# Global, regional, and national epilepsy of unknown cause incidence and mortality, 1990–2036: cross-national health inequalities and predictive analytics

**DOI:** 10.3389/fneur.2025.1526984

**Published:** 2025-06-30

**Authors:** Yi Wang, Yichen Wang, Xinyu Qiu, Zhenjie Yu, Lijun Wang

**Affiliations:** ^1^Tianjin Medical University, Tianjin, China; ^2^Tianjin University, Tianjin, China; ^3^Department of Infectious Diseases and Public Health, Jockey Club College of Veterinary Medicine and Life Sciences, City University of Hong Kong, Kowloon, Hong Kong SAR, China; ^4^Tianjin Fourth Central Hospital Aliated to Tianjin Medical University, Tianjin, China

**Keywords:** global burden disease, epliepsy, epilepsy of unknown cause, YLD, years lived with a disability, health inequalities analysis, predictive analytics, BAPC prediction model

## Abstract

**Background:**

The responsibility for the disease burden associated with epilepsy of unknown cause is unevenly distributed across different nations. It is crucial to describe and forecast cross-national health disparities in terms of years lived with disability (YLDs) for the forthcoming 15-year period. We examined and projected trends in the burden of disease and cross-national inequalities in epilepsy of unknown cause globally, by region and country from 1990 to 2036.

**Methods:**

Our dataset, sourced from the Global Burden of Disease Study 2021, details the number of deaths, morbidity instances, and YLDs due to epilepsy of unknown cause. The distribution of YLDs across varying levels of sociodemographic indices (SDI) was quantified using the slope index of inequality (SII) and the relative concentration index (RCI). Additionally, autoregressive integrated moving average models were utilized to predict future trends in SII and RCI. We used Bayesian age-period-cohort (BAPC) models to predict global, national, and regional trends in age-standardized mortality rates (ASDR), age-standardized incidence rates (ASIR), and age-standardized young-onset rates (ASYR) for epilepsy of unknown cause over the next 15 years. We excluded countries or regions with a total number of age-group cases <5 (42 cases in total) because of potential problems with the sparsity of age-group data in countries with very small populations, resulting in the non-convergence of the BAPC model.

**Results:**

In 2021, epilepsy of unknown cause was responsible for 0.14 million deaths worldwide, with 3.27 million morbidity cases and 7.27 million YLDs recorded. Correlation analysis revealed a significant negative association between ASDR, ASYR, and SDI, while ASIR showed a weak and statistically insignificant positive correlation with SDI. The 1990–2021 SII and RCI values for epilepsy of unknown cause YLDs have been negative. The SII and RCI for YLDs show a continuing downward trend, which is expected to continue over the next 15 years. Projections for the next 15 years show that both ASIR (71.04%) and ASYR (55.74%) will increase in most countries or regions while ASDR (75.41%) will decrease in most countries or regions.

**Conclusion:**

Health inequalities in the world’s idiopathic epileptic YLDs will continue to increase in the future, and the disease burden of idiopathic epileptic YLDs will become more concentrated in low-income countries.

## Background

Epilepsy, a clinical syndrome characterized by highly synchronized abnormal neuronal discharges in the brain due to a variety of causes, remains one of the most prevalent chronic severe neurological disorders worldwide, affecting approximately 50 million people (1). The onset of epilepsy often imposes substantial psychological and financial burdens on both the individual and their family. Compounded by widespread misunderstanding, fear, and discrimination against its clinical symptoms, these factors contribute to delays in effective management. However, advancements in social awareness and global economic growth are anticipated to alleviate the overall disease burden of epilepsy.

According to the latest Global Burden of Disease (GBD) study, epilepsy is categorized as secondary epilepsy when epilepsy is thought to have an underlying cause, for example, head trauma or stroke. Whereas if there is no underlying cause, it is categorized as epilepsy of unknown cause (1). The GBD database provides a comprehensive overview of the burden of disease in epilepsy of unknown cause, and therefore, this paper will focus on the burden of disease in epilepsy of unknown cause. In 2021, epilepsy of unknown cause constituted 44.7% of all epilepsy-related impairments. Moreover, it ranked third in the number of Years Lived with Disability (YLDs) within the GBD Neurological Diseases Subset, following only dementia-related disorders such as migraine and Alzheimer’s Disease. The burden of epilepsy is notably more severe in developing countries due to relatively poorer economic conditions, leading to higher morbidity and mortality rates than those observed in more affluent nations (2, 3). This disparity is likely linked to inadequate healthcare funding, limited knowledge about epilepsy, and restricted access to advanced treatments, including surgical interventions (4–6).

Additionally, prior studies have demonstrated the unequal distribution of this burden across different sociodemographic indices (SDI), with a notable concentration in lower SDI countries in 2019 and a reduction in health inequalities in disability-adjusted life years (DALYs) compared to 1990 (7, 8). Although the trend of health inequality in YLDs associated with epilepsy of unknown cause across various SDI regions has been noted, detailed studies on this trend are lacking. Understanding these trends could elucidate differences in the quality of life among epilepsy patients of various social statuses and aid in formulating targeted policies by nations within diverse SDI regions.

Therefore, using the most recent data from the GBD database, we present the worldwide burden of disease for epilepsy of unknown cause in 2021, highlighting disparities in the burden of disease across countries and regions and predicting trends over the next 15 years. This effort seeks to provide empirical support for enhancing the quality of life for those with epilepsy of unknown cause in varied regions and countries, reducing health disparities, and diminishing the global burden of the disease.

## Methods

### Data source and definitions

The GBD 2021 Global Burden of Diseases, Injuries, and Risk Factors study uses 100,983 data sources and the latest standardized methodologies to provide a comprehensive assessment of the burden of disease for 371 diseases and injuries globally and in 7 super-regions, 21 regions, and 204 countries and territories. We considered similar trends in mortality and disability-adjusted life years (DALYs), and our study used mortality, morbidity, and YLDs to describe the burden of disease in an integrated manner. For each disease and injury, YLDs were calculated by multiplying cause-age-sex-location-year-specific prevalence of sequelae by their respective disability weights. Our study also used SDI, an indicator that measures the level of economic development and social welfare of a country or region based on several aspects such as per capita income, health status, and fertility, etc., and GBD 2021 categorizes different countries and territories around the world into five different regions according to SDI (9). We selected the number of deaths, morbidities, YLDs, mortality rates, incidence rates, YLDs rates and age-standardized mortality rates (ASDR), age-standardized incidence rates (ASIR), and age-standardized YLDs rates (ASYR) for epilepsy of unknown cause in different age groups and different sexes globally, across 21 regions, and in 204 countries from the GBD database. The diagnosis of epilepsy of unknown cause here is based on the Epidemiological Study Guide for Epilepsy published by the International League Against Epilepsy (ILAE) (10).

Additionally, we used the age structure provided by the World Health Organization’s Global Demographic Criteria and extracted population data for 1990–2021 and projected population data for 2022–2036 from the Global Health Data Exchange.^1^ The GBD study employs a standardized statistical modeling framework to impute values for regions with missing or incomplete data. These models integrate covariates (for example, socioeconomic, demographic, or geographic variables) and leverage data from neighboring regions or populations with similar epidemiological profiles. Detailed methodologies are described in the GBD technical appendices and adhere to the Guidelines for Accurate and Transparent Health Estimates Reporting (GATHER) (11).

### Health inequality and auto-regressive integrated moving average model analysis

The slope index of inequality (SII) and the relative concentration index (RCI) are standardized indicators of absolute and relative health inequality, respectively, used to quantify inequality distribution in epilepsy of unknown cause YLDs across countries. SII was calculated from regression analyses established between the relative position of gross national product and national epilepsy of unknown cause YLDs, with positive values indicating that the burden of disease is concentrated in higher-income populations and negative values indicating that the burden of disease is focused on lower-income populations. The RCI was calculated based on the Lorenz curve, comparing differences in the distribution of YLDs between different groups. Larger absolute values of both represent more significant health inequalities.

We calculated each year’s SII and RCI values from 1990 to 2021 and predicted the trends over the next 15 years through an auto-regressive integrated moving average (ARIMA) forecasting model. A description of the specific details of ARIMA could be found in a previous article (12). We used the auto.arima() function of the R tool to select the best-optimized model and finally chose the (1,1,0) parameter to build the ARIMA model for SII and RCI. Both passed the Ljung-Box Q test and the Breush-Godfrey LM test, the model residuals were white noise, and there was no serial correlation in the residual series. We report the Akaike Information Criterion, Corrected Akaike Information Criterion, Bayesian Information Criterion, Mean Error of the model, Root Mean Squared Error (RMSE), Mean Absolute Error (MAE), Mean Percentage Error, Mean Absolute Percentage Error (MAPE), Mean Absolute Scaled Error, Autocorrelation Function at lag 1 to assess the predictive power of the model. Subsequently, a more extensive evaluation of the model’s robustness and precision was conducted through time series cross-validation (tsCV) and rolling-window validation.

### Bayesian age-period-cohort model analysis

Bayesian age-period-cohort (BAPC) modeling combines the strengths of Bayesian methods with the complexity of time-series data for high accuracy in predicting disease burden. The specific details of the BAPC model have been described in previous articles (13, 14). Since very low-population countries may have age group data sparsity problems, resulting in the BAPC model not converging, so we excluded countries or regions with total age group cases <5 (42 in total), and finally, we included the world, 21 regions, and 162 countries and regions to predict the trend of the burden of epilepsy of unknown cause over the next 15 years, using the “BAPC” and “INLA” packages of R. “INLA” package to predict trends in the burden of disease in epilepsy of unknown cause over the next 15 years. We conducted an *a priori* sensitivity analysis to assess the model’s robustness by systematically varying key hyperparameters in our Bayesian Age-Period-Cohort framework. This included testing alternative configurations with: (1) stronger smoothing priors [loggamma(1, 0.001)], (2) weaker smoothing priors [loggamma(1, 1e−06)], (3) exclusion of the cohort component, and (4) default: Age, period, and cohort were used with RW2 *a priori* [loggamma(1, 5e−05)]. The stability of the model predictions was quantified by the maximum relative difference (MaxRD) over the prediction period—a maximum relative variance of <10% was considered relatively stable for the model. Finally, we rigorously assessed the model’s predictive performance through an enhanced time-series cross-validation framework (k-fold with train_window = 12, test_window = 3), employing a rolling-origin evaluation strategy across [n_iter] temporal partitions. The accuracy of the model predictions is assessed by a combination of time series cross-validation results: R-squared (*R*^2^), Root Mean Squared Error, Mean Absolute Error, and Mean Absolute Percentage Error. If *R*^2^ is greater than 0.7, the model is initially considered to have good predictive ability; 0.5 ≤ *R*^2^ ≤ 0.7, the model is considered to have medium predictive ability; and if *R*^2^ is less than 0.5, the model is considered to have poor predictive ability. For models with *R*^2^ greater than 0.7 but unstable, we provide the posterior distribution density plots of the sensitivity analysis for reference. The a posteriori distribution density plot shows the difference in the a posteriori distribution of the parameters under different configurations through the Kernel Density Estimation curve.

### Statistical analysis

Mortality, morbidity, YLD rates, and ASDR, ASIR, and ASYR in our study were expressed as numbers per 100,000 people, all displaying 95% uncertainty interval (UI) values. We used curve fitting methods and Pearson correlation analysis to demonstrate the relationship between SDI and ASDR, ASIR, and ASYR. *p* < 0.05 was considered statistically significant. All the analysis results were obtained using the R tools (version 4.2.3), and the data we used and analyzed are shown in the attached table.

## Results

**Figure 1:**
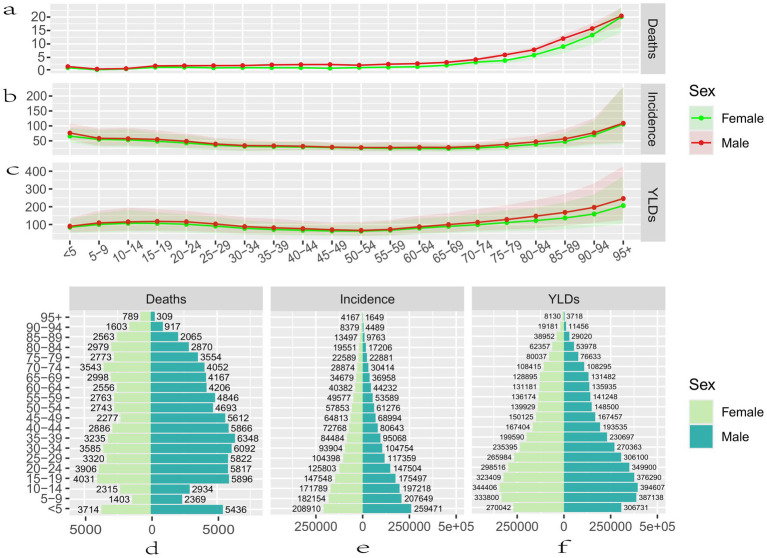
The disease burden of idiopathic epilepsy in different age groups and genders worldwide in 2021. Mortality rate **(a)**, Incidence rate **(b)**, YLDs rate **(c)**, Number of deaths **(d)**, Number of incidence **(e)**, Number of YLDs **(f)**. YLDs, years lived with disability; all rates are expressed per 100,000 people.

In 2021, there were 0.14 million (95% UI: 0.12, 0.15) deaths due to epilepsy of unknown cause, 59.97% of which were in males, with the highest number of deaths occurring between the ages of 35–39 years, amounting to 6,348 (95% UI: 4,727, 7,438). In females, the highest number of deaths occurred between the ages of 15–19 years, amounting to 4,031 (95% UI: 2,880, 5,461). The ASDR was 1.74 (95% UI: 1.46, 1.92) per 100,000 people (Figure 1d). In children and adolescents, mortality rates were significantly higher in the <5 and 15–19 age groups than in the other two age groups, respectively 1.39 (95% UI: 0.95, 1.74) per 100,000 and 1.59 (95% UI: 1.30, 1.88) per 100,000. After that, the change in mortality rates leveled off at all ages until a marked increase in mortality began at ages 60–64, with mortality rates rising to a maximum of 20.14 (95% UI: 14.83, 23.30) per 100,000 persons at ages >95 years (Figure 1a).

The number of incidences of epilepsy of unknown cause tended to increase with age. The total number of incidences was 3.27 million (95% UI: 2.4, 4.13) (Figure 1e), and the ASIR was 42.82 (95% UI: 31.24, 53.72) per 100,000 people. It decreased to a minimum in the 65–69 age group, prior to which incidence tended to decrease with age. After this age group, the prevalence tended to increase with age, and, as with the mortality rate, it rose to a maximum at >95 years of age (Figure 1b).

The total YLDs for epilepsy of unknown cause in 2021 were 7.27 million (95% UI: 4.42, 10.97), with YLDs being most extraordinary in the age group 10–14 years, after which they gradually began to decline (Figure 1c). The overall ASYR was 92.87 (95% UI: 56.44, 140.48) per 100,000 people; the rate of YLDs increased with age until adulthood, then decreased with age to a minimum in the age group 50–54, after which there was a tendency for YLDs to increase with age, in the same way as mortality and morbidity, rising to a maximum at >95 years (Figure 1f).

**Figure 2:**
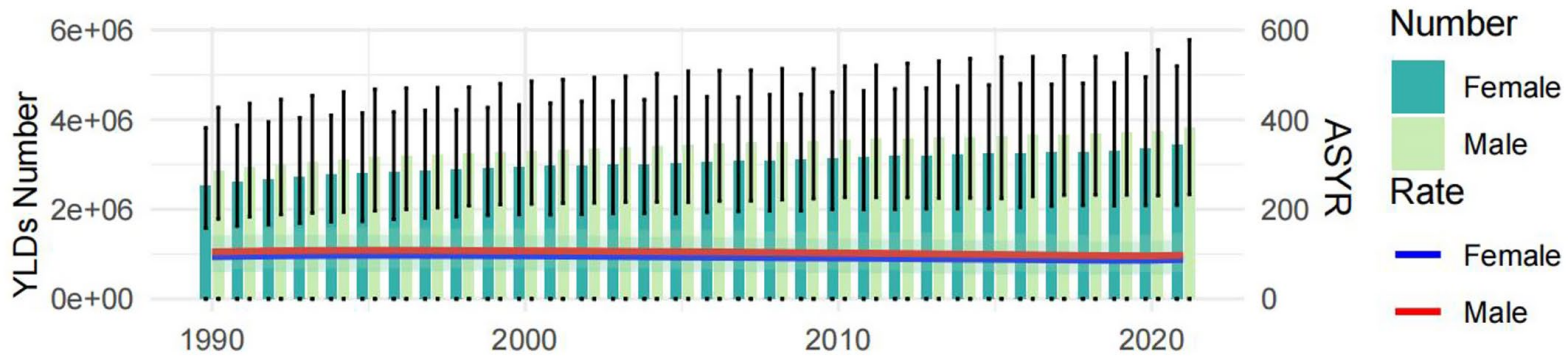
The global disease burden associated with idiopathic epilepsy, categorized by gender, over the period spanning 1990–2021. ASYR, Age-standardized YLDs rate; YLDs, years lived with disability. All rates are expressed per 100,000 people.

**Figure 3:**
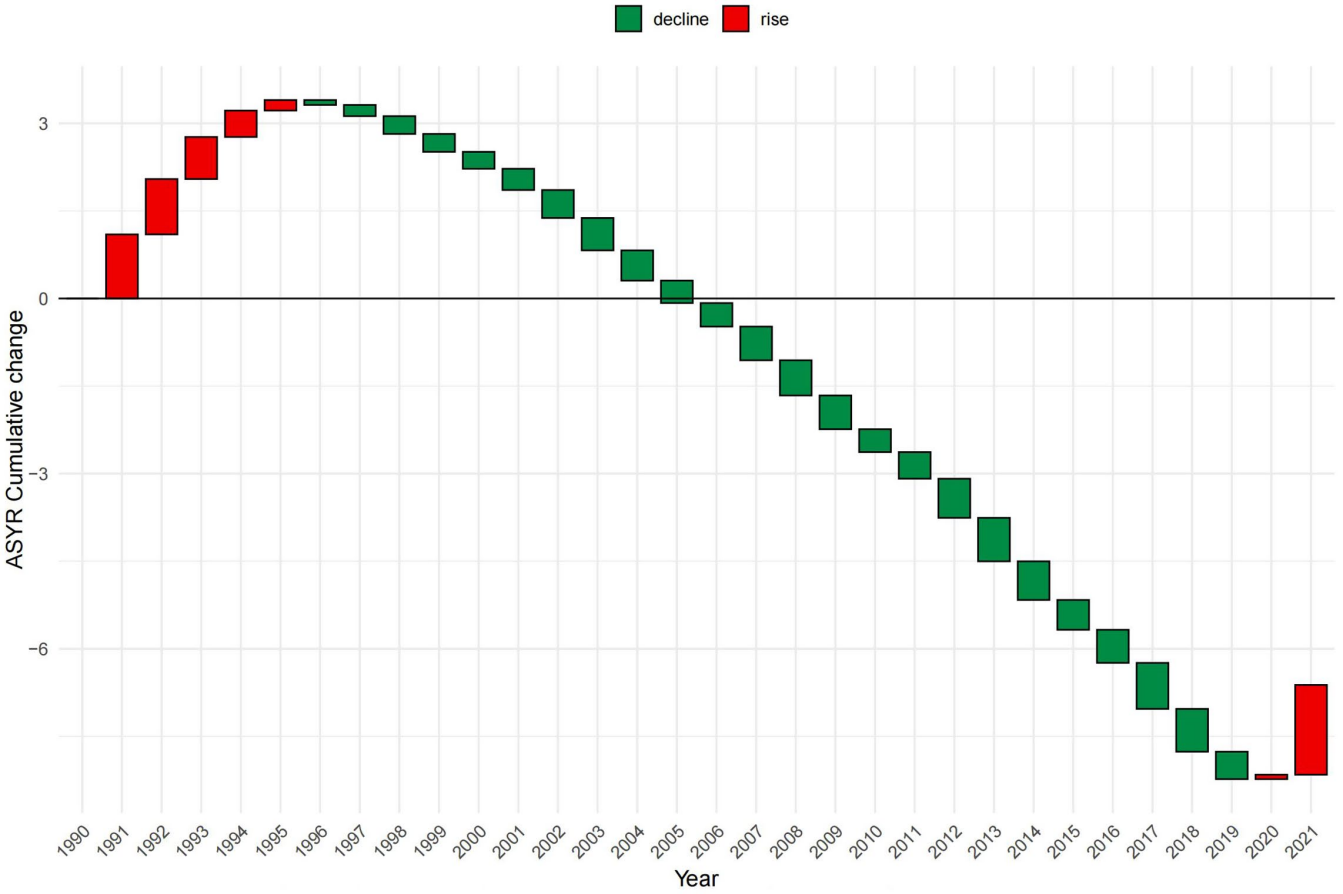
Trends in global total population burden of disease for idiopathic epilepsy, 1990–2021, with 1990 as the zero value. ASYR, Age-standardized YLDs rate; YLDs, years lived with disability. All rates are expressed per 100,000 people.

The number of deaths, incidences, and YLDs due to epilepsy of unknown cause increased globally each year from 1990 to 2021, showing increases of 34.01, 54.29, and 34.79%, respectively, over these 32 years. However, since 2018, following the first decline in deaths from the previous year, the growth in deaths slowed to a 0.8% increase in 2019 and then turned negative again in 2020, resulting in a 0.57% decline from 2019 to 2021. The overall trends in ASDR and ASYR have decreased by 15.76 and 6.65%, respectively, over these 32 years. However, ASDR increased from 2013 to 2017, and ASYR increased from 1990 to 1995 before following a decreasing trend until 2020, when it showed an increase of 1.77% compared to 2019. ASIR increased yearly, with a total increase of 12.32%. There was an upward trend until 2015, after which the ASIR showed a downward trend until 2020, when it again showed an increase, reaching a maximum value of 42.92 (95% UI: 31.24, 53.72) per 100,000 people in 2021. Regardless of the indicator, men were consistently higher than women, but the overall trend was similar for both. (Figures 2, 3 and Supplementary Figures 3.1–3.4).

### Distribution of epilepsy of unknown cause disease burden by country and region

**Figure 4:**
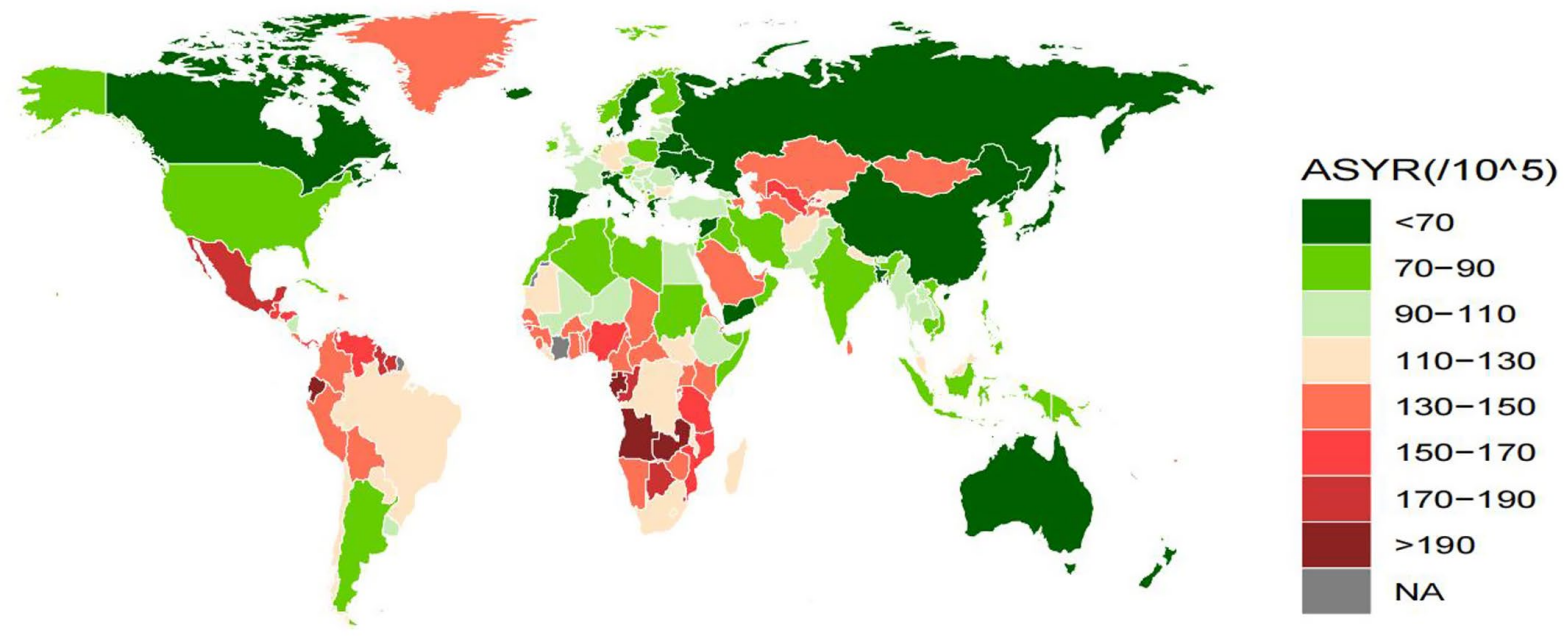
Global distribution map of ASYR for idiopathic epilepsy in 2021. ASYR, Age-standardized YLDs rate; YLDs, years lived with disability. All values are per 100,000 population.

Comparing the ASDR, ASIR, and ASYR for epilepsy of unknown cause in different countries in 2021, the countries with the lowest rates for these three were Vietnam (0.08, 95% UI: 0.01, 0.32), North Korea (21.74, 95% UI: 5.9, 38.69), Sweden (53.6, 95% UI: 16.28, 116.87), and the highest countries were Zambia (12.94, 95% UI: 9.47, 17.08), Ecuador (94.94, 95% UI: 29.94, 160.51), Equatorial Guinea (229.82, 95% UI: 51.37, 481.74). As illustrated by the global heat map, the ASDR, ASIR, and ASYR were relatively low in countries such as Canada, Sweden, Spain, Ukraine, Belarus, Russia, China, North Korea, Japan, and Australia. In contrast, all three indicators exhibited relatively high values in East Africa, West Africa, and Central Africa. (Figure 4 and Supplementary Figures 5.1, 5.2).

**Figure 5:**
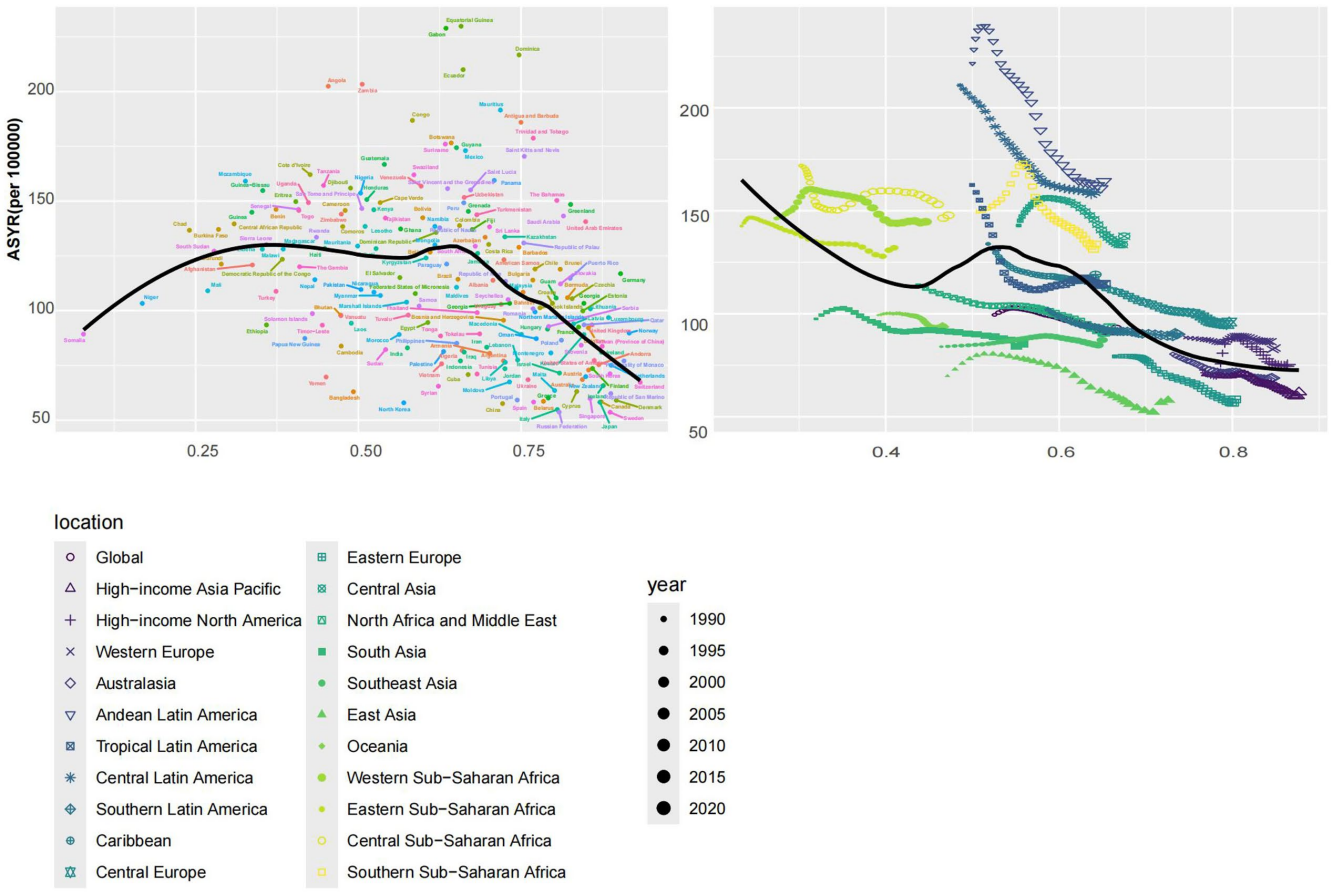
The left panel shows the relationship between the burden of idiopathic epilepsy and the SDI in 204 countries or regions in 2021, and the right panel shows the relationship between the burden of idiopathic epilepsy and the SDI globally and in 21 regions from 1990 to 2021. ASYR, Age-standardized YLDs rate; SDI, socio-demographic index; YLDs, years lived with disability. All values are per 100,000 population.

**Figure 6:**
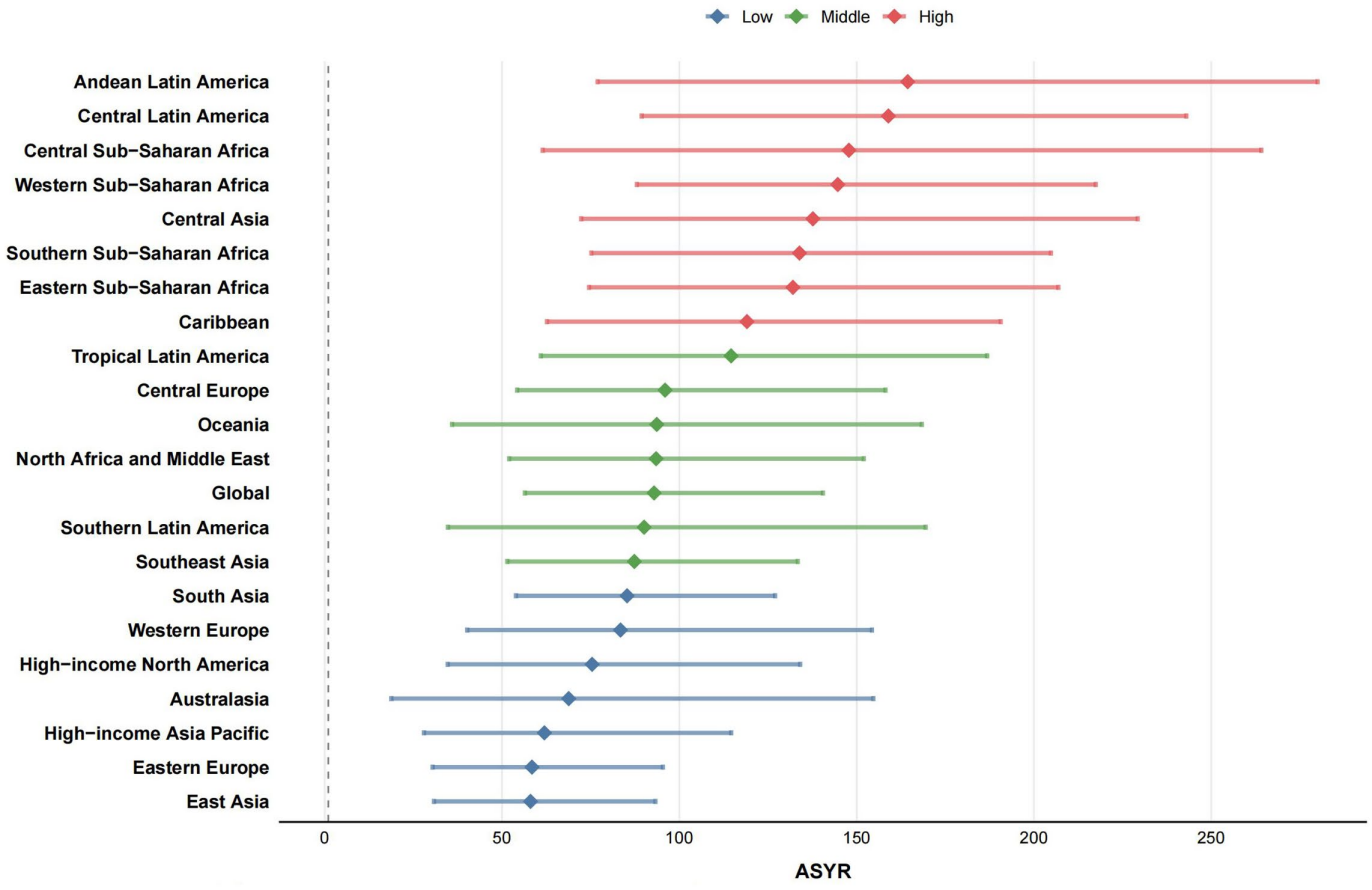
ASYR burden of idiopathic epilepsy globally and in 21 regions in 2021, categorized into three categories based on high and low burden, with high, middle and low indicated in red, green and blue, respectively. ASYR, Age-standardized YLDs rate; YLDs, years lived with disability. All rates are expressed per 100,000 people.

ASDR, ASYR, and SDI in 204 countries showed a significant negative correlation (*R* = −0.69, *p* < 0.001, *R* = −0.37, *p* < 0.001). Similar results were obtained between ASDR, ASYR, and SDI in 21 regions and globally (*R* = −0.72, *p* < 0.001, *R* = −0.51, *p* < 0.001), and ASIR had a weak positive correlation with SDI. However, it was not statistically significant (*R* = 0.11, *p* = 0.132, *R* = 0.03, *p* = 0.445). (Figure 5 and Supplementary Figures 4.1, 4.2). In 2021, Sub-Saharan Africa will have high ASIR, ASYR, and ASDR, while Latin America will have relatively low levels of ASDR, although ASIR and ASYR are also very high. We can see that the three indicators of epilepsy of unknown cause are low in high-income regions such as East Asia and Eastern Europe (Figure 6 and Supplementary Figures 4.3, 4.4).

### Global, regional, and national changes in the burden of disease for epilepsy of unknown cause in the next 15 years

**Figure 7:**
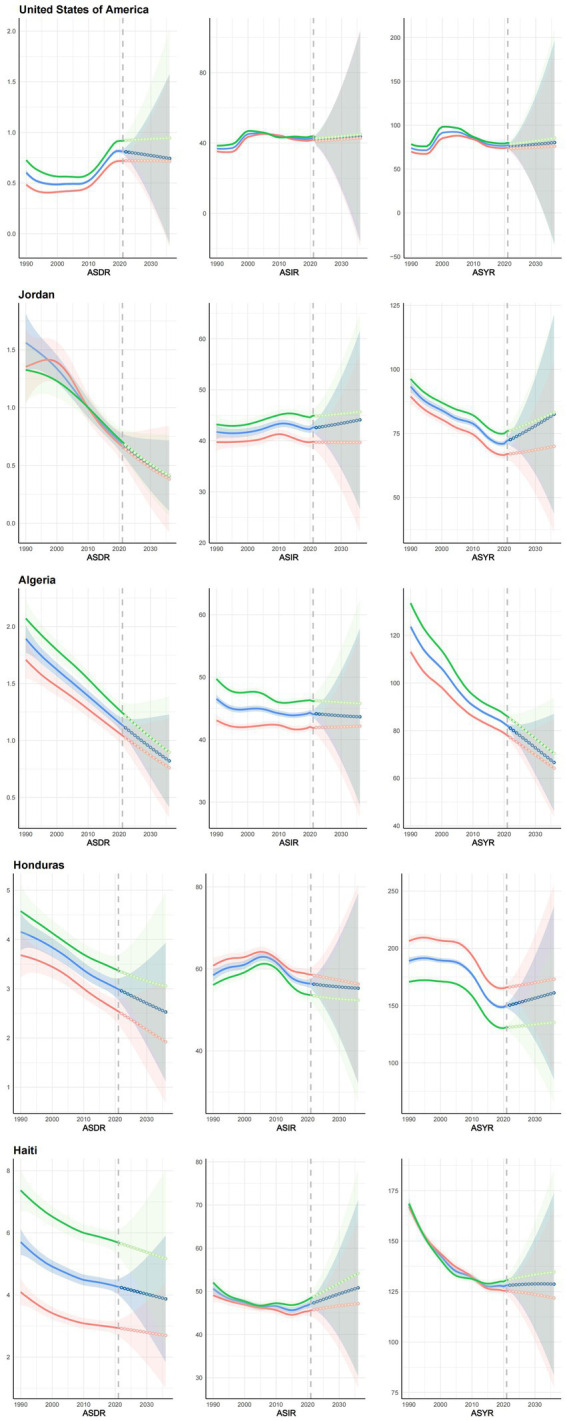
Trend projections for ASDR, ASIR and ASYR for representative countries globally and in each SDI region, 1990–2036. The name of the country is labeled in the upper left corner, and the shaded area represents the 95% confidence interval. ASDR, Age-standardized death rate; ASIR, Age-standardized incidence rate; ASYR, Age-standardized YLDs rate; SDI, socio-demographic index; YLDs, years lived with disability. All values are per 100,000 population.

Utilizing the Bayesian age-period-cohort (BAPC) prediction model, we forecasted the future trends of ASDR, ASIR, and ASYR for different regions, countries, and genders over the next 15 years, as shown in Figure 7 and Supplementary Figures 1, 2. Using Supplementary Tables 1, 2, we find 102 (55.74%) regions or countries that will experience an upward trend in ASYR in the future, with the East Asia region (*R*^2^: 0.02; MaxRD: 27.9%) and Bangladesh (*R*^2^: 0.94; MaxRD: 24.8%) demonstrating the most pronounced increase. By 2036, the region of Andean Latin America (*R*^2^: 0.61; MaxRD: 8%) and the country of Uzbekistan (*R*^2^: 0.79; MaxRD: 37%) will have the highest ASYR with 185.89 (81.59, 290.20) persons/100,000 and 284.22 (77.04, 491.41) persons/100,000, respectively. However, prediction results are highly unstable. The most pronounced decline among the 162 countries is in Germany (*R*^2^: 0.62; MaxRD: 27.4%), with 66.22 (25.81, 106.63) persons/100,000 by 2036. The lowest ASYR by 2036 is in Sweden (*R*^2^: 0.79; MaxRD: 10.4%), with 49.56 (29.07, 70.04) persons/100,000. Among the high SDI regions, the ASYR of the future high-income Asia-Pacific region (*R*^2^: 0.73; MaxRD: 24.5%) and the high-income North America region (*R*^2^: 0.92; MaxRD: 4.6%) are increasing. The Republic of Korea (*R*^2^: 0.91; MaxRD: 30.2%) is exhibiting the highest rate of growth. Nineteen countries (55.88%) in the high SDI region will see their ASYR decline over the next 15 years. Among the low SDI regions, the subsequent 21 countries (65.63%) demonstrate an upward trajectory, with Nepal (*R*^2^: 0.01; MaxRD: 15%) exhibiting the most pronounced increase and Côte d’Ivoire (*R*^2^: 0.48; MaxRD: 3.4%) exhibiting the highest ASYR of 196.17 (123.80, 268.54) persons/100,000 by 2036. Eastern Sub-Saharan Africa (*R*^2^: 0.54; MaxRD: 2.3%) is also on an upward trend, in contrast to Western Sub-Saharan Africa (*R*^2^: 0.7; MaxRD: 1.7%), which is on a downward trend, albeit a small one. Because of space constraints, the ASDR and ASIR for each region and country are not repeated here and are detailed in Supplementary Tables 1, 2.

BAPC modeling was performed for males, females, and total populations globally, in 21 regions and 162 countries or territories, respectively. In total, we built 1,656 BAPC prediction models, and the results of the model prior sensitivity analysis and tsCV showed that only 187 models had *R*^2^ > 0.7 and MaxRD < 10%. Of the 162 countries or regions, 33 of ASDR’s forecasting models are good and robust, including 23 countries. In addition, 83 models were good but unstable, and 91 models were moderately good, including 19 robust ones. ASIR’s prediction models included 64 good and robust models, 83 good but unstable models, and 86 moderately predictive models, 43 of which were robust. ASYR’s prediction models had 56 models that predicted well and were robust, 120 models that predicted well but the models were unstable, and 82 models that predicted moderately well, of which 39 models were robust. Predicted performance indicators for specific countries and regions are shown in Supplementary Tables 3, 4.

In order to provide a more detailed analysis of the distinctions between the various SDI regions or countries, we selected representative countries from each of the different SDI regions for examination. Among all the countries and regions, only Algeria and Haiti have stable predictive models with good predictive performance for the three indicators for women, so we chose to show the visualization results of the two countries as a representative of the middle SDI and low SDI regions, respectively. In the total population, the model prediction performance of ASDR, ASIR, and ASYR of Honduras and Jordan are very good, so we chose these countries to represent low-middle SDI and high-middle SDI regions, respectively. Finally, we chose the United States of America to represent the high SDI region.

From Figure 7, it can be seen that the ASDR for the total US population (*R*^2^: 0.15; MaxRD: 13.3%) and females (*R*^2^: 0; MaxRD: 17%) showed a decreasing trend, but males showed the opposite trend, and this opposite trend may be a result of the poorer predictive accuracy of the model. The ASIR (Both: *R*^2^: 0.93; MaxRD: 7.6%, Female: *R*^2^: 0.91; MaxRD: 8.9%, Male: *R*^2^: 0.86; MaxRD: 4.3%) and ASYR (Both: *R*^2^: 0.92; MaxRD: 5.1%, Female: *R*^2^: 0.94; MaxRD: 7.1%, Male: *R*^2^: 0.88; MaxRD: 3.6%) in the US will trend upward in the future, but to a lesser extent, and will generally be relatively stable. Jordan’s ASIR (Both: *R*^2^: 0.89; MaxRD: 29.8%, Female: *R*^2^: 0.75; MaxRD: 18.8%, Male: *R*^2^: 0.95; MaxRD: 26.3%) and ASYR (Both: *R*^2^: 0.9; MaxRD: 31.6%, Female: *R*^2^: 0.92; MaxRD: 36.3%, Male: *R*^2^: 0.89; MaxRD: 44.9%) are trending upward like the US, and the ASDR (Both: *R*^2^: 0.84; MaxRD: 6.7%, Female: *R*^2^: 0.1; MaxRD: 23.3%, Male: *R*^2^: 0.57; MaxRD: 25.4%) is trending downward, but to a greater extent. In addition, Jordan’s long-term forecast performance for the three indicators is less uncertain compared to the US Algeria’s future ASDR (Both: *R*^2^: 0.97; MaxRD: 2.2%, Female: *R*^2^: 0.92; MaxRD: 2.6%, Male: *R*^2^: 0.97; MaxRD: 4.3%) trends are similar to Jordan’s, showing a clear downward trend. However, ASIR (Both: *R*^2^: 0.68; MaxRD: 11.7%, Female: *R*^2^: 0.76; MaxRD: 7.8%, Male: *R*^2^: 0.16; MaxRD: 10.1%) and ASYR (Both: *R*^2^: 0.96; MaxRD: 6%, Female: *R*^2^: 0.97; MaxRD: 7.2%, Male: *R*^2^: 0.94; MaxRD: 5.1%) show a downward trend contrary to the US and Jordan, especially ASYR, with a clear downward trend and less uncertainty in the long-term forecast.

Honduras (Both: *R*^2^: 0.77; MaxRD: 6%, Female: *R*^2^: 0.65; MaxRD: 8.7%, Male: *R*^2^: 0.72; MaxRD: 5.5%) and Haiti’s (Both: *R*^2^: 0.49; MaxRD: 5.7%, Female: *R*^2^: 0.72; MaxRD: 6.9%, Male: *R*^2^: 0.24; MaxRD: 6.8%) future ASDRs are also trending downward, but the uncertainty in the long-term future projections is greater compared to the previous three countries. Honduras (Both: *R*^2^: 0.87; MaxRD: 6.2%, Female: *R*^2^: 0.83; MaxRD: 5.3%, Male: *R*^2^: 0.9; MaxRD: 13.3%) and Haiti’s (Both: *R*^2^: 0.75; MaxRD: 5.1%, Female: *R*^2^: 0.81; MaxRD: 6.5%, Male: *R*^2^: 0.49; MaxRD: 12.2%) future ASIRs show opposite trends, with the former trending downward and the latter trending upward. Honduras’ future ASYR (Both: *R*^2^: 0.84; MaxRD: 18.7%, Female: *R*^2^: 0.54; MaxRD: 10.6%, Male: *R*^2^: 0.93; MaxRD: 17.8%) performs similarly to Jordan. Surprisingly, Haiti’s male and female ASYR (Both: *R*^2^: 0.95; MaxRD: 6.1%, Female: *R*^2^: 0.95; MaxRD: 3.7%, Male: *R*^2^: 0.89; MaxRD: 5.6%) trends show opposite results, with females showing a downward trend.

The overall trends in ASDR, ASIR, and ASYR are similar between the sexes, with males outnumbering females in most regions and countries. However, the burden of epilepsy of unknown cause disease in women may exceed that of men in some countries in the next 15 years, such as ASDR (Female: *R*^2^: 0; MaxRD: 11.5%, Male: *R*^2^: 0.01; MaxRD: 5.7%), ASIR (Female: *R*^2^: 0.27; MaxRD: 2.8%, Male: *R*^2^: 0.87; MaxRD: 1%), ASYR (Female: *R*^2^: 0.77; MaxRD: 9.3%, Male: *R*^2^: 0.64; MaxRD: 9.4%) in Colombia, ASIR (Female: *R*^2^: 0.88; MaxRD: 29.2%, Male: *R*^2^: 0.85; MaxRD: 30.3%), ASYR (Female: *R*^2^: 0.63; MaxRD: 21.6%, Male: *R*^2^: 0.58; MaxRD: 22.7%) in Germany. There are also countries or regions where the burden of epilepsy of unknown cause disease is consistently higher in females than in males, for example, ASDR (Female: *R*^2^: 0.41; MaxRD: 10.6%, Male: *R*^2^: 0.05; MaxRD: 18.6%), ASIR (Female: *R*^2^: 0.19; MaxRD: 4.4%, Male: *R*^2^: 0.83; MaxRD: 19.9%), ASYR (Female: *R*^2^: 0.38; MaxRD: 14.2%, Male: *R*^2^: 0.61; MaxRD: 30.3%) in Pakistan, ASIR (Female: *R*^2^: 0.38; MaxRD: 28.1%, Male: *R*^2^: 0.63; MaxRD: 5.2%), ASYR (Female: *R*^2^: 0.49; MaxRD: 29.9%, Male: *R*^2^: 0.48; MaxRD: 9.8%) in the Philippines, and ASDR (Female: *R*^2^: 0.73; MaxRD: 4.1%, Male: *R*^2^: 0.93; MaxRD: 11.9%) in Nepal. It is important to note that the confidence intervals for the long-term forecasts of almost all forecasting models are significantly larger, except for a few countries, for example, Iran (Islamic Republic of), where the long-term forecasts of the ASDR (Both: *R*^2^: 0.95; MaxRD: 39.3%, Female: *R*^2^: 0.98; MaxRD: 32.6%, Male: *R*^2^: 0.85; MaxRD: 49.4%) are relatively stable. Trends for all countries and regions are shown in Supplementary Figures 1, 2.

### Future trends in cross-country inequalities

**Figure 8:**
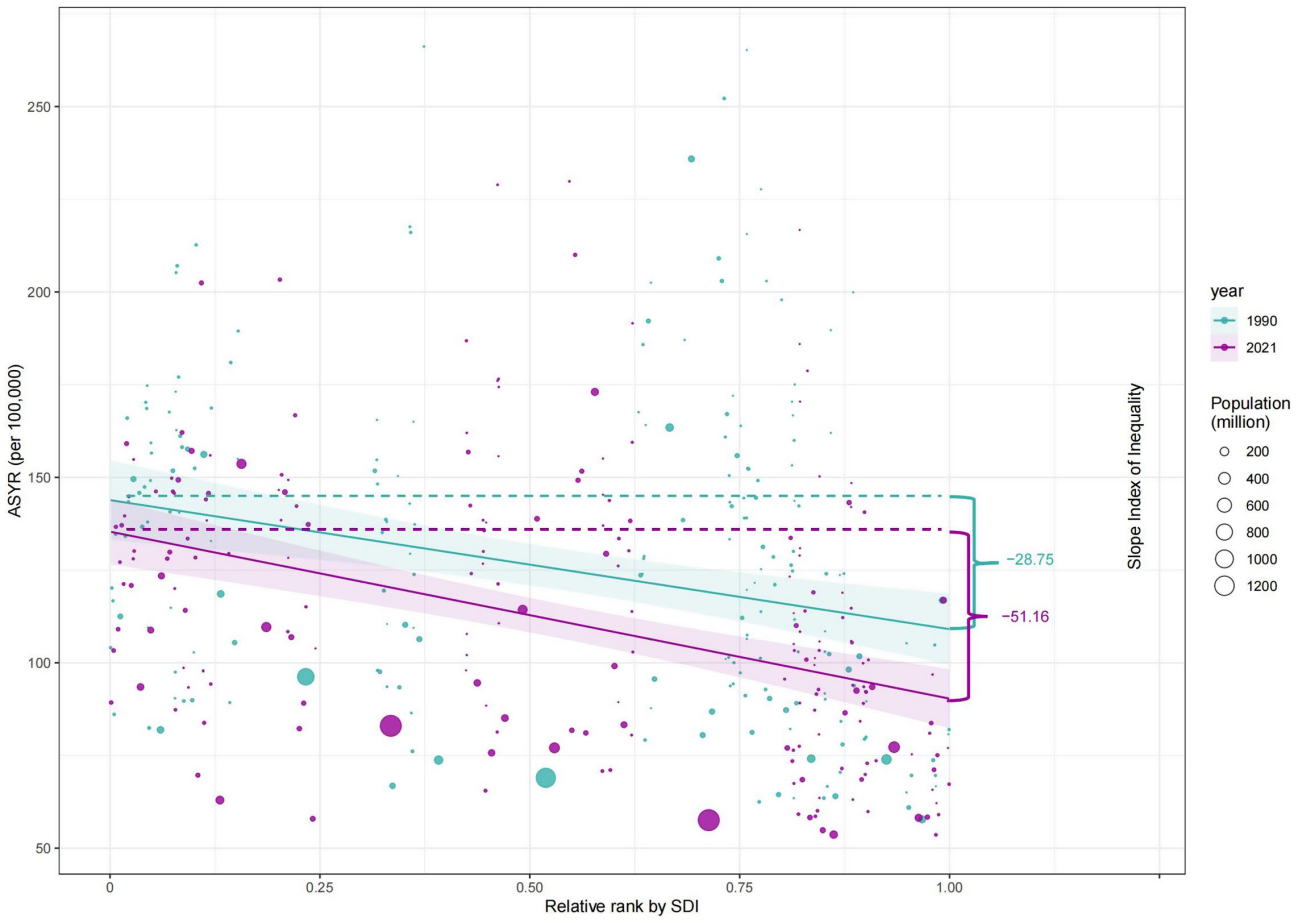
Health inequality regression curves for the ASYR of idiopathic epilepsy worldwide, 1990 and 2021. ASYR, Age-standardized YLDs rate; YLDs, years lived with disability; SDI, socio-demographic index.

**Figure 9:**
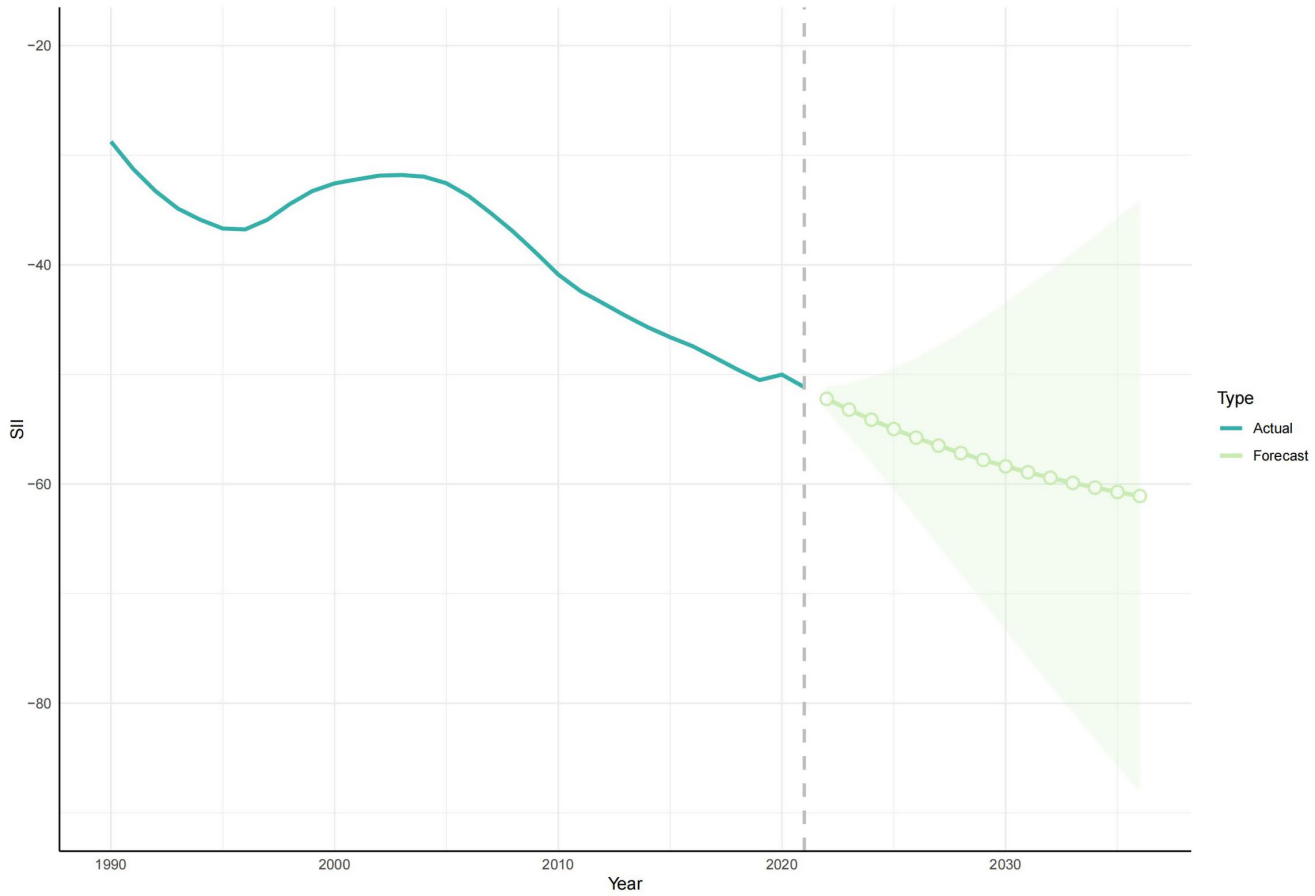
The development trend of SII in 1990–2021 and the future development trend of SII in 2022–2036. SII, Slope index of inequality.

**Figure 10:**
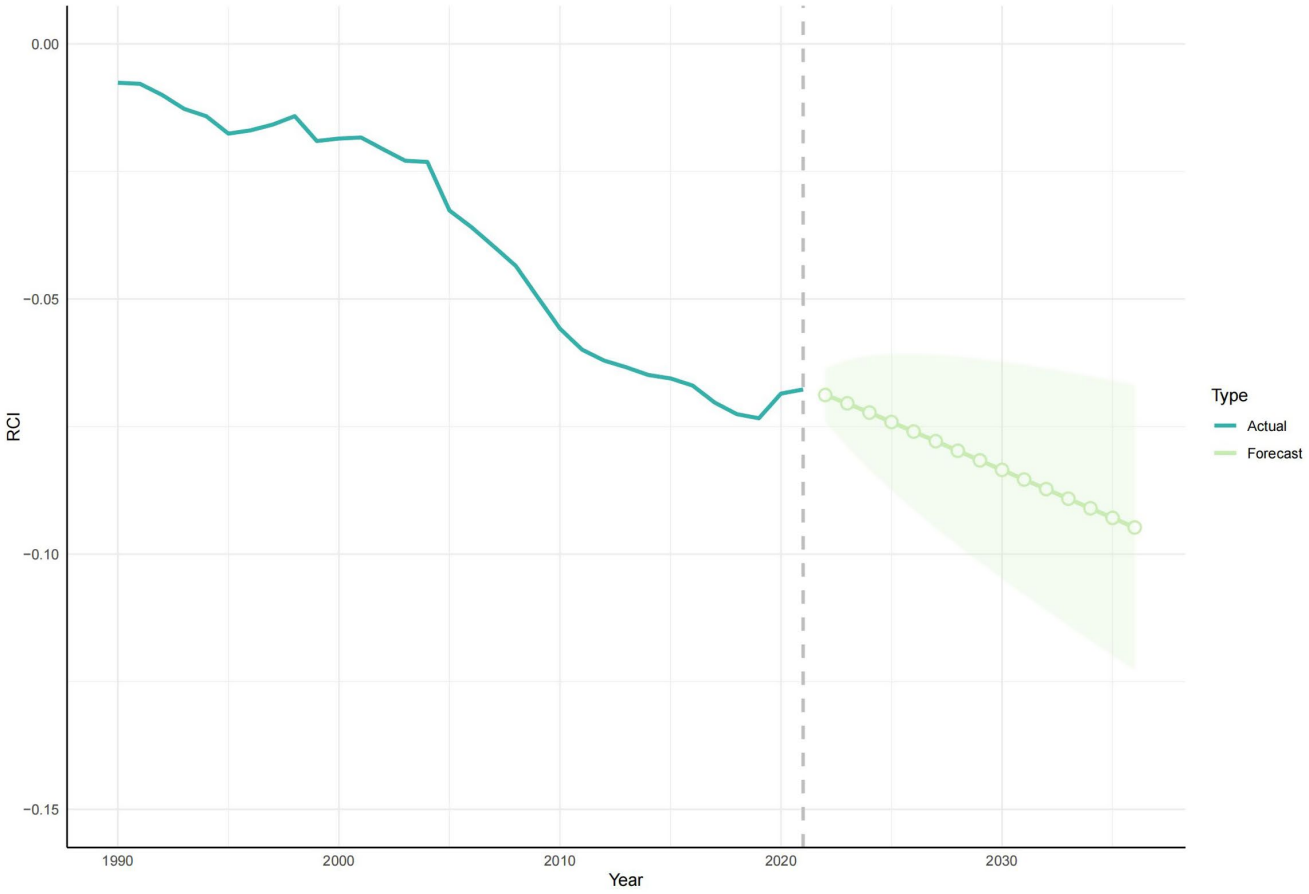
The development trend of RCI in 1990–2021 and the future development trend of RCI in 2022–2036. RCI, relative concentration index.

**Figure 11:**
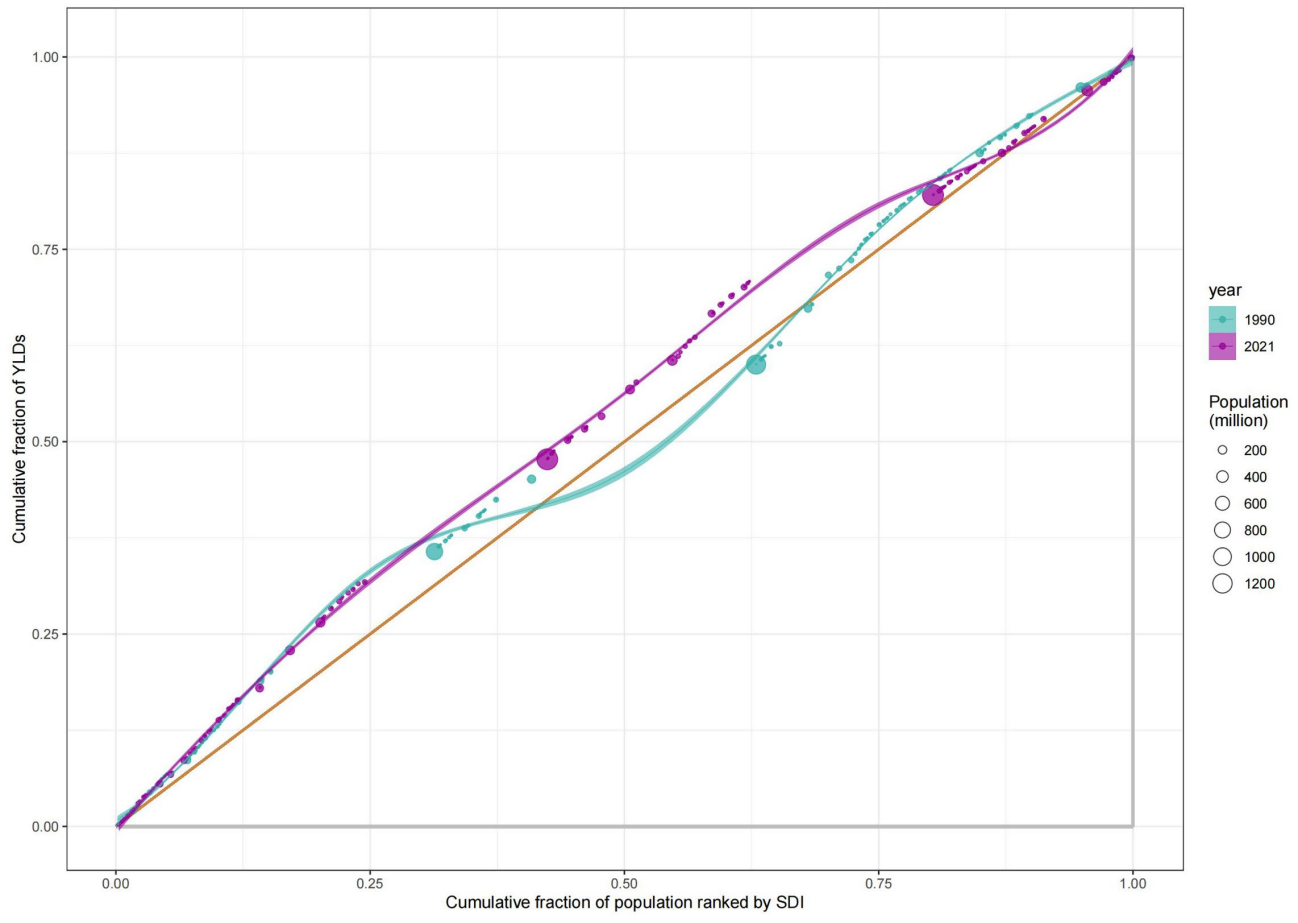
Health inequality concentration curves for the YLDs of idiopathic epilepsy worldwide, 1990 and 2021. YLDs, years lived with disability; SDI, socio-demographic index.

According to the health inequality analysis (Figure 8), the absolute value of the SII increased in 2021 compared to 1990, and the gap between the countries with the highest SDI and those with the lowest SDI increased by 22.41 ASYR per 100,000 people. Except for the period 1996–2003, when the SII was on an upward trend, the SII has been on a downward trend, and the results of the projections for the next 15 years show this trend. The SII forecast trend, generated using the ARIMA model and without the introduction of any additional interventions, demonstrated a decline to −61.1 (95% CI: −88.04, −34.15) by 2036 (Figure 9). The RCI had progressively decreased from 1990 to 2021, including the subsequent trend predicted using the ARIMA model, and was also on a downward trend for the next 15 years (Figure 10). The 2019 Concentration Index curve exhibits an S-shaped curve, with the medium SDI region lying below the diagonal. The 2021 concentration index curves had been overwhelmingly above the absolute equity line, similar to the SII, suggesting that the burden of epilepsy of unknown cause YLDs was concentrated mainly in countries with low SDI levels (Figure 11).

We performed a series of evaluations of the model, and the MAE and MAPE were 1.2 and 2.86% for SII and 0.0067 and 4.3% for RCI, respectively. The RMSE obtained from the time series CV and the MAE obtained from the rolling window validation for SII were 1.45 and 7.25, respectively, and the RMSE obtained from the time series CV and the MAE obtained from the rolling window validation for RCI were 0.0027 and 0.0085, respectively (Table 1). In addition, Figures 9, 10 show that the confidence intervals for the predictions of SII are large, and the uncertainty is high, while the trend of the predictions for RCI is relatively stable. The exact magnitude of the values can be viewed in Table 2.

**Table 1:** Model parameters associated with the ARIMA prediction model.

Name	SII	RCI
tsCV RMSE	1.387896	0.00302456
Rolling MAE	8.844658	0.02108864
Rolling RMSE	9.006269	0.02179945
AIC	56.64	−275.96
AICc	57.07	−275.07
BIC	59.51	−271.65
ME	−0.03581164	−1.82E−05
MAE	0.3840752	0.001783314
RMSE	0.5392908	0.002518747
MPE	0.1177469	−0.8577987
MAPE	1.002803	7.081815
MASE	0.3571049	0.694135
ACF1	0.02279351	−0.02624679

AIC, Akaike Information Criterion; AICc, Corrected Akaike Information Criterion; BIC, Bayesian Information Criterion; ME, Mean Error; RMSE, Root Mean Squared Error; MAE, Mean Absolute Error; MPE, Mean Percentage Error; MAPE, Mean Absolute Percentage Error; MAPE, Mean Absolute Scaled Error; ACF1, Autocorrelation Function at lag 1; tsCV, Time series cross-validation; Rolling, rolling-window validation; SII, Slope index of inequality; RCI, Relative concentration index.

**Table 2:** Actual and projected values of SII and RCI for 1990–2036.

Year	SII	95%Lower	95%Upper	RCI	95%Lower	95%Upper
1990	−28.75163283			−0.007617849		
1991	−31.2574436			−0.007815319		
1992	−33.28168057			−0.010000962		
1993	−34.87477578			−0.012739908		
1994	−35.88556036			−0.014172177		
1995	−36.68630378			−0.017580091		
1996	−36.77133519			−0.016940079		
1997	−35.88273045			−0.015814648		
1998	−34.45264861			−0.014155982		
1999	−33.27362725			−0.019037723		
2000	−32.56830851			−0.018542955		
2001	−32.20133564			−0.018334676		
2002	−31.86265617			−0.020647161		
2003	−31.80616076			−0.022901172		
2004	−31.9576571			−0.023129637		
2005	−32.55415501			−0.032648426		
2006	−33.72769017			−0.035929392		
2007	−35.30562458			−0.039673414		
2008	−36.99747437			−0.04350287		
2009	−38.89595075			−0.049687678		
2010	−40.88541794			−0.05580858		
2011	−42.40030581			−0.059918379		
2012	−43.50301424			−0.062066921		
2013	−44.63873922			−0.063377562		
2014	−45.69152752			−0.064888704		
2015	−46.60152317			−0.065558859		
2016	−47.4187693			−0.066954047		
2017	−48.46510677			−0.070289626		
2018	−49.54013589			−0.072563481		
2019	−50.51644723			−0.073365084		
2020	−50.01731599			−0.068526537		
2021	−51.16429228			−0.067724025		
2022	−52.22821162	−53.31987	−51.13655646	−0.068803458	−0.073989173	−0.063617742
2023	−53.21508847	−55.58566	−50.84451501	−0.070444094	−0.078941921	−0.061946267
2024	−54.13050179	−57.98832	−50.27268683	−0.072252084	−0.083385642	−0.061118527
2025	−54.97962652	−60.47584	−49.48341148	−0.07410998	−0.087440261	−0.060779699
2026	−55.76726287	−63.01397	−48.52055306	−0.075982758	−0.091215605	−0.060749911
2027	−56.49786345	−65.57885	−47.41687708	−0.077859974	−0.094788154	−0.060931793
2028	−57.17555844	−68.15318	−46.19793345	−0.079738513	−0.09820851	−0.061268515
2029	−57.80417893	−70.72413	−44.88423252	−0.081617446	−0.101510521	−0.061724372
2030	−58.38727861	−73.28198	−43.49257577	−0.083496498	−0.104717544	−0.062275451
2031	−58.9281538	−75.81938	−42.03692849	−0.085375584	−0.10784629	−0.062904878
2032	−59.42986214	−78.3307	−40.52902396	−0.087254681	−0.110909129	−0.063600234
2033	−59.89523985	−80.81168	−38.97879991	−0.089133781	−0.113915493	−0.064352069
2034	−60.32691779	−83.25911	−37.39472467	−0.091012882	−0.116872768	−0.065152997
2035	−60.72733626	−85.67062	−35.78404768	−0.092891984	−0.119786855	−0.065997112
2036	−61.0987589	−88.04452	−34.15299617	−0.094771085	−0.122662562	−0.066879607

SII, Slope index of inequality; RCI, Relative concentration index.

## Discussion

In 2021, the ASIR and ASYR were predominantly high among older individuals, children and adolescents. Conversely, the older age group exhibited the lowest incidence of cases and YLDs. The ASDR for epilepsy of unknown cause generally rose with age from year to year and, like the ASIR and ASYR, was highest at >95 years. This was basically the same as the results of previous studies (7, 15–17). Elevated morbidity risks in children and adolescents, particularly neonates, are primarily linked to genetic factors, congenital brain malformations, and infections (18–20). In older age groups, increased morbidity and ASYR are often due to metabolic disorders, traumatic brain injuries, and strokes (1, 16). Both younger and older patient groups face significant challenges, including stigmatization and discrimination. Prolonged medication use exacerbates the psychological and life burdens for patients and their families (16, 21).

Globally, the number of deaths, morbidities, and YLDs linked to epilepsy of unknown cause has risen annually from 1990 to 2021. However, this increase has slowed over the last 4 years. Despite a decline in ASDR and ASYR from 2015 to 2020, ASIR has recently shown an upward trend. This suggests that the growth in these metrics may result from a combination of a larger and aging population base and an increased risk of morbidity despite improvements in medical care and health awareness (1, 7, 22, 23). The rising ASIR is particularly concerning, potentially reflecting more frequent patient visits and diagnoses due to improved diagnostic capabilities and greater health awareness, coupled with an increase in risk factors such as stressful lifestyles, drinking habits, and an aging population (1, 15, 23). Men have consistently been at higher risk than women for all indicators, a trend supported by other studies (7, 24). The reasons behind this gender disparity remain elusive, potentially tied to differences in neurodevelopment, hormone levels, genetics, and exposure to risk factors (25, 26).

A notable rise in ASIR was particularly evident in 2020–2021, a period marked by the global outbreak of the novel coronavirus. Recent studies suggest a possible link between novel coronavirus infection and the onset of new epilepsy cases or the exacerbation of symptoms in existing patients (27), which may explain the unusual trends in epilepsy burden during this period. A meta-analysis confirmed that vaccination against the novel coronavirus does not induce seizures (28). Thus, moving forward, it is imperative to enhance vaccination rates, increase public awareness about epilepsy risk factors, and implement additional preventative measures, especially among the more vulnerable younger and older male populations, emphasizing the need for heightened societal attention.

We find that ASYR negatively correlates with SDI and that the YLD burden is more concentrated in low and medium SDI areas. Contrary to earlier cross-national analyses of epilepsy of unknown cause DALYs (7), our study suggests that health inequalities in YLDs have worsened in 2021 compared to 1990, indicating a more concentrated burden among lower socioeconomic groups. Conversely, inequalities in Years of Life Lost (YLLs) due to early deaths may have diminished, pointing to an evolving landscape of health disparities influenced by both societal progress and persistent challenges. We applied ARIMA predictive modeling to the SII and RCI metrics and then evaluated the models extensively. The results demonstrate the effectiveness of the models in predicting outcomes; however, the application of ARIMA modeling falls short of accurately capturing the long-term trend. Our projections for CI and SII show a downward trend in both SII and CI in the future, suggesting that the unequal distribution of YLD in epilepsy of unknown cause will continue to increase.

In the future, YLD in epilepsy of unknown cause will be more concentrated in low SDI regions, and the health resource gap will require a concerted global effort to reduce it. Of course, this finding may also be related to potential reporting bias in low SDI countries and variations in diagnostic criteria across regions and over time. The persistence of treatment gaps, particularly in low SDI regions, is exacerbated by the tendency of public health administrations to overlook epilepsy, coupled with widespread ignorance about the condition (22, 29, 30).

Ethiopia will continue to experience an increase in ASYR (Both: *R*^2^: 0.69; MaxRD: 1.2%, Female: *R*^2^: 0.63; MaxRD: 3.5%, Male: *R*^2^: 0.73; MaxRD: 1.3%) in the future. The prevalence of poor quality of life was higher in Ethiopian patients with epilepsy (45.07, 95% CI: 39.73–50.42%), and illiteracy, anxiety and depression were significantly associated with quality of life in patients with epilepsy (31). The burden of medication non-adherence among Ethiopians with epilepsy is also high and is associated with anxiety, depression and stigma (32, 33). This psychological state is associated with poor understanding and perception of epilepsy and unfavorable attitudes toward people with epilepsy among the majority of the Ethiopian population (34, 35). The issue of the cost of medicines is also linked to patient non-adherence (36). To address the above situation, on the one hand, we propose to follow China’s “band purchasing” experience and establish a drug purchasing alliance with representatives from various countries to reduce the unit price of drugs by expanding the market scale, and on the other hand, we can encourage drug companies to adopt a tiered pricing strategy, maintaining profit revenue in high SDI countries and supplying drugs at cost in low SDI countries, and subsidizing drug companies with the international health insurance fund, and use international health insurance funds to subsidize drug companies. The stigmatization of epilepsy of unknown cause in low SDI regions can be addressed by increasing knowledge about epilepsy in schools, large corporations, and government employees.

Our analysis revealed a previously undescribed weak positive correlation between ASIR and SDI, challenging prior assertions (7, 37). This finding suggests a complex scenario for the diagnosis of epilepsy of unknown cause in low SDI regions and potentially underestimates the actual burden (38, 39). When analyzing the projections of future ASIR, we found that most countries or regions (71.04%) show an increasing trend. There is a severe shortage of neurologists and neurophysiologists in resource-limited countries or regions, and the lack of diagnostic equipment, such as EEGs in primary healthcare facilities, contributes to the high rate of underdiagnosis of idiopathic epilepsies in low SDI regions (40). We recommend scientific and systematic training of primary care physicians and the use of tele-digital medicine to provide epilepsy screening to increase the treatment rate of patients with epilepsy and to reduce the rate of misdiagnosis and underdiagnosis. Destigmatization and improved diagnostic and treatment technologies will lead to higher patient attendance, lower misdiagnosis and underdiagnosis and, thus, higher ASIR in epilepsy of unknown cause. For example, in Pakistan, the local government has initiated a program in recent years to reduce disparities and stigma in epilepsy treatment, which may contribute to a future increase in ASIR (Both: *R*^2^: 0.75; MaxRD: 13.7%, Female: *R*^2^: 0.19; MaxRD: 4.4%, Male: *R*^2^: 0.83; MaxRD: 29.9%) for epilepsy of unknown cause in Pakistan (41). Industrialization in low-income countries is accompanied by unchecked environmental pollution, which exposes people to a variety of neurotoxic substances and thus increases the incidence of epilepsy (42, 43), for example high levels of lead contamination in Zambia and high blood lead levels in children increase the incidence of epilepsy in local children (44). In areas with a high SDI, changes in the lifestyle of people under high stress, such as sleep deprivation, more frequent exposure to light stimulation and high-sugar diets, can increase the risk of seizures (45–47). We recommend that a calibrated model of the ASIR for epilepsy of unknown cause be established to distinguish between true increases in incidence and data bias due to improved diagnostic capabilities, that various neurotoxic substances be closely monitored and promptly managed, and that intervenable risk factors for seizures be publicized and controlled in high-risk populations, such as reducing alcohol consumption and maintaining physical and mental relaxation (48).

Through BAPC modeling and correlation analysis, we found that most countries and regions (75.41%) showed a decreasing trend in ASDR and that ASDR was negatively correlated with SDI levels. Advances in epigenetic technology have provided new tools for the prevention of epilepsy and the definitive diagnosis of refractory epilepsy (49), and advances in epilepsy surgery techniques, including the use of brain-computer interfaces, have made cures for refractory epilepsy more likely (50). The increasing standardization of epilepsy care and the availability of a large number of generic versions of epilepsy drugs after patent expiry have led to further improvements in epilepsy care in low- and middle-income countries or regions, all of which have reduced the likelihood of death (51).

The burden of epilepsy of unknown cause is generally higher in men than in women; however, certain countries or regions exhibit a more significant disease burden in women. Additionally, there are areas where the burden in women may surpass that in men in the future. Men head most families in Pakistan, and women are less likely to receive support from their families and marry earlier, making the female population more exposed to seizure risk factors and less likely to receive proper medical care (52). It is essential for these regions to focus on female populations, address misconceptions and discrimination surrounding epilepsy, and ensure equitable access to healthcare services for both men and women. We recommend that these countries undertake community activities and establish female role models to reduce discrimination against women. In health care, establishing green lanes for women’s health care and women’s health insurance funds will further protect women’s rights and interests in accessing health care.

This study leveraged the latest GBD database data to innovatively visualize and predict trends in health inequalities related to YLDs from epilepsy of unknown cause over the next 15 years. We conducted BAPC prediction modeling for 21 regions and 162 countries to elucidate the disparities in mortality, morbidity, and YLDs. This analysis aims to assist policymakers in comprehending the variations in the burden of epilepsy across regions and countries with differing levels of social development.

Although the BAPC model has been widely used in the field of disease burden prediction, this study found significant heterogeneity in its predictive efficacy in cross-country comparisons. BAPC has shown high accuracy in countries with good health data infrastructure and abundant case numbers (for example, Germany and the United Kingdom). However, it has wide prediction intervals and poor predictive efficacy in low and middle-income countries or countries with small populations. It is limited by underreporting diseases, incomplete data, and lagging demographic dynamics. For models with moderate predictive power (0.5 ≤ *R*^2^ ≤ 0.7) and *a priori* sensitivity suggesting that the model is more stable, this may be because the model does not fully capture the dynamic changes in the data and future research could improve the predictive power by incorporating covariates such as socio-economic or policy changes. In contrast, for unstable models, a hierarchical model can be used to reduce parameter uncertainty. For models with poor predictive ability (*R*^2^ < 0.5), other modeling methods should be tried, such as ARIMA models, machine learning, etc. For models with good but unstable predictive power, we provide visualizations of posterior distributions from *a priori* sensitivity analyses (Supplementary Figures 6, 7). We can initially determine the sensitivity of the prior for different effects by the degree of dispersion of the curves; the higher the degree of dispersion, the more sensitive it is. For age effect sensitivity, we suggest merging age groups to reduce data sparsity for remodeling. For period effect sensitivity, we suggest a priori remodeling or adding dummy variables to control for confounding events. The model can be simplified for cohort effect sensitivity by removing cohort effects. The overdispersion effect is sensitive, suggesting that the model may be missing important variables and that random effects need to be added to refine the model. A more detailed reading of the posterior distribution can be found in a previous article (53). After comprehensive analysis, the model a priori level can be adjusted and the model can be optimized by dealing with anomalously sensitive data. Although the absolute values of the individual country projections must be interpreted with caution, the relative trends can still be used to prioritize regional health resource allocation, particularly in allocating budgets for the purchase of antiepileptic drugs and in planning the training of primary neurologists, which has an early warning value. In addition, we find that the long-term results of the BAPC forecasting model are uncertain. Policymakers should be more cautious about the long-term forecast results and focus on the forecast results in the last 5–10 years, where timely policy interventions and changes in the world economy may change them.

Suppose further information is needed about future trends in the burden of disease for epilepsy of unknown cause in countries with poor BAPC model predictions. In that case, the calibration results of the BAPC model can be interpreted according to our previous tips. The choice can be made to either improve the BAPC model or abandon the BAPC model and select another model for predictive analysis. Currently, clinicians may pay more attention to the burden of disease for different causes of death in epilepsy of unknown cause, which may lead clinicians to pay more attention to the main causes of death in patients with epilepsy of unknown cause. Therefore, future studies could follow the example of the current study on the burden of disease in stroke and analyze in more detail the description of the burden of ASDR in patients with epilepsy attributed to different causes. While the GBD’s imputation methods provide critical estimates for under-resourced regions, their reliance on cross-regional extrapolation may obscure local contextual drivers of disease. We acknowledge that uncertainty intervals for these estimates are typically wider, reflecting limited primary data availability.

## Conclusion

Our study underscores the troubling forecast that health inequalities in YLDs associated with epilepsy of unknown cause are likely to intensify in the coming years. Policymakers are urged to implement targeted interventions aimed at narrowing the disparities in quality of life between patients from varied social and economic backgrounds.

Future policy efforts should concentrate on these dynamics, ensuring that strategies are comprehensive and inclusive, thereby enhancing the overall management and support structures for individuals affected by epilepsy of unknown cause. By acknowledging and addressing these disparities, we can move toward a more equitable health landscape where the burden of epilepsy is more evenly distributed across all segments of society.

## Data Availability

The original contributions presented in the study are included in the article/Supplementary material, further inquiries can be directed to the corresponding authors.

## Footnotes

^1^ http://ghdx.healthdata.org
