# Correction: Global, regional, and national epilepsy of unknown cause incidence and mortality, 1990–2036: cross-national health inequalities and predictive analytics

**DOI:** 10.3389/fneur.2025.1679359

**Published:** 2025-08-29

**Authors:** Yi Wang, Yichen Wang, Xinyu Qiu, Zhenjie Yu, Lijun Wang

**Affiliations:** ^1^Tianjin Medical University, Tianjin, China; ^2^Tianjin University, Tianjin, China; ^3^Department of Infectious Diseases and Public Health, Jockey Club College of Veterinary Medicine and Life Sciences, City University of Hong Kong, Kowloon, Hong Kong SAR, China; ^4^Tianjin Fourth Central Hospital Affiliated to Tianjin Medical University, Tianjin, China

**Keywords:** global burden disease, epliepsy, epilepsy of unknown cause, YLD, years lived with a disability, health inequalities analysis, predictive analytics, BAPC prediction model

In the published article, there was a mistake in the order of authors in the author list. The order of authors in the author list of the published paper was erroneously given as: “Lijun Wang^*^, Zhenjie Yu^*^, Yi Wang, Yichen Wang, Xinyu Qiu”. The correct author list reads: “Yi Wang^†^, Yichen Wang^†^, Xinyu Qiu, Zhenjie Yu^*^, Lijun Wang^*^.

The following was omitted from the published article:

^†^These authors have contributed equally to this work

The original version of this article has been updated.

