# Correction: Novice teachers' emotional labor: a study of volunteer Chinese teachers in African Confucius Institutes

**DOI:** 10.3389/fpsyg.2025.1724863

**Published:** 2025-10-27

**Authors:** Mengying Wu, Wenle Yan

**Affiliations:** Research Institute for International and Comparative Education, Shanghai Normal University, Shanghai, China

**Keywords:** emotional rules, emotional labor strategies, volunteer Chinese teachers, Africa, novice teachers

In the published article, there was an error in the Funding statement. The article incorrectly stated: “The author(s) declare that financial support was received for the research and/or publication of this article. This research was supported by the General Project of Humanities and Social Sciences of the Ministry of Education of China (2025): ‘Research on International Pathways and Chinese Construction of Public Diplomacy in Basic Education for Enhancing National Image'.” The correct Funding statement appears below.

