# Supplementary material for: Global, regional, and national epilepsy of unknown cause incidence and mortality, 1990–2036: cross-national health inequalities and predictive analytics
Source: Front Neurol. 2025 Jun 30;16:1526984. doi: 10.3389/fneur.2025.1526984 (PMC12256229; doi:10.3389/fneur.2025.1526984)
Supplement: Supplementary file 1 [file Data_Sheet_1.pdf]

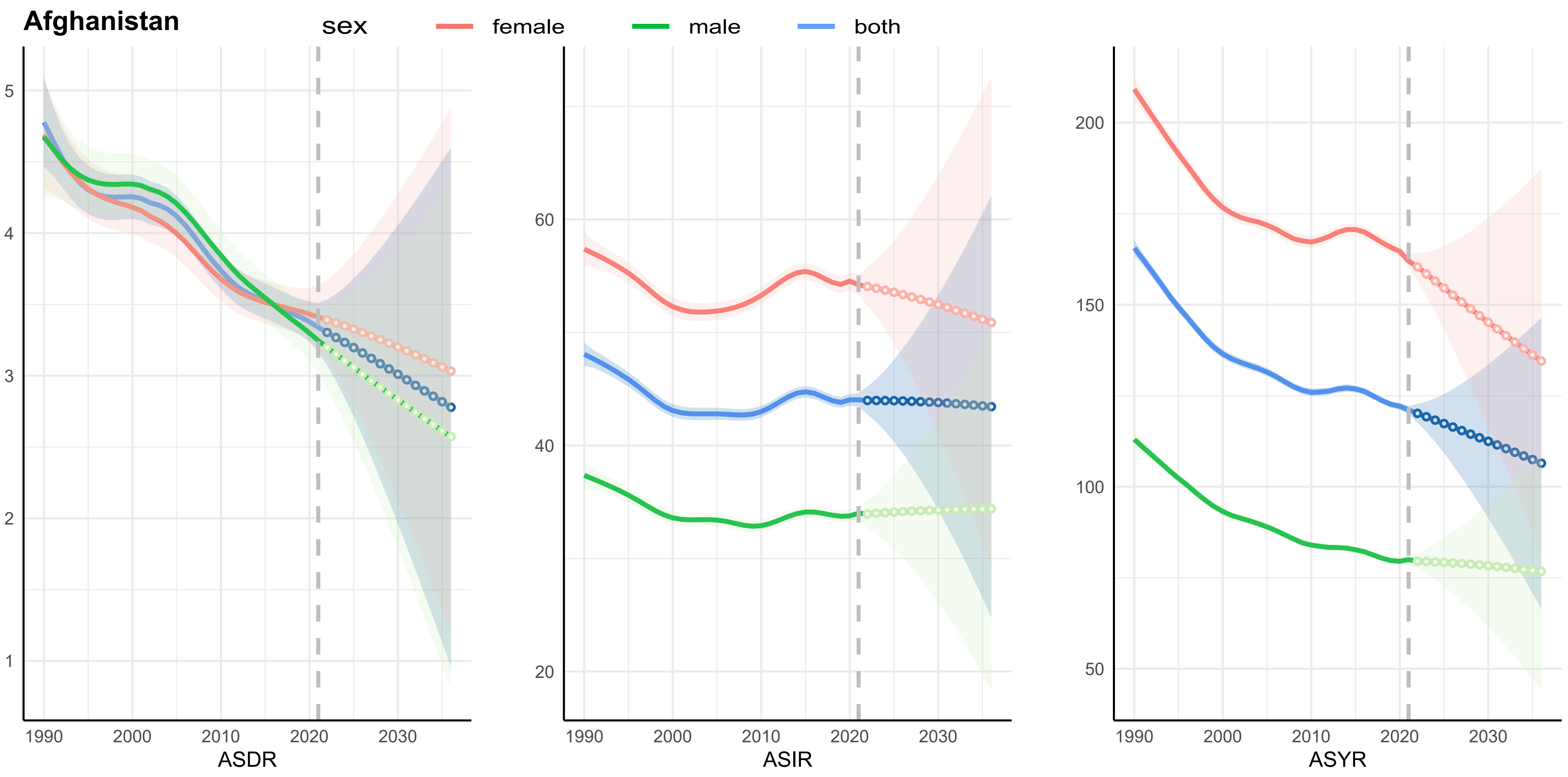

Afghanistan
sex
female
male
both
5
4
3
2
1
1990
2000
2010
2020
2030
ASDR
60
40
20
ASIR
200
150
100
50
ASYR

Albania

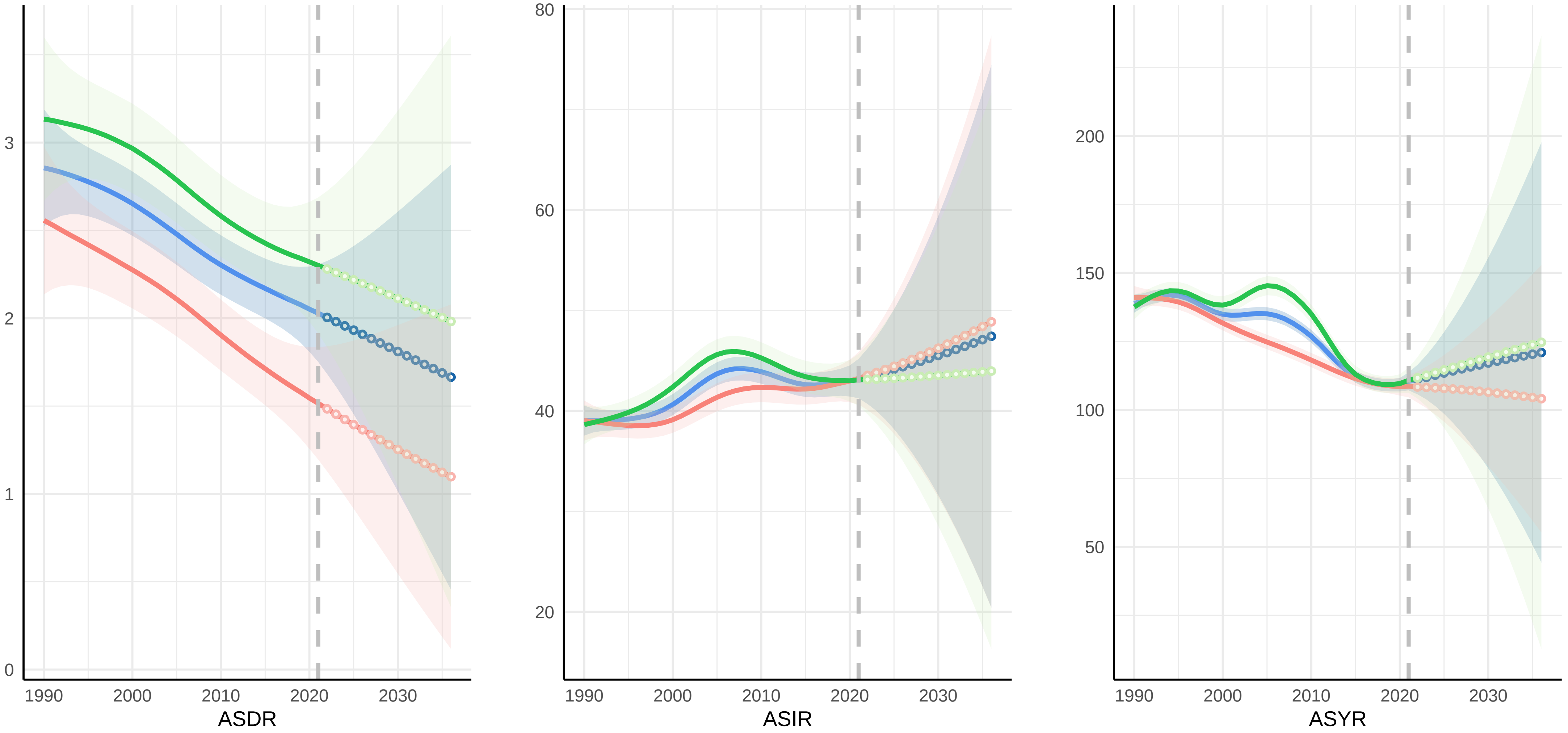

3
2
1
0
1990
2000
2010
2020
2030
ASDR
80
60
40
20
1990
2000
2010
2020
2030
ASIR
200
150
100
50
1990
2000
2010
2020
2030
ASYR

Algeria

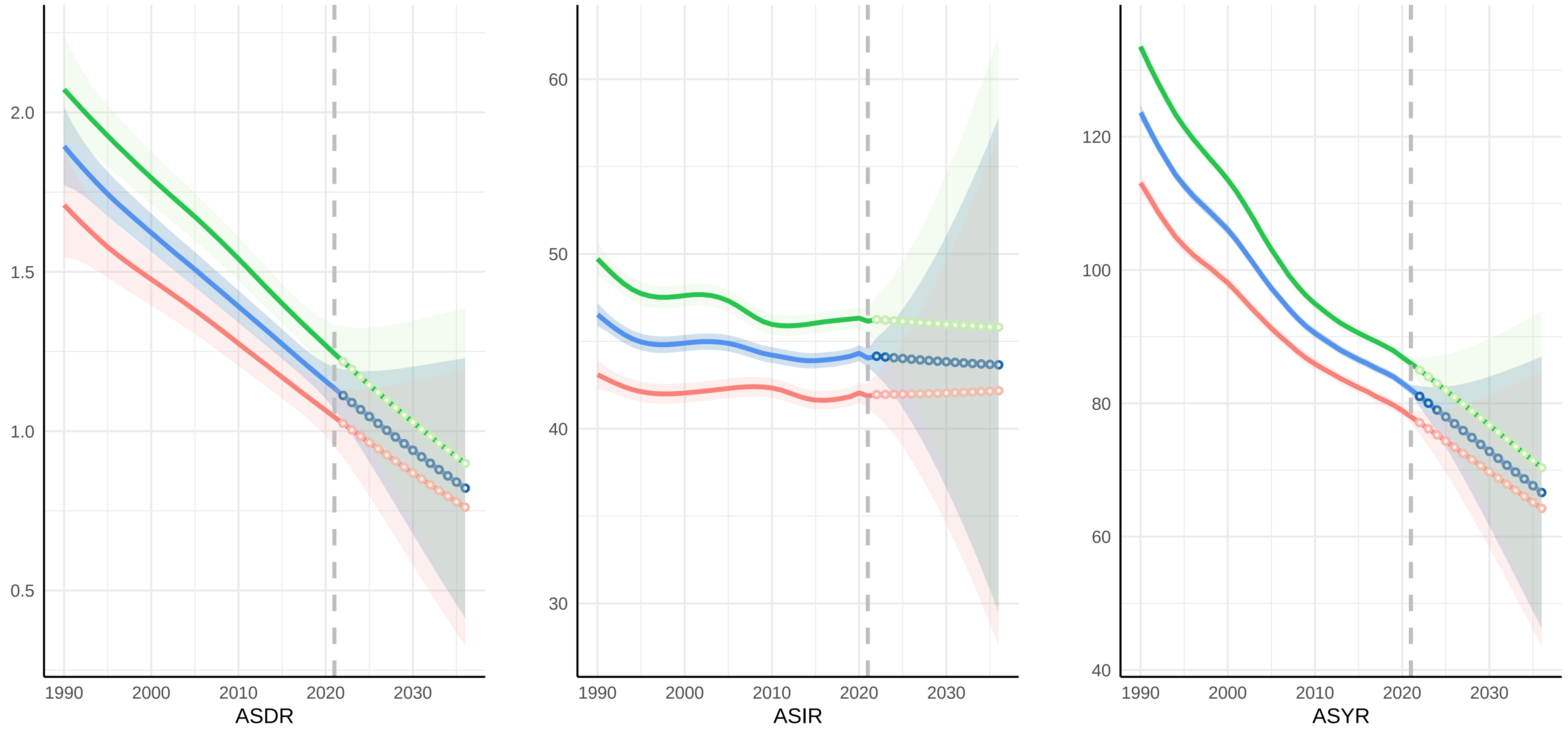

2.0
1.5
1.0
0.5
1990
2000
2010
2020
2030
ASDR
60
50
40
30
1990
2000
2010
2020
2030
ASIR
120
100
80
60
40
1990
2000
2010
2020
2030
ASYR

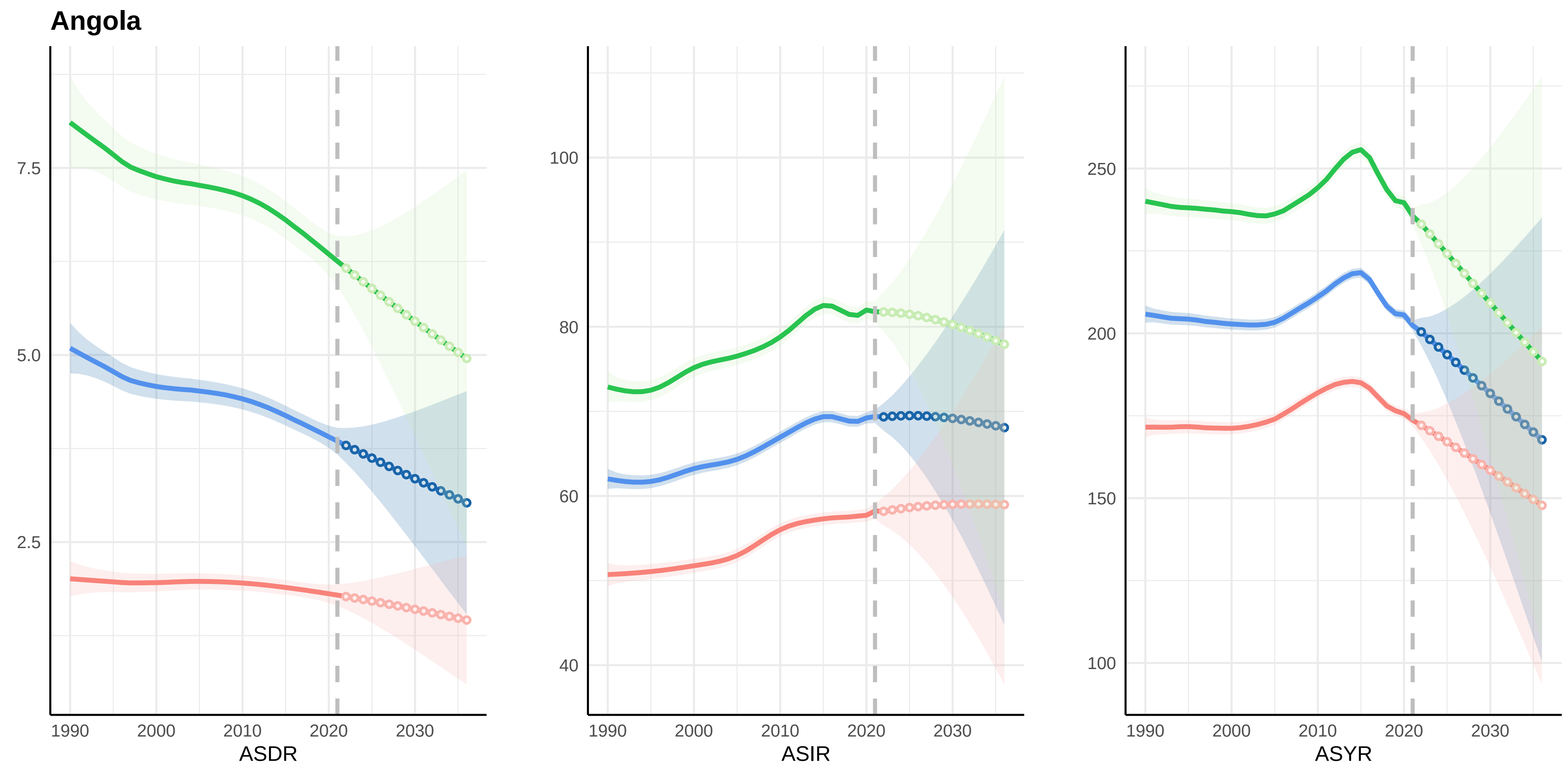
Angola
7.5
5.0
2.5
1990
2000
2010
2020
2030
ASDR
100
80
60
40
1990
2000
2010
2020
2030
ASIR
250
200
150
100
1990
2000
2010
2020
2030
ASYR

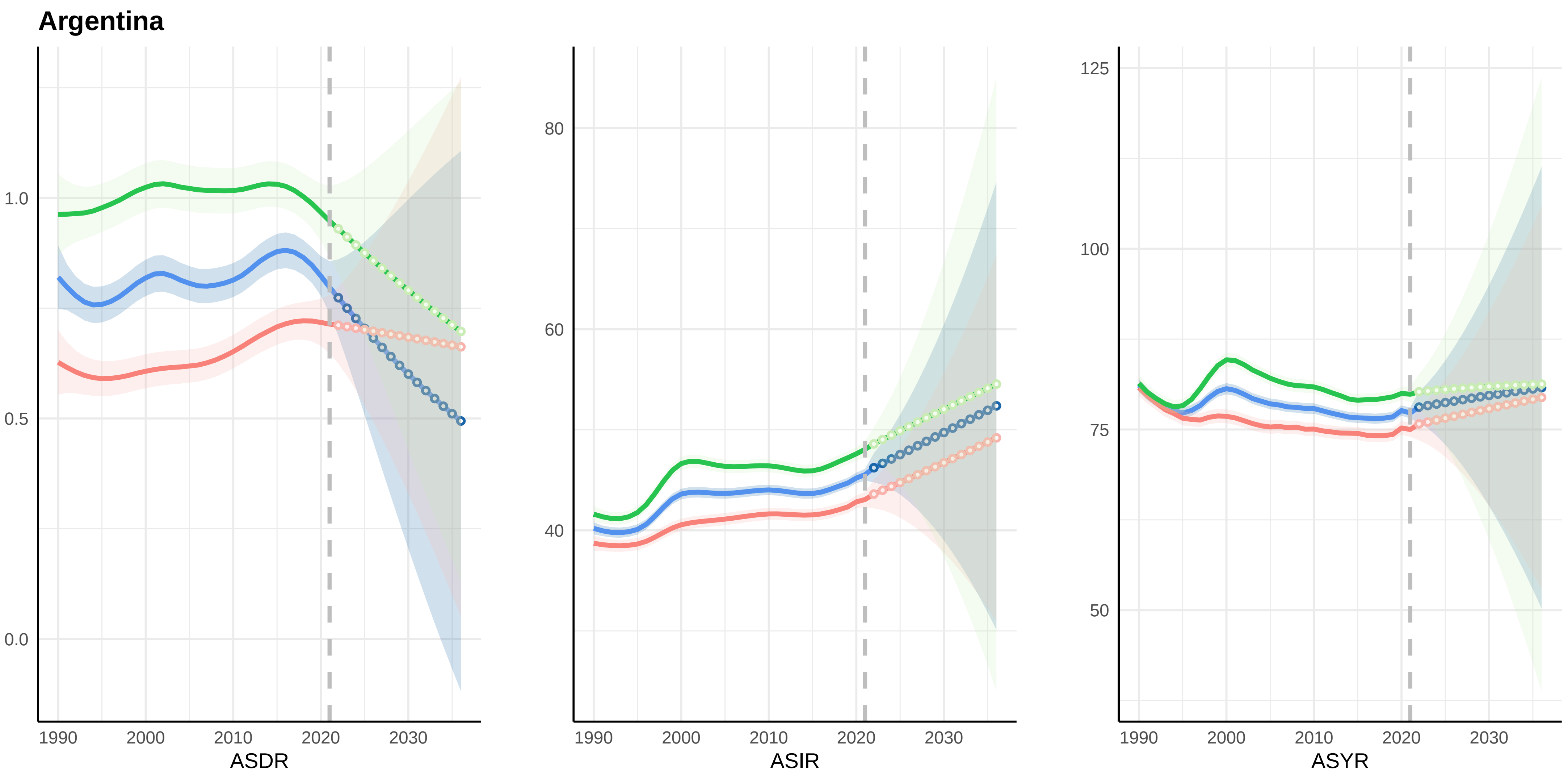

Argentina
1.0
0.5
0.0
1990
2000
2010
2020
2030
ASDR
80
60
40
1990
2000
2010
2020
2030
ASIR
125
100
75
50
1990
2000
2010
2020
2030
ASYR

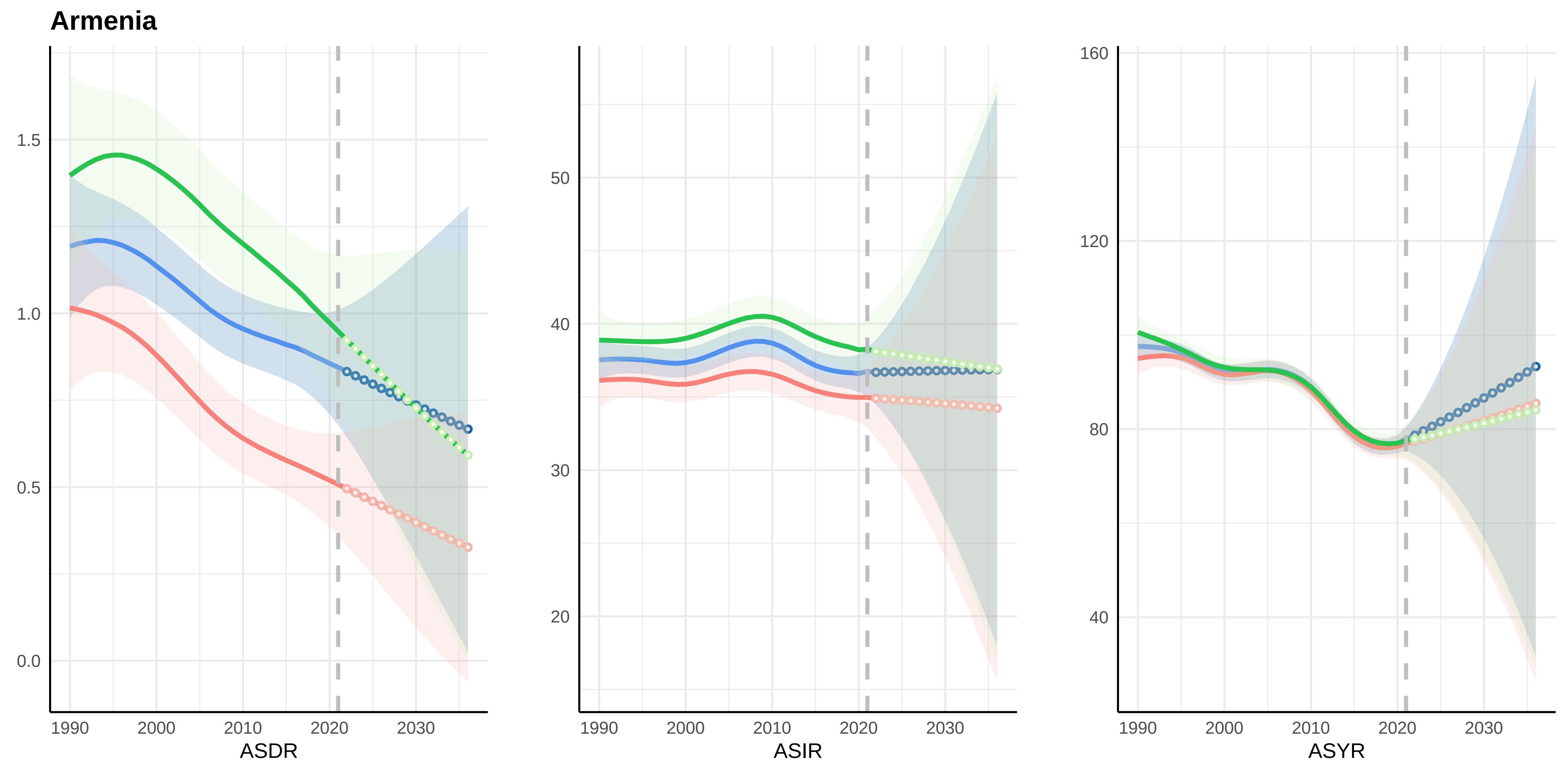

Armenia
1.5
1.0
0.5
0.0
1990
2000
2010
2020
2030
ASDR
50
40
30
20
1990
2000
2010
2020
2030
ASIR
160
120
80
40
1990
2000
2010
2020
2030
ASYR

Australia

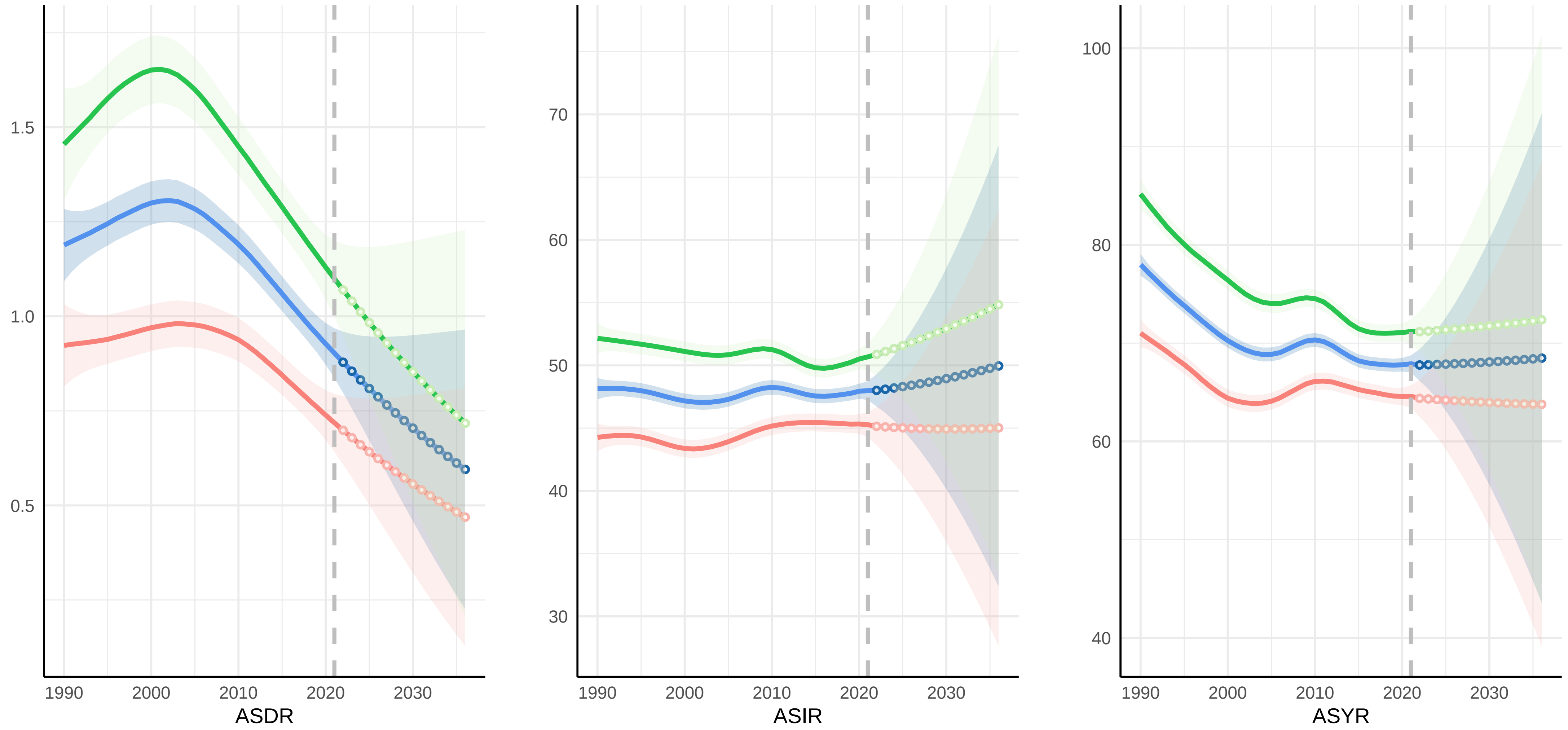

1.5
1.0
0.5
1990
2000
2010
2020
2030
ASDR
70
60
50
40
30
1990
2000
2010
2020
2030
ASIR
100
80
60
40
1990
2000
2010
2020
2030
ASYR

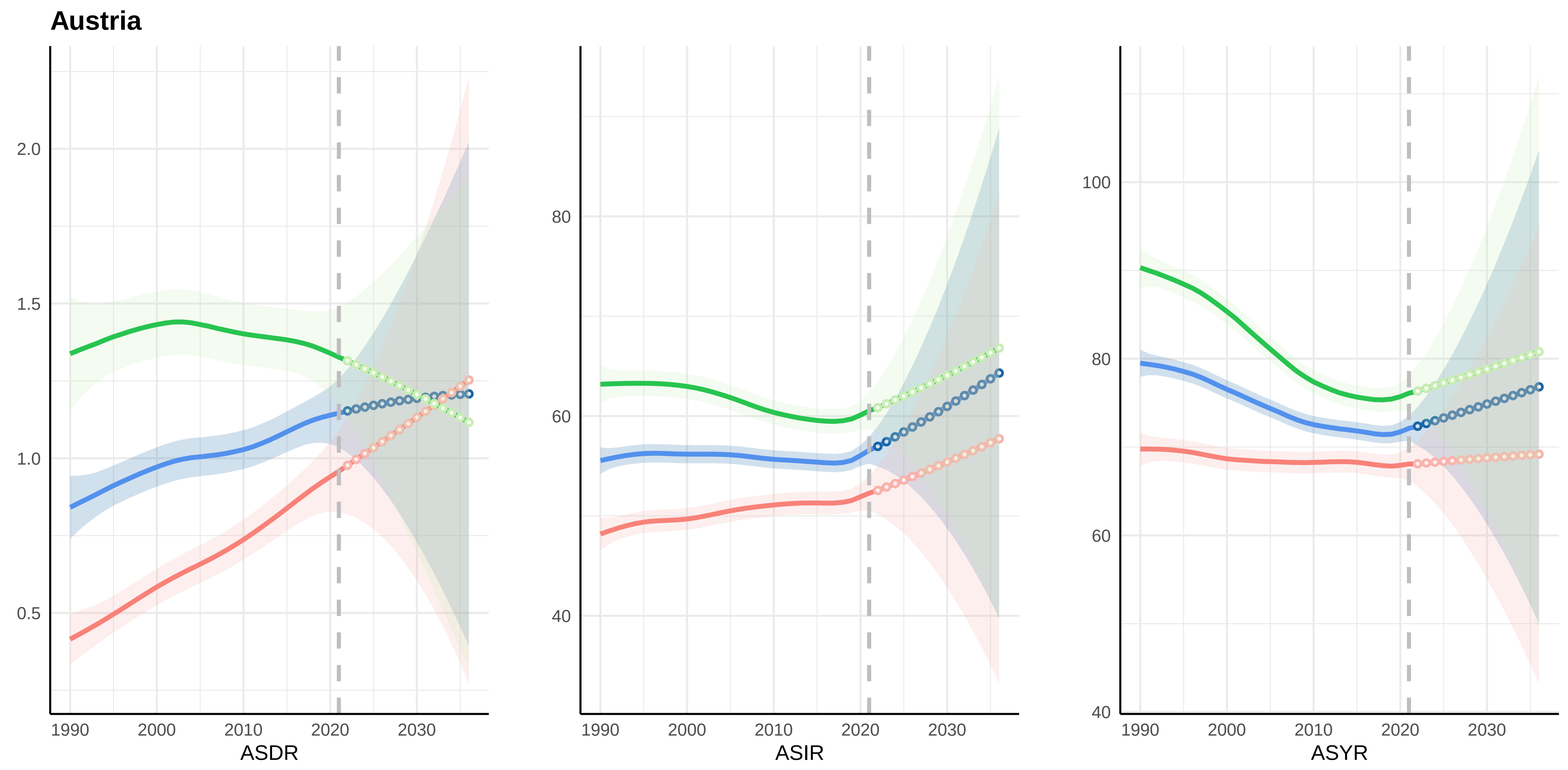

Austria
2.0
1.5
1.0
0.5
1990
2000
2010
2020
2030
ASDR
80
60
40
1990
2000
2010
2020
2030
ASIR
100
80
60
40
1990
2000
2010
2020
2030
ASYR

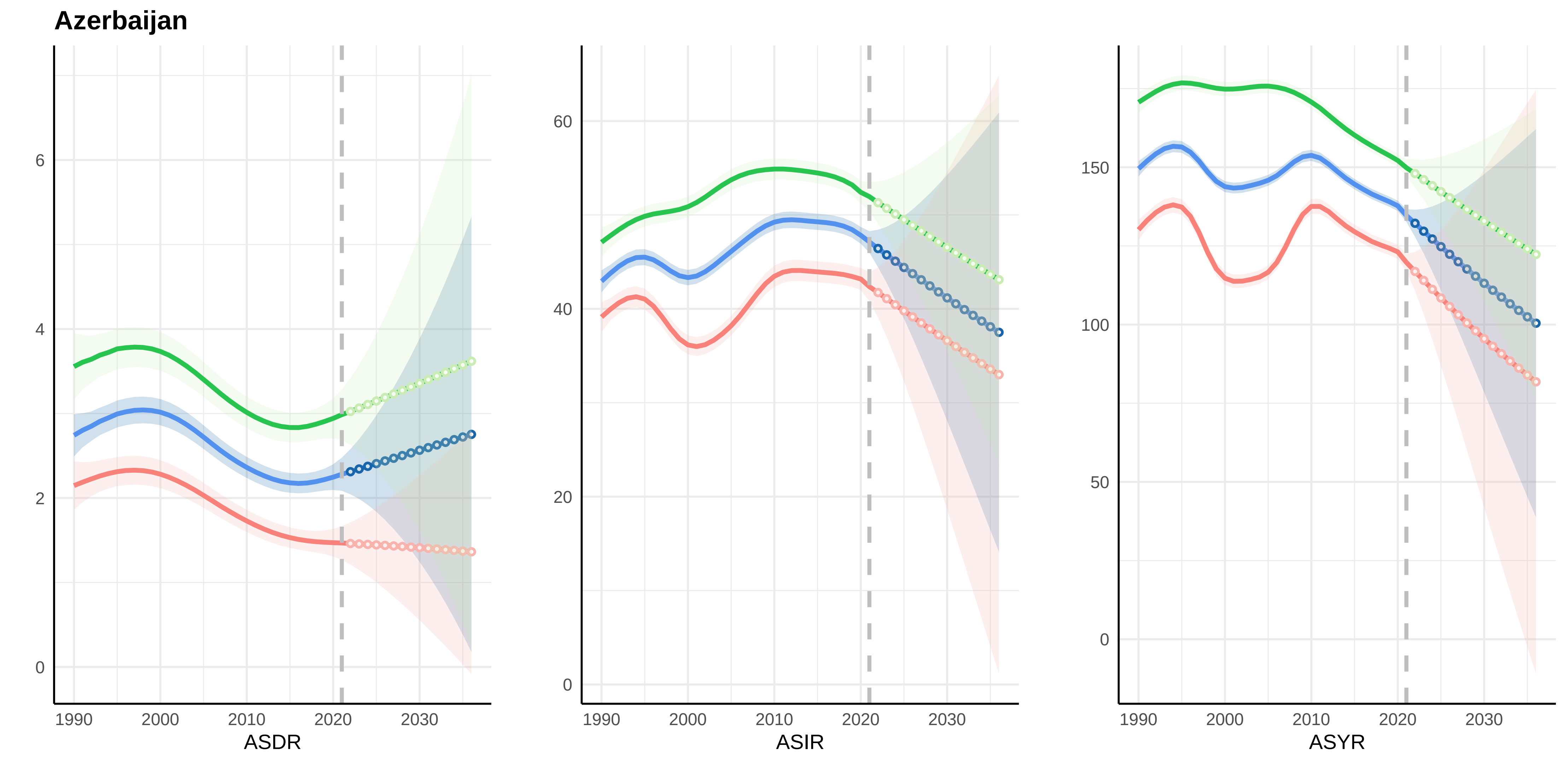

Azerbaijan
6
4
2
0
1990
2000
2010
2020
2030
ASDR
60
40
20
0
1990
2000
2010
2020
2030
ASIR
150
100
50
0
1990
2000
2010
2020
2030
ASYR

Bahrain

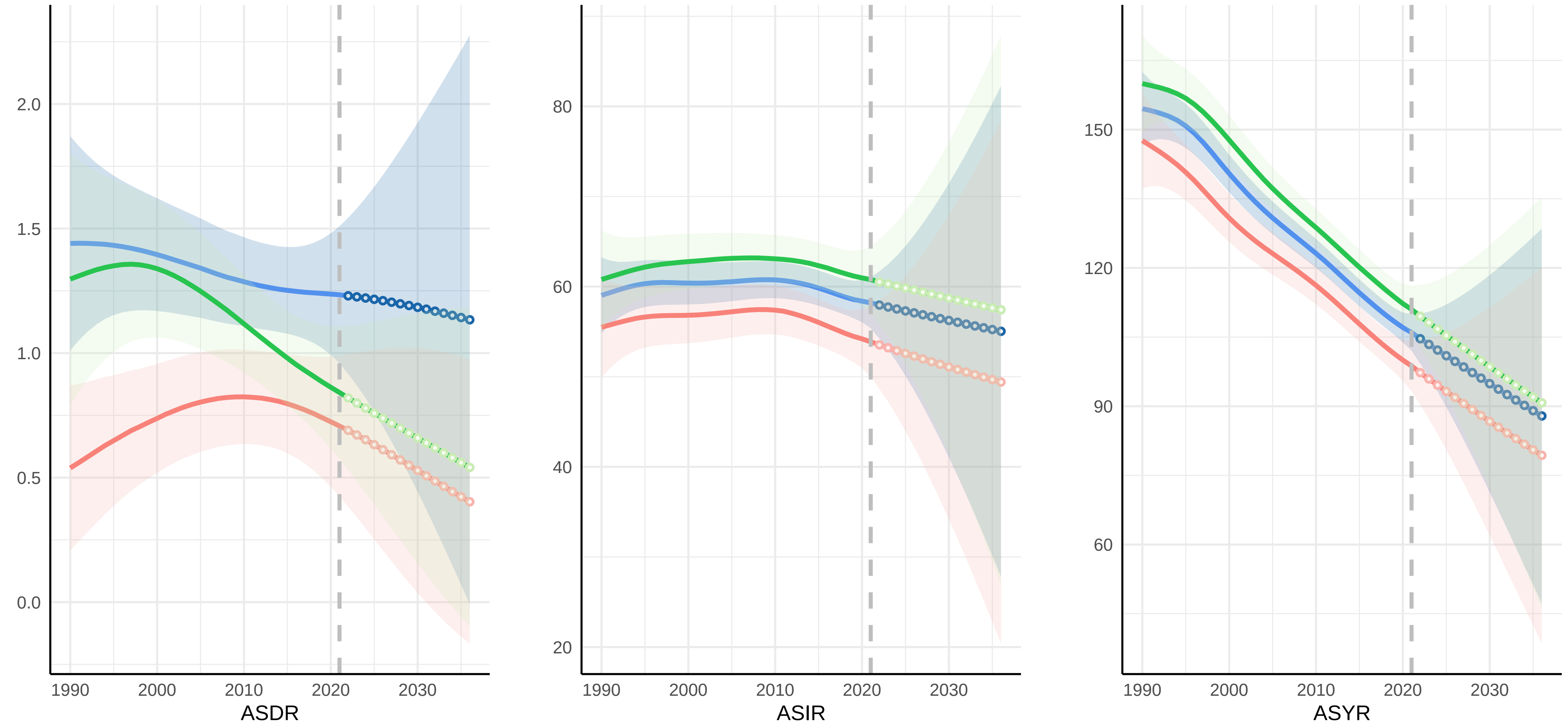

2.0
1.5
1.0
0.5
0.0
1990
2000
2010
2020
2030
ASDR
80
60
40
20
1990
2000
2010
2020
2030
ASIR
150
120
90
60
1990
2000
2010
2020
2030
ASYR

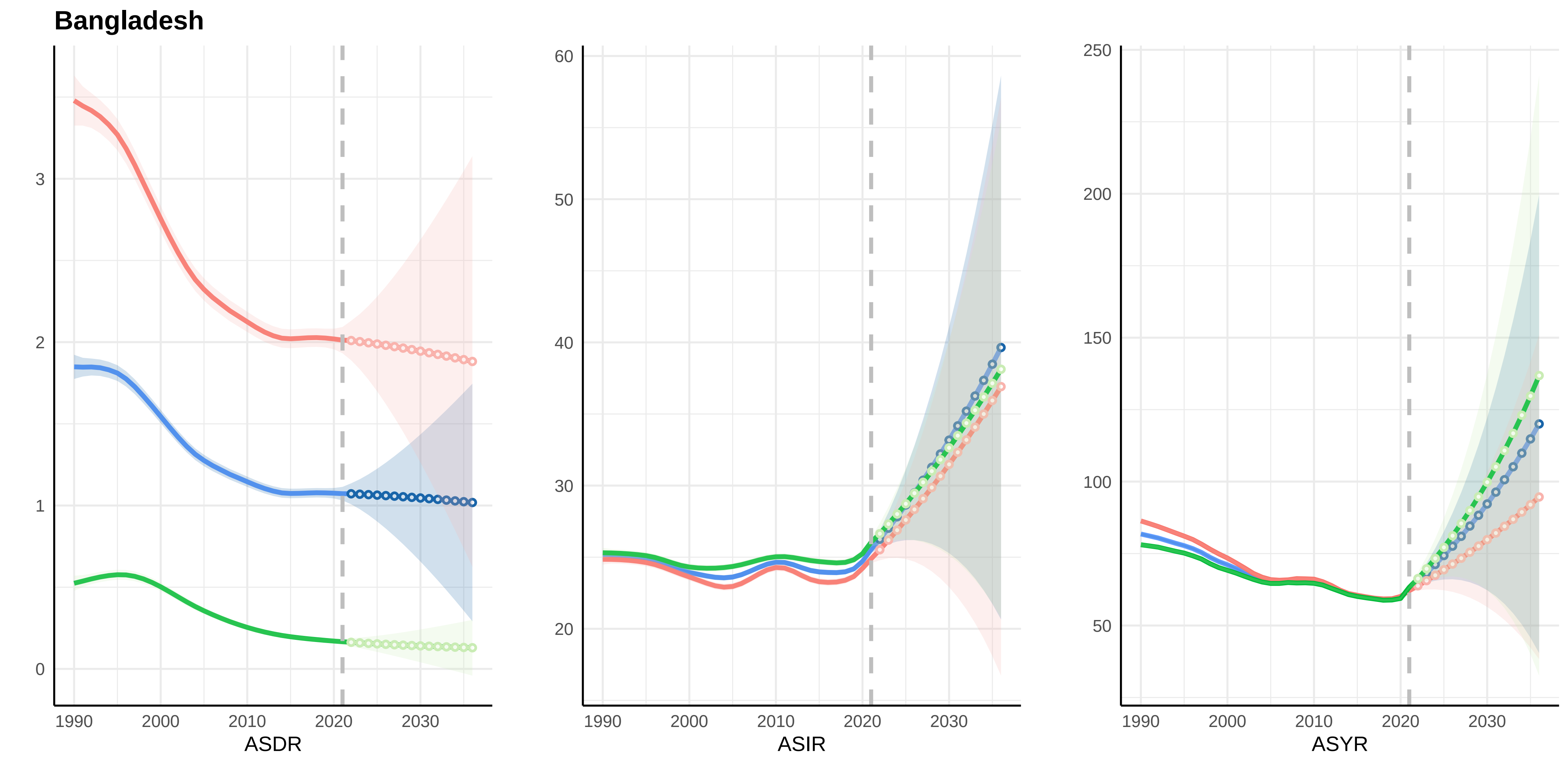
Bangladesh
3
2
1
0
1990
2000
2010
2020
2030
ASDR
60
50
40
30
20
1990
2000
2010
2020
2030
ASIR
250
200
150
100
50
1990
2000
2010
2020
2030
ASYR

Belarus

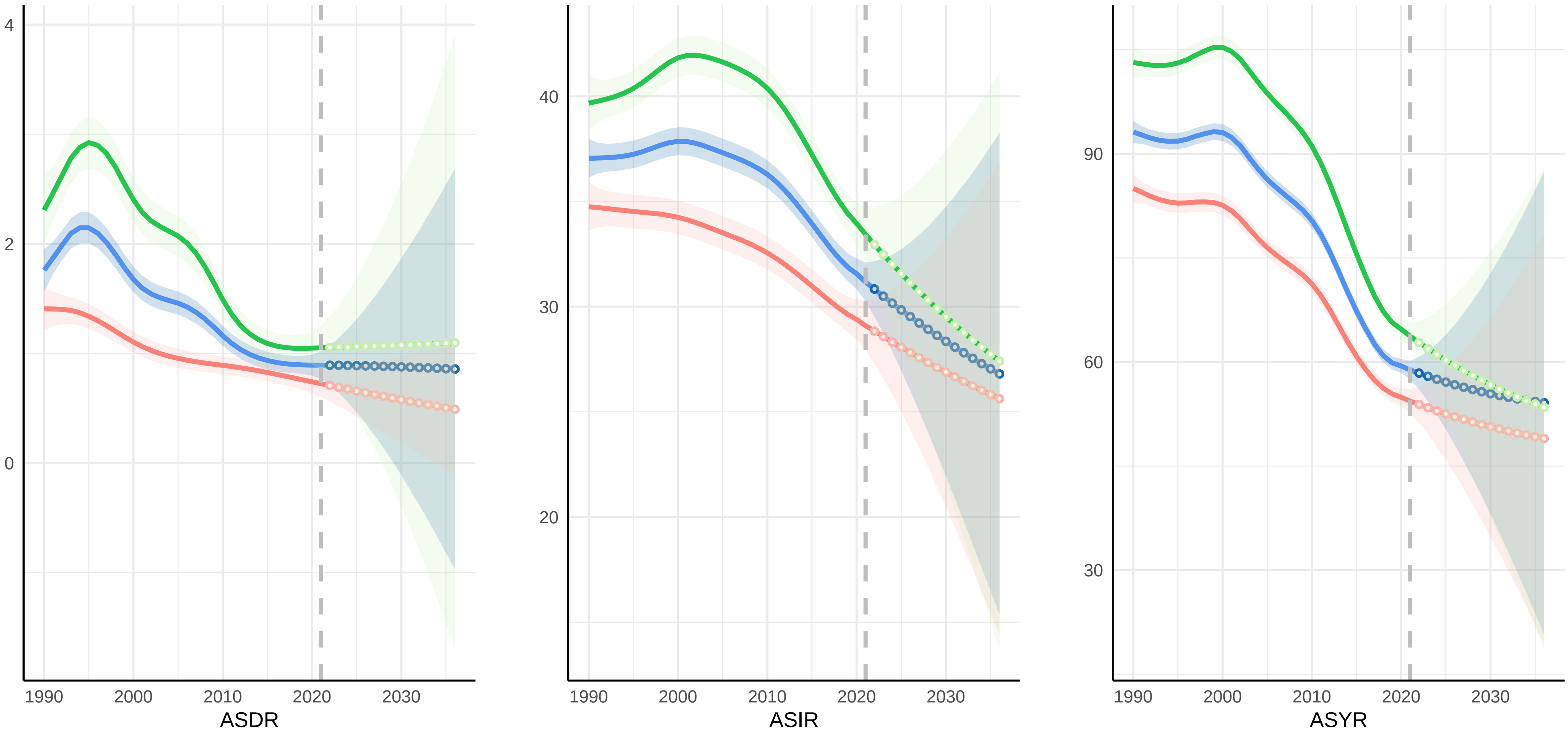
4
2
0
1990
2000
2010
2020
2030
ASDR
40
30
20
1990
2000
2010
2020
2030
ASIR
90
60
30
1990
2000
2010
2020
2030
ASYR

Belgium

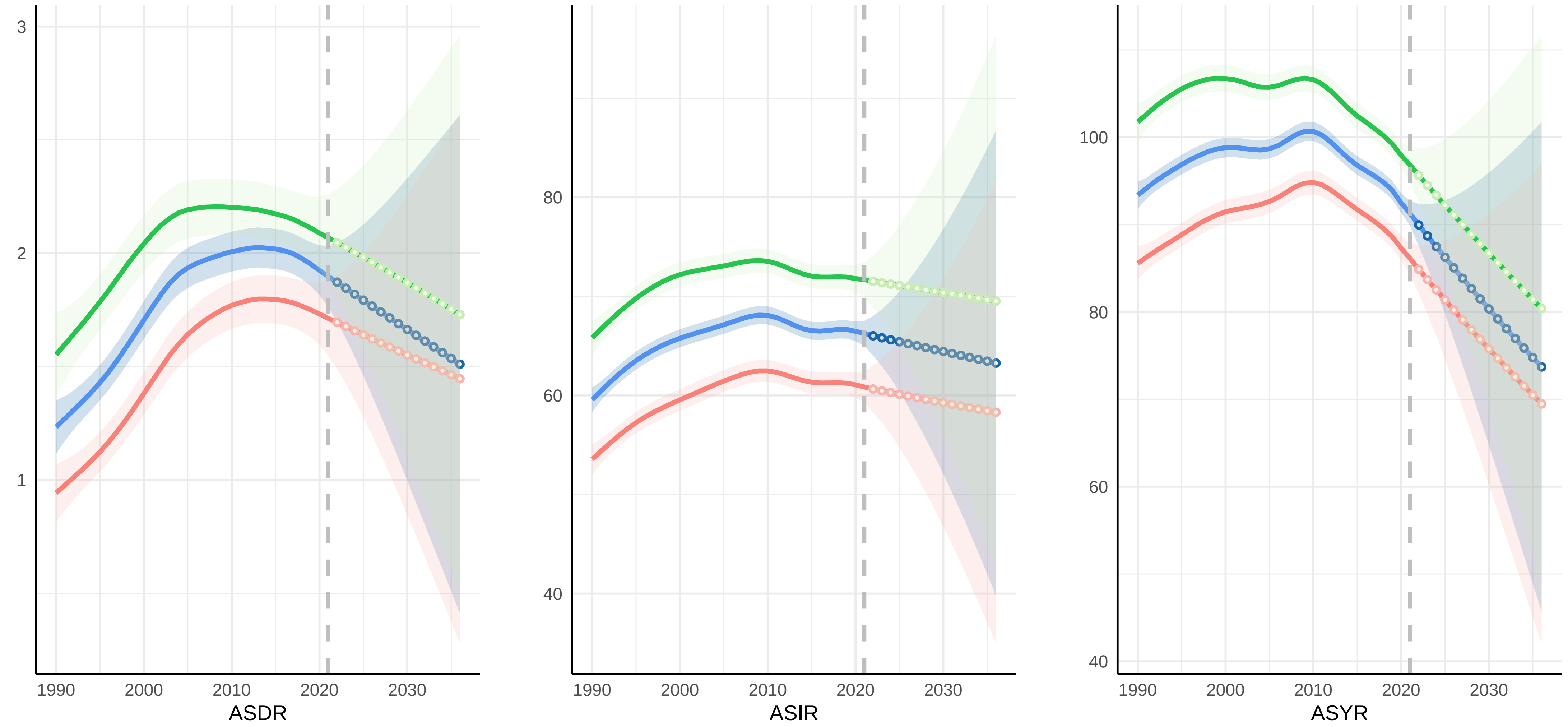

3
2
1
1990
2000
2010
2020
2030
ASDR
80
60
40
1990
2000
2010
2020
2030
ASIR
100
80
60
40
1990
2000
2010
2020
2030
ASYR

Benin

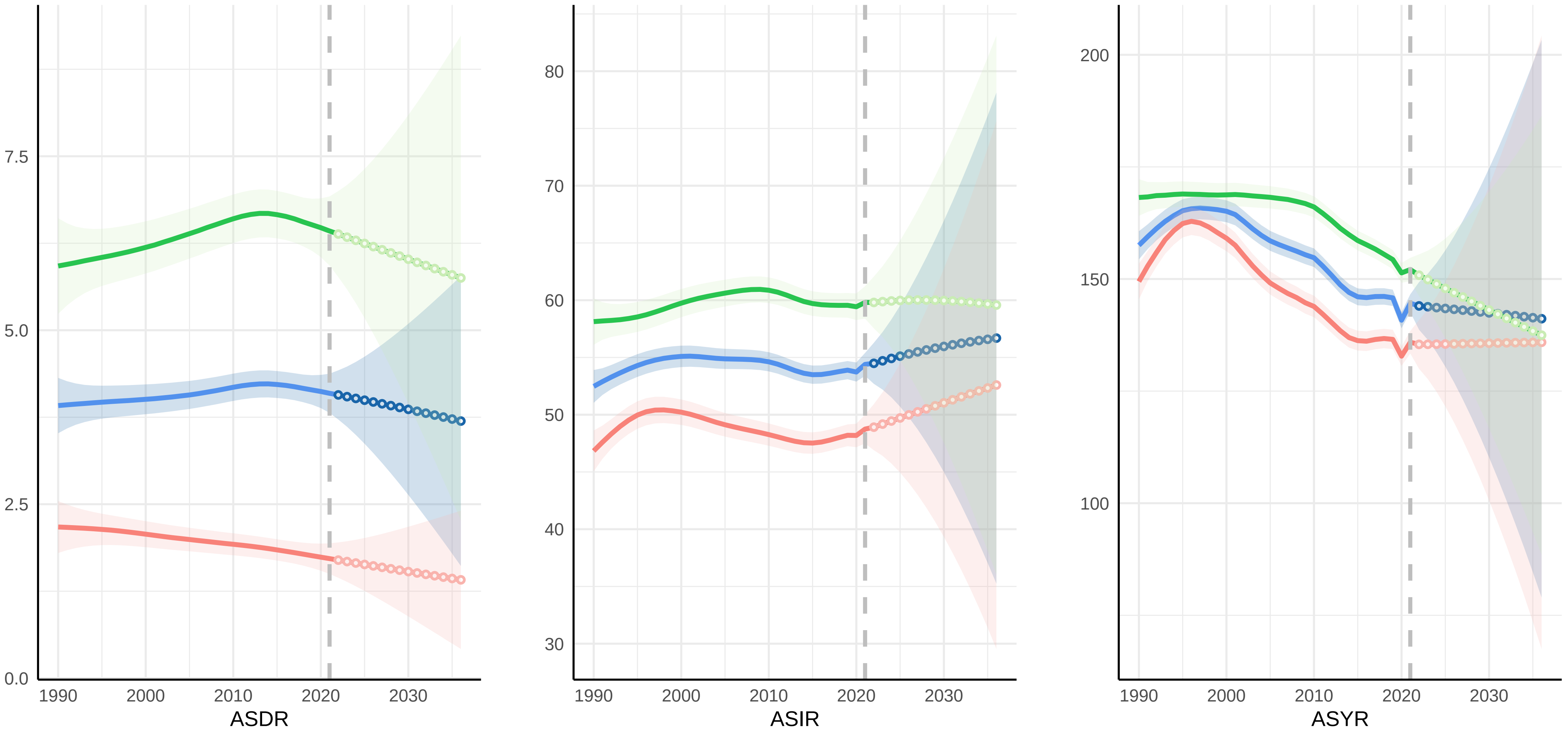

7.5
5.0
2.5
0.0
1990
2000
2010
2020
2030
ASDR
80
70
60
50
40
30
ASIR
200
150
100
ASYR

Bhutan

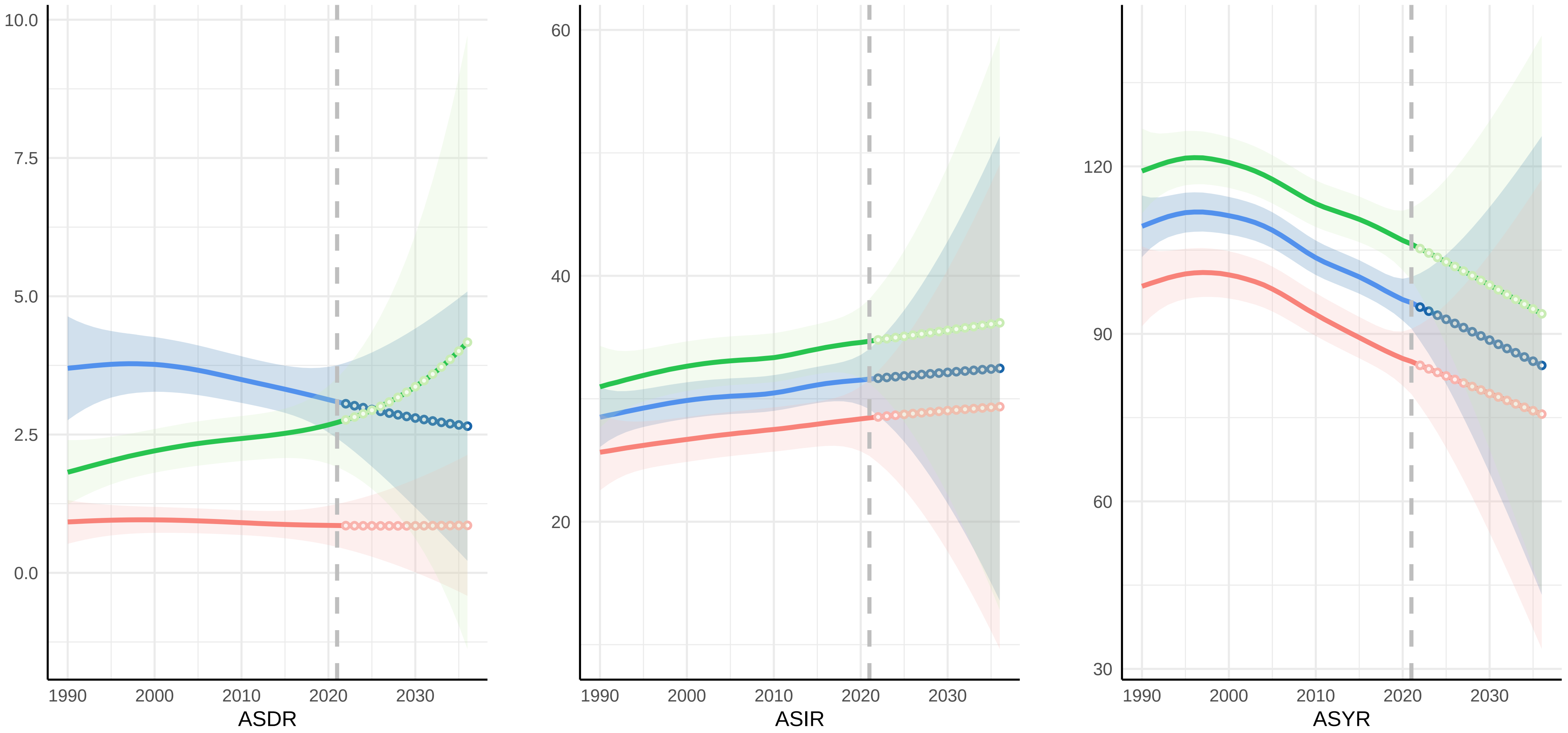

10.0
7.5
5.0
2.5
0.0
1990
2000
2010
2020
2030
ASDR
60
40
20
1990
2000
2010
2020
2030
ASIR
120
90
60
30
1990
2000
2010
2020
2030
ASYR

Bolivia (Plurinational State of)

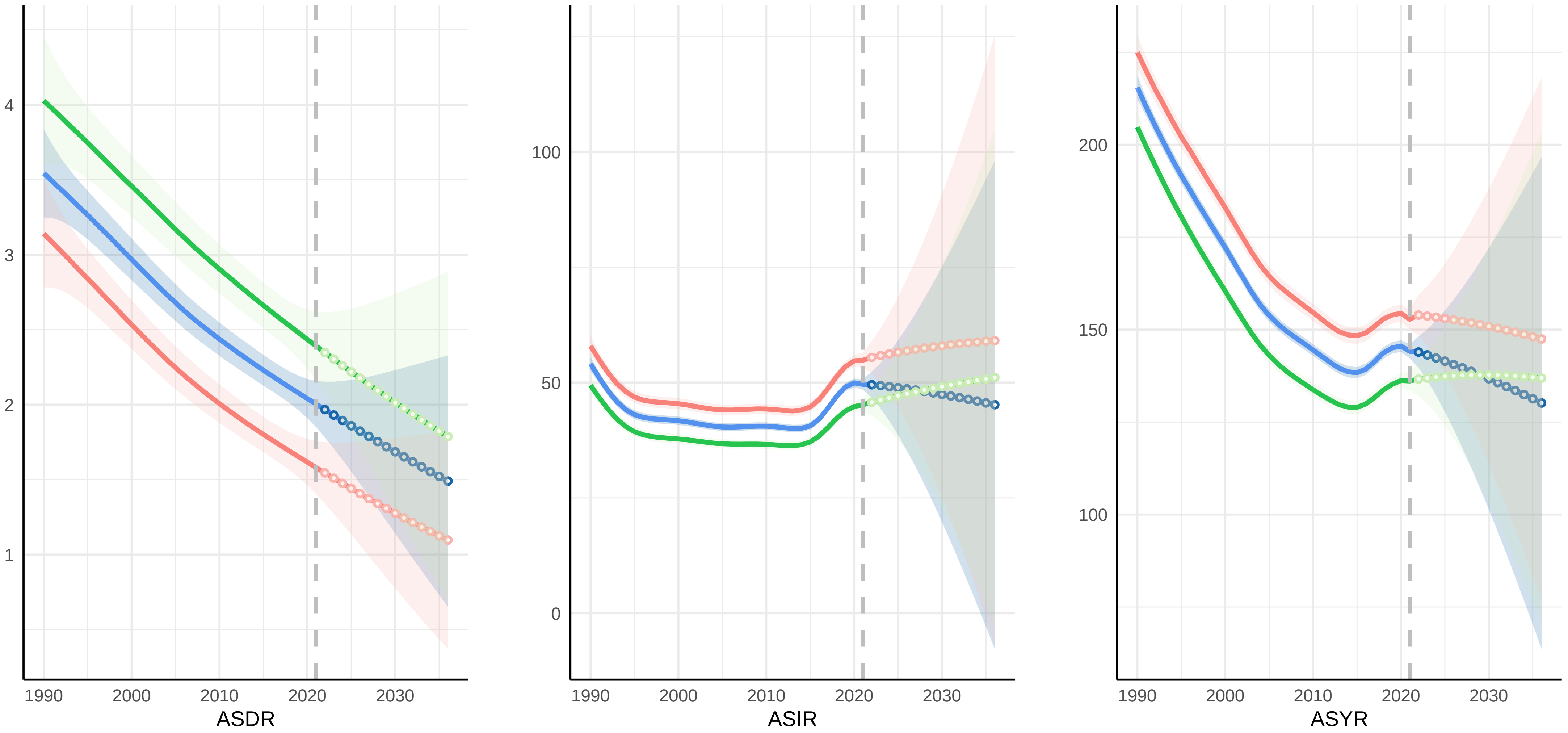

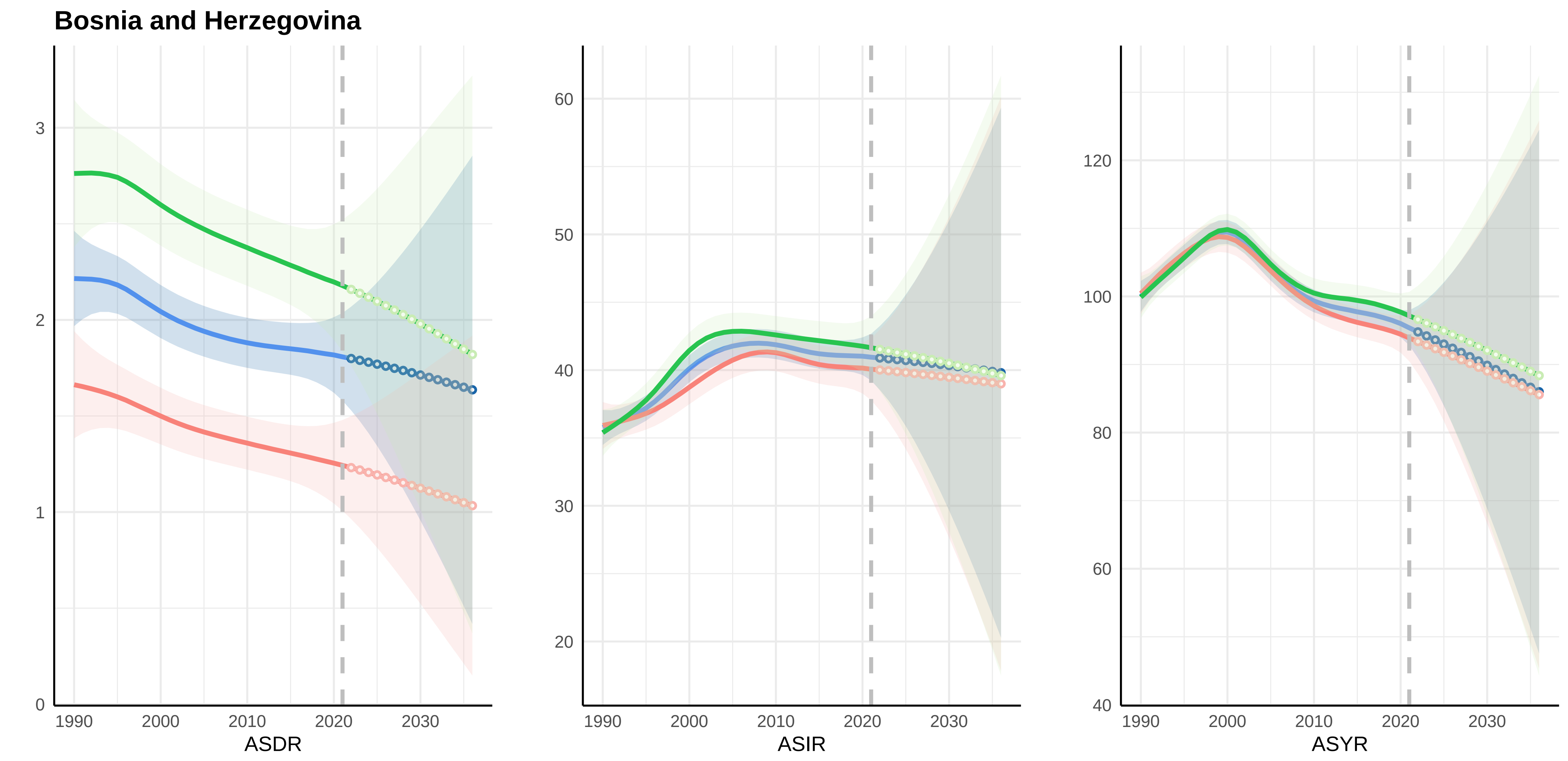

Bosnia and Herzegovina
3
2
1
0
1990
2000
2010
2020
2030
ASDR
60
50
40
30
20
ASIR
120
100
80
60
40
ASYR

Botswana

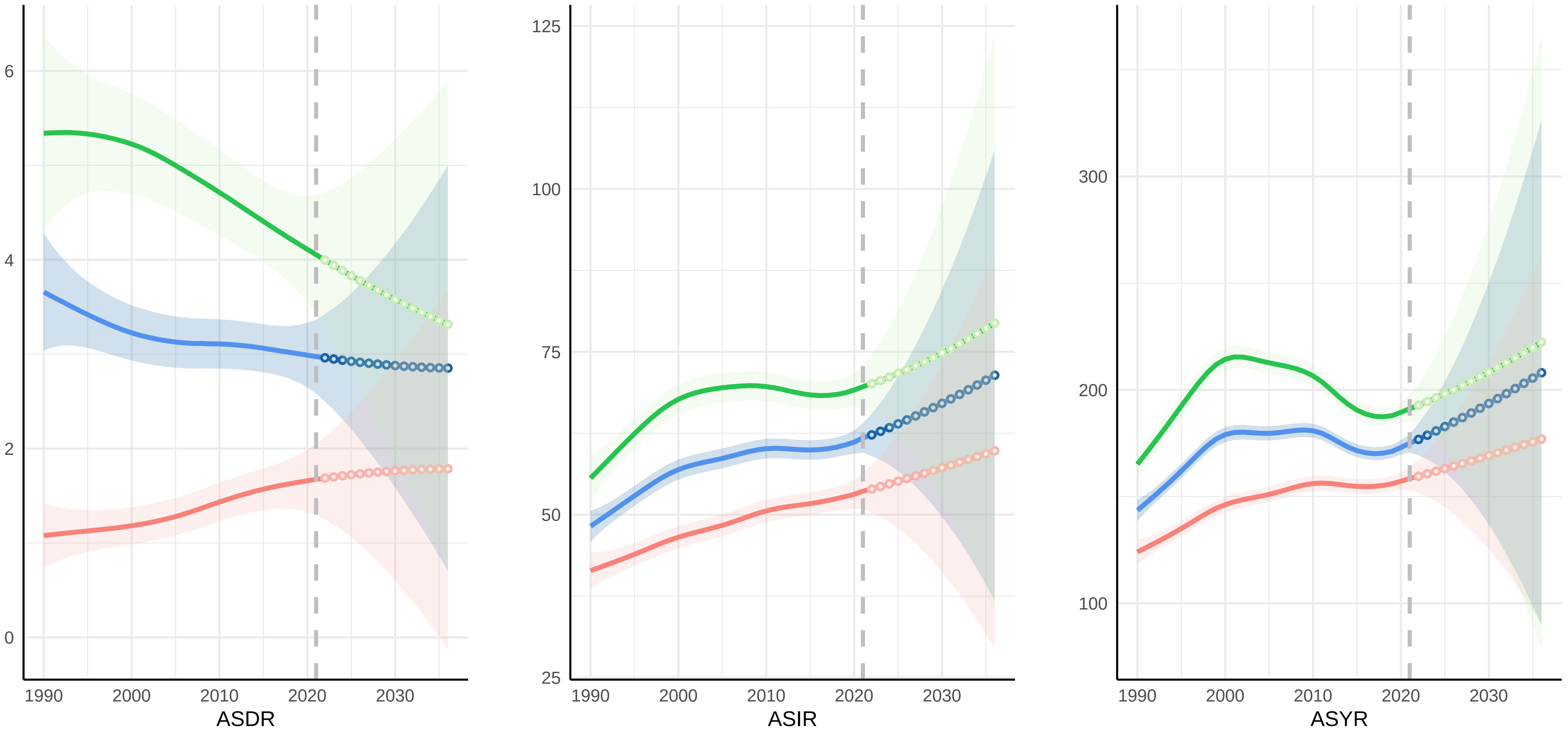

6
4
2
0
1990
2000
2010
2020
2030
ASDR
125
100
75
50
25
1990
2000
2010
2020
2030
ASIR
300
200
100
1990
2000
2010
2020
2030
ASYR

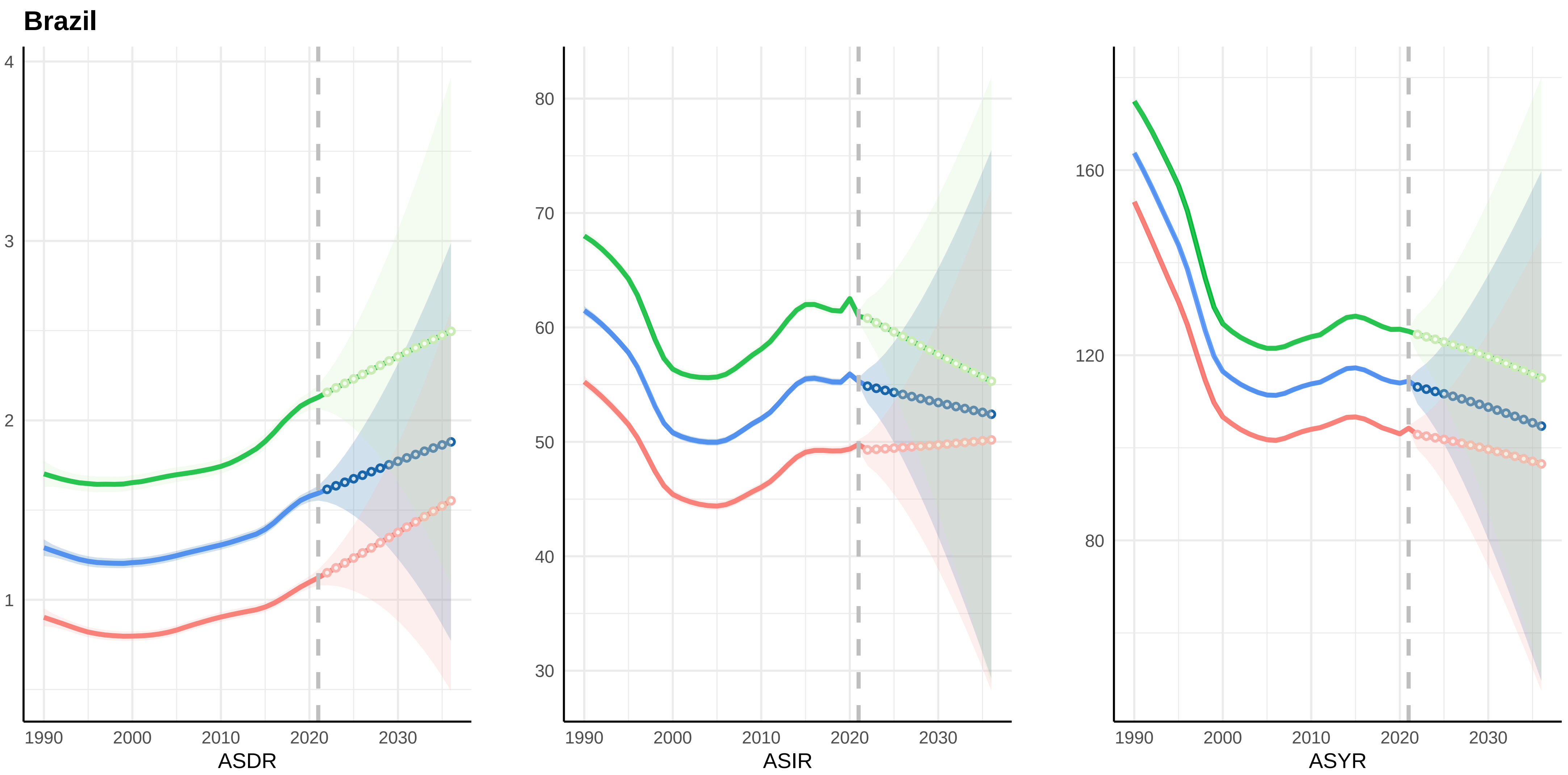
Brazil
4
3
2
1
1990
2000
2010
2020
2030
ASDR
80
70
60
50
40
30
1990
2000
2010
2020
2030
ASIR
160
120
80
1990
2000
2010
2020
2030
ASYR

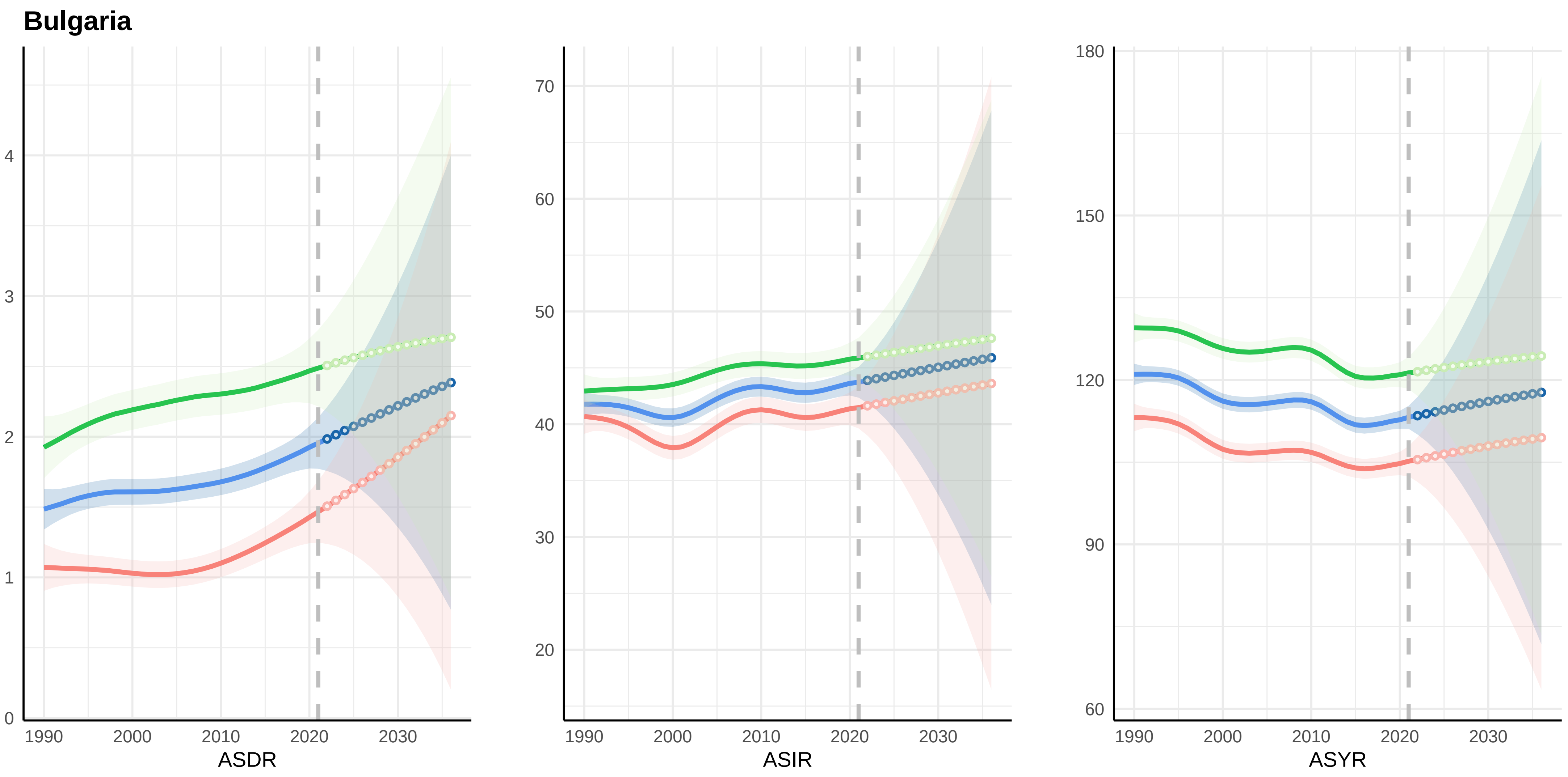
Bulgaria
4
3
2
1
0
1990
2000
2010
2020
2030
ASDR
70
60
50
40
30
20
1990
2000
2010
2020
2030
ASIR
180
150
120
90
60
1990
2000
2010
2020
2030
ASYR

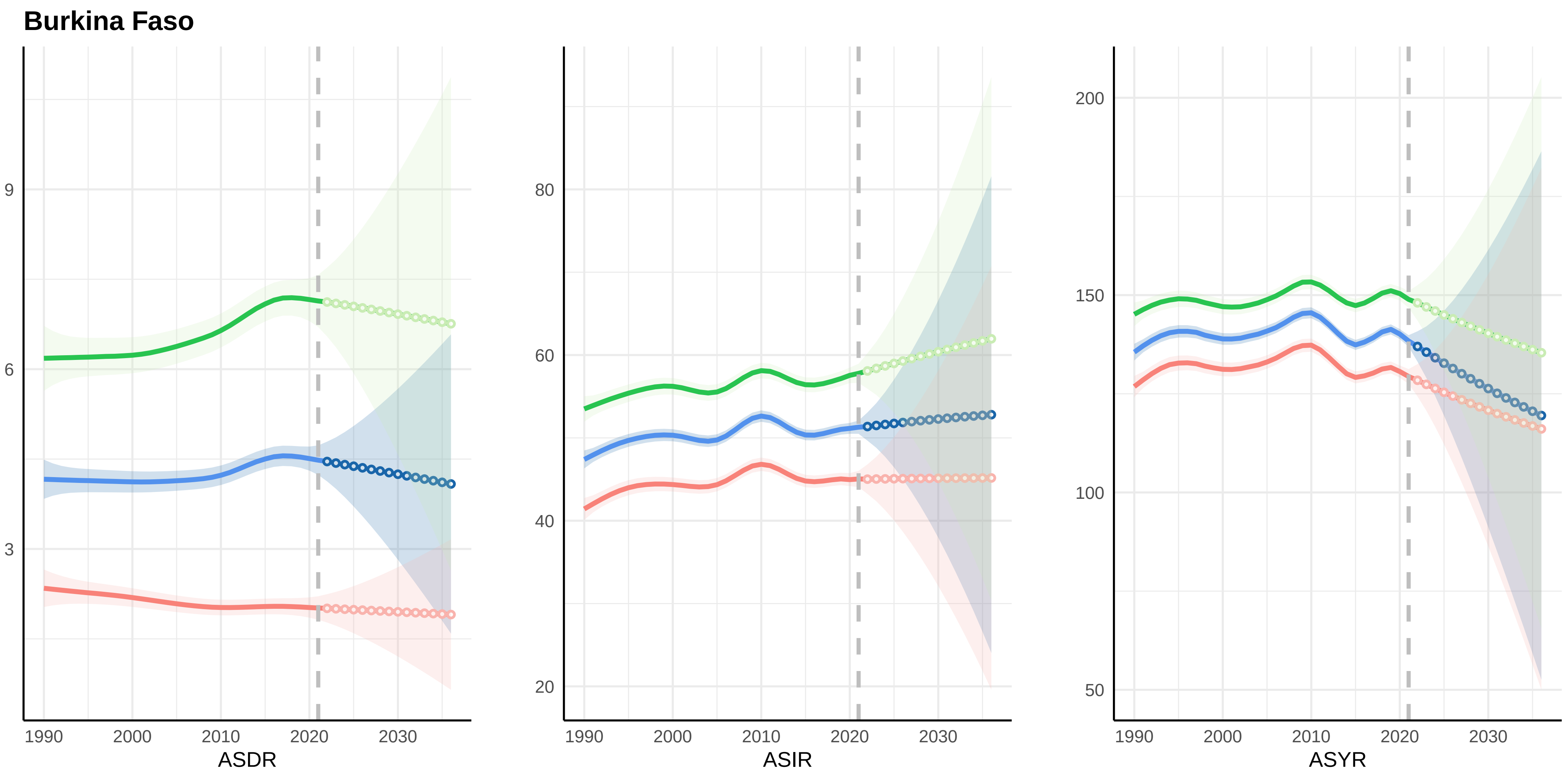
Burkina Faso
9
6
3
1990
2000
2010
2020
2030
ASDR
80
60
40
20
1990
2000
2010
2020
2030
ASIR
200
150
100
50
1990
2000
2010
2020
2030
ASYR

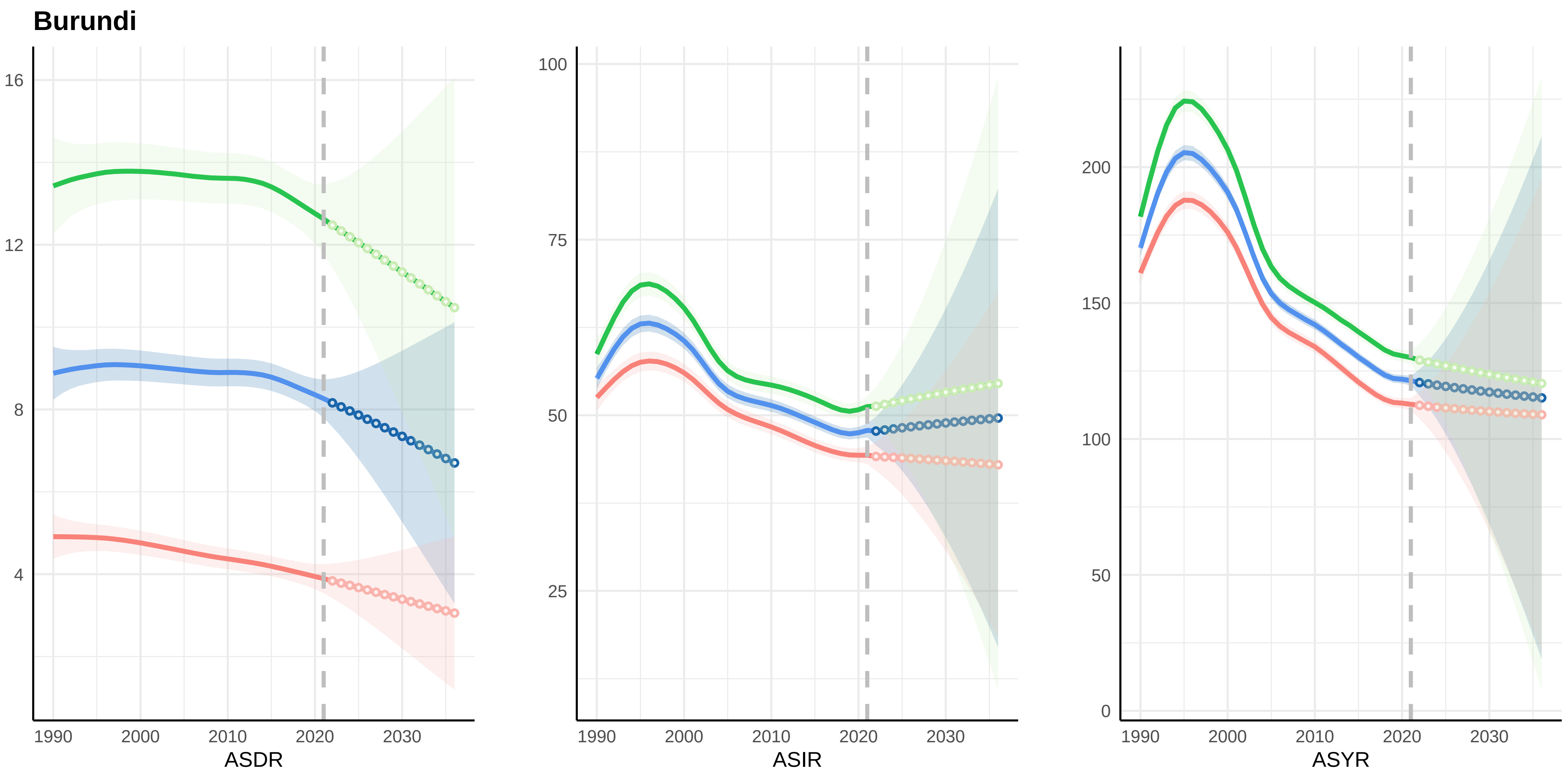

Burundi
16
12
8
4
1990
2000
2010
2020
2030
ASDR
100
75
50
25
1990
2000
2010
2020
2030
ASIR
200
150
100
50
0
1990
2000
2010
2020
2030
ASYR

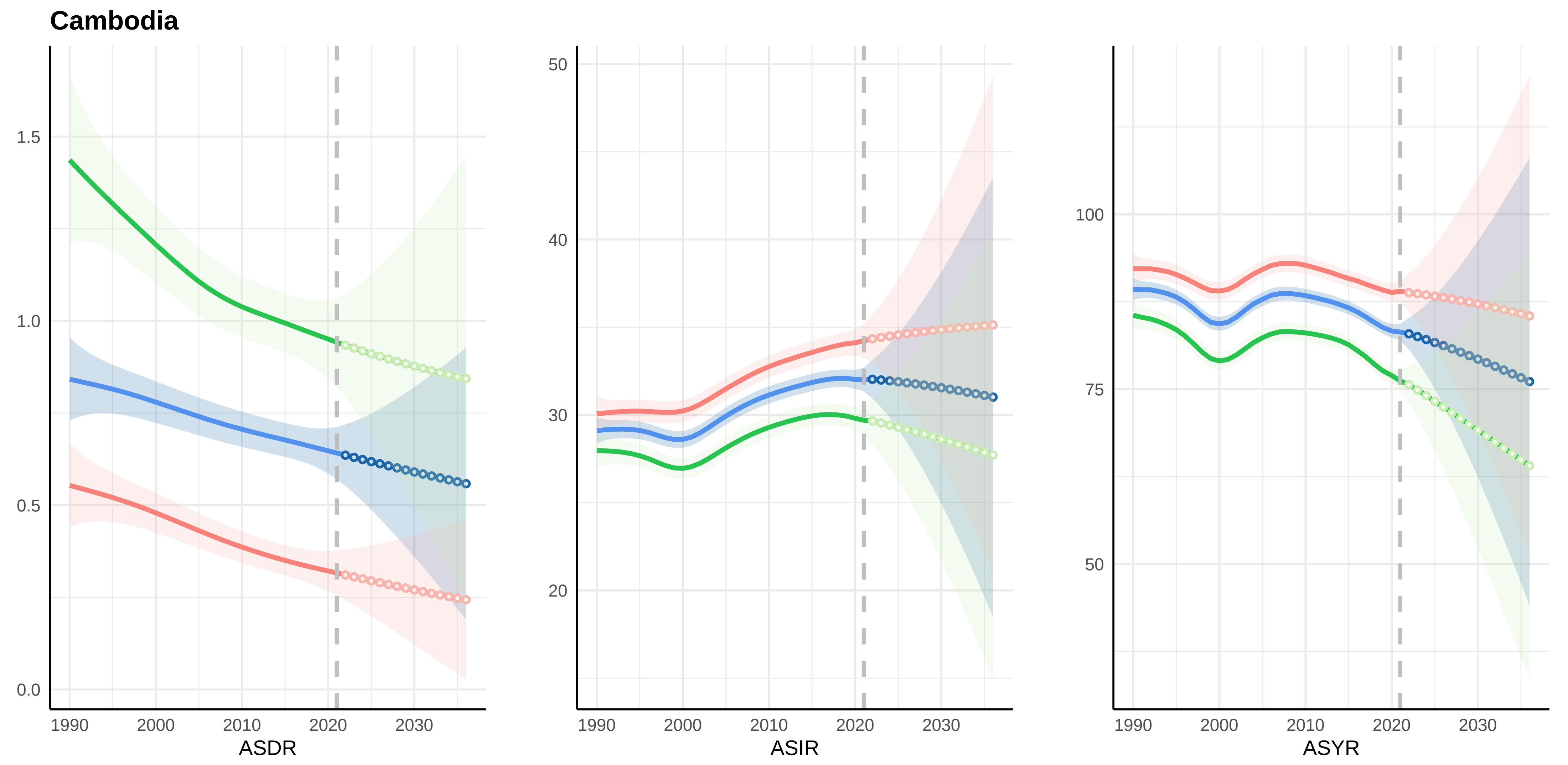
Cambodia
1.5
1.0
0.5
0.0
1990
2000
2010
2020
2030
ASDR
50
40
30
20
1990
2000
2010
2020
2030
ASIR
100
75
50
1990
2000
2010
2020
2030
ASYR

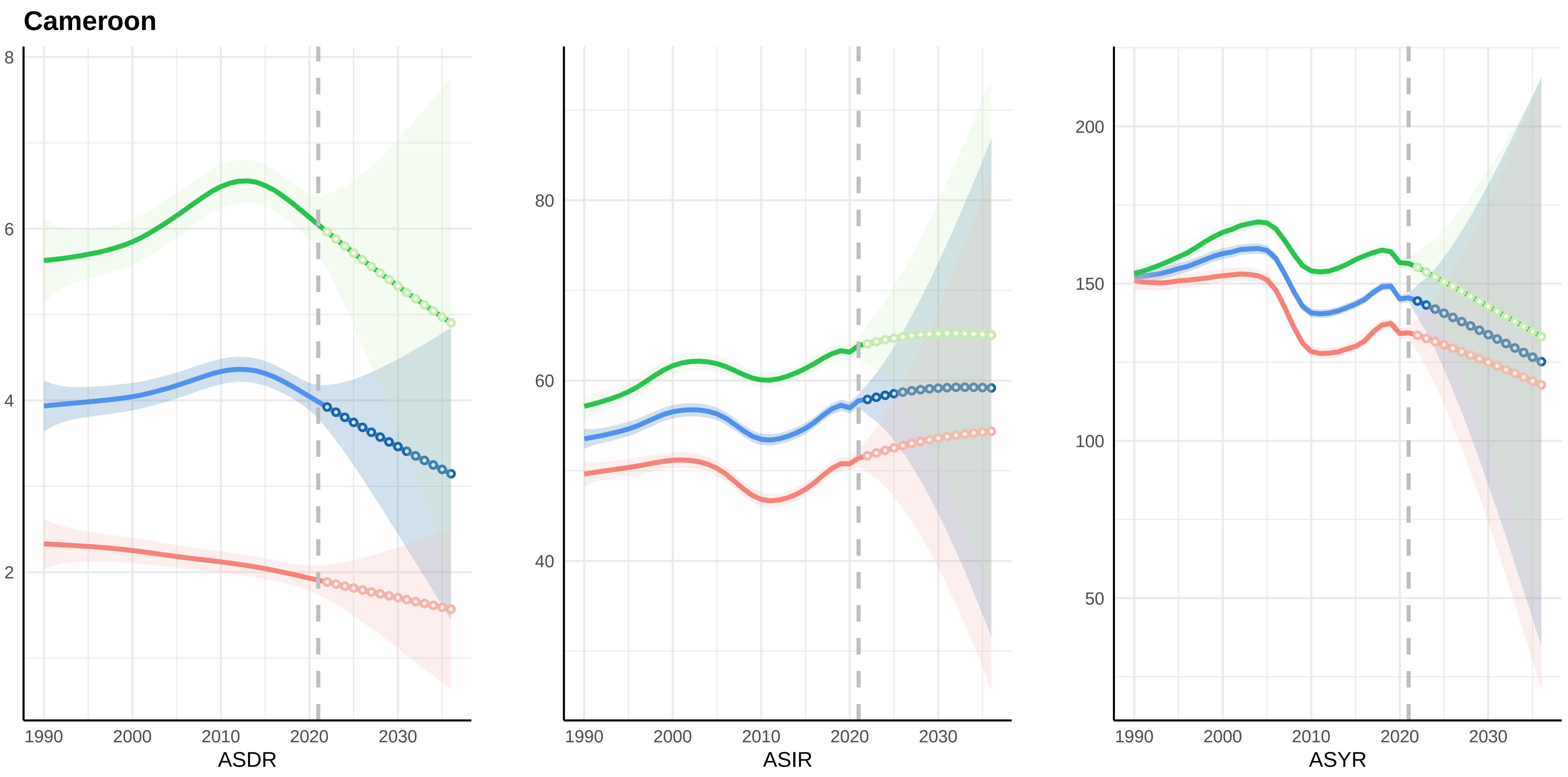

Cameroon
8
6
4
2
1990
2000
2010
2020
2030
ASDR
80
60
40
1990
2000
2010
2020
2030
ASIR
200
150
100
50
1990
2000
2010
2020
2030
ASYR

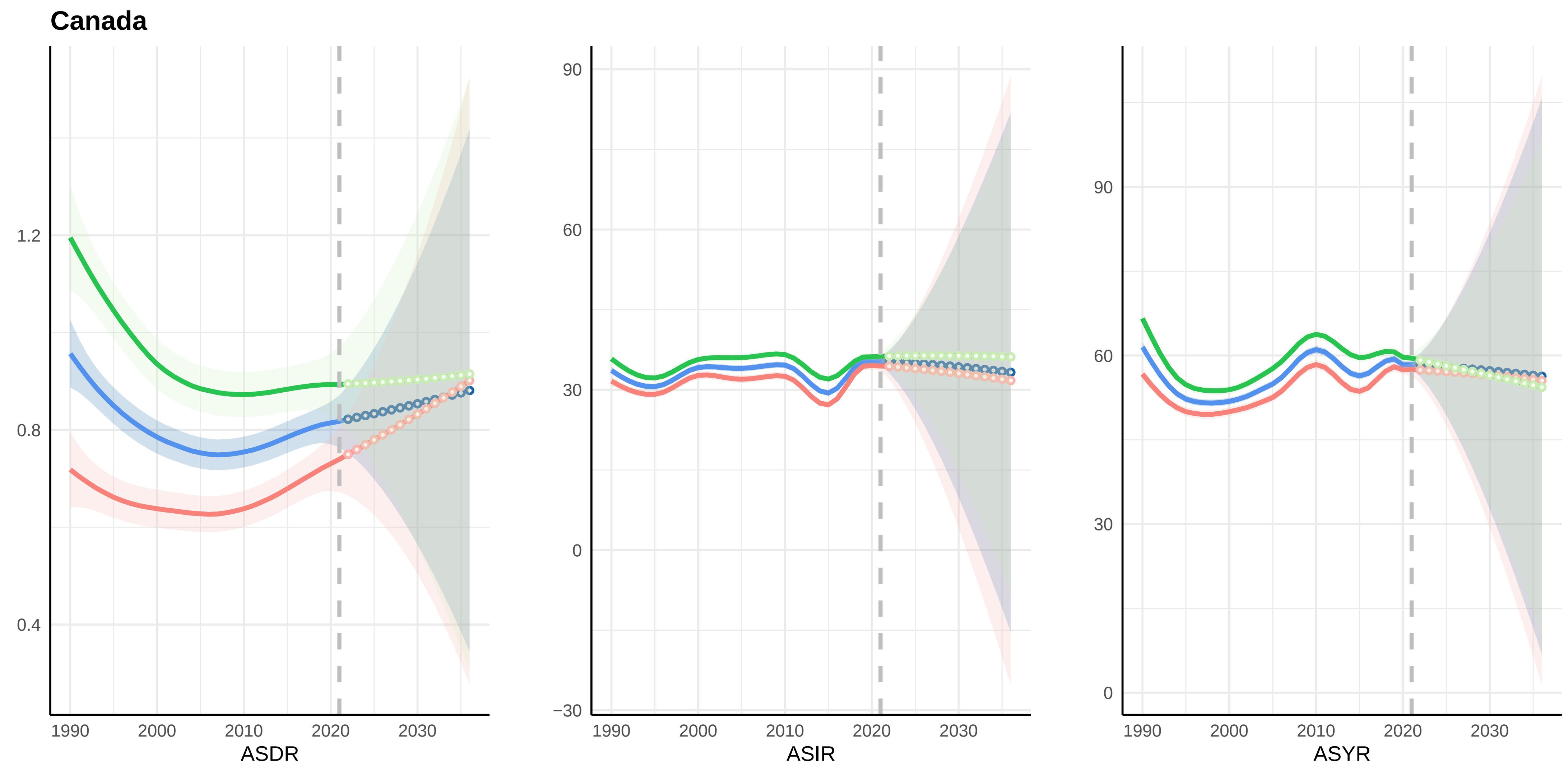

Canada
1.2
0.8
0.4
1990
2000
2010
2020
2030
ASDR
90
60
30
0
−30
1990
2000
2010
2020
2030
ASIR
90
60
30
0
1990
2000
2010
2020
2030
ASYR

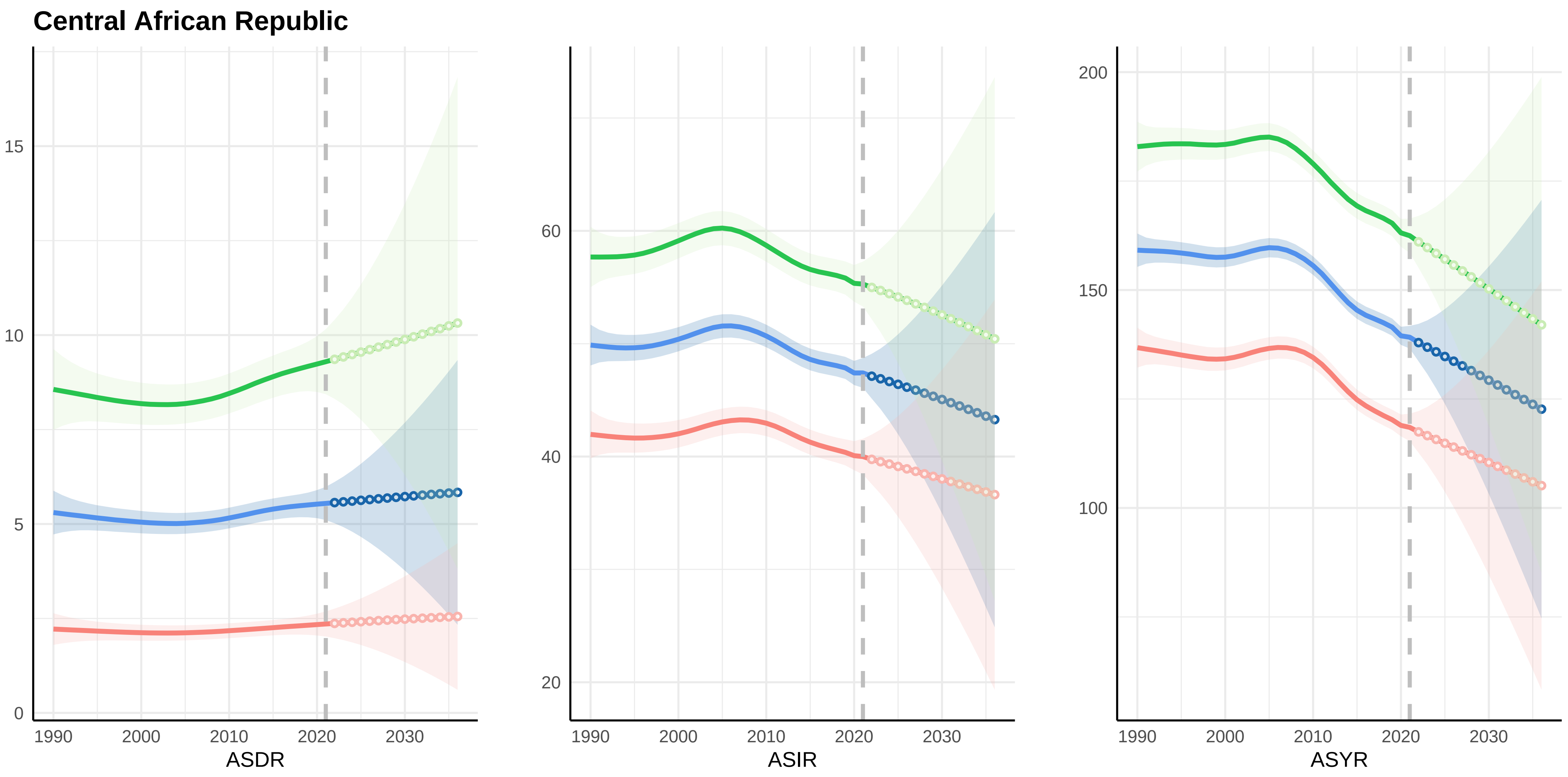
Central African Republic
15
10
5
0
1990
2000
2010
2020
2030
ASDR
60
40
20
1990
2000
2010
2020
2030
ASIR
200
150
100
1990
2000
2010
2020
2030
ASYR

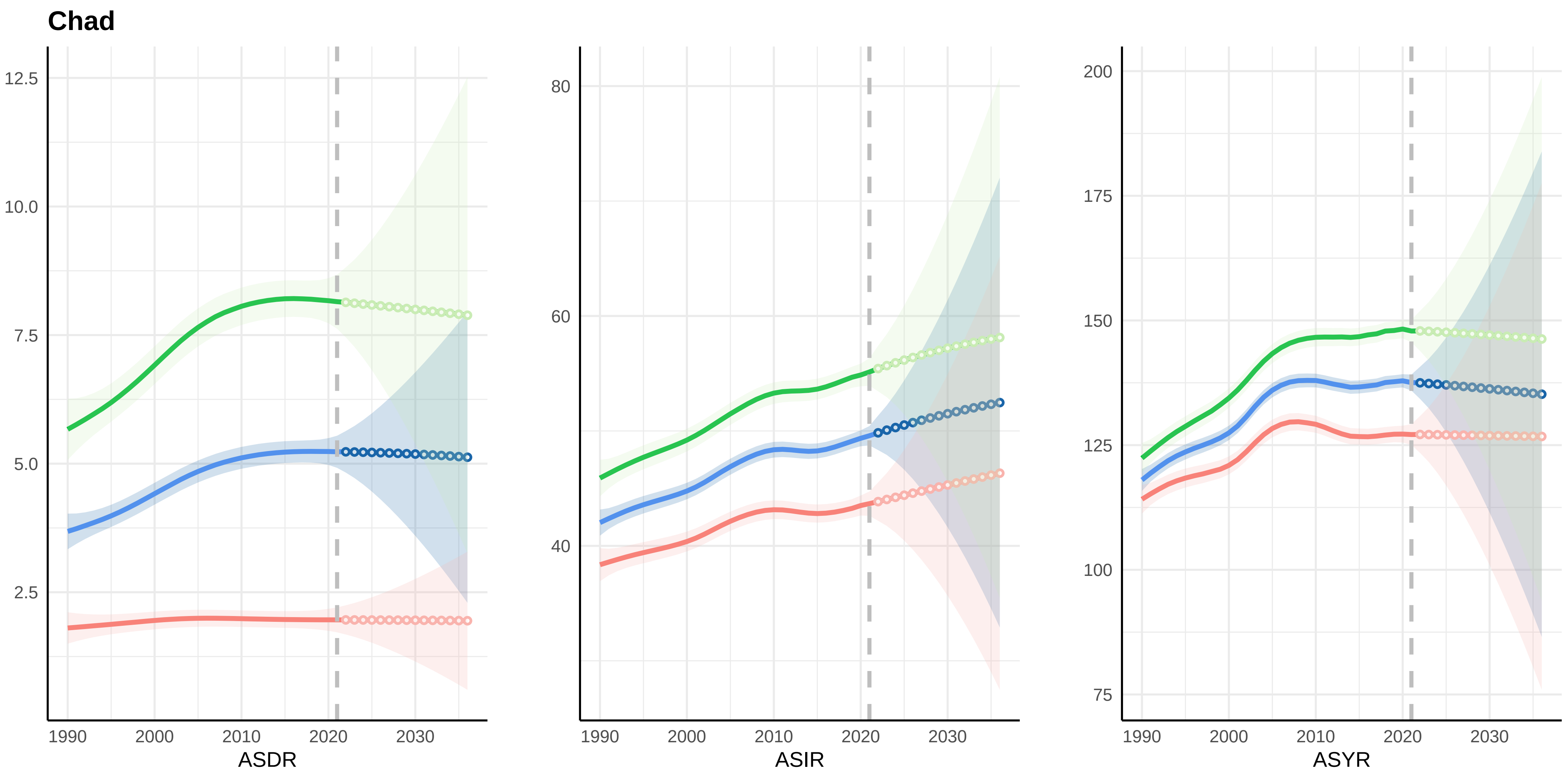

Chad
12.5
10.0
7.5
5.0
2.5
1990
2000
2010
2020
2030
ASDR
80
60
40
1990
2000
2010
2020
2030
ASIR
200
175
150
125
100
75
1990
2000
2010
2020
2030
ASYR

Chile

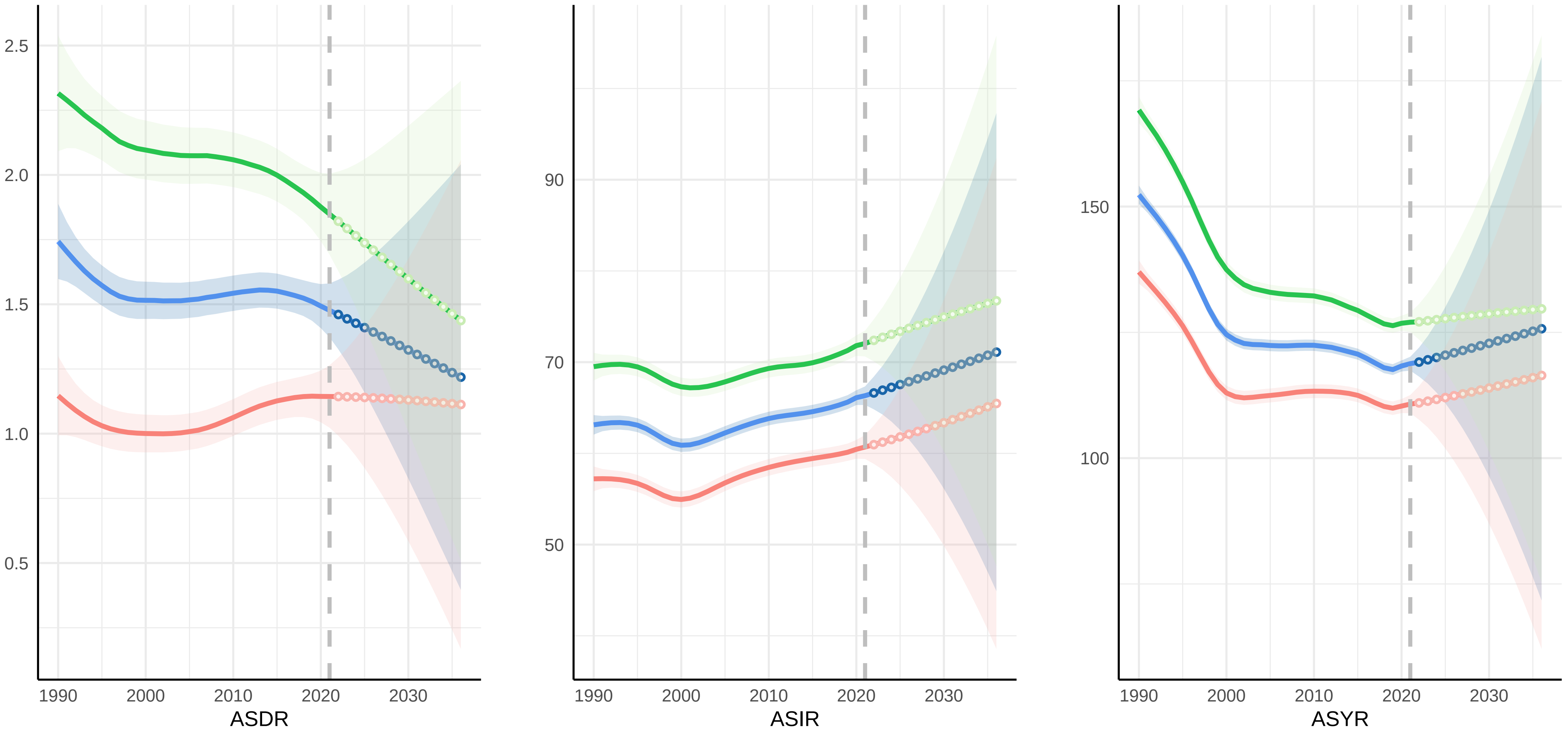

2.5
2.0
1.5
1.0
0.5
1990
2000
2010
2020
2030
ASDR
90
70
50
1990
2000
2010
2020
2030
ASIR
150
100
1990
2000
2010
2020
2030
ASYR

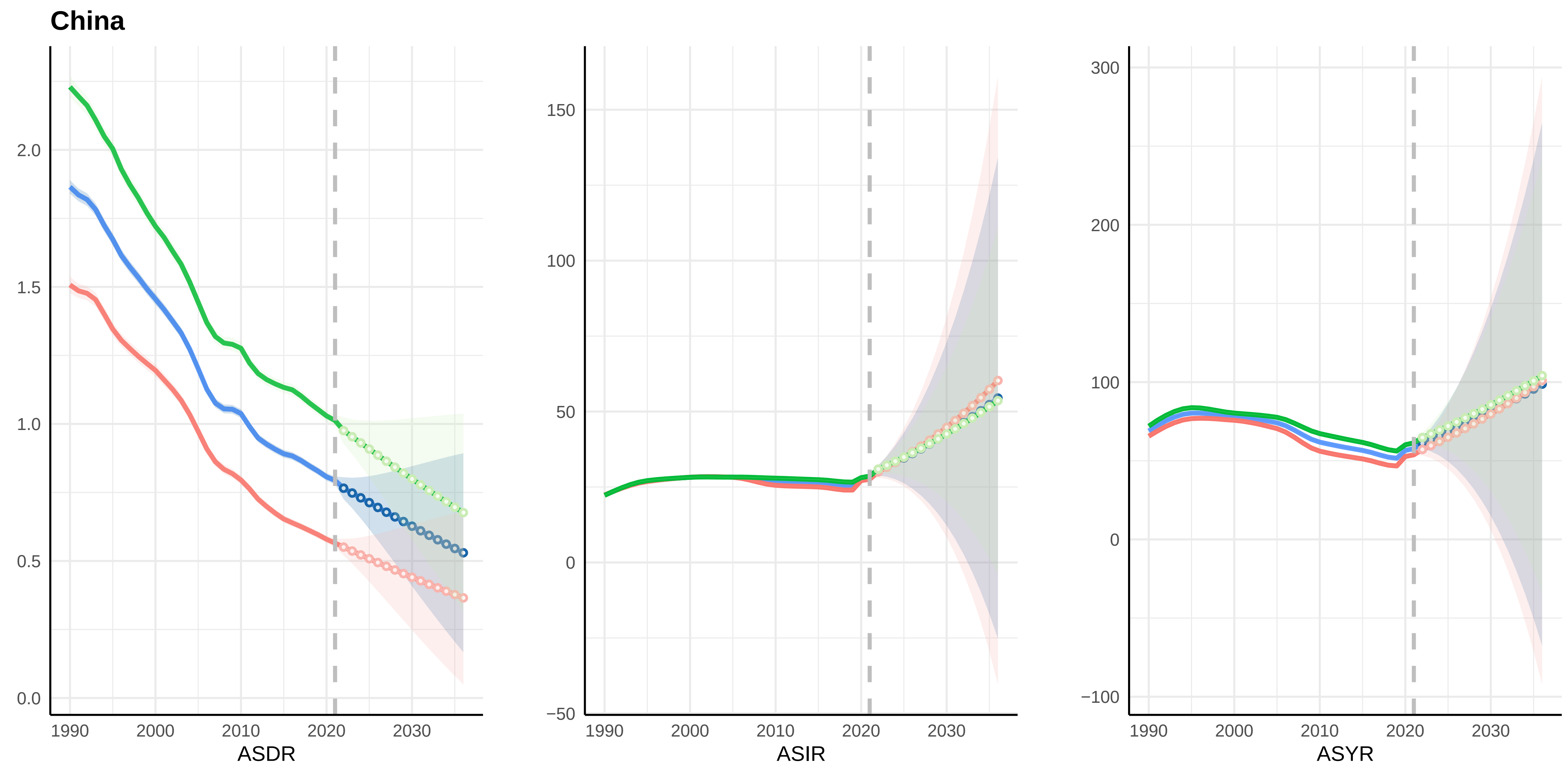

China
2.0
1.5
1.0
0.5
0.0
1990
2000
2010
2020
2030
ASDR
150
100
50
0
−50
1990
2000
2010
2020
2030
ASIR
300
200
100
0
−100
1990
2000
2010
2020
2030
ASYR

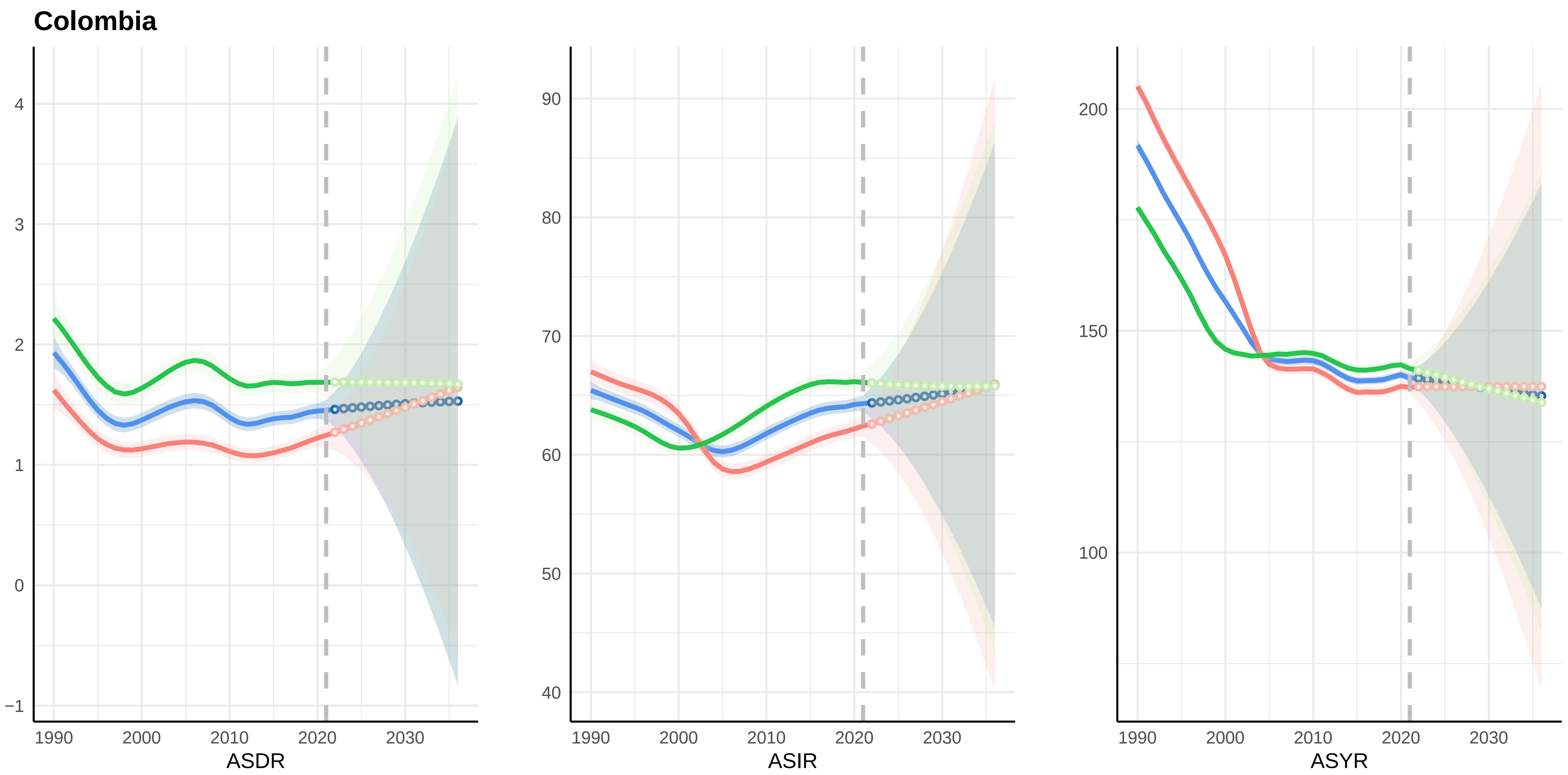

Colombia
4
3
2
1
0
−1
1990
2000
2010
2020
2030
ASDR
90
80
70
60
50
40
ASIR
200
150
100
ASYR

Comoros

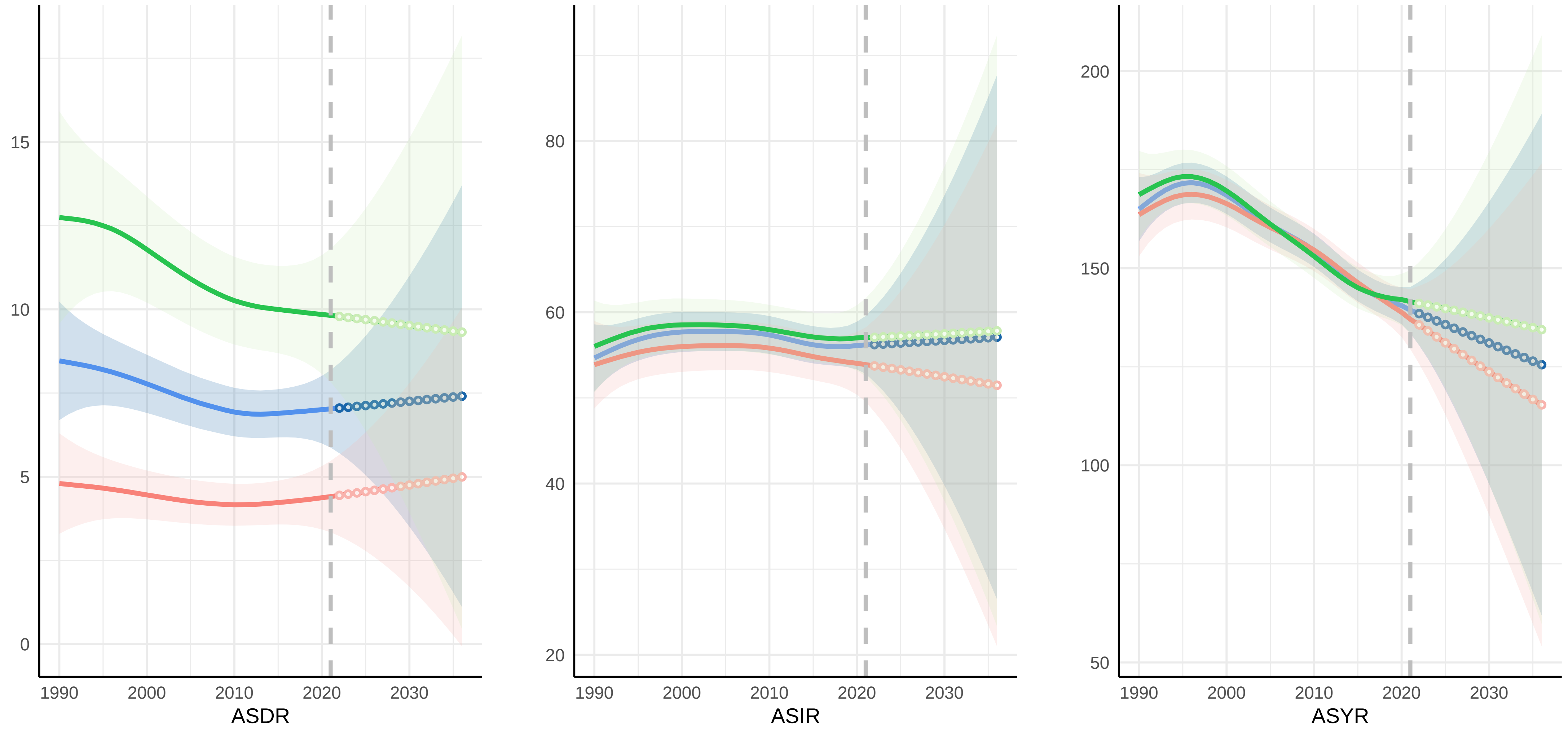
15
10
5
0
1990
2000
2010
2020
2030
ASDR
80
60
40
20
1990
2000
2010
2020
2030
ASIR
200
150
100
50
1990
2000
2010
2020
2030
ASYR

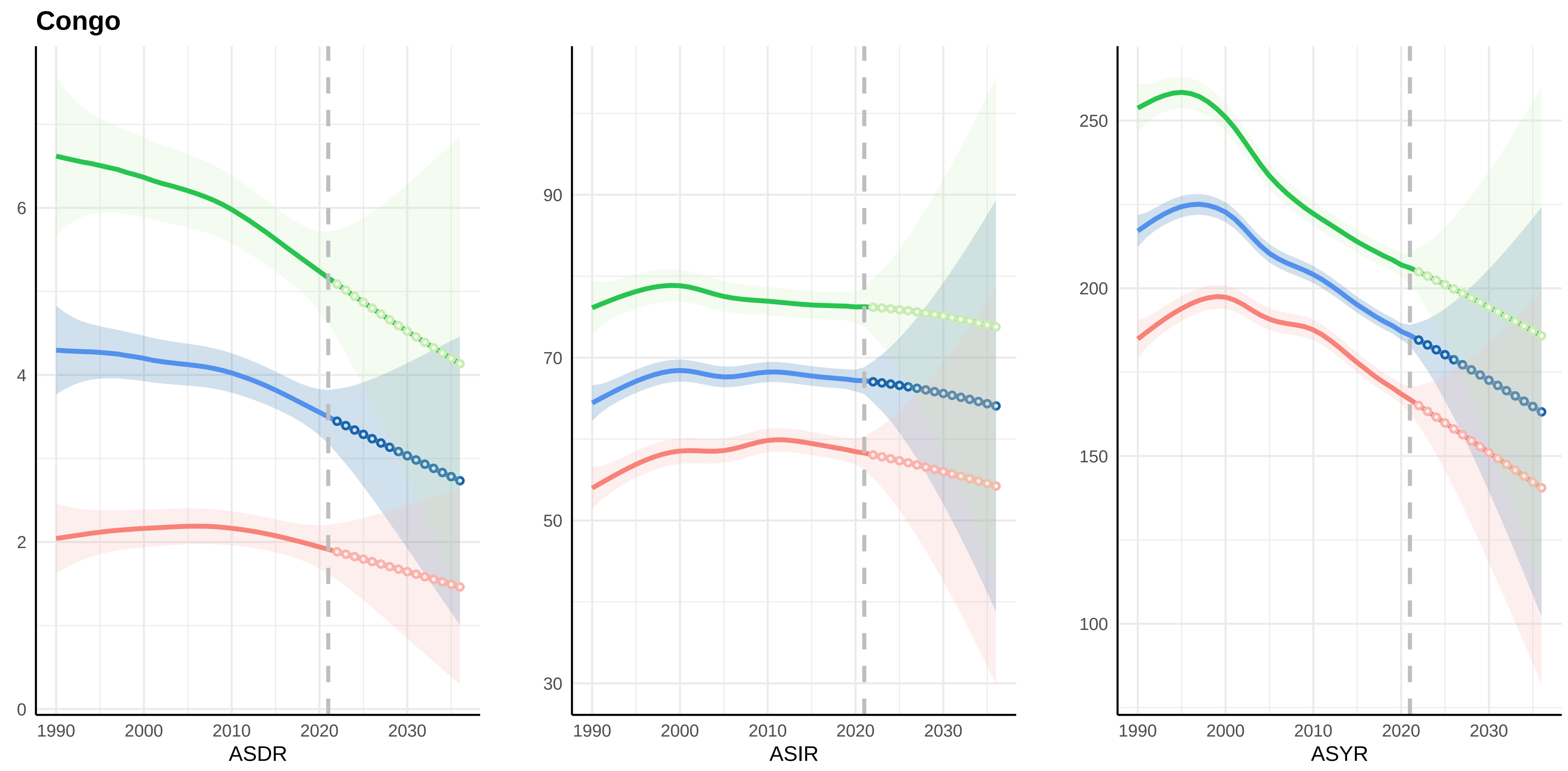

Congo
6
4
2
0
1990
2000
2010
2020
2030
ASDR
90
70
50
30
ASIR
250
200
150
100
ASYR

Costa Rica

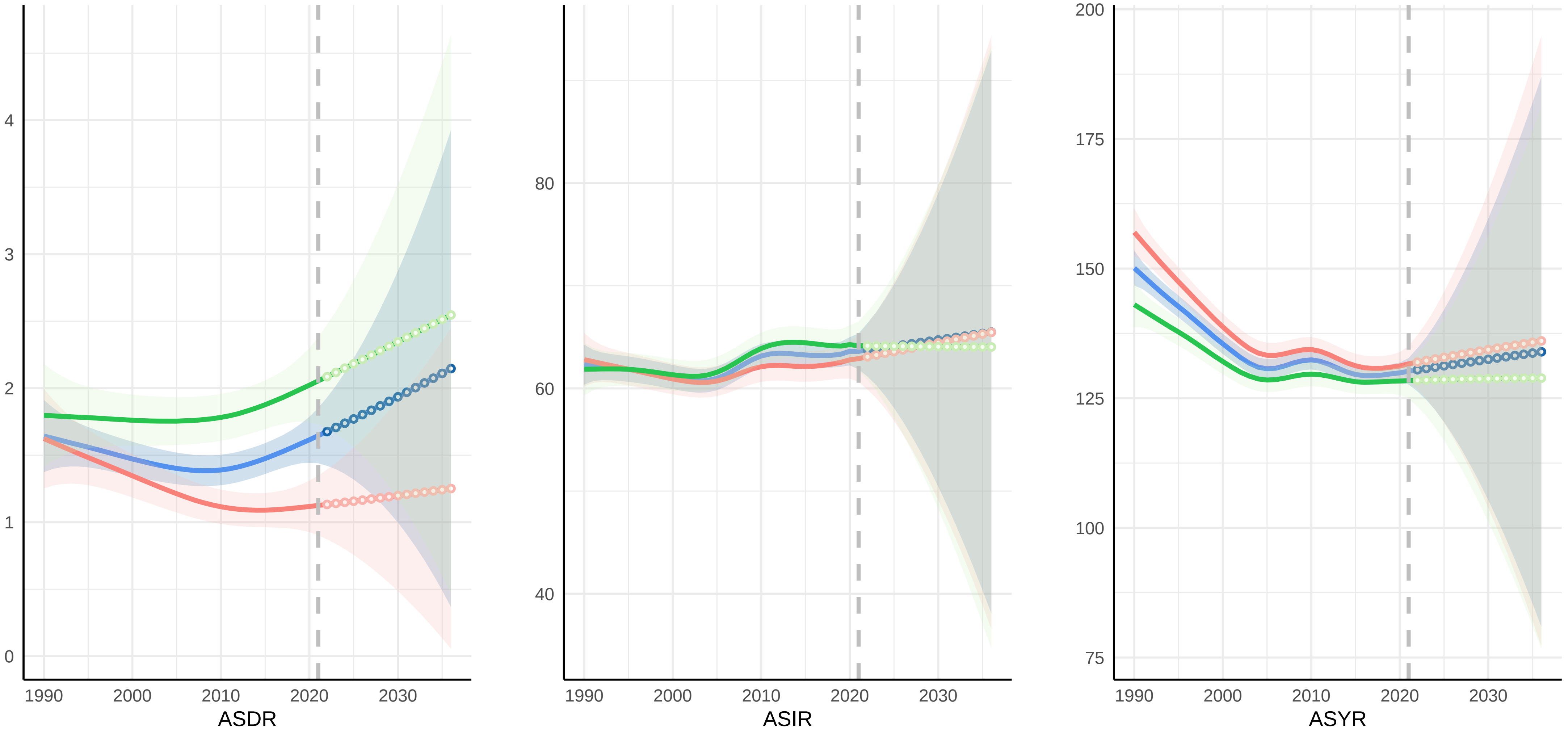

0
1
2
3
4
1990
2000
2010
2020
2030
ASDR
40
60
80
1990
2000
2010
2020
2030
ASIR
75
100
125
150
175
200
1990
2000
2010
2020
2030
ASYR

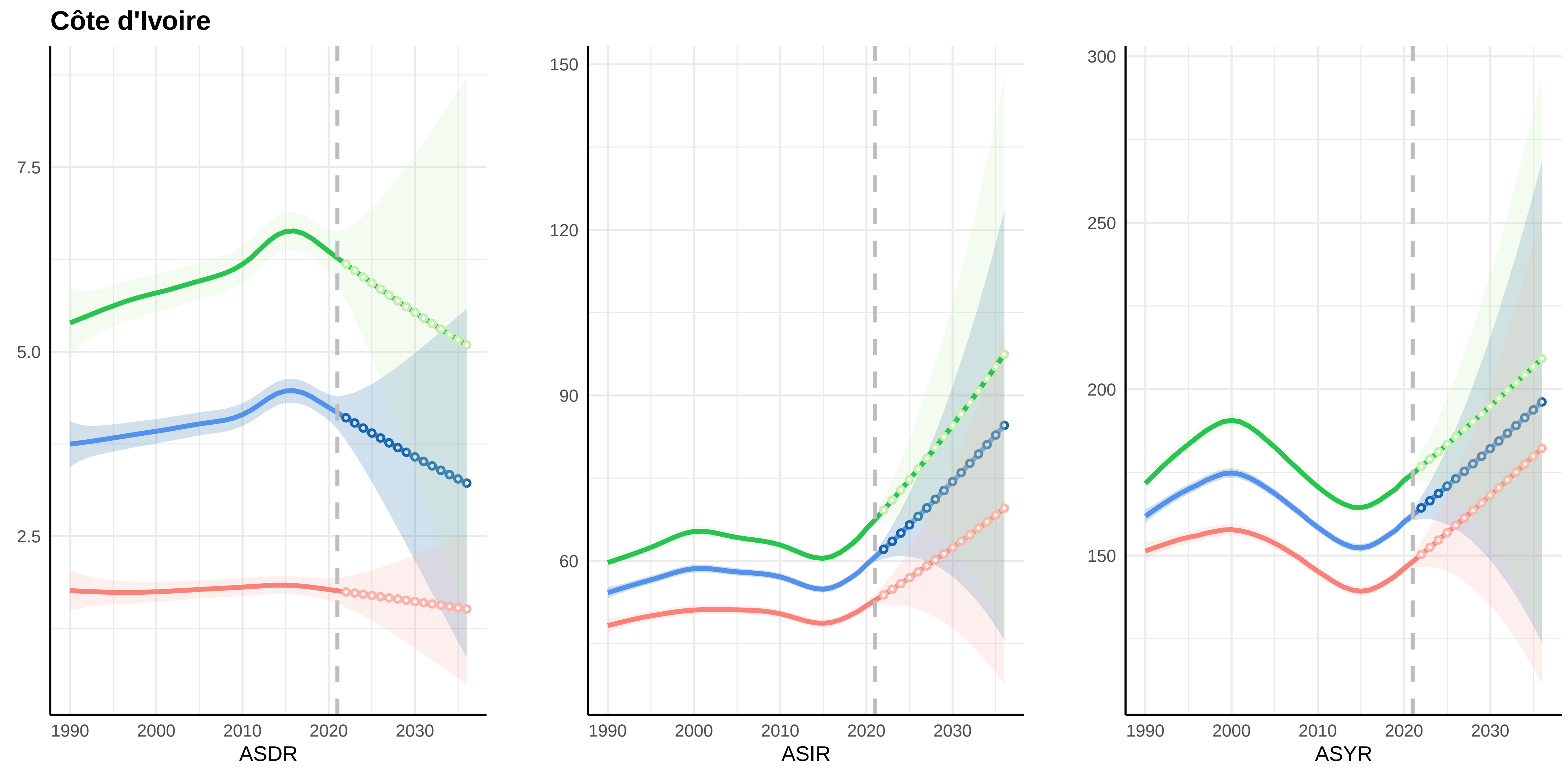

Côte d'Ivoire
7.5
5.0
2.5
1990
2000
2010
2020
2030
ASDR
150
120
90
60
1990
2000
2010
2020
2030
ASIR
300
250
200
150
1990
2000
2010
2020
2030
ASYR

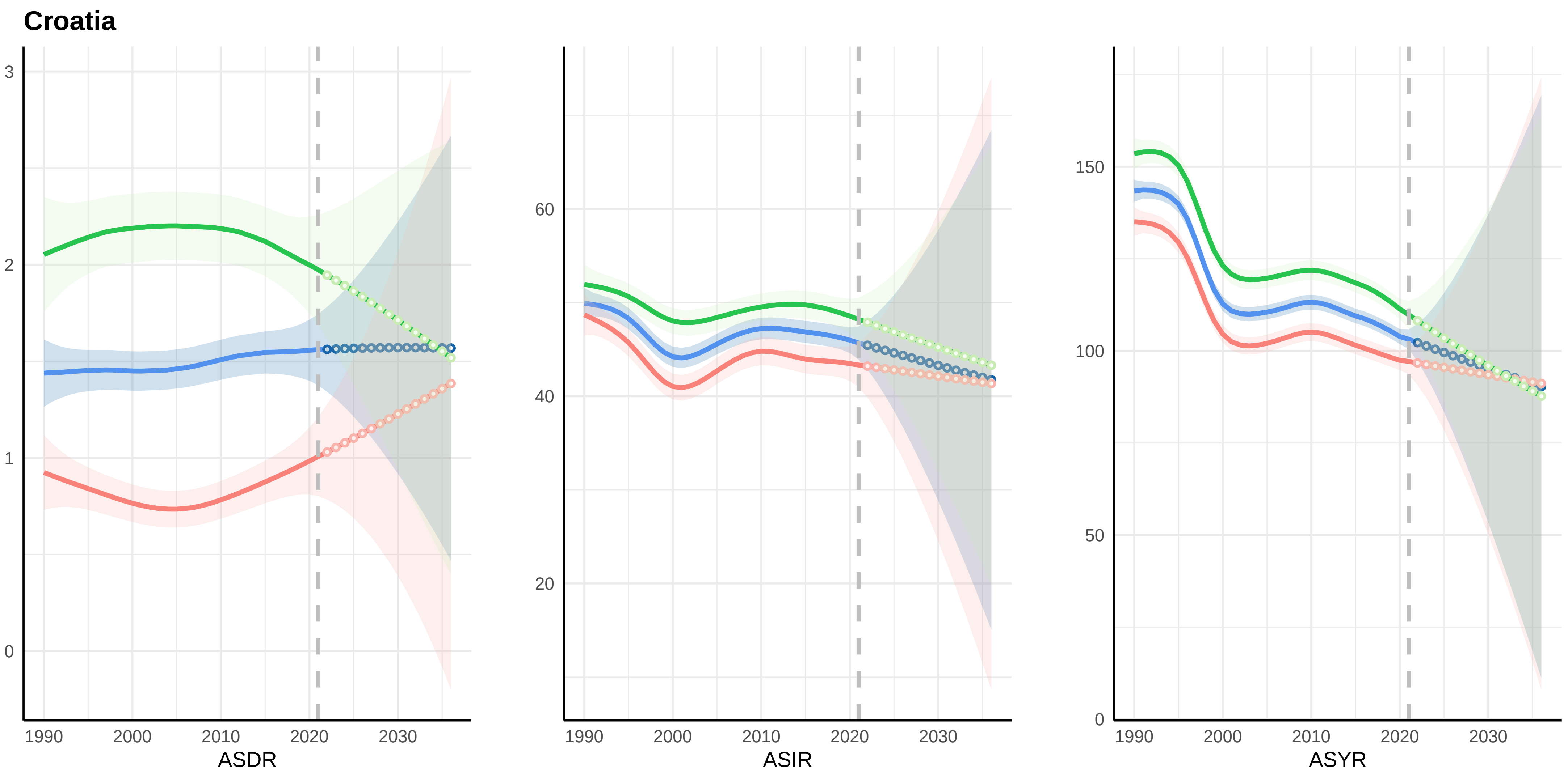

Croatia
3
2
1
0
1990
2000
2010
2020
2030
ASDR
60
40
20
ASIR
150
100
50
0
ASYR

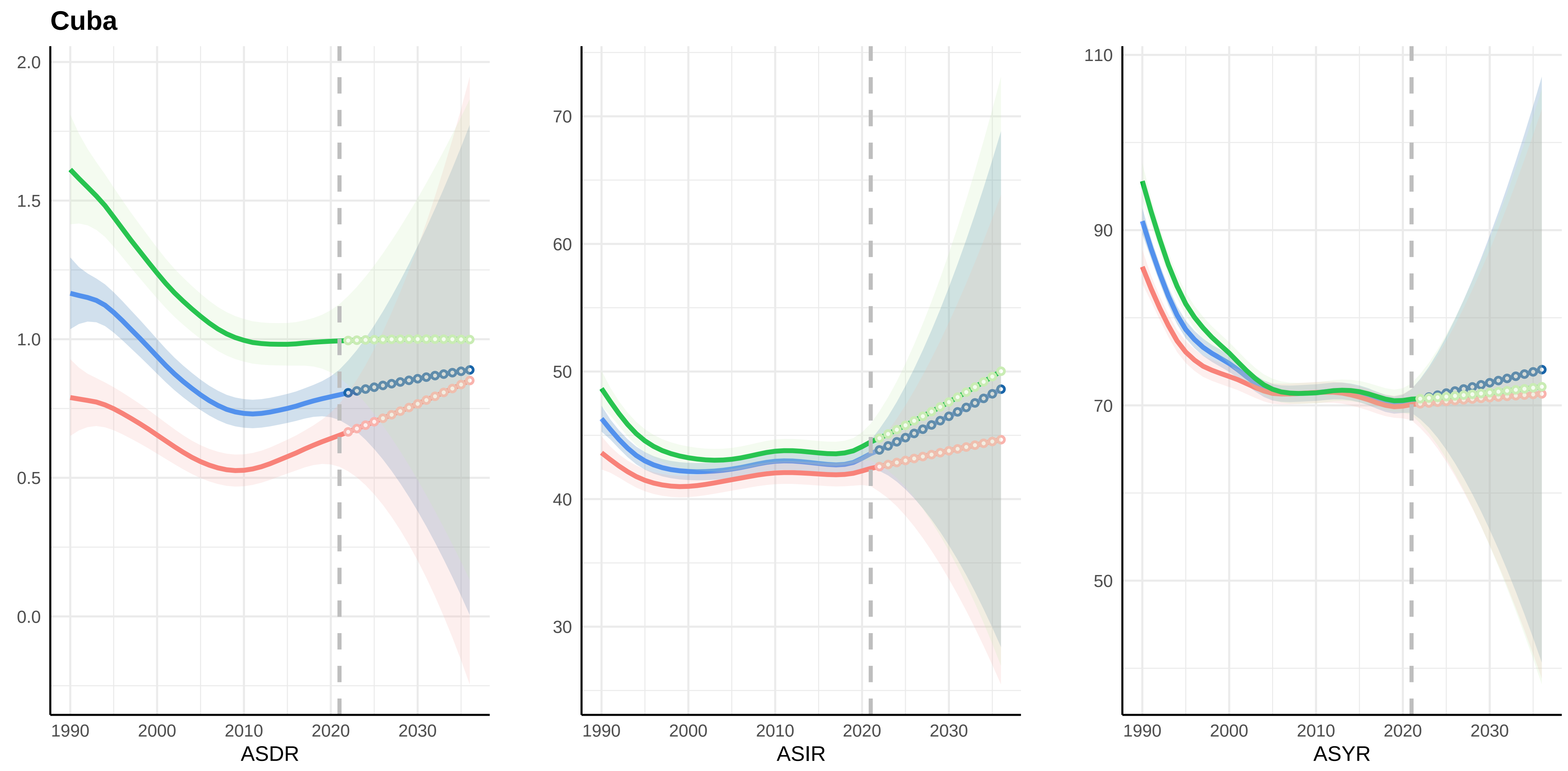
Cuba
2.0
1.5
1.0
0.5
0.0
1990
2000
2010
2020
2030
ASDR
70
60
50
40
30
ASIR
110
90
70
50
ASYR

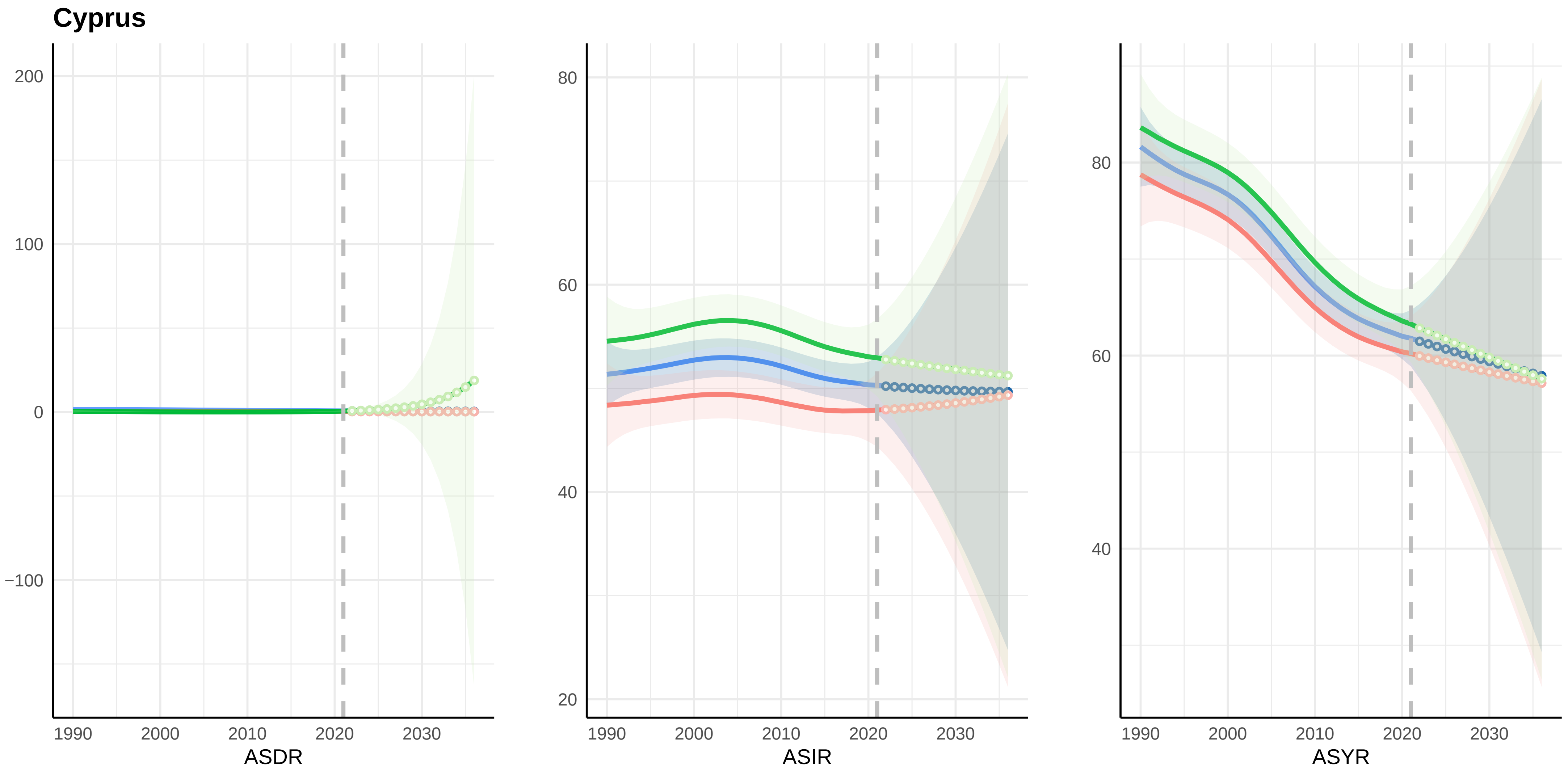

Cyprus
200
100
0
−100
1990
2000
2010
2020
2030
ASDR
80
60
40
20
1990
2000
2010
2020
2030
ASIR
80
60
40
1990
2000
2010
2020
2030
ASYR

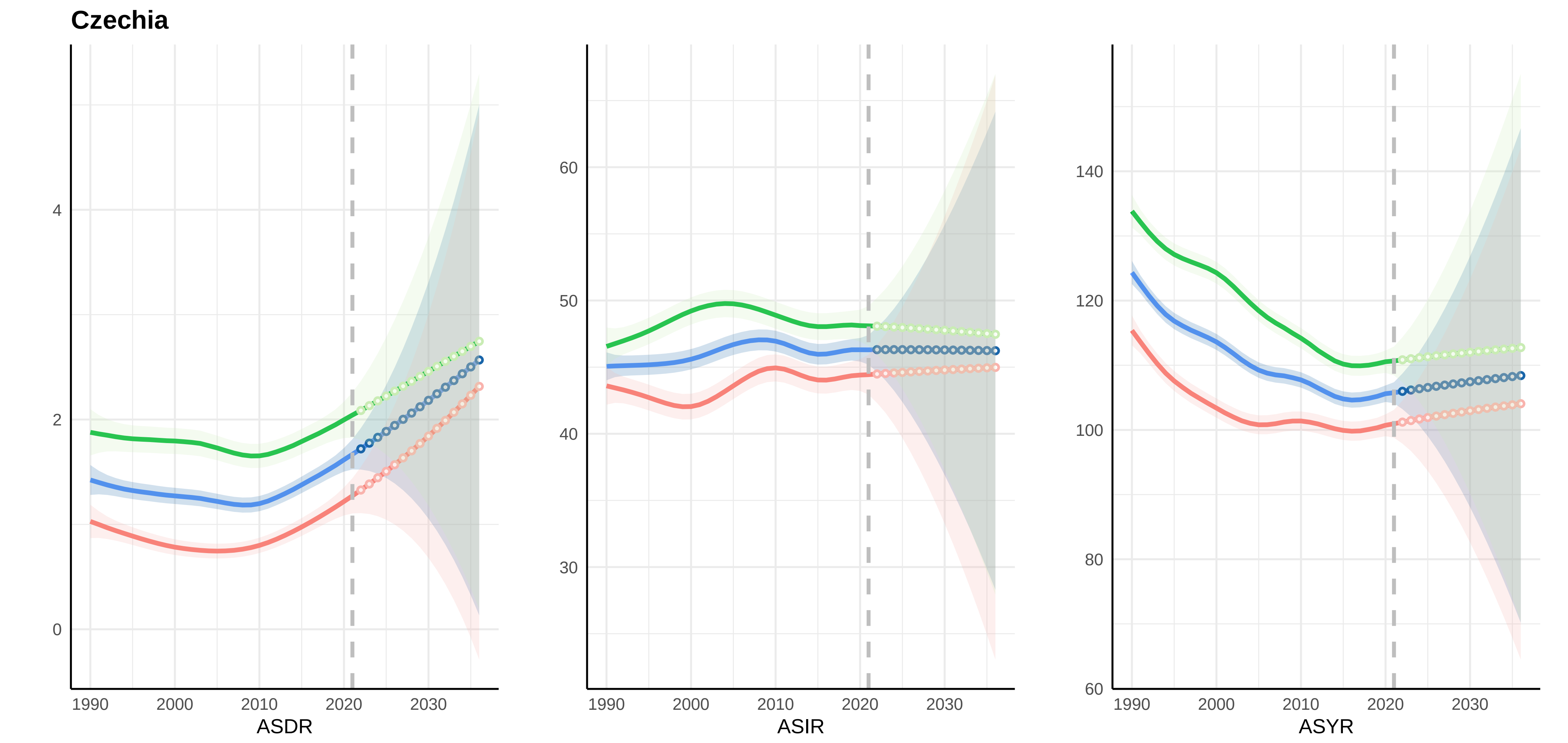

Czechia
0
2
4
60
50
40
30
140
120
100
80
60
1990
2000
2010
2020
2030
ASDR
ASIR
ASYR

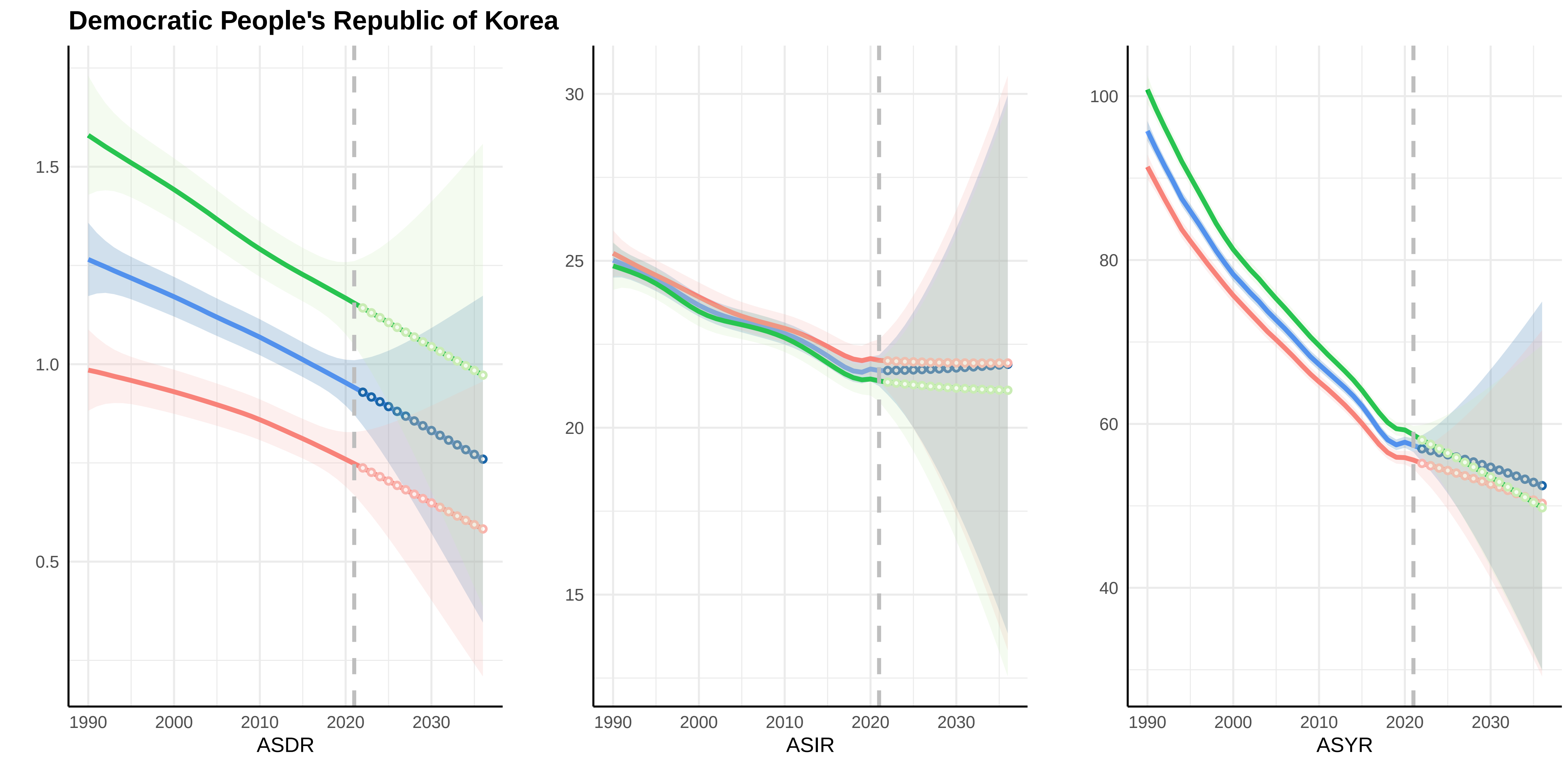
Democratic People's Republic of Korea
1.5
1.0
0.5
1990
2000
2010
2020
2030
ASDR
30
25
20
15
1990
2000
2010
2020
2030
ASIR
100
80
60
40
1990
2000
2010
2020
2030
ASYR

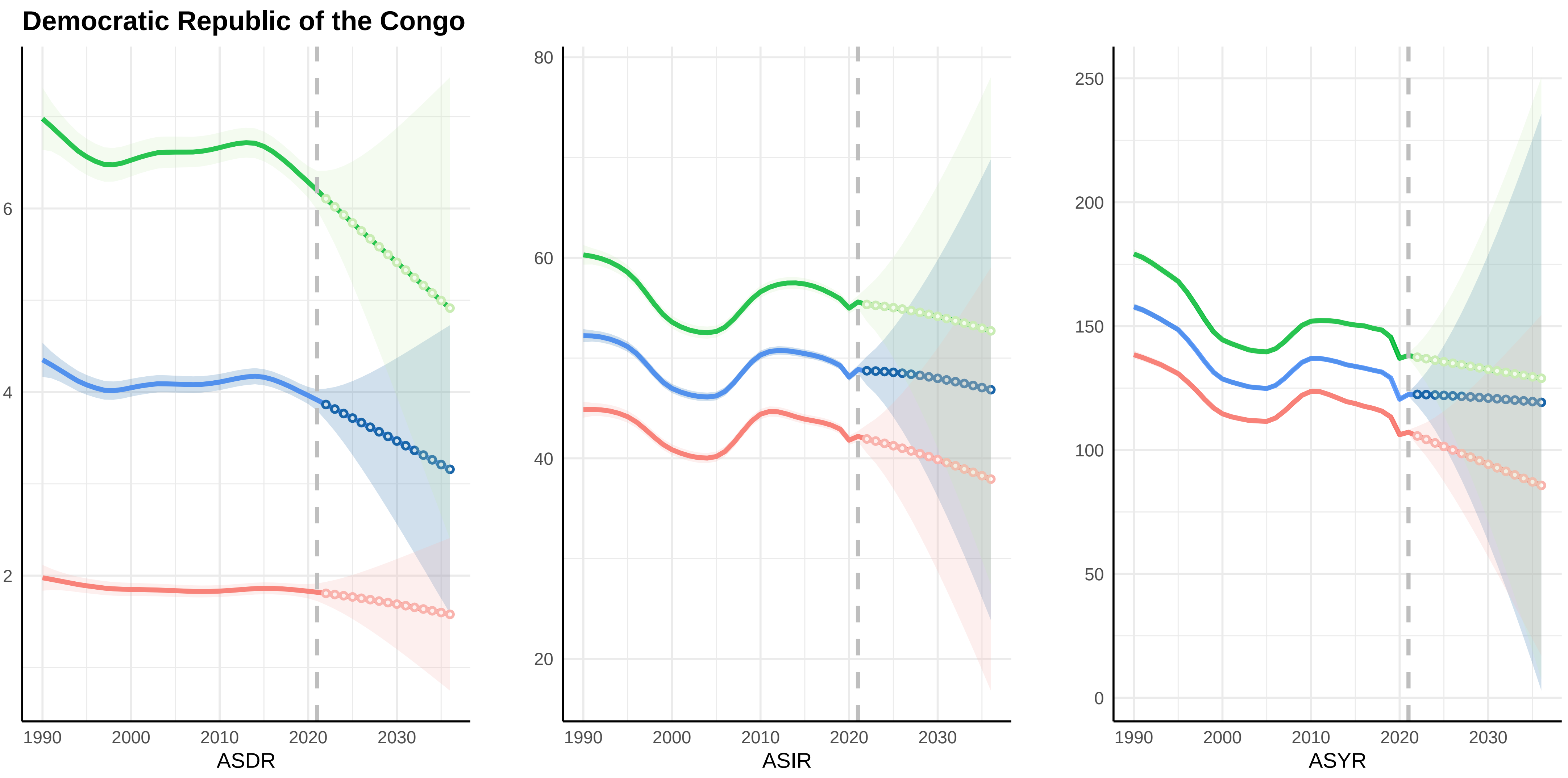

Democratic Republic of the Congo
6
4
2
1990
2000
2010
2020
2030
ASDR
80
60
40
20
1990
2000
2010
2020
2030
ASIR
250
200
150
100
50
0
1990
2000
2010
2020
2030
ASYR

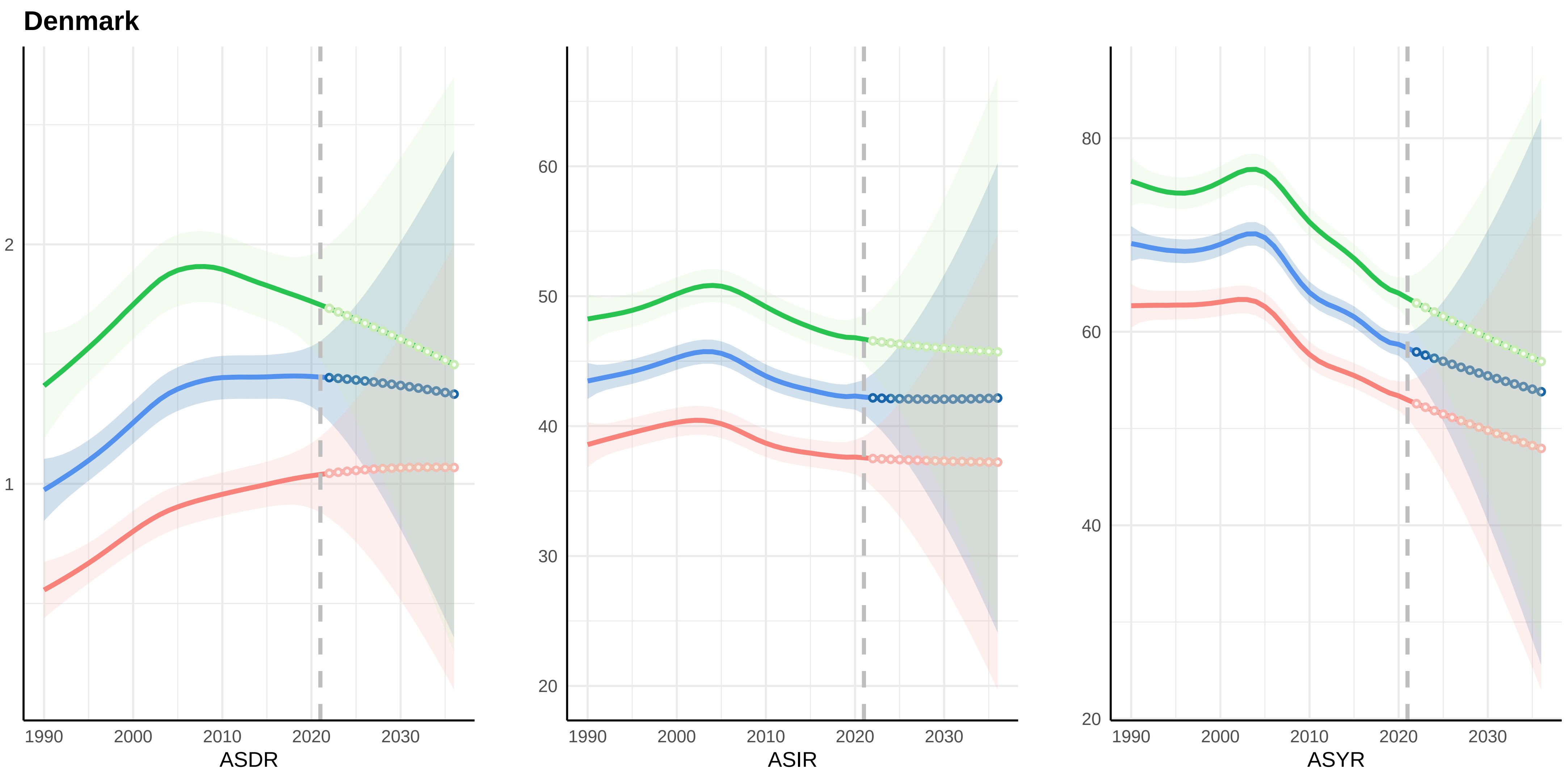
Denmark
ASDR
ASIR
ASYR
2
1
60
50
40
30
20
80
60
40
20
1990
2000
2010
2020
2030

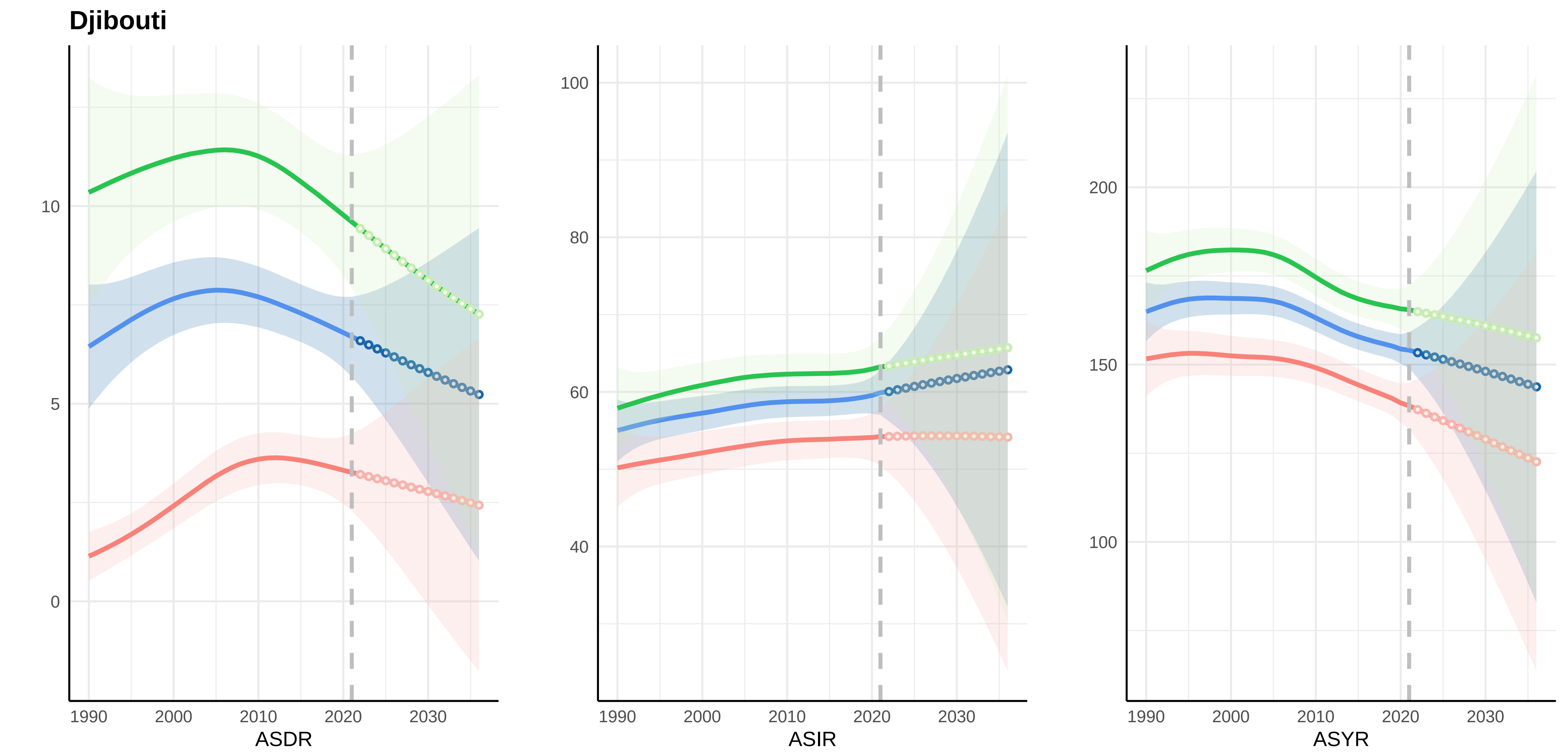
Djibouti
10
5
0
1990
2000
2010
2020
2030
ASDR
100
80
60
40
1990
2000
2010
2020
2030
ASIR
200
150
100
1990
2000
2010
2020
2030
ASYR

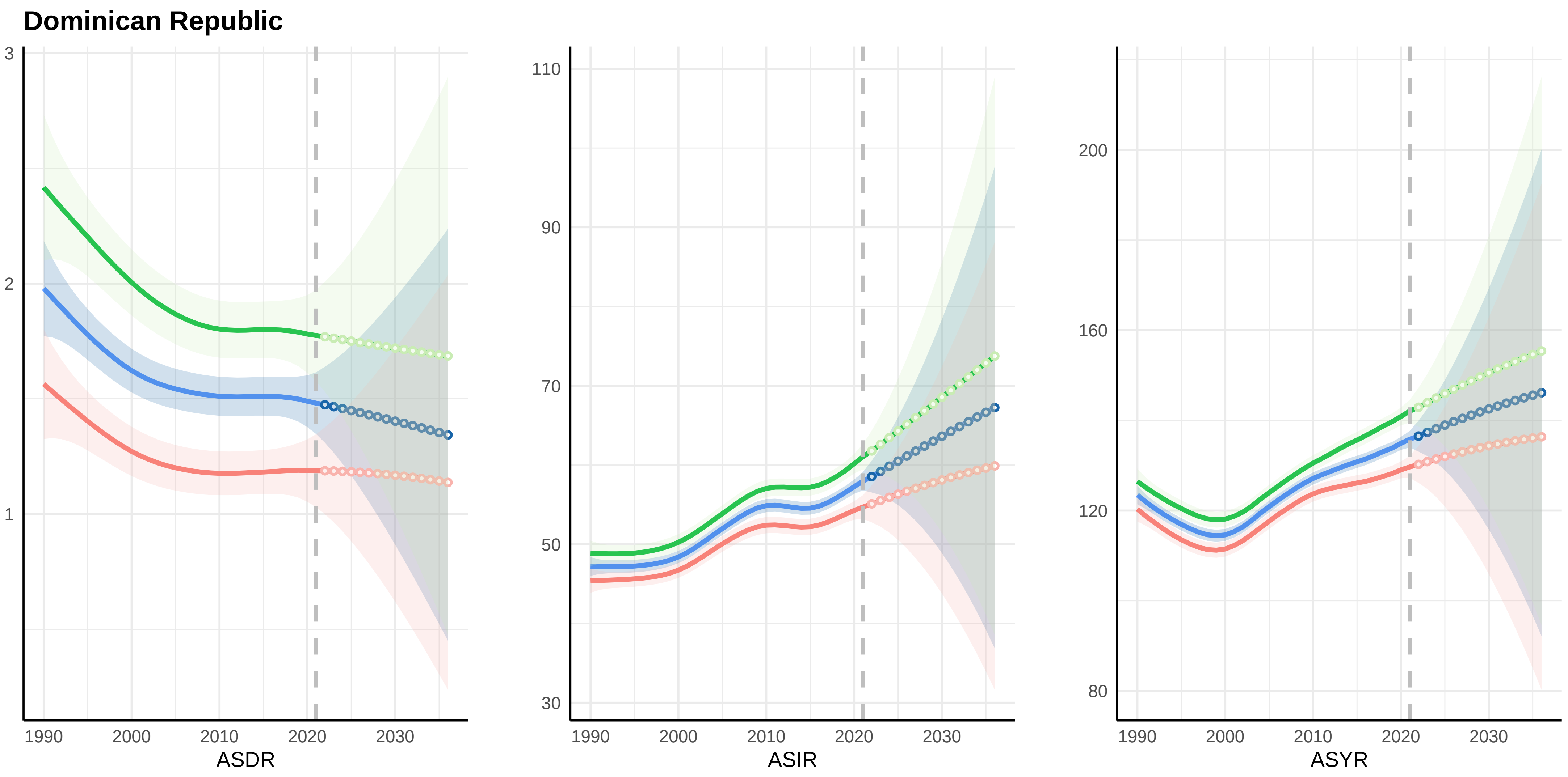

Dominican Republic
3
2
1
1990
2000
2010
2020
2030
ASDR
110
90
70
50
30
1990
2000
2010
2020
2030
ASIR
200
160
120
80
1990
2000
2010
2020
2030
ASYR

Ecuador

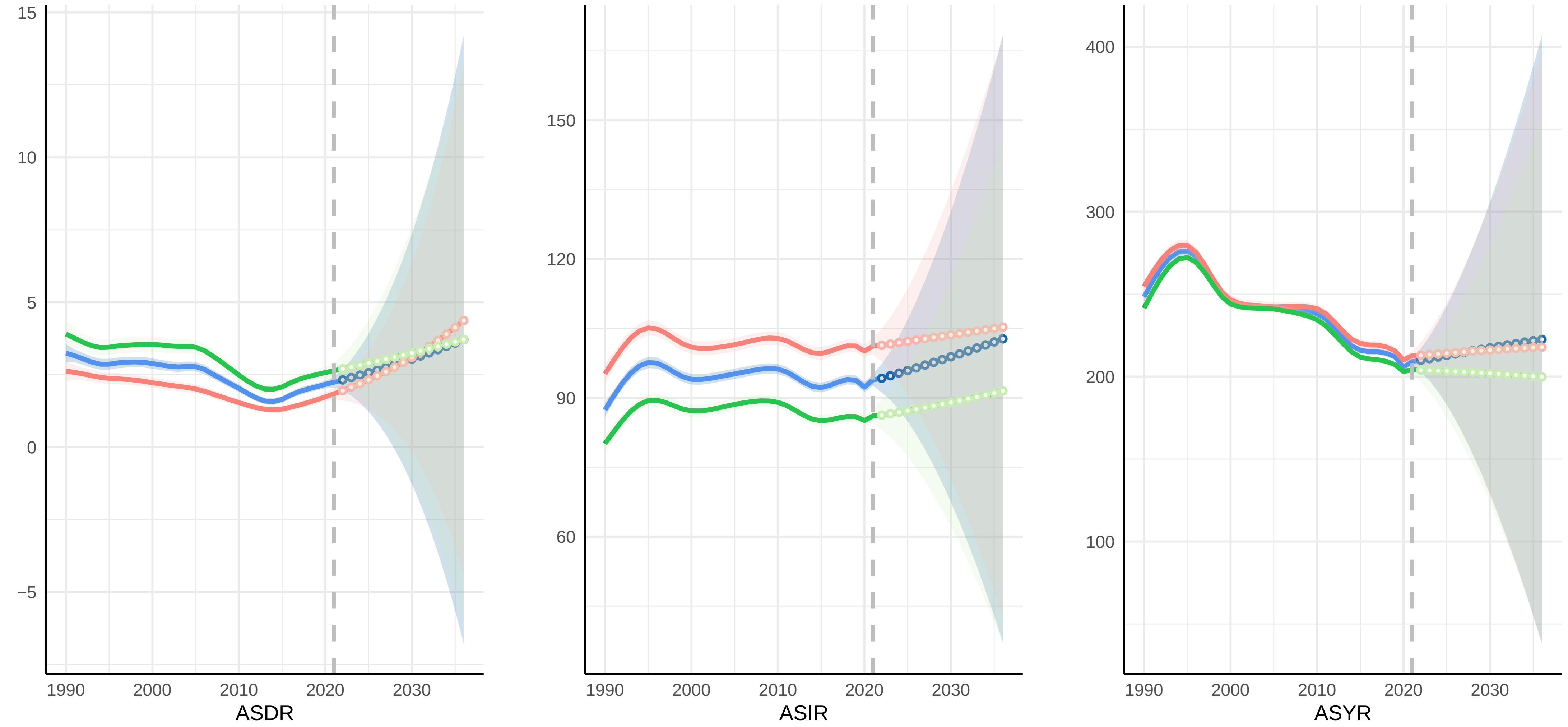
15
10
5
0
−5
1990
2000
2010
2020
2030
ASDR
150
120
90
60
1990
2000
2010
2020
2030
ASIR
400
300
200
100
1990
2000
2010
2020
2030
ASYR

Egypt

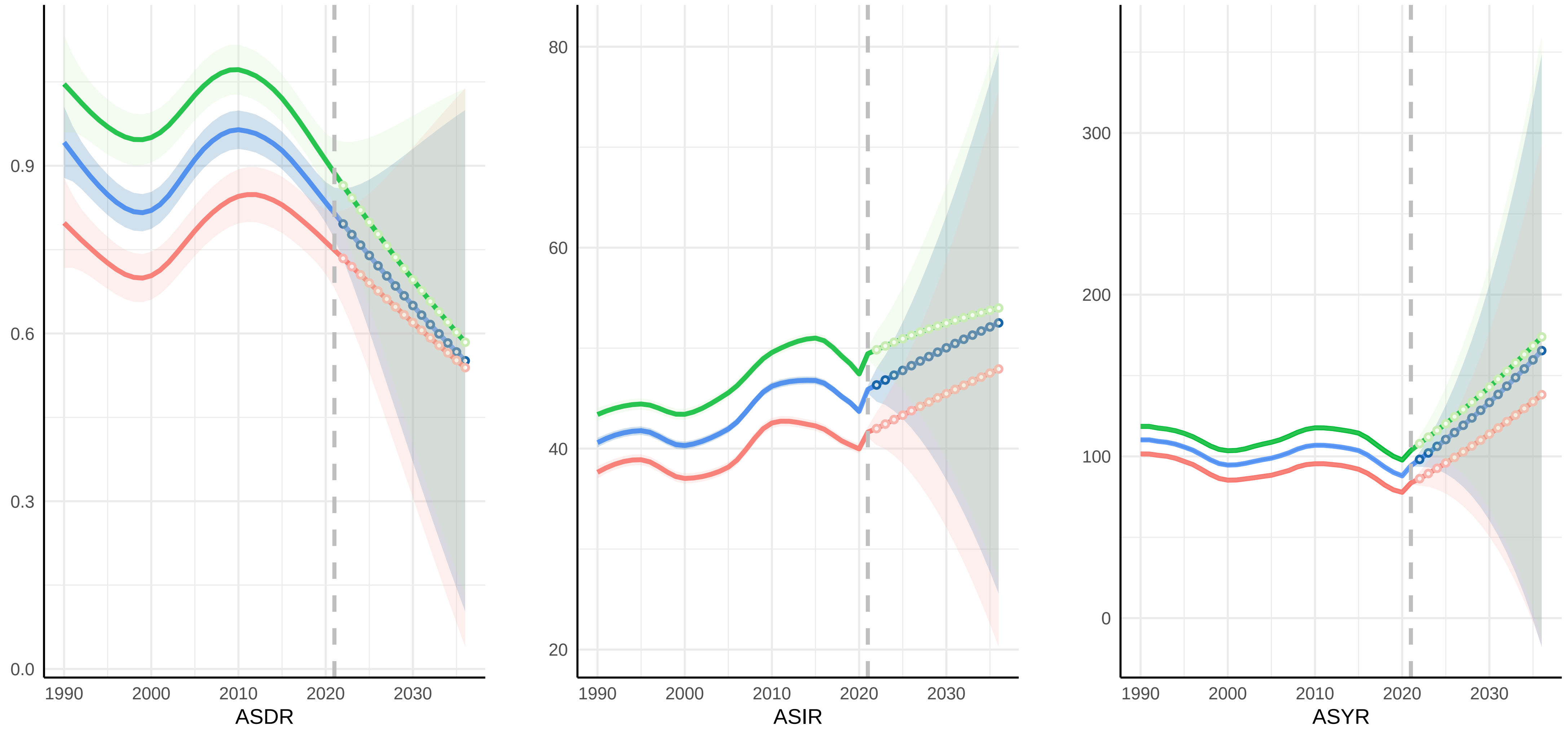
0.9
0.6
0.3
0.0
1990
2000
2010
2020
2030
ASDR
80
60
40
20
1990
2000
2010
2020
2030
ASIR
300
200
100
0
1990
2000
2010
2020
2030
ASYR

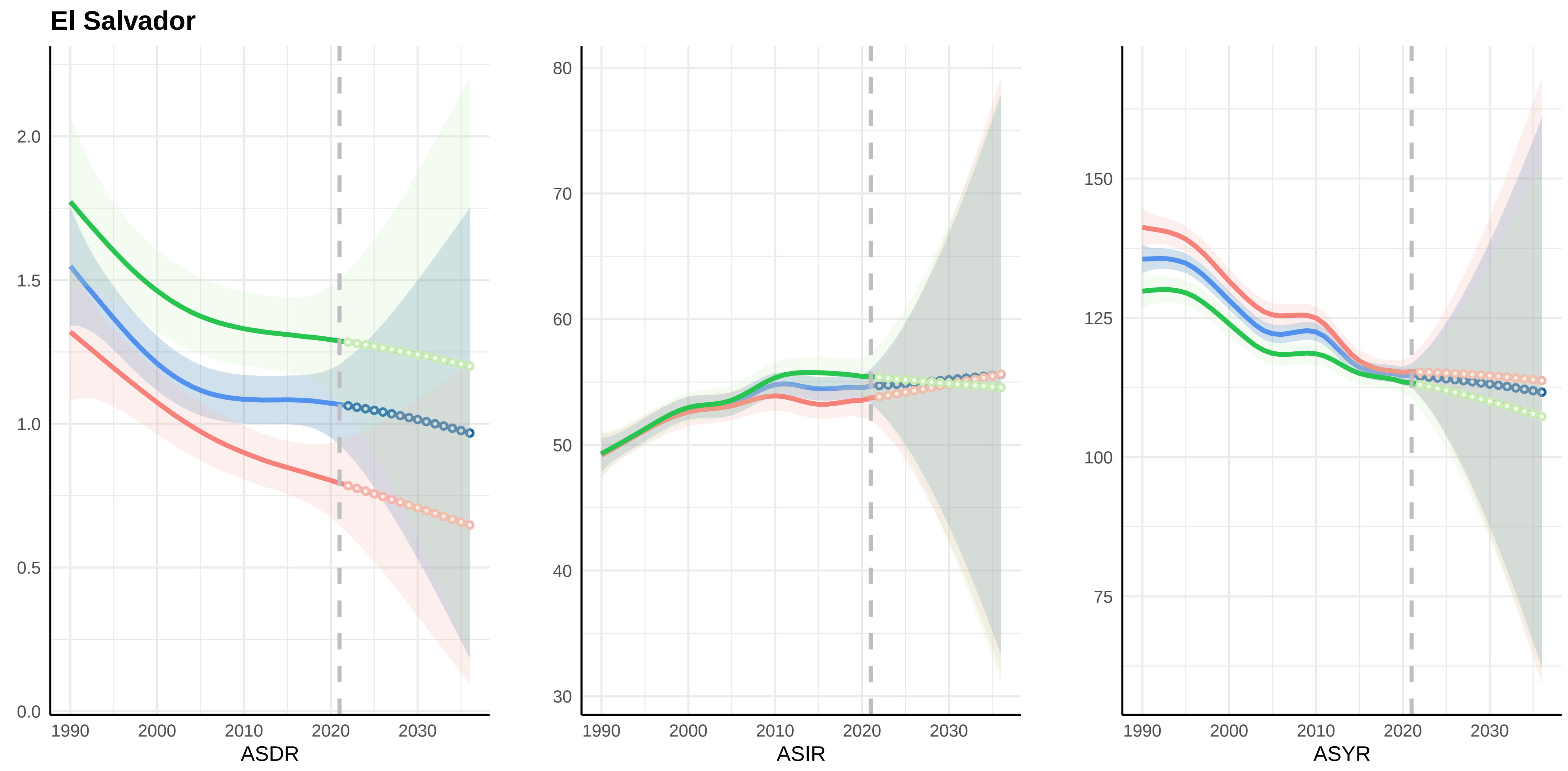

El Salvador
0.0
0.5
1.0
1.5
2.0
1990
2000
2010
2020
2030
ASDR
30
40
50
60
70
80
1990
2000
2010
2020
2030
ASIR
75
100
125
150
1990
2000
2010
2020
2030
ASYR

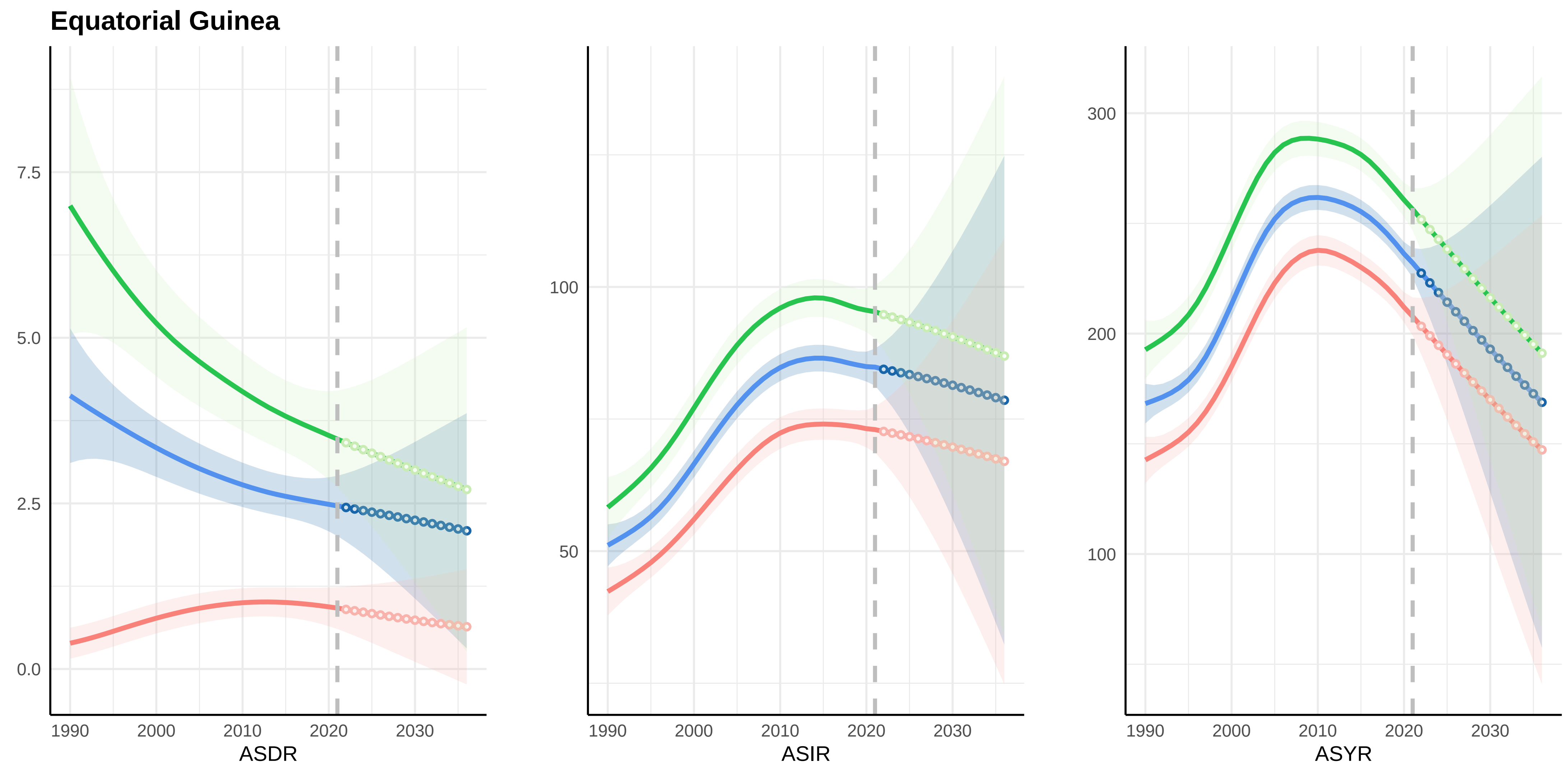

Equatorial Guinea
7.5
5.0
2.5
0.0
1990
2000
2010
2020
2030
ASDR
100
50
ASIR
300
200
100
ASYR

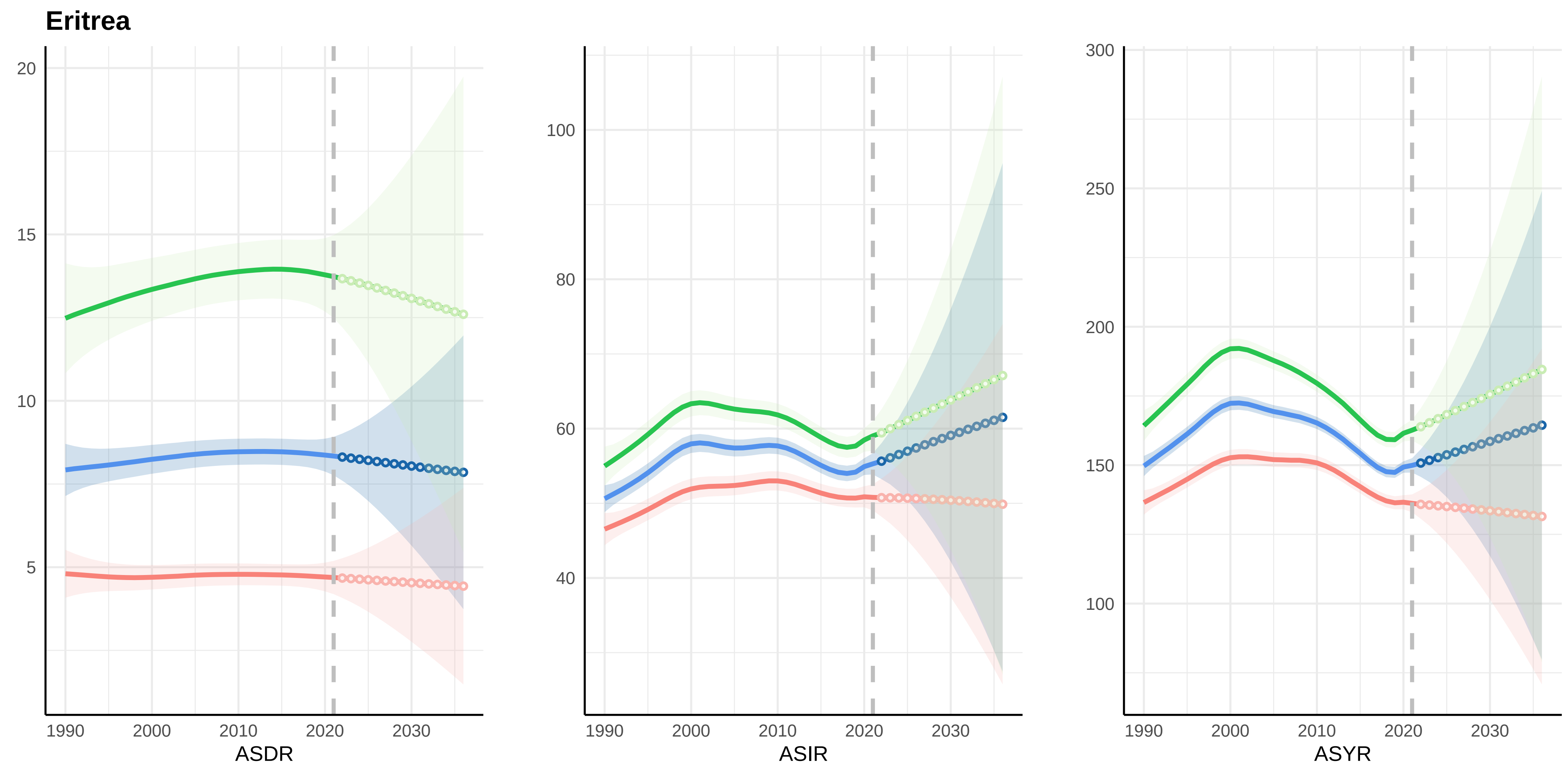

Eritrea
20
15
10
5
1990
2000
2010
2020
2030
ASDR
100
80
60
40
1990
2000
2010
2020
2030
ASIR
300
250
200
150
100
1990
2000
2010
2020
2030
ASYR

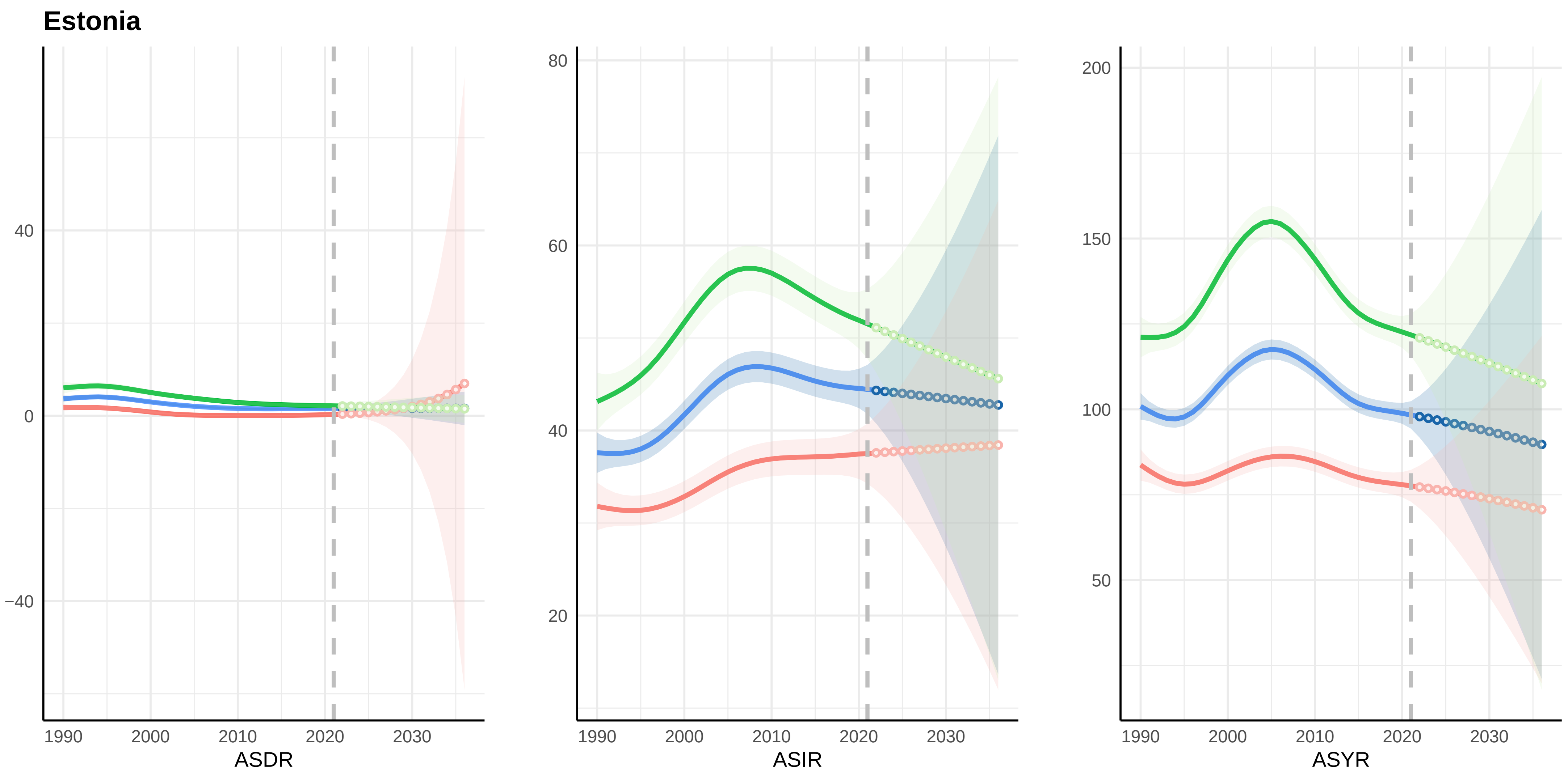

Estonia
40
0
−40
1990
2000
2010
2020
2030
ASDR
80
60
40
20
1990
2000
2010
2020
2030
ASIR
200
150
100
50
1990
2000
2010
2020
2030
ASYR

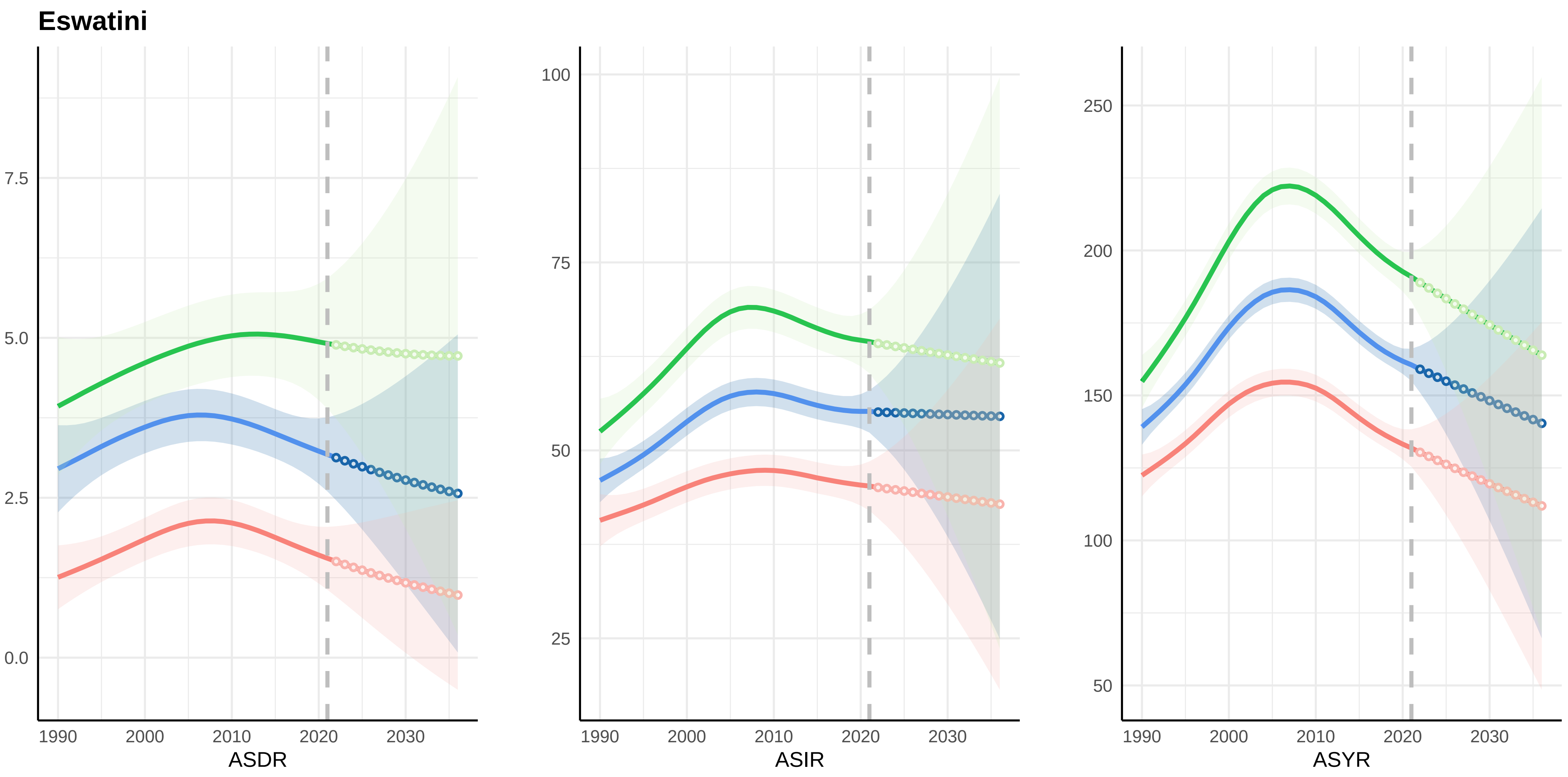

Eswatini
7.5
5.0
2.5
0.0
1990
2000
2010
2020
2030
ASDR
100
75
50
25
ASIR
250
200
150
100
50
ASYR

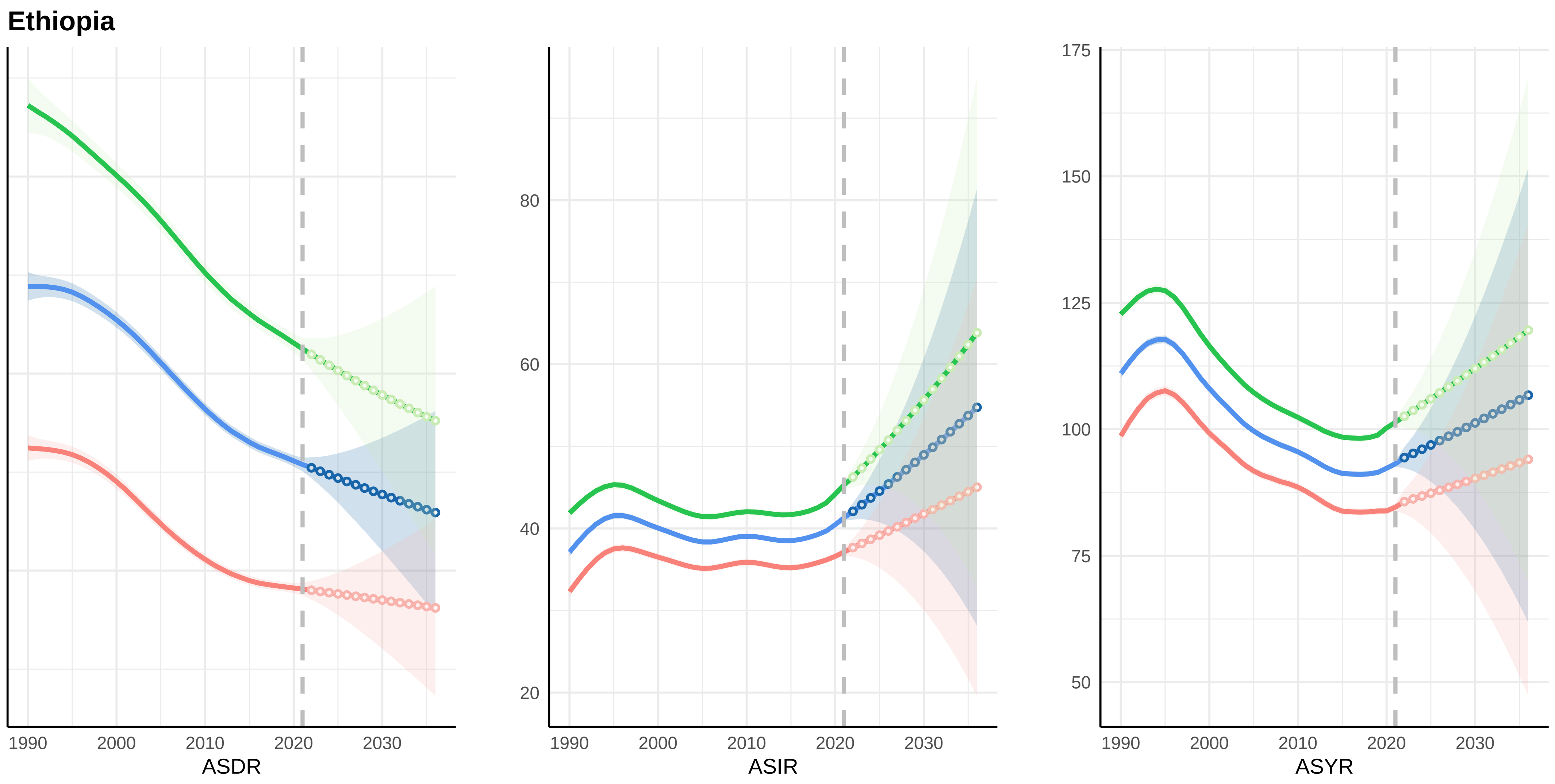

Ethiopia
15
10
5
1990
2000
2010
2020
2030
ASDR
80
60
40
20
1990
2000
2010
2020
2030
ASIR
175
150
125
100
75
50
1990
2000
2010
2020
2030
ASYR

Fiji

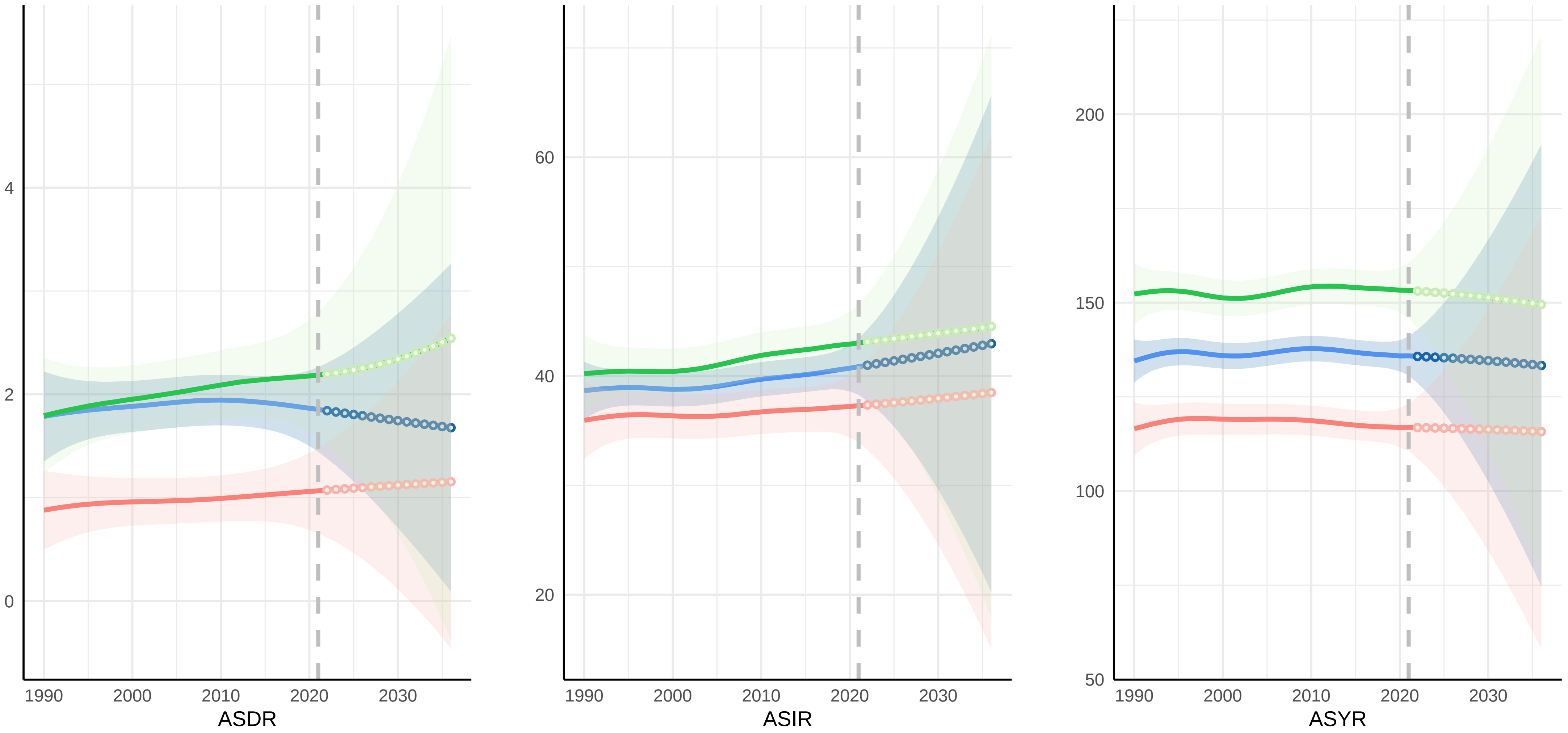

4
2
0
1990
2000
2010
2020
2030
ASDR
60
40
20
1990
2000
2010
2020
2030
ASIR
200
150
100
50
1990
2000
2010
2020
2030
ASYR

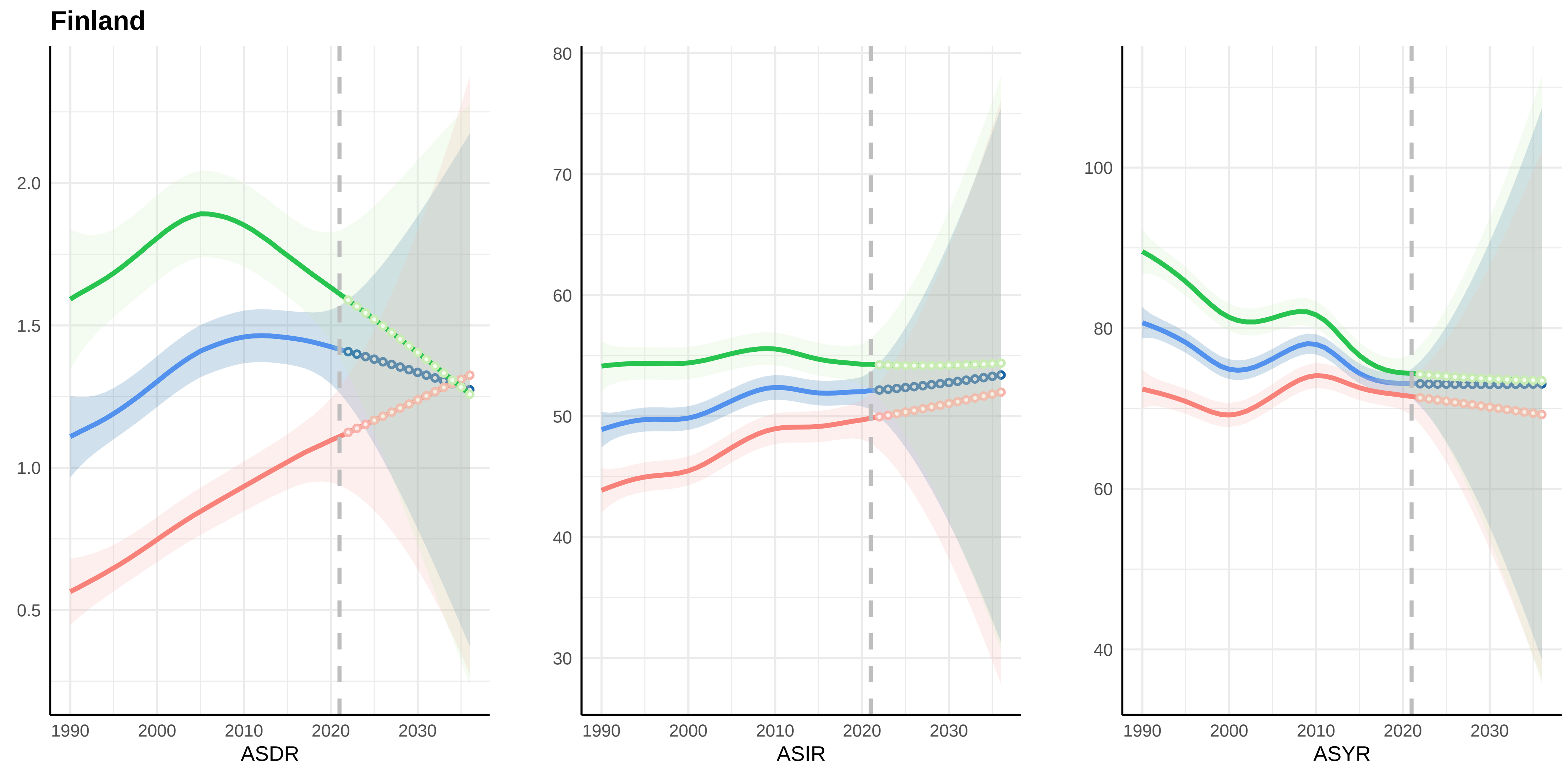

Finland
2.0
1.5
1.0
0.5
1990
2000
2010
2020
2030
ASDR
80
70
60
50
40
30
ASIR
100
80
60
40
ASYR

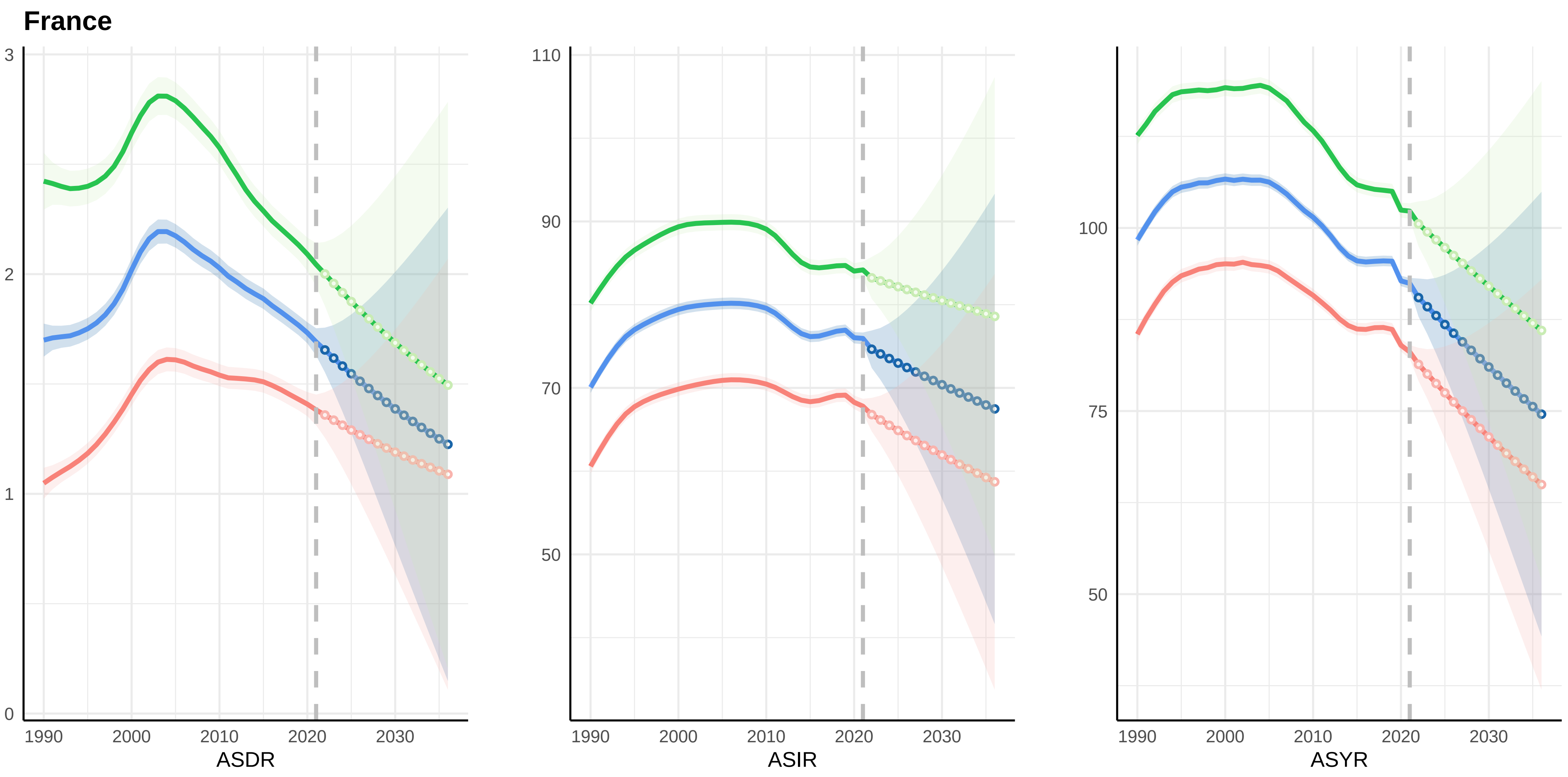

France
ASDR
ASIR
ASYR
0
1
2
3
50
70
90
110
50
75
100
1990
2000
2010
2020
2030

Gabon

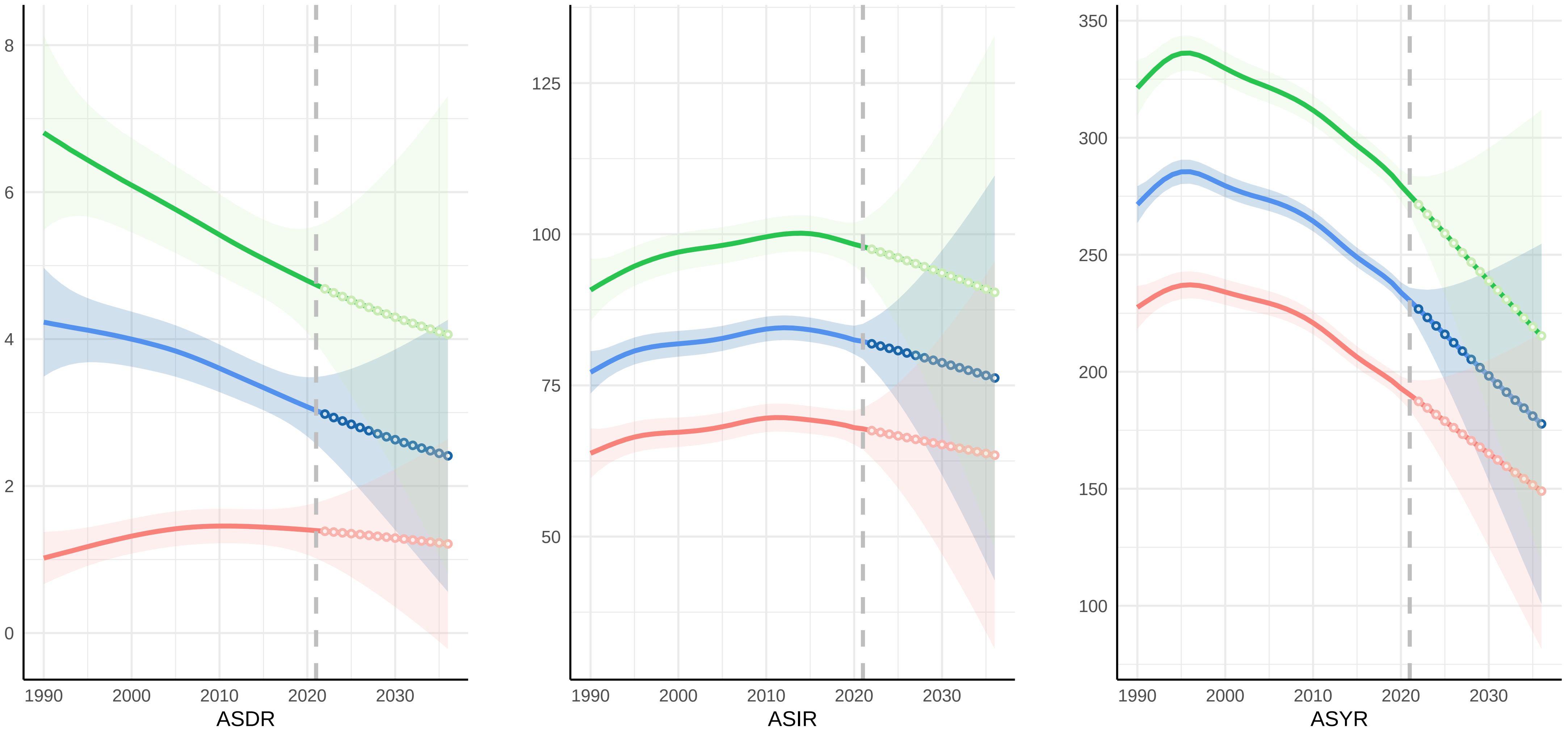

8
6
4
2
0
1990
2000
2010
2020
2030
ASDR
125
100
75
50
1990
2000
2010
2020
2030
ASIR
350
300
250
200
150
100
1990
2000
2010
2020
2030
ASYR

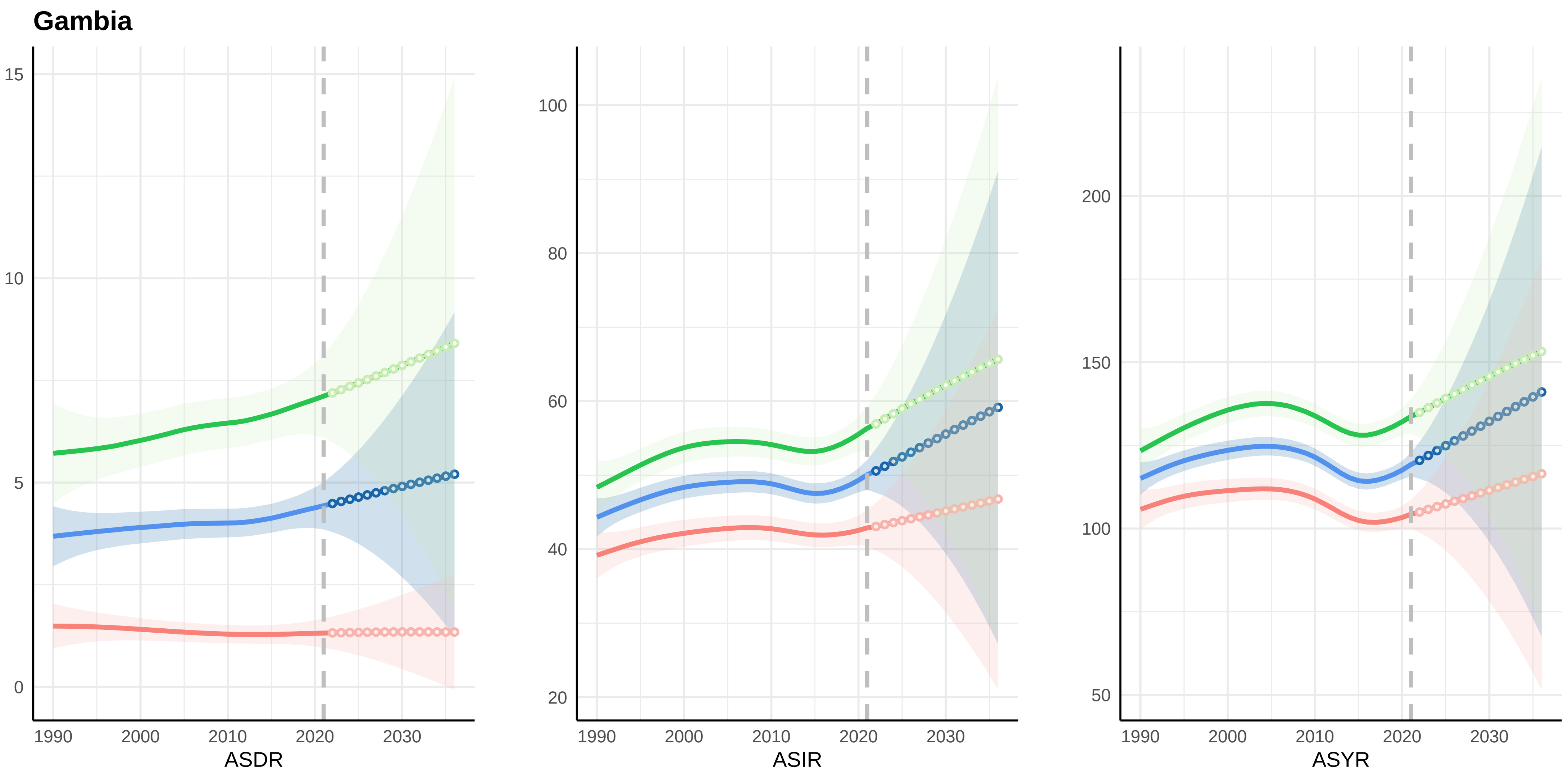

Gambia
15
10
5
0
1990
2000
2010
2020
2030
ASDR
100
80
60
40
20
1990
2000
2010
2020
2030
ASIR
200
150
100
50
1990
2000
2010
2020
2030
ASYR

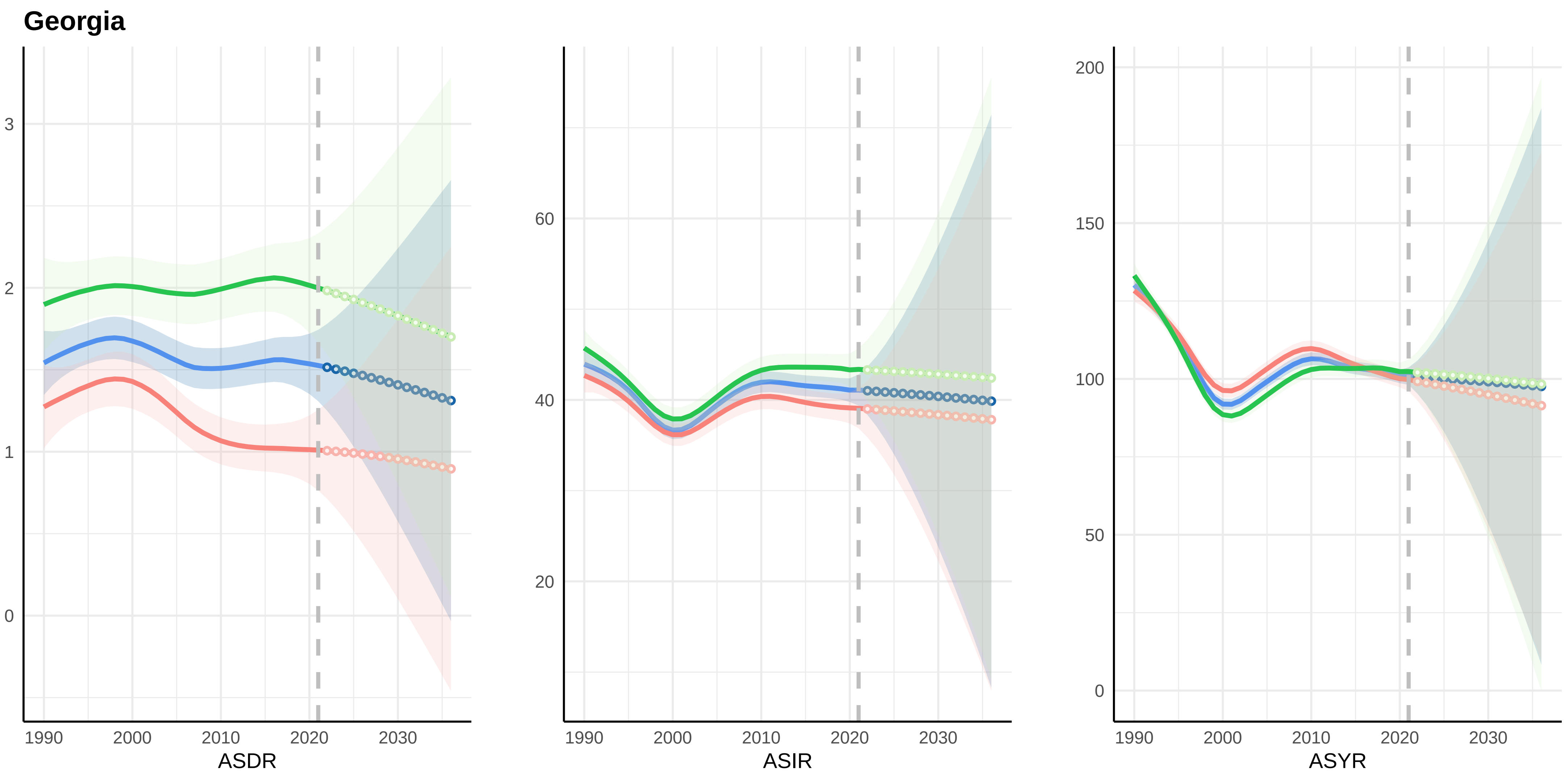

Georgia
3
2
1
0
1990
2000
2010
2020
2030
ASDR
60
40
20
ASIR
200
150
100
50
ASYR

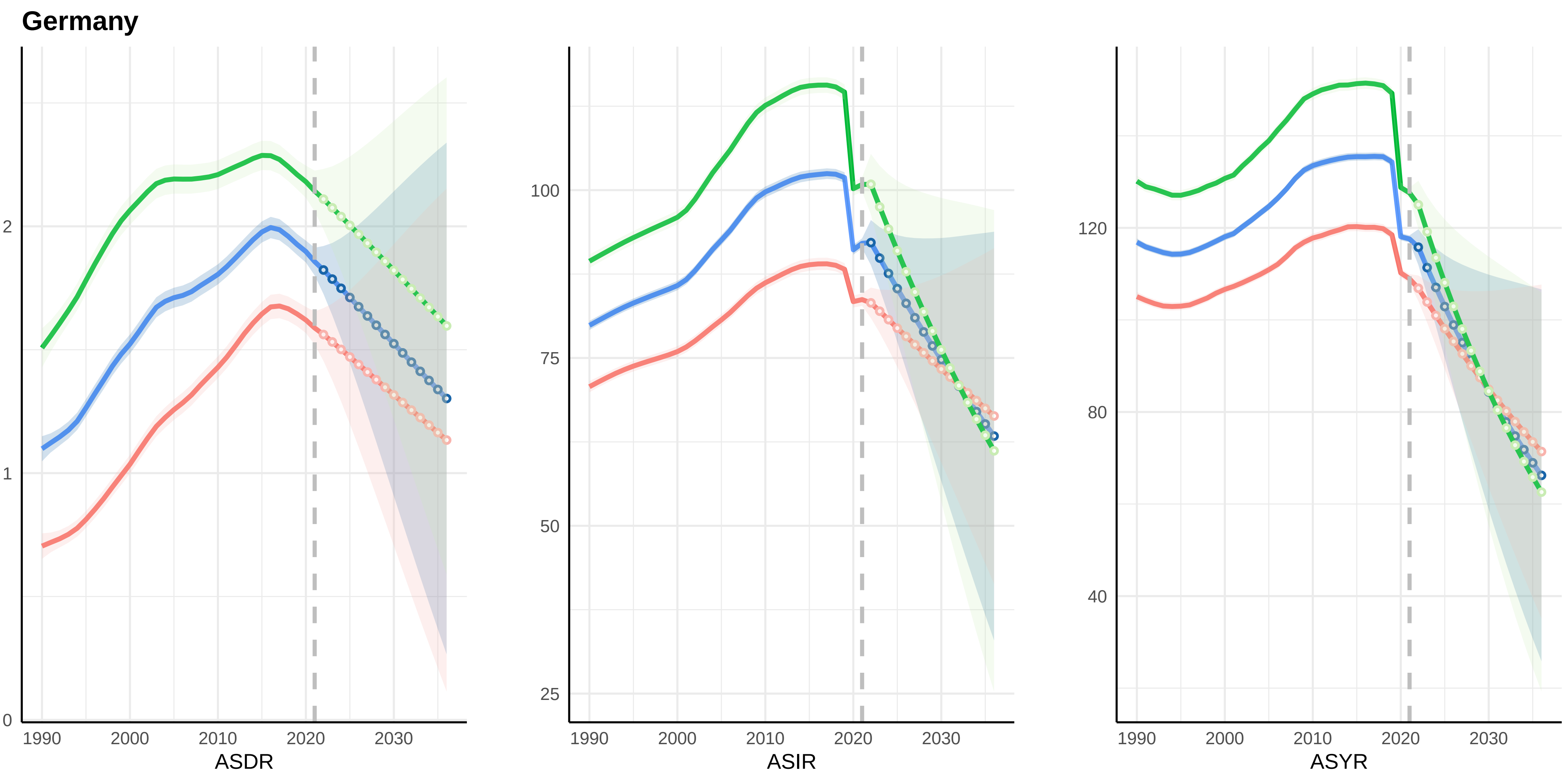

Germany
2
1
0
1990
2000
2010
2020
2030
ASDR
100
75
50
25
ASIR
120
80
40
ASYR

Ghana

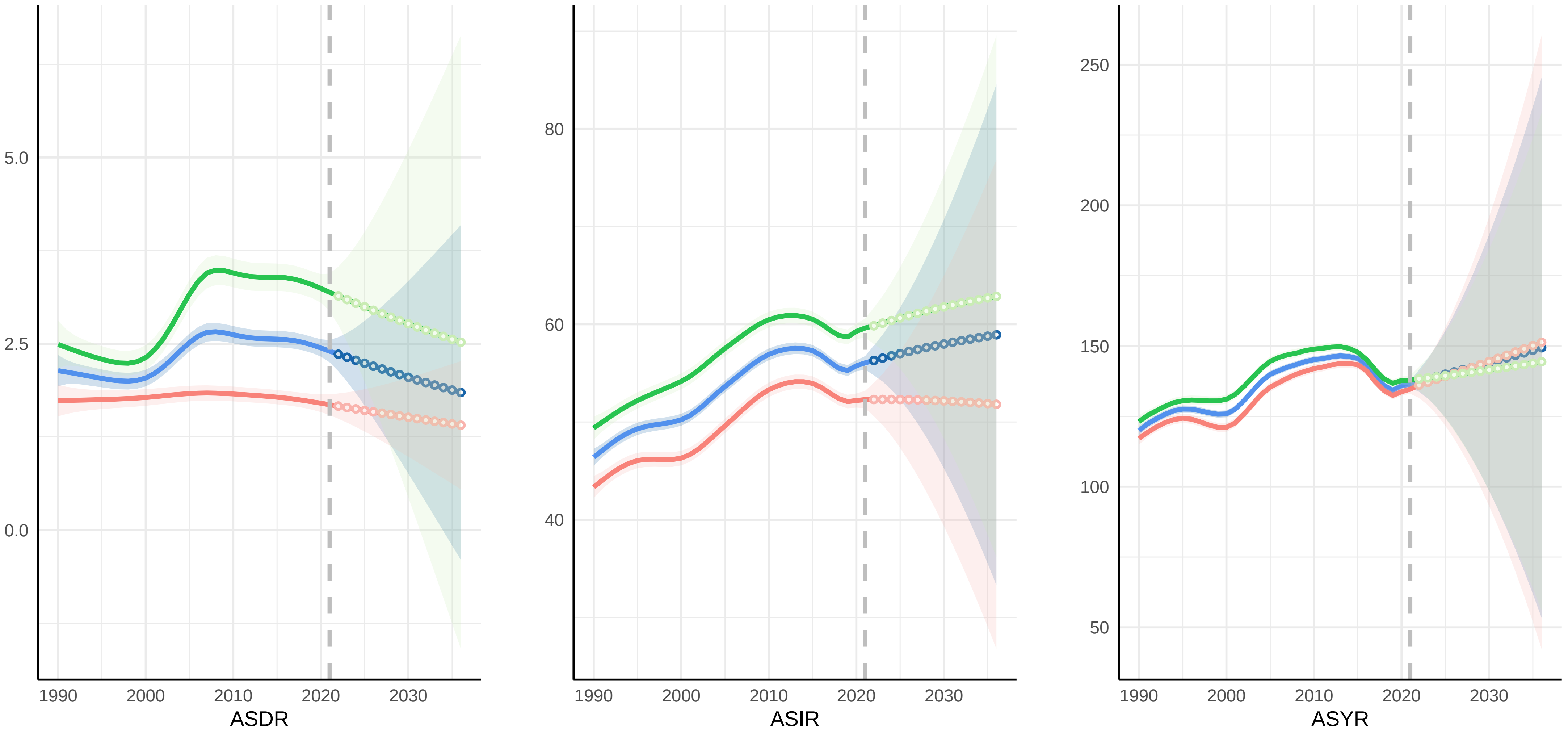

5.0
2.5
0.0
1990
2000
2010
2020
2030
ASDR
80
60
40
1990
2000
2010
2020
2030
ASIR
250
200
150
100
50
1990
2000
2010
2020
2030
ASYR

Greece

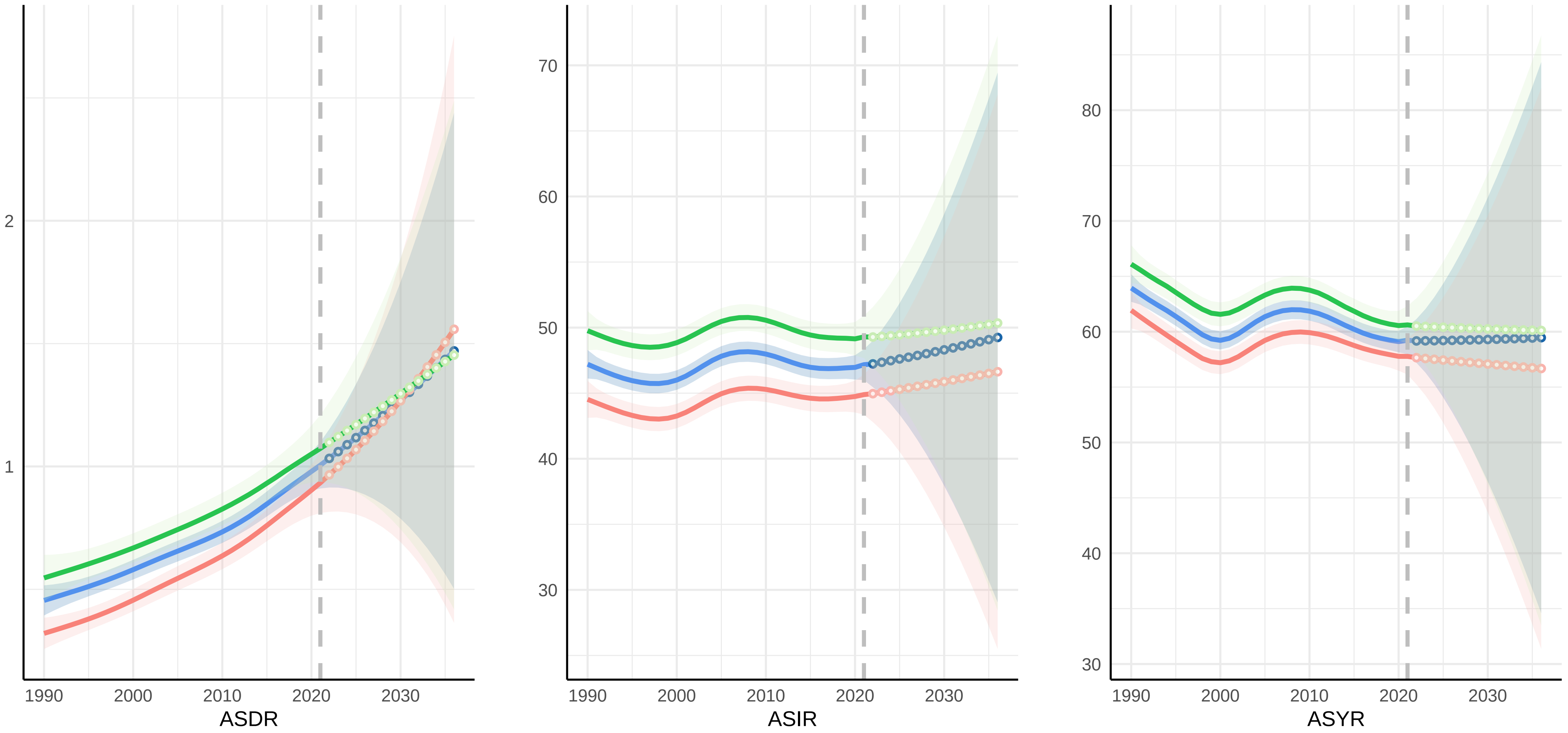

Guatemala

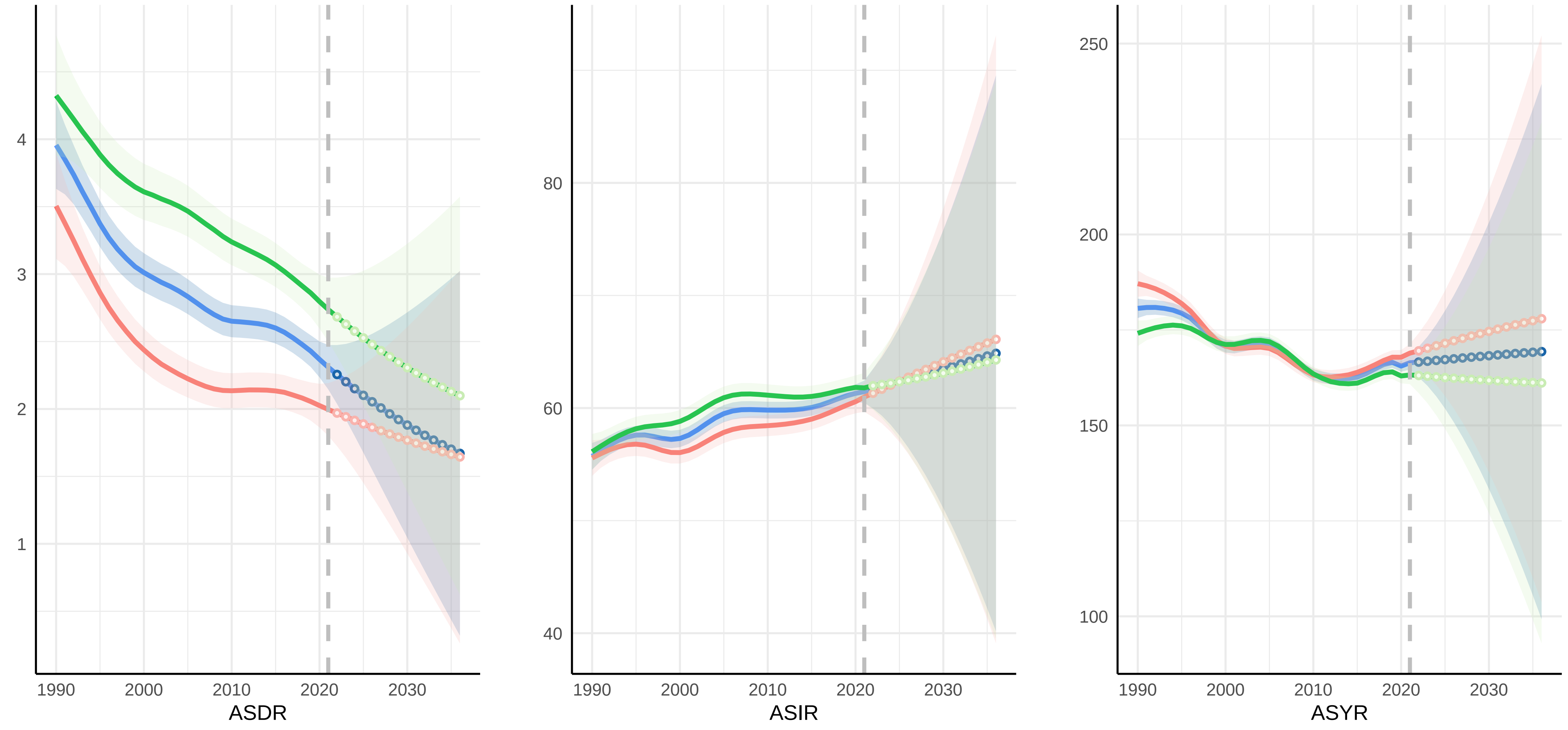

4
3
2
1
1990
2000
2010
2020
2030
ASDR
80
60
40
1990
2000
2010
2020
2030
ASIR
250
200
150
100
1990
2000
2010
2020
2030
ASYR

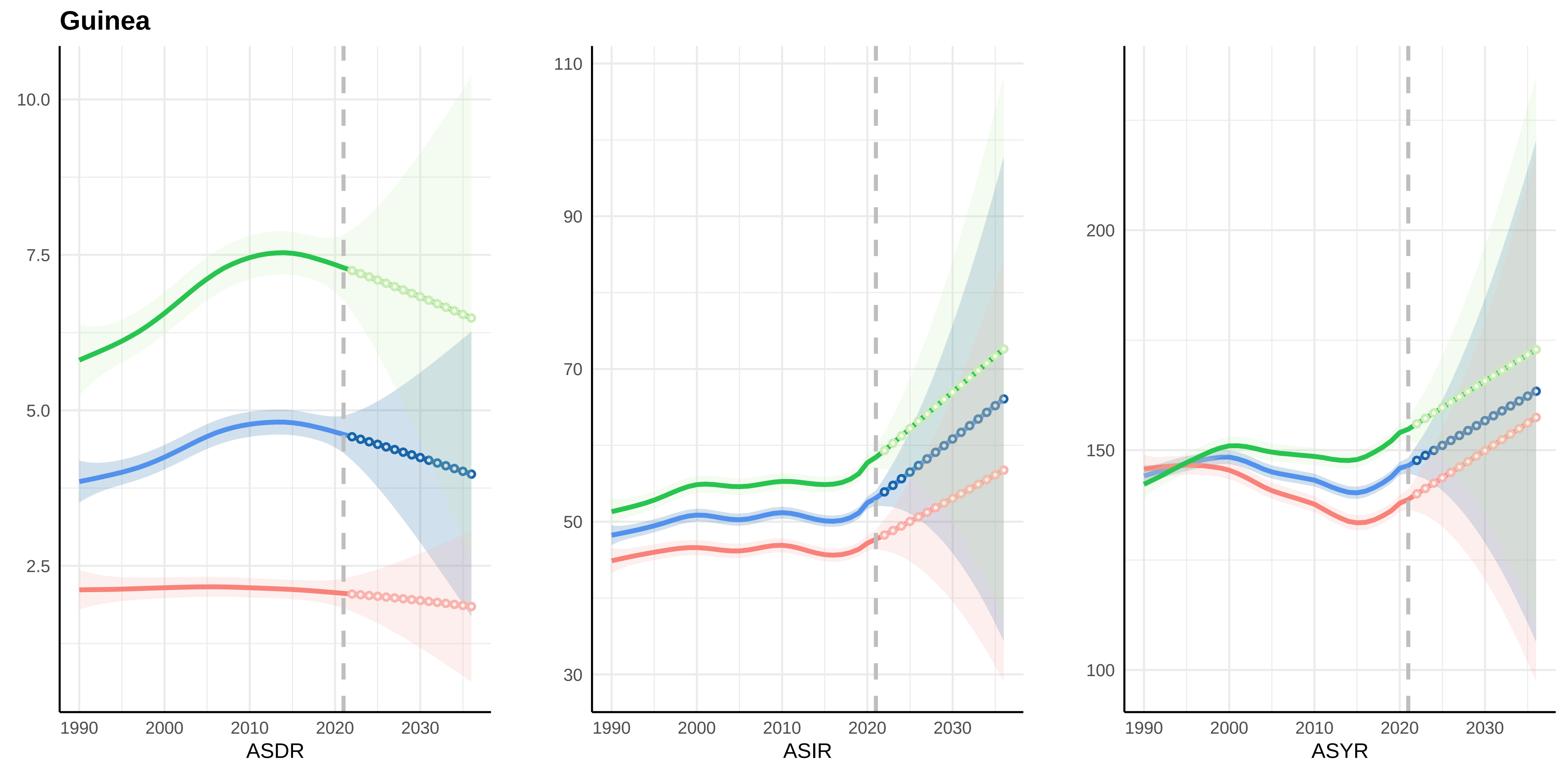

Guinea
10.0
7.5
5.0
2.5
1990
2000
2010
2020
2030
ASDR
110
90
70
50
30
1990
2000
2010
2020
2030
ASIR
200
150
100
1990
2000
2010
2020
2030
ASYR

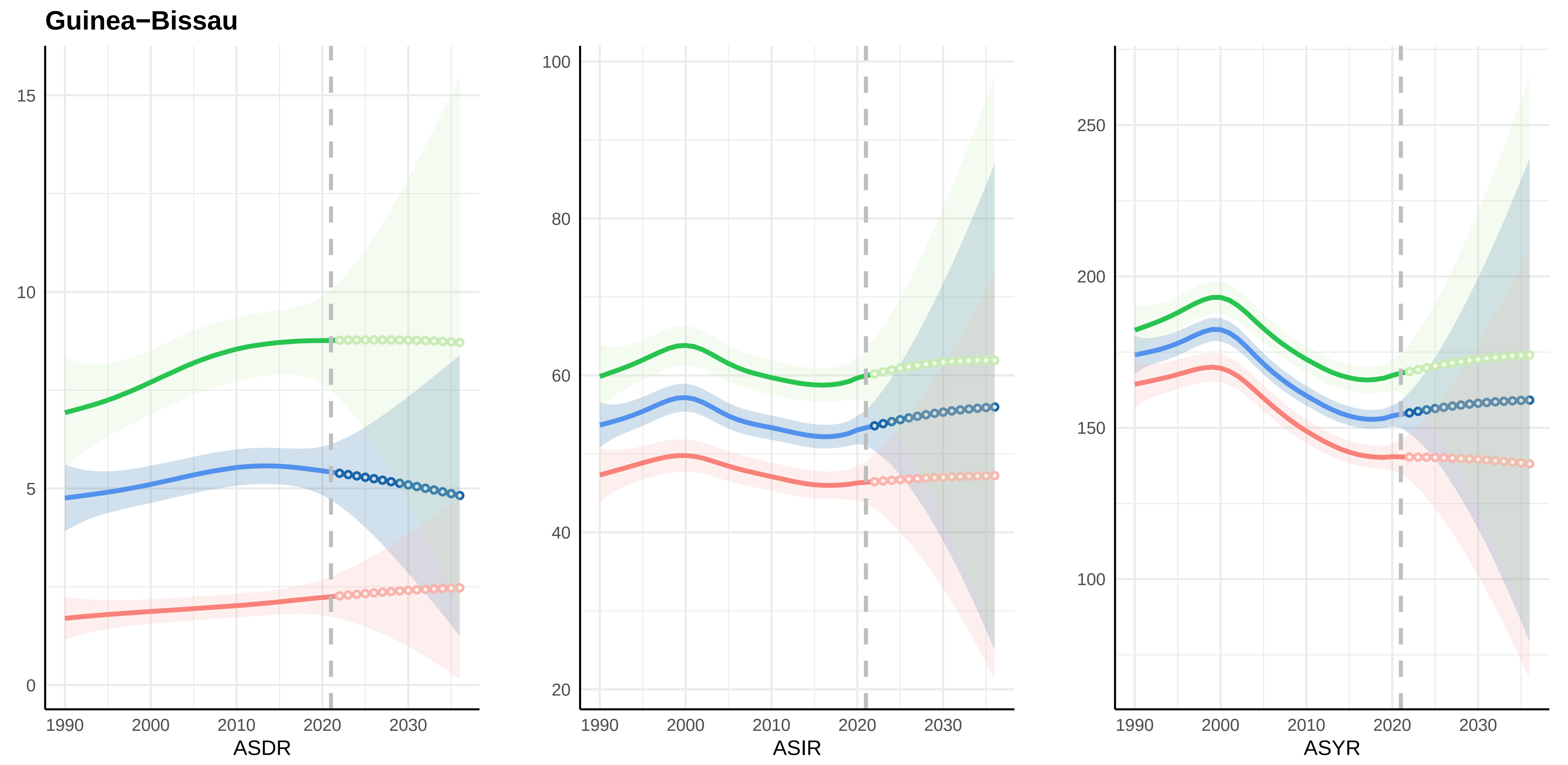

Guinea−Bissau
15
10
5
0
1990
2000
2010
2020
2030
ASDR
100
80
60
40
20
ASIR
250
200
150
100
ASYR

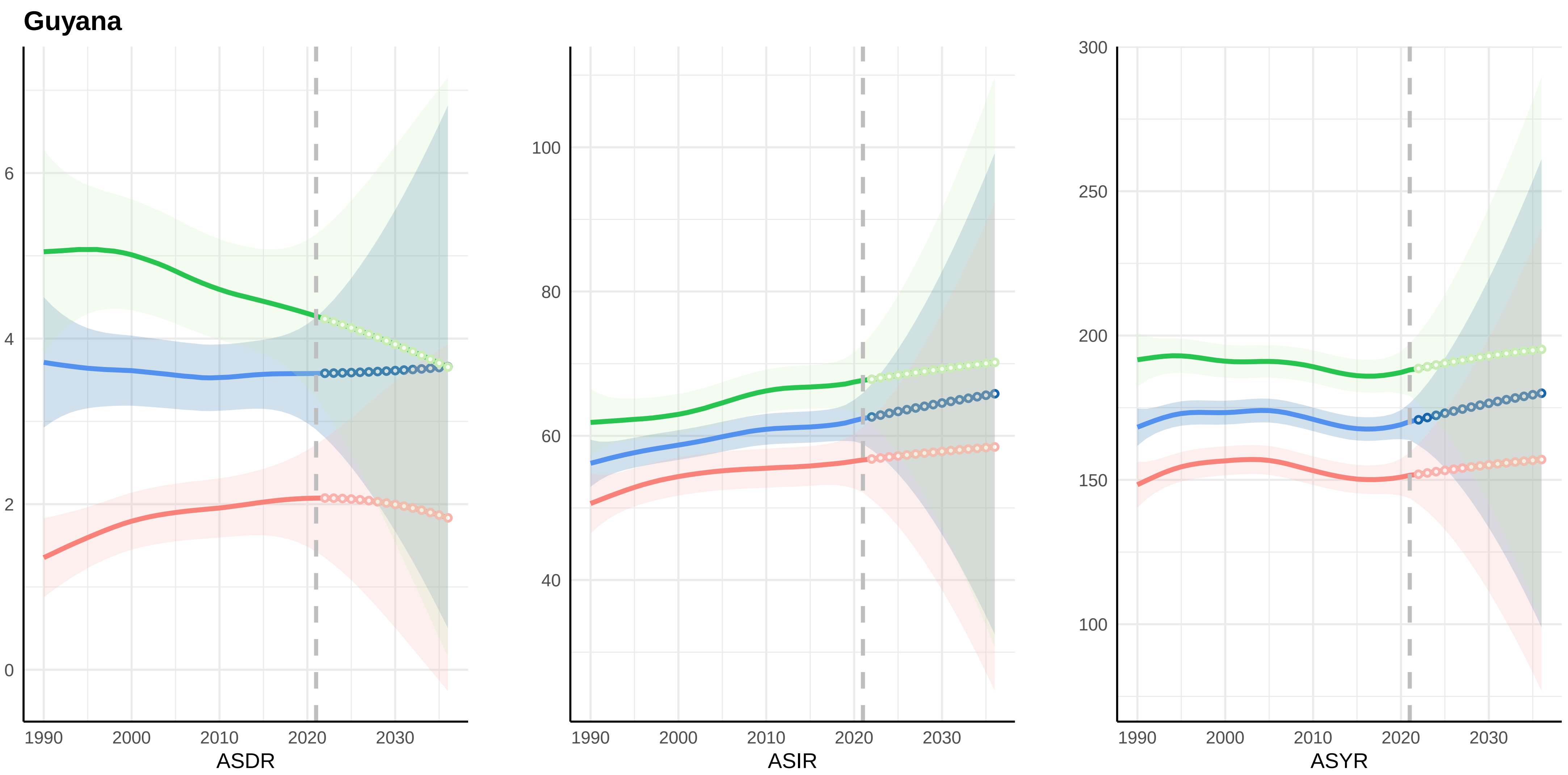
Guyana
6
4
2
0
1990
2000
2010
2020
2030
ASDR
100
80
60
40
1990
2000
2010
2020
2030
ASIR
300
250
200
150
100
1990
2000
2010
2020
2030
ASYR

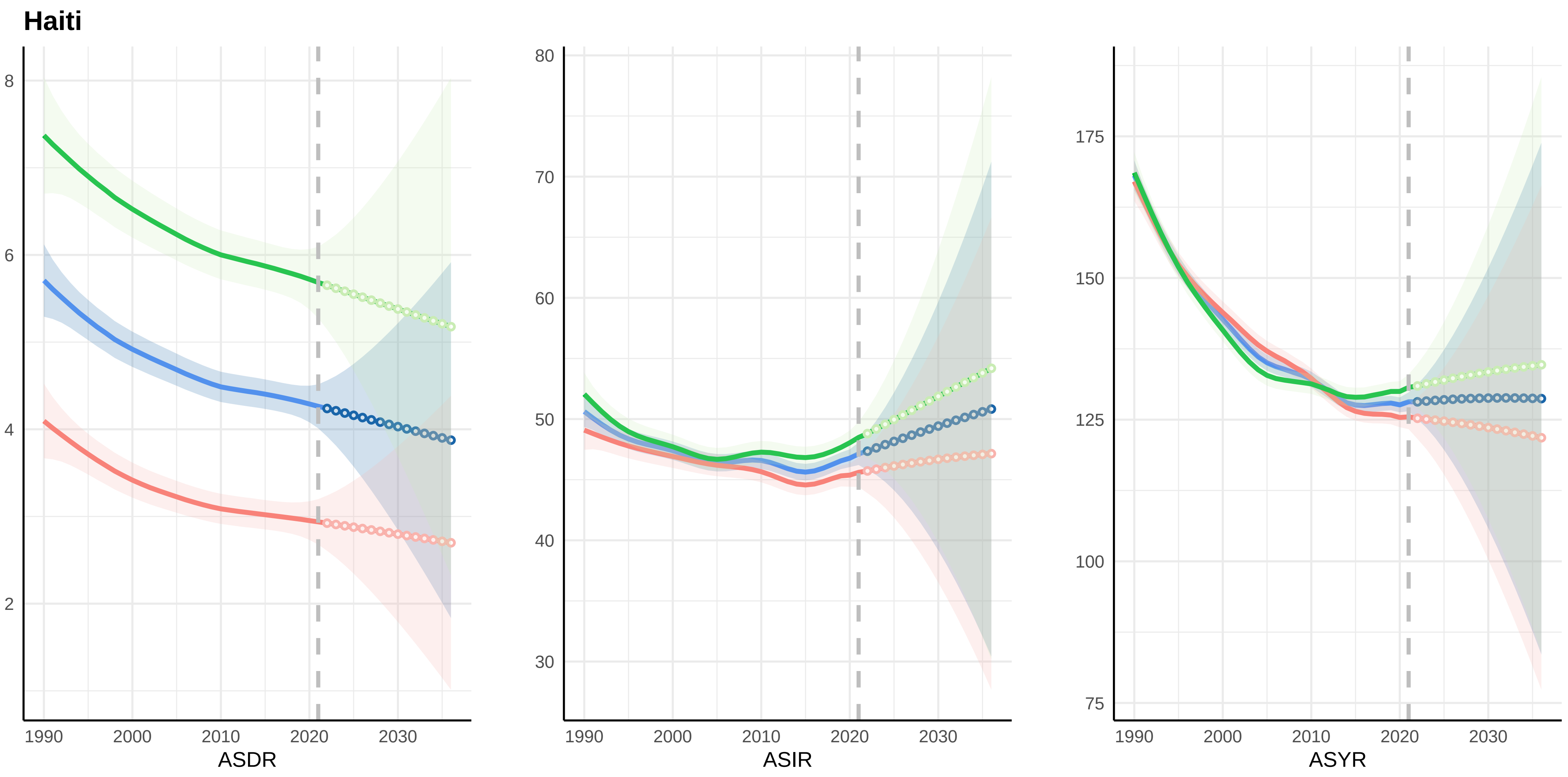

Haiti
8
6
4
2
1990
2000
2010
2020
2030
ASDR
80
70
60
50
40
30
1990
2000
2010
2020
2030
ASIR
175
150
125
100
75
1990
2000
2010
2020
2030
ASYR

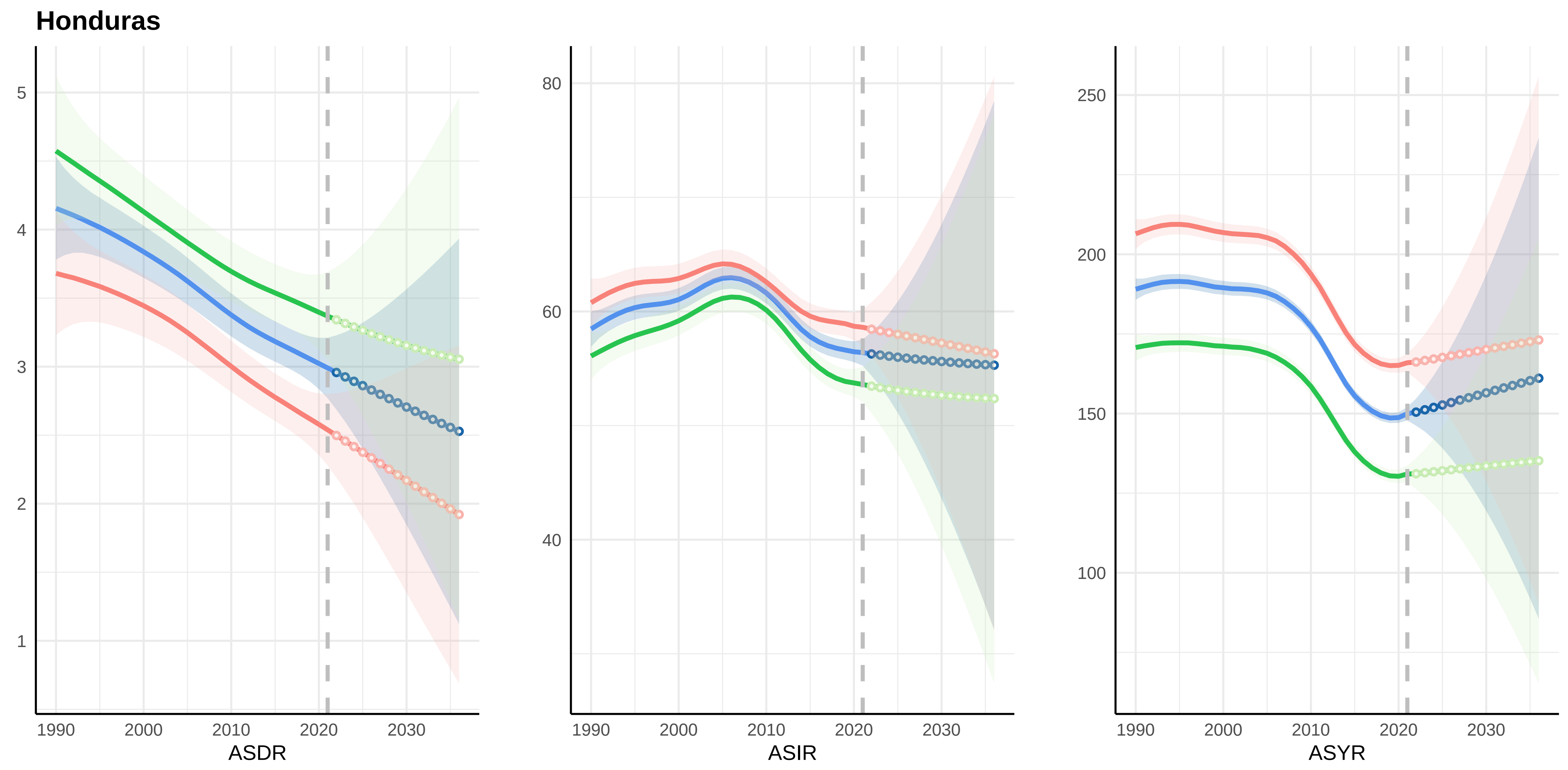

Honduras
5
4
3
2
1
1990
2000
2010
2020
2030
ASDR
80
60
40
1990
2000
2010
2020
2030
ASIR
250
200
150
100
1990
2000
2010
2020
2030
ASYR

Hungary

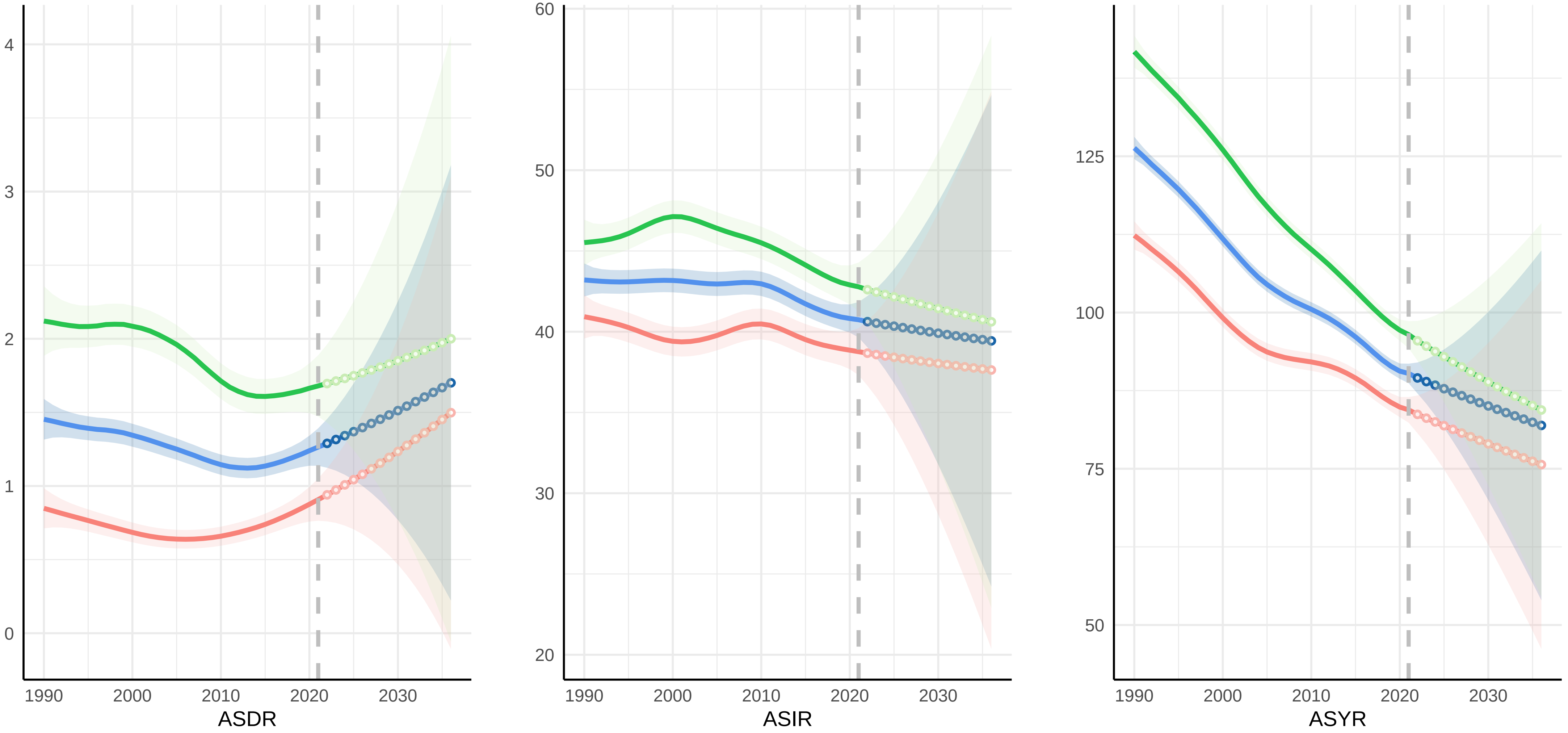

4
3
2
1
0
1990
2000
2010
2020
2030
ASDR
60
50
40
30
20
1990
2000
2010
2020
2030
ASIR
125
100
75
50
1990
2000
2010
2020
2030
ASYR

India

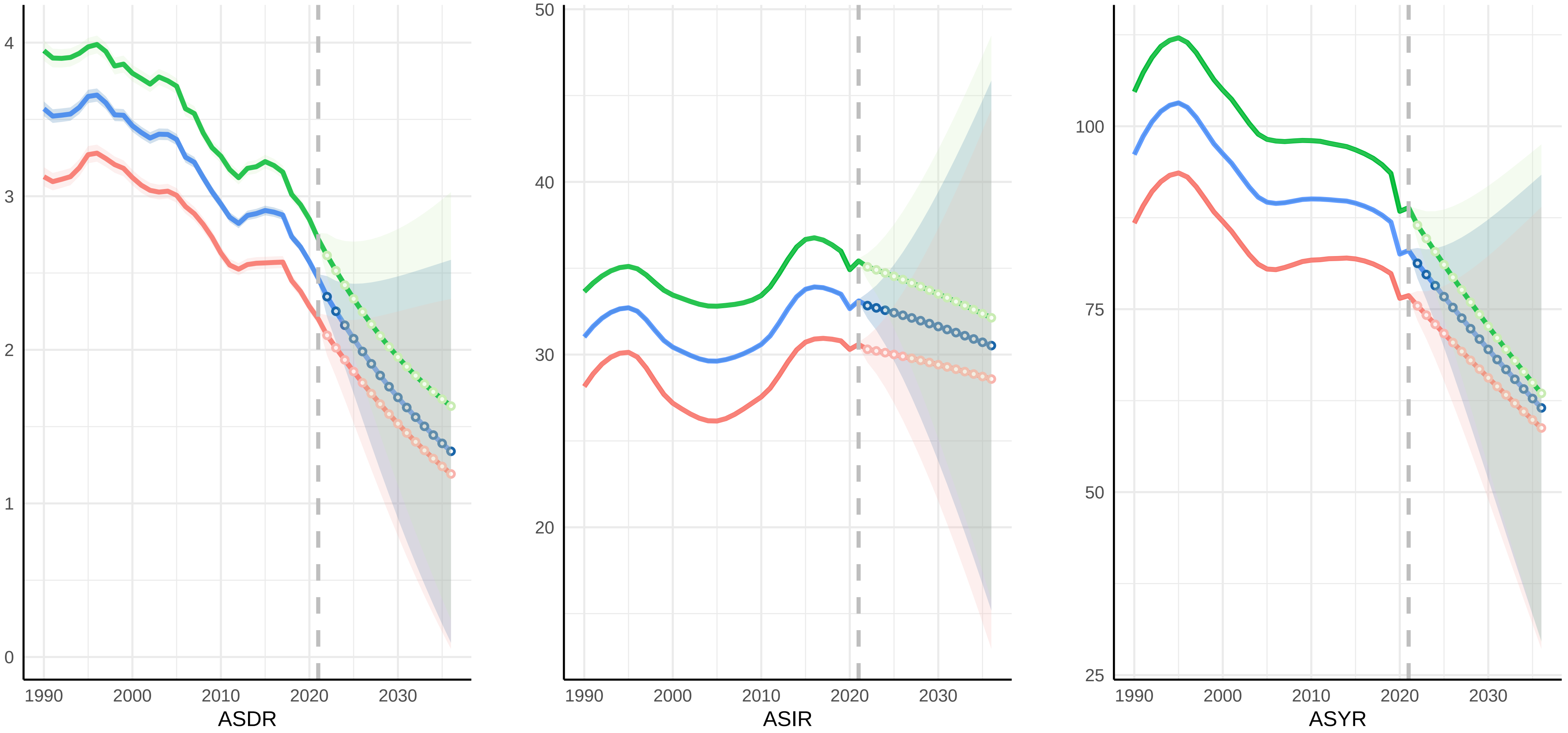

4
3
2
1
0
1990
2000
2010
2020
2030
ASDR
50
40
30
20
ASIR
100
75
50
25
ASYR

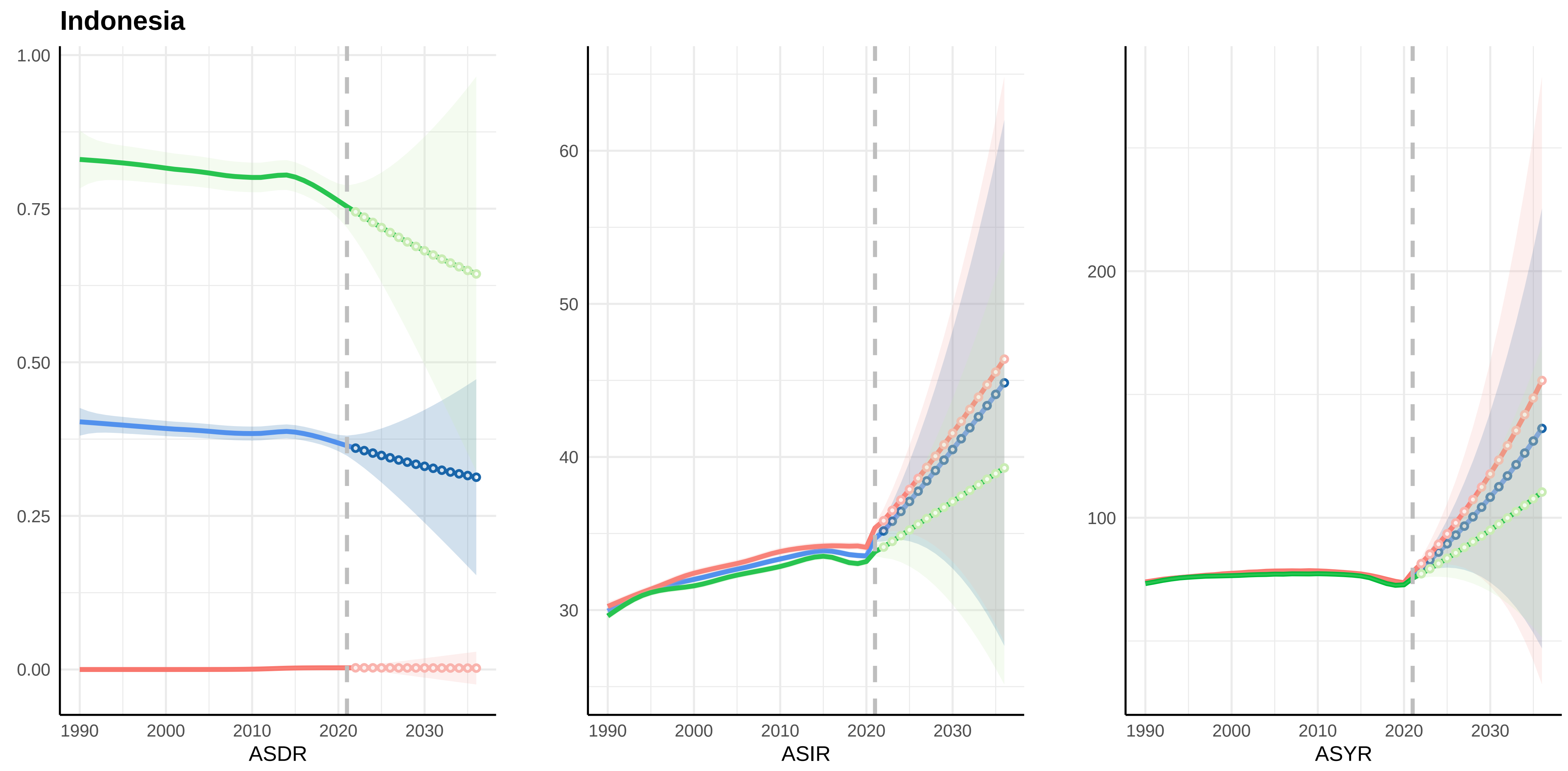

Indonesia
1.00
0.75
0.50
0.25
0.00
1990
2000
2010
2020
2030
ASDR
60
50
40
30
ASIR
200
100
ASYR

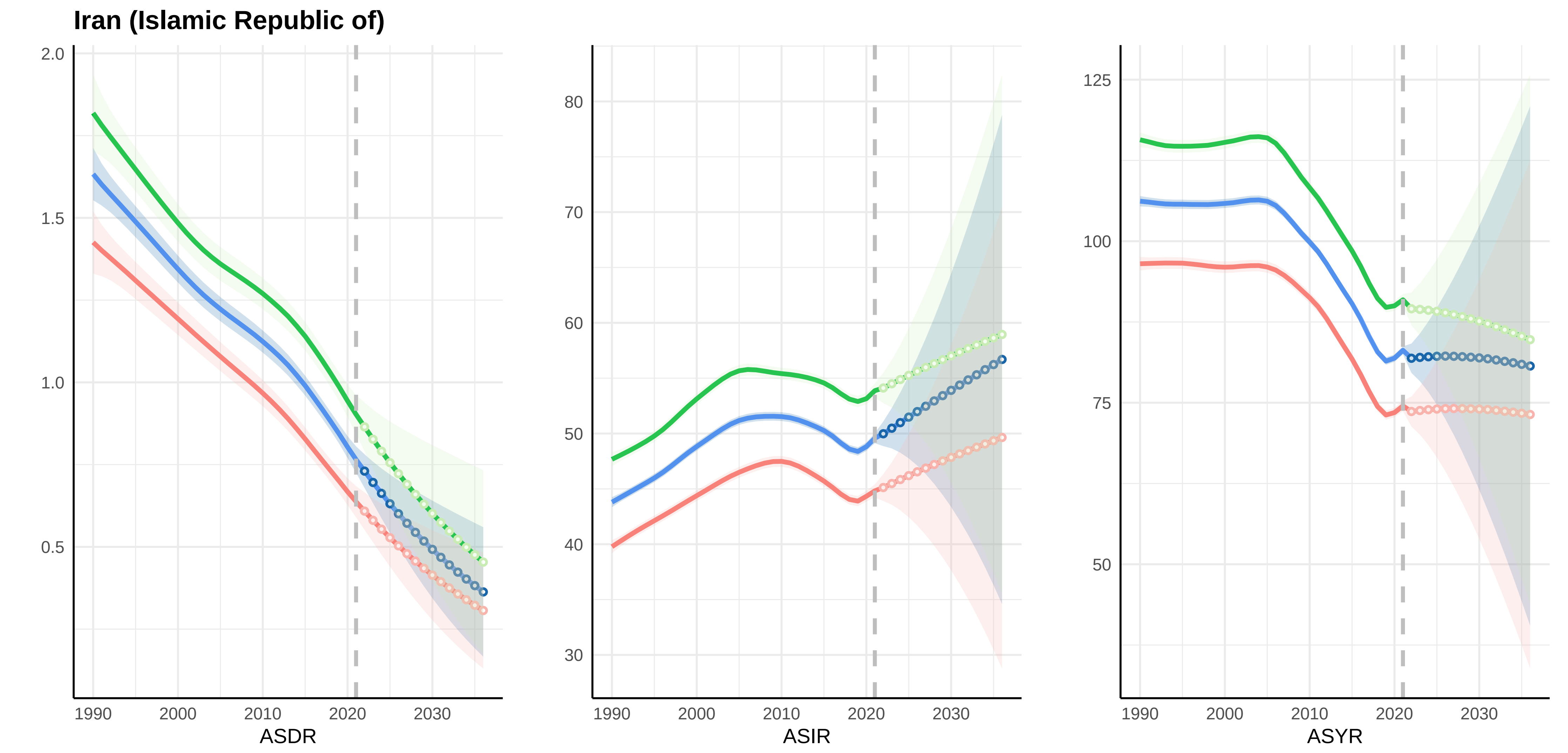

Iran (Islamic Republic of)
2.0
1.5
1.0
0.5
1990
2000
2010
2020
2030
ASDR
80
70
60
50
40
30
1990
2000
2010
2020
2030
ASIR
125
100
75
50
1990
2000
2010
2020
2030
ASYR

Iraq

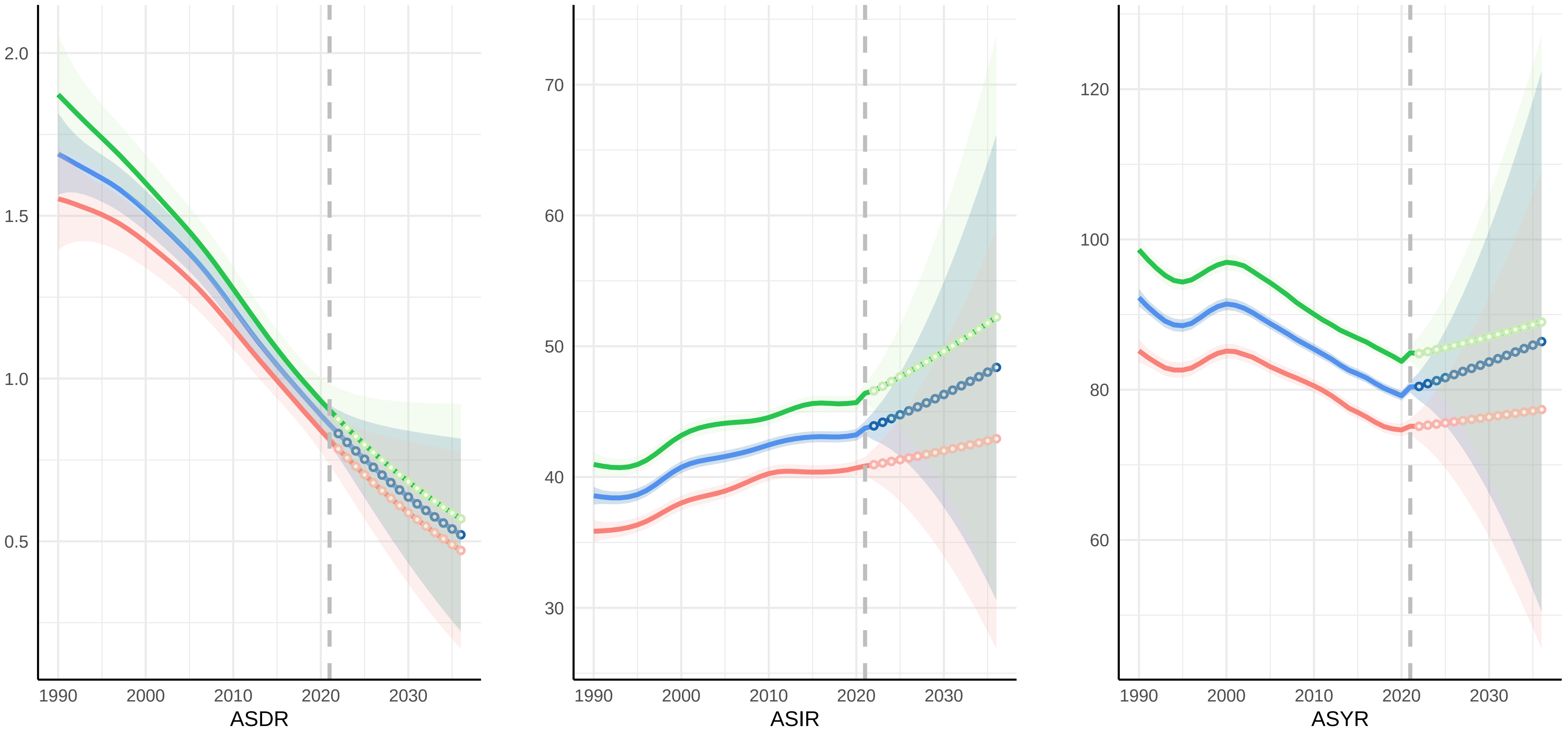

2.0
1.5
1.0
0.5
1990
2000
2010
2020
2030
ASDR
70
60
50
40
30
1990
2000
2010
2020
2030
ASIR
120
100
80
60
1990
2000
2010
2020
2030
ASYR

Ireland

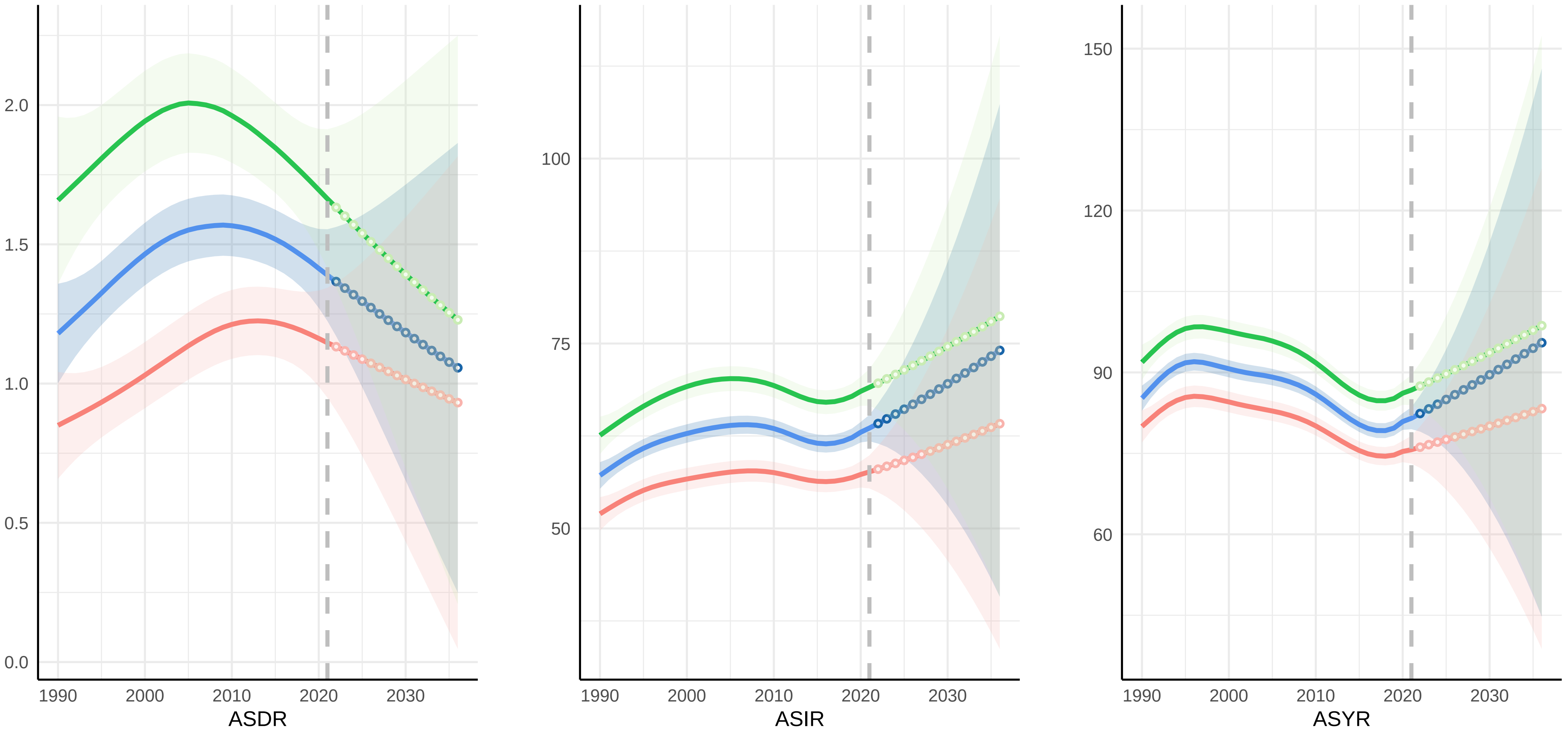

0.0
0.5
1.0
1.5
2.0
1990
2000
2010
2020
2030
ASDR
50
75
100
1990
2000
2010
2020
2030
ASIR
60
90
120
150
1990
2000
2010
2020
2030
ASYR

Israel

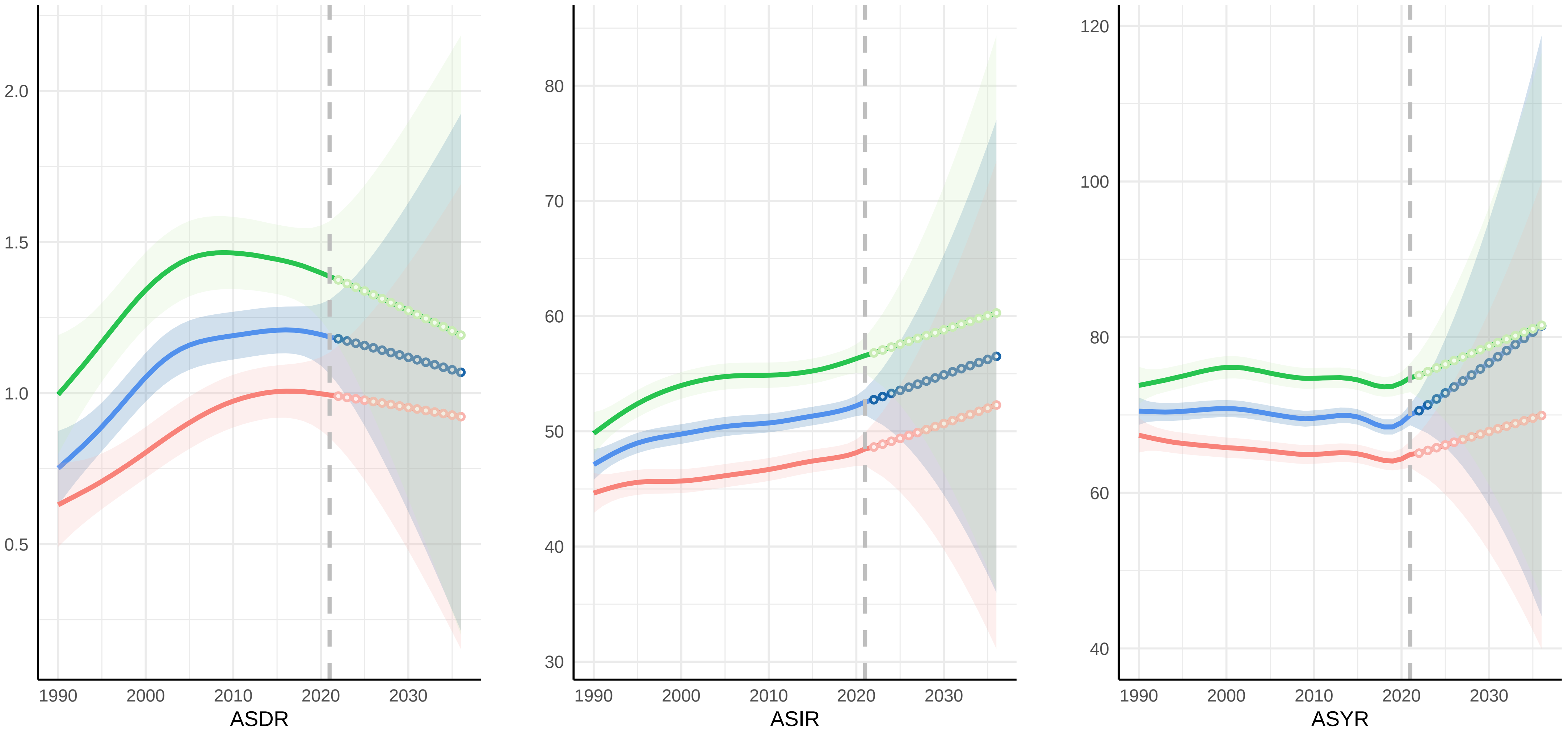

2.0
1.5
1.0
0.5
1990
2000
2010
2020
2030
ASDR
80
70
60
50
40
30
1990
2000
2010
2020
2030
ASIR
120
100
80
60
40
1990
2000
2010
2020
2030
ASYR

Italy

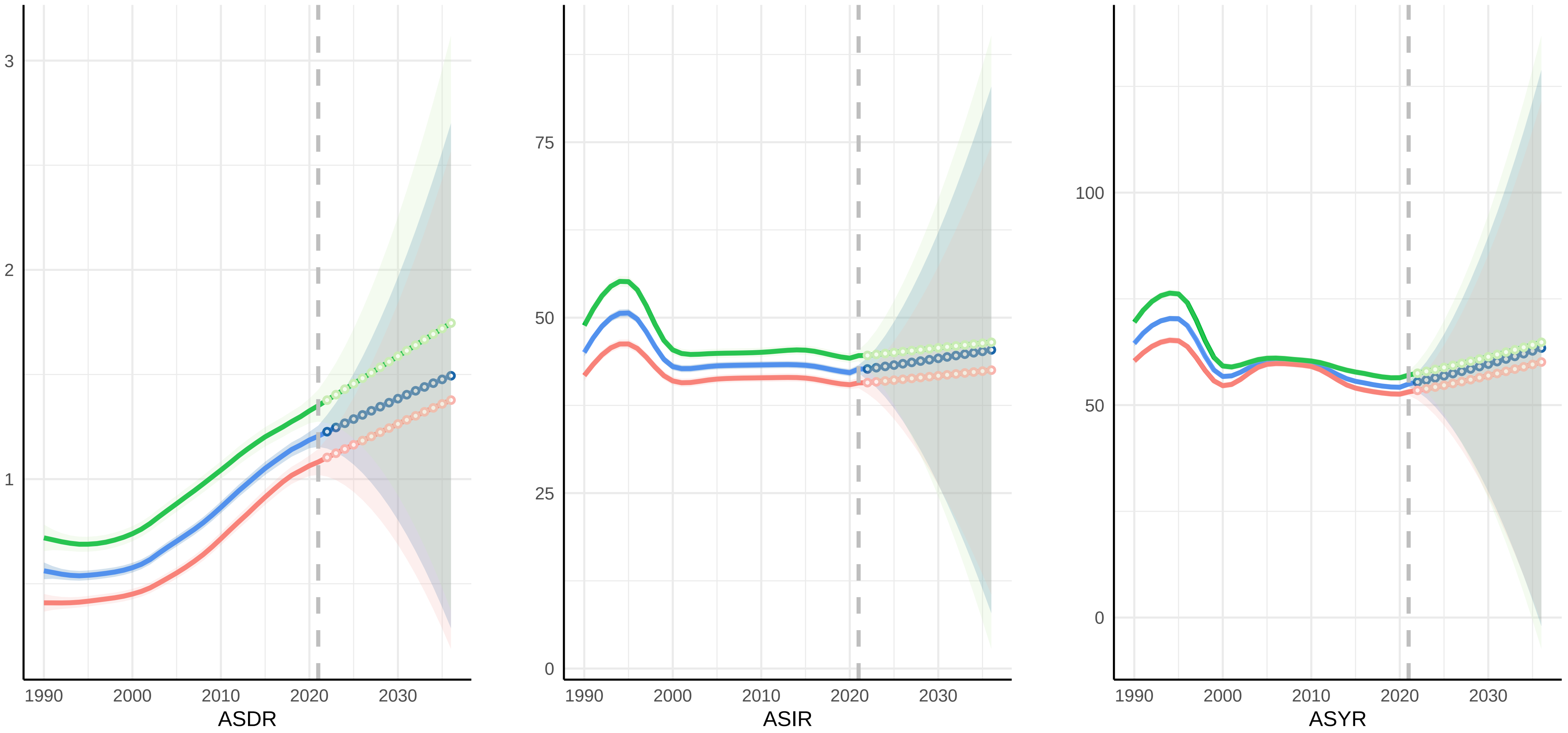

3
2
1
1990
2000
2010
2020
2030
ASDR
75
50
25
0
1990
2000
2010
2020
2030
ASIR
100
50
0
1990
2000
2010
2020
2030
ASYR

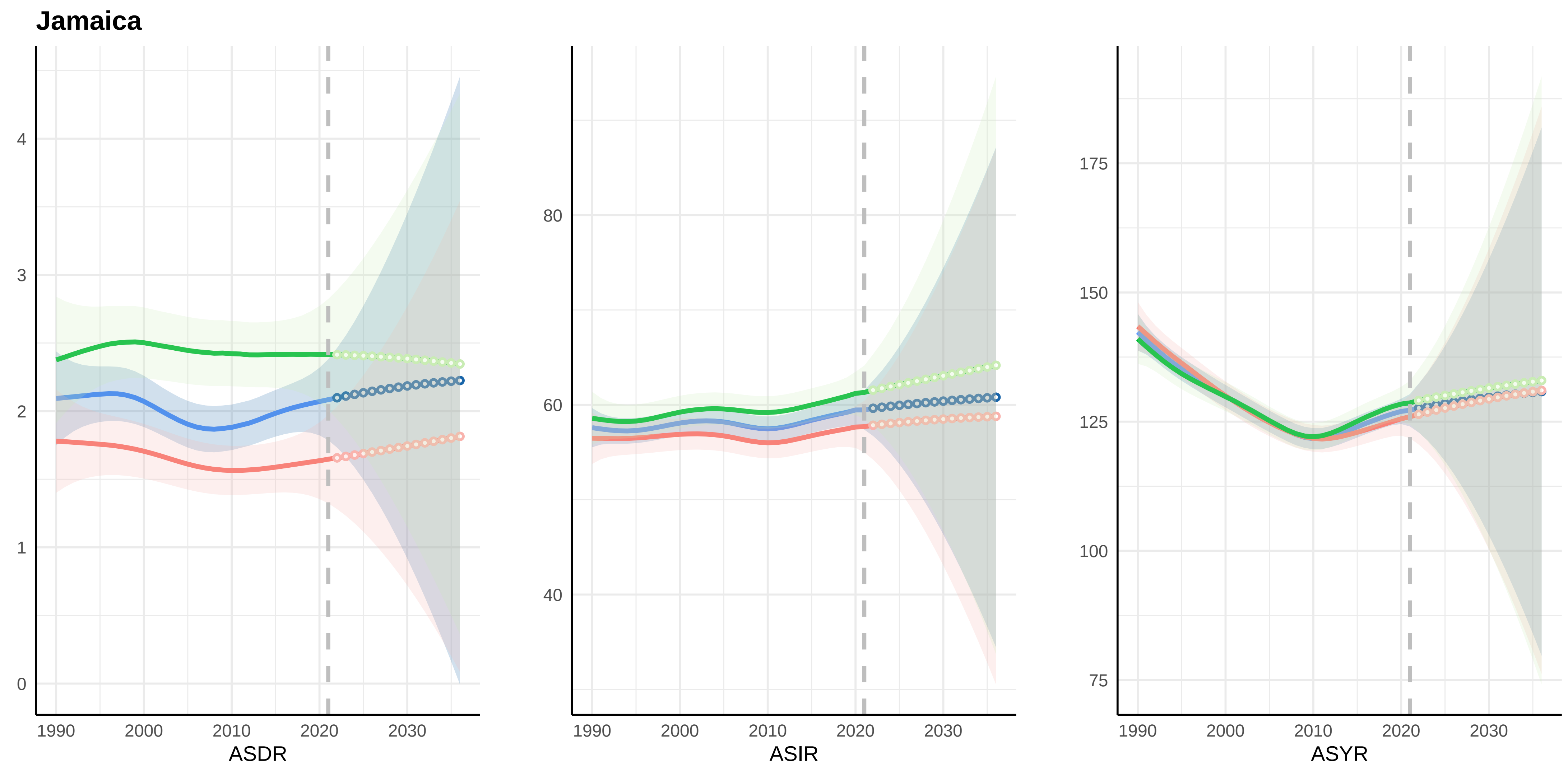

Jamaica
4
3
2
1
0
1990
2000
2010
2020
2030
ASDR
80
60
40
1990
2000
2010
2020
2030
ASIR
175
150
125
100
75
1990
2000
2010
2020
2030
ASYR

Japan

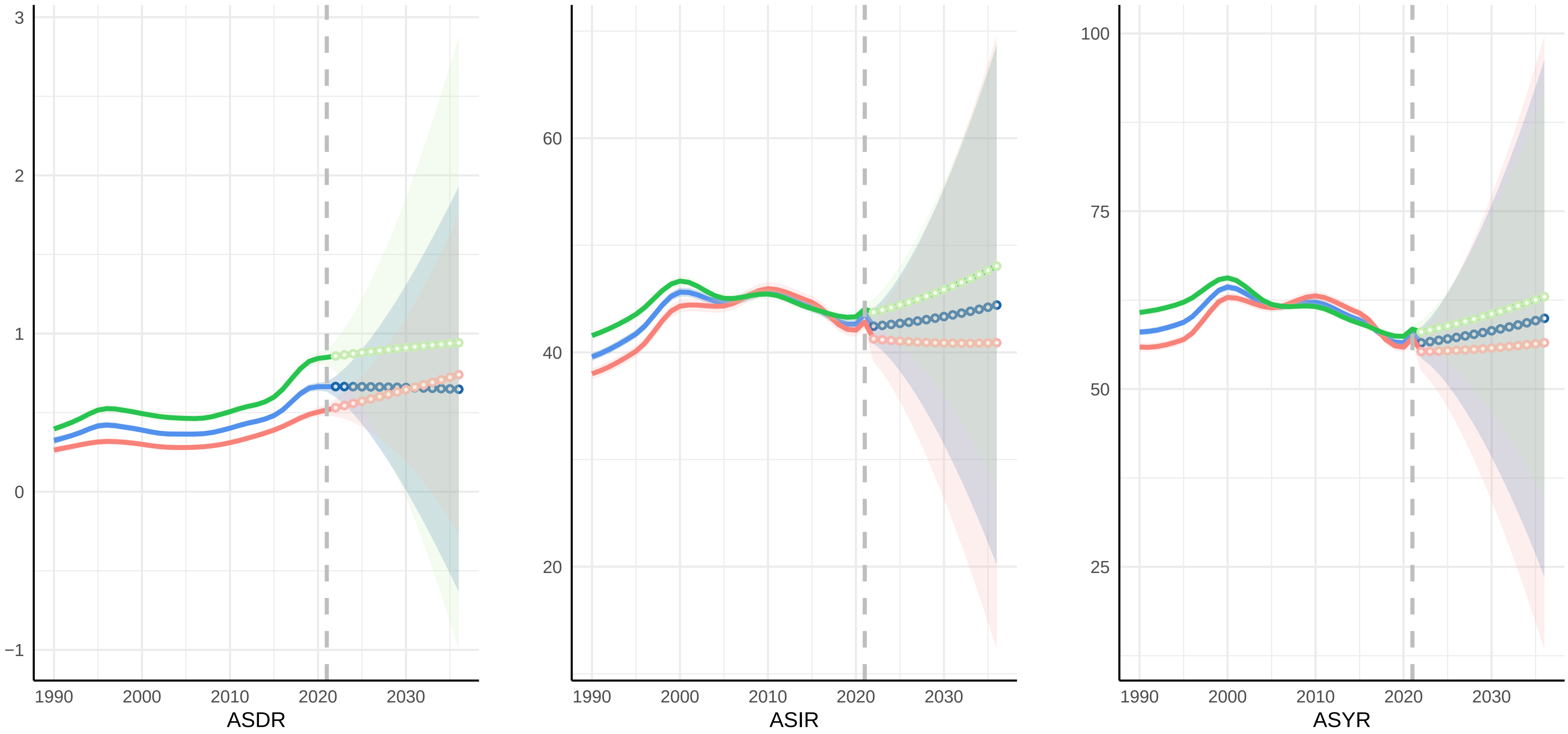

3
2
1
0
−1
1990
2000
2010
2020
2030
ASDR
60
40
20
ASIR
100
75
50
25
ASYR

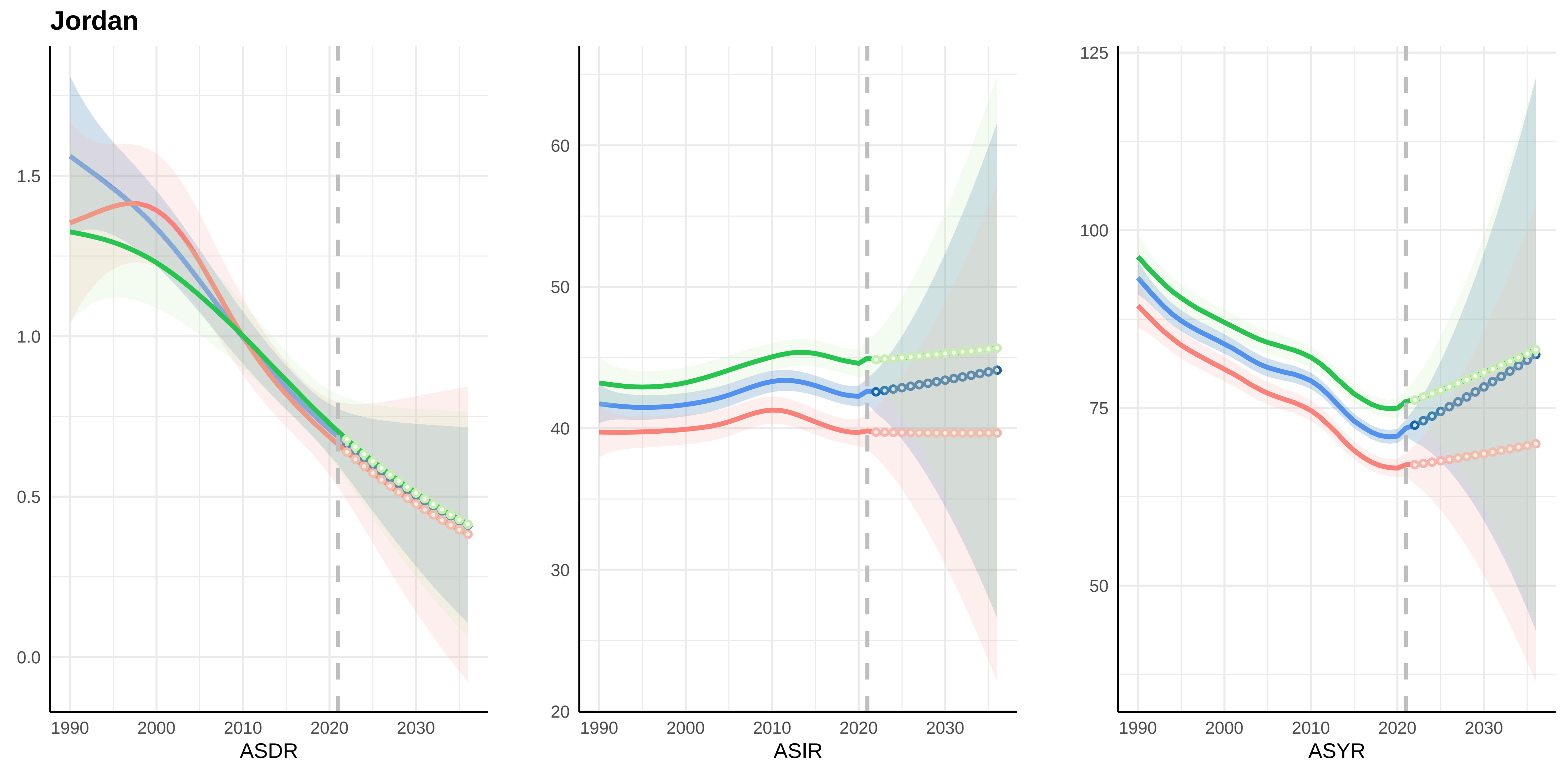

Jordan
1.5
1.0
0.5
0.0
1990
2000
2010
2020
2030
ASDR
60
50
40
30
20
1990
2000
2010
2020
2030
ASIR
125
100
75
50
1990
2000
2010
2020
2030
ASYR

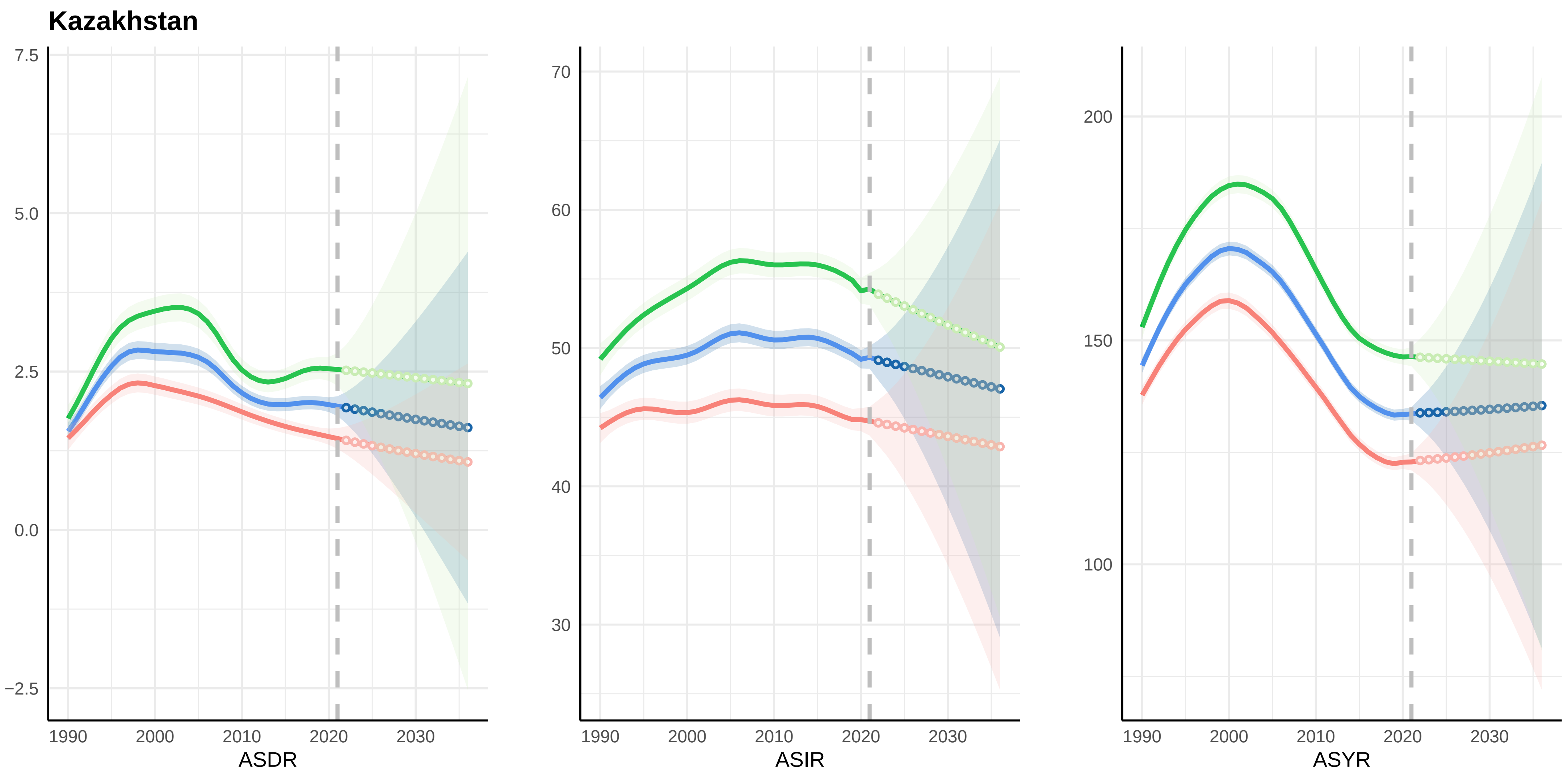

Kazakhstan
7.5
5.0
2.5
0.0
−2.5
1990
2000
2010
2020
2030
ASDR
70
60
50
40
30
1990
2000
2010
2020
2030
ASIR
200
150
100
1990
2000
2010
2020
2030
ASYR

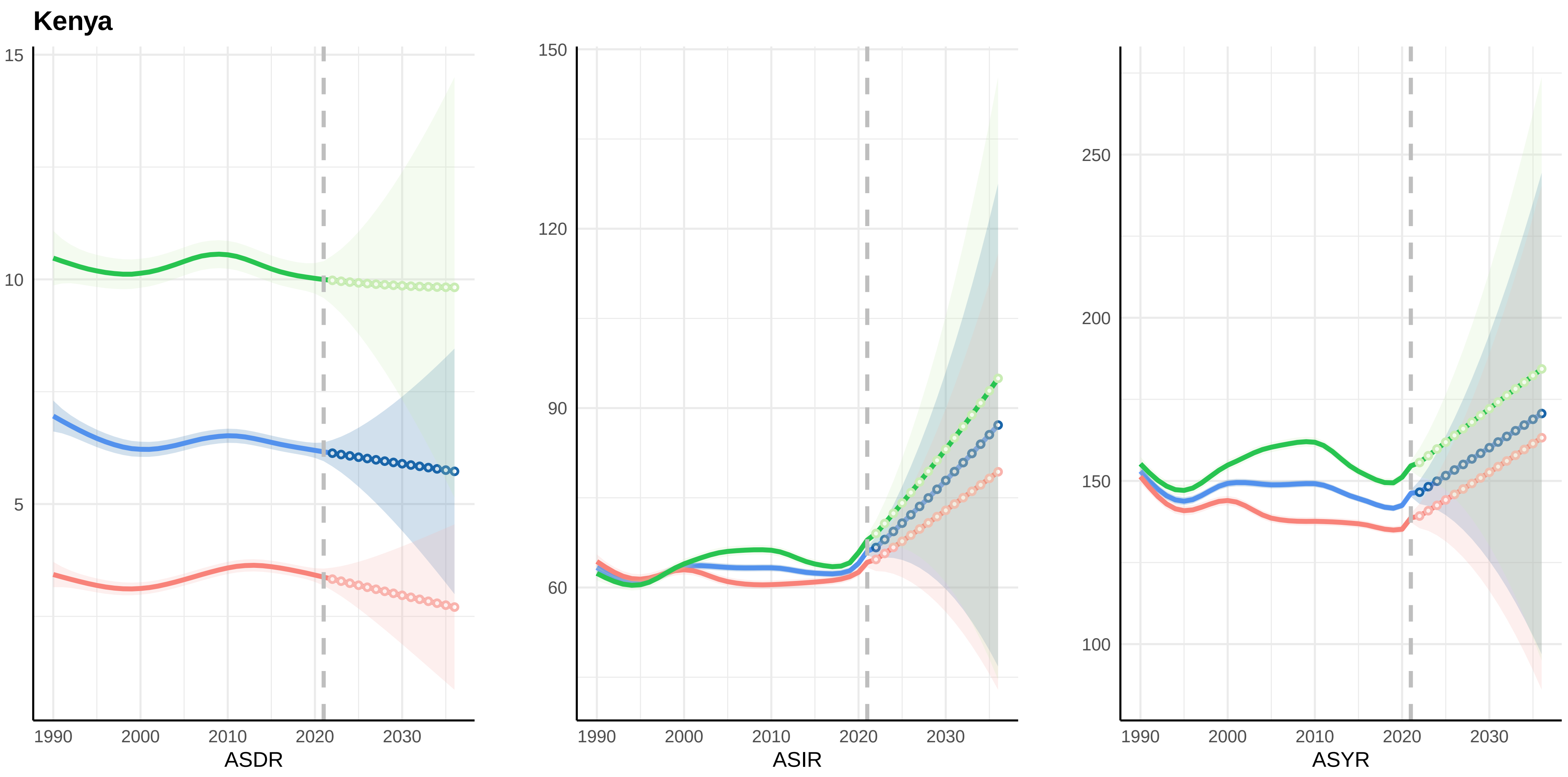
Kenya
15
10
5
1990
2000
2010
2020
2030
ASDR
150
120
90
60
1990
2000
2010
2020
2030
ASIR
250
200
150
100
1990
2000
2010
2020
2030
ASYR

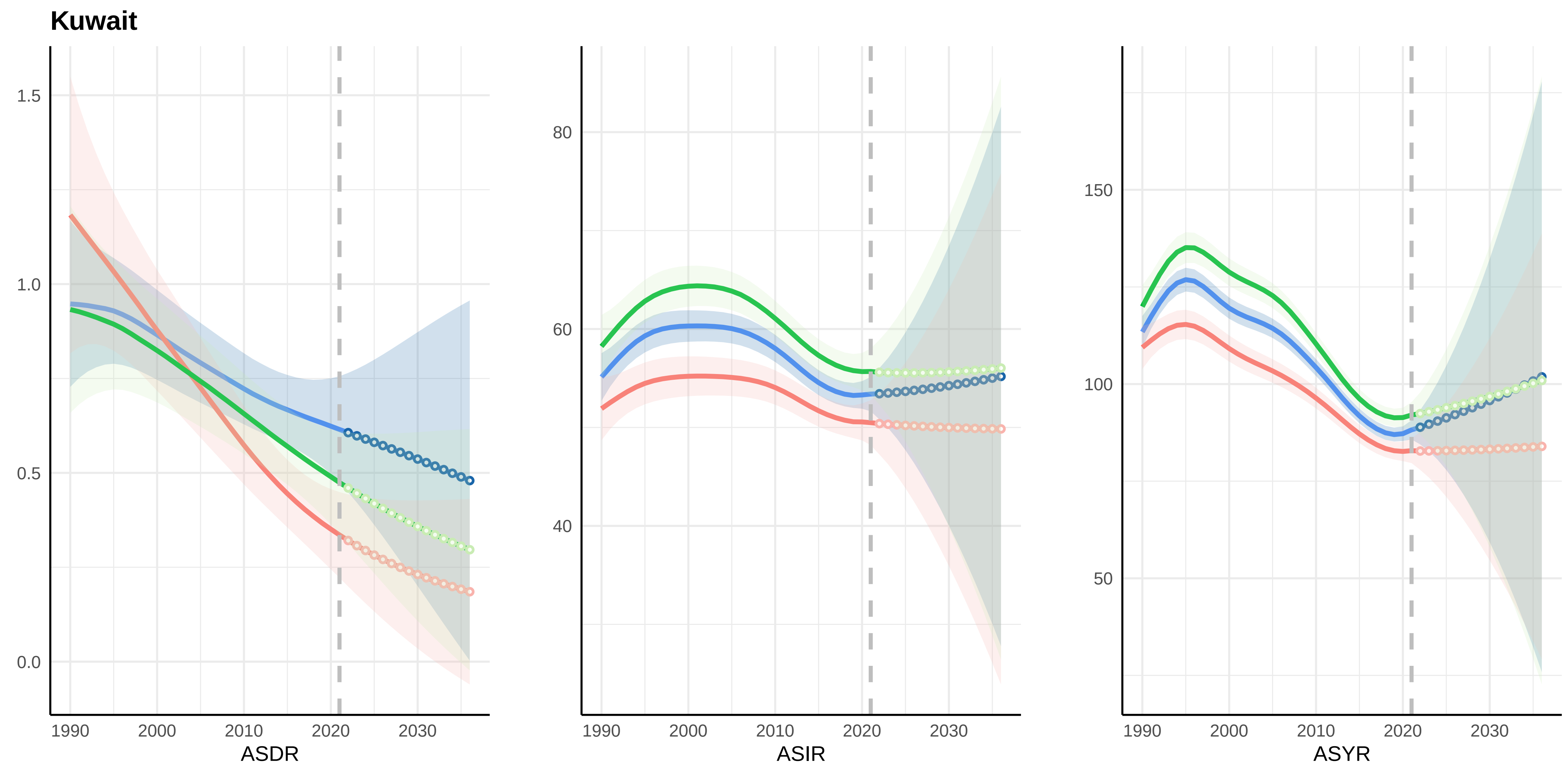
Kuwait
1.5
1.0
0.5
0.0
1990
2000
2010
2020
2030
ASDR
80
60
40
1990
2000
2010
2020
2030
ASIR
150
100
50
1990
2000
2010
2020
2030
ASYR

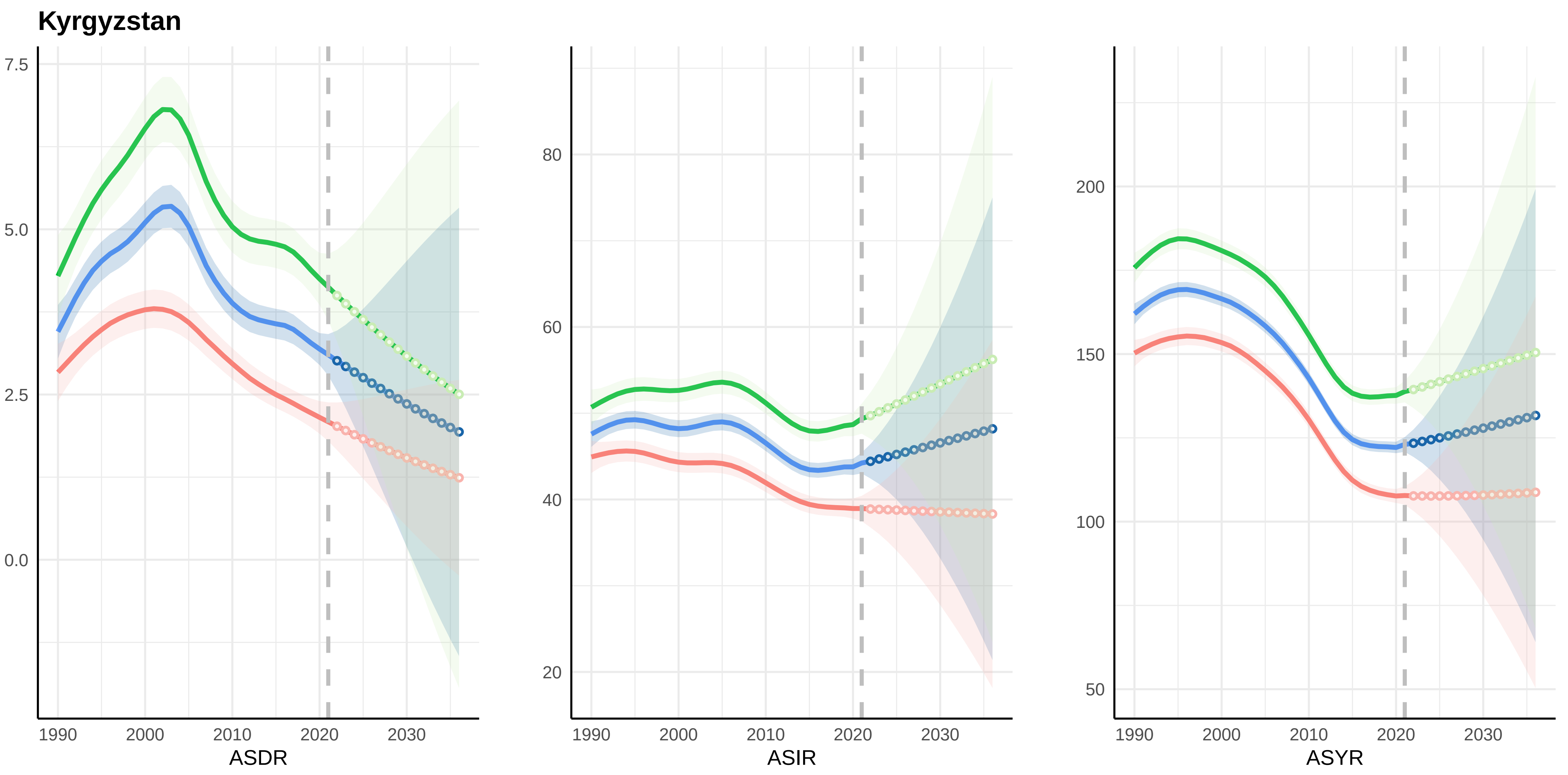
Kyrgyzstan
7.5
5.0
2.5
0.0
1990
2000
2010
2020
2030
ASDR
80
60
40
20
1990
2000
2010
2020
2030
ASIR
200
150
100
50
1990
2000
2010
2020
2030
ASYR

Lao People's Democratic Republic

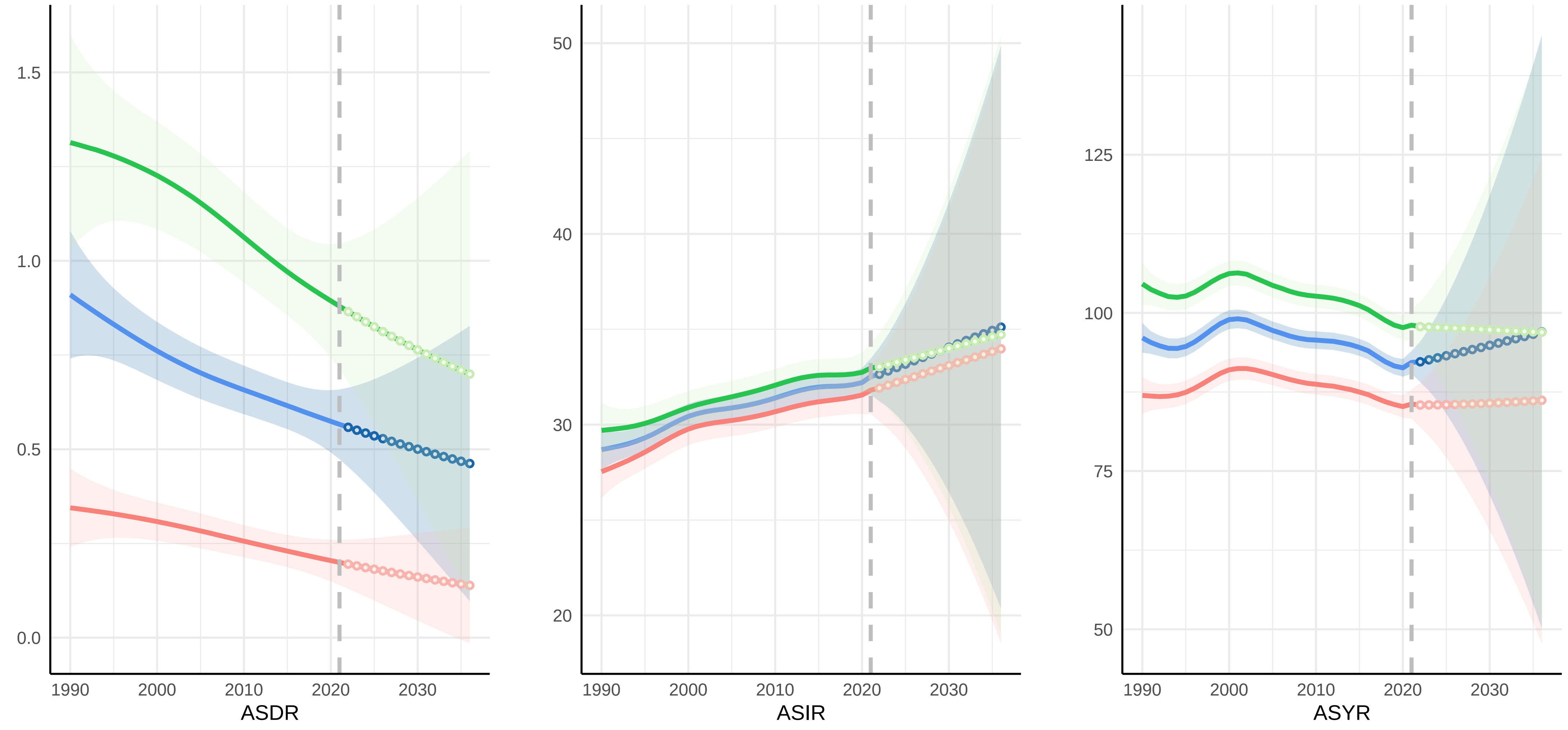

Latvia

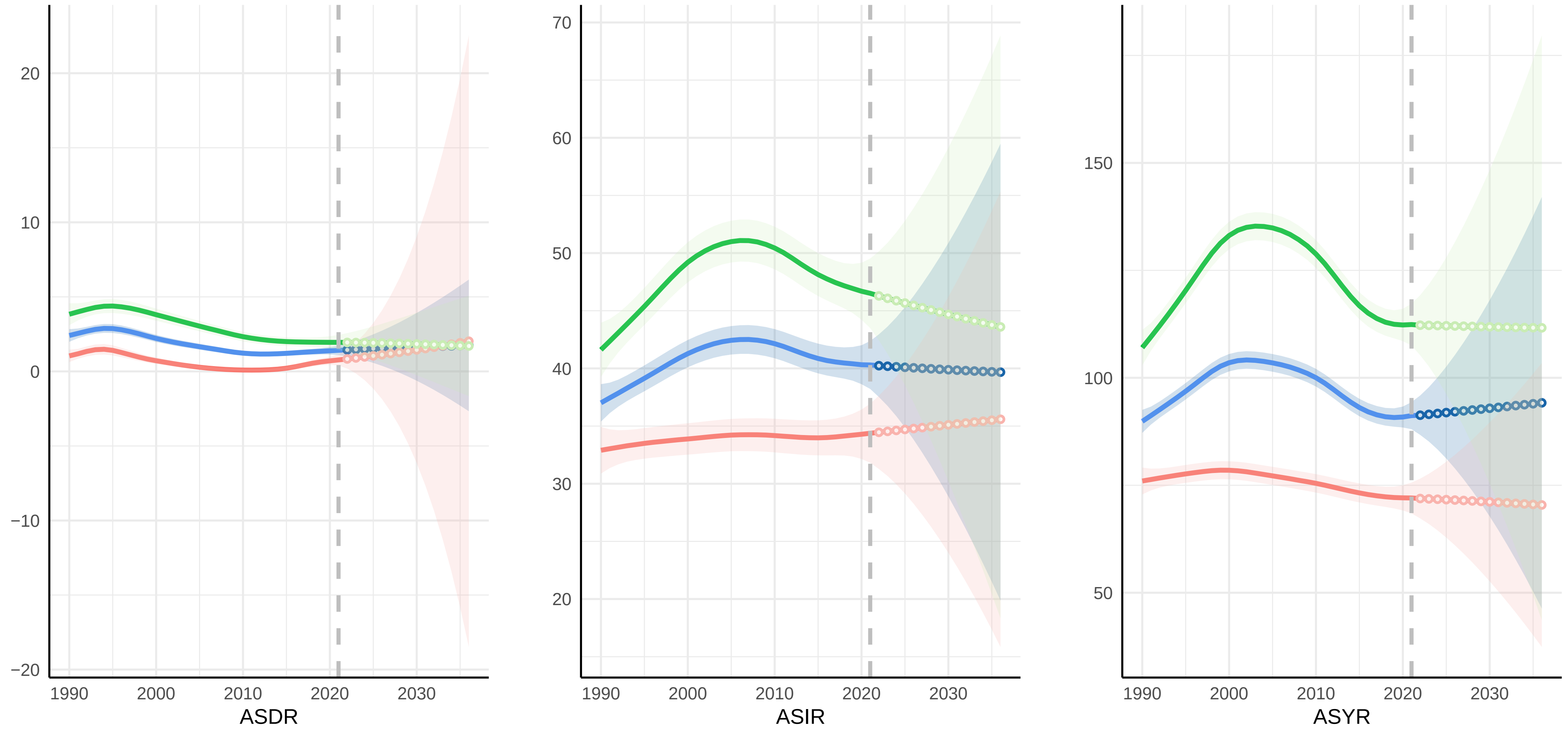

20
10
0
−10
−20
1990
2000
2010
2020
2030
ASDR
70
60
50
40
30
20
ASIR
150
100
50
ASYR

Lebanon

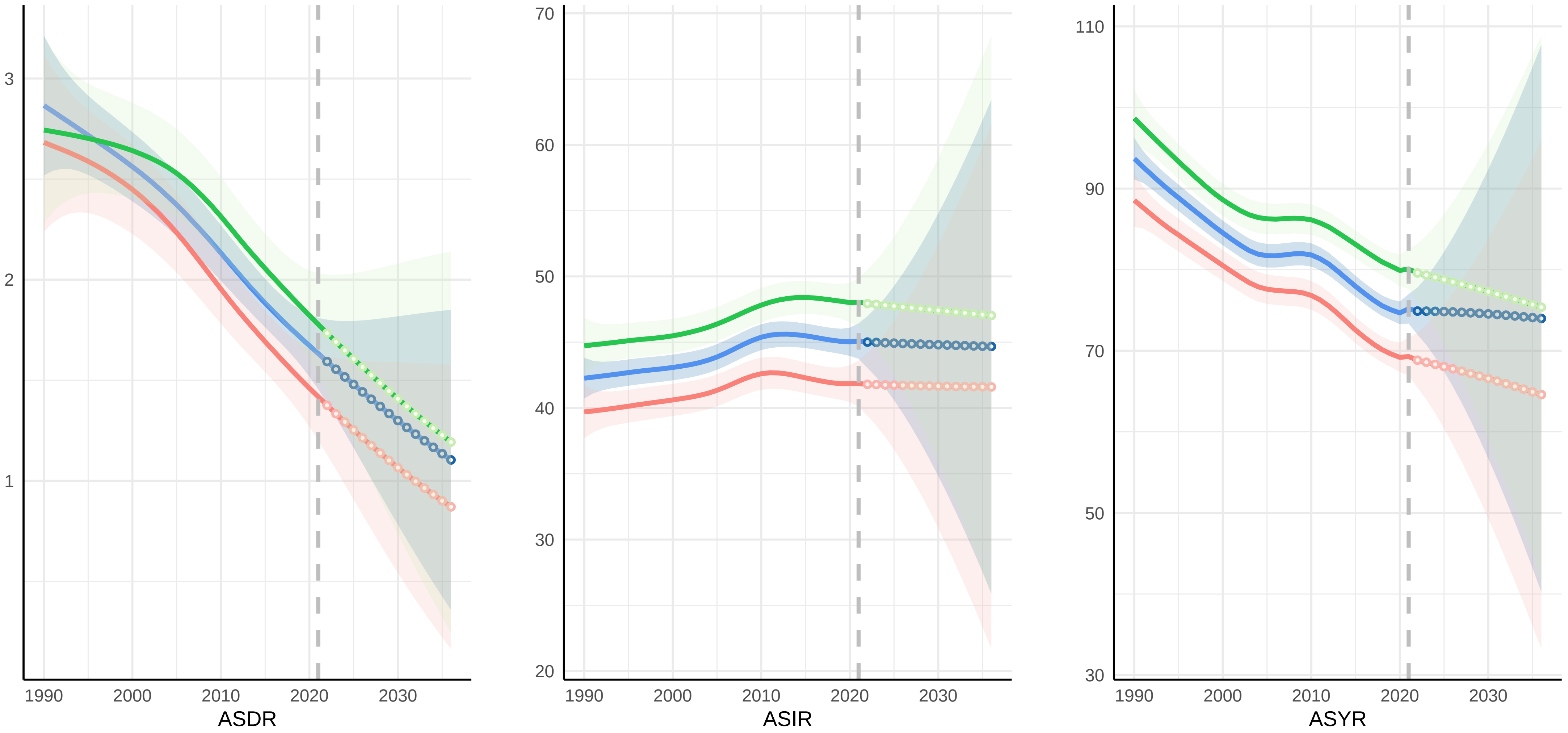

3
2
1
1990
2000
2010
2020
2030
ASDR
70
60
50
40
30
20
1990
2000
2010
2020
2030
ASIR
110
90
70
50
30
1990
2000
2010
2020
2030
ASYR

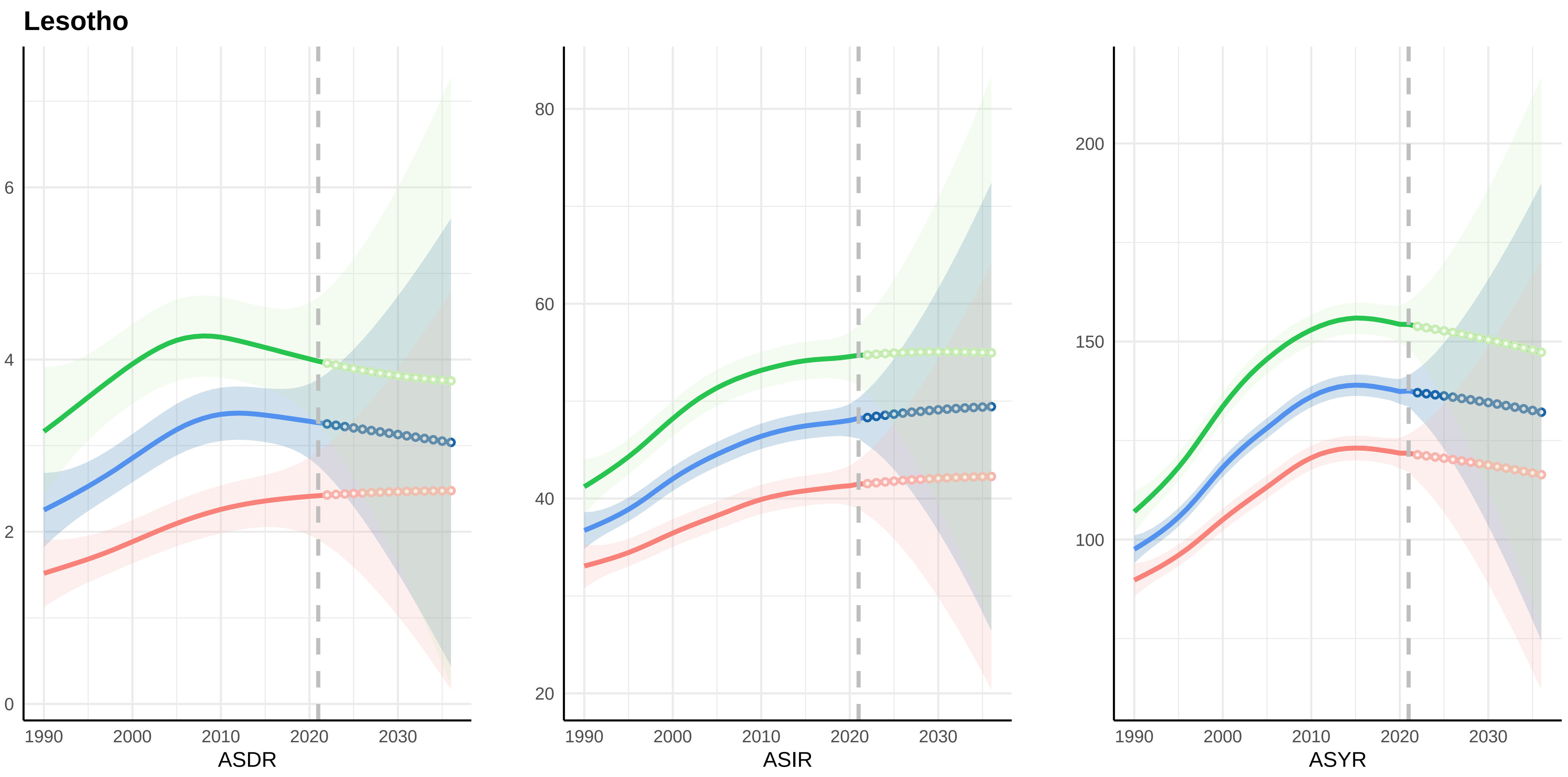
Lesotho
0
2
4
6
1990
2000
2010
2020
2030
ASDR
20
40
60
80
1990
2000
2010
2020
2030
ASIR
100
150
200
1990
2000
2010
2020
2030
ASYR

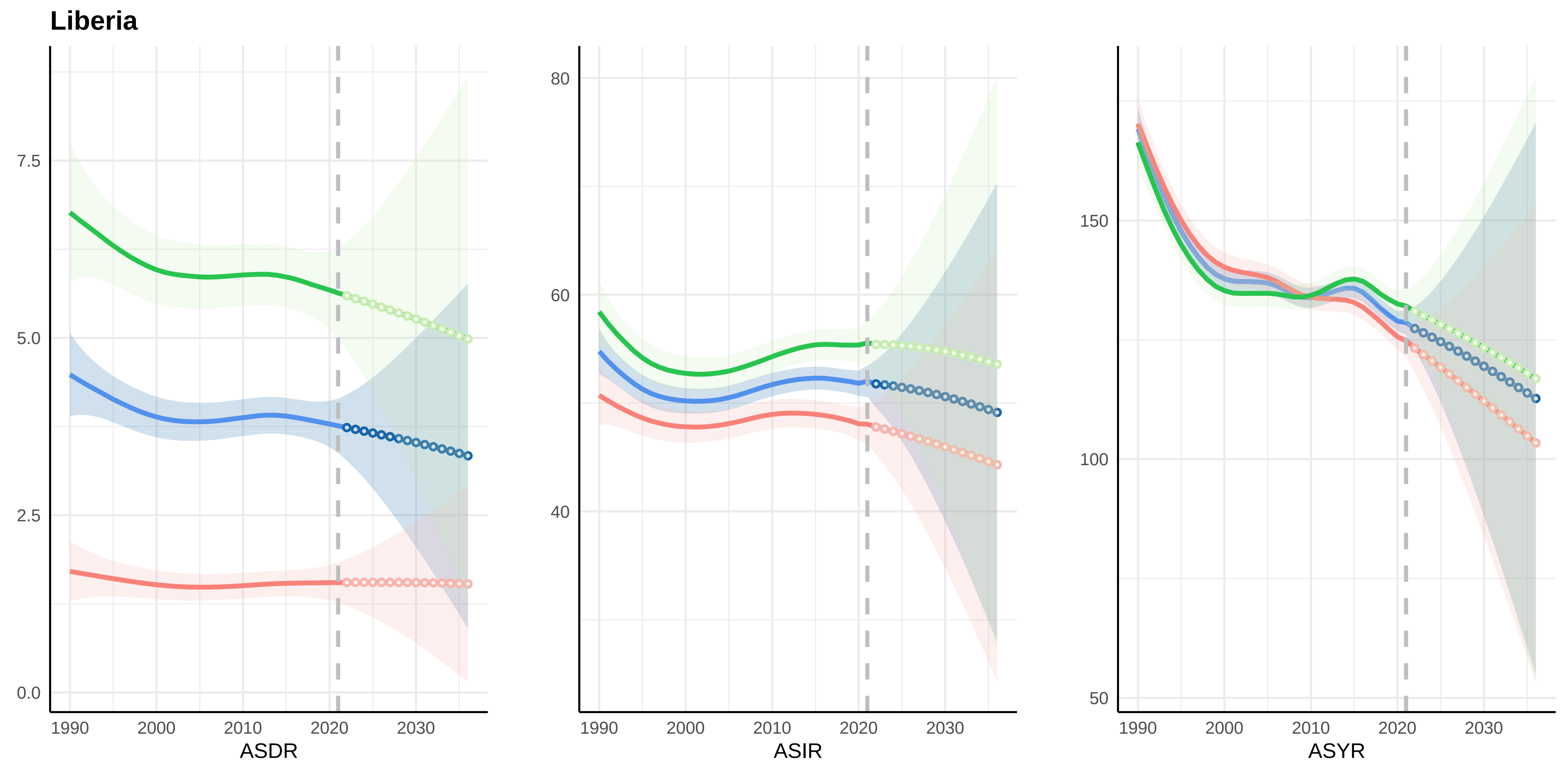

Liberia
7.5
5.0
2.5
0.0
1990
2000
2010
2020
2030
ASDR
80
60
40
1990
2000
2010
2020
2030
ASIR
150
100
50
1990
2000
2010
2020
2030
ASYR

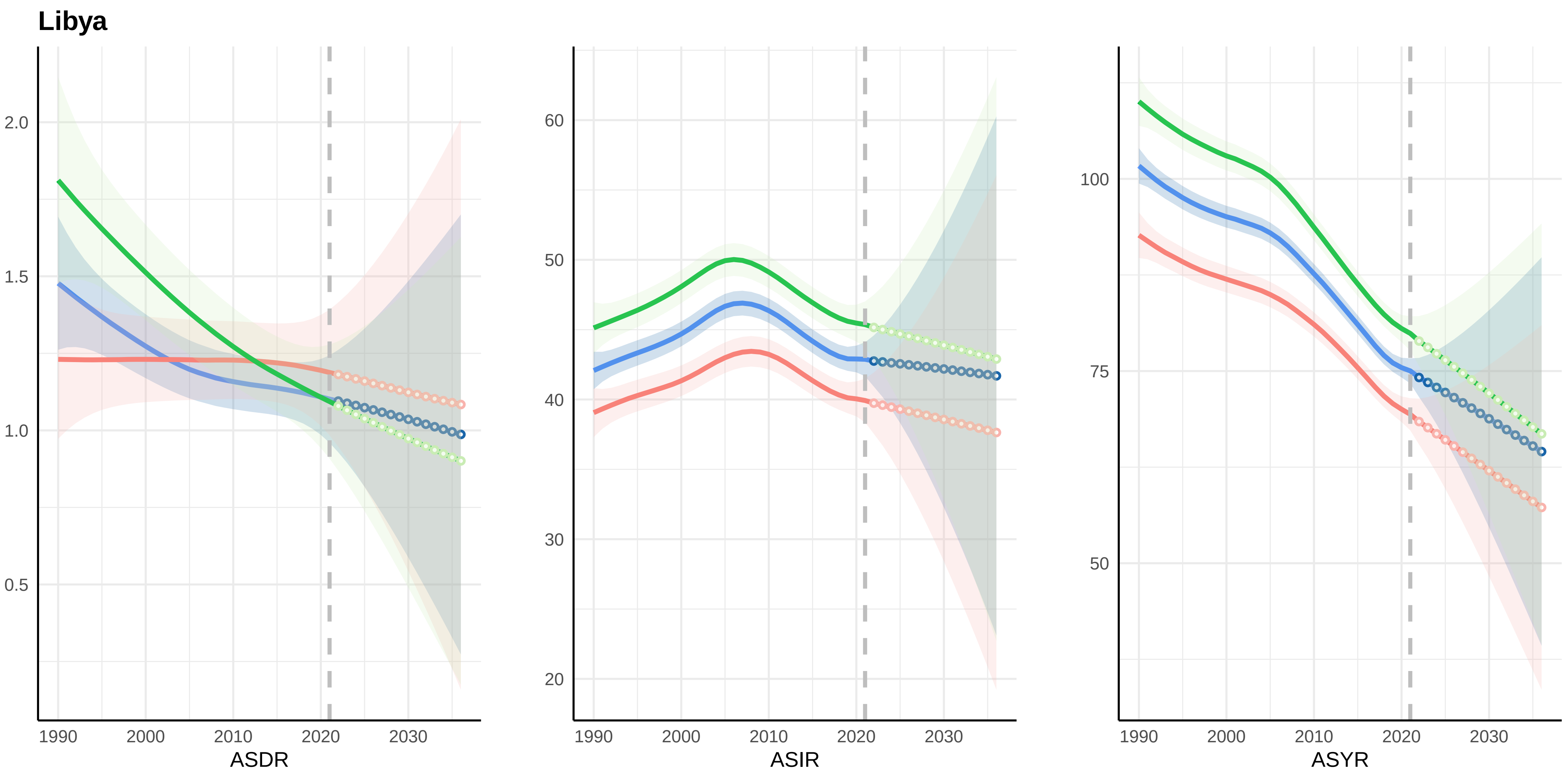

Libya
2.0
1.5
1.0
0.5
1990
2000
2010
2020
2030
ASDR
60
50
40
30
20
1990
2000
2010
2020
2030
ASIR
100
75
50
1990
2000
2010
2020
2030
ASYR

Lithuania

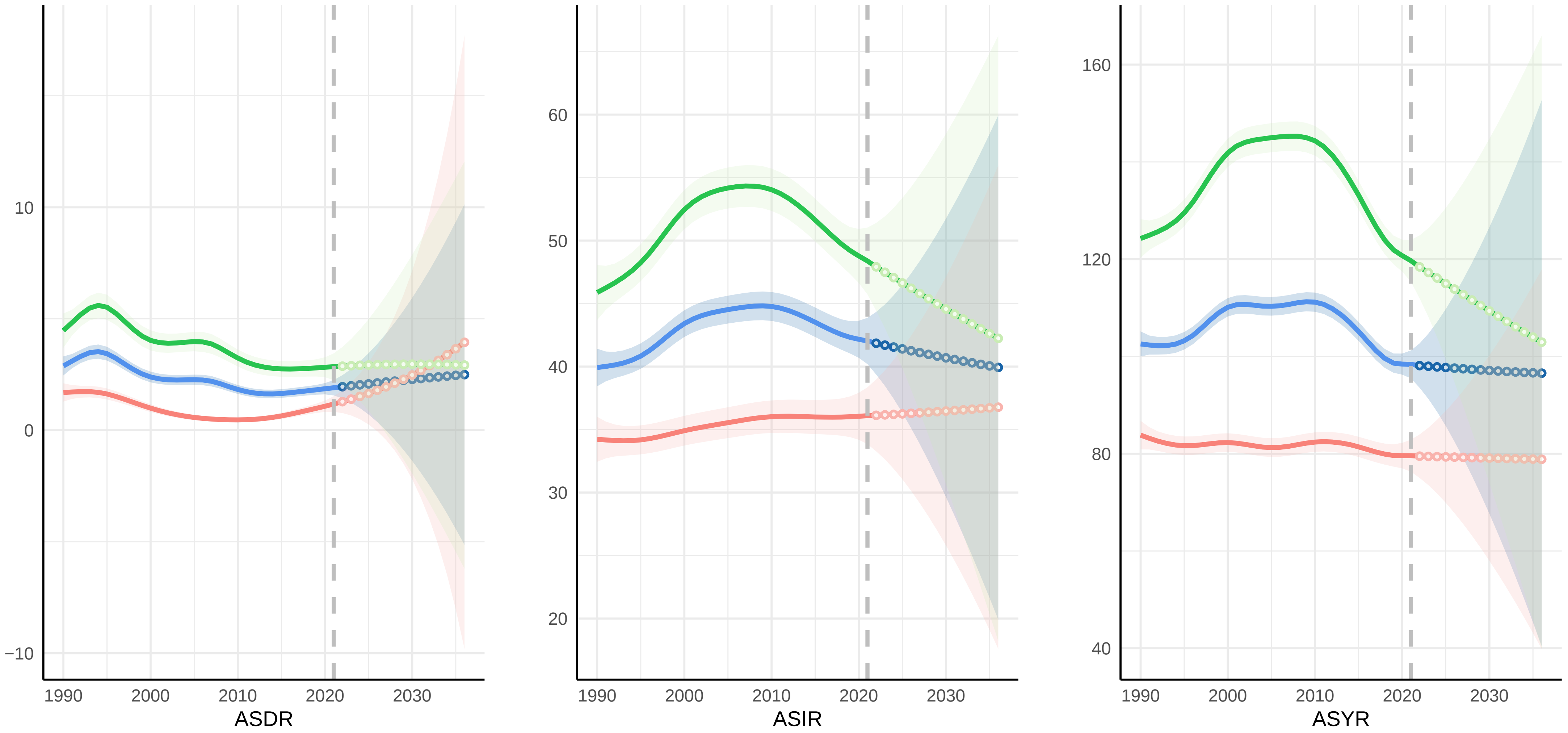

10
0
−10
1990
2000
2010
2020
2030
ASDR
60
50
40
30
20
1990
2000
2010
2020
2030
ASIR
160
120
80
40
1990
2000
2010
2020
2030
ASYR

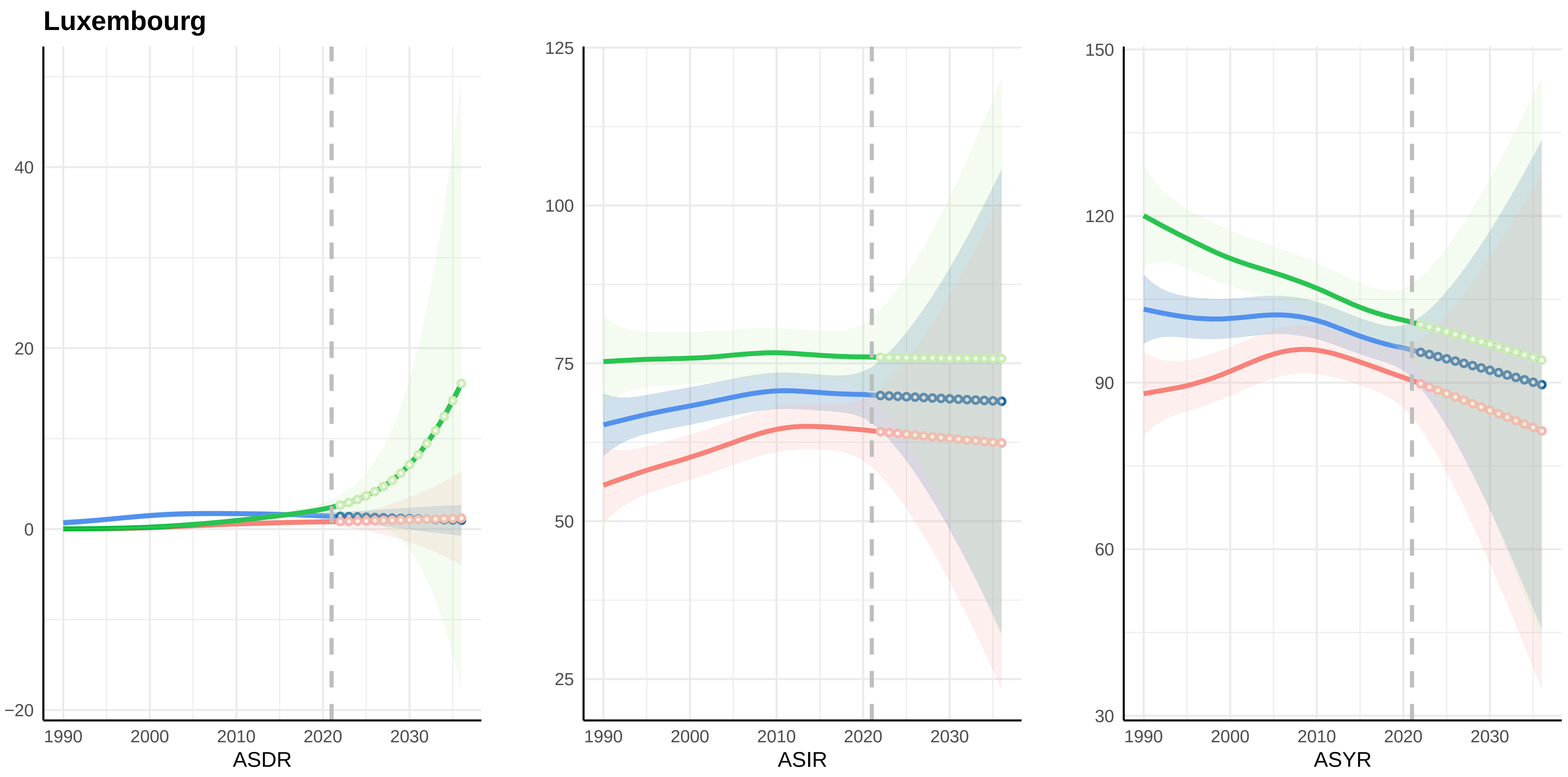
Luxembourg
40
20
0
−20
1990
2000
2010
2020
2030
ASDR
125
100
75
50
25
ASIR
150
120
90
60
30
ASYR

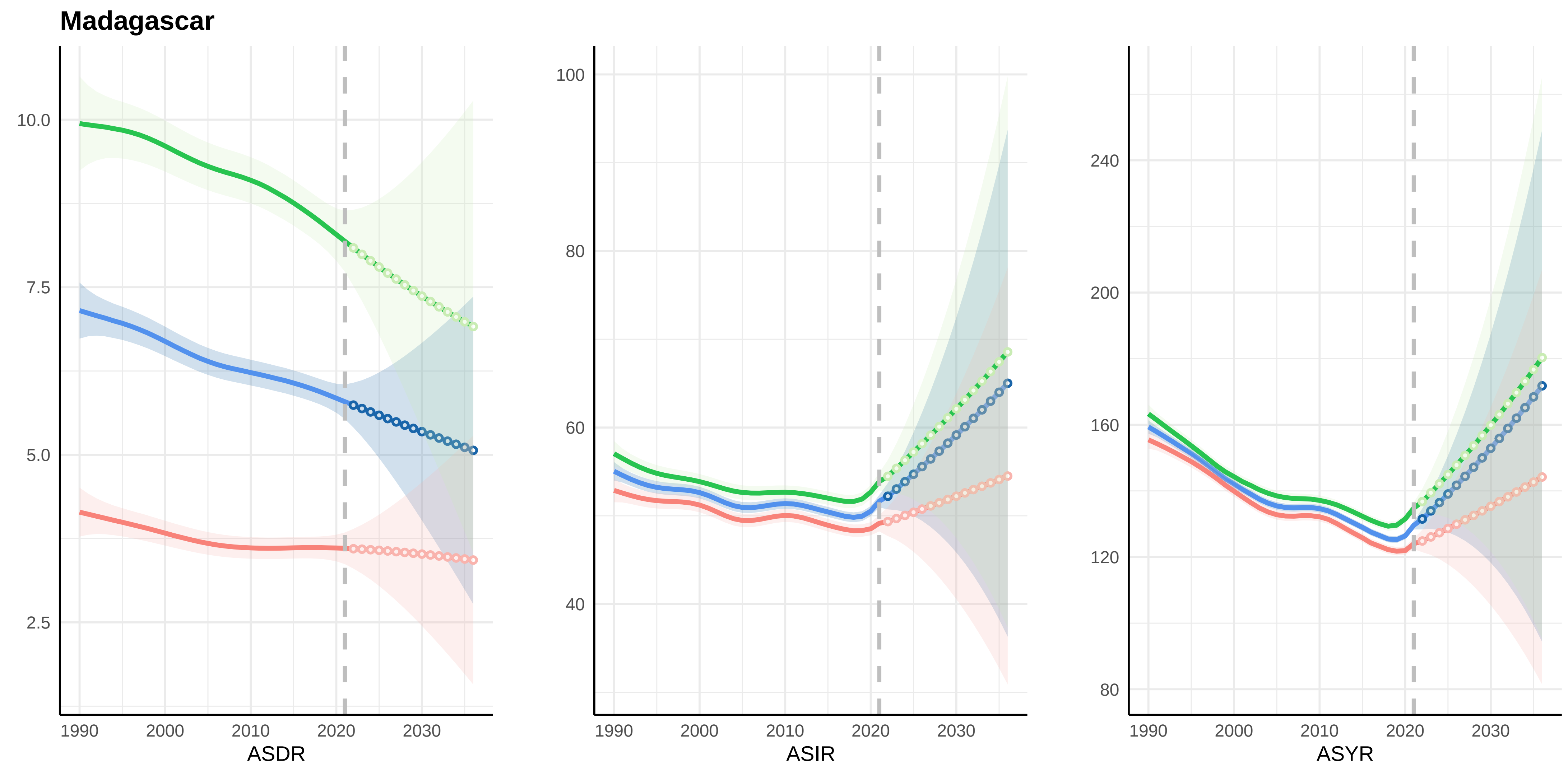

Madagascar
10.0
7.5
5.0
2.5
1990
2000
2010
2020
2030
ASDR
100
80
60
40
ASIR
240
200
160
120
80
ASYR

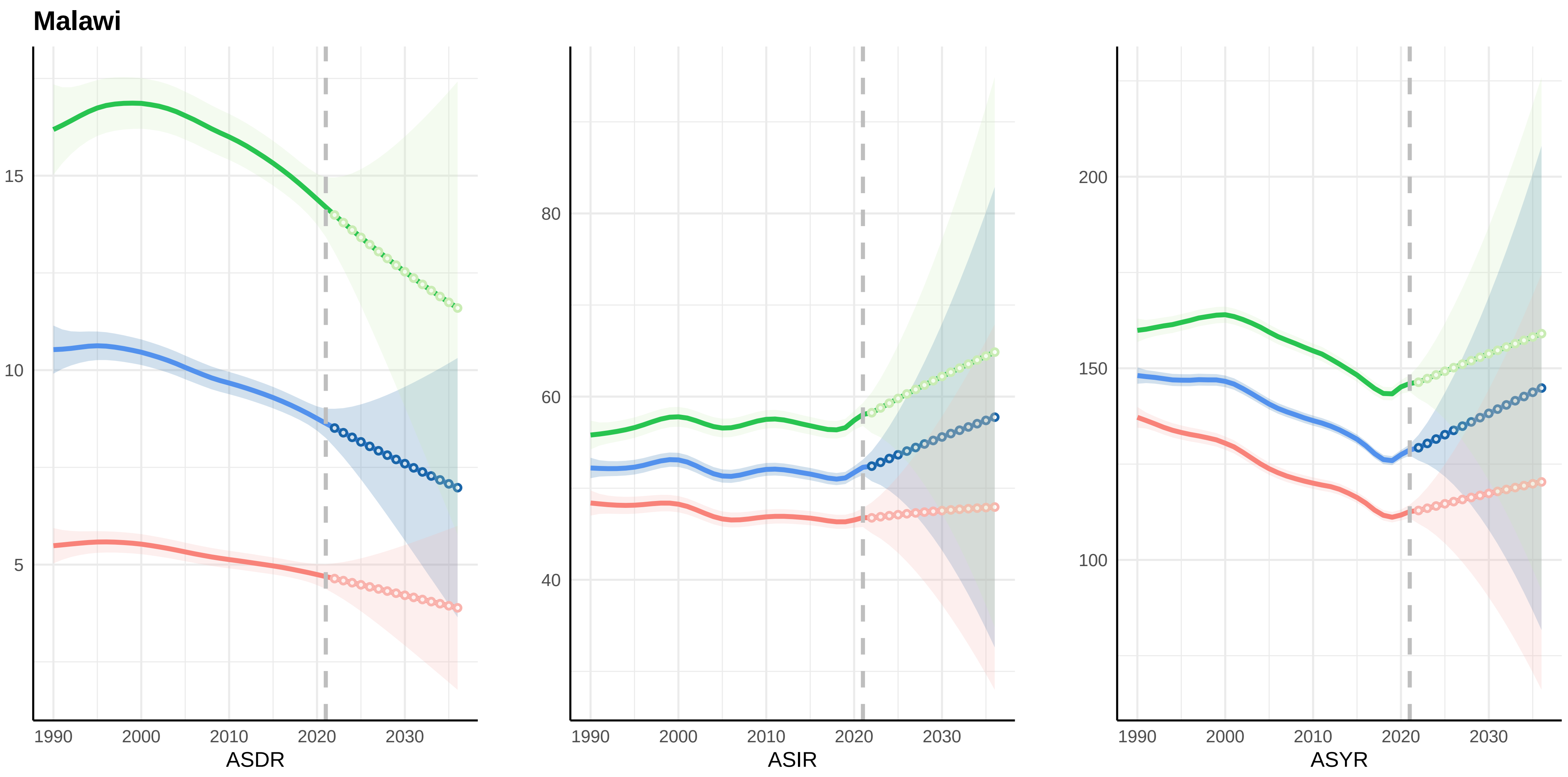

Malawi
15
10
5
1990
2000
2010
2020
2030
ASDR
80
60
40
1990
2000
2010
2020
2030
ASIR
200
150
100
1990
2000
2010
2020
2030
ASYR

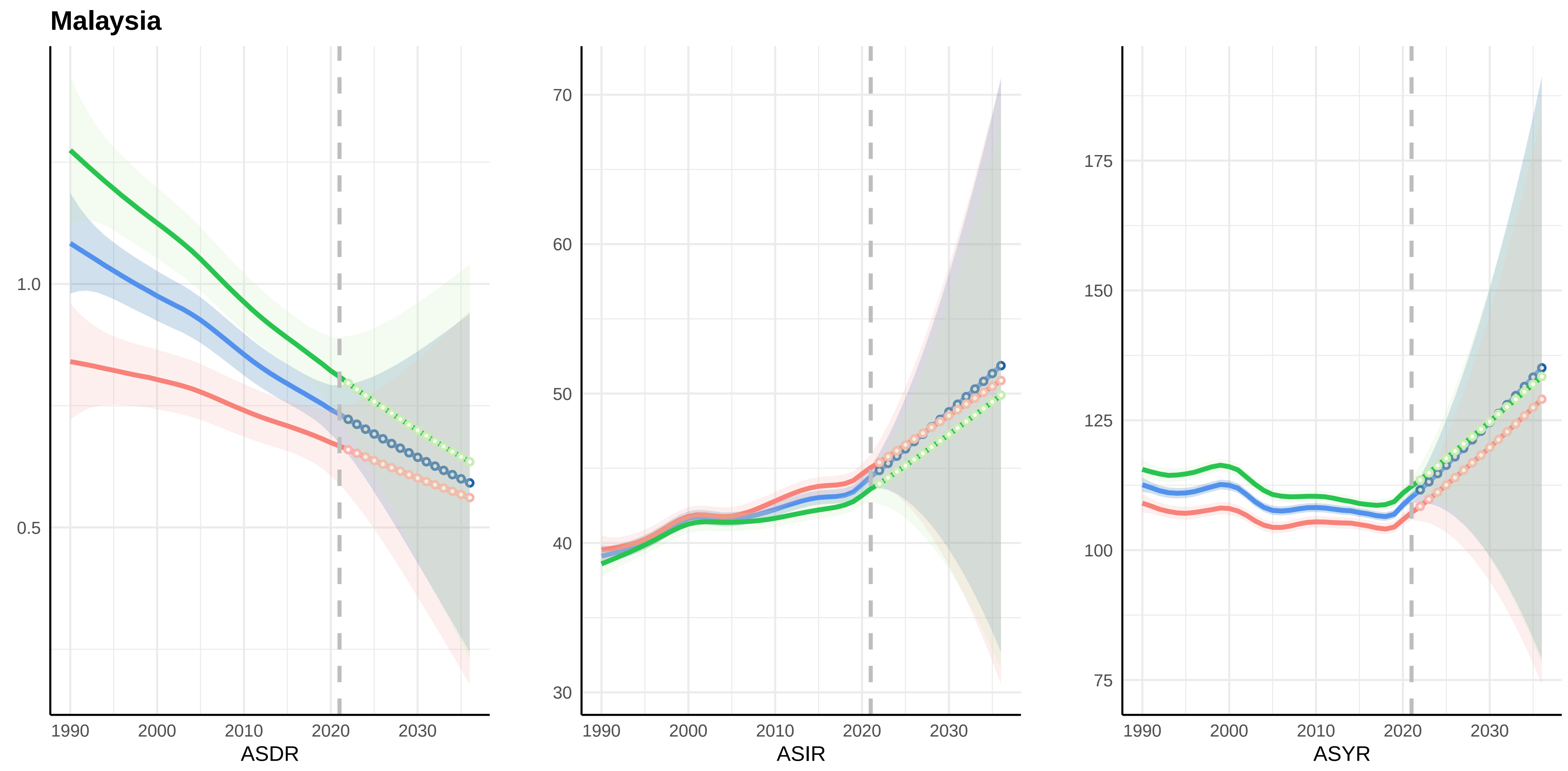

Malaysia
1.0
0.5
1990
2000
2010
2020
2030
ASDR
70
60
50
40
30
1990
2000
2010
2020
2030
ASIR
175
150
125
100
75
1990
2000
2010
2020
2030
ASYR

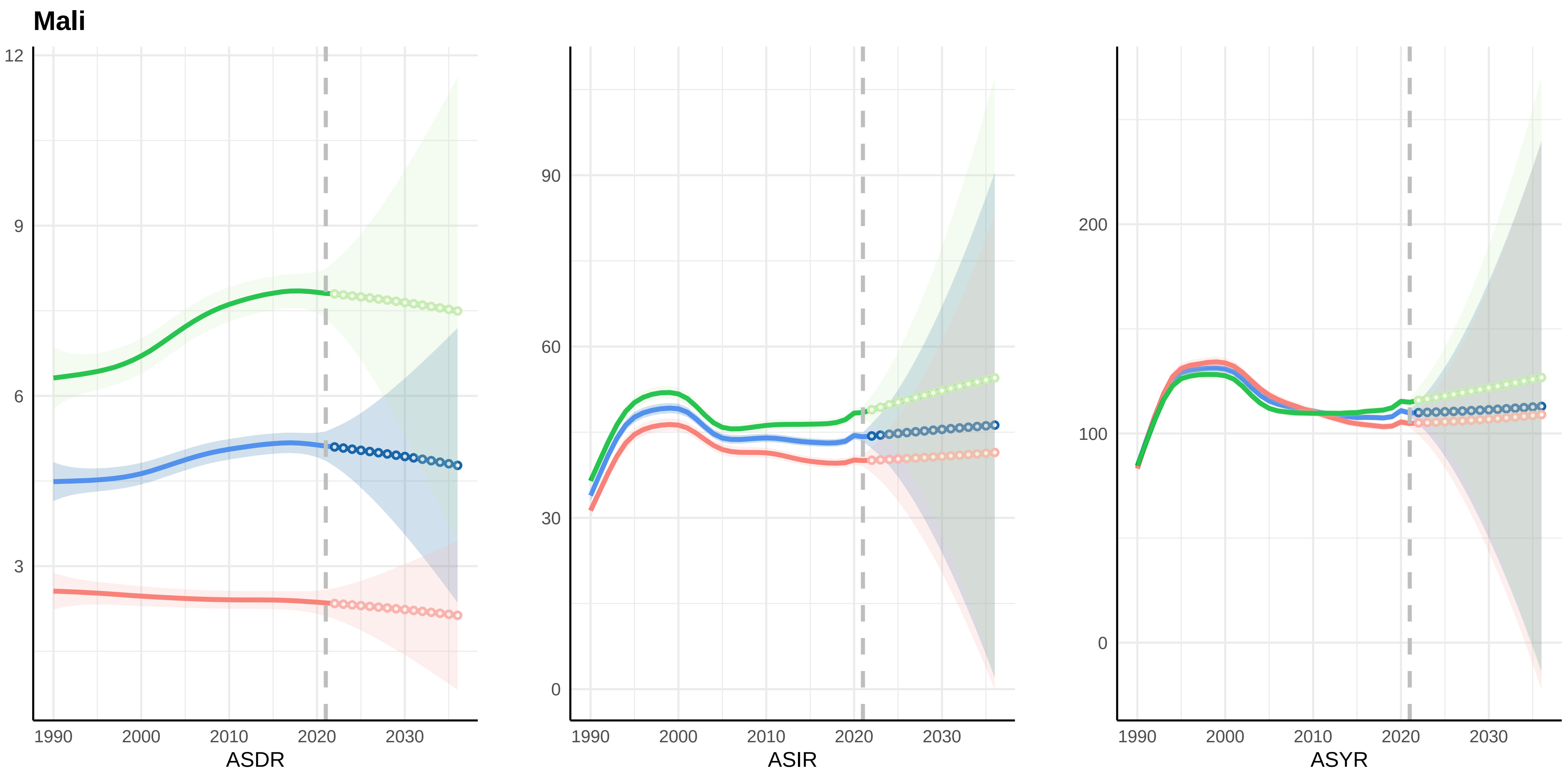

Mali
12
9
6
3
1990
2000
2010
2020
2030
ASDR
90
60
30
0
ASIR
200
100
0
ASYR

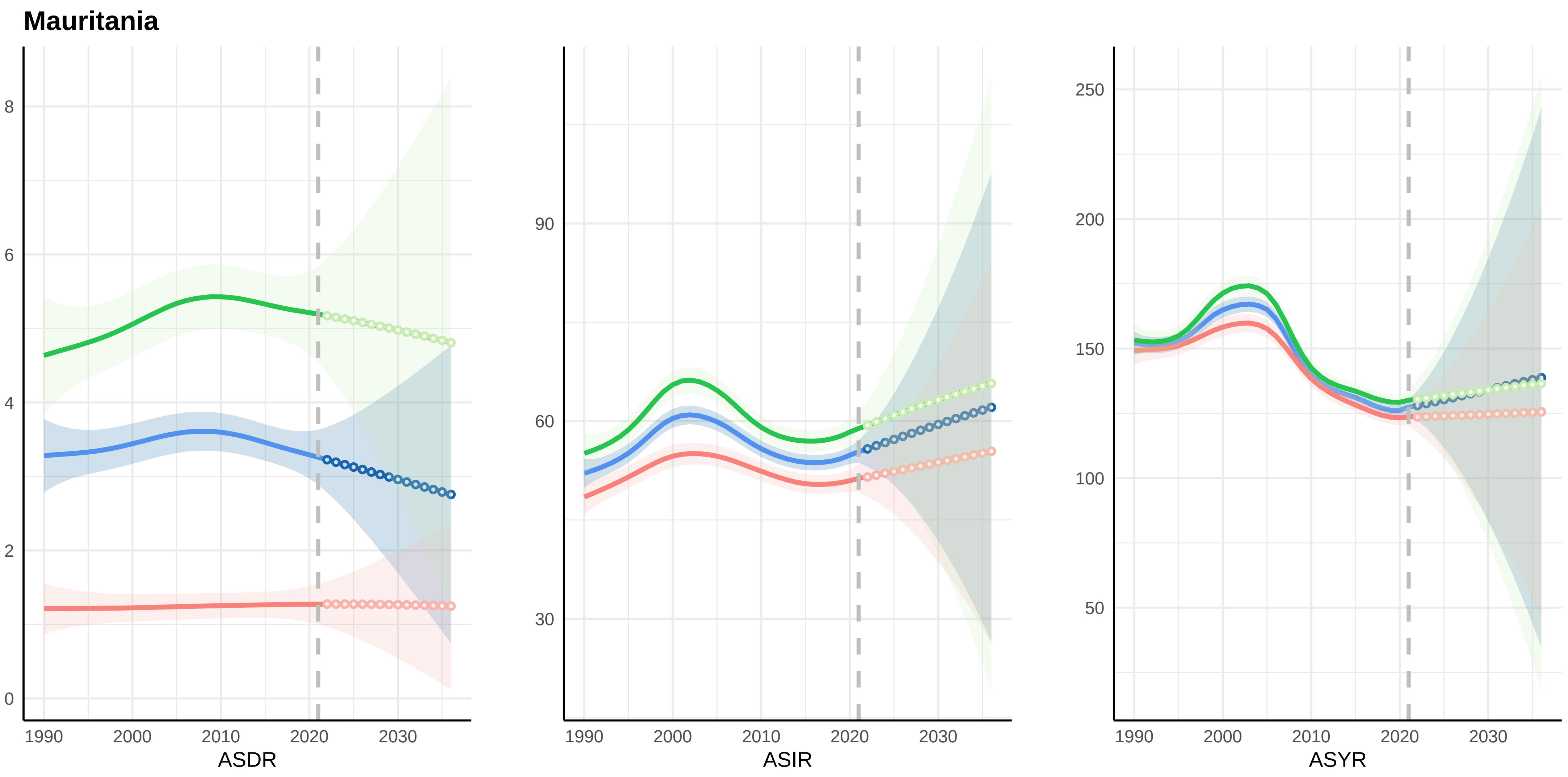

Mauritania
8
6
4
2
0
1990
2000
2010
2020
2030
ASDR
90
60
30
ASIR
250
200
150
100
50
ASYR

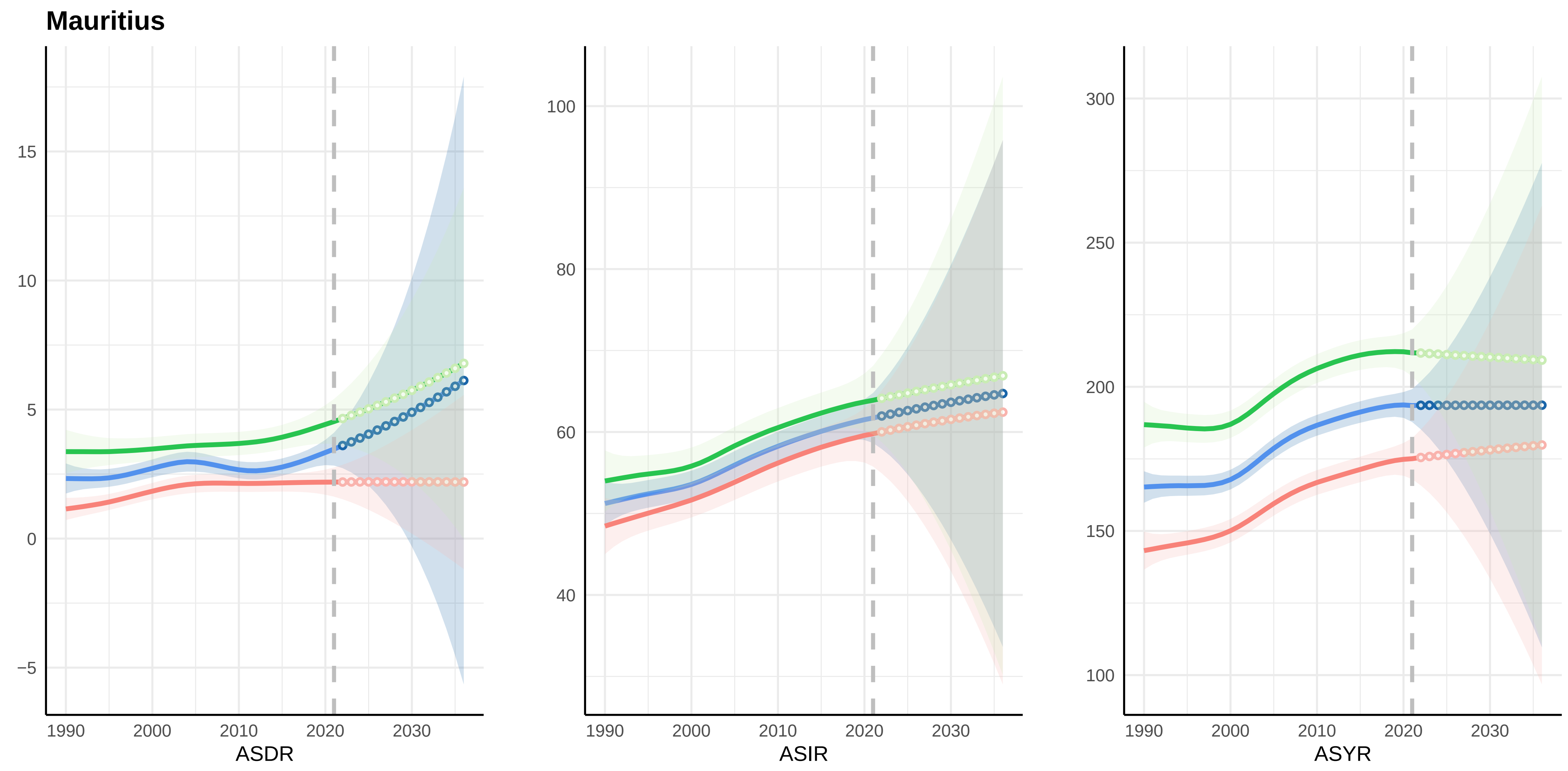

Mauritius
15
10
5
0
−5
1990
2000
2010
2020
2030
ASDR
100
80
60
40
1990
2000
2010
2020
2030
ASIR
300
250
200
150
100
1990
2000
2010
2020
2030
ASYR

Mexico

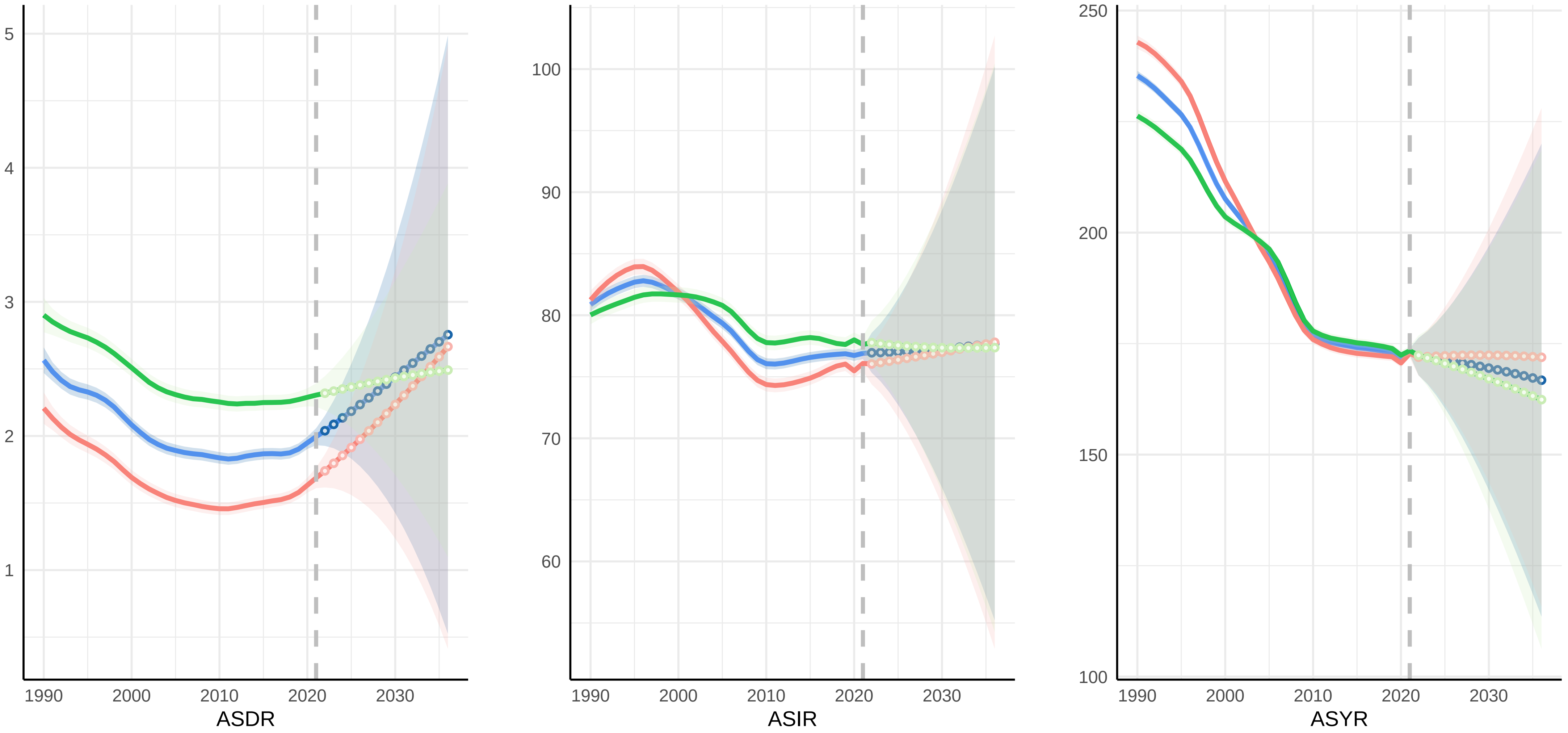

5
4
3
2
1
1990
2000
2010
2020
2030
ASDR
100
90
80
70
60
1990
2000
2010
2020
2030
ASIR
250
200
150
100
1990
2000
2010
2020
2030
ASYR

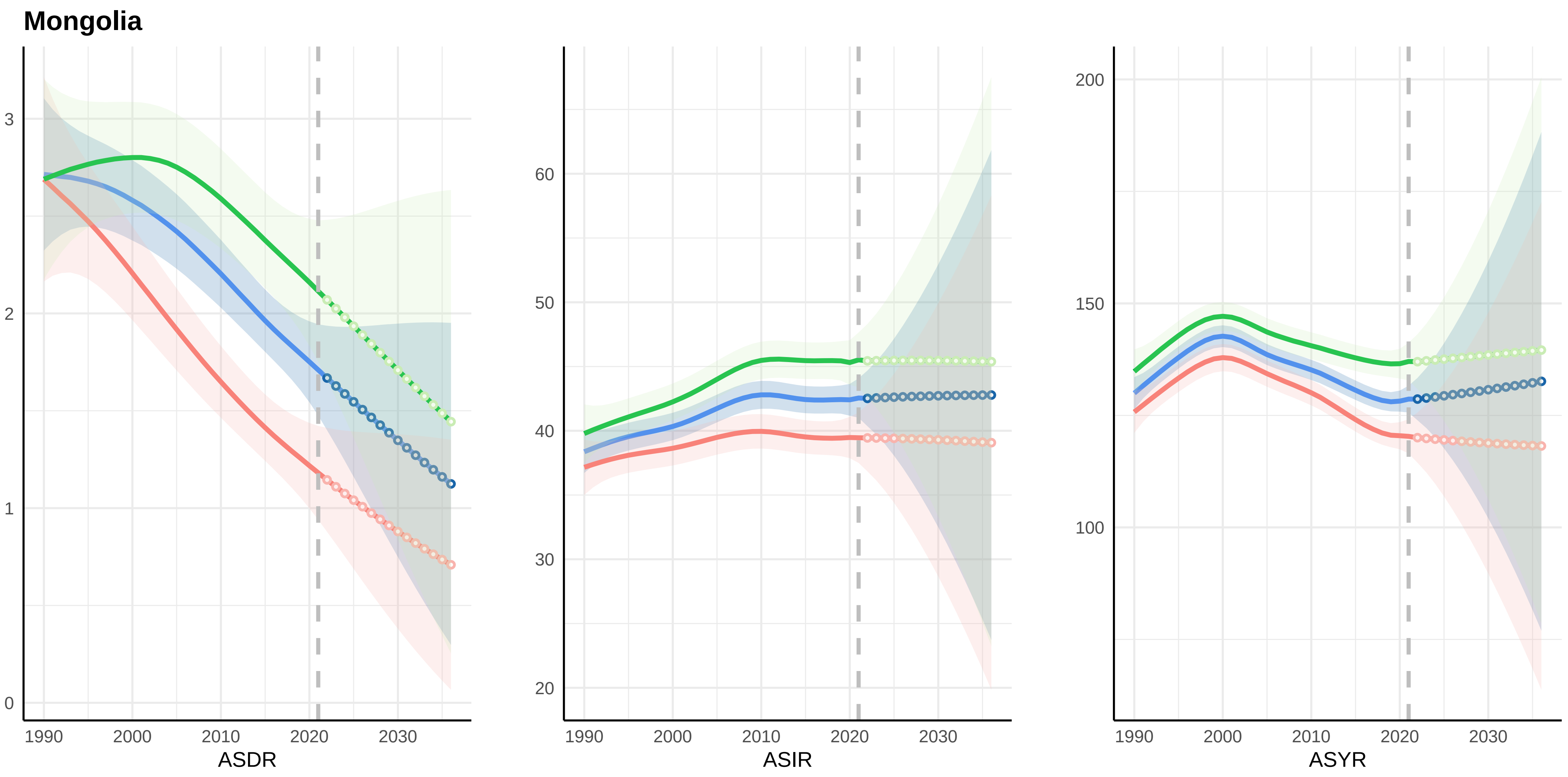

Mongolia
3
2
1
0
1990
2000
2010
2020
2030
ASDR
60
50
40
30
20
ASIR
200
150
100
ASYR

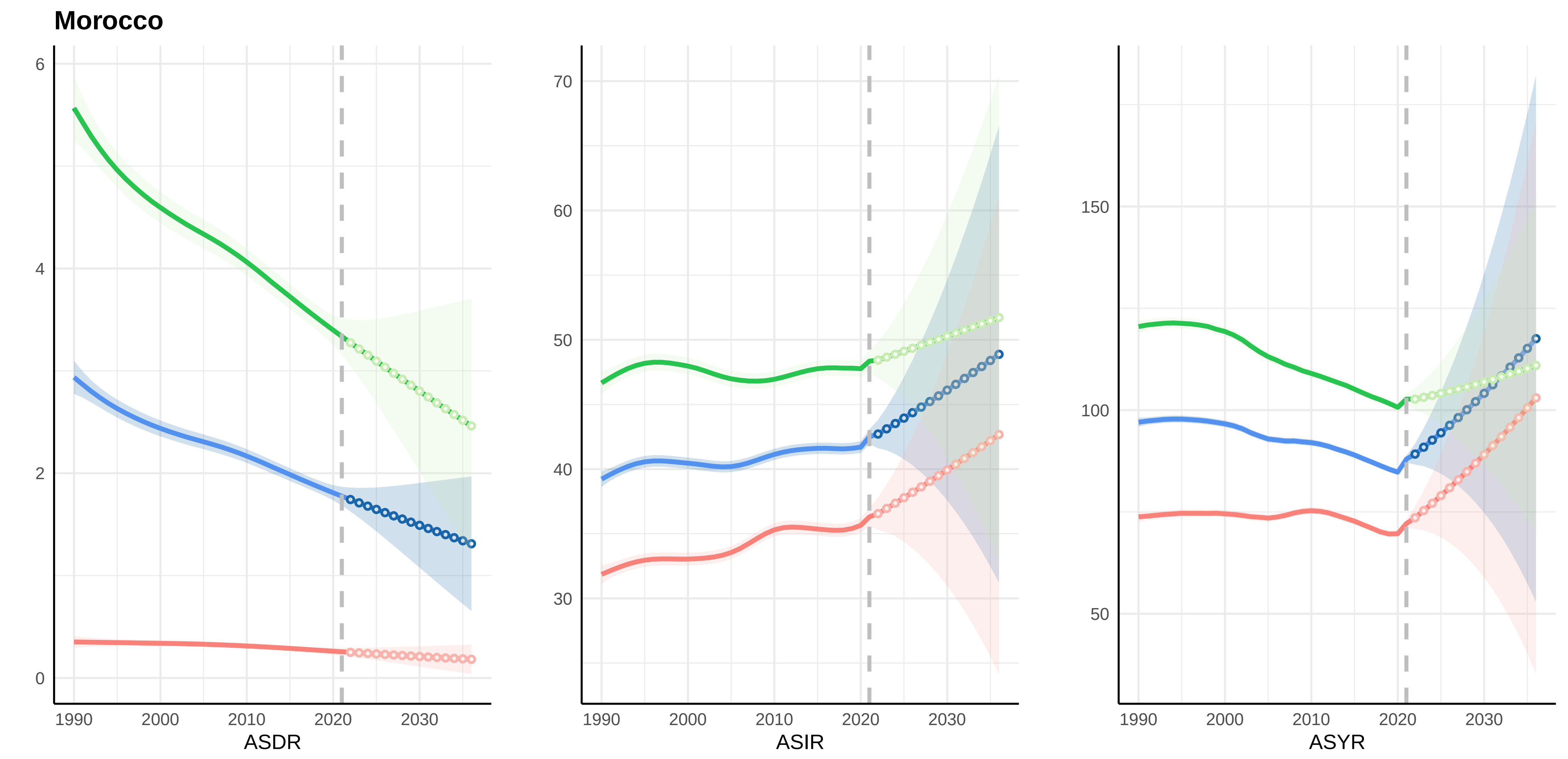

Morocco
6
4
2
0
1990
2000
2010
2020
2030
ASDR
70
60
50
40
30
1990
2000
2010
2020
2030
ASIR
150
100
50
1990
2000
2010
2020
2030
ASYR

Mozambique

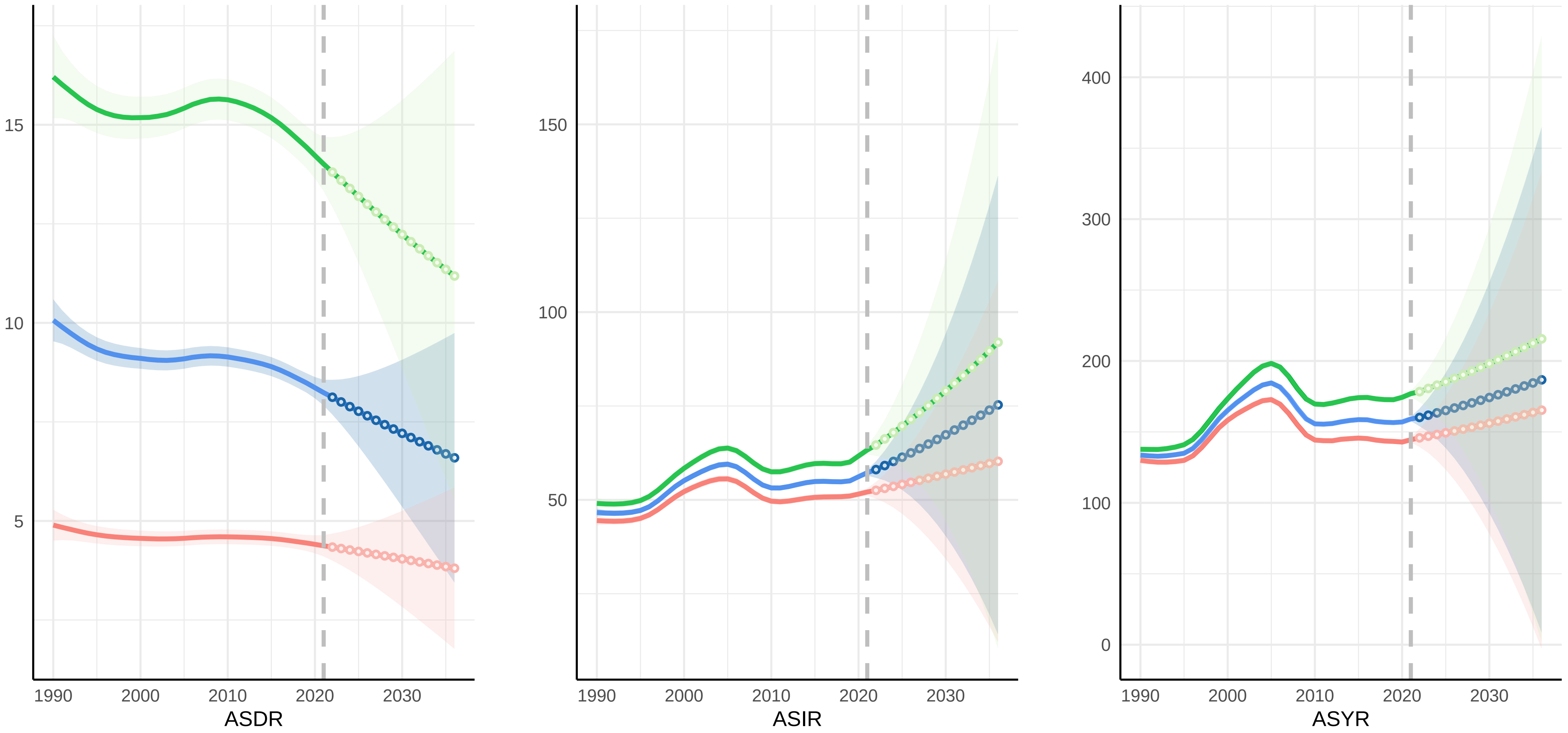

15
10
5
1990
2000
2010
2020
2030
ASDR
150
100
50
1990
2000
2010
2020
2030
ASIR
400
300
200
100
0
1990
2000
2010
2020
2030
ASYR

Myanmar

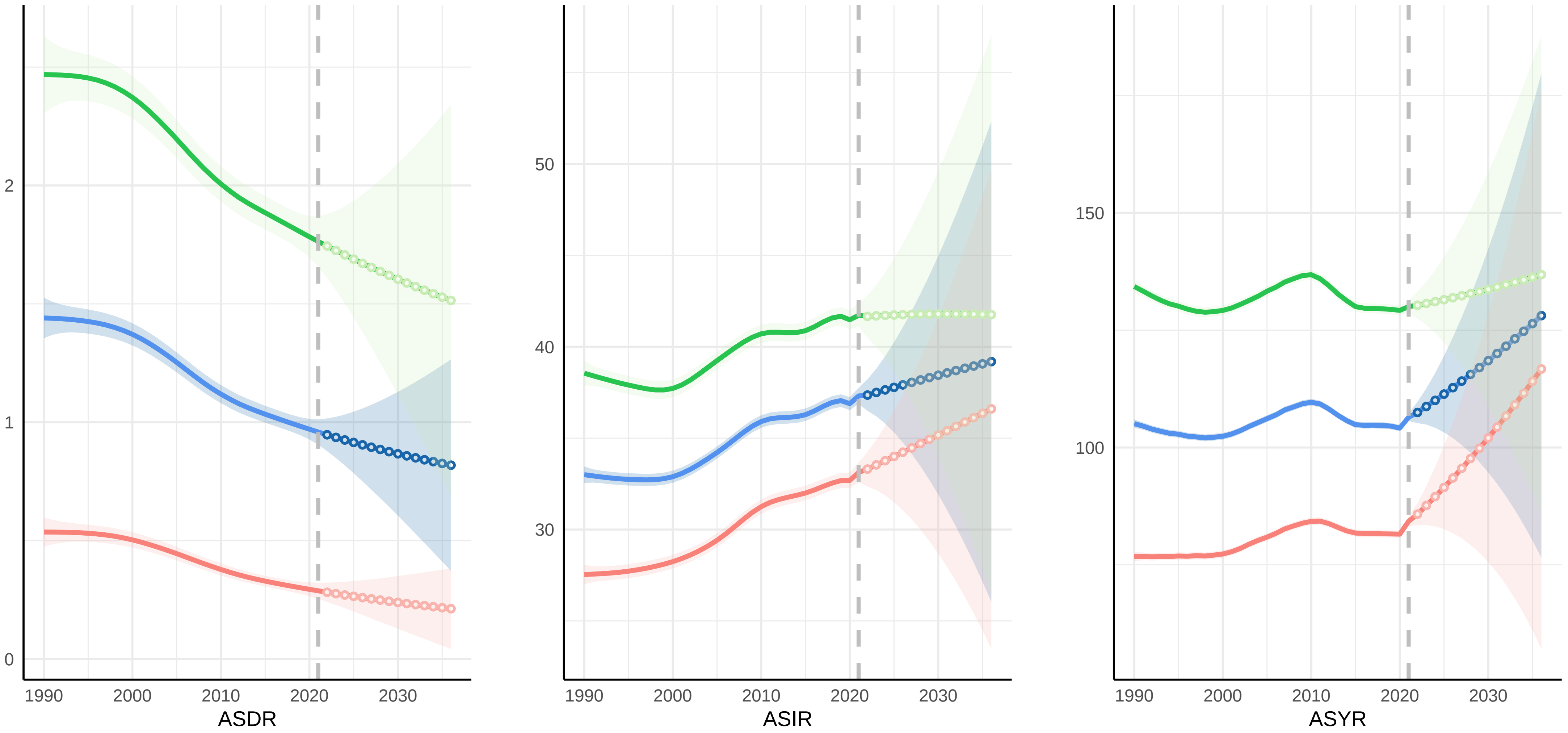

Namibia

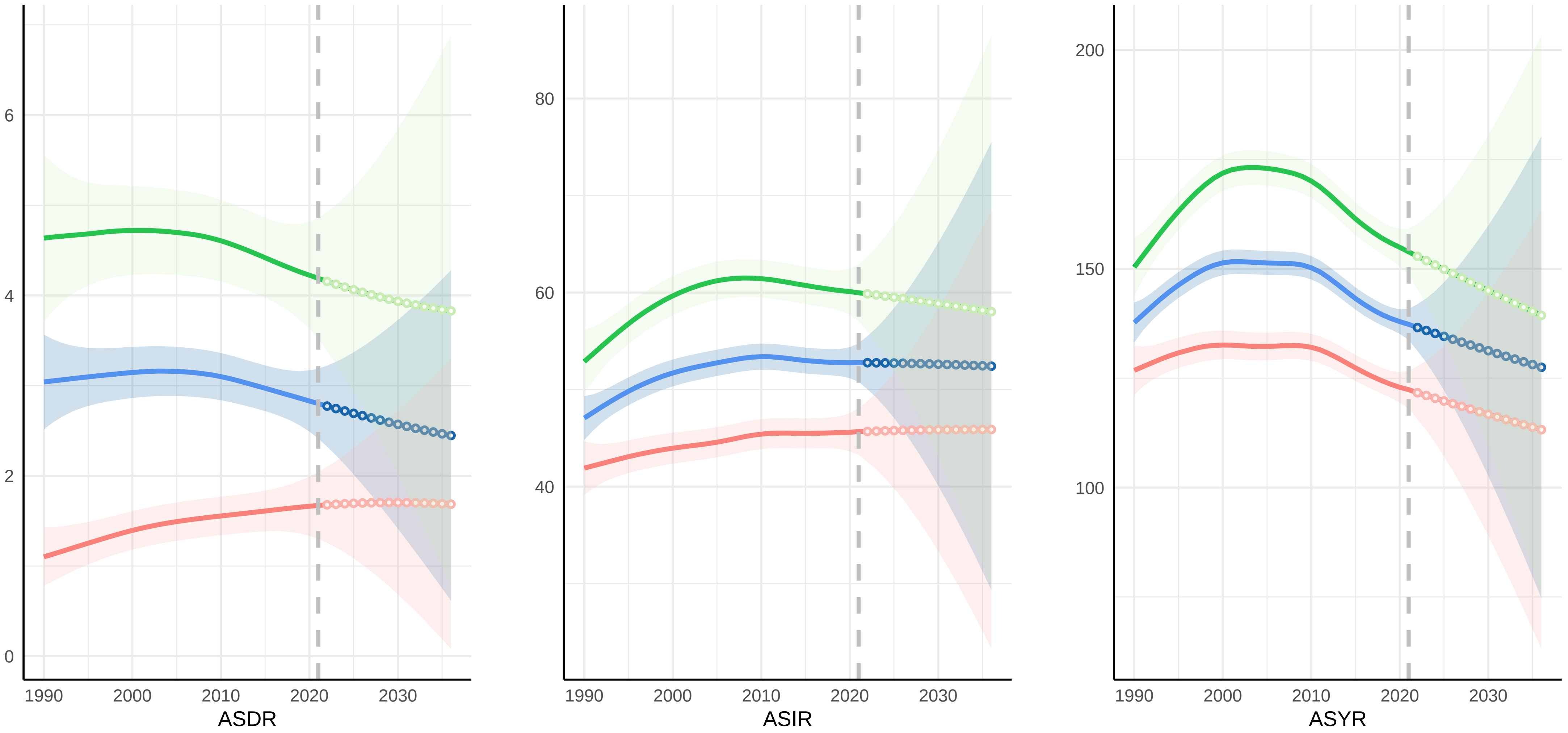

6
4
2
0
1990
2000
2010
2020
2030
ASDR
80
60
40
1990
2000
2010
2020
2030
ASIR
200
150
100
1990
2000
2010
2020
2030
ASYR

Nepal

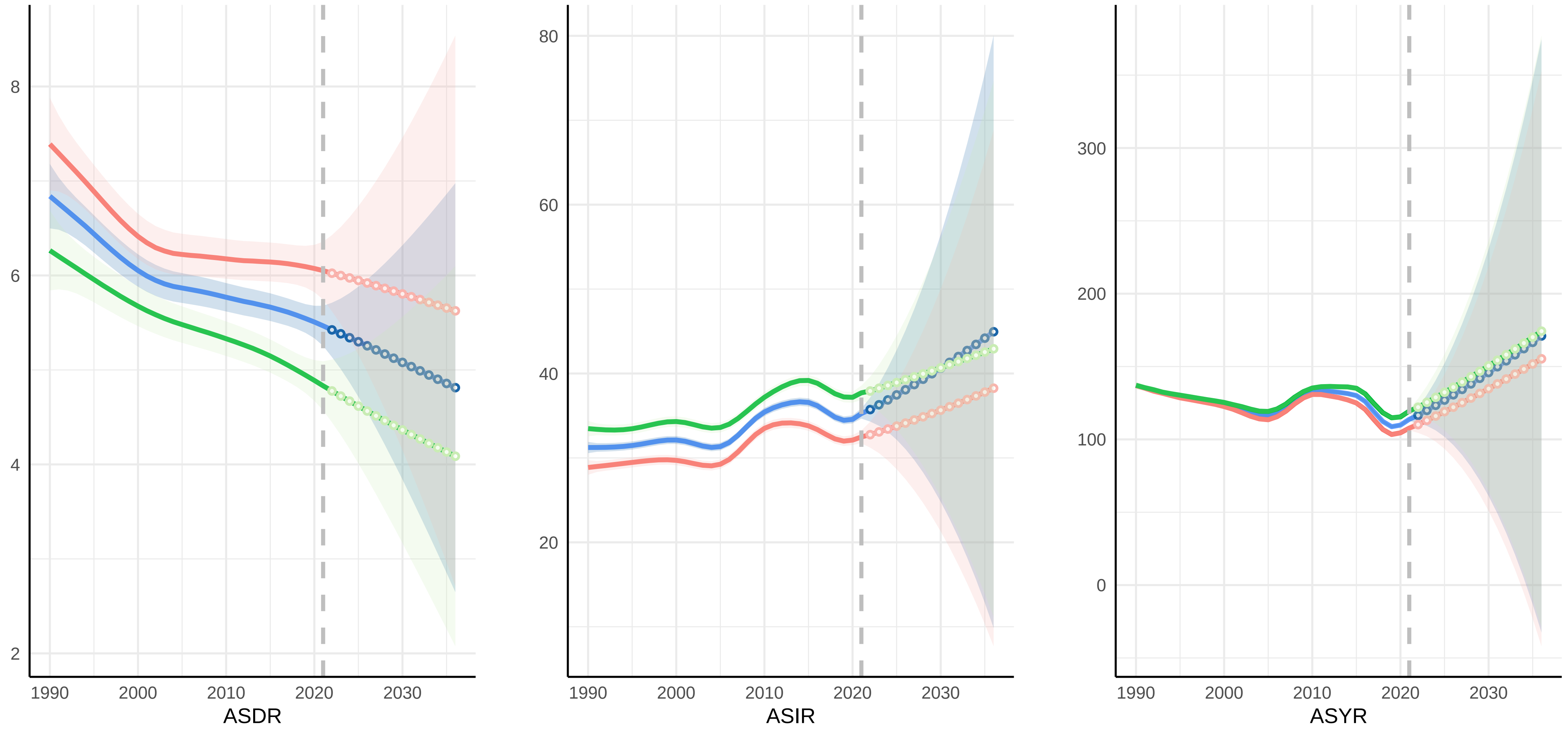

8
6
4
2
1990
2000
2010
2020
2030
ASDR
80
60
40
20
1990
2000
2010
2020
2030
ASIR
300
200
100
0
1990
2000
2010
2020
2030
ASYR

Netherlands

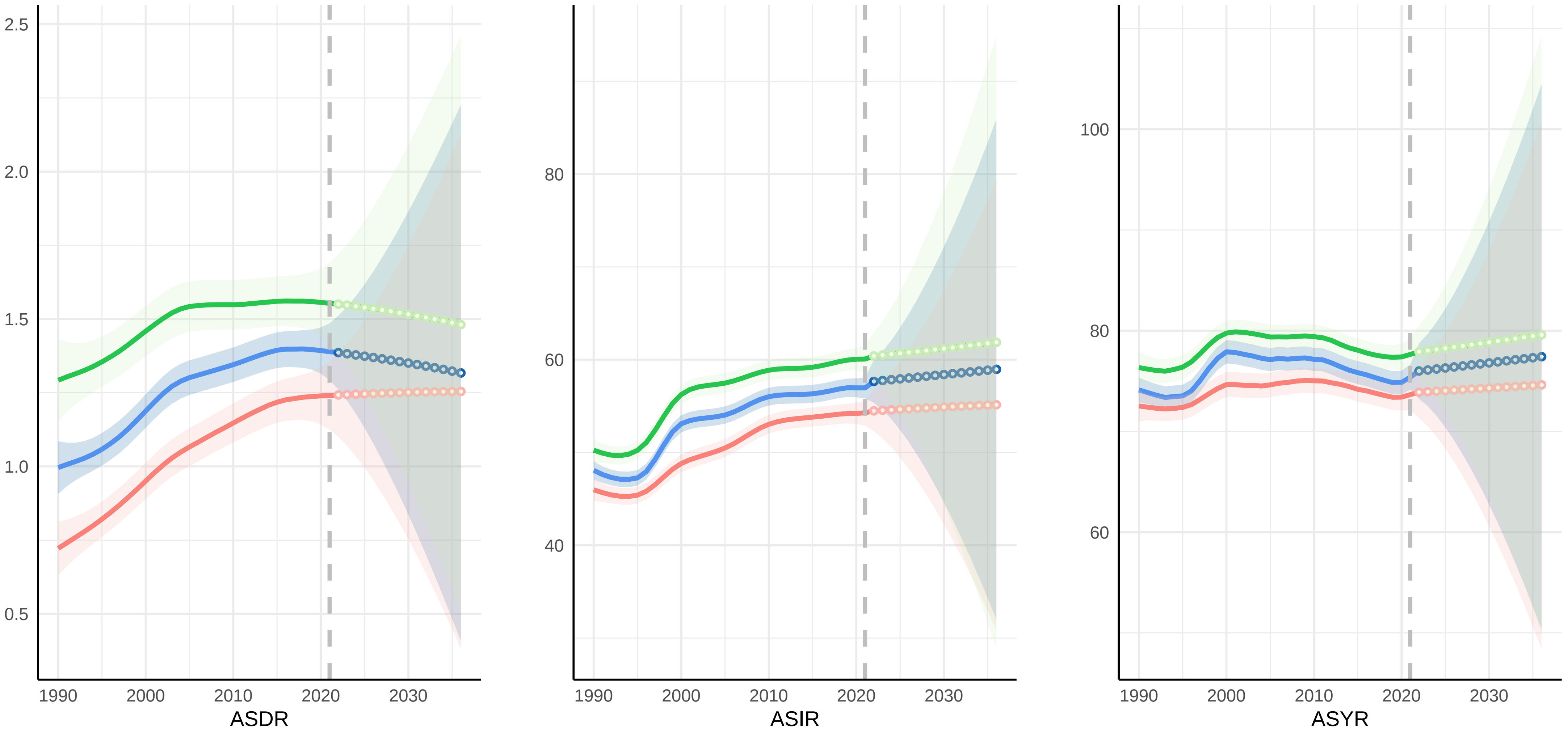

New Zealand

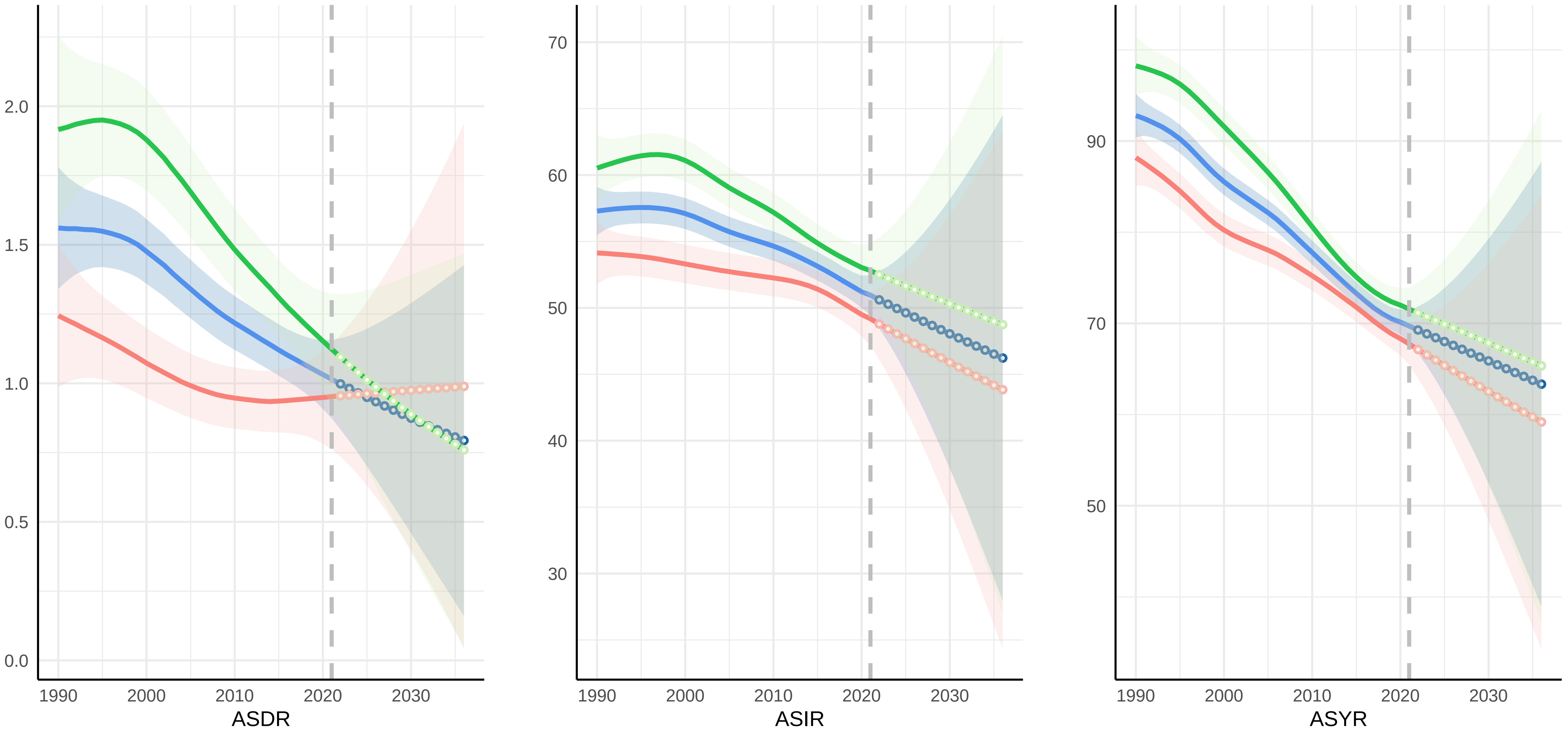

2.0
1.5
1.0
0.5
0.0
1990
2000
2010
2020
2030
ASDR
70
60
50
40
30
ASIR
90
70
50
ASYR

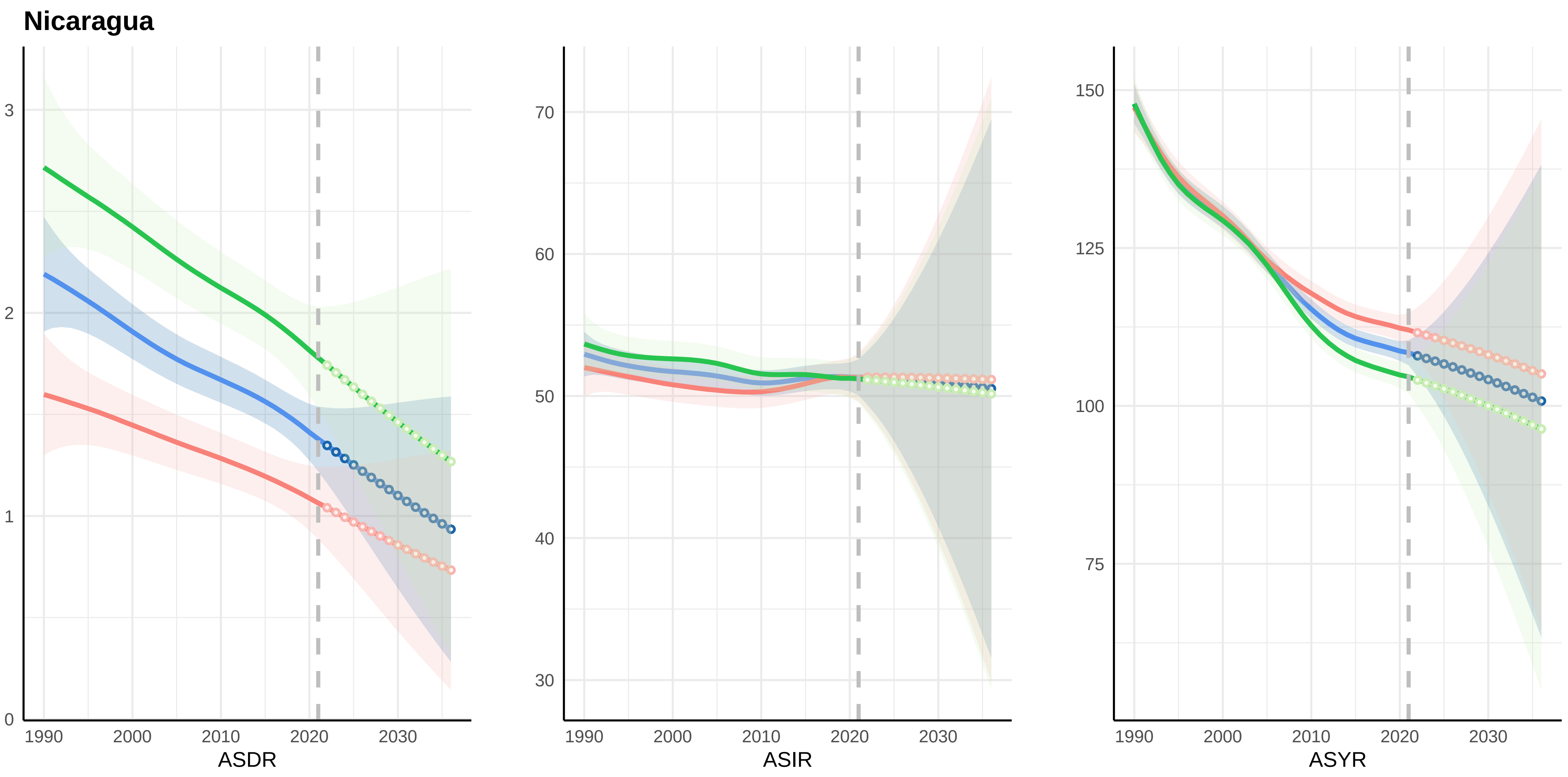
Nicaragua
3
2
1
0
1990
2000
2010
2020
2030
ASDR
70
60
50
40
30
1990
2000
2010
2020
2030
ASIR
150
125
100
75
1990
2000
2010
2020
2030
ASYR

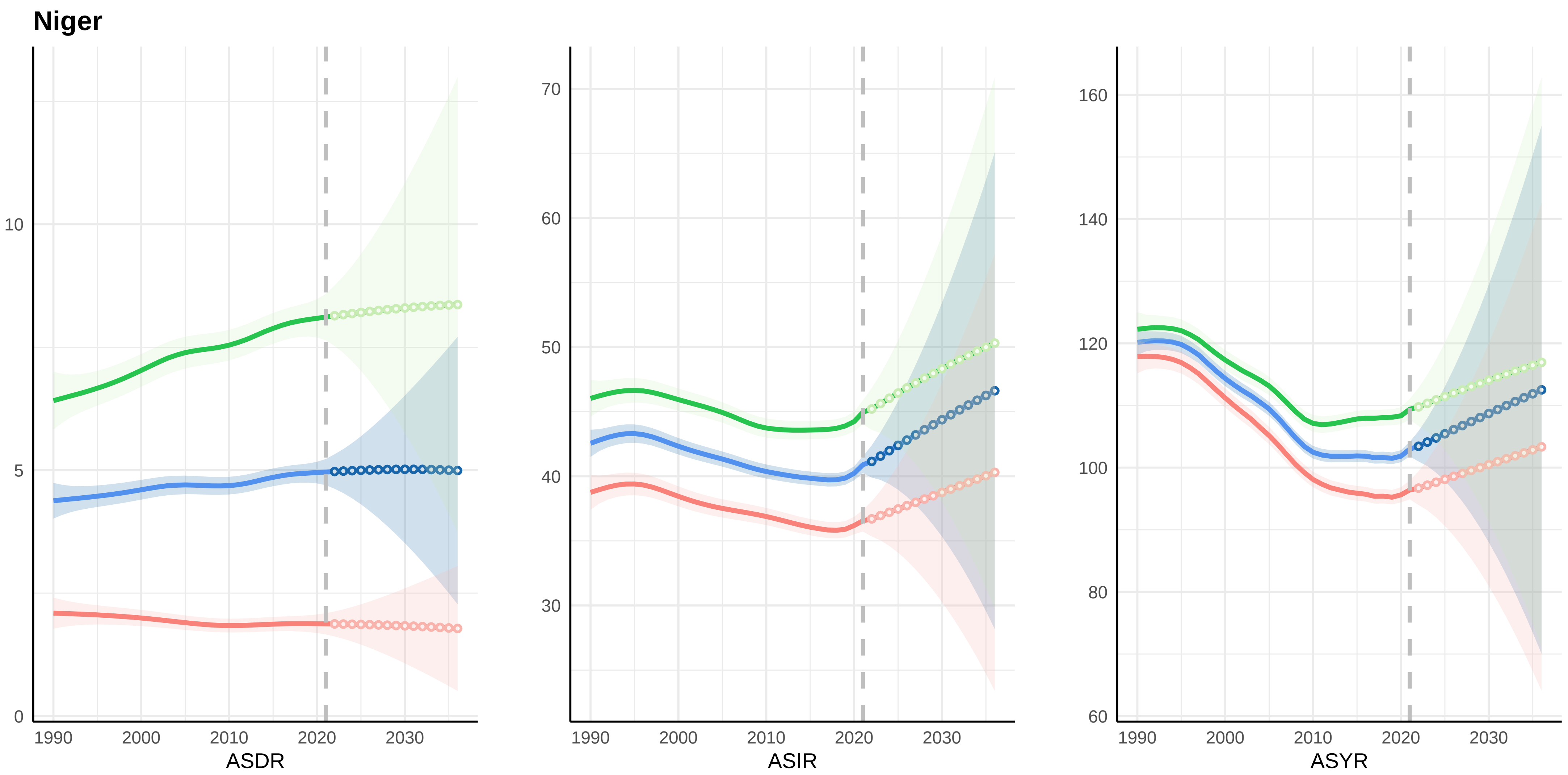

Niger
10
5
0
1990
2000
2010
2020
2030
ASDR
70
60
50
40
30
ASIR
160
140
120
100
80
60
ASYR

Nigeria

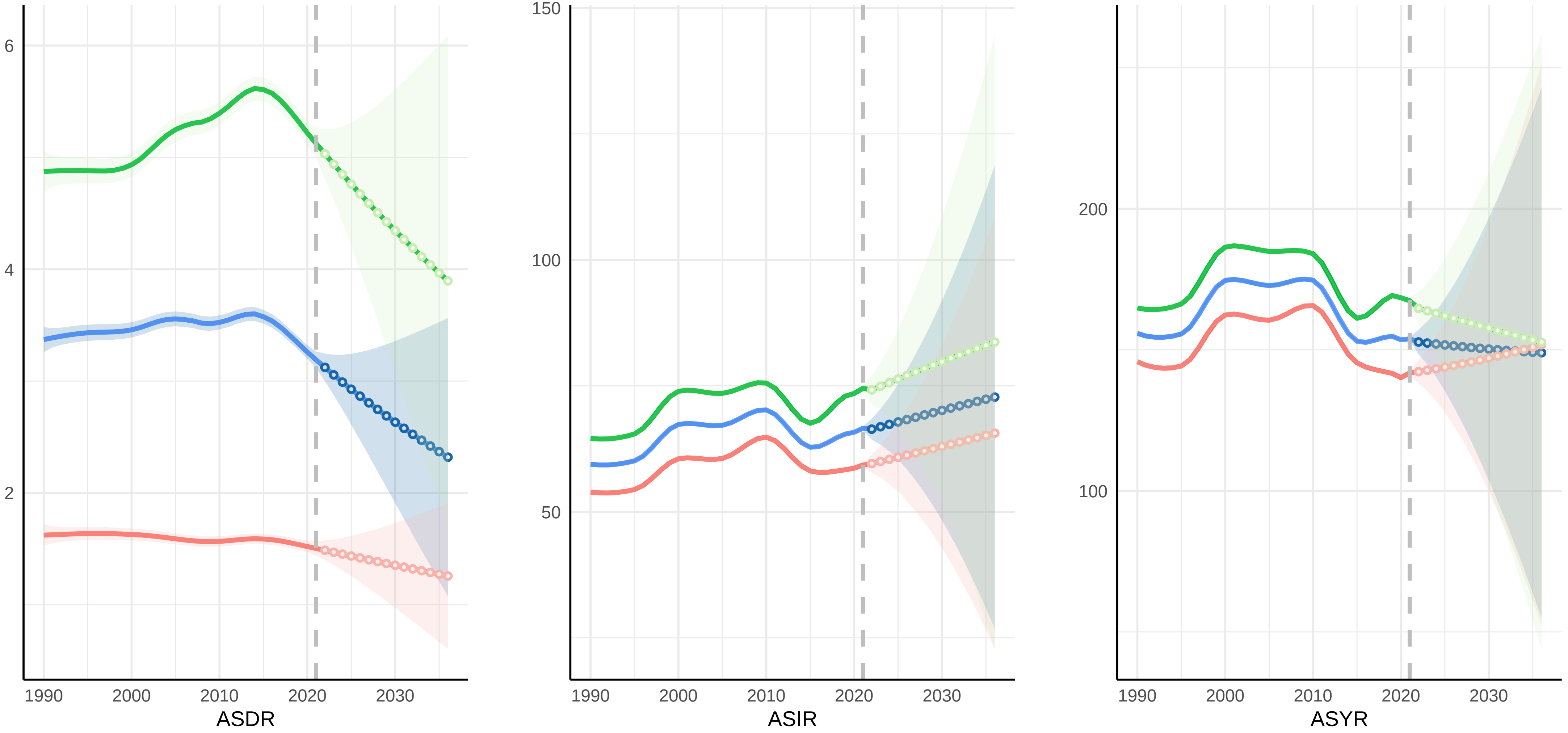

6
4
2
1990
2000
2010
2020
2030
ASDR
150
100
50
1990
2000
2010
2020
2030
ASIR
200
100
1990
2000
2010
2020
2030
ASYR

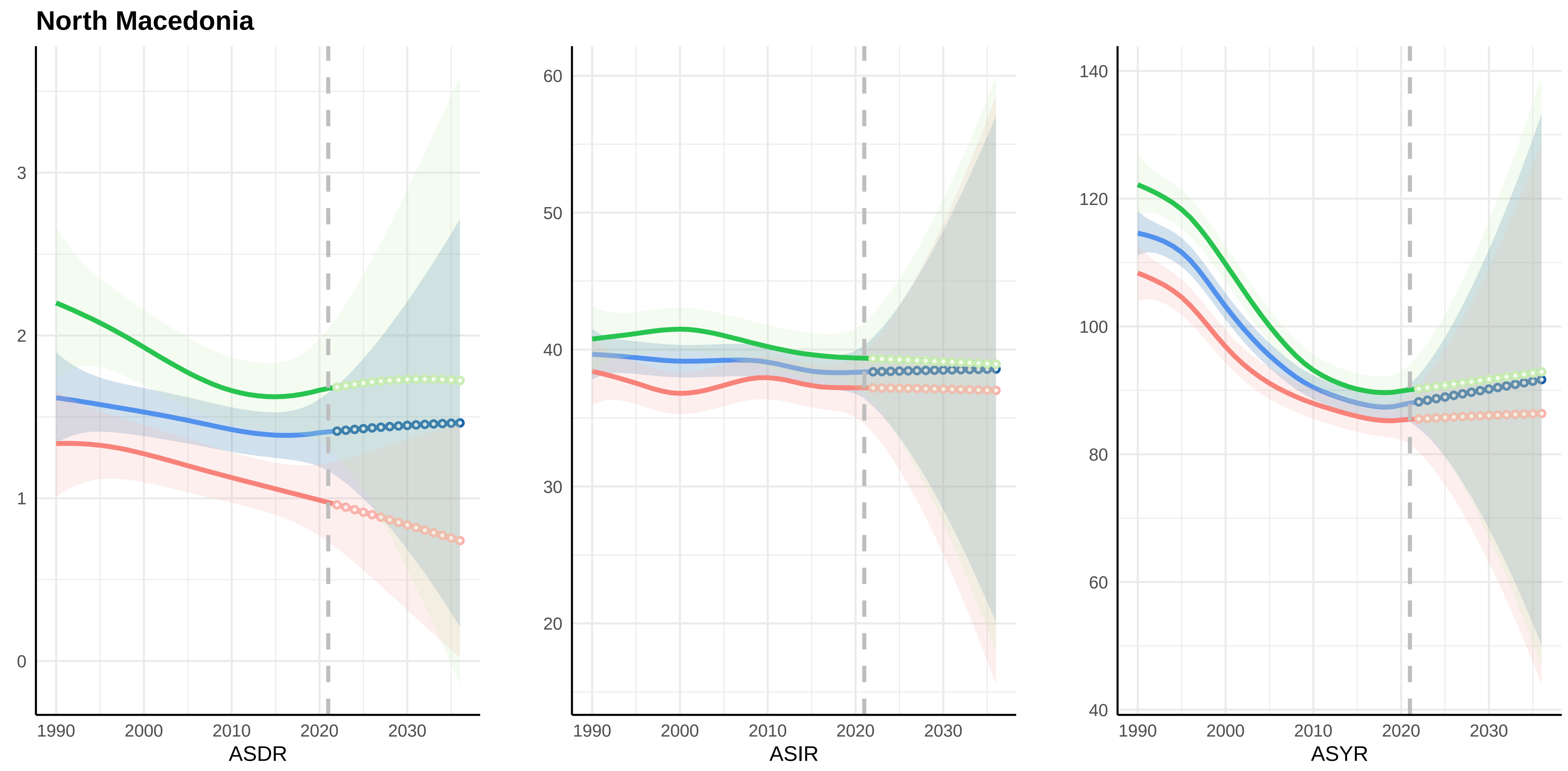

North Macedonia
0
1
2
3
1990
2000
2010
2020
2030
ASDR
20
30
40
50
60
1990
2000
2010
2020
2030
ASIR
40
60
80
100
120
140
1990
2000
2010
2020
2030
ASYR

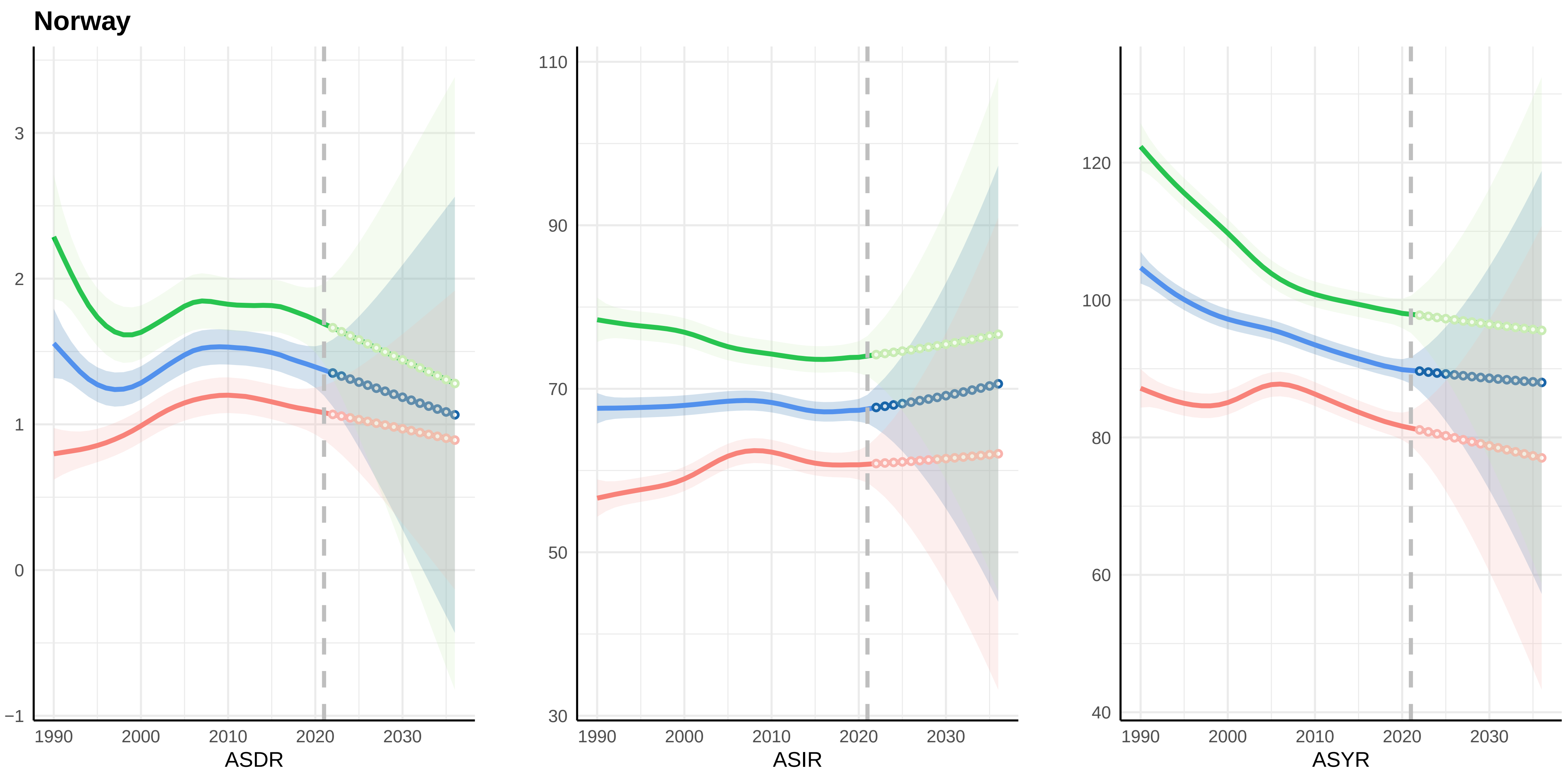
Norway
3
2
1
0
−1
1990
2000
2010
2020
2030
ASDR
110
90
70
50
30
ASIR
120
100
80
60
40
ASYR

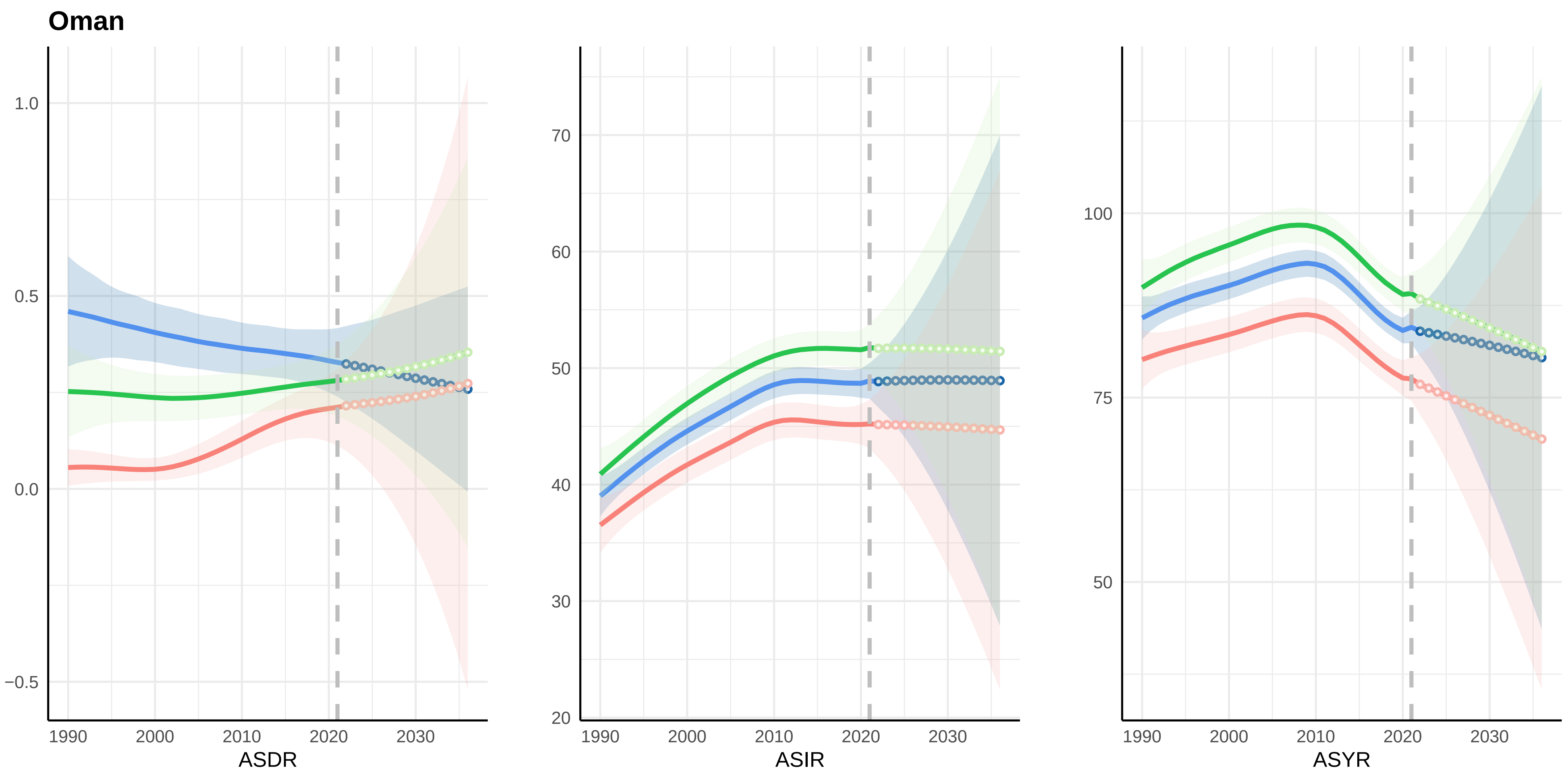

Oman
1.0
0.5
0.0
−0.5
1990
2000
2010
2020
2030
ASDR
70
60
50
40
30
20
1990
2000
2010
2020
2030
ASIR
100
75
50
1990
2000
2010
2020
2030
ASYR

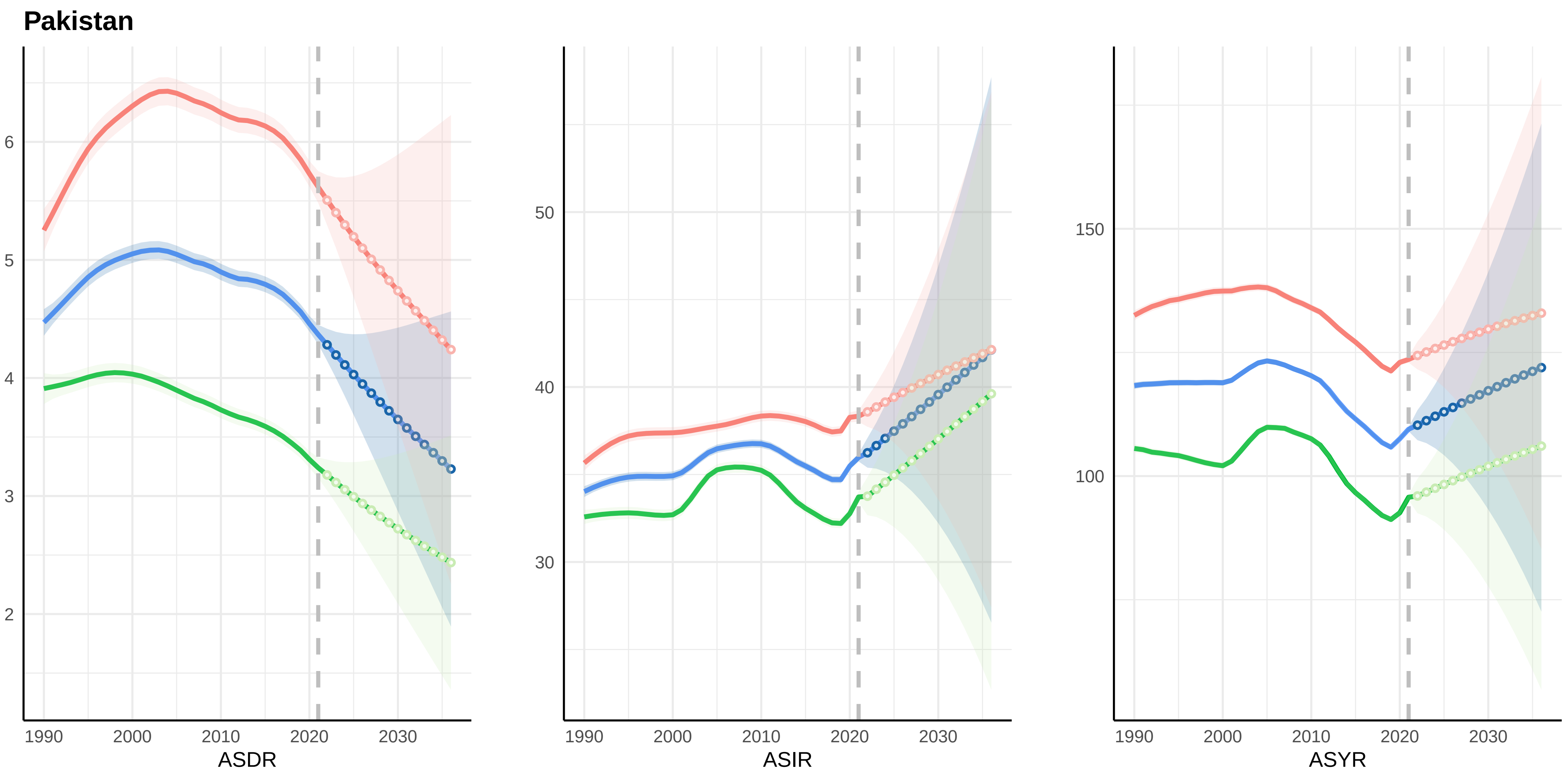
Pakistan
6
5
4
3
2
1990
2000
2010
2020
2030
ASDR
50
40
30
ASIR
150
100
ASYR

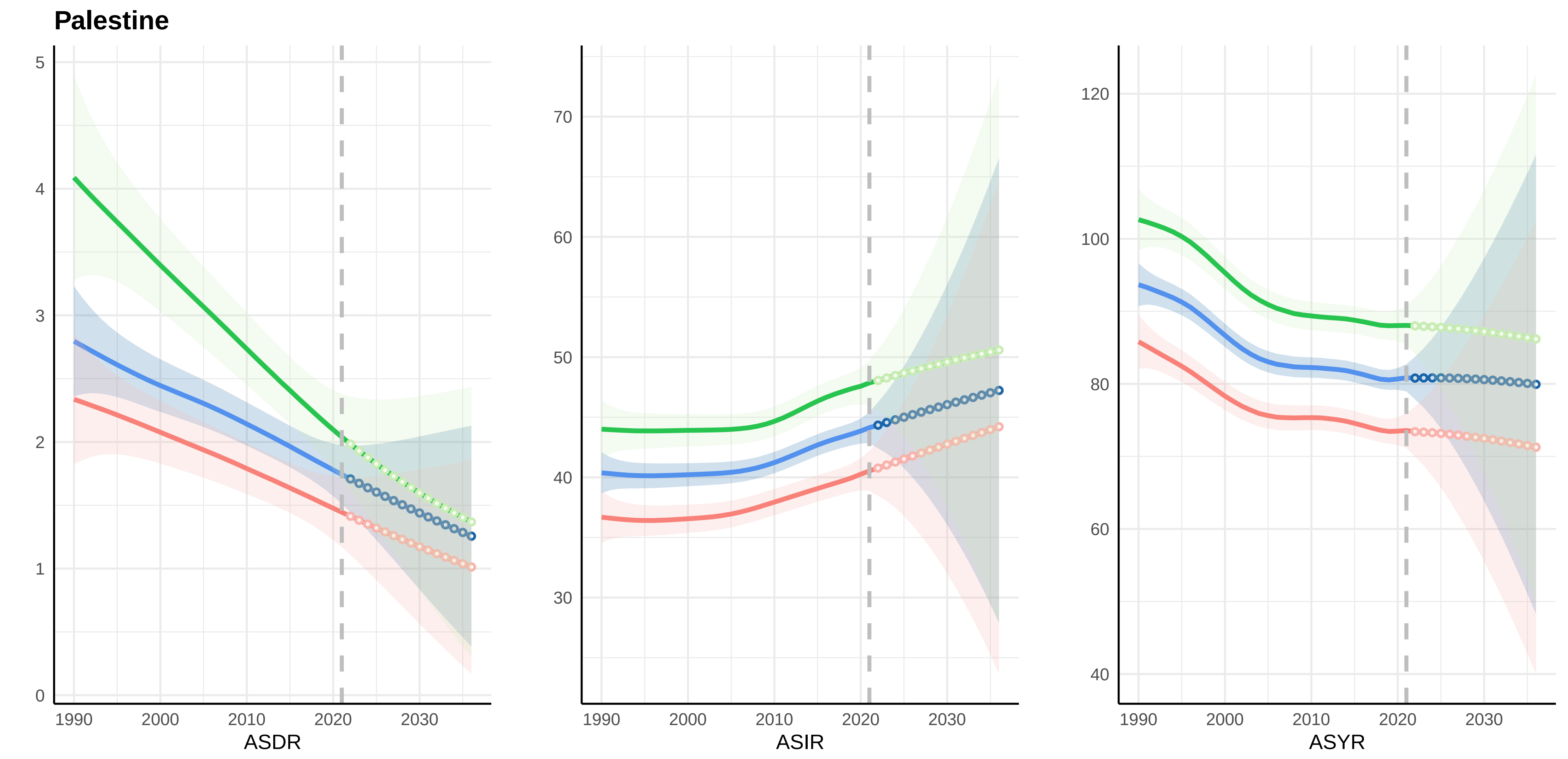

Palestine
5
4
3
2
1
0
1990
2000
2010
2020
2030
ASDR
70
60
50
40
30
1990
2000
2010
2020
2030
ASIR
120
100
80
60
40
1990
2000
2010
2020
2030
ASYR

Panama

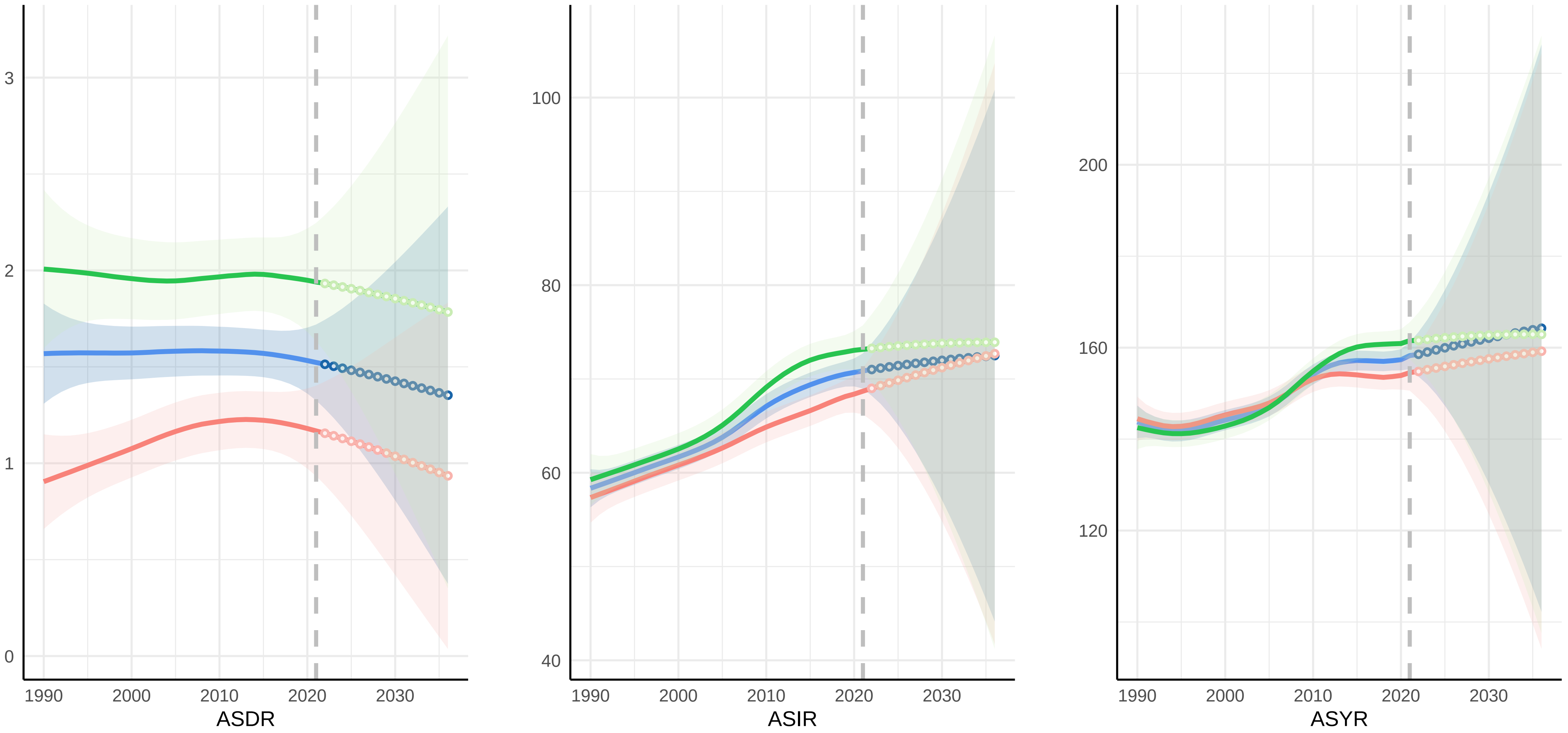

3
2
1
0
1990
2000
2010
2020
2030
ASDR
100
80
60
40
1990
2000
2010
2020
2030
ASIR
200
160
120
1990
2000
2010
2020
2030
ASYR

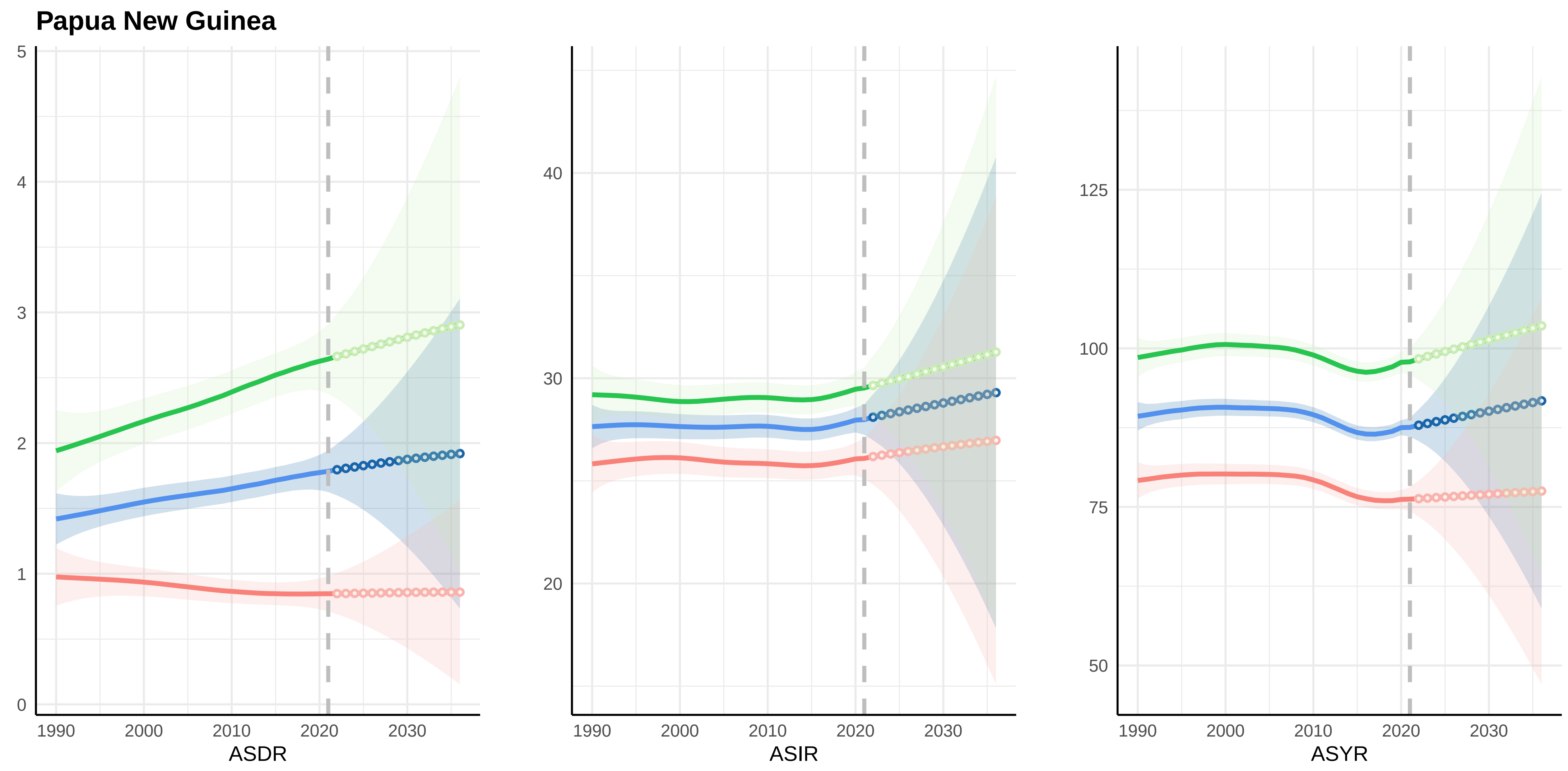

Papua New Guinea
0
1
2
3
4
5
1990
2000
2010
2020
2030
ASDR
20
30
40
1990
2000
2010
2020
2030
ASIR
50
75
100
125
1990
2000
2010
2020
2030
ASYR

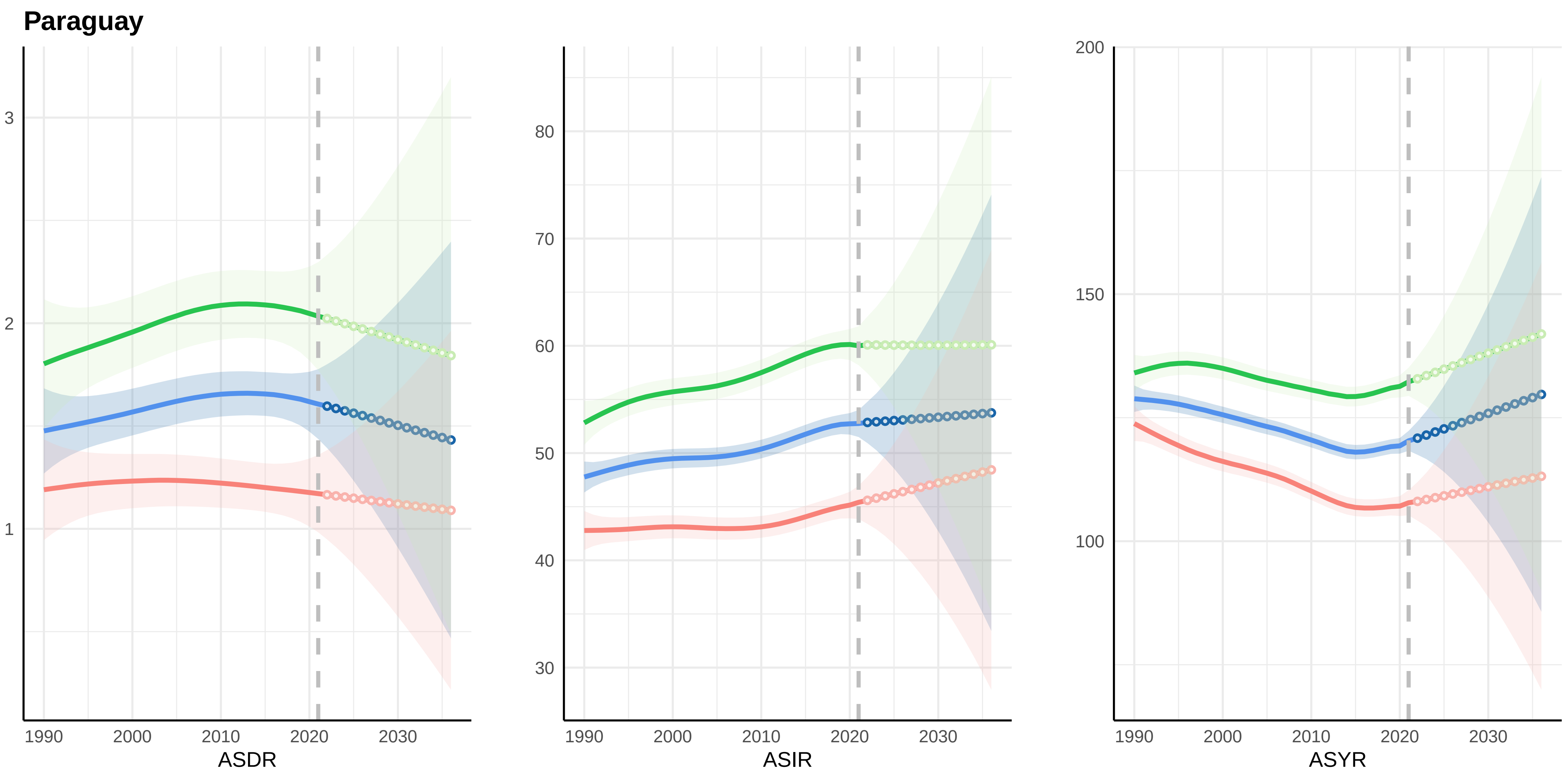

Paraguay
3
2
1
1990
2000
2010
2020
2030
ASDR
80
70
60
50
40
30
1990
2000
2010
2020
2030
ASIR
200
150
100
1990
2000
2010
2020
2030
ASYR

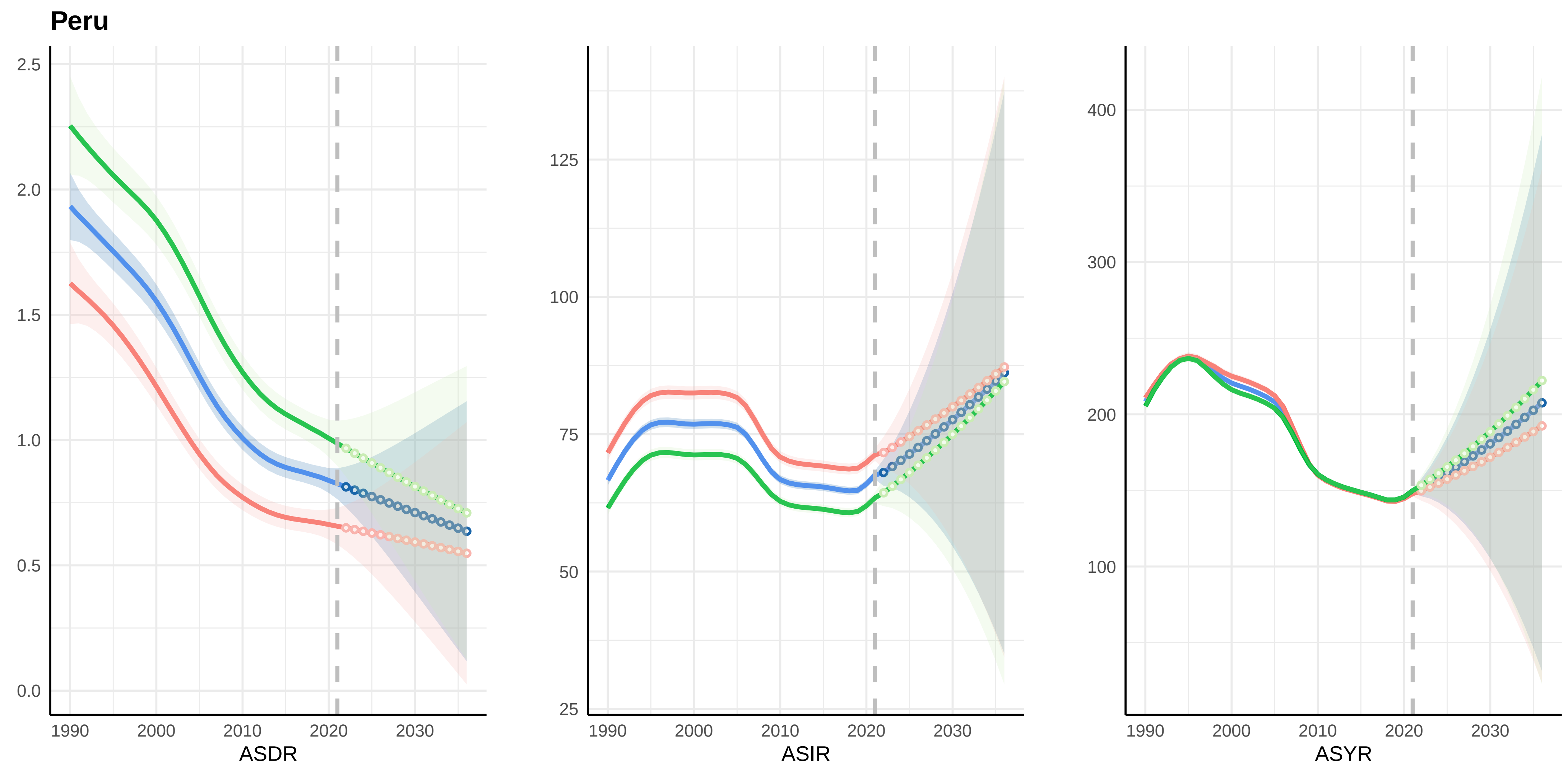
Peru
2.5
2.0
1.5
1.0
0.5
0.0
1990
2000
2010
2020
2030
ASDR
125
100
75
50
25
1990
2000
2010
2020
2030
ASIR
400
300
200
100
1990
2000
2010
2020
2030
ASYR

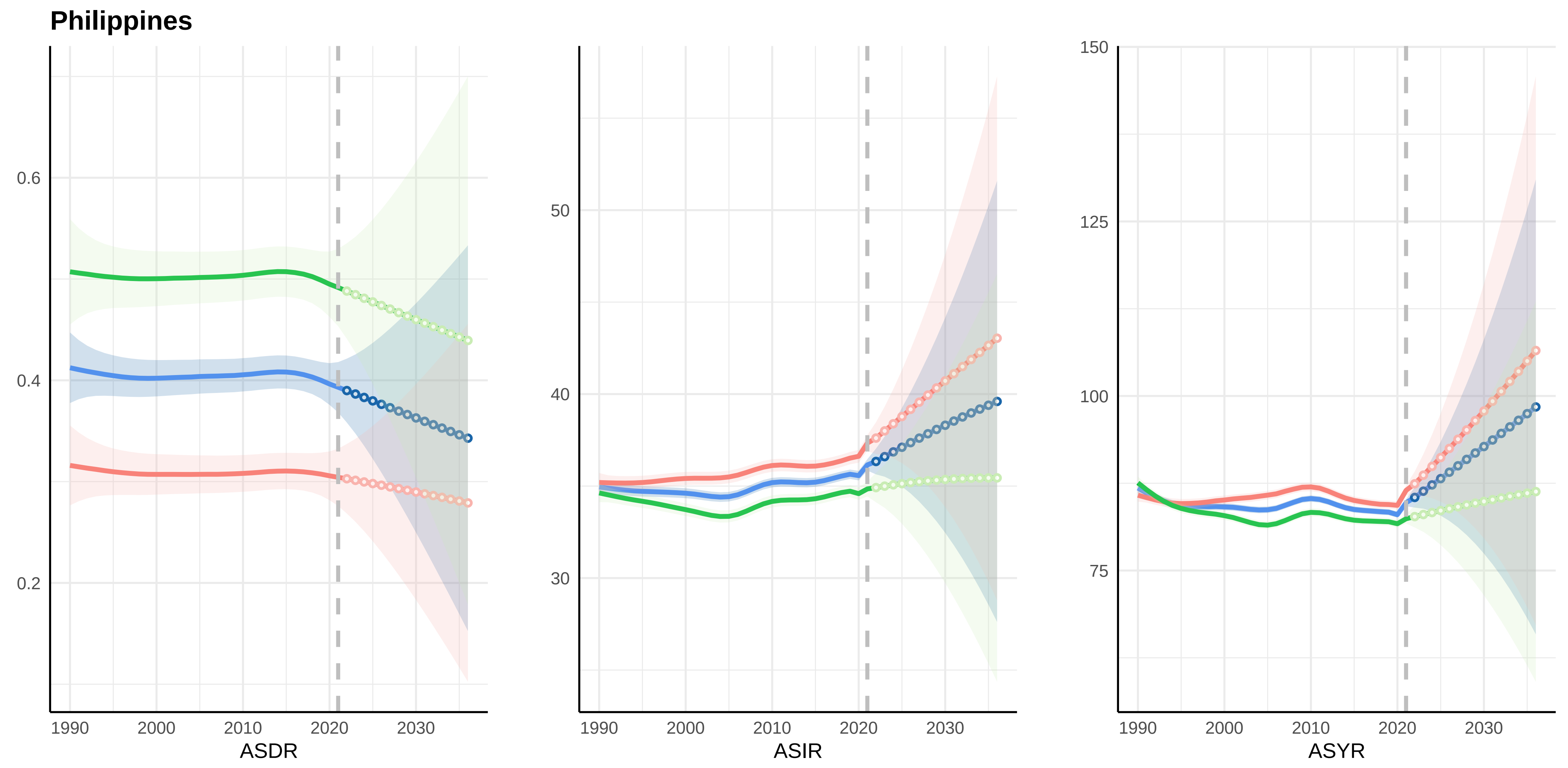
Philippines
0.6
0.4
0.2
1990
2000
2010
2020
2030
ASDR
50
40
30
ASIR
150
125
100
75
ASYR

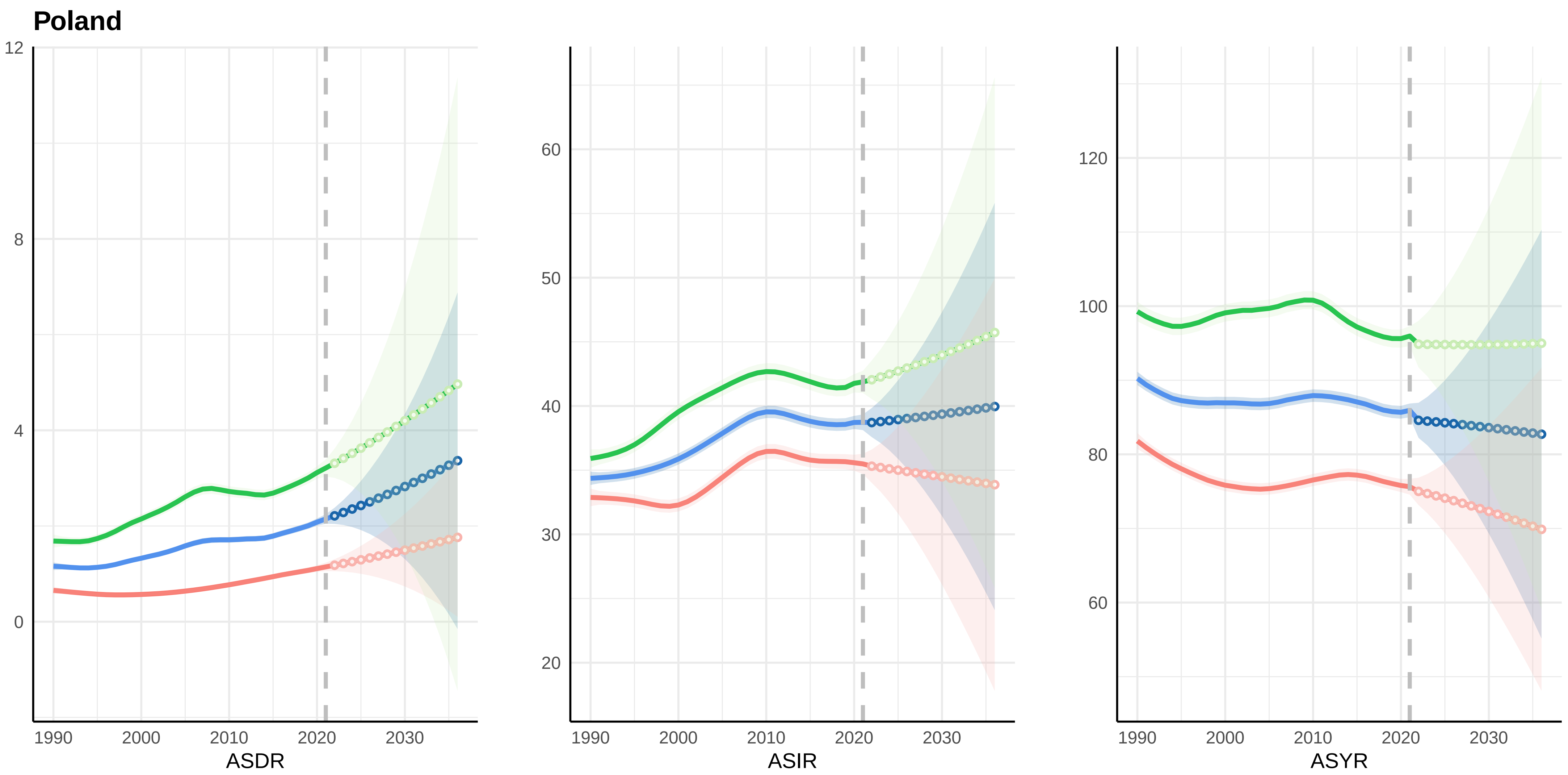

Poland
12
8
4
0
1990
2000
2010
2020
2030
ASDR
60
50
40
30
20
1990
2000
2010
2020
2030
ASIR
120
100
80
60
1990
2000
2010
2020
2030
ASYR

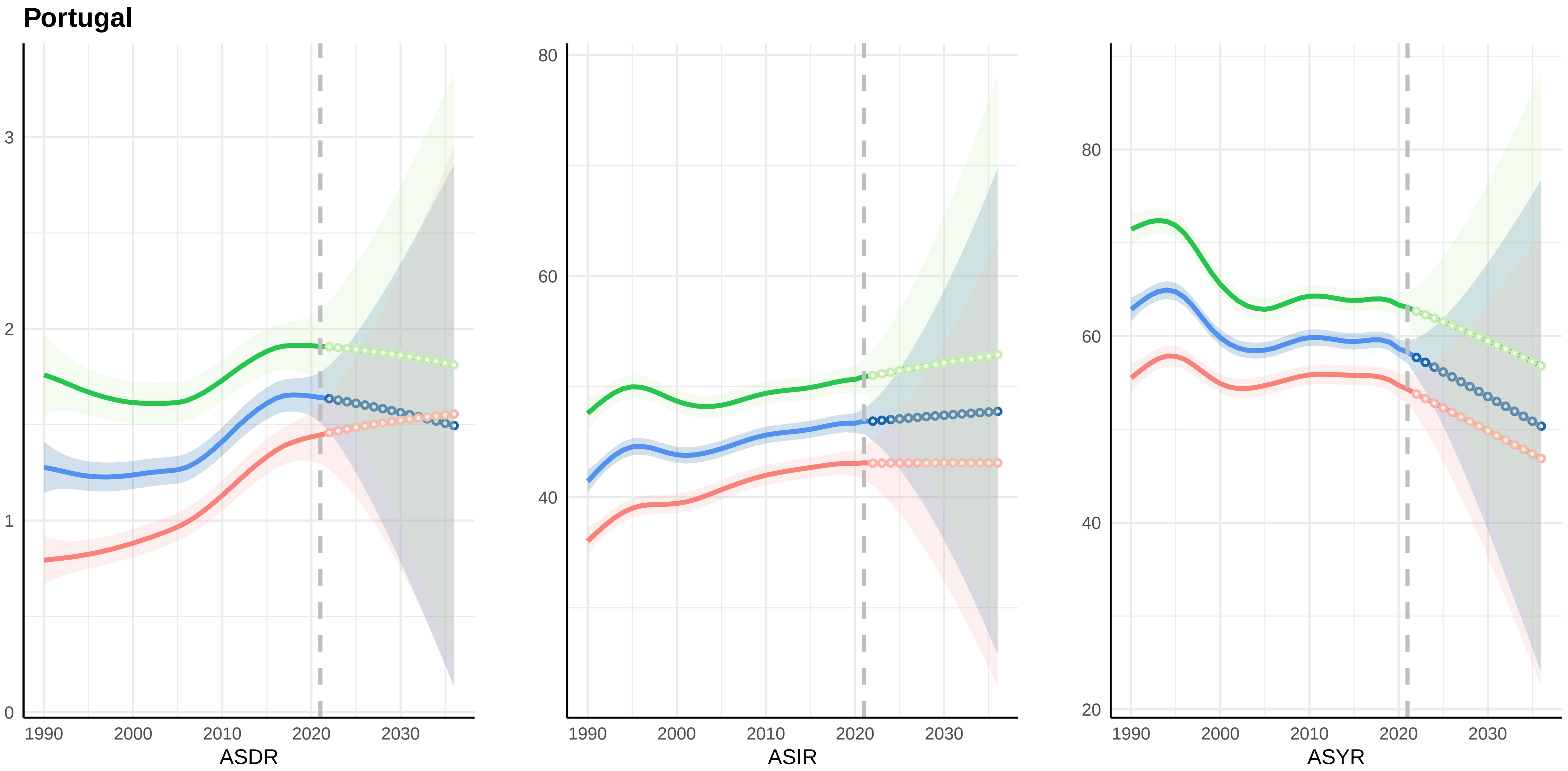

Portugal
3
2
1
0
1990
2000
2010
2020
2030
ASDR
80
60
40
1990
2000
2010
2020
2030
ASIR
80
60
40
20
1990
2000
2010
2020
2030
ASYR

Puerto Rico

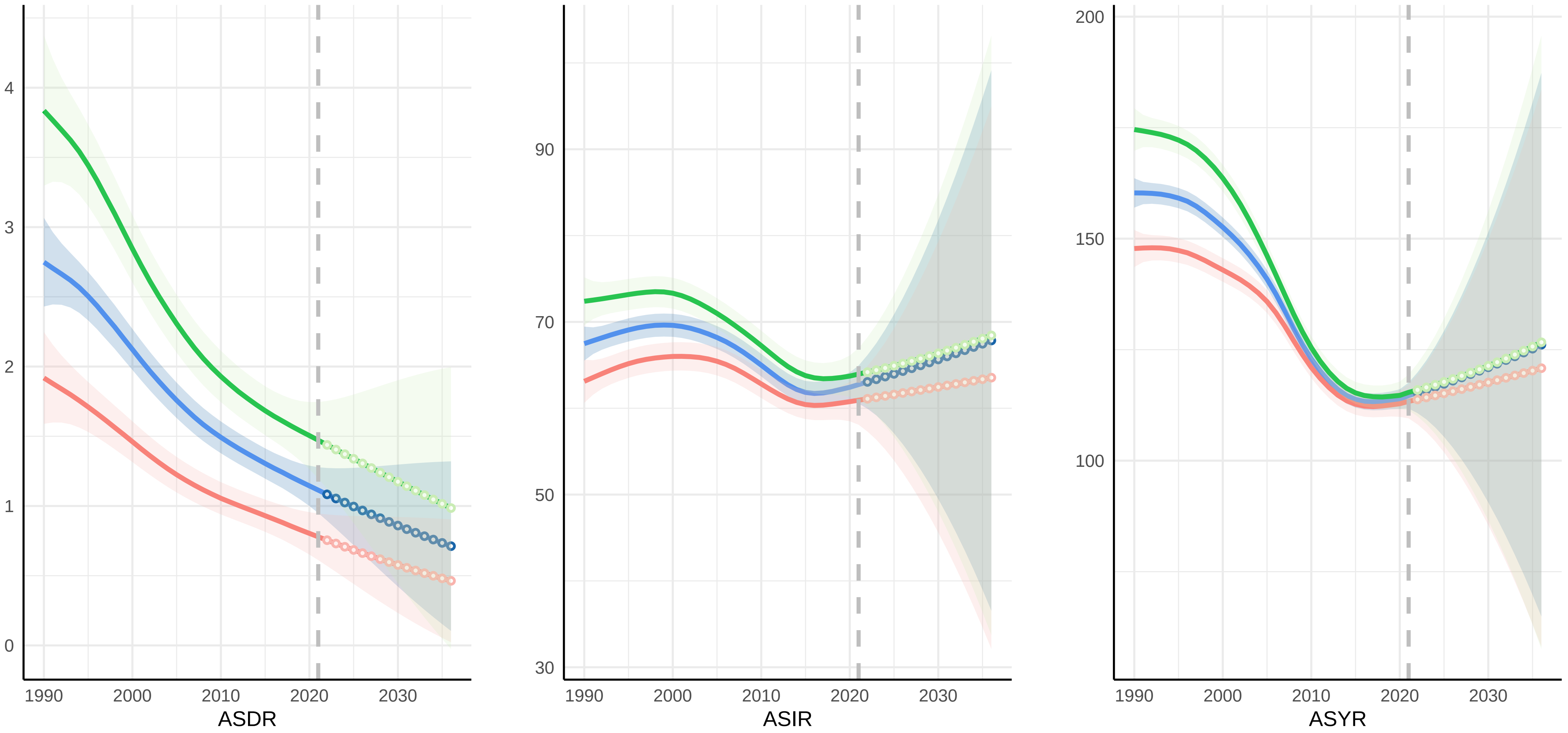

4
3
2
1
0
1990
2000
2010
2020
2030
ASDR
90
70
50
30
1990
2000
2010
2020
2030
ASIR
200
150
100
1990
2000
2010
2020
2030
ASYR

Republic of Korea

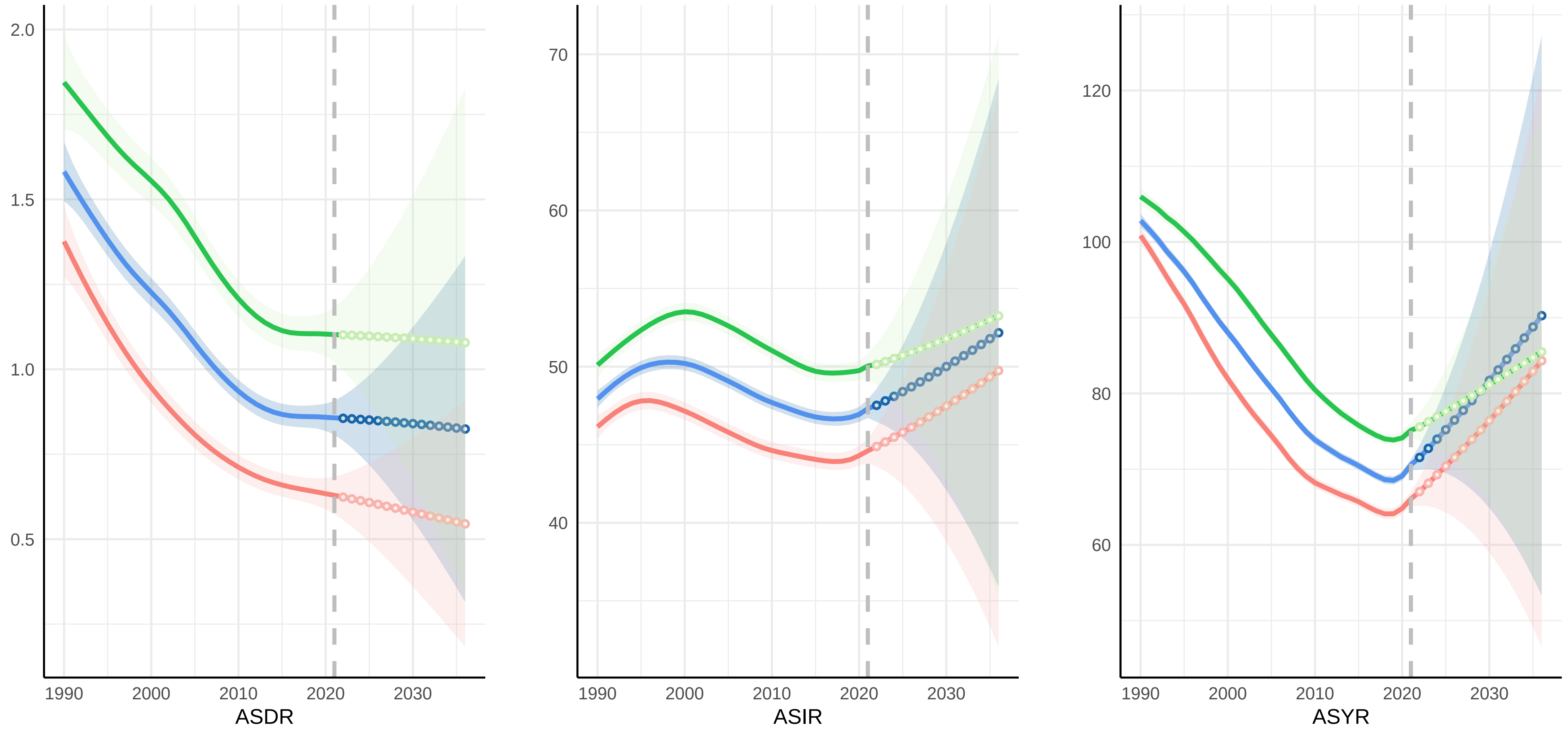

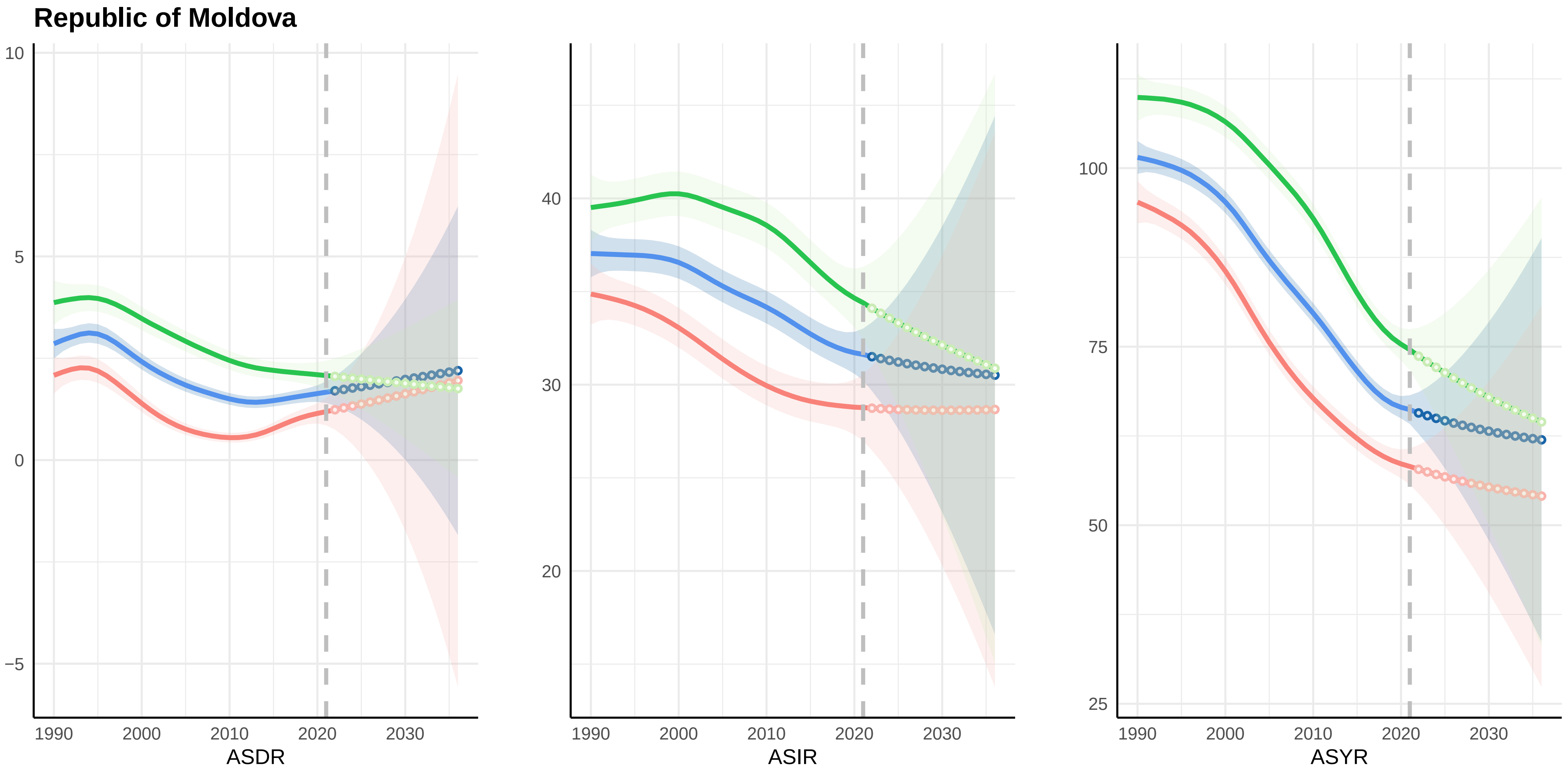
Republic of Moldova
10
5
0
−5
1990
2000
2010
2020
2030
ASDR
40
30
20
ASIR
100
75
50
25
ASYR

Romania

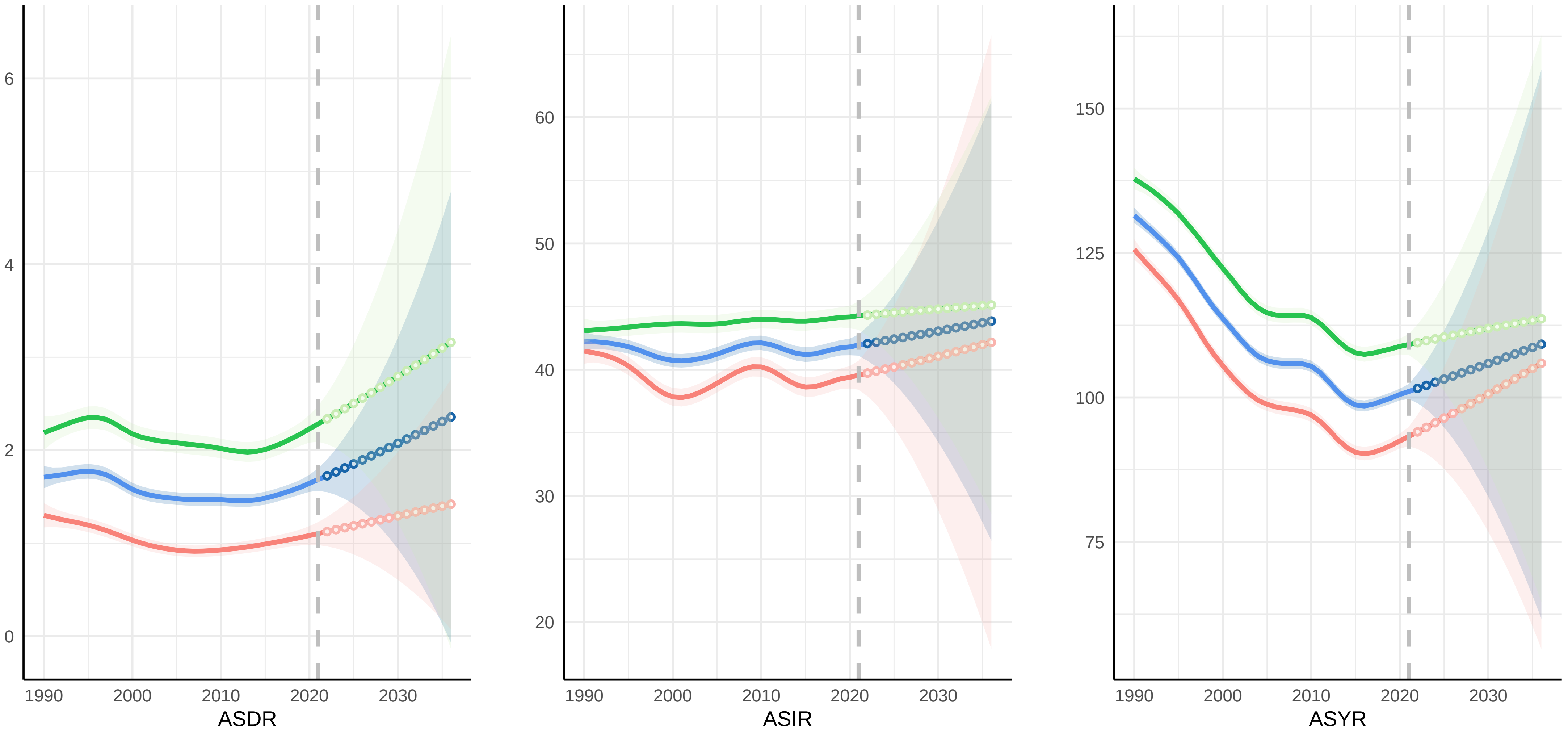

0
2
4
6
1990
2000
2010
2020
2030
ASDR
20
30
40
50
60
ASIR
75
100
125
150
ASYR

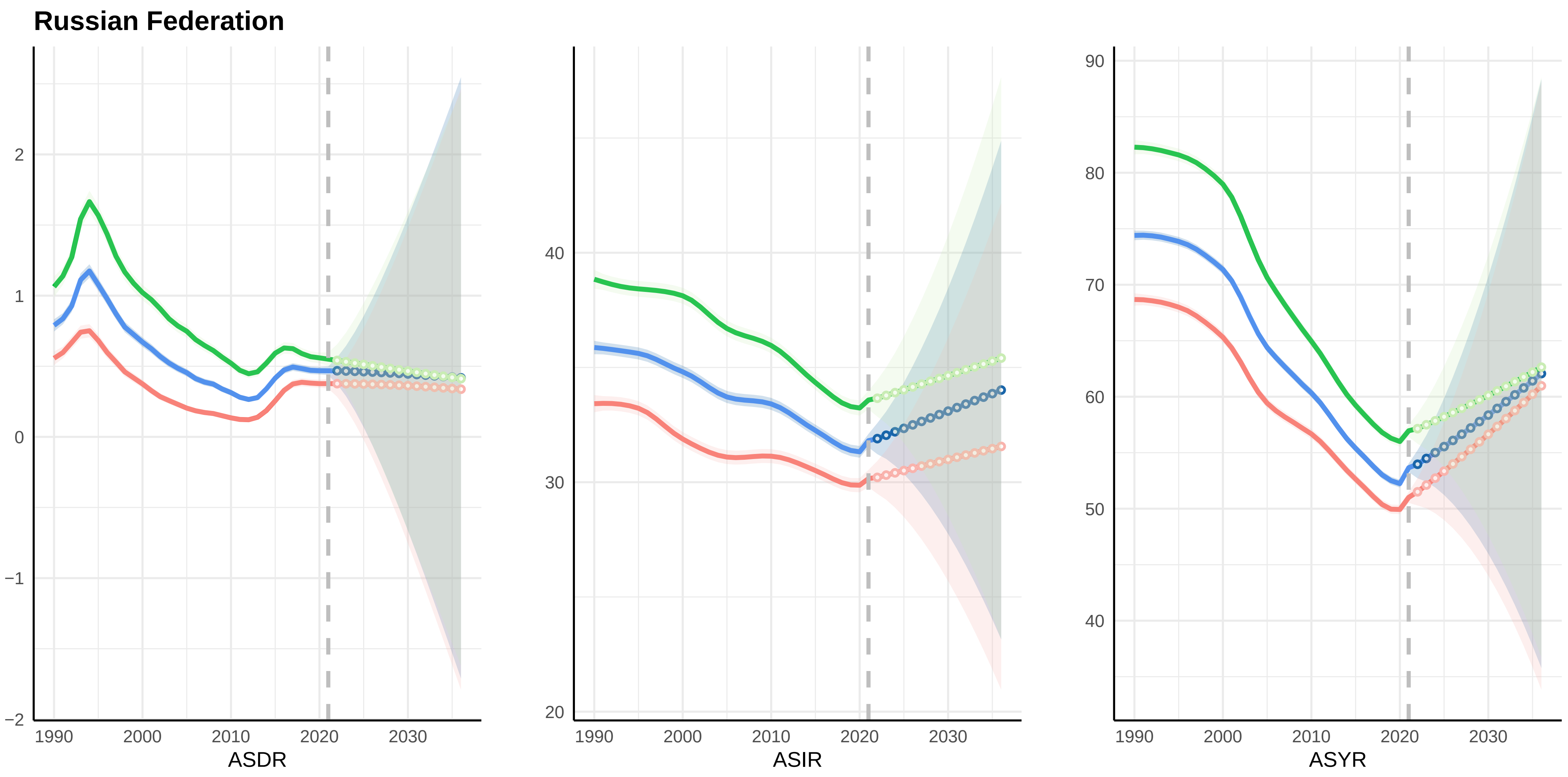

Russian Federation
2
1
0
−1
−2
1990
2000
2010
2020
2030
ASDR
40
30
20
1990
2000
2010
2020
2030
ASIR
90
80
70
60
50
40
1990
2000
2010
2020
2030
ASYR

Rwanda

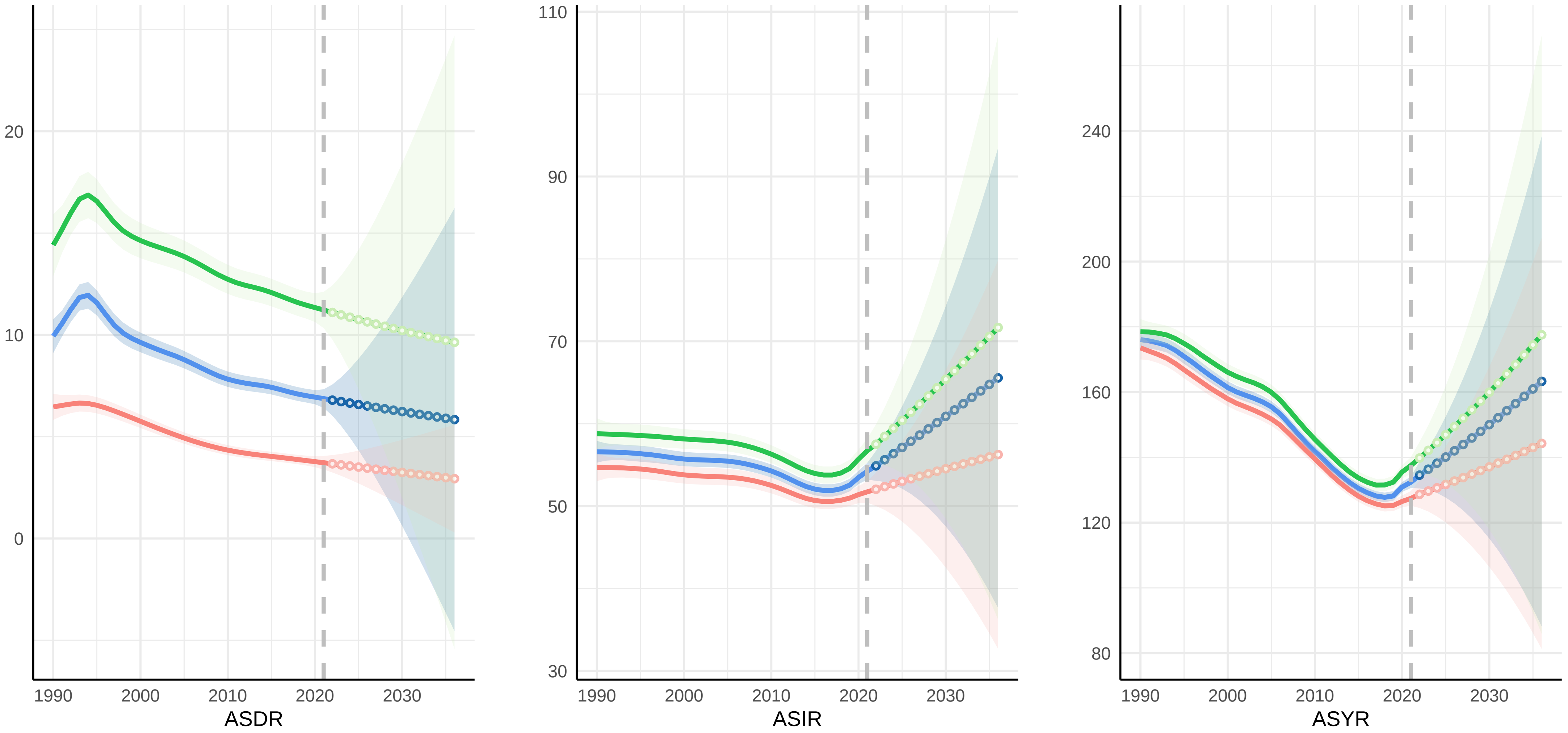

20
10
0
1990
2000
2010
2020
2030
ASDR
110
90
70
50
30
ASIR
240
200
160
120
80
ASYR

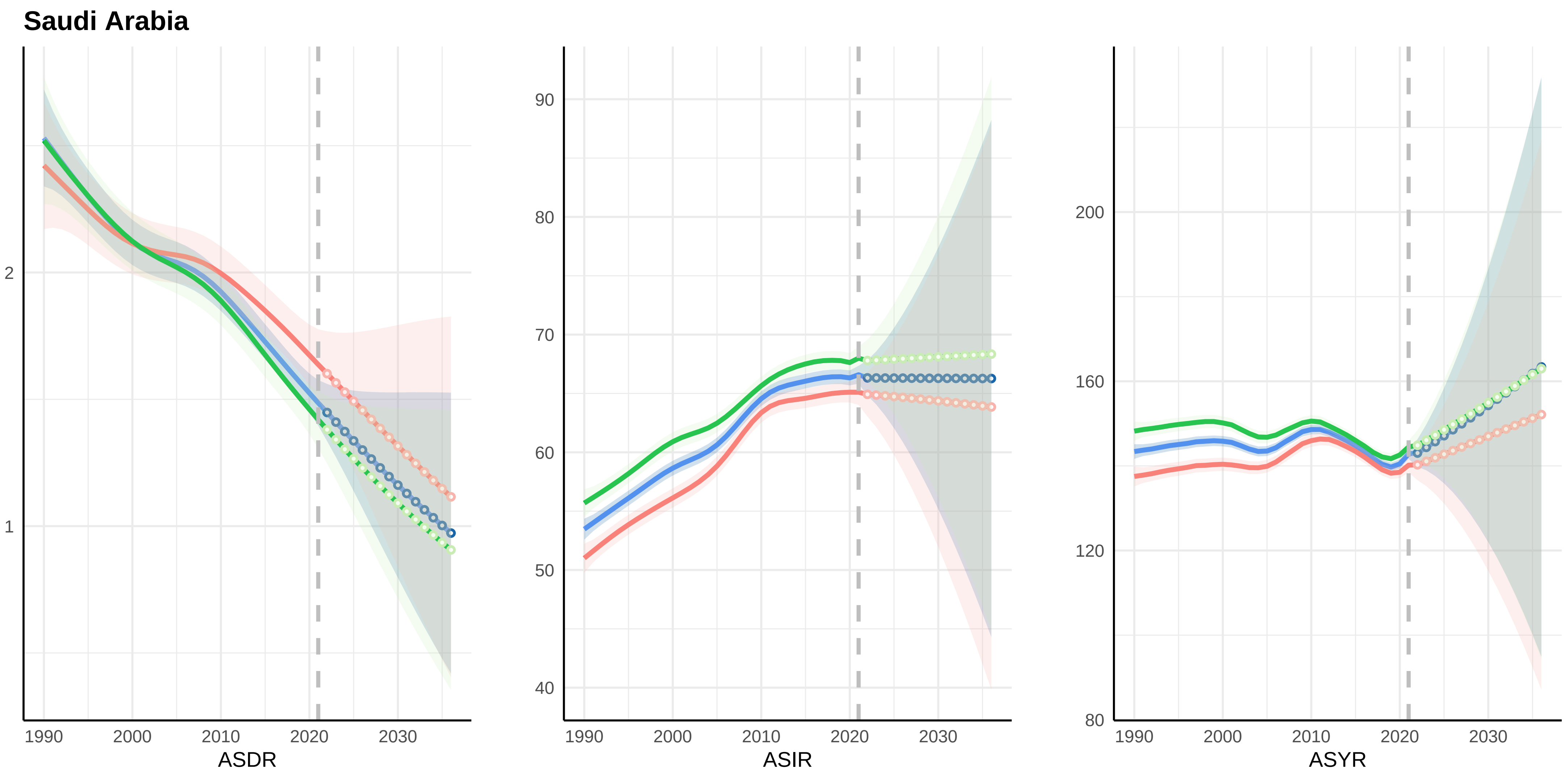

Saudi Arabia
2
1
1990
2000
2010
2020
2030
ASDR
90
80
70
60
50
40
1990
2000
2010
2020
2030
ASIR
200
160
120
80
1990
2000
2010
2020
2030
ASYR

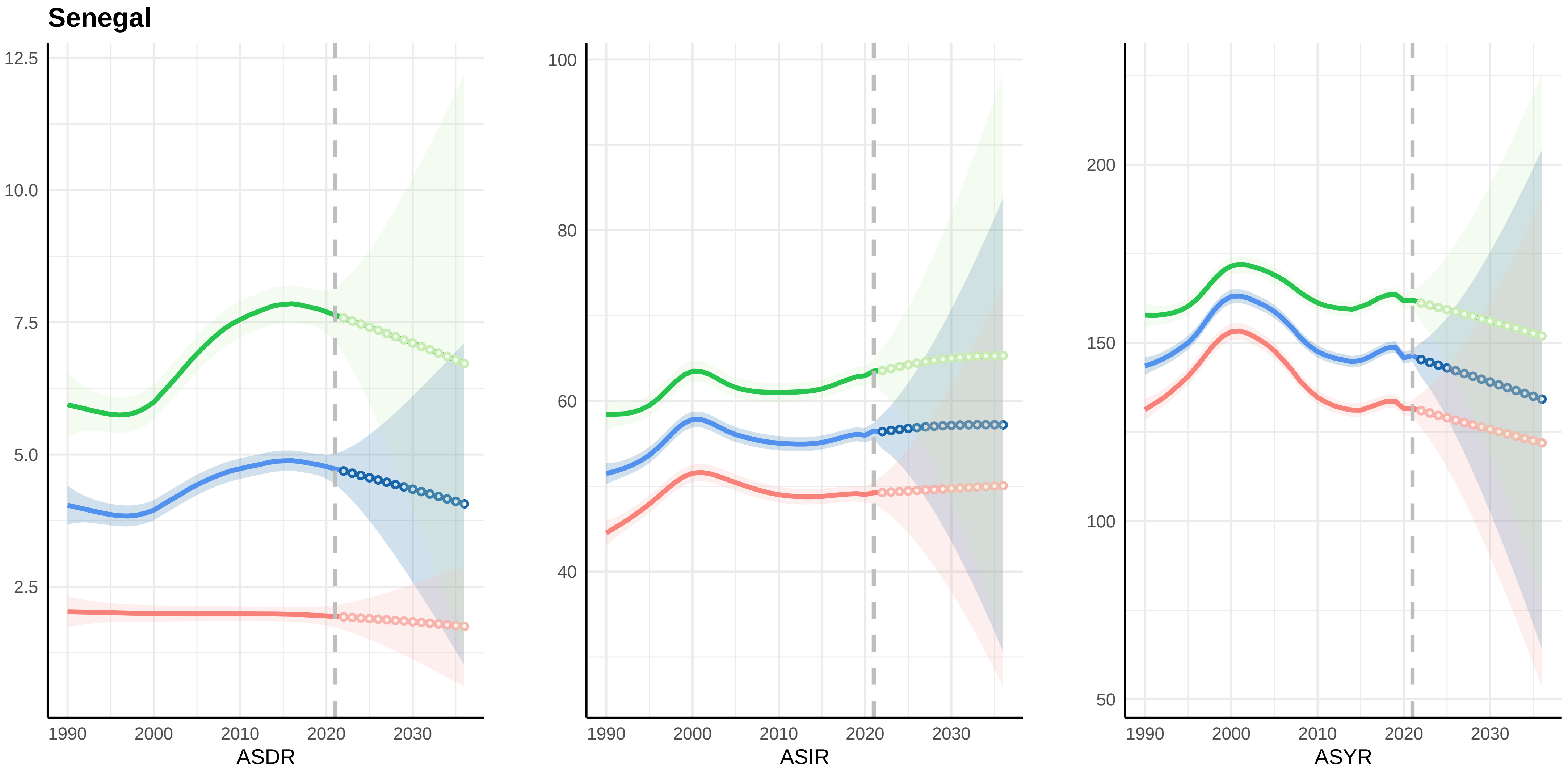
Senegal
12.5
10.0
7.5
5.0
2.5
1990
2000
2010
2020
2030
ASDR
100
80
60
40
1990
2000
2010
2020
2030
ASIR
200
150
100
50
1990
2000
2010
2020
2030
ASYR

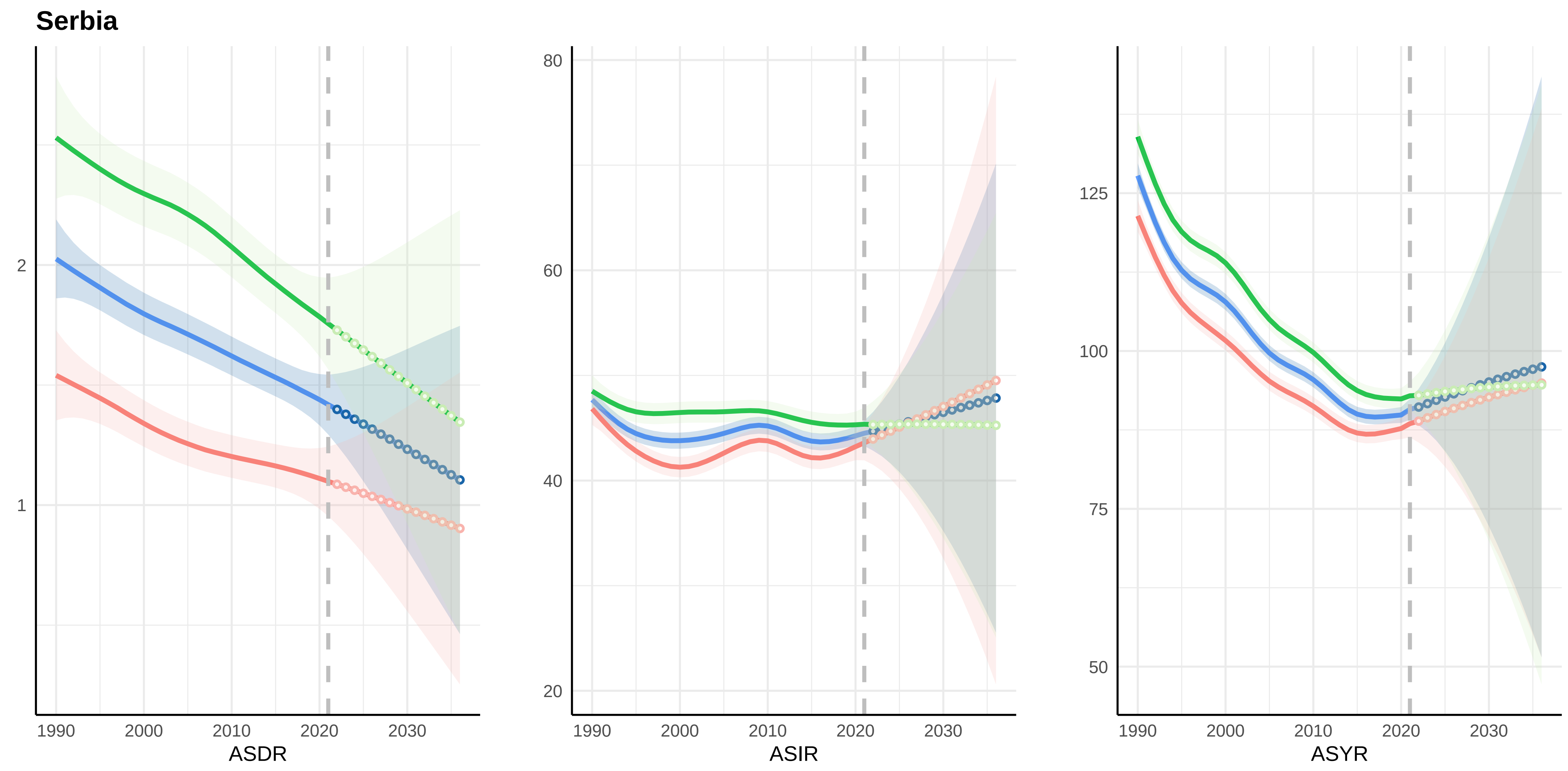
Serbia
2
1
1990
2000
2010
2020
2030
ASDR
80
60
40
20
ASIR
125
100
75
50
ASYR

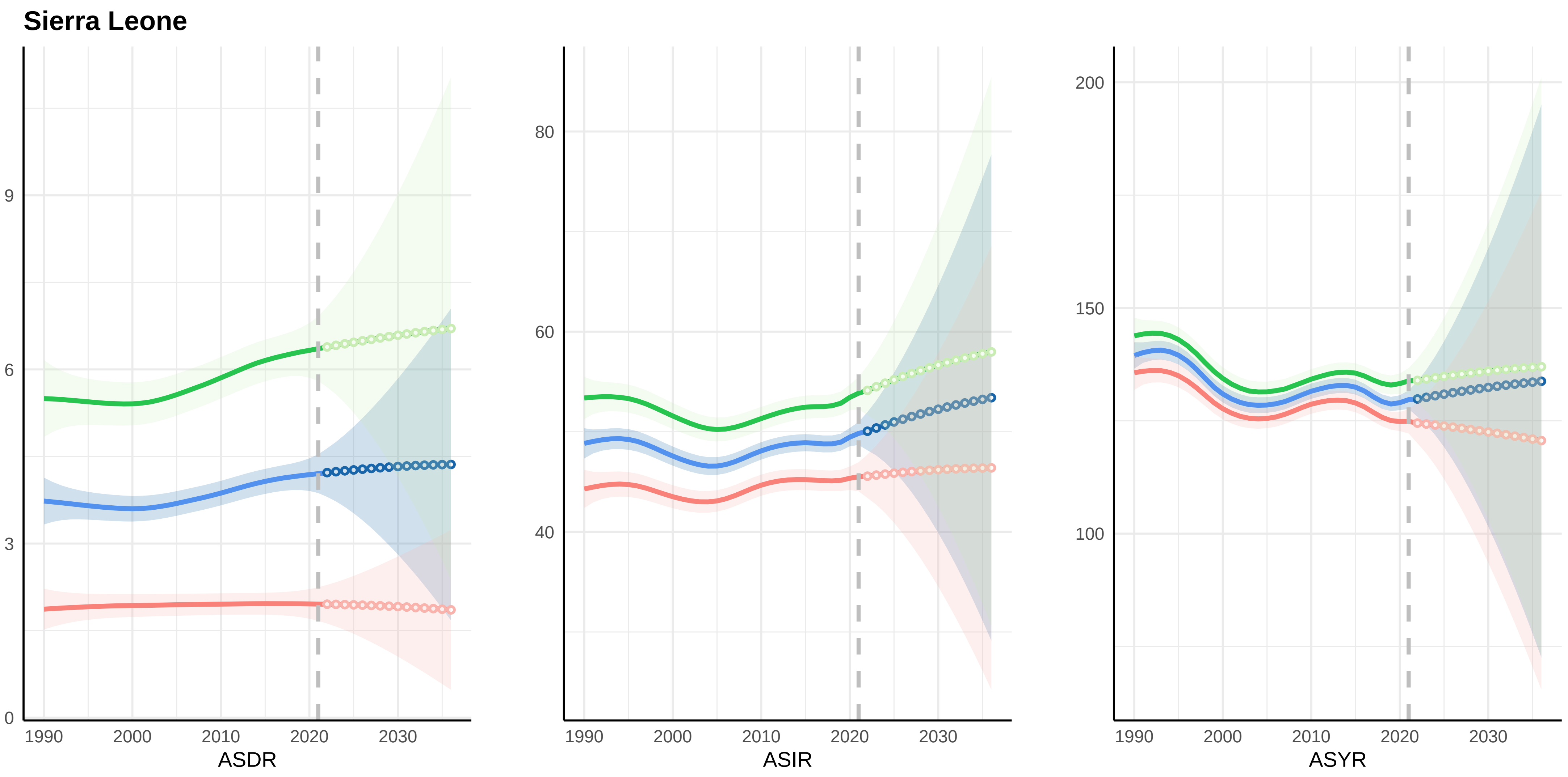

Sierra Leone
9
6
3
0
1990
2000
2010
2020
2030
ASDR
80
60
40
1990
2000
2010
2020
2030
ASIR
200
150
100
1990
2000
2010
2020
2030
ASYR

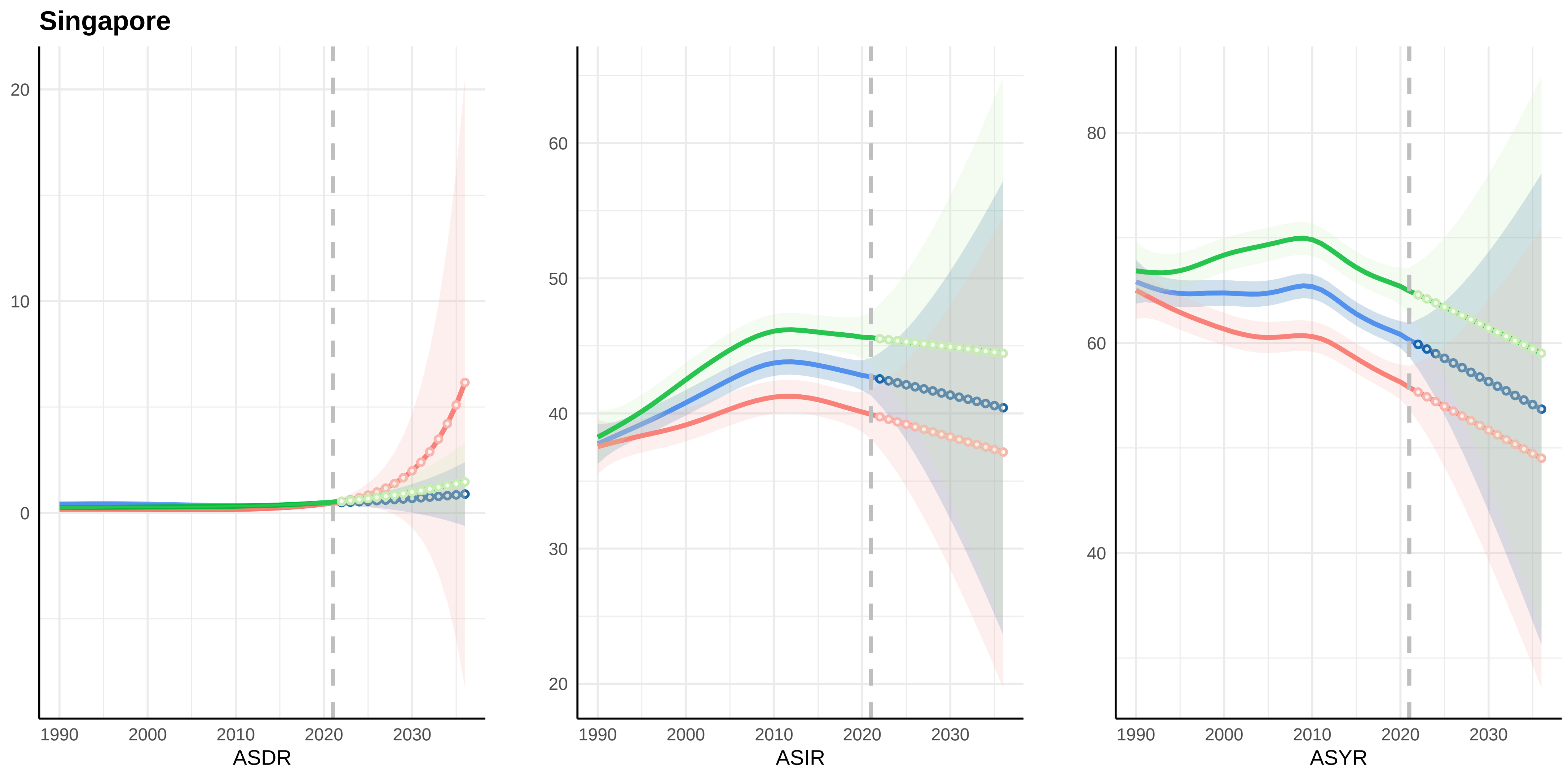

Singapore
20
10
0
1990
2000
2010
2020
2030
ASDR
60
50
40
30
20
ASIR
80
60
40
ASYR

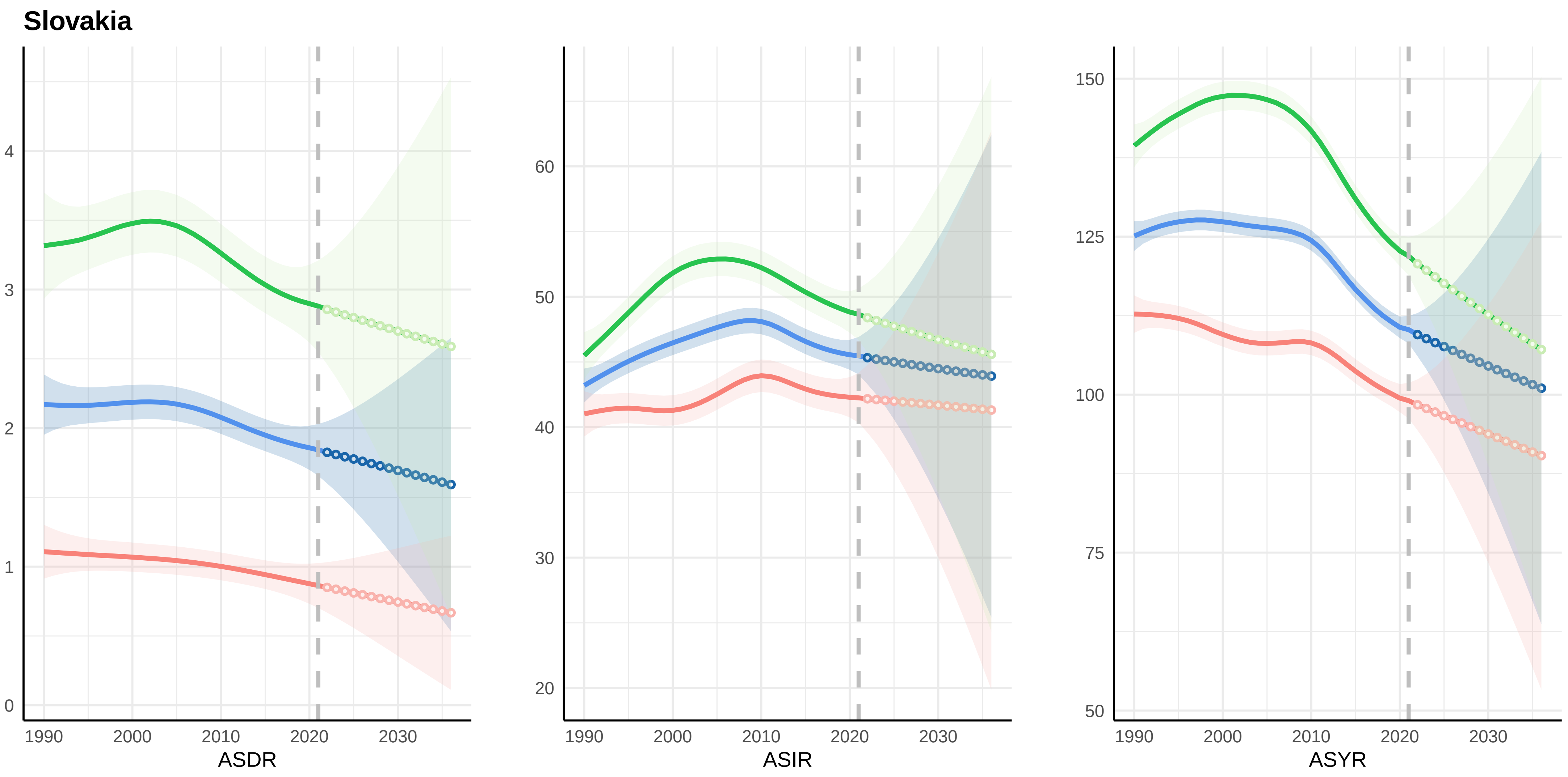

Slovakia
4
3
2
1
0
1990
2000
2010
2020
2030
ASDR
60
50
40
30
20
1990
2000
2010
2020
2030
ASIR
150
125
100
75
50
1990
2000
2010
2020
2030
ASYR

Slovenia

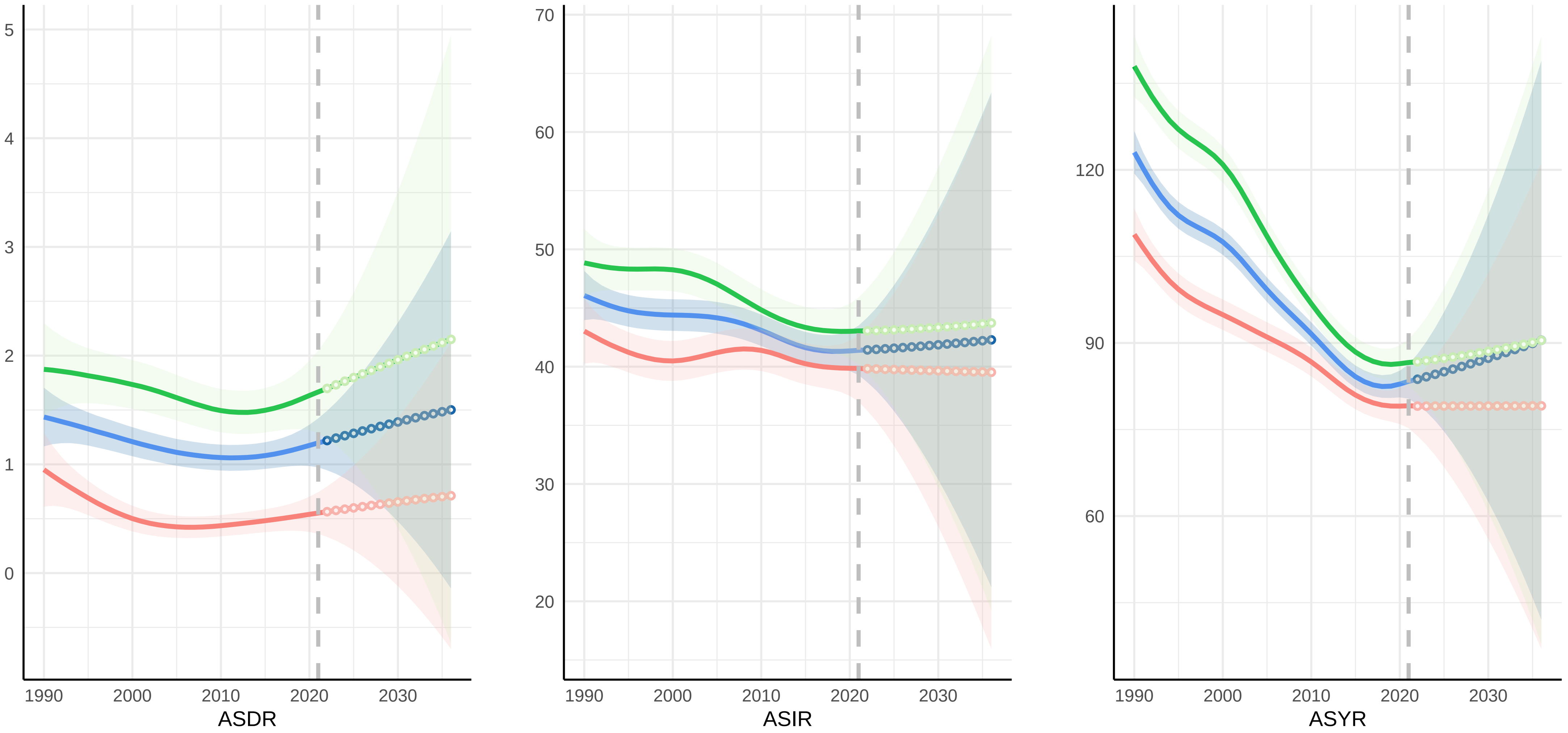

5
4
3
2
1
0
1990
2000
2010
2020
2030
ASDR
70
60
50
40
30
20
1990
2000
2010
2020
2030
ASIR
120
90
60
1990
2000
2010
2020
2030
ASYR

Somalia

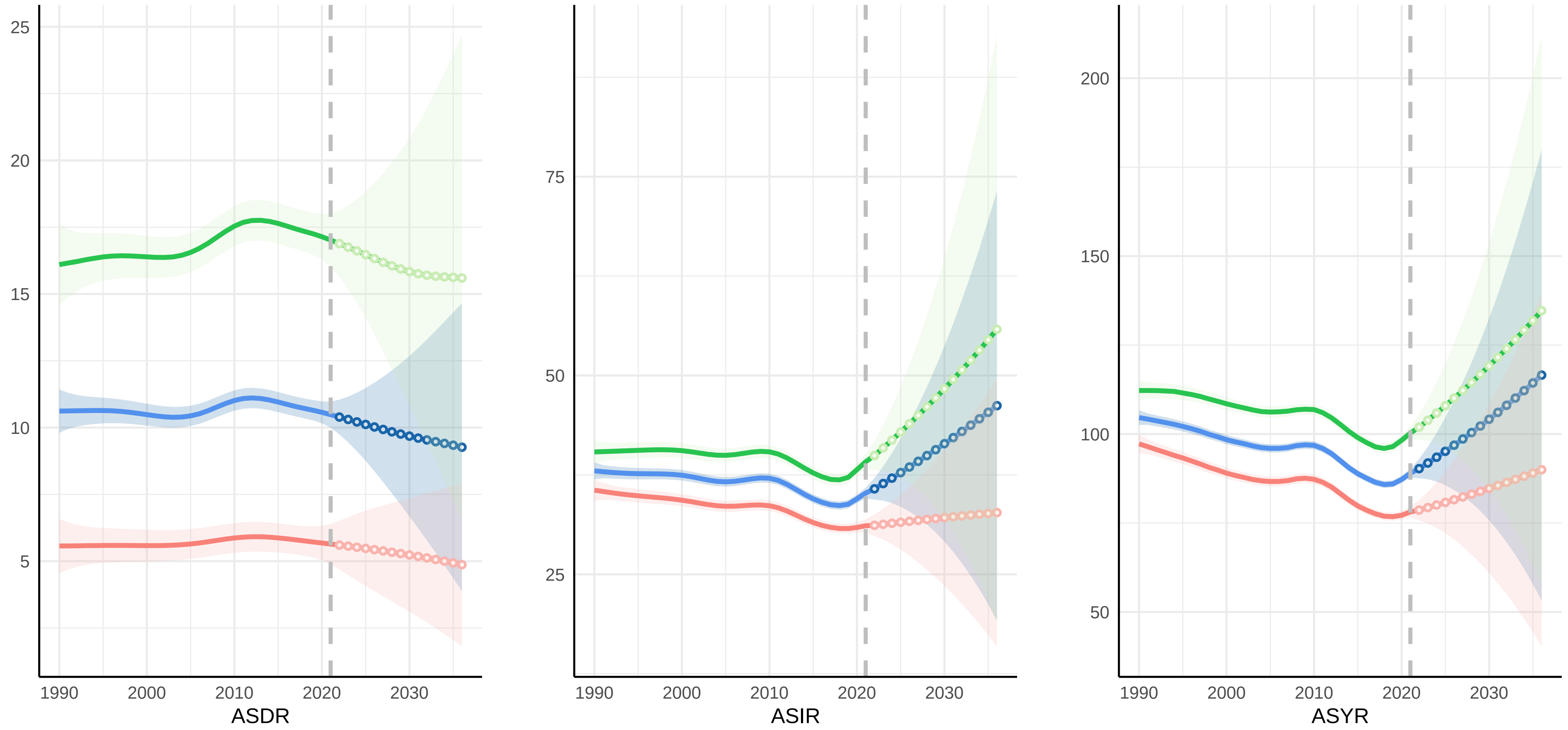

25
20
15
10
5
1990
2000
2010
2020
2030
ASDR
75
50
25
1990
2000
2010
2020
2030
ASIR
200
150
100
50
1990
2000
2010
2020
2030
ASYR

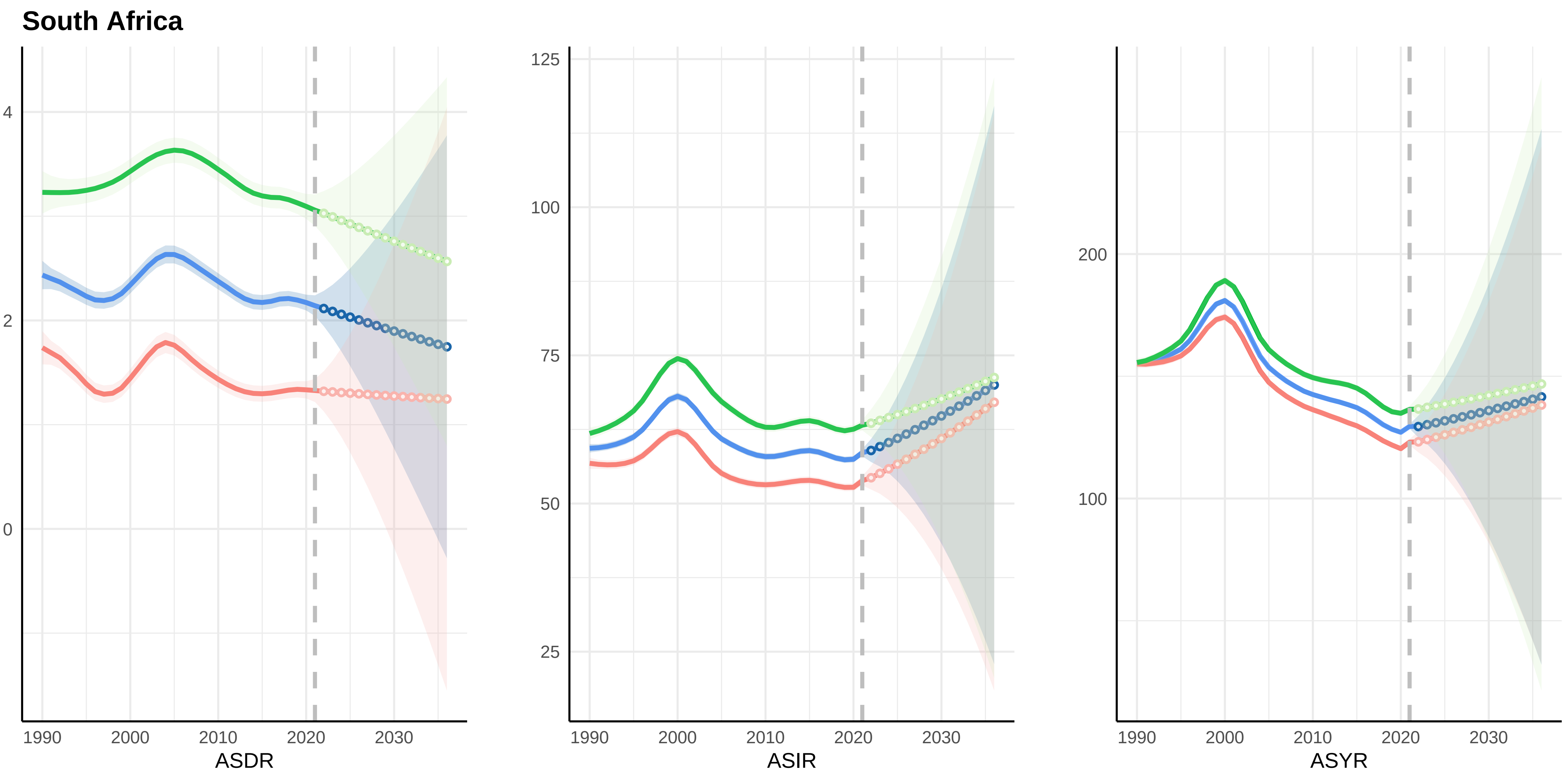

South Africa
4
2
0
1990
2000
2010
2020
2030
ASDR
125
100
75
50
25
1990
2000
2010
2020
2030
ASIR
200
100
1990
2000
2010
2020
2030
ASYR

South Sudan

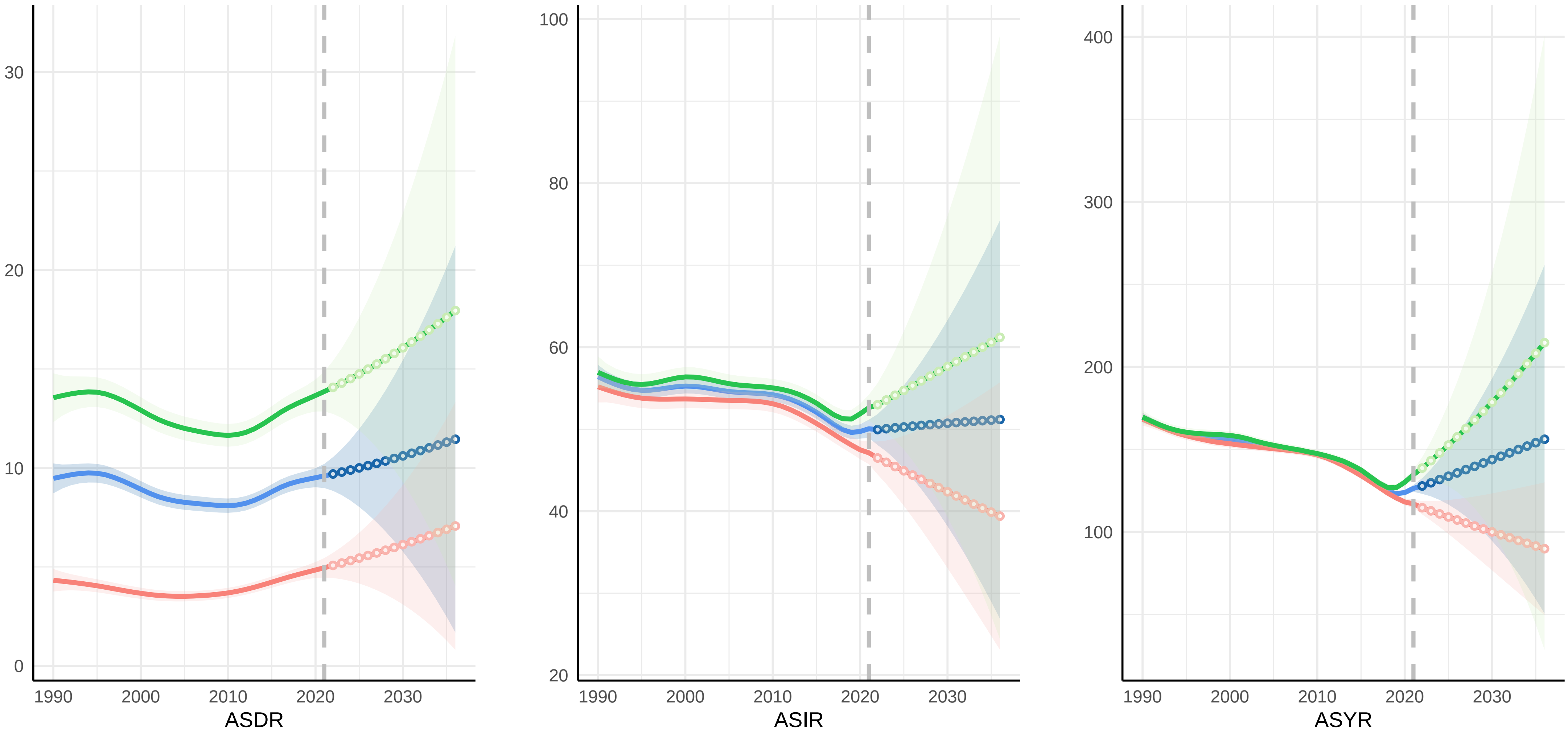

30
20
10
0
1990
2000
2010
2020
2030
ASDR
100
80
60
40
20
ASIR
400
300
200
100
ASYR

Spain

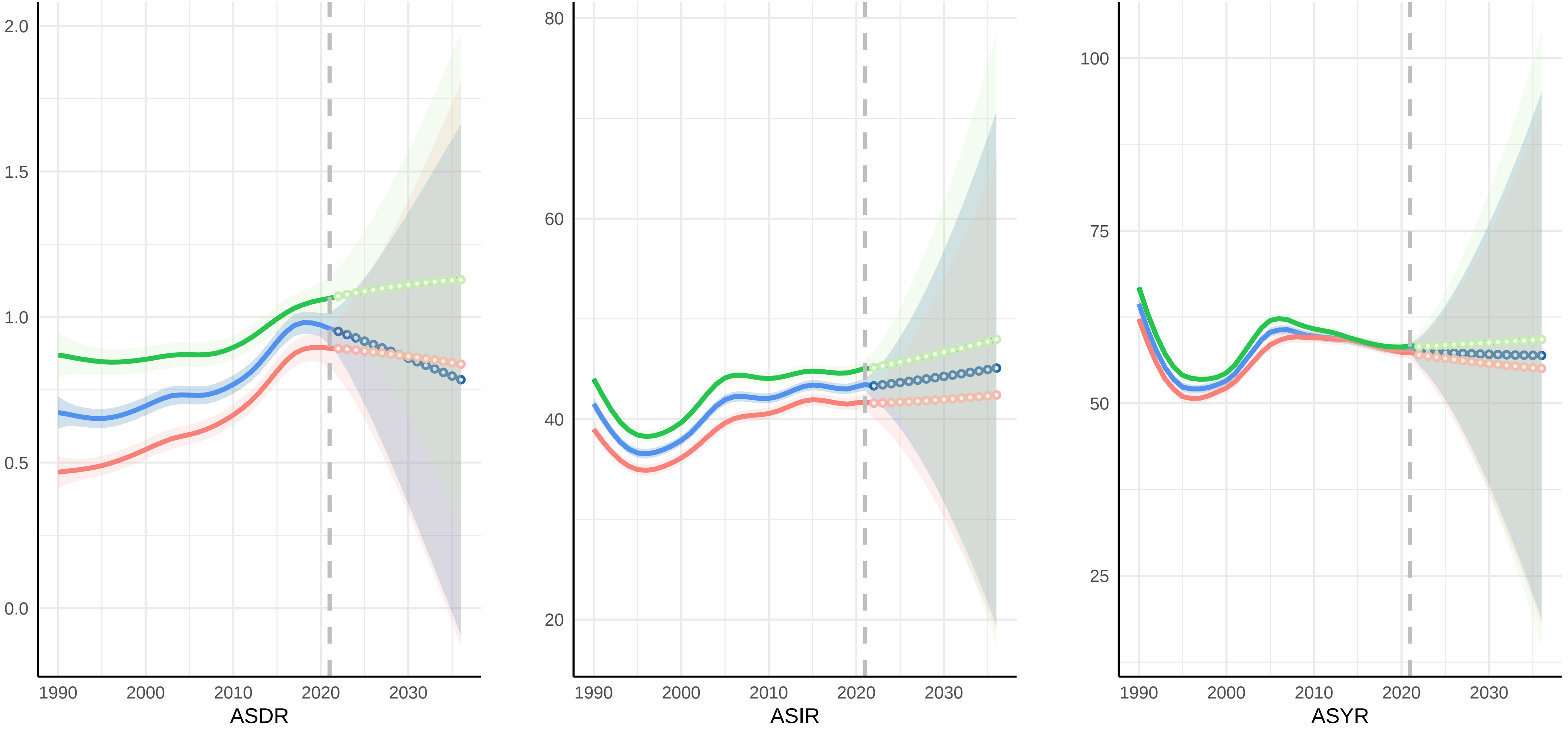

2.0
1.5
1.0
0.5
0.0
1990
2000
2010
2020
2030
ASDR
80
60
40
20
1990
2000
2010
2020
2030
ASIR
100
75
50
25
1990
2000
2010
2020
2030
ASYR

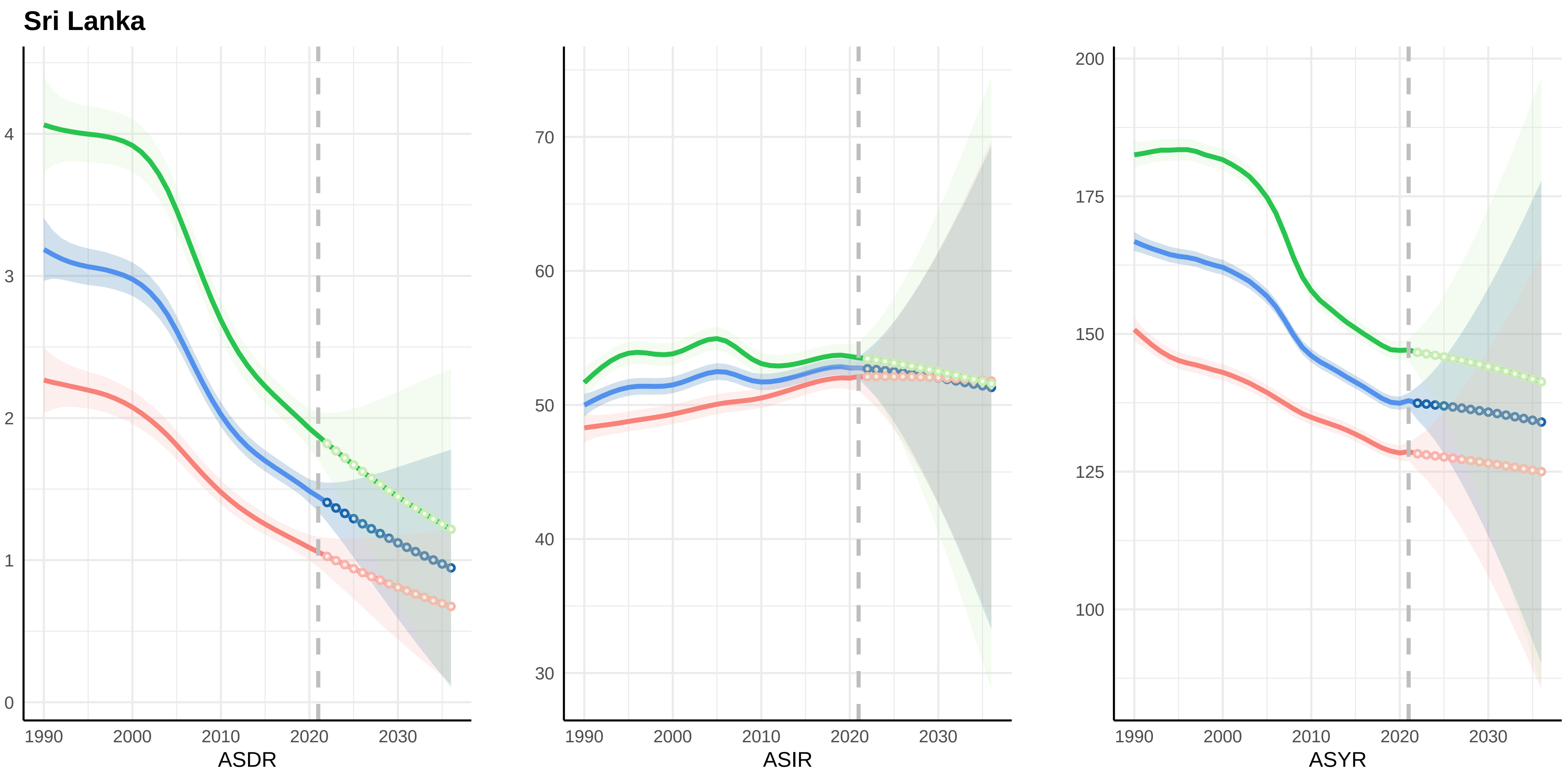

Sri Lanka
4
3
2
1
0
1990
2000
2010
2020
2030
ASDR
70
60
50
40
30
1990
2000
2010
2020
2030
ASIR
200
175
150
125
100
1990
2000
2010
2020
2030
ASYR

Sudan

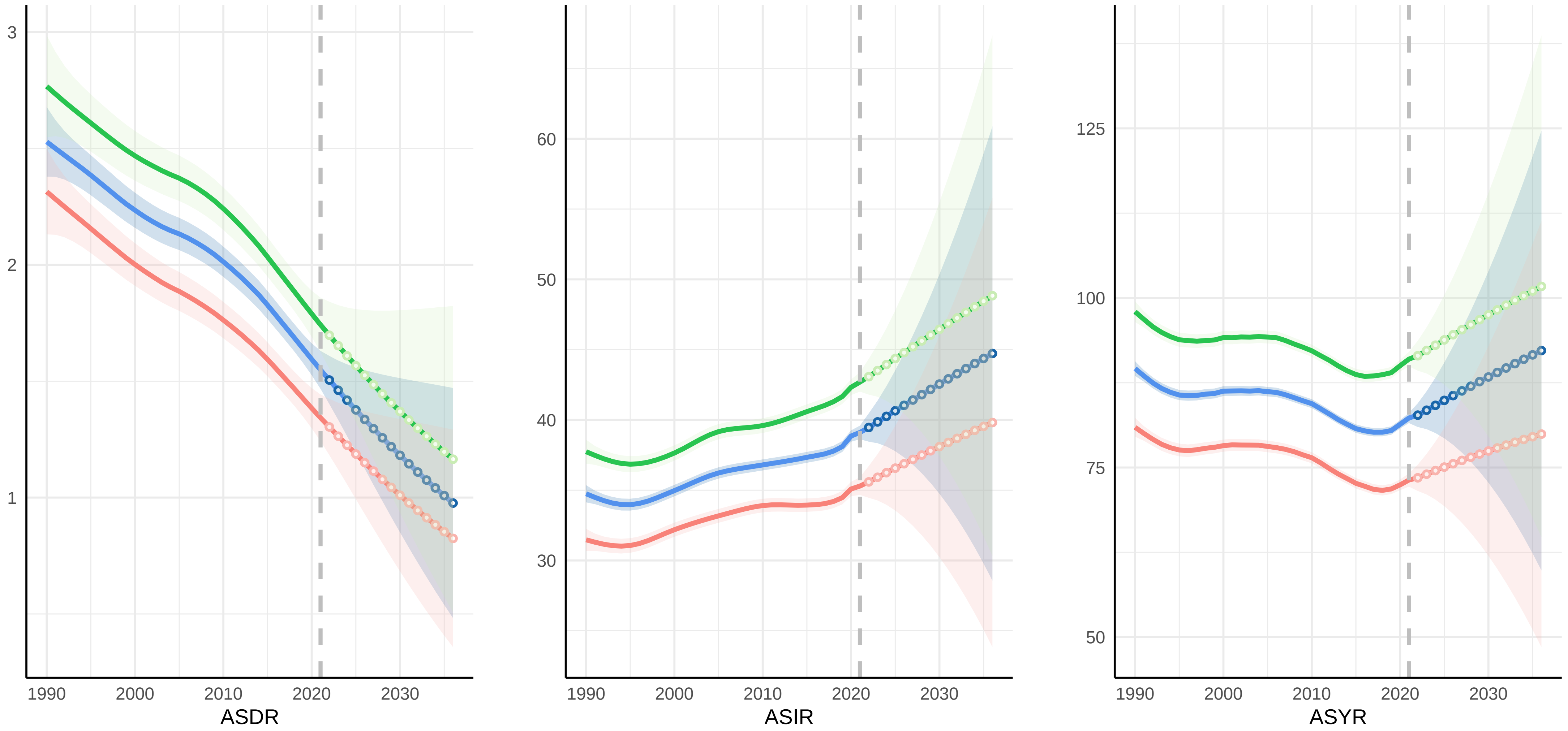

3
2
1
1990
2000
2010
2020
2030
ASDR
60
50
40
30
1990
2000
2010
2020
2030
ASIR
125
100
75
50
1990
2000
2010
2020
2030
ASYR

Suriname

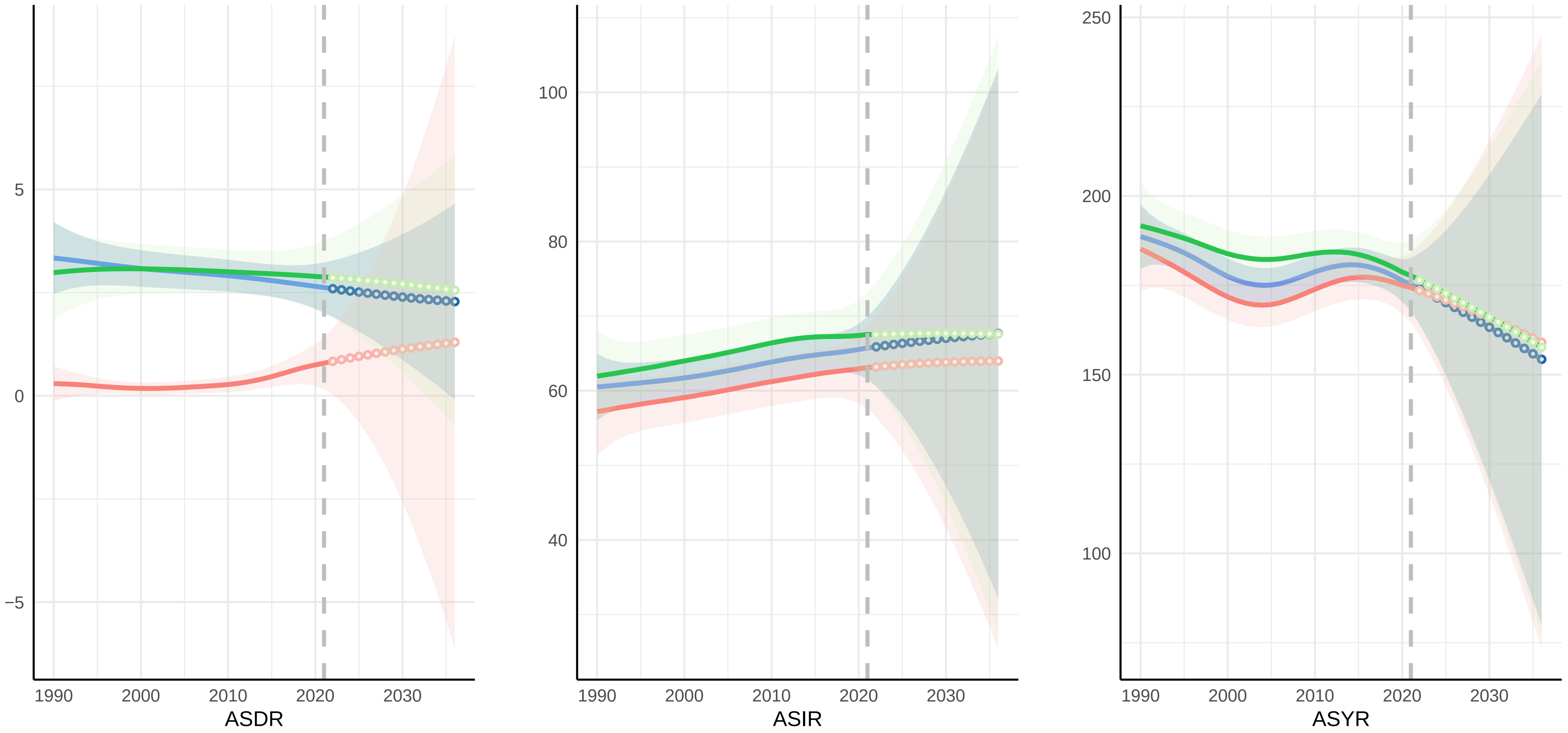
5
0
−5
1990
2000
2010
2020
2030
ASDR
100
80
60
40
ASIR
250
200
150
100
ASYR

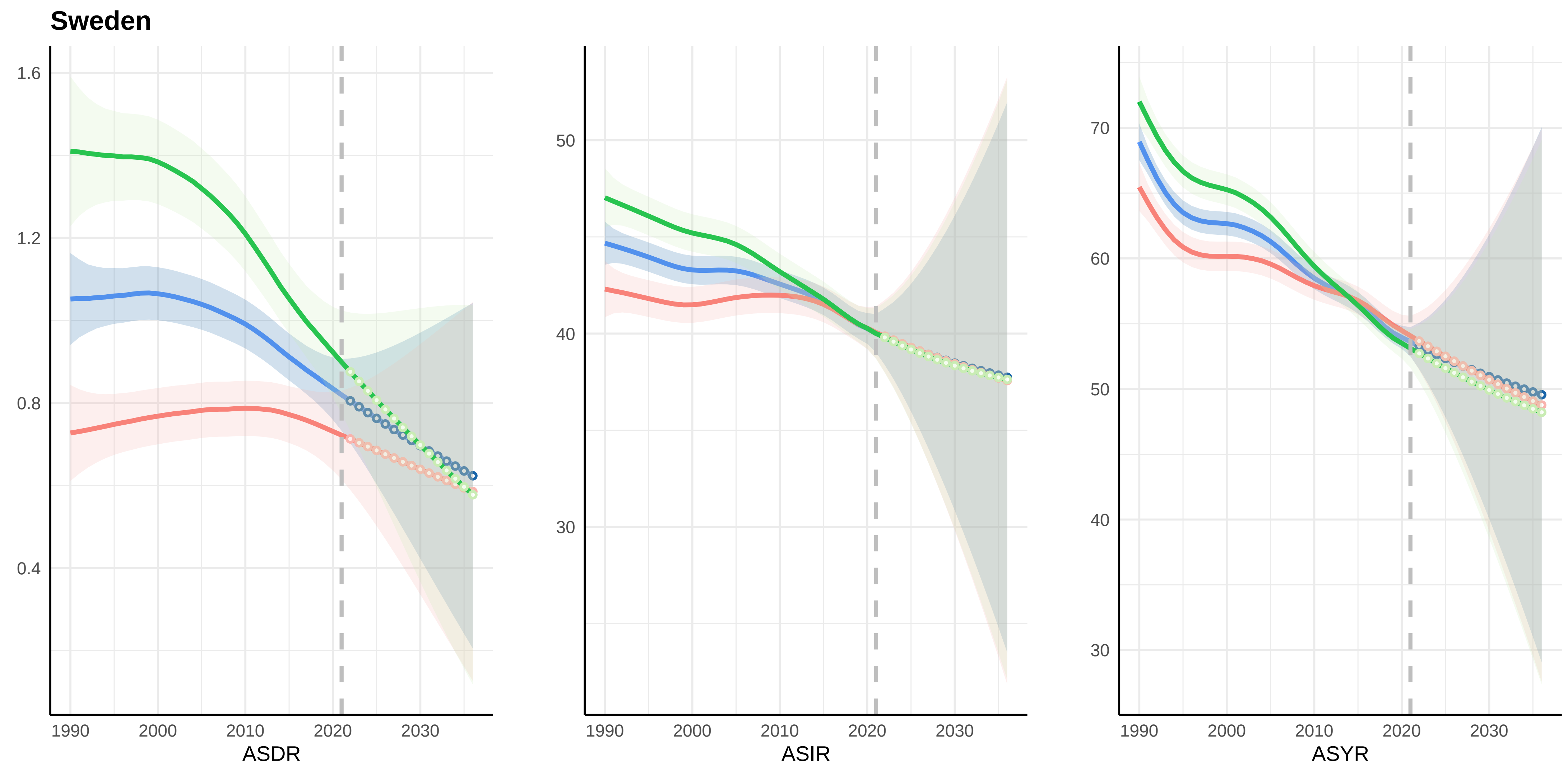
Sweden
1.6
1.2
0.8
0.4
1990
2000
2010
2020
2030
ASDR
50
40
30
1990
2000
2010
2020
2030
ASIR
70
60
50
40
30
1990
2000
2010
2020
2030
ASYR

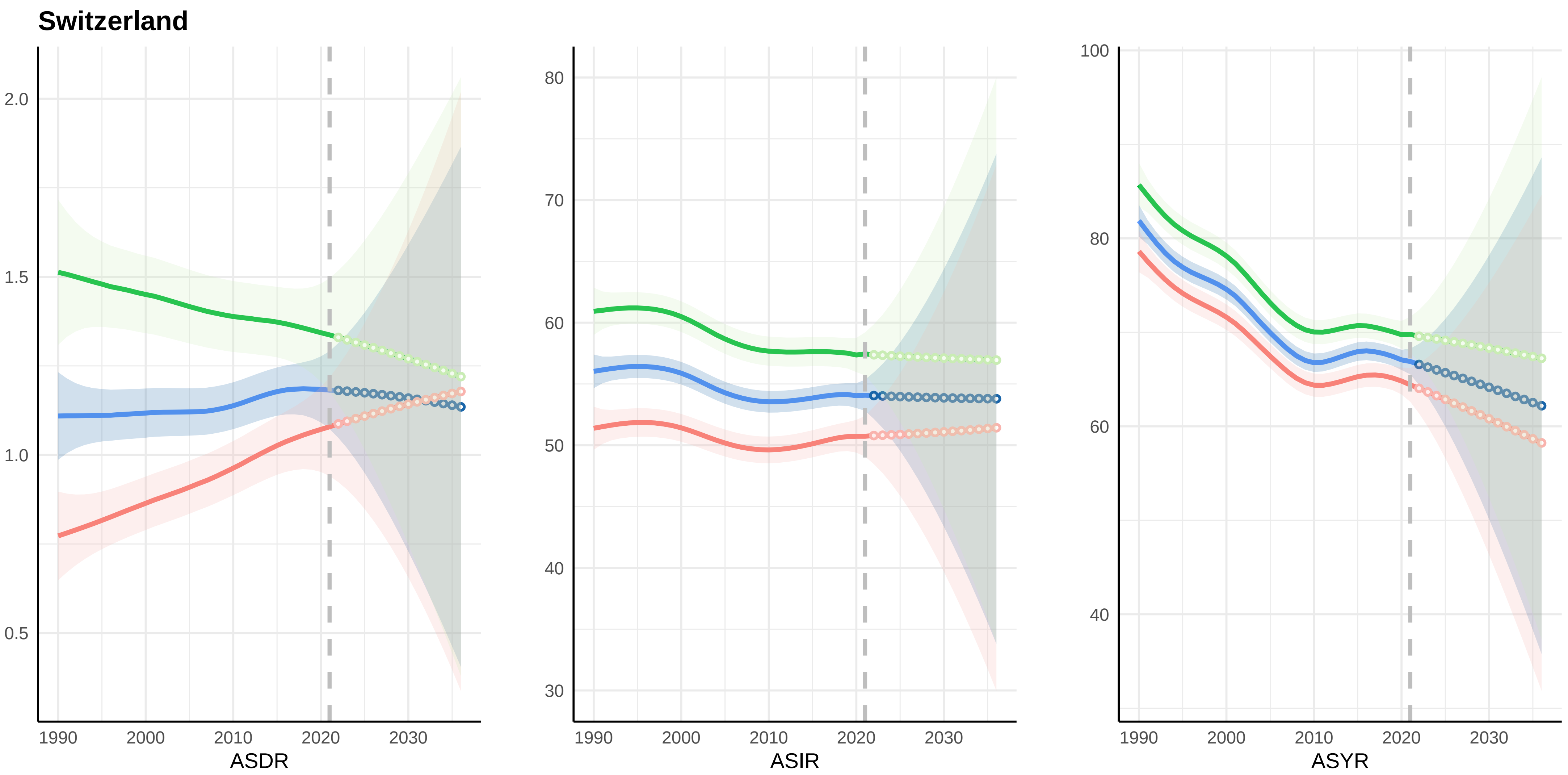

Switzerland
2.0
1.5
1.0
0.5
1990
2000
2010
2020
2030
ASDR
80
70
60
50
40
30
1990
2000
2010
2020
2030
ASIR
100
80
60
40
1990
2000
2010
2020
2030
ASYR

Syrian Arab Republic

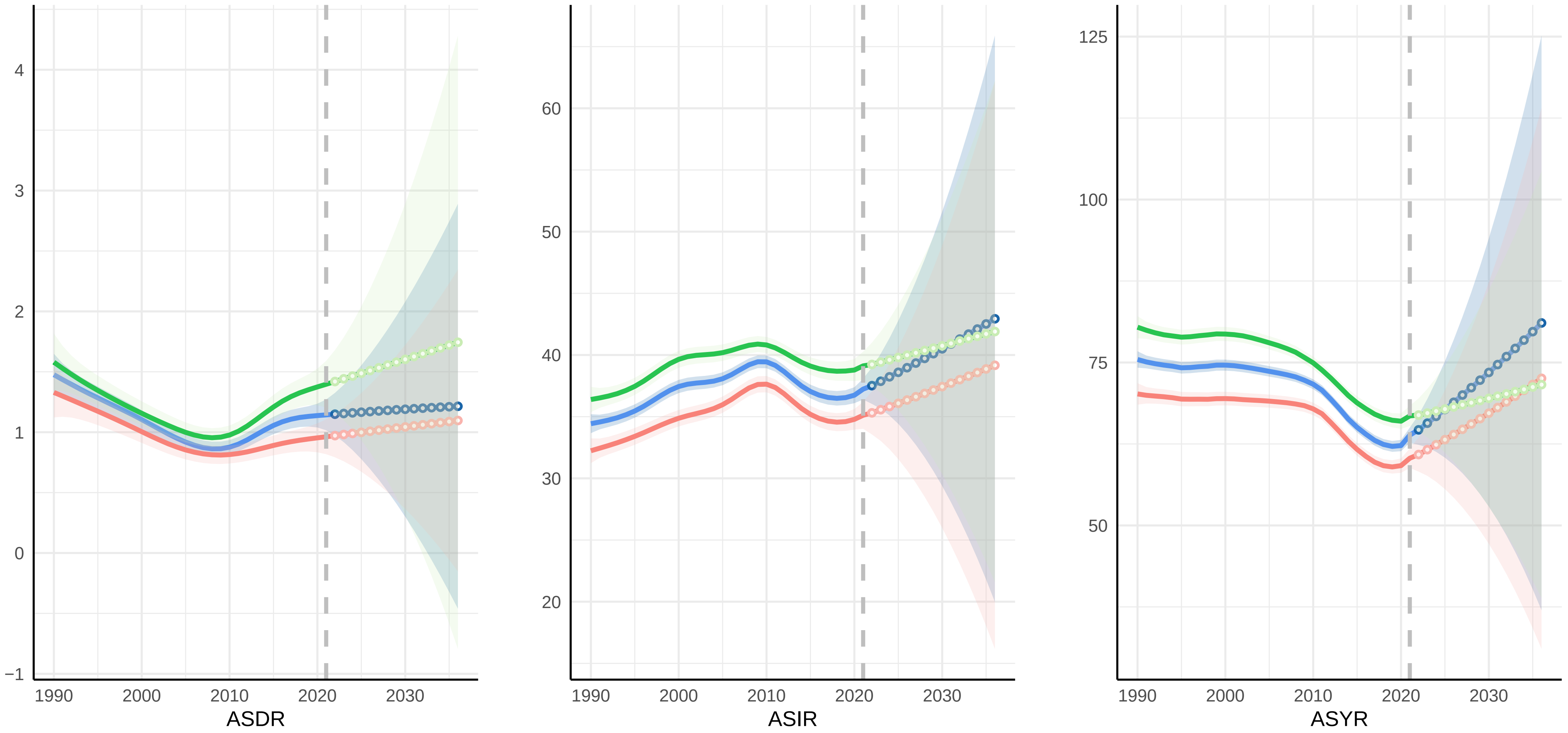

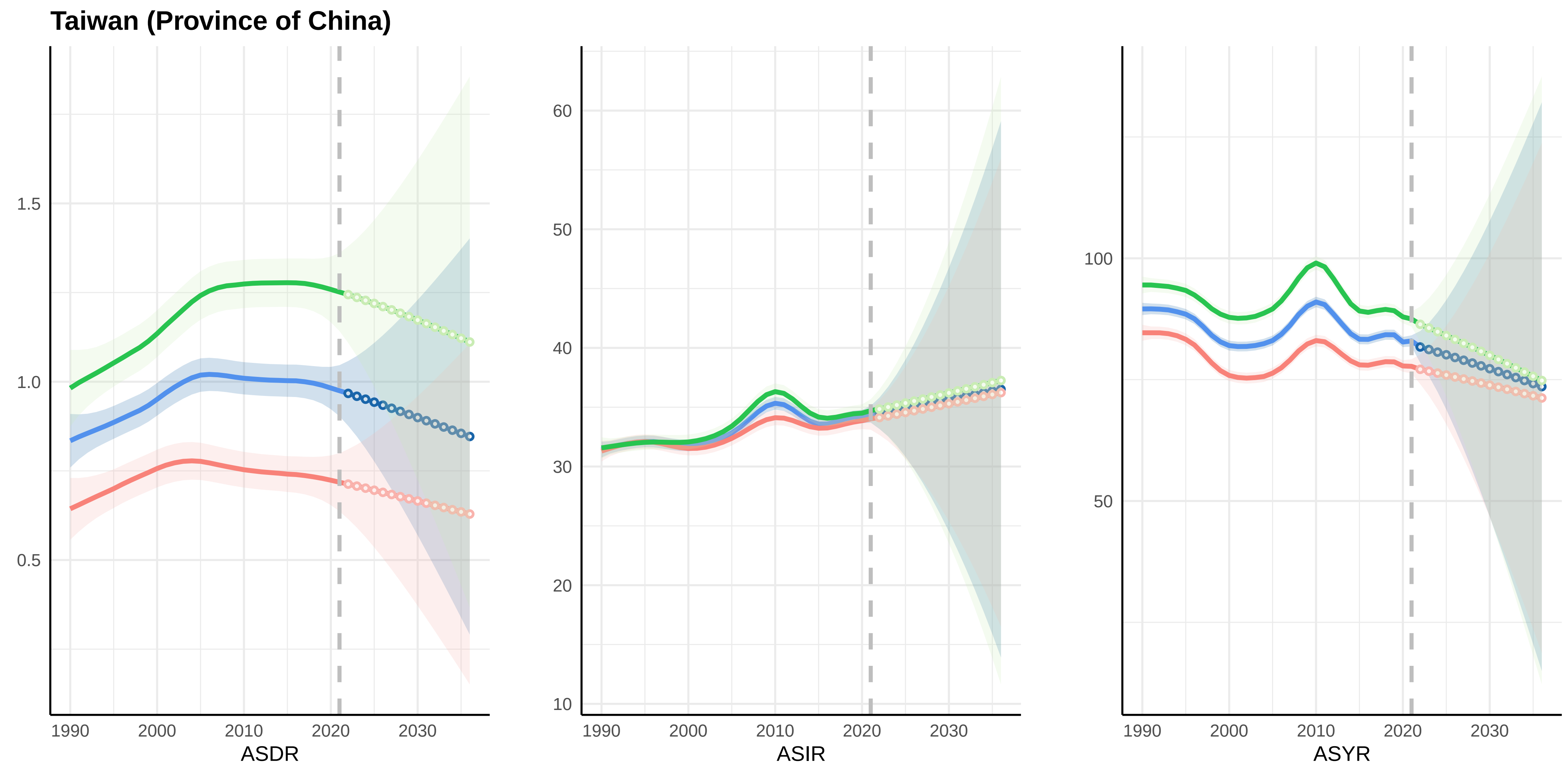

Taiwan (Province of China)
1.5
1.0
0.5
1990
2000
2010
2020
2030
ASDR
60
50
40
30
20
10
ASIR
100
50
ASYR

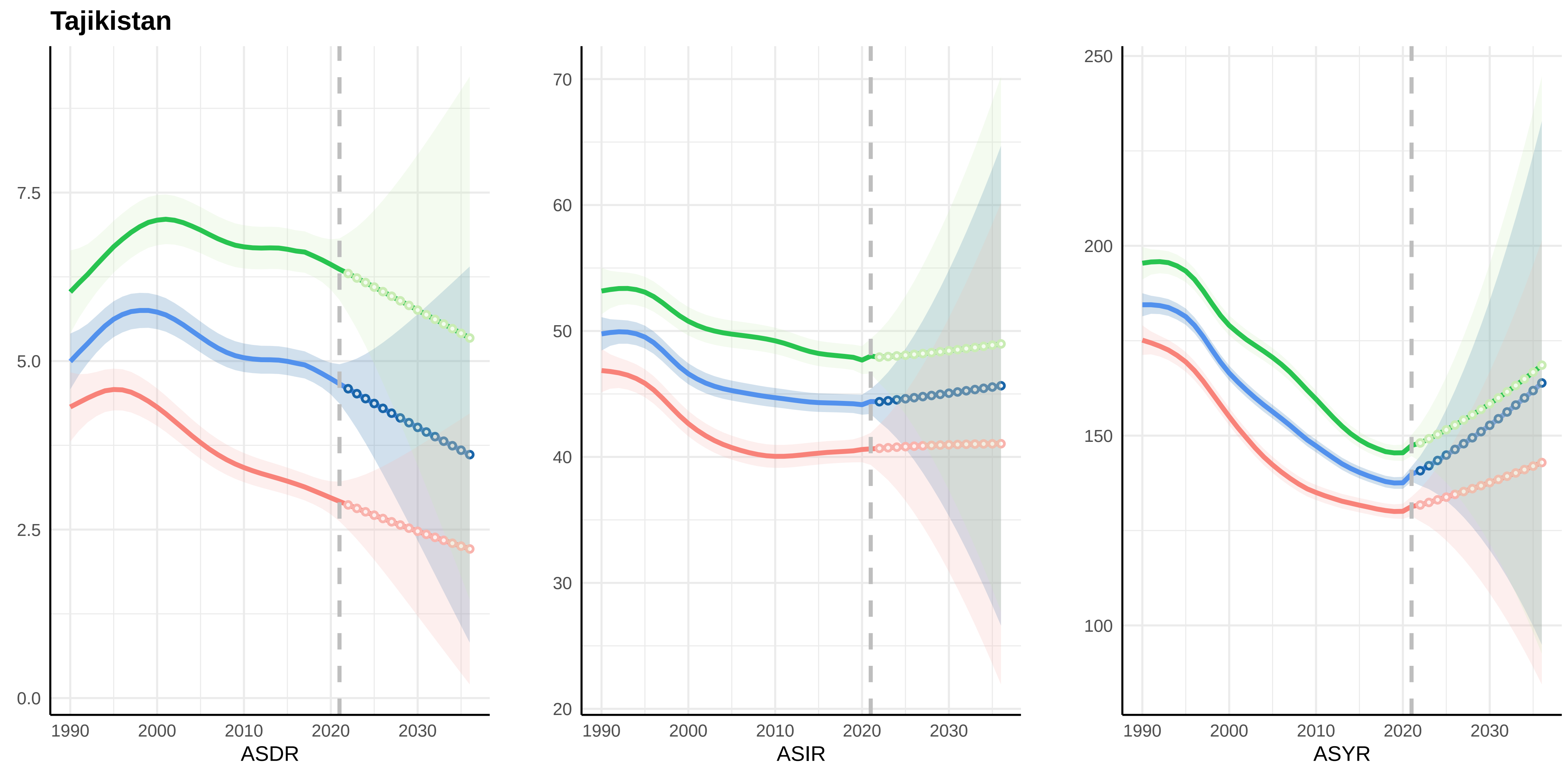

Tajikistan
7.5
5.0
2.5
0.0
1990
2000
2010
2020
2030
ASDR
70
60
50
40
30
20
1990
2000
2010
2020
2030
ASIR
250
200
150
100
1990
2000
2010
2020
2030
ASYR

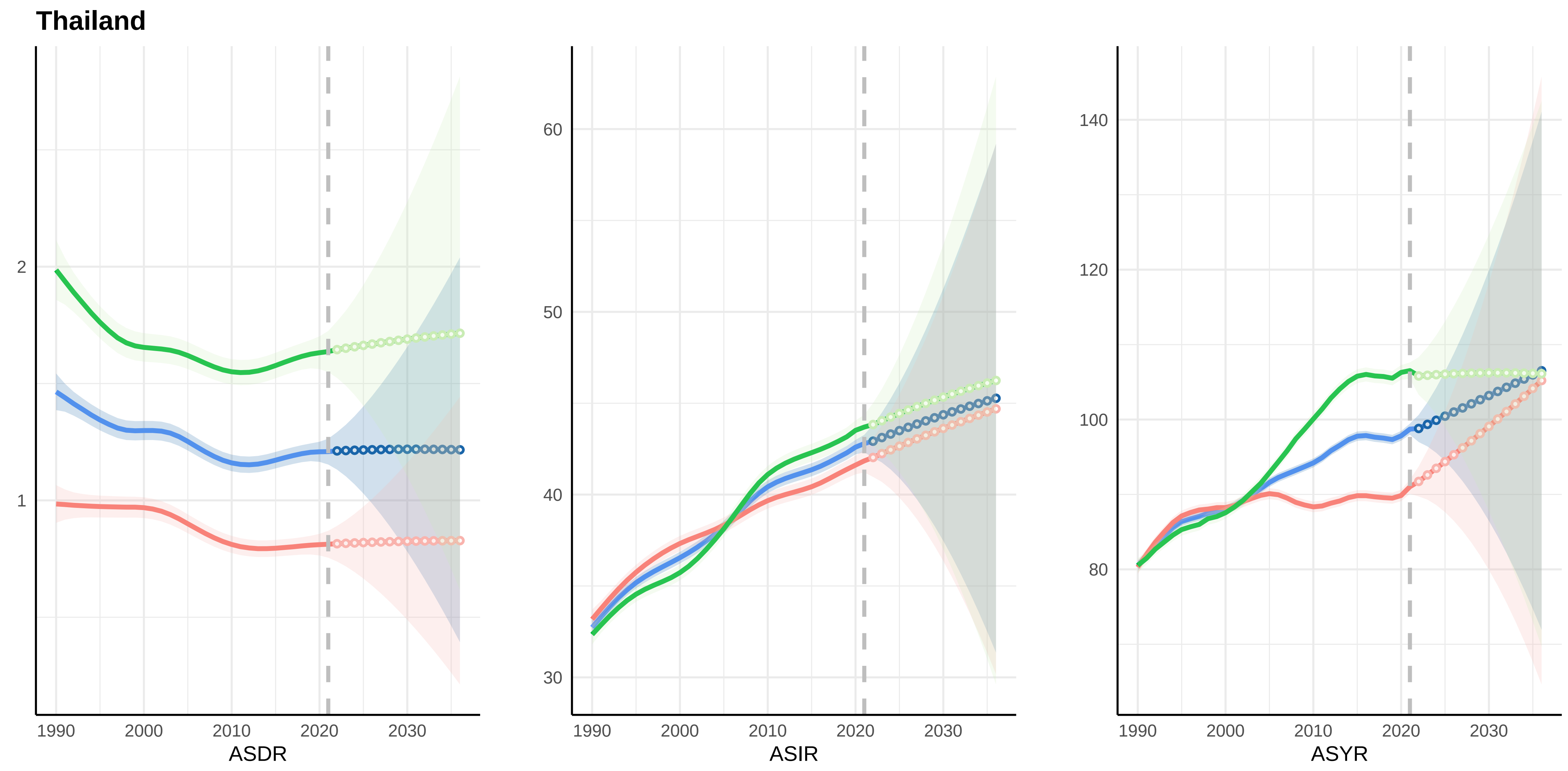

Thailand
2
1
1990
2000
2010
2020
2030
ASDR
60
50
40
30
1990
2000
2010
2020
2030
ASIR
140
120
100
80
1990
2000
2010
2020
2030
ASYR

Togo

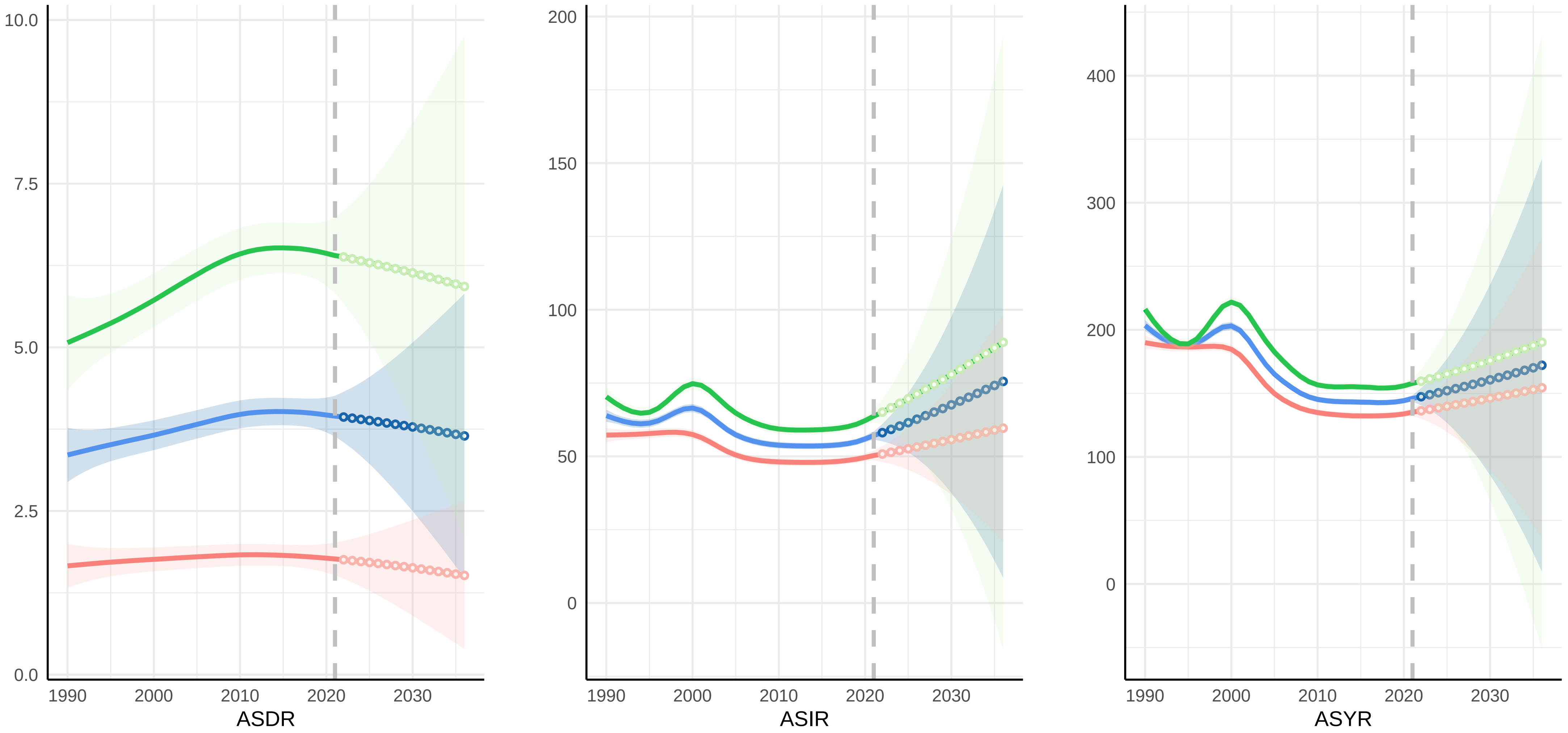

10.0
7.5
5.0
2.5
0.0
1990
2000
2010
2020
2030
ASDR
200
150
100
50
0
1990
2000
2010
2020
2030
ASIR
400
300
200
100
0
1990
2000
2010
2020
2030
ASYR

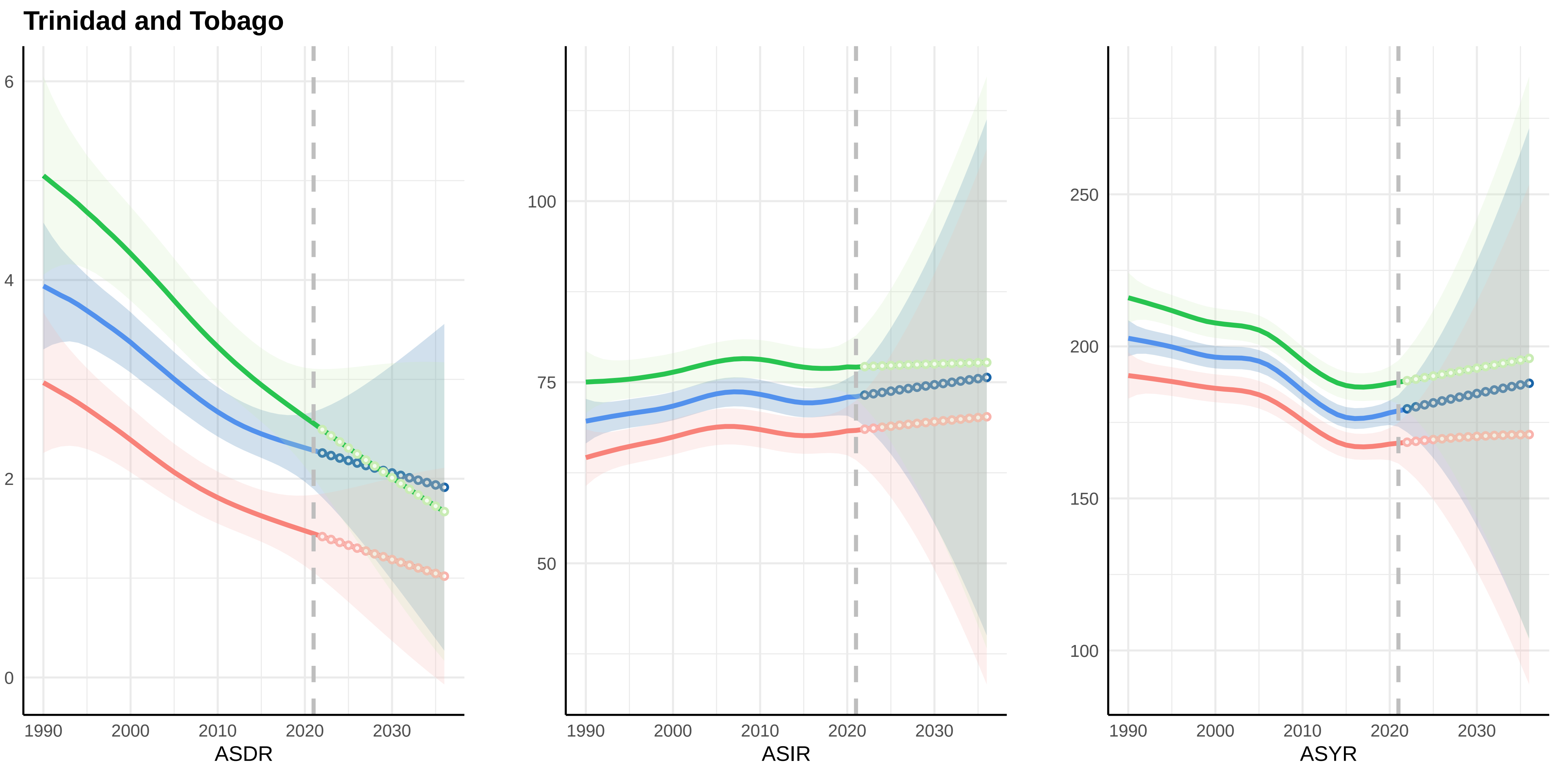
Trinidad and Tobago
6
4
2
0
1990
2000
2010
2020
2030
ASDR
100
75
50
1990
2000
2010
2020
2030
ASIR
250
200
150
100
1990
2000
2010
2020
2030
ASYR

Tunisia

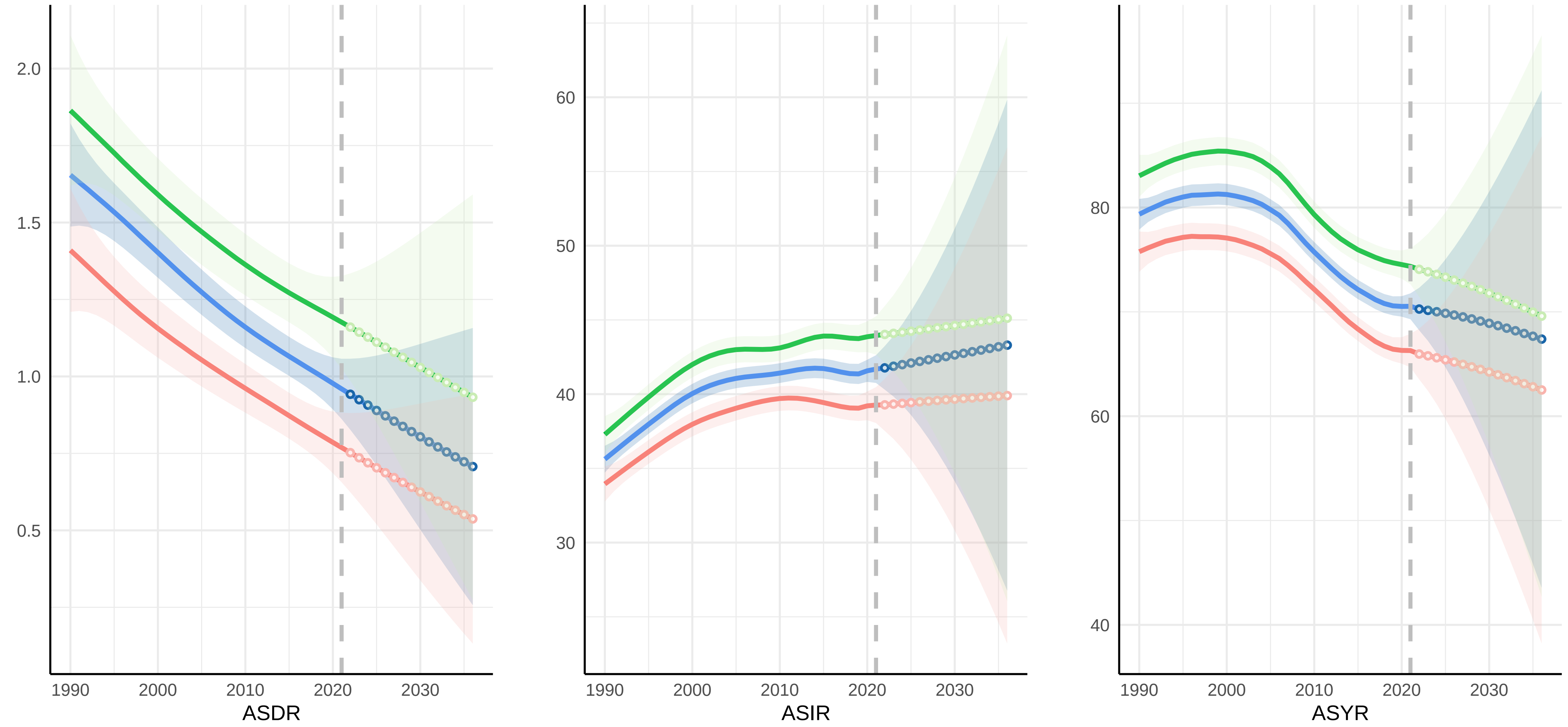

2.0
1.5
1.0
0.5
1990
2000
2010
2020
2030
ASDR
60
50
40
30
ASIR
80
60
40
ASYR

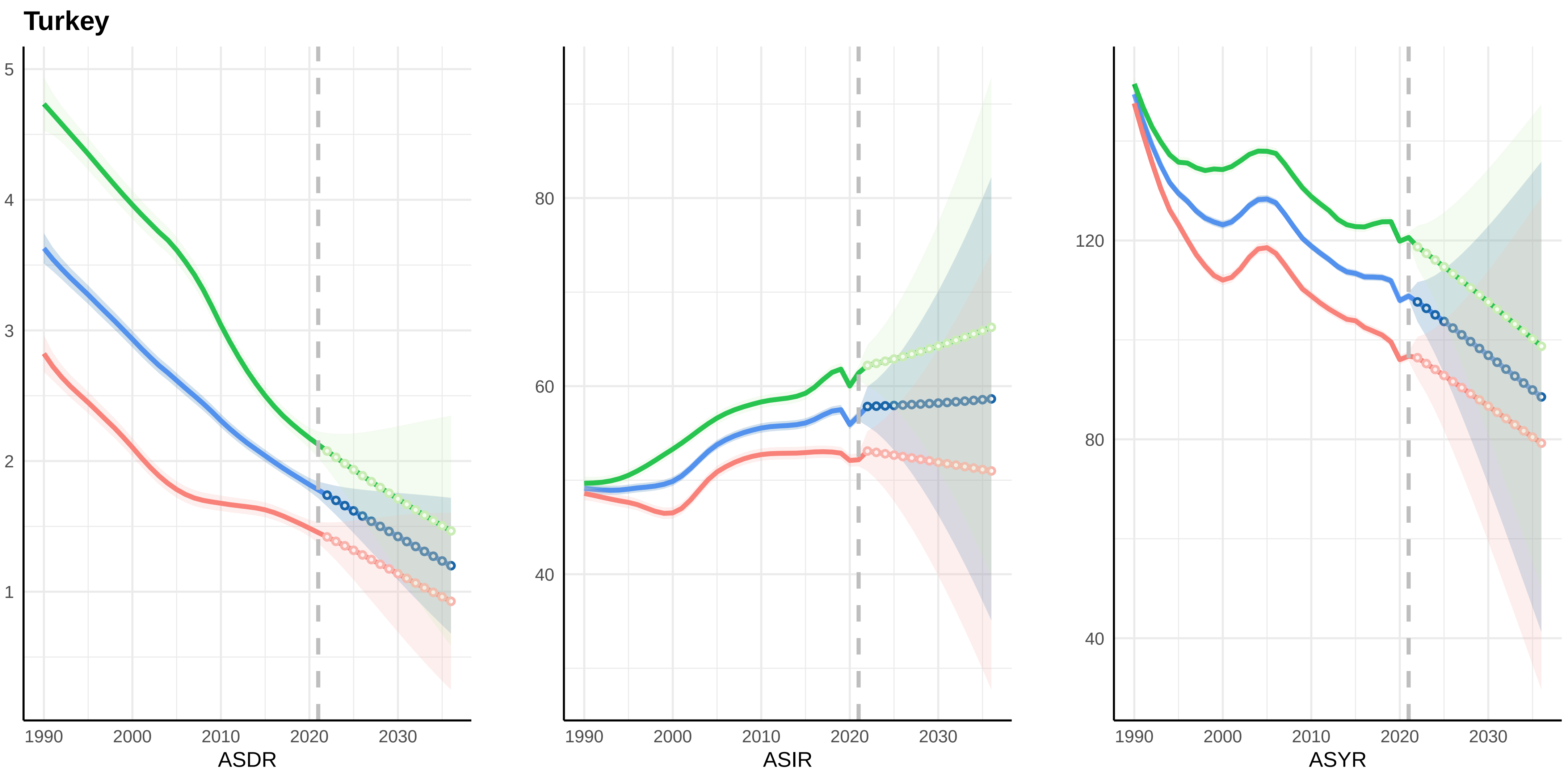

Turkey
5
4
3
2
1
1990
2000
2010
2020
2030
ASDR
80
60
40
1990
2000
2010
2020
2030
ASIR
120
80
40
1990
2000
2010
2020
2030
ASYR

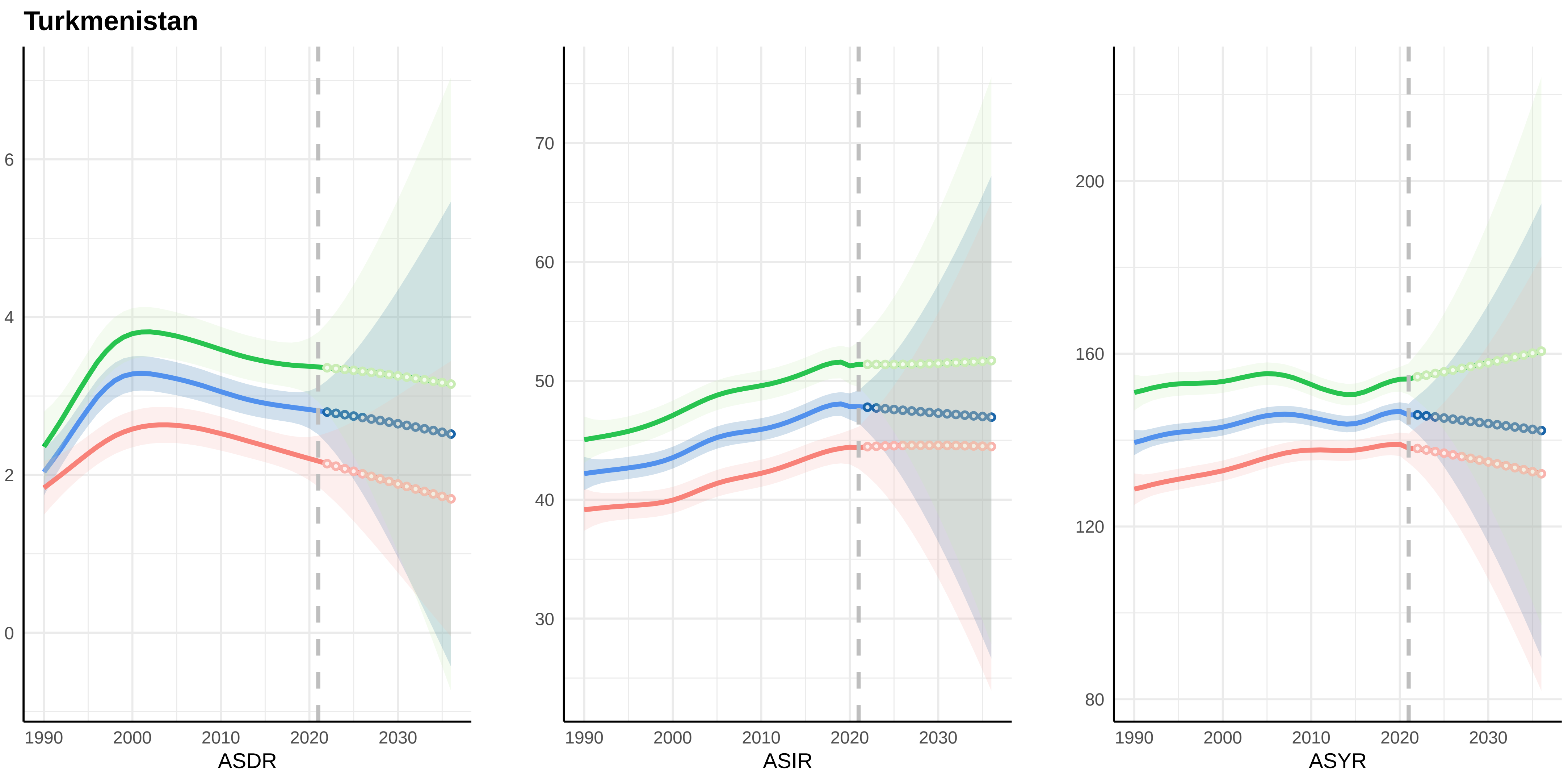

Turkmenistan
6
4
2
0
1990
2000
2010
2020
2030
ASDR
70
60
50
40
30
1990
2000
2010
2020
2030
ASIR
200
160
120
80
1990
2000
2010
2020
2030
ASYR

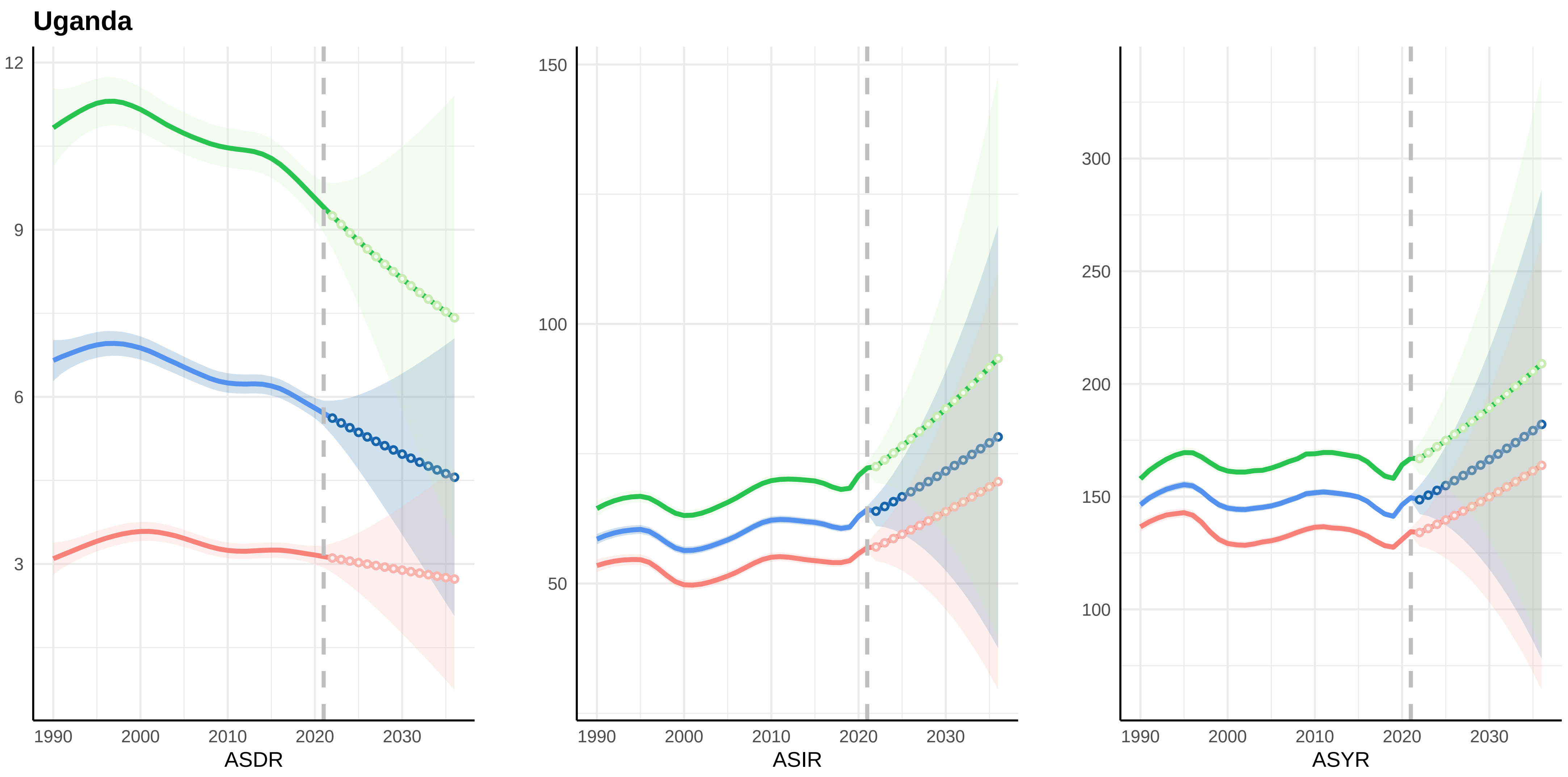

Uganda
12
9
6
3
1990
2000
2010
2020
2030
ASDR
150
100
50
ASIR
300
250
200
150
100
ASYR

Ukraine

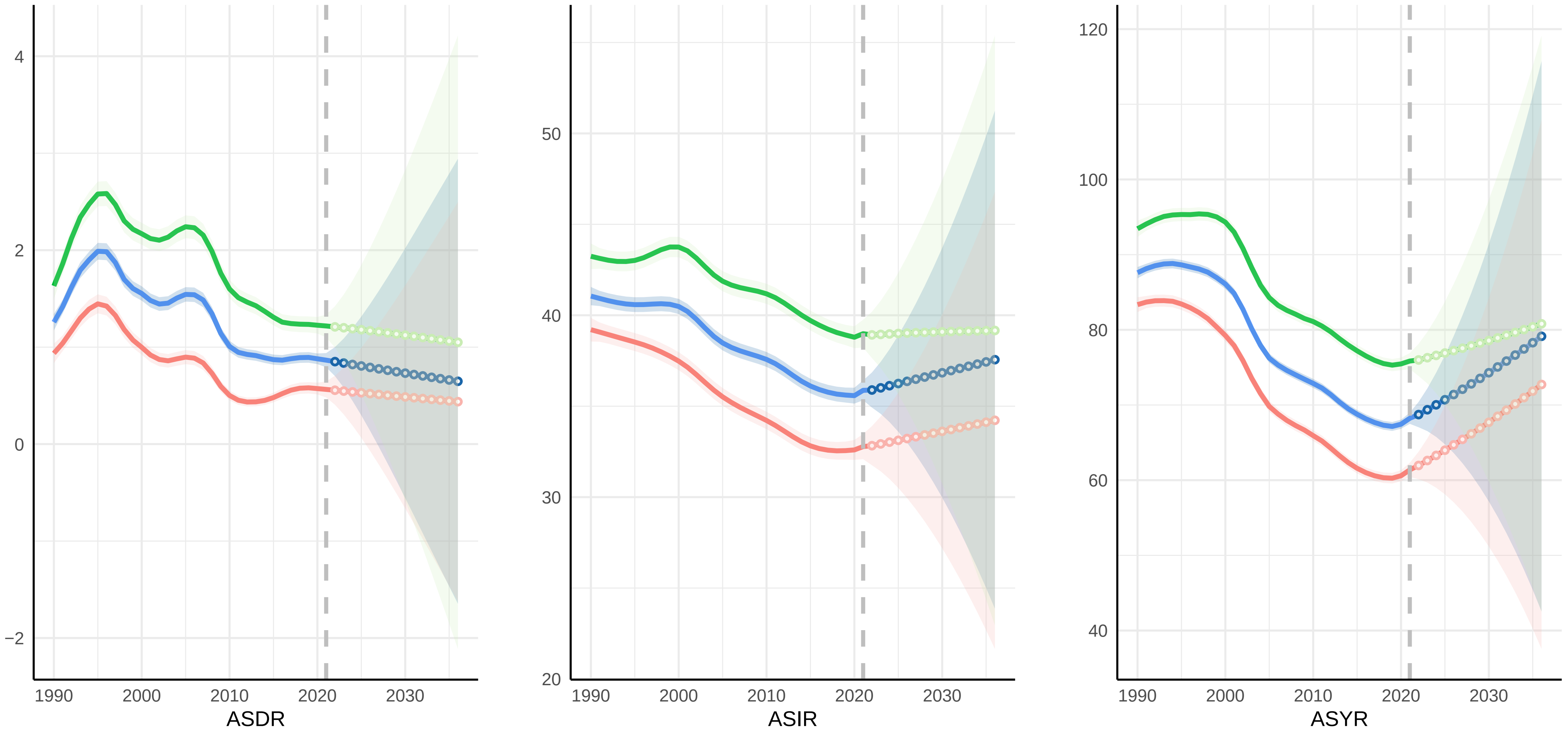

4
2
0
−2
1990
2000
2010
2020
2030
ASDR
50
40
30
20
ASIR
120
100
80
60
40
ASYR

United Arab Emirates

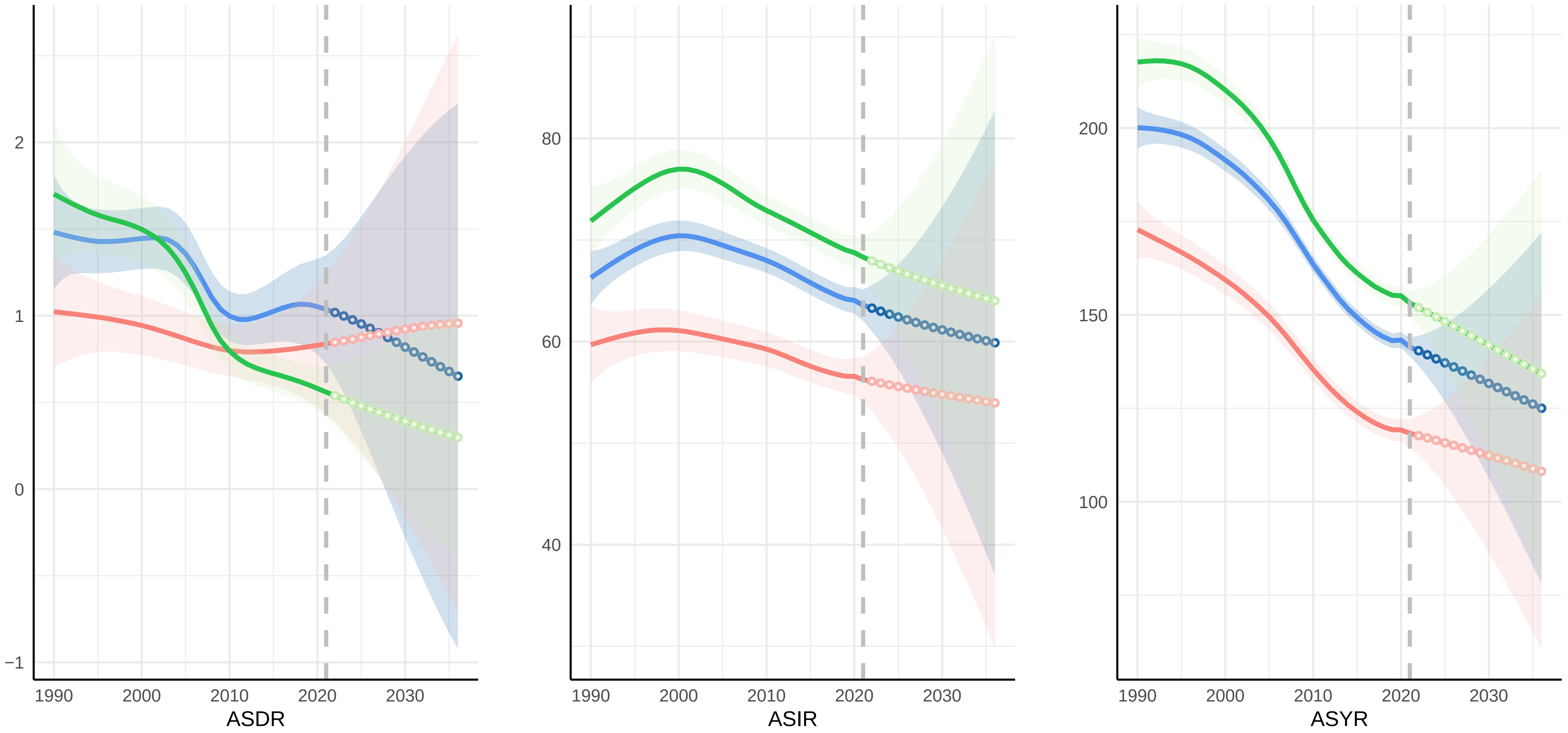

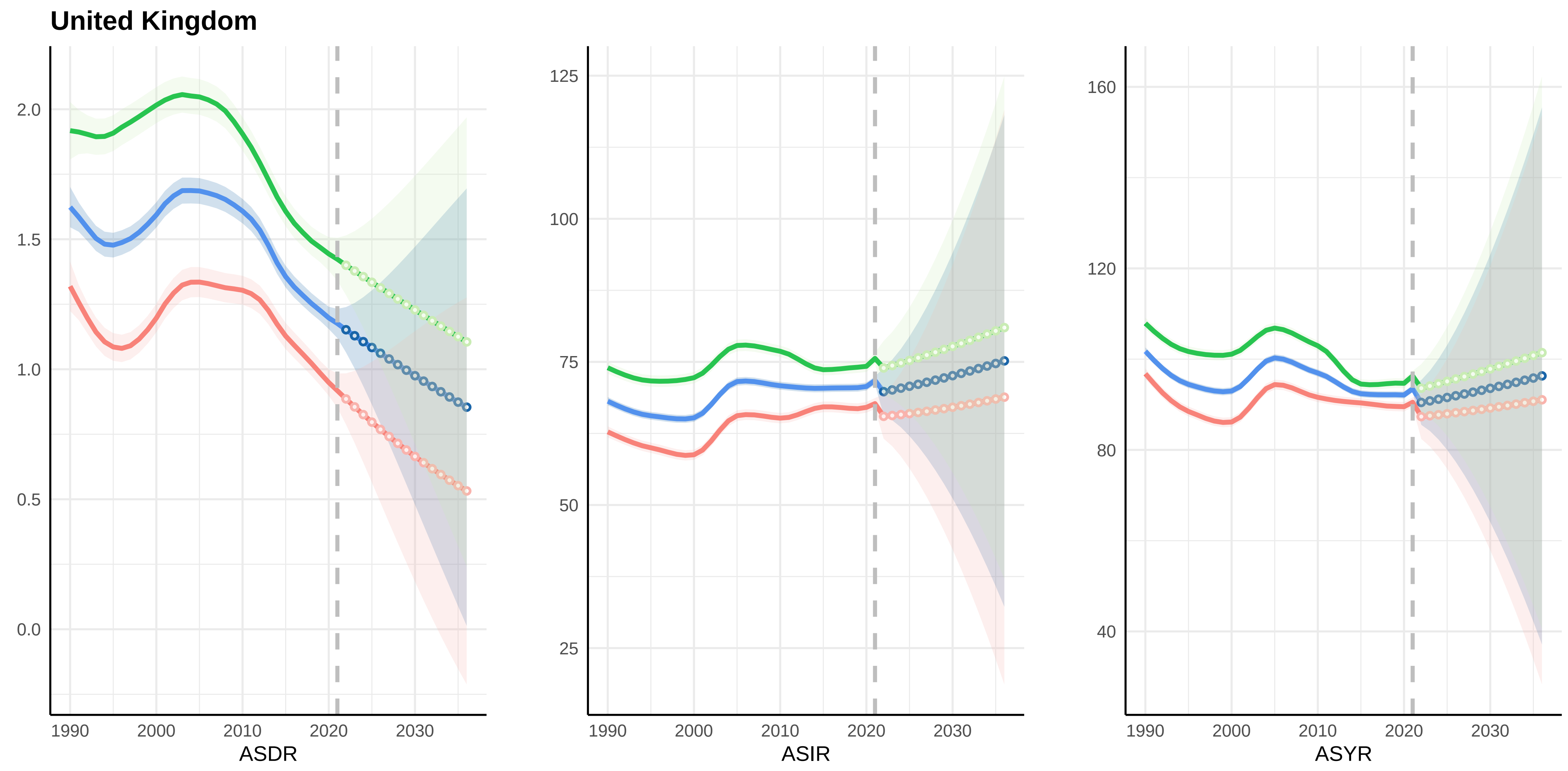
United Kingdom
2.0
1.5
1.0
0.5
0.0
1990
2000
2010
2020
2030
ASDR
125
100
75
50
25
1990
2000
2010
2020
2030
ASIR
160
120
80
40
1990
2000
2010
2020
2030
ASYR

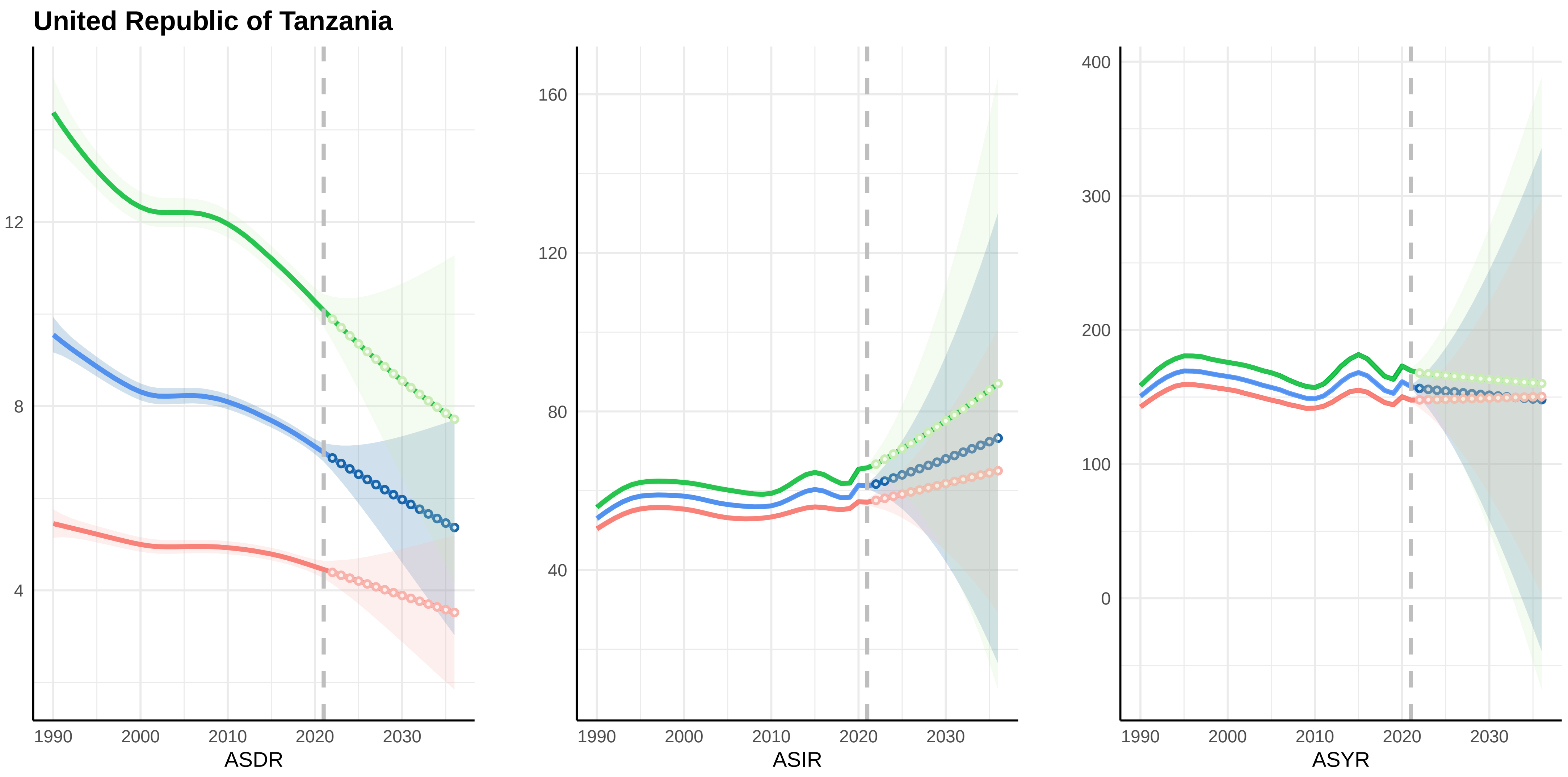

United Republic of Tanzania
12
8
4
1990
2000
2010
2020
2030
ASDR
160
120
80
40
1990
2000
2010
2020
2030
ASIR
400
300
200
100
0
1990
2000
2010
2020
2030
ASYR

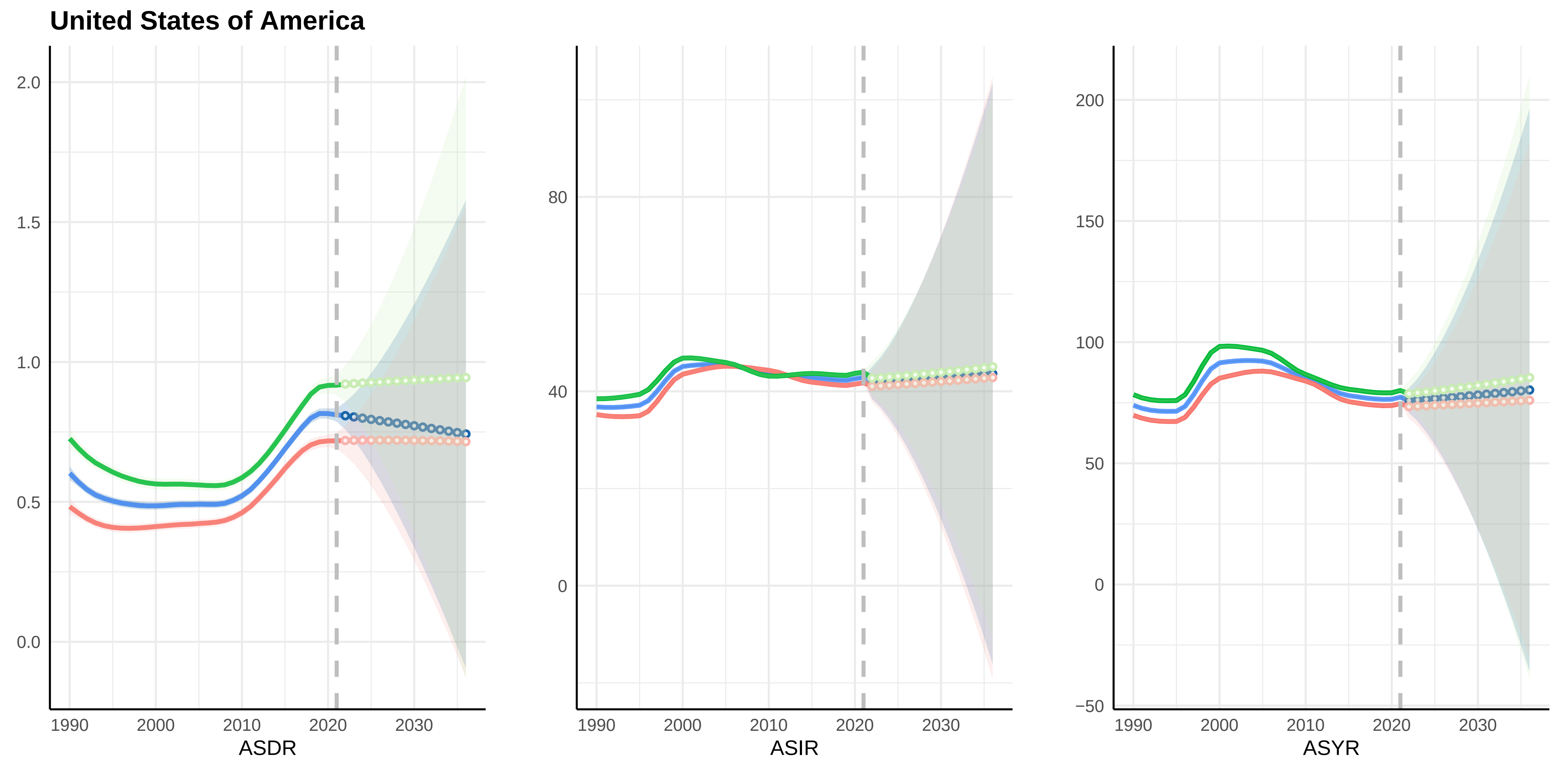
United States of America
2.0
1.5
1.0
0.5
0.0
1990
2000
2010
2020
2030
ASDR
80
40
0
ASIR
200
150
100
50
0
−50
ASYR

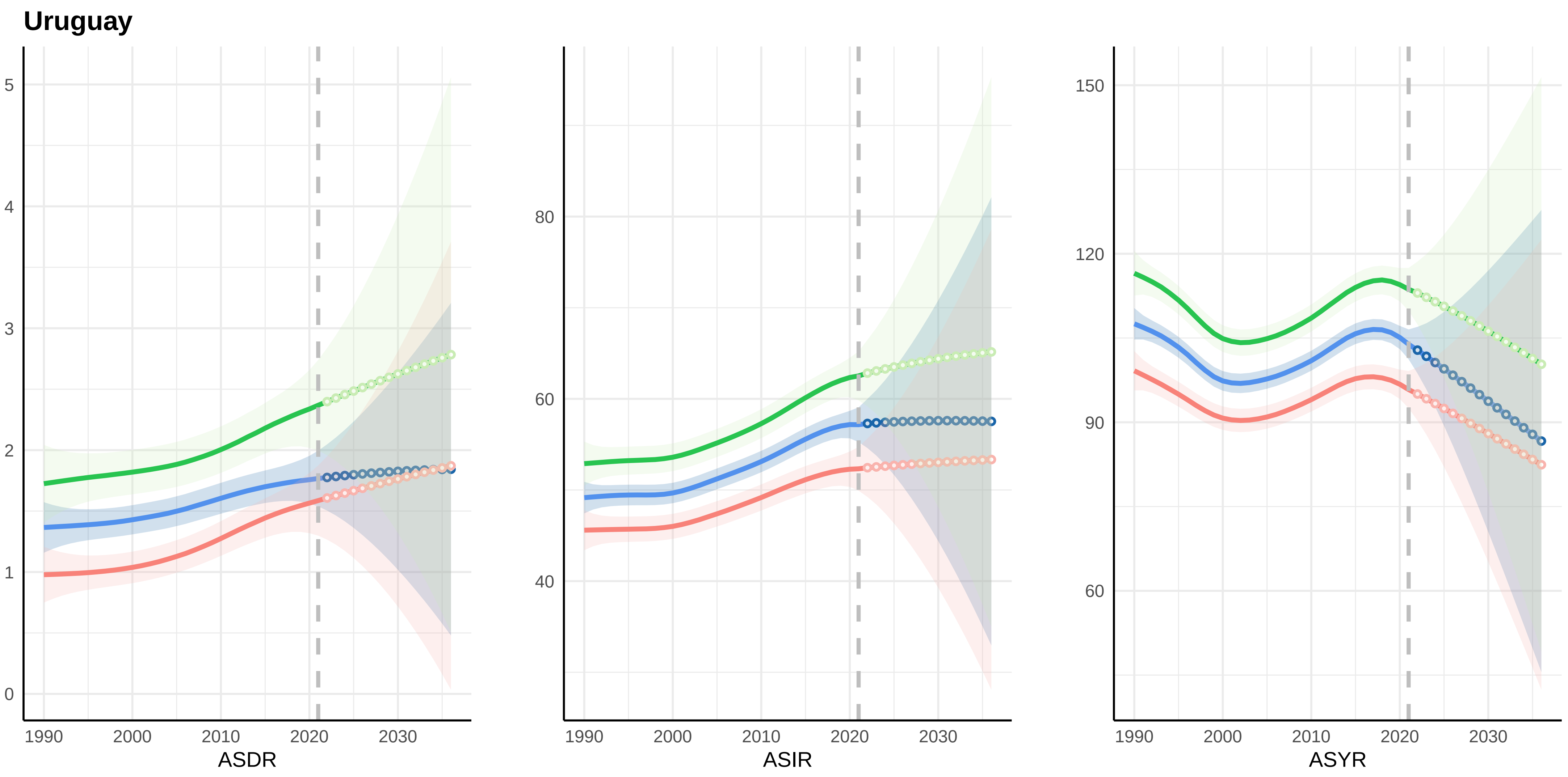

Uruguay
5
4
3
2
1
0
1990
2000
2010
2020
2030
ASDR
80
60
40
1990
2000
2010
2020
2030
ASIR
150
120
90
60
1990
2000
2010
2020
2030
ASYR

Uzbekistan

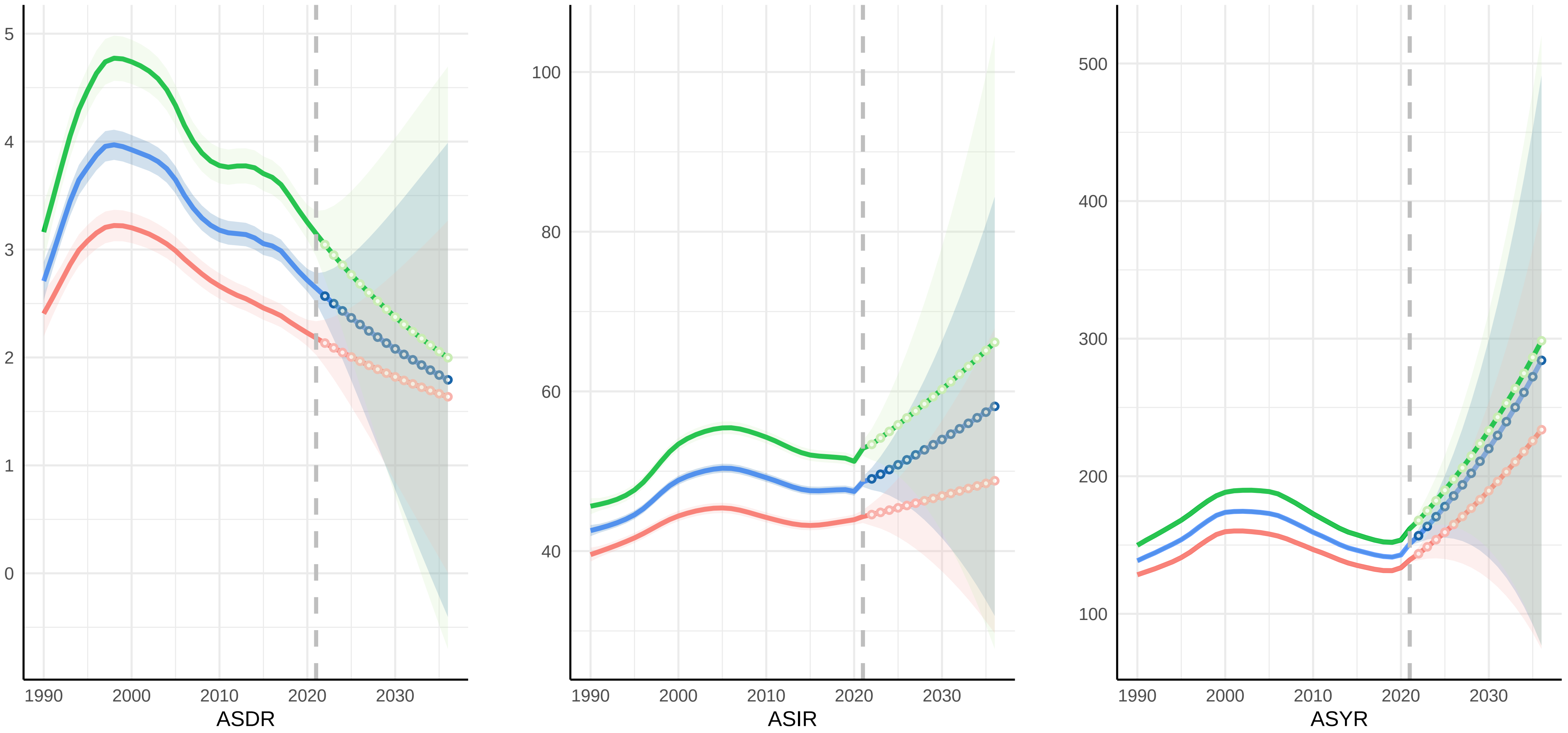

5
4
3
2
1
0
1990
2000
2010
2020
2030
ASDR
100
80
60
40
1990
2000
2010
2020
2030
ASIR
500
400
300
200
100
1990
2000
2010
2020
2030
ASYR

Venezuela (Bolivarian Republic of)

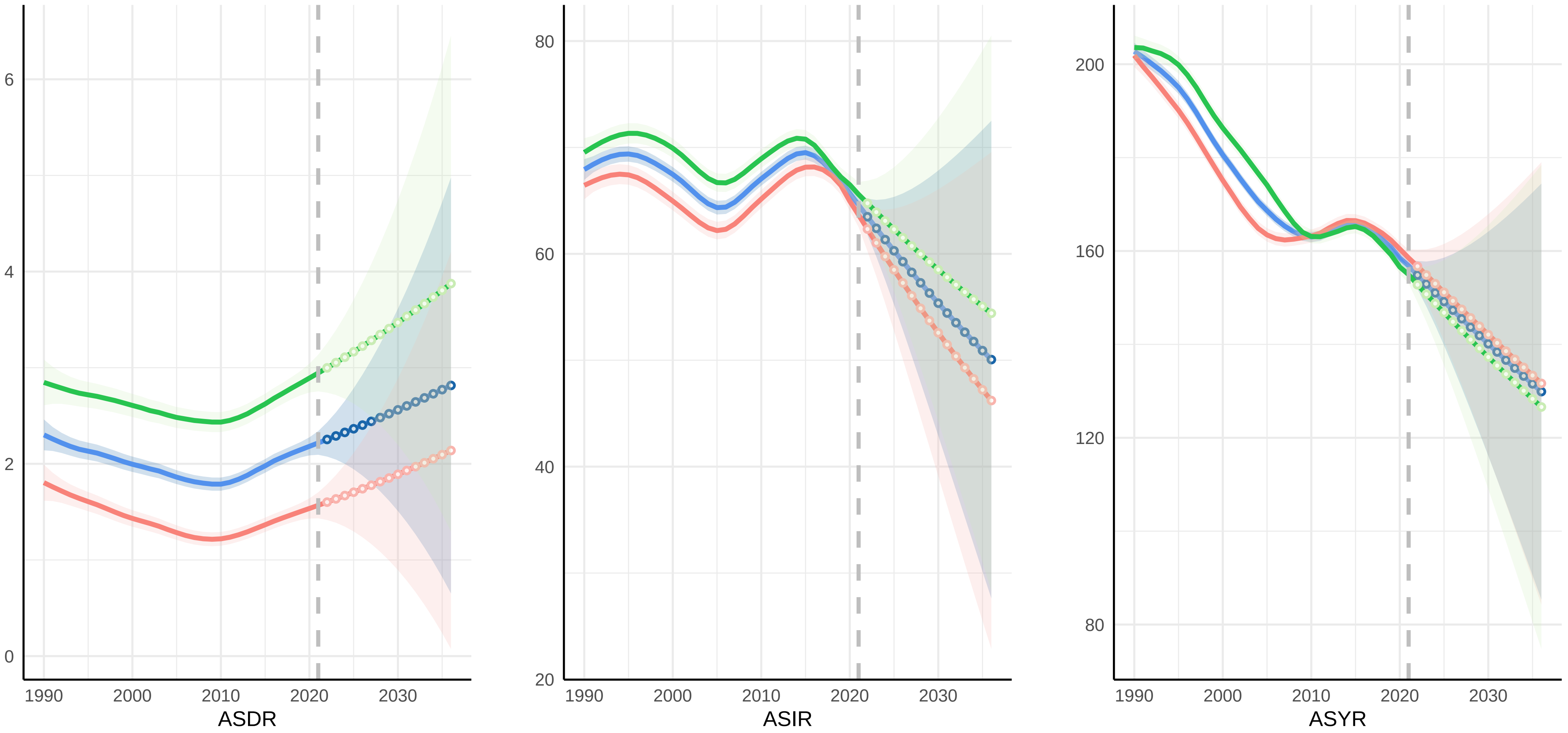

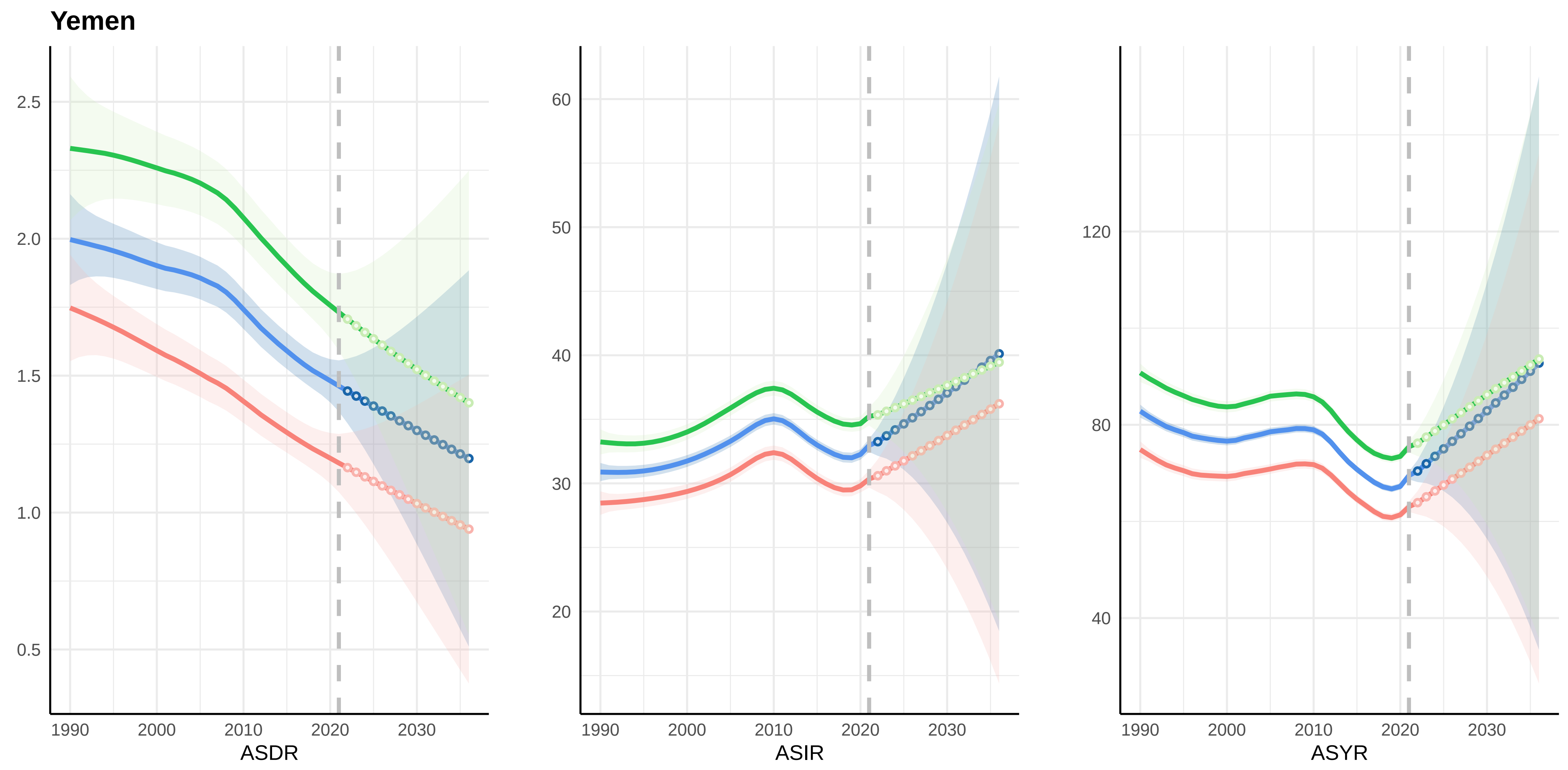

Yemen
2.5
2.0
1.5
1.0
0.5
1990
2000
2010
2020
2030
ASDR
60
50
40
30
20
ASIR
120
80
40
ASYR

Zambia

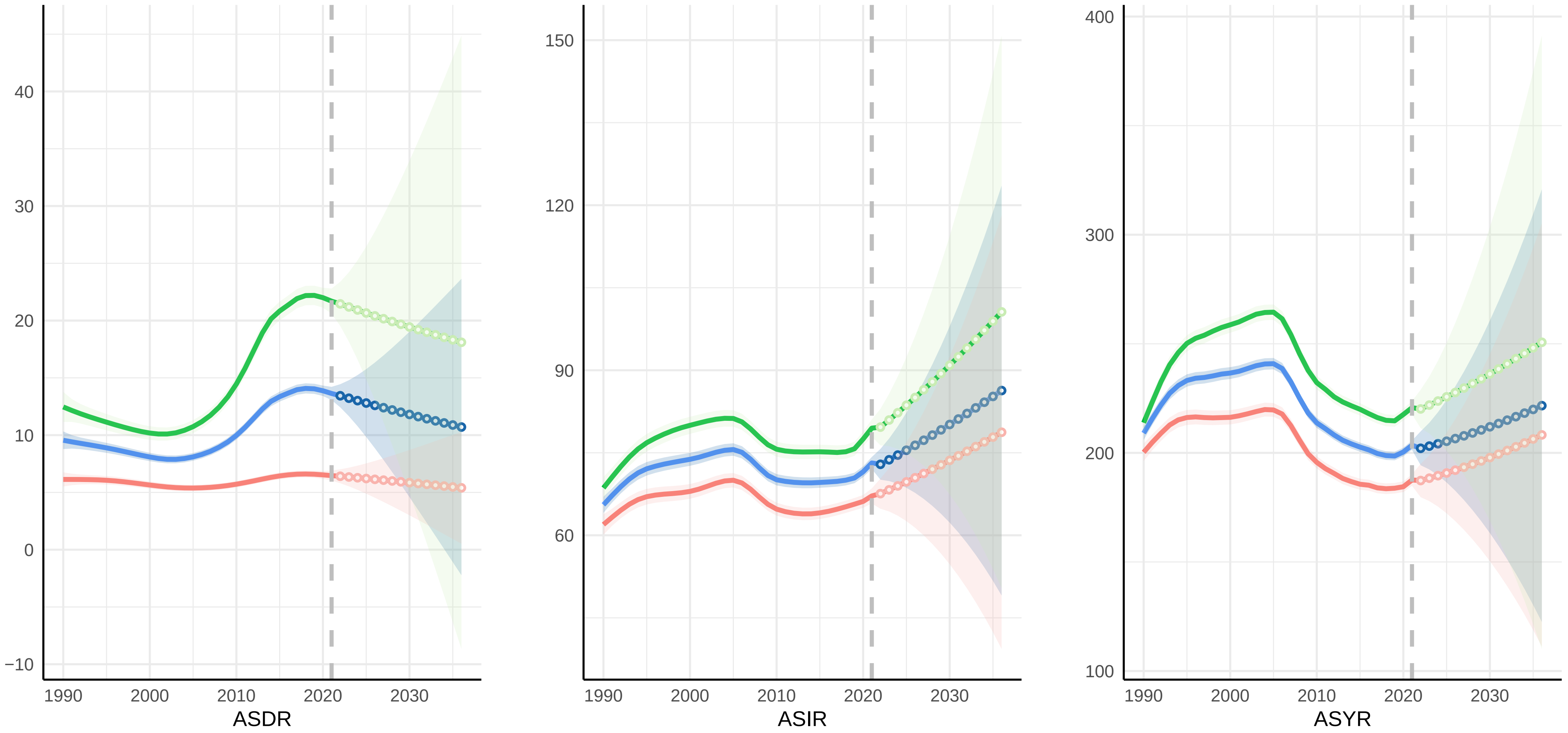

40
30
20
10
0
−10
1990
2000
2010
2020
2030
ASDR
150
120
90
60
1990
2000
2010
2020
2030
ASIR
400
300
200
100
1990
2000
2010
2020
2030
ASYR

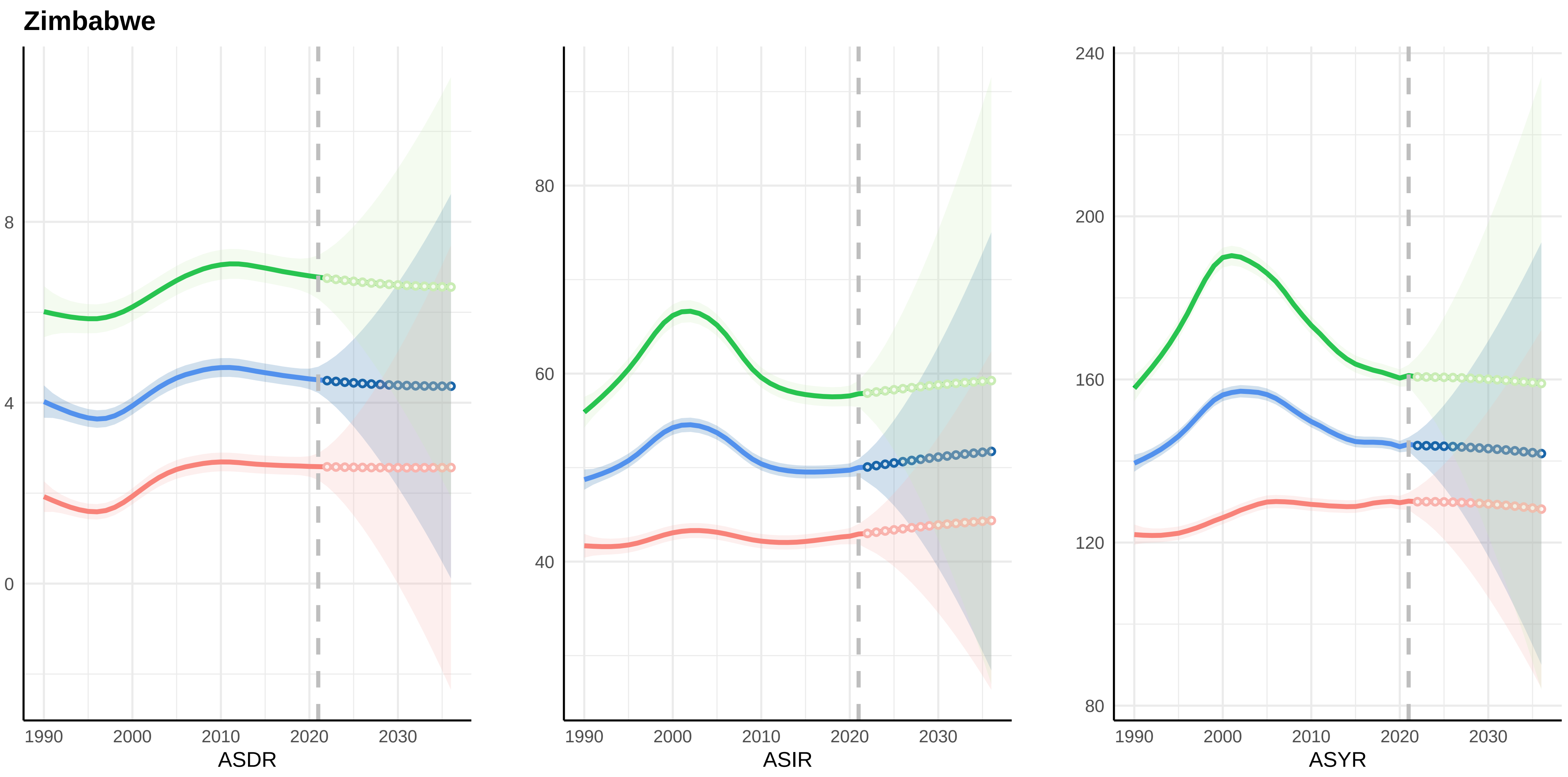
Zimbabwe
8
4
0
1990
2000
2010
2020
2030
ASDR
80
60
40
1990
2000
2010
2020
2030
ASIR
240
200
160
120
80
1990
2000
2010
2020
2030
ASYR
