# Supplementary material for: Global, regional, and national epilepsy of unknown cause incidence and mortality, 1990–2036: cross-national health inequalities and predictive analytics
Source: Front Neurol. 2025 Jun 30;16:1526984. doi: 10.3389/fneur.2025.1526984 (PMC12256229; doi:10.3389/fneur.2025.1526984)
Supplement: Supplementary file 2 [file Data_Sheet_2.pdf]

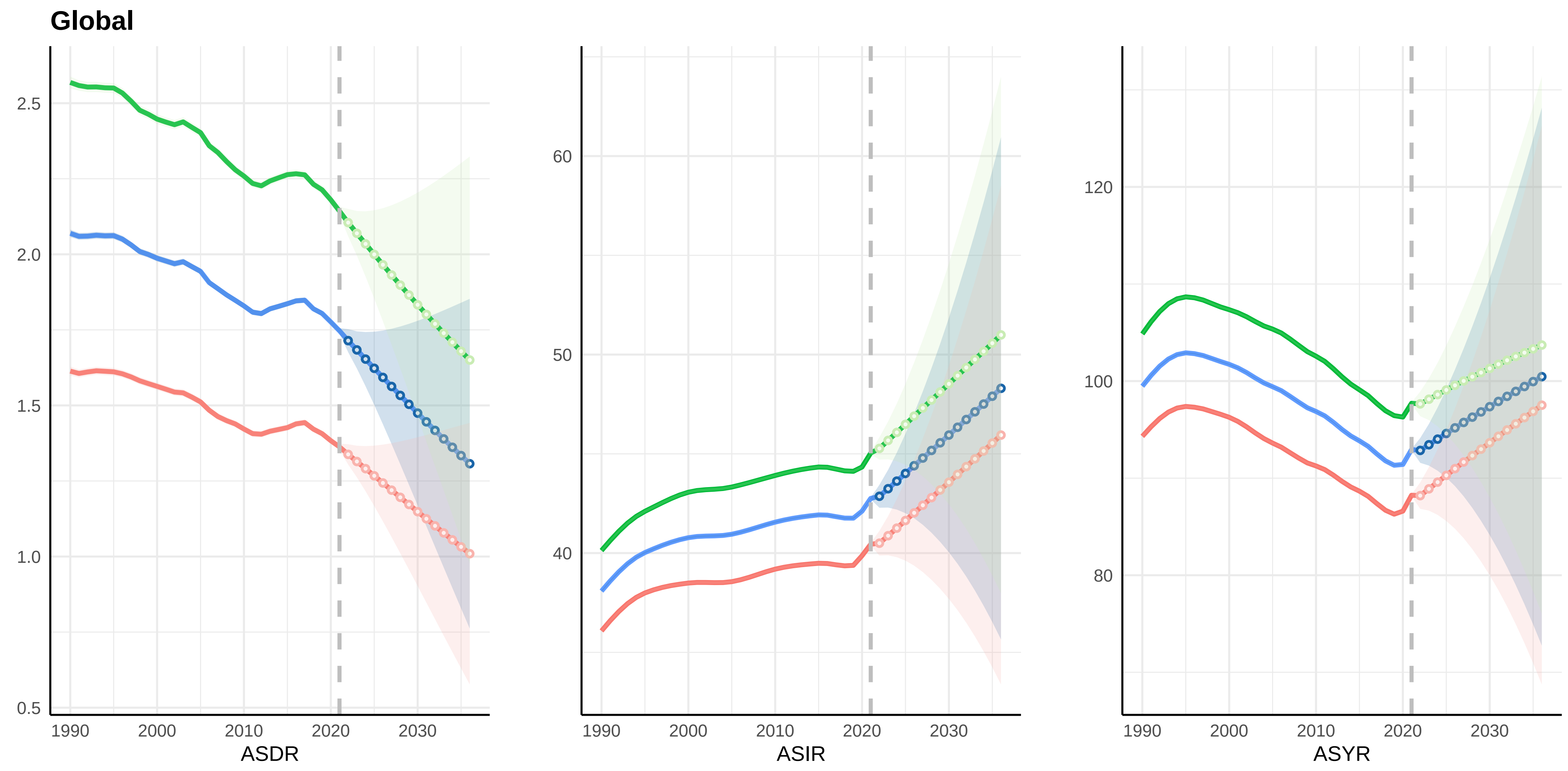

Global
2.5
2.0
1.5
1.0
0.5
1990
2000
2010
2020
2030
ASDR
60
50
40
1990
2000
2010
2020
2030
ASIR
120
100
80
1990
2000
2010
2020
2030
ASYR

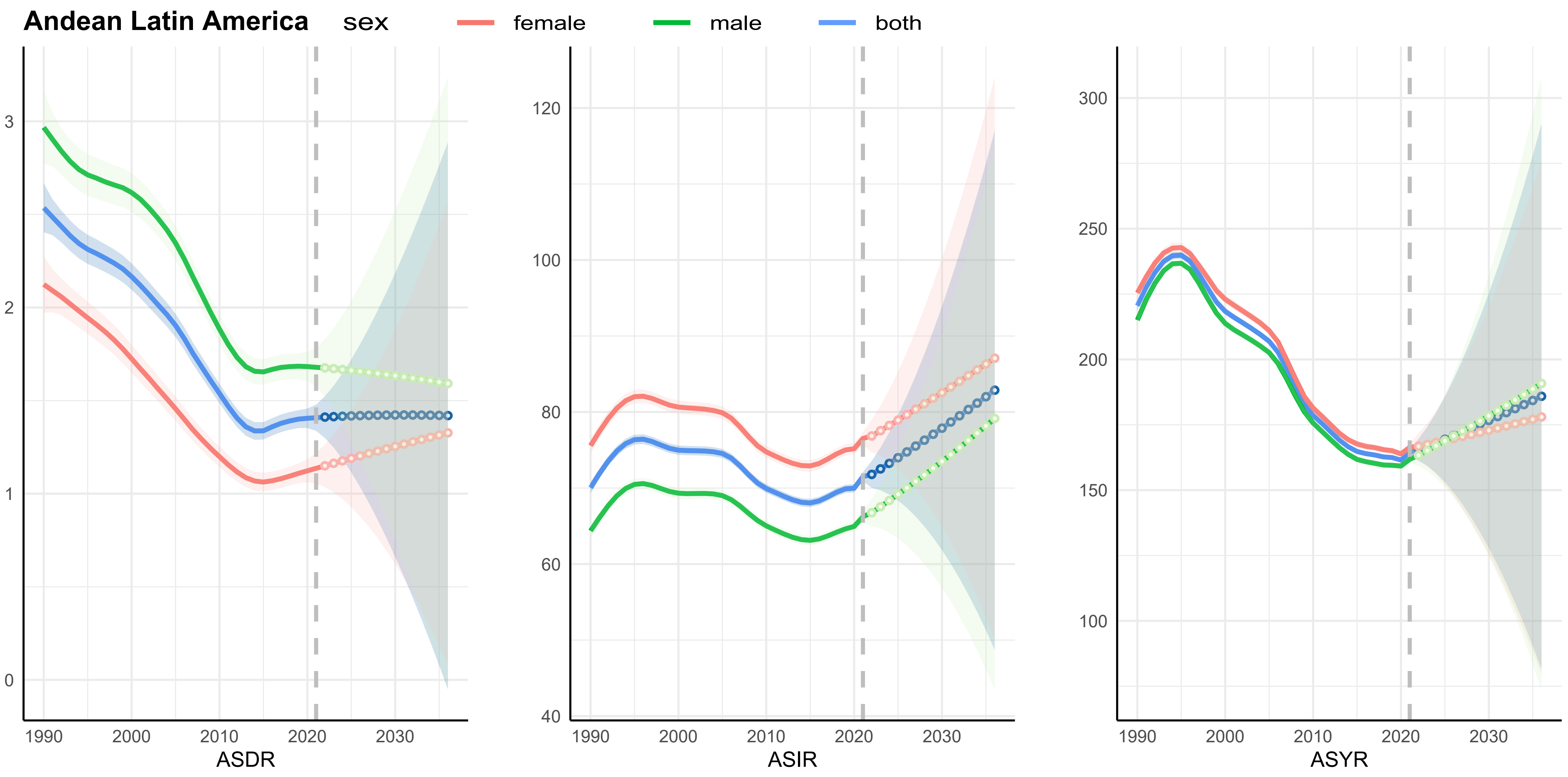

Andean Latin America
sex
female
male
both
3
2
1
0
1990
2000
2010
2020
2030
ASDR
120
100
80
60
40
ASIR
300
250
200
150
100
ASYR

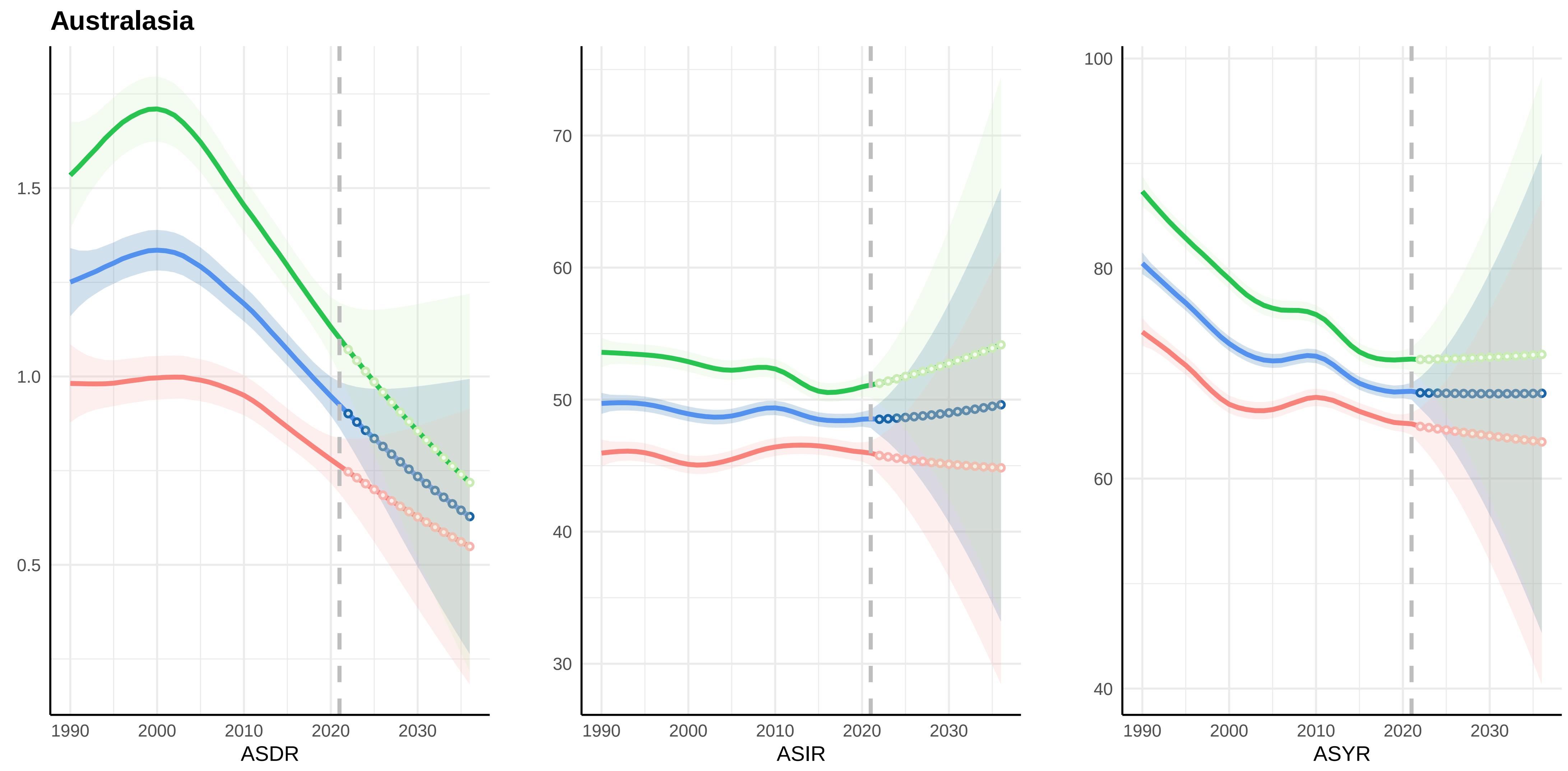

Australasia
1.5
1.0
0.5
1990
2000
2010
2020
2030
ASDR
70
60
50
40
30
1990
2000
2010
2020
2030
ASIR
100
80
60
40
1990
2000
2010
2020
2030
ASYR

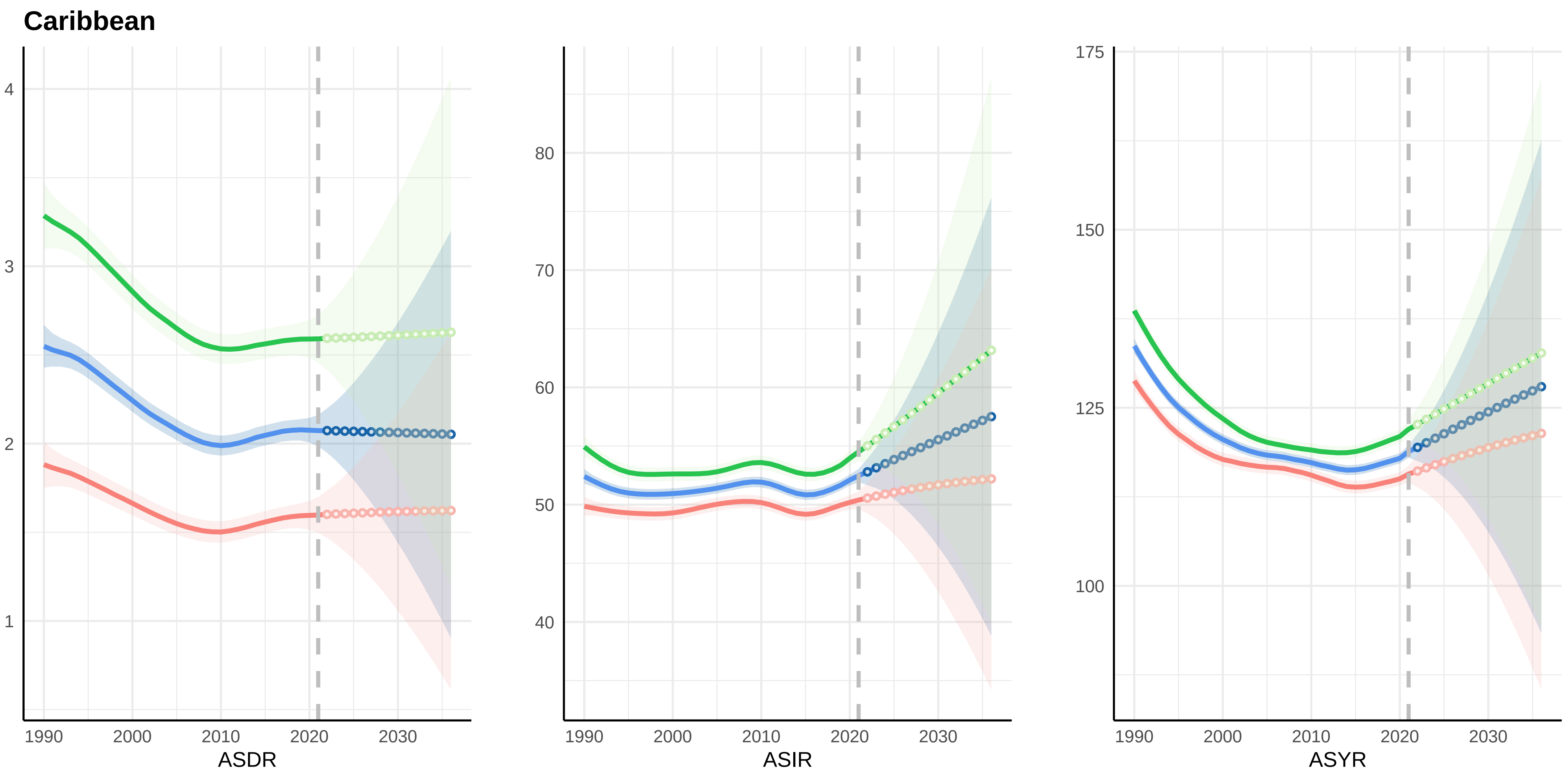
Caribbean
4
3
2
1
1990
2000
2010
2020
2030
ASDR
80
70
60
50
40
1990
2000
2010
2020
2030
ASIR
175
150
125
100
1990
2000
2010
2020
2030
ASYR

Central Asia

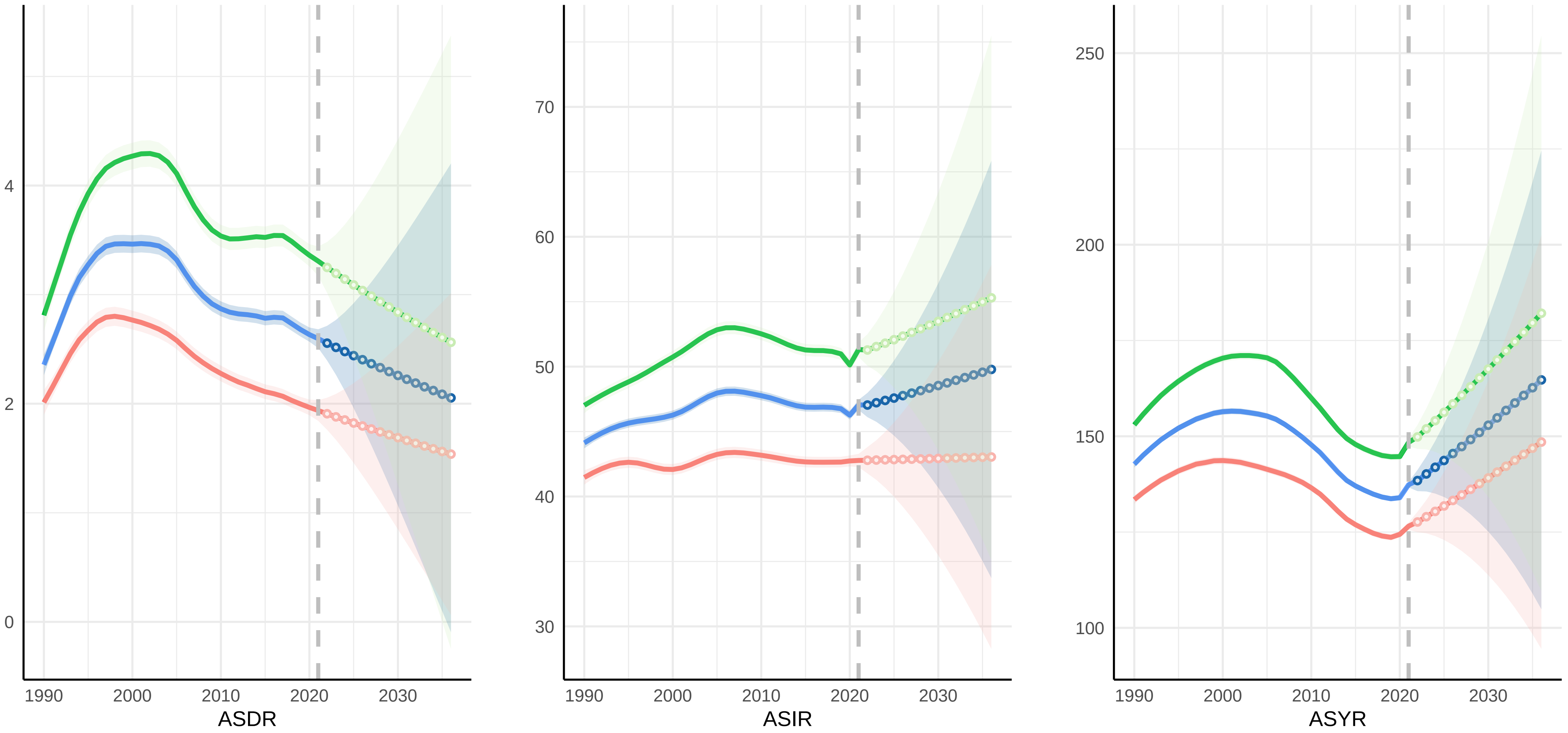

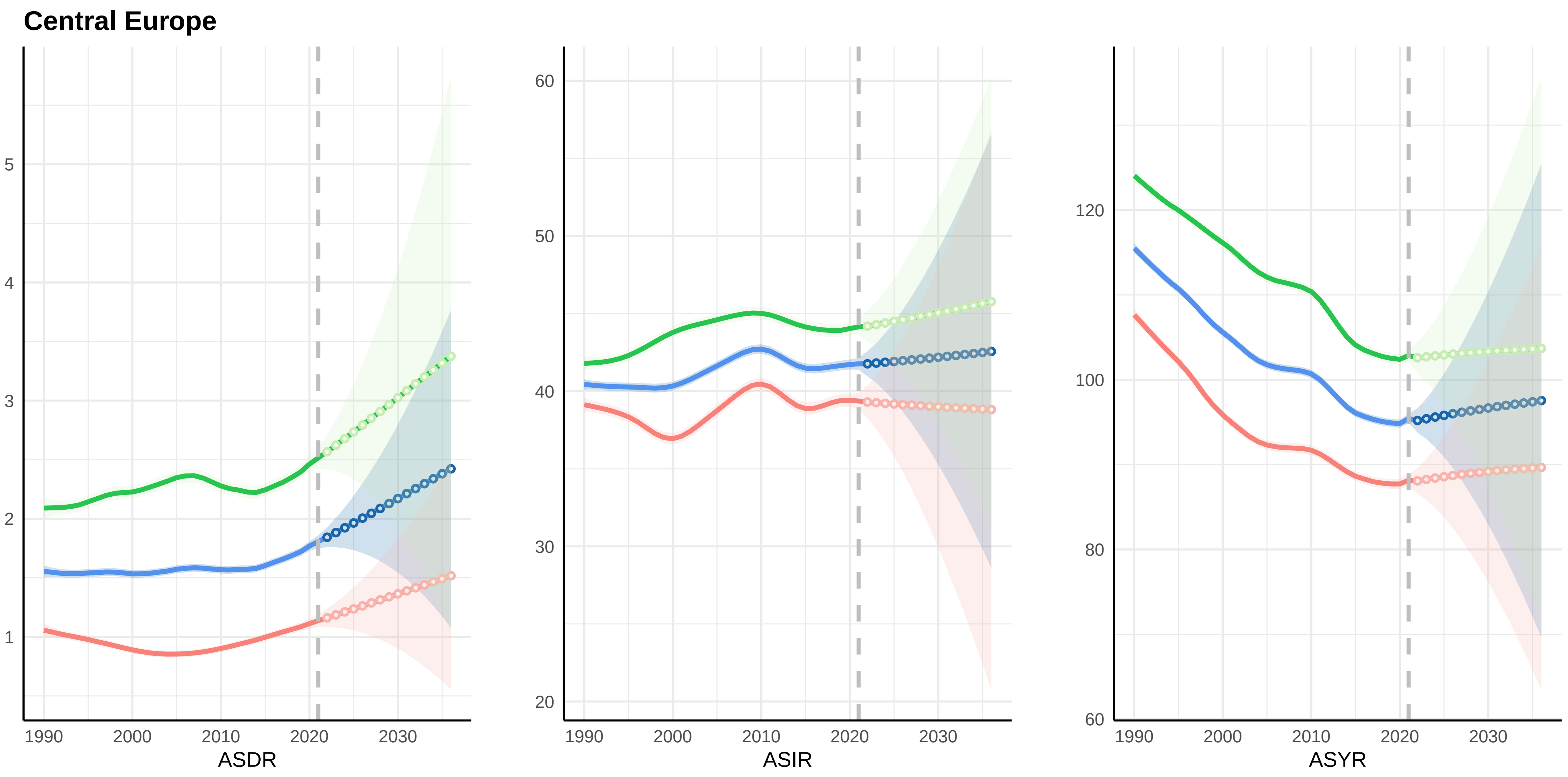
Central Europe
5
4
3
2
1
1990
2000
2010
2020
2030
ASDR
60
50
40
30
20
1990
2000
2010
2020
2030
ASIR
120
100
80
60
1990
2000
2010
2020
2030
ASYR

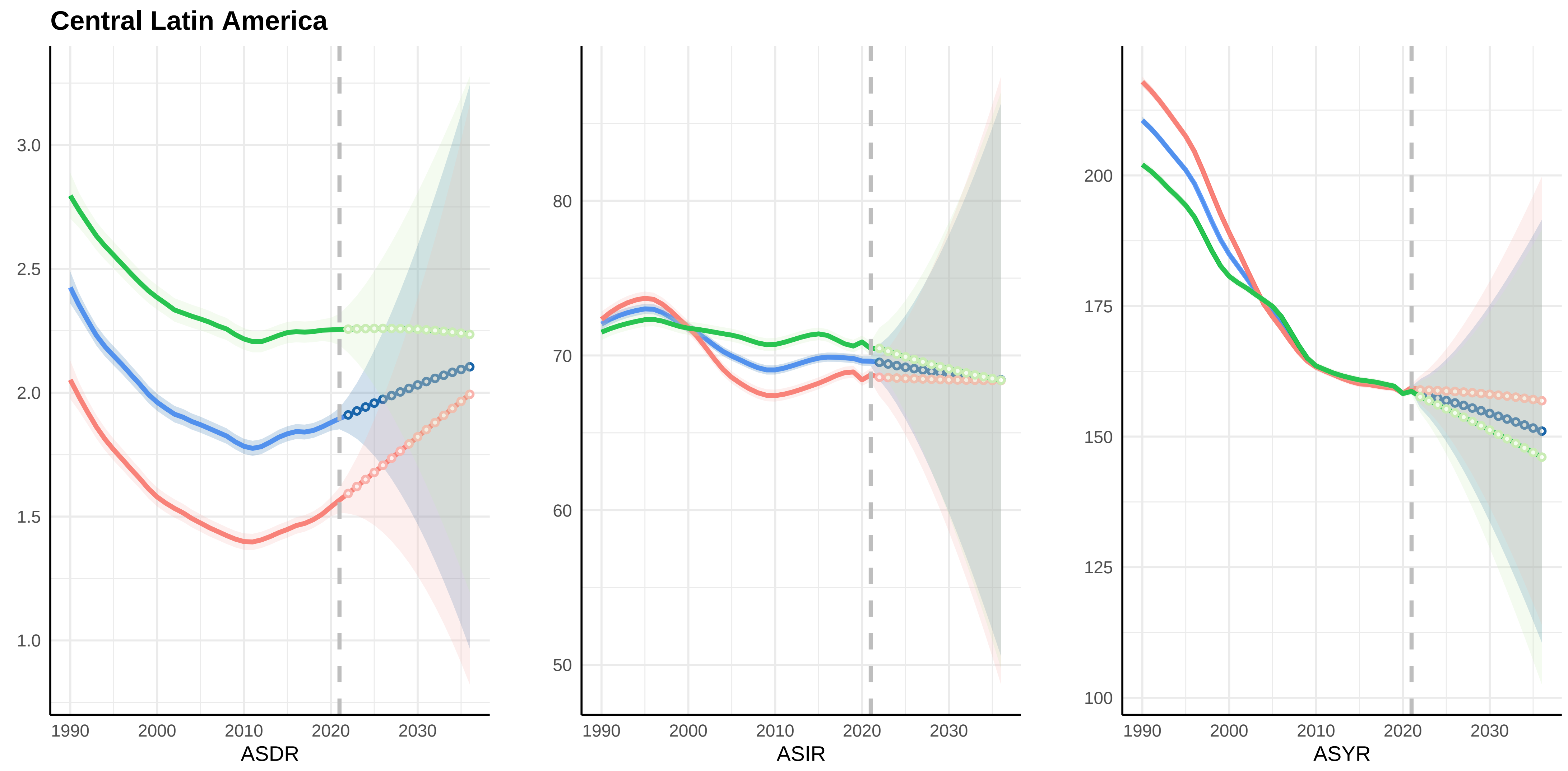

Central Latin America
3.0
2.5
2.0
1.5
1.0
1990
2000
2010
2020
2030
ASDR
80
70
60
50
1990
2000
2010
2020
2030
ASIR
200
175
150
125
100
1990
2000
2010
2020
2030
ASYR

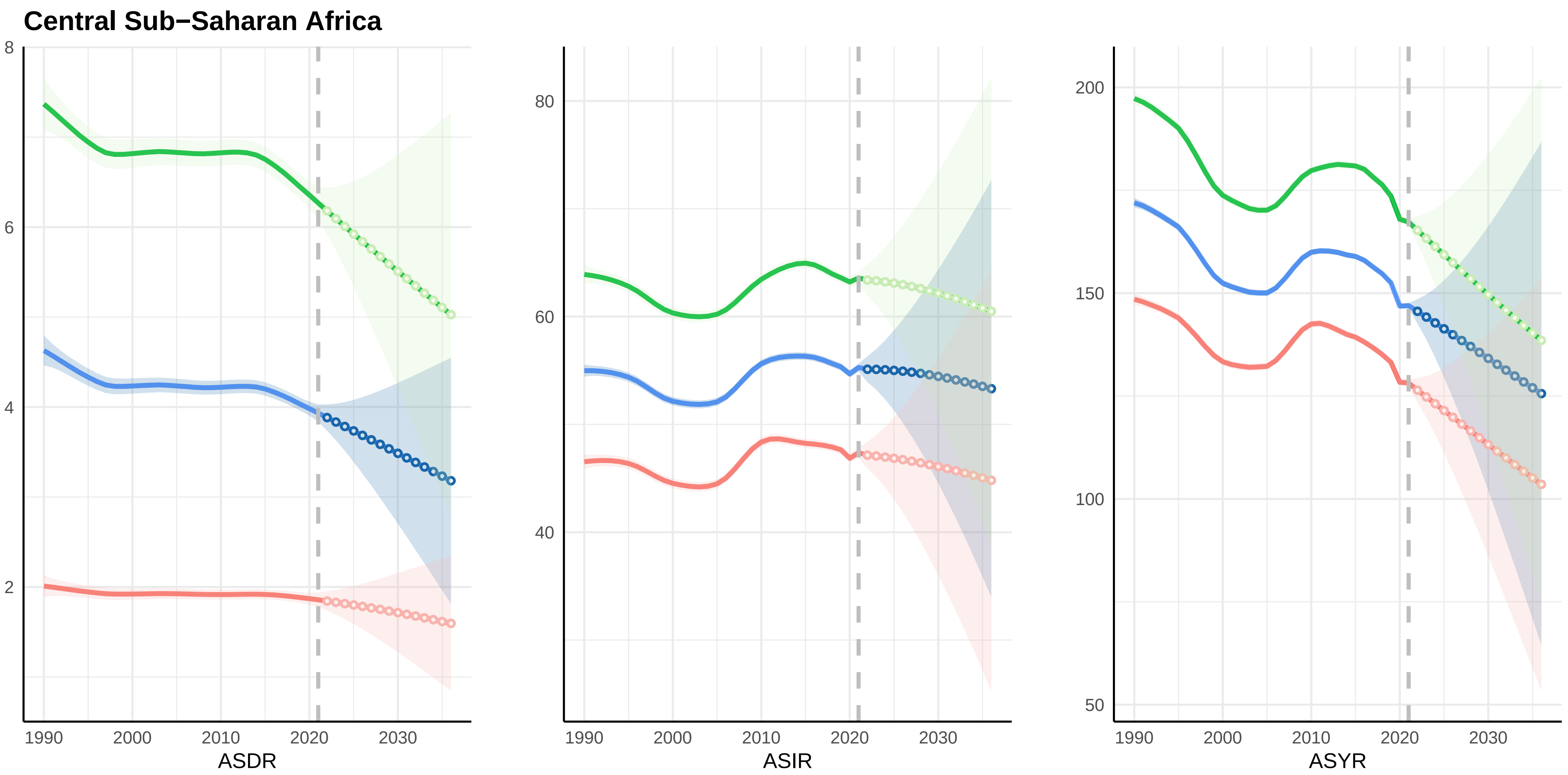

Central Sub−Saharan Africa
8
6
4
2
1990
2000
2010
2020
2030
ASDR
80
60
40
ASIR
200
150
100
50
ASYR

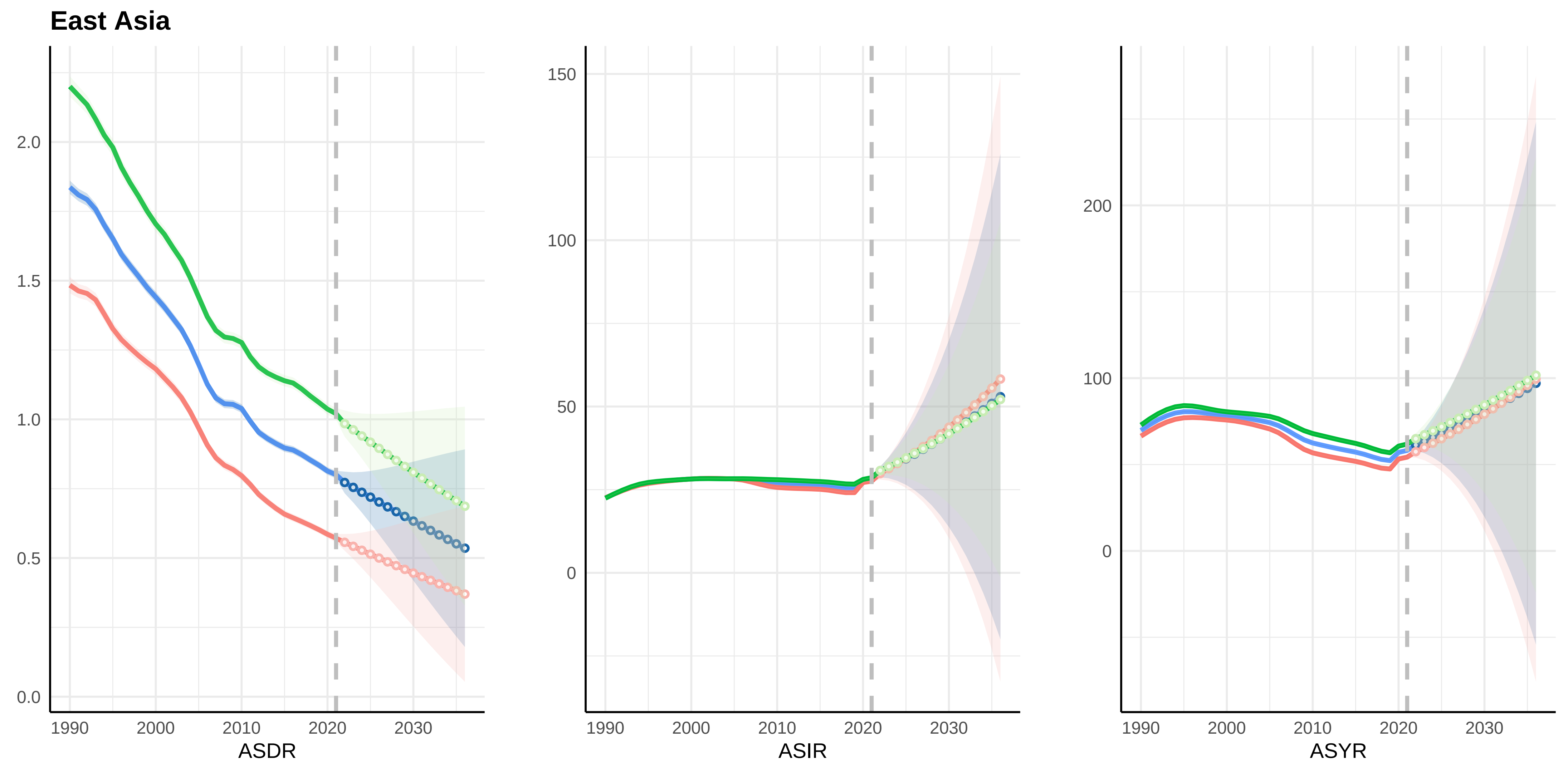

East Asia
2.0
1.5
1.0
0.5
0.0
1990
2000
2010
2020
2030
ASDR
150
100
50
0
1990
2000
2010
2020
2030
ASIR
200
100
0
1990
2000
2010
2020
2030
ASYR

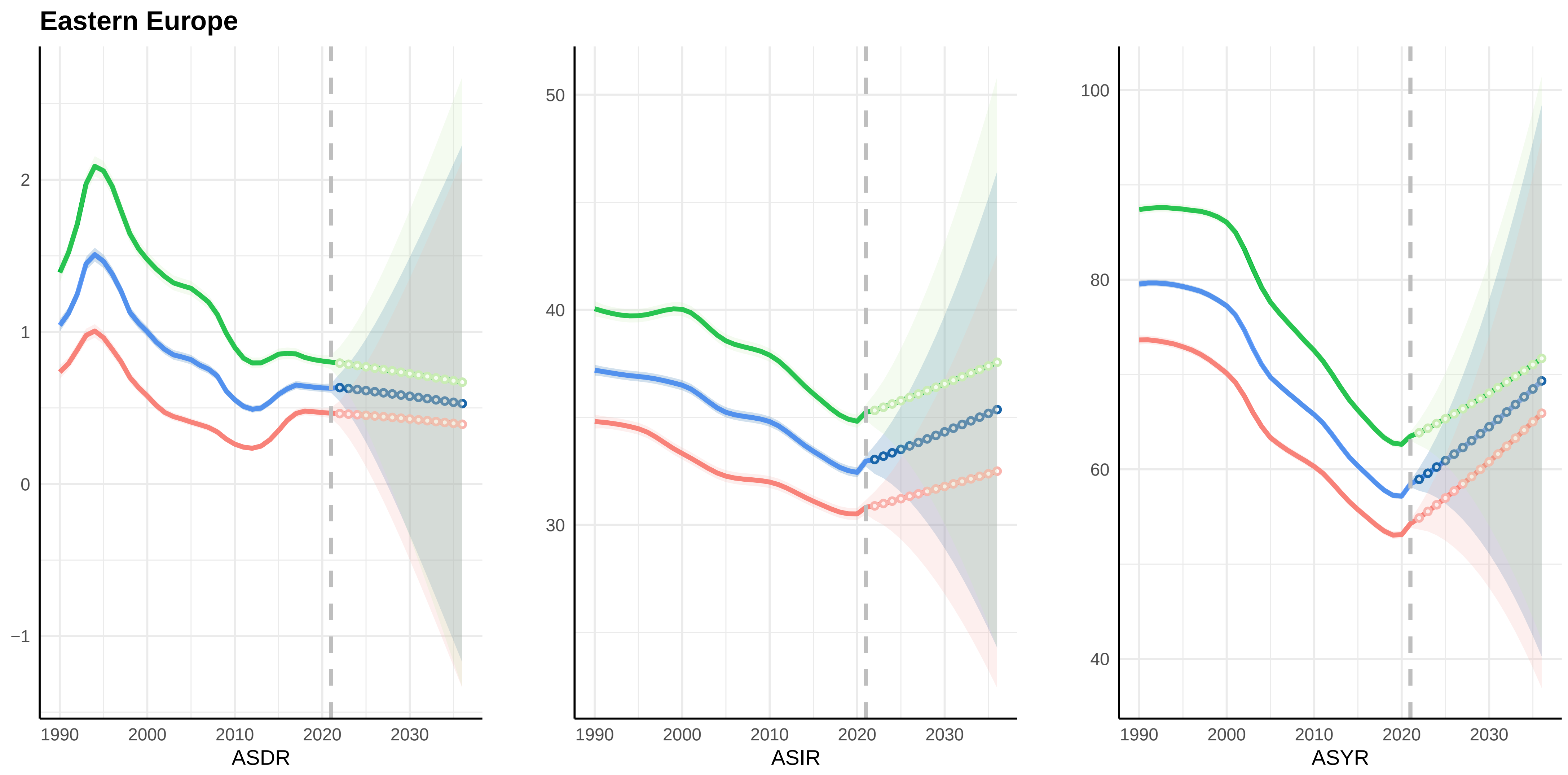
Eastern Europe
2
1
0
−1
1990
2000
2010
2020
2030
ASDR
50
40
30
1990
2000
2010
2020
2030
ASIR
100
80
60
40
1990
2000
2010
2020
2030
ASYR

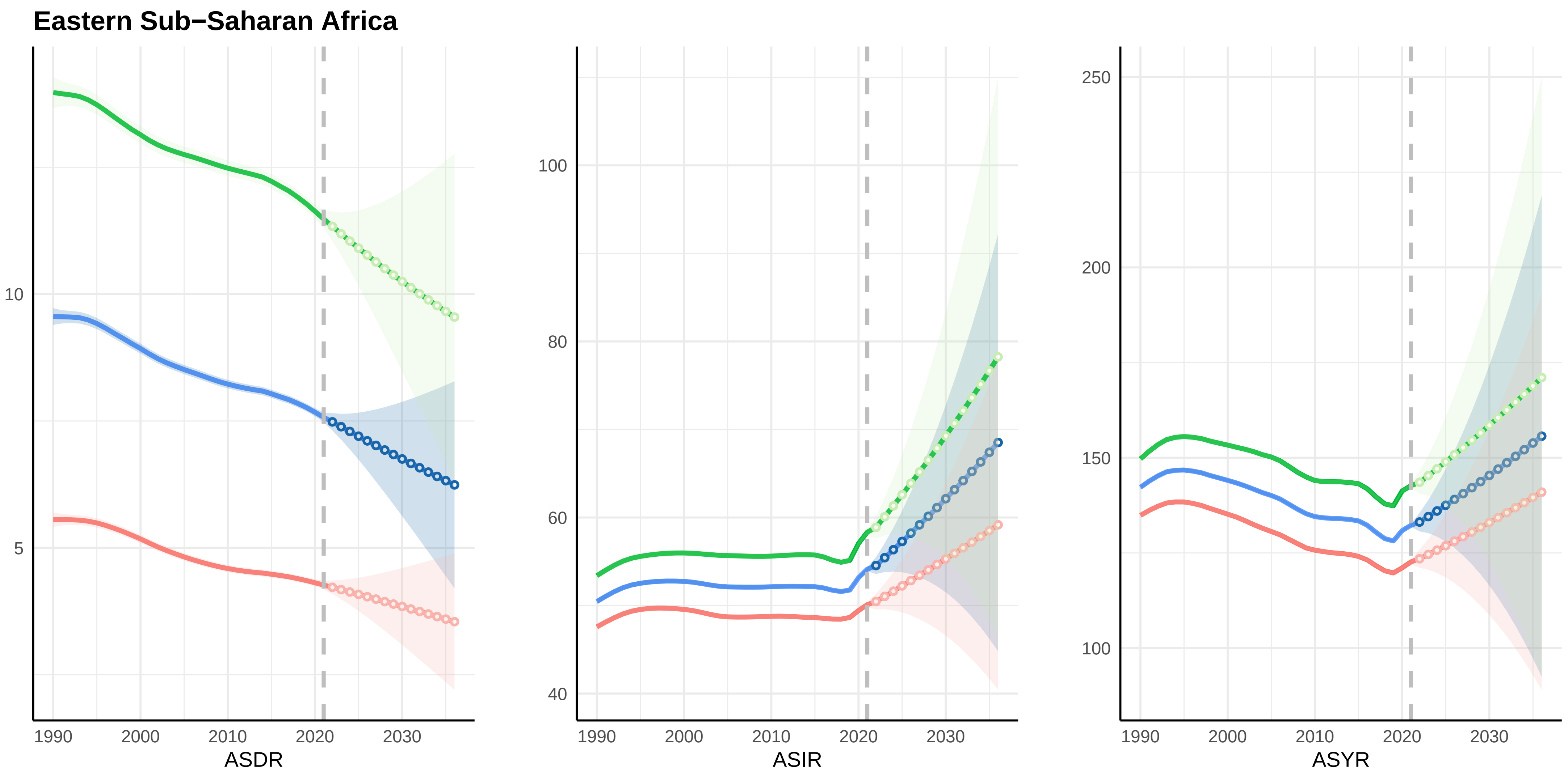

Eastern Sub−Saharan Africa
10
5
1990
2000
2010
2020
2030
ASDR
100
80
60
40
ASIR
250
200
150
100
ASYR

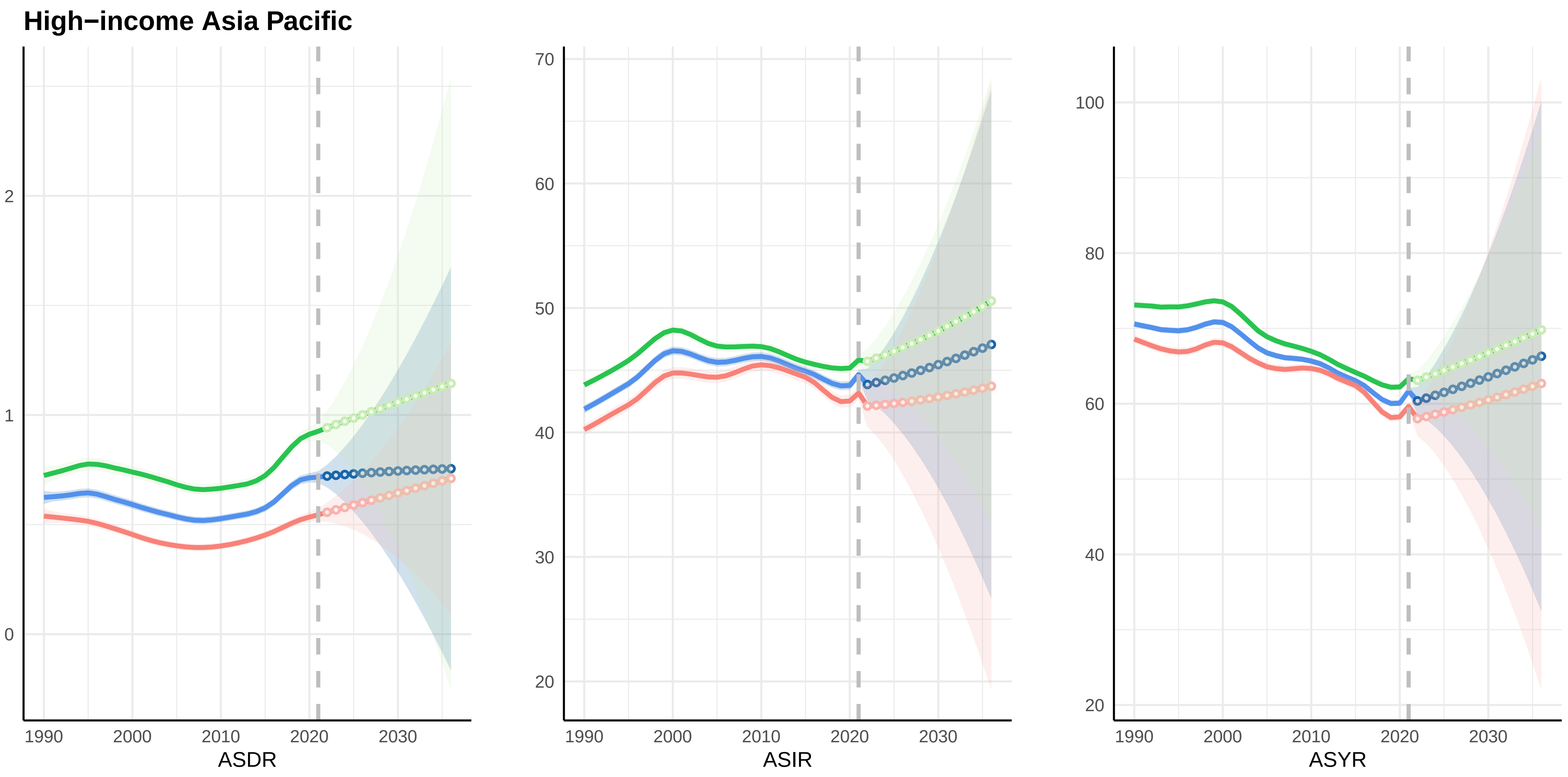

High−income Asia Pacific
2
1
0
1990
2000
2010
2020
2030
ASDR
70
60
50
40
30
20
1990
2000
2010
2020
2030
ASIR
100
80
60
40
20
1990
2000
2010
2020
2030
ASYR

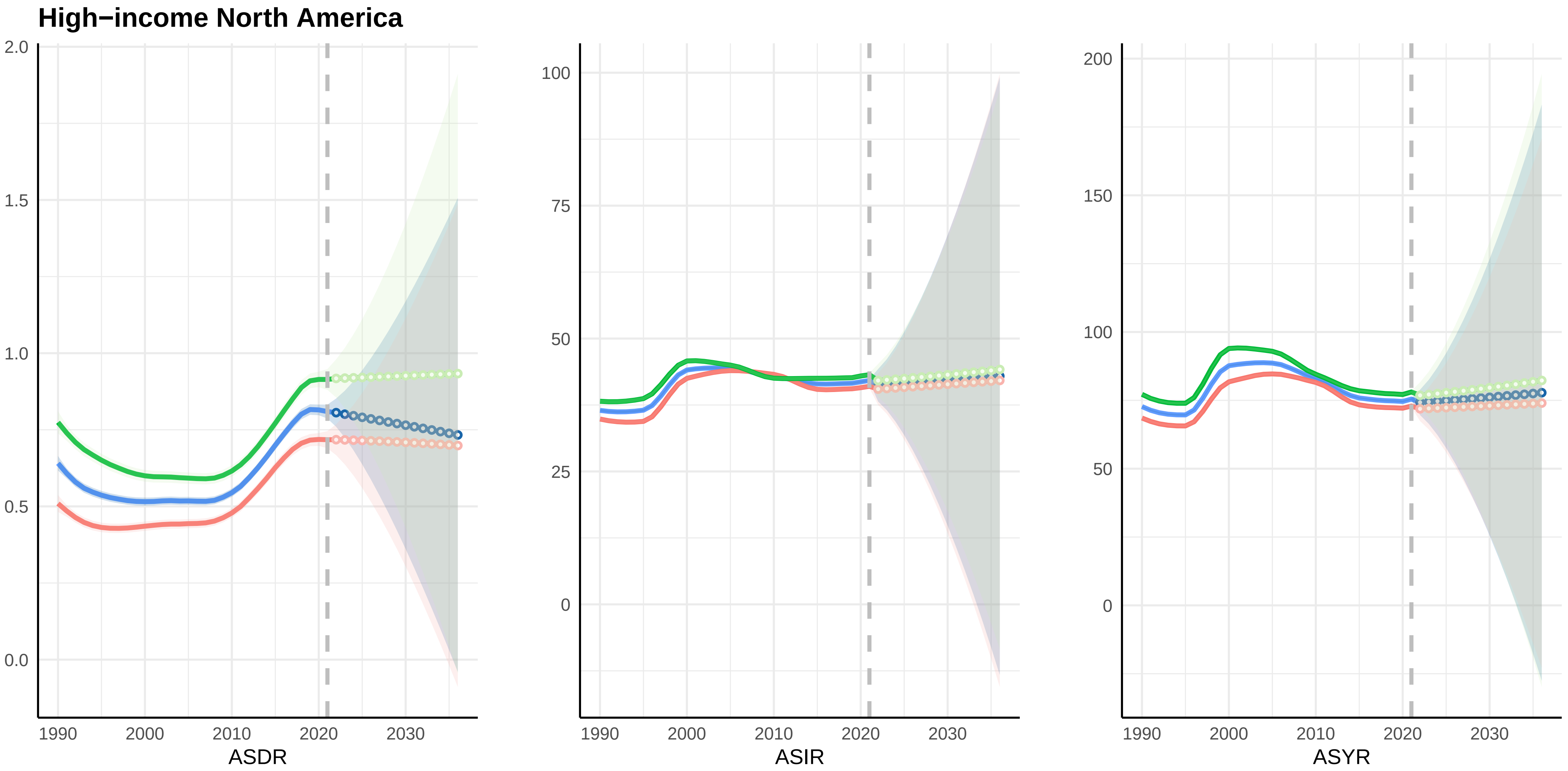

High−income North America
2.0
1.5
1.0
0.5
0.0
1990
2000
2010
2020
2030
ASDR
100
75
50
25
0
1990
2000
2010
2020
2030
ASIR
200
150
100
50
0
1990
2000
2010
2020
2030
ASYR

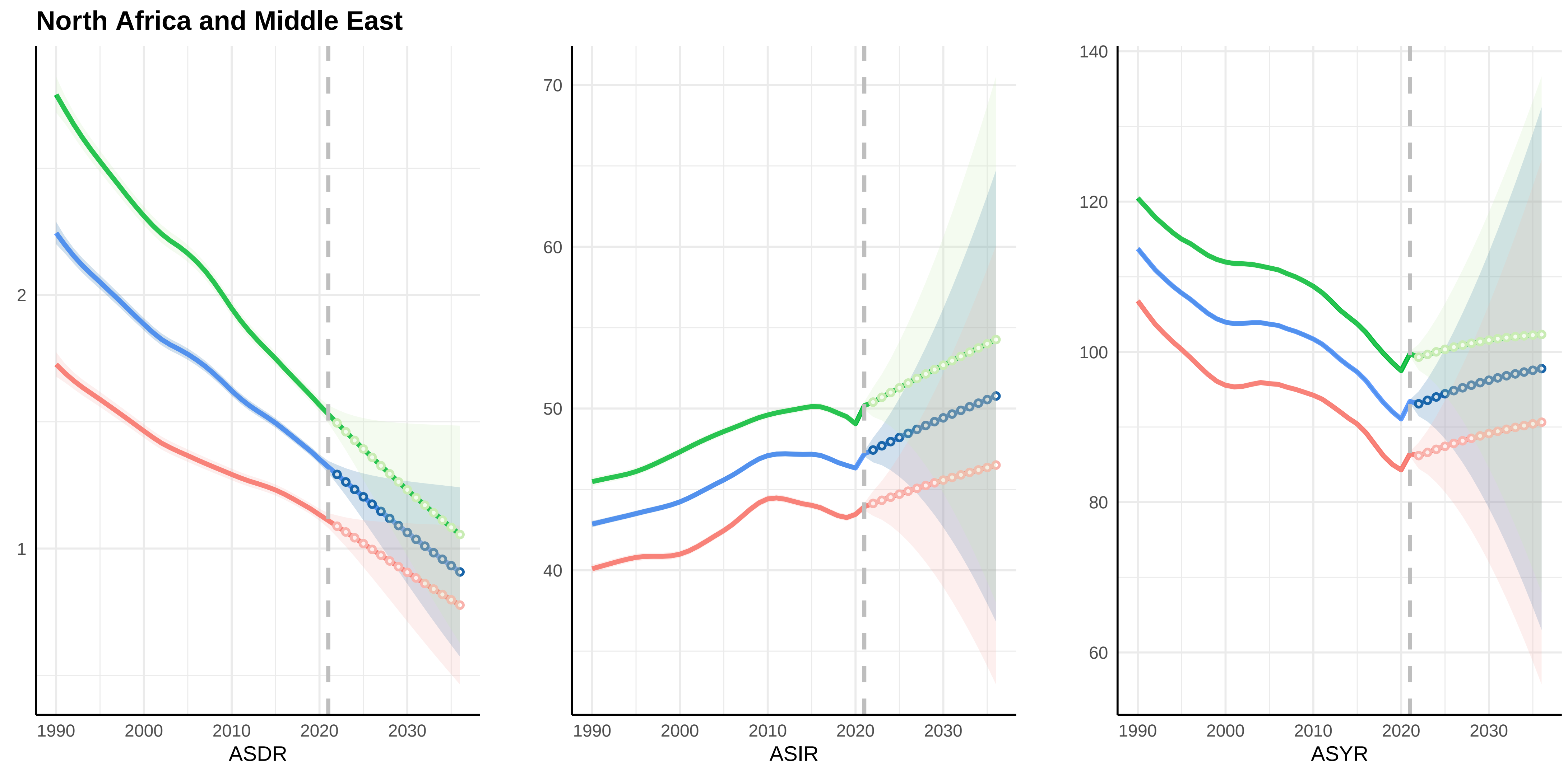

North Africa and Middle East
2
1
1990
2000
2010
2020
2030
ASDR
70
60
50
40
ASIR
140
120
100
80
60
ASYR

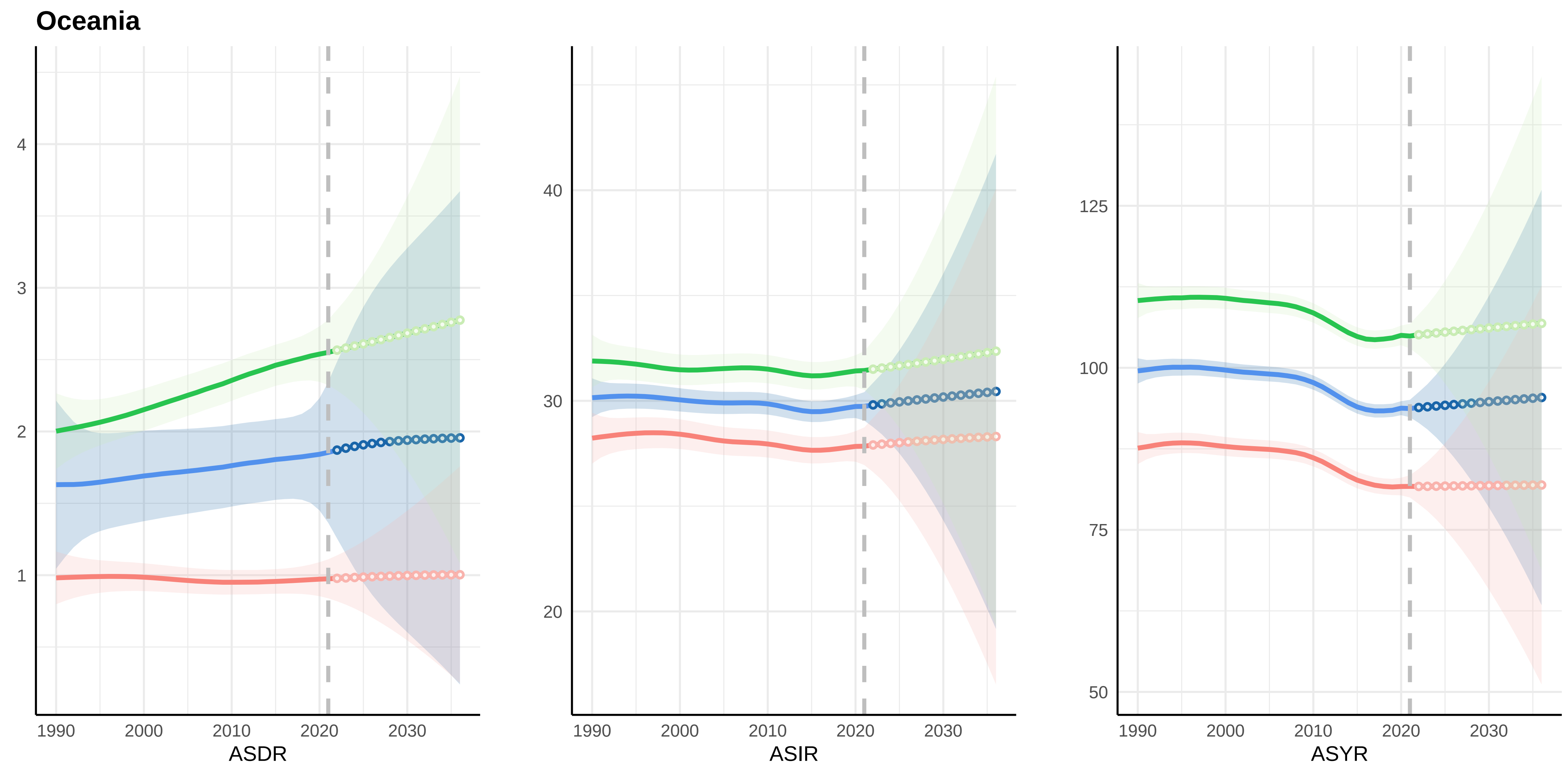

Oceania
4
3
2
1
1990
2000
2010
2020
2030
ASDR
40
30
20
1990
2000
2010
2020
2030
ASIR
125
100
75
50
1990
2000
2010
2020
2030
ASYR

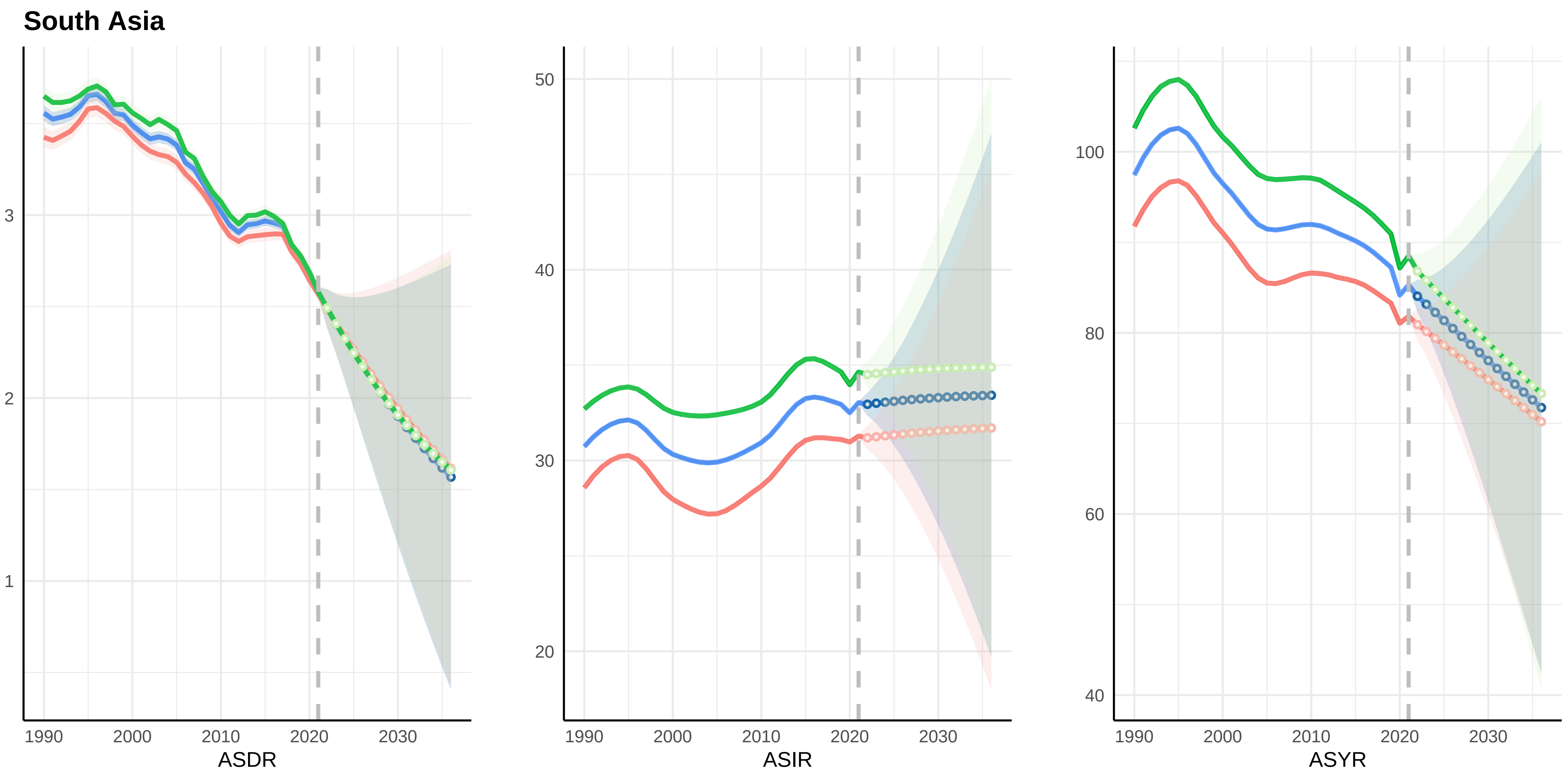
South Asia
3
2
1
1990
2000
2010
2020
2030
ASDR
50
40
30
20
1990
2000
2010
2020
2030
ASIR
100
80
60
40
1990
2000
2010
2020
2030
ASYR

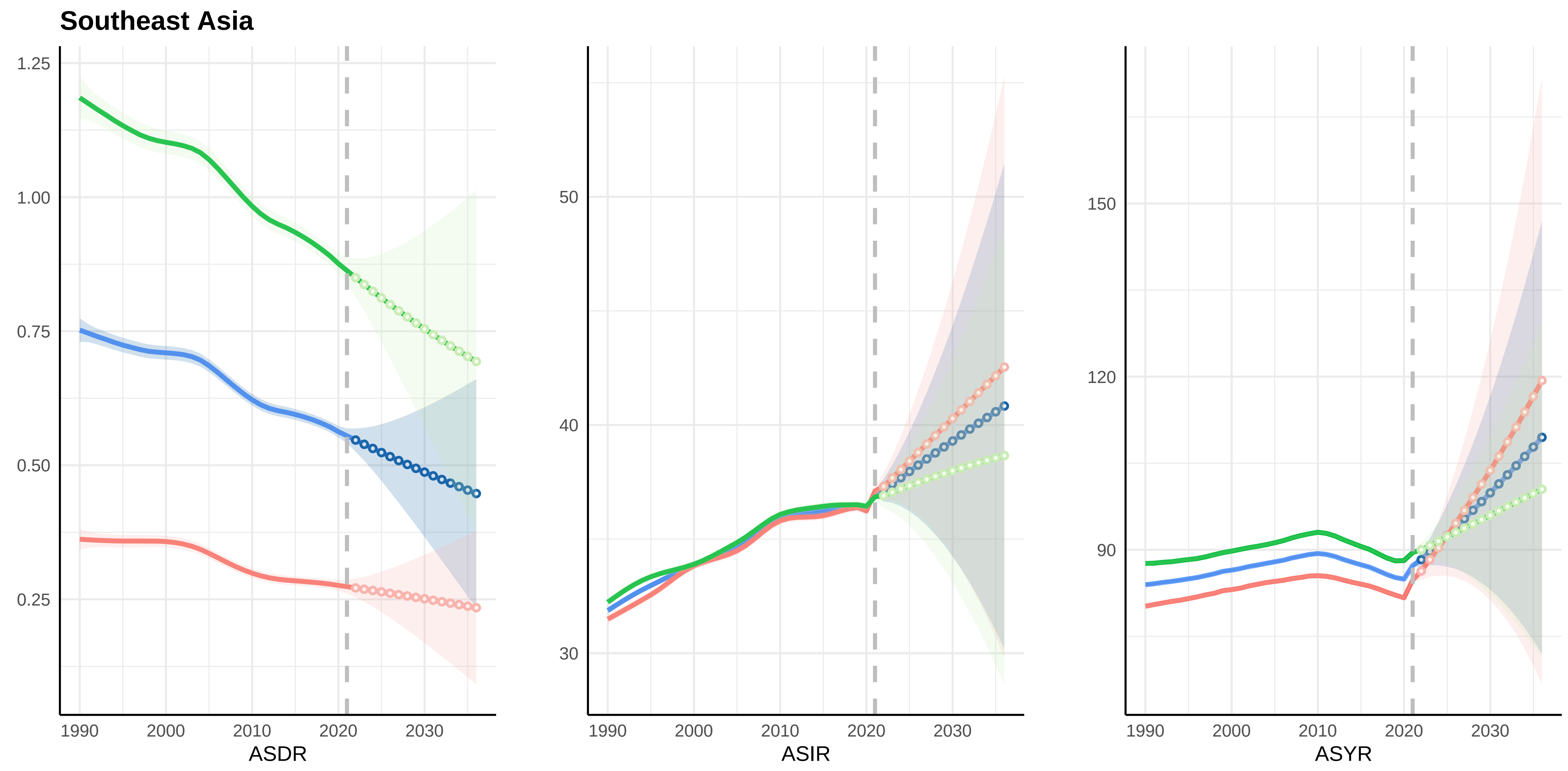

Southeast Asia
1.25
1.00
0.75
0.50
0.25
1990
2000
2010
2020
2030
ASDR
50
40
30
1990
2000
2010
2020
2030
ASIR
150
120
90
1990
2000
2010
2020
2030
ASYR

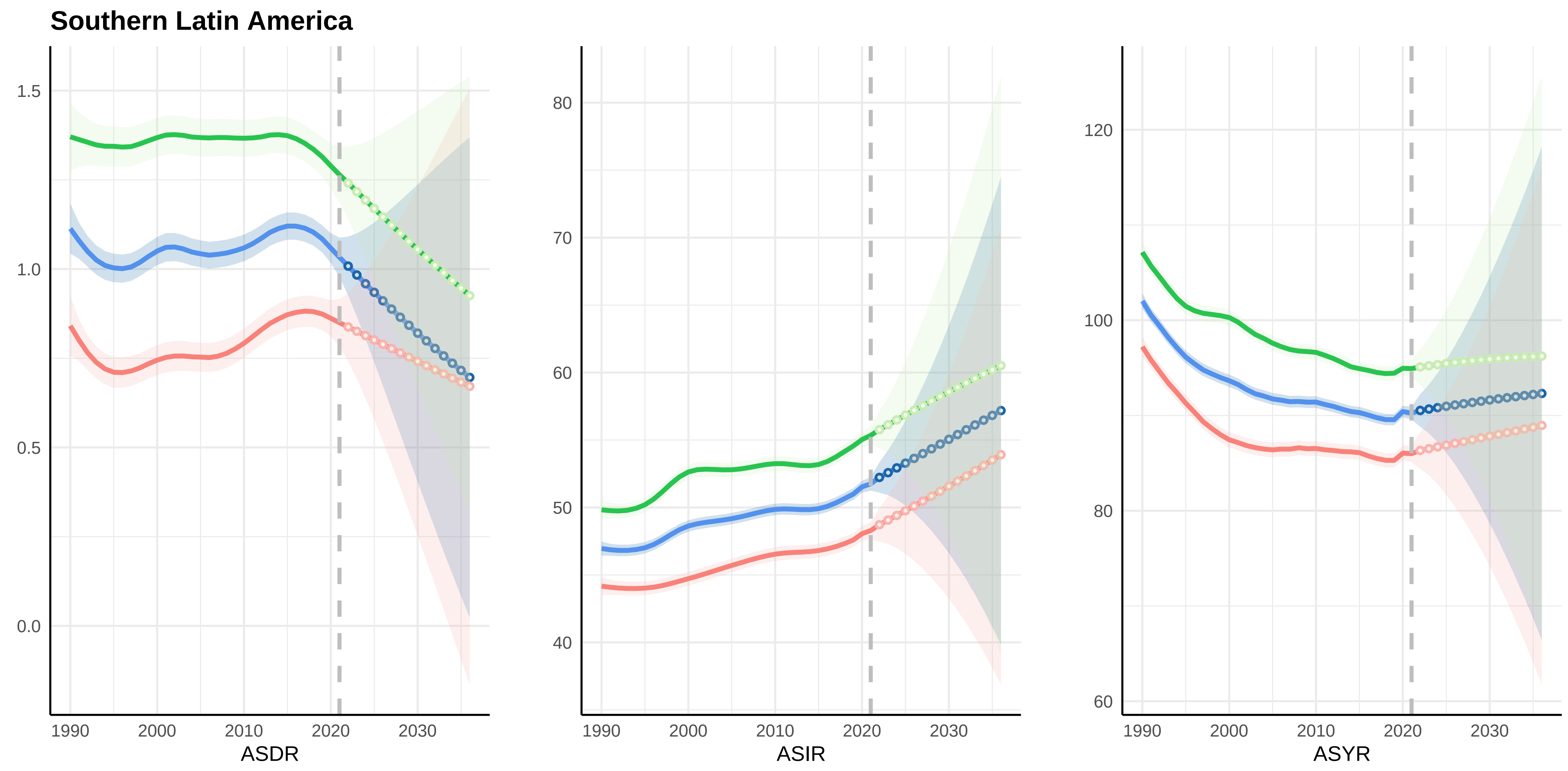

Southern Latin America
1.5
1.0
0.5
0.0
1990
2000
2010
2020
2030
ASDR
80
70
60
50
40
1990
2000
2010
2020
2030
ASIR
120
100
80
60
1990
2000
2010
2020
2030
ASYR

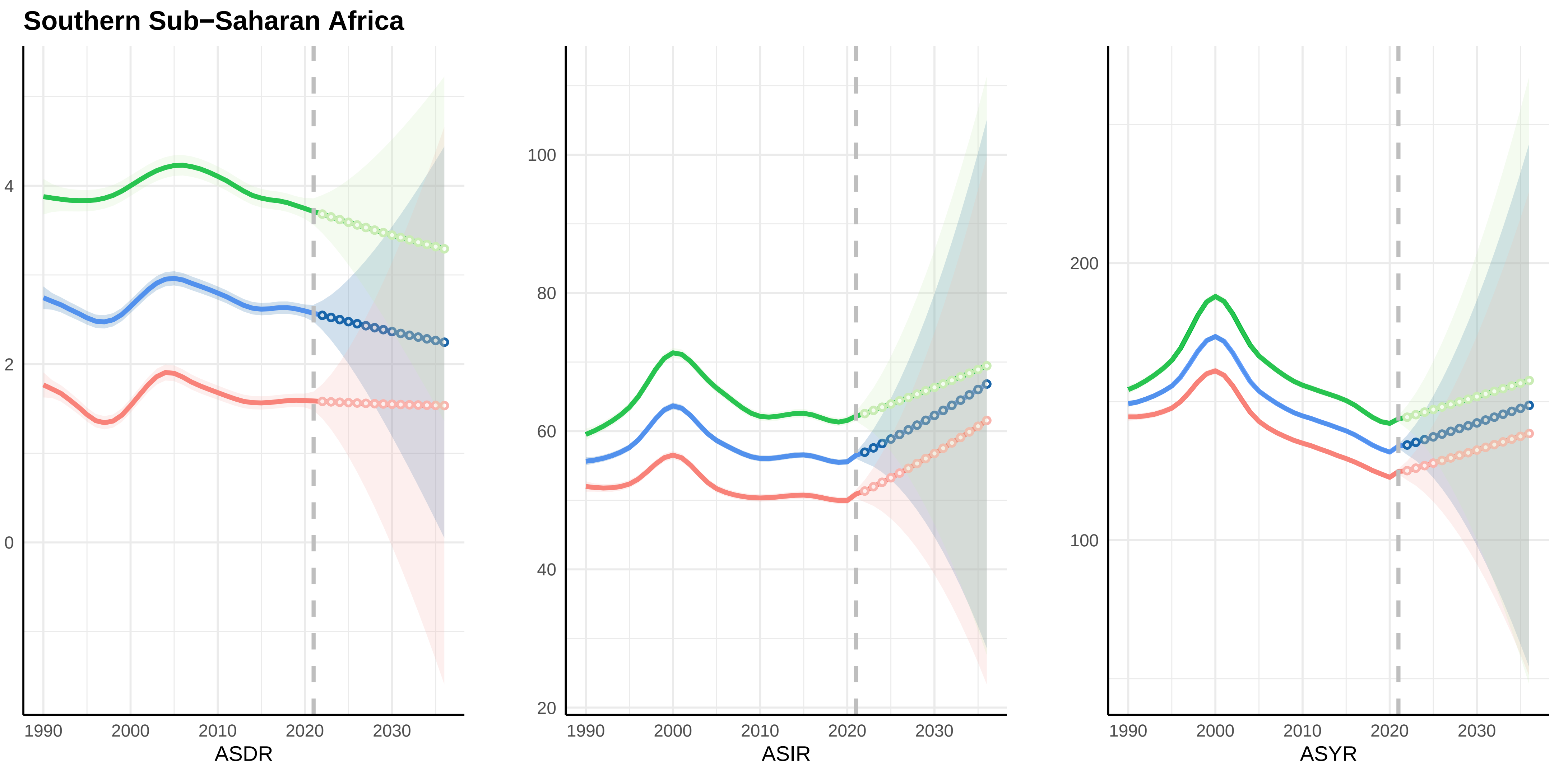

Southern Sub−Saharan Africa
4
2
0
1990
2000
2010
2020
2030
ASDR
100
80
60
40
20
1990
2000
2010
2020
2030
ASIR
200
100
1990
2000
2010
2020
2030
ASYR

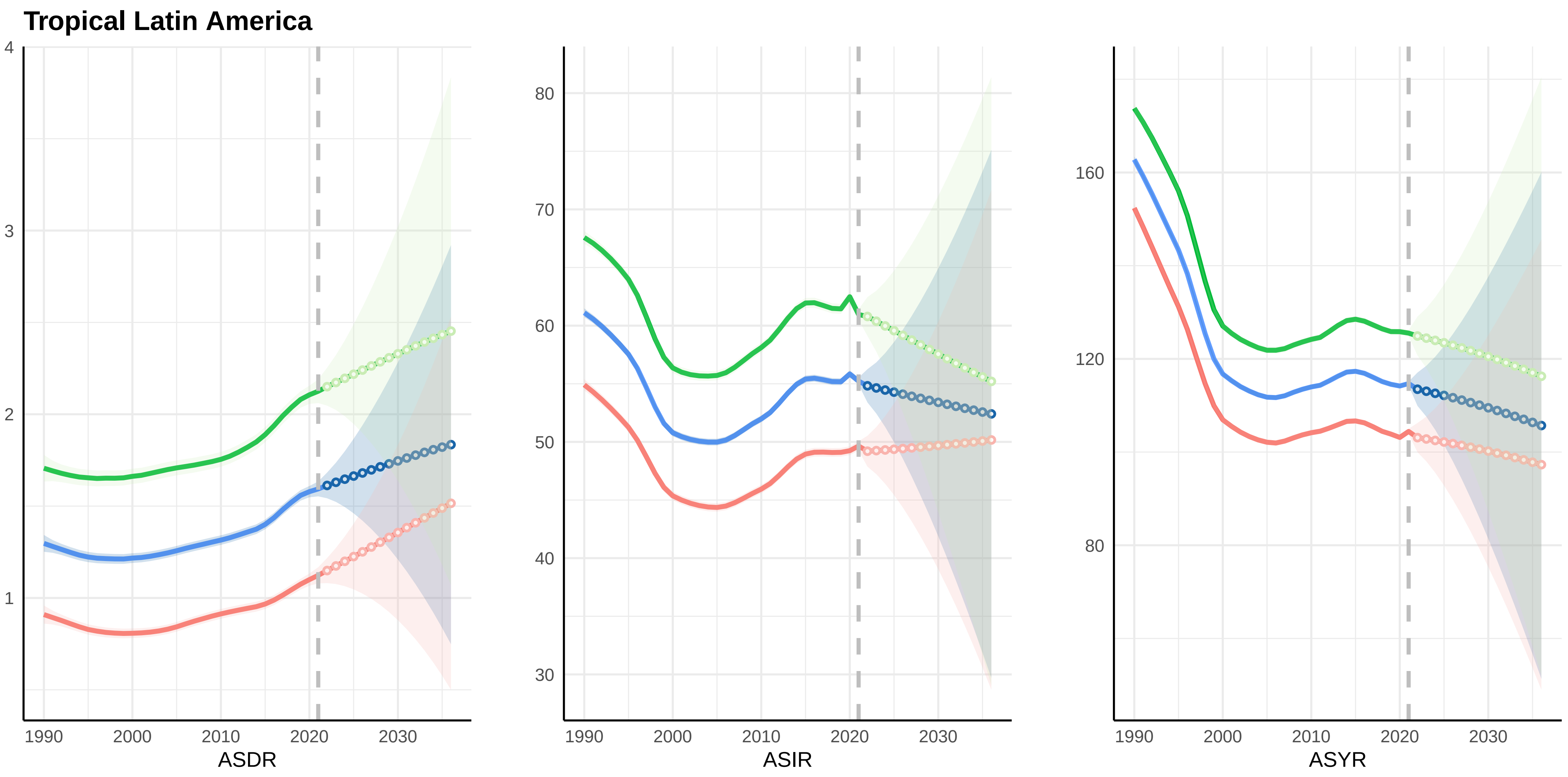
Tropical Latin America
ASDR
ASIR
ASYR
1990
2000
2010
2020
2030
1
2
3
4
30
40
50
60
70
80
120
160

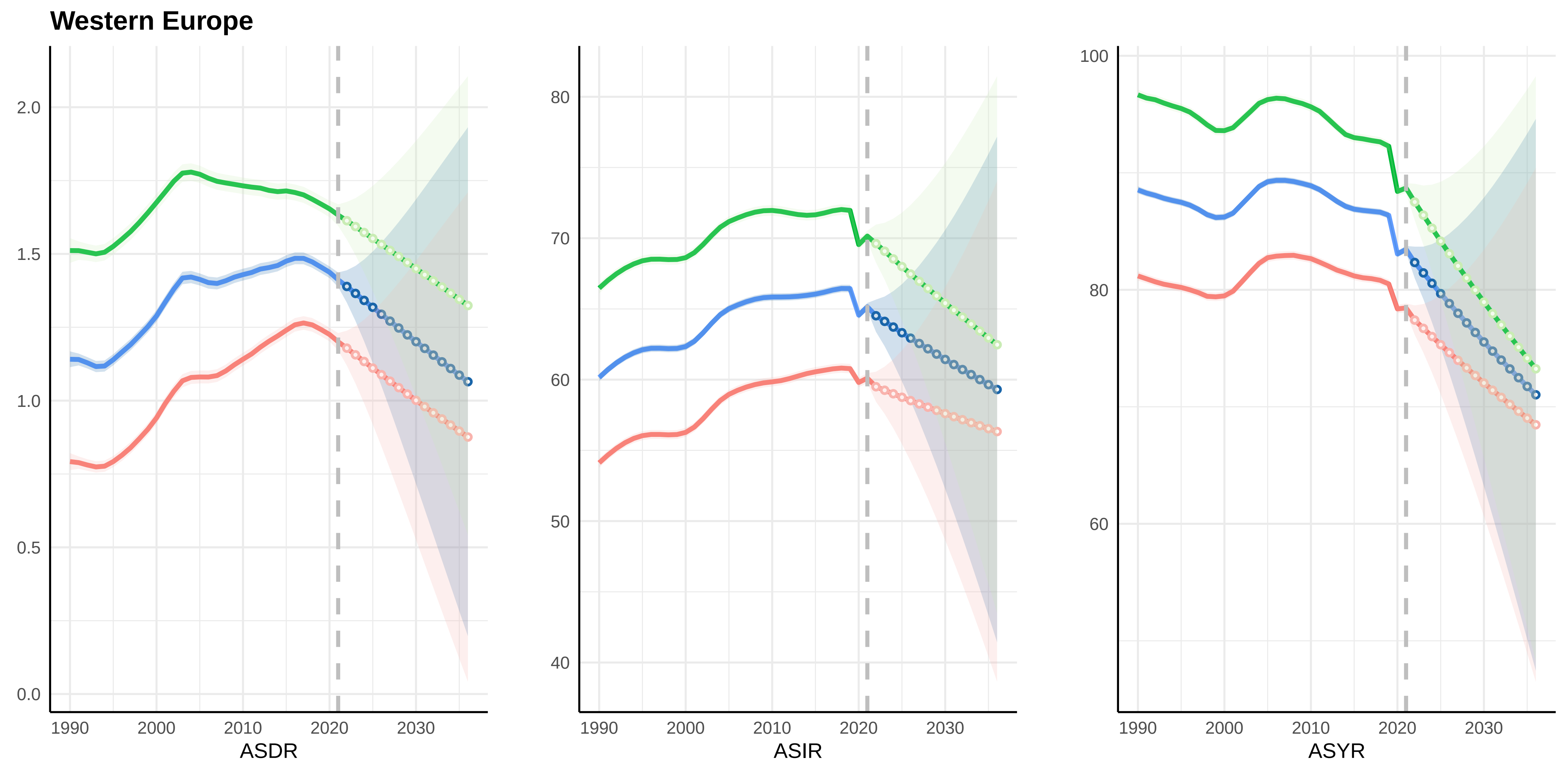

Western Europe
2.0
1.5
1.0
0.5
0.0
1990
2000
2010
2020
2030
ASDR
80
70
60
50
40
1990
2000
2010
2020
2030
ASIR
100
80
60
1990
2000
2010
2020
2030
ASYR

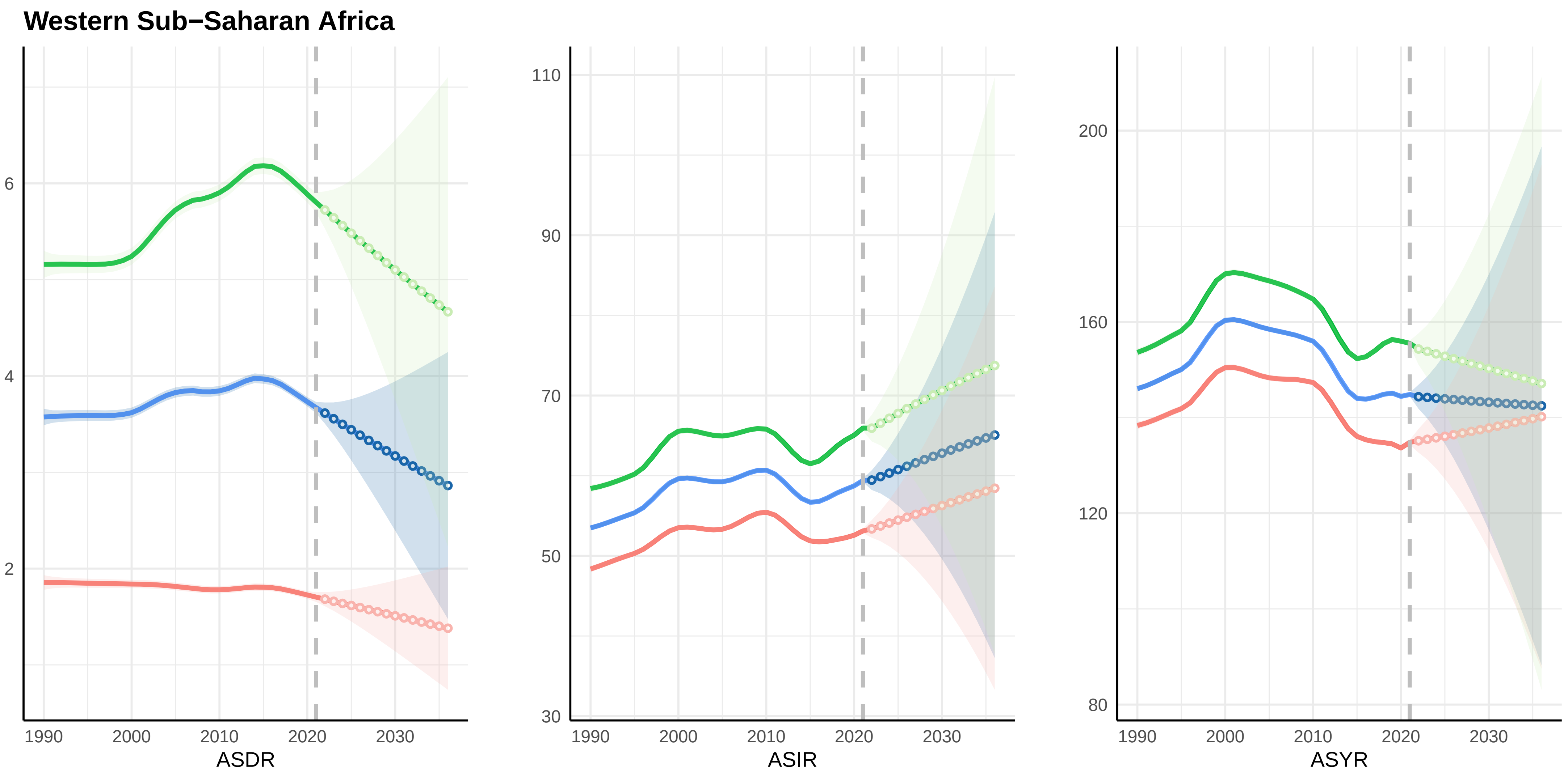
Western Sub−Saharan Africa
6
4
2
1990
2000
2010
2020
2030
ASDR
110
90
70
50
30
1990
2000
2010
2020
2030
ASIR
200
160
120
80
1990
2000
2010
2020
2030
ASYR
