# Supplementary material for: Global, regional, and national epilepsy of unknown cause incidence and mortality, 1990–2036: cross-national health inequalities and predictive analytics
Source: Front Neurol. 2025 Jun 30;16:1526984. doi: 10.3389/fneur.2025.1526984 (PMC12256229; doi:10.3389/fneur.2025.1526984)
Supplement: Supplementary file 3 [file Data_Sheet_3.pdf]

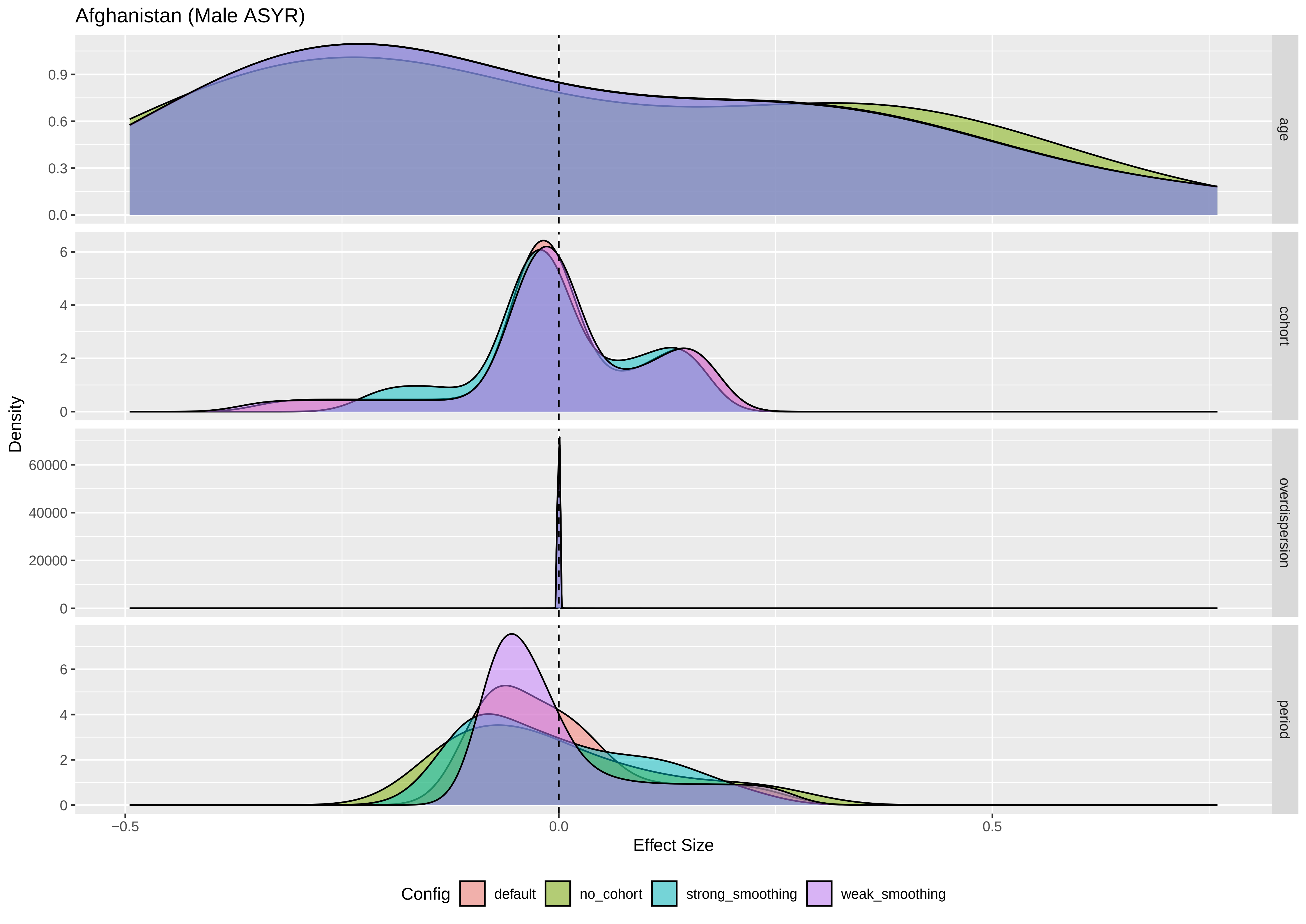

Afghanistan (Male ASYR)
Density
age
cohort
overdispersion
period
0.9
0.6
0.3
0.0
6
4
2
0
60000
40000
20000
0
−0.5
0.0
0.5
Effect Size
Config
default
no_cohort
strong_smoothing
weak_smoothing

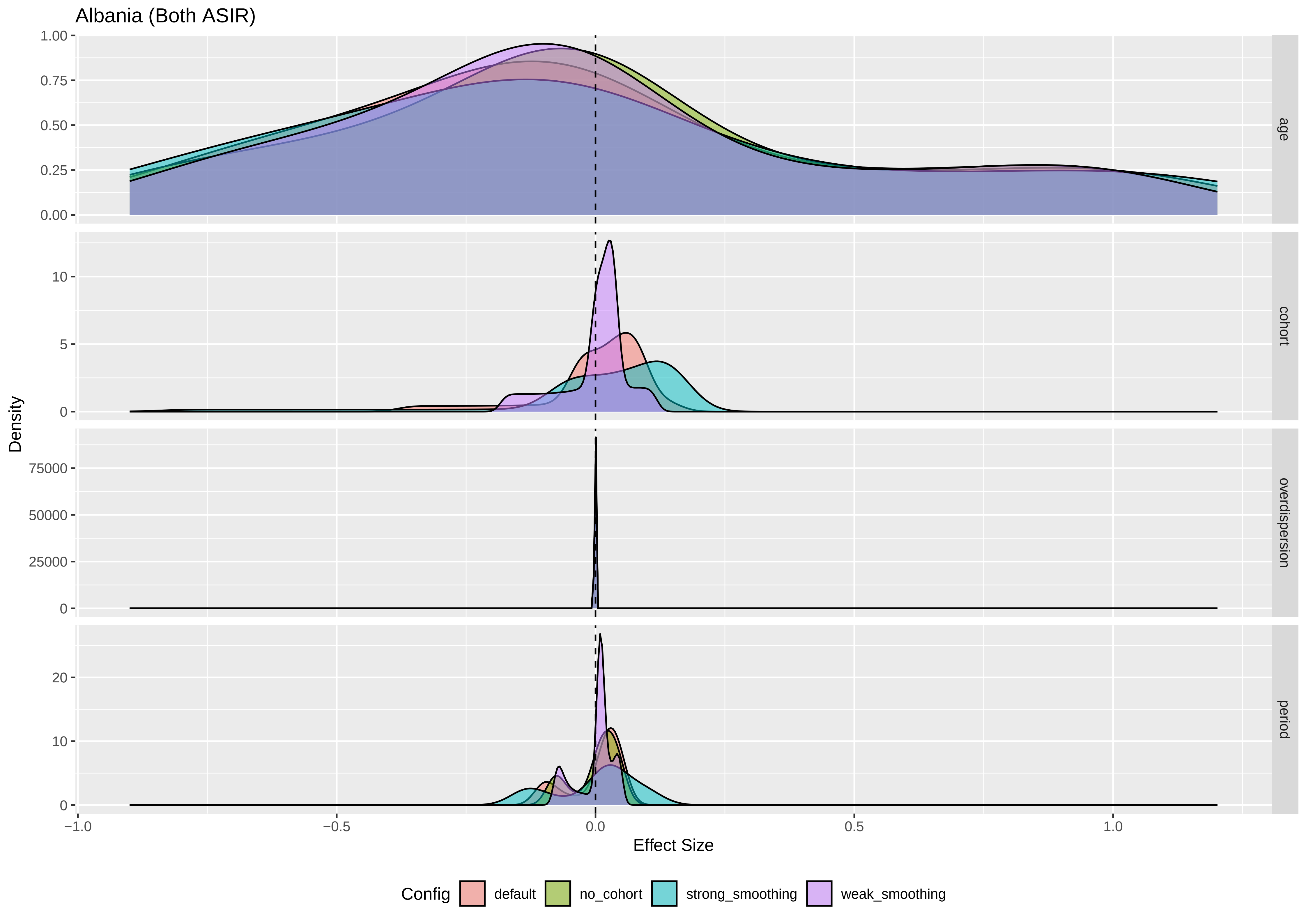

Albania (Both ASIR)
Density
Effect Size
age
cohort
overdispersion
period
1.00
0.75
0.50
0.25
0.00
10
5
0
75000
50000
25000
0
20
10
0
−1.0
−0.5
0.0
0.5
1.0
Config
default
no_cohort
strong_smoothing
weak_smoothing

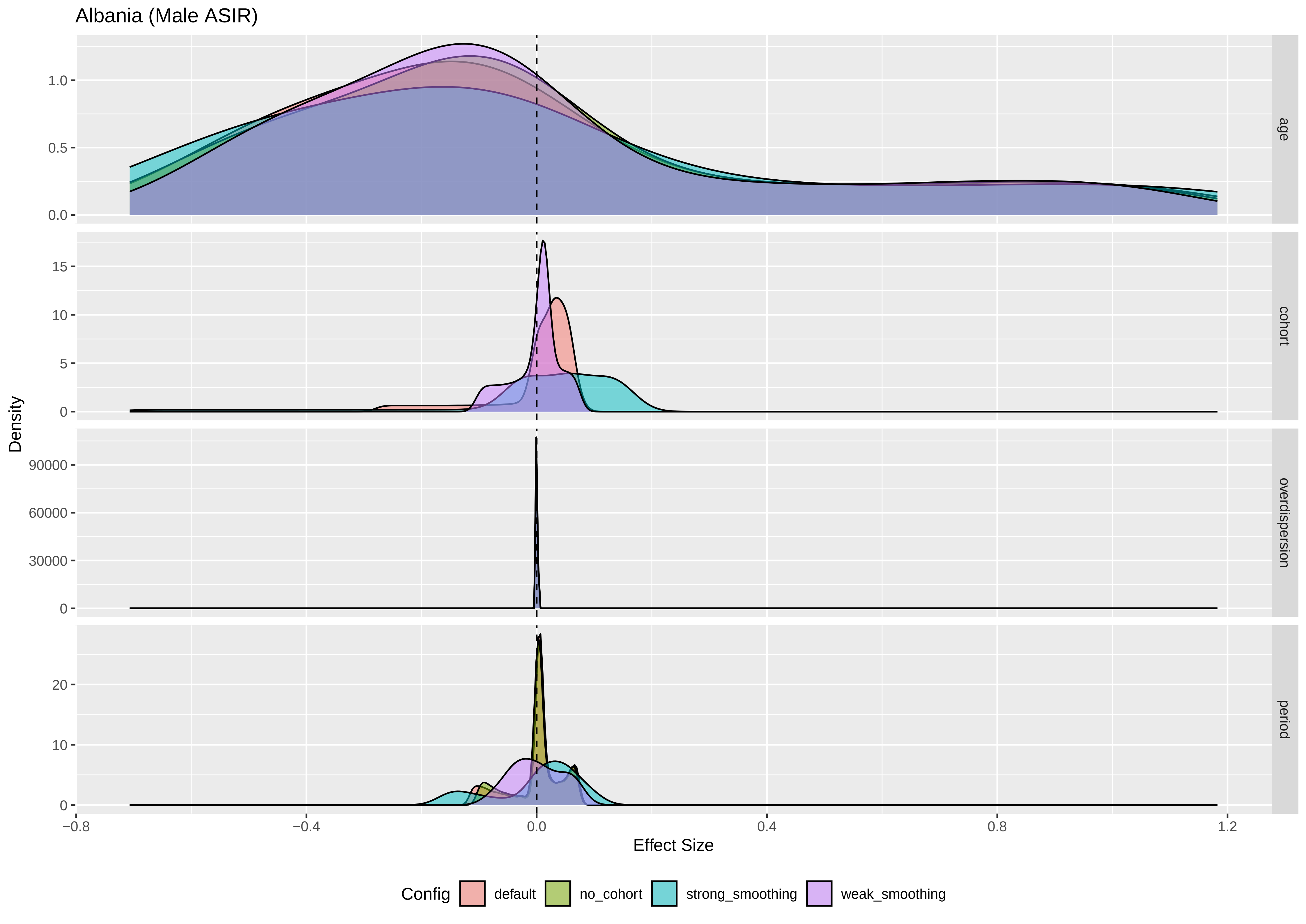

Albania (Male ASIR)
age
cohort
overdispersion
period
Density
Effect Size
-0.8
-0.4
0.0
0.4
0.8
1.2
0.0
0.5
1.0
0
5
10
15
30000
60000
90000
20
Config
default
no_cohort
strong_smoothing
weak_smoothing

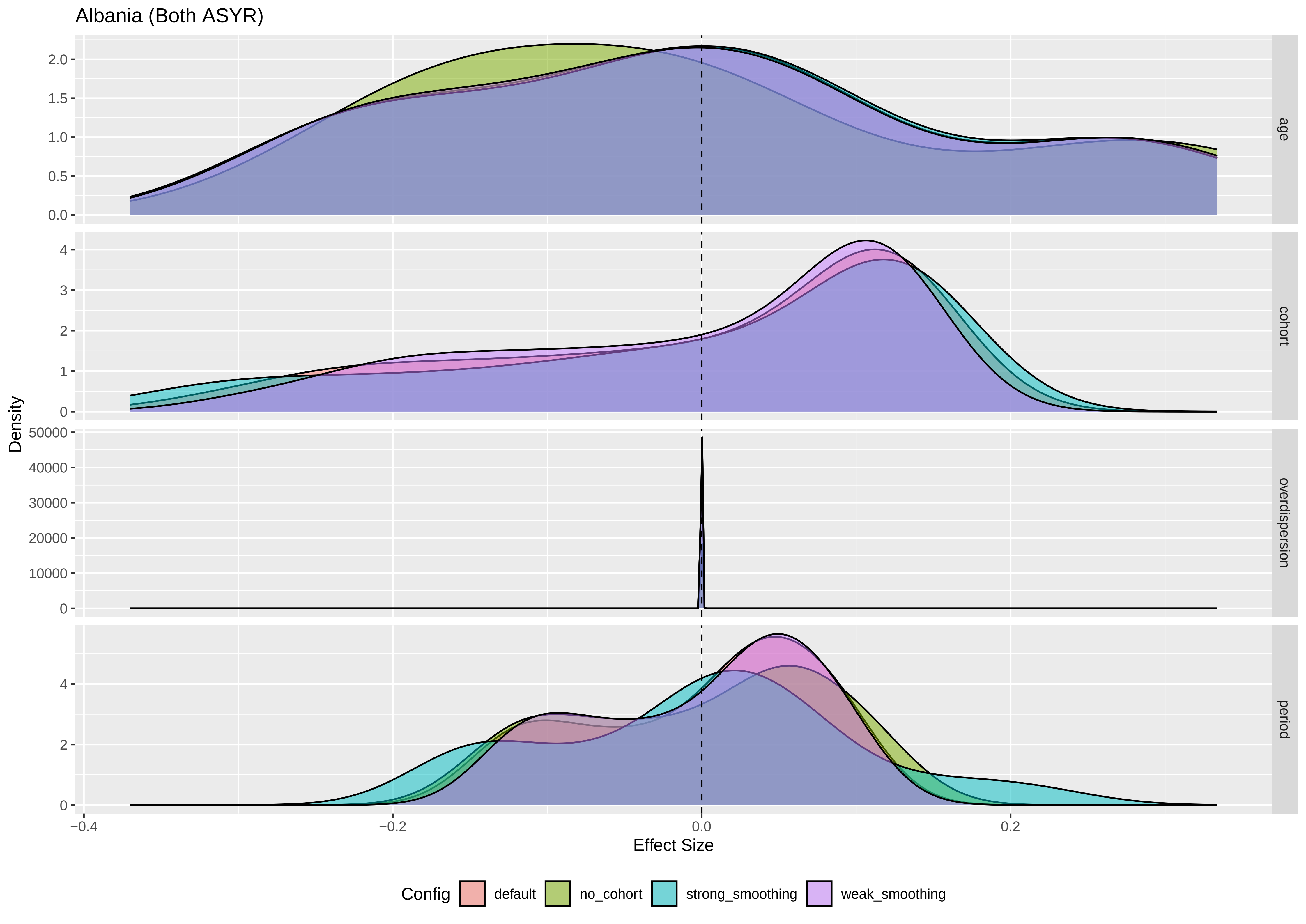

Albania (Both ASYR)
age
cohort
overdispersion
period
Density
Effect Size
2.0
1.5
1.0
0.5
0.0
4
3
2
1
0
50000
40000
30000
20000
10000
0
4
2
0
−0.4
−0.2
0.0
0.2
Config
default
no_cohort
strong_smoothing
weak_smoothing

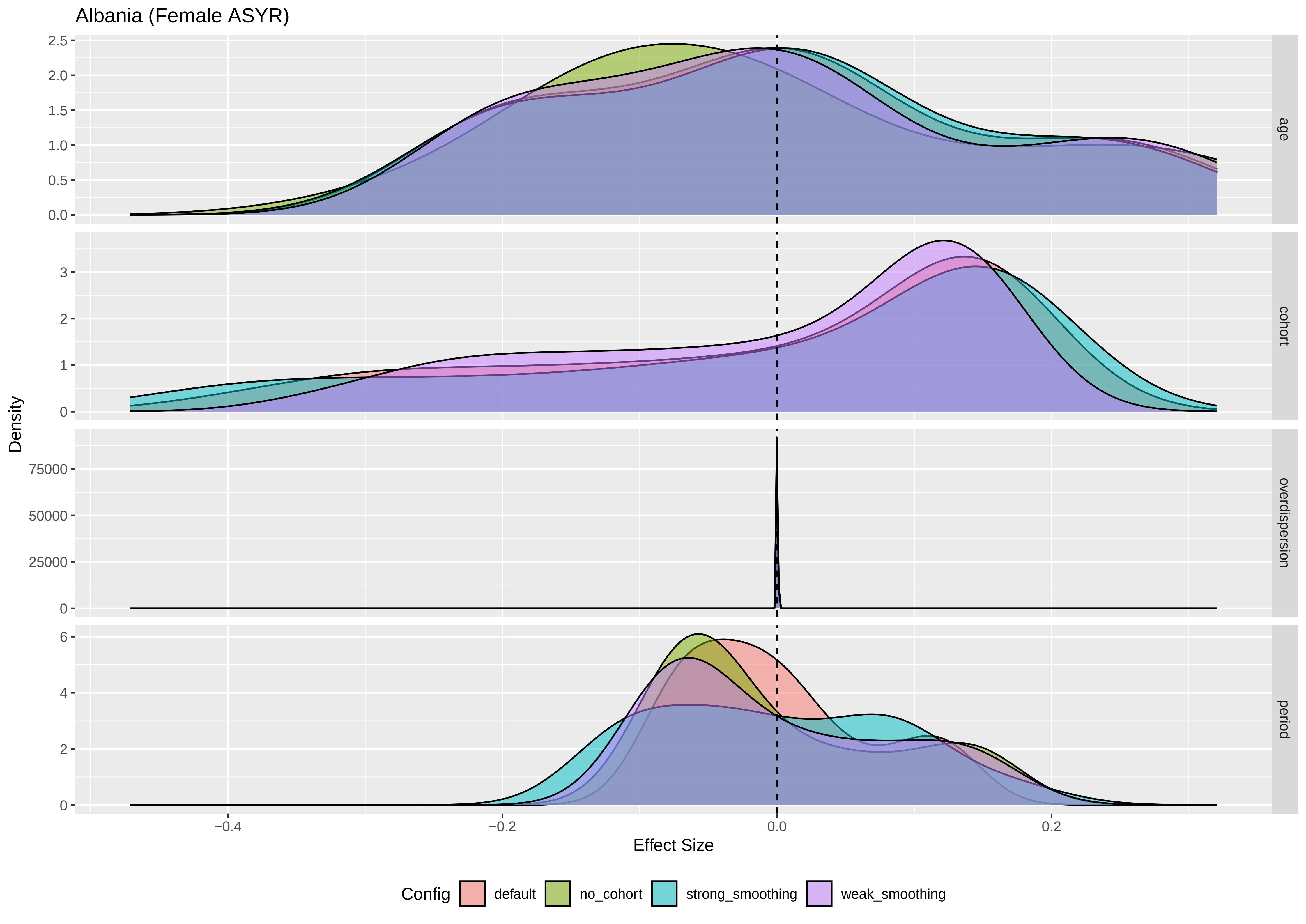

Albania (Female ASYR)
Density
Effect Size
age
cohort
overdispersion
period
2.5
2.0
1.5
1.0
0.5
0.0
3
2
1
0
75000
50000
25000
0
6
4
2
0
−0.4
−0.2
0.0
0.2
Config
default
no_cohort
strong_smoothing
weak_smoothing

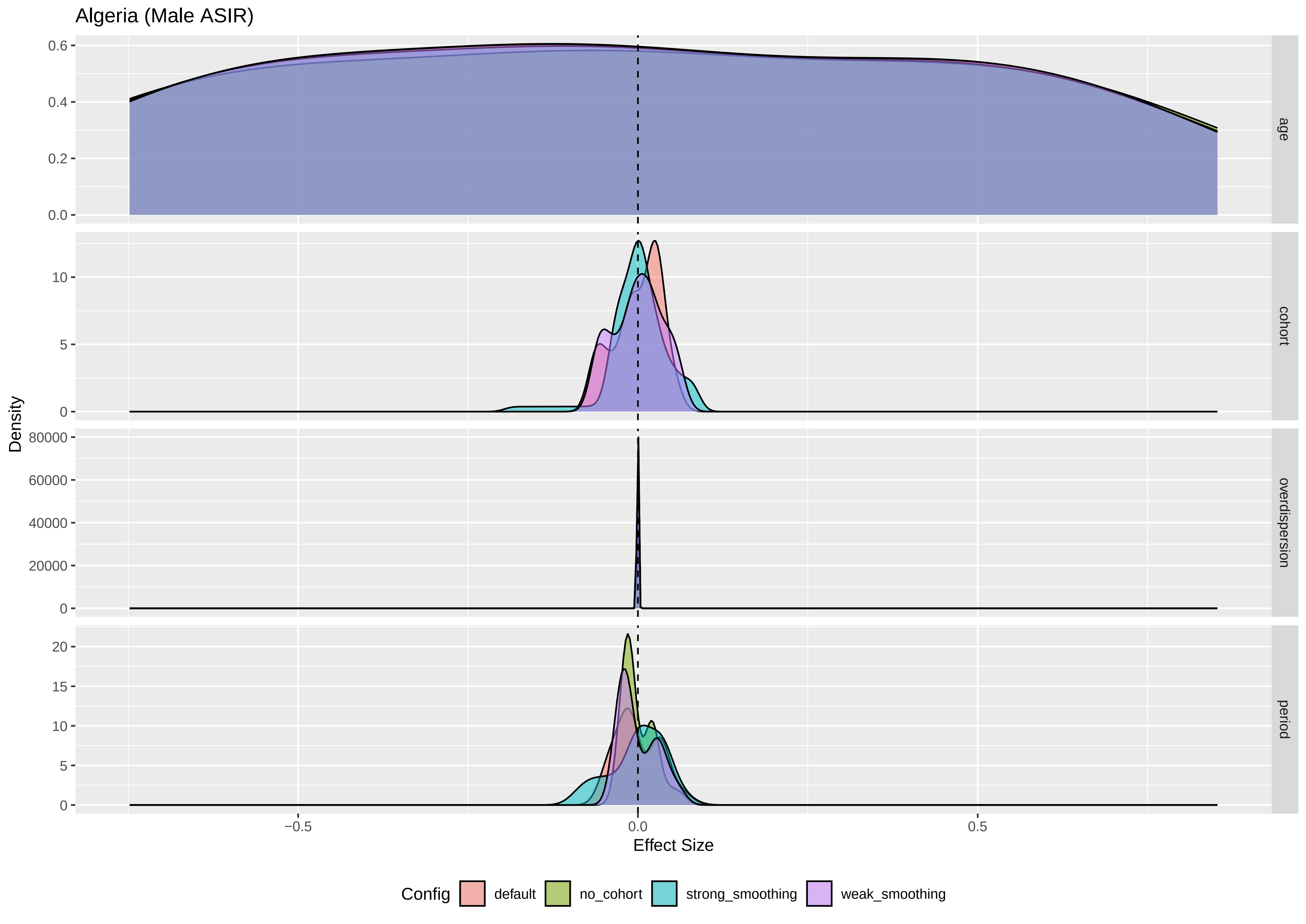

Algeria (Male ASIR)
0.6
0.4
0.2
0.0
10
5
0
80000
60000
40000
20000
0
20
15
10
5
0
Density
age
cohort
overdispersion
period
−0.5
0.0
0.5
Effect Size
Config
default
no_cohort
strong_smoothing
weak_smoothing

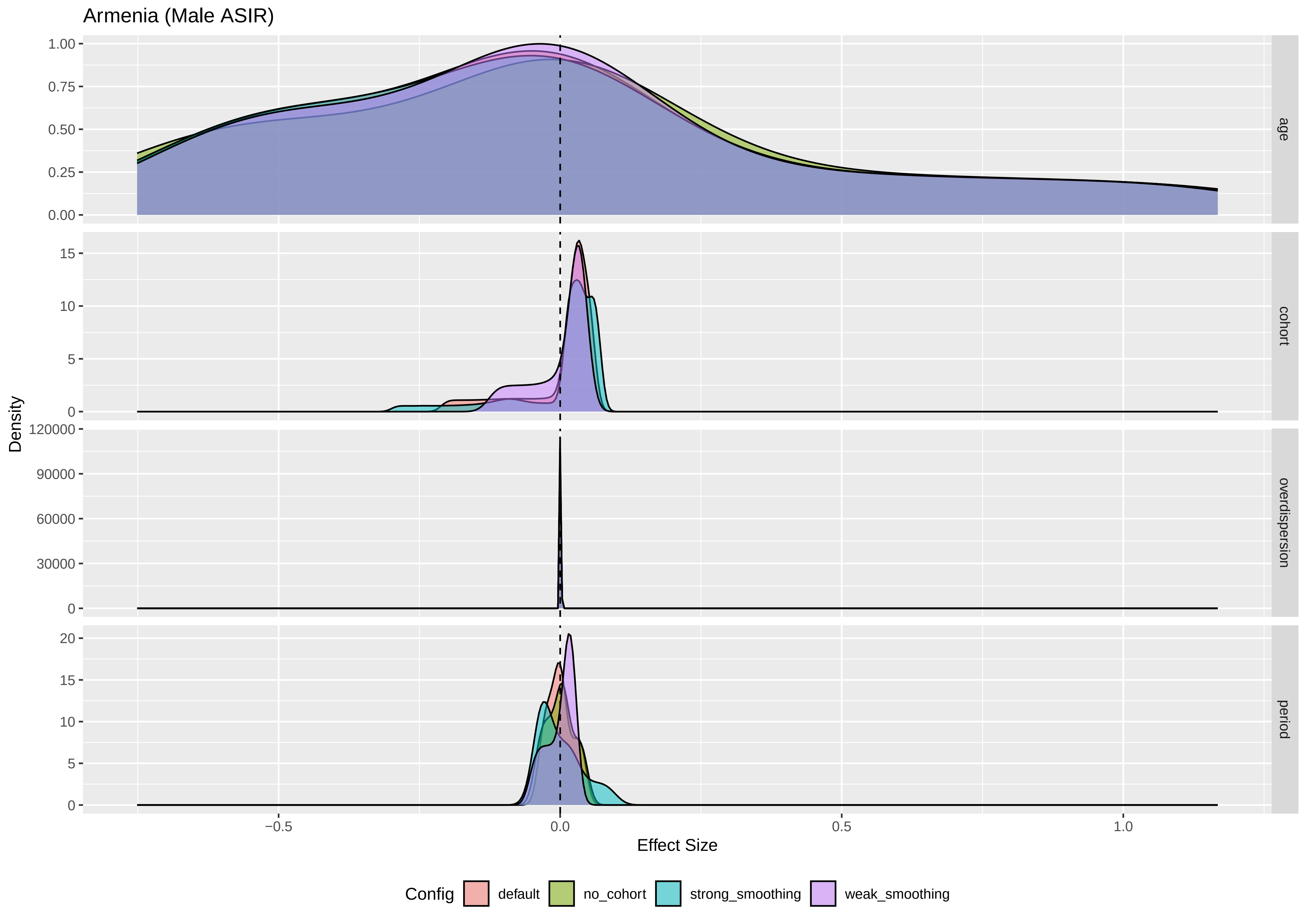

Armenia (Male ASIR)
1.00
0.75
0.50
0.25
0.00
15
10
5
0
120000
90000
60000
30000
0
20
15
10
5
0
age
cohort
overdispersion
period
Density
−0.5
0.0
0.5
1.0
Effect Size
Config
default
no_cohort
strong_smoothing
weak_smoothing

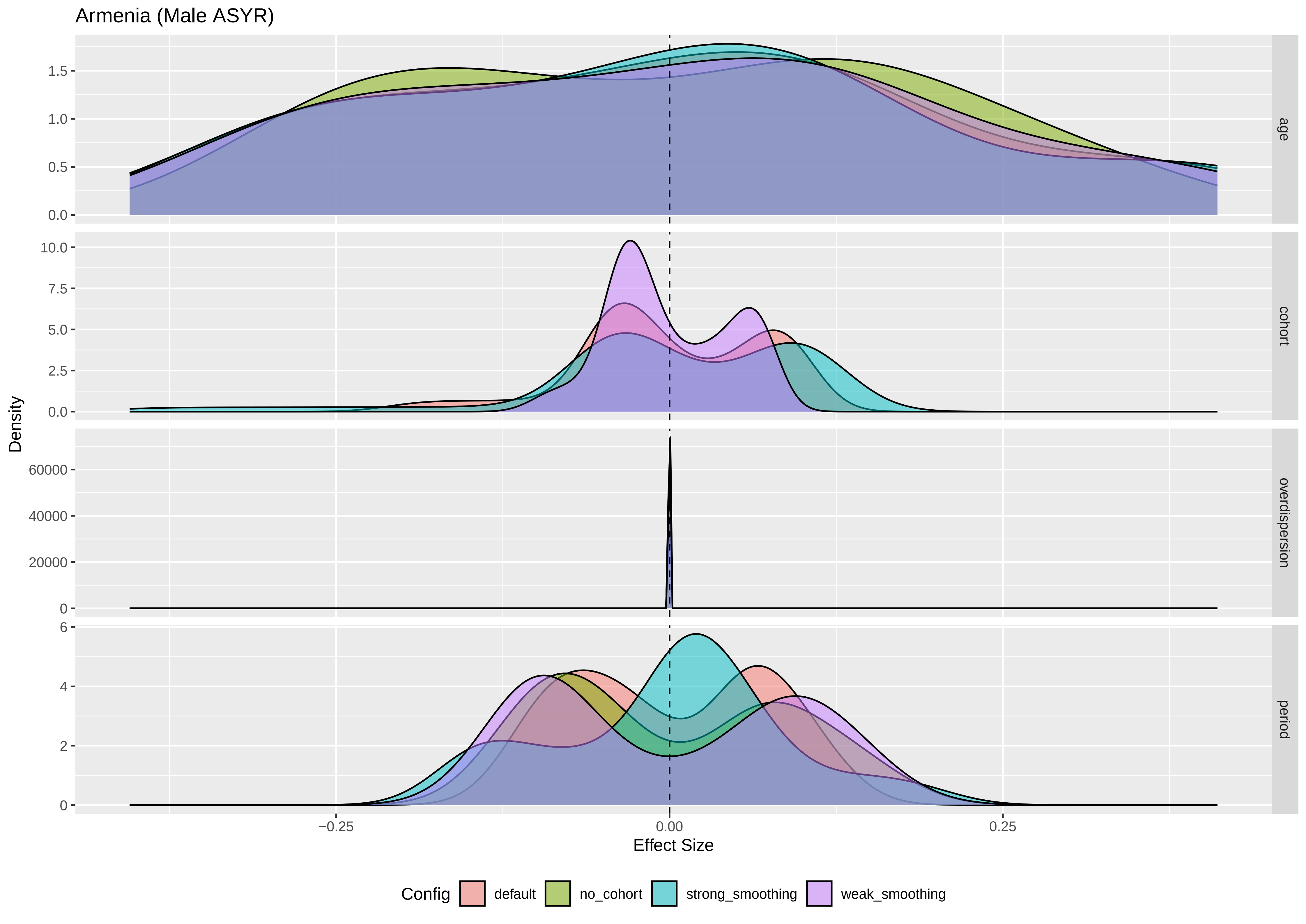

Armenia (Male ASYR)
age
cohort
overdispersion
period
Density
Effect Size
0.0
0.5
1.0
1.5
2.5
5.0
7.5
10.0
0
20000
40000
60000
2
4
6
−0.25
0.00
0.25
Config
default
no_cohort
strong_smoothing
weak_smoothing

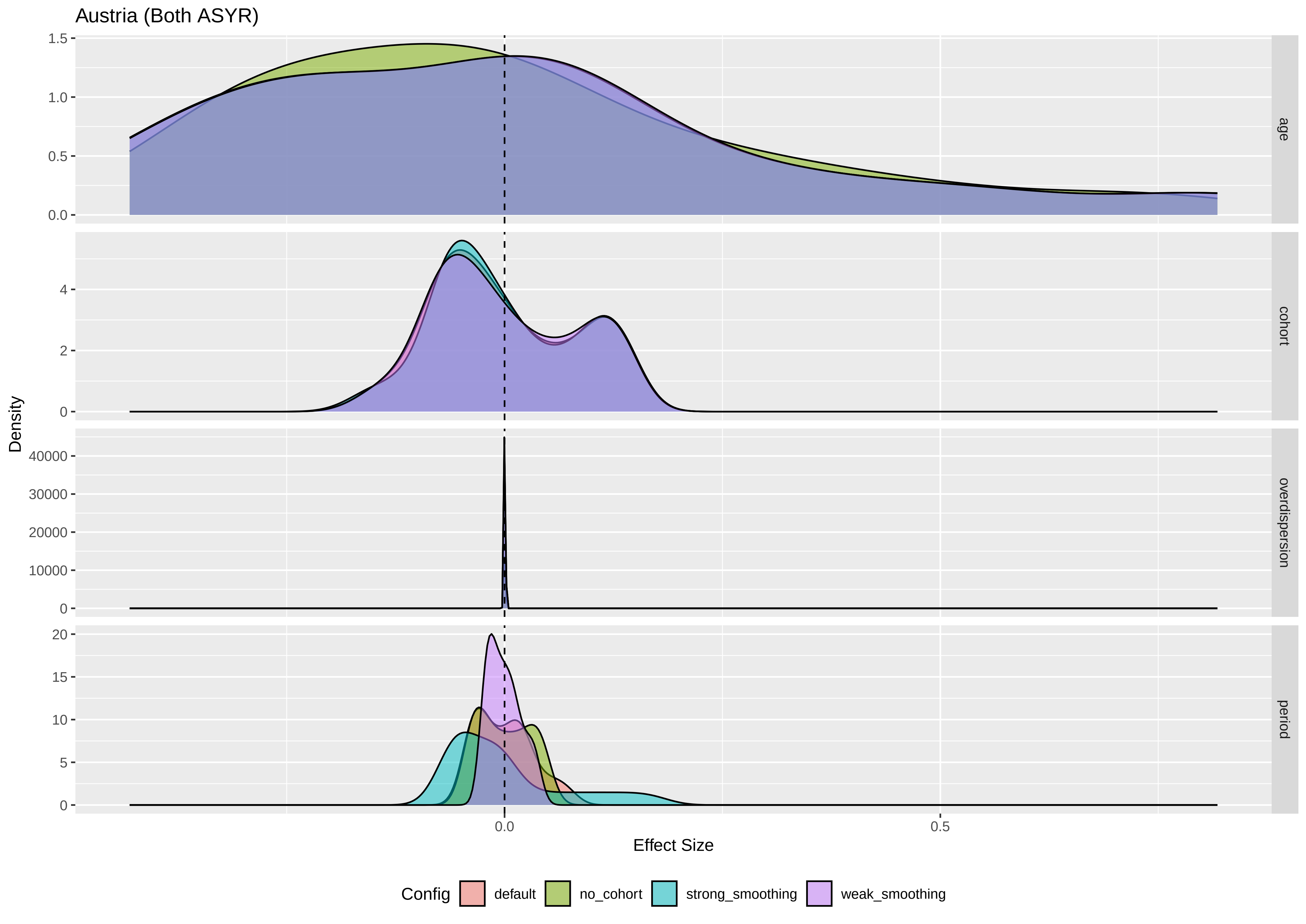
Austria (Both ASYR)
age
cohort
overdispersion
period
Density
Effect Size
0.0
0.5
1.5
1.0
0.5
0.0
4
2
0
40000
30000
20000
10000
0
20
15
10
5
0
Config
default
no_cohort
strong_smoothing
weak_smoothing

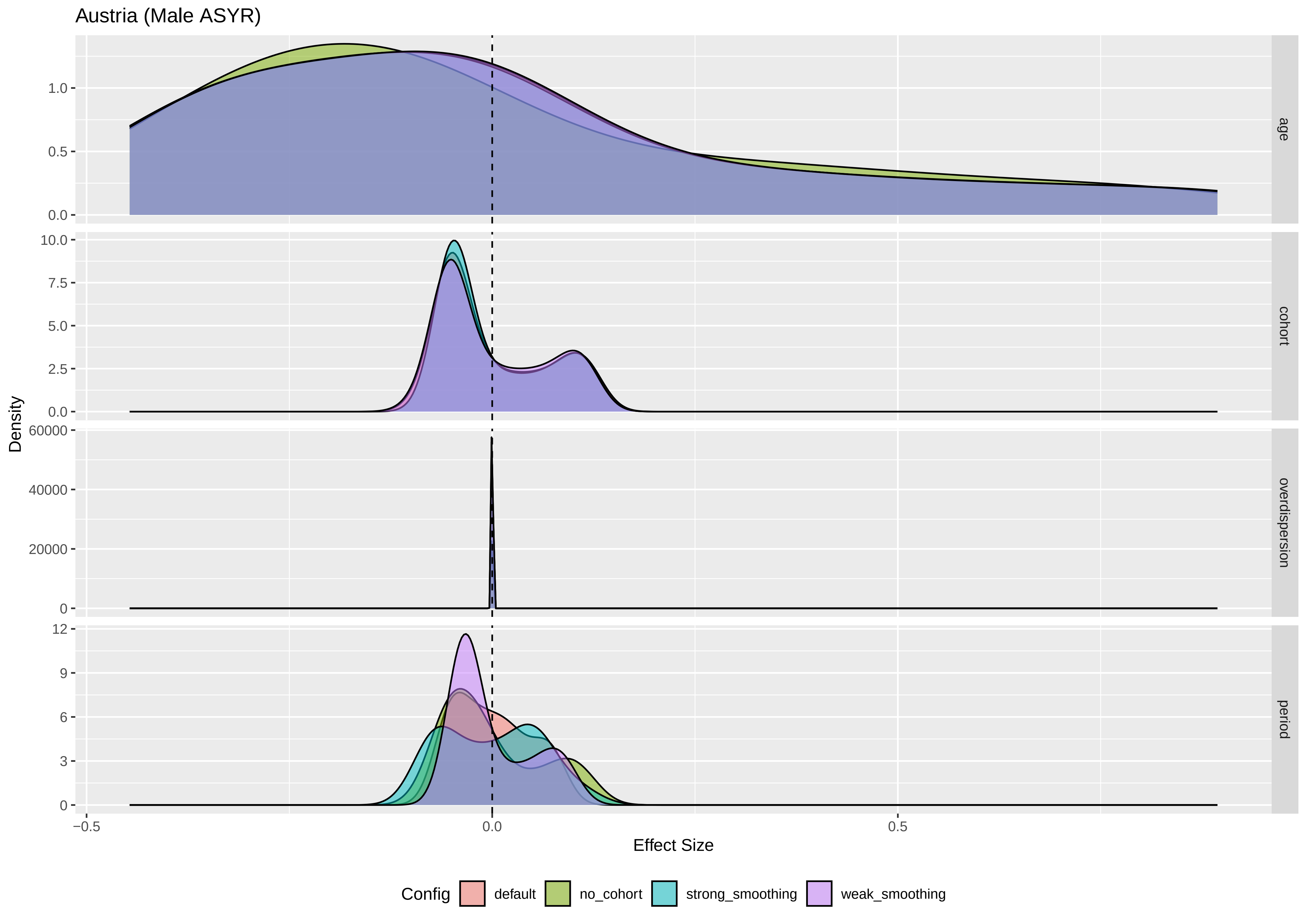

Austria (Male ASYR)
age
cohort
overdispersion
period
Density
Effect Size
-0.5
0.0
0.5
1.0
0
3
6
9
12
2.5
5.0
7.5
10.0
20000
40000
60000
Config
default
no_cohort
strong_smoothing
weak_smoothing

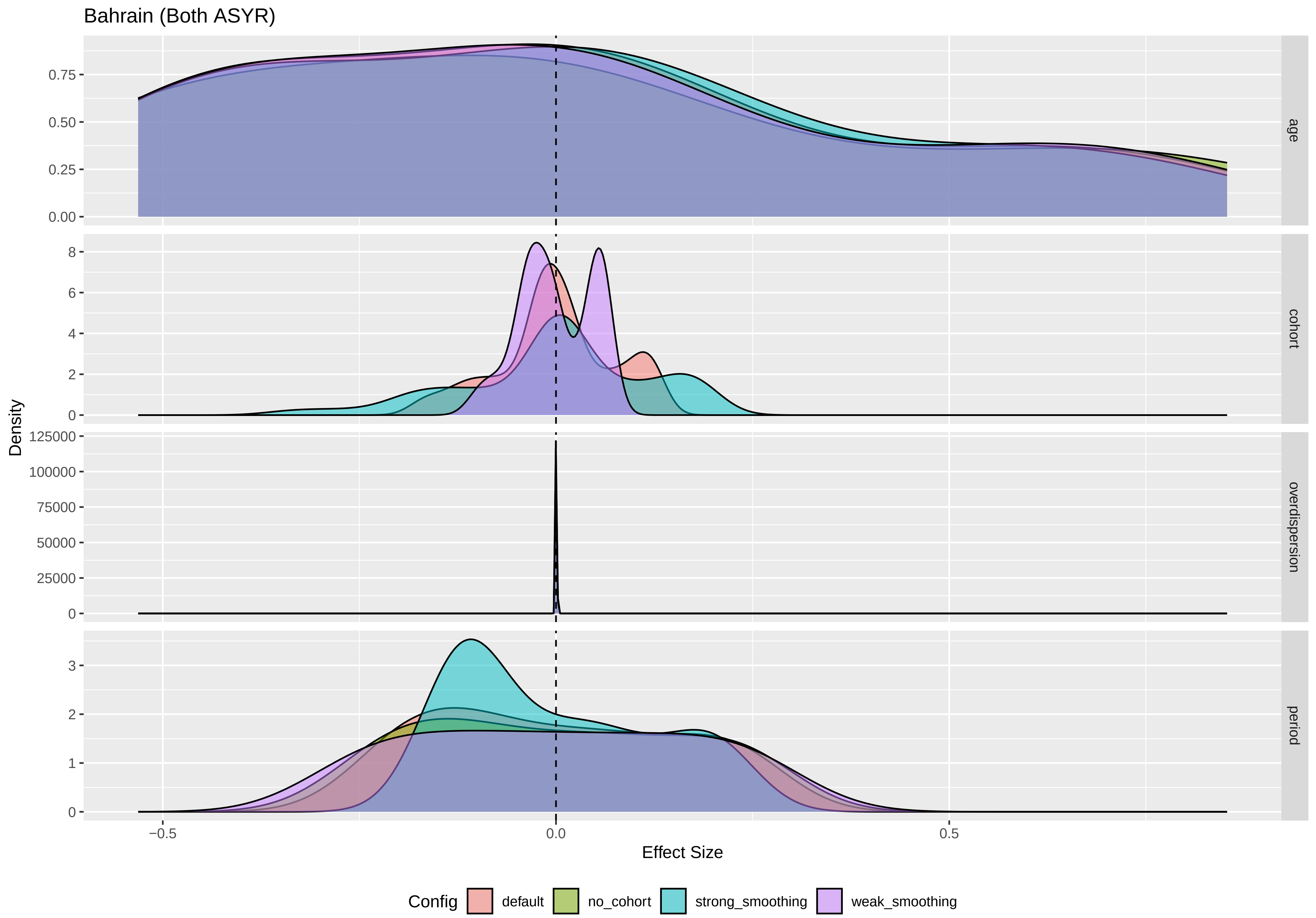

Bahrain (Both ASYR)
0.00
0.25
0.50
0.75
age
0
2
4
6
8
cohort
0
25000
50000
75000
100000
125000
overdispersion
0
1
2
3
period
Density
−0.5
0.0
0.5
Effect Size
Config
default
no_cohort
strong_smoothing
weak_smoothing

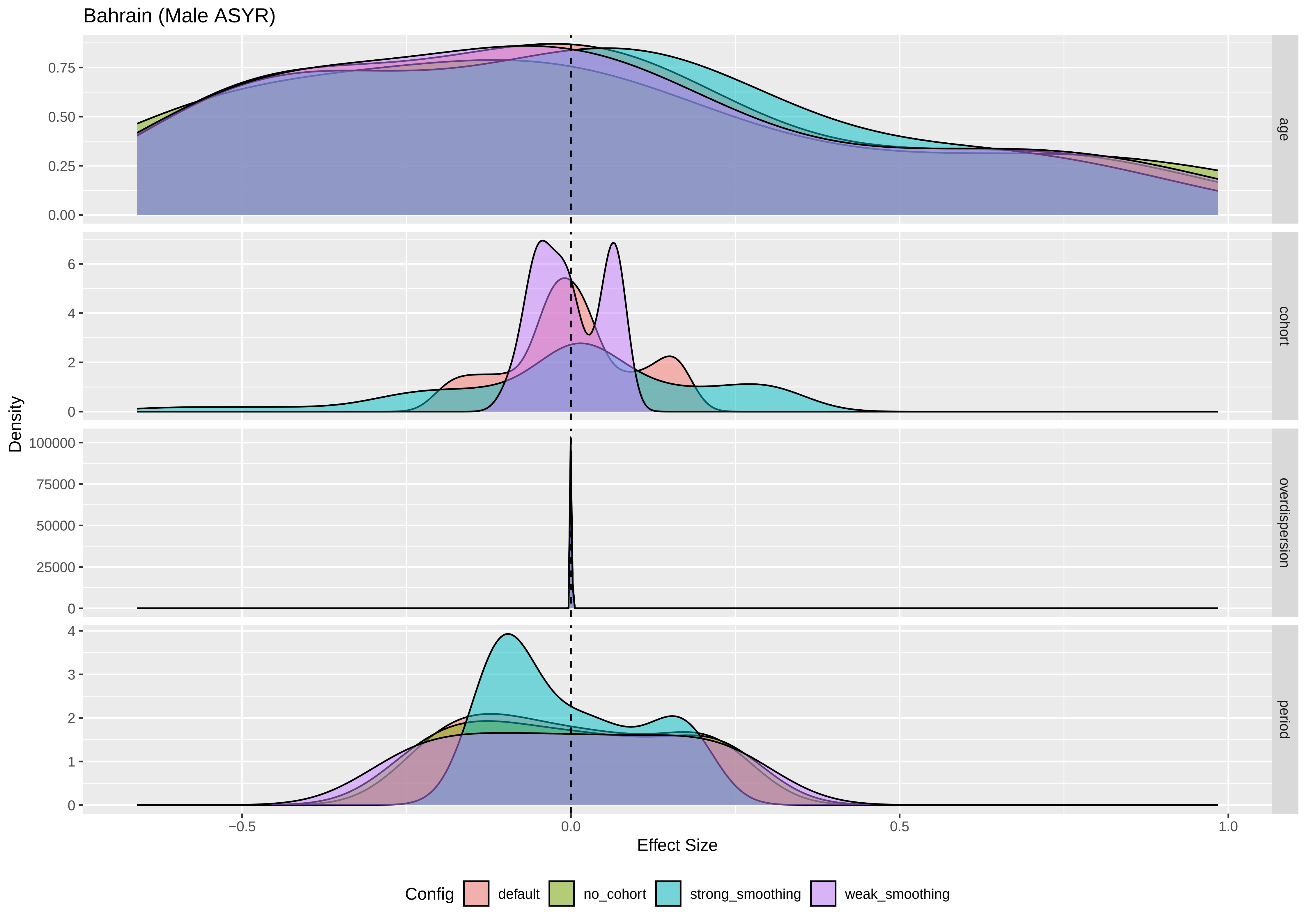

Bahrain (Male ASYR)
age
cohort
overdispersion
period
Density
Effect Size
0.00
0.25
0.50
0.75
0
2
4
6
0
25000
50000
75000
100000
0
1
2
3
4
-0.5
0.0
0.5
1.0
Config
default
no_cohort
strong_smoothing
weak_smoothing

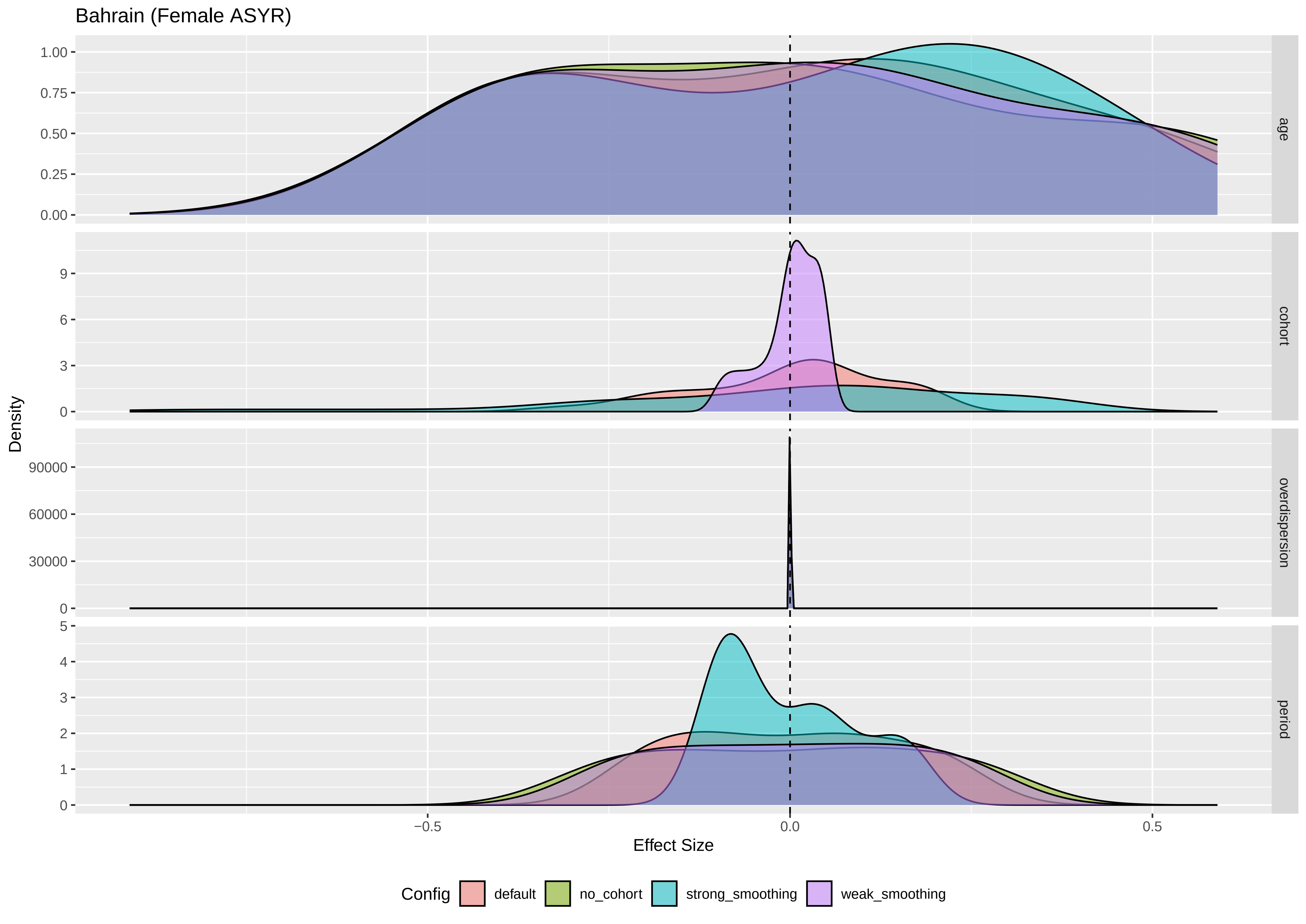

Bahrain (Female ASYR)
1.00
0.75
0.50
0.25
0.00
age
9
6
3
0
cohort
Density
90000
60000
30000
0
overdispersion
5
4
3
2
1
0
period
−0.5
0.0
0.5
Effect Size
Config
default
no_cohort
strong_smoothing
weak_smoothing

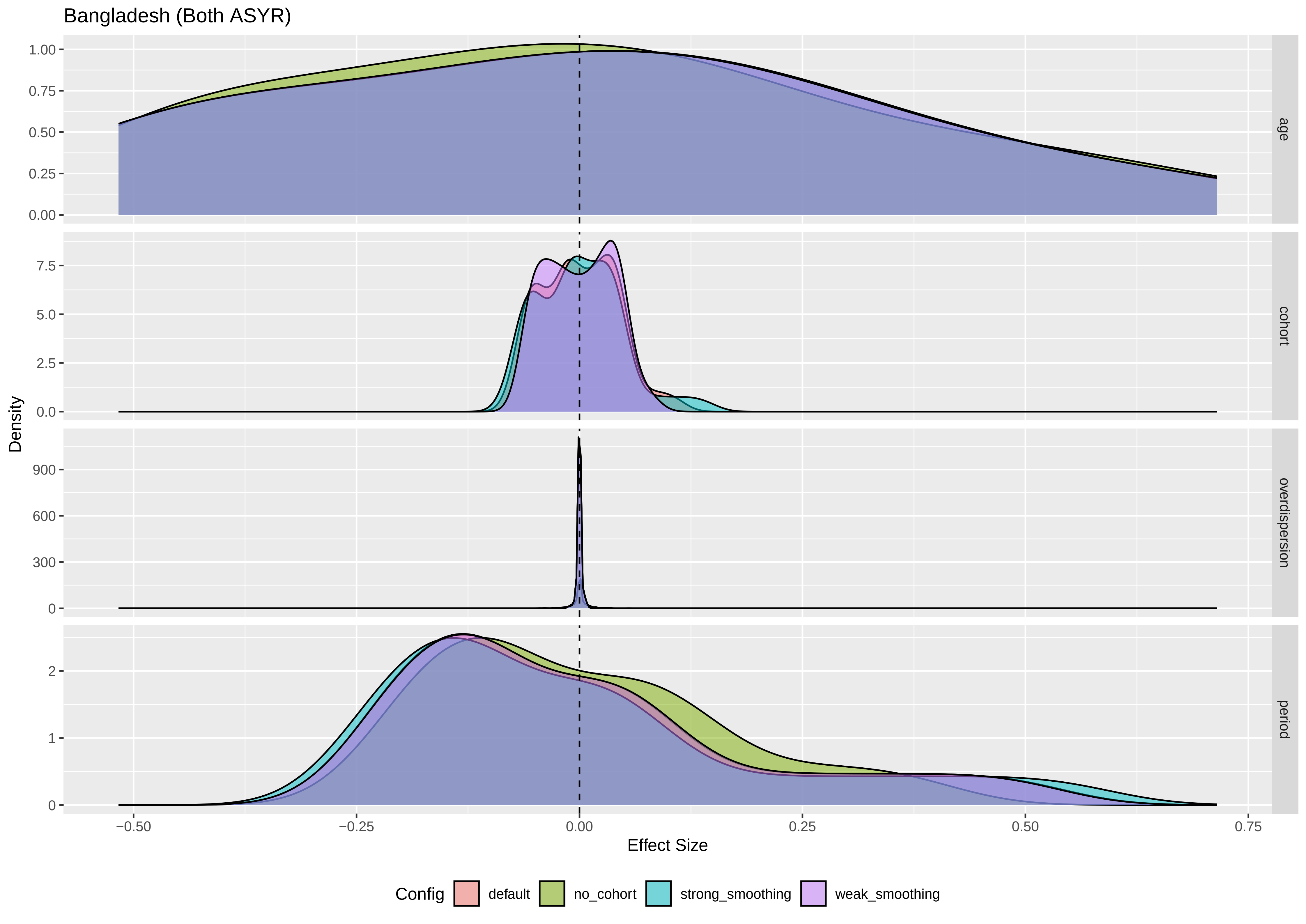

Bangladesh (Both ASYR)
age
cohort
overdispersion
period
Density
Effect Size
1.00
0.75
0.50
0.25
0.00
7.5
5.0
2.5
0.0
900
600
300
0
2
1
0
−0.50
−0.25
0.00
0.25
0.50
0.75
Config
default
no_cohort
strong_smoothing
weak_smoothing

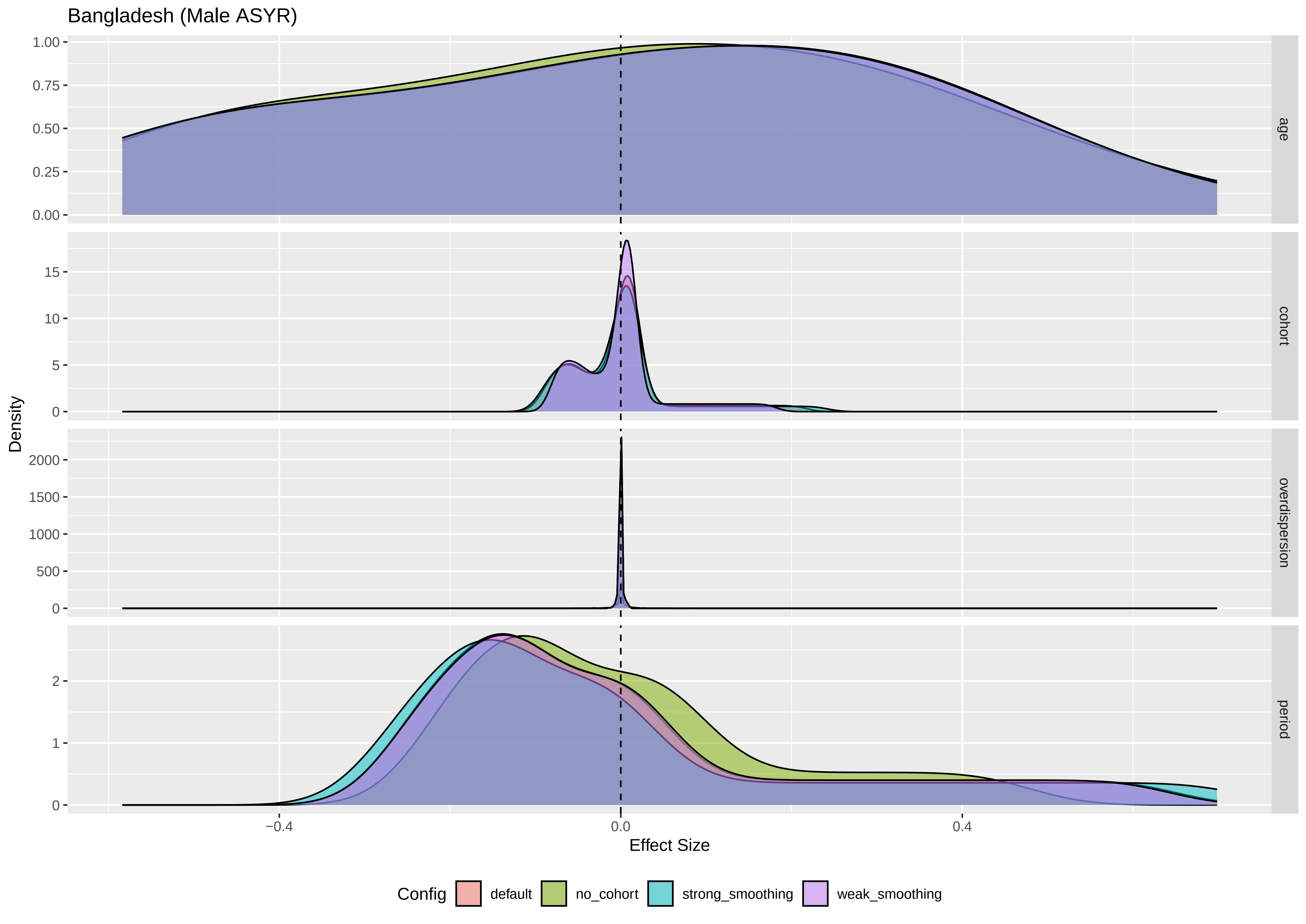

Bangladesh (Male ASYR)
Density
Effect Size
age
cohort
overdispersion
period
1.00
0.75
0.50
0.25
0.00
15
10
5
0
2000
1500
1000
500
0
2
1
0
−0.4
0.0
0.4
Config
default
no_cohort
strong_smoothing
weak_smoothing

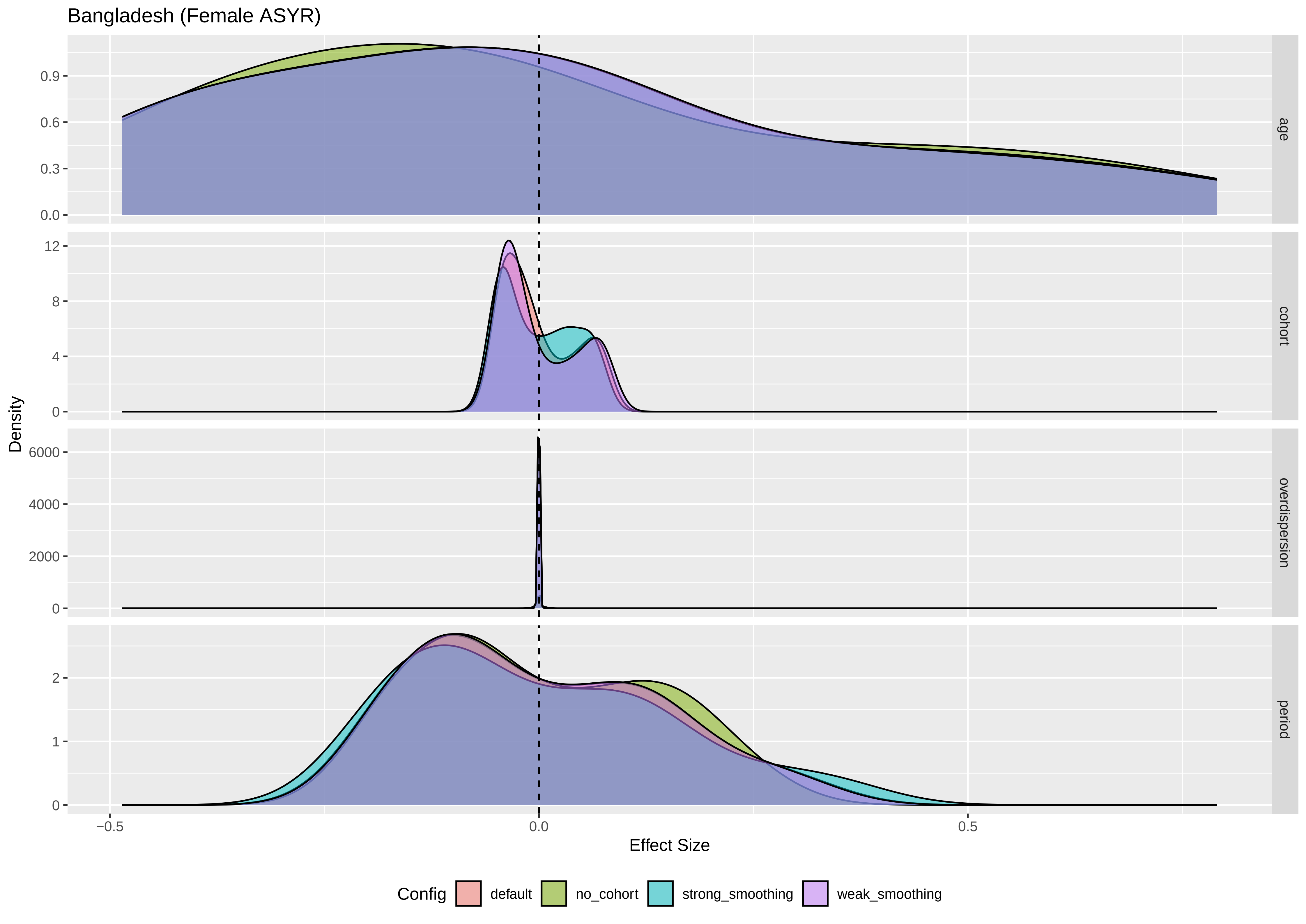
Bangladesh (Female ASYR)
age
cohort
overdispersion
period
Density
Effect Size
0.0
0.3
0.6
0.9
0
4
8
12
0
2000
4000
6000
0
1
2
−0.5
0.0
0.5
Config
default
no_cohort
strong_smoothing
weak_smoothing

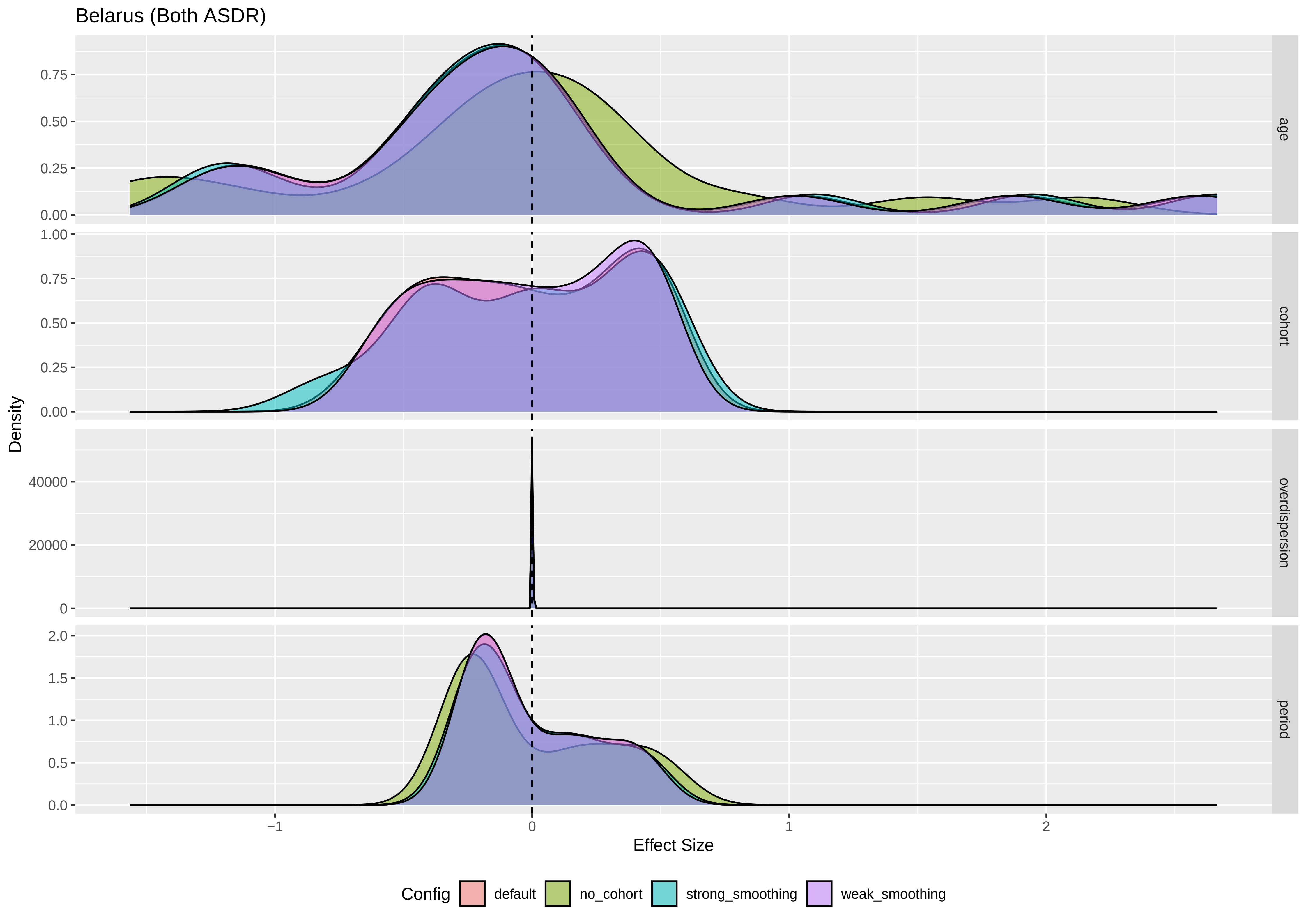

Belarus (Both ASDR)
Density
Effect Size
age
cohort
overdispersion
period
0.00
0.25
0.50
0.75
1.00
0
20000
40000
0.0
0.5
1.0
1.5
2.0
−1
0
1
2
Config
default
no_cohort
strong_smoothing
weak_smoothing

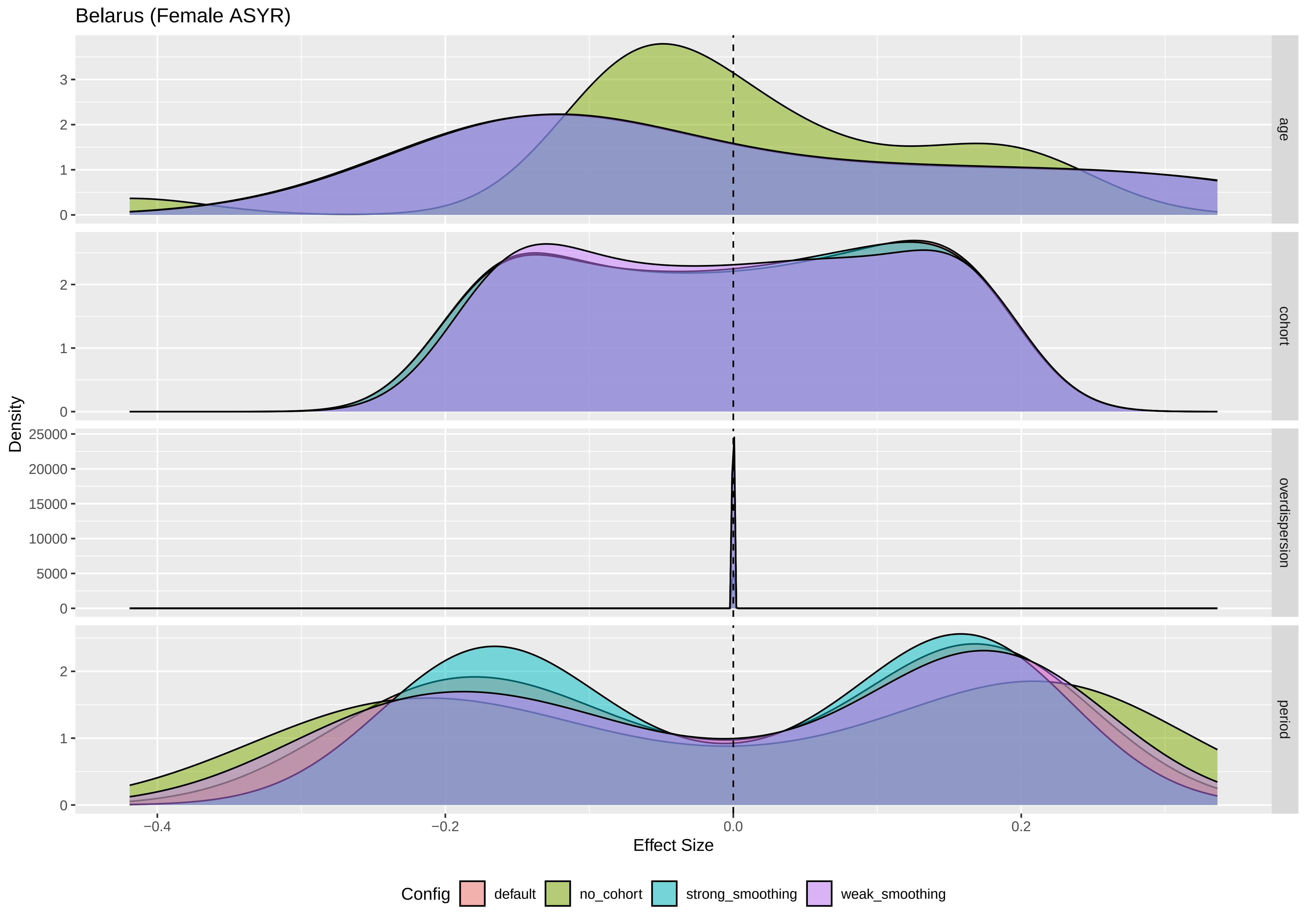

Belarus (Female ASYR)
Density
Effect Size
age
cohort
overdispersion
period
3
2
1
0
25000
20000
15000
10000
5000
−0.4
−0.2
0.0
0.2
Config
default
no_cohort
strong_smoothing
weak_smoothing

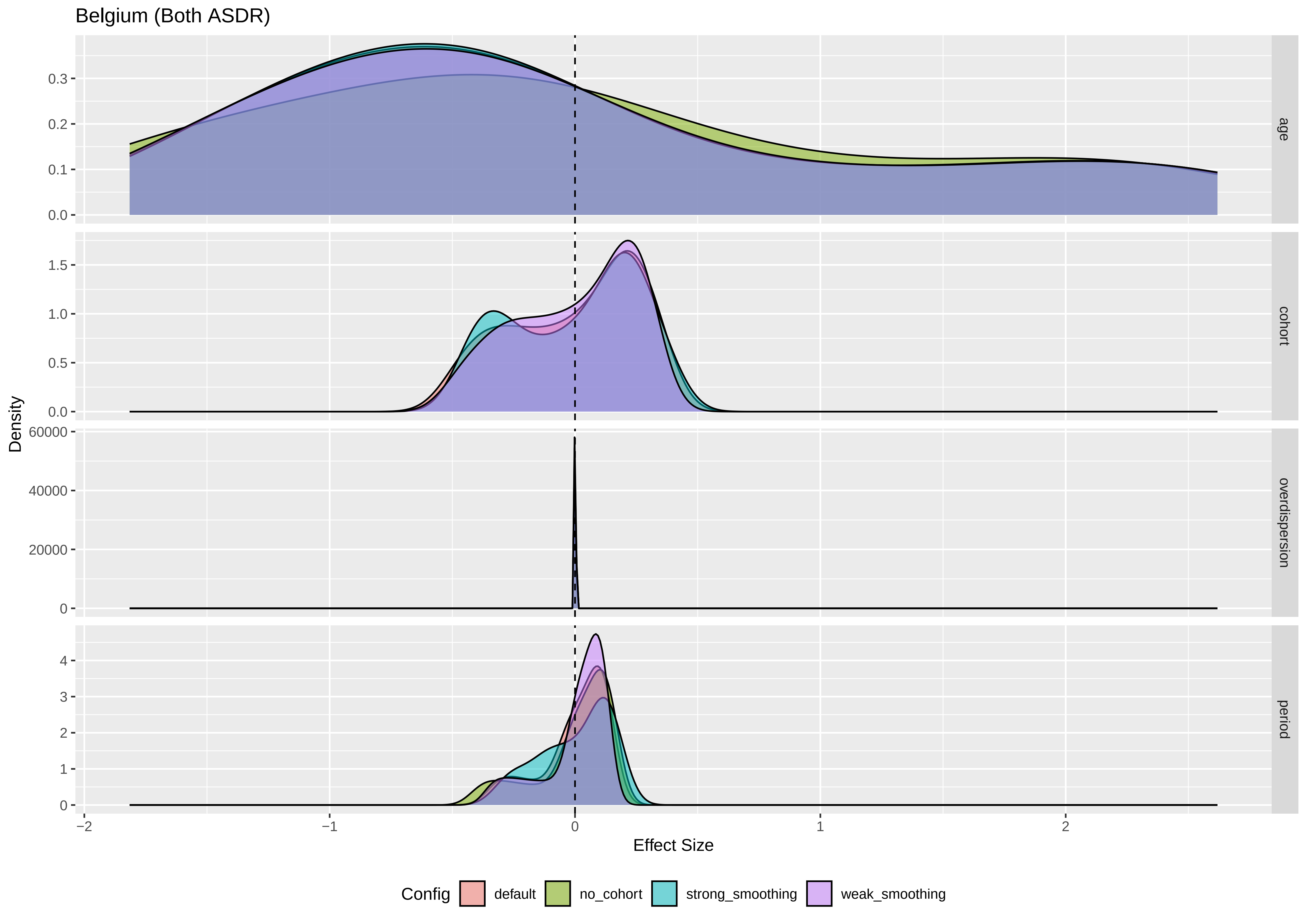

Belgium (Both ASDR)
age
cohort
overdispersion
period
Density
Effect Size
0.0
0.1
0.2
0.3
0.5
1.0
1.5
0
20000
40000
60000
1
2
3
4
−2
−1
Config
default
no_cohort
strong_smoothing
weak_smoothing

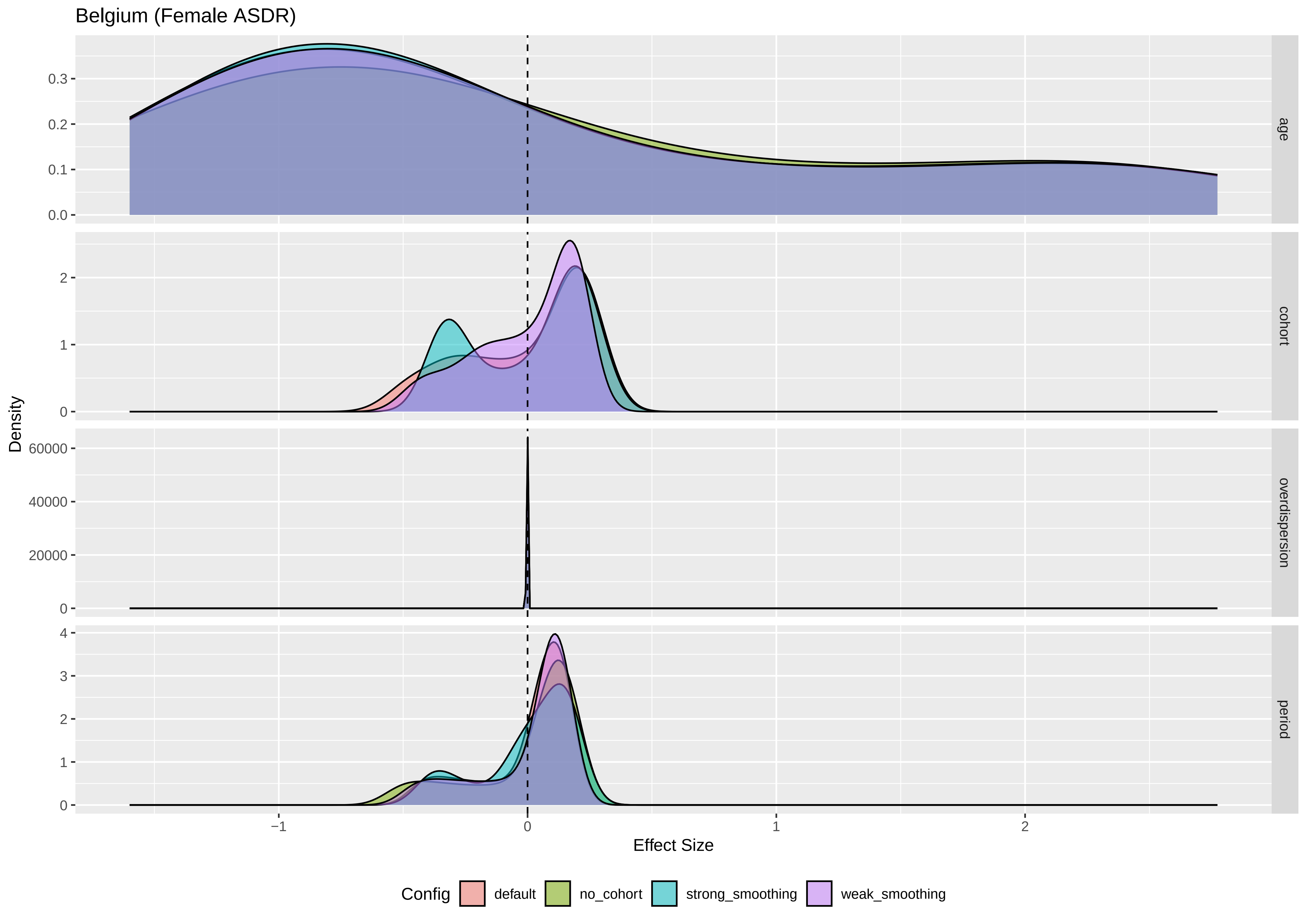

Belgium (Female ASDR)
age
cohort
overdispersion
period
Density
Effect Size
0.0
0.1
0.2
0.3
0
1
2
20000
40000
60000
3
4
−1
Config
default
no_cohort
strong_smoothing
weak_smoothing

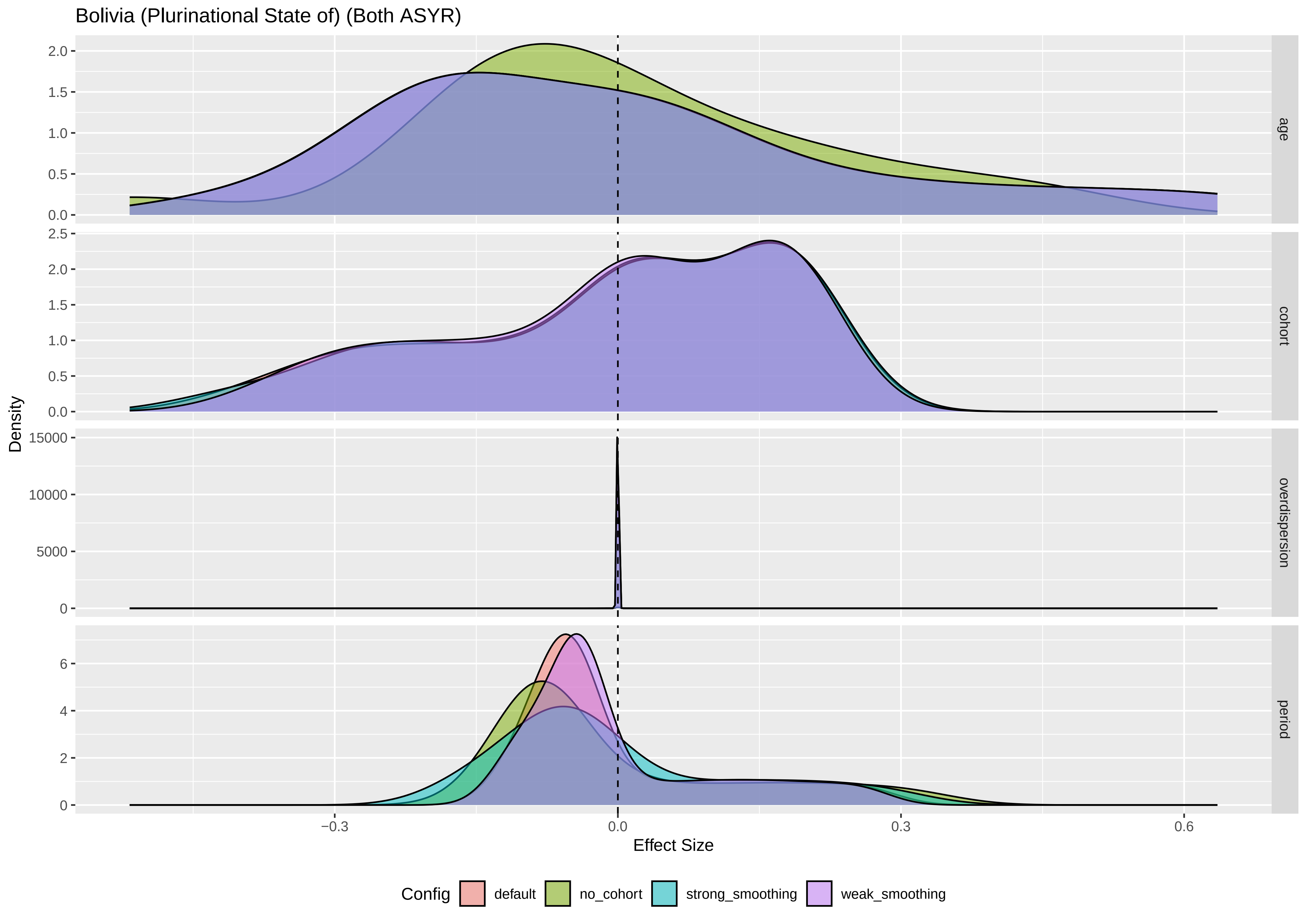

Bolivia (Plurinational State of) (Both ASYR)
Density
Effect Size
age
cohort
overdispersion
period
2.0
1.5
1.0
0.5
0.0
2.5
2.0
1.5
1.0
0.5
0.0
15000
10000
5000
0
6
4
2
0
−0.3
0.0
0.3
0.6
Config
default
no_cohort
strong_smoothing
weak_smoothing

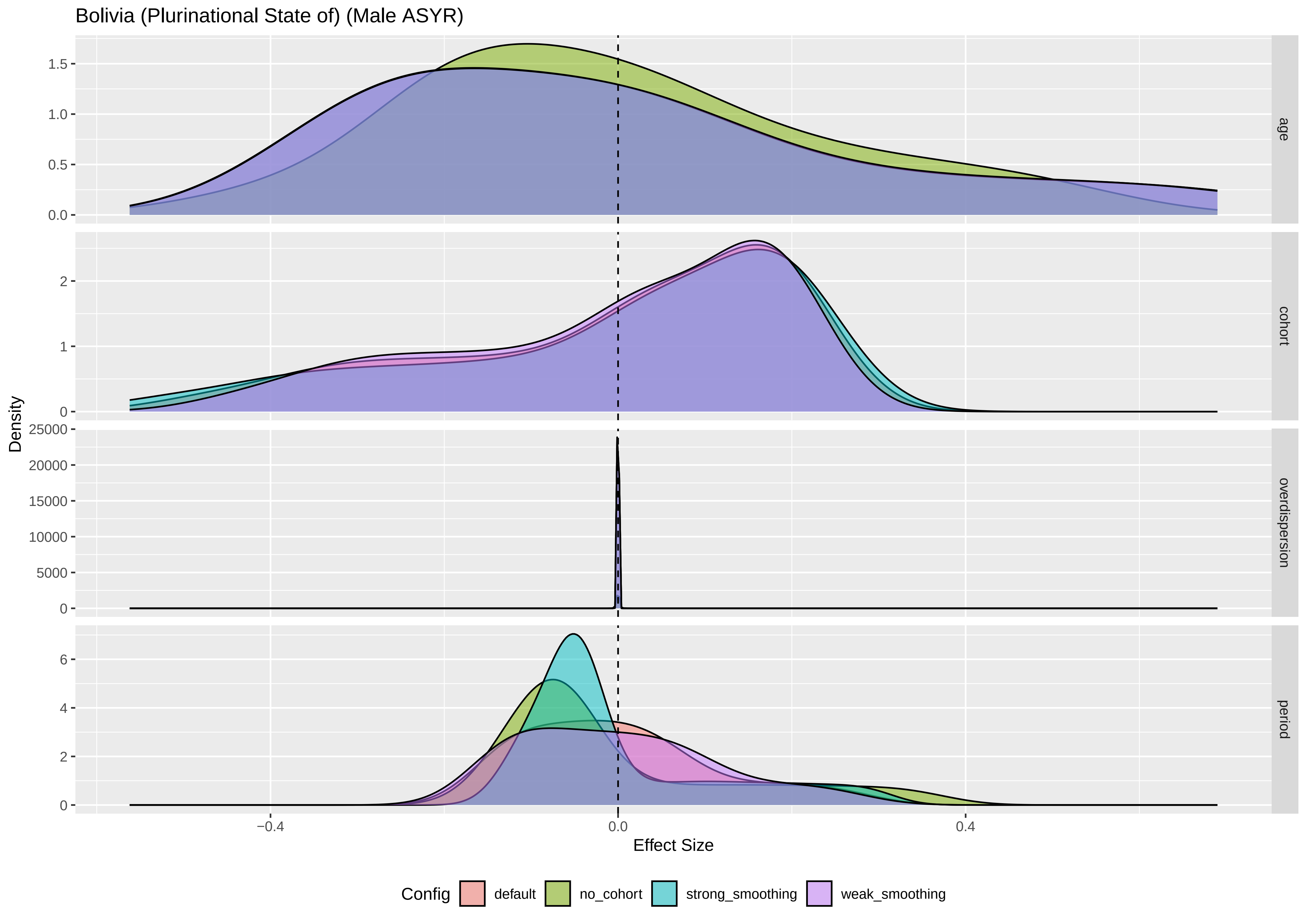

Bolivia (Plurinational State of) (Male ASYR)
Density
Effect Size
age
cohort
overdispersion
period
0.0
0.5
1.0
1.5
0
1
2
5000
10000
15000
20000
25000
4
6
−0.4
0.0
0.4
Config
default
no_cohort
strong_smoothing
weak_smoothing

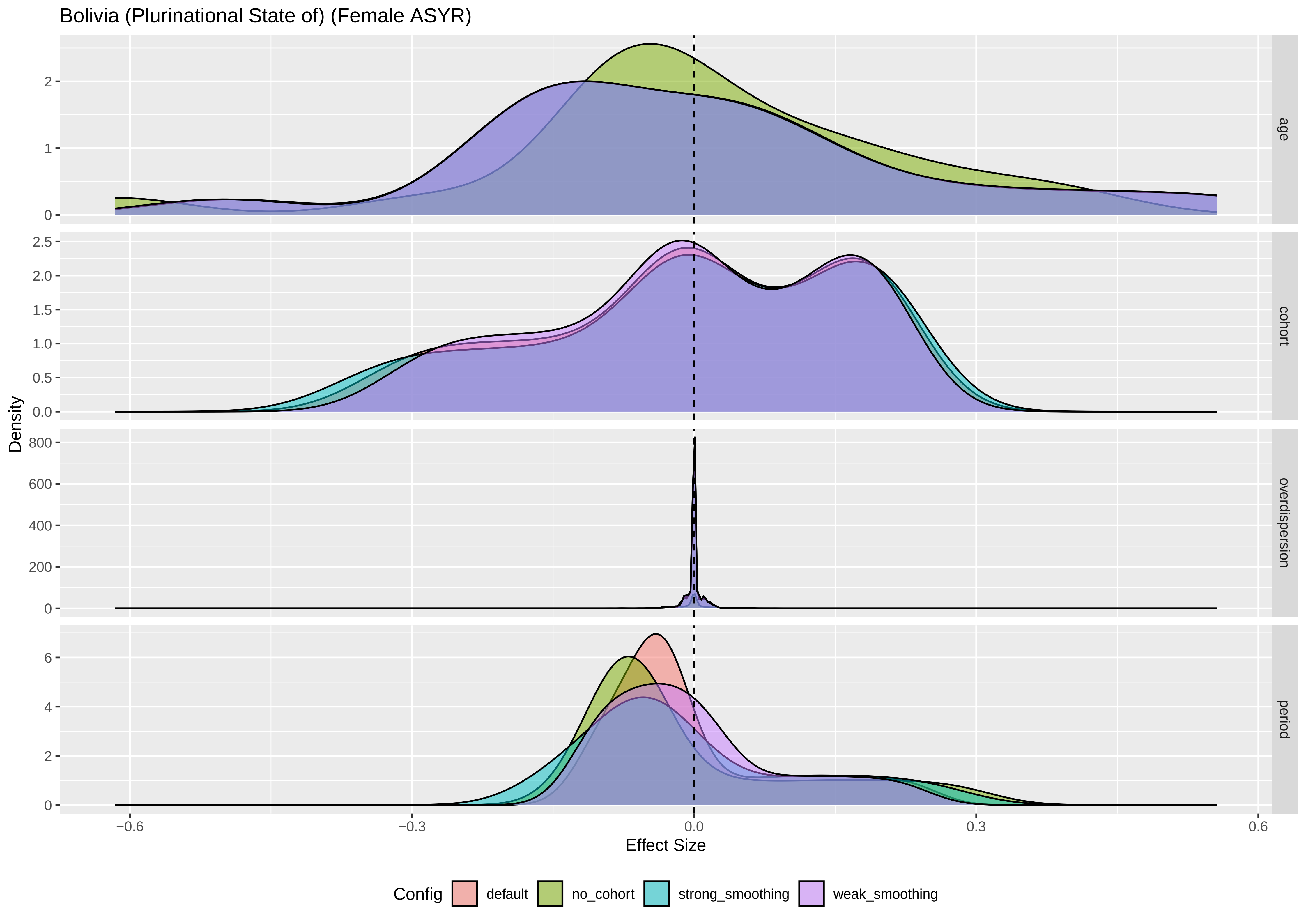

Bolivia (Plurinational State of) (Female ASYR)
age
cohort
overdispersion
period
Density
Effect Size
−0.6
−0.3
0.0
0.3
0.6
0
1
2
0.0
0.5
1.0
1.5
2.0
2.5
200
400
600
800
4
6
Config
default
no_cohort
strong_smoothing
weak_smoothing

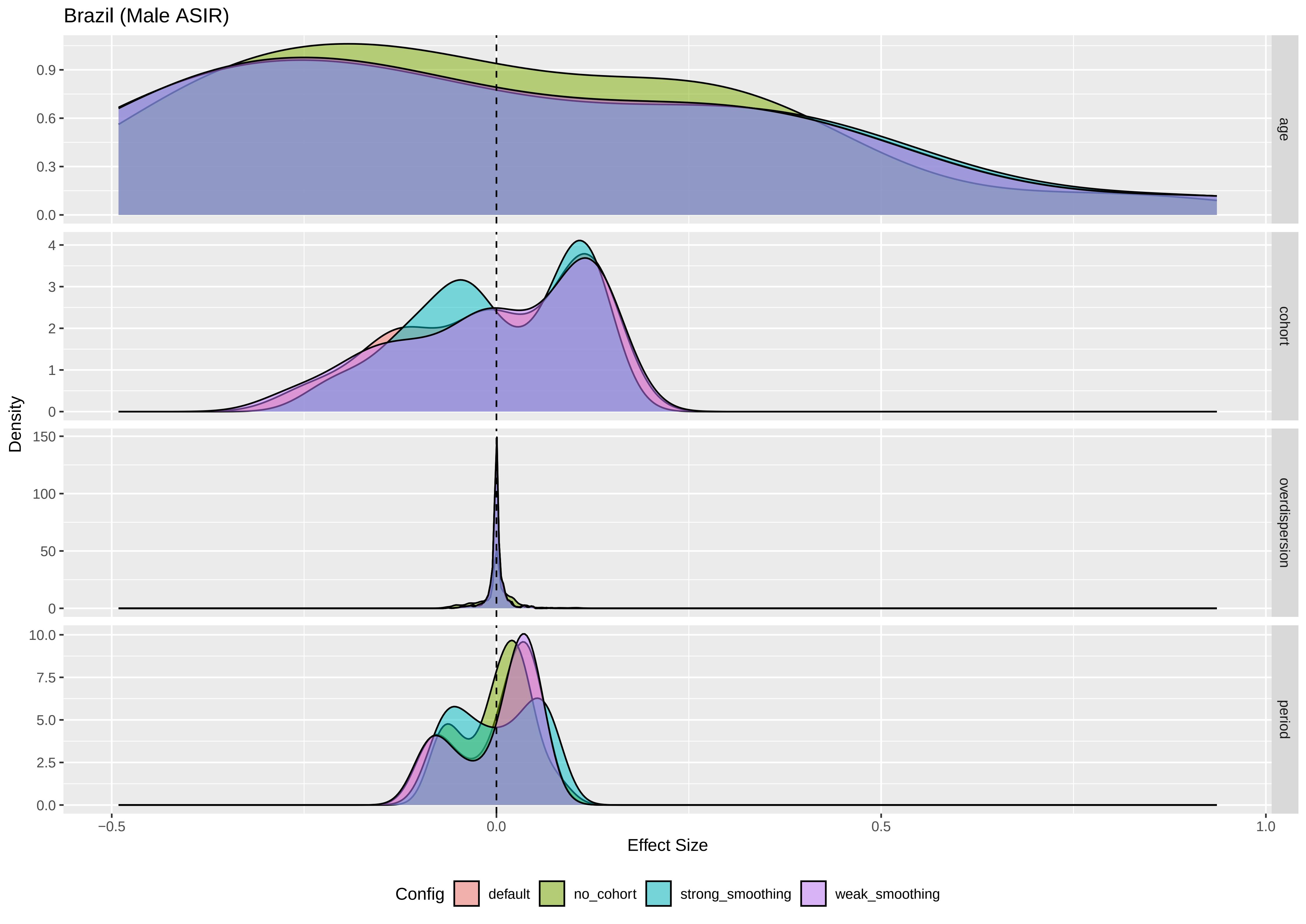

Brazil (Male ASIR)
0.9
0.6
0.3
0.0
age
4
3
2
1
0
cohort
150
100
50
0
overdispersion
10.0
7.5
5.0
2.5
0.0
period
Density
−0.5
0.0
0.5
1.0
Effect Size
Config
default
no_cohort
strong_smoothing
weak_smoothing

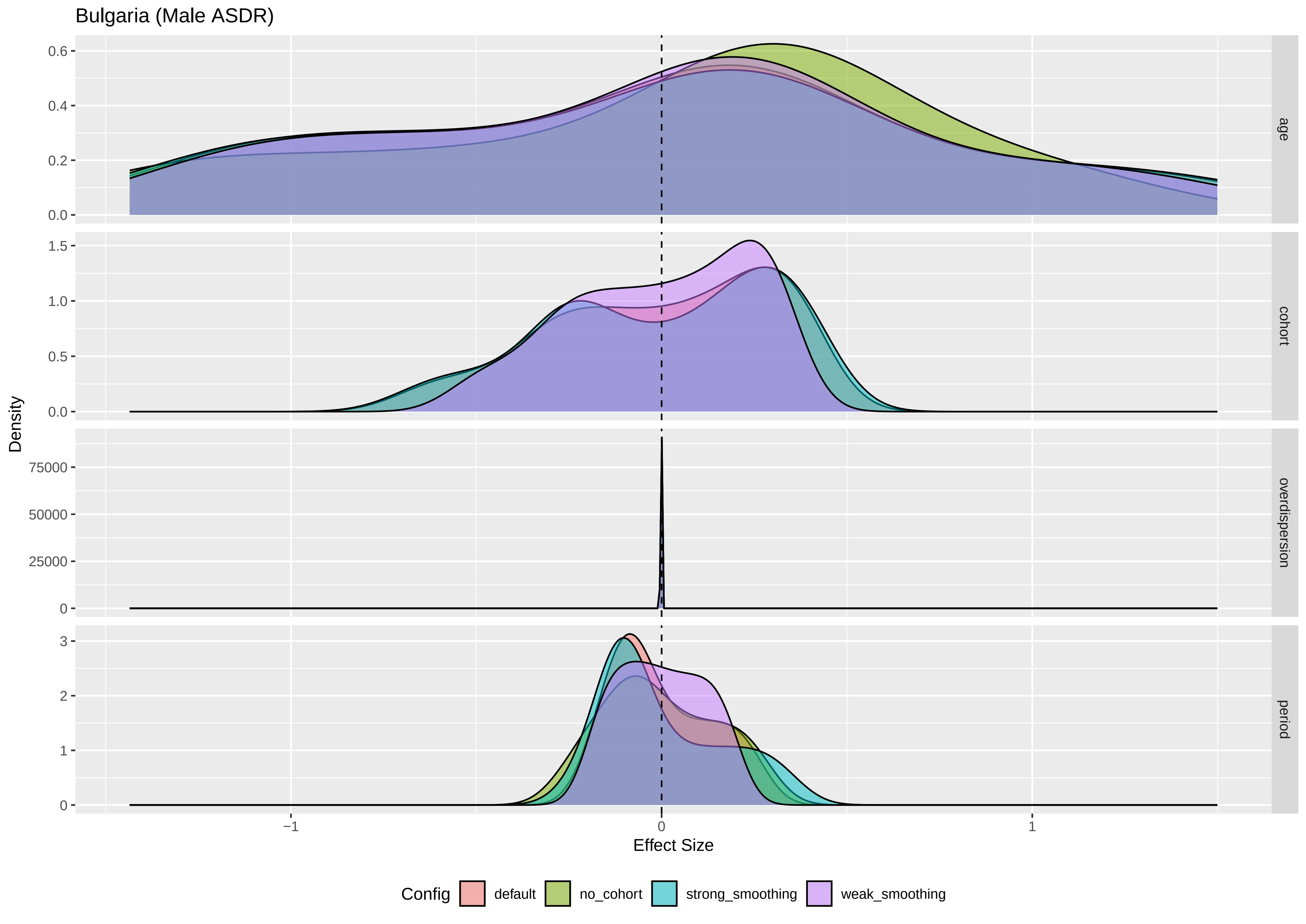

Bulgaria (Male ASDR)
Density
Effect Size
age
cohort
overdispersion
period
0.0
0.2
0.4
0.6
0.5
1.0
1.5
0
25000
50000
75000
1
2
3
−1
0
1
Config
default
no_cohort
strong_smoothing
weak_smoothing

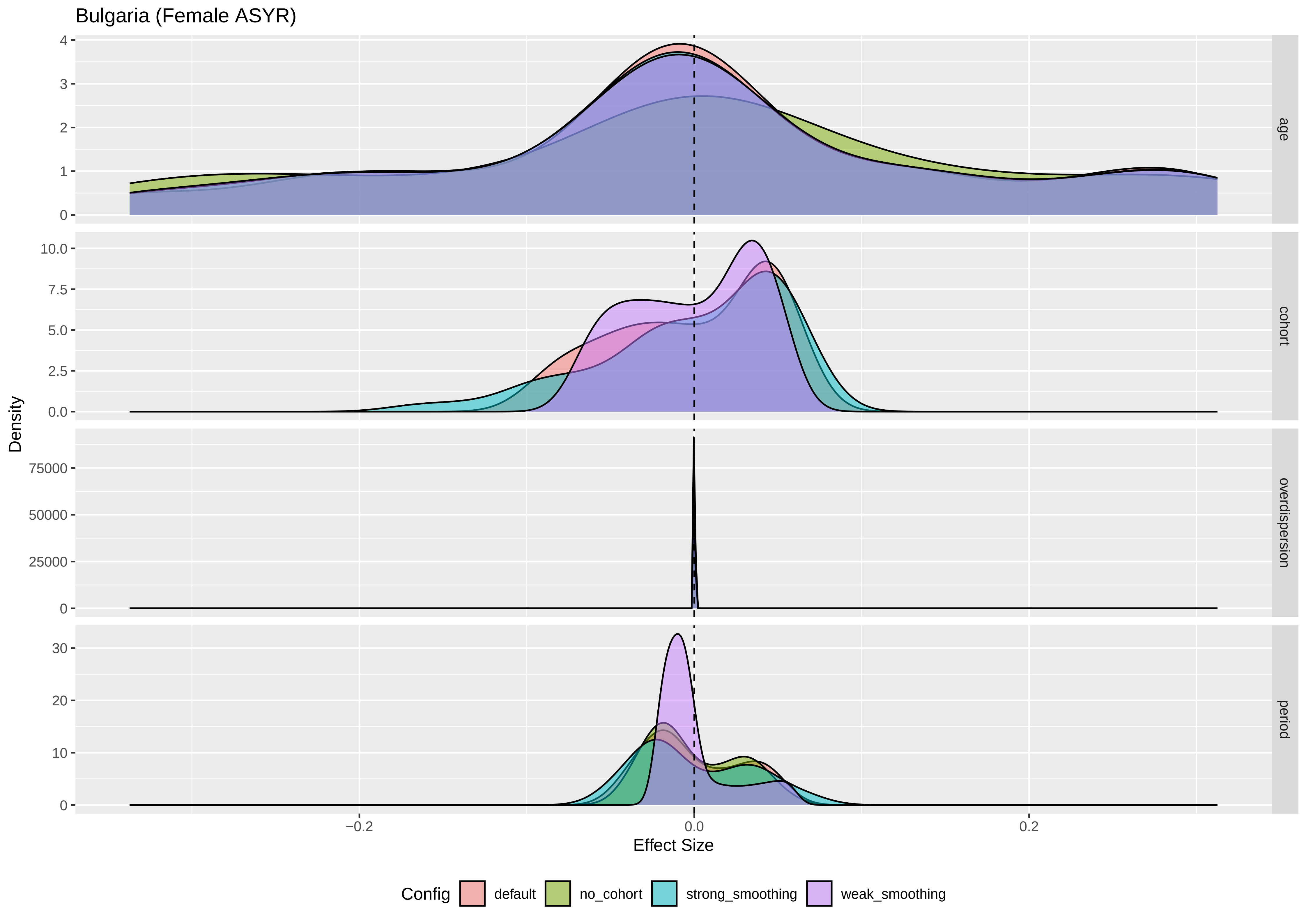

Bulgaria (Female ASYR)
age
cohort
overdispersion
period
Density
Effect Size
−0.2
0.0
0.2
4
3
2
1
0
10.0
7.5
5.0
2.5
0.0
75000
50000
25000
0
30
20
10
0
Config
default
no_cohort
strong_smoothing
weak_smoothing

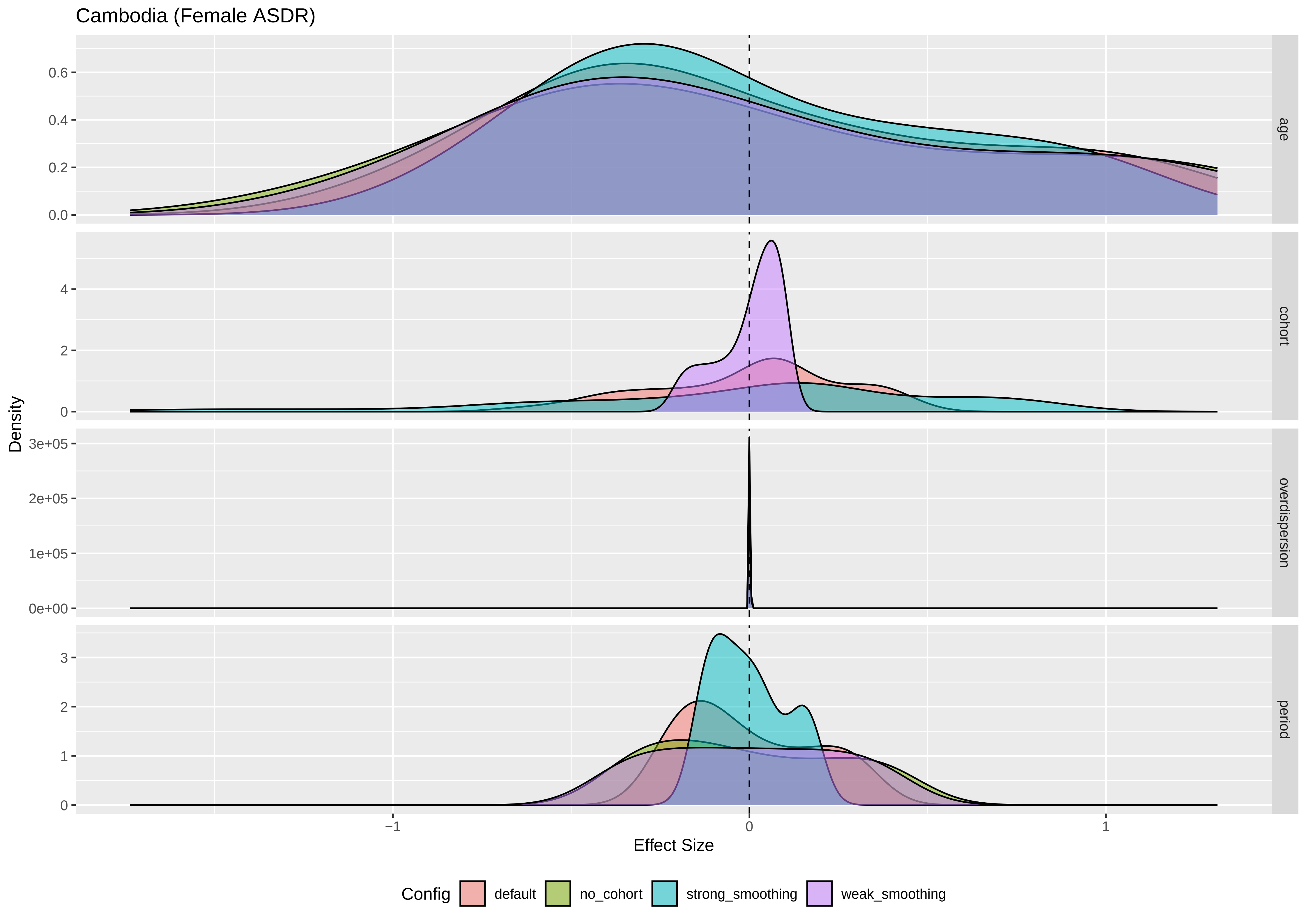

Cambodia (Female ASDR)
Density
0.0
0.2
0.4
0.6
age
0
2
4
cohort
0e+00
1e+05
2e+05
3e+05
overdispersion
0
1
2
3
period
−1
0
1
Effect Size
Config
default
no_cohort
strong_smoothing
weak_smoothing

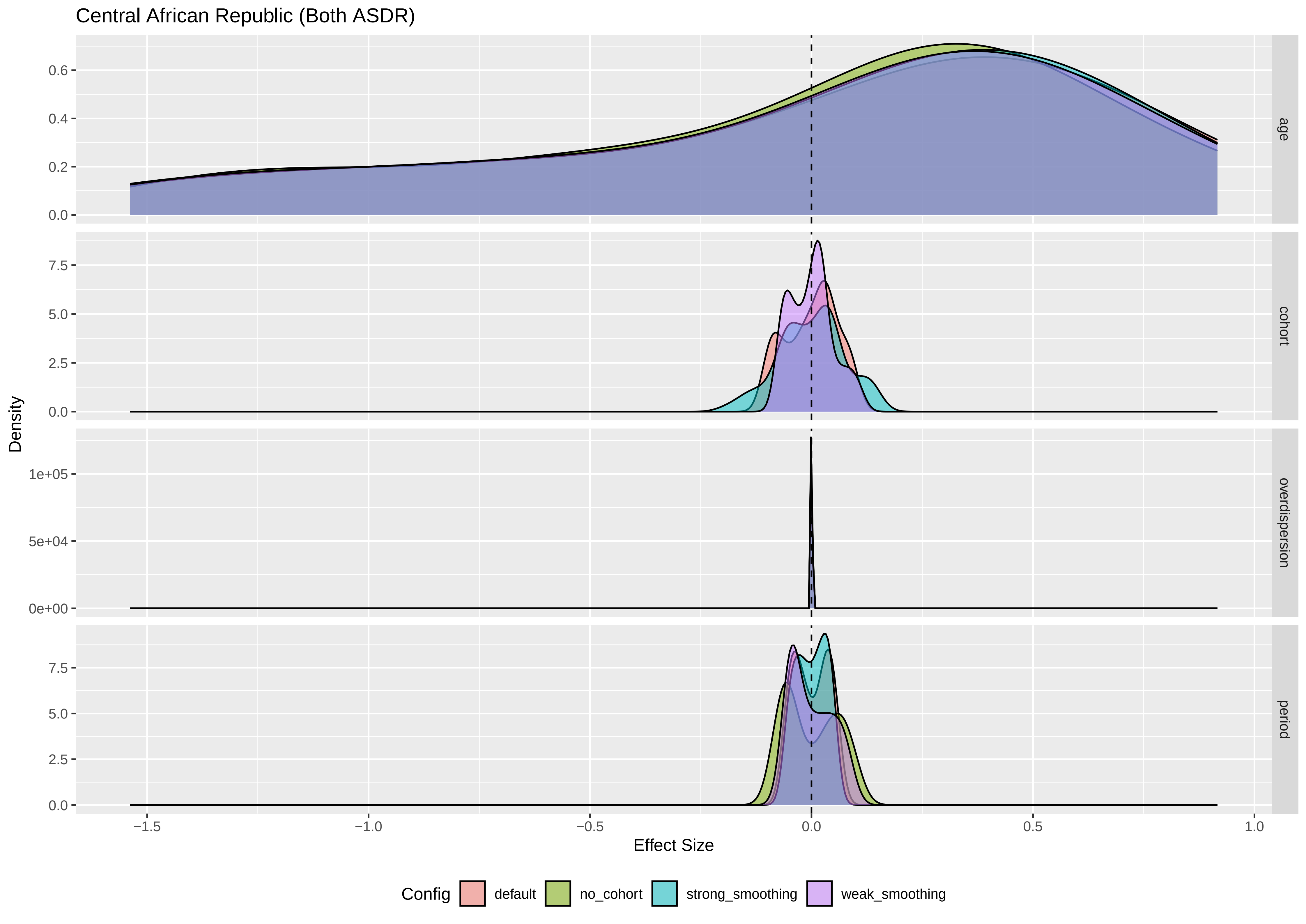

Central African Republic (Both ASDR)
age
cohort
overdispersion
period
Density
Effect Size
0.0
0.2
0.4
0.6
2.5
5.0
7.5
0e+00
5e+04
1e+05
−1.5
−1.0
−0.5
0.0
0.5
1.0
Config
default
no_cohort
strong_smoothing
weak_smoothing

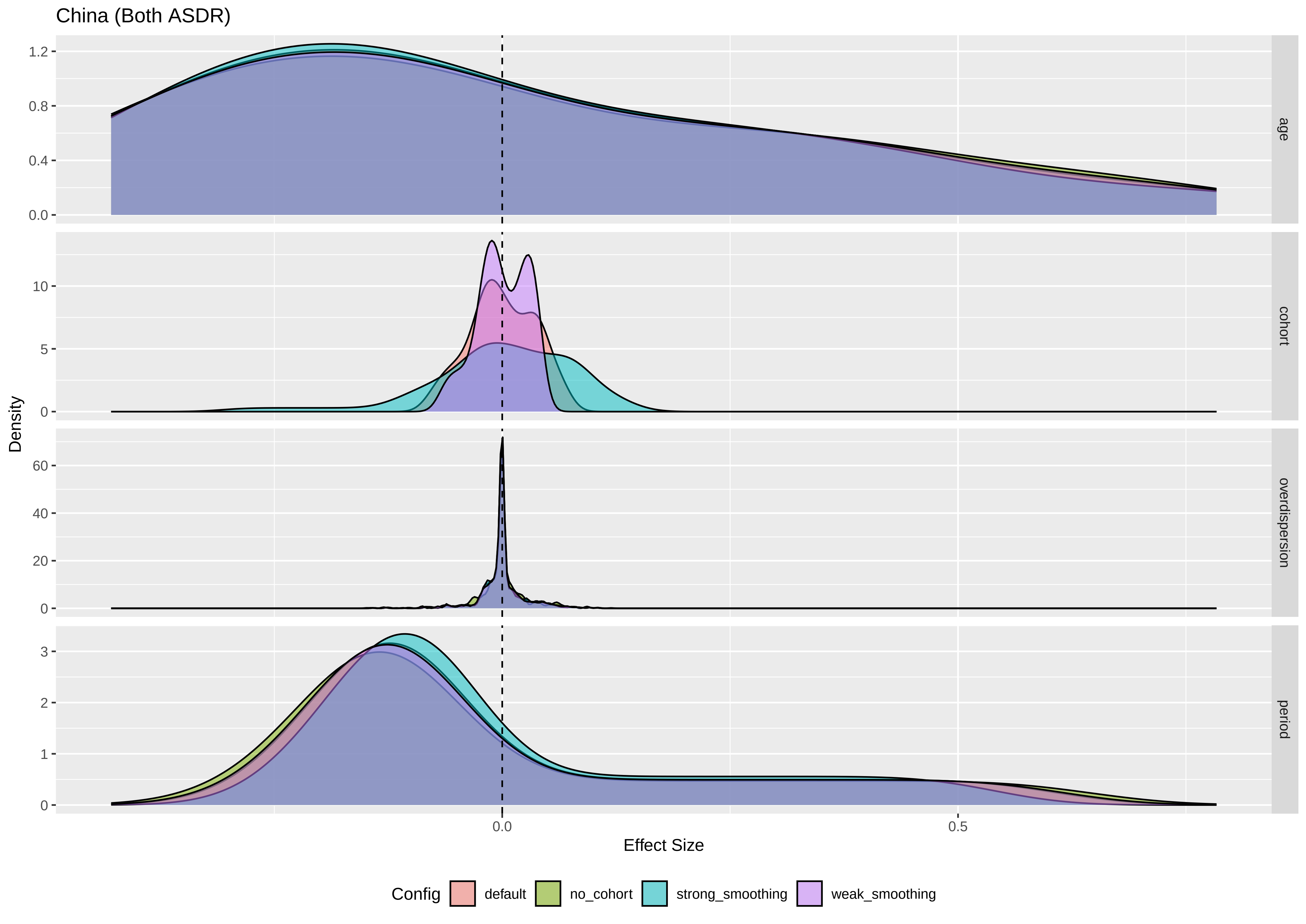

China (Both ASDR)
Density
Effect Size
age
cohort
overdispersion
period
0.0
0.5
Config
default
no_cohort
strong_smoothing
weak_smoothing

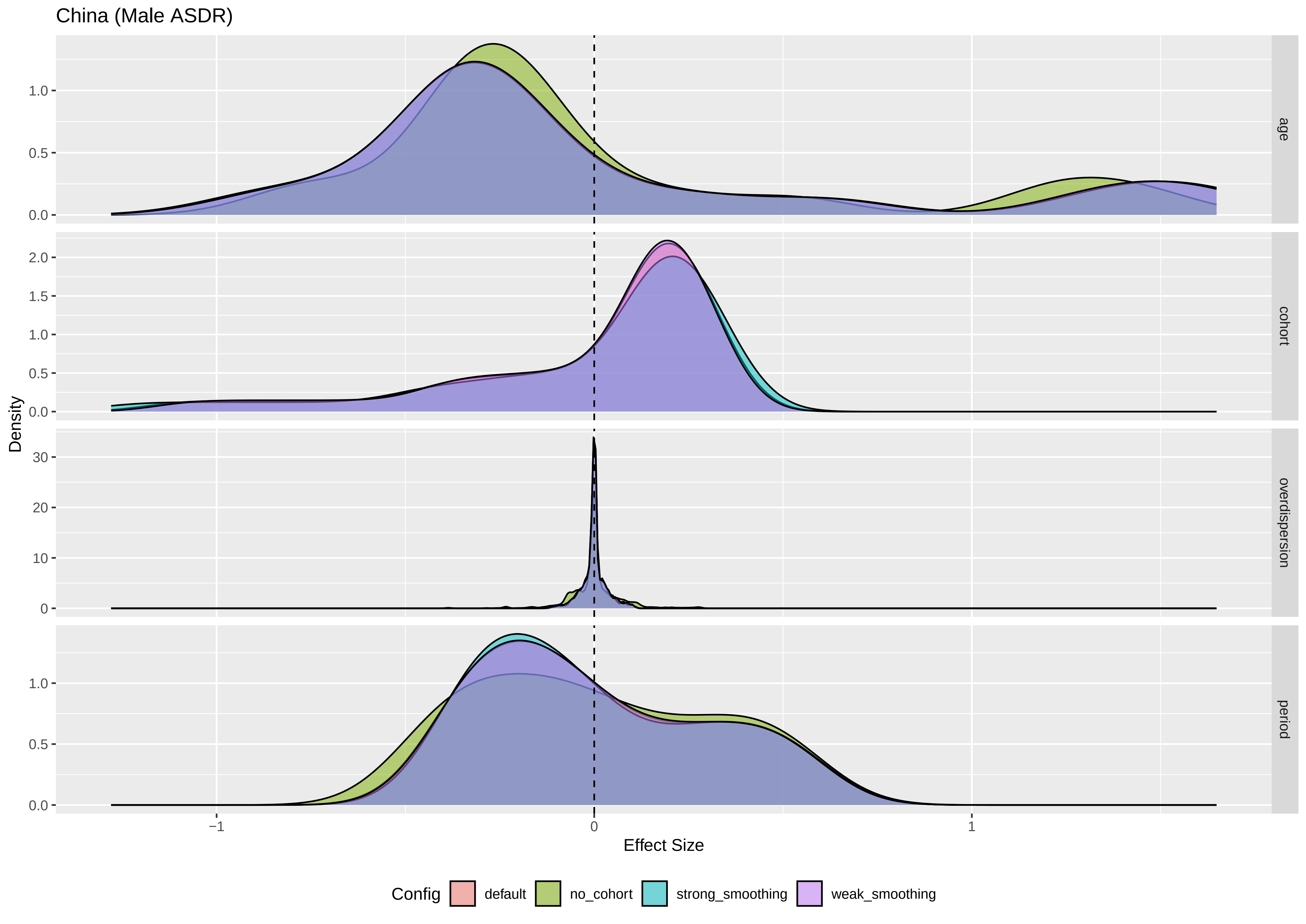
China (Male ASDR)
age
cohort
overdispersion
period
Density
Effect Size
1.0
0.5
0.0
2.0
1.5
30
20
10
0
−1
1
Config
default
no_cohort
strong_smoothing
weak_smoothing

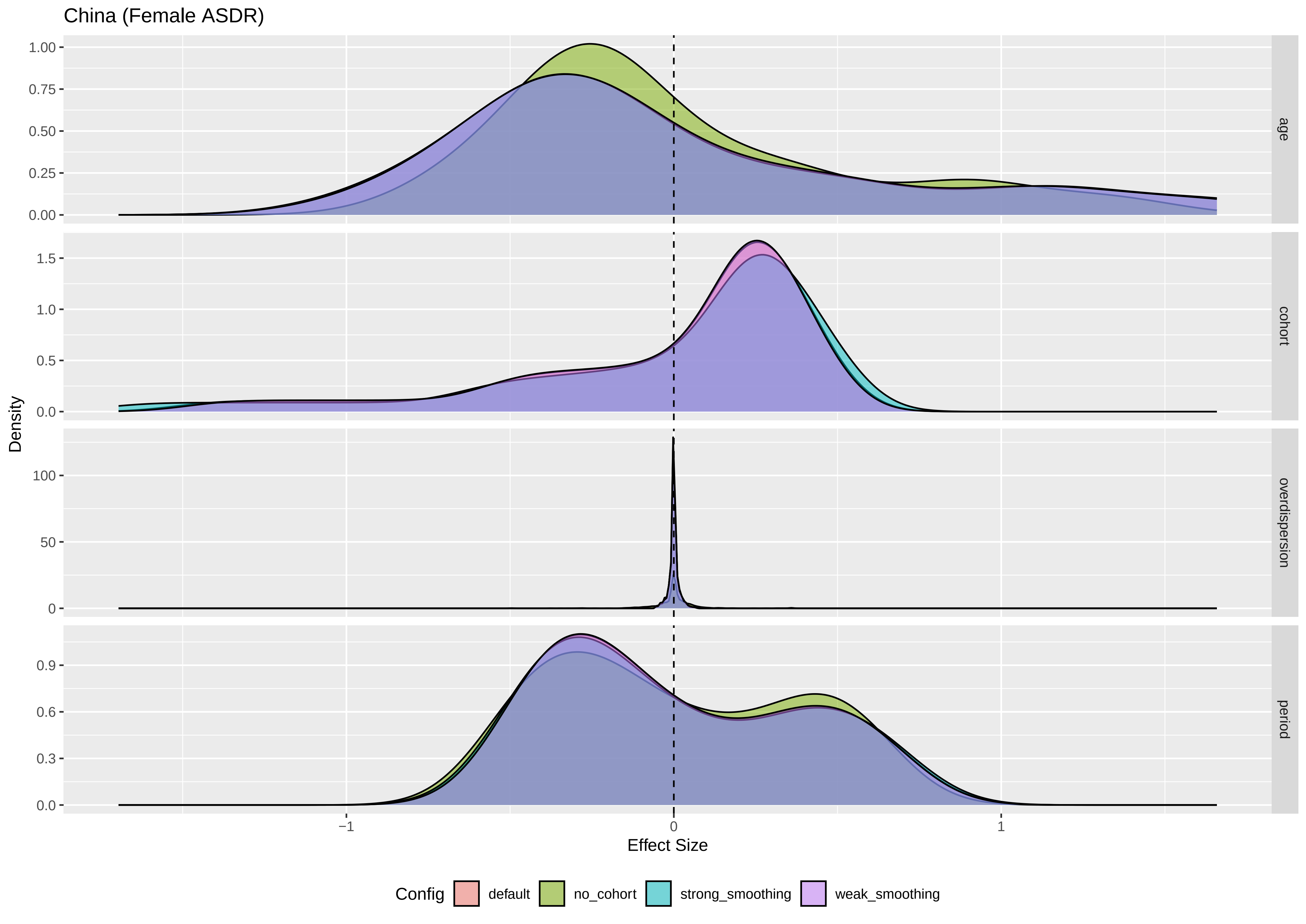

China (Female ASDR)
age
cohort
overdispersion
period
Density
Effect Size
1.00
0.75
0.50
0.25
0.00
1.5
1.0
0.5
0.0
100
50
0
0.9
0.6
0.3
0.0
−1
0
1
Config
default
no_cohort
strong_smoothing
weak_smoothing

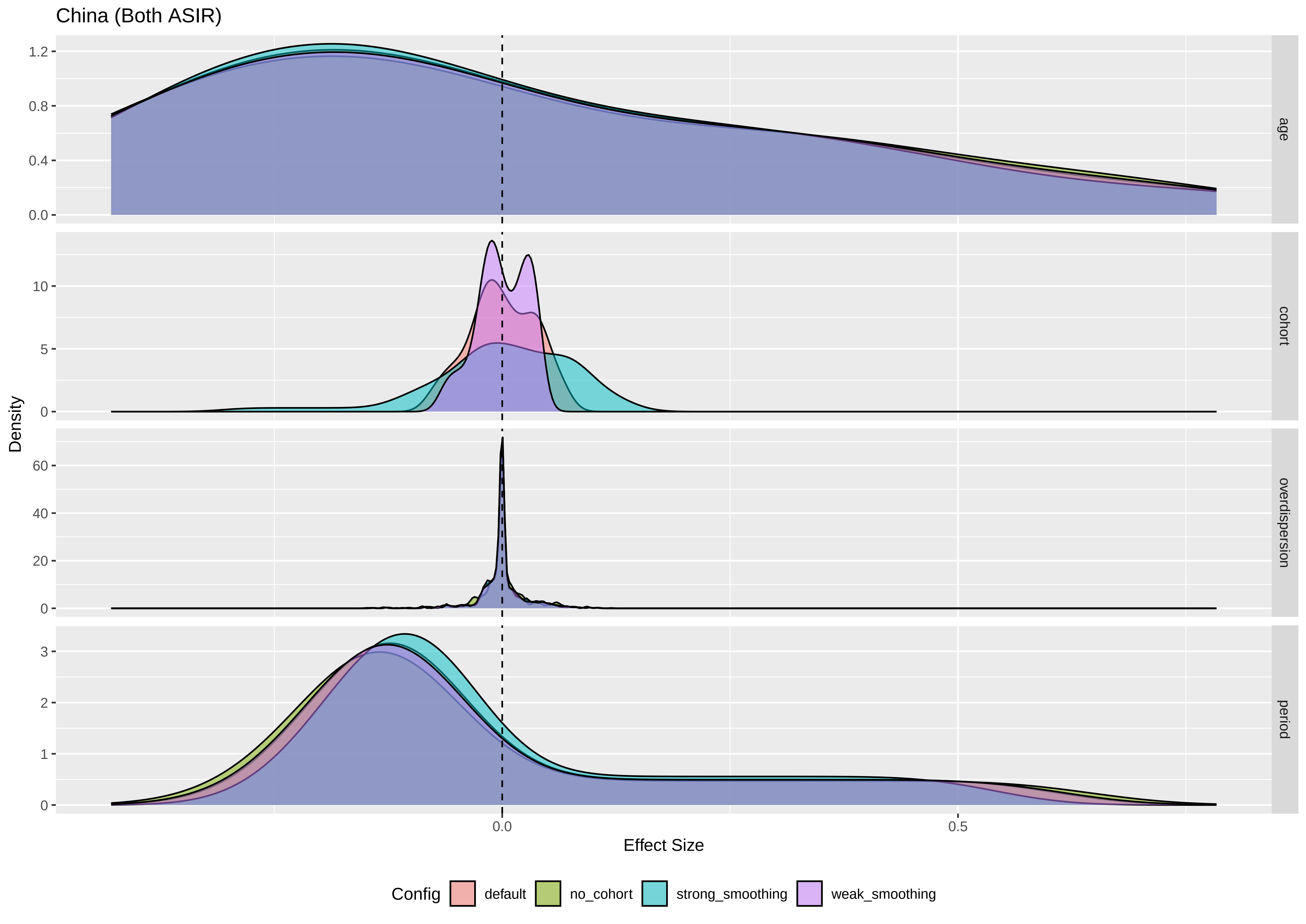

China (Both ASIR)
Density
Effect Size
age
cohort
overdispersion
period
0.0
0.4
0.8
1.2
0
5
10
0
20
40
60
0
1
2
3
0.0
0.5
Config
default
no_cohort
strong_smoothing
weak_smoothing

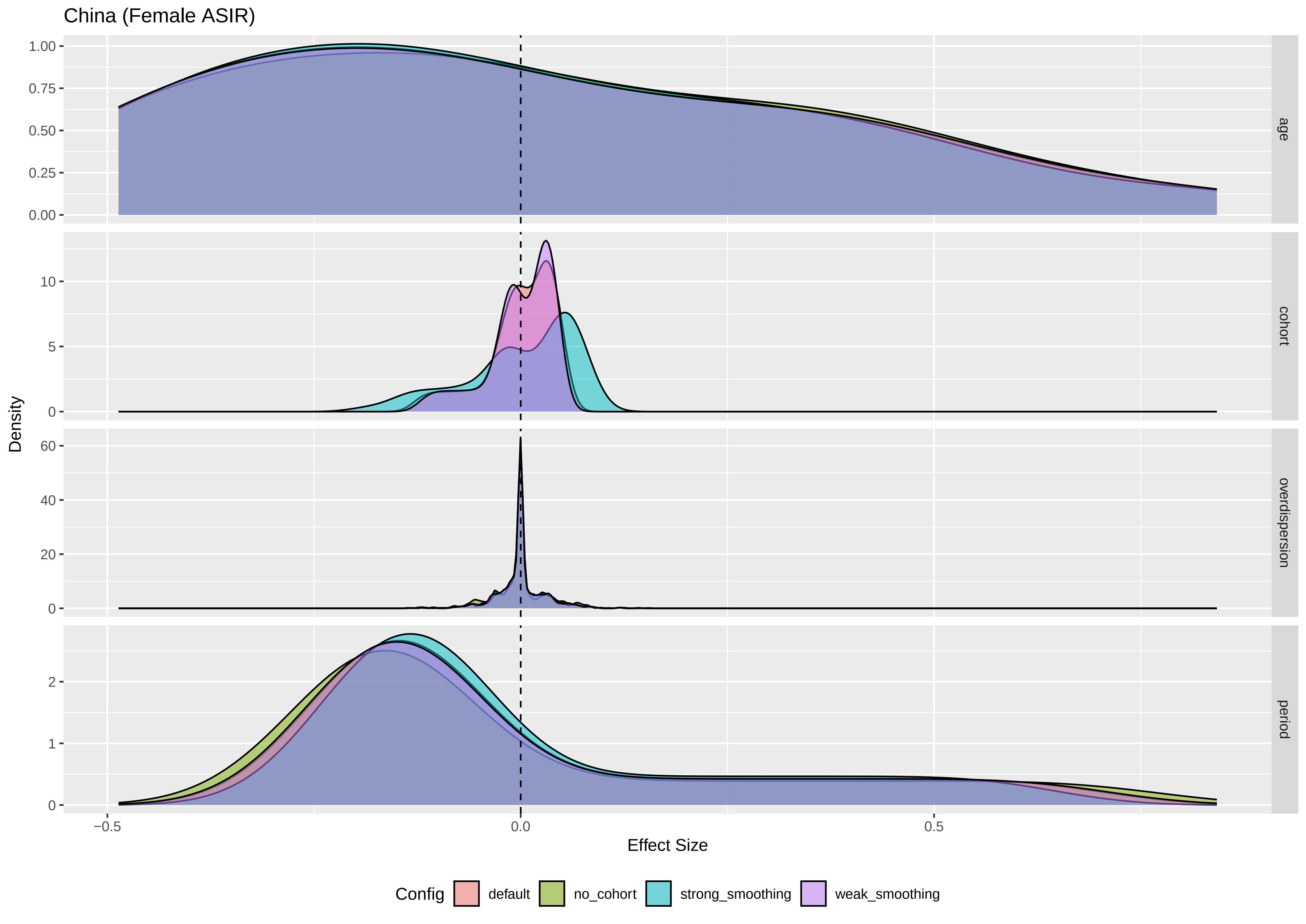

China (Female ASIR)
age
cohort
overdispersion
period
Density
Effect Size
1.00
0.75
0.50
0.25
0.00
10
5
0
60
40
20
0
2
1
0
−0.5
0.0
0.5
Config
default
no_cohort
strong_smoothing
weak_smoothing

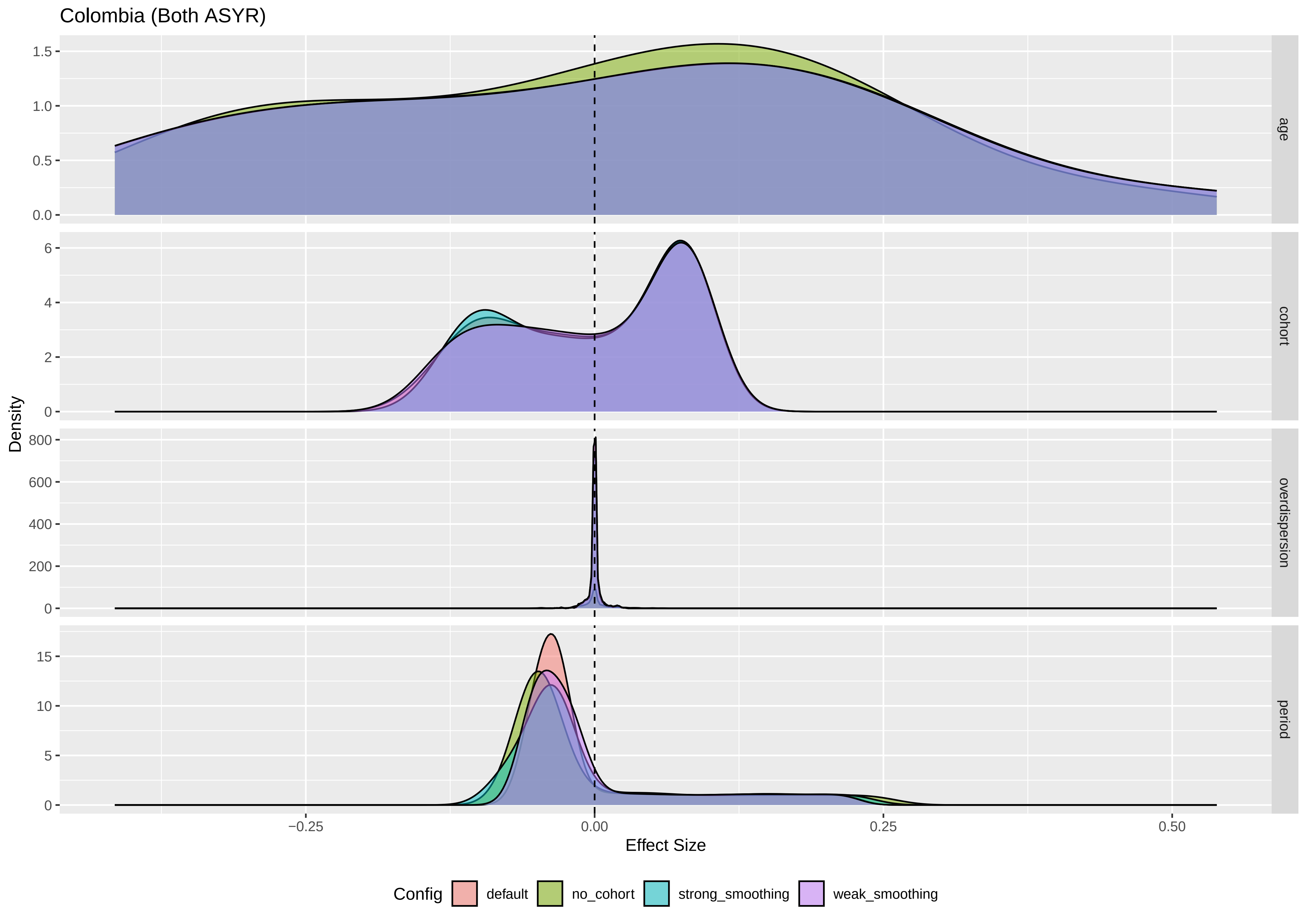

Colombia (Both ASYR)
age
cohort
overdispersion
period
Density
Effect Size
−0.25
0.00
0.25
0.50
0.0
0.5
1.0
1.5
0
2
4
6
0
200
400
600
800
0
5
10
15
Config
default
no_cohort
strong_smoothing
weak_smoothing

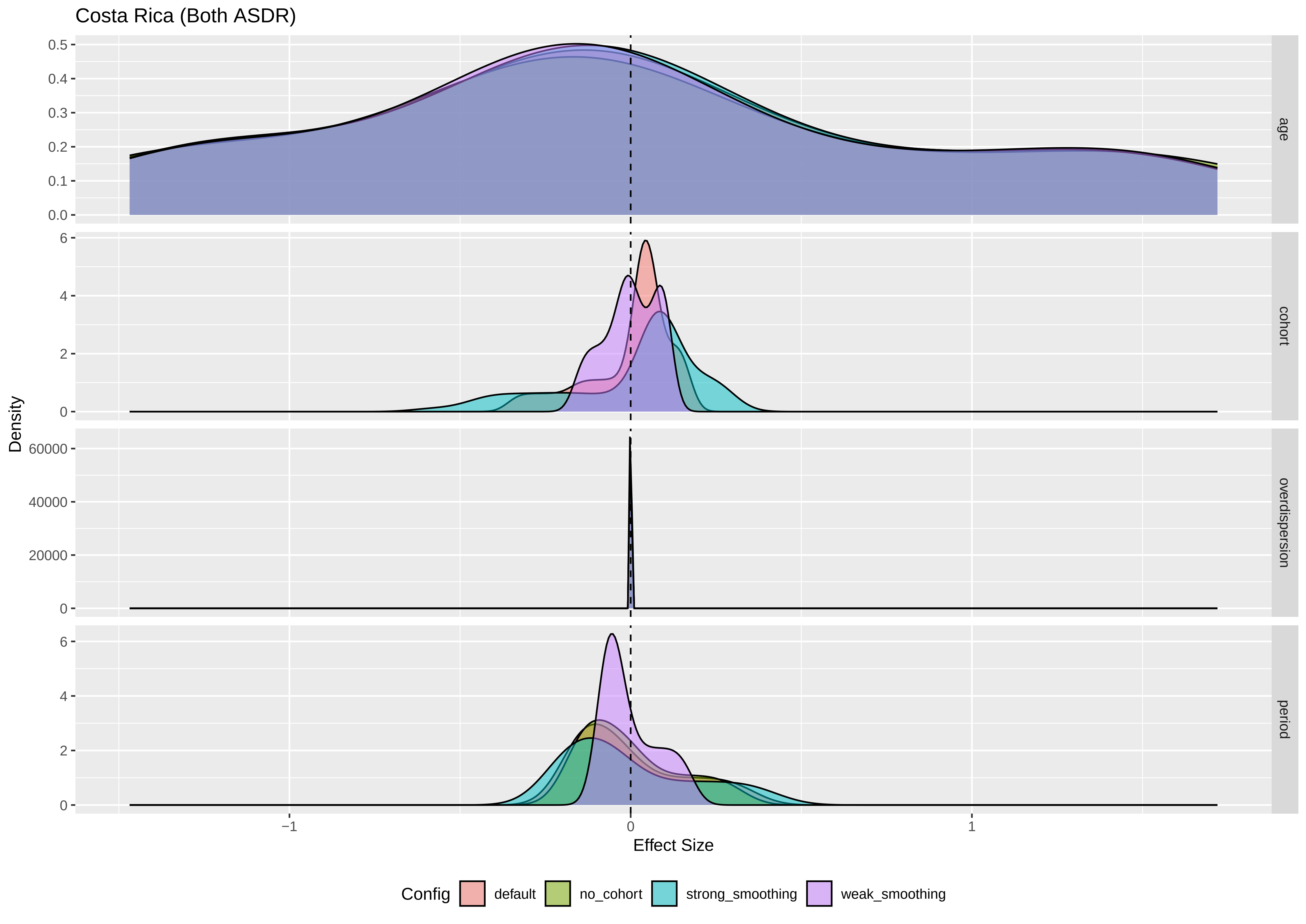

Costa Rica (Both ASDR)
Density
Effect Size
age
cohort
overdispersion
period
0.0
0.1
0.2
0.3
0.4
0.5
0
2
4
6
0
20000
40000
60000
−1
0
1
Config
default
no_cohort
strong_smoothing
weak_smoothing

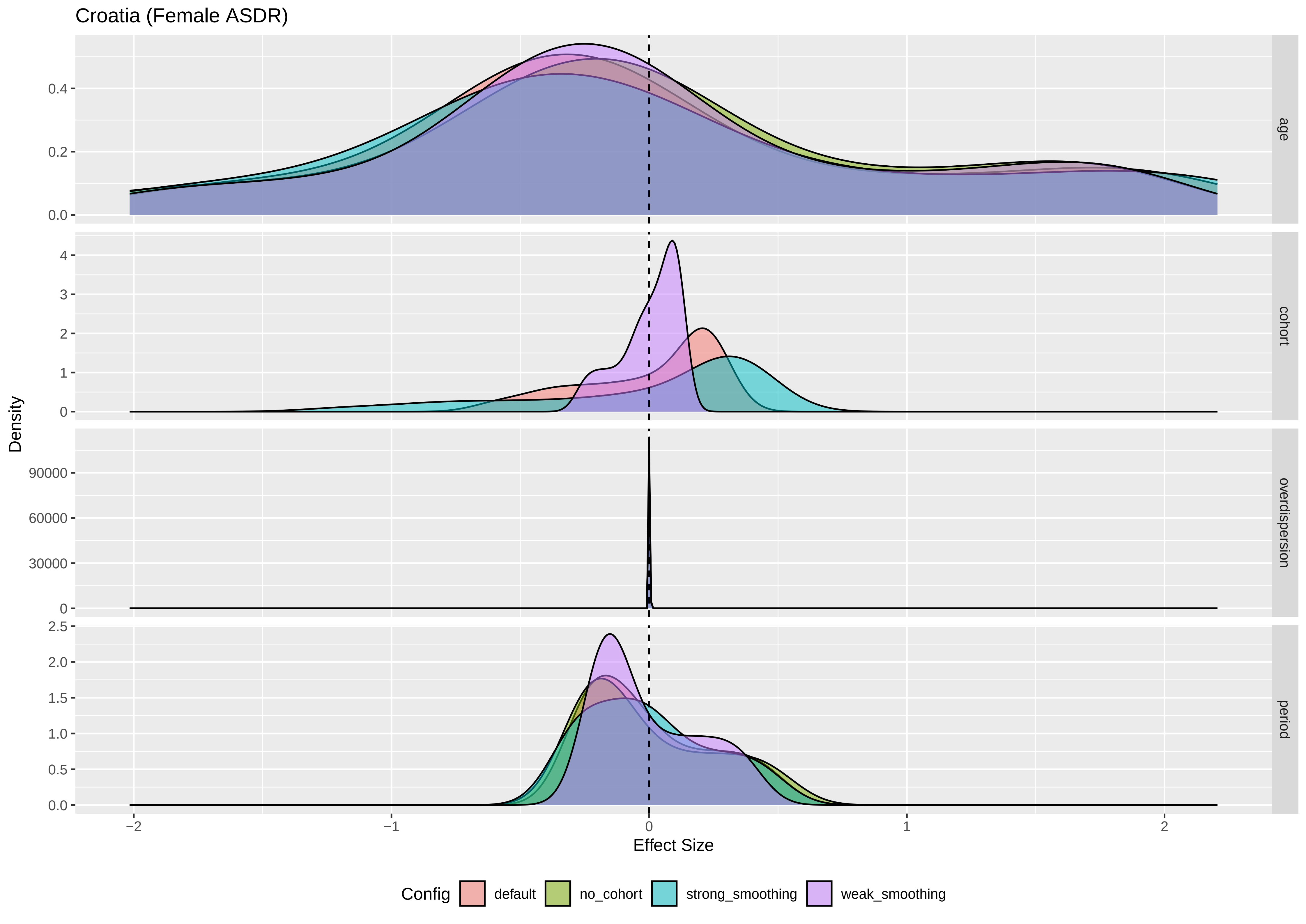

Croatia (Female ASDR)
Density
0.4
0.2
0.0
4
3
2
1
0
90000
60000
30000
0
2.5
2.0
1.5
1.0
0.5
0.0
age
cohort
overdispersion
period
−2
−1
0
1
2
Effect Size
Config
default
no_cohort
strong_smoothing
weak_smoothing

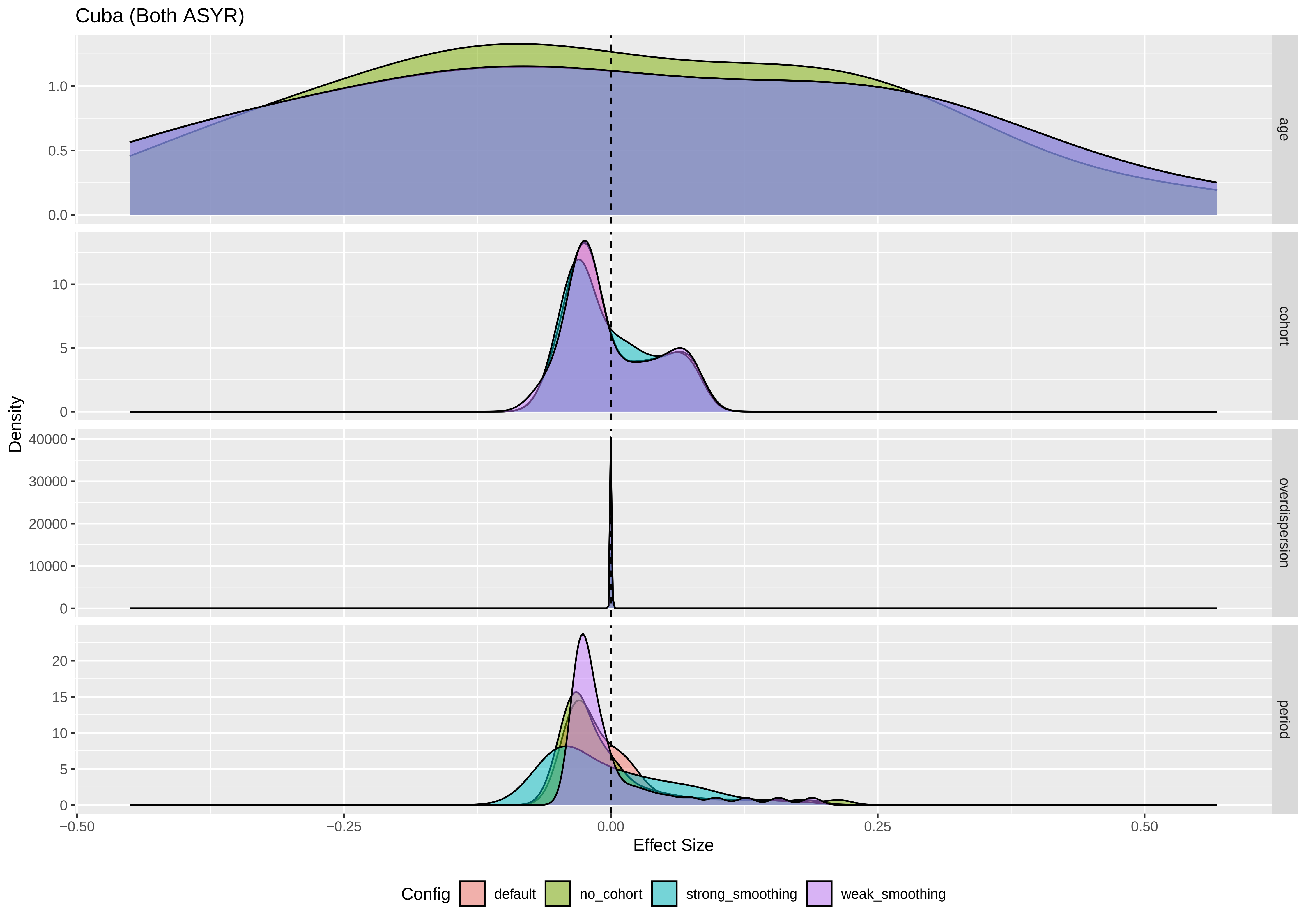

Cuba (Both ASYR)
age
cohort
overdispersion
period
Density
Effect Size
1.0
0.5
0.0
10
5
0
40000
30000
20000
10000
0
20
15
10
5
0
−0.50
−0.25
0.00
0.25
0.50
Config
default
no_cohort
strong_smoothing
weak_smoothing

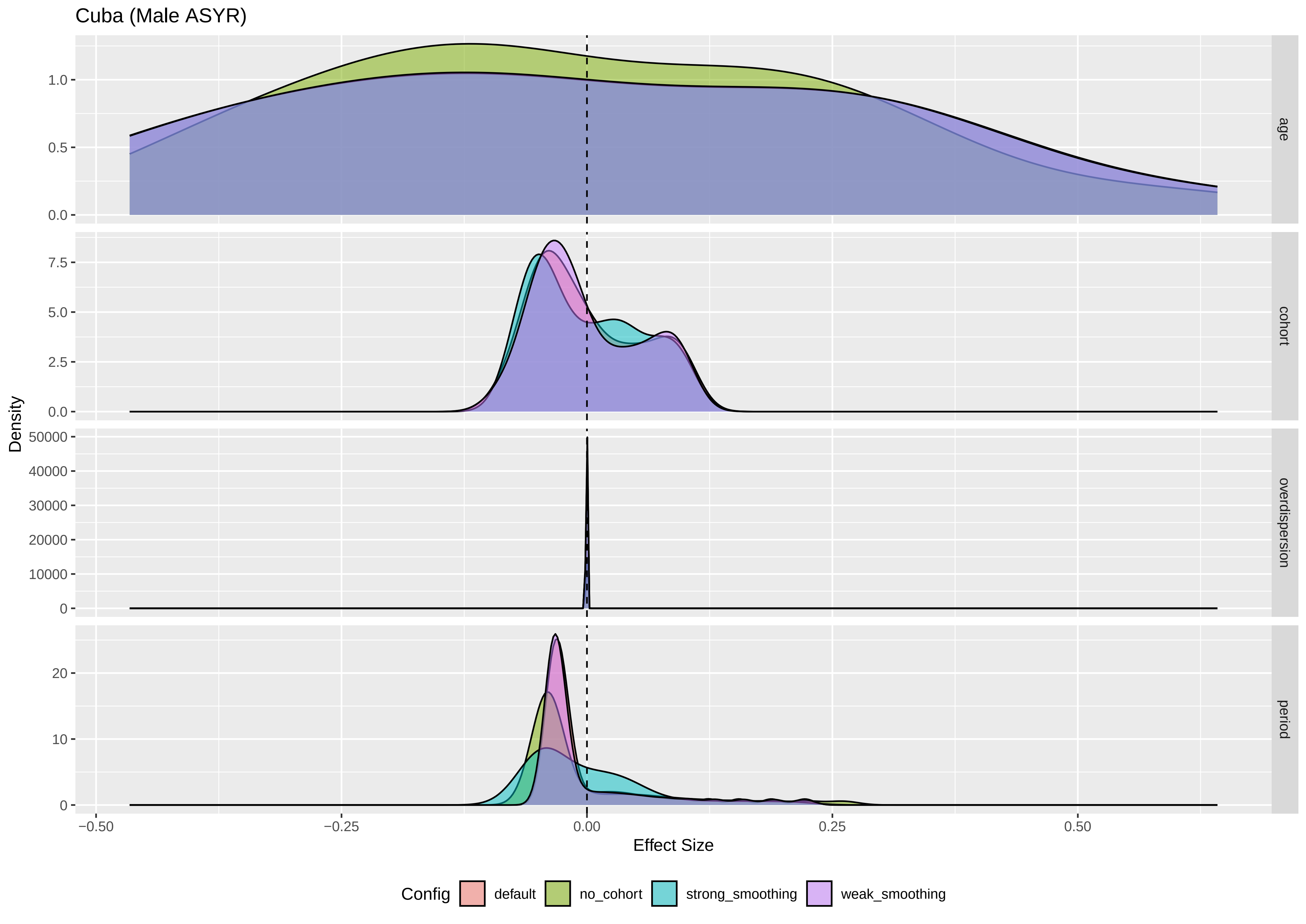
Cuba (Male ASYR)
Density
Effect Size
age
cohort
overdispersion
period
1.0
0.5
0.0
7.5
5.0
2.5
0.0
50000
40000
30000
20000
10000
0
20
10
0
−0.50
−0.25
0.00
0.25
0.50
Config
default
no_cohort
strong_smoothing
weak_smoothing

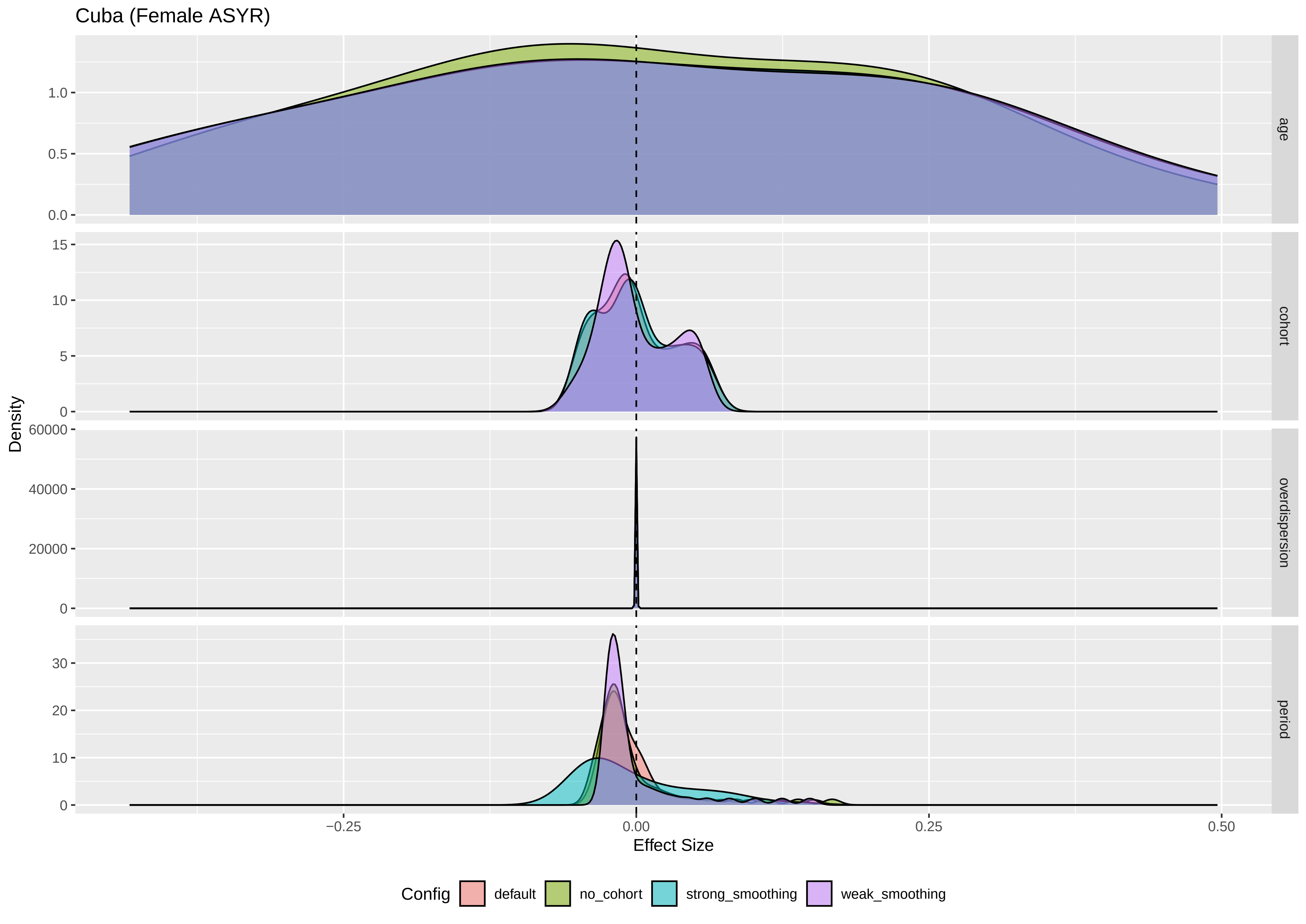
Cuba (Female ASYR)
Density
Effect Size
age
cohort
overdispersion
period
1.0
0.5
0.0
15
10
5
0
60000
40000
20000
0
30
20
10
0
−0.25
0.00
0.25
0.50
Config
default
no_cohort
strong_smoothing
weak_smoothing

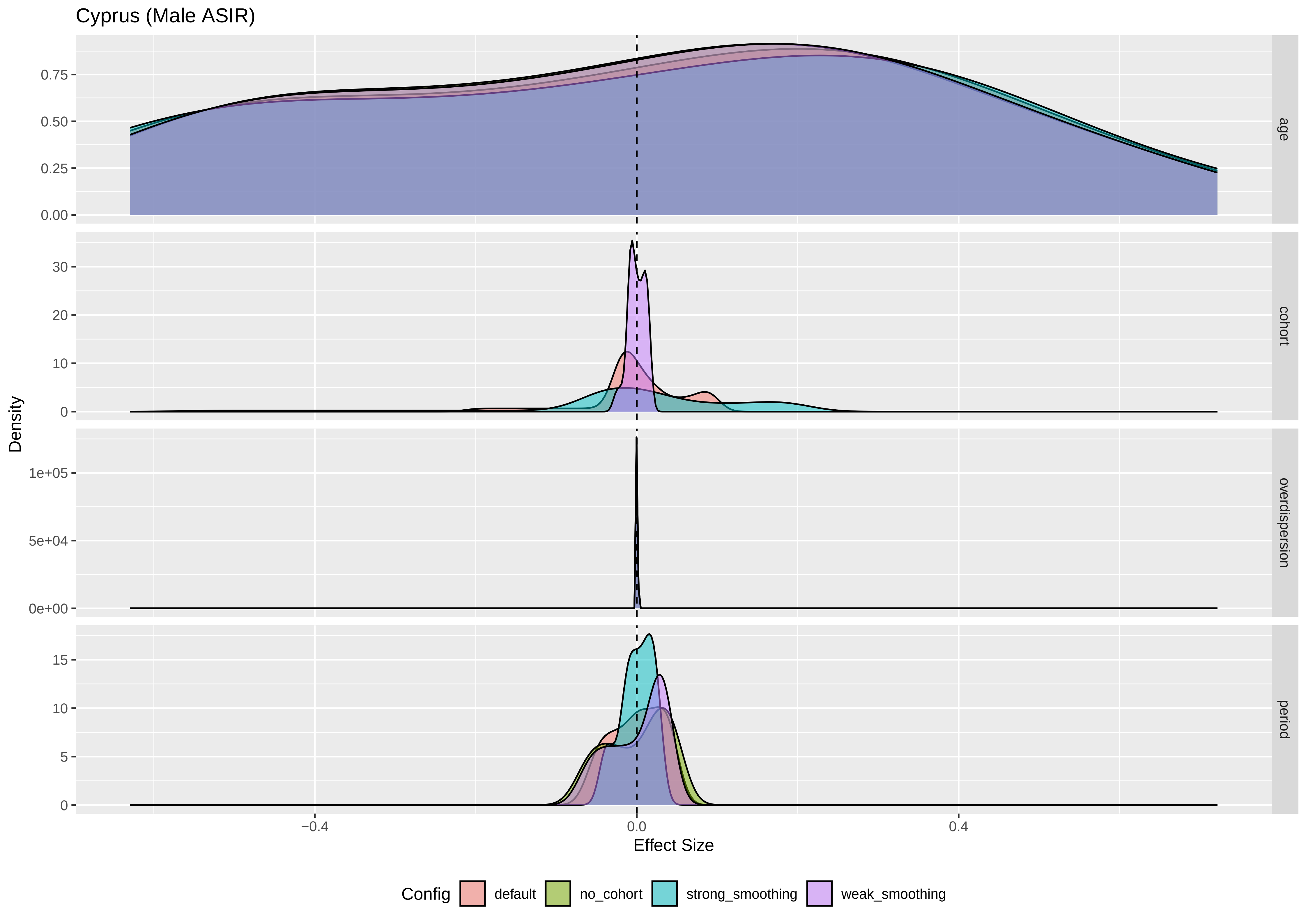

Cyprus (Male ASIR)
age
cohort
overdispersion
period
Density
Effect Size
0.00
0.25
0.50
0.75
0
10
20
30
0e+00
5e+04
1e+05
0
5
10
15
−0.4
0.0
0.4
Config
default
no_cohort
strong_smoothing
weak_smoothing

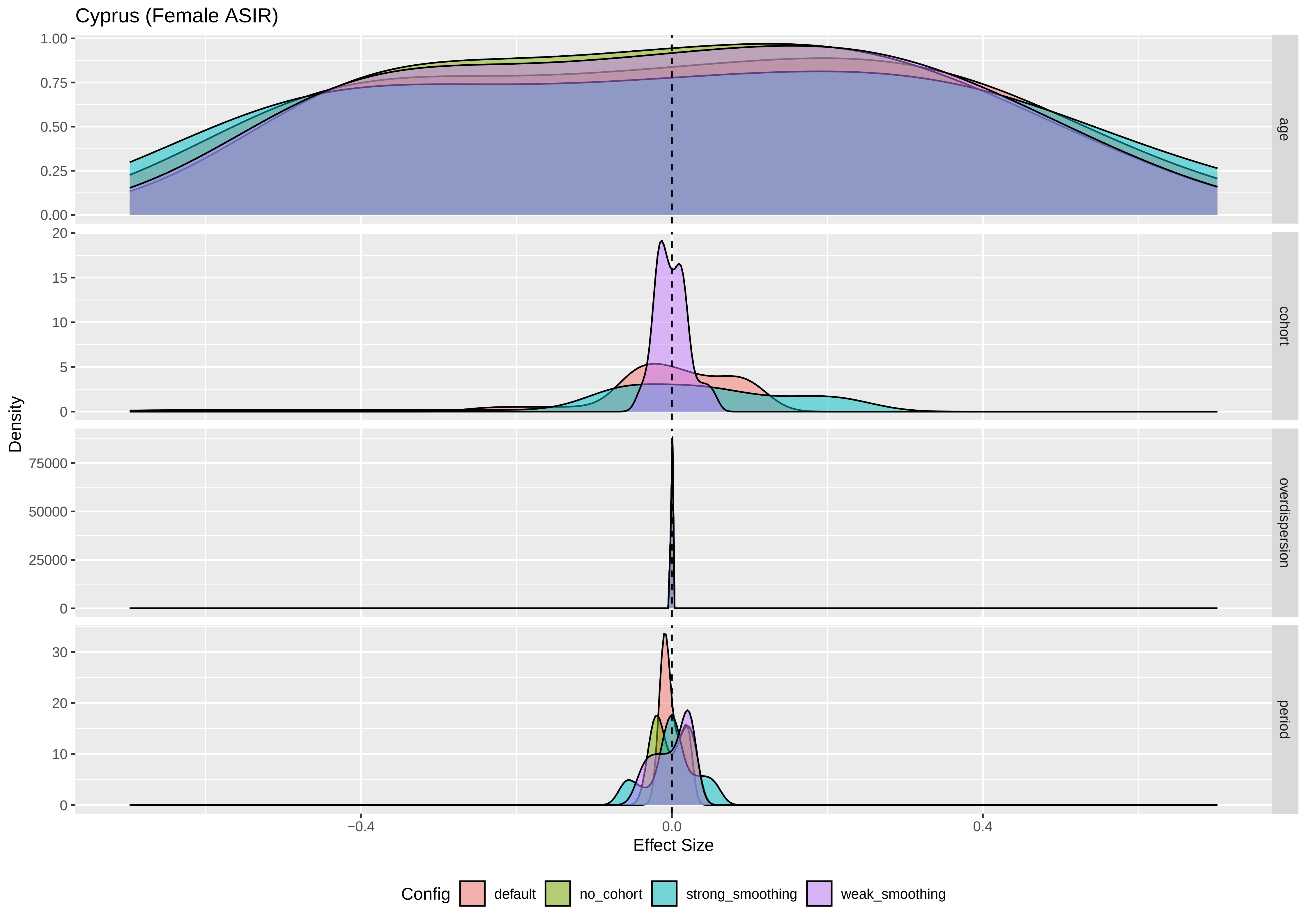

Cyprus (Female ASIR)
1.00
0.75
0.50
0.25
0.00
age
20
15
10
5
0
cohort
75000
50000
25000
0
overdispersion
30
20
10
0
period
Density
−0.4
0.0
0.4
Effect Size
Config
default
no_cohort
strong_smoothing
weak_smoothing

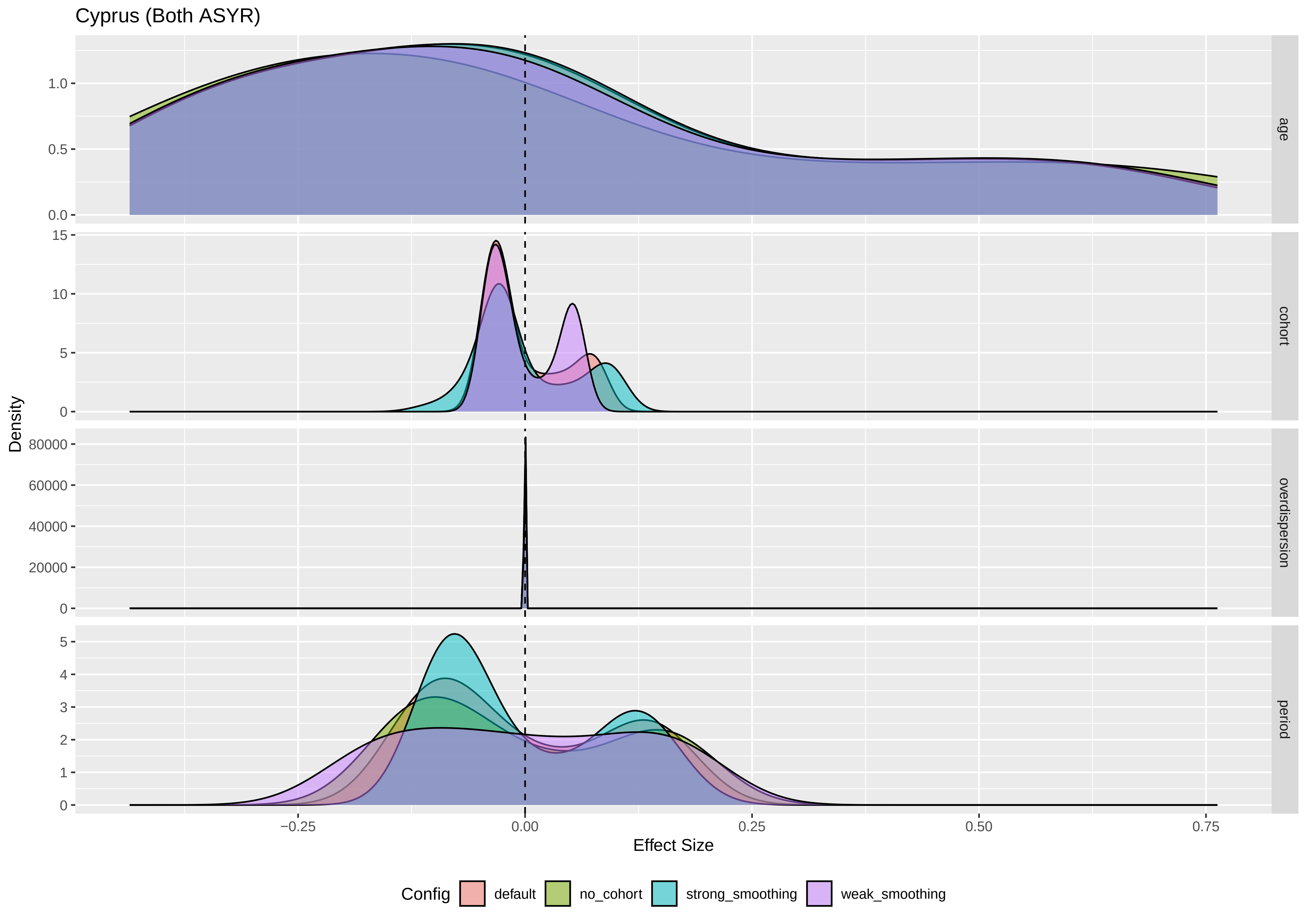

Cyprus (Both ASYR)
age
cohort
overdispersion
period
Density
Effect Size
−0.25
0.00
0.25
0.50
0.75
0.0
0.5
1.0
0
5
10
15
0
20000
40000
60000
80000
0
1
2
3
4
5
Config
default
no_cohort
strong_smoothing
weak_smoothing

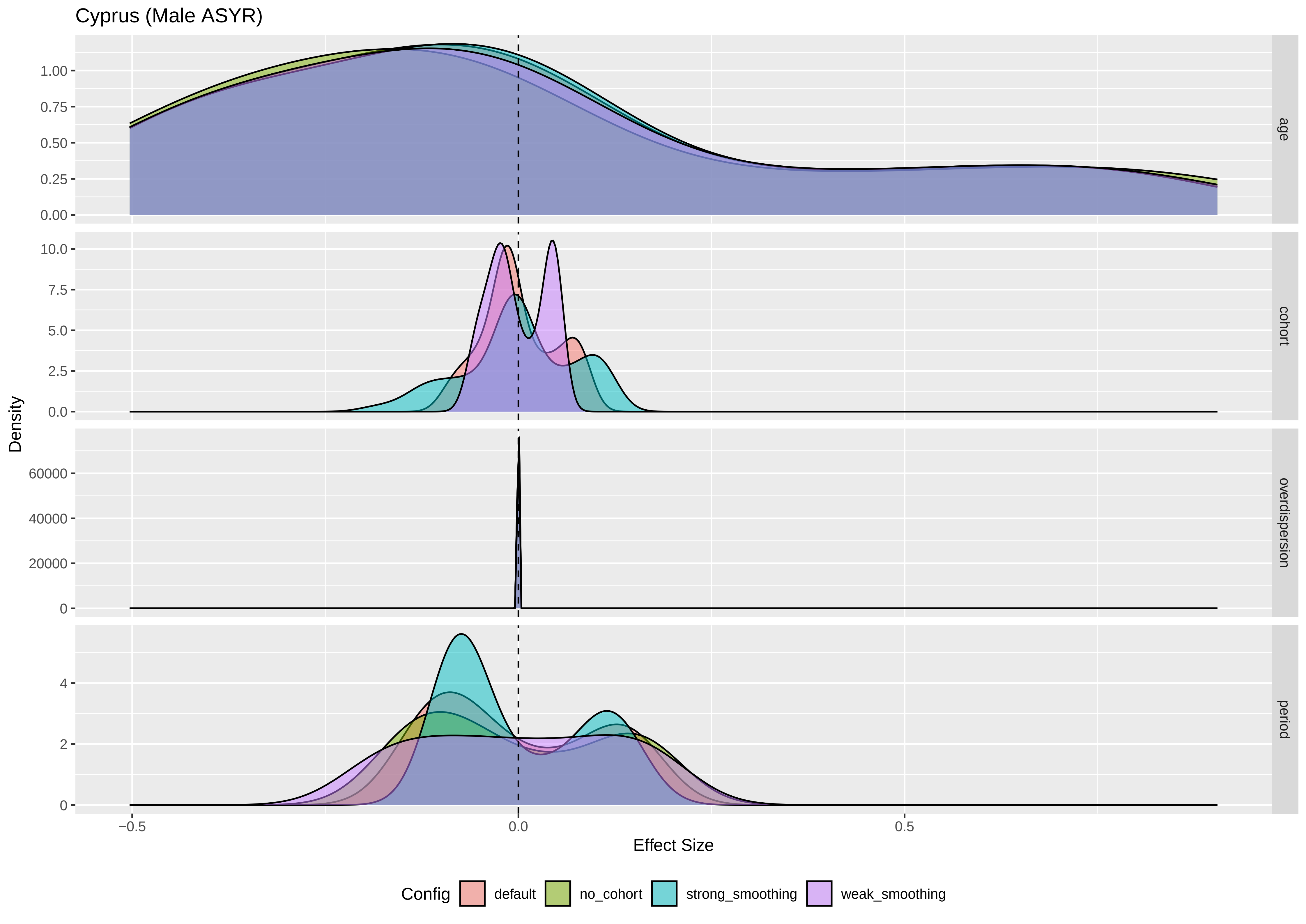

Cyprus (Male ASYR)
age
cohort
overdispersion
period
Density
Effect Size
-0.5
0.0
0.5
1.00
0.75
0.50
0.25
0.00
10.0
7.5
5.0
2.5
0.0
60000
40000
20000
0
4
2
0
Config
default
no_cohort
strong_smoothing
weak_smoothing

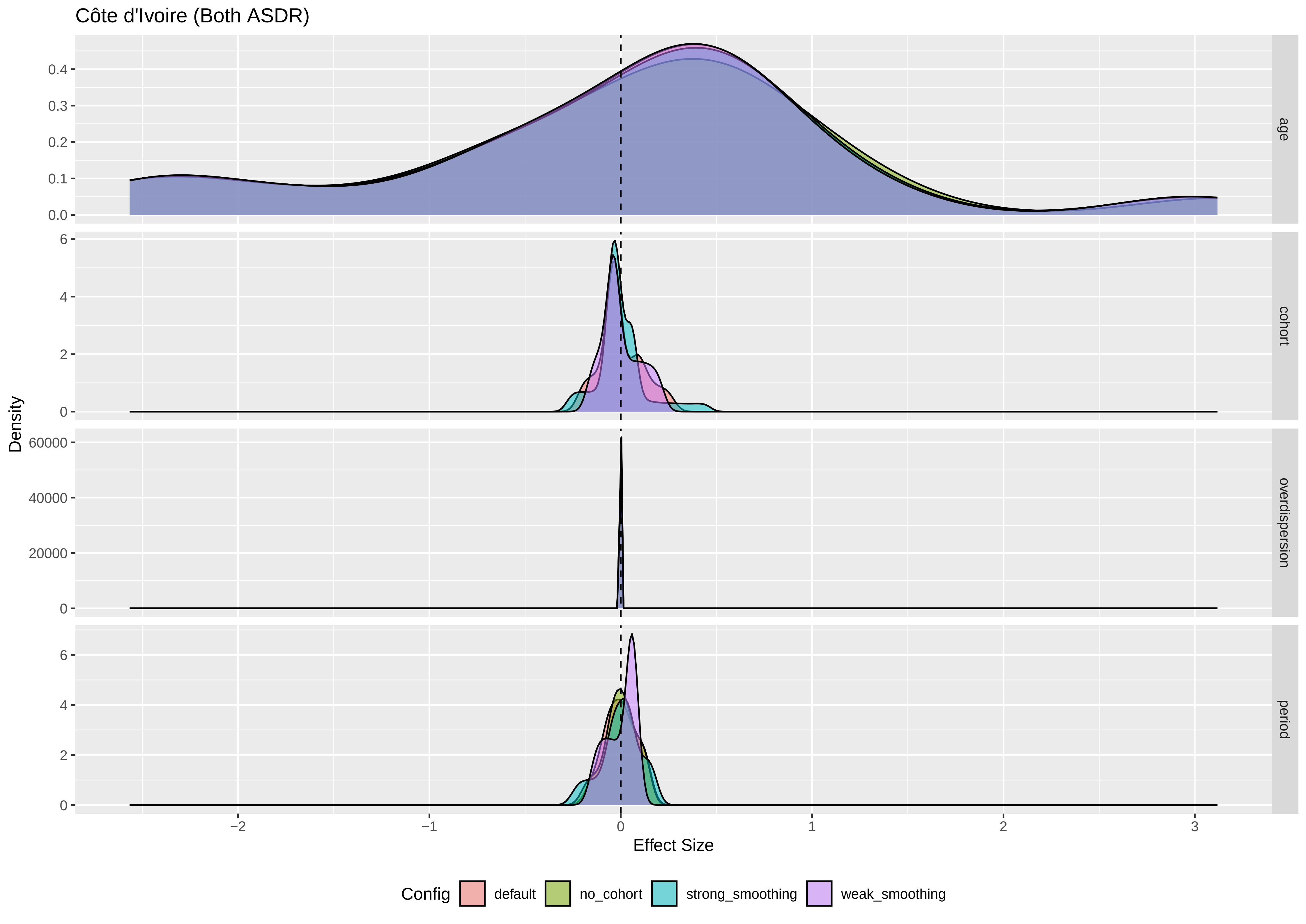

Côte d'Ivoire (Both ASDR)
Density
Effect Size
age
cohort
overdispersion
period
0.0
0.1
0.2
0.3
0.4
0
2
4
6
0
20000
40000
60000
0
2
4
6
−2
−1
0
1
2
3
Config
default
no_cohort
strong_smoothing
weak_smoothing

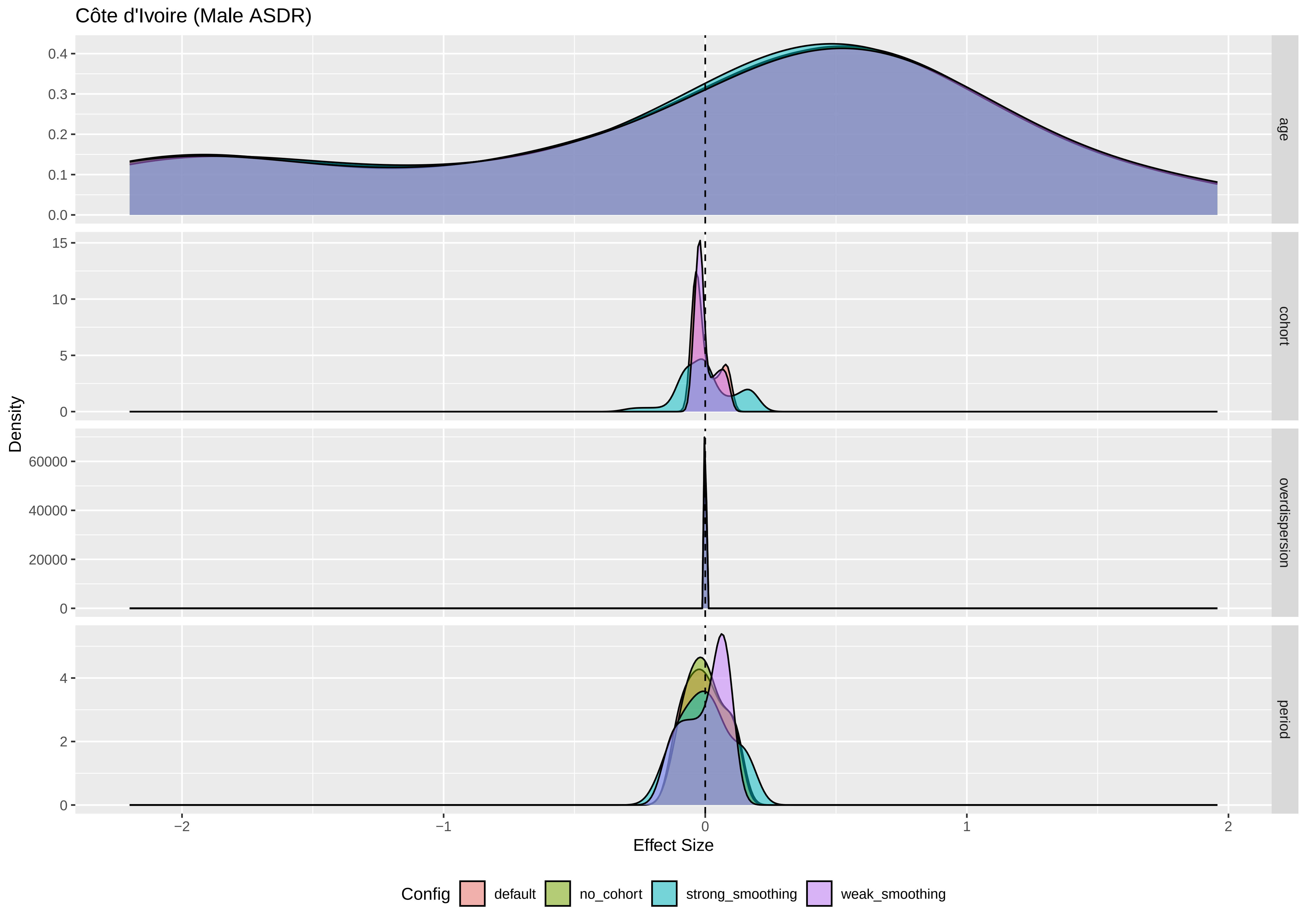

Côte d'Ivoire (Male ASDR)
Density
Effect Size
age
cohort
overdispersion
period
0.0
0.1
0.2
0.3
0.4
0
5
10
15
0
20000
40000
60000
0
2
4
−2
−1
0
1
2
Config
default
no_cohort
strong_smoothing
weak_smoothing

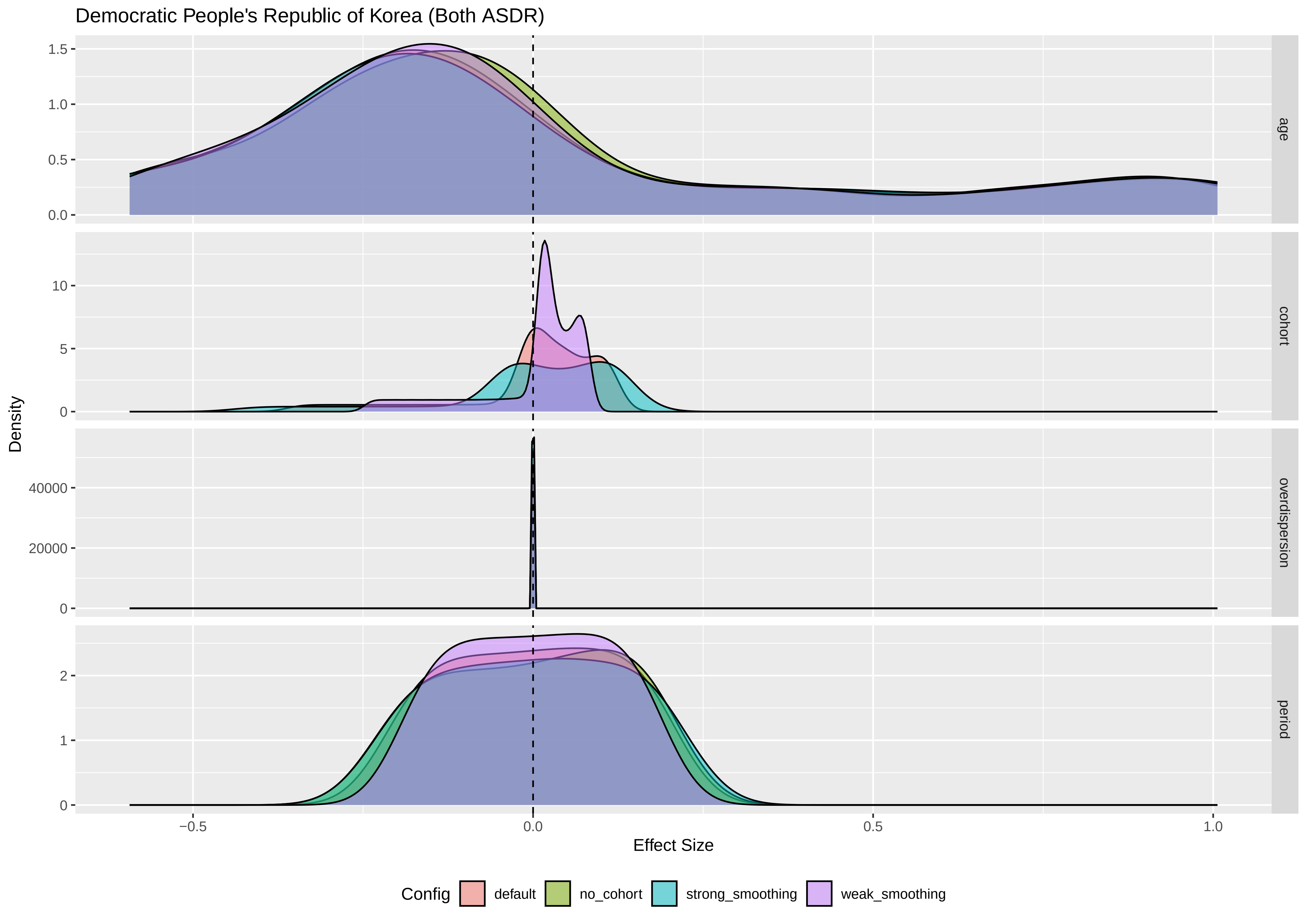

Democratic People's Republic of Korea (Both ASDR)
Density
Effect Size
age
cohort
overdispersion
period
1.5
1.0
0.5
0.0
10
5
0
40000
20000
0
2
1
0
−0.5
0.0
0.5
1.0
Config
default
no_cohort
strong_smoothing
weak_smoothing

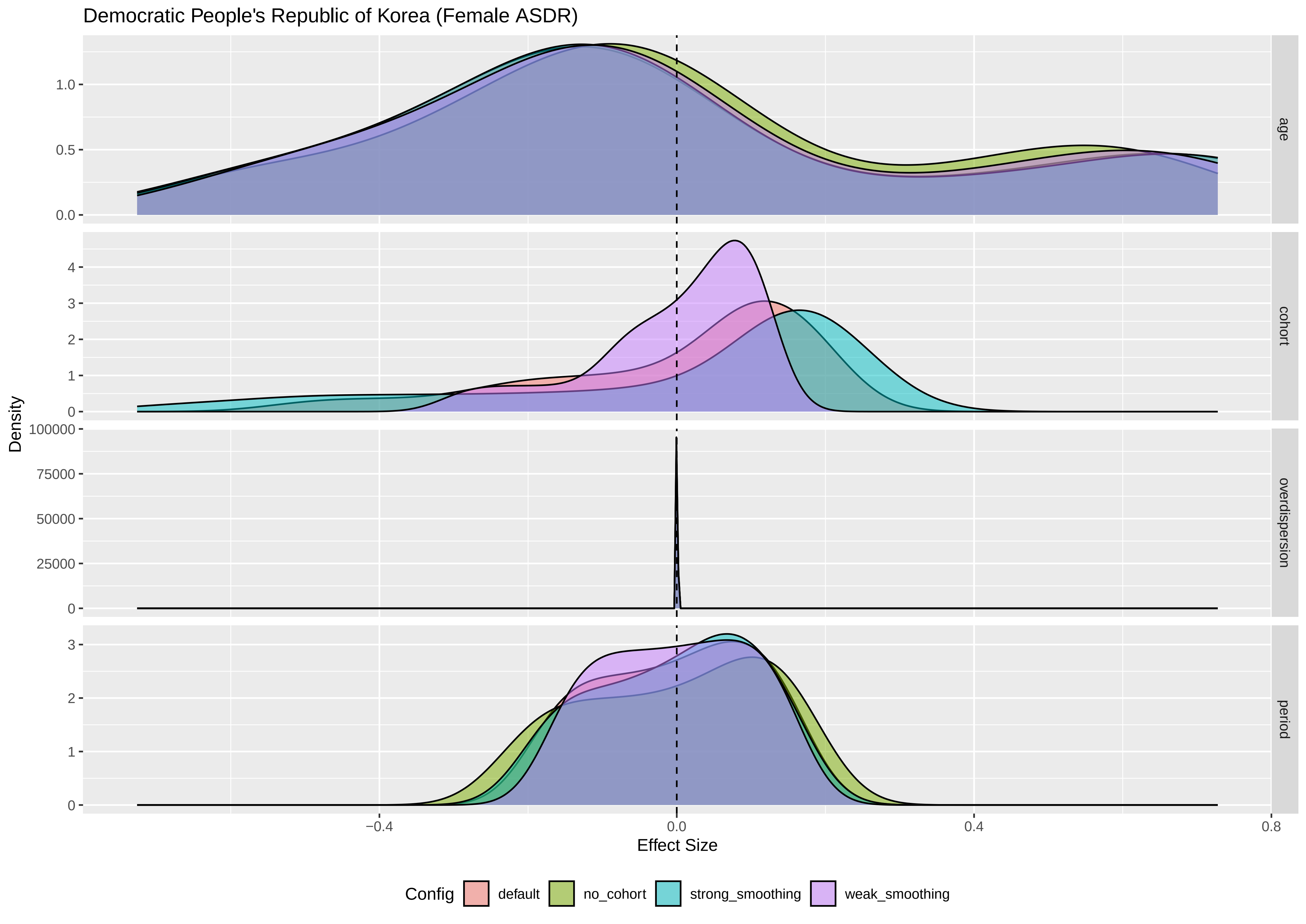

Democratic People's Republic of Korea (Female ASDR)
Density
Effect Size
age
cohort
overdispersion
period
1.0
0.5
0.0
4
3
2
1
0
100000
75000
50000
25000
0
3
2
1
0
−0.4
0.0
0.4
0.8
Config
default
no_cohort
strong_smoothing
weak_smoothing

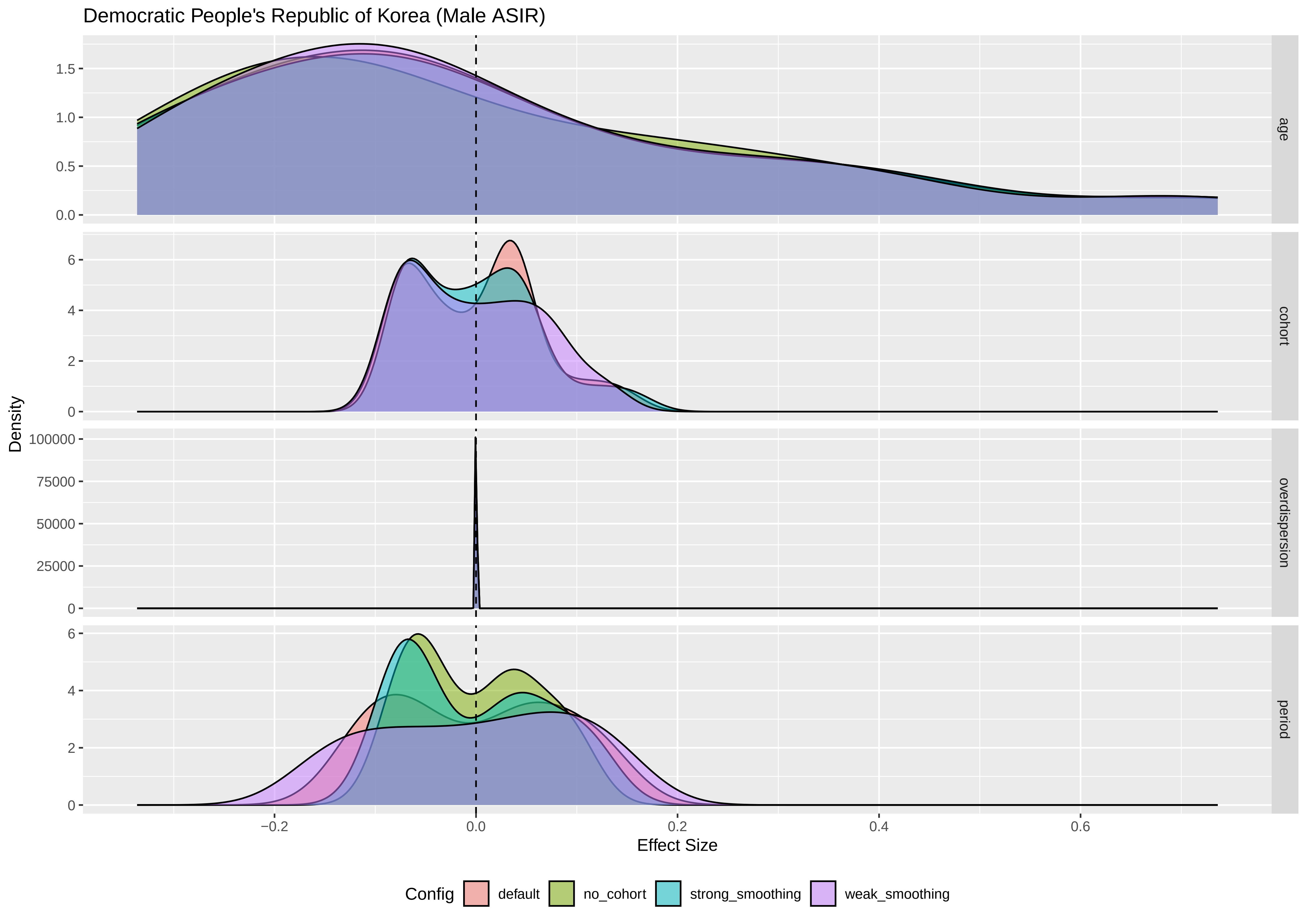
Democratic People's Republic of Korea (Male ASIR)
Density
Effect Size
age
cohort
overdispersion
period
1.5
1.0
0.5
0.0
6
4
2
0
100000
75000
50000
25000
0
6
4
2
0
−0.2
0.0
0.2
0.4
0.6
Config
default
no_cohort
strong_smoothing
weak_smoothing

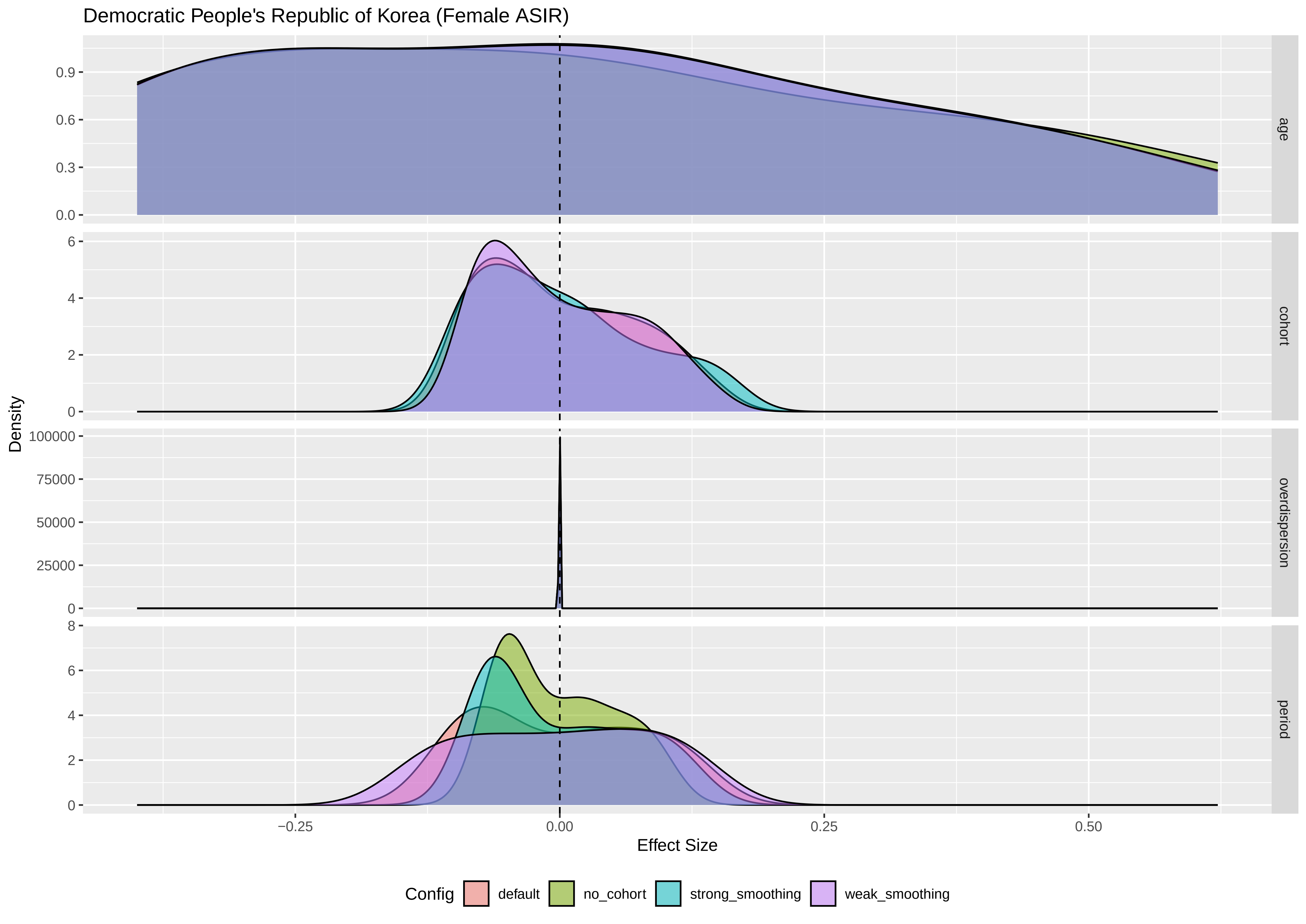

Democratic People's Republic of Korea (Female ASIR)
age
cohort
overdispersion
period
Density
Effect Size
0.0
0.3
0.6
0.9
0
2
4
6
0
25000
50000
75000
100000
8
−0.25
0.00
0.25
0.50
Config
default
no_cohort
strong_smoothing
weak_smoothing

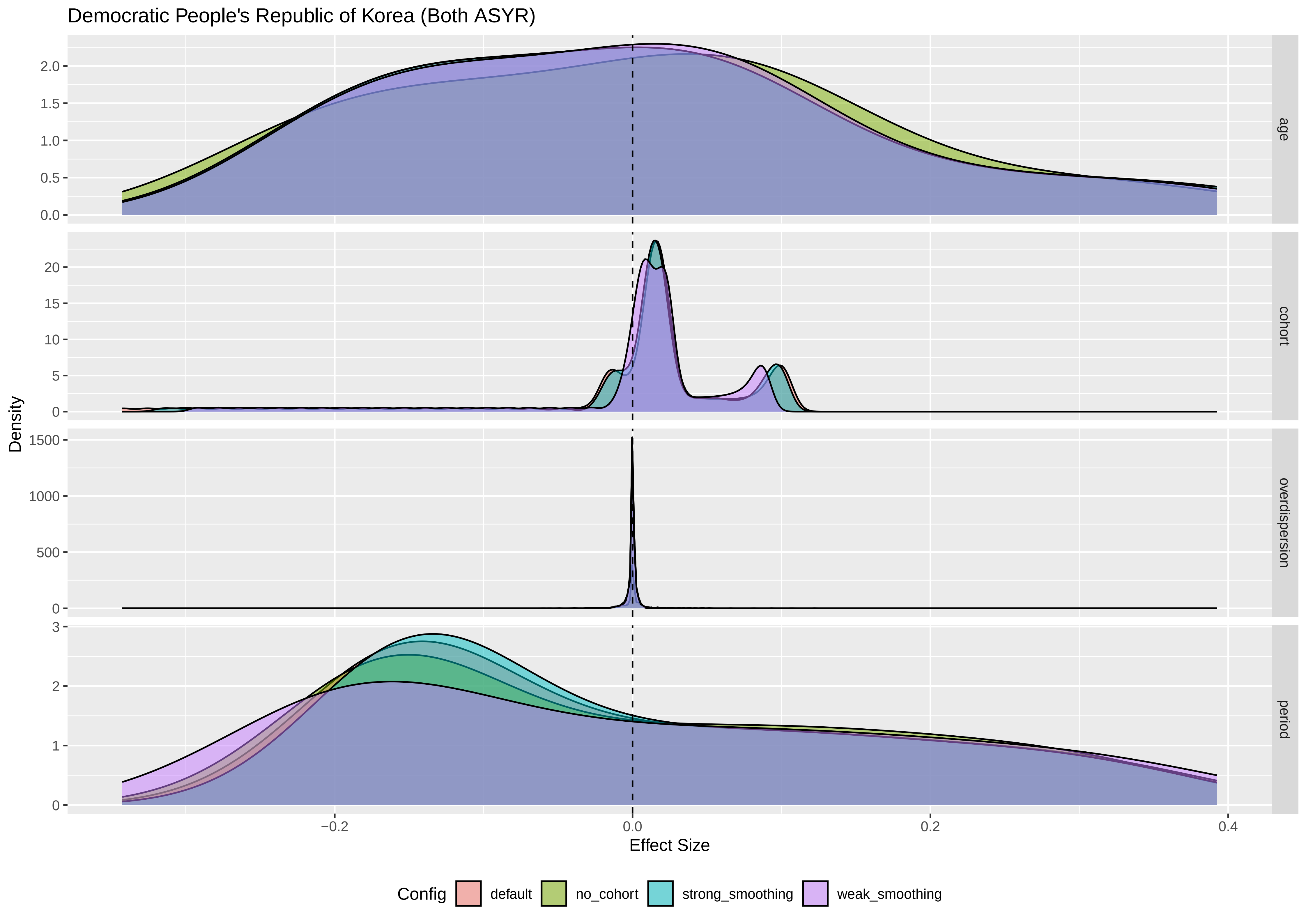

Democratic People's Republic of Korea (Both ASYR)
age
cohort
overdispersion
period
Density
Effect Size
−0.2
0.0
0.2
0.4
2.0
1.5
1.0
0.5
0.0
20
15
10
5
0
1500
1000
500
0
3
2
1
0
Config
default
no_cohort
strong_smoothing
weak_smoothing

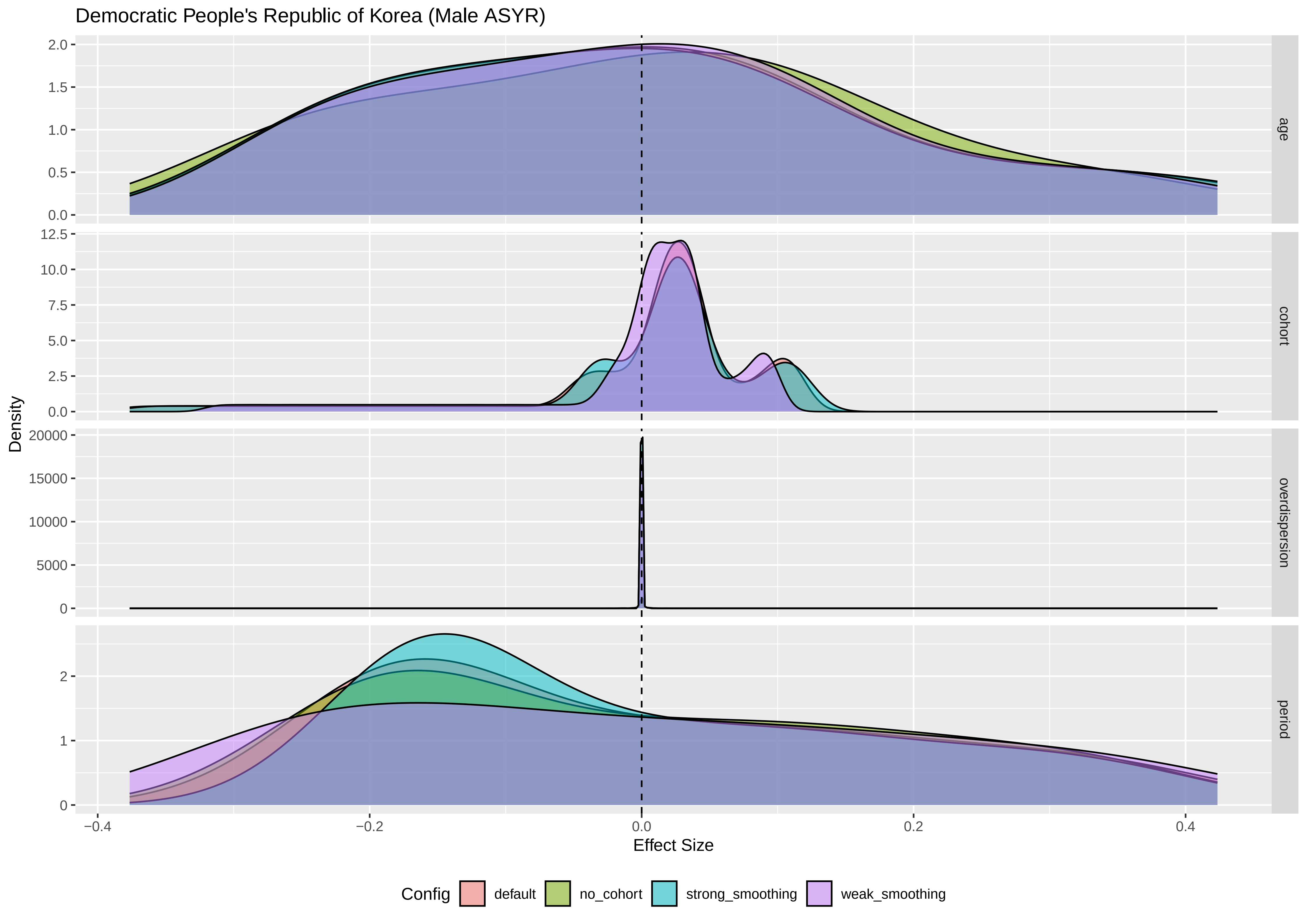

Democratic People's Republic of Korea (Male ASYR)
age
cohort
overdispersion
period
Density
Effect Size
Config
default
no_cohort
strong_smoothing
weak_smoothing

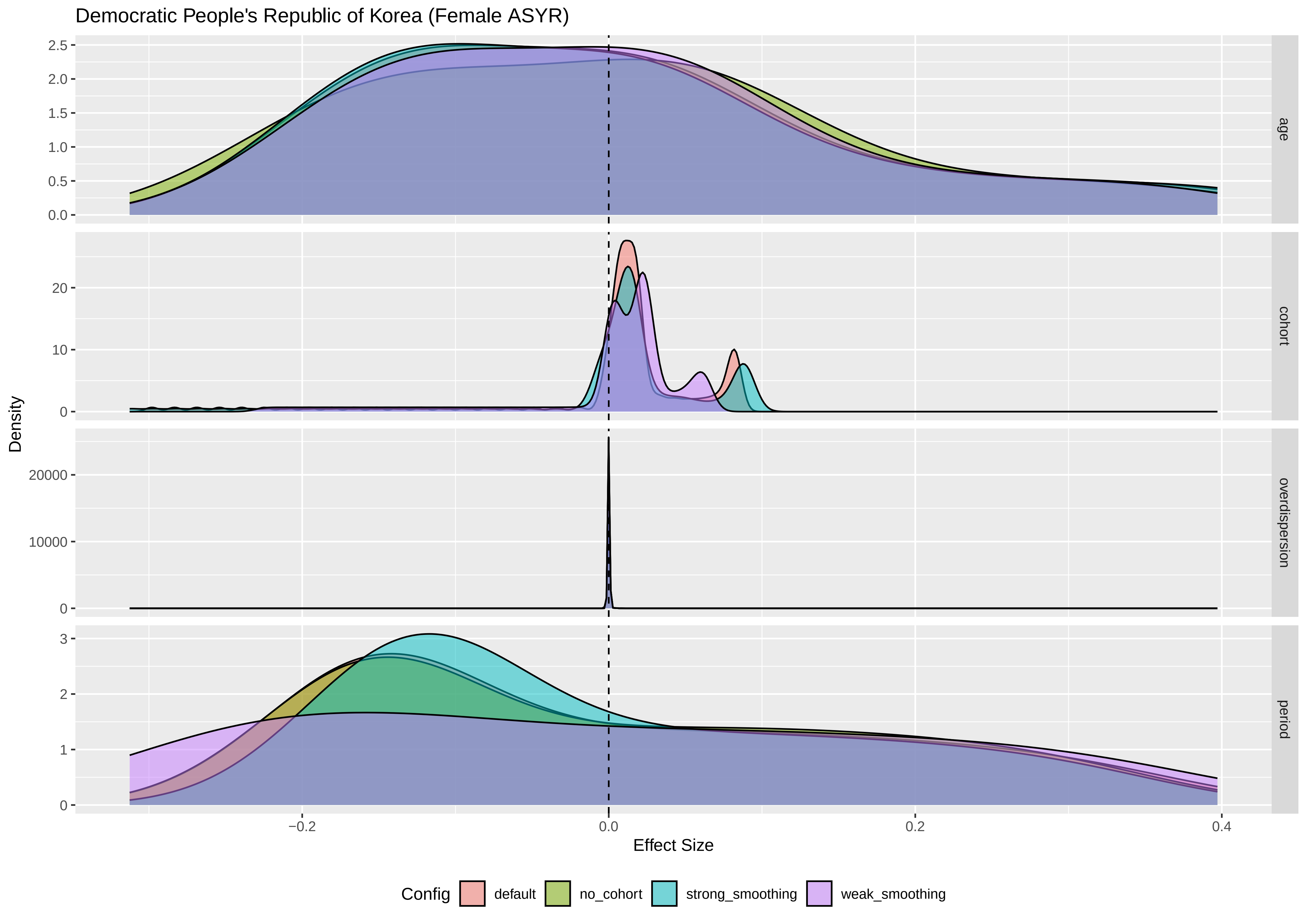
Democratic People's Republic of Korea (Female ASYR)
age
cohort
overdispersion
period
Density
Effect Size
−0.2
0.0
0.2
0.4
2.5
2.0
1.5
1.0
0.5
0.0
20
10
0
20000
10000
0
3
2
1
0
Config
default
no_cohort
strong_smoothing
weak_smoothing

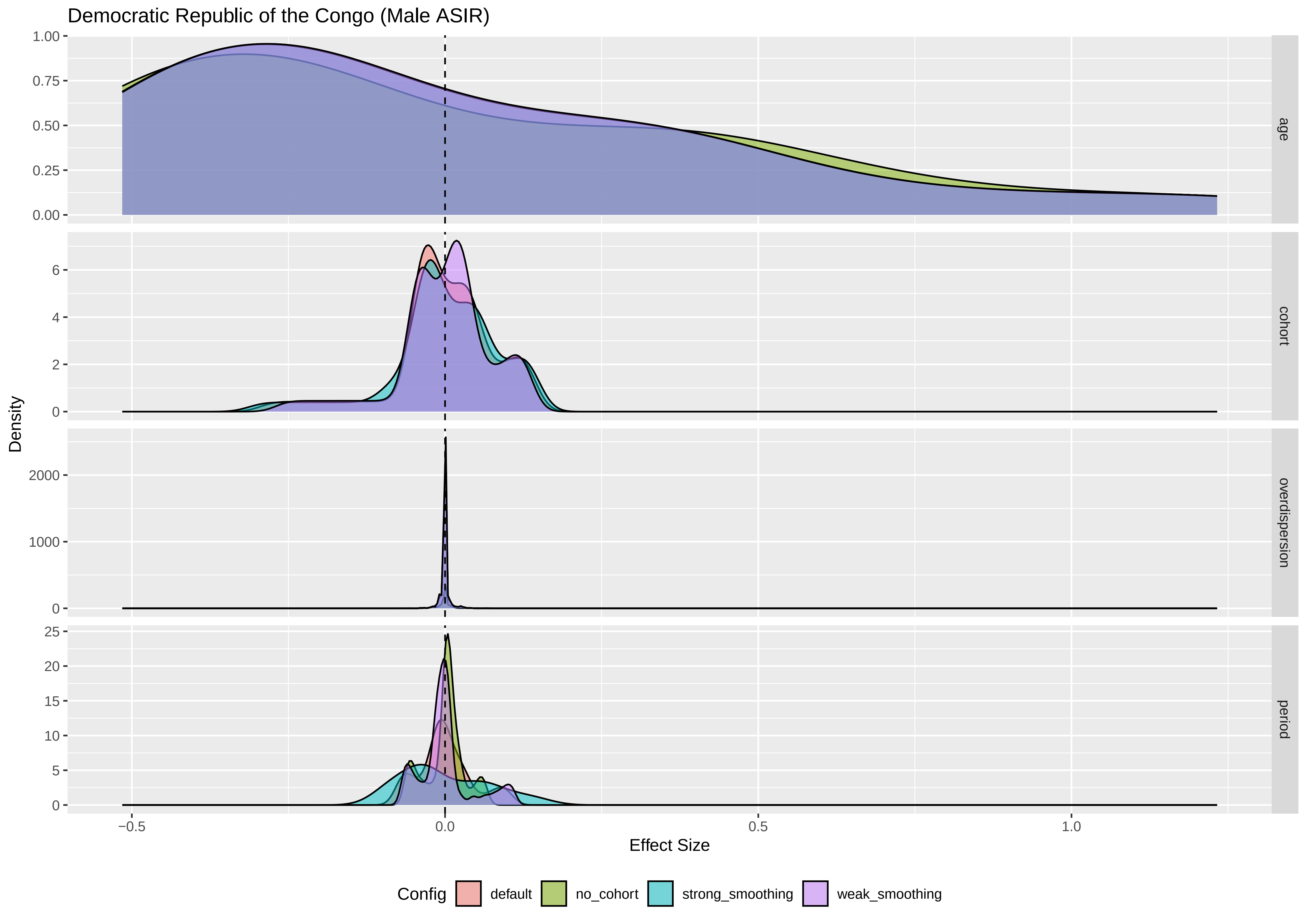

Democratic Republic of the Congo (Male ASIR)
age
cohort
overdispersion
period
Density
Effect Size
1.00
0.75
0.50
0.25
0.00
6
4
2
0
2000
1000
0
25
20
15
10
5
0
-0.5
0.0
0.5
1.0
Config
default
no_cohort
strong_smoothing
weak_smoothing

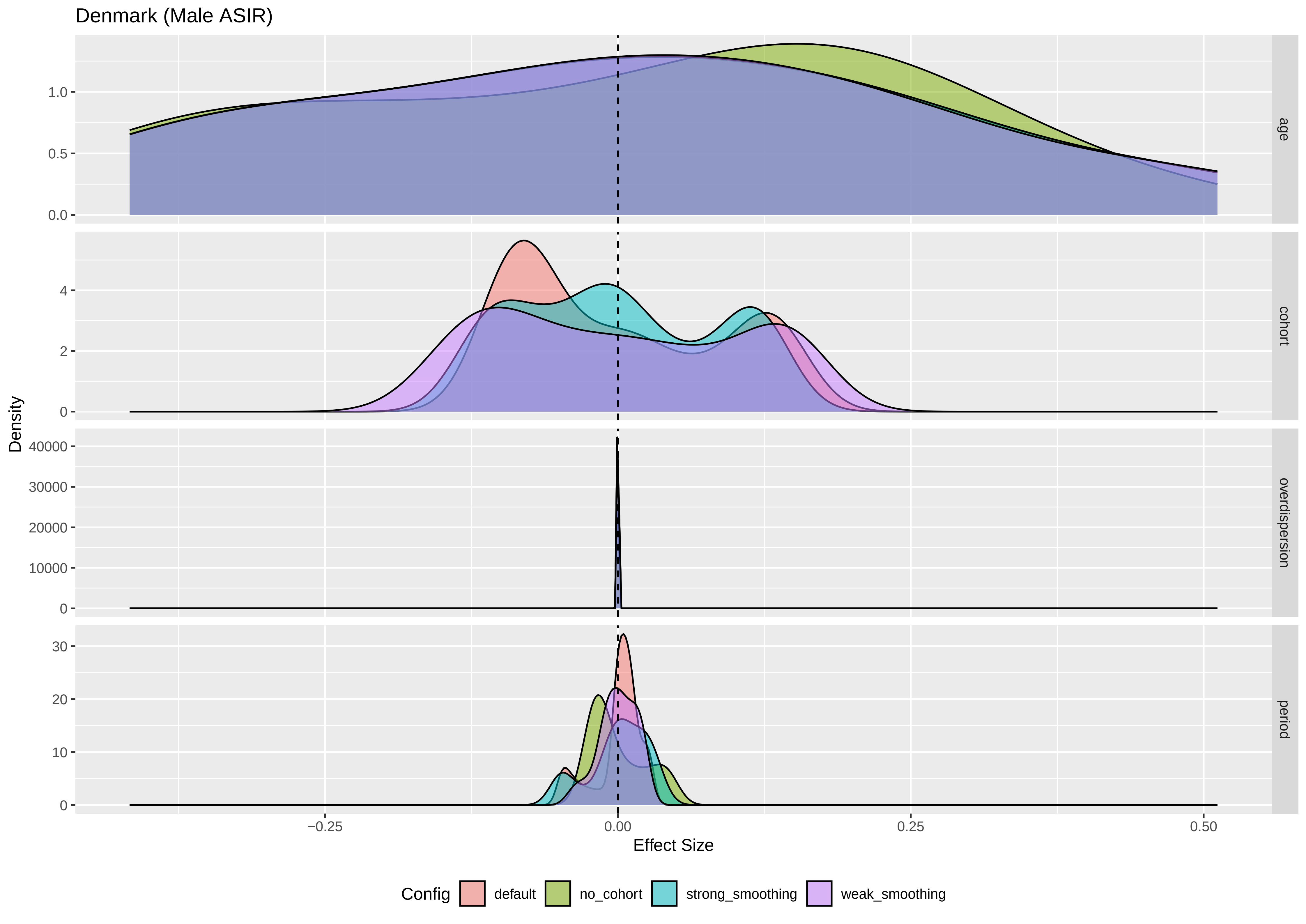

Denmark (Male ASIR)
Density
Effect Size
age
cohort
overdispersion
period
0.0
0.5
1.0
0
2
4
0
10000
20000
30000
40000
0
10
20
30
−0.25
0.00
0.25
0.50
Config
default
no_cohort
strong_smoothing
weak_smoothing

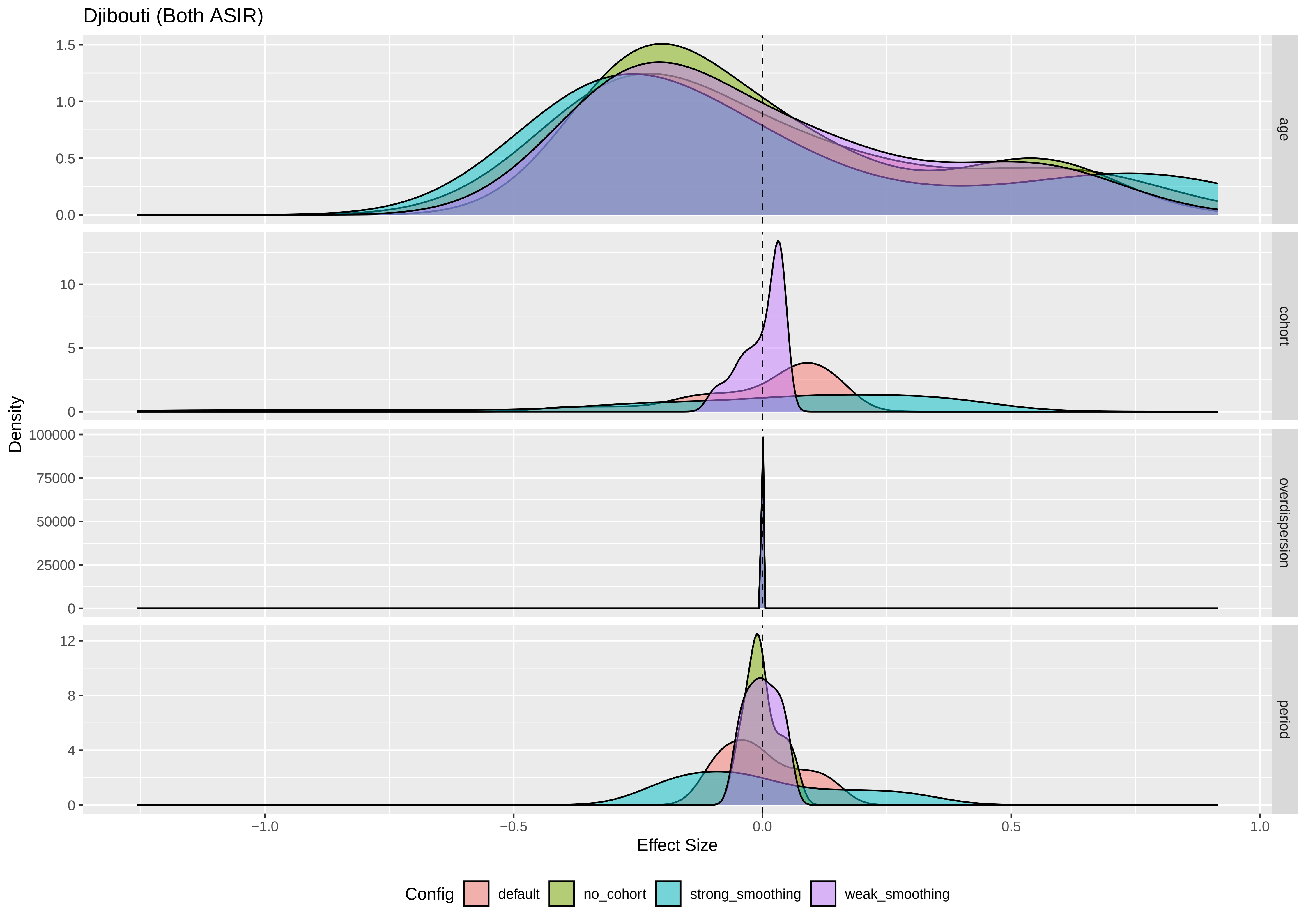
Djibouti (Both ASIR)
Density
Effect Size
age
cohort
overdispersion
period
1.5
1.0
0.5
0.0
10
5
0
100000
75000
50000
25000
0
12
8
4
0
−1.0
−0.5
0.0
0.5
1.0
Config
default
no_cohort
strong_smoothing
weak_smoothing

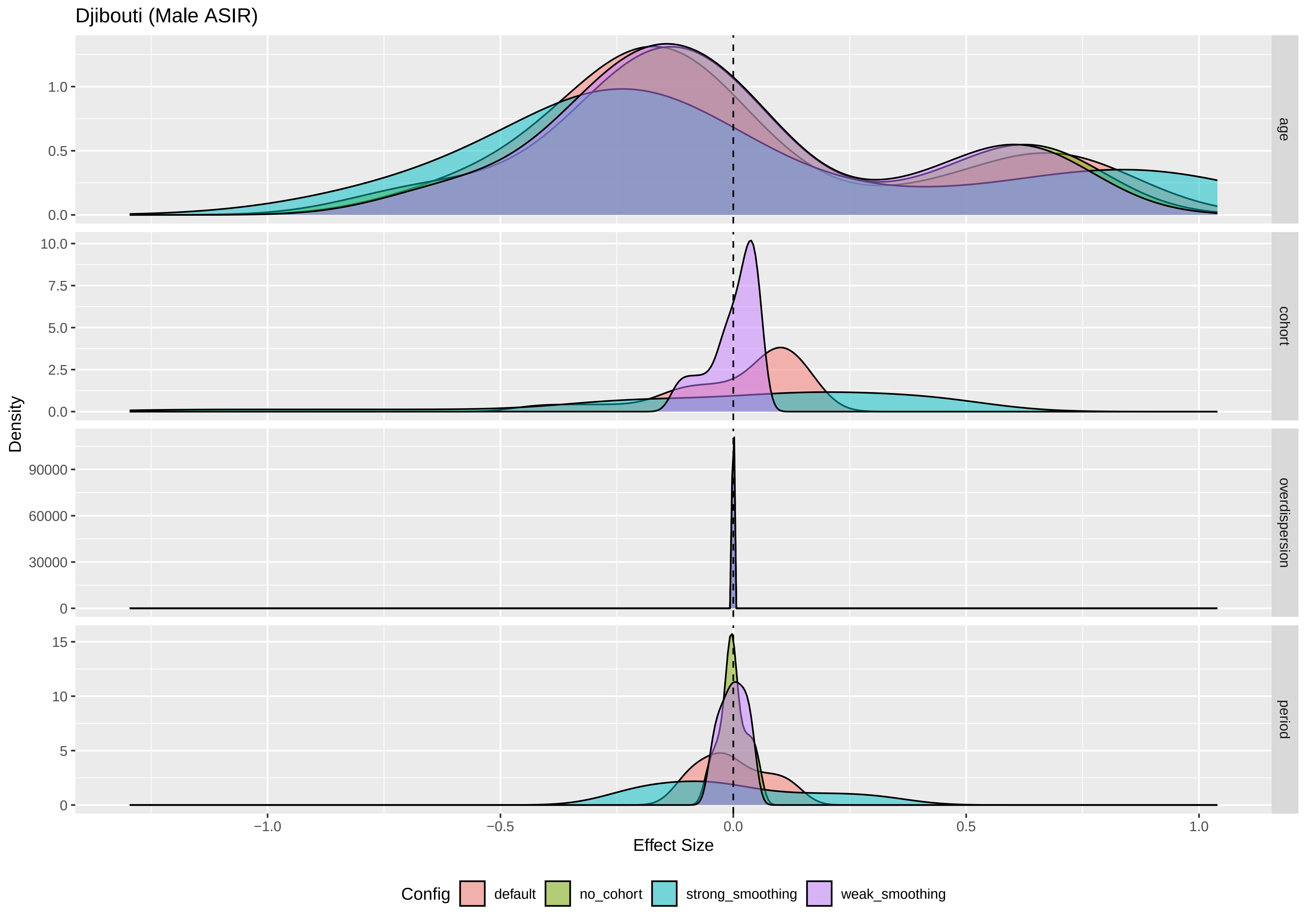

Djibouti (Male ASIR)
Density
Effect Size
age
cohort
overdispersion
period
1.0
0.5
0.0
10.0
7.5
5.0
2.5
0.0
90000
60000
30000
0
15
10
5
0
−1.0
−0.5
0.0
0.5
1.0
Config
default
no_cohort
strong_smoothing
weak_smoothing

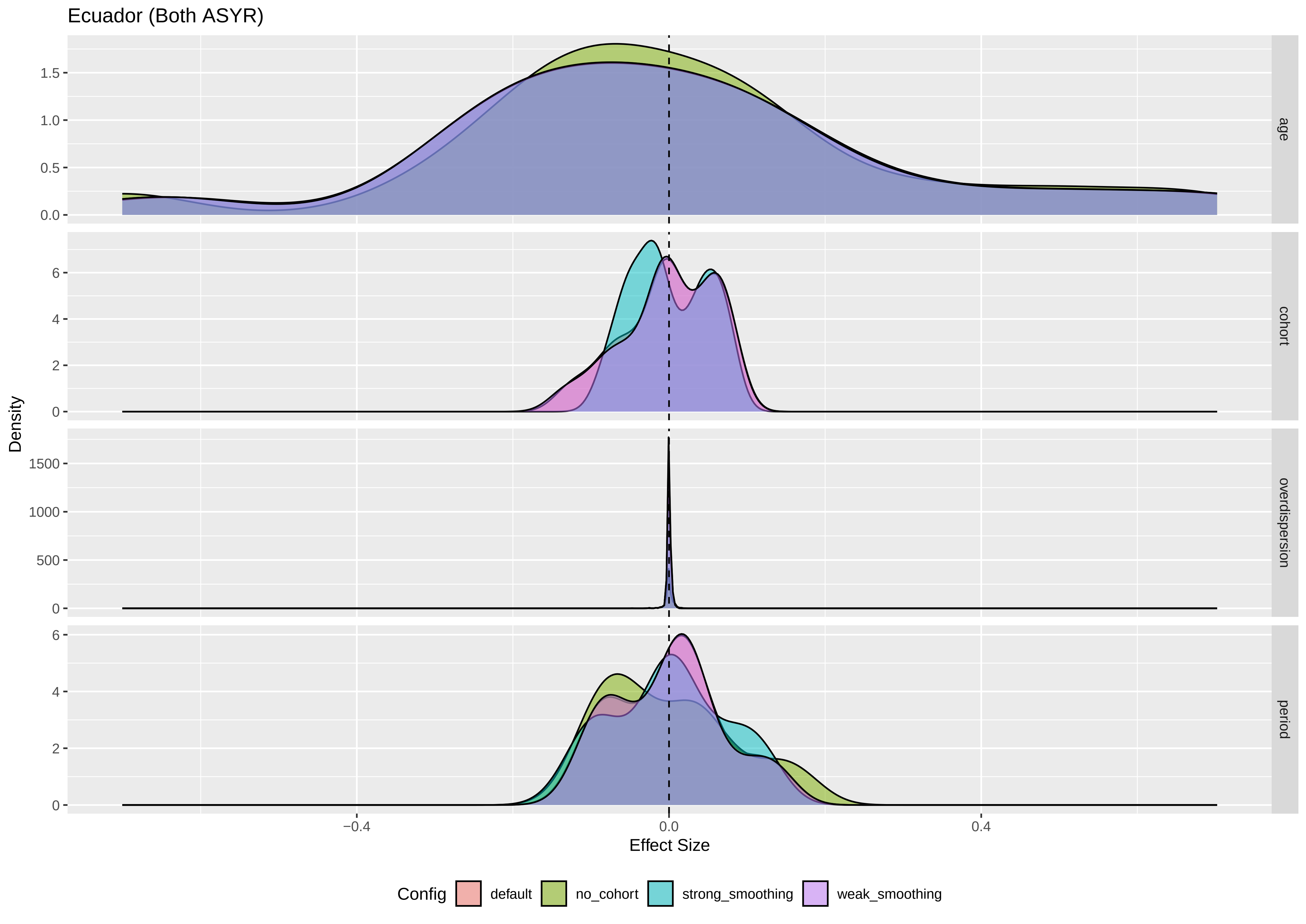
Ecuador (Both ASYR)
Density
Effect Size
age
cohort
overdispersion
period
1.5
1.0
0.5
0.0
6
4
2
0
1500
1000
500
0
−0.4
0.0
0.4
Config
default
no_cohort
strong_smoothing
weak_smoothing

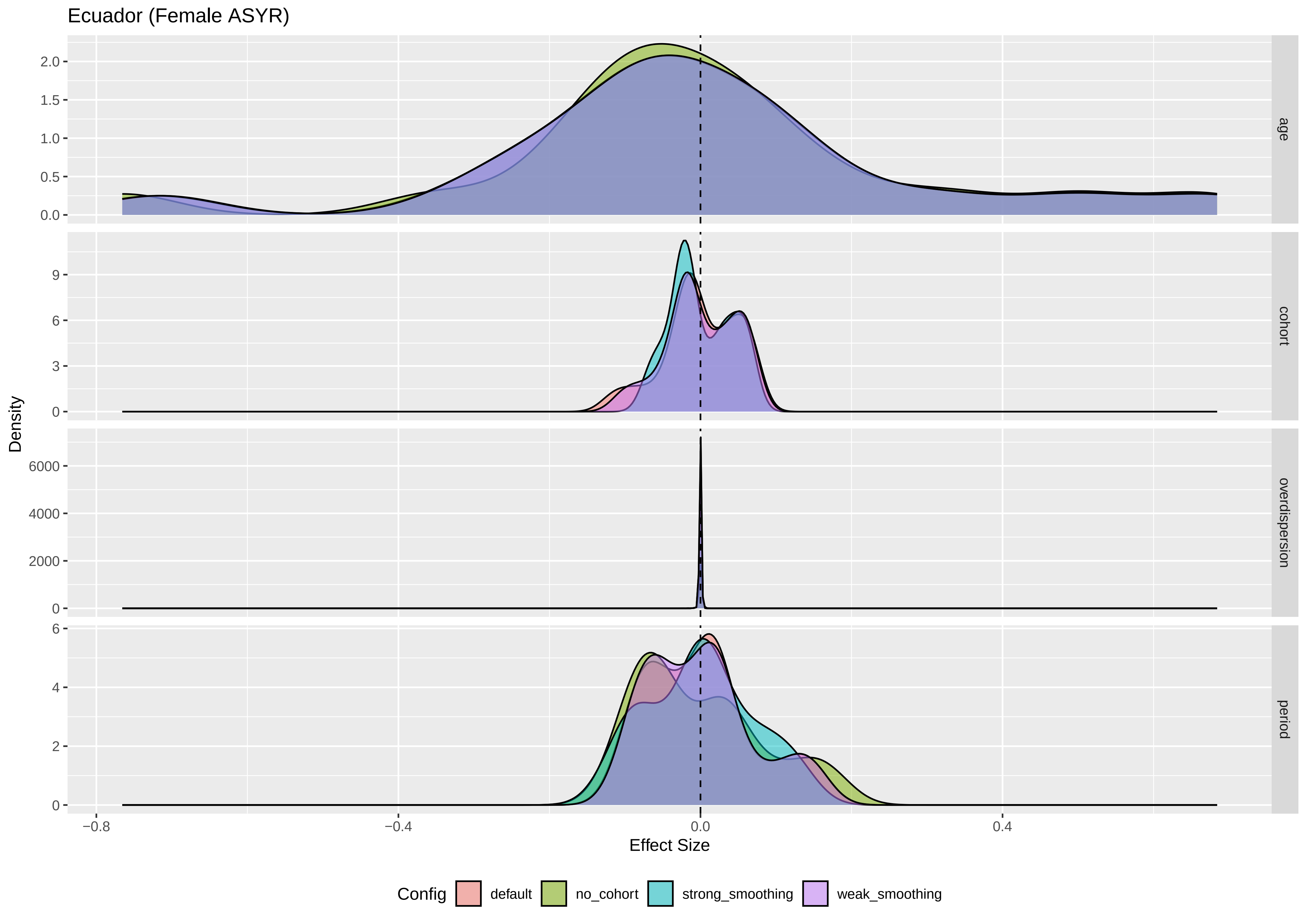

Ecuador (Female ASYR)
Density
Effect Size
age
cohort
overdispersion
period
2.0
1.5
1.0
0.5
0.0
9
6
3
0
6000
4000
2000
0
6
4
2
0
−0.8
−0.4
0.0
0.4
Config
default
no_cohort
strong_smoothing
weak_smoothing

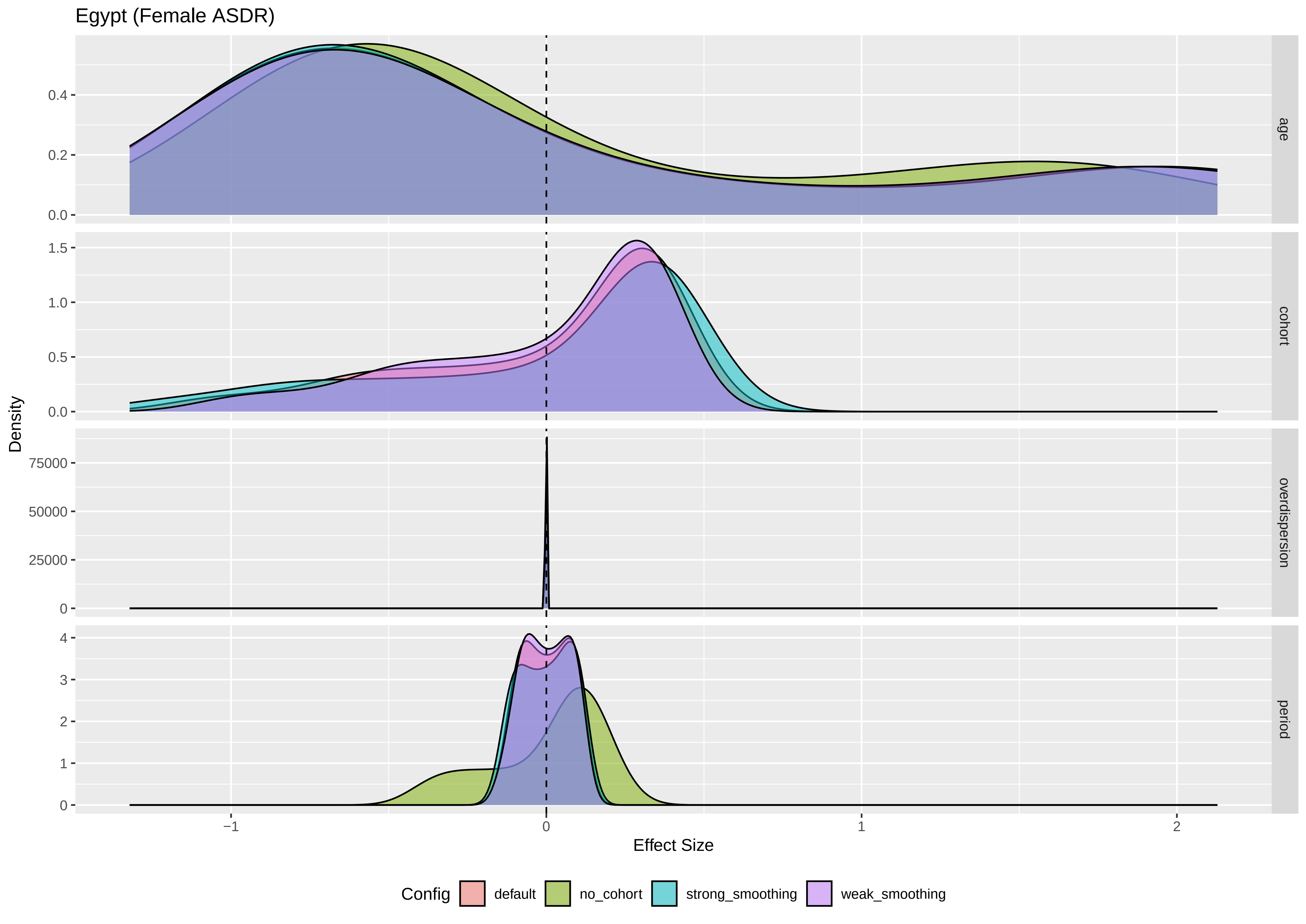

Egypt (Female ASDR)
age
cohort
overdispersion
period
Density
Effect Size
0.0
0.2
0.4
0.5
1.0
1.5
0
25000
50000
75000
1
2
3
4
−1
0
1
2
Config
default
no_cohort
strong_smoothing
weak_smoothing

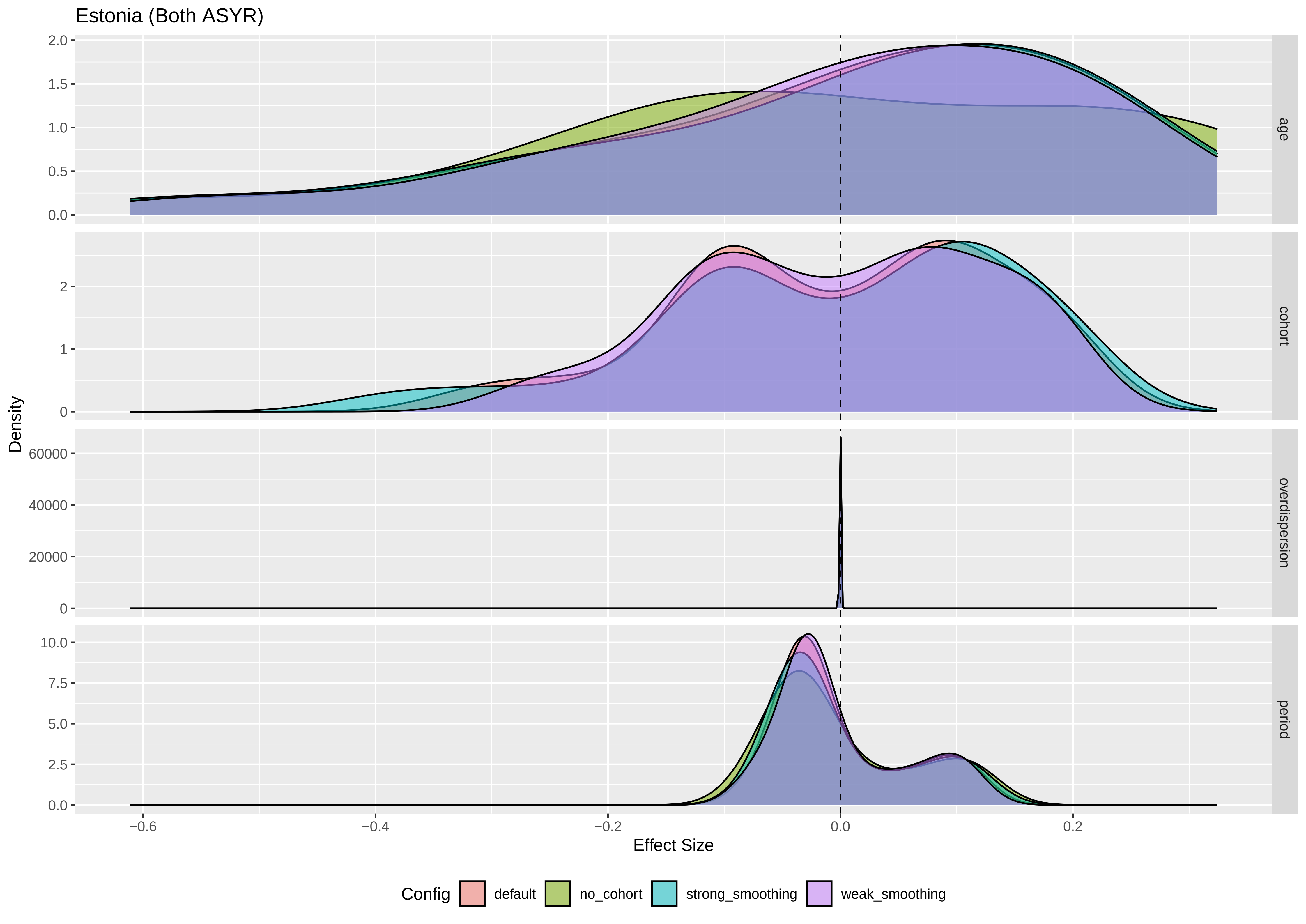

Estonia (Both ASYR)
Density
age
cohort
overdispersion
period
2.0
1.5
1.0
0.5
0.0
2
1
0
60000
40000
20000
0
10.0
7.5
5.0
2.5
0.0
−0.6
−0.4
−0.2
0.0
0.2
Effect Size
Config
default
no_cohort
strong_smoothing
weak_smoothing

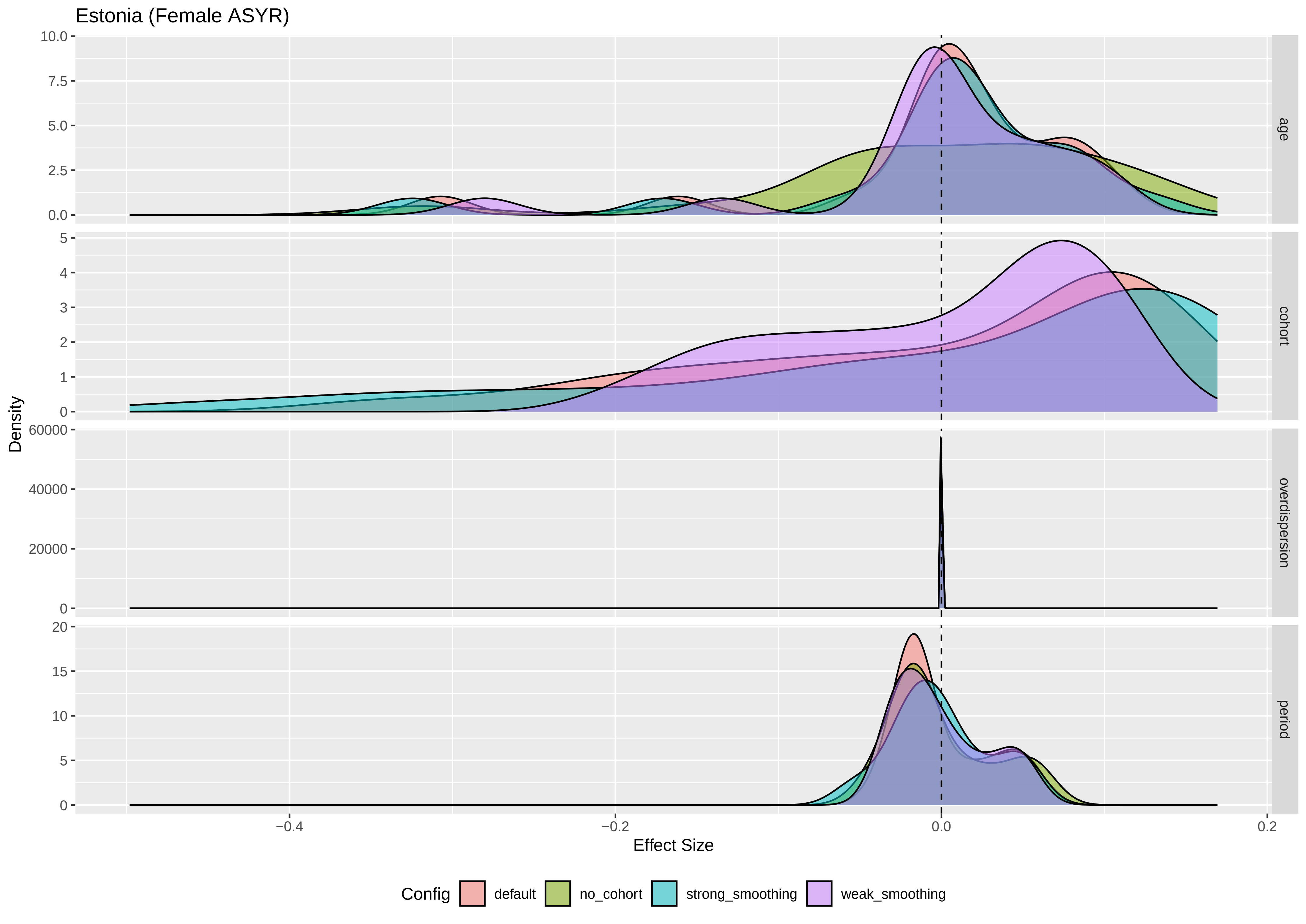

Estonia (Female ASYR)
10.0
7.5
5.0
2.5
0.0
age
5
4
3
2
1
0
cohort
60000
40000
20000
0
overdispersion
20
15
10
5
0
period
Density
−0.4
−0.2
0.0
0.2
Effect Size
Config
default
no_cohort
strong_smoothing
weak_smoothing

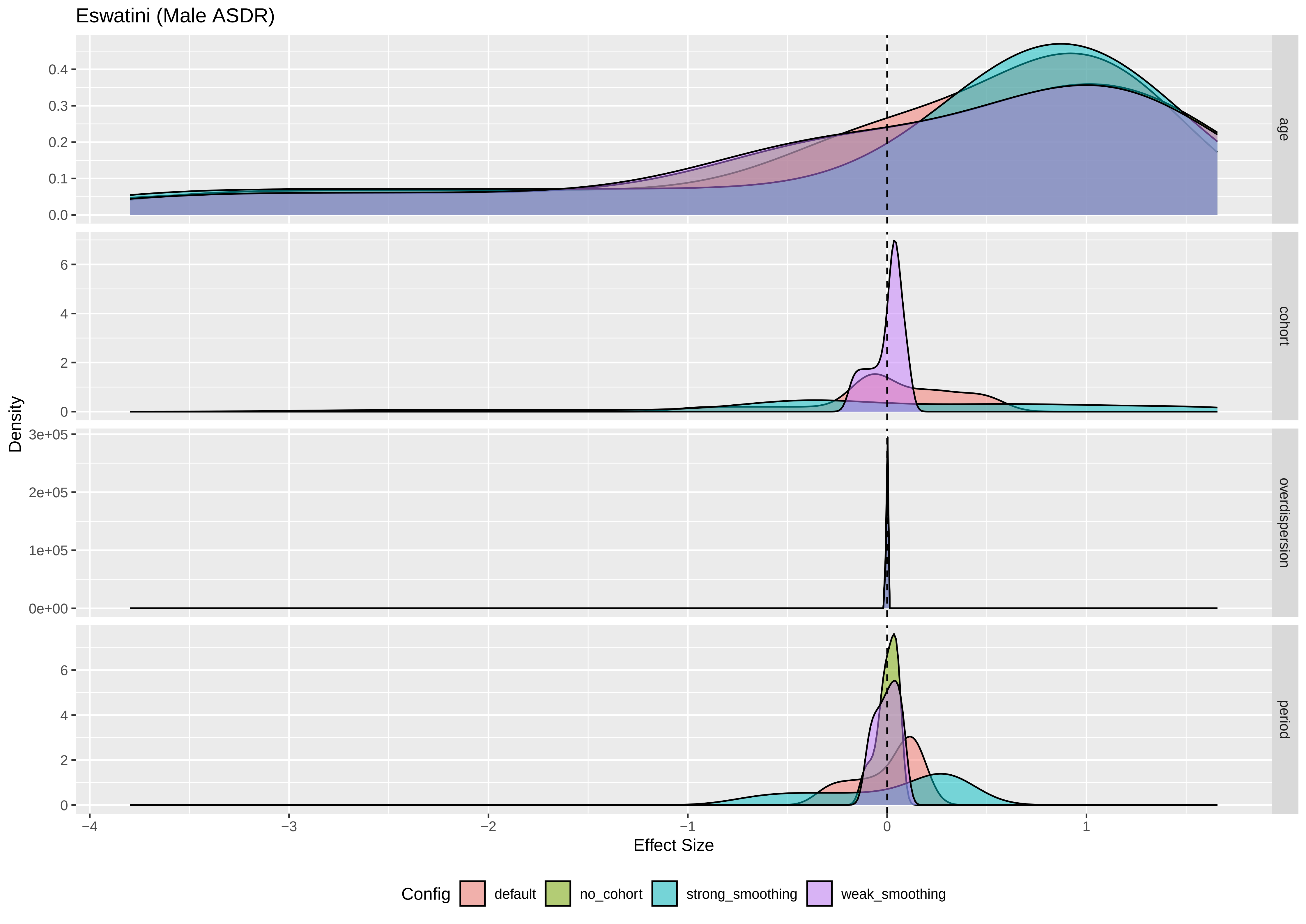

Eswatini (Male ASDR)
Density
Effect Size
age
cohort
overdispersion
period
0.0
0.1
0.2
0.3
0.4
0
2
4
6
0e+00
1e+05
2e+05
3e+05
−4
−3
−2
−1
0
1
Config
default
no_cohort
strong_smoothing
weak_smoothing

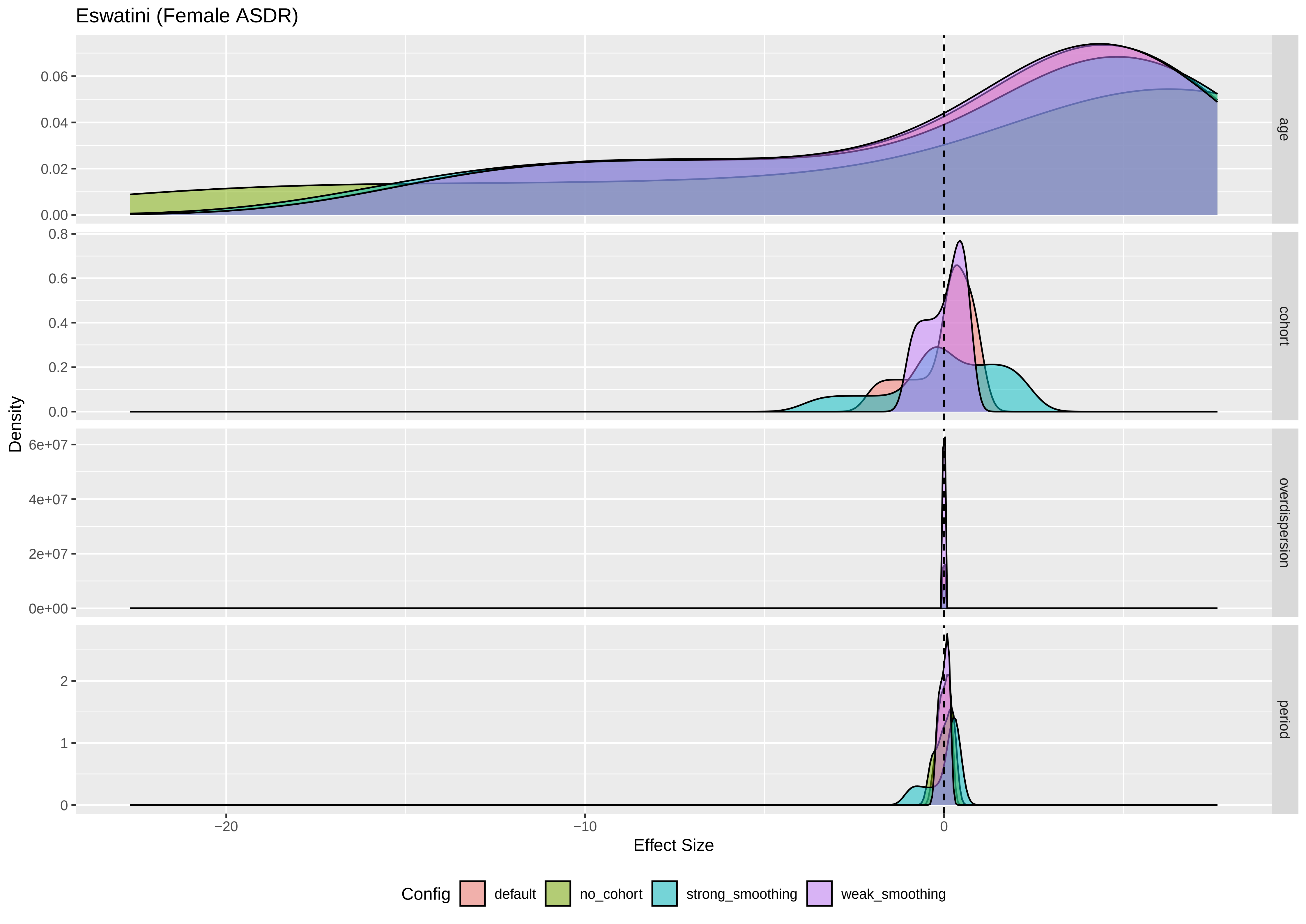
Eswatini (Female ASDR)
0.06
0.04
0.02
0.00
age
0.8
0.6
0.4
0.2
0.0
cohort
6e+07
4e+07
2e+07
0e+00
overdispersion
2
1
0
period
Density
−20
−10
0
Effect Size
Config
default
no_cohort
strong_smoothing
weak_smoothing

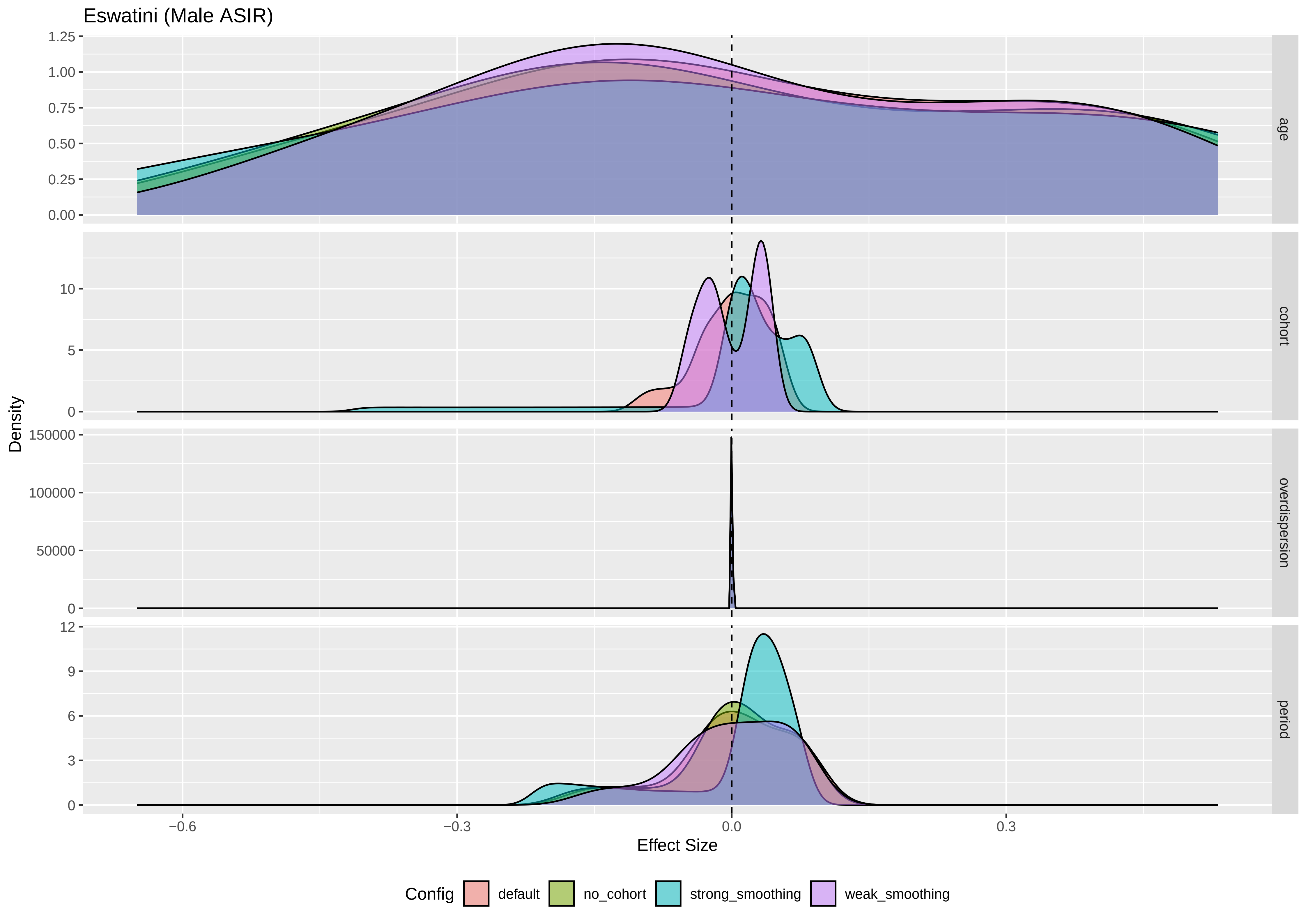

Eswatini (Male ASIR)
Density
Effect Size
age
cohort
overdispersion
period
1.25
1.00
0.75
0.50
0.25
0.00
10
5
0
150000
100000
50000
0
12
9
6
3
0
−0.6
−0.3
0.0
0.3
Config
default
no_cohort
strong_smoothing
weak_smoothing

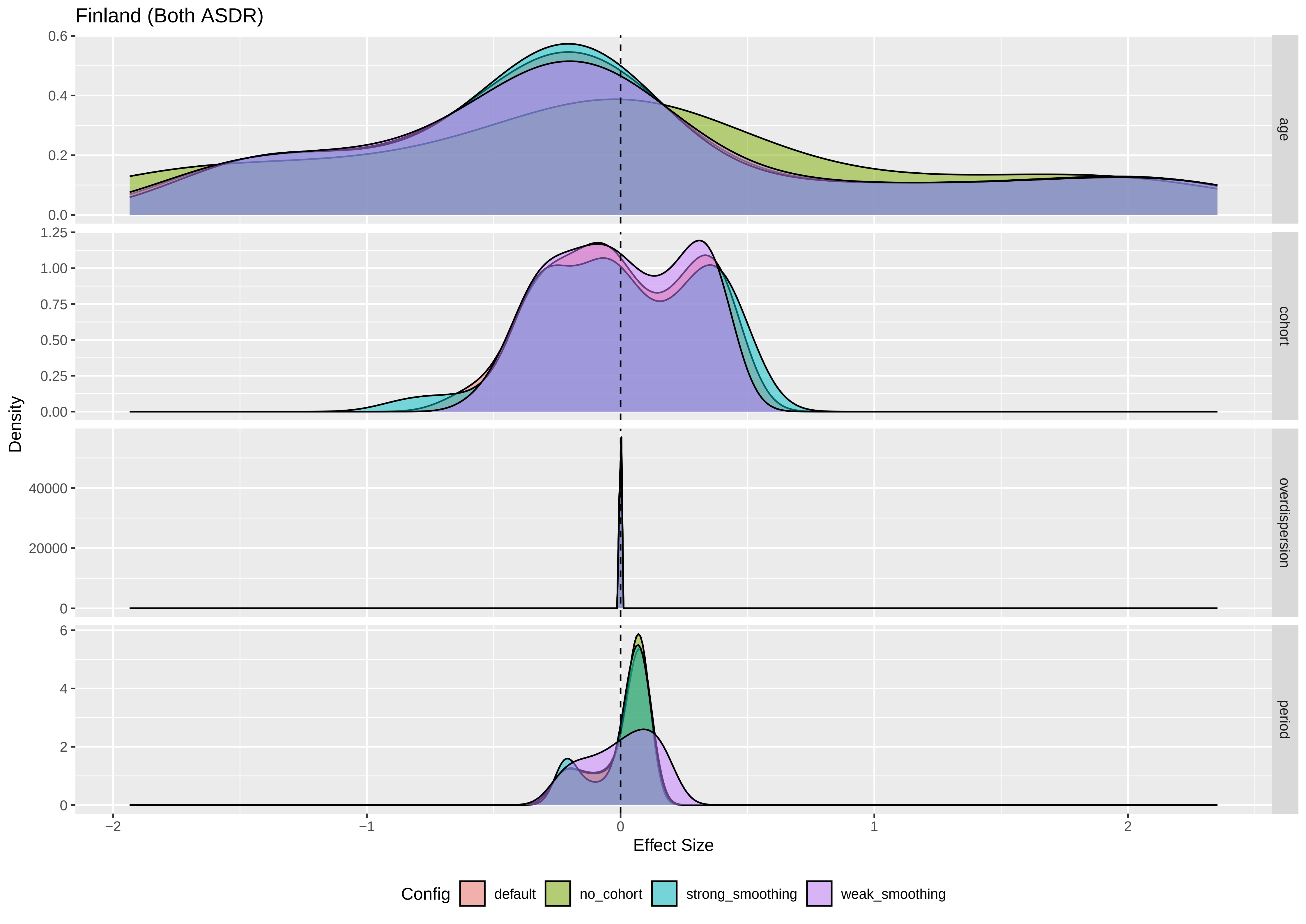

Finland (Both ASDR)
Density
Effect Size
age
cohort
overdispersion
period
0.0
0.2
0.4
0.6
0.00
0.25
0.50
0.75
1.00
1.25
0
20000
40000
0
2
4
6
−2
−1
0
1
2
Config
default
no_cohort
strong_smoothing
weak_smoothing

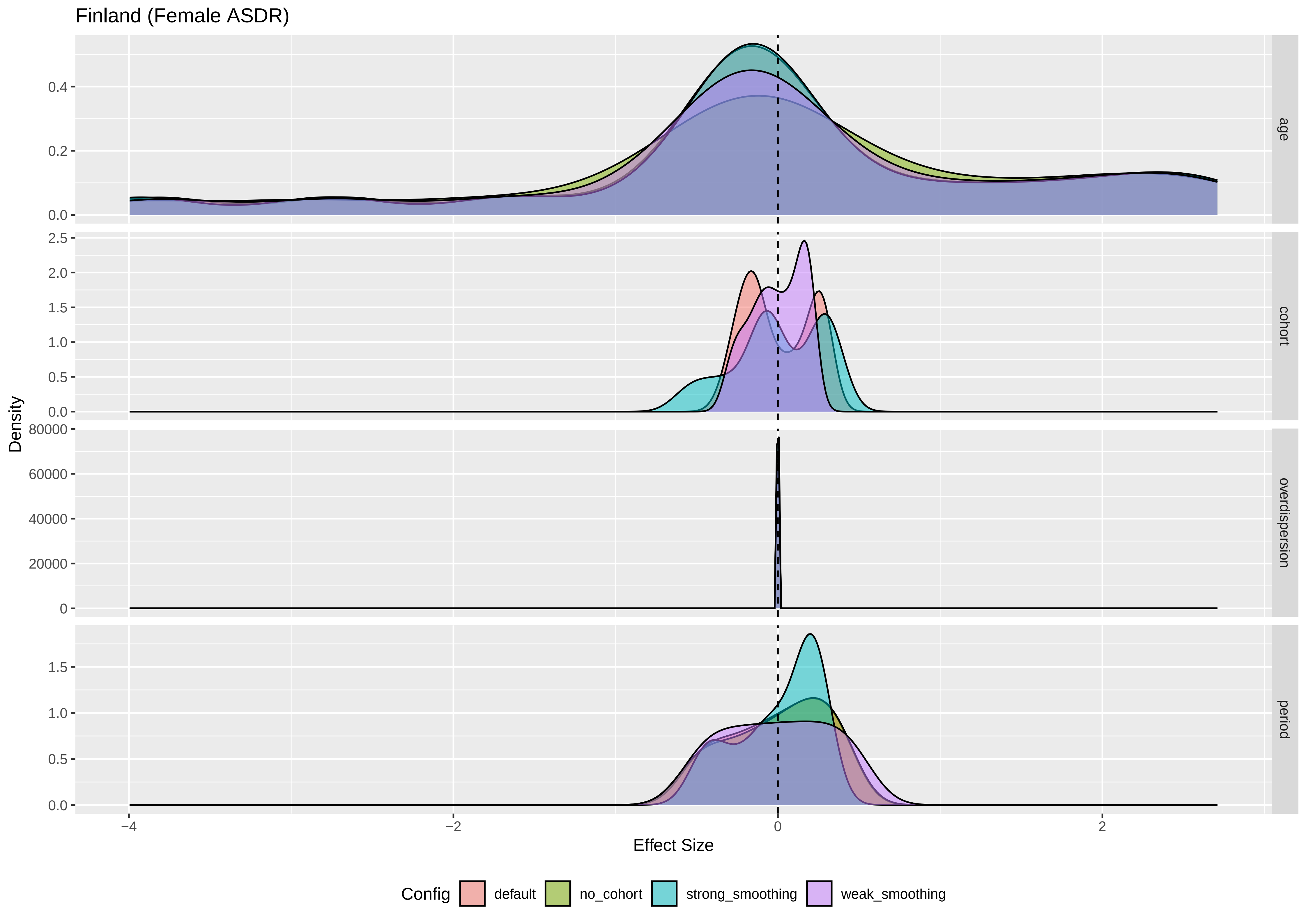

Finland (Female ASDR)
Density
Effect Size
age
cohort
overdispersion
period
0.0
0.2
0.4
0.5
1.0
1.5
2.0
2.5
0
20000
40000
60000
80000
−4
−2
0
2
Config
default
no_cohort
strong_smoothing
weak_smoothing

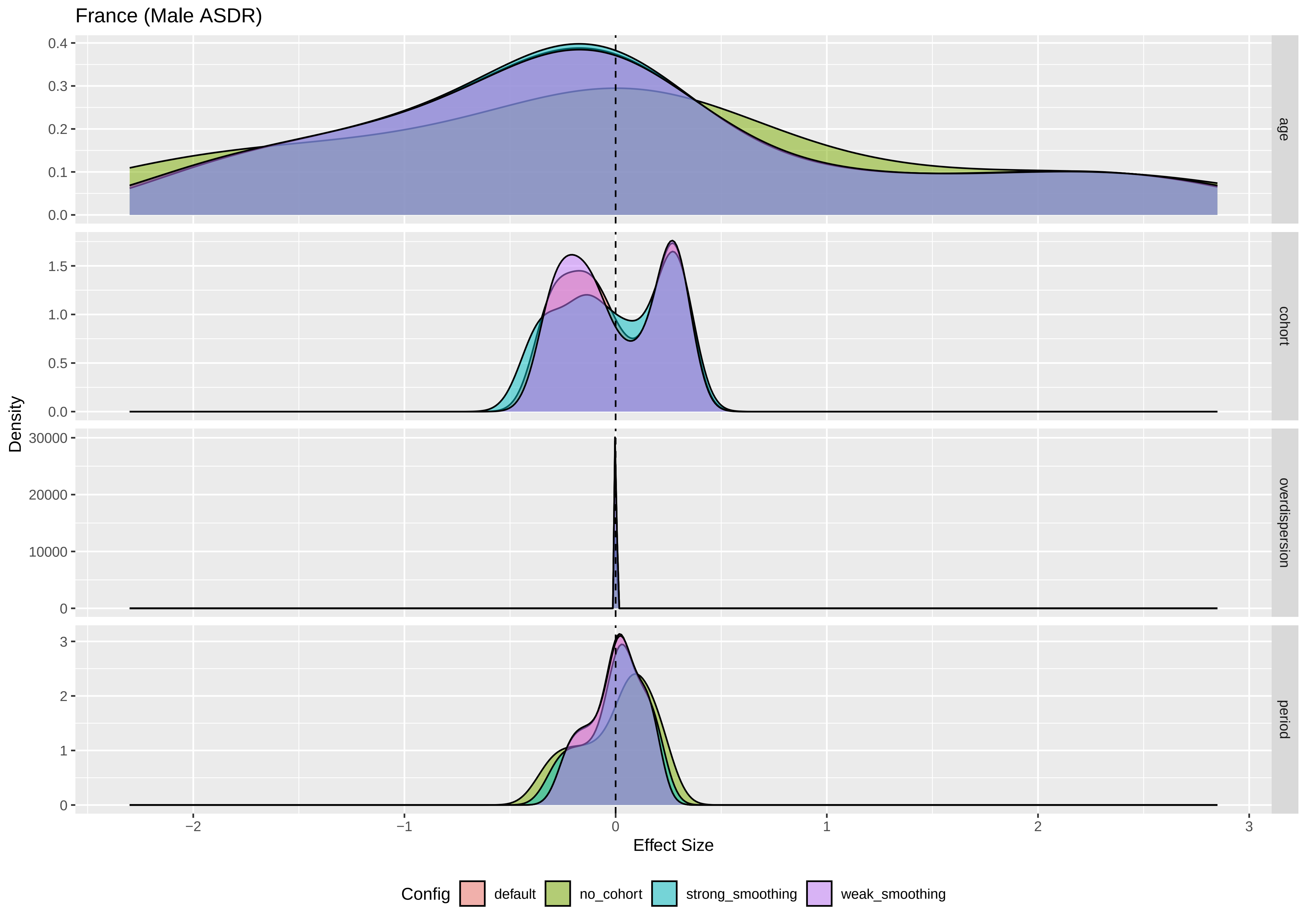

France (Male ASDR)
Density
Effect Size
age
cohort
overdispersion
period
0.0
0.1
0.2
0.3
0.4
0.5
1.0
1.5
0
10000
20000
30000
1
2
3
−2
−1
Config
default
no_cohort
strong_smoothing
weak_smoothing

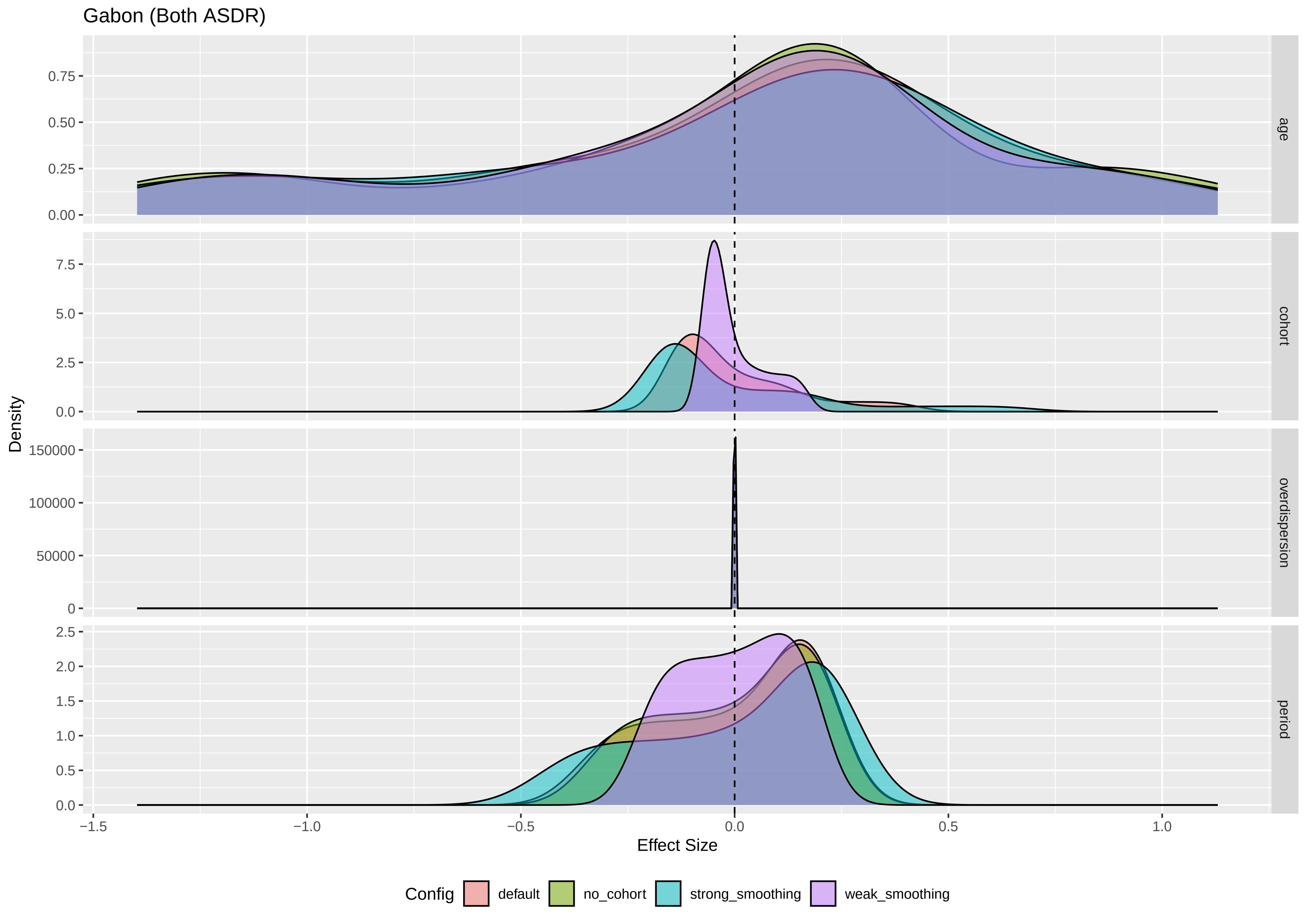

Gabon (Both ASDR)
Density
0.00
0.25
0.50
0.75
age
0.0
2.5
5.0
7.5
cohort
0
50000
100000
150000
overdispersion
0.0
0.5
1.0
1.5
2.0
2.5
period
−1.5
−1.0
−0.5
0.0
0.5
1.0
Effect Size
Config
default
no_cohort
strong_smoothing
weak_smoothing

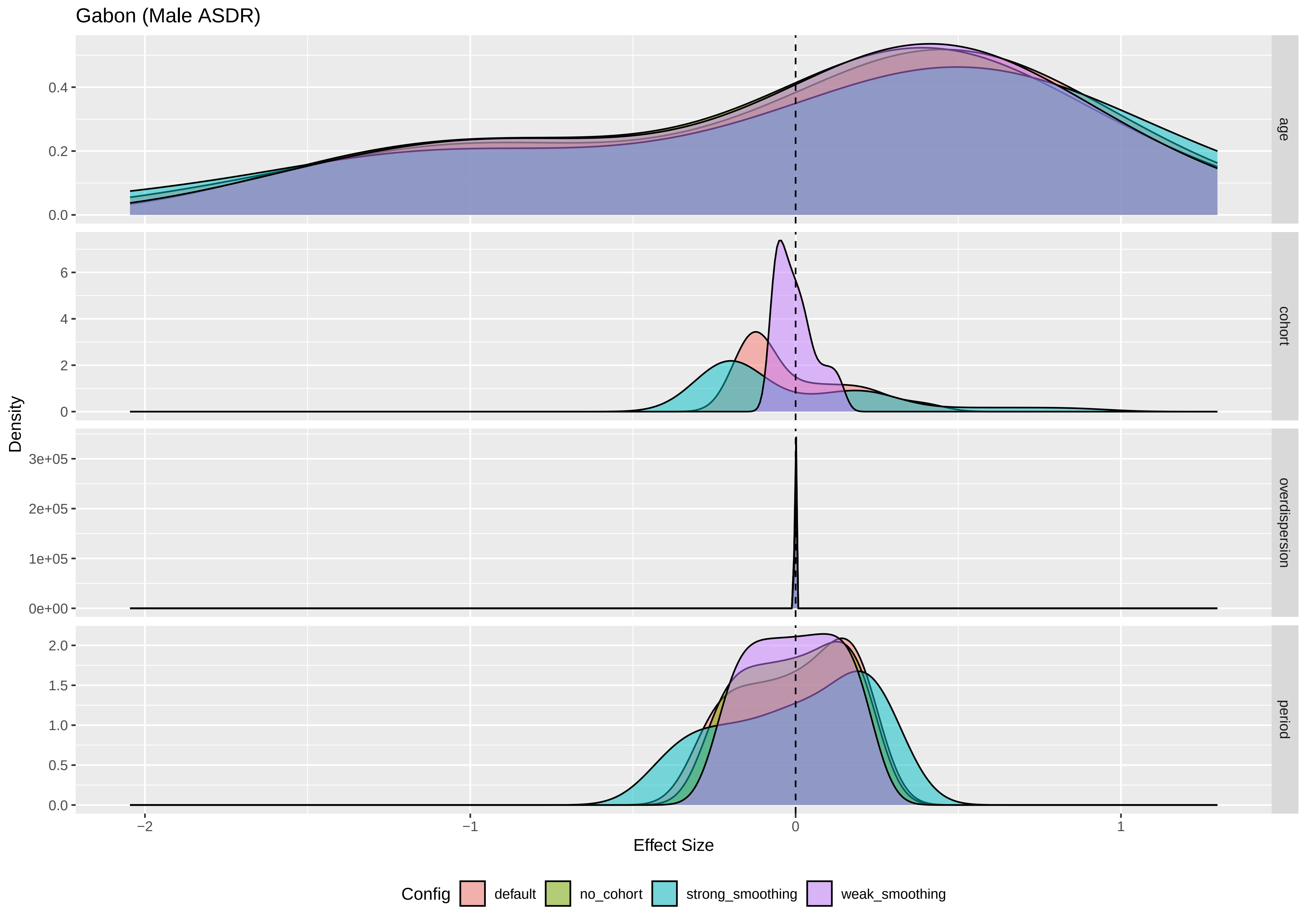

Gabon (Male ASDR)
age
cohort
overdispersion
period
Density
Effect Size
0.0
0.2
0.4
0
2
4
6
0e+00
1e+05
2e+05
3e+05
0.0
0.5
1.0
1.5
2.0
−2
−1
0
1
Config
default
no_cohort
strong_smoothing
weak_smoothing

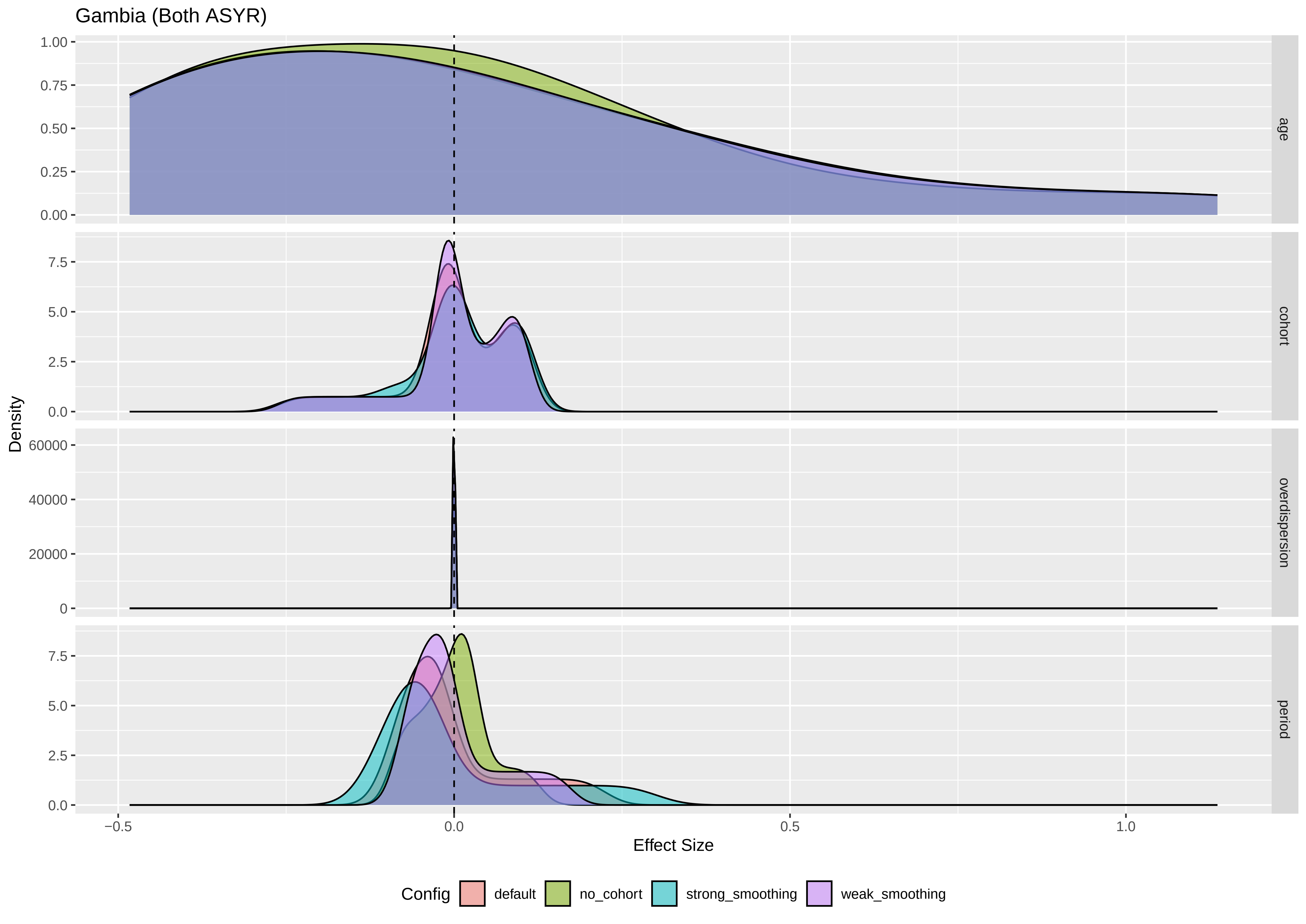

Gambia (Both ASYR)
age
cohort
overdispersion
period
Density
Effect Size
−0.5
0.0
0.5
1.0
Config
default
no_cohort
strong_smoothing
weak_smoothing

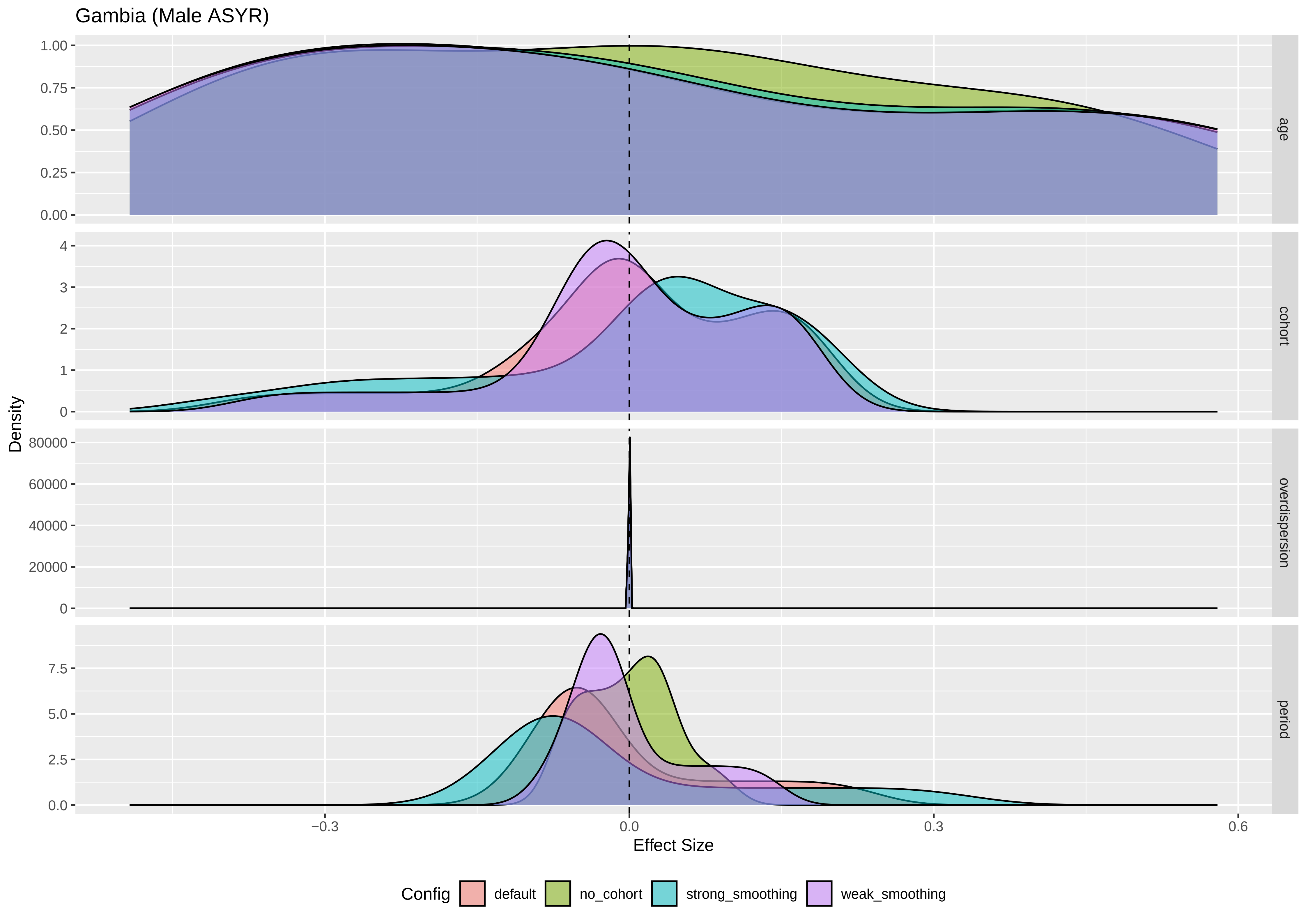

Gambia (Male ASYR)
Density
Effect Size
age
cohort
overdispersion
period
Config
default
no_cohort
strong_smoothing
weak_smoothing

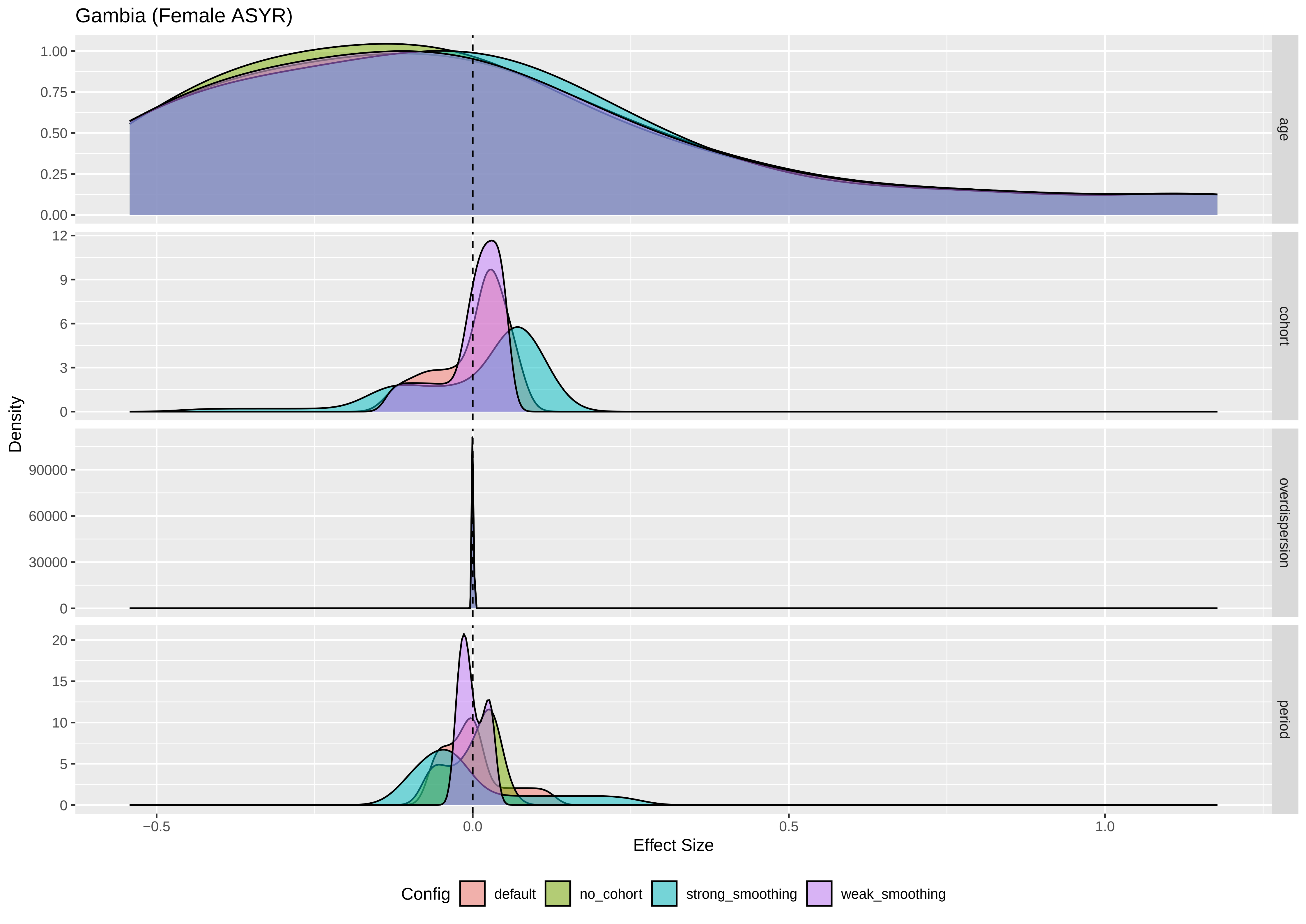
Gambia (Female ASYR)
Density
Effect Size
age
cohort
overdispersion
period
1.00
0.75
0.50
0.25
0.00
12
9
6
3
0
90000
60000
30000
0
20
15
10
5
0
−0.5
0.0
0.5
1.0
Config
default
no_cohort
strong_smoothing
weak_smoothing

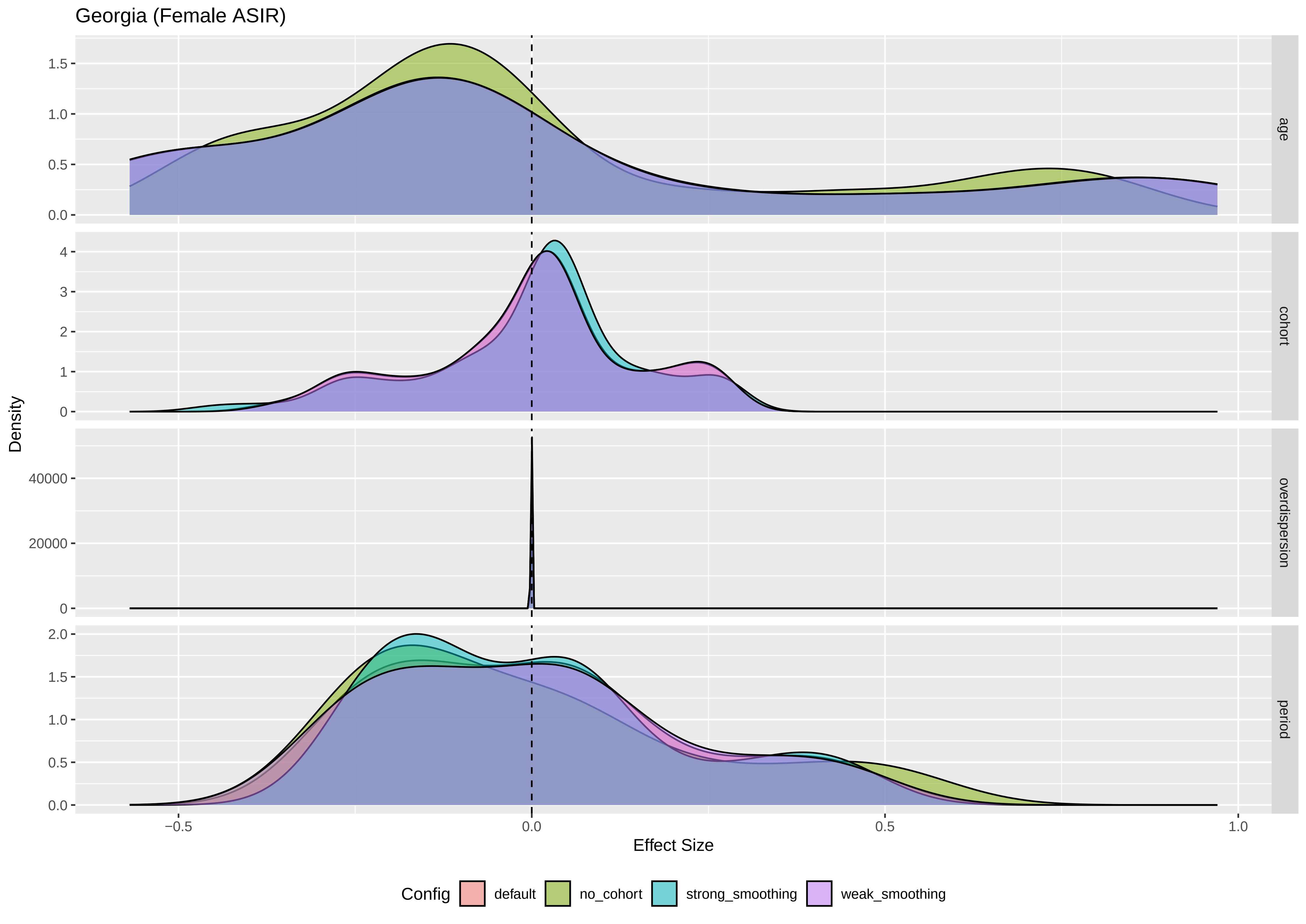

Georgia (Female ASIR)
age
cohort
overdispersion
period
Density
Effect Size
0.0
0.5
1.0
1.5
0
1
2
3
4
0
20000
40000
2.0
−0.5
1.0
Config
default
no_cohort
strong_smoothing
weak_smoothing

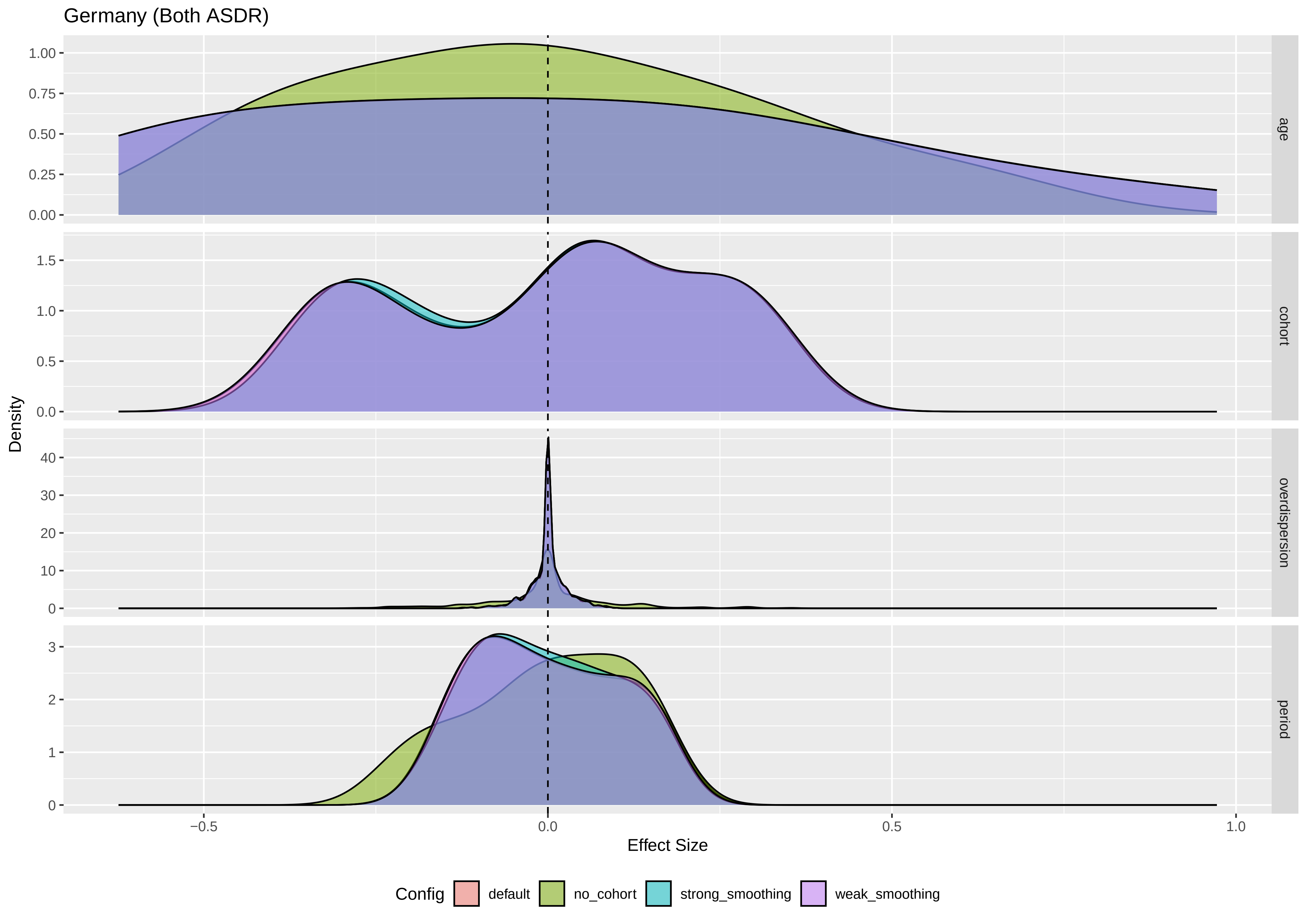

Germany (Both ASDR)
Density
age
cohort
overdispersion
period
1.00
0.75
0.50
0.25
0.00
1.5
1.0
0.5
0.0
40
30
20
10
0
3
2
1
0
−0.5
0.0
0.5
1.0
Effect Size
Config
default
no_cohort
strong_smoothing
weak_smoothing

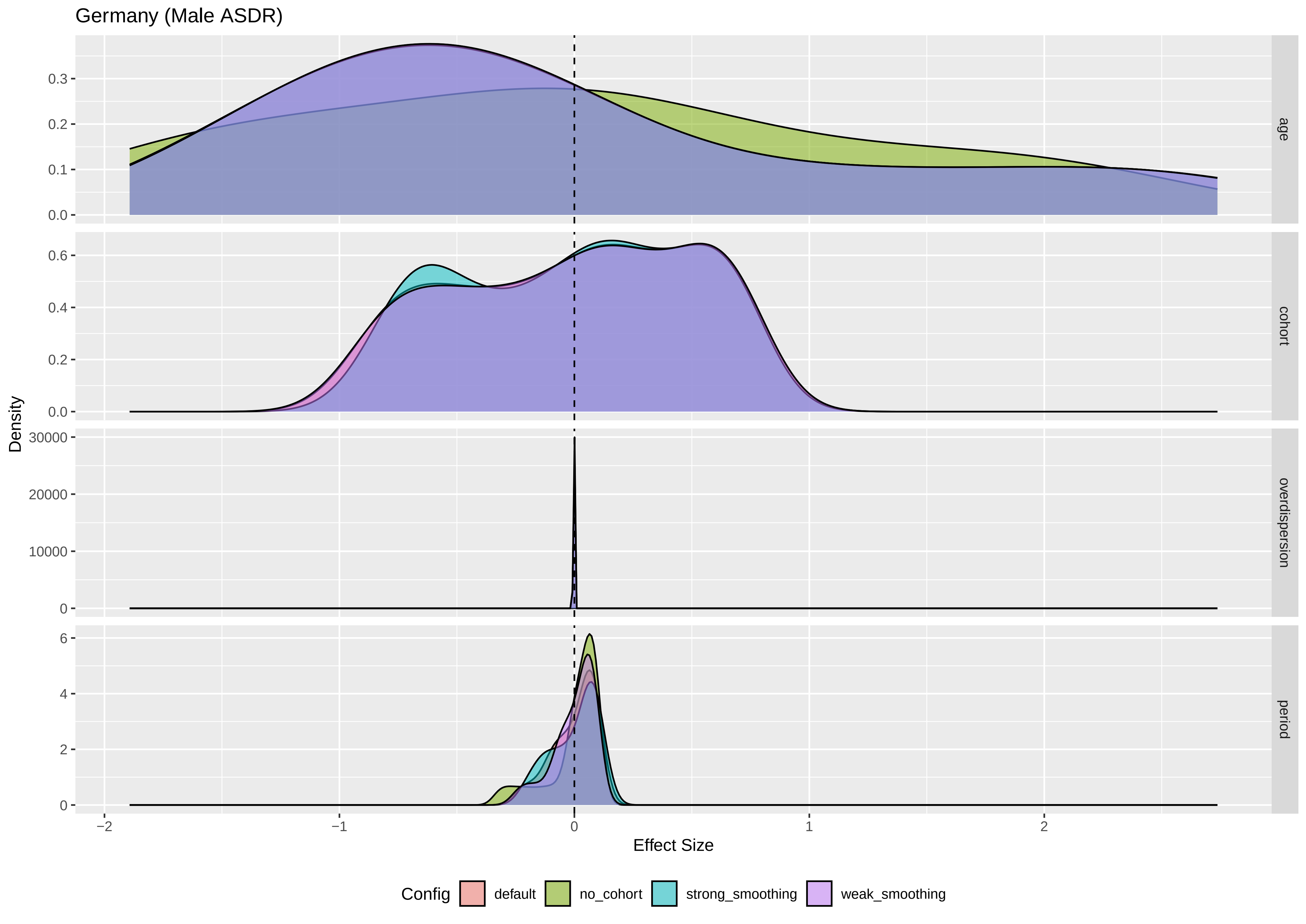

Germany (Male ASDR)
age
cohort
overdispersion
period
Density
Effect Size
0.0
0.1
0.2
0.3
0.0
0.2
0.4
0.6
0
10000
20000
30000
0
2
4
6
−2
−1
0
1
2
Config
default
no_cohort
strong_smoothing
weak_smoothing

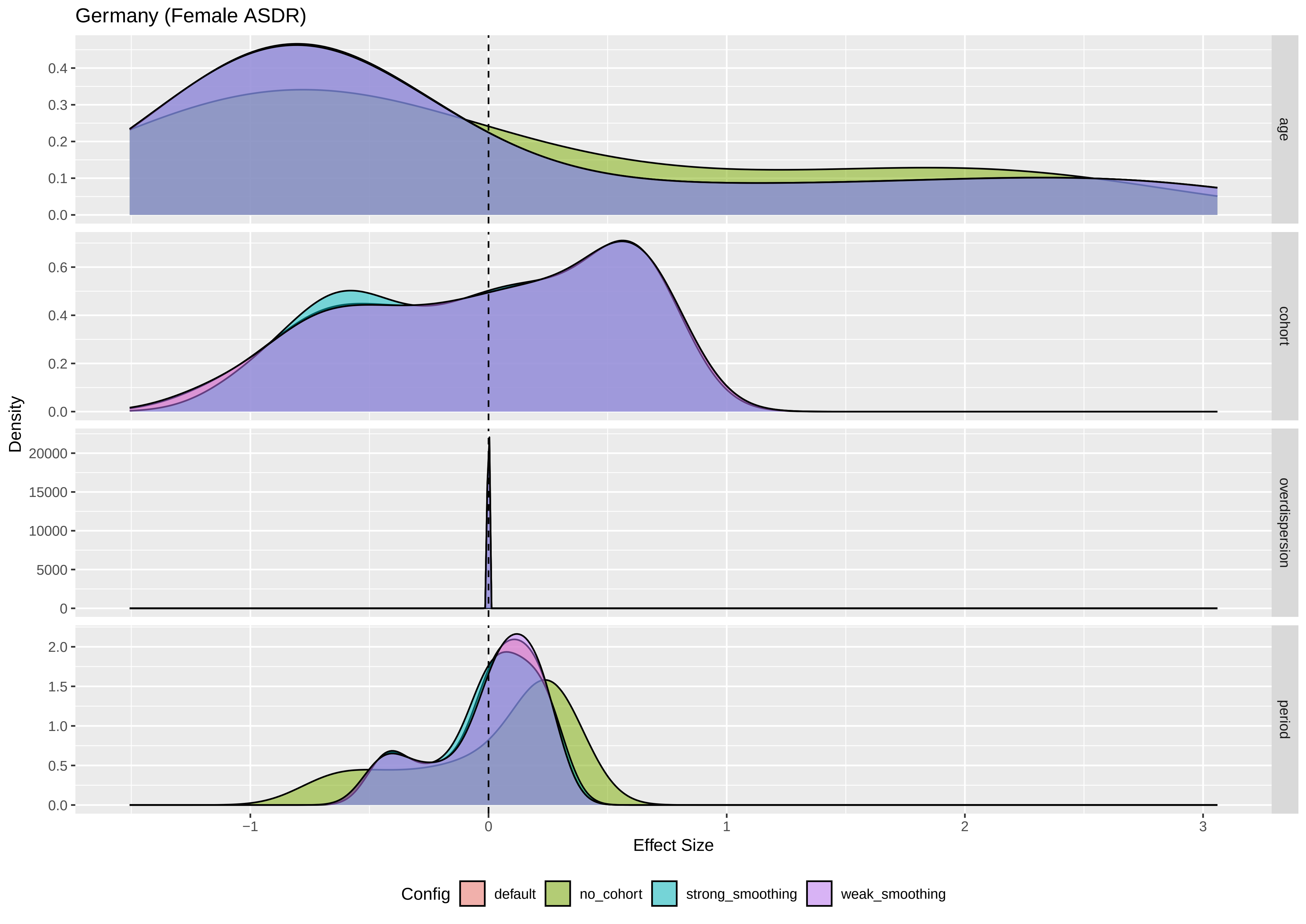

Germany (Female ASDR)
Density
Effect Size
age
cohort
overdispersion
period
0.0
0.1
0.2
0.3
0.4
0.6
0
5000
10000
15000
20000
0.5
1.0
1.5
2.0
−1
0
1
2
3
Config
default
no_cohort
strong_smoothing
weak_smoothing

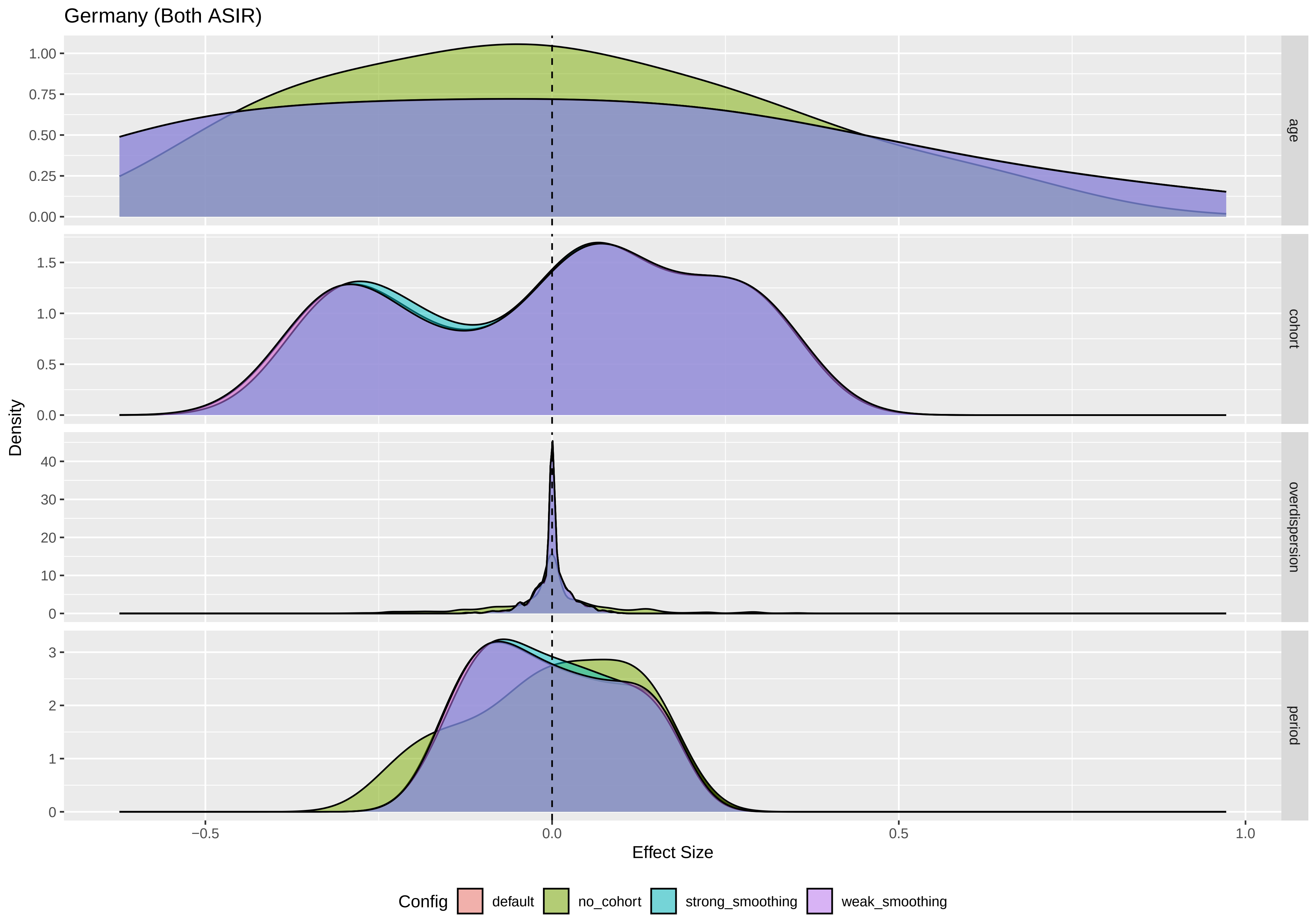

Germany (Both ASIR)
Density
Effect Size
age
cohort
overdispersion
period
1.00
0.75
0.50
0.25
0.00
1.5
1.0
0.5
0.0
40
30
20
10
0
3
2
1
0
−0.5
0.0
0.5
1.0
Config
default
no_cohort
strong_smoothing
weak_smoothing

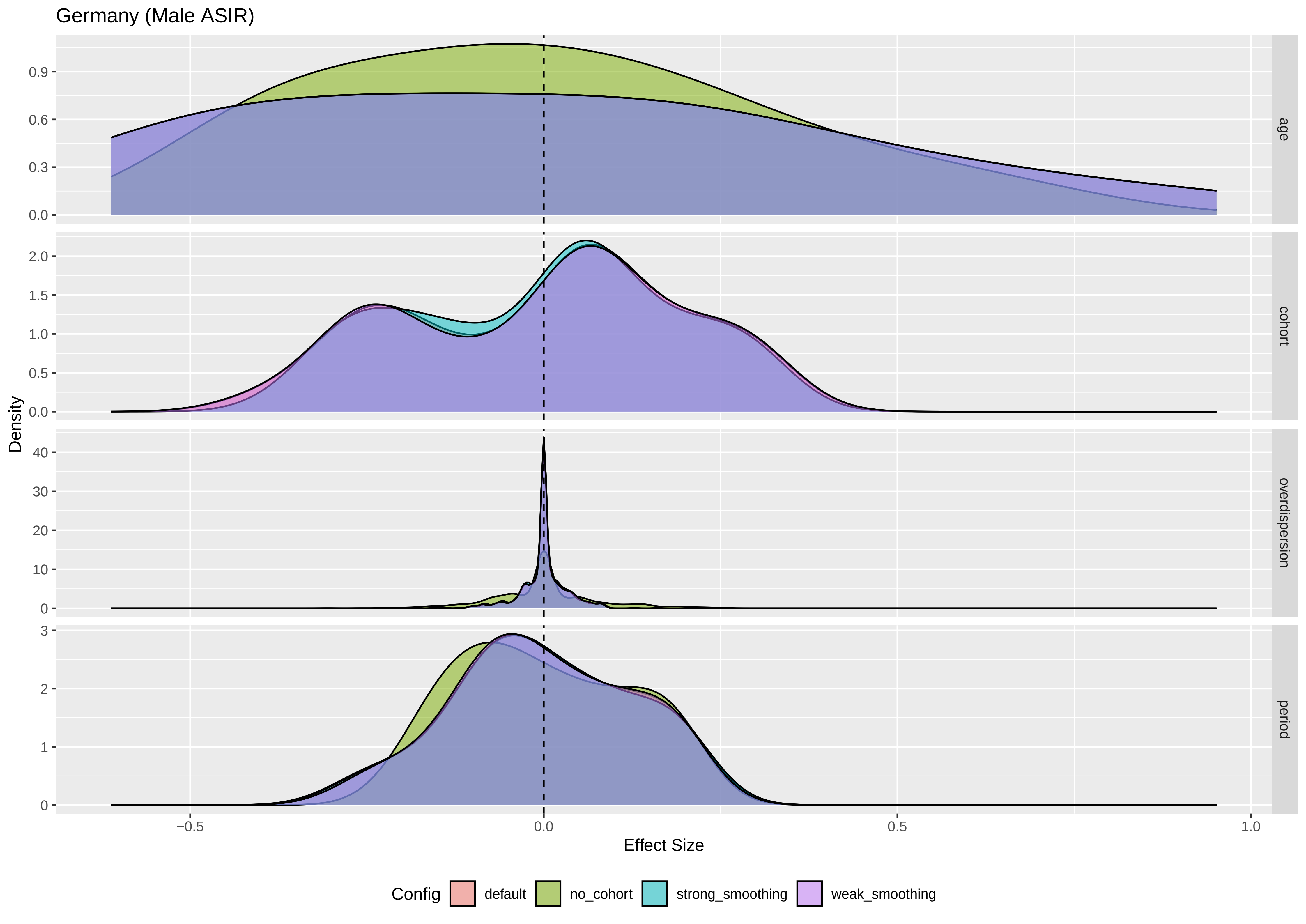

Germany (Male ASIR)
age
cohort
overdispersion
period
Density
Effect Size
−0.5
0.0
0.5
1.0
0.0
0.3
0.6
0.9
0.5
1.0
1.5
2.0
0
10
20
30
40
1
2
3
Config
default
no_cohort
strong_smoothing
weak_smoothing

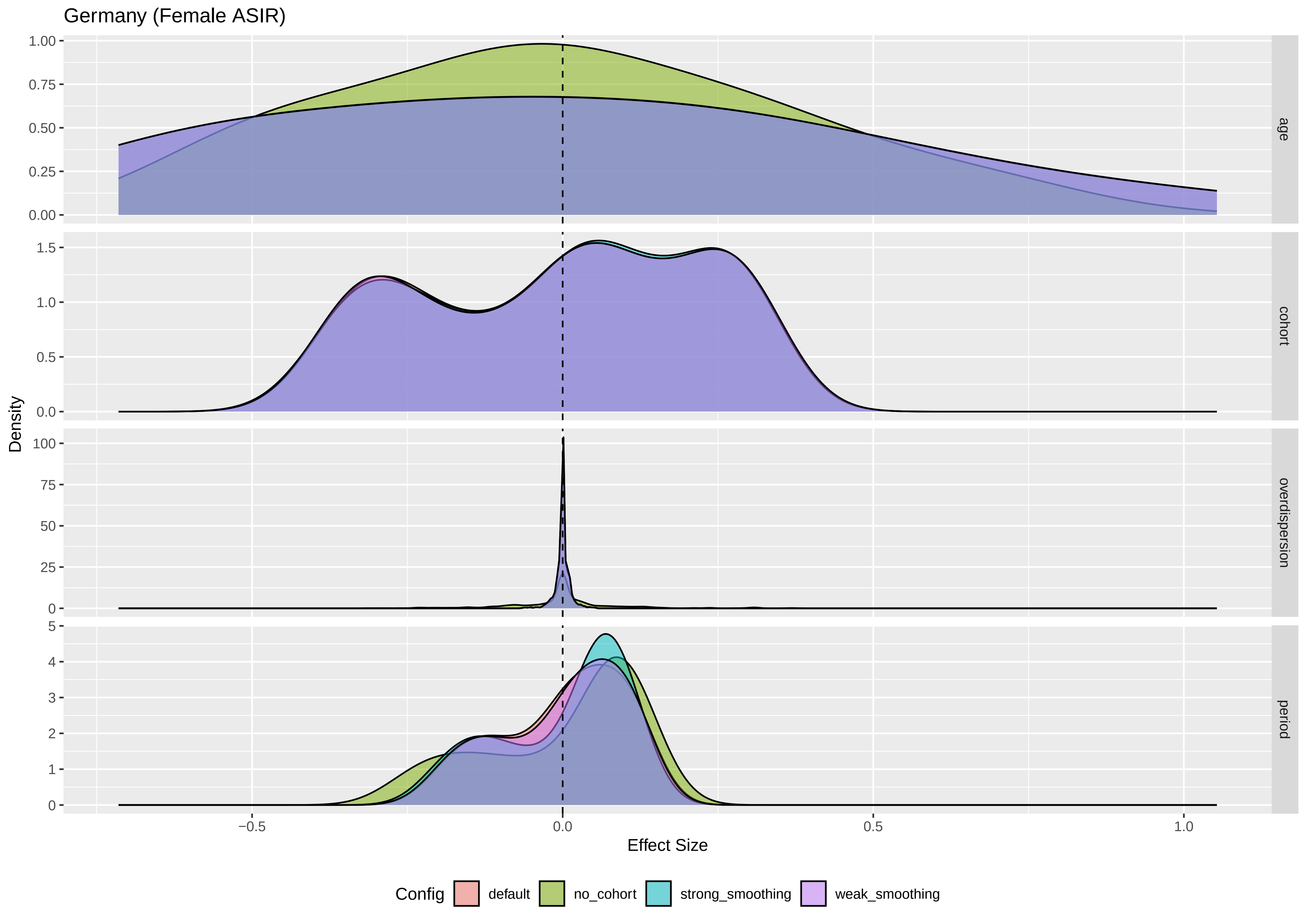

Germany (Female ASIR)
age
cohort
overdispersion
period
Density
Effect Size
Config
default
no_cohort
strong_smoothing
weak_smoothing

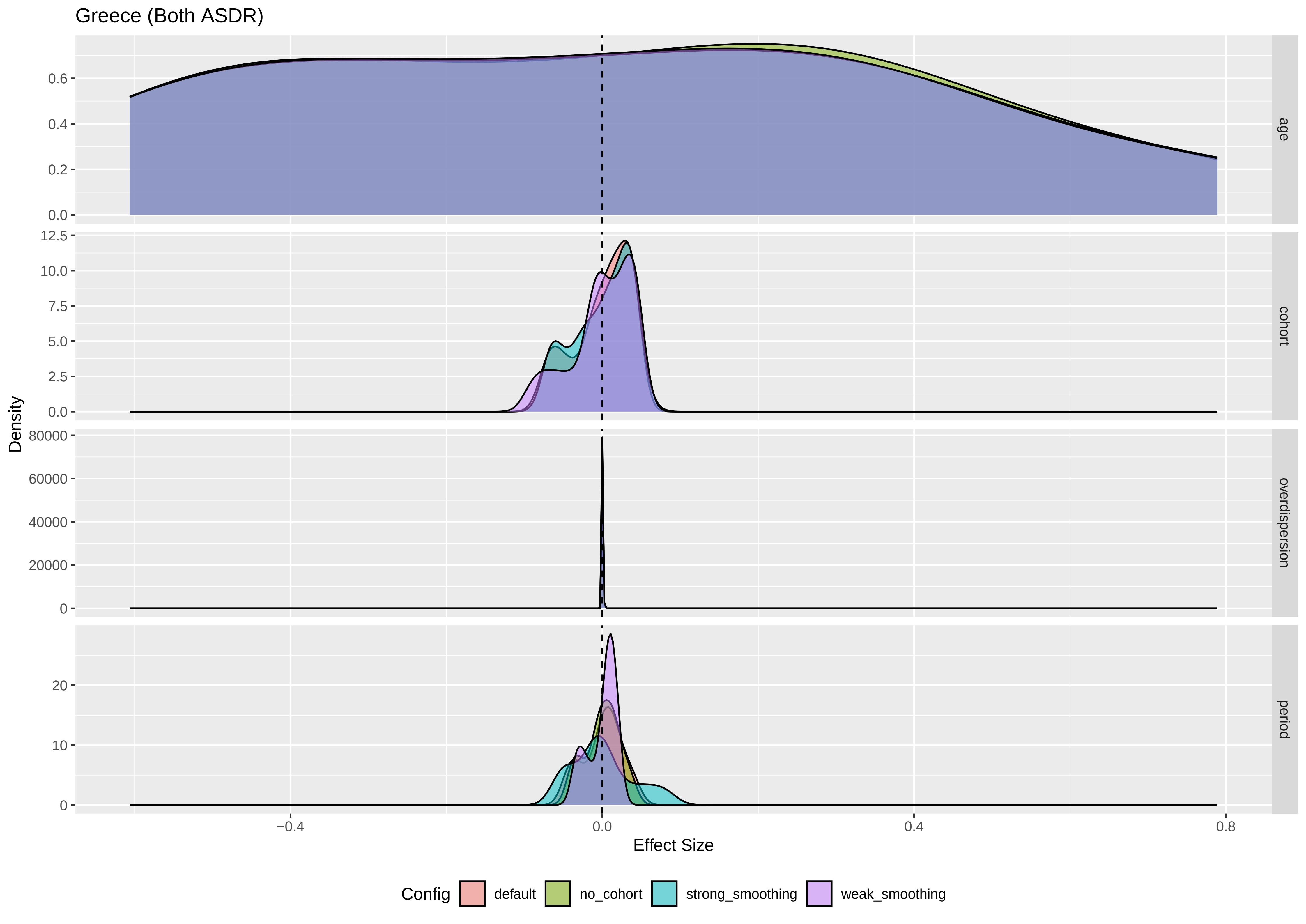
Greece (Both ASDR)
age
cohort
overdispersion
period
Density
Effect Size
0.0
0.2
0.4
0.6
12.5
10.0
7.5
5.0
2.5
80000
60000
40000
20000
0
20
10
−0.4
0.0
0.4
0.8
Config
default
no_cohort
strong_smoothing
weak_smoothing

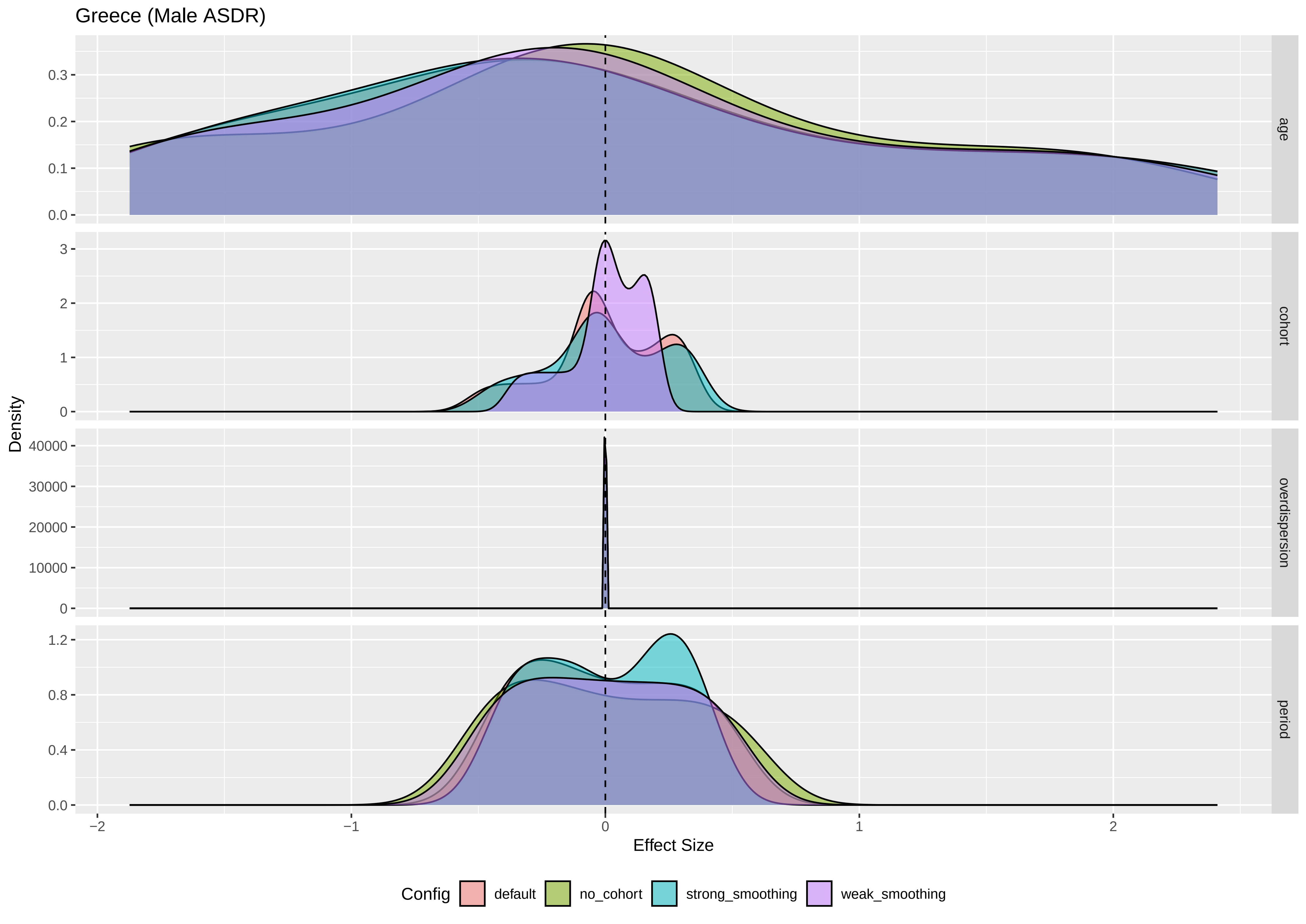
Greece (Male ASDR)
Density
Effect Size
age
cohort
overdispersion
period
0.0
0.1
0.2
0.3
0
1
2
3
0
10000
20000
30000
40000
0.0
0.4
0.8
1.2
−2
−1
0
1
2
Config
default
no_cohort
strong_smoothing
weak_smoothing

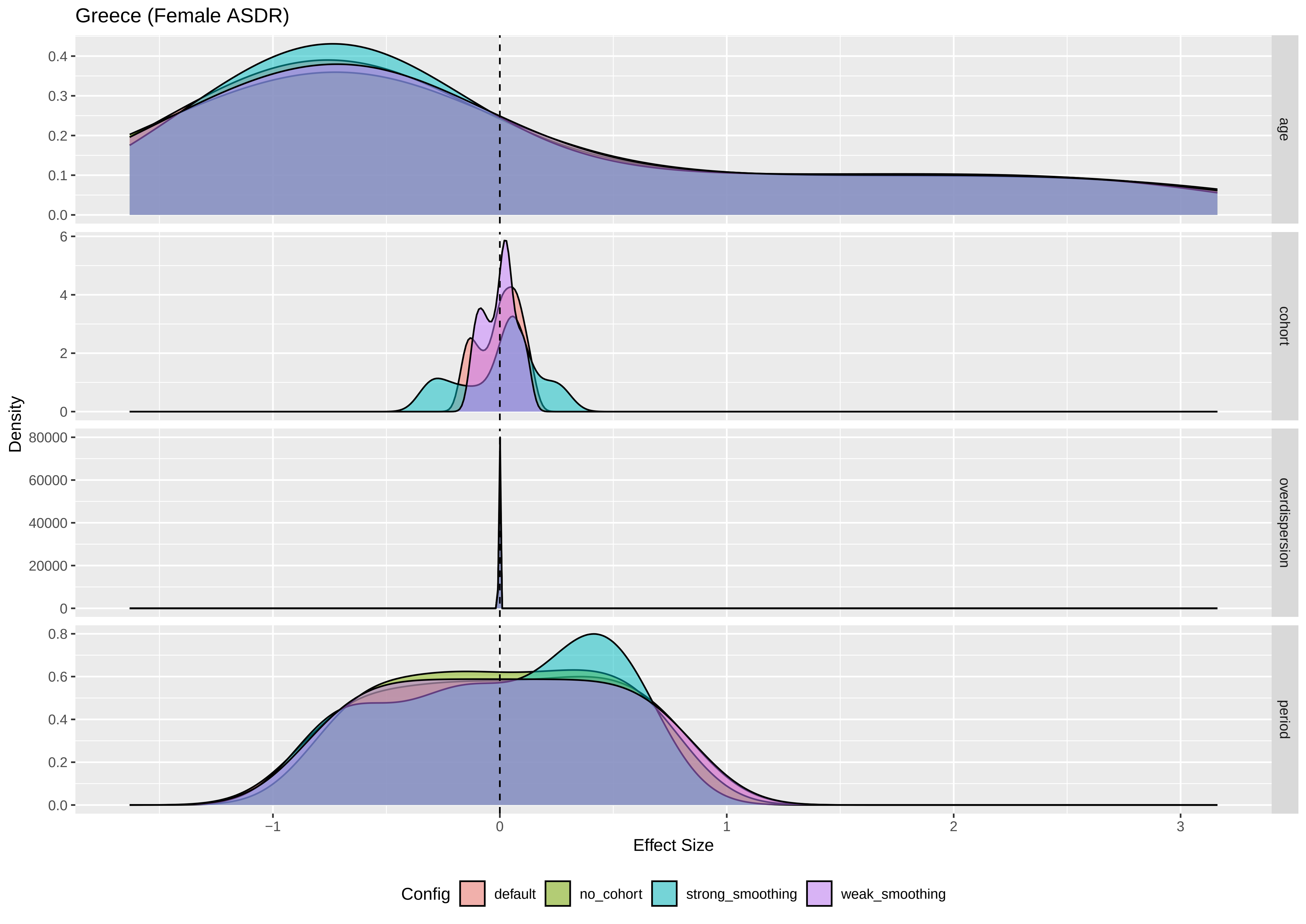

Greece (Female ASDR)
age
cohort
overdispersion
period
Density
Effect Size
0.0
0.1
0.2
0.3
0.4
0
2
4
6
0
20000
40000
60000
80000
0.0
0.2
0.4
0.6
0.8
−1
0
1
2
3
Config
default
no_cohort
strong_smoothing
weak_smoothing

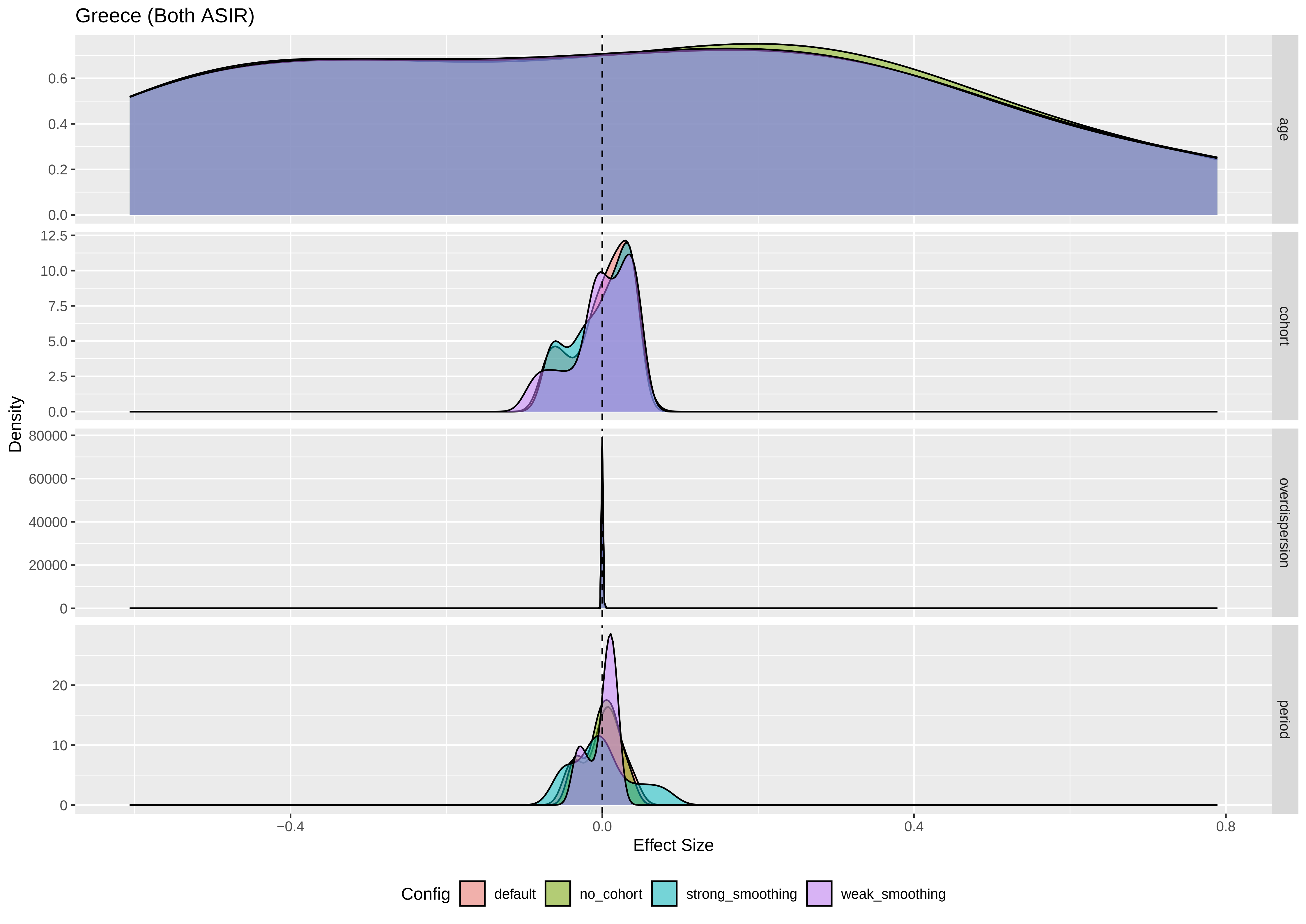

Greece (Both ASIR)
age
cohort
overdispersion
period
Density
Effect Size
0.0
0.2
0.4
0.6
12.5
10.0
7.5
5.0
2.5
0.0
80000
60000
40000
20000
0
20
10
0
−0.4
0.0
0.4
0.8
Config
default
no_cohort
strong_smoothing
weak_smoothing

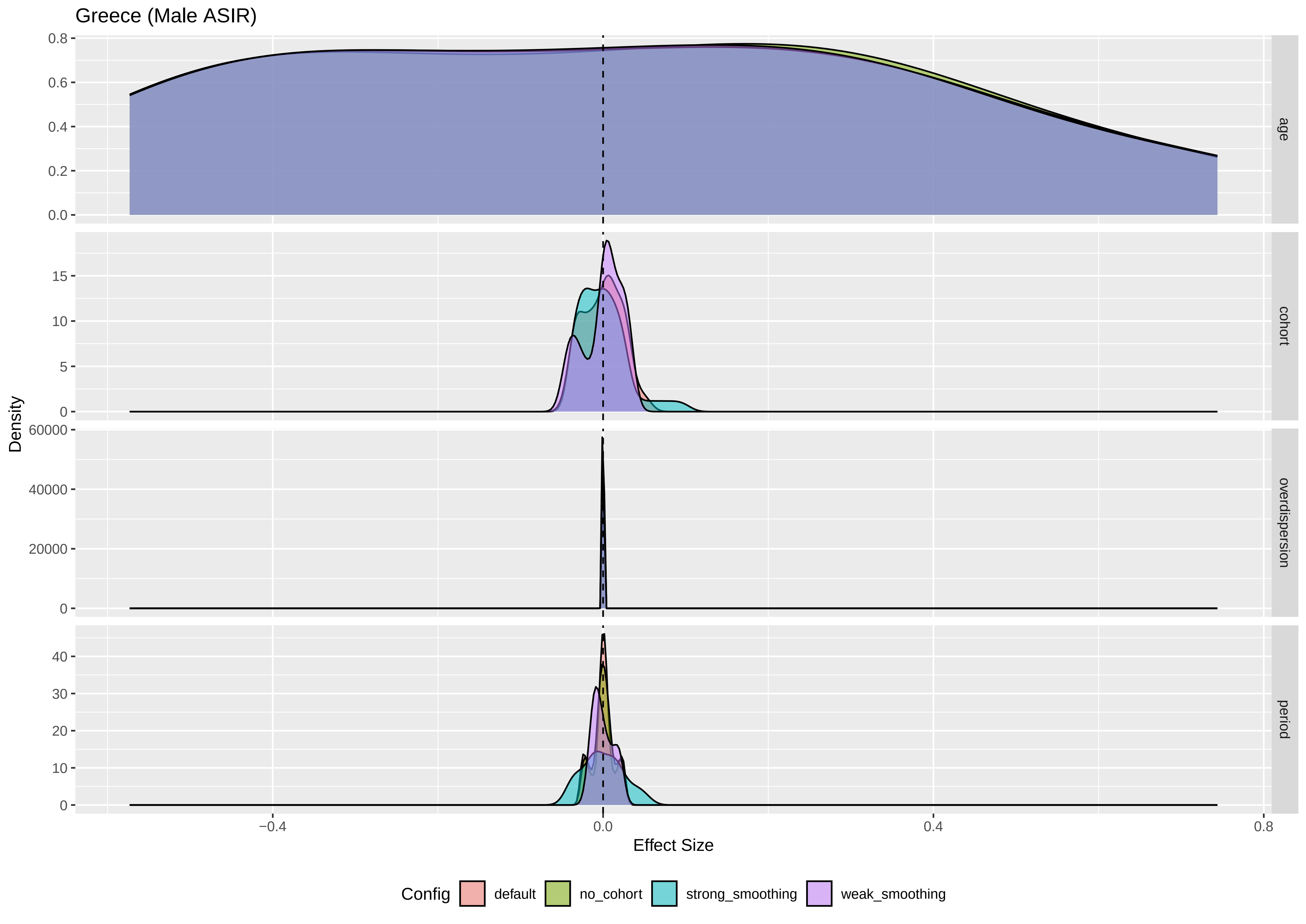

Greece (Male ASIR)
0.8
0.6
0.4
0.2
0.0
age
15
10
5
0
cohort
60000
40000
20000
0
overdispersion
40
30
20
10
0
period
Density
−0.4
0.0
0.4
0.8
Effect Size
Config
default
no_cohort
strong_smoothing
weak_smoothing

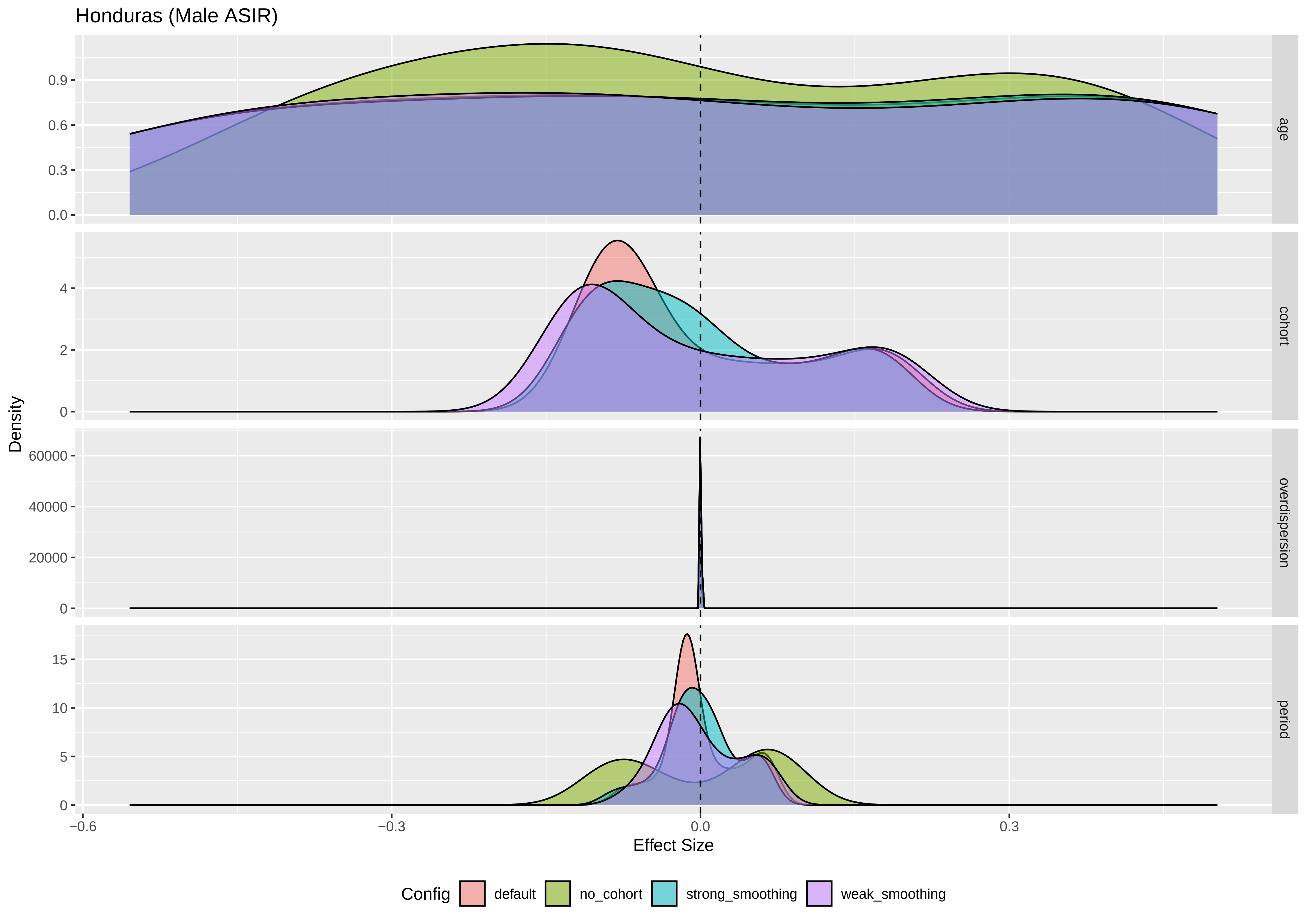

Honduras (Male ASIR)
0.9
0.6
0.3
0.0
age
4
2
0
cohort
60000
40000
20000
0
overdispersion
15
10
5
0
period
Density
−0.6
−0.3
0.0
0.3
Effect Size
Config
default
no_cohort
strong_smoothing
weak_smoothing

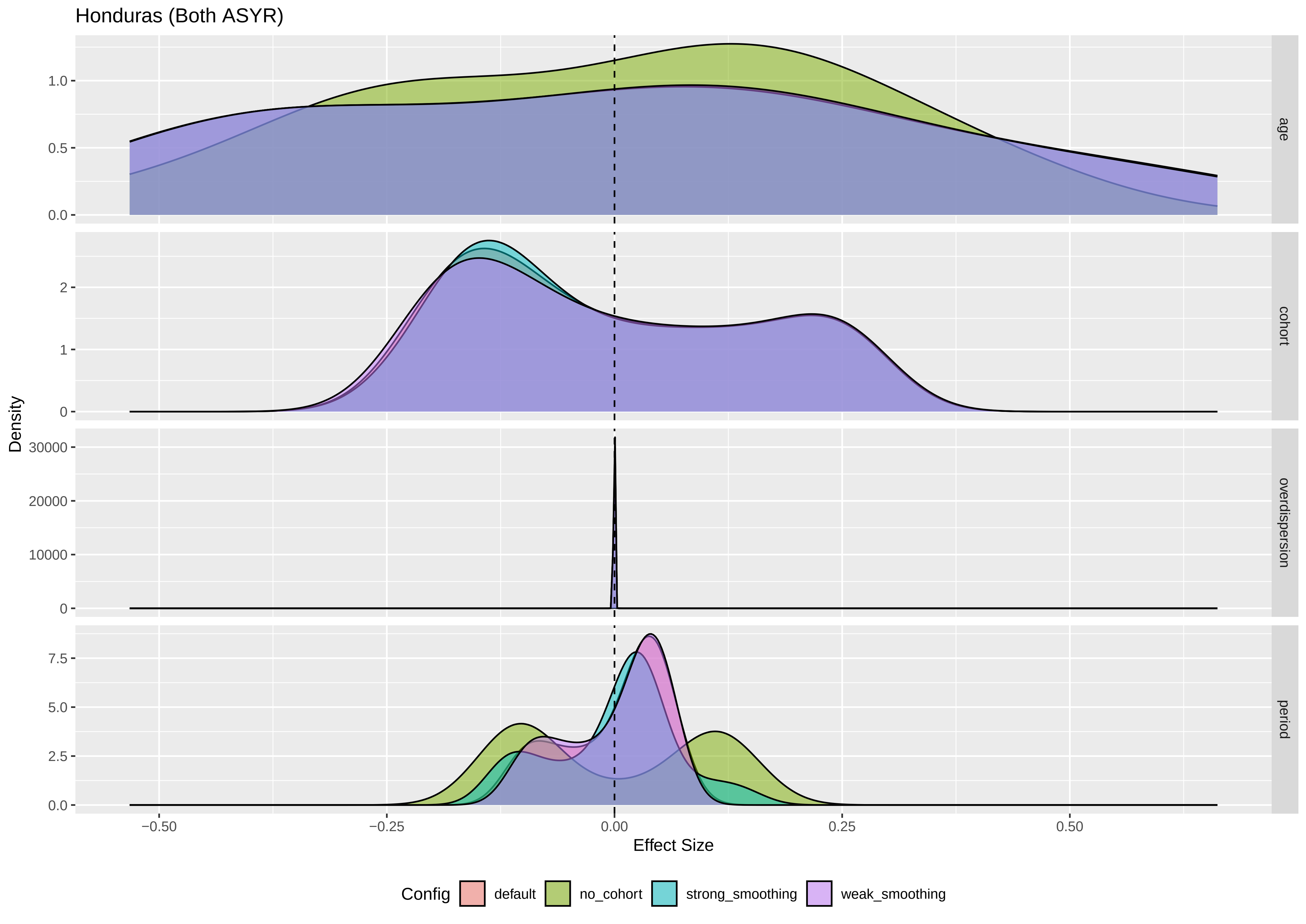
Honduras (Both ASYR)
Density
Effect Size
age
cohort
overdispersion
period
1.0
0.5
0.0
2
1
0
30000
20000
10000
0
7.5
5.0
2.5
0.0
−0.50
−0.25
0.00
0.25
0.50
Config
default
no_cohort
strong_smoothing
weak_smoothing

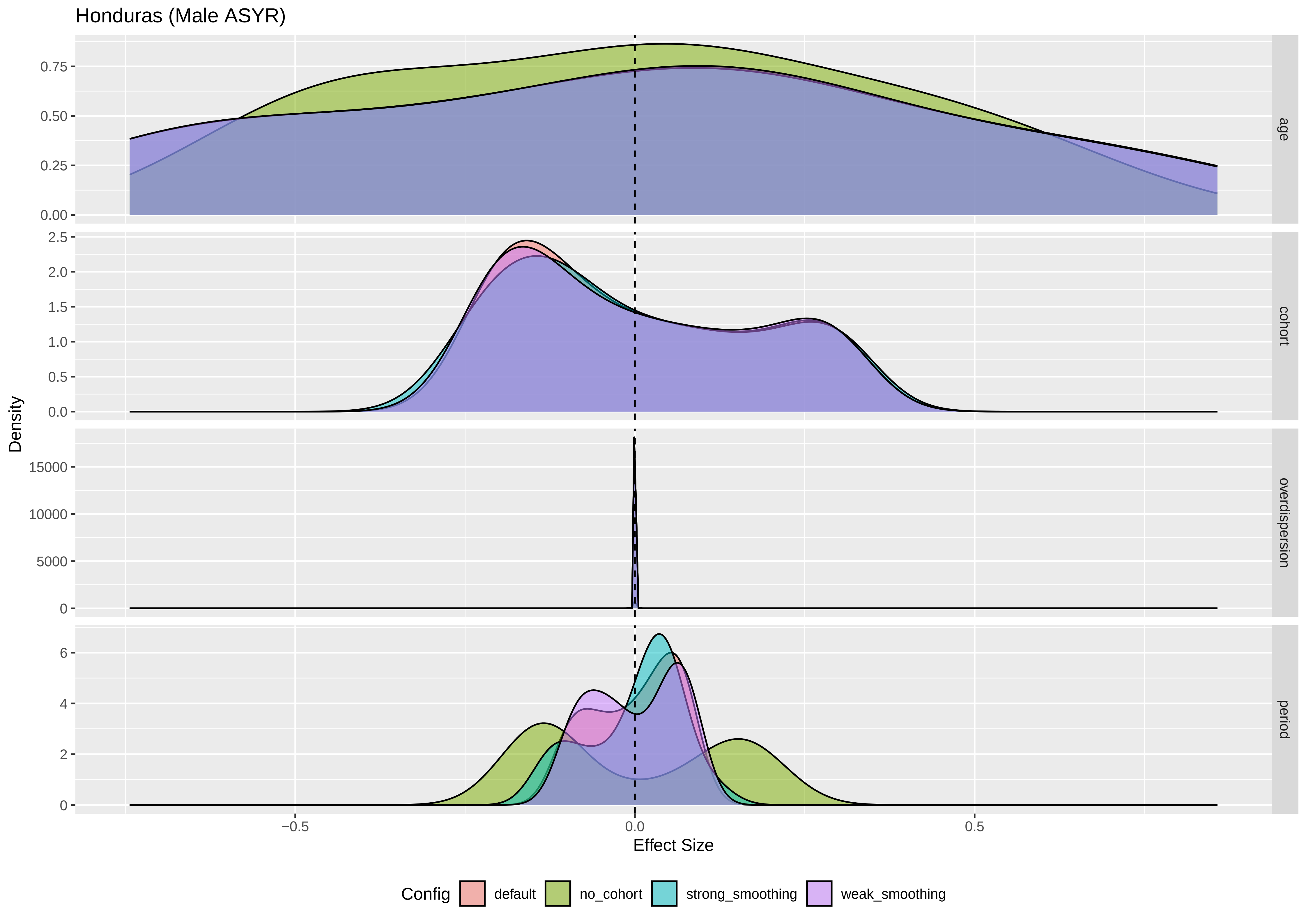

Honduras (Male ASYR)
age
cohort
overdispersion
period
Density
Effect Size
0.00
0.25
0.50
0.75
0.0
0.5
1.0
1.5
2.0
2.5
0
5000
10000
15000
0
2
4
6
−0.5
0.0
0.5
Config
default
no_cohort
strong_smoothing
weak_smoothing

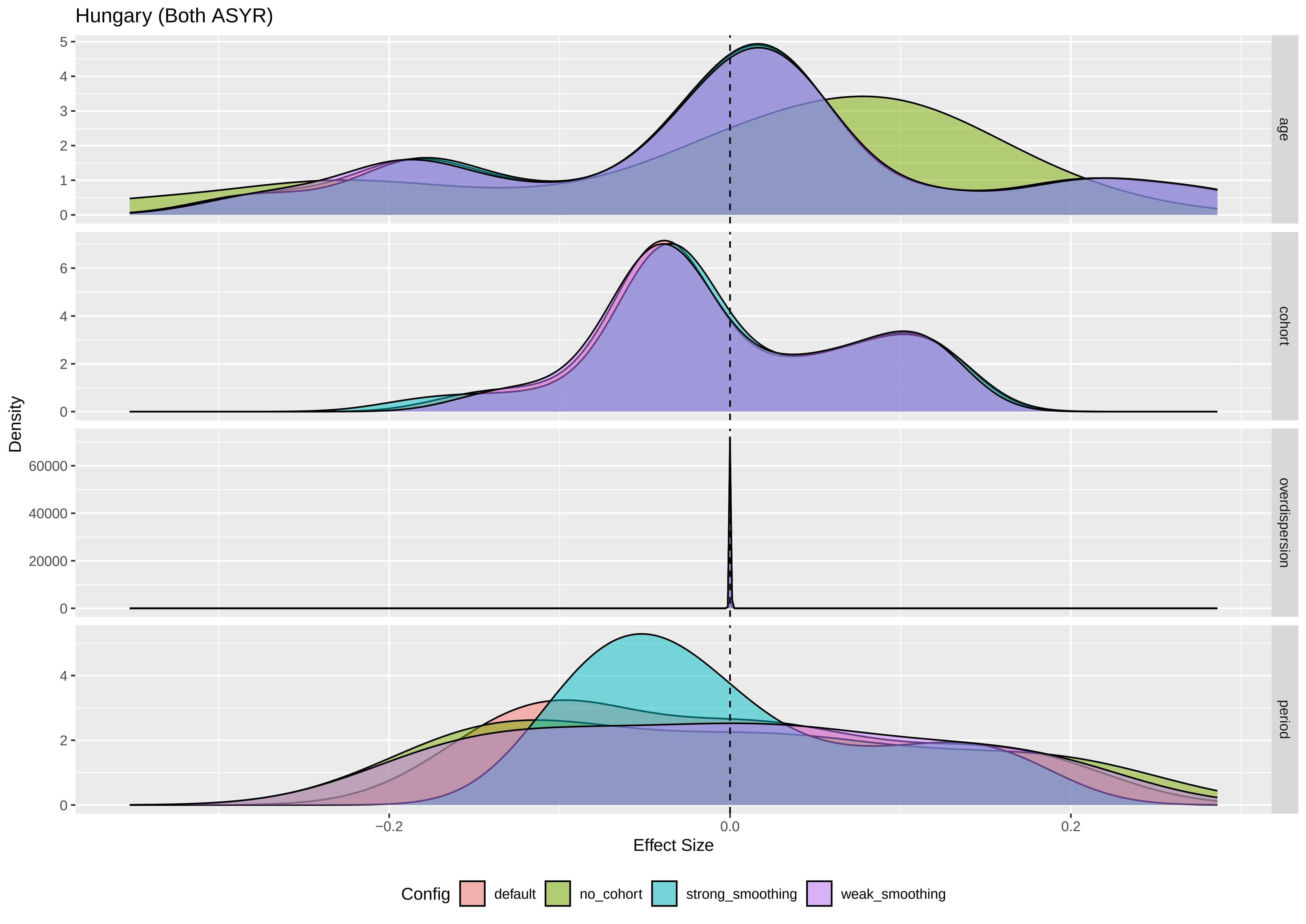

Hungary (Both ASYR)
Density
age
cohort
overdispersion
period
0
1
2
3
4
5
6
20000
40000
60000
−0.2
0.0
0.2
Effect Size
Config
default
no_cohort
strong_smoothing
weak_smoothing

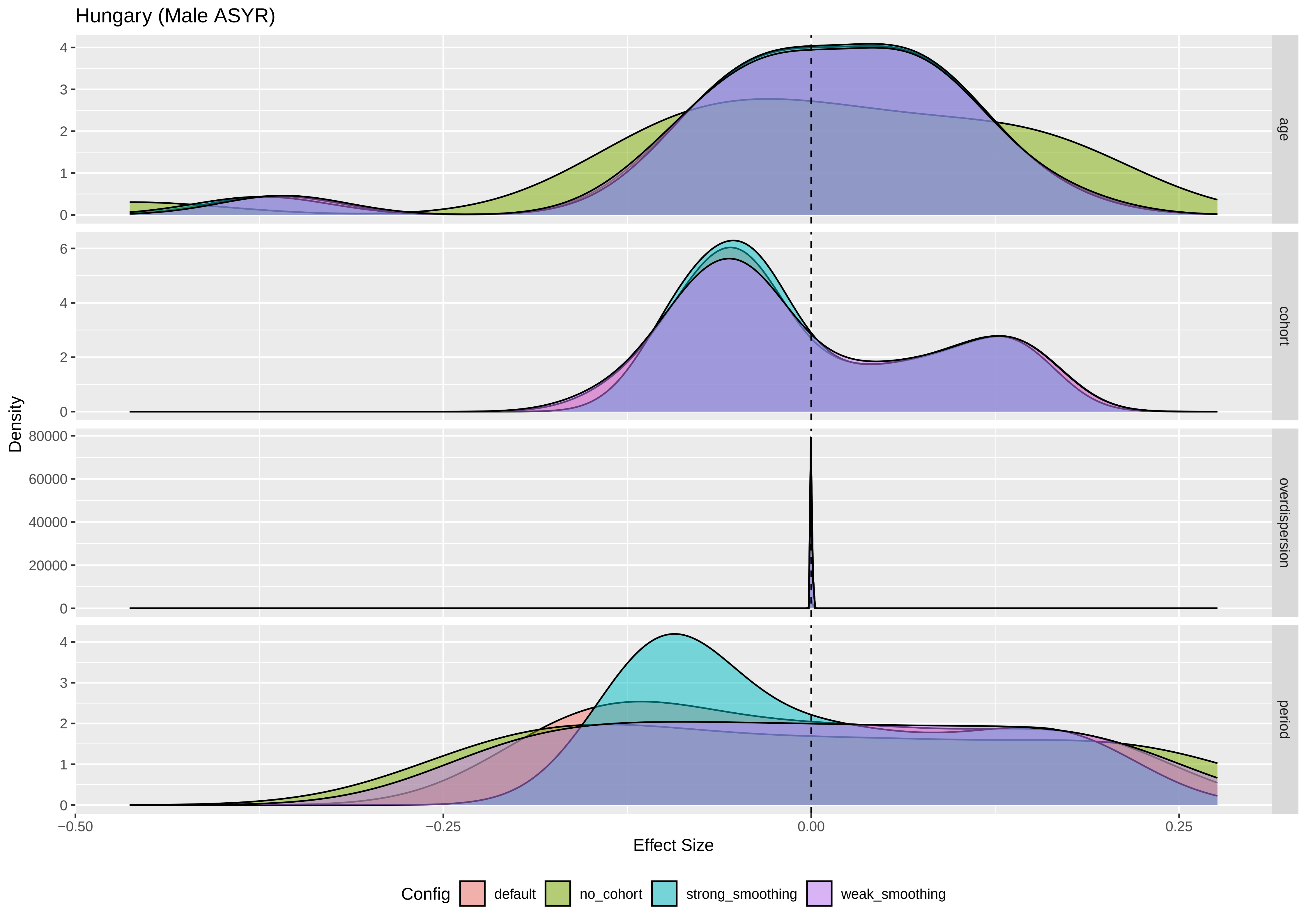

Hungary (Male ASYR)
Density
Effect Size
age
cohort
overdispersion
period
4
3
2
1
0
6
4
2
0
80000
60000
40000
20000
0
4
3
2
1
0
−0.50
−0.25
0.00
0.25
Config
default
no_cohort
strong_smoothing
weak_smoothing

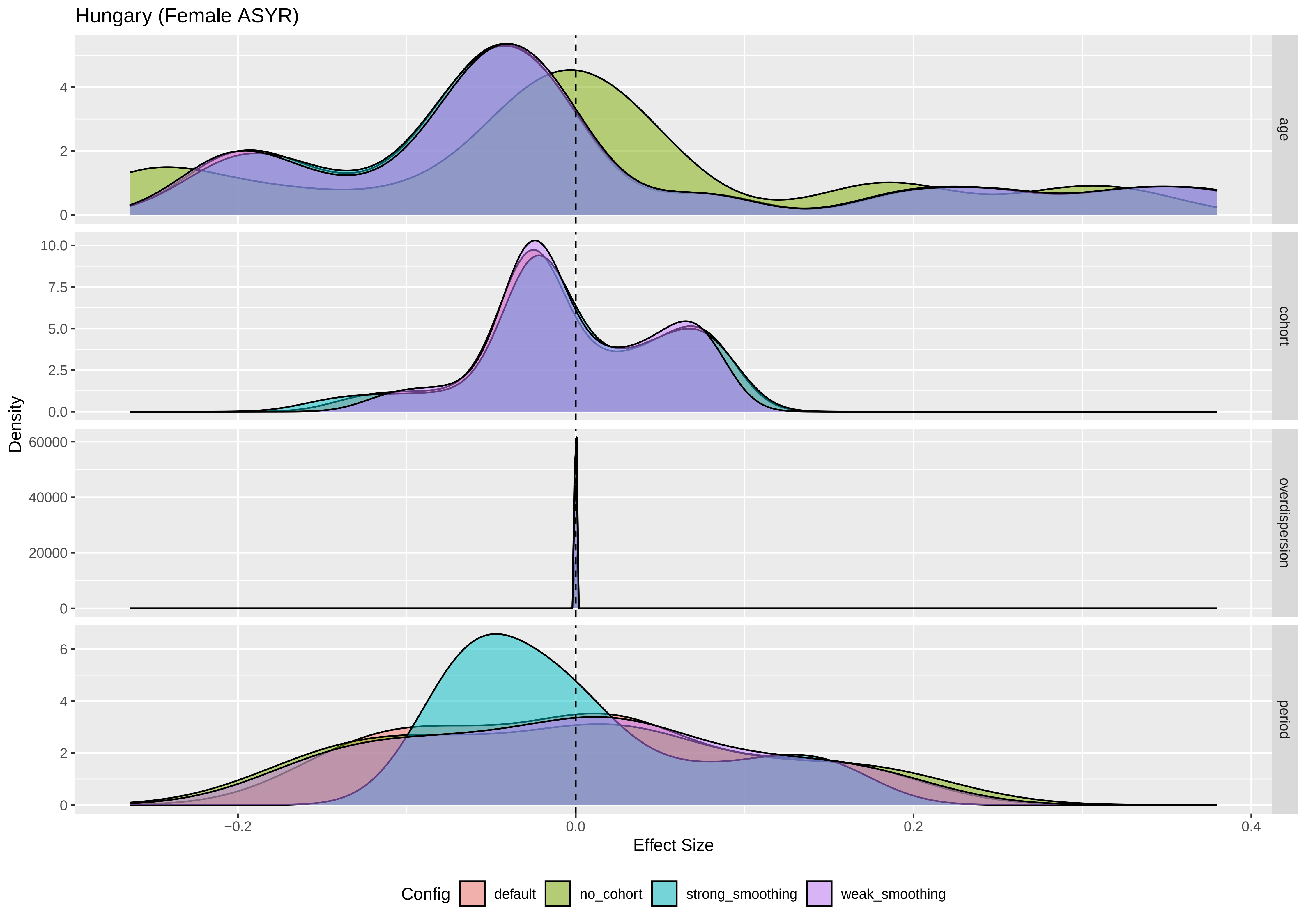

Hungary (Female ASYR)
Density
Effect Size
age
cohort
overdispersion
period
0
2
4
0.0
2.5
5.0
7.5
10.0
0
20000
40000
60000
6
−0.2
0.0
0.2
0.4
Config
default
no_cohort
strong_smoothing
weak_smoothing

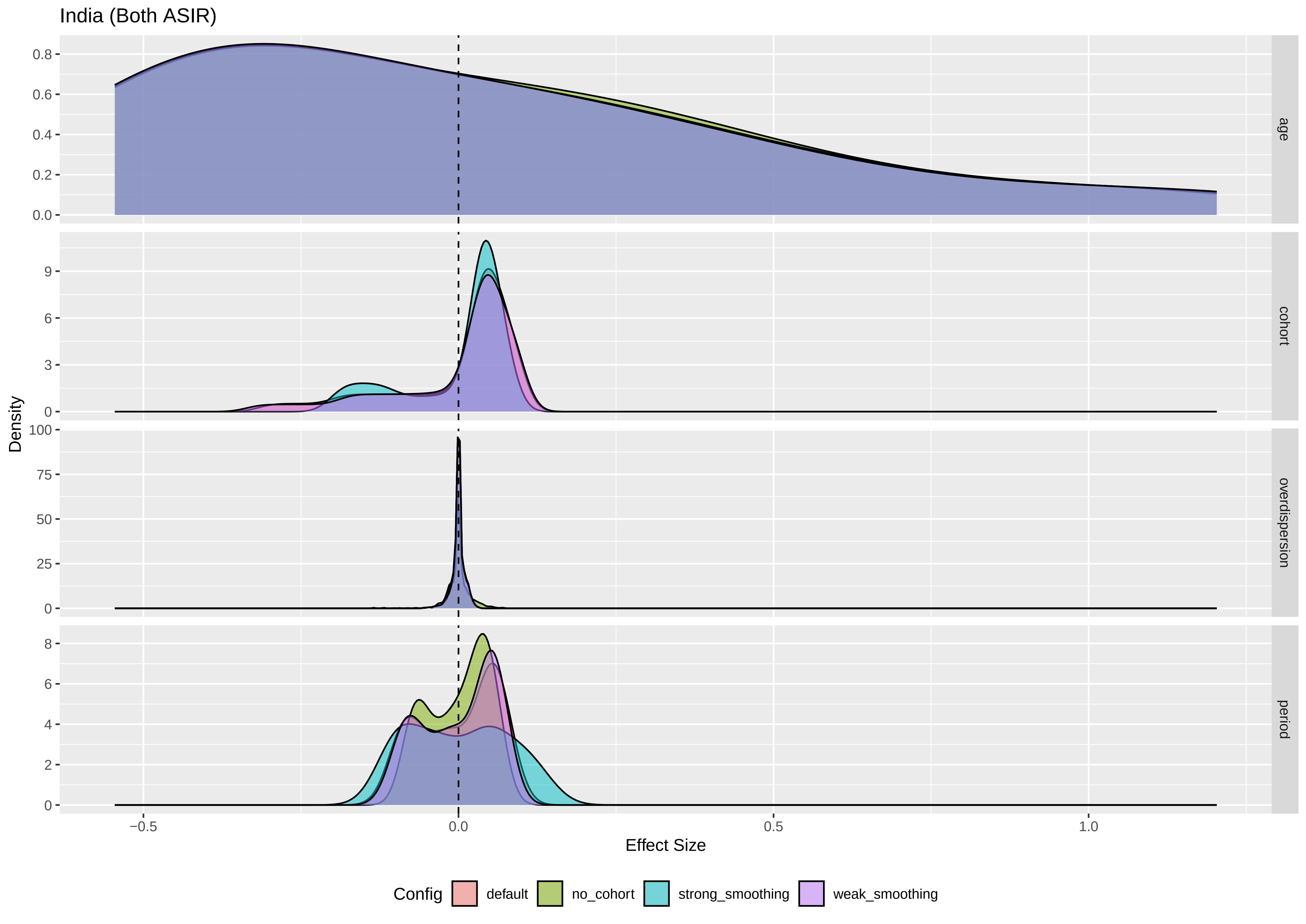

India (Both ASIR)
Density
0.8
0.6
0.4
0.2
0.0
age
9
6
3
0
cohort
100
75
50
25
0
overdispersion
8
6
4
2
0
period
−0.5
0.0
0.5
1.0
Effect Size
Config
default
no_cohort
strong_smoothing
weak_smoothing

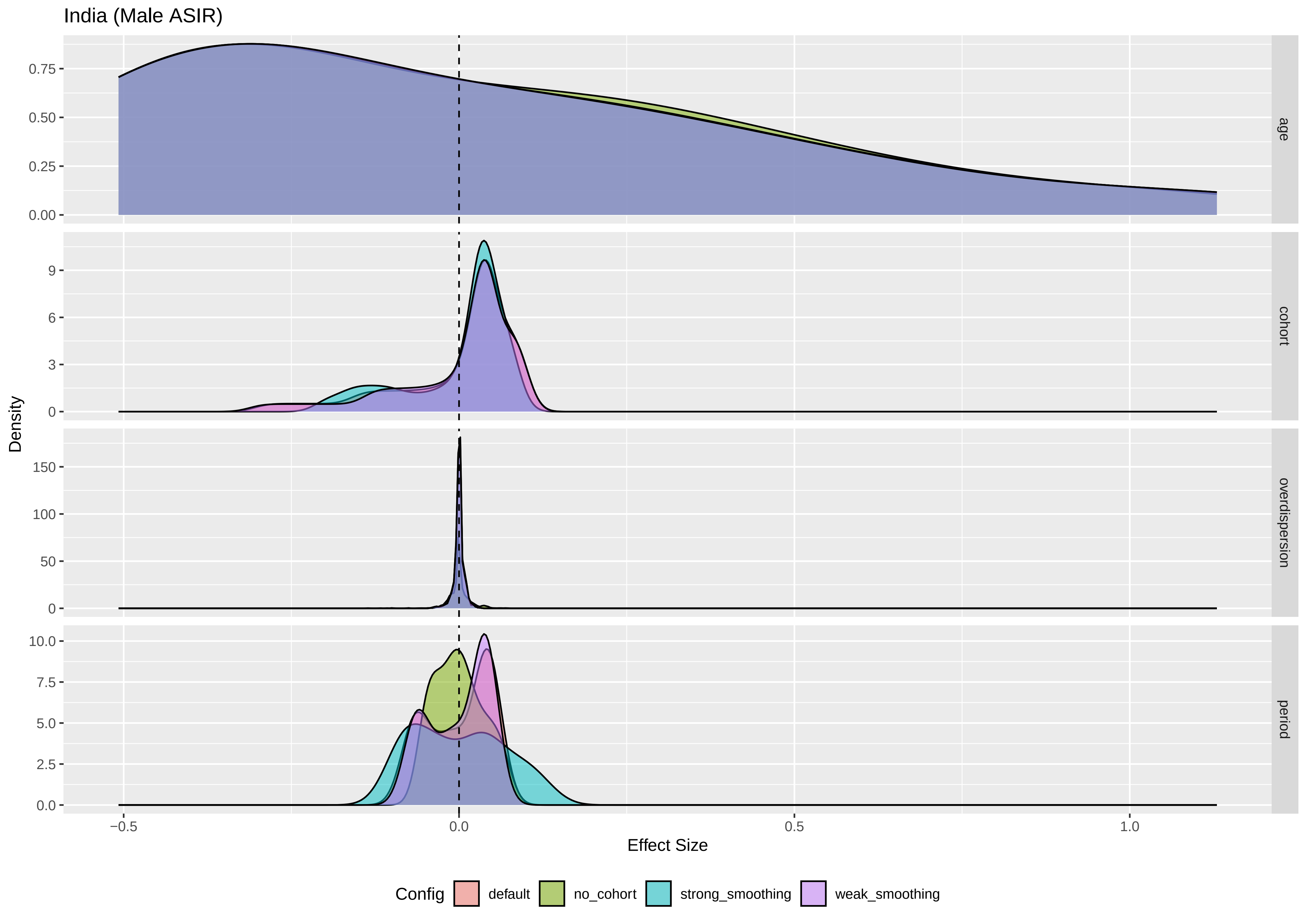
India (Male ASIR)
Density
age
cohort
overdispersion
period
0.75
0.50
0.25
0.00
9
6
3
0
150
100
50
0
10.0
7.5
5.0
2.5
0.0
-0.5
0.0
0.5
1.0
Effect Size
Config
default
no_cohort
strong_smoothing
weak_smoothing

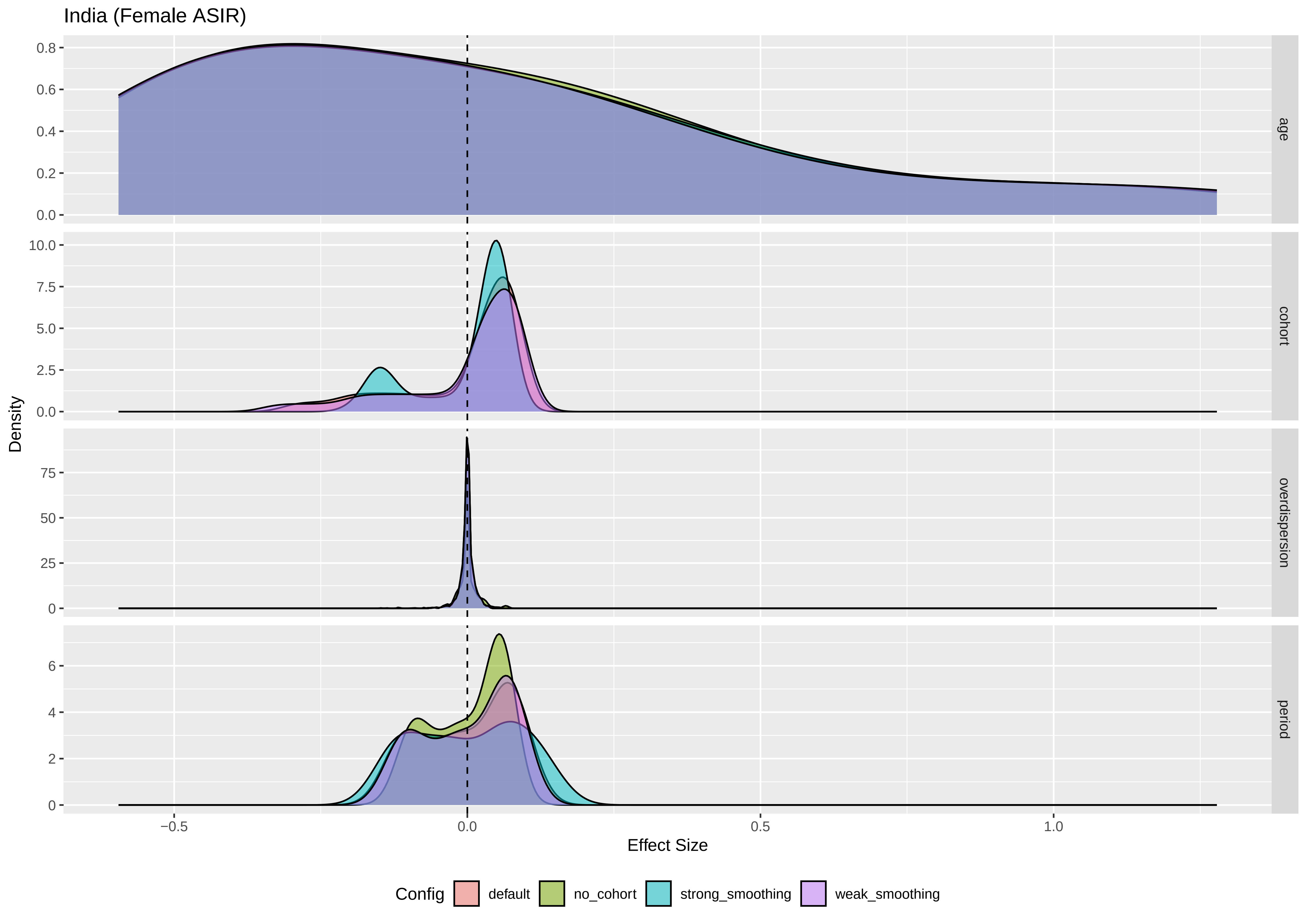
India (Female ASIR)
Density
Effect Size
age
cohort
overdispersion
period
−0.5
0.0
0.5
1.0
Config
default
no_cohort
strong_smoothing
weak_smoothing

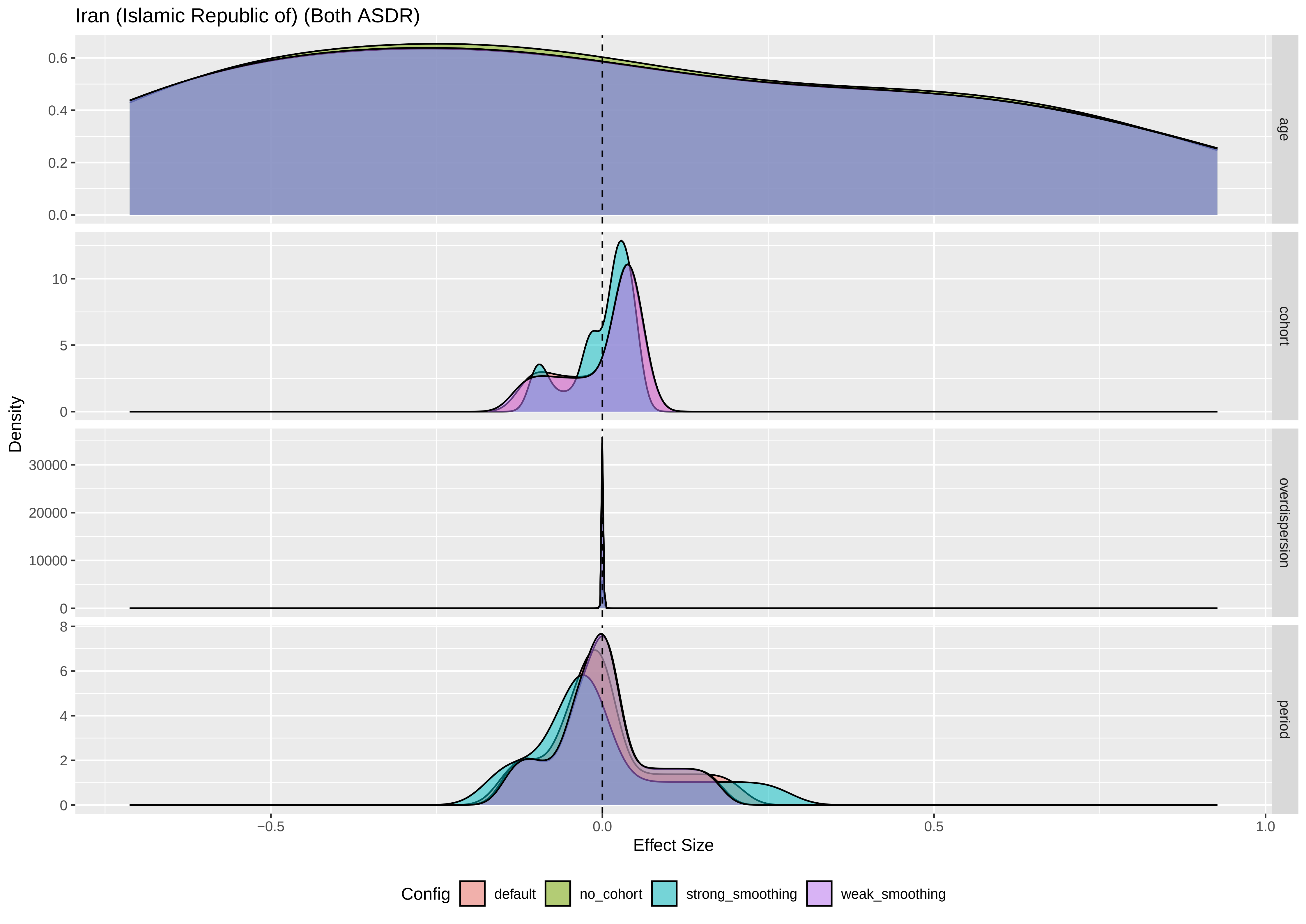

Iran (Islamic Republic of) (Both ASDR)
age
cohort
overdispersion
period
Density
Effect Size
−0.5
0.0
0.5
1.0
0.0
0.2
0.4
0.6
0
5
10
10000
20000
30000
2
4
6
8
Config
default
no_cohort
strong_smoothing
weak_smoothing

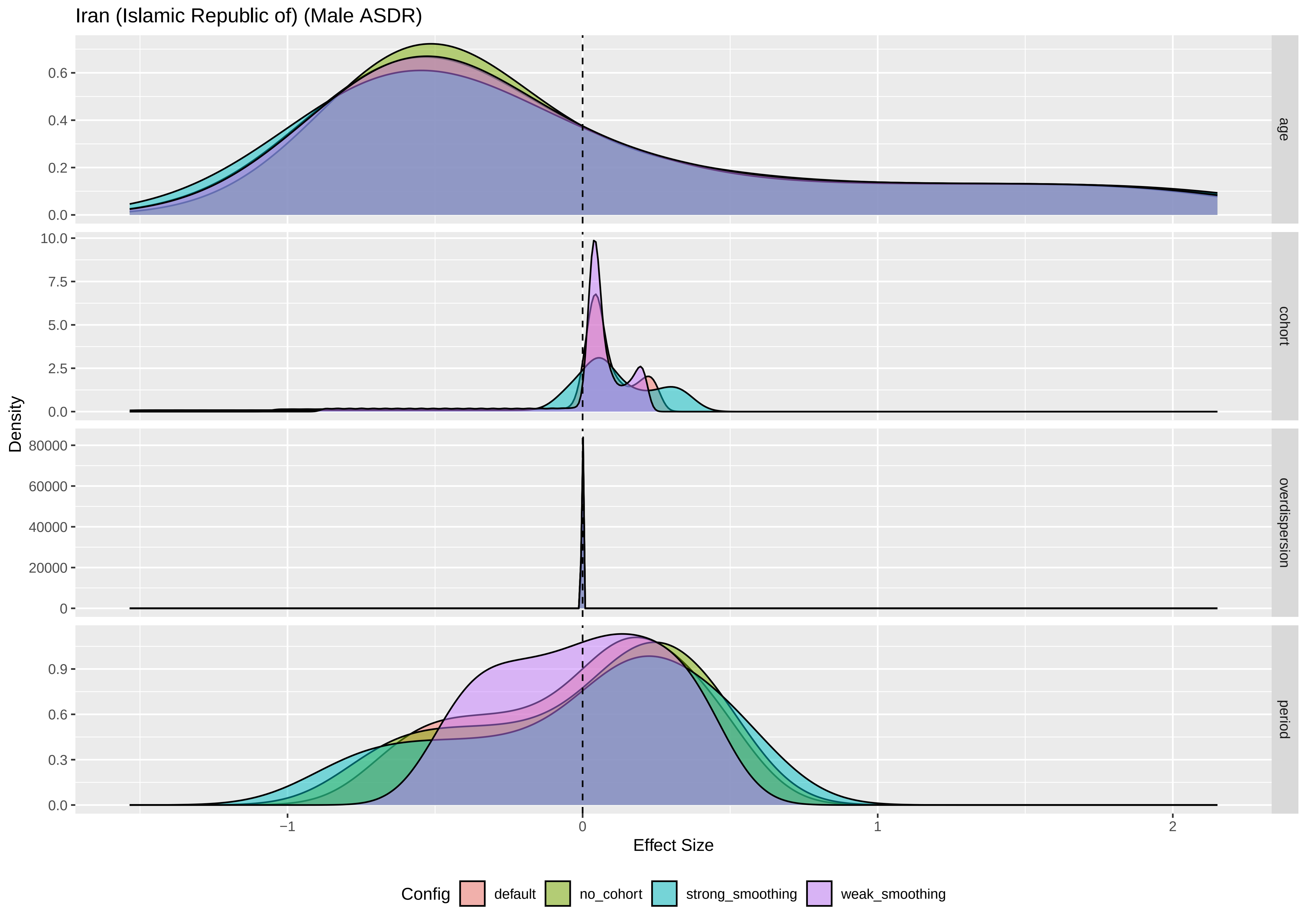

Iran (Islamic Republic of) (Male ASDR)
age
cohort
overdispersion
period
Density
Effect Size
0.0
0.2
0.4
0.6
0.0
2.5
5.0
7.5
10.0
0
20000
40000
60000
80000
0.0
0.3
0.6
0.9
−1
0
1
2
Config
default
no_cohort
strong_smoothing
weak_smoothing

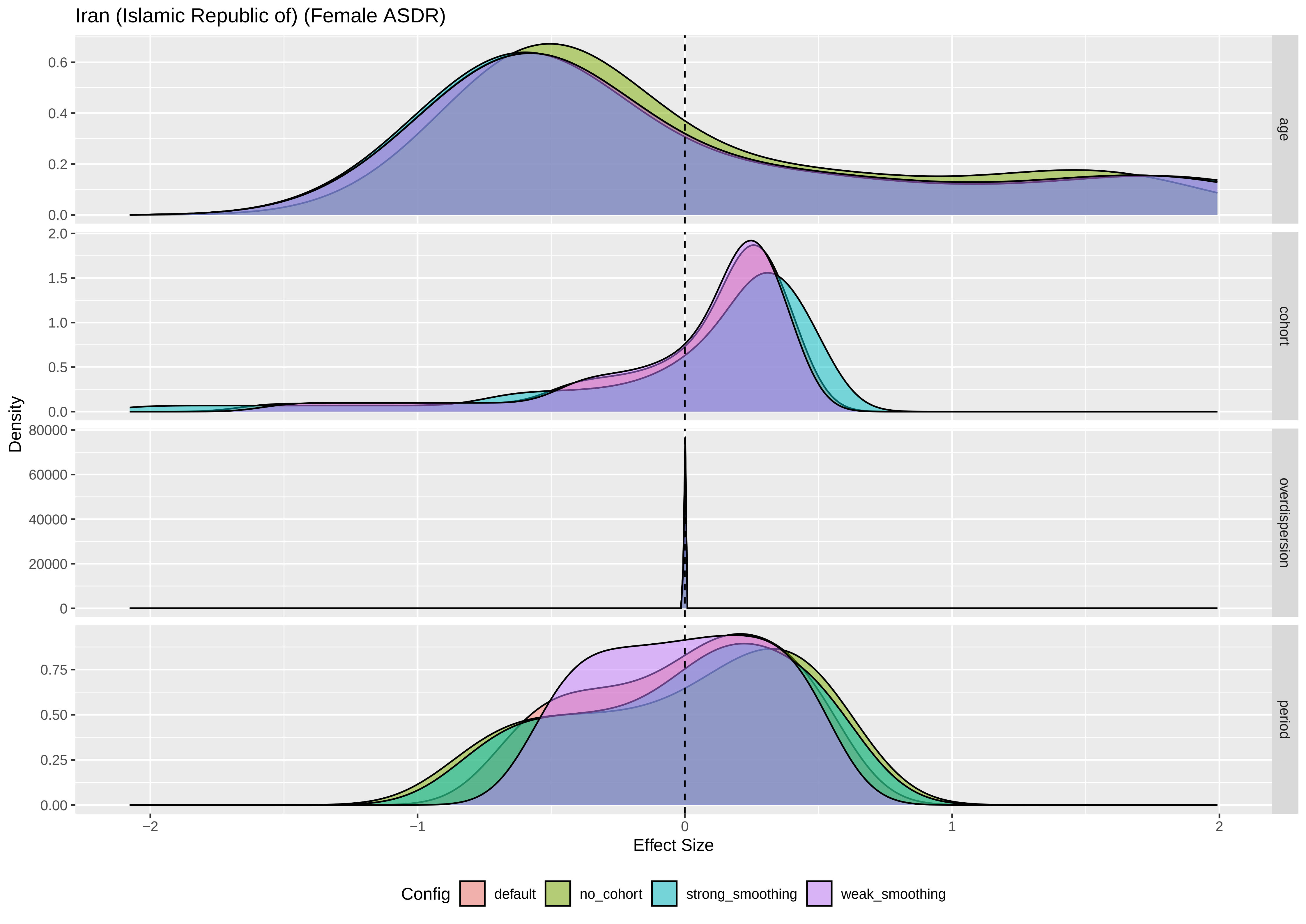

Iran (Islamic Republic of) (Female ASDR)
Density
Effect Size
age
cohort
overdispersion
period
0.0
0.2
0.4
0.6
0.5
1.0
1.5
2.0
0
20000
40000
60000
80000
0.00
0.25
0.50
0.75
−2
−1
0
1
2
Config
default
no_cohort
strong_smoothing
weak_smoothing

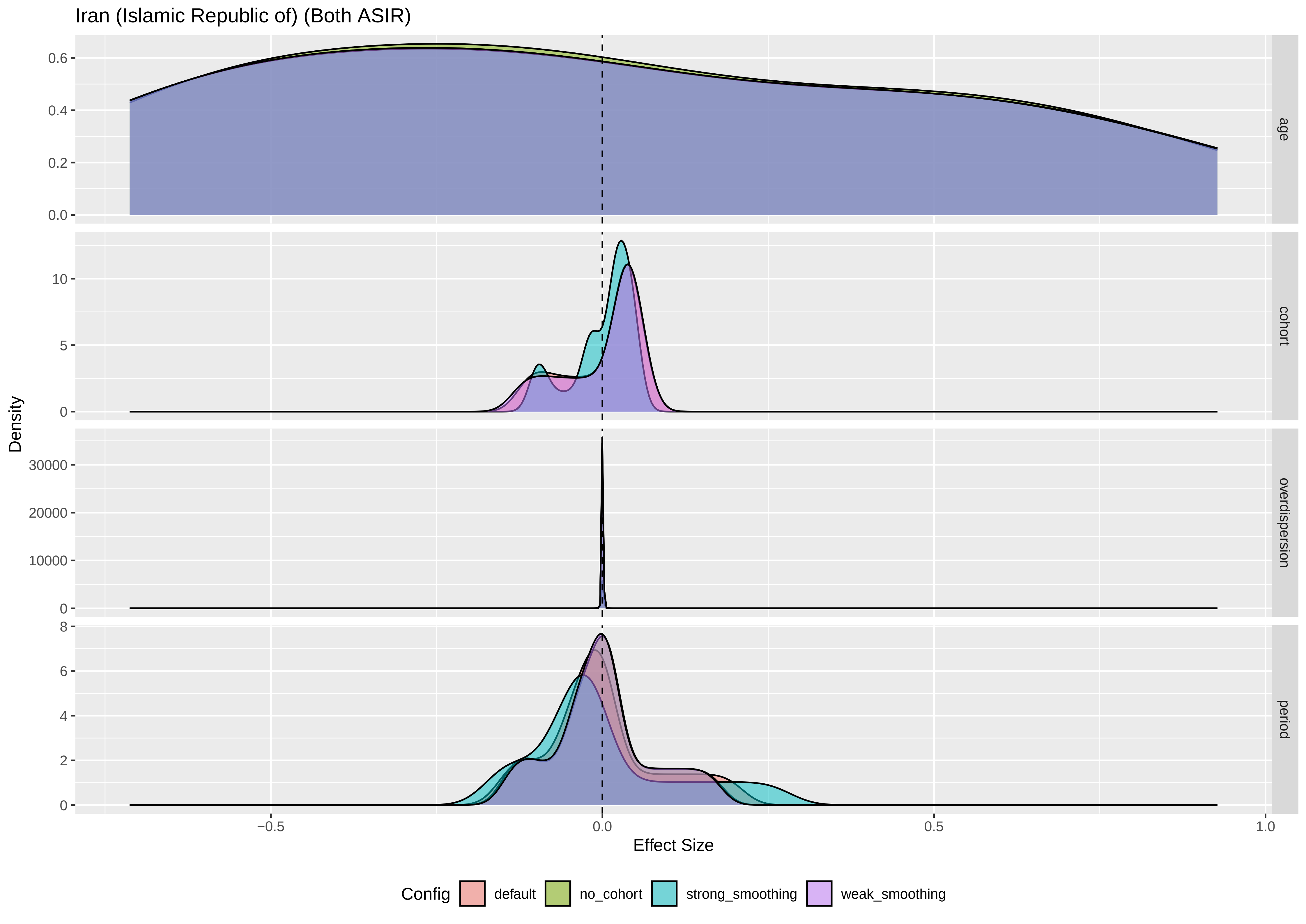
Iran (Islamic Republic of) (Both ASIR)
Density
Effect Size
age
cohort
overdispersion
period
0.0
0.2
0.4
0.6
0
5
10
0
10000
20000
30000
0
2
4
6
8
−0.5
0.0
0.5
1.0
Config
default
no_cohort
strong_smoothing
weak_smoothing

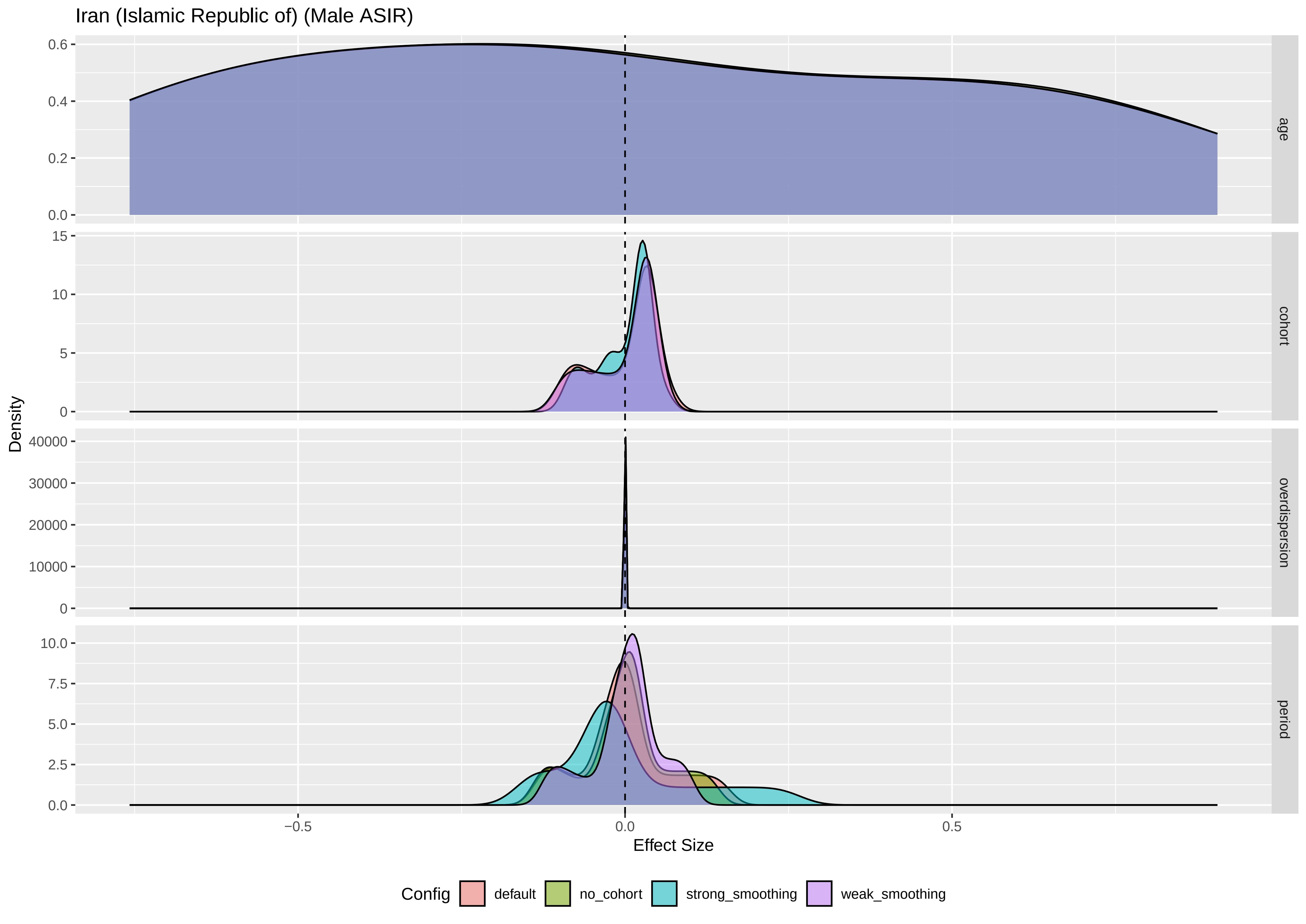

Iran (Islamic Republic of) (Male ASIR)
Density
Effect Size
age
cohort
overdispersion
period
0.6
0.4
0.2
0.0
15
10
5
0
40000
30000
20000
10000
0
10.0
7.5
5.0
2.5
0.0
−0.5
0.0
0.5
Config
default
no_cohort
strong_smoothing
weak_smoothing

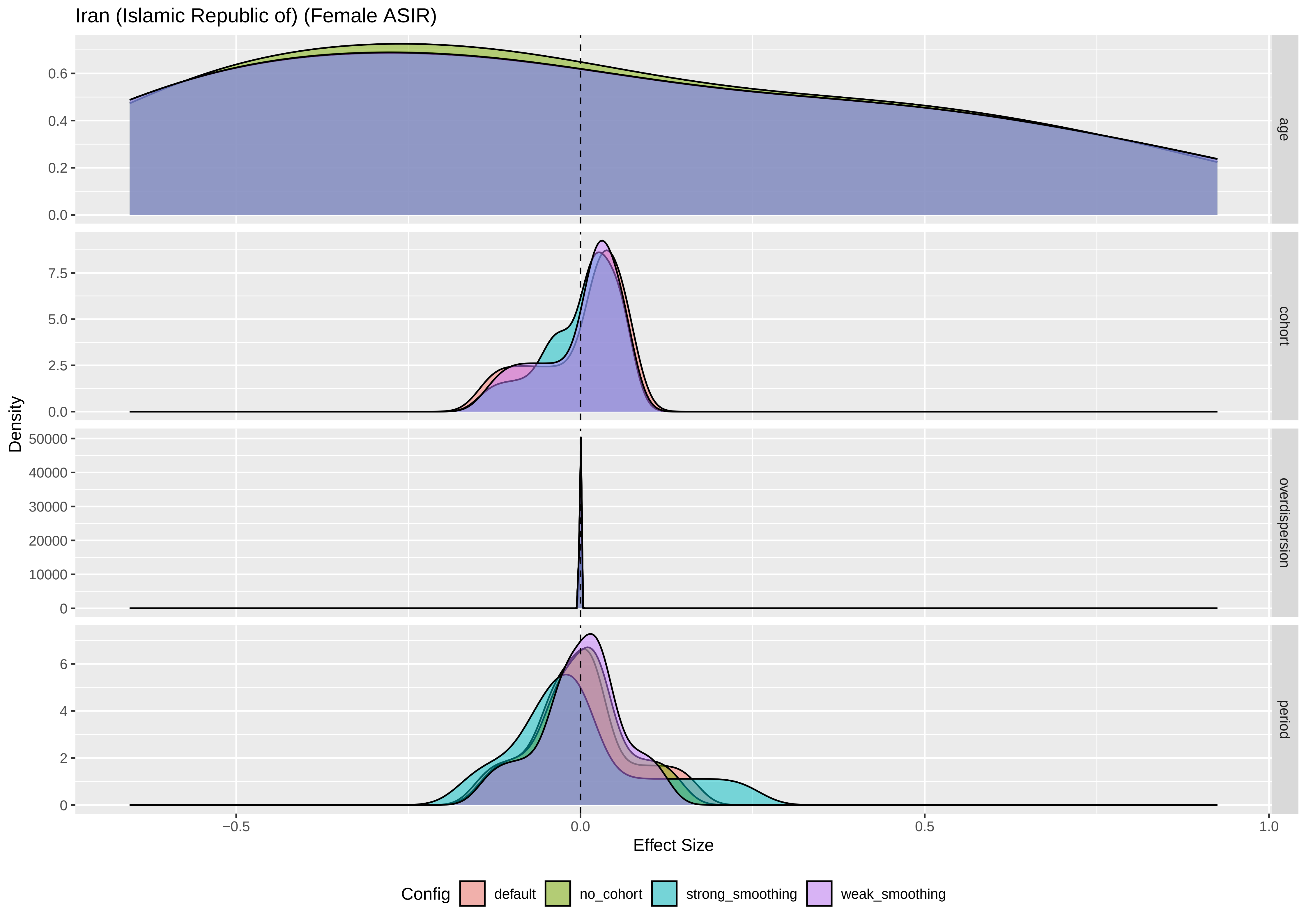

Iran (Islamic Republic of) (Female ASIR)
age
cohort
overdispersion
period
Density
Effect Size
−0.5
0.0
0.5
1.0
0.0
0.2
0.4
0.6
2.5
5.0
7.5
0
10000
20000
30000
40000
50000
2
4
6
Config
default
no_cohort
strong_smoothing
weak_smoothing

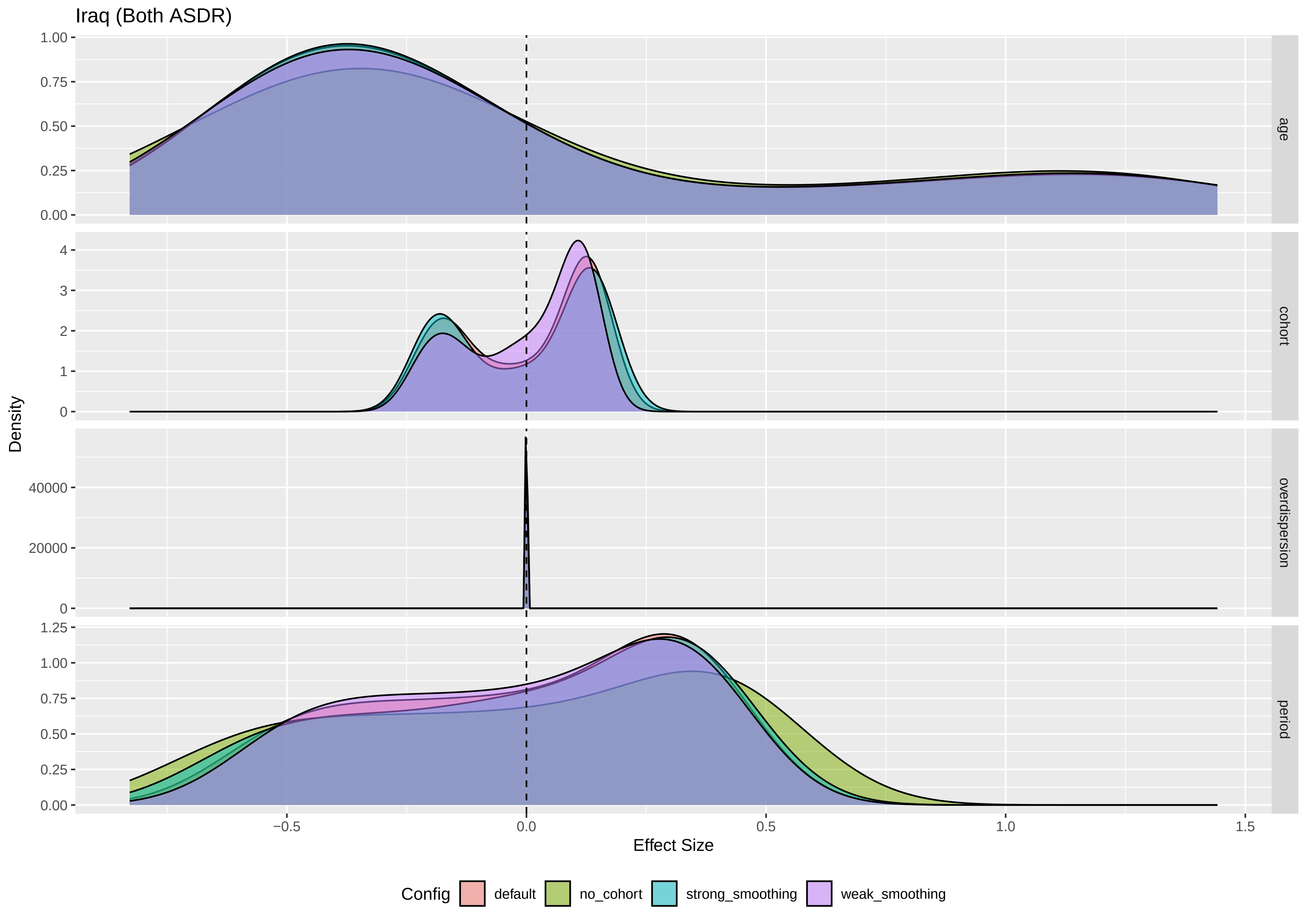

Iraq (Both ASDR)
age
cohort
overdispersion
period
Density
Effect Size
1.00
0.75
0.50
0.25
0.00
4
3
2
1
0
40000
20000
0
1.25
−0.5
0.0
0.5
1.0
1.5
Config
default
no_cohort
strong_smoothing
weak_smoothing

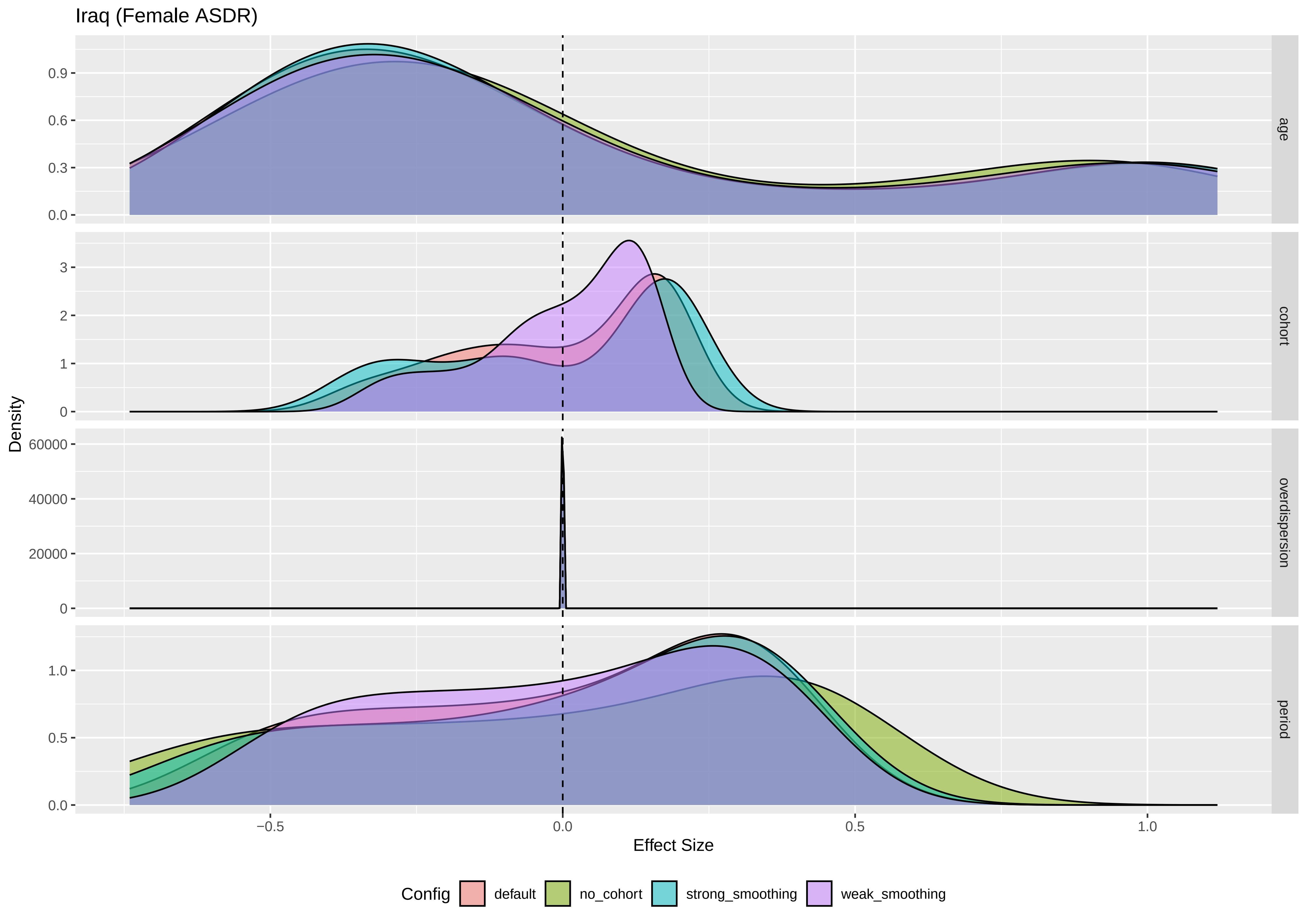

Iraq (Female ASDR)
Density
Effect Size
age
cohort
overdispersion
period
0.0
0.3
0.6
0.9
0
1
2
3
20000
40000
60000
0.5
1.0
−0.5
Config
default
no_cohort
strong_smoothing
weak_smoothing

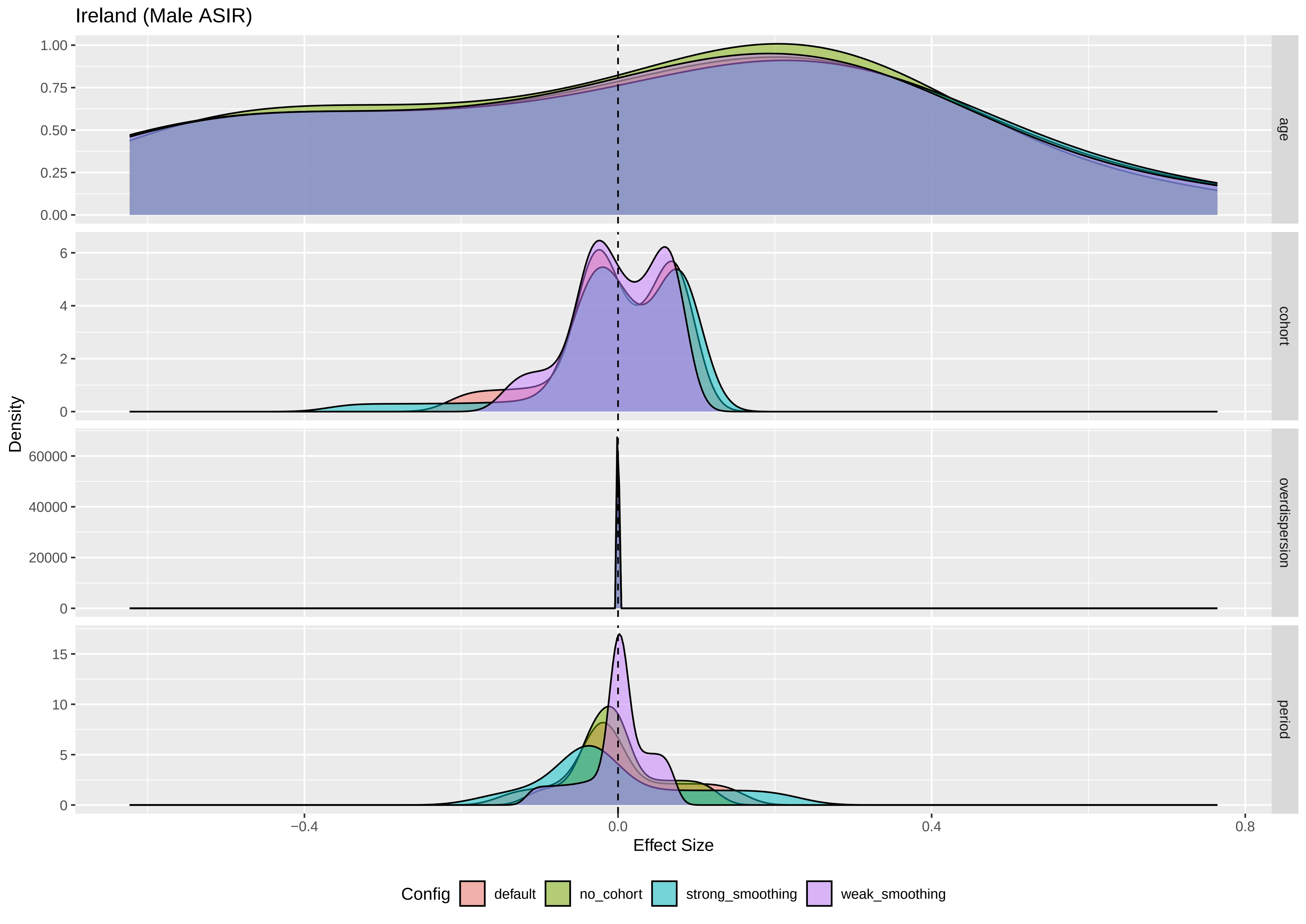

Ireland (Male ASIR)
age
cohort
overdispersion
period
Density
Effect Size
−0.4
0.0
0.4
0.8
0.00
0.25
0.50
0.75
1.00
0
2
4
6
0
20000
40000
60000
0
5
10
15
Config
default
no_cohort
strong_smoothing
weak_smoothing

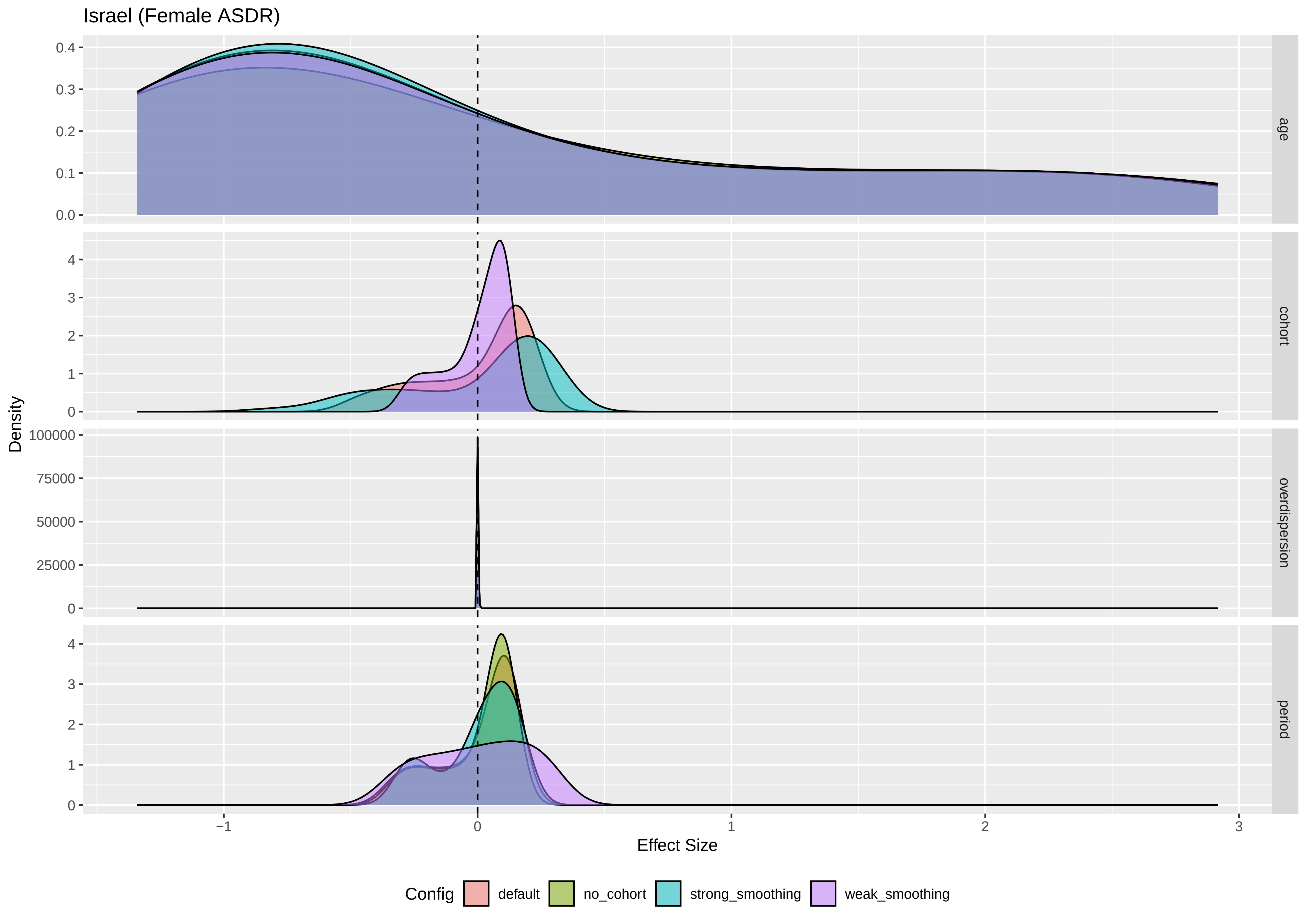

Israel (Female ASDR)
Density
Effect Size
age
cohort
overdispersion
period
0.0
0.1
0.2
0.3
0.4
0
1
2
3
4
0
25000
50000
75000
100000
−1
0
1
2
3
Config
default
no_cohort
strong_smoothing
weak_smoothing

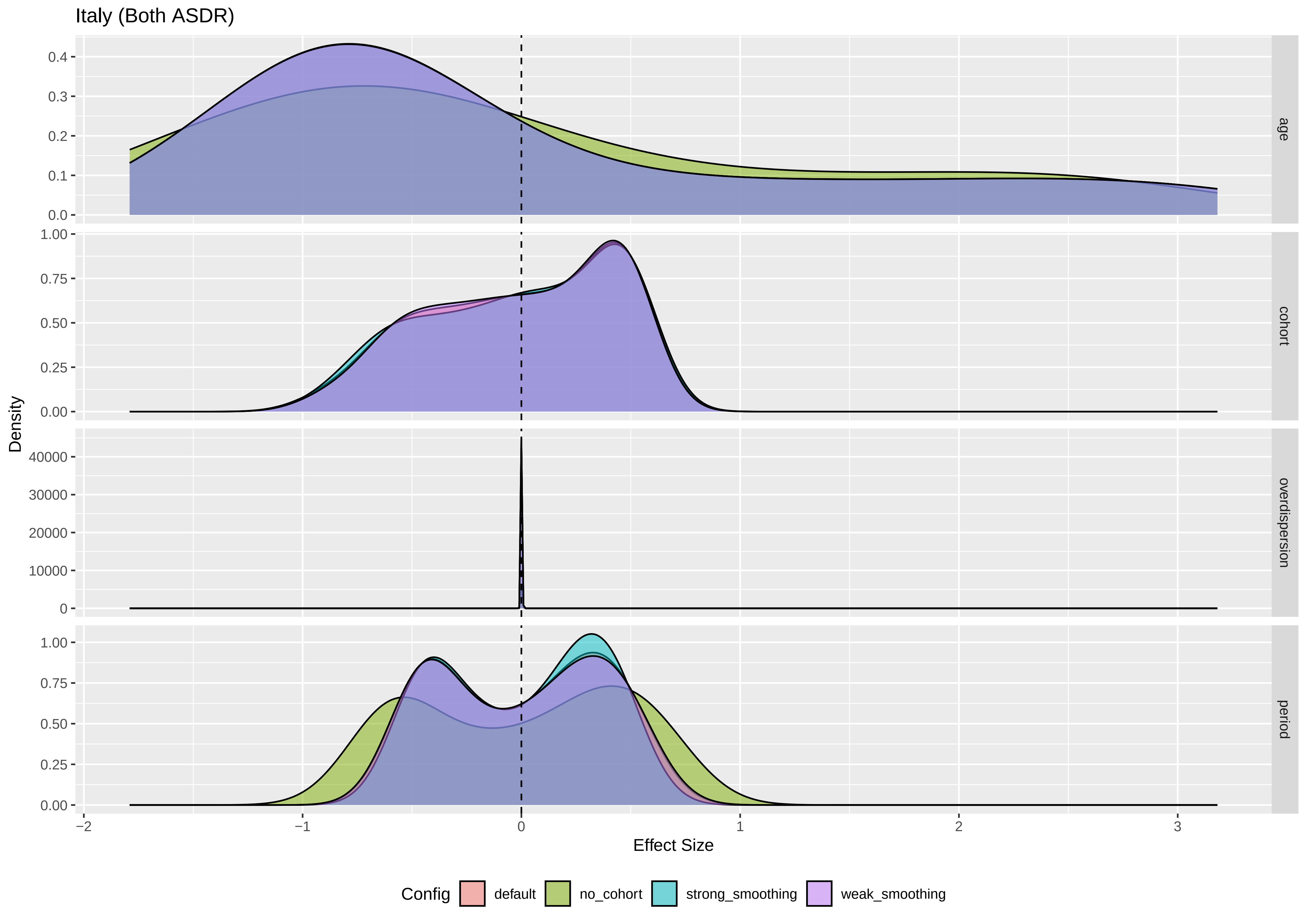

Italy (Both ASDR)
age
cohort
overdispersion
period
Density
Effect Size
0.0
0.1
0.2
0.3
0.4
0.00
0.25
0.50
0.75
1.00
0
10000
20000
30000
40000
−2
−1
0
1
2
3
Config
default
no_cohort
strong_smoothing
weak_smoothing

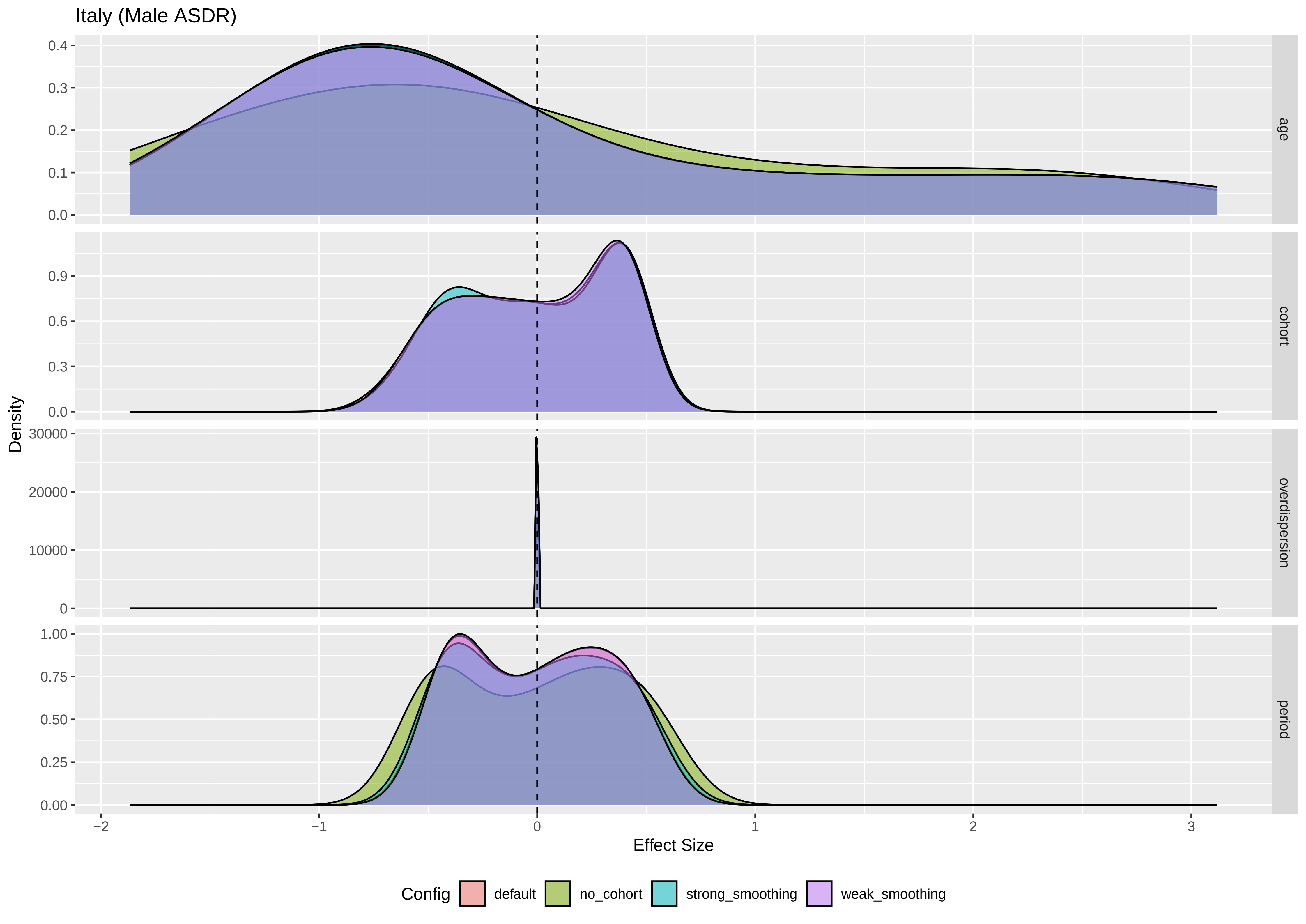

Italy (Male ASDR)
Density
Effect Size
age
cohort
overdispersion
period
0.0
0.1
0.2
0.3
0.4
0.6
0.9
0
10000
20000
30000
0.00
0.25
0.50
0.75
1.00
−2
−1
0
1
2
3
Config
default
no_cohort
strong_smoothing
weak_smoothing

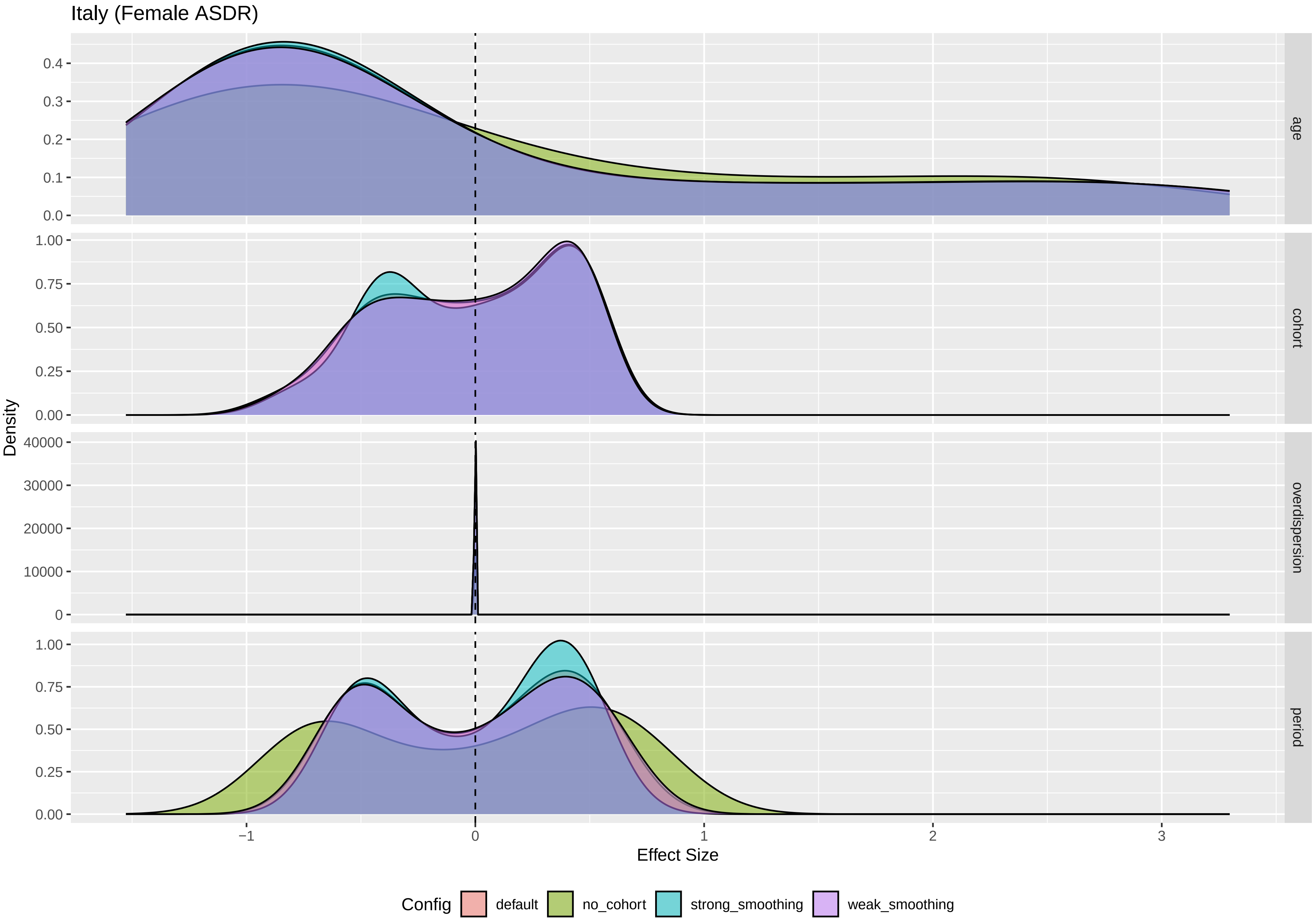

Italy (Female ASDR)
age
cohort
overdispersion
period
Density
Effect Size
0.4
0.3
0.2
0.1
0.0
1.00
0.75
0.50
0.25
0.00
40000
30000
20000
10000
0
−1
0
1
2
3
Config
default
no_cohort
strong_smoothing
weak_smoothing

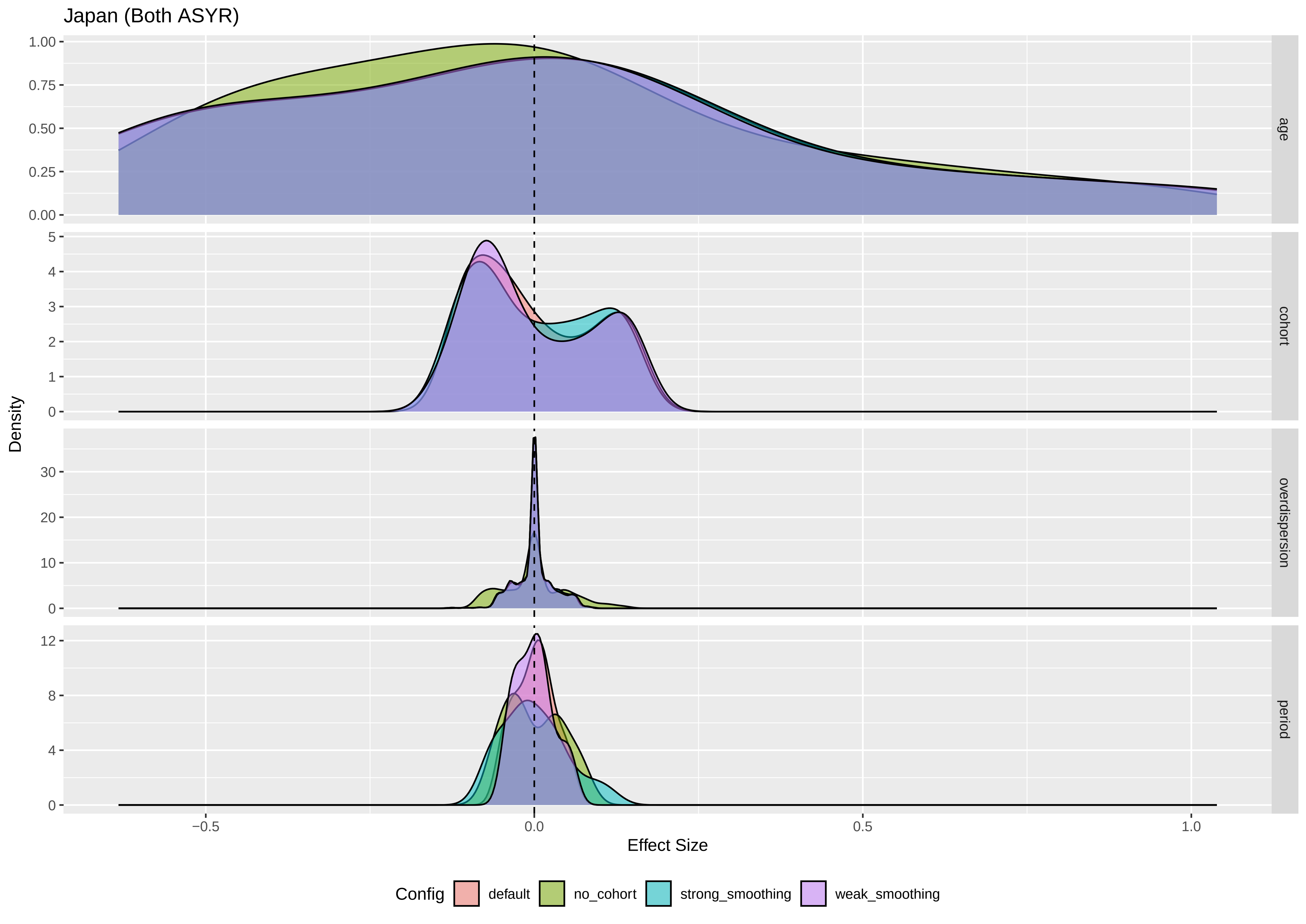

Japan (Both ASYR)
Density
Effect Size
age
cohort
overdispersion
period
Config
default
no_cohort
strong_smoothing
weak_smoothing

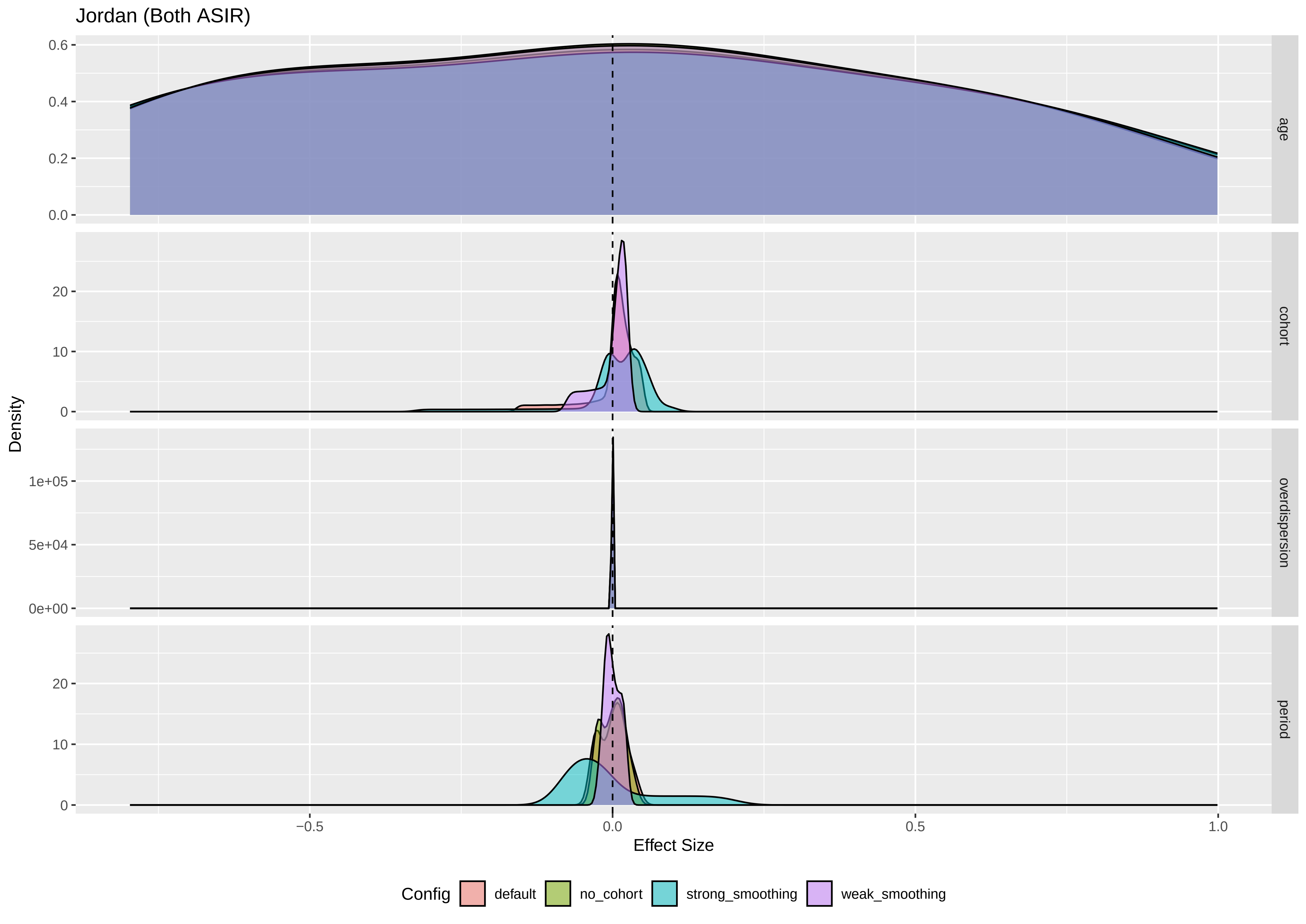

Jordan (Both ASIR)
Density
age
cohort
overdispersion
period
0.6
0.4
0.2
0.0
20
10
0
1e+05
5e+04
0e+00
20
10
0
−0.5
0.0
0.5
1.0
Effect Size
Config
default
no_cohort
strong_smoothing
weak_smoothing

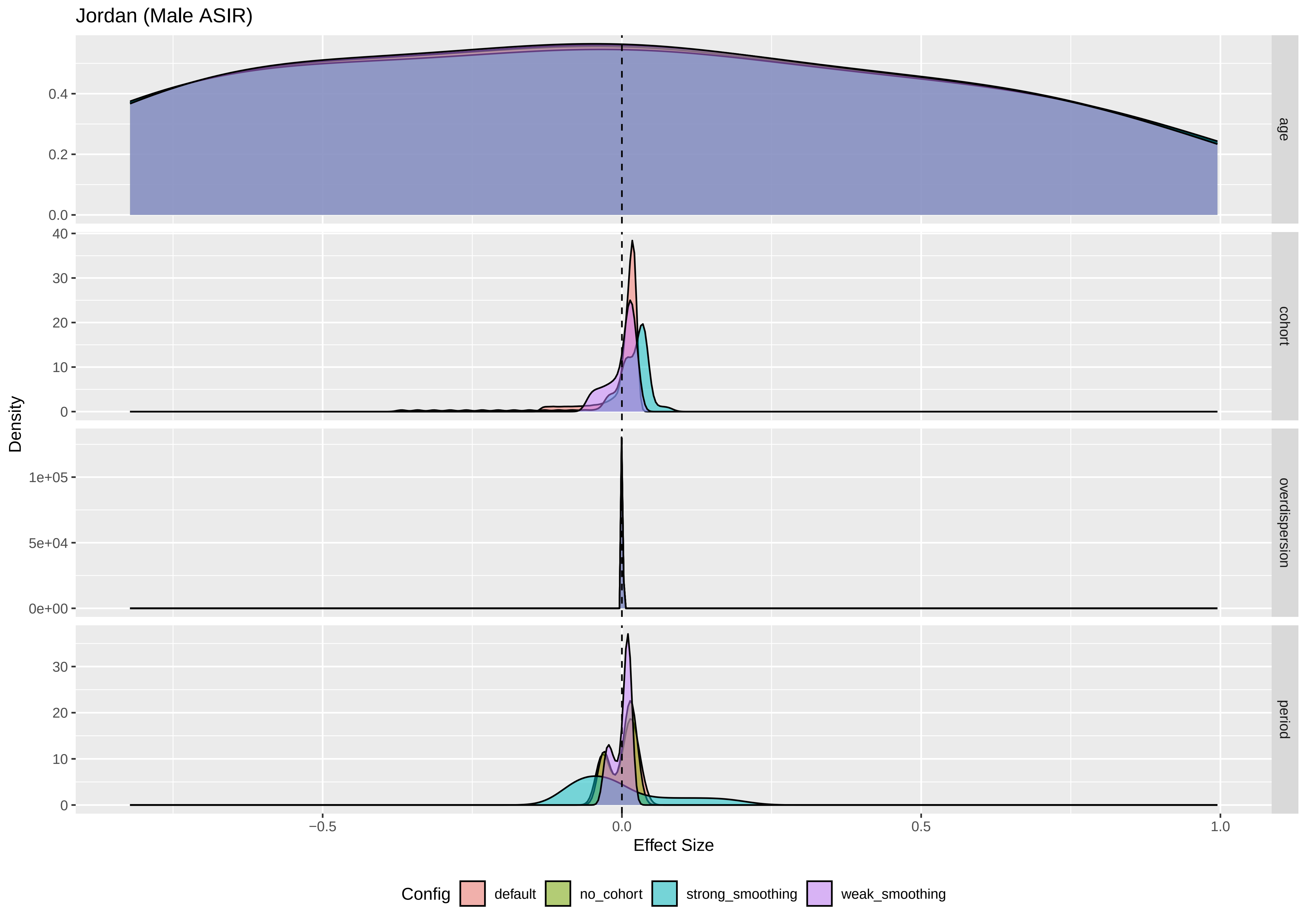

Jordan (Male ASIR)
Density
Effect Size
age
cohort
overdispersion
period
0.0
0.2
0.4
0
10
20
30
40
0e+00
5e+04
1e+05
−0.5
0.0
0.5
1.0
Config
default
no_cohort
strong_smoothing
weak_smoothing

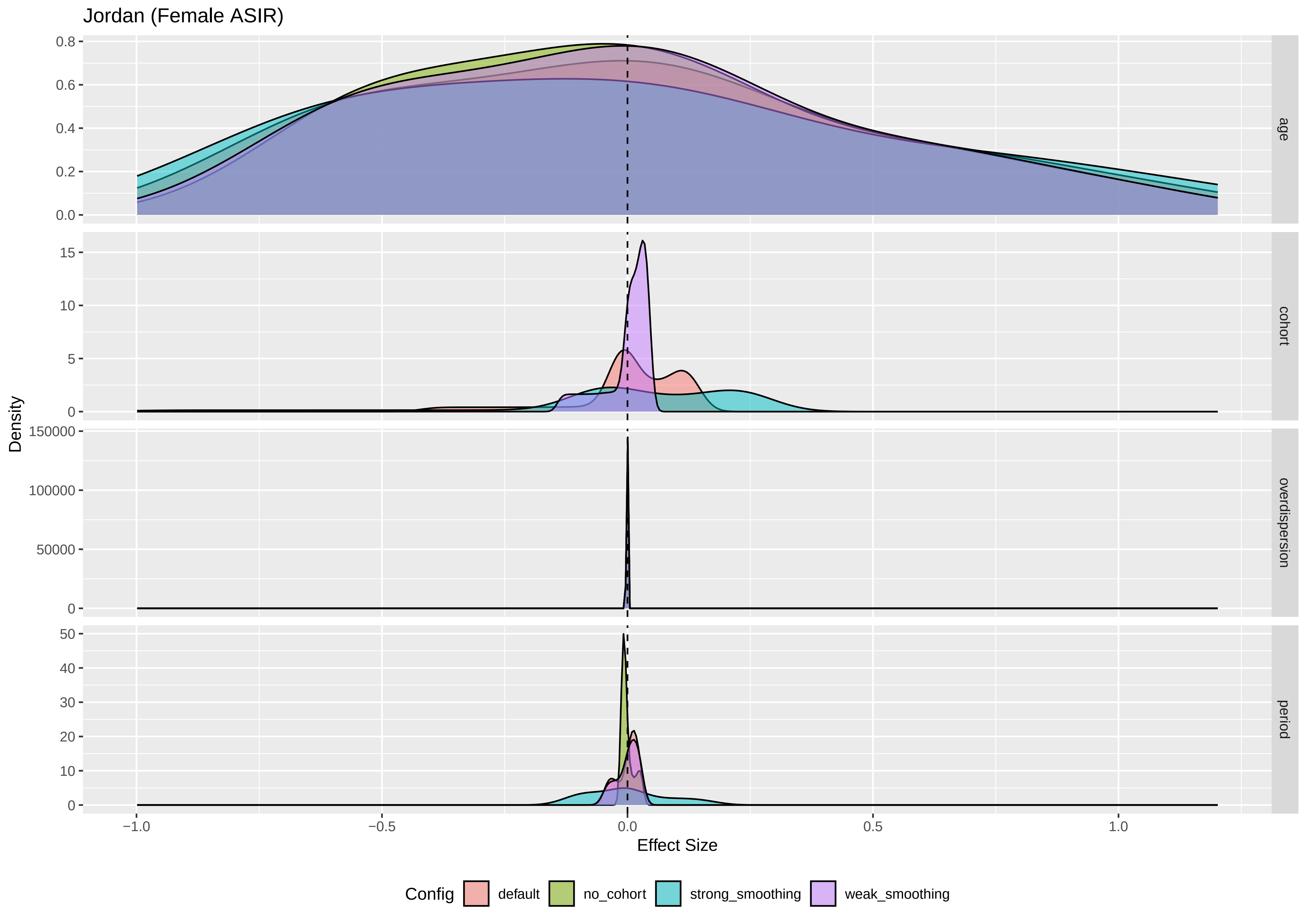

Jordan (Female ASIR)
Density
Effect Size
age
cohort
overdispersion
period
0.0
0.2
0.4
0.6
0.8
0
5
10
15
50000
100000
150000
20
30
40
50
−1.0
−0.5
0.0
0.5
1.0
Config
default
no_cohort
strong_smoothing
weak_smoothing

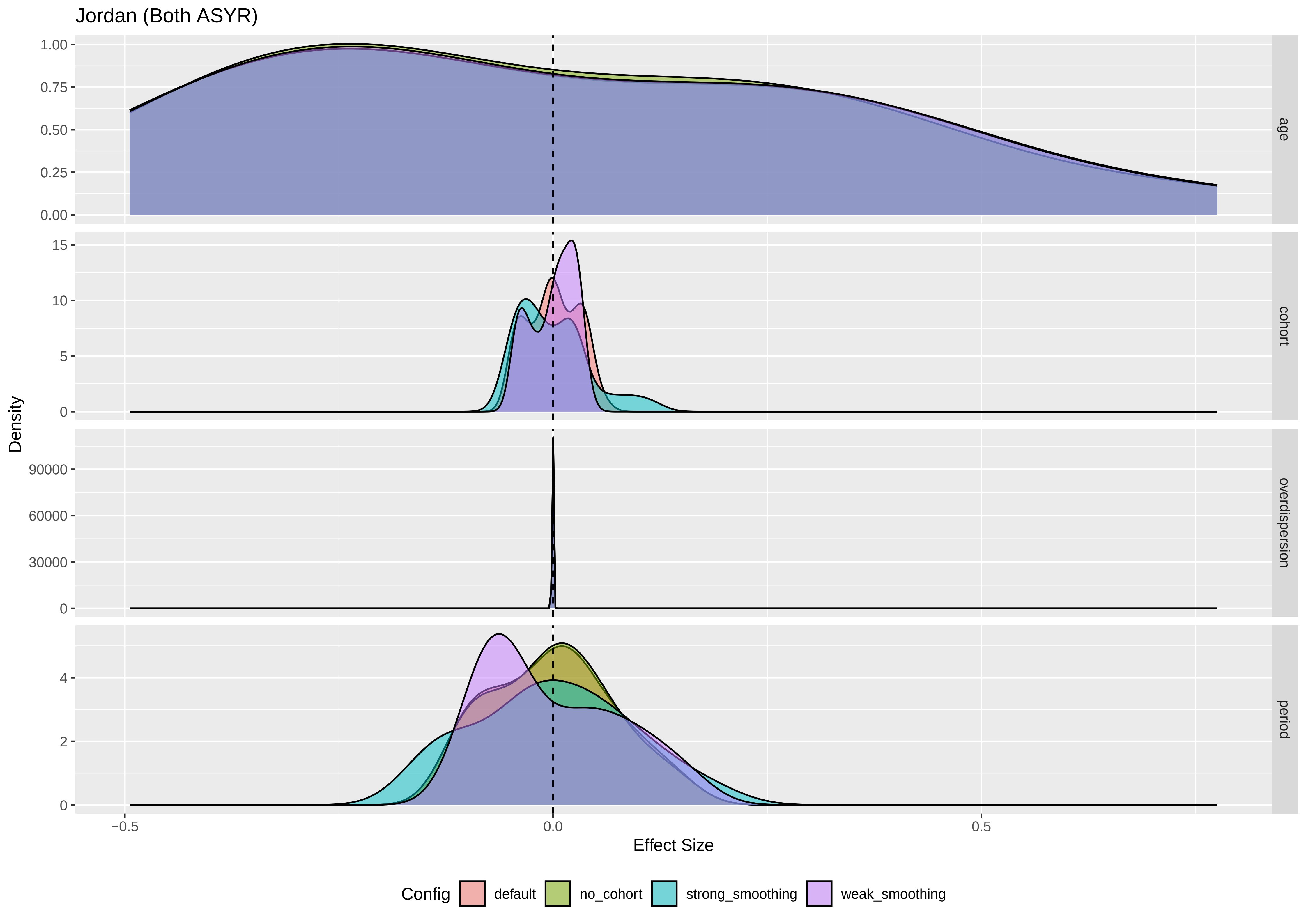

Jordan (Both ASYR)
1.00
0.75
0.50
0.25
0.00
age
15
10
5
0
cohort
Density
90000
60000
30000
0
overdispersion
4
2
0
period
−0.5
0.0
0.5
Effect Size
Config
default
no_cohort
strong_smoothing
weak_smoothing

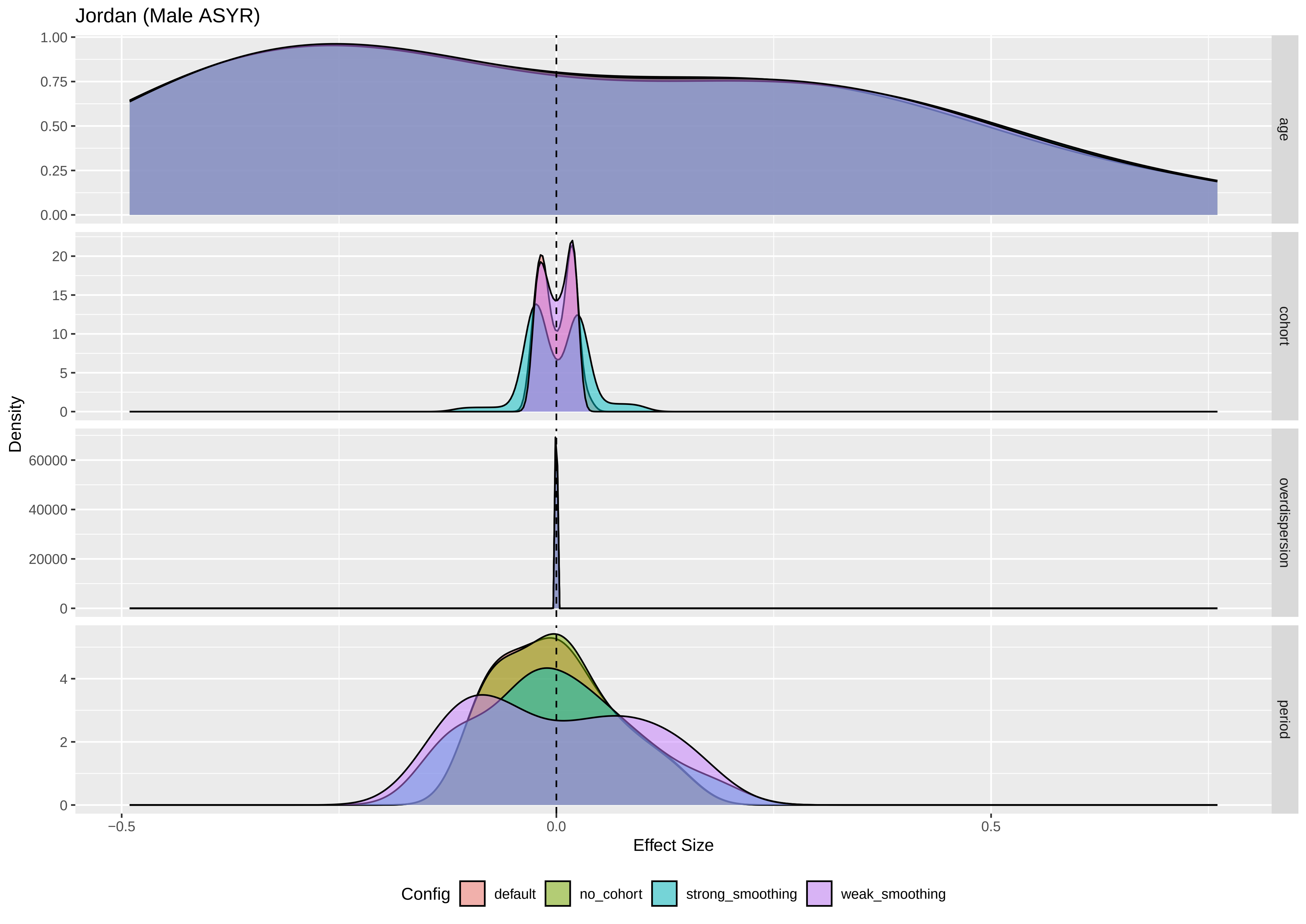

Jordan (Male ASYR)
Density
Effect Size
age
cohort
overdispersion
period
1.00
0.75
0.50
0.25
0.00
20
15
10
5
0
60000
40000
20000
0
4
2
0
−0.5
0.0
0.5
Config
default
no_cohort
strong_smoothing
weak_smoothing

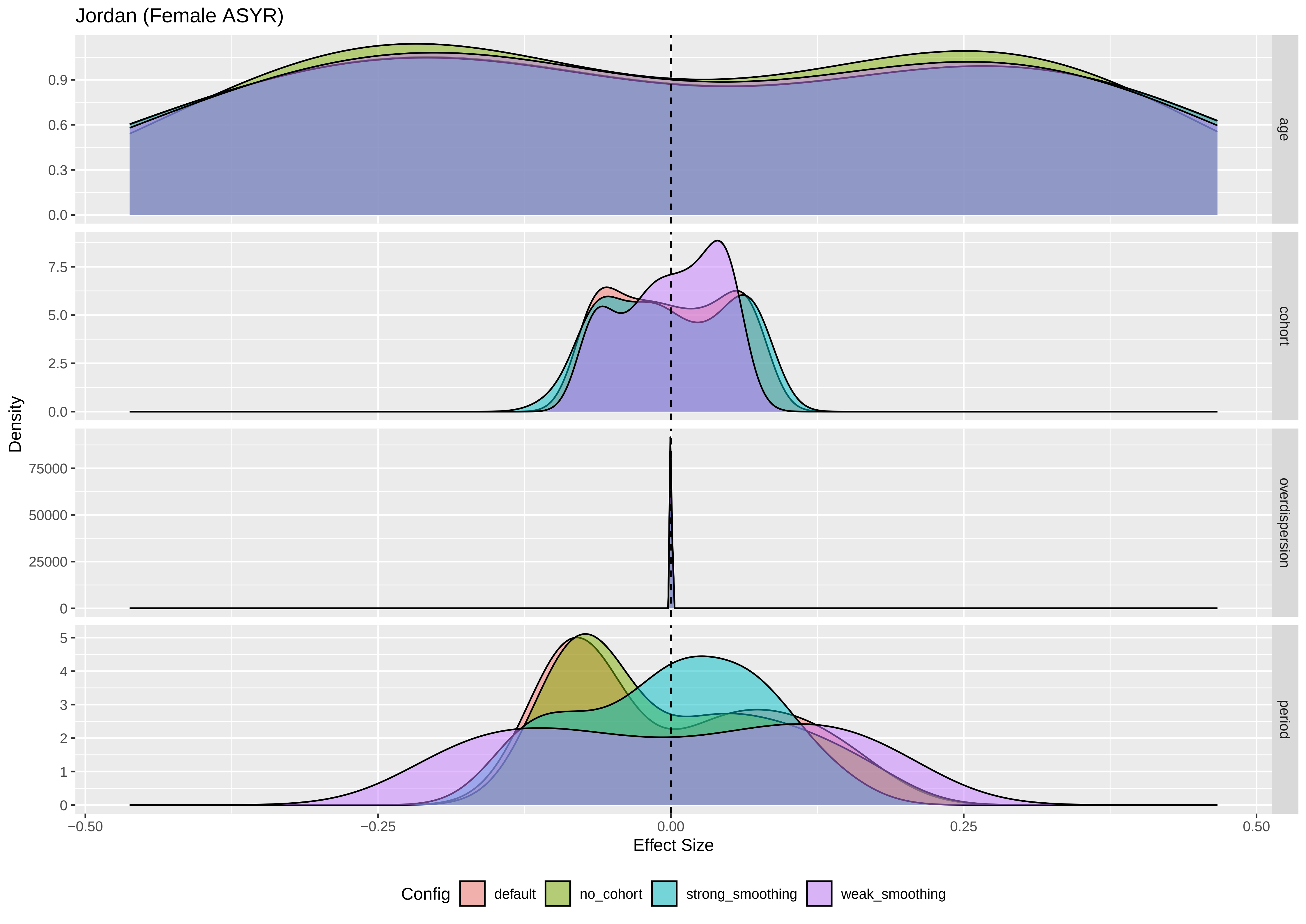

Jordan (Female ASYR)
Density
age
cohort
overdispersion
period
0.0
0.3
0.6
0.9
0.0
2.5
5.0
7.5
0
25000
50000
75000
0
1
2
3
4
5
−0.50
−0.25
0.00
0.25
0.50
Effect Size
Config
default
no_cohort
strong_smoothing
weak_smoothing

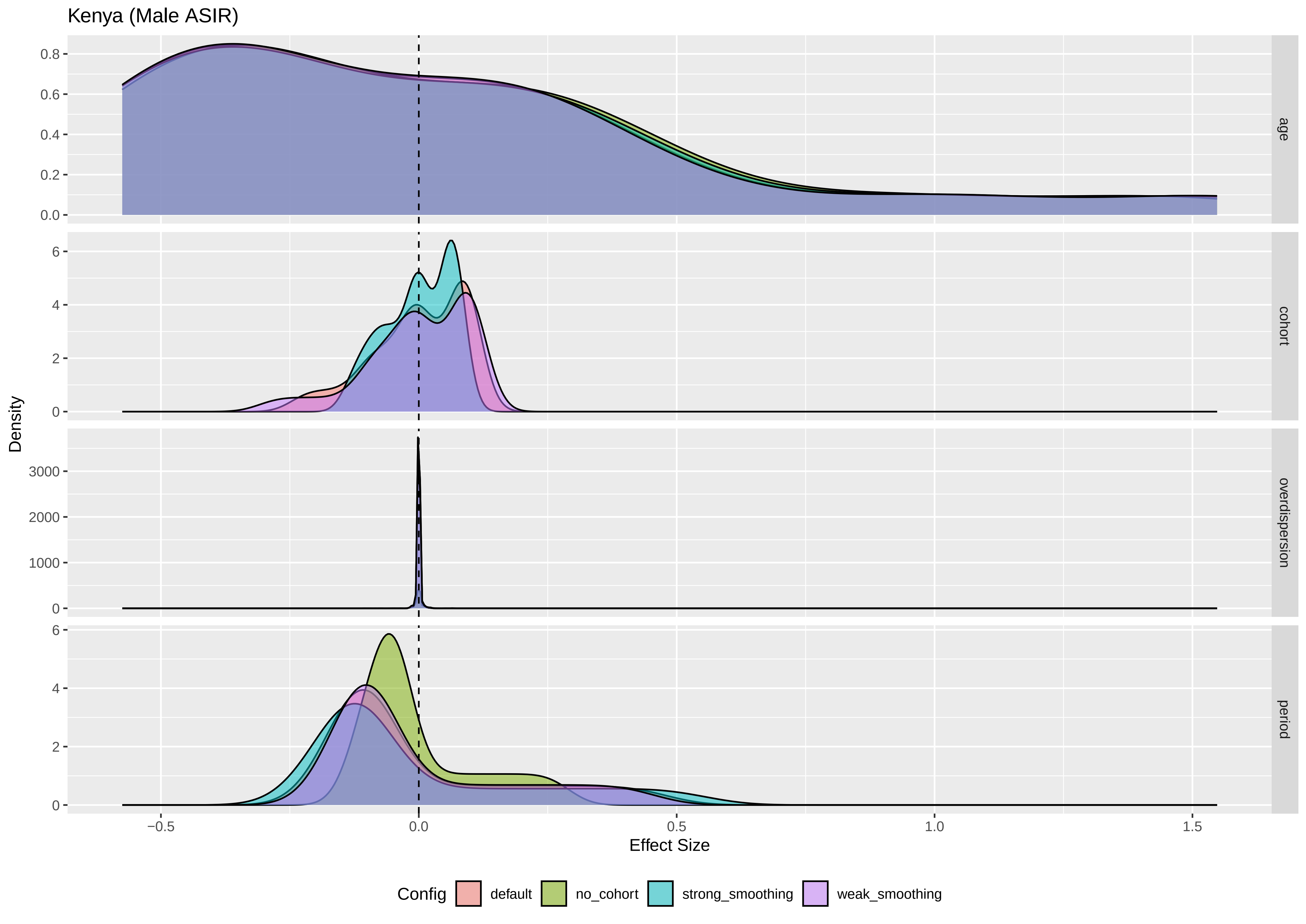
Kenya (Male ASIR)
Density
Effect Size
age
cohort
overdispersion
period
0.0
0.2
0.4
0.6
0.8
0
2
4
6
0
1000
2000
3000
0
2
4
6
−0.5
0.0
0.5
1.0
1.5
Config
default
no_cohort
strong_smoothing
weak_smoothing

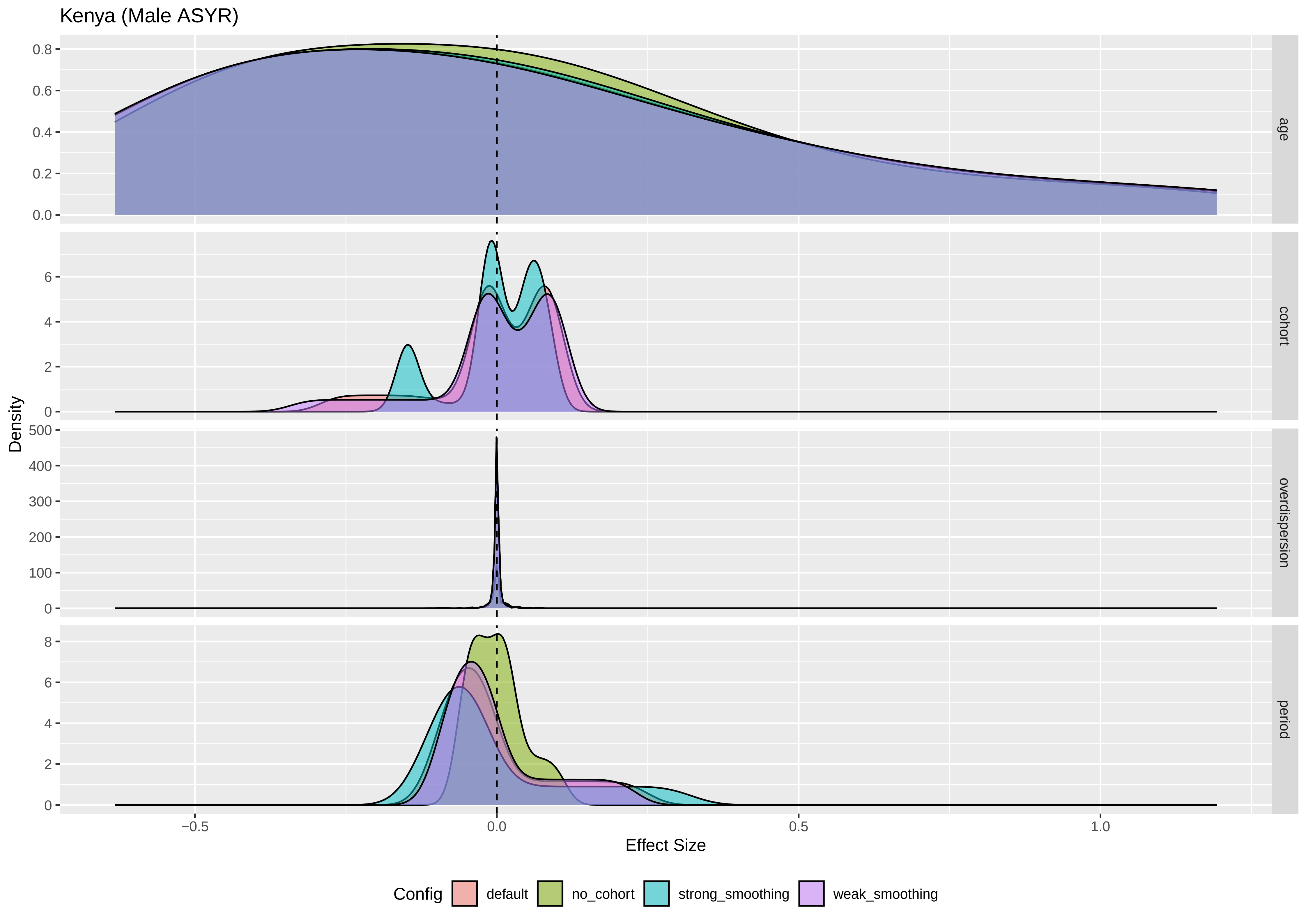

Kenya (Male ASYR)
age
cohort
overdispersion
period
Density
Effect Size
−0.5
0.0
0.5
1.0
0.0
0.2
0.4
0.6
0.8
0
2
4
6
100
200
300
400
500
8
Config
default
no_cohort
strong_smoothing
weak_smoothing

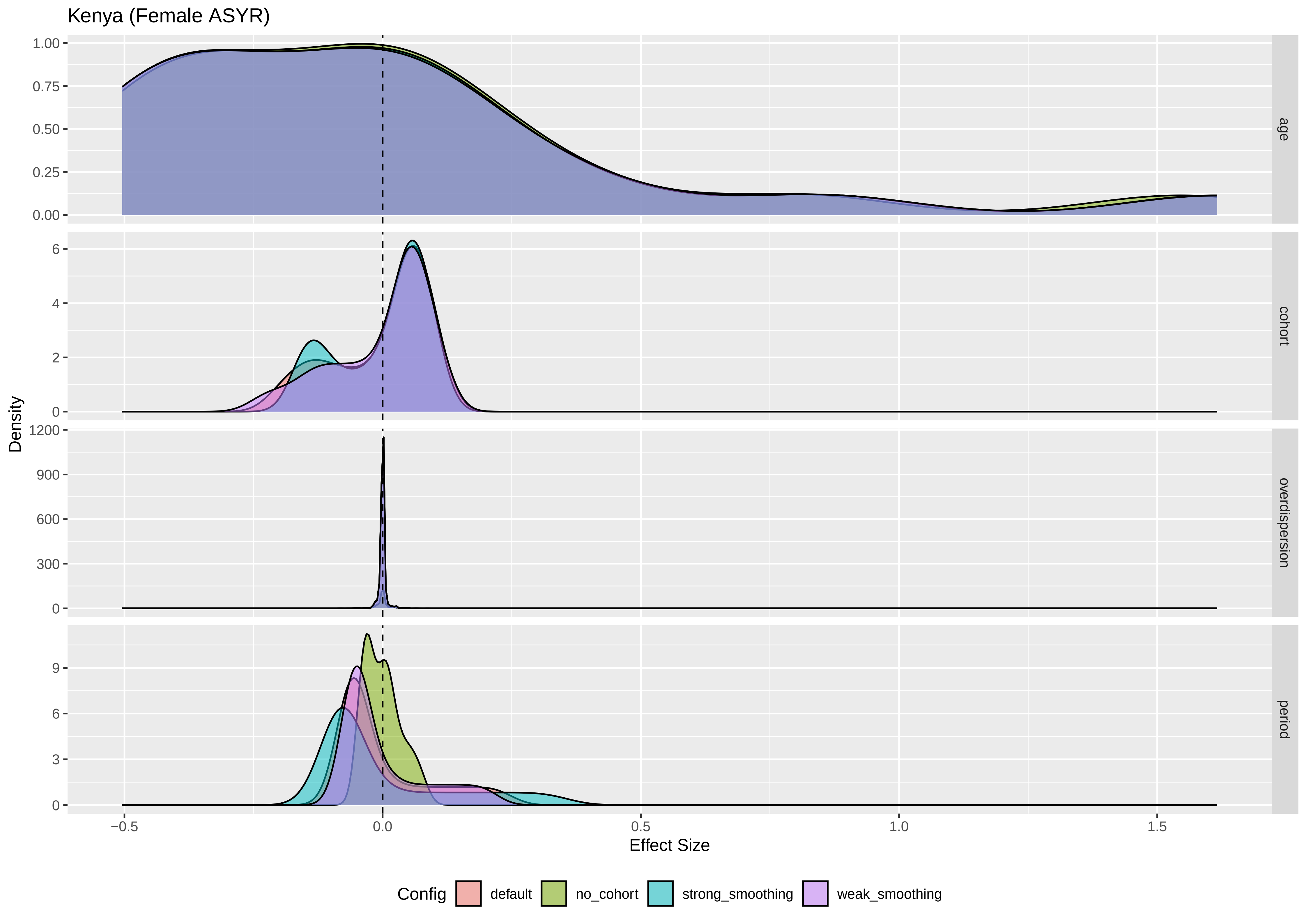
Kenya (Female ASYR)
Density
age
cohort
overdispersion
period
1.00
0.75
0.50
0.25
0.00
6
4
2
0
1200
900
600
300
0
9
6
3
0
−0.5
0.0
0.5
1.0
1.5
Effect Size
Config
default
no_cohort
strong_smoothing
weak_smoothing

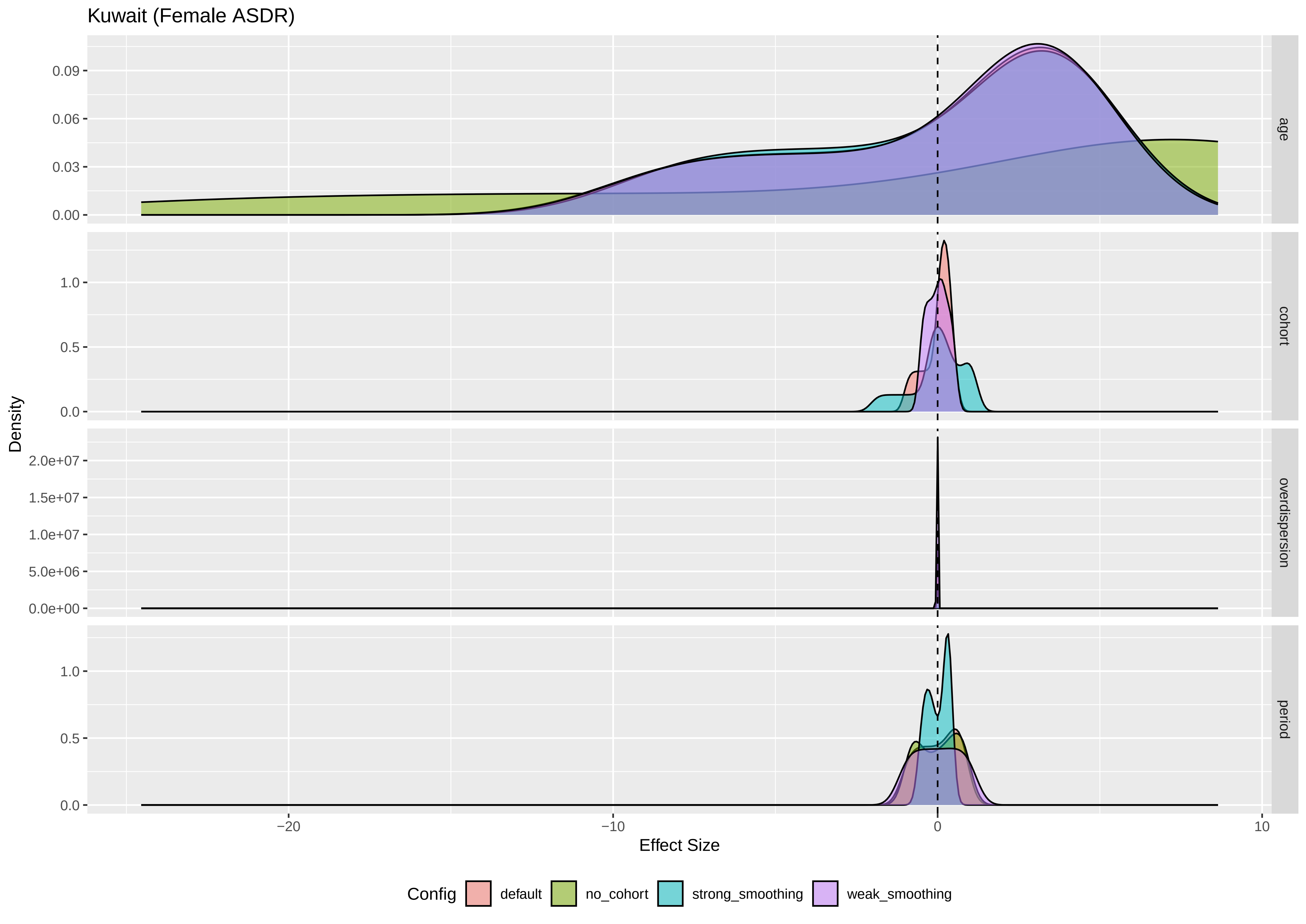

Kuwait (Female ASDR)
Density
0.00
0.03
0.06
0.09
0.0
0.5
1.0
0.0e+00
5.0e+06
1.0e+07
1.5e+07
2.0e+07
0.0
0.5
1.0
age
cohort
overdispersion
period
−20
−10
0
10
Effect Size
Config
default
no_cohort
strong_smoothing
weak_smoothing

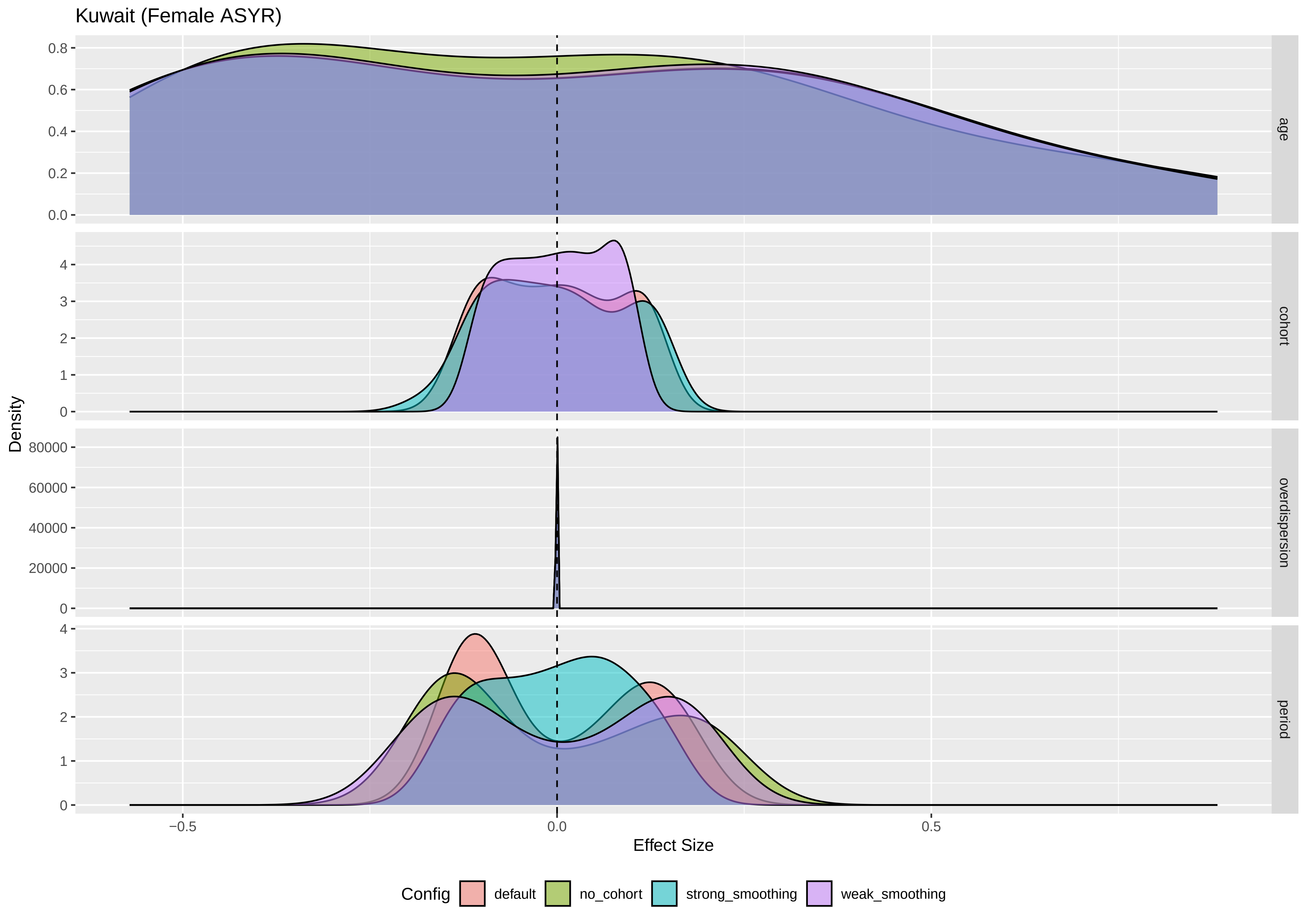

Kuwait (Female ASYR)
0.8
0.6
0.4
0.2
0.0
age
4
3
2
1
0
cohort
80000
60000
40000
20000
0
overdispersion
4
3
2
1
0
period
Density
−0.5
0.0
0.5
Effect Size
Config
default
no_cohort
strong_smoothing
weak_smoothing

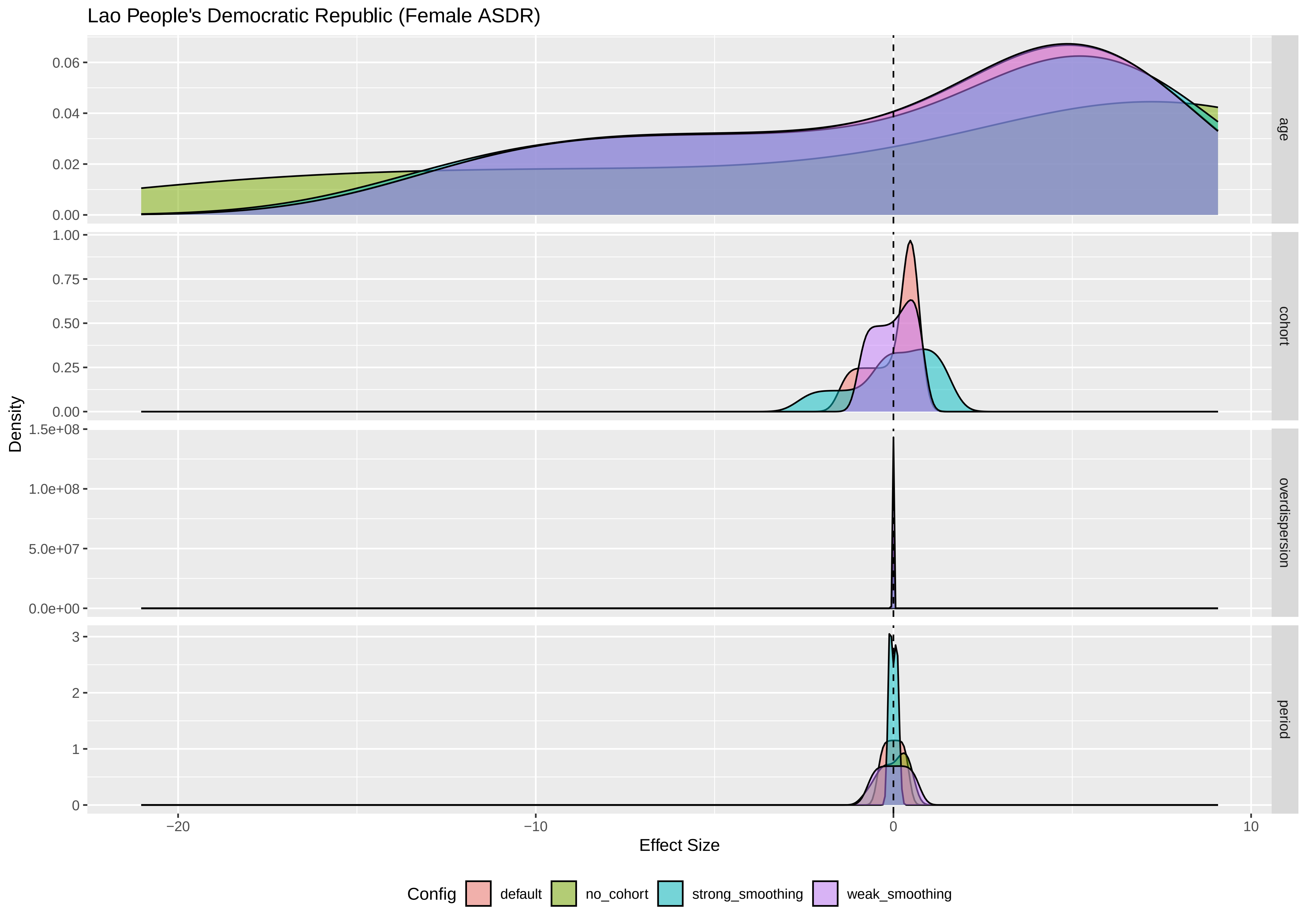

Lao People's Democratic Republic (Female ASDR)
age
cohort
overdispersion
period
Density
Effect Size
0.06
0.04
0.02
0.00
1.00
0.75
0.50
0.25
0.00
1.5e+08
1.0e+08
5.0e+07
0.0e+00
3
2
1
0
−20
−10
0
10
Config
default
no_cohort
strong_smoothing
weak_smoothing

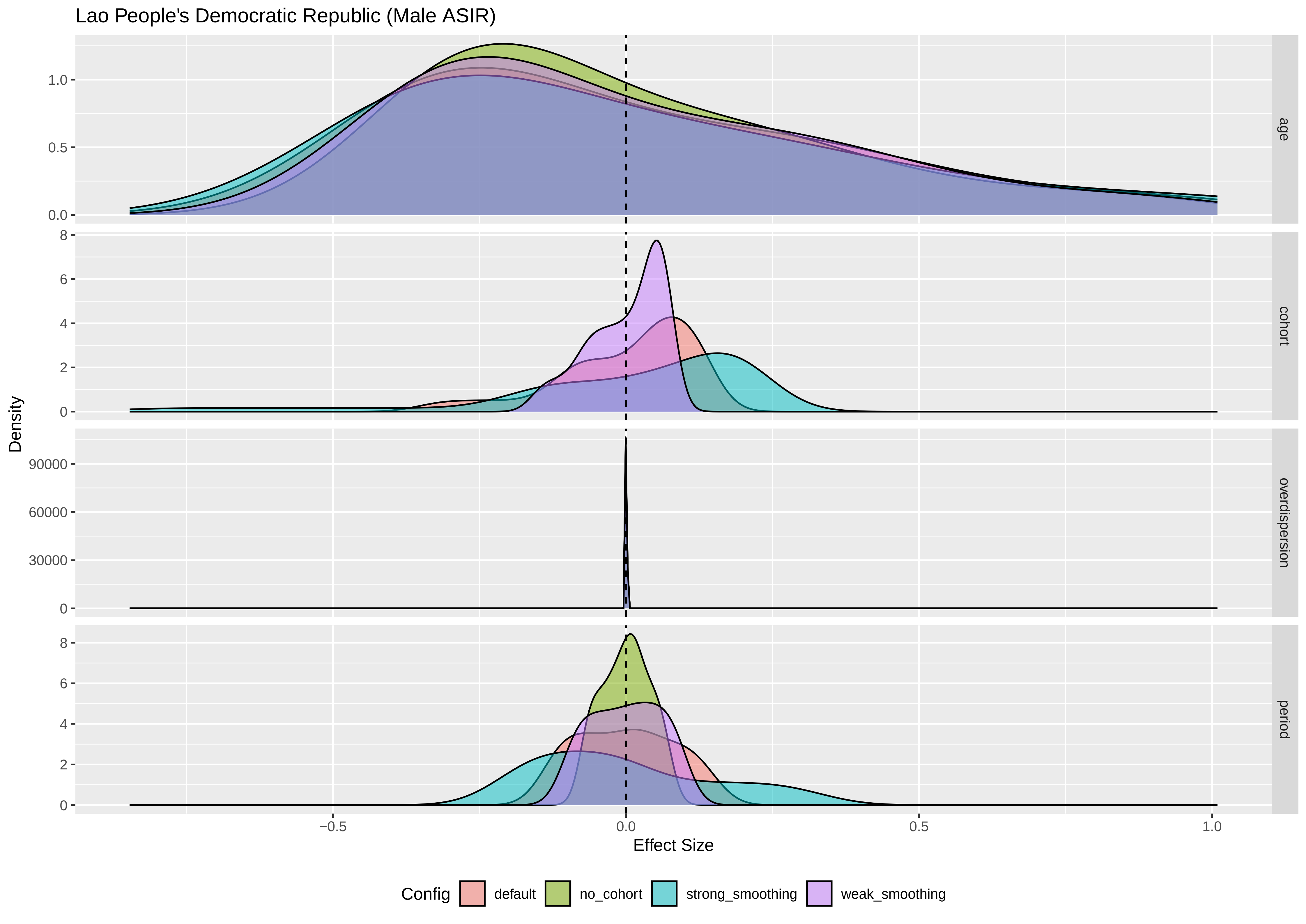

Lao People's Democratic Republic (Male ASIR)
1.0
0.5
0.0
age
8
6
4
2
0
cohort
Density
90000
60000
30000
0
overdispersion
8
6
4
2
0
period
−0.5
0.0
0.5
1.0
Effect Size
Config
default
no_cohort
strong_smoothing
weak_smoothing

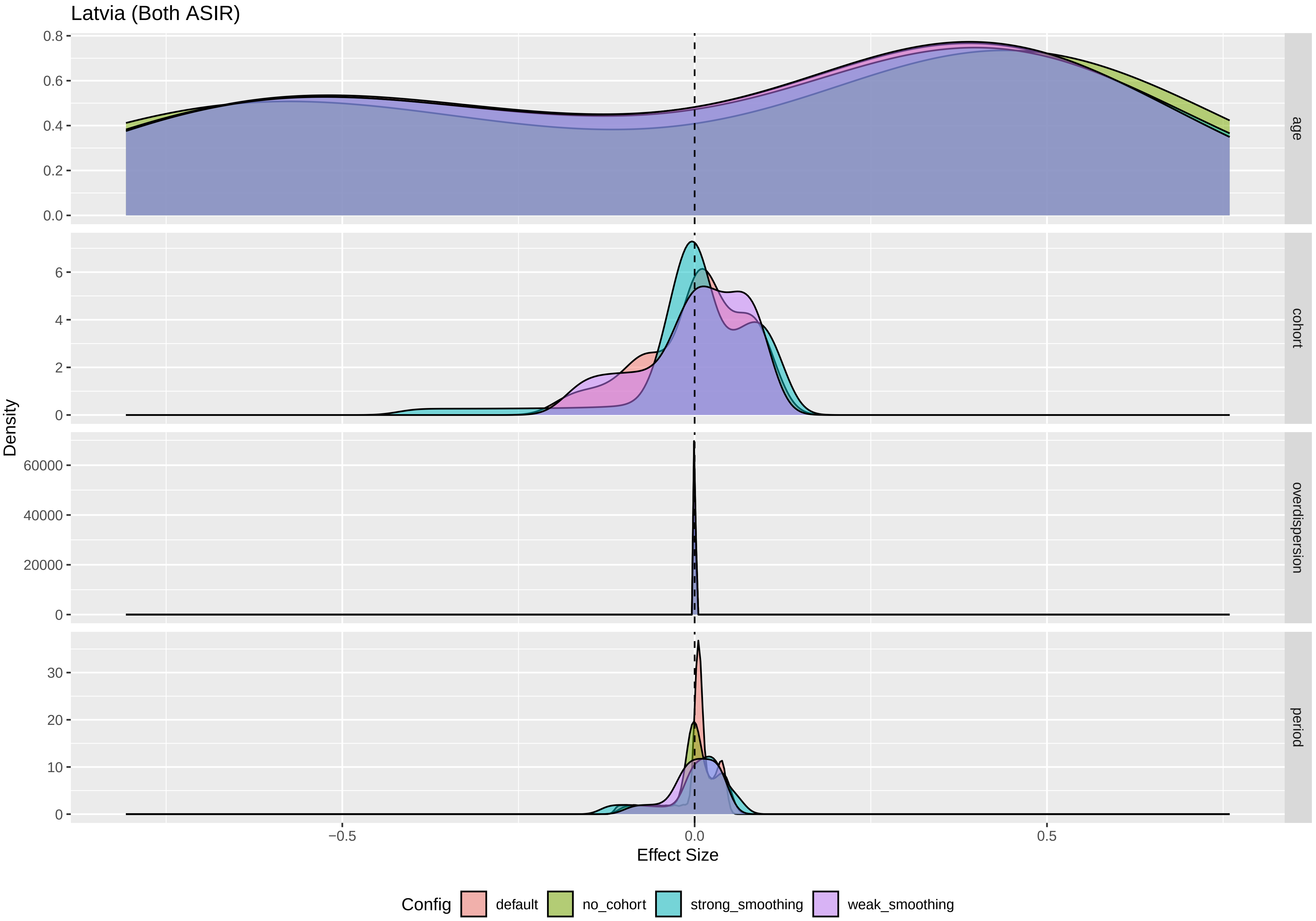

Latvia (Both ASIR)
Density
Effect Size
age
cohort
overdispersion
period
0.0
0.2
0.4
0.6
0.8
0
2
4
6
0
20000
40000
60000
0
10
20
30
−0.5
0.0
0.5
Config
default
no_cohort
strong_smoothing
weak_smoothing

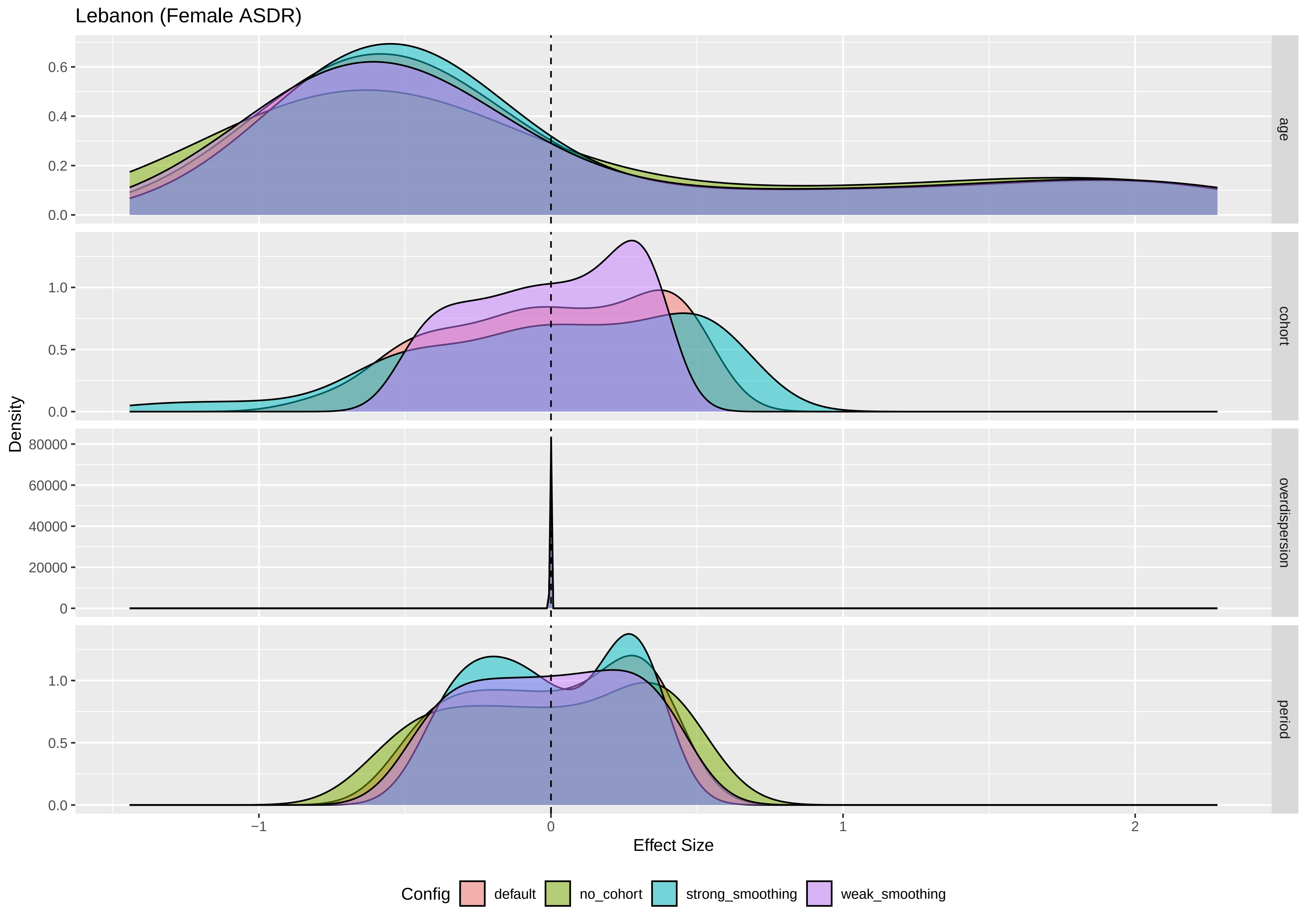

Lebanon (Female ASDR)
Density
Effect Size
age
cohort
overdispersion
period
0.0
0.2
0.4
0.6
0.5
1.0
0
20000
40000
60000
80000
−1
0
1
2
Config
default
no_cohort
strong_smoothing
weak_smoothing

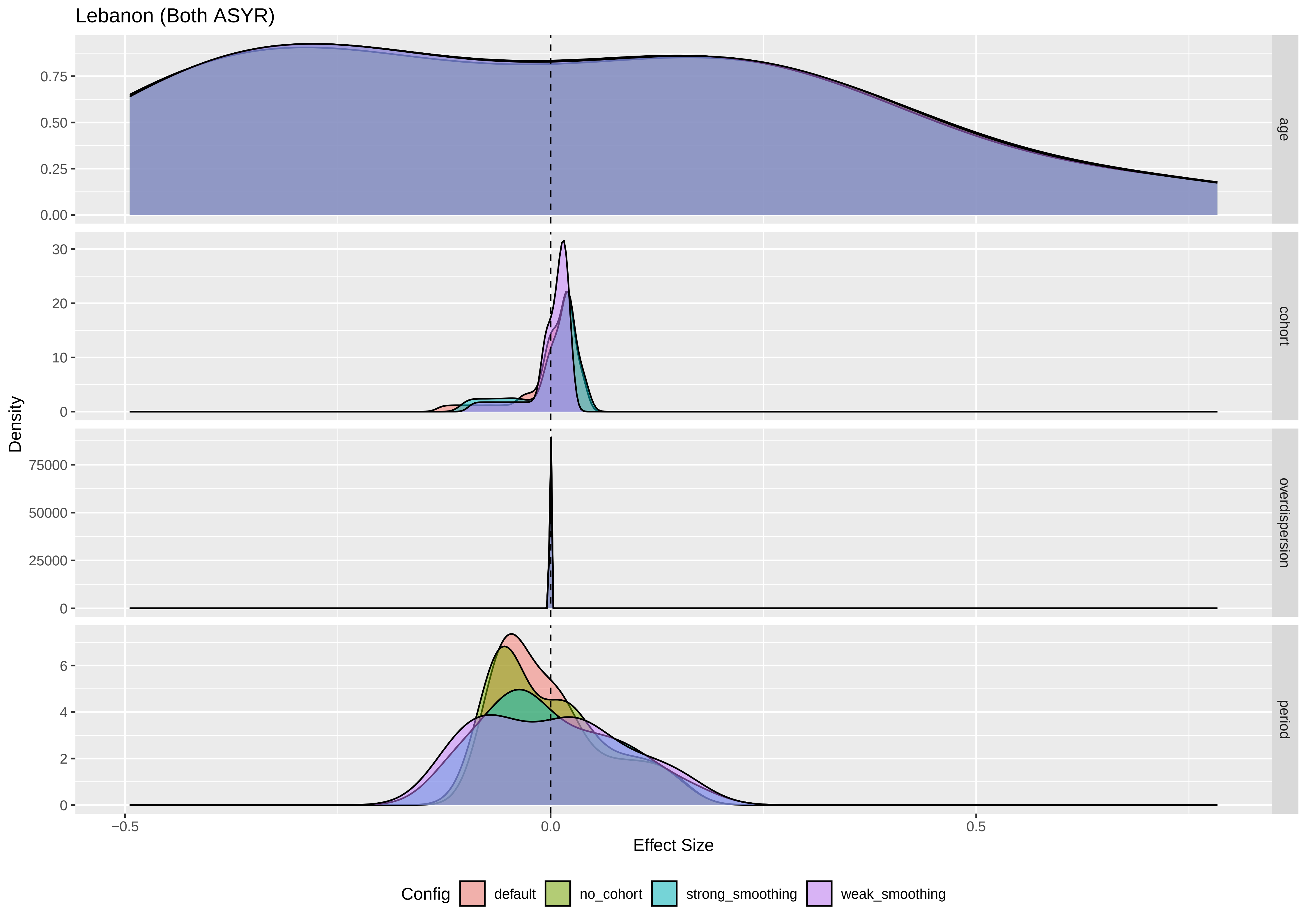

Lebanon (Both ASYR)
Density
Effect Size
age
cohort
overdispersion
period
0.00
0.25
0.50
0.75
0
10
20
30
0
25000
50000
75000
0
2
4
6
−0.5
0.0
0.5
Config
default
no_cohort
strong_smoothing
weak_smoothing

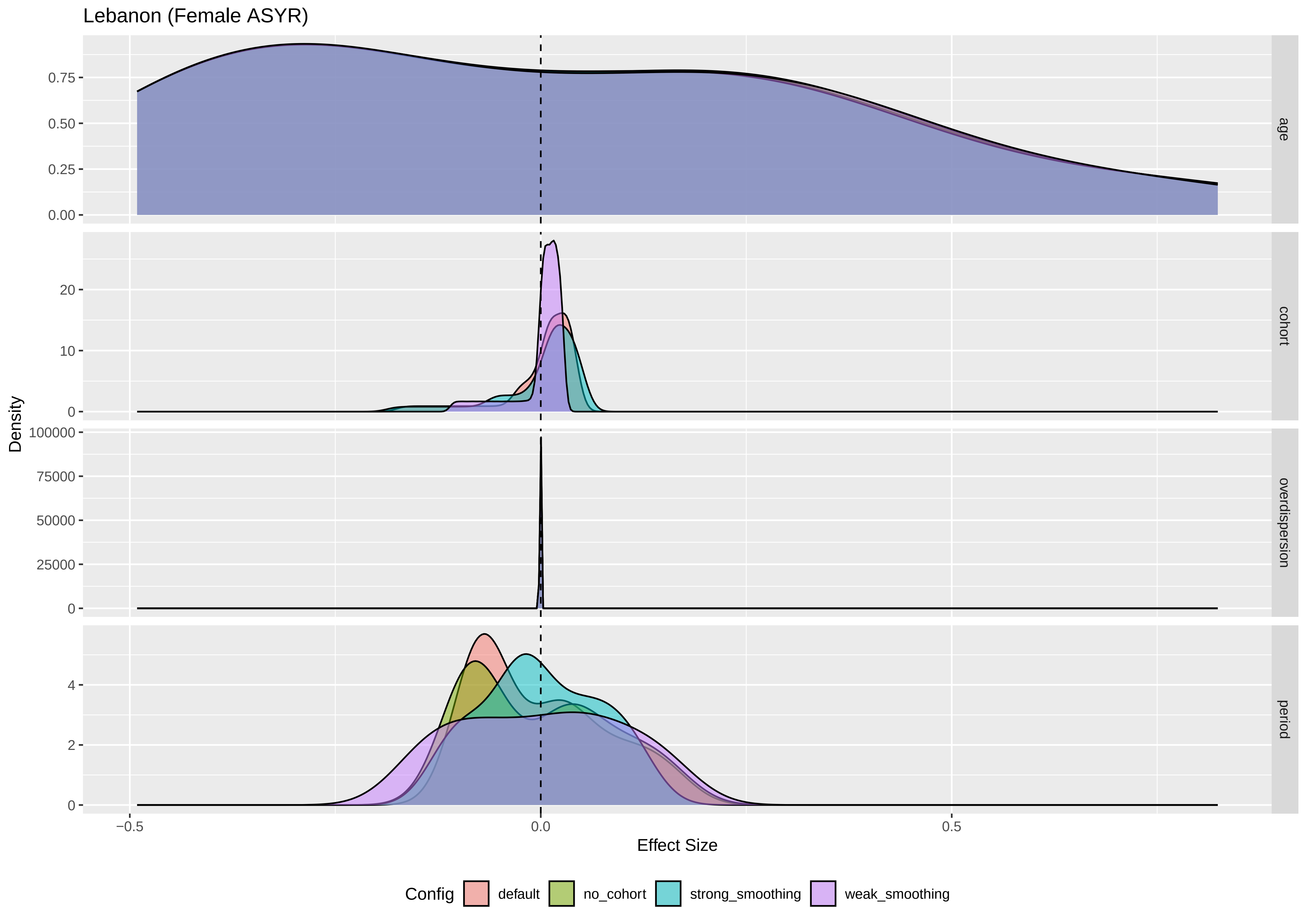
Lebanon (Female ASYR)
age
cohort
overdispersion
period
Density
0.00
0.25
0.50
0.75
0
10
20
0
25000
50000
75000
100000
0
2
4
−0.5
0.0
0.5
Effect Size
Config
default
no_cohort
strong_smoothing
weak_smoothing

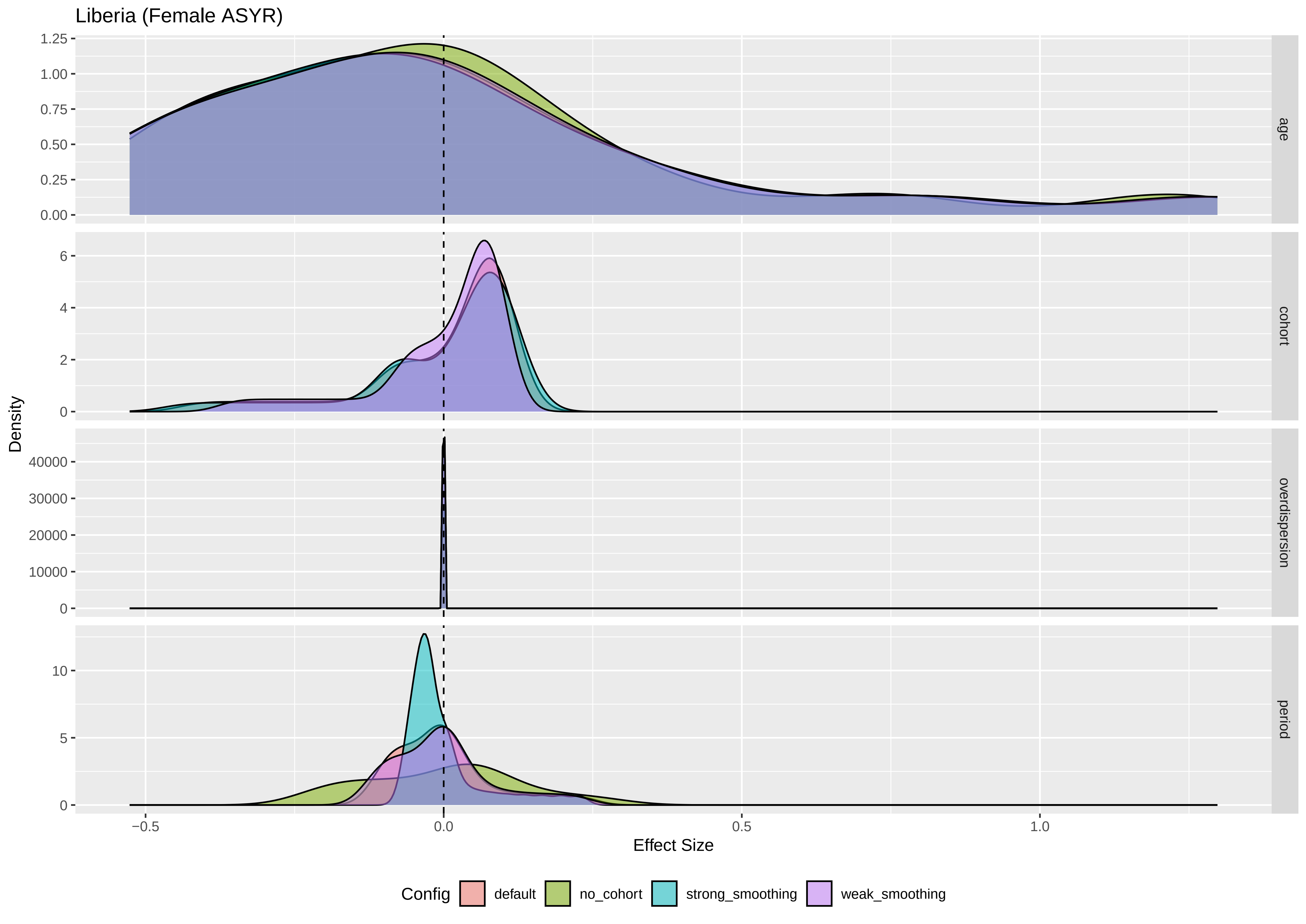

Liberia (Female ASYR)
1.25
1.00
0.75
0.50
0.25
0.00
age
6
4
2
0
cohort
40000
30000
20000
10000
0
overdispersion
10
5
0
period
Density
−0.5
0.0
0.5
1.0
Effect Size
Config
default
no_cohort
strong_smoothing
weak_smoothing

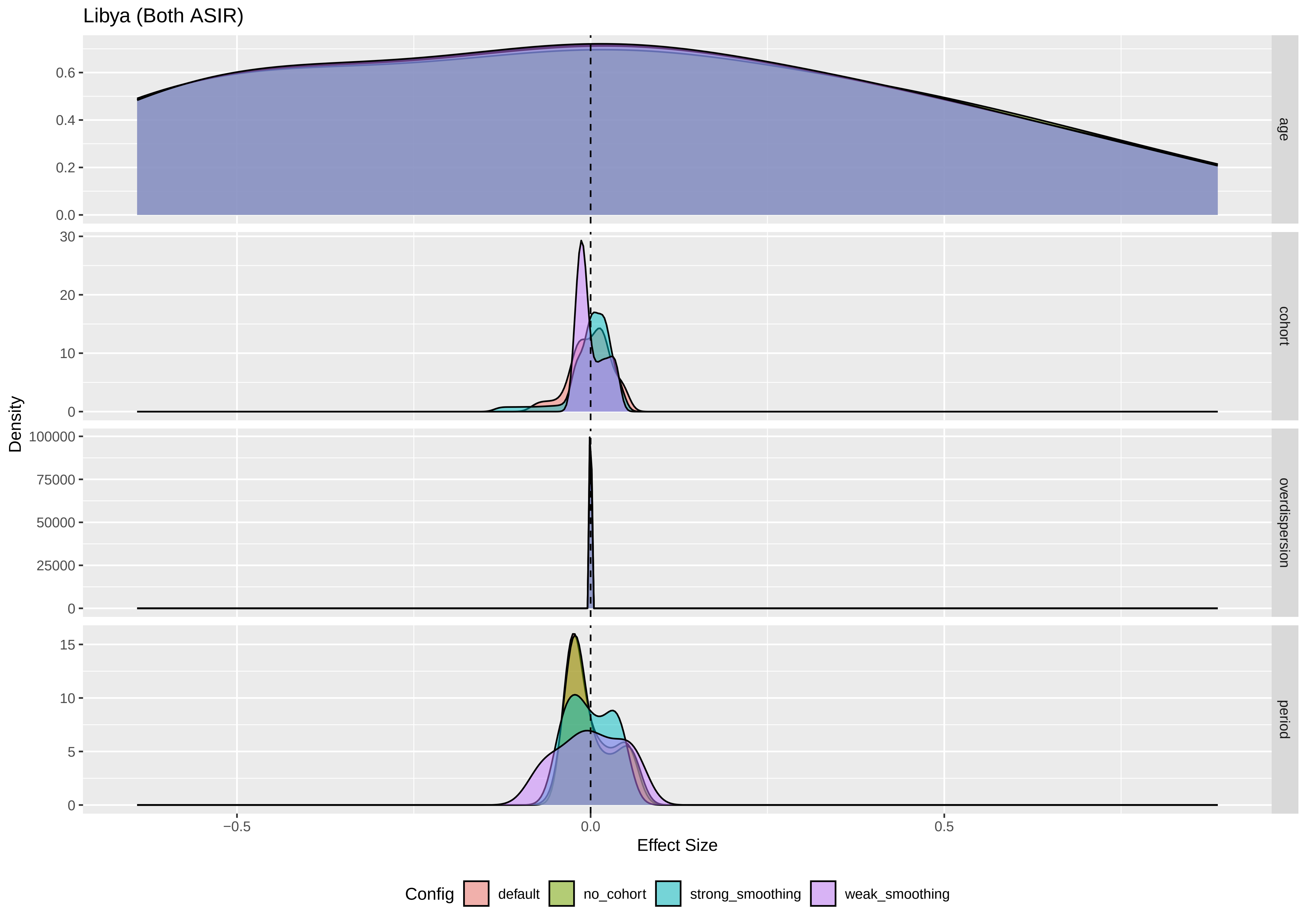

Libya (Both ASIR)
Density
Effect Size
age
cohort
overdispersion
period
0.0
0.2
0.4
0.6
0
10
20
30
0
25000
50000
75000
100000
0
5
10
15
−0.5
0.0
0.5
Config
default
no_cohort
strong_smoothing
weak_smoothing

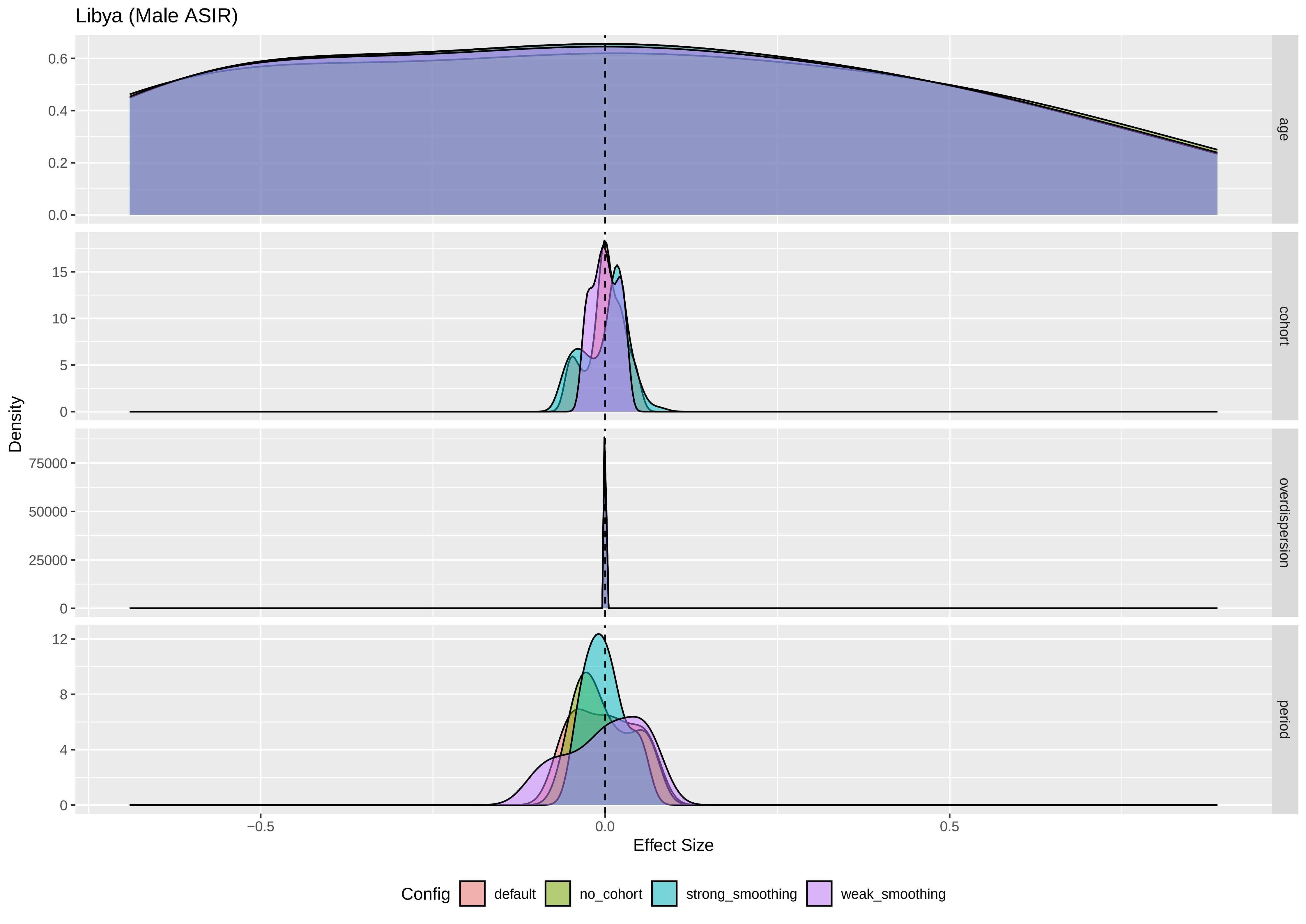

Libya (Male ASIR)
0.6
0.4
0.2
0.0
age
15
10
5
0
cohort
75000
50000
25000
0
overdispersion
12
8
4
0
period
Density
−0.5
0.0
0.5
Effect Size
Config
default
no_cohort
strong_smoothing
weak_smoothing

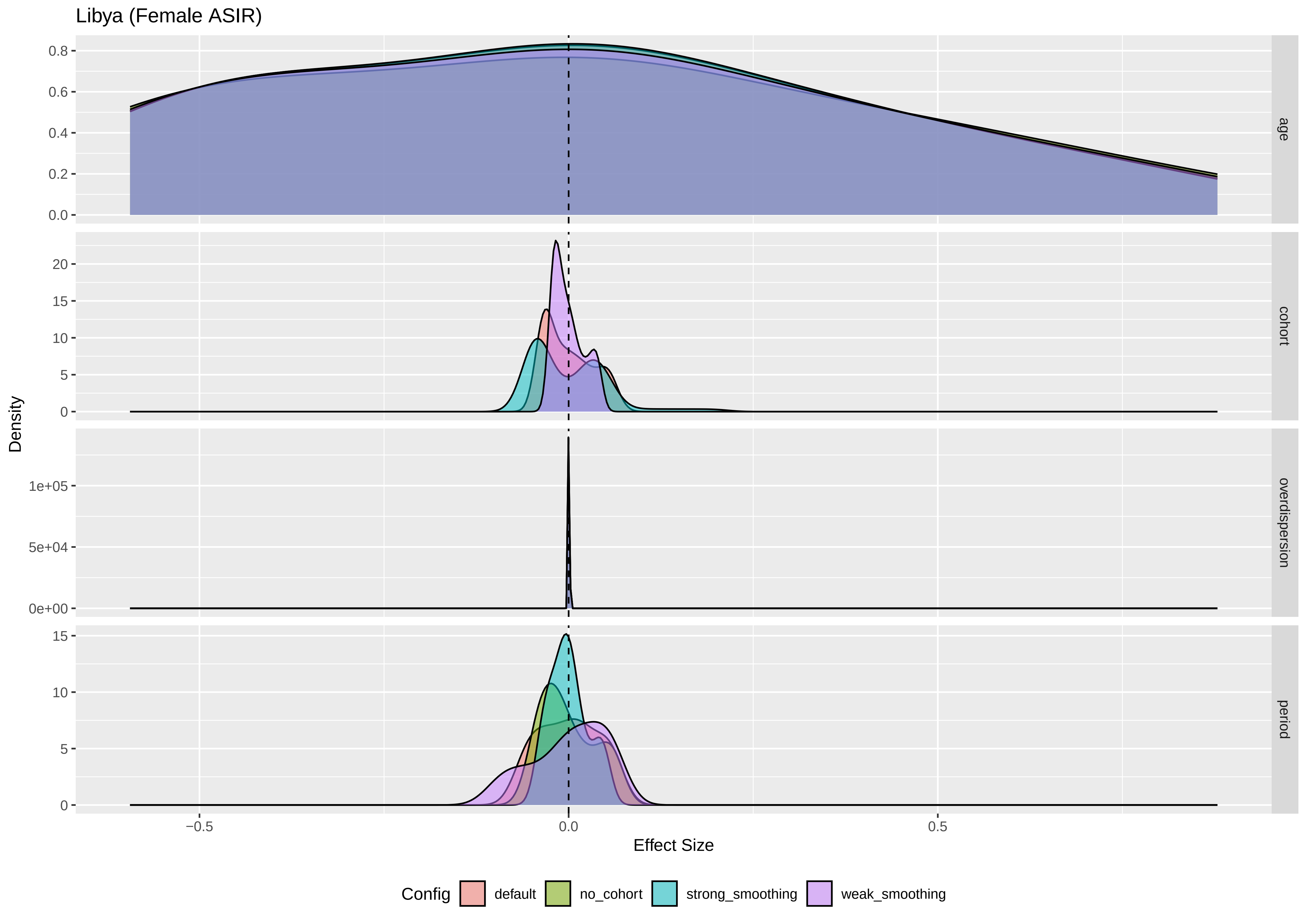

Libya (Female ASIR)
0.8
0.6
0.4
0.2
0.0
age
20
15
10
5
0
cohort
1e+05
5e+04
0e+00
overdispersion
15
10
5
0
period
Density
−0.5
0.0
0.5
Effect Size
Config
default
no_cohort
strong_smoothing
weak_smoothing

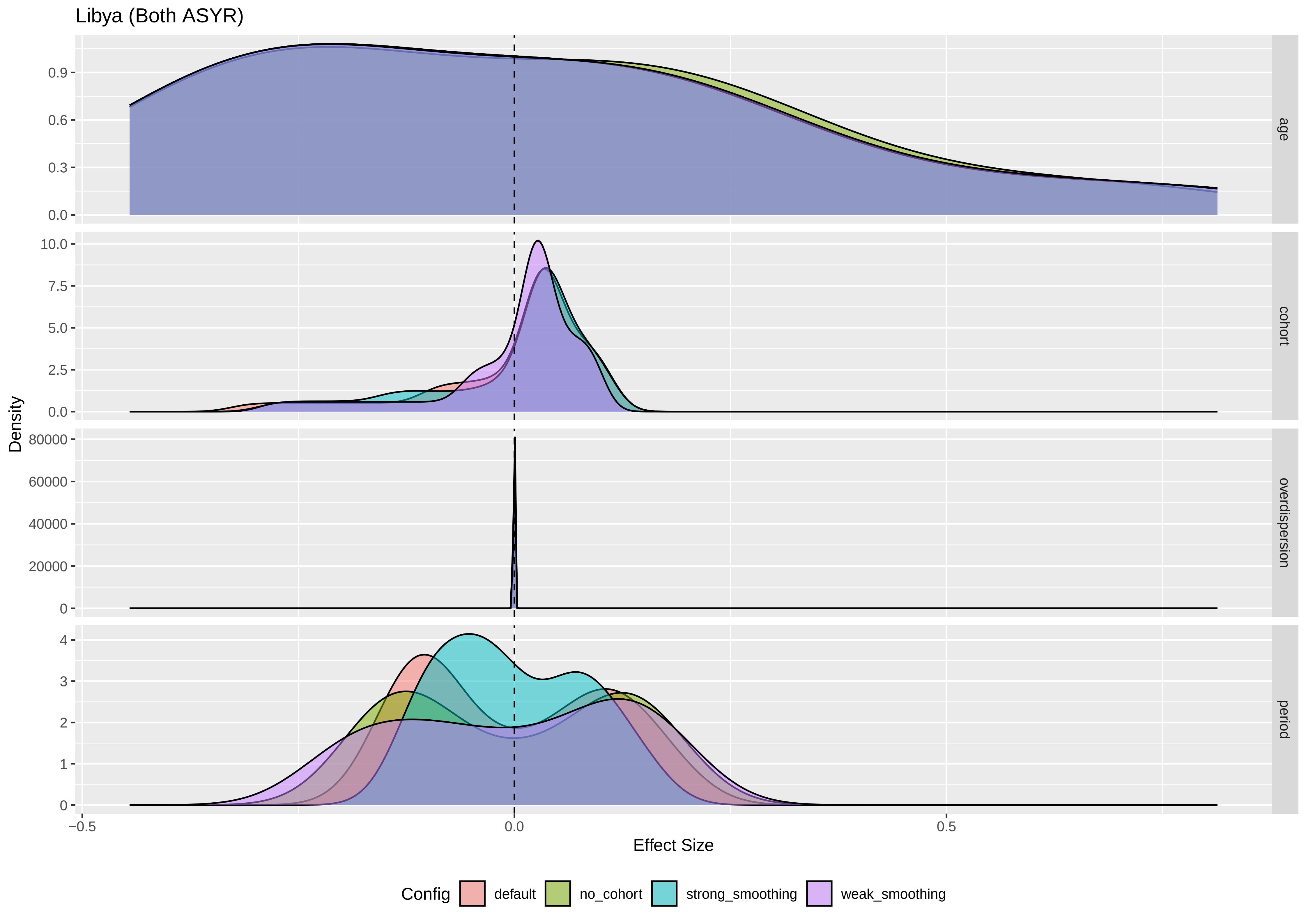

Libya (Both ASYR)
Density
Effect Size
age
cohort
overdispersion
period
0.0
0.3
0.6
0.9
0.0
2.5
5.0
7.5
10.0
0
20000
40000
60000
80000
0
1
2
3
4
−0.5
0.0
0.5
Config
default
no_cohort
strong_smoothing
weak_smoothing

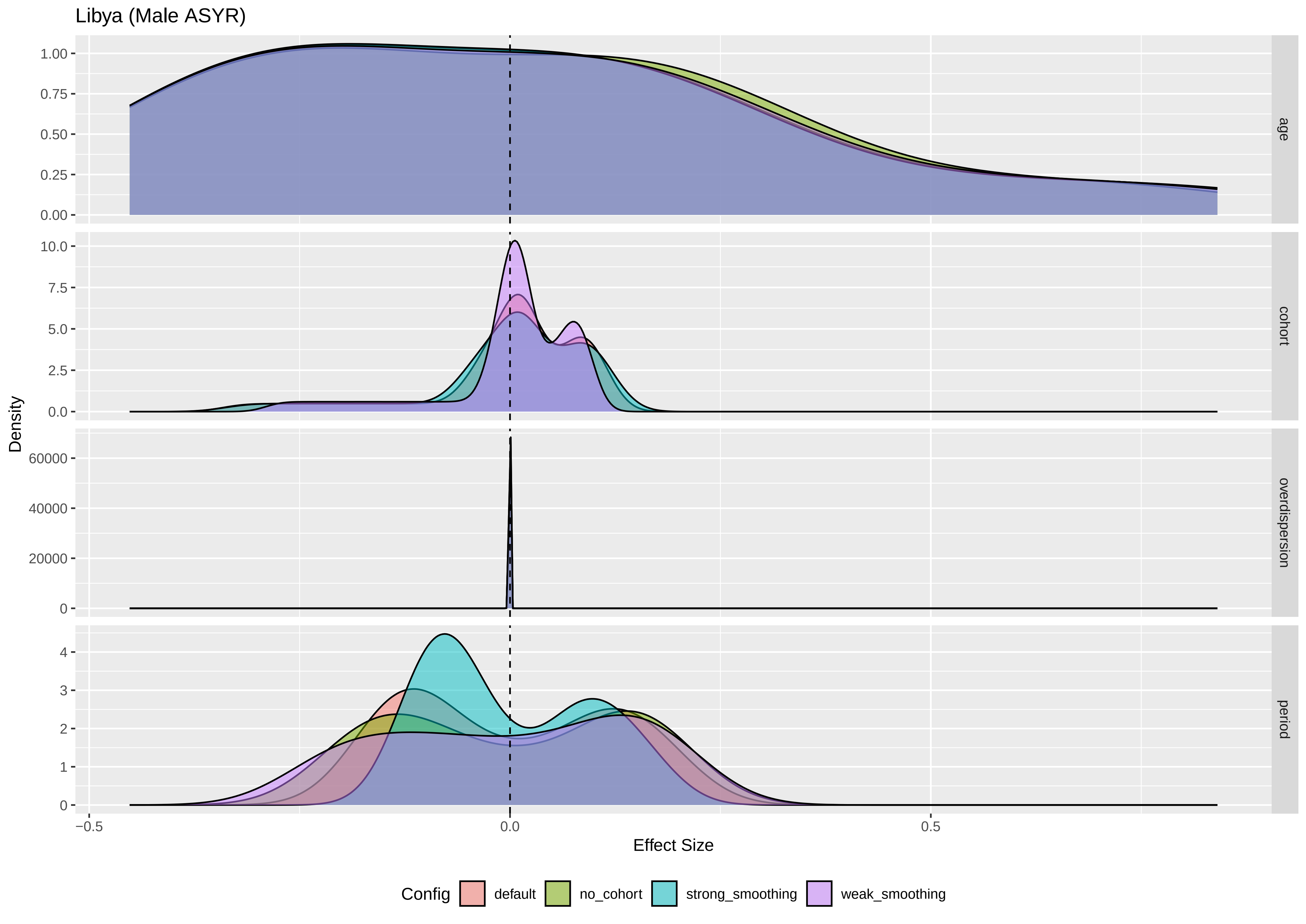

Libya (Male ASYR)
age
cohort
overdispersion
period
1.00
0.75
0.50
0.25
0.00
10.0
7.5
5.0
2.5
0.0
60000
40000
20000
0
4
3
2
1
0
Density
−0.5
0.0
0.5
Effect Size
Config
default
no_cohort
strong_smoothing
weak_smoothing

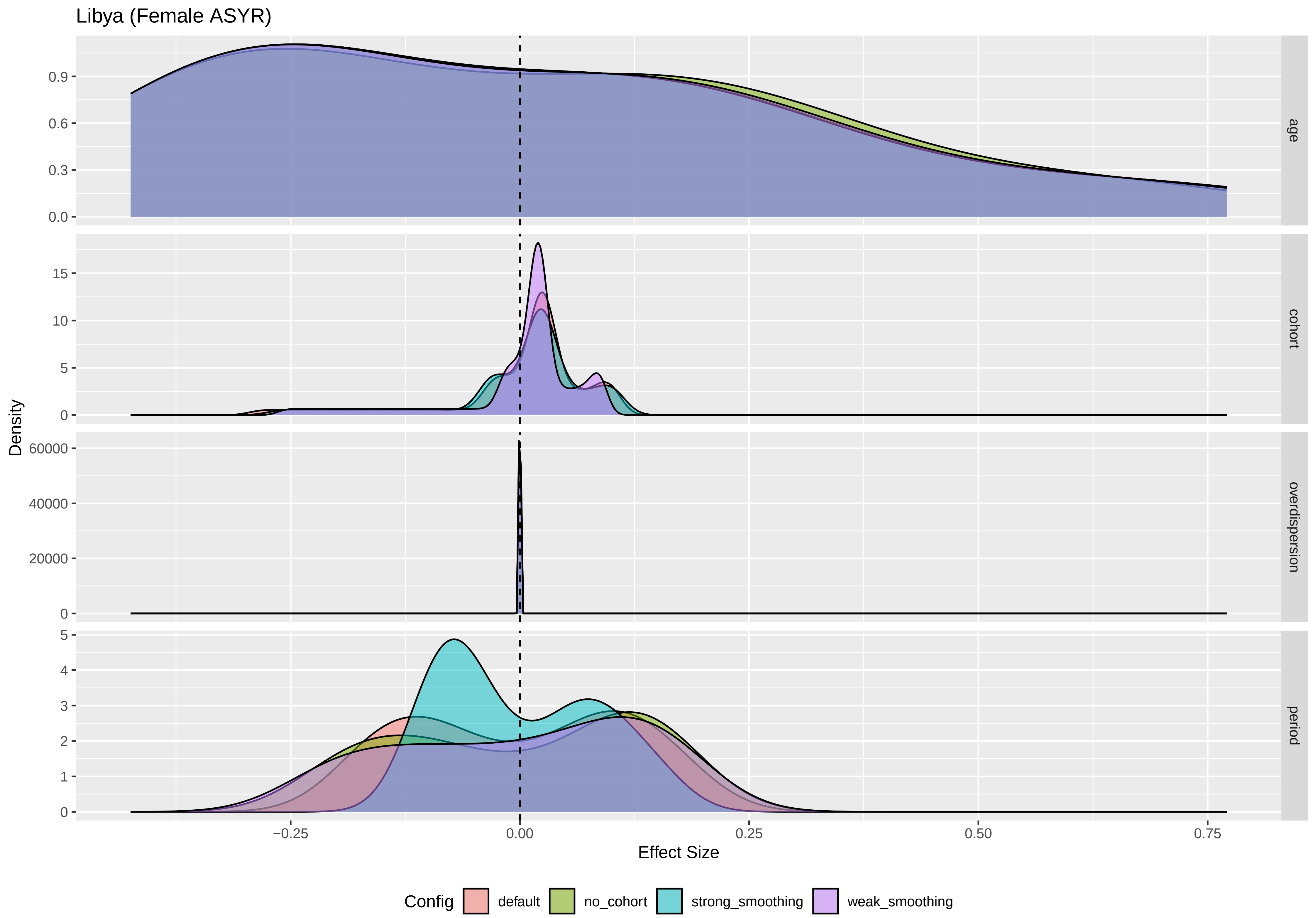

Libya (Female ASYR)
Density
0.9
0.6
0.3
0.0
age
15
10
5
0
cohort
60000
40000
20000
0
overdispersion
5
4
3
2
1
0
period
−0.25
0.00
0.25
0.50
0.75
Effect Size
Config
default
no_cohort
strong_smoothing
weak_smoothing

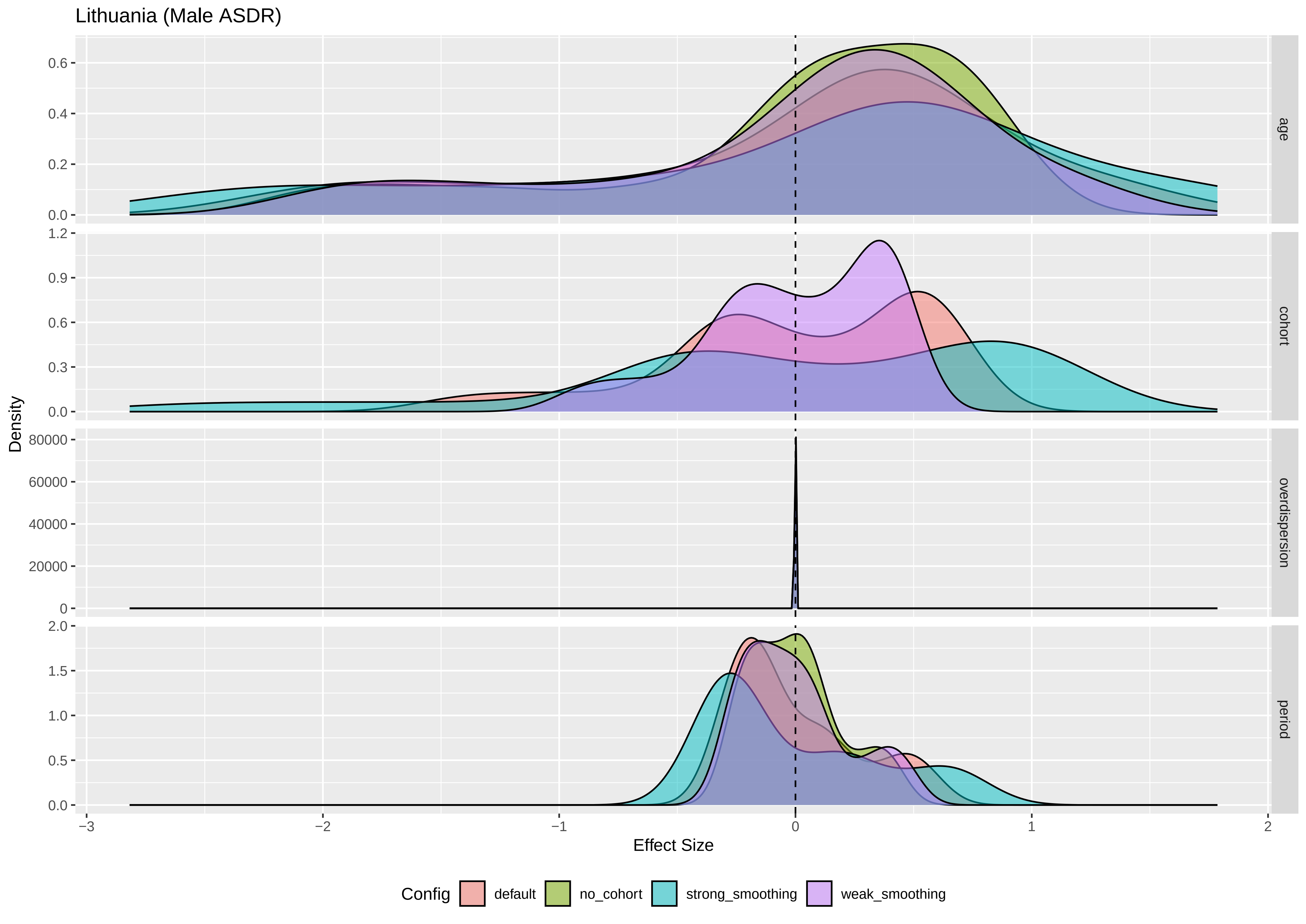

Lithuania (Male ASDR)
Density
Effect Size
age
cohort
overdispersion
period
0.0
0.2
0.4
0.6
0.3
0.6
0.9
1.2
0
20000
40000
60000
80000
0.5
1.0
1.5
2.0
−3
−2
−1
0
1
2
Config
default
no_cohort
strong_smoothing
weak_smoothing

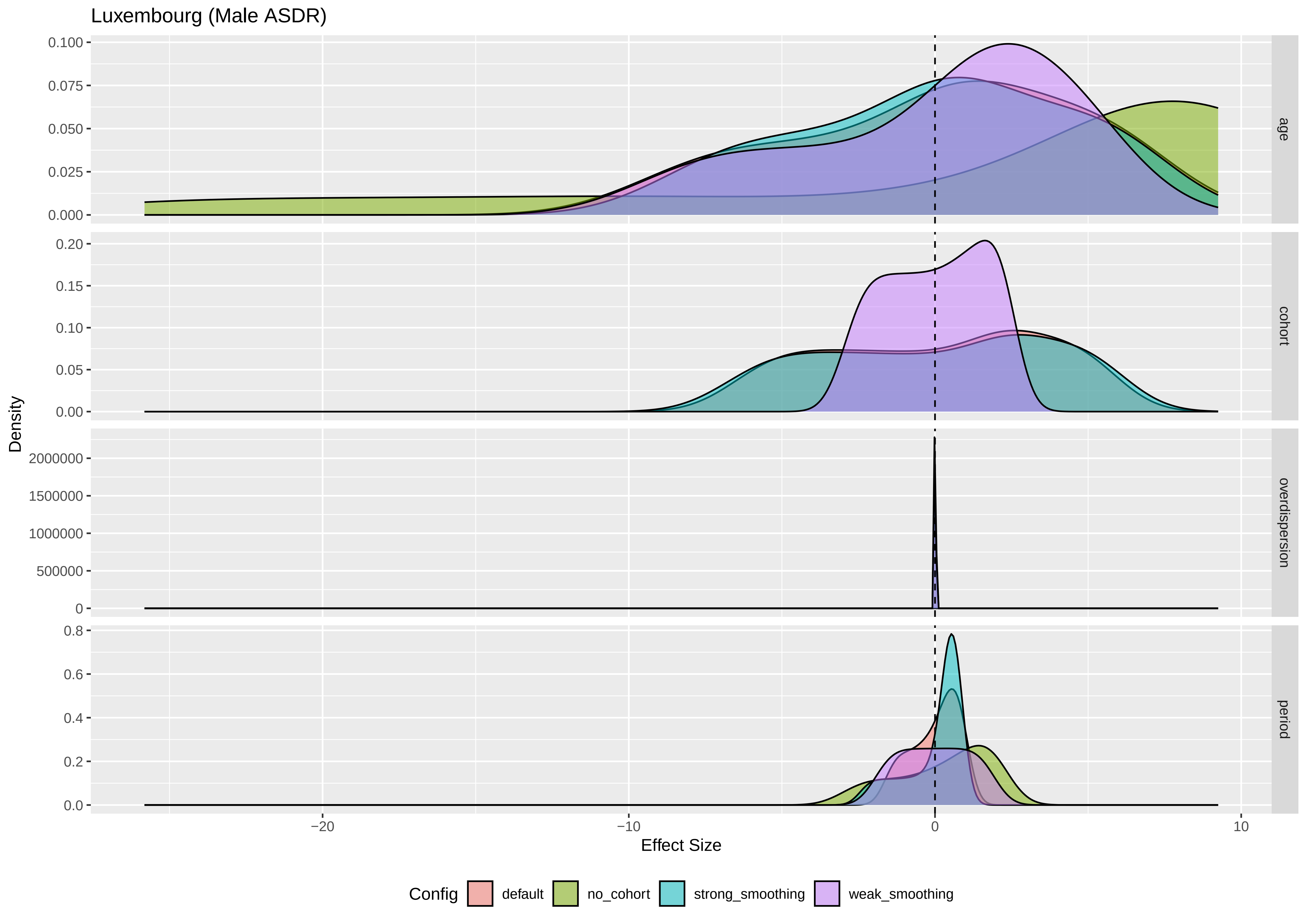
Luxembourg (Male ASDR)
age
cohort
overdispersion
period
Density
Effect Size
0.000
0.025
0.050
0.075
0.100
0.00
0.05
0.10
0.15
0.20
0
500000
1000000
1500000
2000000
0.0
0.2
0.4
0.6
0.8
−20
−10
0
10
Config
default
no_cohort
strong_smoothing
weak_smoothing

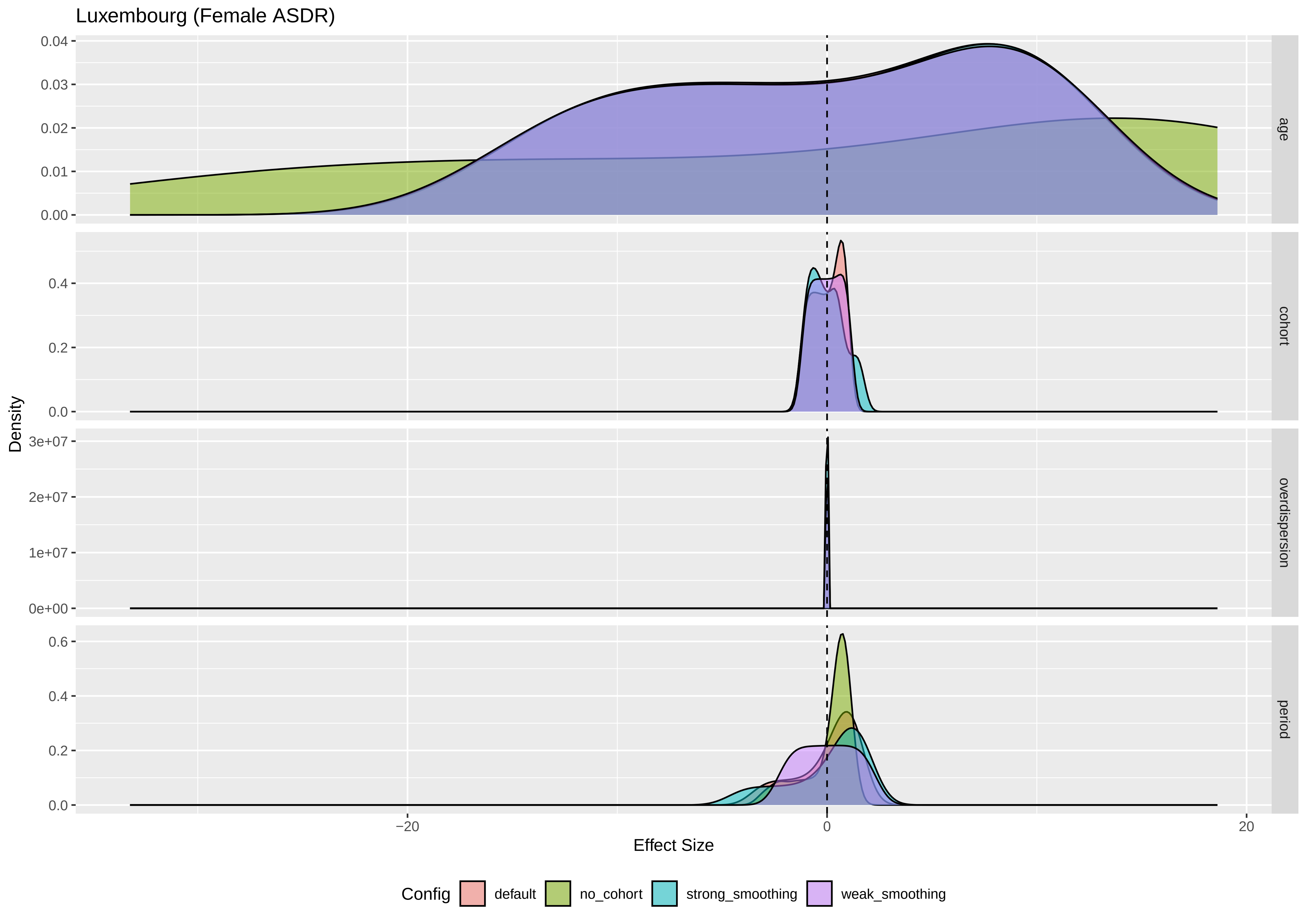
Luxembourg (Female ASDR)
Density
Effect Size
age
cohort
overdispersion
period
0.00
0.01
0.02
0.03
0.04
0.0
0.2
0.4
0e+00
1e+07
2e+07
3e+07
0.6
−20
0
20
Config
default
no_cohort
strong_smoothing
weak_smoothing

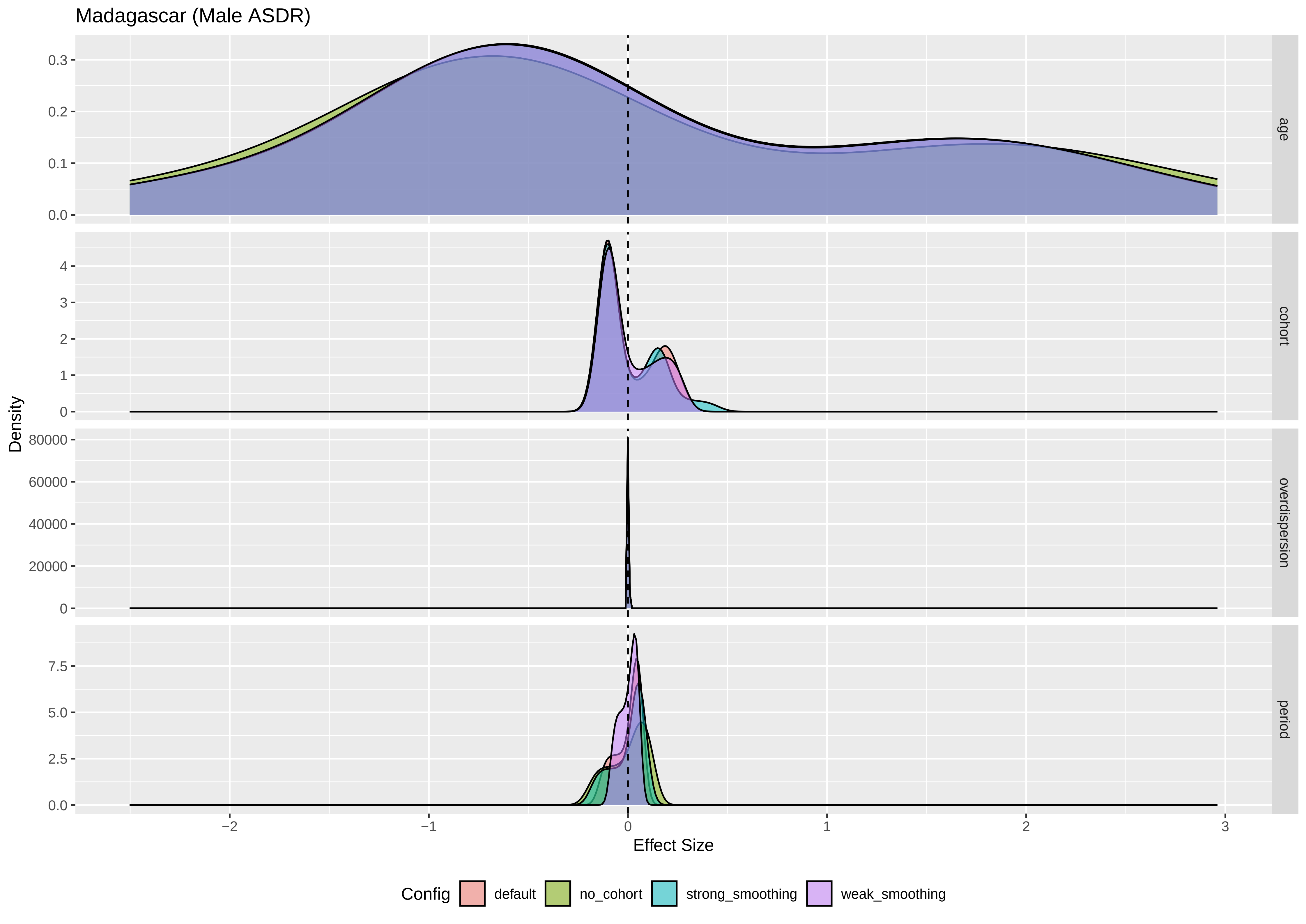

Madagascar (Male ASDR)
Density
Effect Size
age
cohort
overdispersion
period
0.0
0.1
0.2
0.3
0
1
2
3
4
0
20000
40000
60000
80000
0.0
2.5
5.0
7.5
−2
−1
0
1
2
3
Config
default
no_cohort
strong_smoothing
weak_smoothing

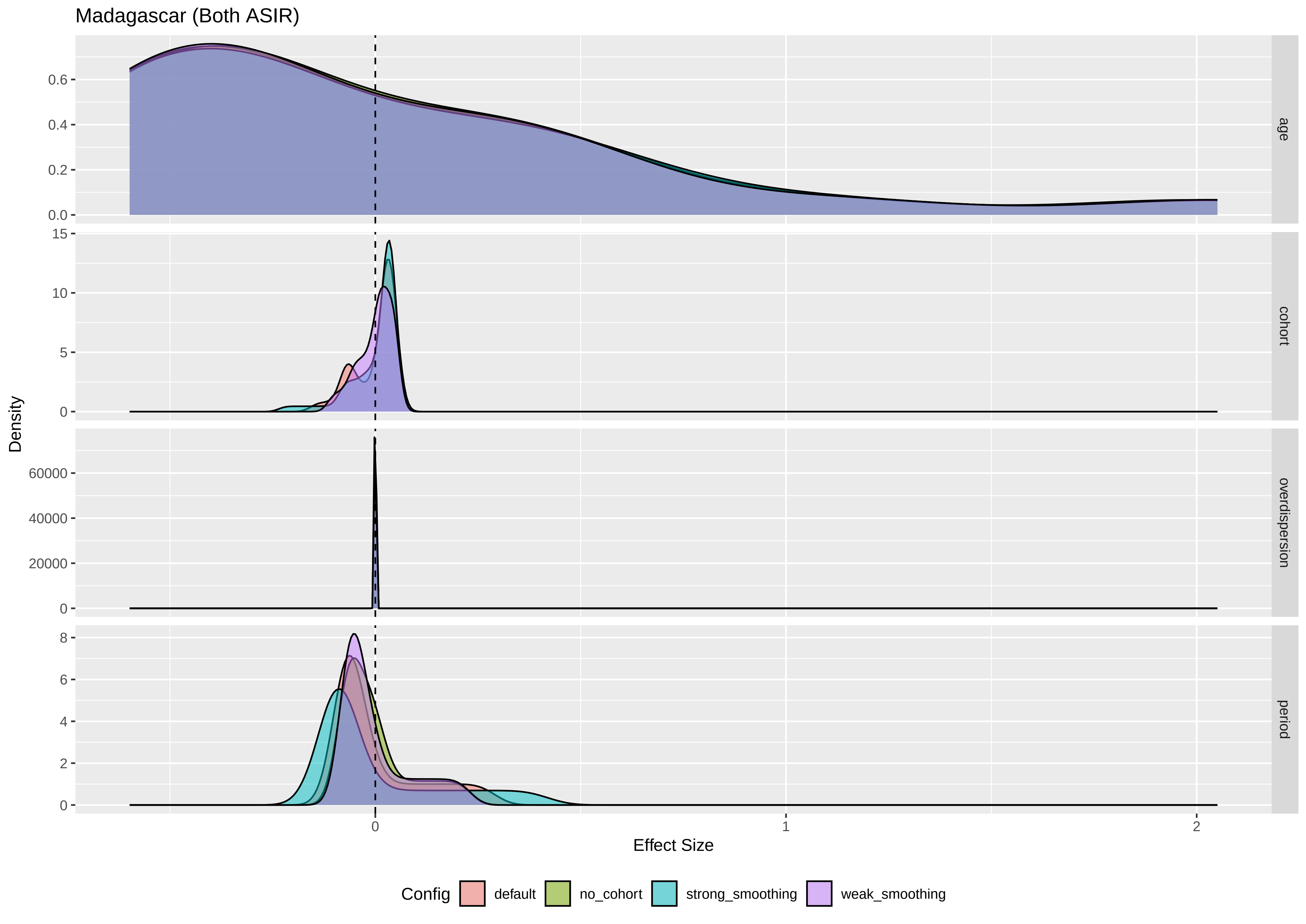

Madagascar (Both ASIR)
Density
Effect Size
age
cohort
overdispersion
period
0.0
0.2
0.4
0.6
0
5
10
15
0
20000
40000
60000
0
2
4
6
8
0
1
2
Config
default
no_cohort
strong_smoothing
weak_smoothing

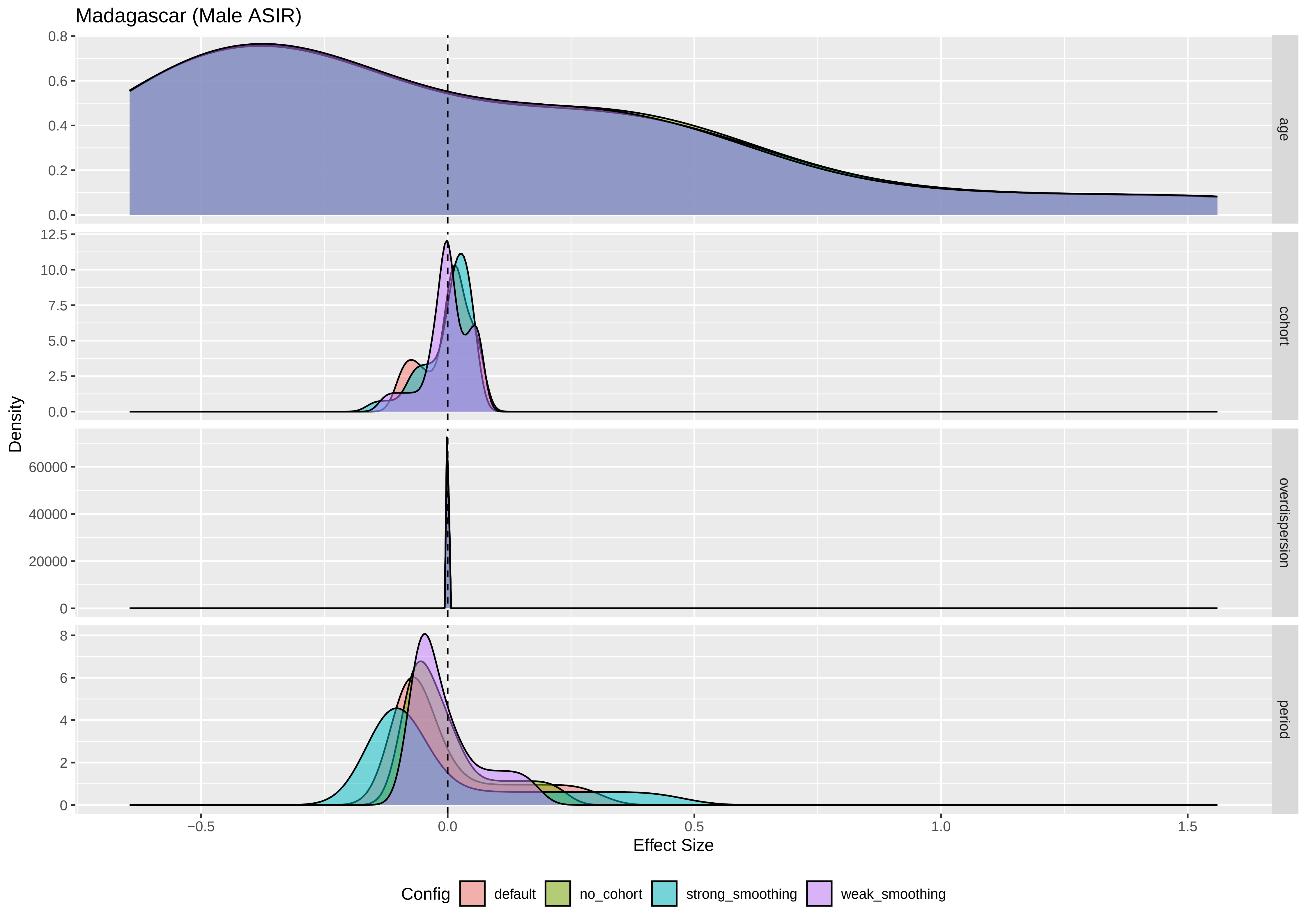

Madagascar (Male ASIR)
age
cohort
overdispersion
period
Density
Effect Size
−0.5
0.0
0.5
1.0
1.5
Config
default
no_cohort
strong_smoothing
weak_smoothing

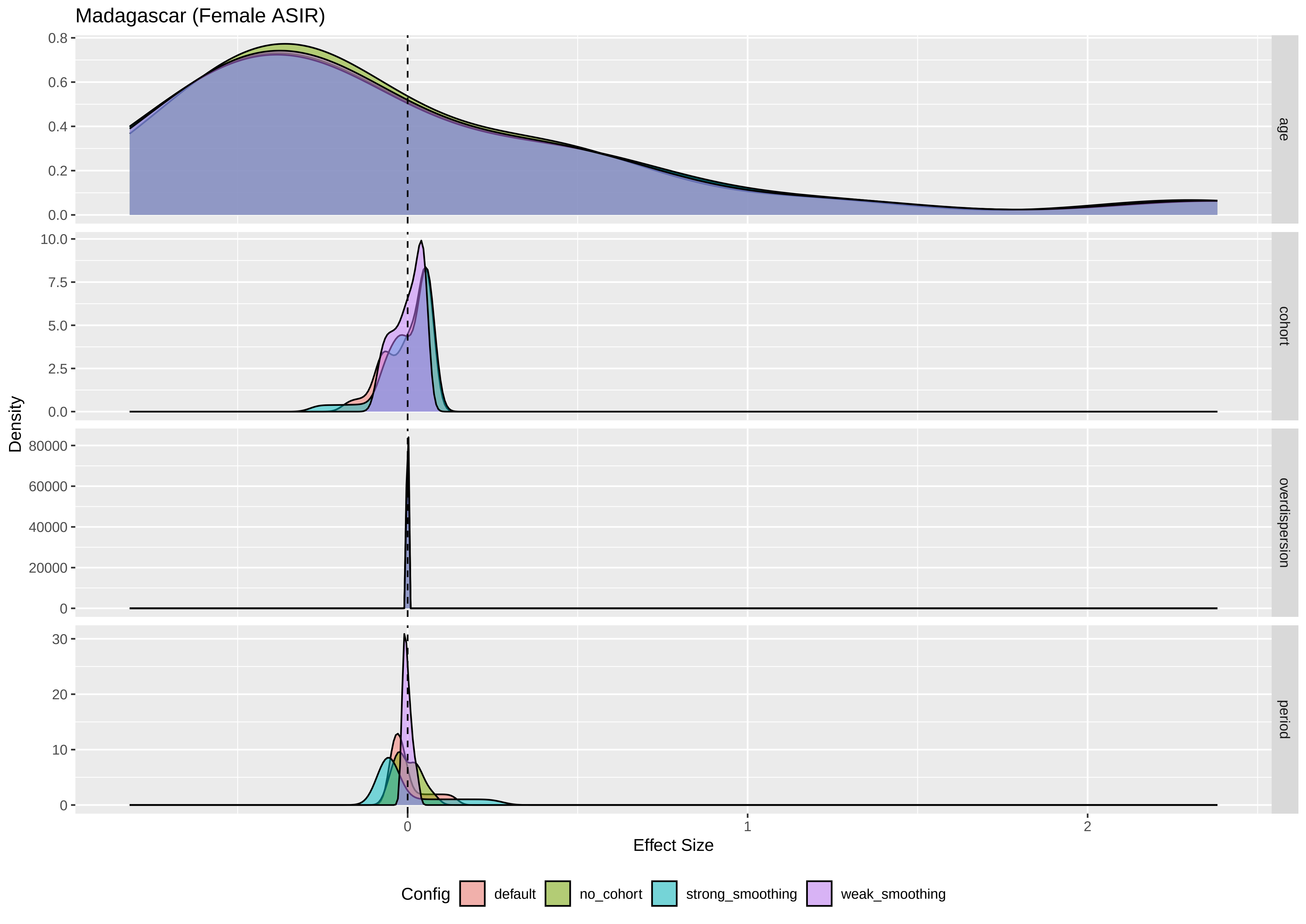

Madagascar (Female ASIR)
Density
Effect Size
age
cohort
overdispersion
period
0.0
0.2
0.4
0.6
0.8
0.0
2.5
5.0
7.5
10.0
0
20000
40000
60000
80000
0
10
20
30
0
1
2
Config
default
no_cohort
strong_smoothing
weak_smoothing

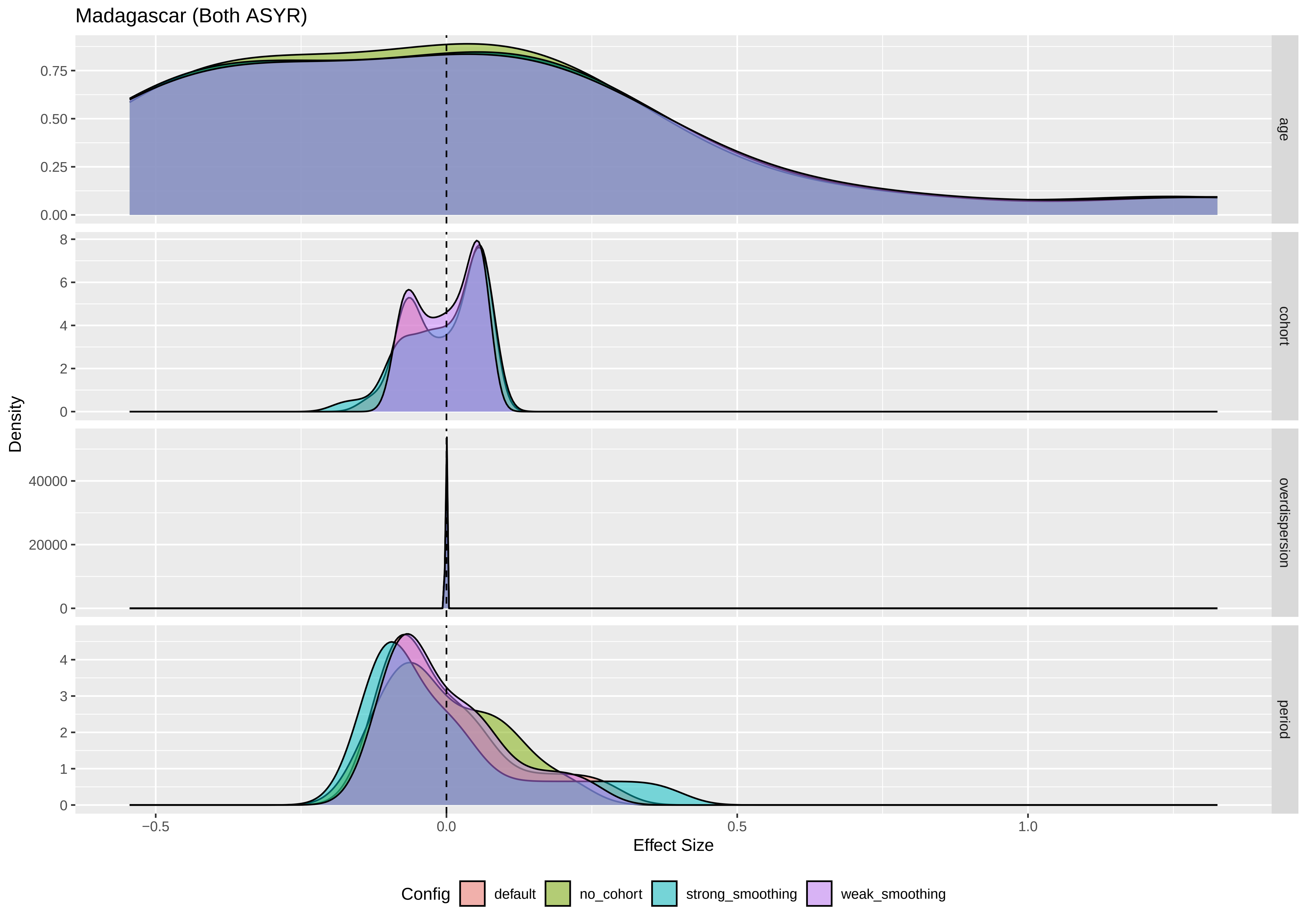

Madagascar (Both ASYR)
age
cohort
overdispersion
period
Density
Effect Size
0.75
0.50
0.25
0.00
8
6
4
2
0
40000
20000
0
4
3
2
1
0
−0.5
0.0
0.5
1.0
Config
default
no_cohort
strong_smoothing
weak_smoothing

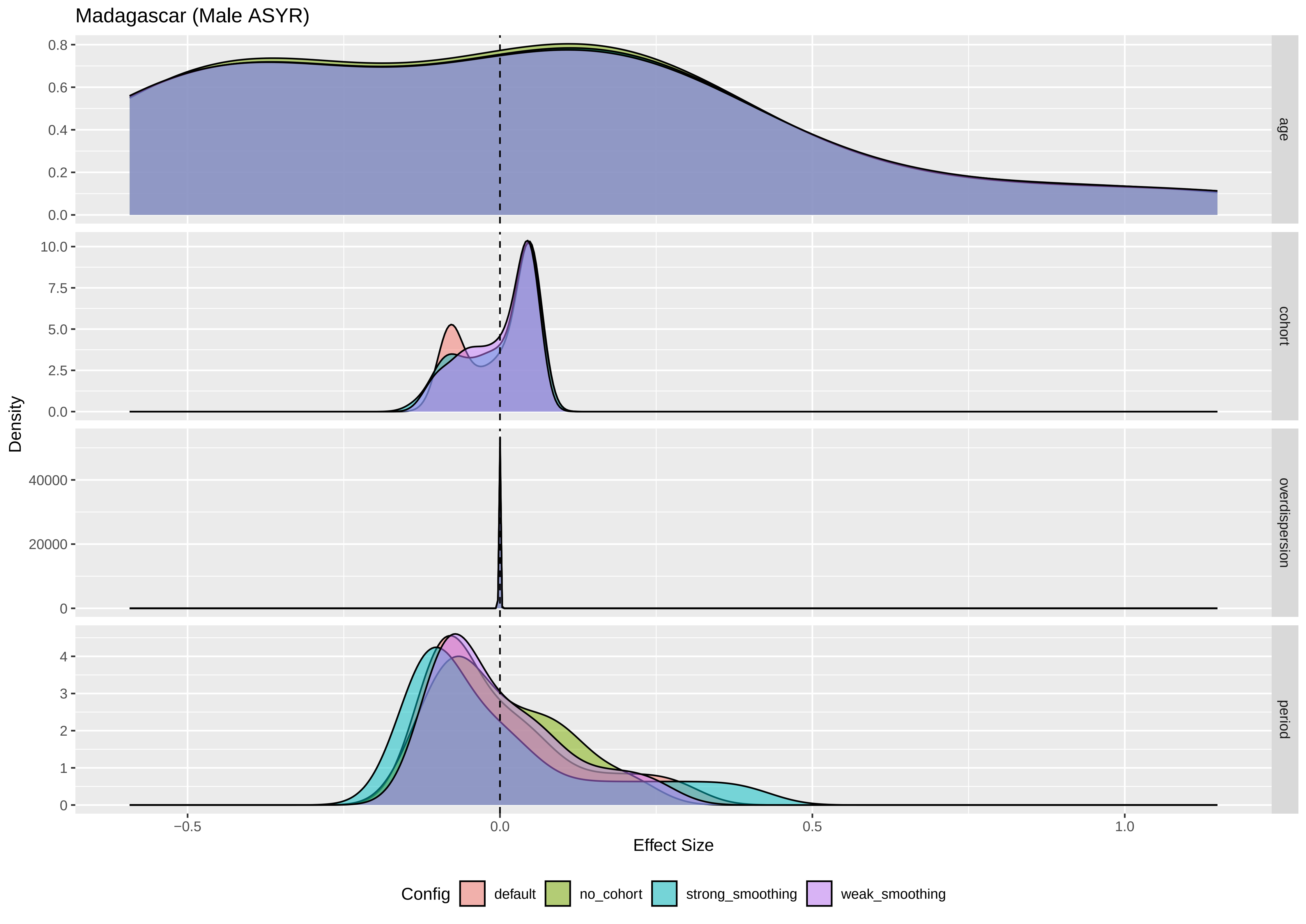

Madagascar (Male ASYR)
Density
Effect Size
age
cohort
overdispersion
period
0.0
0.2
0.4
0.6
0.8
0.0
2.5
5.0
7.5
10.0
0
20000
40000
0
1
2
3
4
−0.5
0.0
0.5
1.0
Config
default
no_cohort
strong_smoothing
weak_smoothing

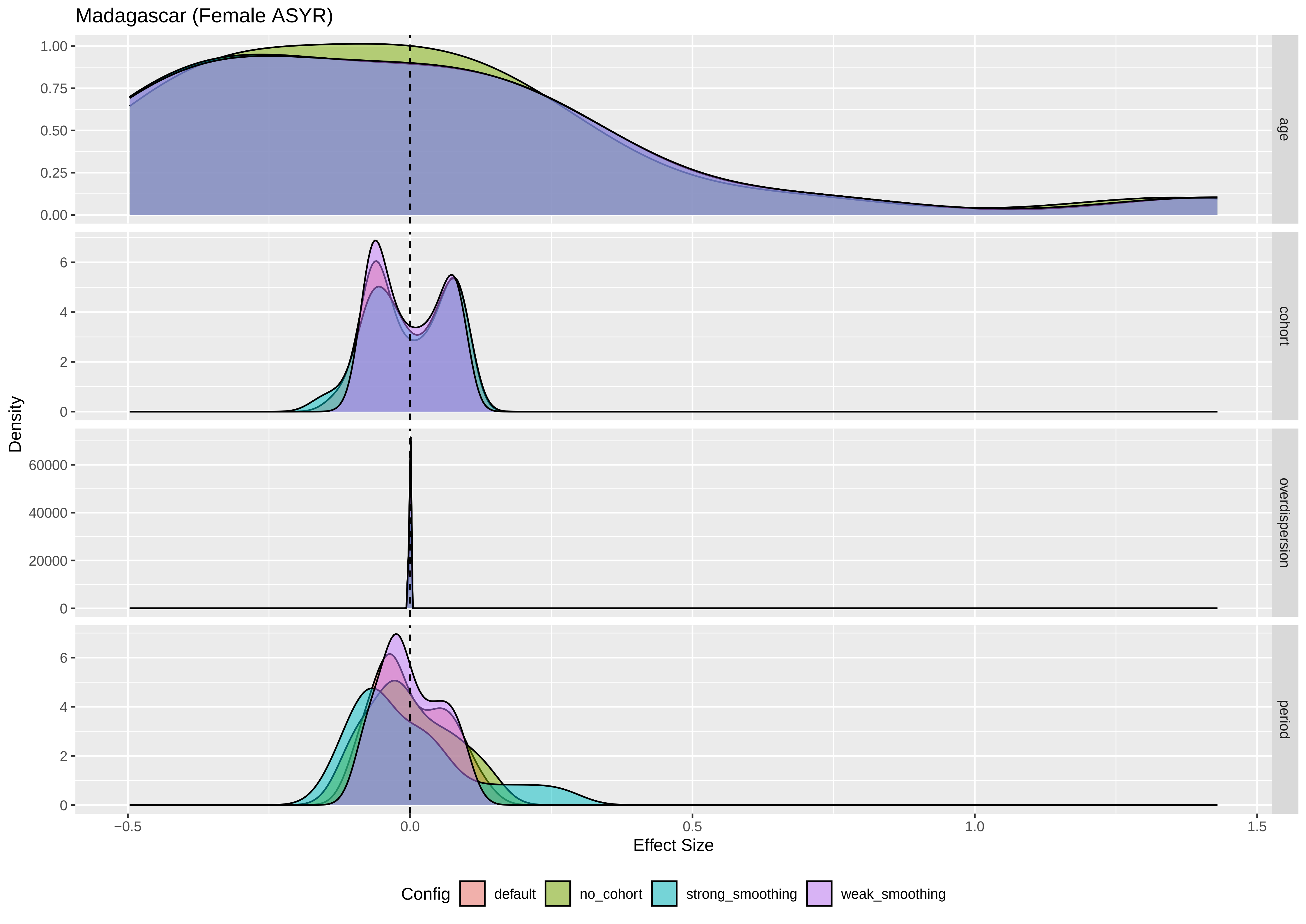

Madagascar (Female ASYR)
Density
Effect Size
age
cohort
overdispersion
period
1.00
0.75
0.50
0.25
0.00
6
4
2
0
60000
40000
20000
0
−0.5
0.0
0.5
1.0
1.5
Config
default
no_cohort
strong_smoothing
weak_smoothing

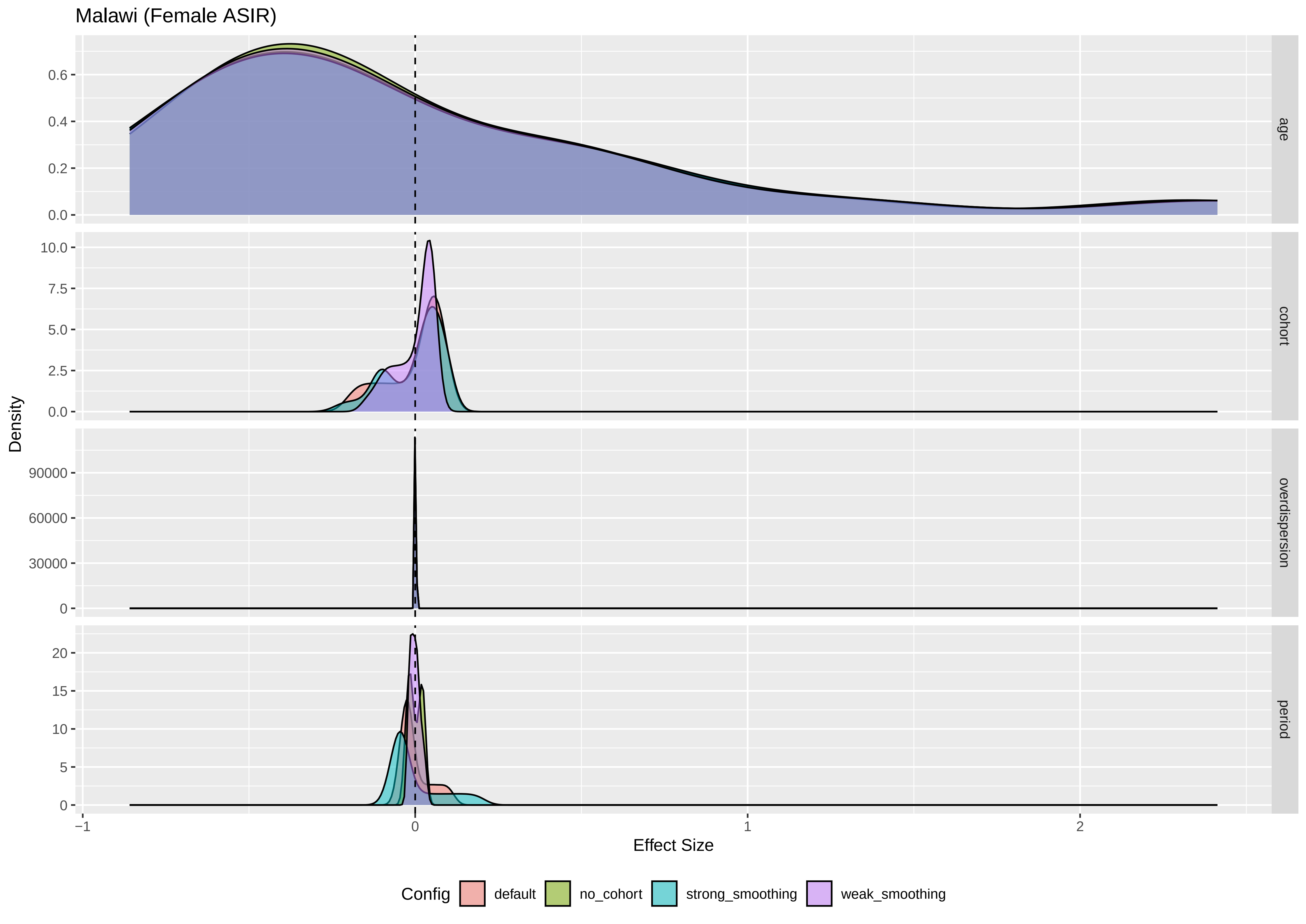

Malawi (Female ASIR)
Density
Effect Size
age
cohort
overdispersion
period
0.0
0.2
0.4
0.6
2.5
5.0
7.5
10.0
0
30000
60000
90000
5
10
15
20
−1
0
1
2
Config
default
no_cohort
strong_smoothing
weak_smoothing

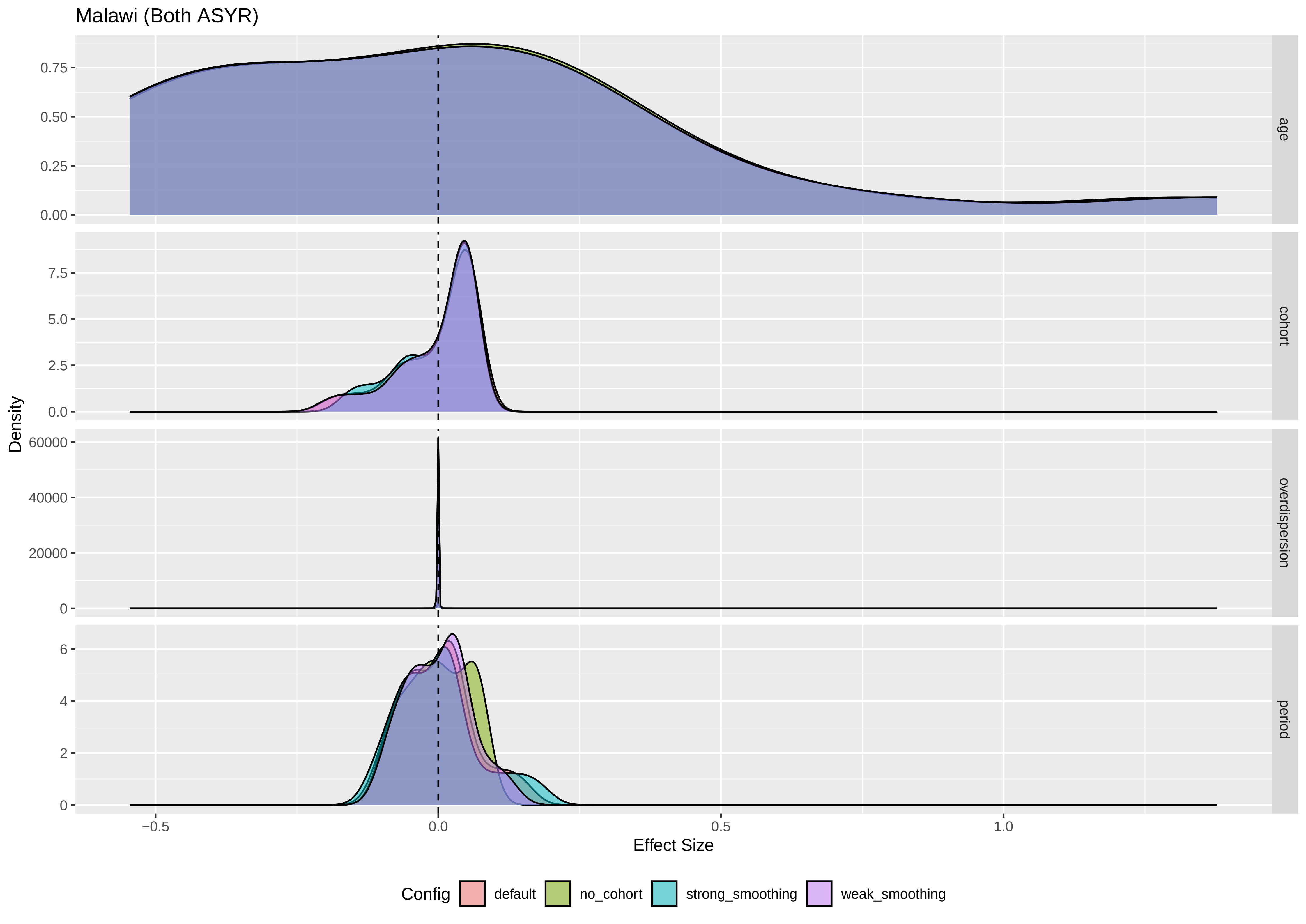

Malawi (Both ASYR)
0.75
0.50
0.25
0.00
age
7.5
5.0
2.5
0.0
cohort
60000
40000
20000
0
overdispersion
6
4
2
0
period
Density
−0.5
0.0
0.5
1.0
Effect Size
Config
default
no_cohort
strong_smoothing
weak_smoothing

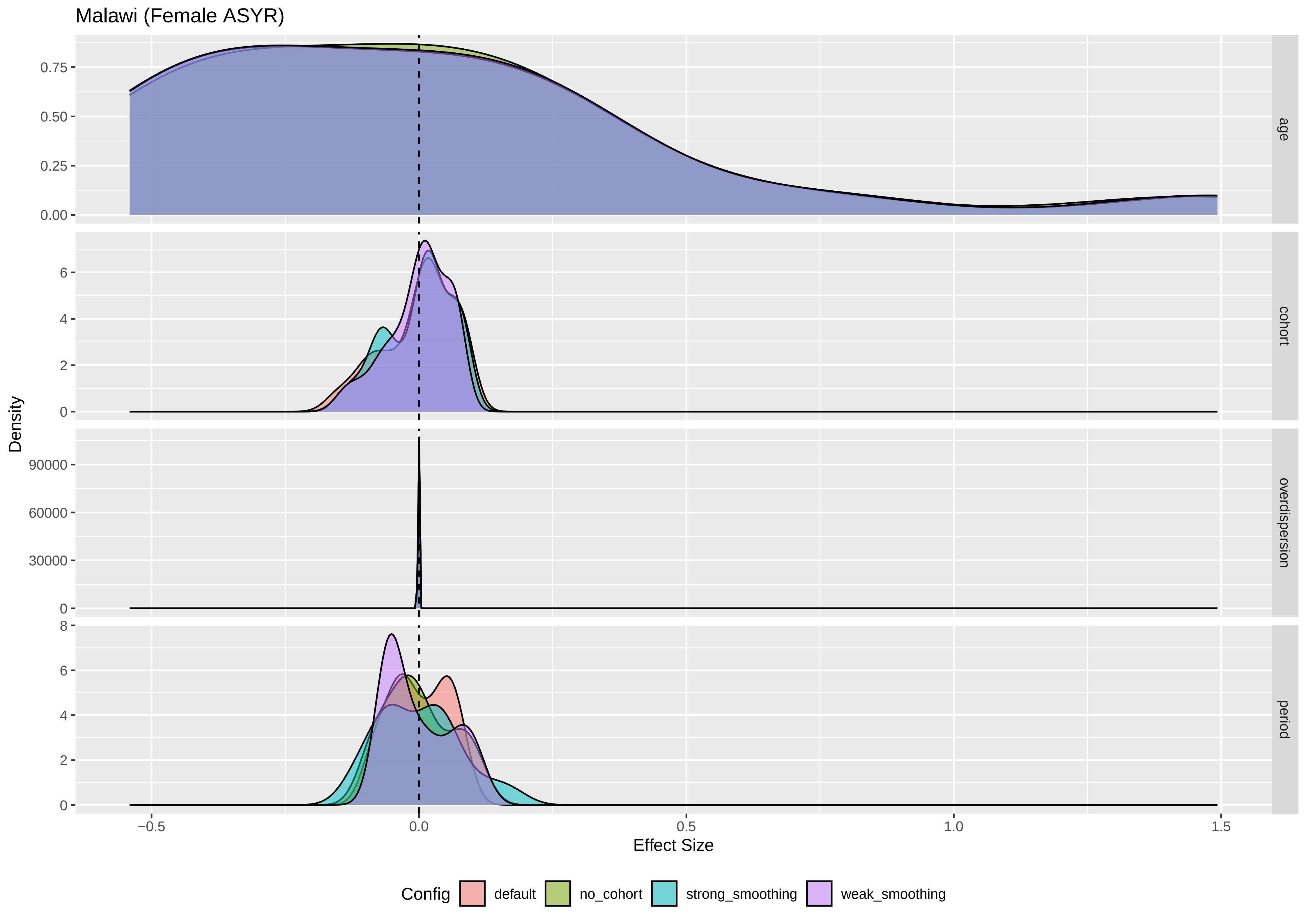

Malawi (Female ASYR)
Density
Effect Size
age
cohort
overdispersion
period
0.00
0.25
0.50
0.75
0
2
4
6
30000
60000
90000
8
−0.5
0.0
0.5
1.0
1.5
Config
default
no_cohort
strong_smoothing
weak_smoothing

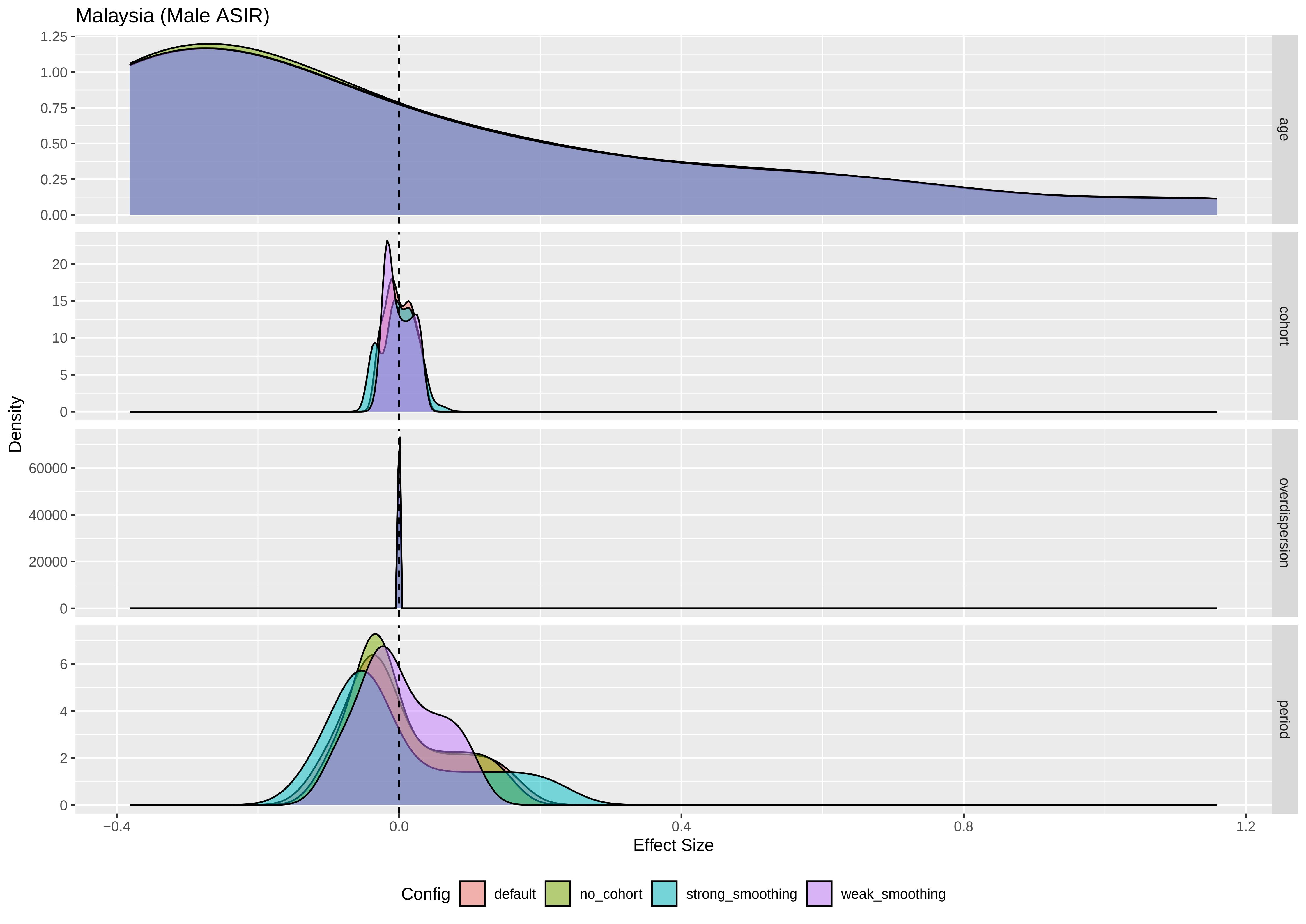

Malaysia (Male ASIR)
age
cohort
overdispersion
period
Density
Effect Size
1.25
1.00
0.75
0.50
0.25
0.00
20
15
10
5
0
60000
40000
20000
0
6
4
2
0
−0.4
0.0
0.4
0.8
1.2
Config
default
no_cohort
strong_smoothing
weak_smoothing

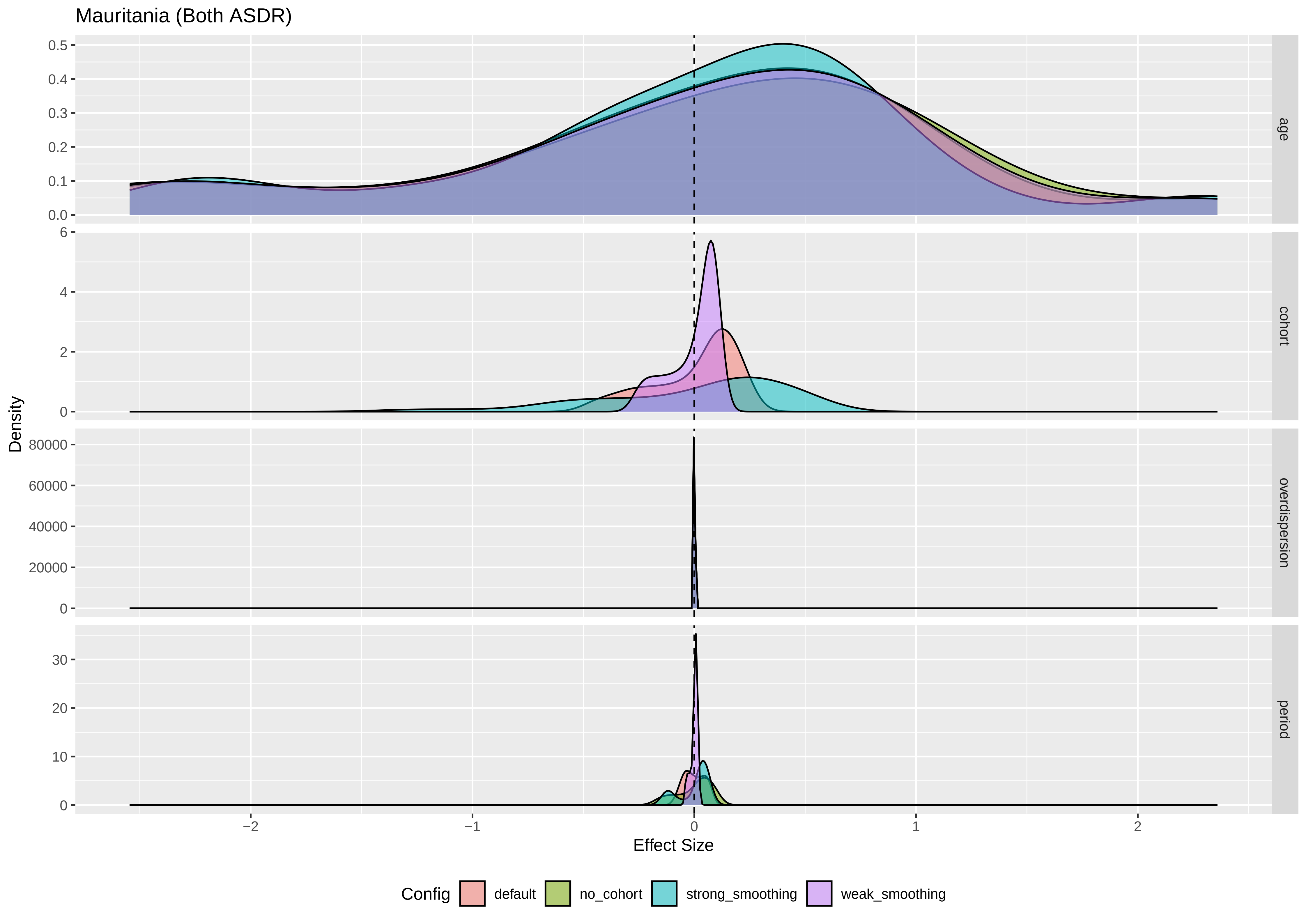

Mauritania (Both ASDR)
Density
Effect Size
age
cohort
overdispersion
period
0.0
0.1
0.2
0.3
0.4
0.5
0
2
4
6
0
20000
40000
60000
80000
0
10
20
30
−2
−1
0
1
2
Config
default
no_cohort
strong_smoothing
weak_smoothing

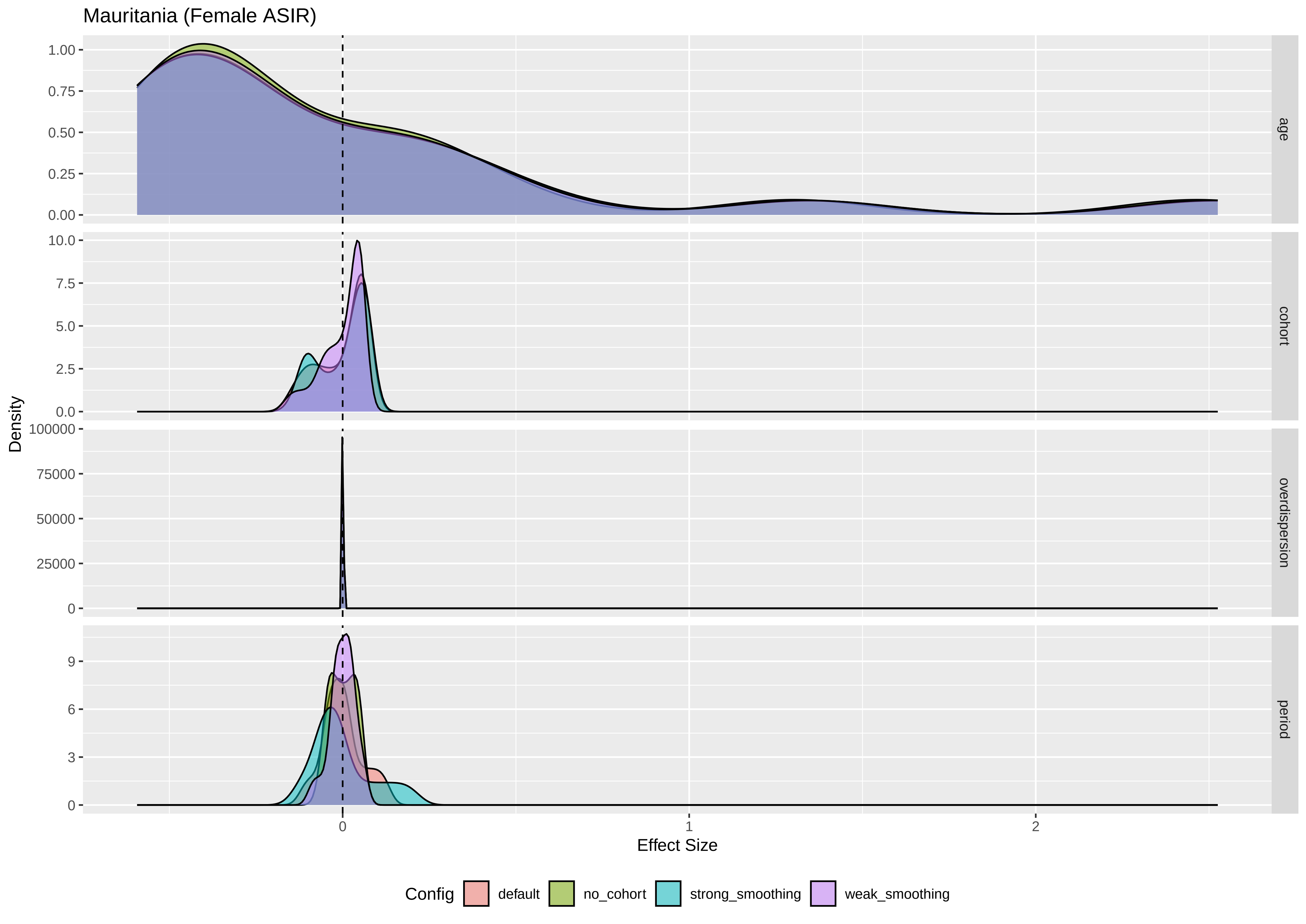

Mauritania (Female ASIR)
age
cohort
overdispersion
period
Density
Effect Size
1.00
0.75
0.50
0.25
0.00
10.0
7.5
5.0
2.5
0.0
100000
75000
50000
25000
0
9
6
3
0
1
2
Config
default
no_cohort
strong_smoothing
weak_smoothing

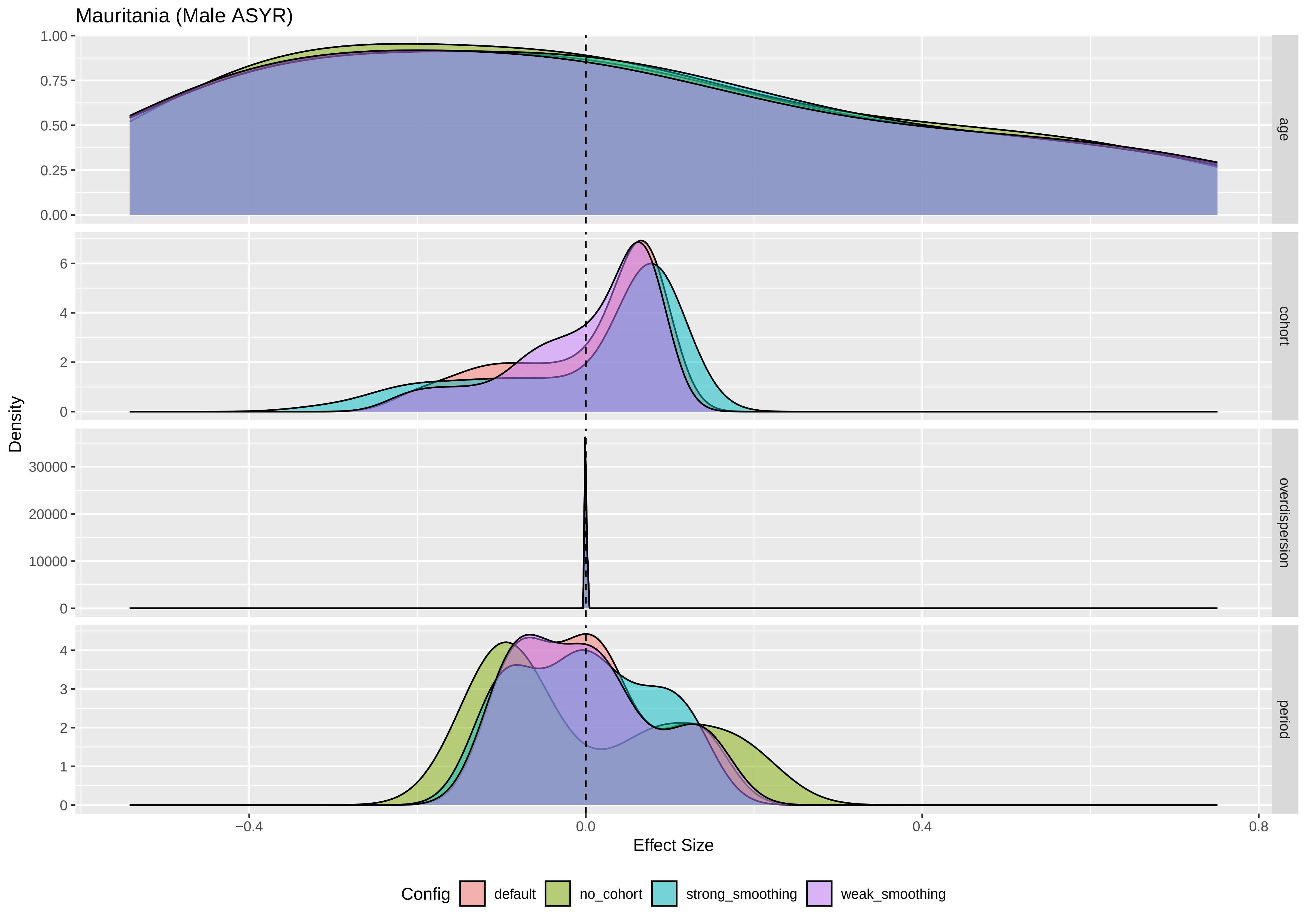

Mauritania (Male ASYR)
Density
Effect Size
age
cohort
overdispersion
period
1.00
0.75
0.50
0.25
0.00
6
4
2
0
30000
20000
10000
0
4
3
2
1
0
−0.4
0.0
0.4
0.8
Config
default
no_cohort
strong_smoothing
weak_smoothing

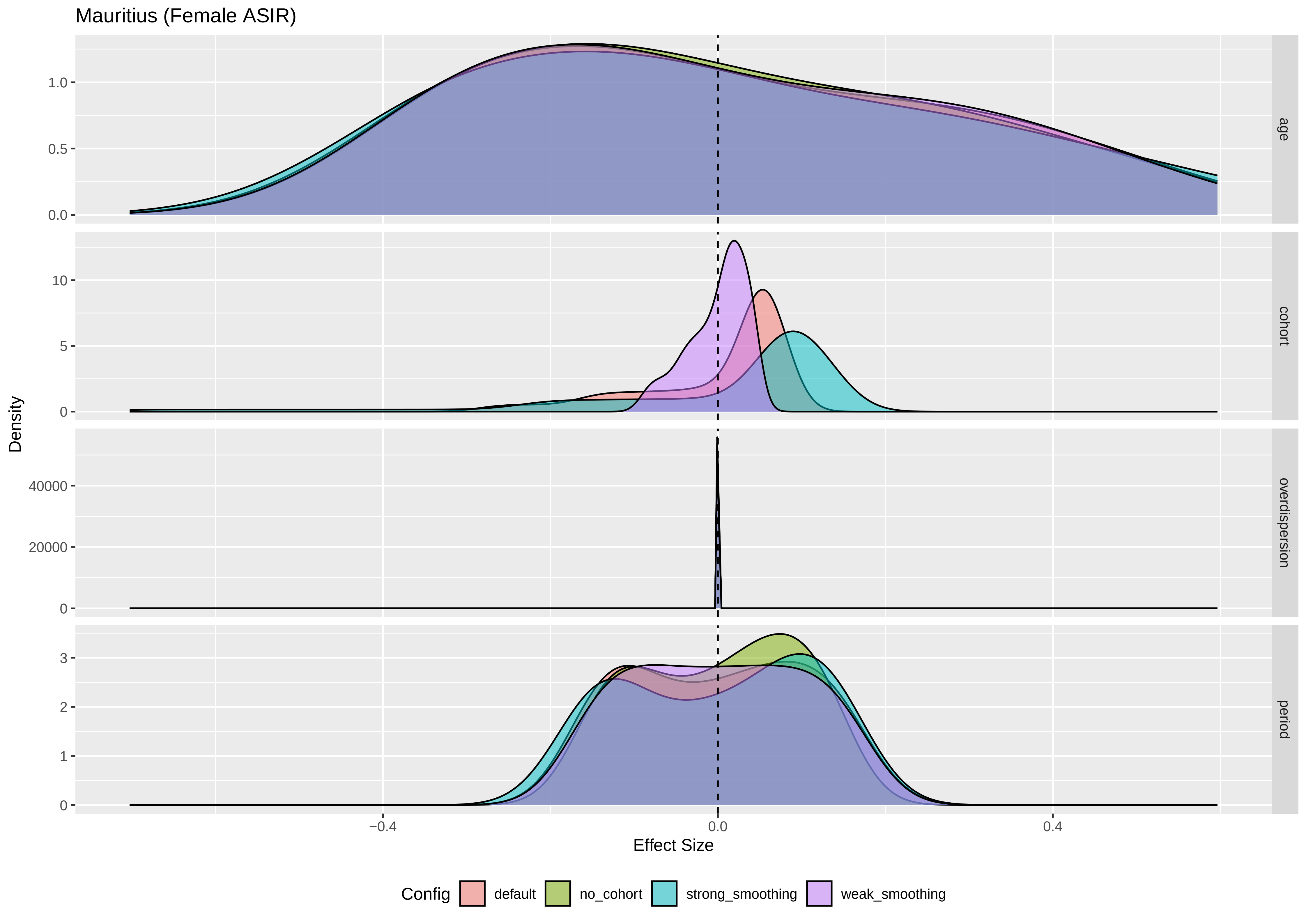

Mauritius (Female ASIR)
Density
Effect Size
age
cohort
overdispersion
period
1.0
0.5
0.0
10
5
0
40000
20000
0
3
2
1
0
−0.4
0.0
0.4
Config
default
no_cohort
strong_smoothing
weak_smoothing

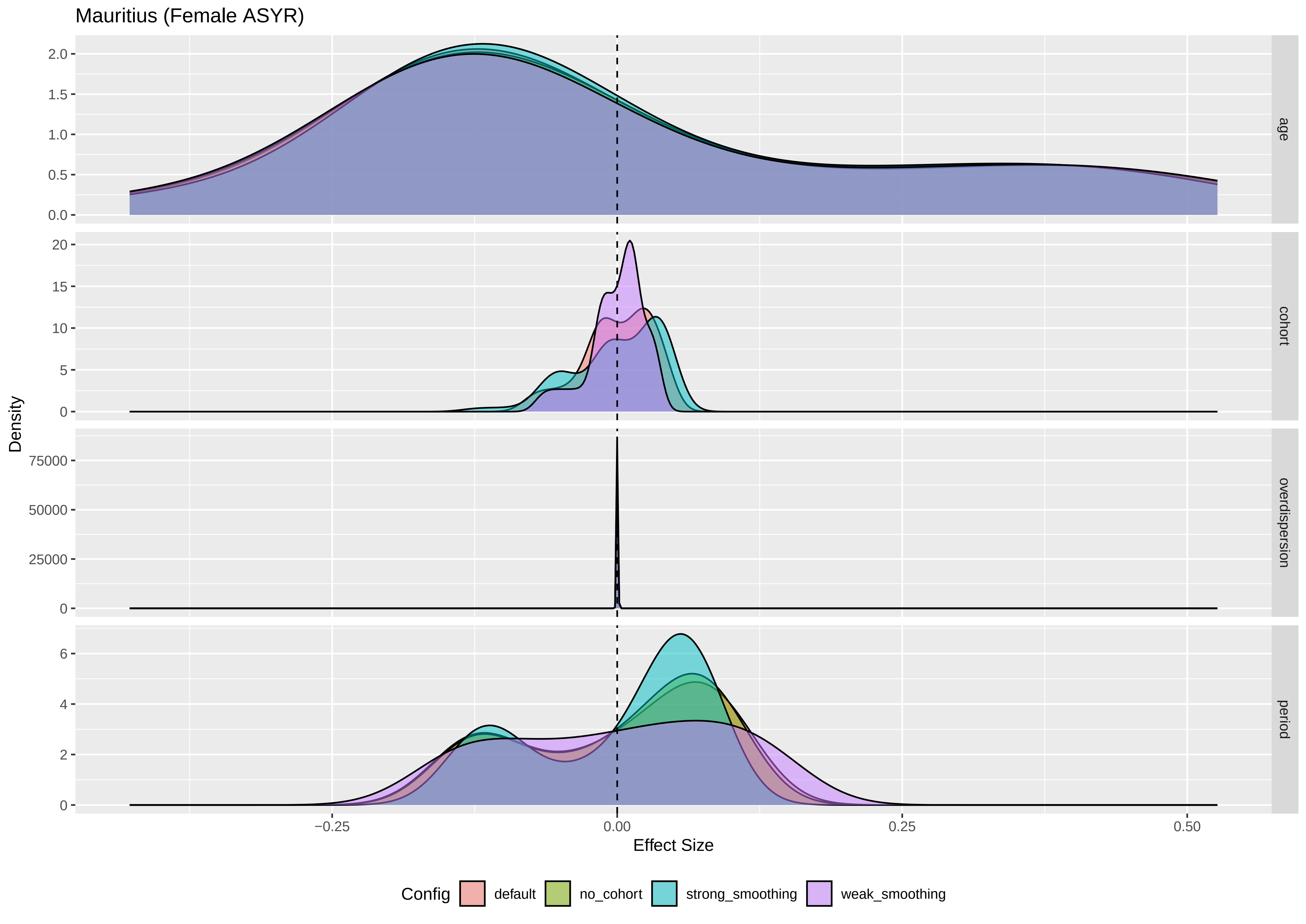

Mauritius (Female ASYR)
Density
Effect Size
age
cohort
overdispersion
period
2.0
1.5
1.0
0.5
0.0
20
15
10
5
0
75000
50000
25000
0
6
4
2
0
−0.25
0.00
0.25
0.50
Config
default
no_cohort
strong_smoothing
weak_smoothing

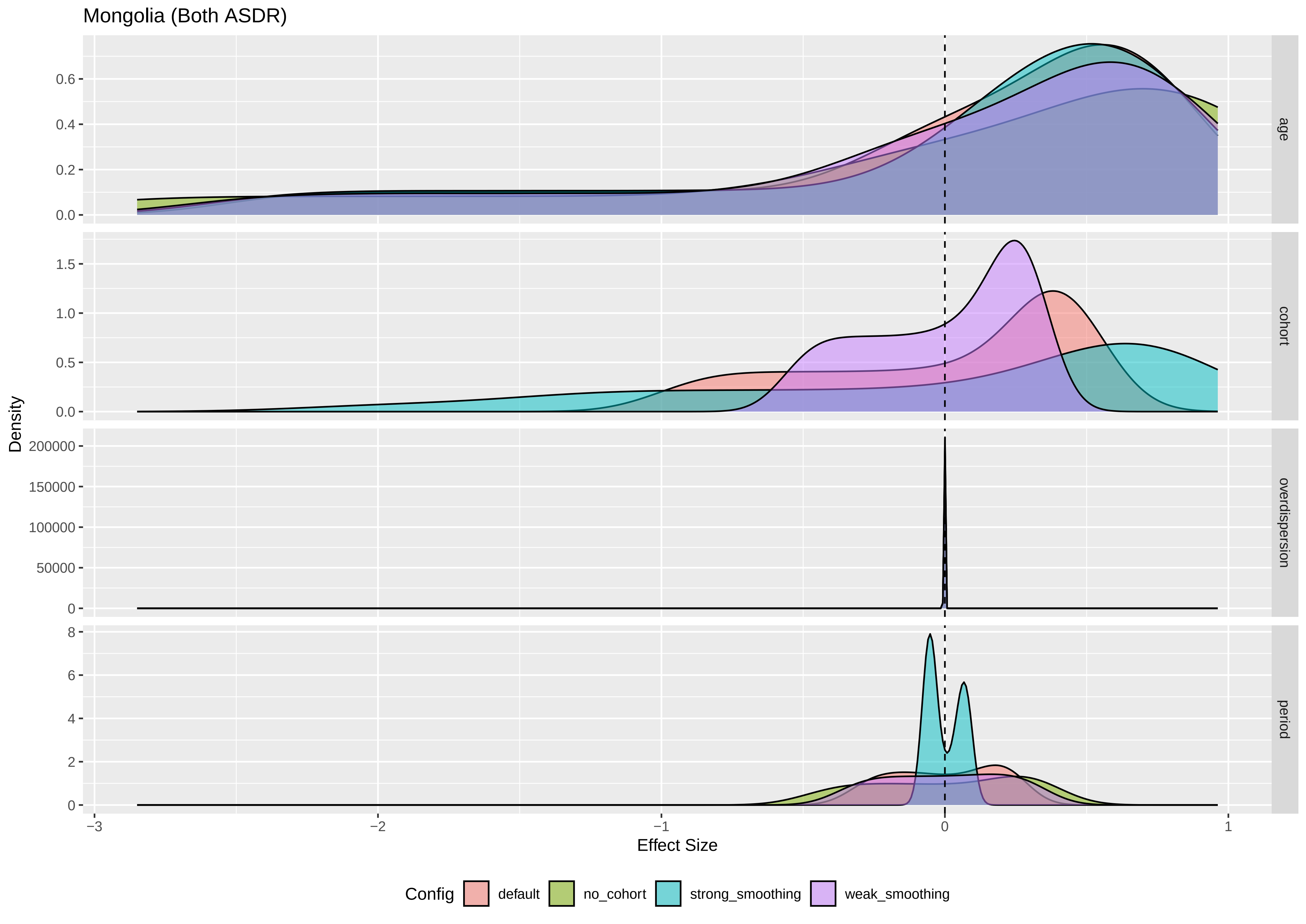

Mongolia (Both ASDR)
Density
Effect Size
age
cohort
overdispersion
period
0.0
0.2
0.4
0.6
0.5
1.0
1.5
0
50000
100000
150000
200000
2
4
6
8
−3
−2
−1
0
1
Config
default
no_cohort
strong_smoothing
weak_smoothing

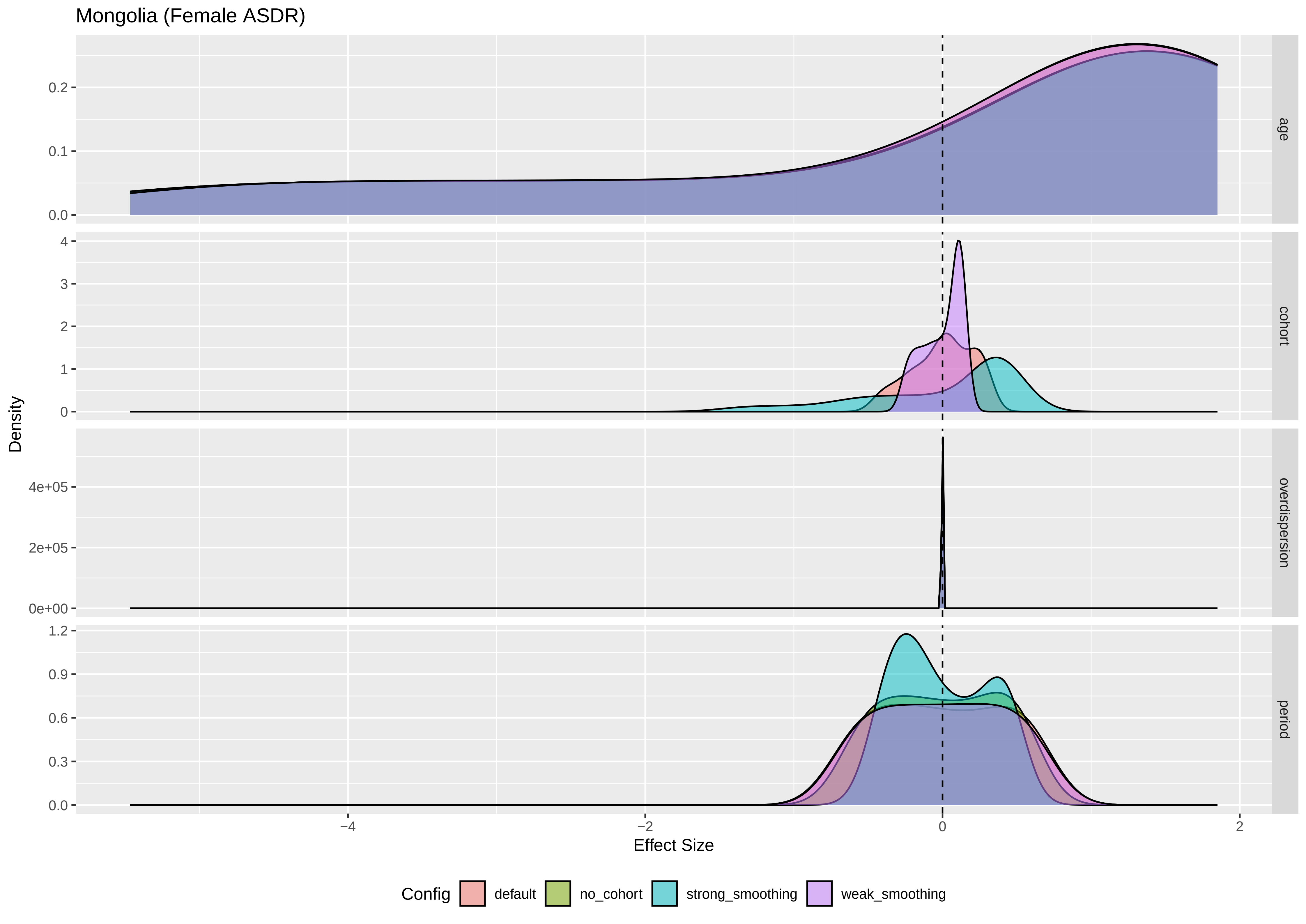
Mongolia (Female ASDR)
Density
Effect Size
age
cohort
overdispersion
period
0.0
0.1
0.2
0
1
2
3
4
0e+00
2e+05
4e+05
0.0
0.3
0.6
0.9
1.2
−4
−2
0
2
Config
default
no_cohort
strong_smoothing
weak_smoothing

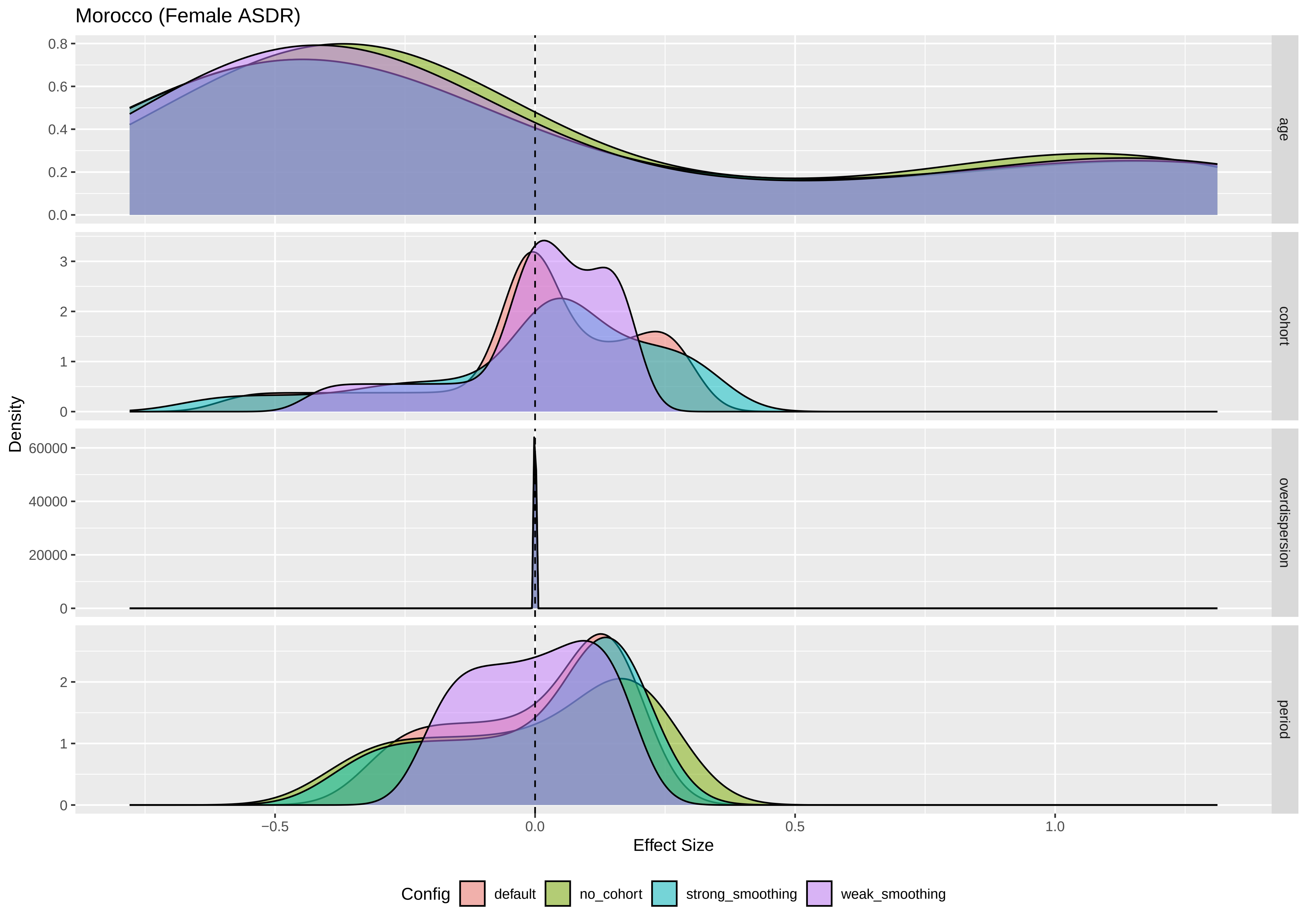
Morocco (Female ASDR)
Density
Effect Size
age
cohort
overdispersion
period
0.8
0.6
0.4
0.2
0.0
3
2
1
0
60000
40000
20000
0
−0.5
0.0
0.5
1.0
Config
default
no_cohort
strong_smoothing
weak_smoothing

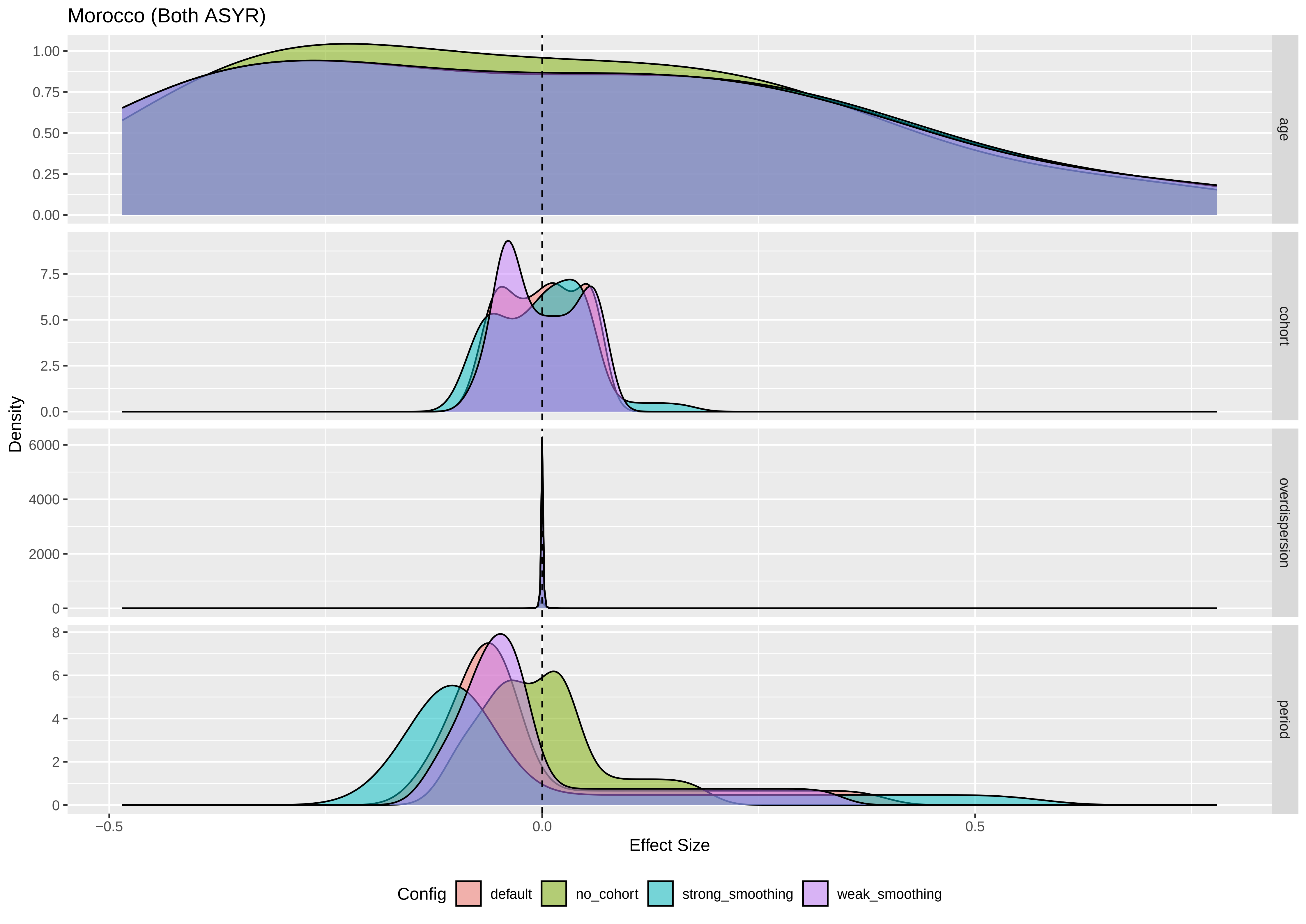
Morocco (Both ASYR)
Density
Effect Size
age
cohort
overdispersion
period
1.00
0.75
0.50
0.25
0.00
7.5
5.0
2.5
0.0
6000
4000
2000
0
8
6
4
2
0
−0.5
0.0
0.5
Config
default
no_cohort
strong_smoothing
weak_smoothing

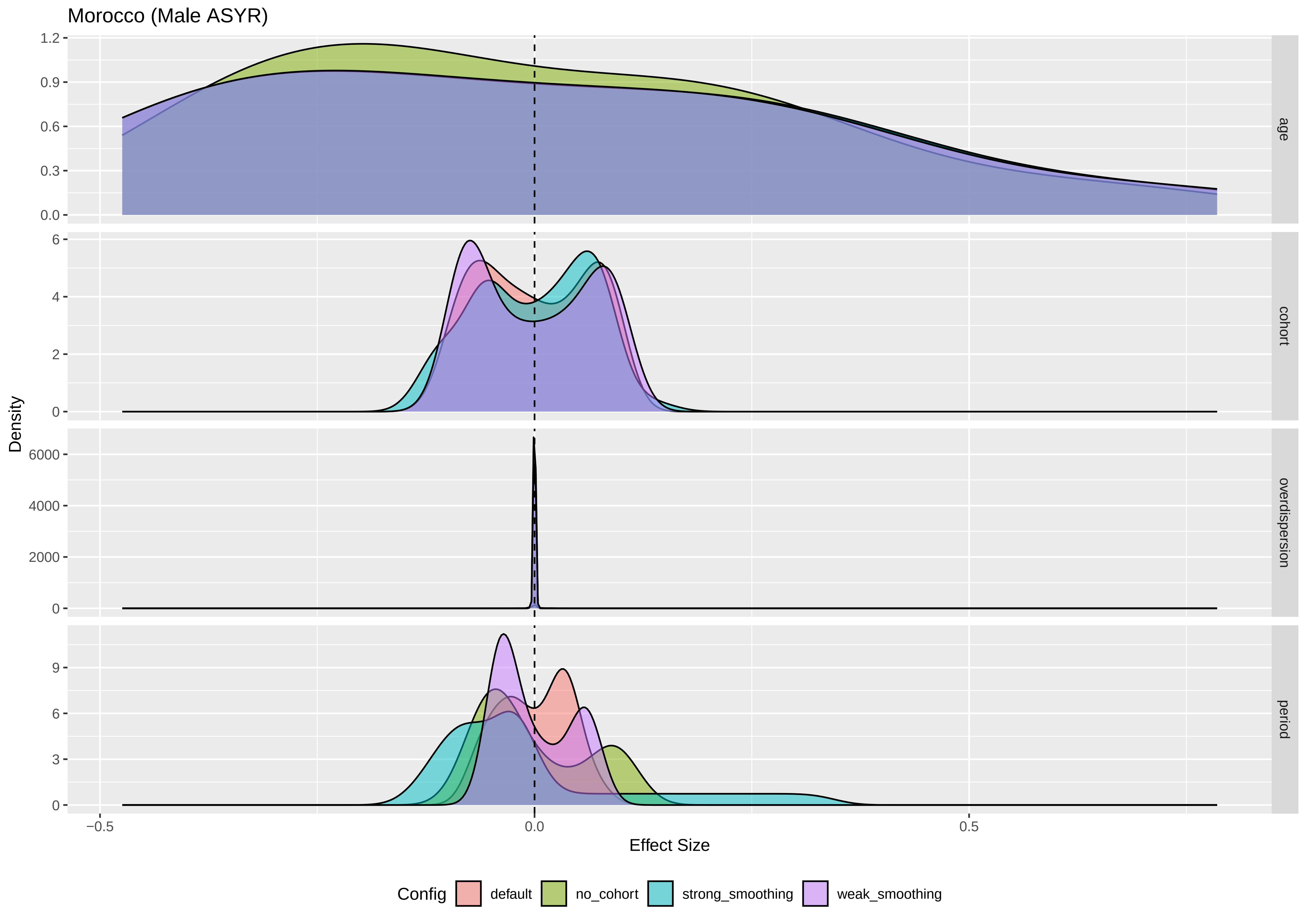

Morocco (Male ASYR)
Density
age
cohort
overdispersion
period
1.2
0.9
0.6
0.3
0.0
6
4
2
0
6000
4000
2000
0
9
6
3
0
−0.5
0.0
0.5
Effect Size
Config
default
no_cohort
strong_smoothing
weak_smoothing

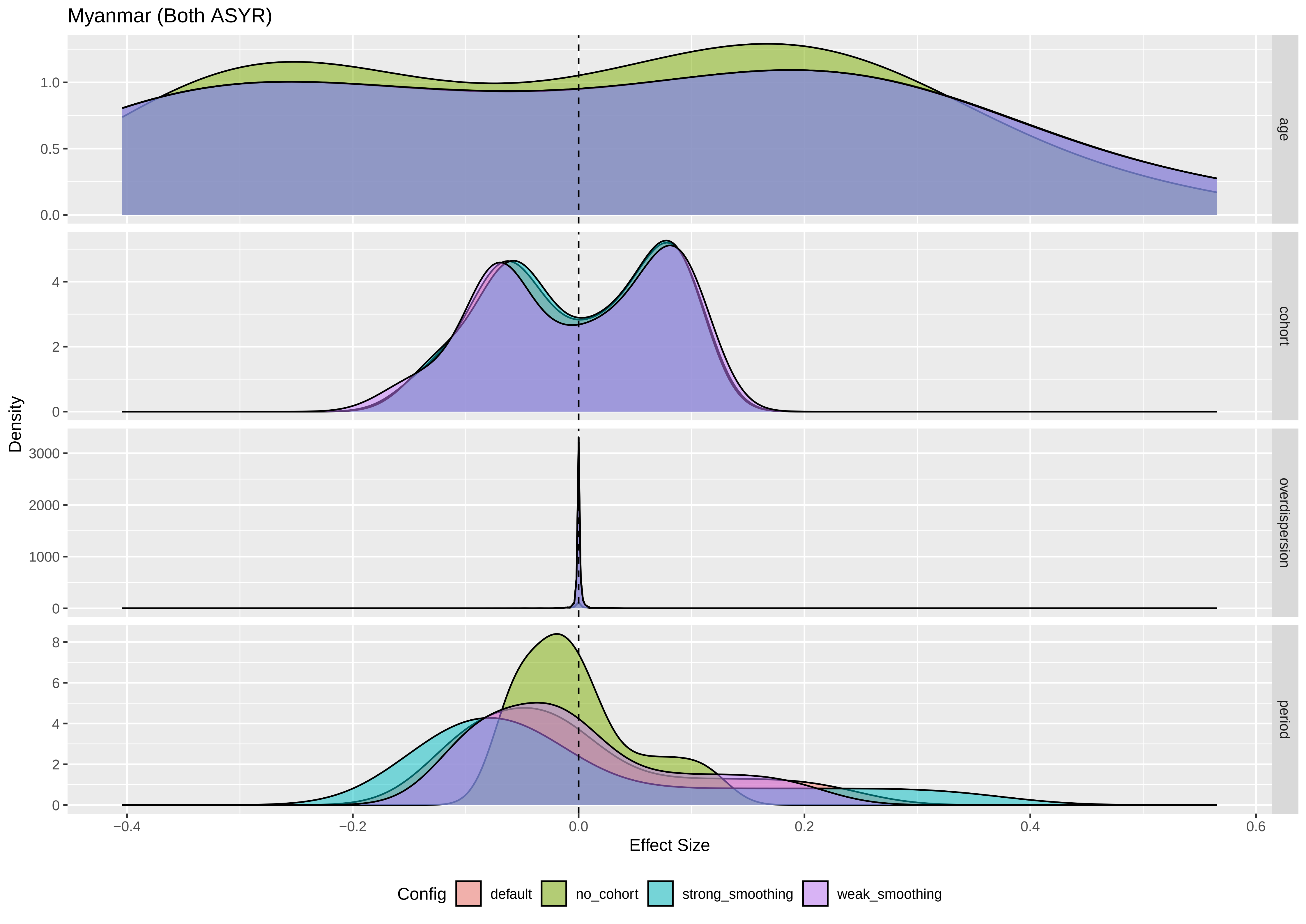

Myanmar (Both ASYR)
age
cohort
overdispersion
period
Density
Effect Size
−0.4
−0.2
0.0
0.2
0.4
0.6
0.0
0.5
1.0
0
2
4
1000
2000
3000
6
8
Config
default
no_cohort
strong_smoothing
weak_smoothing

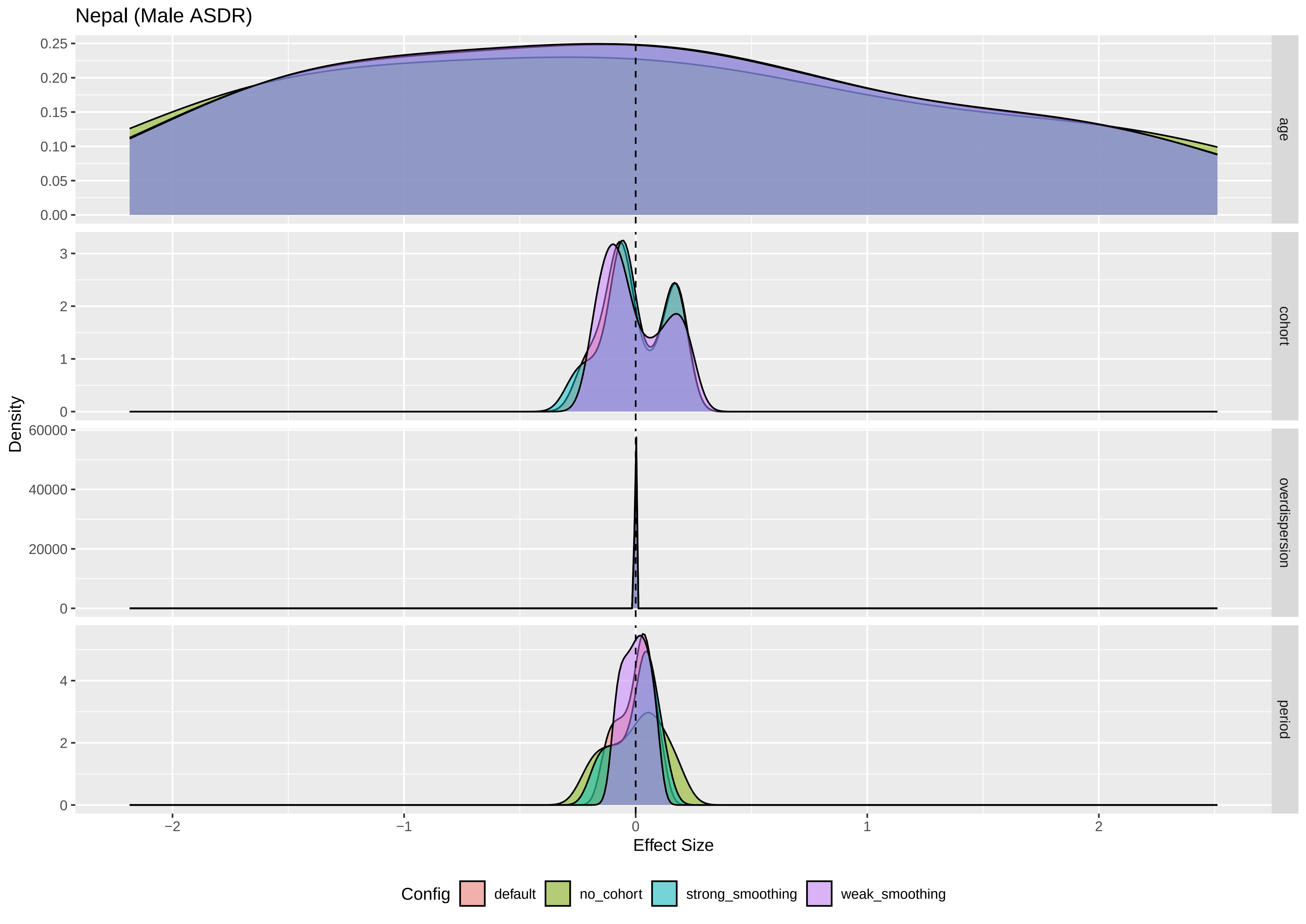

Nepal (Male ASDR)
Density
Effect Size
age
cohort
overdispersion
period
0.25
0.20
0.15
0.10
0.05
0.00
3
2
1
0
60000
40000
20000
0
4
2
0
−2
−1
0
1
2
Config
default
no_cohort
strong_smoothing
weak_smoothing

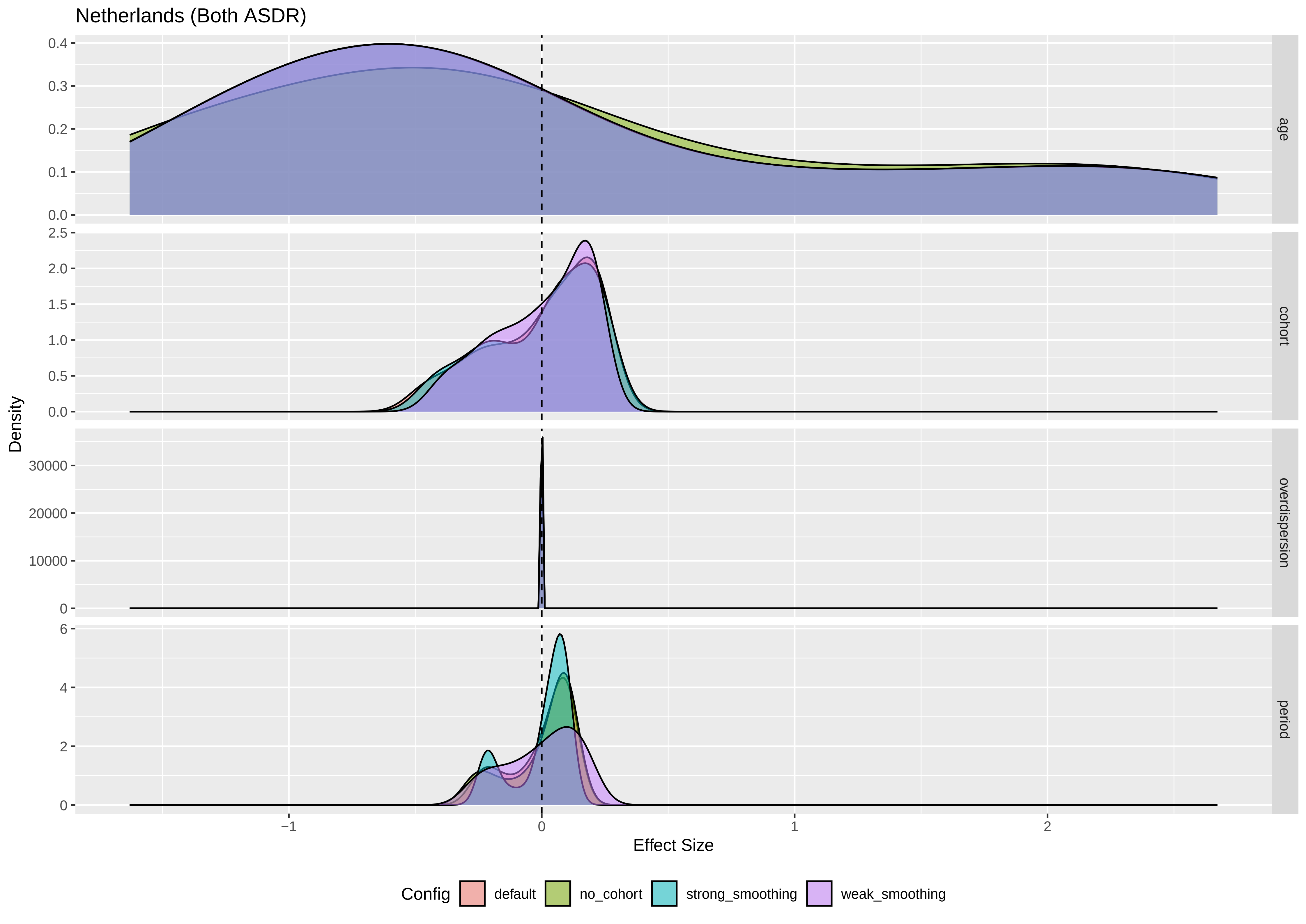

Netherlands (Both ASDR)
age
cohort
overdispersion
period
Density
Effect Size
0.0
0.1
0.2
0.3
0.4
0.5
1.0
1.5
2.0
2.5
0
10000
20000
30000
2
4
6
−1
1
Config
default
no_cohort
strong_smoothing
weak_smoothing

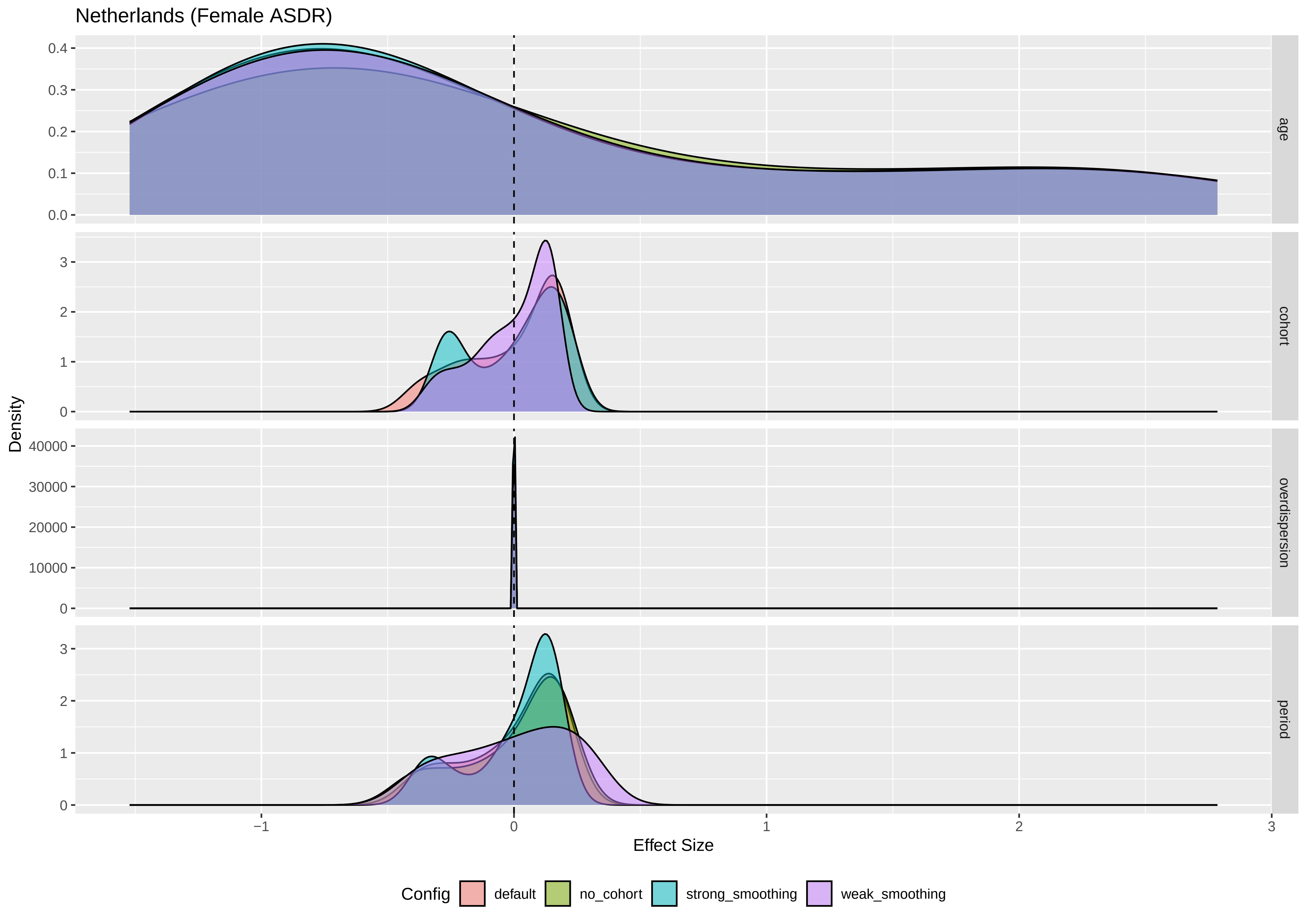

Netherlands (Female ASDR)
0.4
0.3
0.2
0.1
0.0
age
3
2
1
0
cohort
40000
30000
20000
10000
0
overdispersion
3
2
1
0
period
Density
−1
0
1
2
3
Effect Size
Config
default
no_cohort
strong_smoothing
weak_smoothing

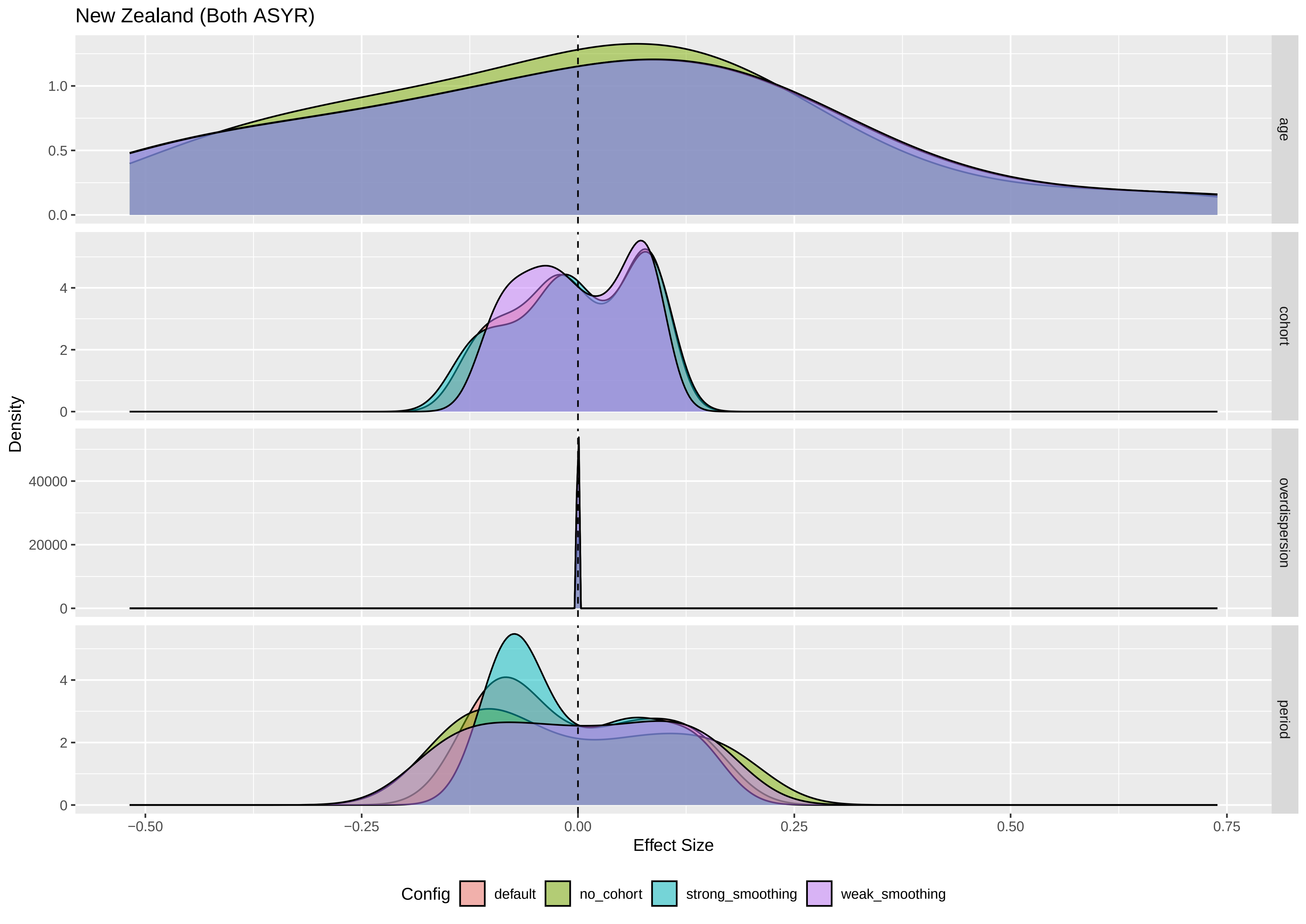
New Zealand (Both ASYR)
age
cohort
overdispersion
period
Density
Effect Size
1.0
0.5
0.0
4
2
0
40000
20000
−0.50
−0.25
0.00
0.25
0.50
0.75
Config
default
no_cohort
strong_smoothing
weak_smoothing

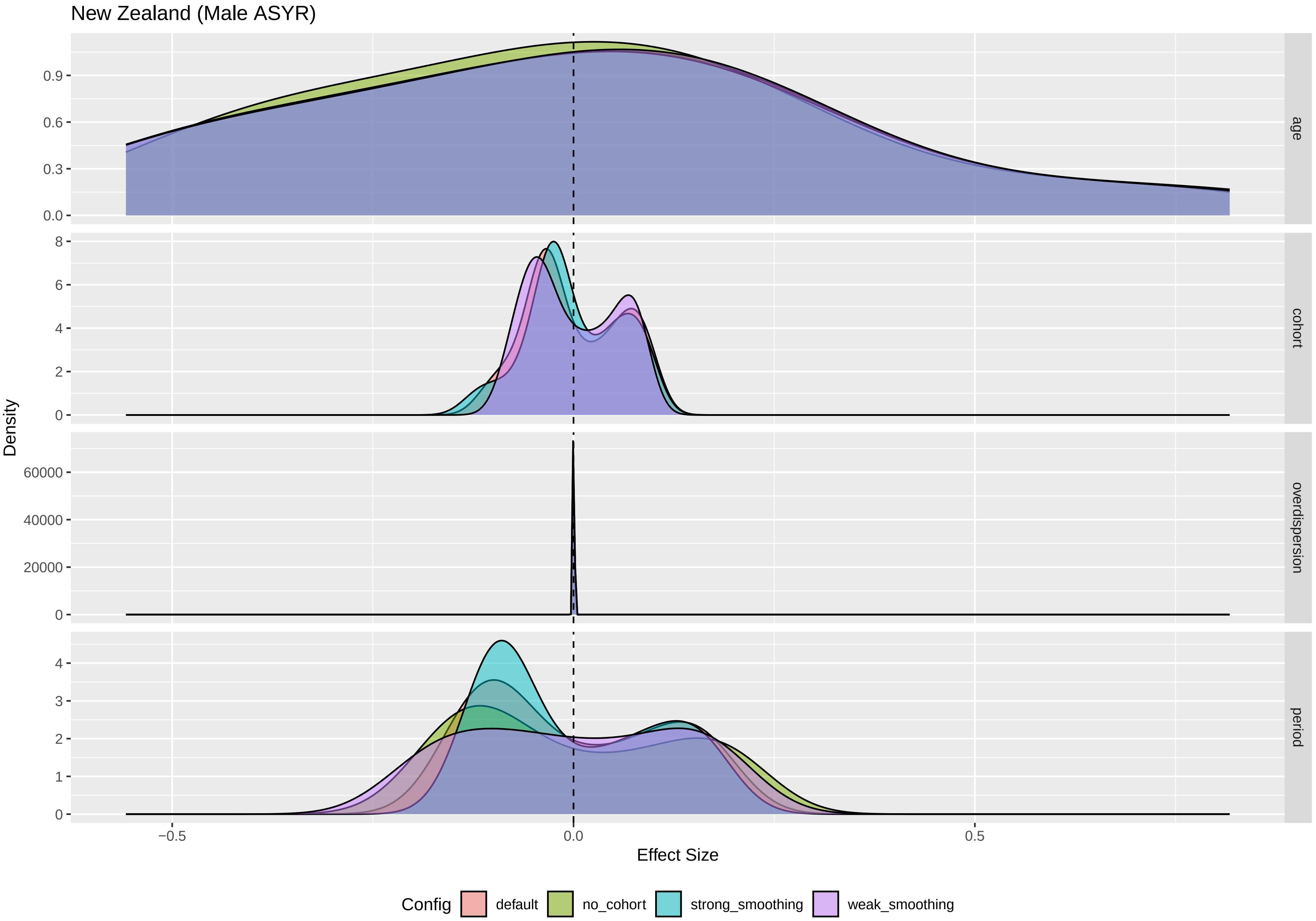

New Zealand (Male ASYR)
age
cohort
overdispersion
period
Density
Effect Size
0.0
0.3
0.6
0.9
0
2
4
6
8
0
20000
40000
60000
0
1
2
3
4
−0.5
0.0
0.5
Config
default
no_cohort
strong_smoothing
weak_smoothing

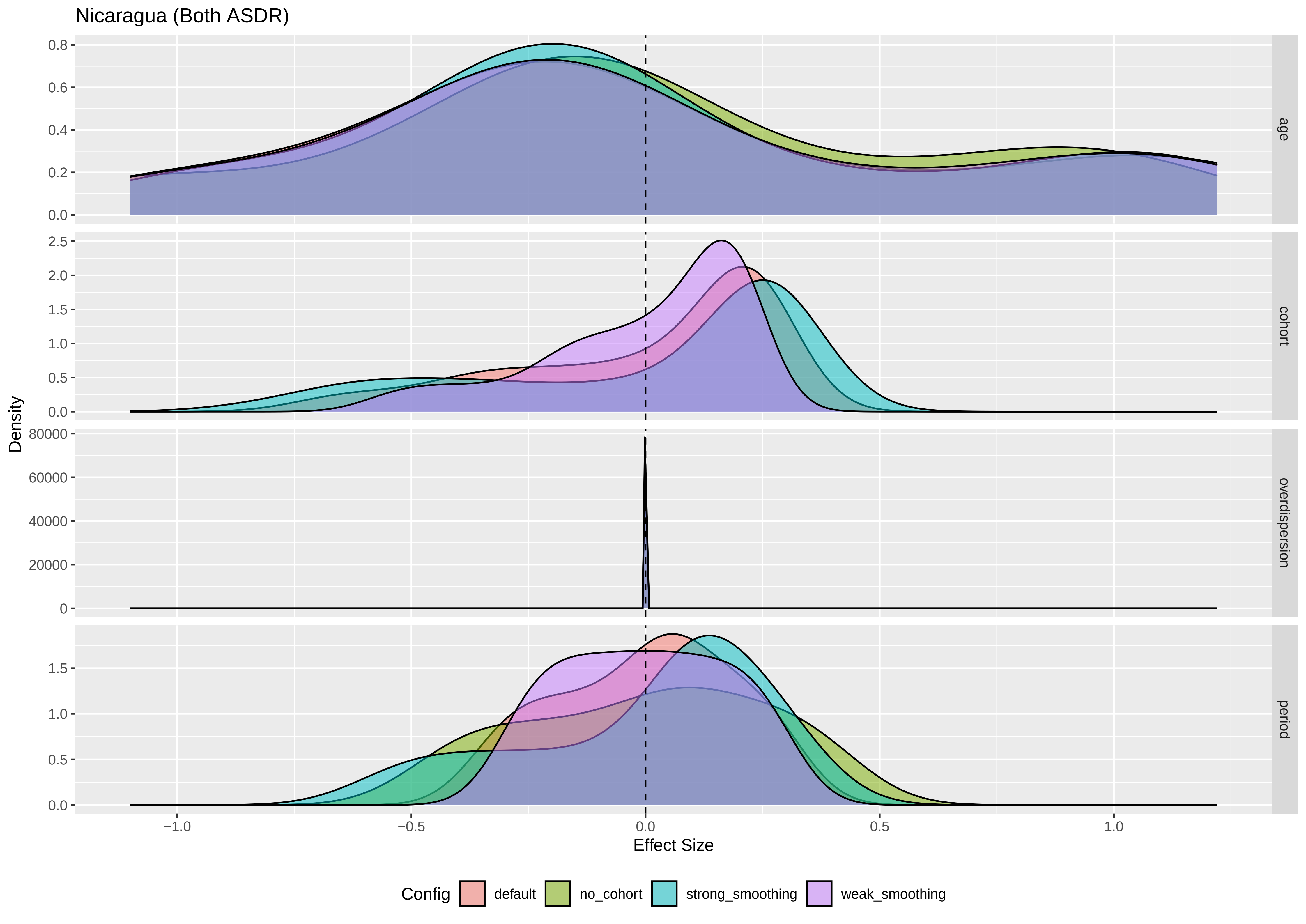

Nicaragua (Both ASDR)
Density
Effect Size
age
cohort
overdispersion
period
0.0
0.2
0.4
0.6
0.8
0.0
0.5
1.0
1.5
2.0
2.5
0
20000
40000
60000
80000
0.0
0.5
1.0
1.5
−1.0
−0.5
0.0
0.5
1.0
Config
default
no_cohort
strong_smoothing
weak_smoothing

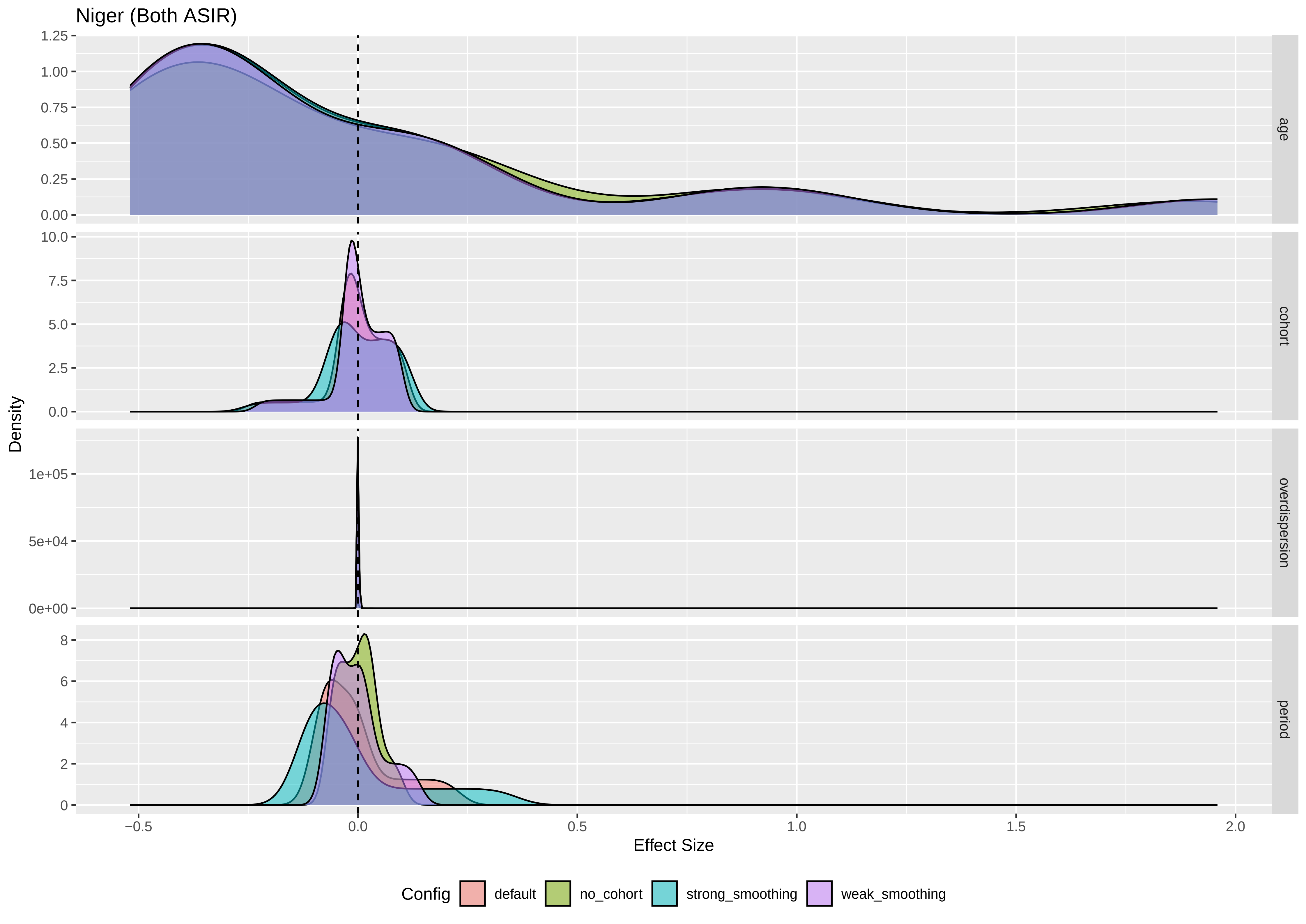

Niger (Both ASIR)
1.25
1.00
0.75
0.50
0.25
0.00
age
10.0
7.5
5.0
2.5
0.0
cohort
Density
1e+05
5e+04
0e+00
overdispersion
8
6
4
2
0
period
−0.5
0.0
0.5
1.0
1.5
2.0
Effect Size
Config
default
no_cohort
strong_smoothing
weak_smoothing

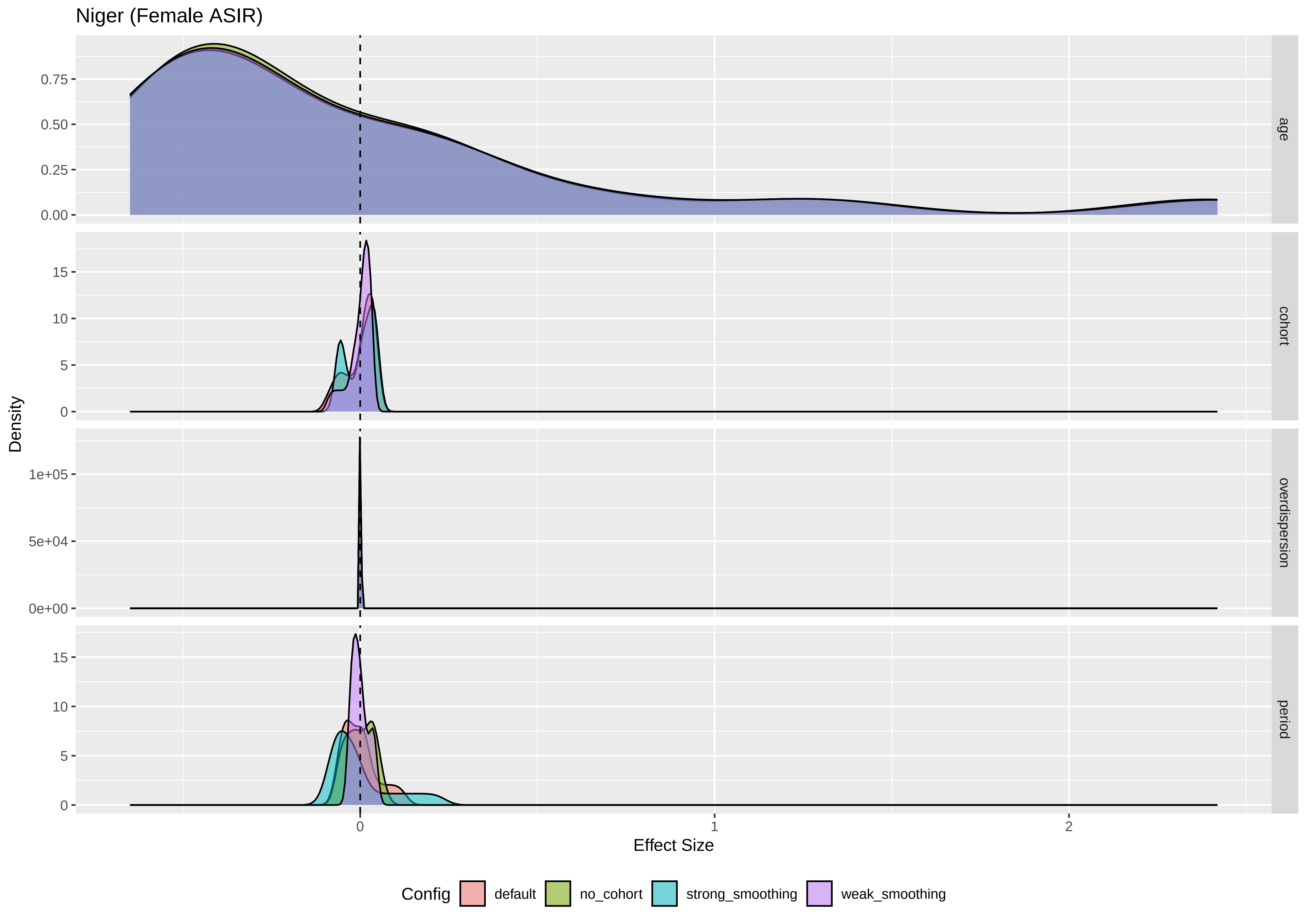

Niger (Female ASIR)
age
cohort
overdispersion
period
Density
0.75
0.50
0.25
0.00
15
10
5
0
1e+05
5e+04
0e+00
0
1
2
Effect Size
Config
default
no_cohort
strong_smoothing
weak_smoothing

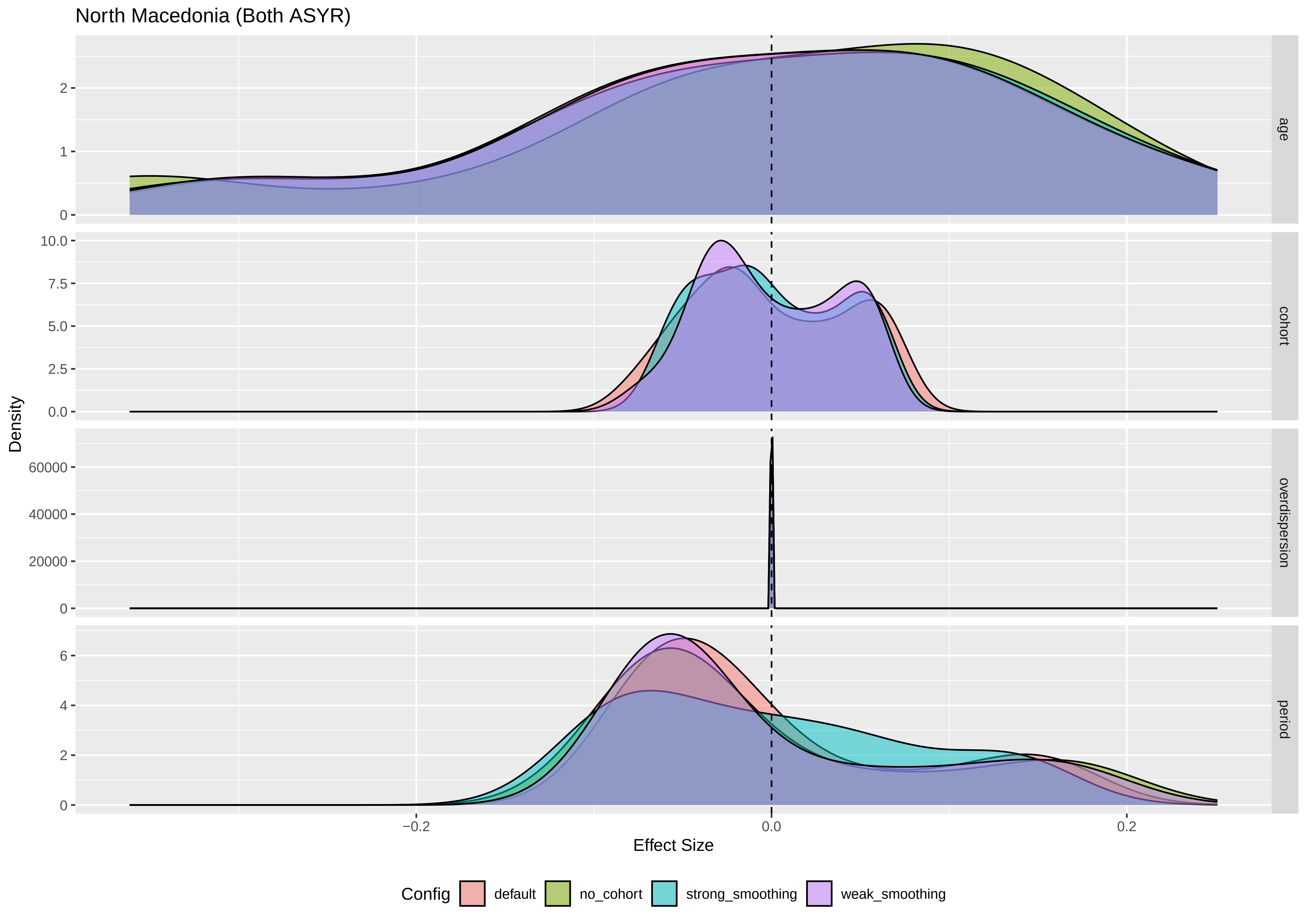

North Macedonia (Both ASYR)
Density
Effect Size
age
cohort
overdispersion
period
0
1
2
0.0
2.5
5.0
7.5
10.0
0
20000
40000
60000
0
2
4
6
−0.2
0.0
0.2
Config
default
no_cohort
strong_smoothing
weak_smoothing

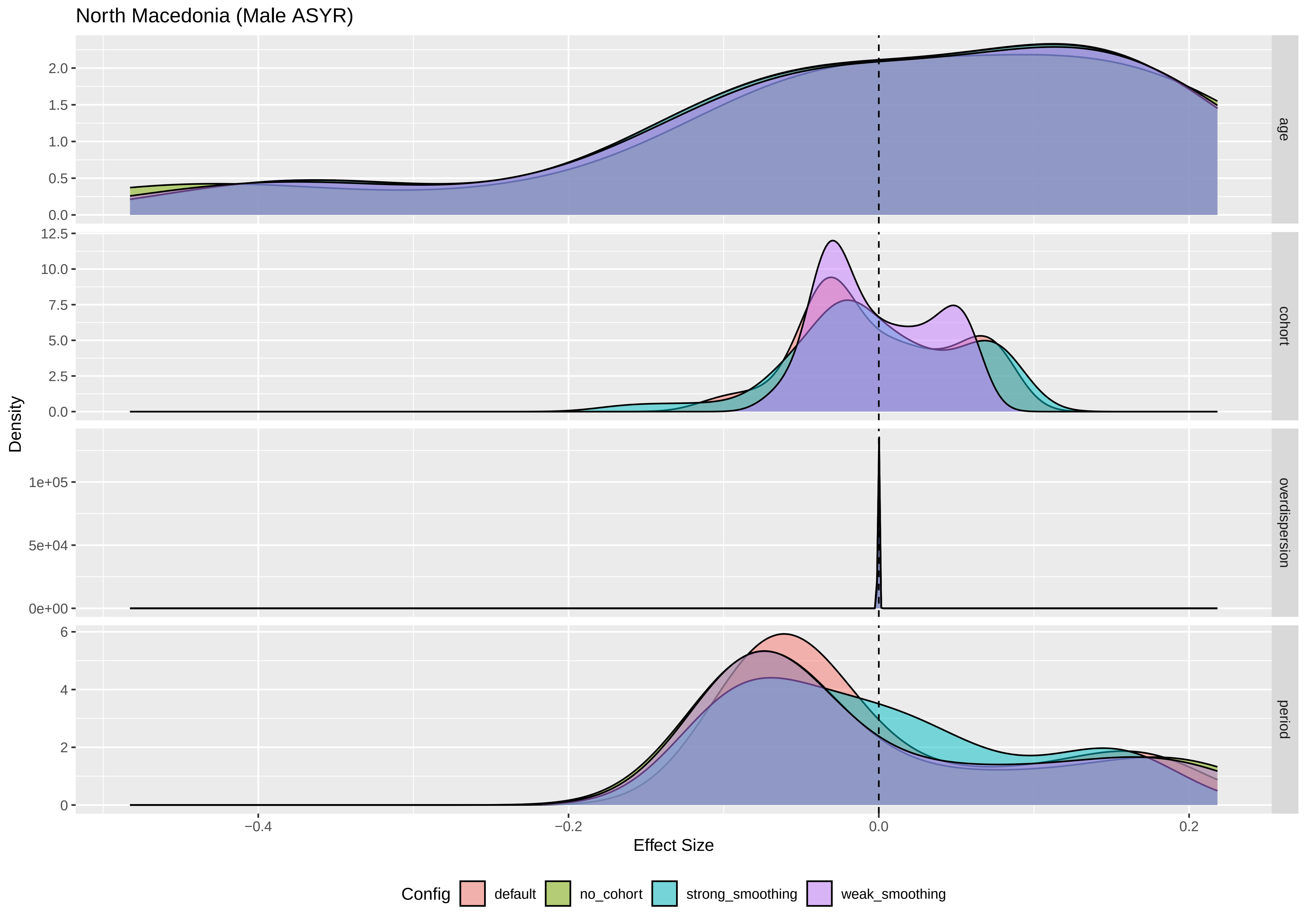

North Macedonia (Male ASYR)
age
cohort
overdispersion
period
Density
Effect Size
2.0
1.5
1.0
0.5
0.0
12.5
10.0
7.5
5.0
2.5
0.0
1e+05
5e+04
0e+00
6
4
2
0
−0.4
−0.2
0.0
0.2
Config
default
no_cohort
strong_smoothing
weak_smoothing

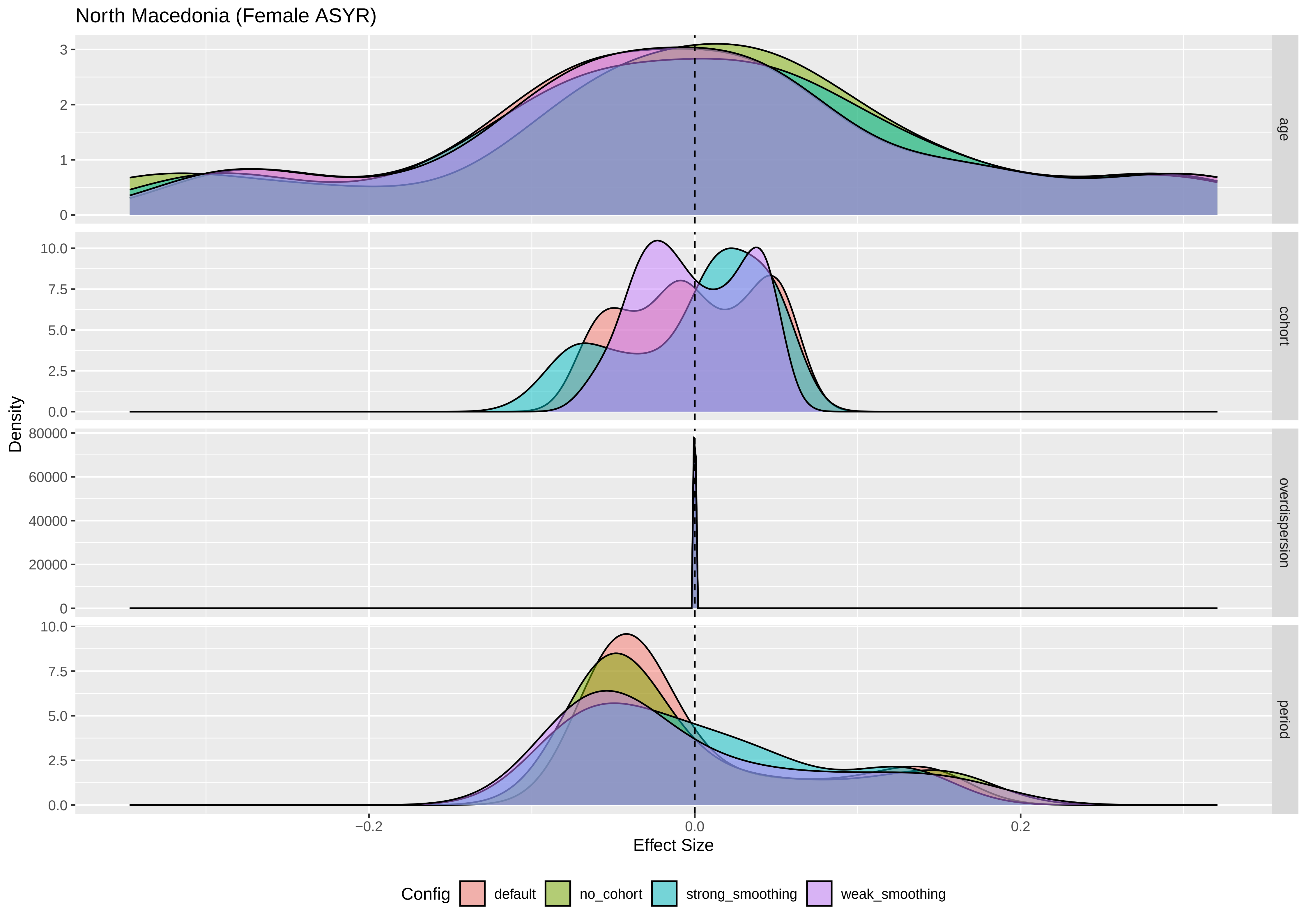
North Macedonia (Female ASYR)
Density
Effect Size
age
cohort
overdispersion
period
3
2
1
0
10.0
7.5
5.0
2.5
0.0
80000
60000
40000
20000
0
−0.2
0.0
0.2
Config
default
no_cohort
strong_smoothing
weak_smoothing

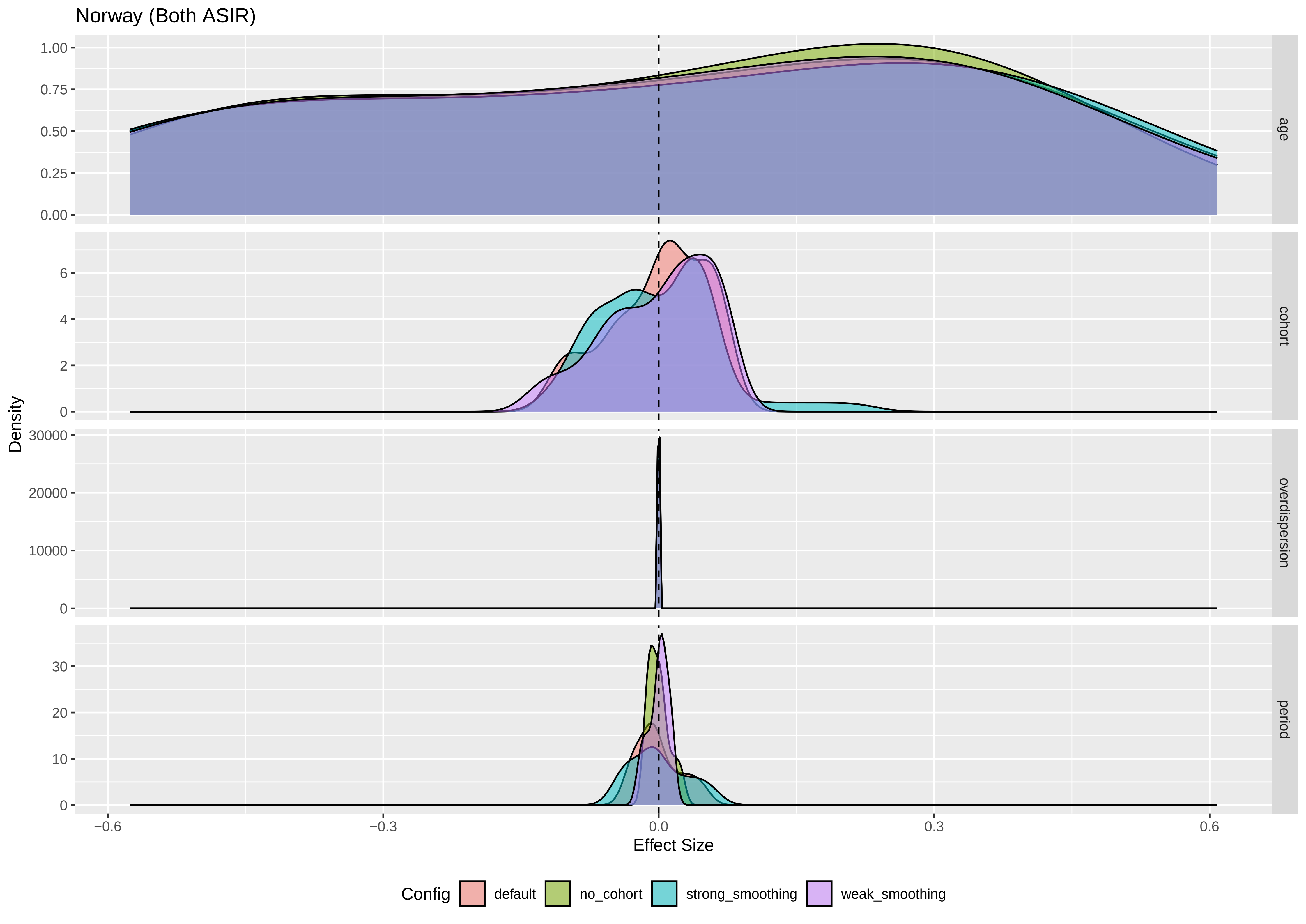

Norway (Both ASIR)
Density
Effect Size
age
cohort
overdispersion
period
1.00
0.75
0.50
0.25
0.00
6
4
2
0
30000
20000
10000
0
30
20
10
0
−0.6
−0.3
0.0
0.3
0.6
Config
default
no_cohort
strong_smoothing
weak_smoothing

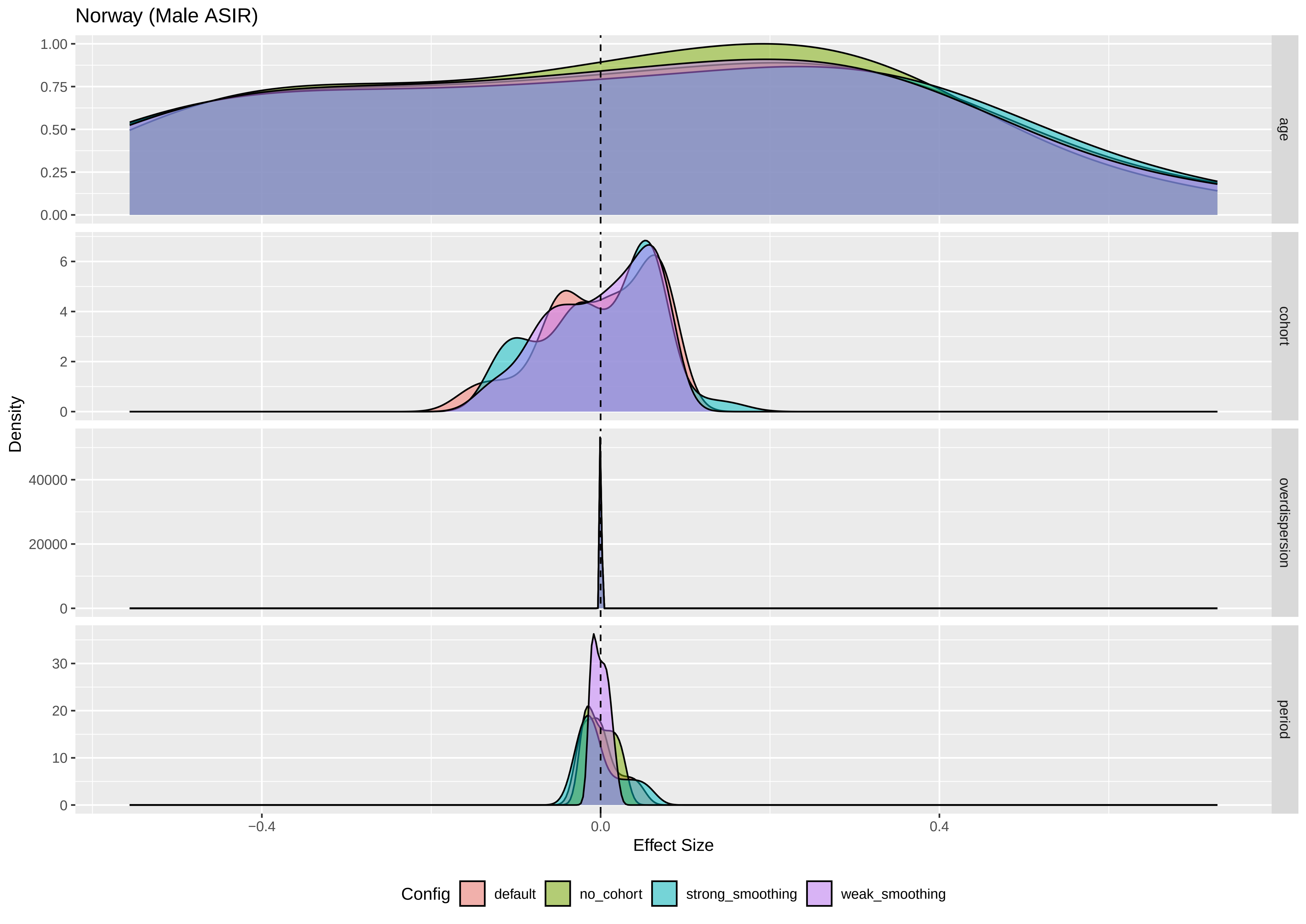

Norway (Male ASIR)
Density
Effect Size
age
cohort
overdispersion
period
1.00
0.75
0.50
0.25
0.00
6
4
2
0
40000
20000
0
30
20
10
0
−0.4
0.0
0.4
Config
default
no_cohort
strong_smoothing
weak_smoothing

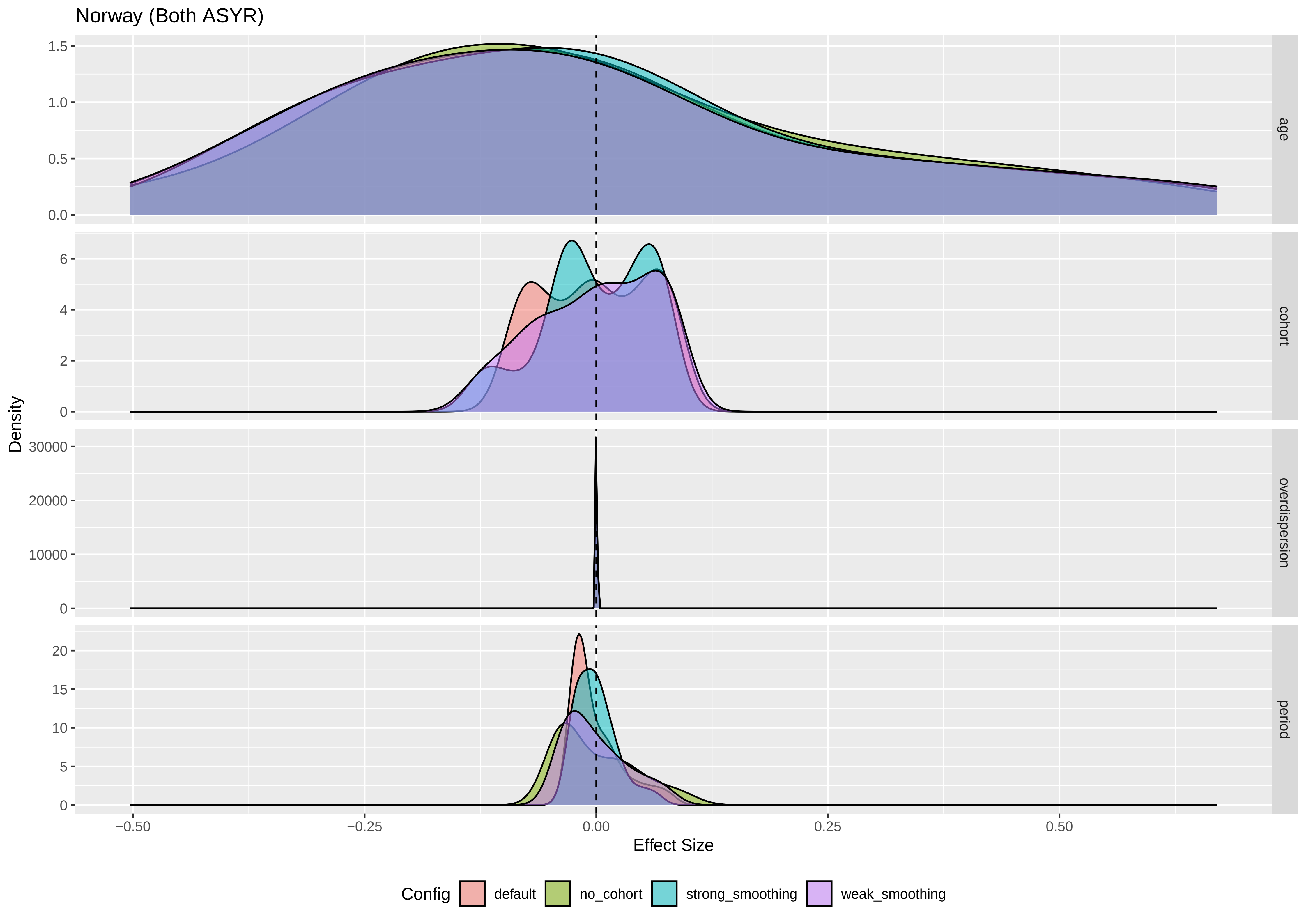

Norway (Both ASYR)
Density
age
cohort
overdispersion
period
1.5
1.0
0.5
0.0
6
4
2
0
30000
20000
10000
0
20
15
10
5
0
−0.50
−0.25
0.00
0.25
0.50
Effect Size
Config
default
no_cohort
strong_smoothing
weak_smoothing

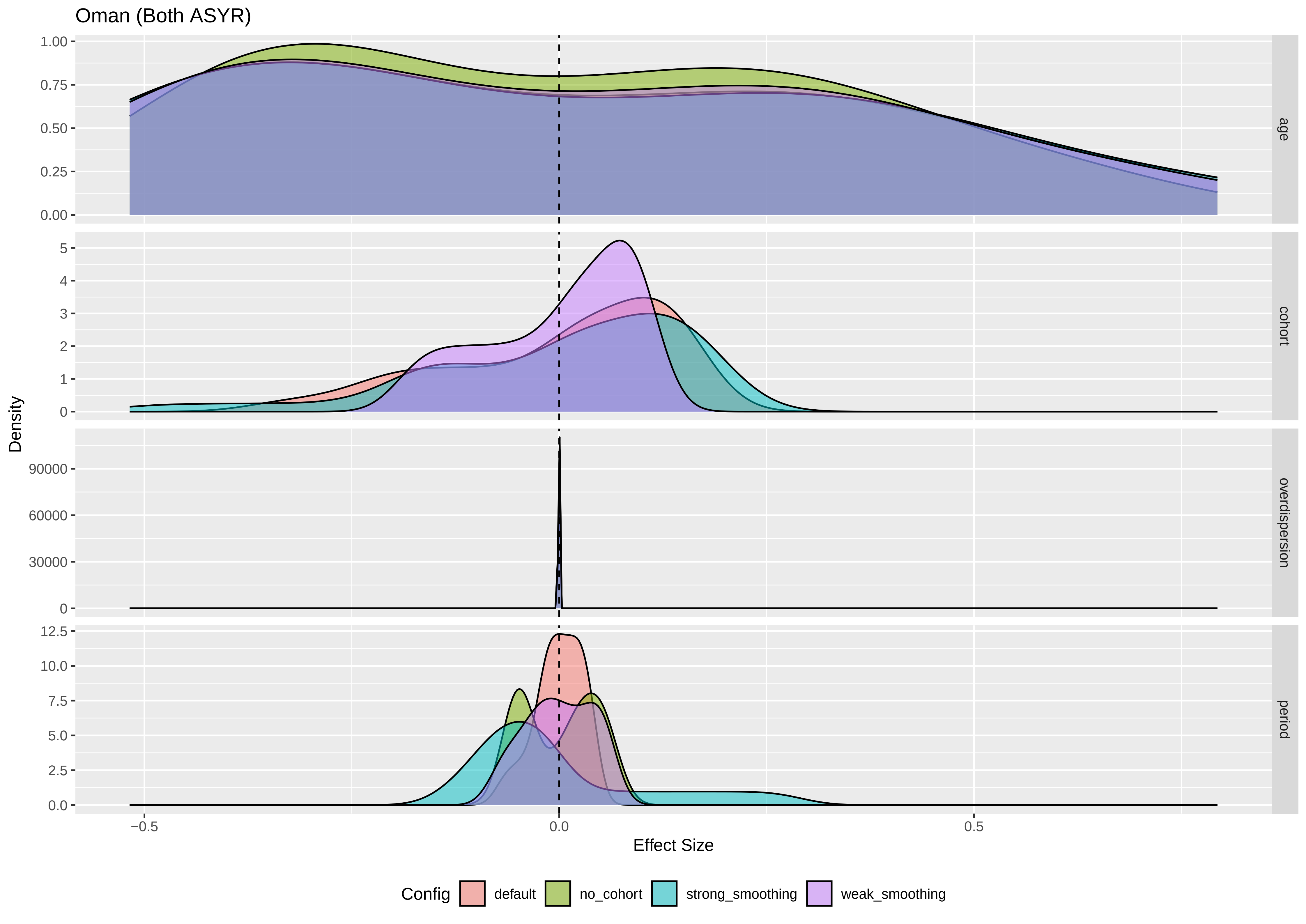

Oman (Both ASYR)
Density
Effect Size
age
cohort
overdispersion
period
1.00
0.75
0.50
0.25
0.00
5
4
3
2
1
0
90000
60000
30000
0
12.5
10.0
7.5
5.0
2.5
0.0
−0.5
0.0
0.5
Config
default
no_cohort
strong_smoothing
weak_smoothing

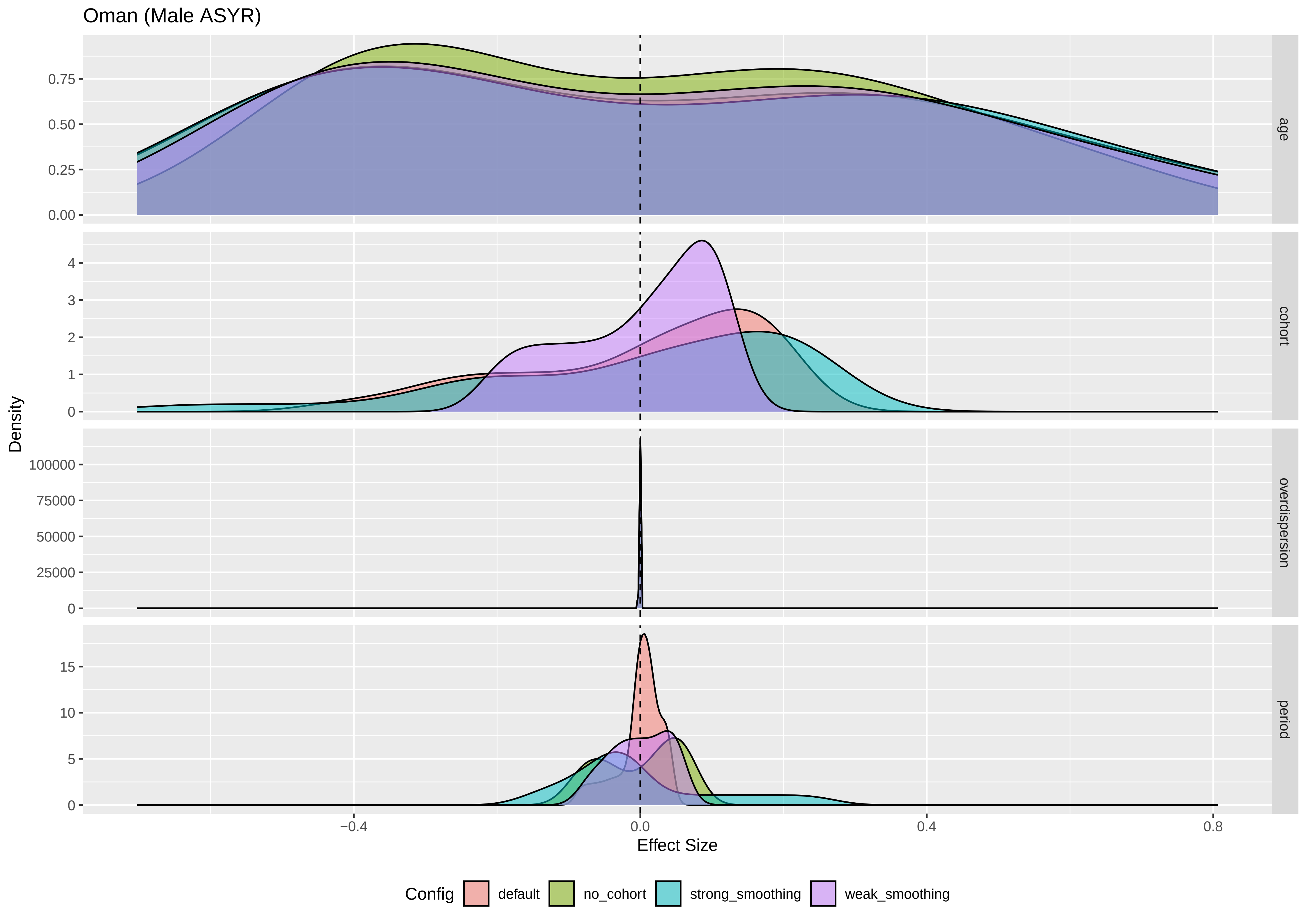

Oman (Male ASYR)
Density
Effect Size
age
cohort
overdispersion
period
0.00
0.25
0.50
0.75
0
1
2
3
4
0
25000
50000
75000
100000
0
5
10
15
-0.4
0.0
0.4
0.8
Config
default
no_cohort
strong_smoothing
weak_smoothing

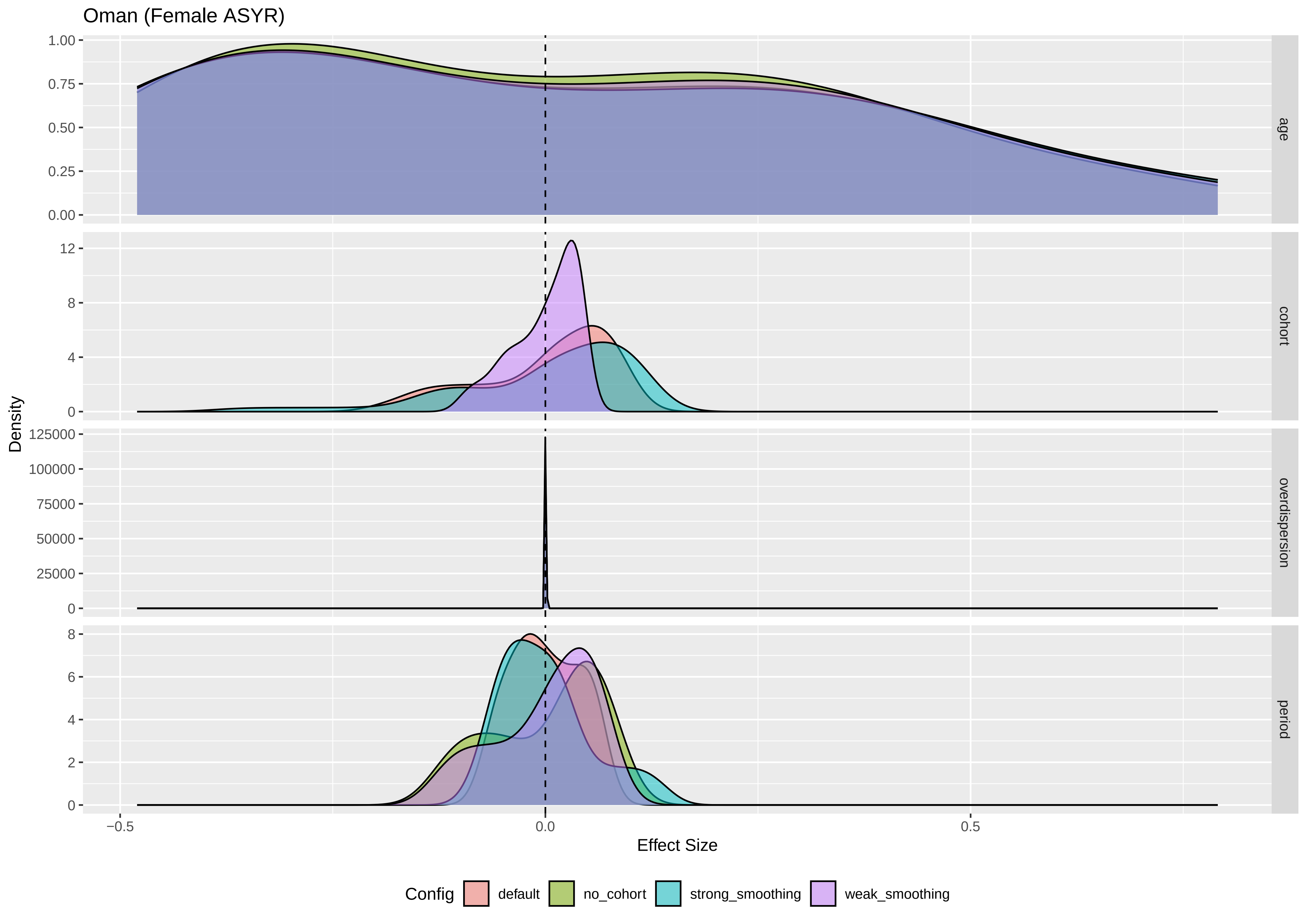

Oman (Female ASYR)
Density
Effect Size
age
cohort
overdispersion
period
1.00
0.75
0.50
0.25
0.00
12
8
4
0
125000
100000
75000
50000
25000
0
8
6
4
2
0
−0.5
0.0
0.5
Config
default
no_cohort
strong_smoothing
weak_smoothing

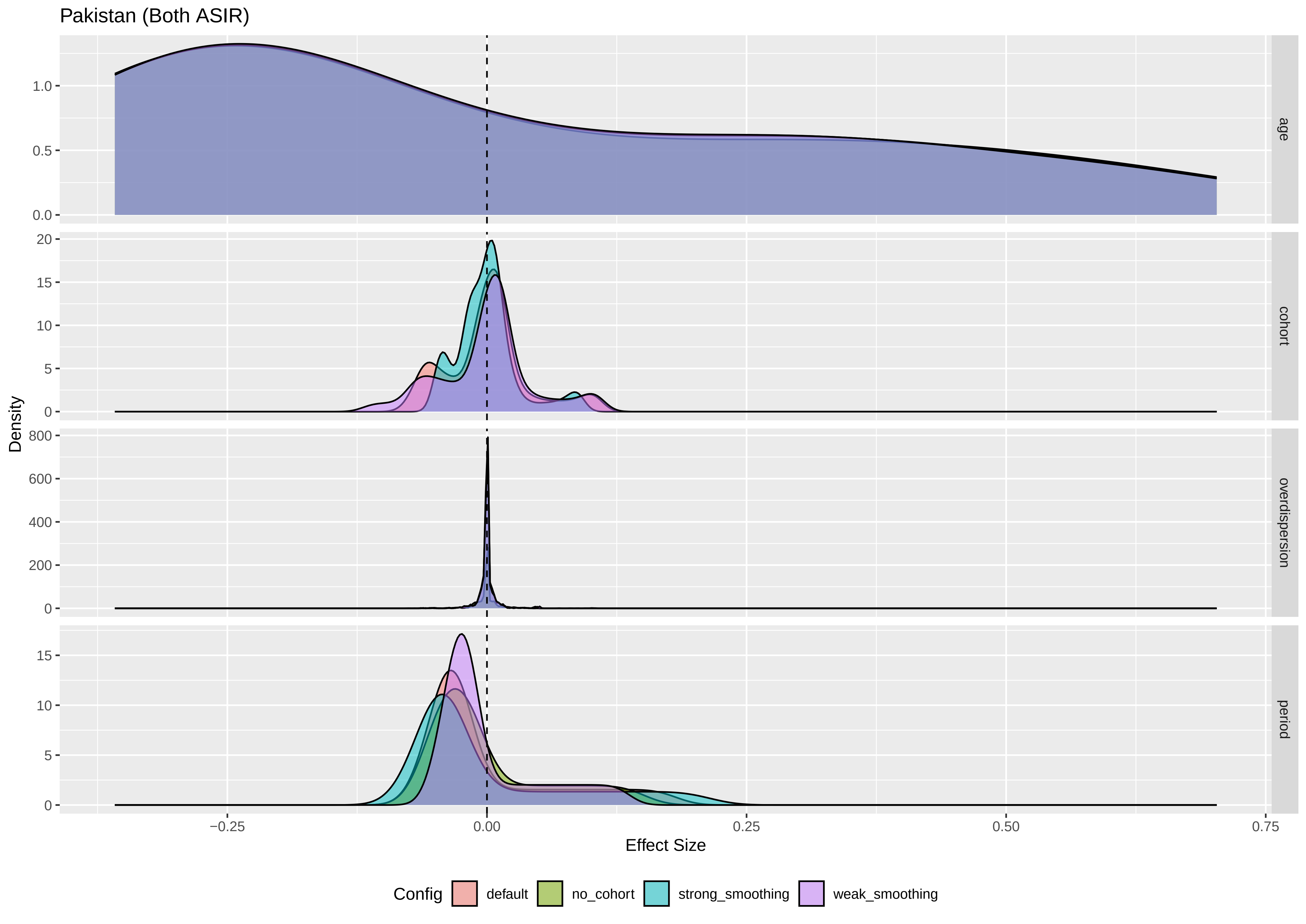

Pakistan (Both ASIR)
age
cohort
overdispersion
period
Density
Effect Size
−0.25
0.00
0.25
0.50
0.75
Config
default
no_cohort
strong_smoothing
weak_smoothing

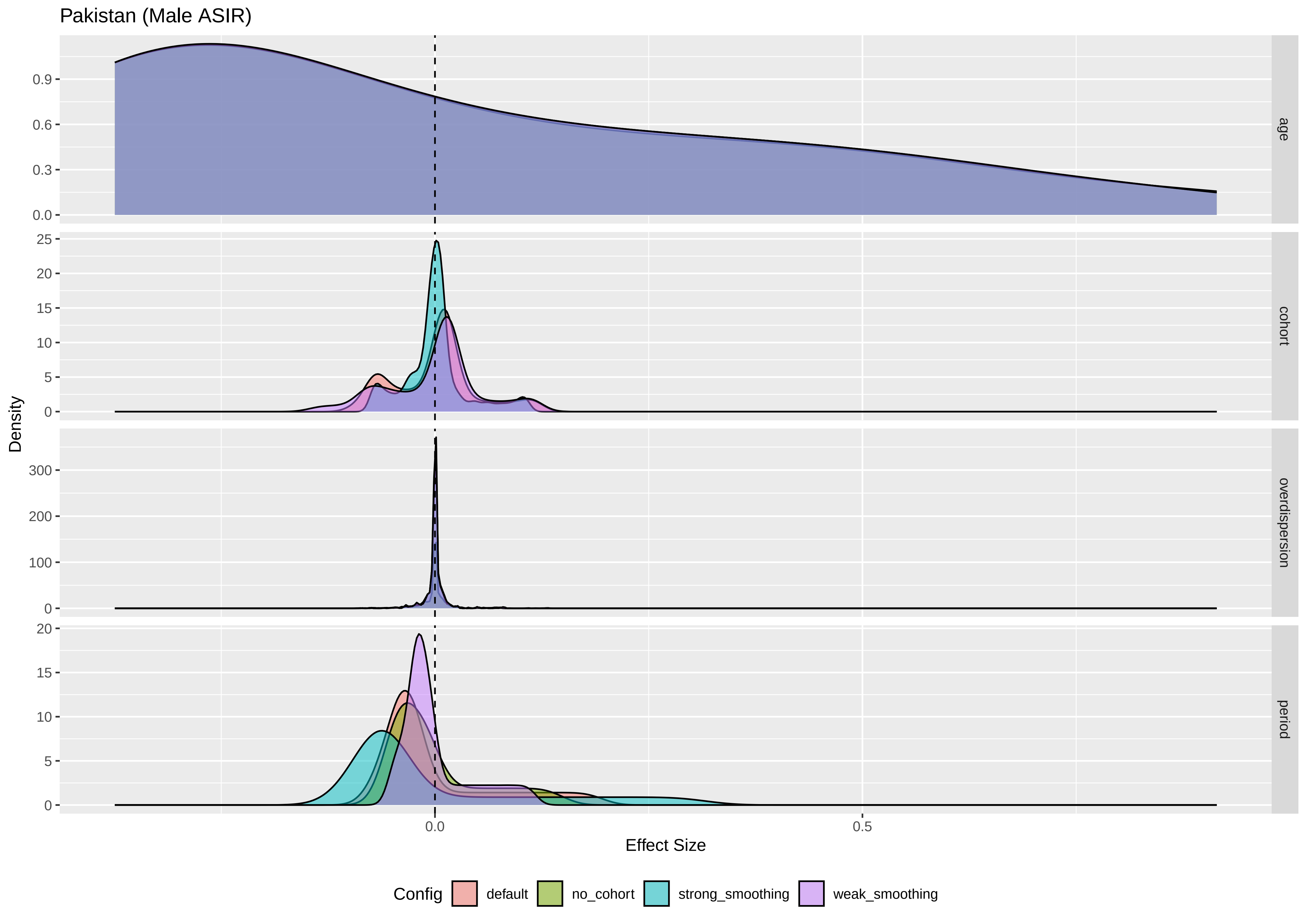

Pakistan (Male ASIR)
age
cohort
overdispersion
period
Density
Effect Size
0.0
0.5
0.0
0.3
0.6
0.9
0
5
10
15
20
25
0
100
200
300
0
5
10
15
20
Config
default
no_cohort
strong_smoothing
weak_smoothing

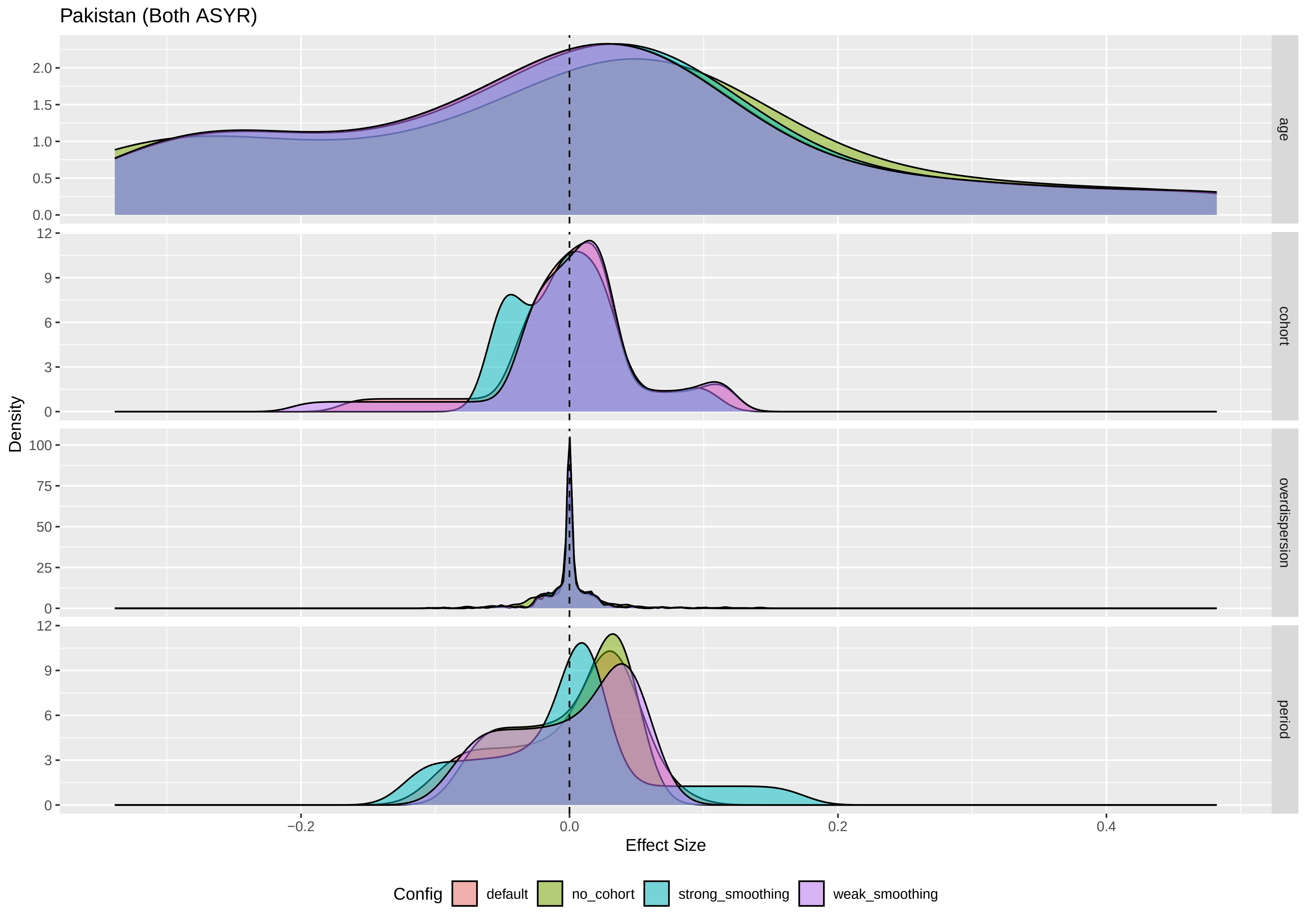

Pakistan (Both ASYR)
age
cohort
overdispersion
period
Density
Effect Size
−0.2
0.0
0.2
0.4
0.0
0.5
1.0
1.5
2.0
0
3
6
9
12
0
25
50
75
100
Config
default
no_cohort
strong_smoothing
weak_smoothing

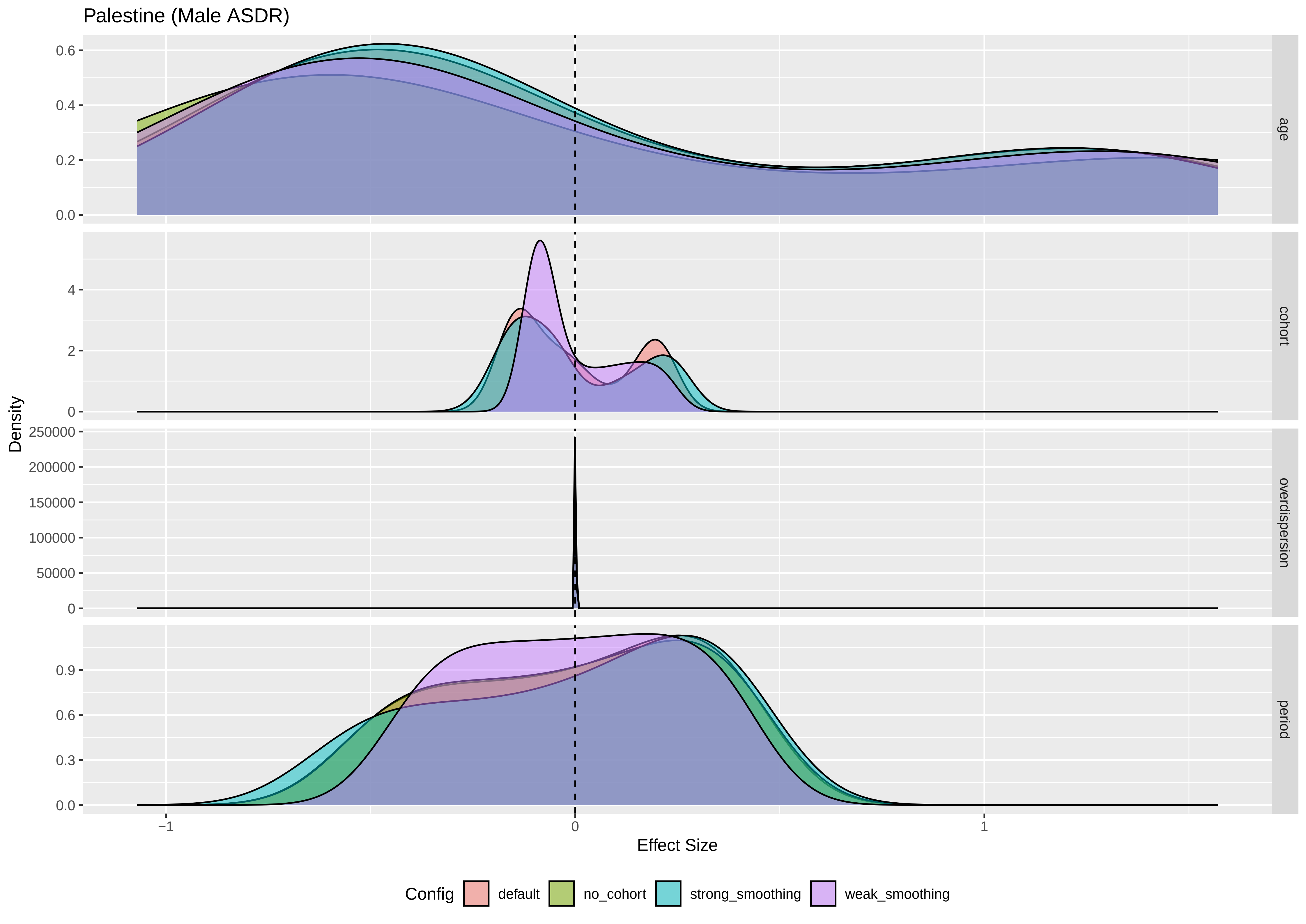
Palestine (Male ASDR)
Density
Effect Size
age
cohort
overdispersion
period
−1
0
1
0.0
0.2
0.4
0.6
0
2
4
250000
200000
150000
100000
50000
0
0.0
0.3
0.6
0.9
Config
default
no_cohort
strong_smoothing
weak_smoothing

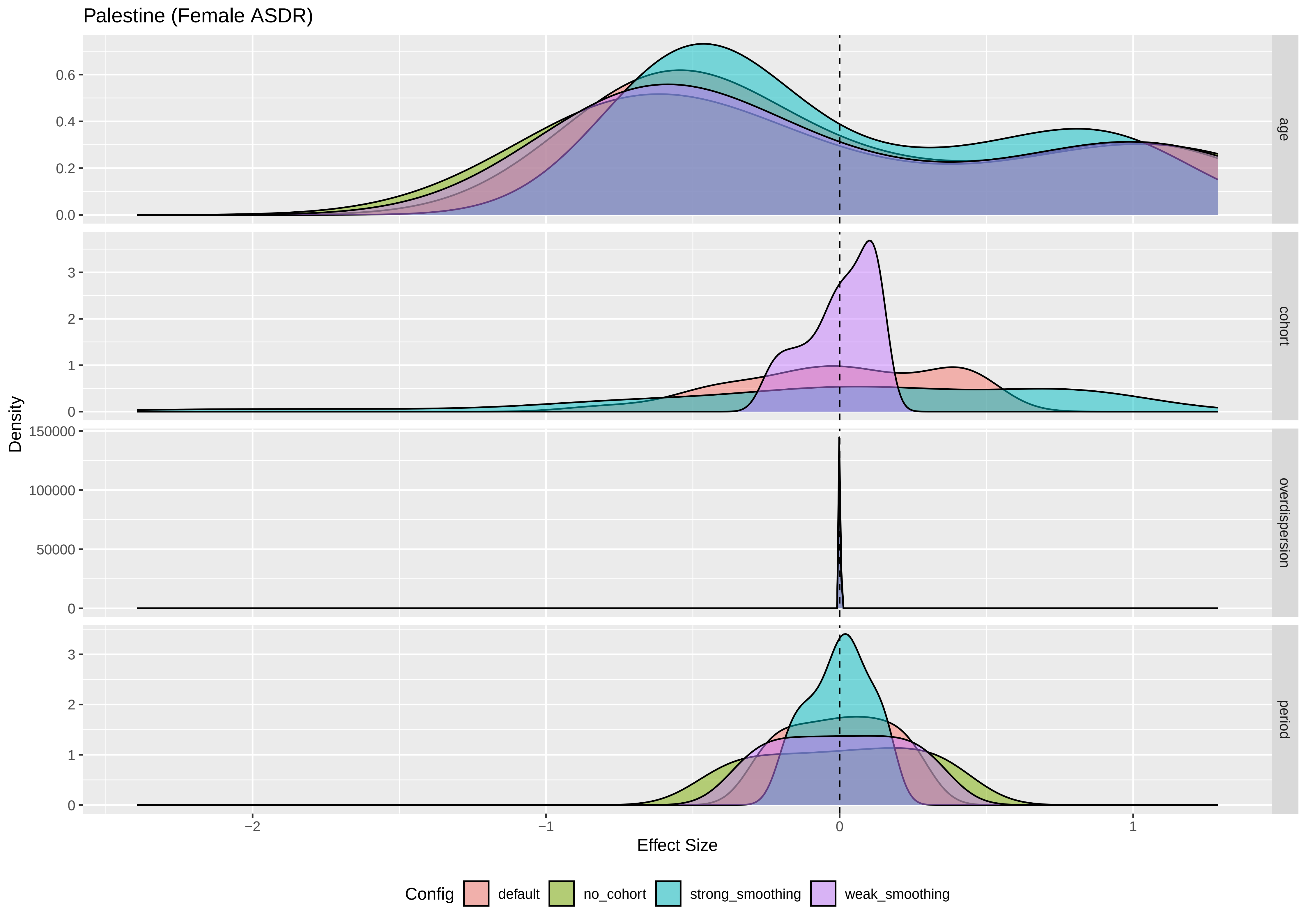

Palestine (Female ASDR)
age
cohort
overdispersion
period
Density
Effect Size
0.0
0.2
0.4
0.6
0
1
2
3
0
50000
100000
150000
−2
−1
0
1
Config
default
no_cohort
strong_smoothing
weak_smoothing

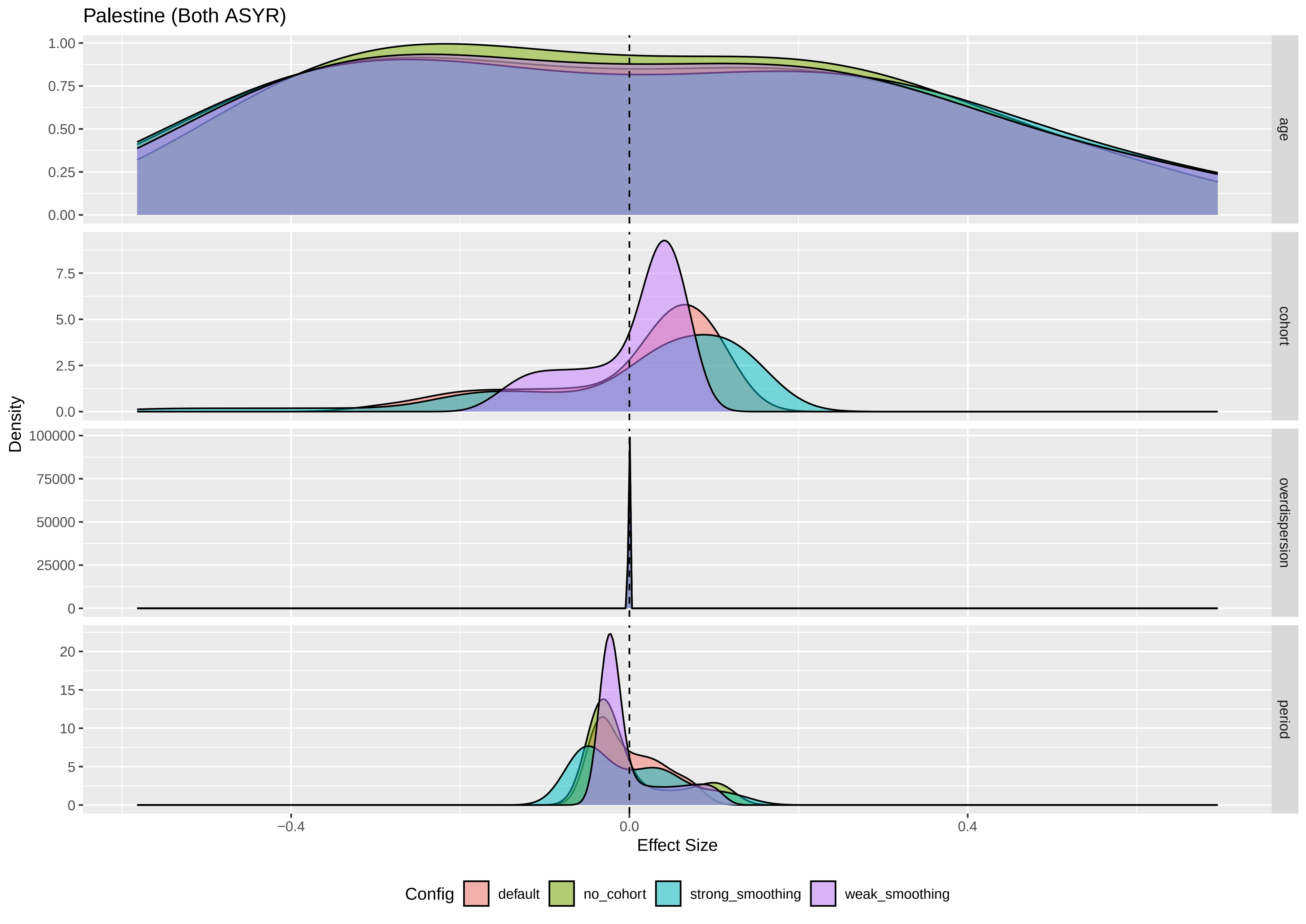

Palestine (Both ASYR)
Density
1.00
0.75
0.50
0.25
0.00
7.5
5.0
2.5
0.0
100000
75000
50000
25000
0
20
15
10
5
0
age
cohort
overdispersion
period
−0.4
0.0
0.4
Effect Size
Config
default
no_cohort
strong_smoothing
weak_smoothing

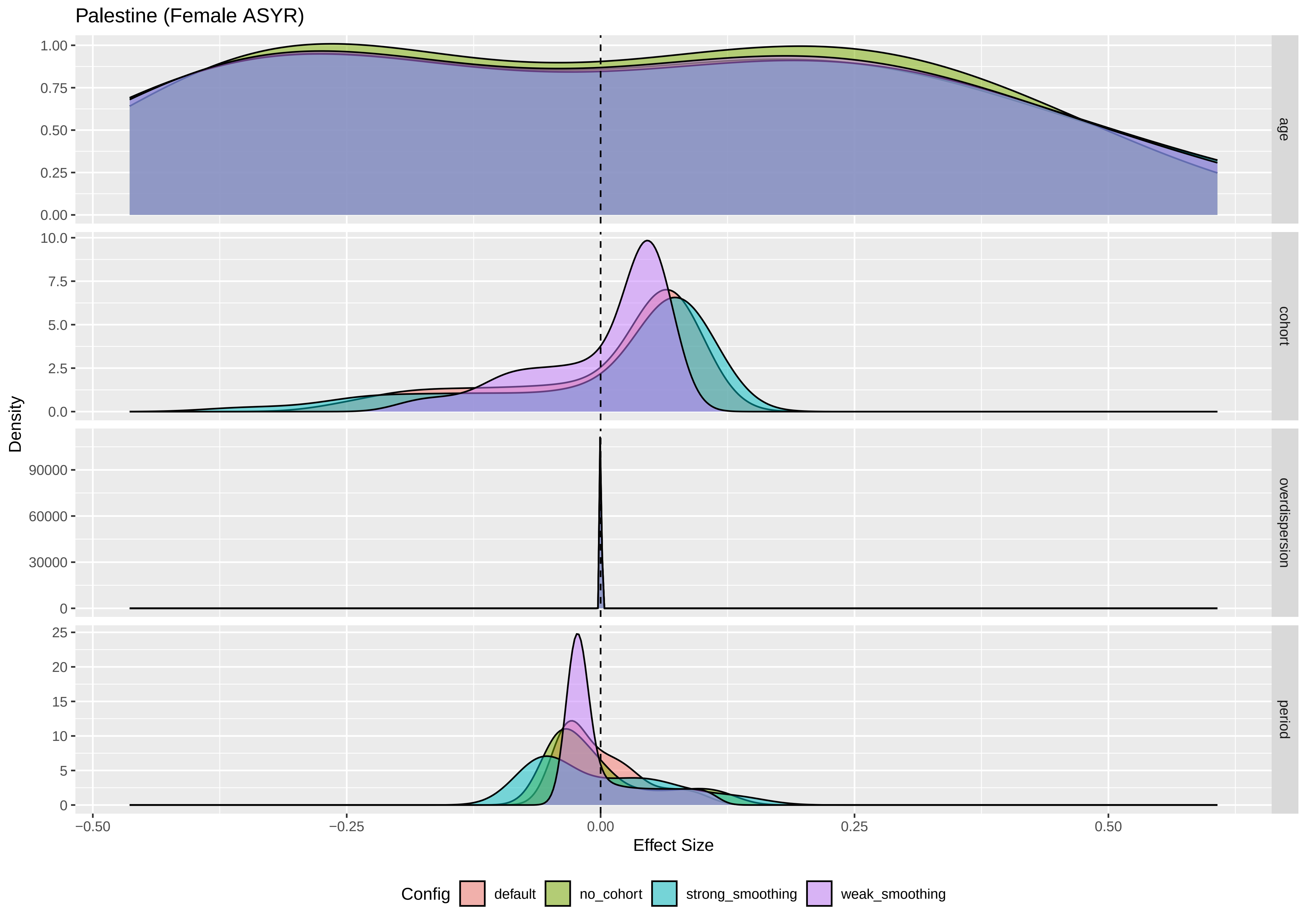

Palestine (Female ASYR)
age
cohort
overdispersion
period
Density
Effect Size
1.00
0.75
0.50
0.25
0.00
10.0
7.5
5.0
2.5
0.0
90000
60000
30000
0
25
20
15
10
5
0
−0.50
−0.25
0.00
0.25
0.50
Config
default
no_cohort
strong_smoothing
weak_smoothing

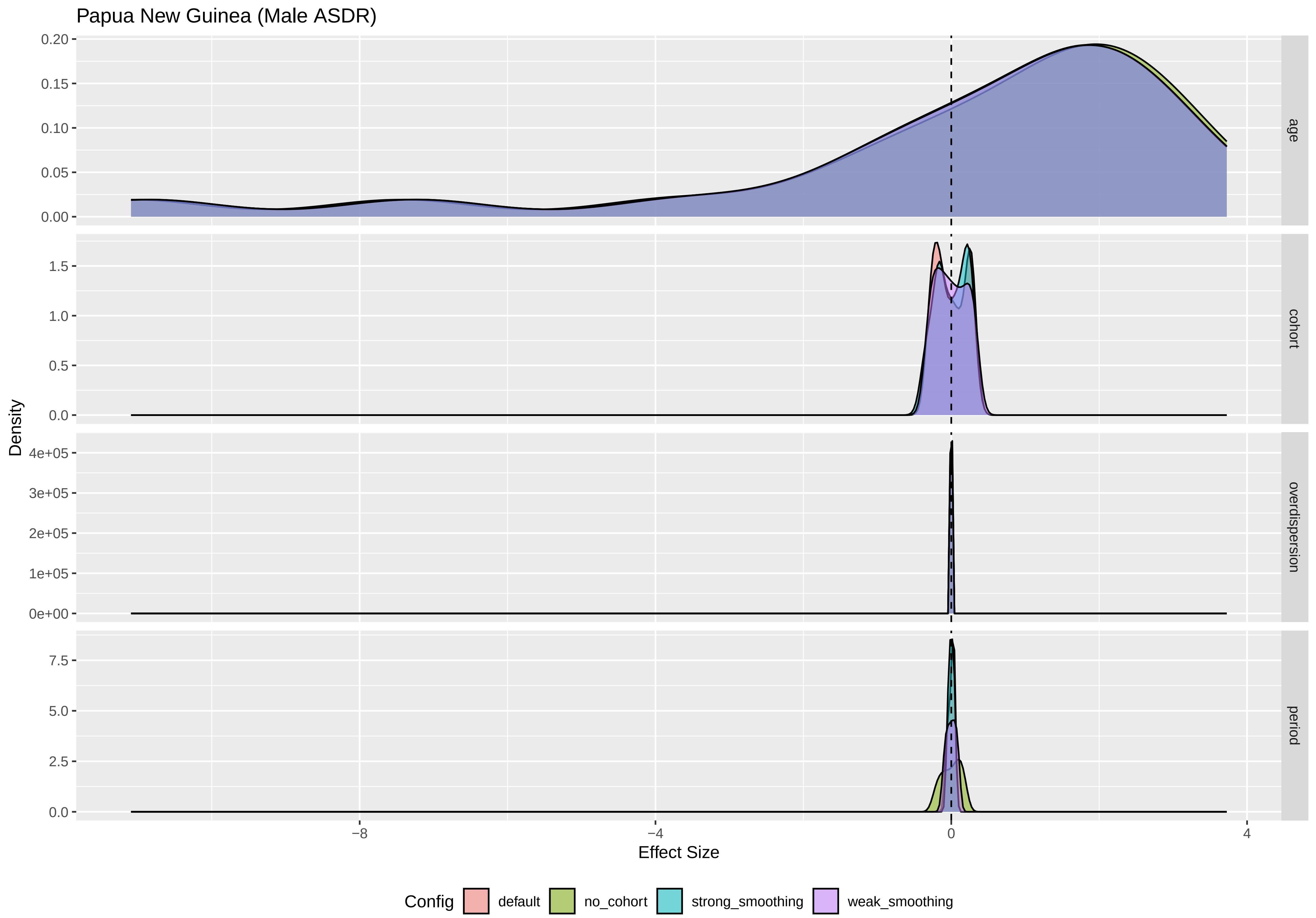

Papua New Guinea (Male ASDR)
Density
Effect Size
age
cohort
overdispersion
period
0.20
0.15
0.10
0.05
0.00
1.5
1.0
0.5
0.0
4e+05
3e+05
2e+05
1e+05
0e+00
7.5
5.0
2.5
0.0
−8
−4
0
4
Config
default
no_cohort
strong_smoothing
weak_smoothing

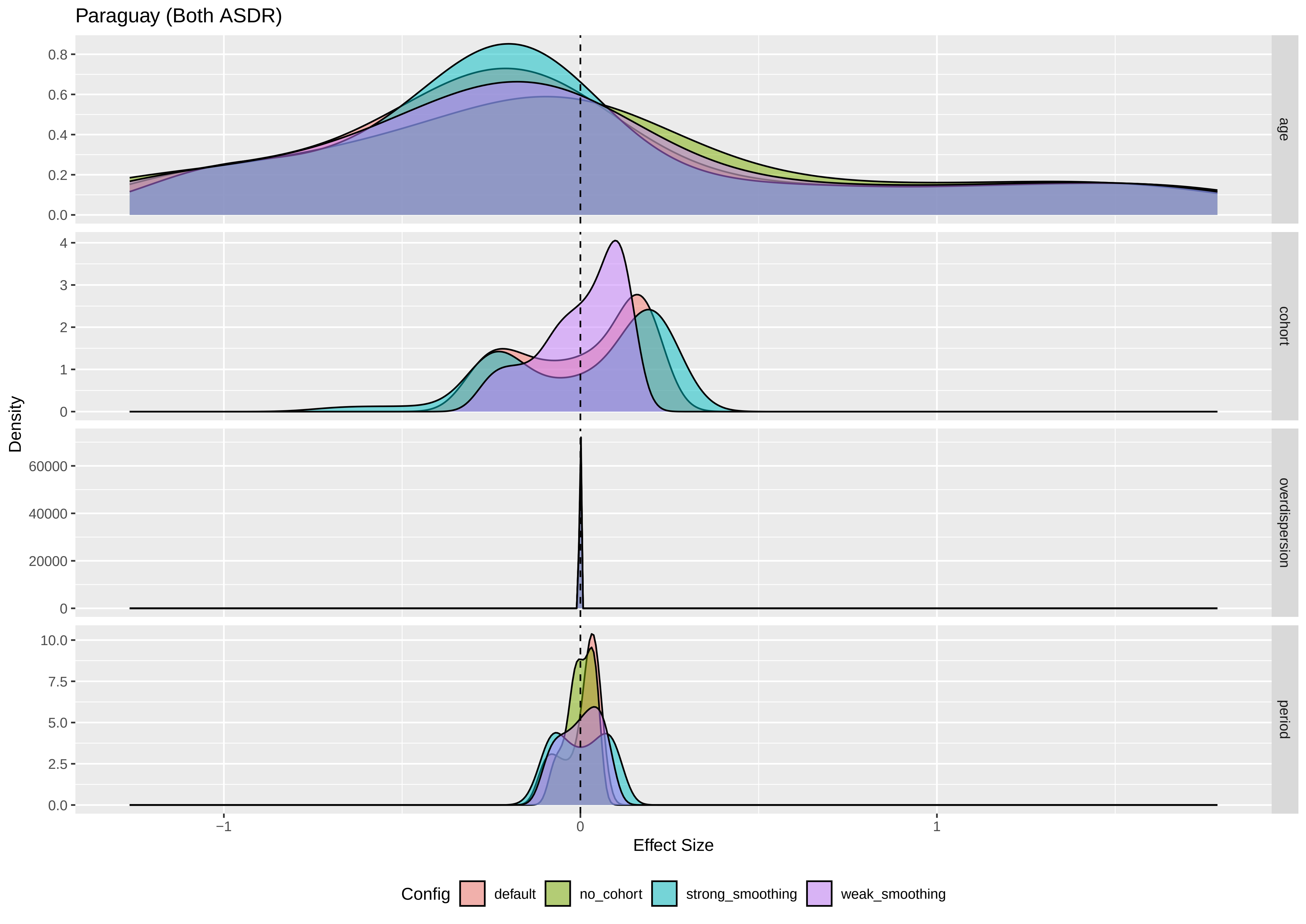

Paraguay (Both ASDR)
Density
Effect Size
age
cohort
overdispersion
period
0.0
0.2
0.4
0.6
0.8
0
1
2
3
4
0
20000
40000
60000
0.0
2.5
5.0
7.5
10.0
−1
0
1
Config
default
no_cohort
strong_smoothing
weak_smoothing

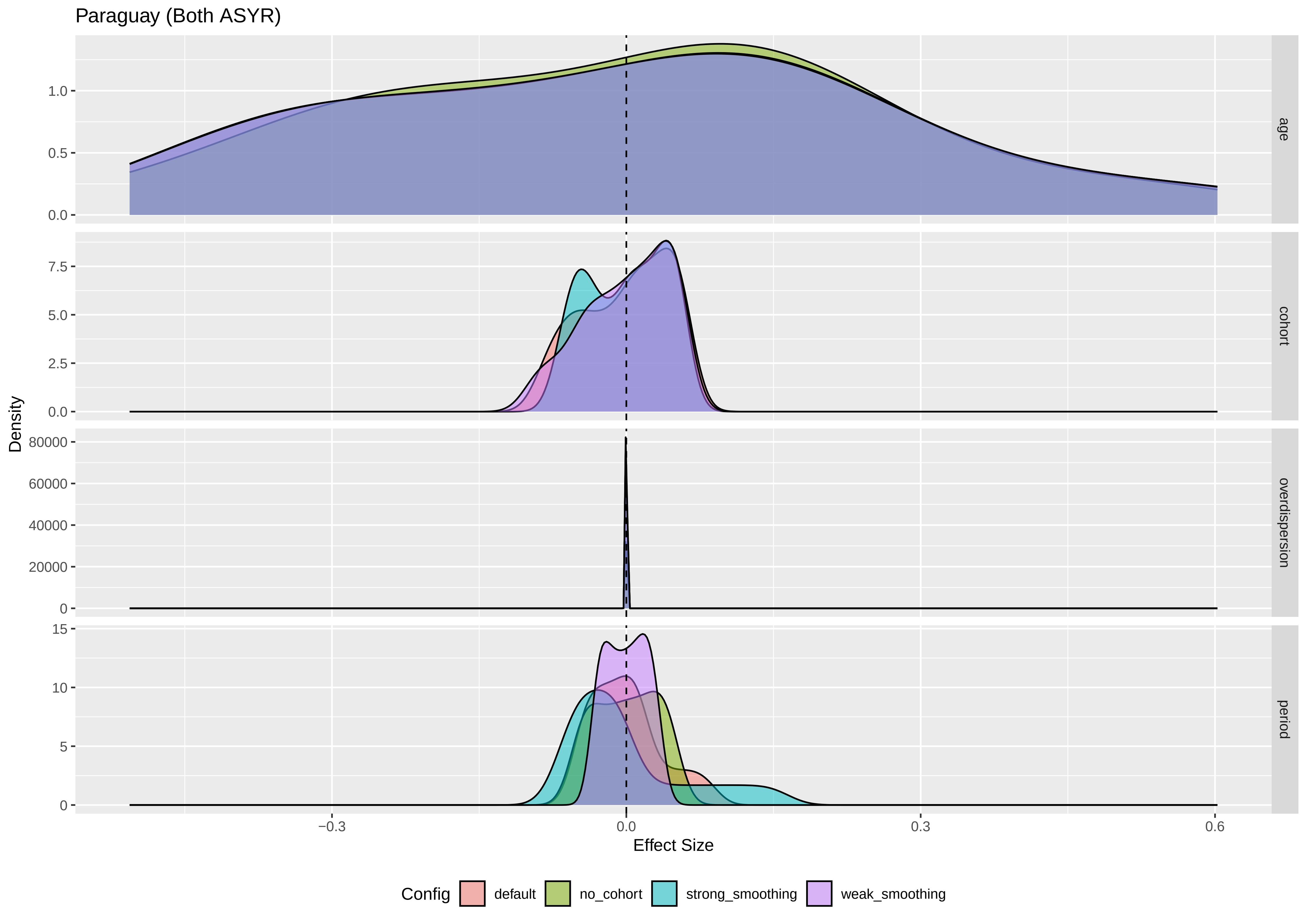

Paraguay (Both ASYR)
Density
Effect Size
age
cohort
overdispersion
period
1.0
0.5
0.0
7.5
5.0
2.5
0.0
80000
60000
40000
20000
0
15
10
5
0
−0.3
0.0
0.3
0.6
Config
default
no_cohort
strong_smoothing
weak_smoothing

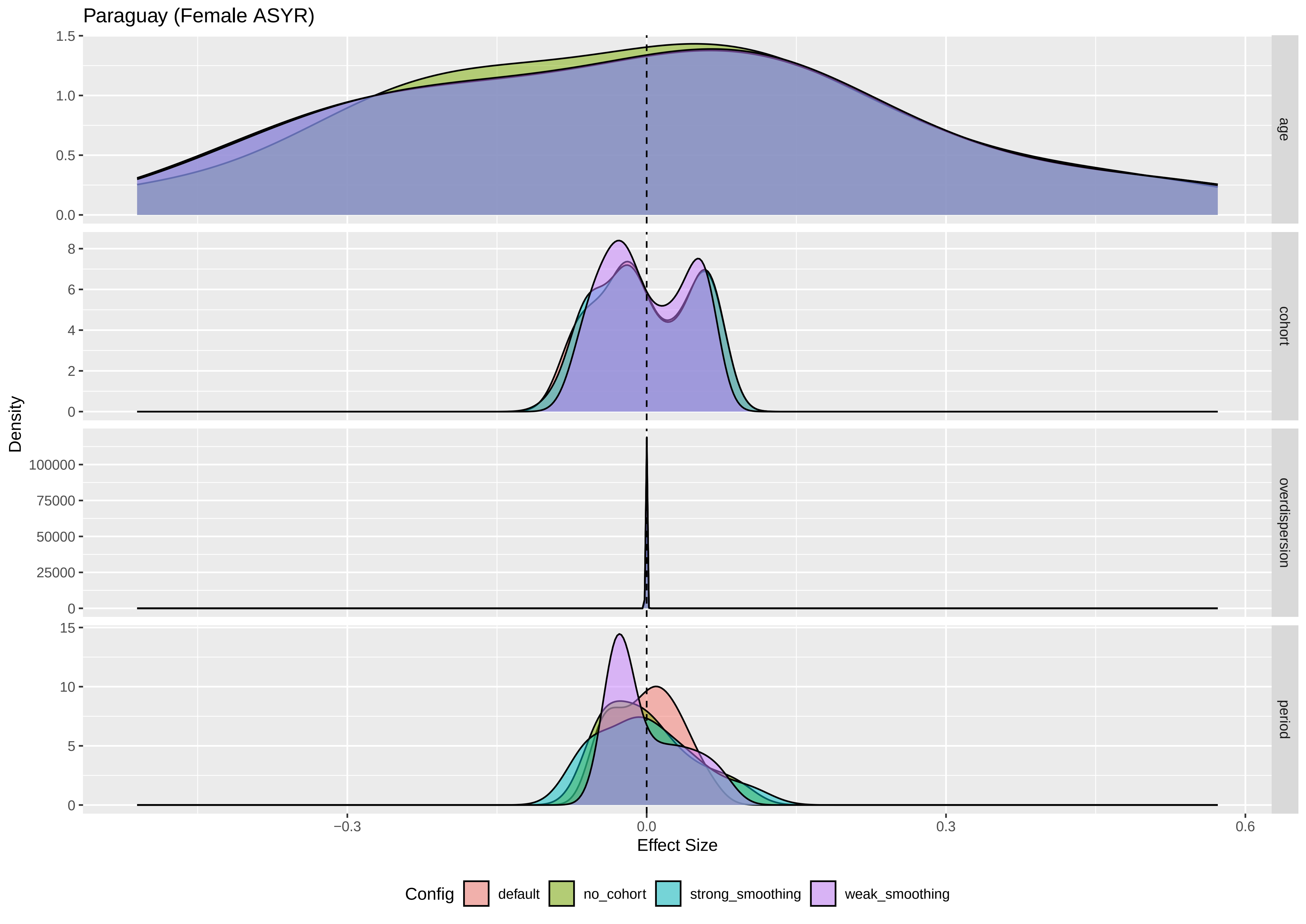

Paraguay (Female ASYR)
Density
Effect Size
age
cohort
overdispersion
period
1.5
1.0
0.5
0.0
8
6
4
2
0
100000
75000
50000
25000
0
15
10
5
0
−0.3
0.0
0.3
0.6
Config
default
no_cohort
strong_smoothing
weak_smoothing

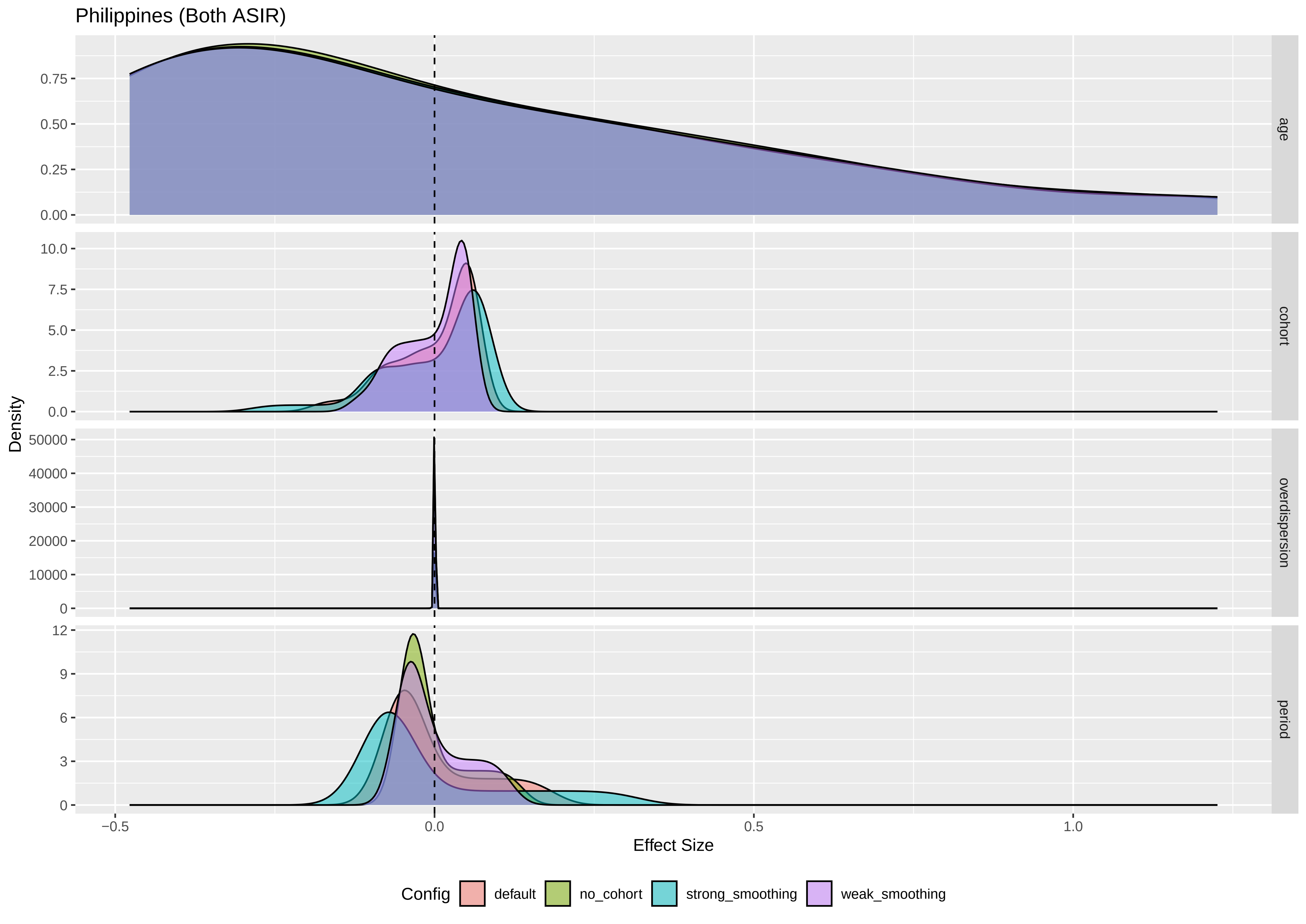

Philippines (Both ASIR)
0.75
0.50
0.25
0.00
age
10.0
7.5
5.0
2.5
0.0
cohort
50000
40000
30000
20000
10000
0
overdispersion
12
9
6
3
0
period
Density
−0.5
0.0
0.5
1.0
Effect Size
Config
default
no_cohort
strong_smoothing
weak_smoothing

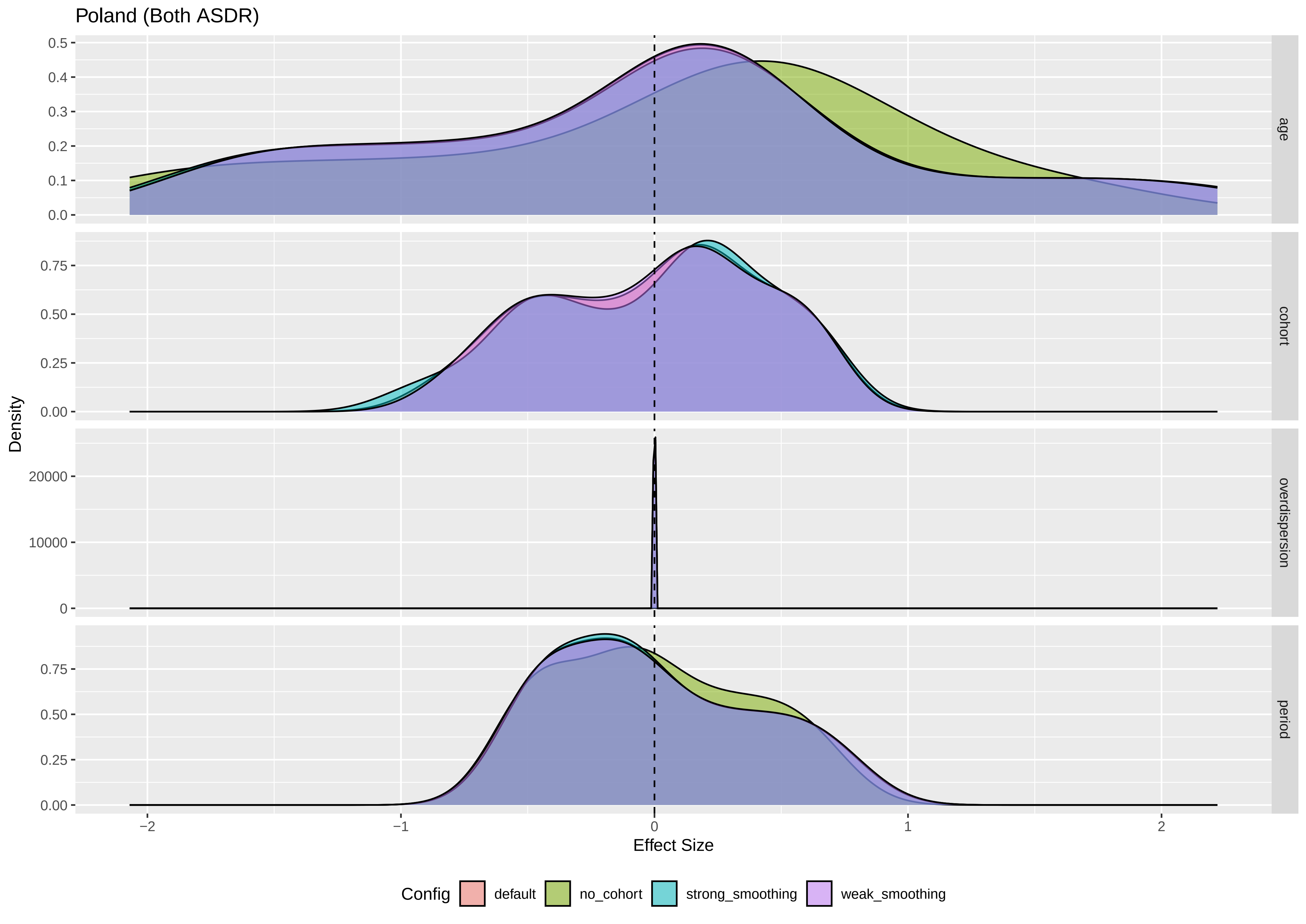

Poland (Both ASDR)
age
cohort
overdispersion
period
Density
Effect Size
0.0
0.1
0.2
0.3
0.4
0.5
0.00
0.25
0.50
0.75
0
10000
20000
−2
−1
0
1
2
Config
default
no_cohort
strong_smoothing
weak_smoothing

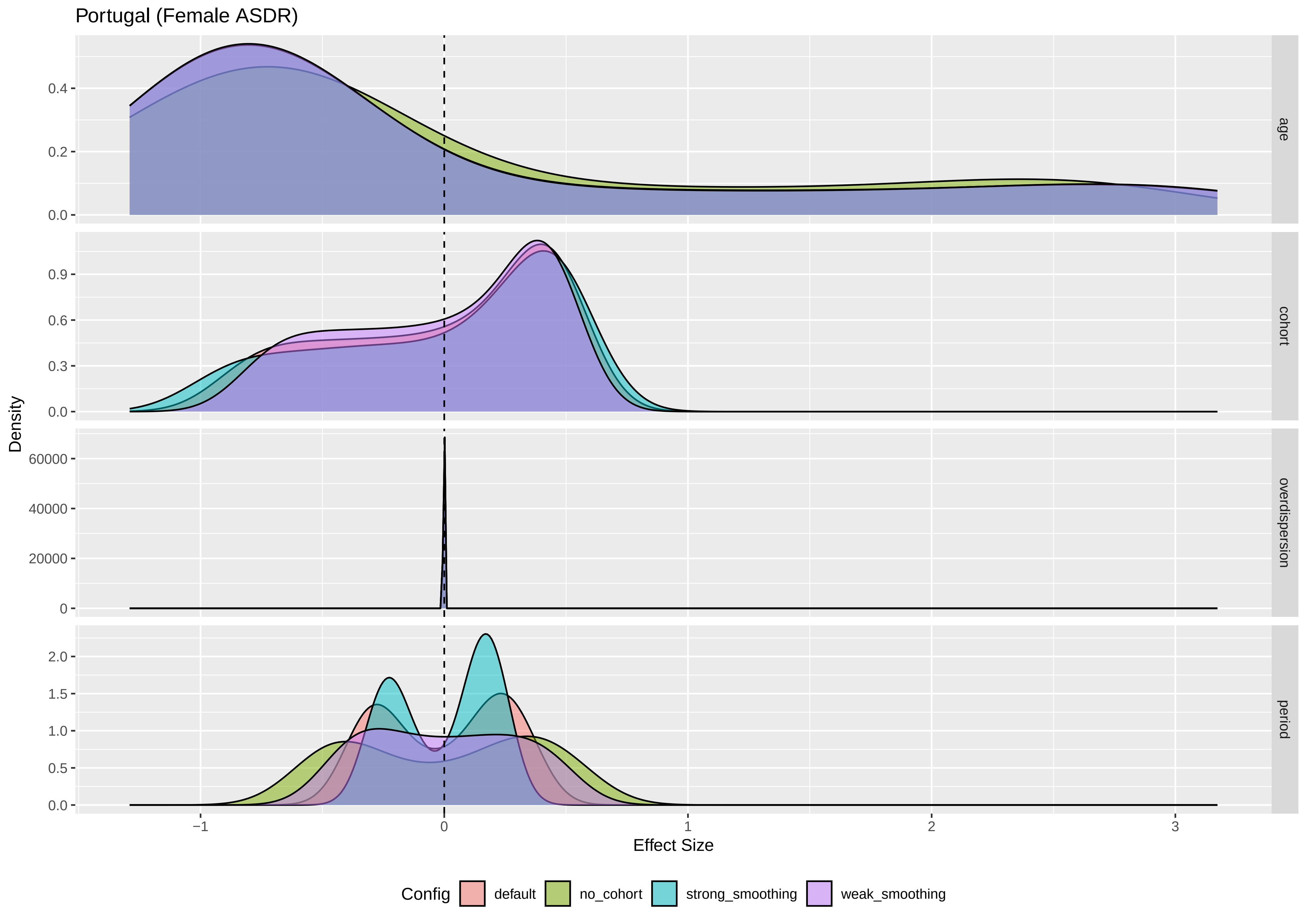

Portugal (Female ASDR)
Density
Effect Size
age
cohort
overdispersion
period
0.0
0.2
0.4
0.3
0.6
0.9
0
20000
40000
60000
0.5
1.0
1.5
2.0
−1
0
1
2
3
Config
default
no_cohort
strong_smoothing
weak_smoothing

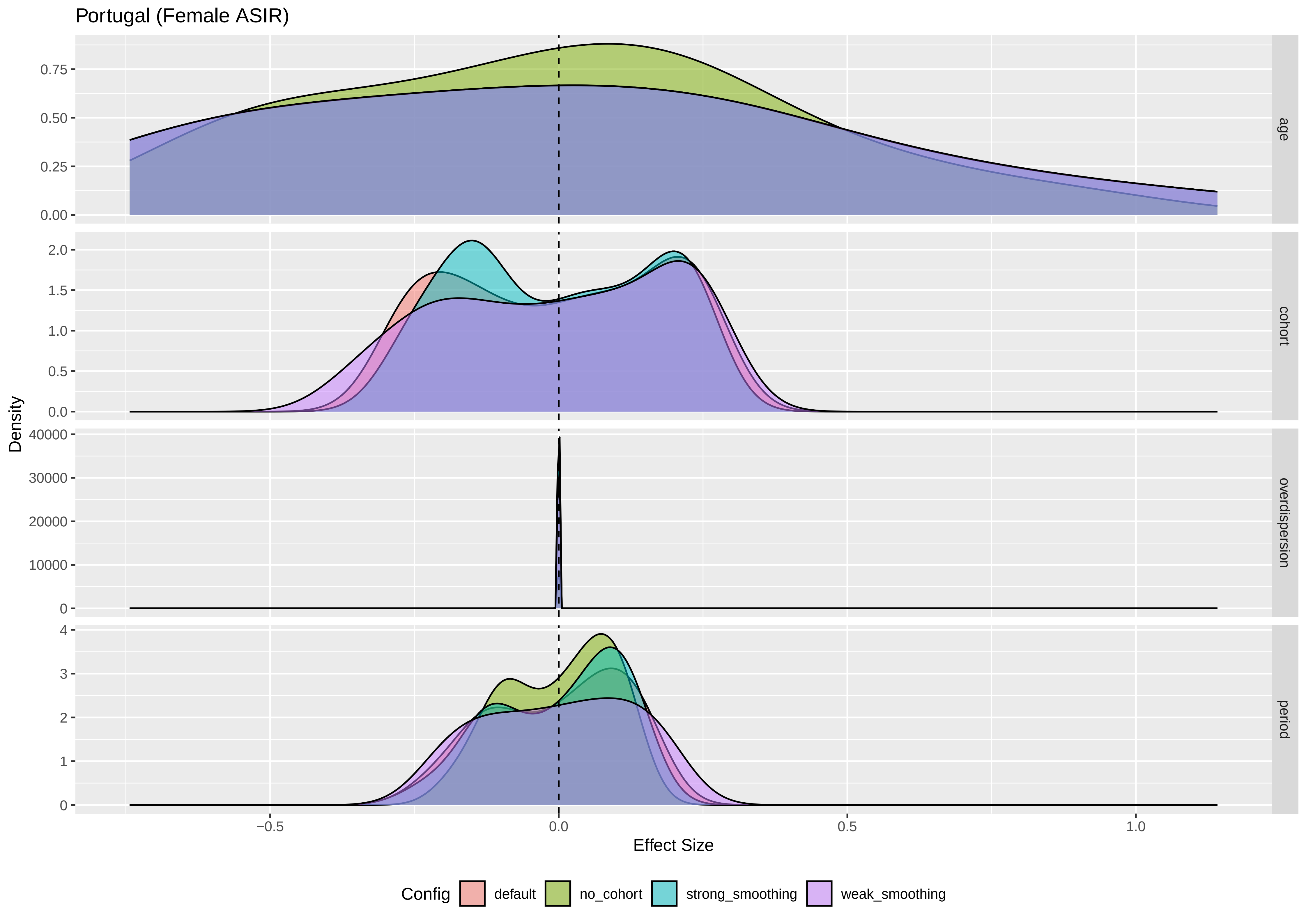

Portugal (Female ASIR)
Density
Effect Size
age
cohort
overdispersion
period
0.00
0.25
0.50
0.75
0.0
0.5
1.0
1.5
2.0
0
10000
20000
30000
40000
0
1
2
3
4
−0.5
0.0
0.5
1.0
Config
default
no_cohort
strong_smoothing
weak_smoothing

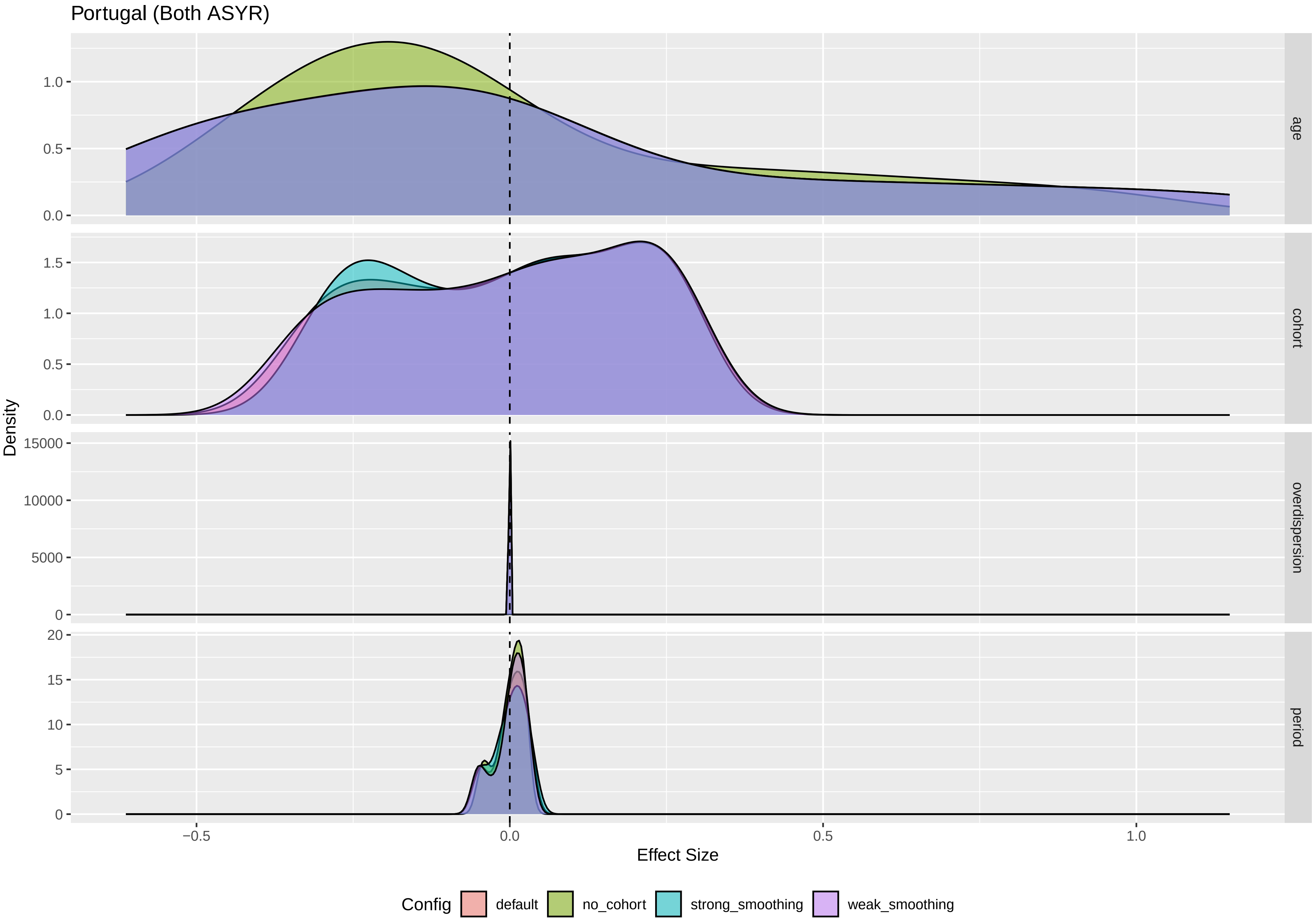

Portugal (Both ASYR)
age
cohort
overdispersion
period
Density
Effect Size
1.0
0.5
0.0
1.5
15000
10000
5000
0
20
15
10
5
−0.5
0.0
0.5
1.0
Config
default
no_cohort
strong_smoothing
weak_smoothing

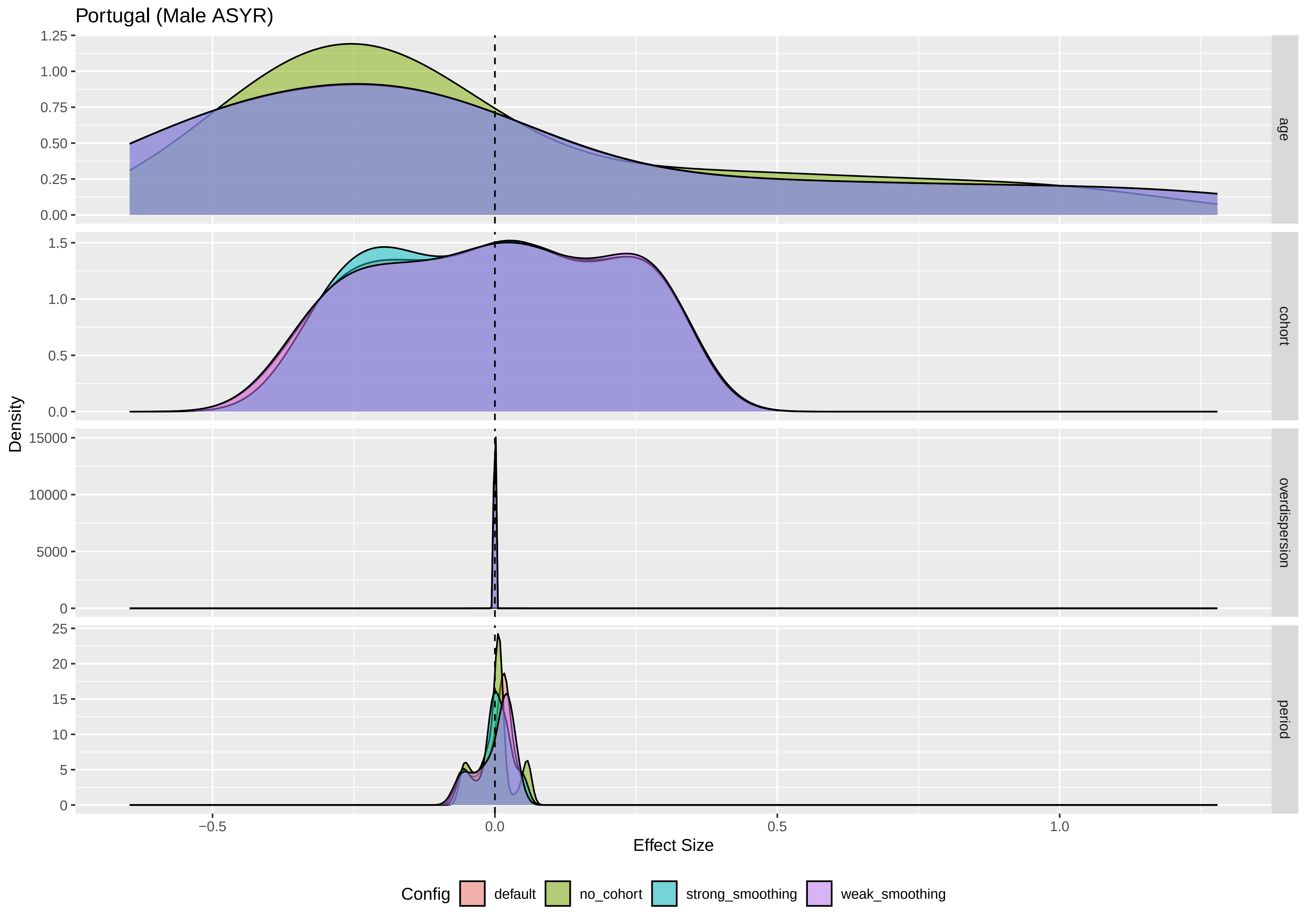
Portugal (Male ASYR)
Density
Effect Size
age
cohort
overdispersion
period
1.25
1.00
0.75
0.50
0.25
0.00
1.5
1.0
0.5
0.0
15000
10000
5000
0
25
20
15
10
5
0
−0.5
0.0
0.5
1.0
Config
default
no_cohort
strong_smoothing
weak_smoothing

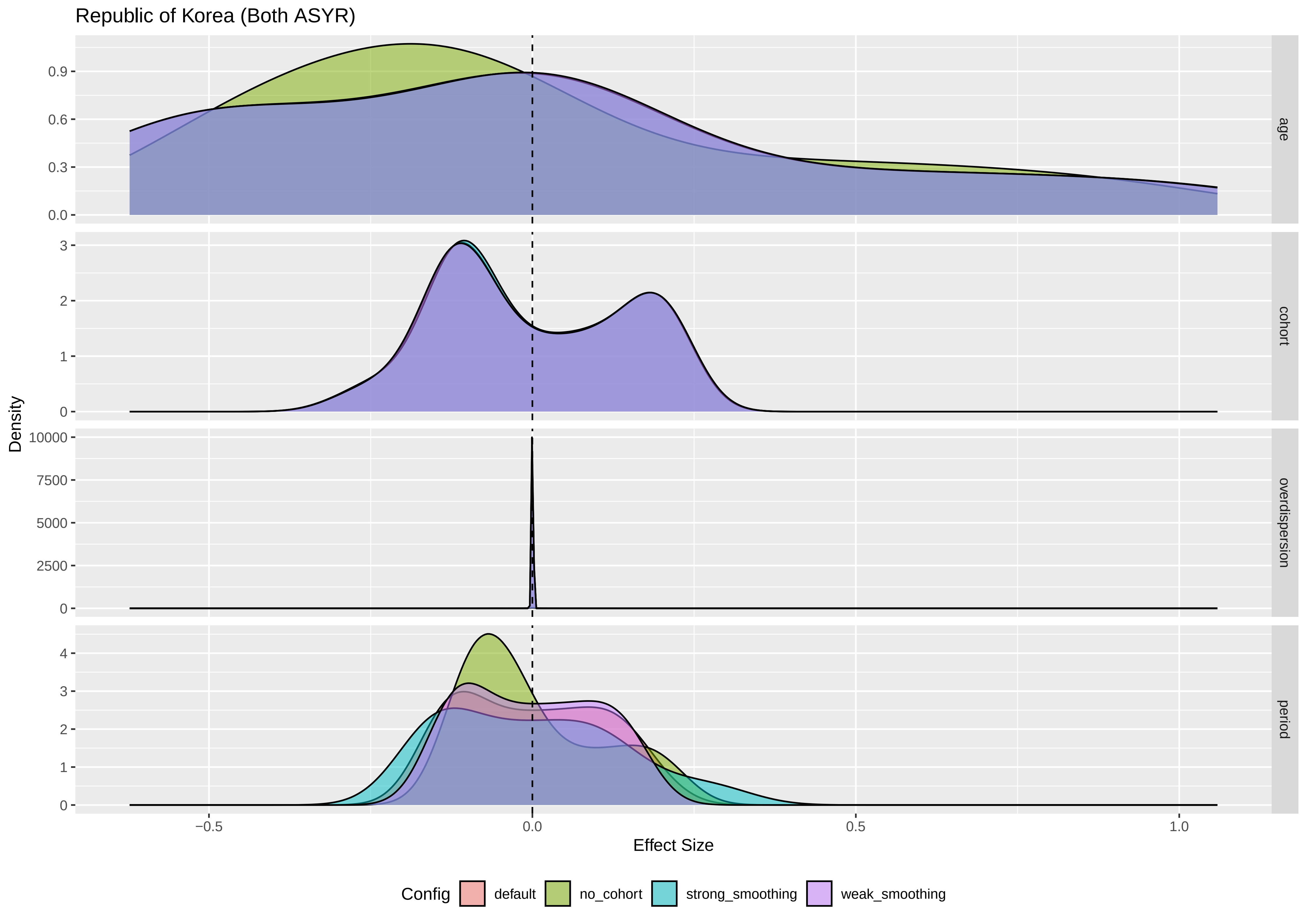

Republic of Korea (Both ASYR)
age
cohort
overdispersion
period
Density
Effect Size
0.0
0.3
0.6
0.9
0
1
2
3
2500
5000
7500
10000
4
−0.5
0.0
0.5
1.0
Config
default
no_cohort
strong_smoothing
weak_smoothing

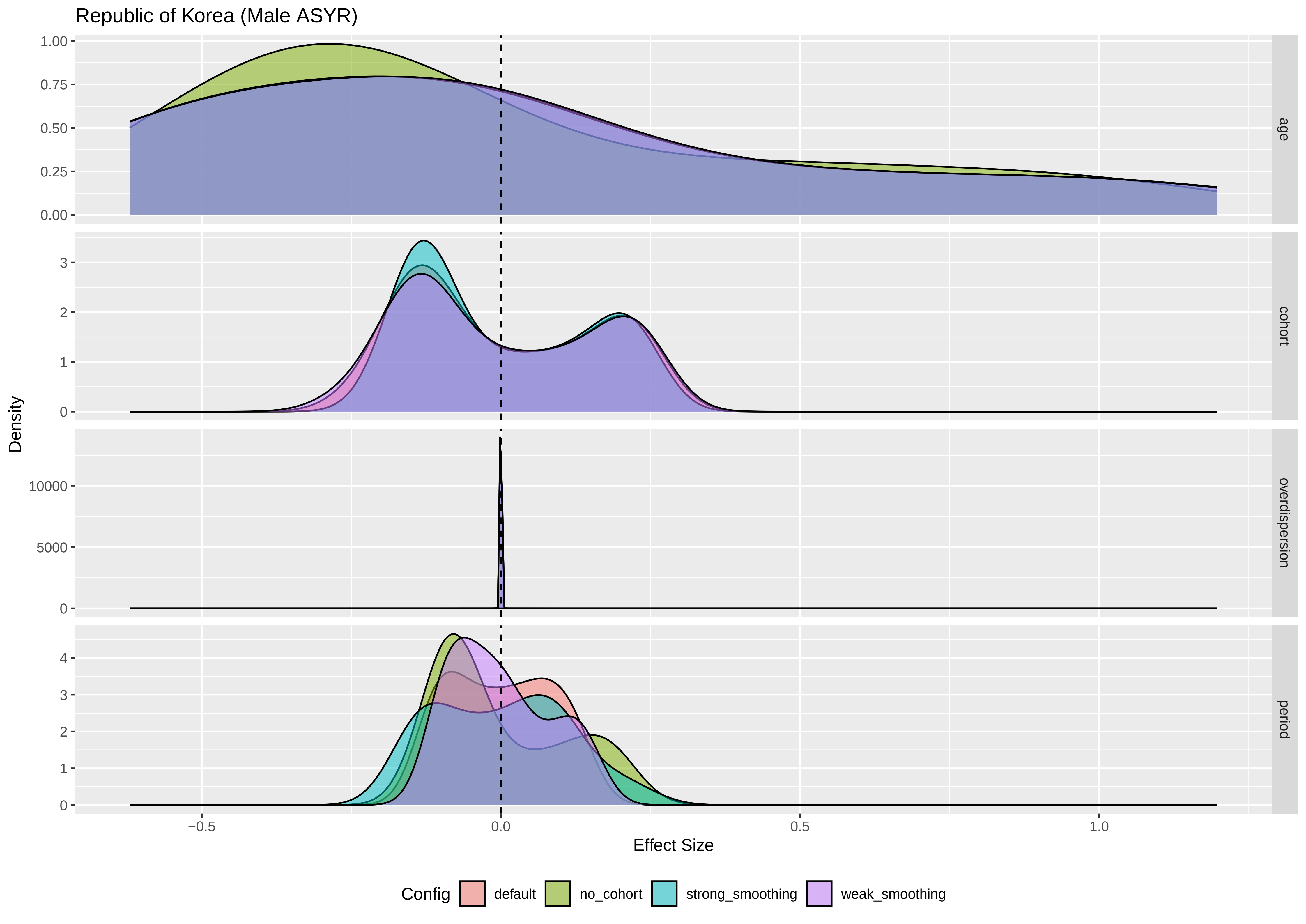

Republic of Korea (Male ASYR)
Density
Effect Size
age
cohort
overdispersion
period
1.00
0.75
0.50
0.25
0.00
3
2
1
0
10000
5000
0
4
3
2
1
0
−0.5
0.0
0.5
1.0
Config
default
no_cohort
strong_smoothing
weak_smoothing

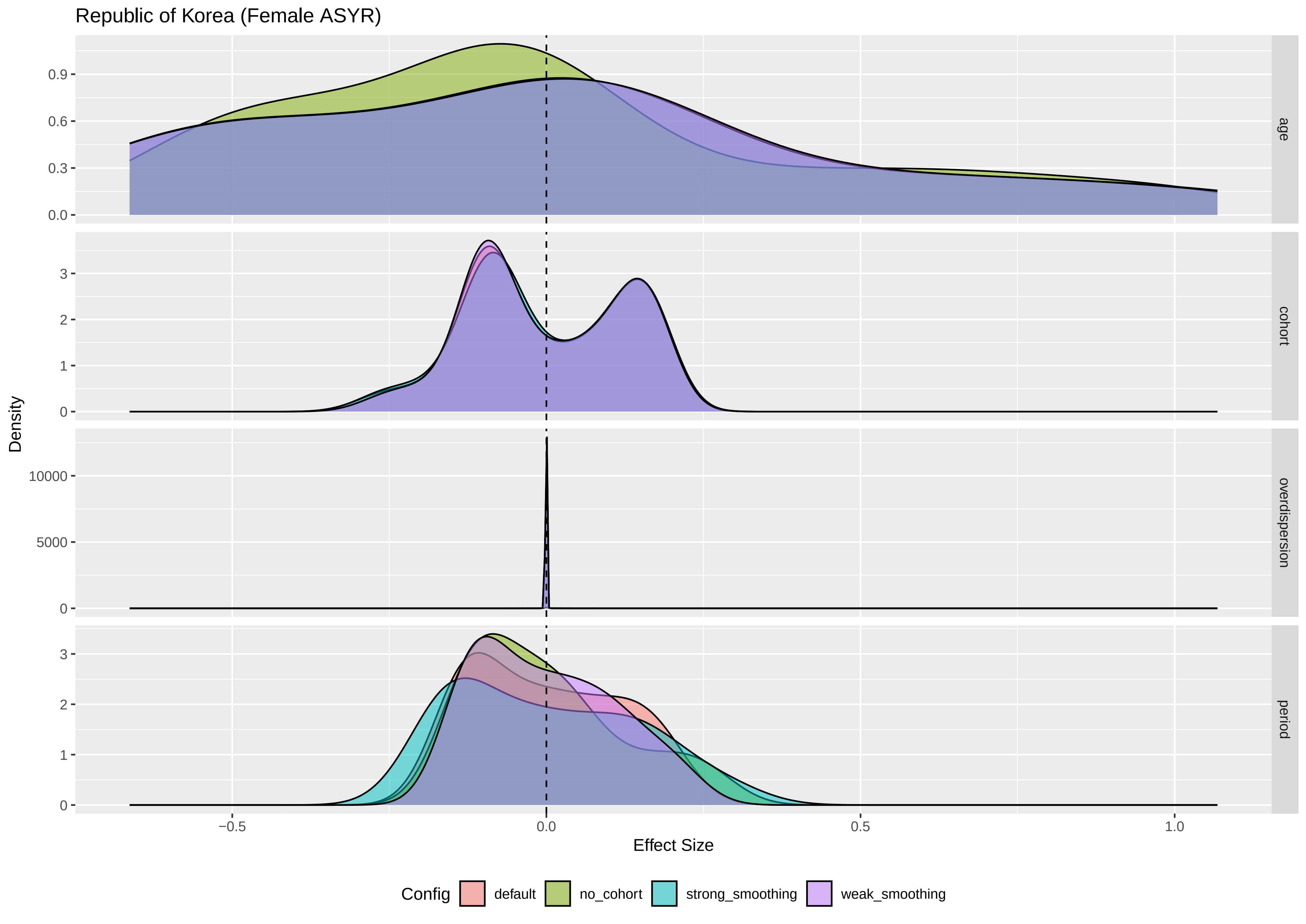
Republic of Korea (Female ASYR)
age
cohort
overdispersion
period
Density
Effect Size
0.0
0.3
0.6
0.9
0
1
2
3
0
5000
10000
−0.5
0.0
0.5
1.0
Config
default
no_cohort
strong_smoothing
weak_smoothing

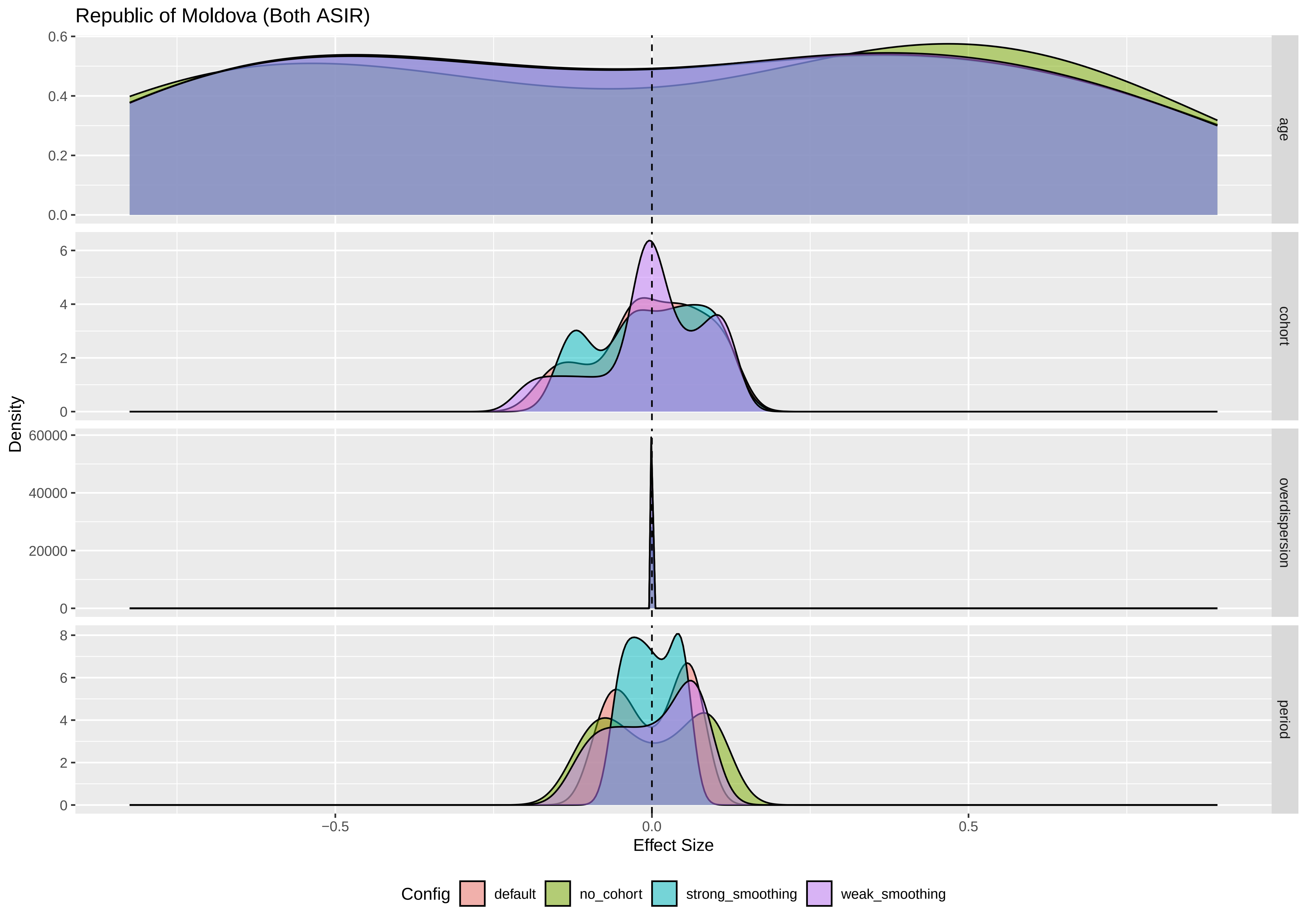

Republic of Moldova (Both ASIR)
Density
Effect Size
age
cohort
overdispersion
period
0.0
0.2
0.4
0.6
0
2
4
6
0
20000
40000
60000
0
2
4
6
8
−0.5
0.0
0.5
Config
default
no_cohort
strong_smoothing
weak_smoothing

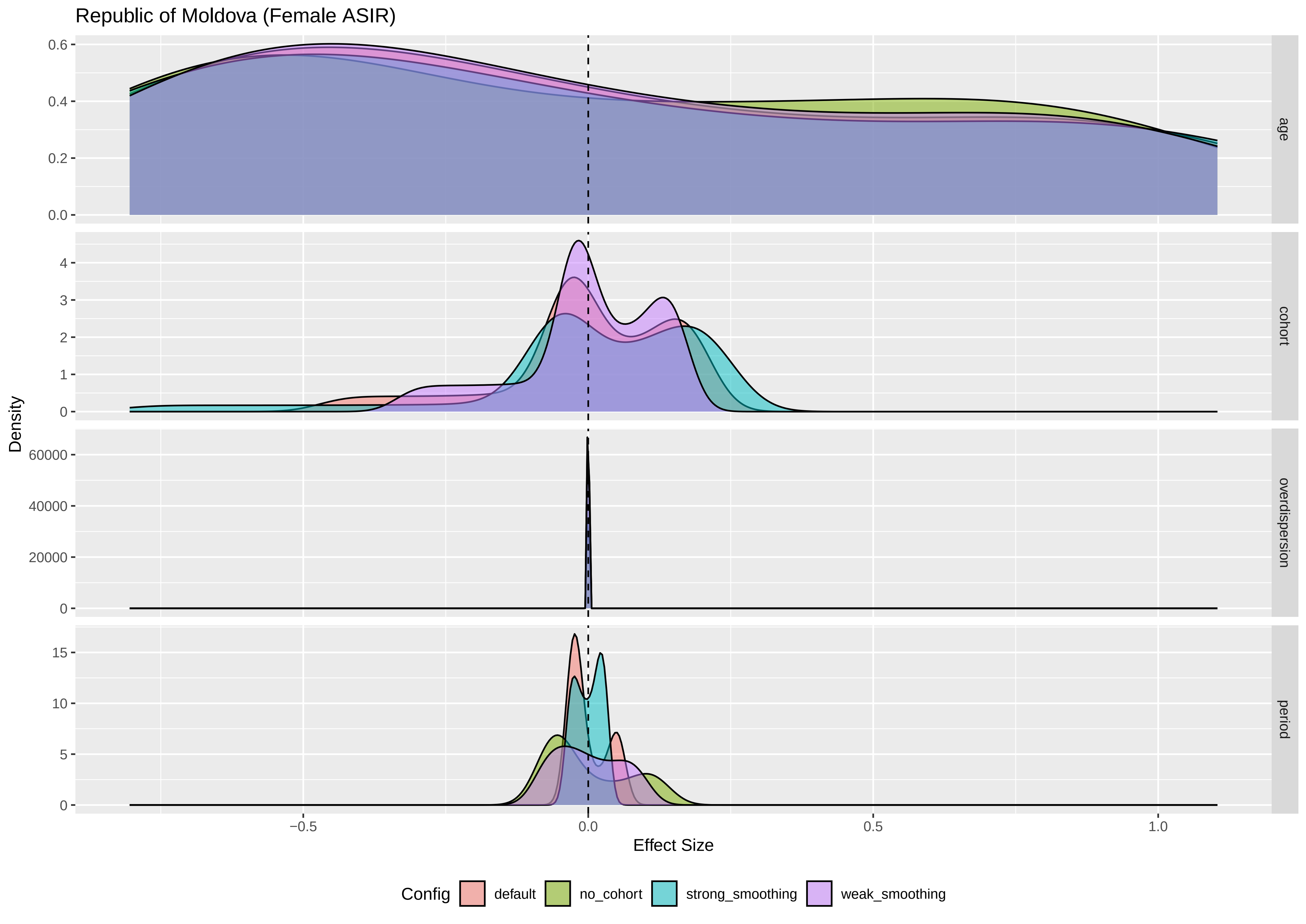

Republic of Moldova (Female ASIR)
Density
Effect Size
age
cohort
overdispersion
period
0.0
0.2
0.4
0.6
0
1
2
3
4
20000
40000
60000
5
10
15
−0.5
0.0
0.5
1.0
Config
default
no_cohort
strong_smoothing
weak_smoothing

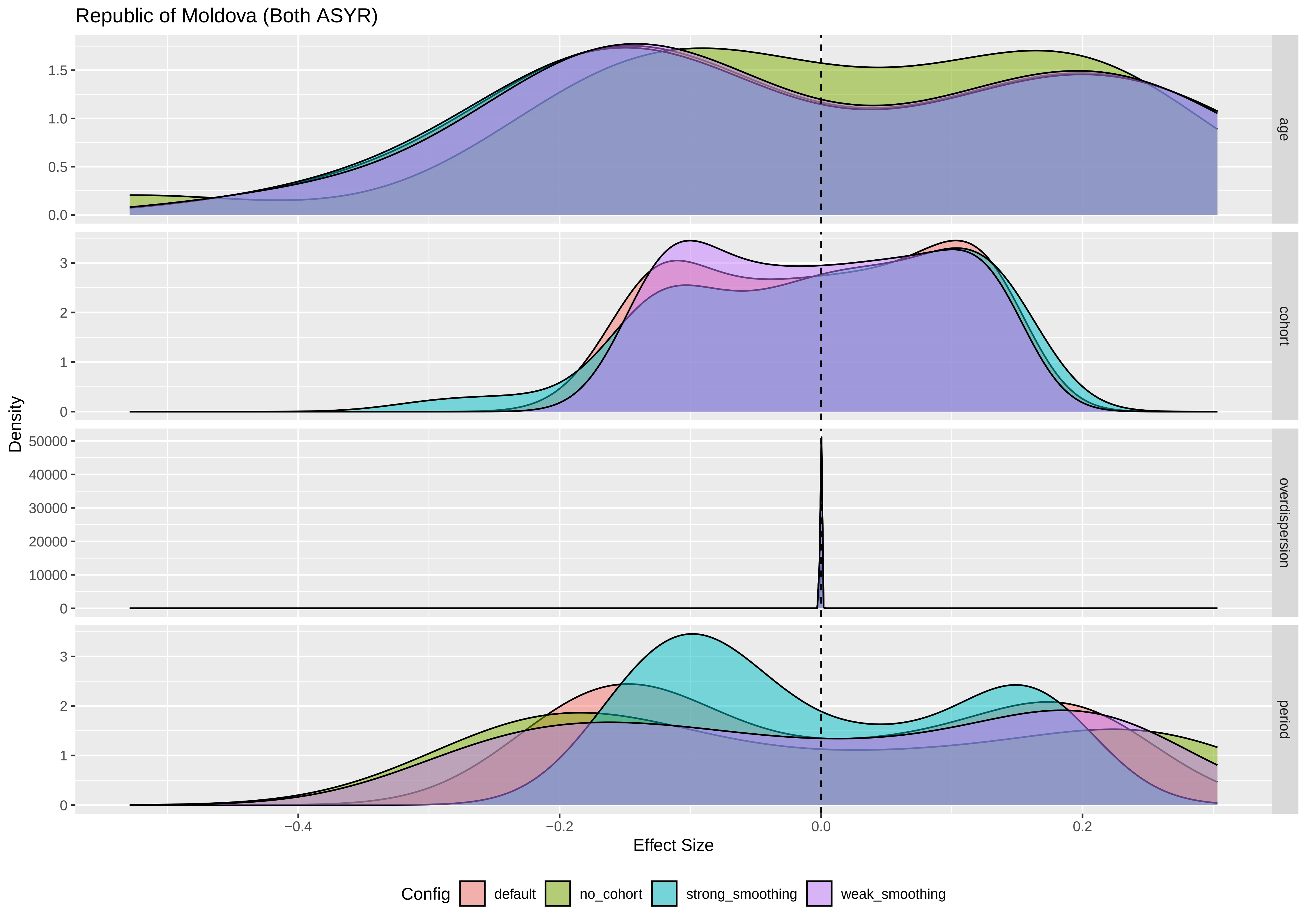

Republic of Moldova (Both ASYR)
age
cohort
overdispersion
period
Density
Effect Size
1.5
1.0
0.5
0.0
3
2
1
0
50000
40000
30000
20000
10000
0
−0.4
−0.2
0.0
0.2
Config
default
no_cohort
strong_smoothing
weak_smoothing

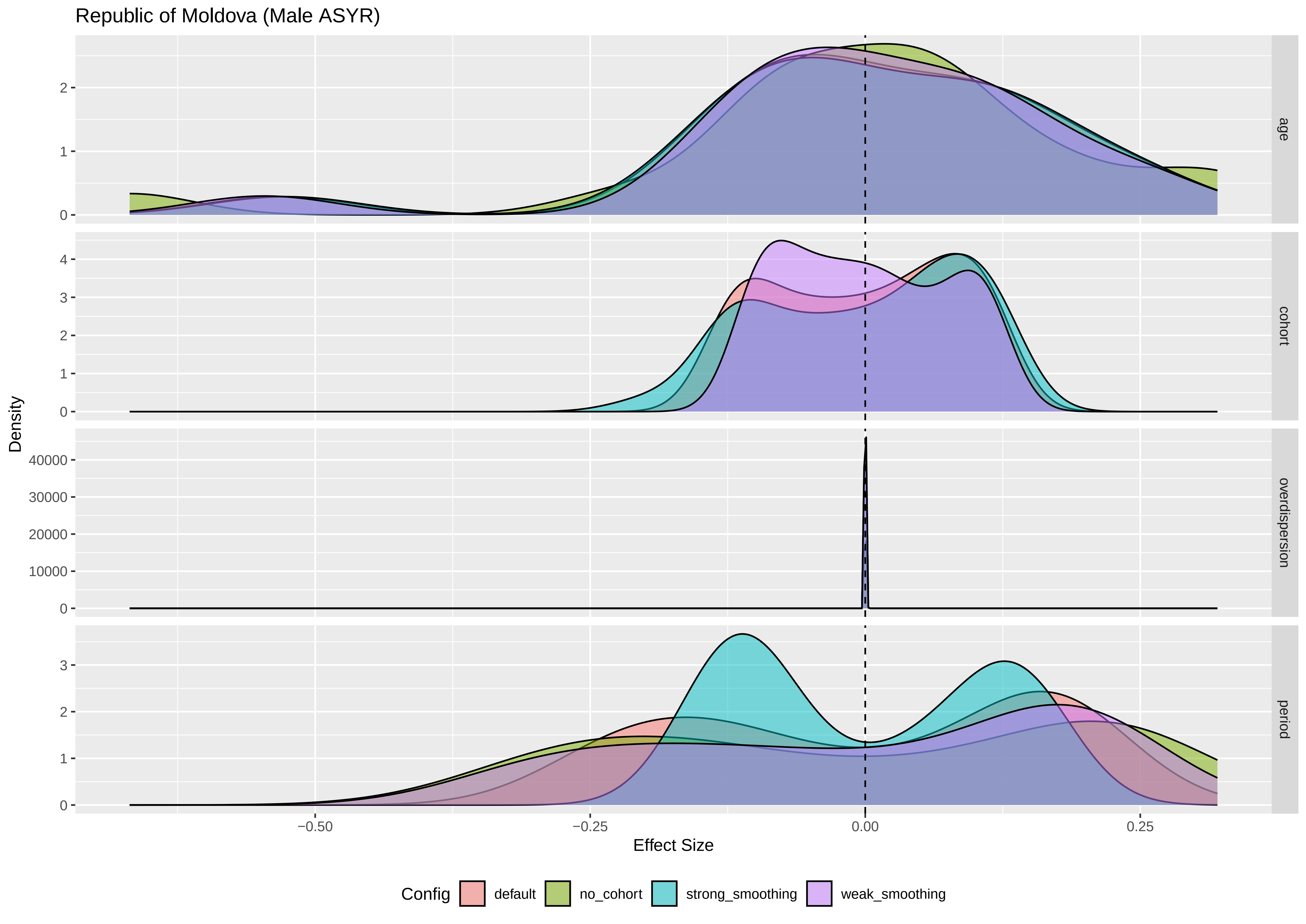

Republic of Moldova (Male ASYR)
age
cohort
overdispersion
period
Density
Effect Size
−0.50
−0.25
0.00
0.25
0
1
2
3
4
10000
20000
30000
40000
Config
default
no_cohort
strong_smoothing
weak_smoothing

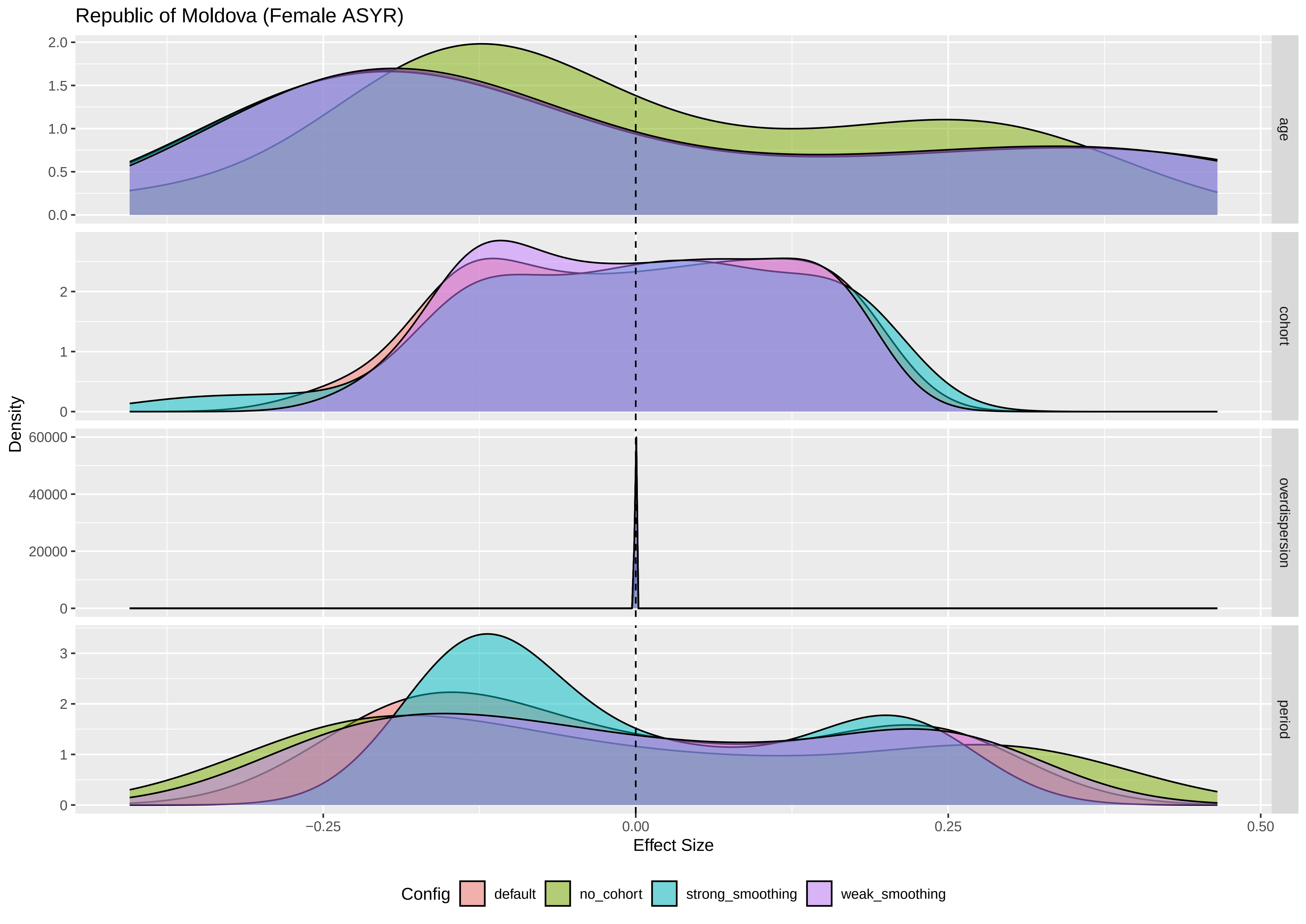

Republic of Moldova (Female ASYR)
age
cohort
overdispersion
period
Density
Effect Size
2.0
1.5
1.0
0.5
0.0
2
1
0
60000
40000
20000
0
3
2
1
0
−0.25
0.00
0.25
0.50
Config
default
no_cohort
strong_smoothing
weak_smoothing

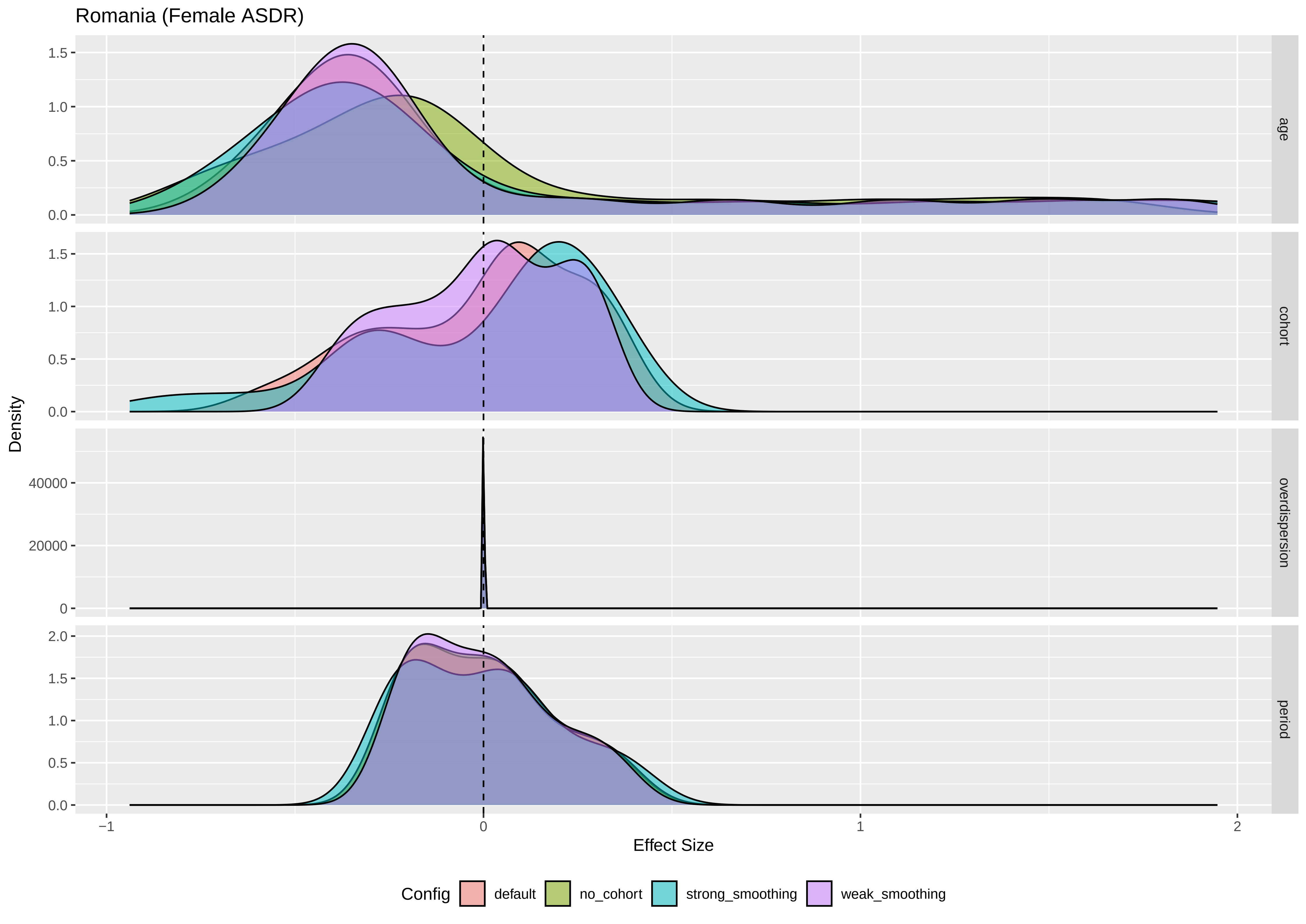

Romania (Female ASDR)
Density
Effect Size
age
cohort
overdispersion
period
1.5
1.0
0.5
0.0
40000
20000
0
2.0
−1
0
1
2
Config
default
no_cohort
strong_smoothing
weak_smoothing

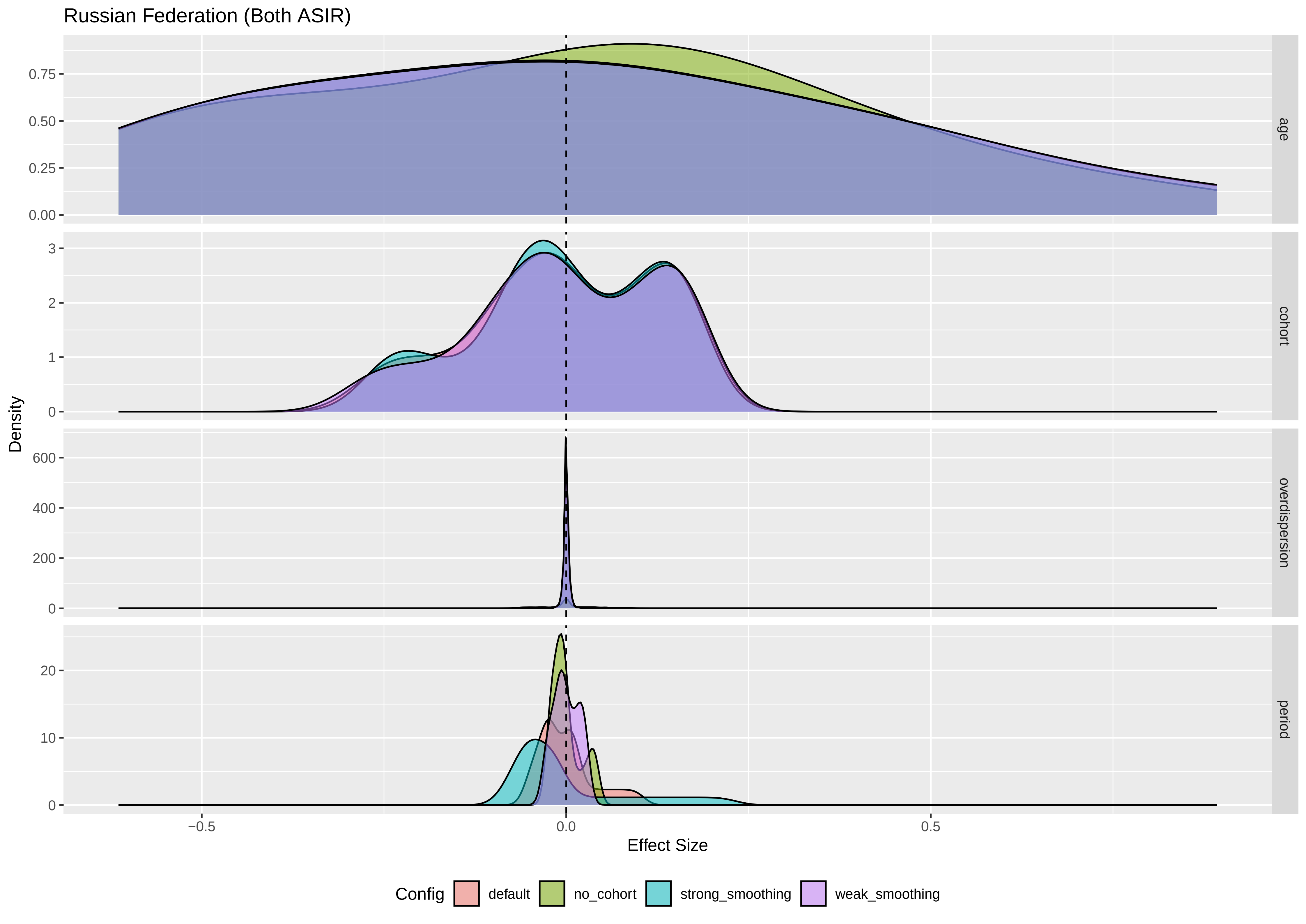

Russian Federation (Both ASIR)
age
cohort
overdispersion
period
Density
0.00
0.25
0.50
0.75
0
1
2
3
0
200
400
600
0
10
20
−0.5
0.0
0.5
Effect Size
Config
default
no_cohort
strong_smoothing
weak_smoothing

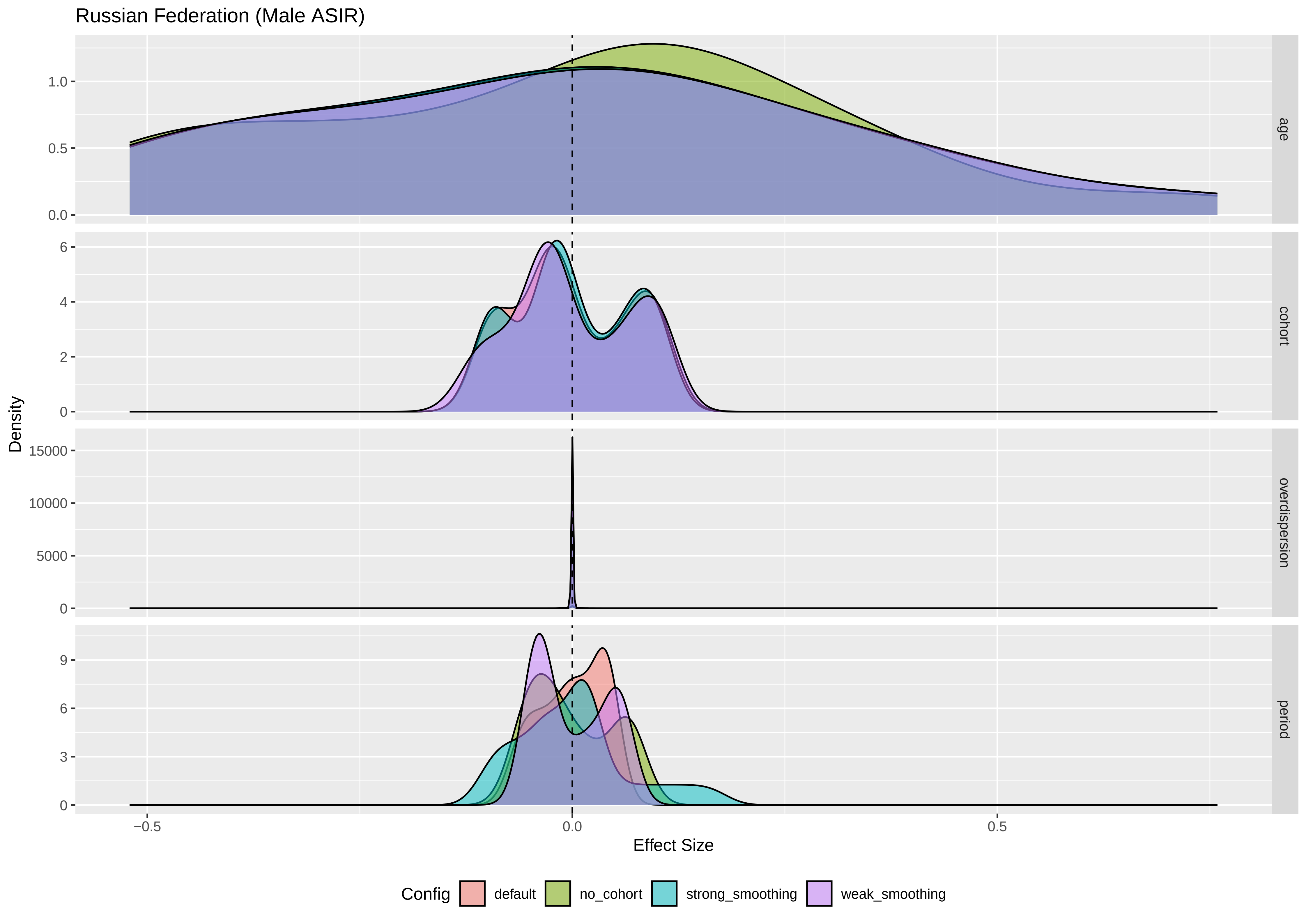

Russian Federation (Male ASIR)
age
cohort
overdispersion
period
Density
Effect Size
−0.5
0.0
0.5
Config
default
no_cohort
strong_smoothing
weak_smoothing

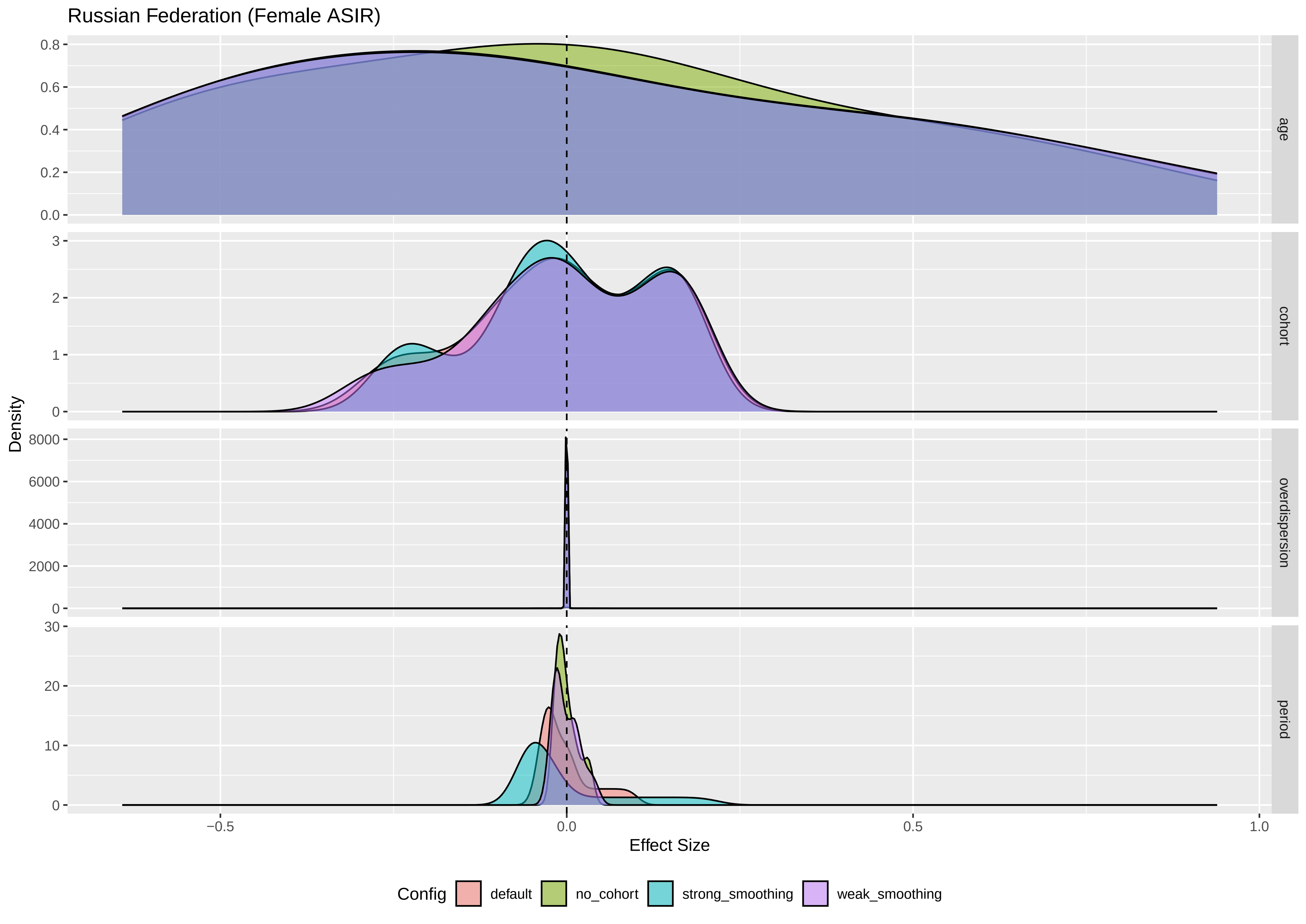

Russian Federation (Female ASIR)
age
cohort
overdispersion
period
Density
Effect Size
−0.5
0.0
0.5
1.0
Config
default
no_cohort
strong_smoothing
weak_smoothing

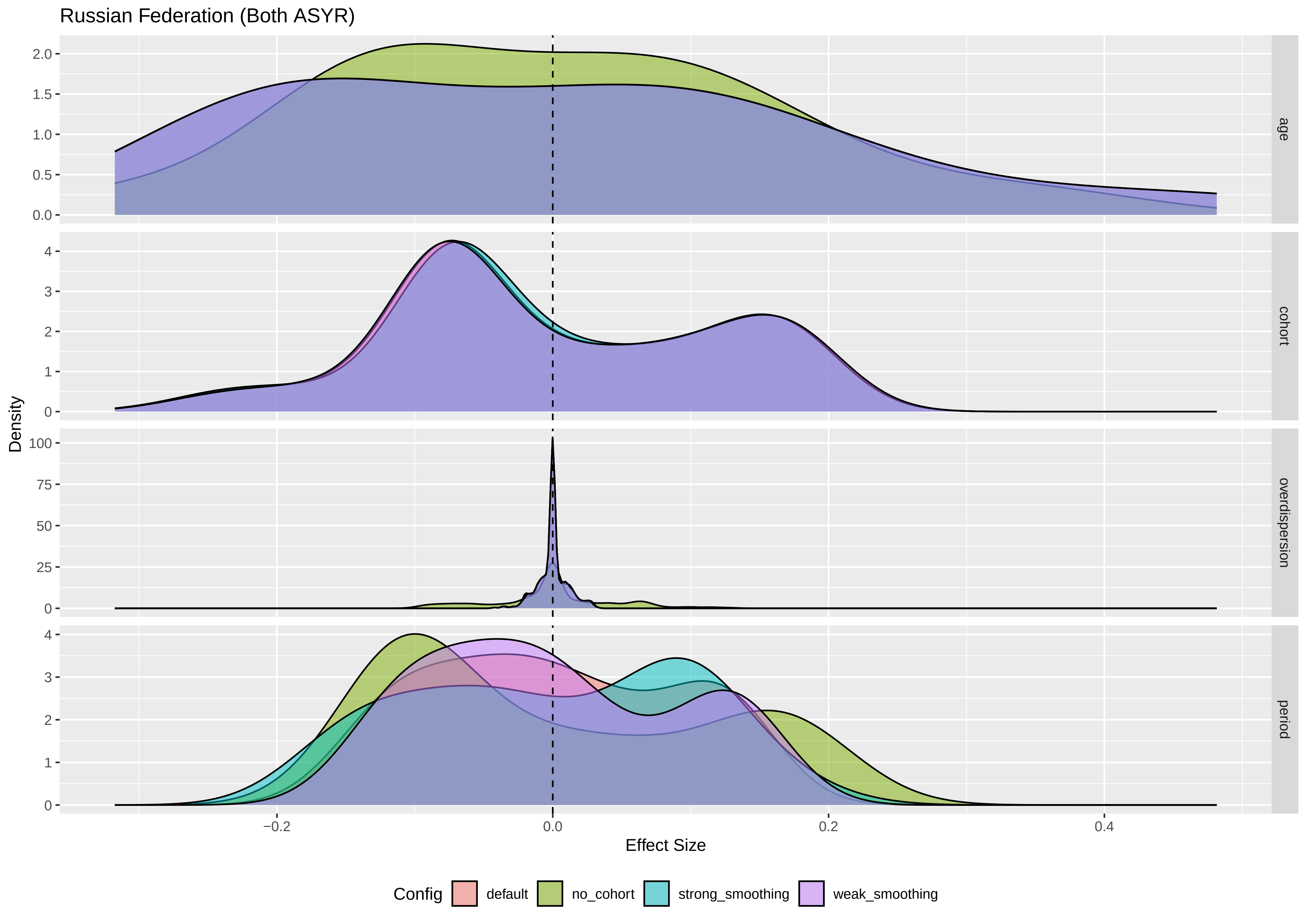

Russian Federation (Both ASYR)
Density
age
cohort
overdispersion
period
2.0
1.5
1.0
0.5
0.0
4
3
2
1
0
100
75
50
25
0
−0.2
0.0
0.2
0.4
Effect Size
Config
default
no_cohort
strong_smoothing
weak_smoothing

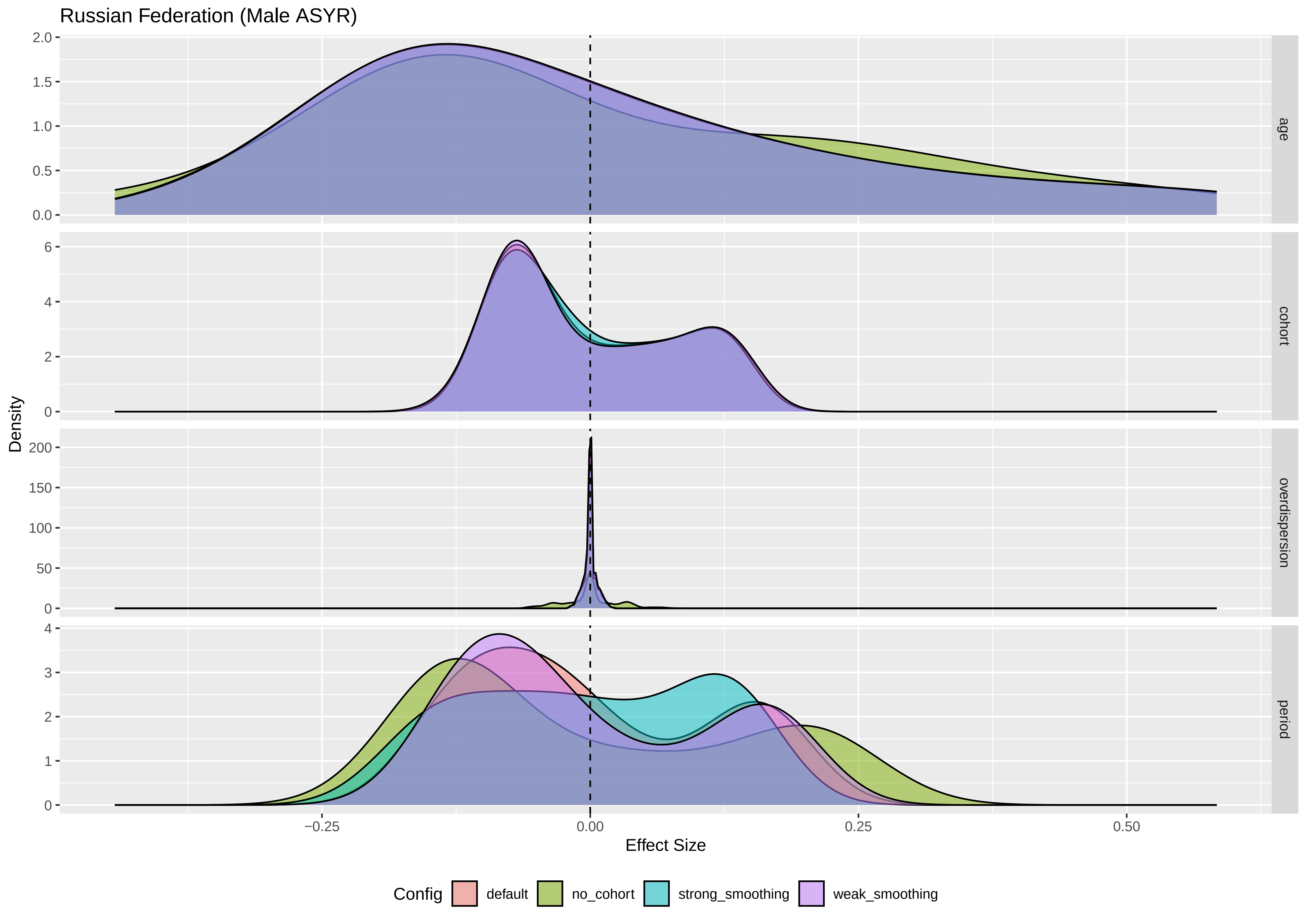

Russian Federation (Male ASYR)
Density
Effect Size
age
cohort
overdispersion
period
2.0
1.5
1.0
0.5
0.0
6
4
2
0
200
150
100
50
0
4
3
2
1
0
−0.25
0.00
0.25
0.50
Config
default
no_cohort
strong_smoothing
weak_smoothing

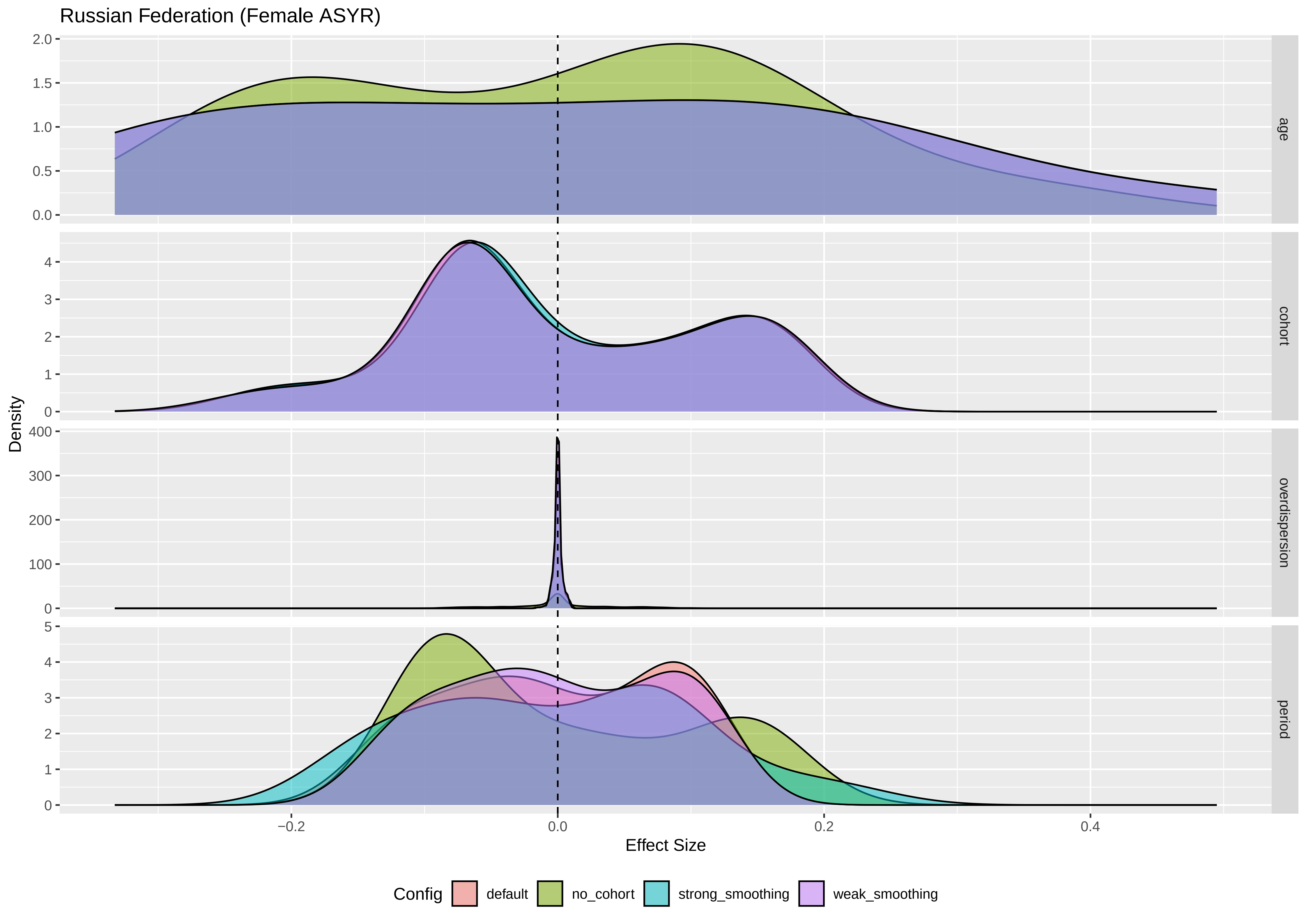

Russian Federation (Female ASYR)
Density
Effect Size
age
cohort
overdispersion
period
2.0
1.5
1.0
0.5
0.0
4
3
2
1
0
400
300
200
100
0
5
4
3
2
1
0
−0.2
0.0
0.2
0.4
Config
default
no_cohort
strong_smoothing
weak_smoothing

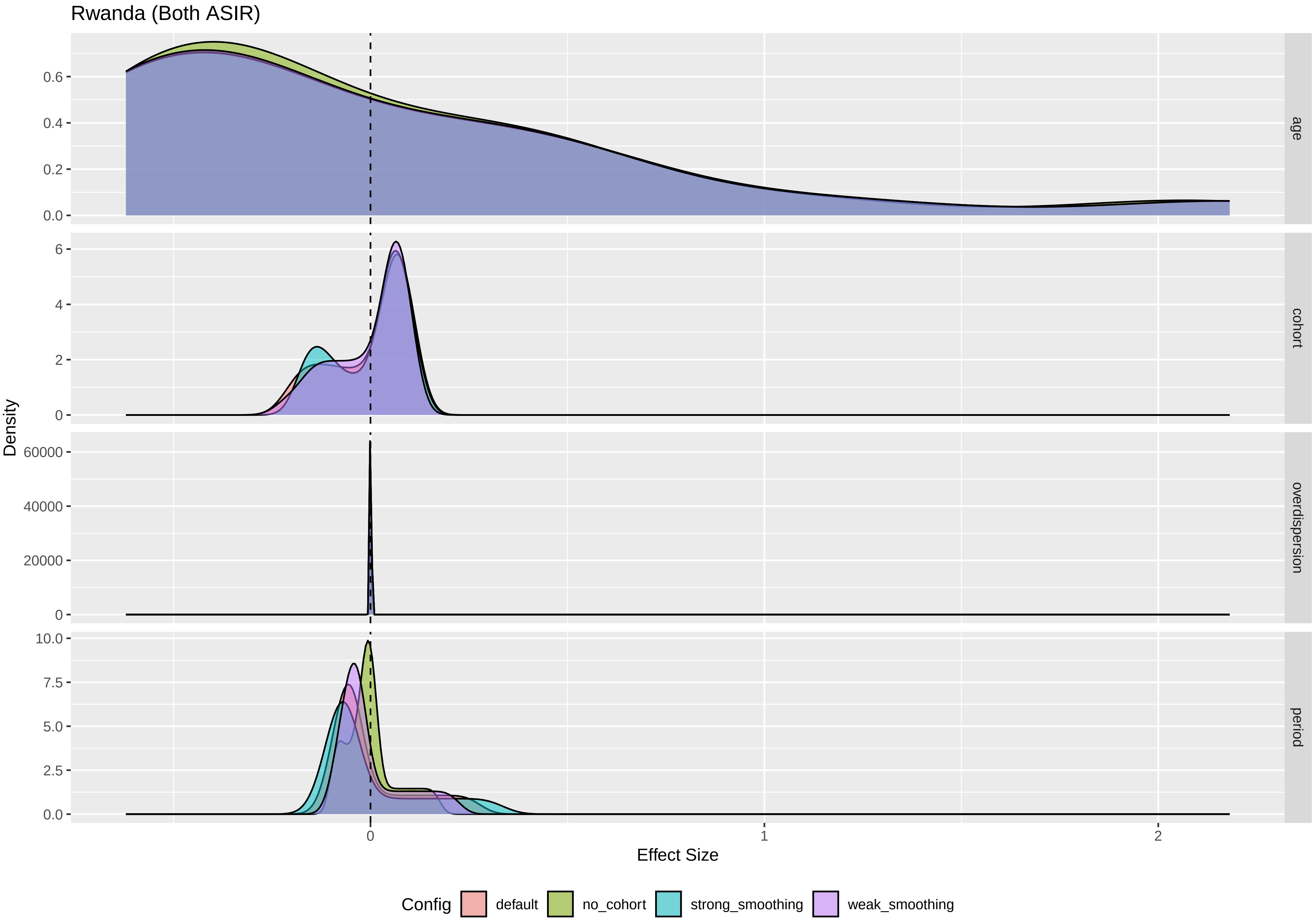

Rwanda (Both ASIR)
age
cohort
overdispersion
period
Density
Effect Size
0.0
0.2
0.4
0.6
0
2
4
6
0
20000
40000
60000
0.0
2.5
5.0
7.5
10.0
0
1
2
Config
default
no_cohort
strong_smoothing
weak_smoothing

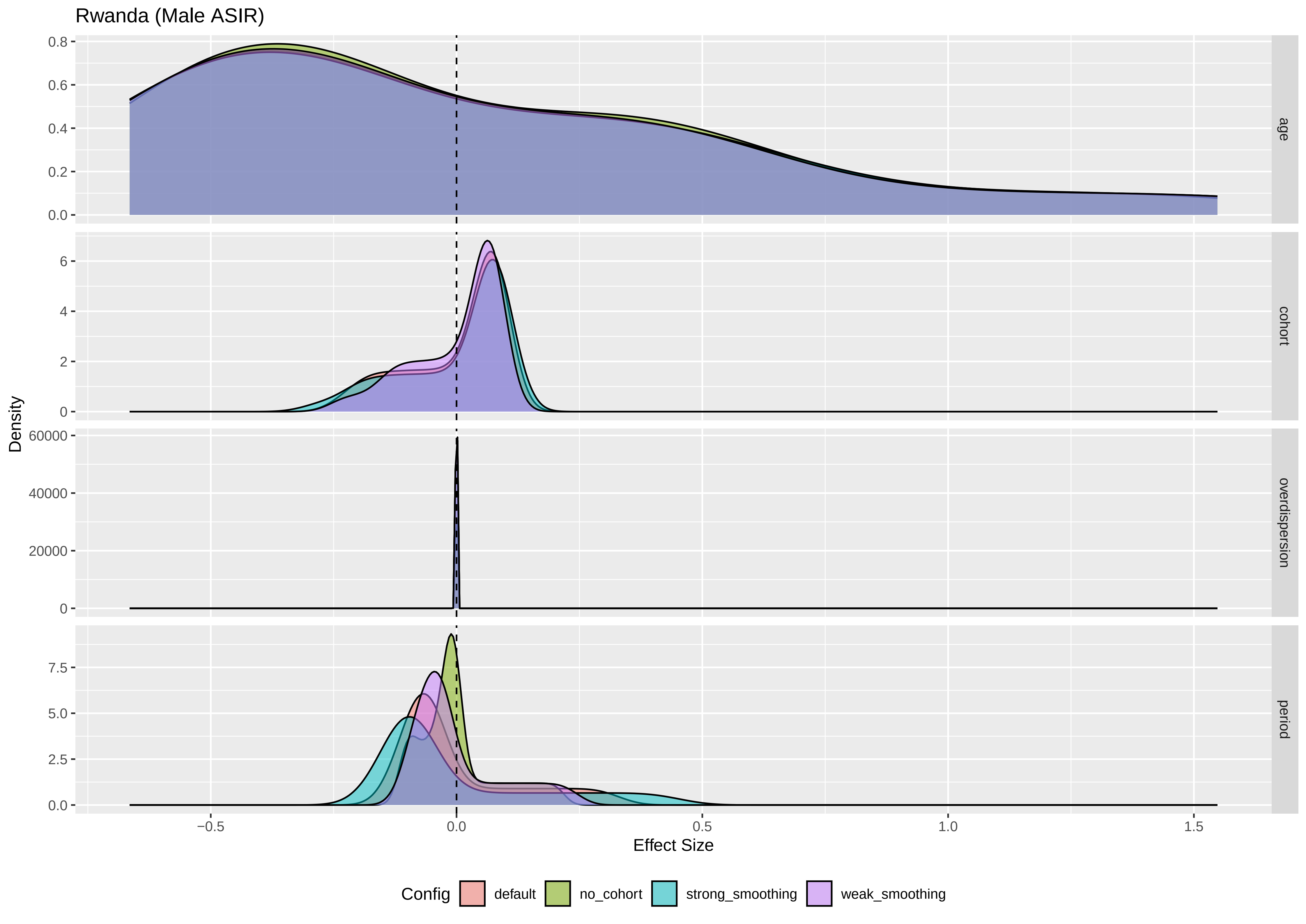

Rwanda (Male ASIR)
Density
Effect Size
age
cohort
overdispersion
period
0.0
0.2
0.4
0.6
0.8
0
2
4
6
20000
40000
60000
2.5
5.0
7.5
−0.5
0.0
0.5
1.0
1.5
Config
default
no_cohort
strong_smoothing
weak_smoothing

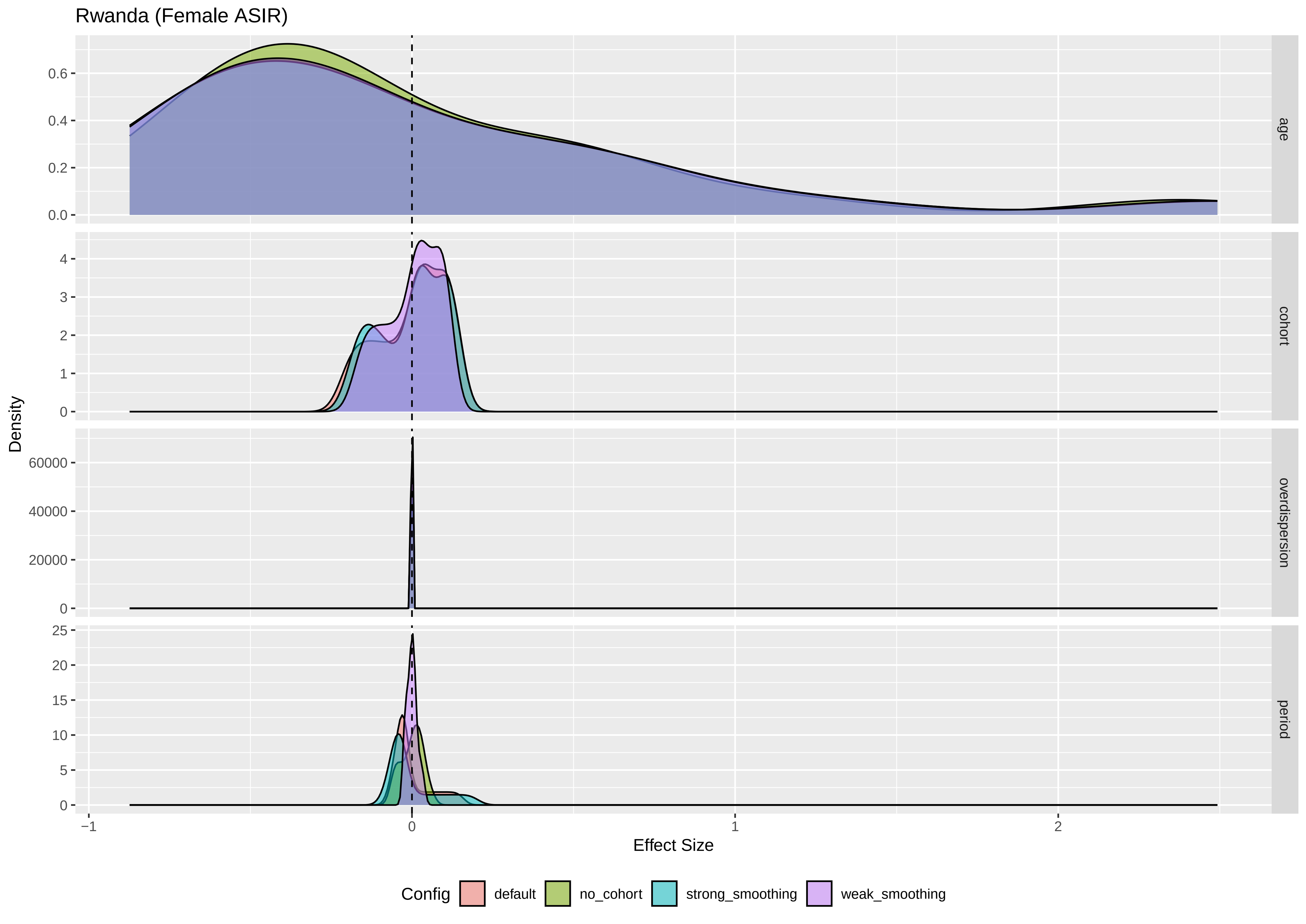

Rwanda (Female ASIR)
0.6
0.4
0.2
0.0
age
4
3
2
1
0
cohort
60000
40000
20000
0
overdispersion
25
20
15
10
5
0
period
−1
0
1
2
Effect Size
Density
Config
default
no_cohort
strong_smoothing
weak_smoothing

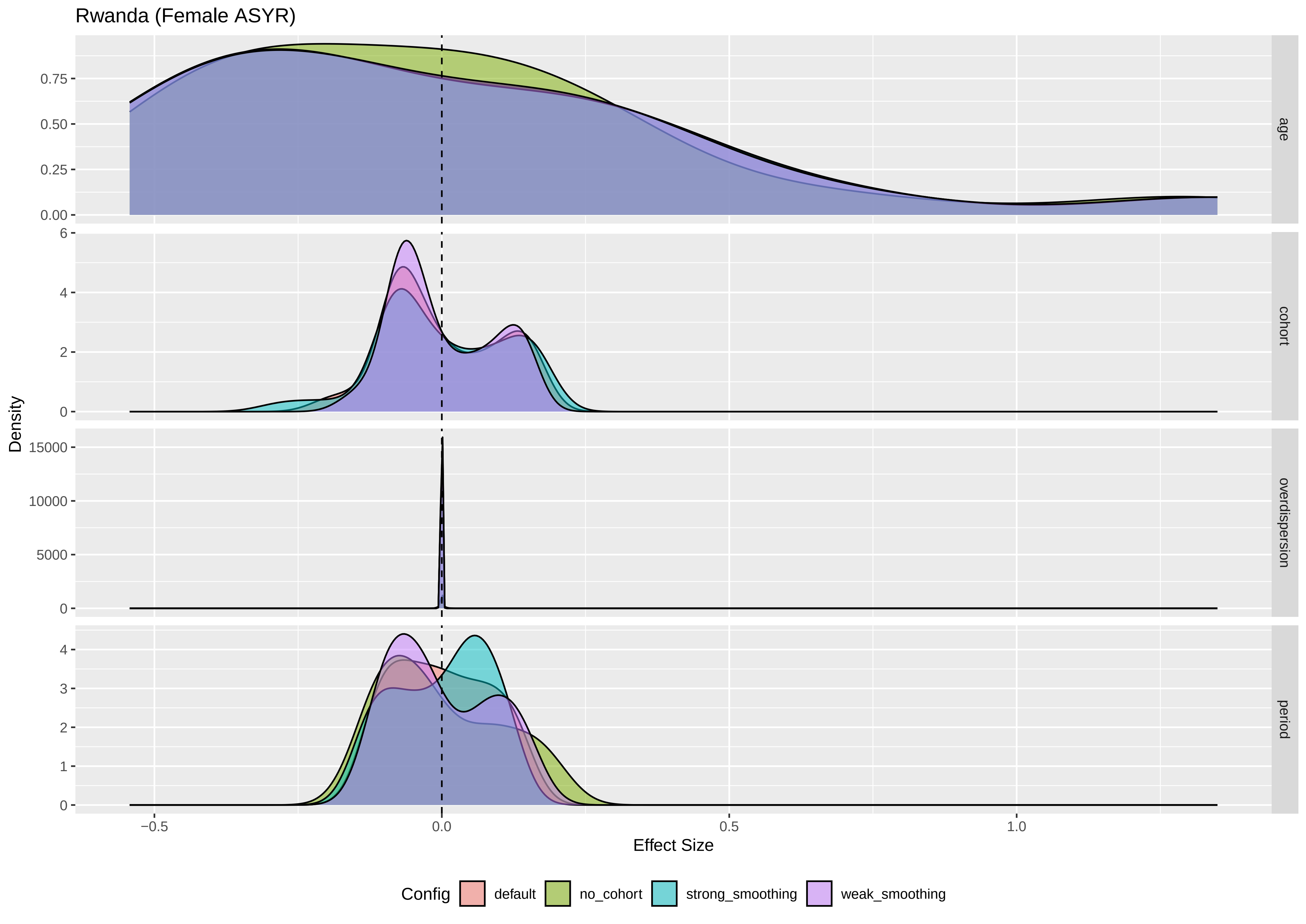
Rwanda (Female ASYR)
age
cohort
overdispersion
period
Density
Effect Size
0.00
0.25
0.50
0.75
0
2
4
6
5000
10000
15000
1
3
−0.5
0.0
0.5
1.0
Config
default
no_cohort
strong_smoothing
weak_smoothing

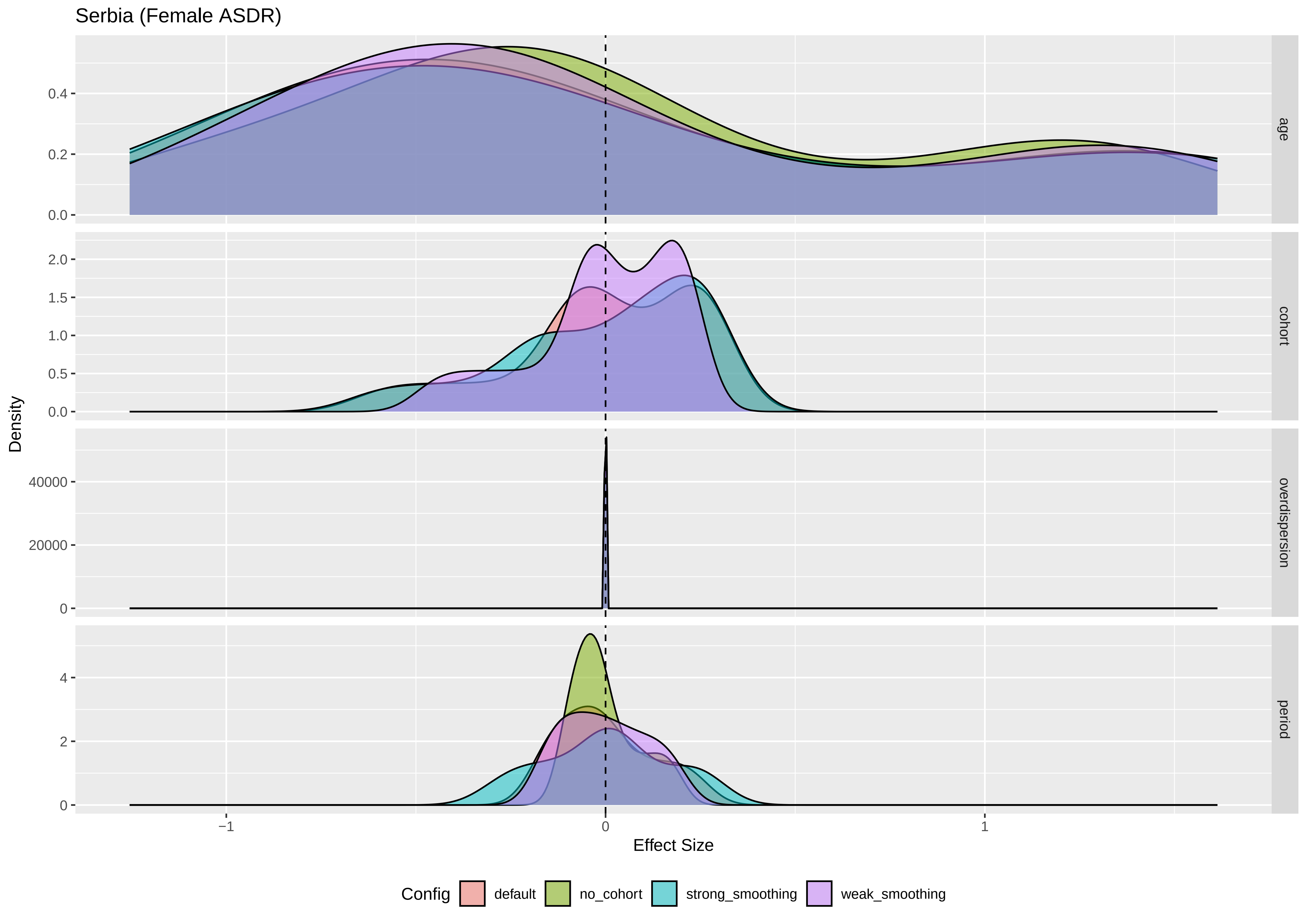

Serbia (Female ASDR)
Density
0.0
0.2
0.4
0.0
0.5
1.0
1.5
2.0
0
20000
40000
0
2
4
age
cohort
overdispersion
period
−1
0
1
Effect Size
Config
default
no_cohort
strong_smoothing
weak_smoothing

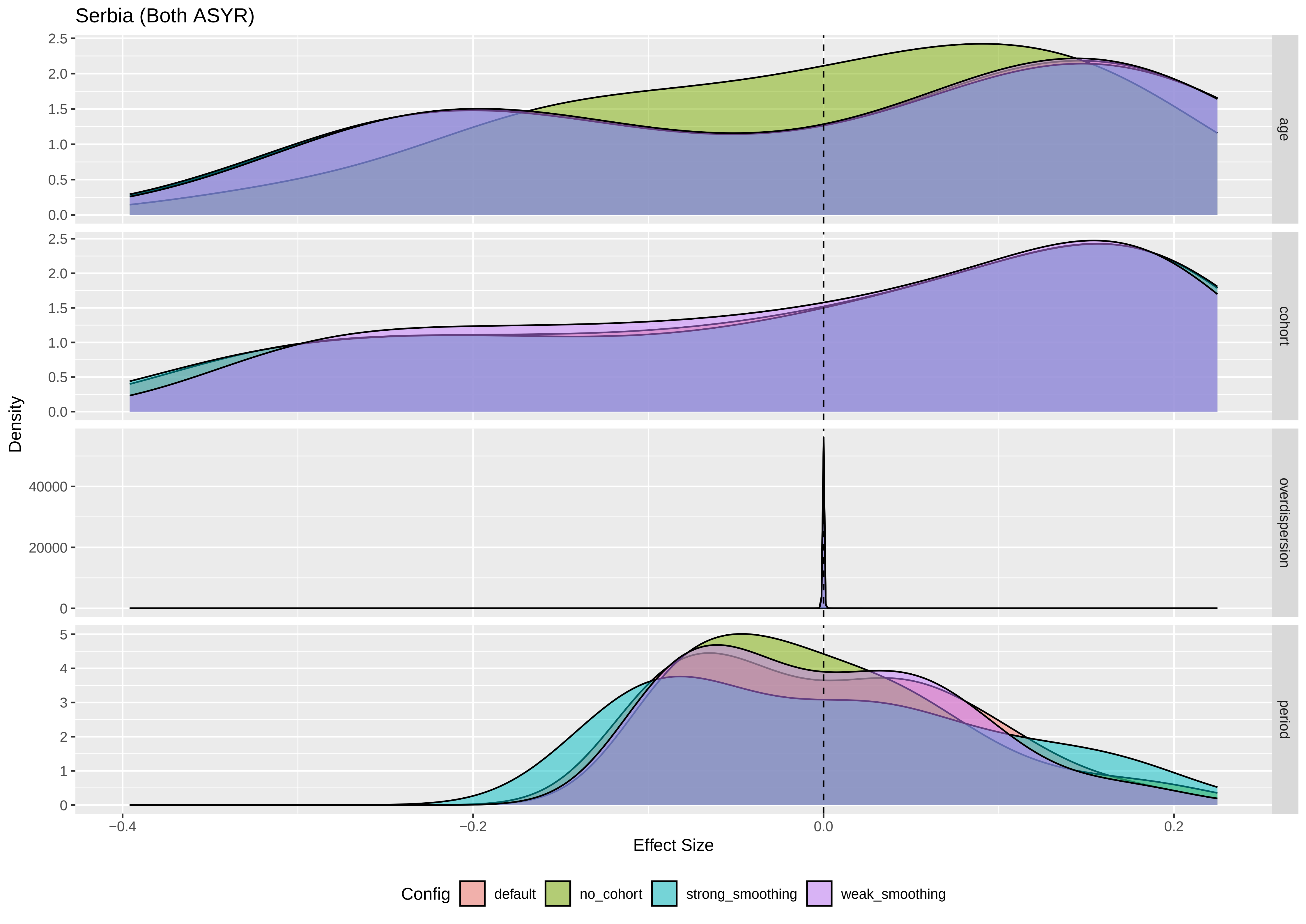
Serbia (Both ASYR)
Density
Effect Size
age
cohort
overdispersion
period
2.5
2.0
1.5
1.0
0.5
0.0
40000
20000
0
5
4
3
2
1
−0.4
−0.2
0.0
0.2
Config
default
no_cohort
strong_smoothing
weak_smoothing

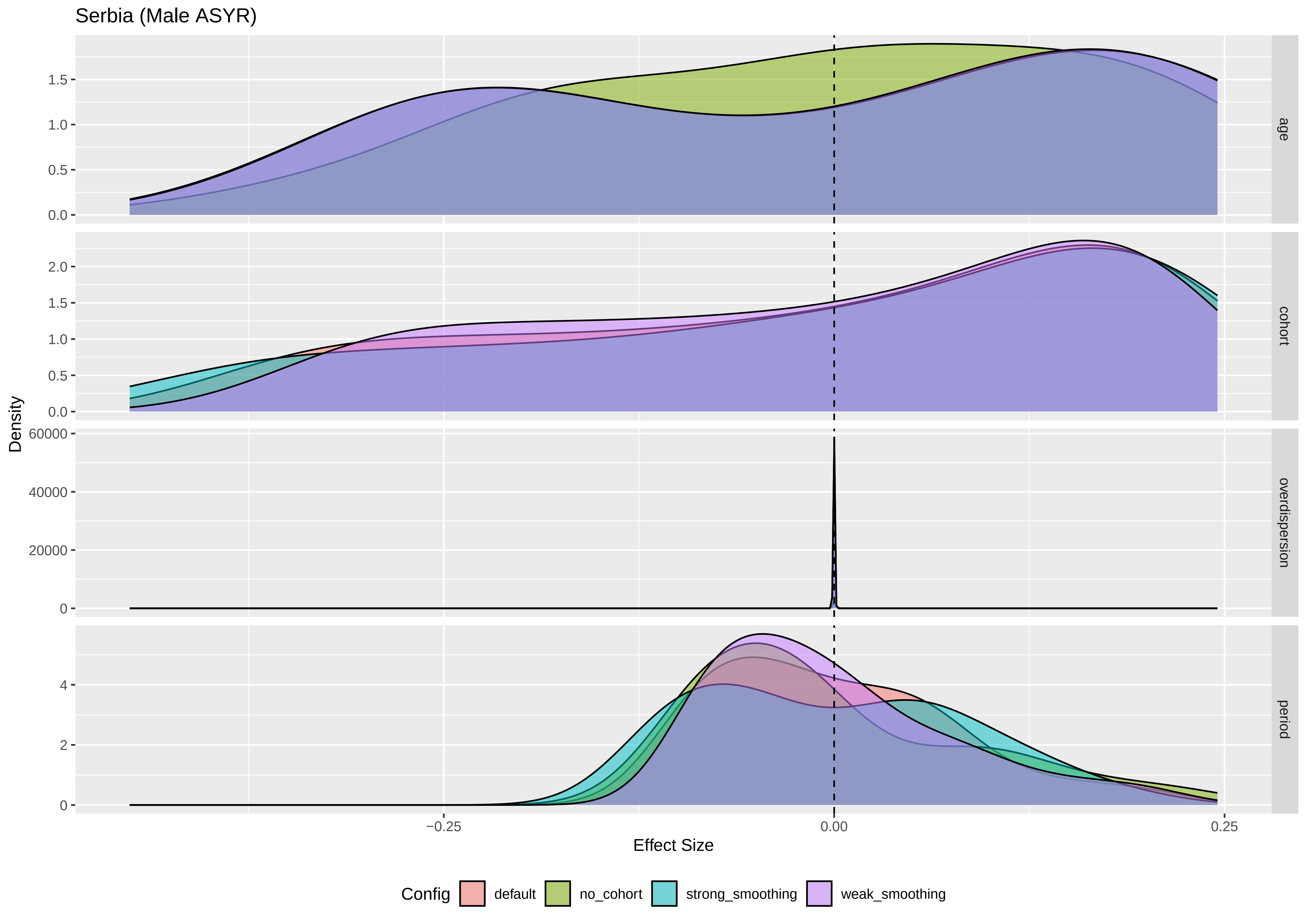

Serbia (Male ASYR)
age
cohort
overdispersion
period
Density
Effect Size
1.5
1.0
0.5
0.0
2.0
60000
40000
20000
0
4
2
−0.25
0.00
0.25
Config
default
no_cohort
strong_smoothing
weak_smoothing

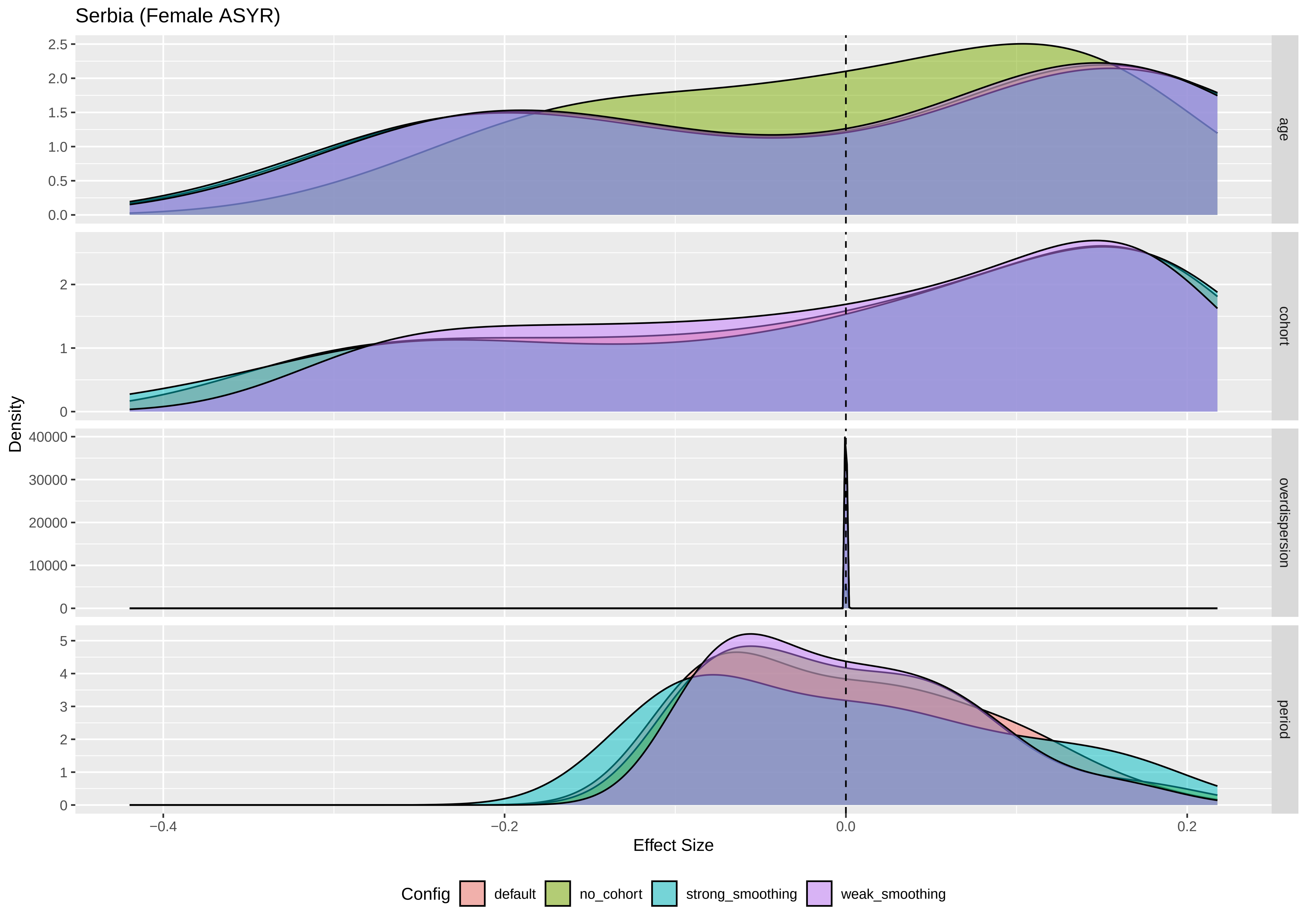

Serbia (Female ASYR)
Density
Effect Size
age
cohort
overdispersion
period
2.5
2.0
1.5
1.0
0.5
0.0
2
1
0
40000
30000
20000
10000
0
5
4
3
2
1
0
−0.4
−0.2
0.0
0.2
Config
default
no_cohort
strong_smoothing
weak_smoothing

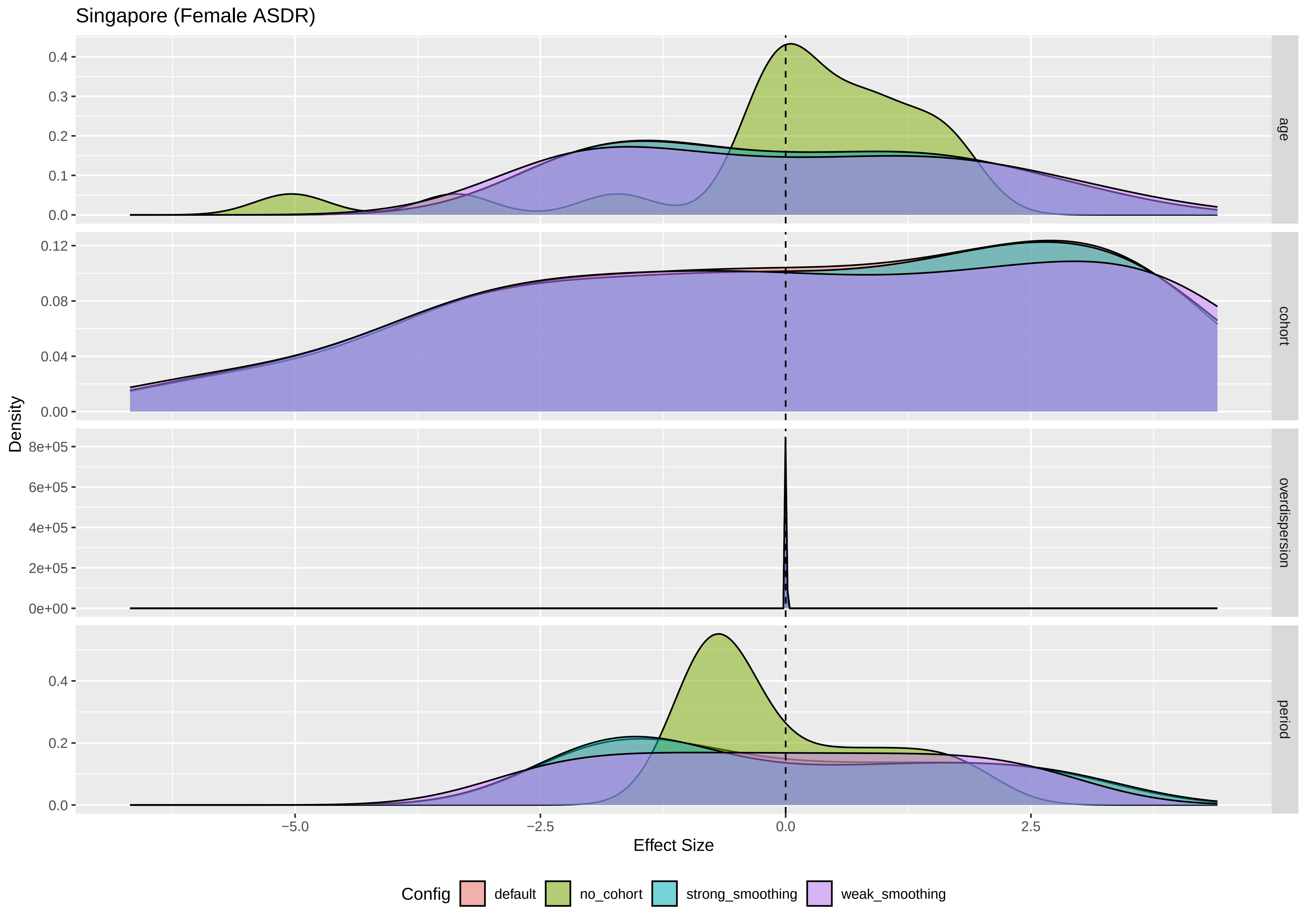

Singapore (Female ASDR)
Density
Effect Size
age
cohort
overdispersion
period
0.0
0.1
0.2
0.3
0.4
0.00
0.04
0.08
0.12
0e+00
2e+05
4e+05
6e+05
8e+05
−5.0
−2.5
0.0
2.5
Config
default
no_cohort
strong_smoothing
weak_smoothing

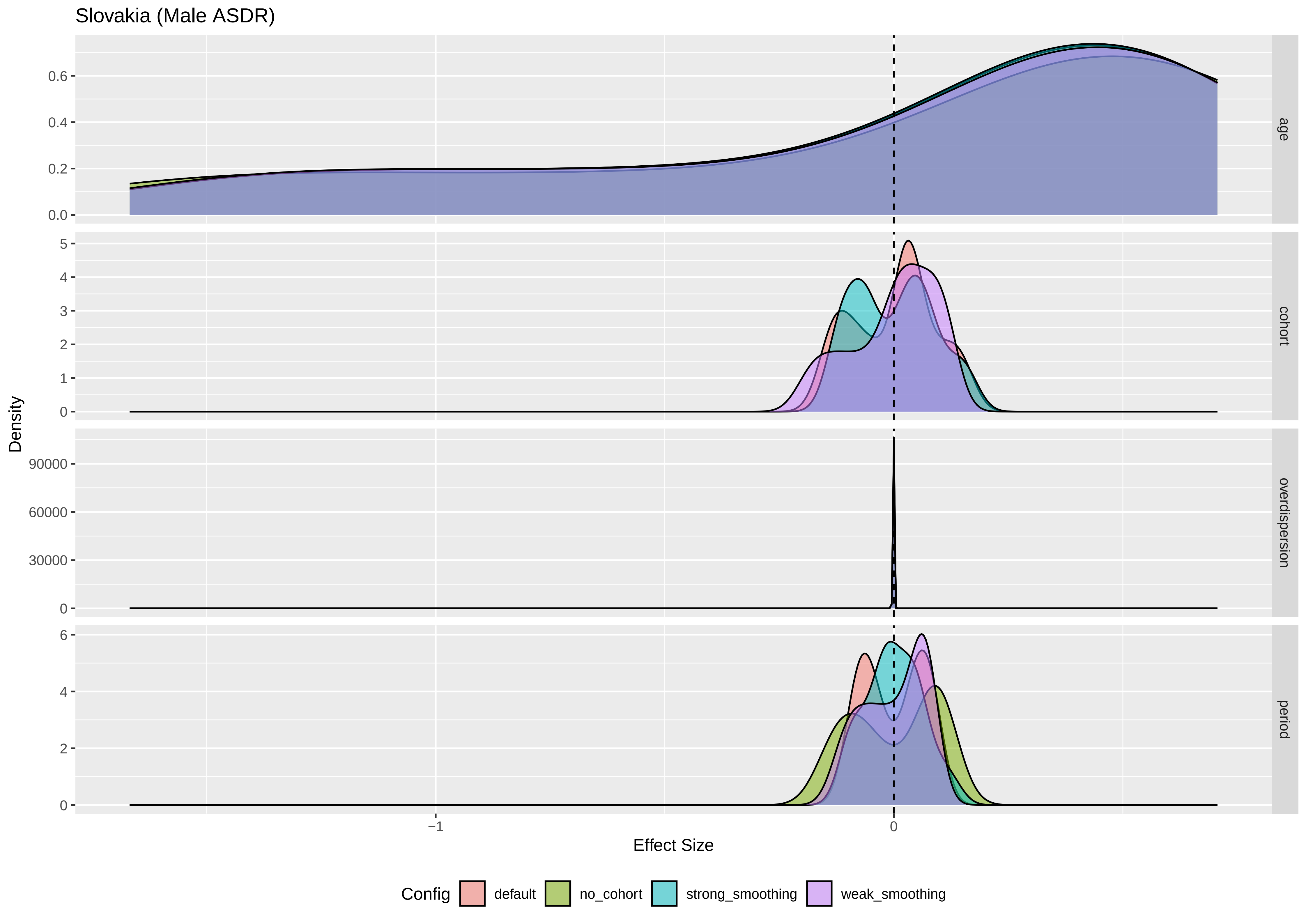

Slovakia (Male ASDR)
Density
Effect Size
age
cohort
overdispersion
period
0.0
0.2
0.4
0.6
0
1
2
3
4
5
0
30000
60000
90000
0
2
4
6
−1
0
Config
default
no_cohort
strong_smoothing
weak_smoothing

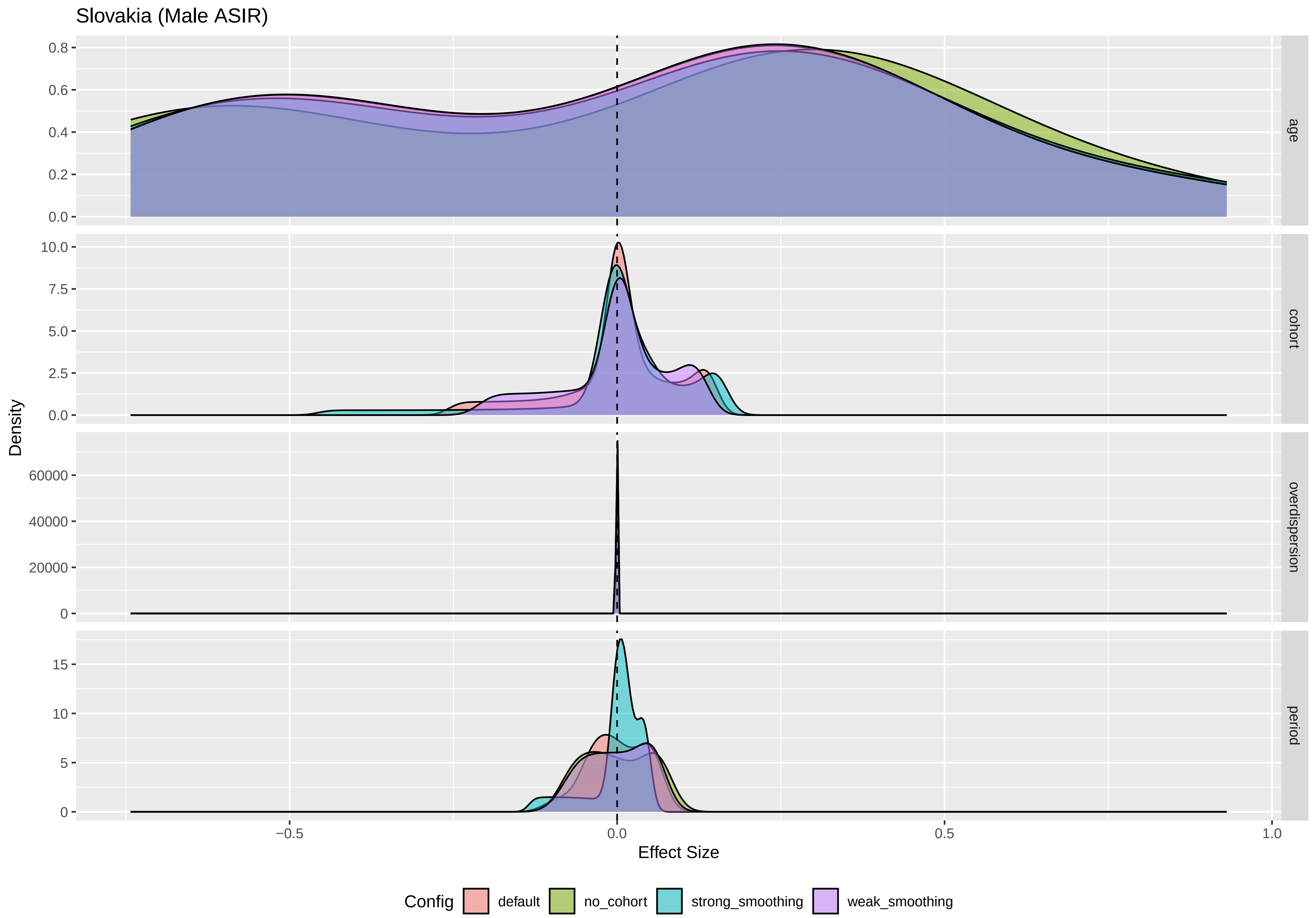
Slovakia (Male ASIR)
Density
Effect Size
age
cohort
overdispersion
period
0.0
0.2
0.4
0.6
0.8
10.0
7.5
5.0
2.5
60000
40000
20000
0
15
10
5
−0.5
0.0
0.5
1.0
Config
default
no_cohort
strong_smoothing
weak_smoothing

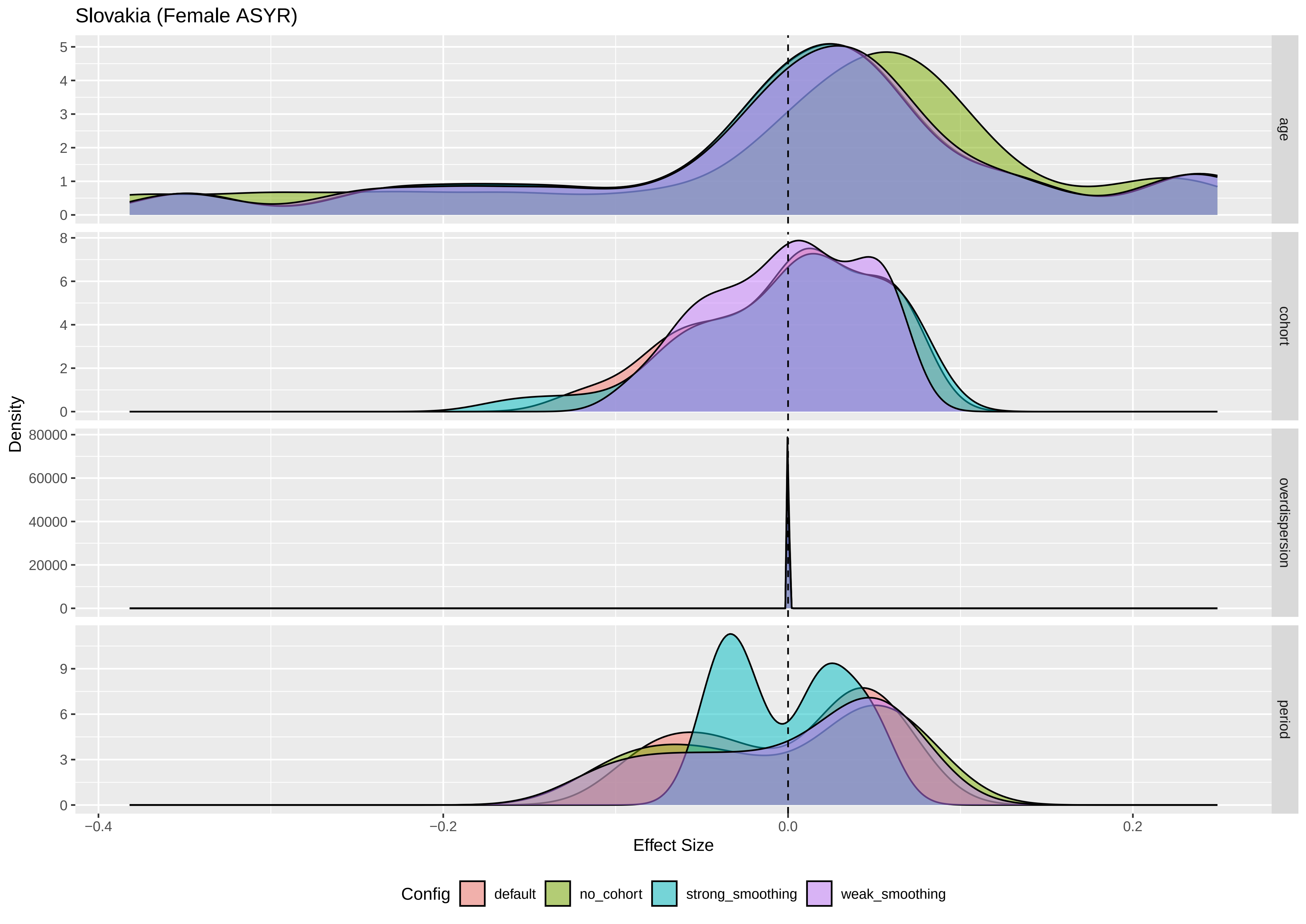

Slovakia (Female ASYR)
Density
Effect Size
age
cohort
overdispersion
period
0
1
2
3
4
5
0
2
4
6
8
0
20000
40000
60000
80000
0
3
6
9
−0.4
−0.2
0.0
0.2
Config
default
no_cohort
strong_smoothing
weak_smoothing

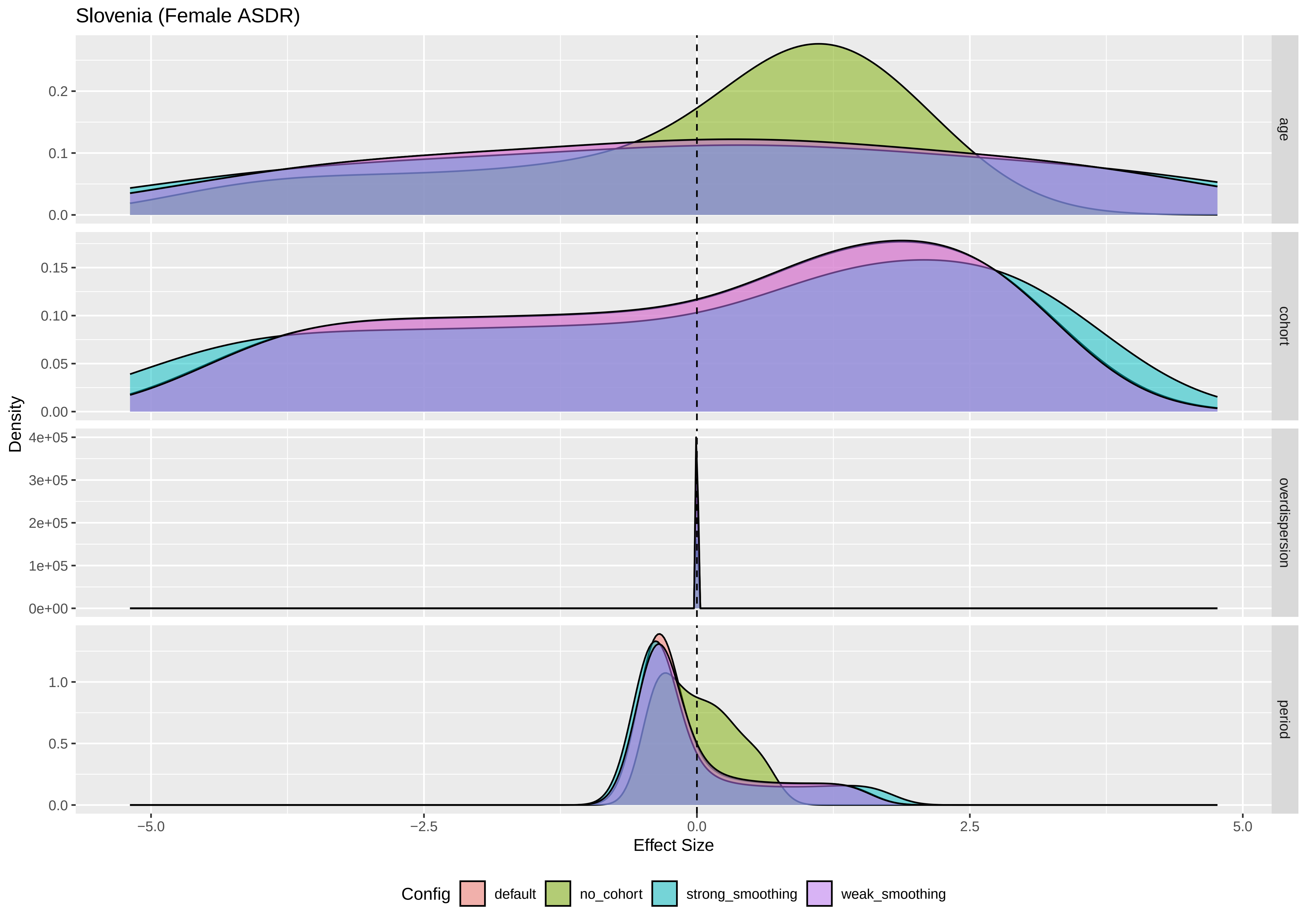

Slovenia (Female ASDR)
Density
Effect Size
age
cohort
overdispersion
period
0.0
0.1
0.2
0.00
0.05
0.10
0.15
0e+00
1e+05
2e+05
3e+05
4e+05
0.5
1.0
−5.0
−2.5
0.0
2.5
5.0
Config
default
no_cohort
strong_smoothing
weak_smoothing

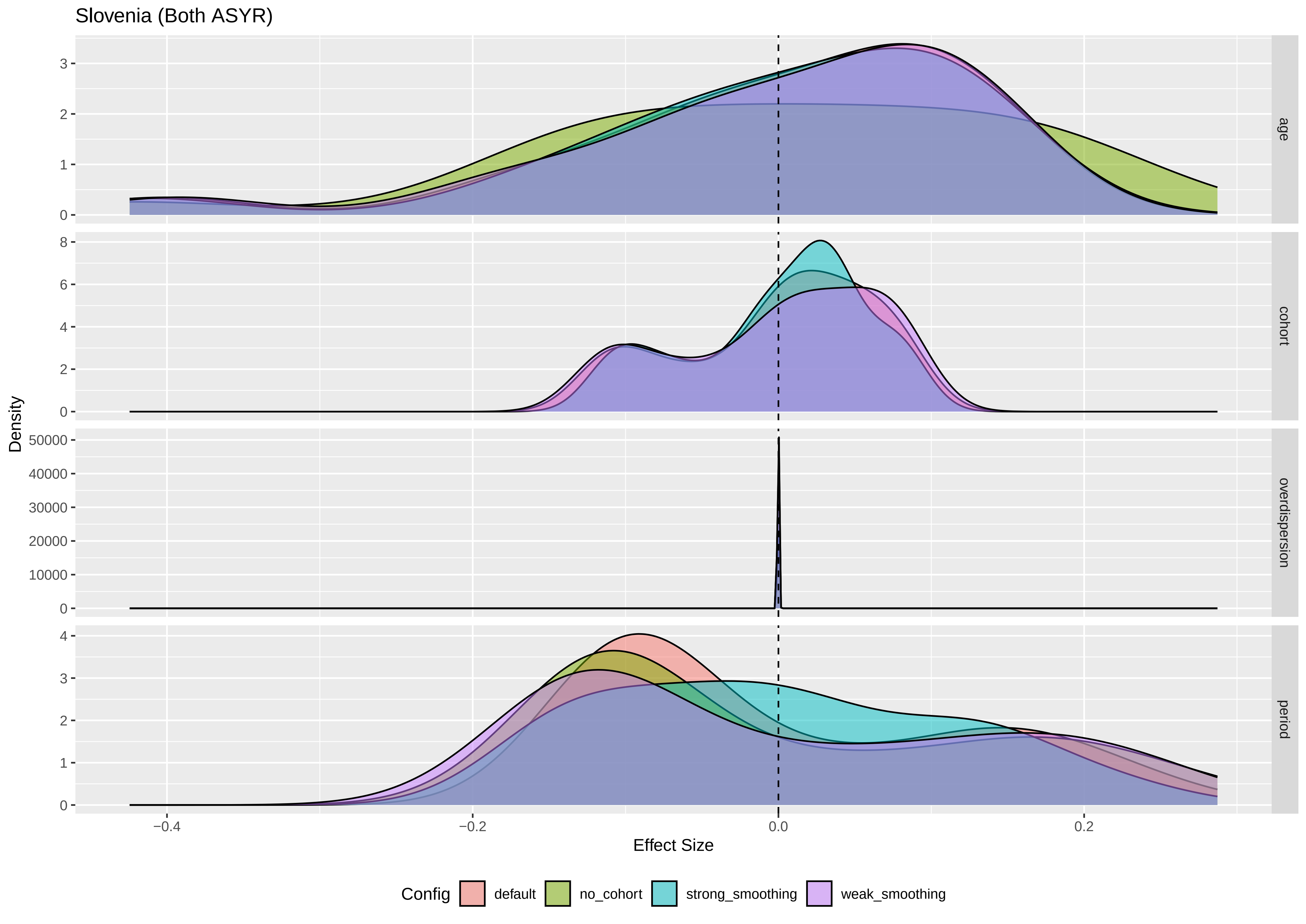

Slovenia (Both ASYR)
Density
Effect Size
age
cohort
overdispersion
period
−0.4
−0.2
0.0
0.2
0
1
2
3
4
6
8
10000
20000
30000
40000
50000
Config
default
no_cohort
strong_smoothing
weak_smoothing

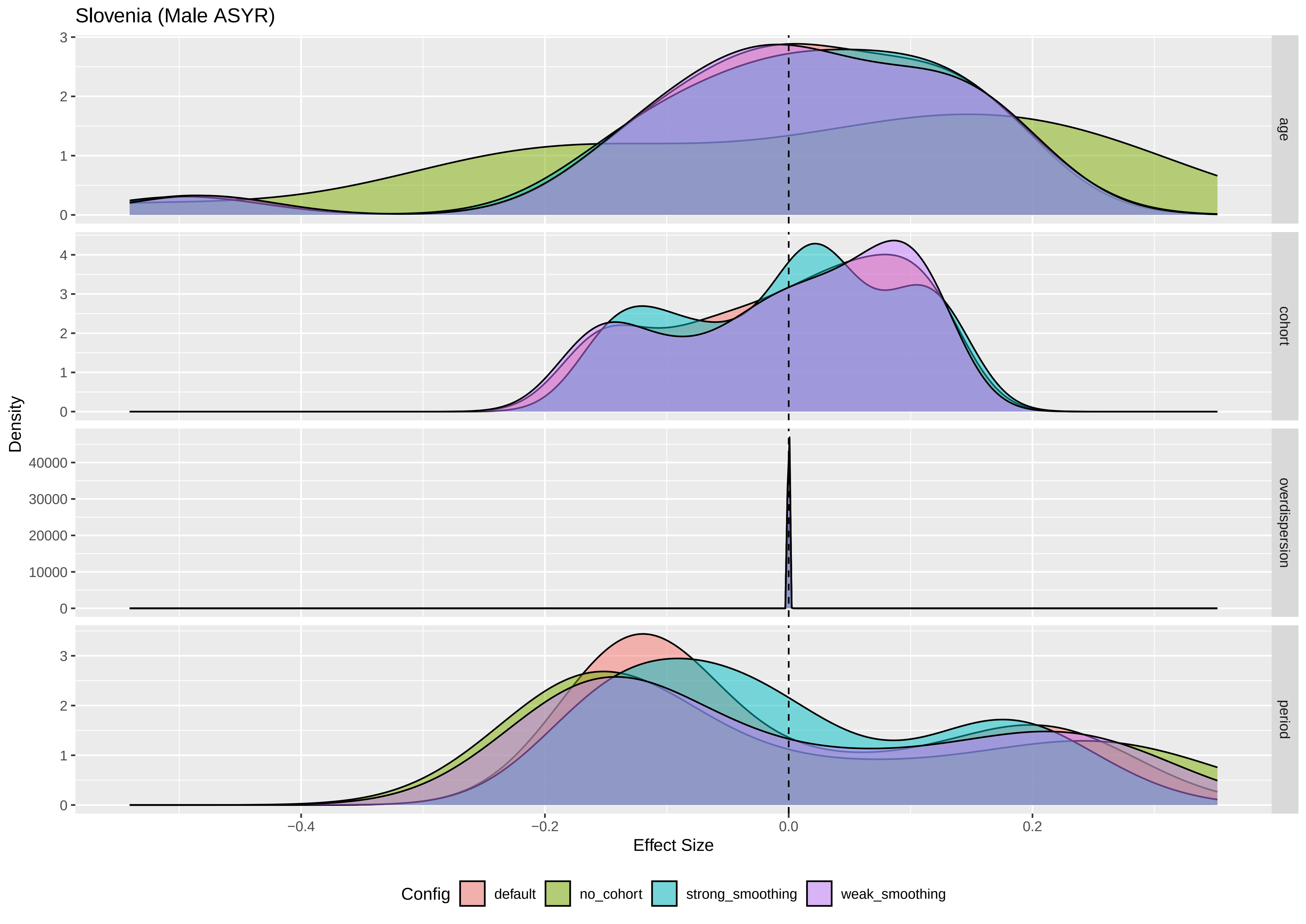
Slovenia (Male ASYR)
Density
Effect Size
age
cohort
overdispersion
period
−0.4
−0.2
0.0
0.2
0
1
2
3
4
10000
20000
30000
40000
Config
default
no_cohort
strong_smoothing
weak_smoothing

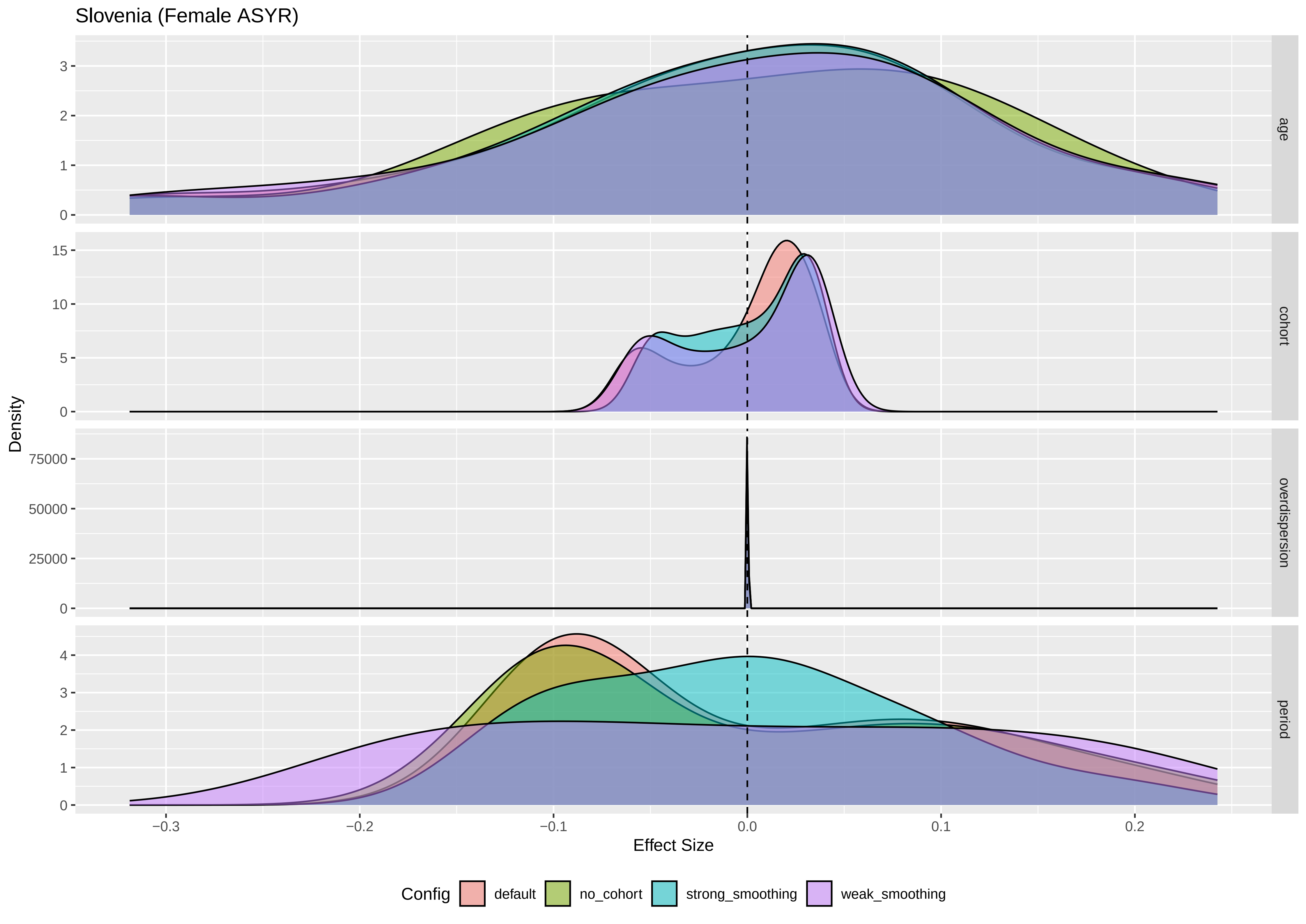

Slovenia (Female ASYR)
Density
Effect Size
age
cohort
overdispersion
period
3
2
1
0
15
10
5
0
75000
50000
25000
0
4
3
2
1
0
−0.3
−0.2
−0.1
0.0
0.1
0.2
Config
default
no_cohort
strong_smoothing
weak_smoothing

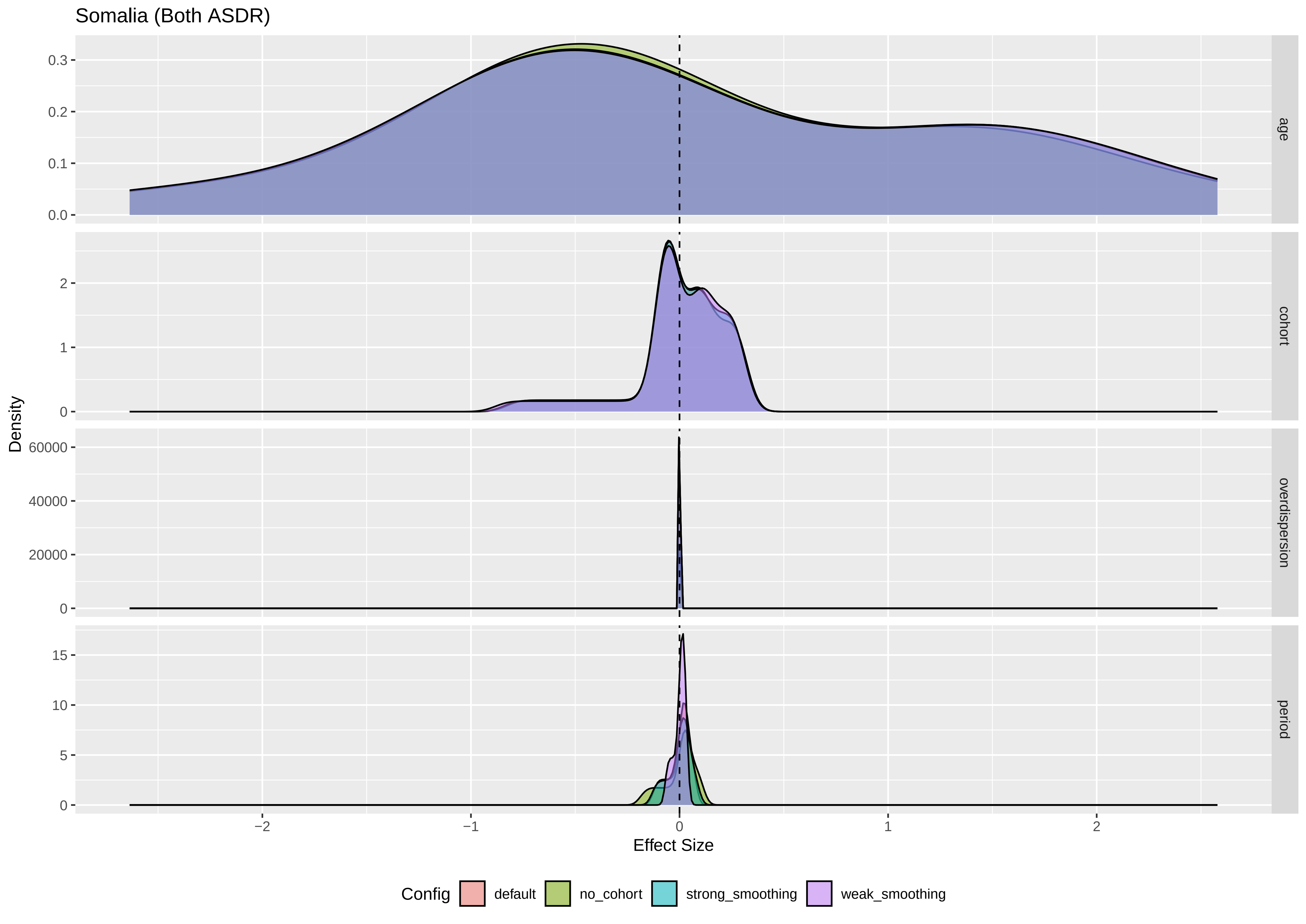

Somalia (Both ASDR)
Density
Effect Size
age
cohort
overdispersion
period
0.0
0.1
0.2
0.3
0
1
2
0
20000
40000
60000
0
5
10
15
−2
−1
0
1
2
Config
default
no_cohort
strong_smoothing
weak_smoothing

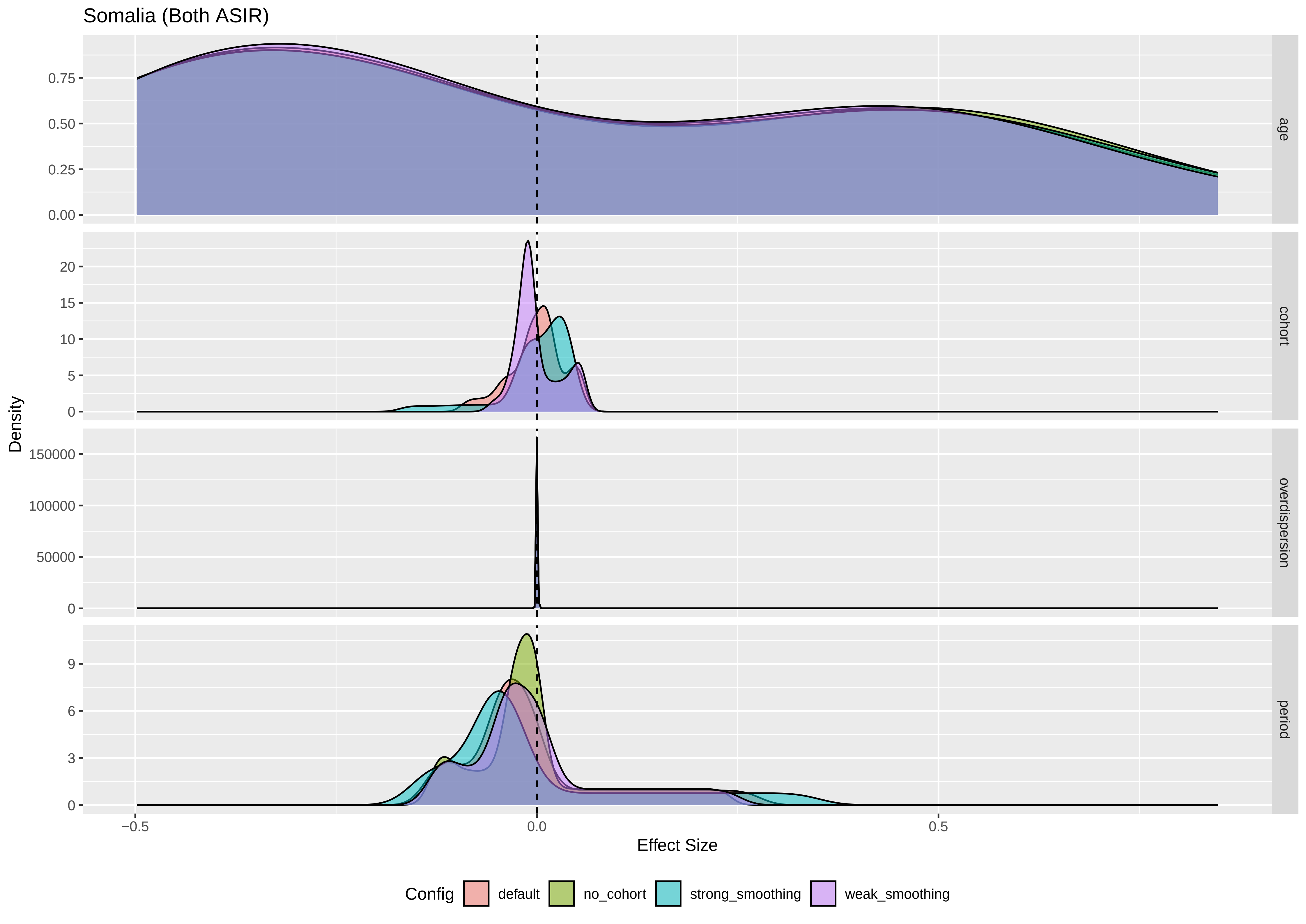

Somalia (Both ASIR)
0.75
0.50
0.25
0.00
age
20
15
10
5
0
cohort
Density
150000
100000
50000
0
overdispersion
9
6
3
0
period
−0.5
0.0
0.5
Effect Size
Config
default
no_cohort
strong_smoothing
weak_smoothing

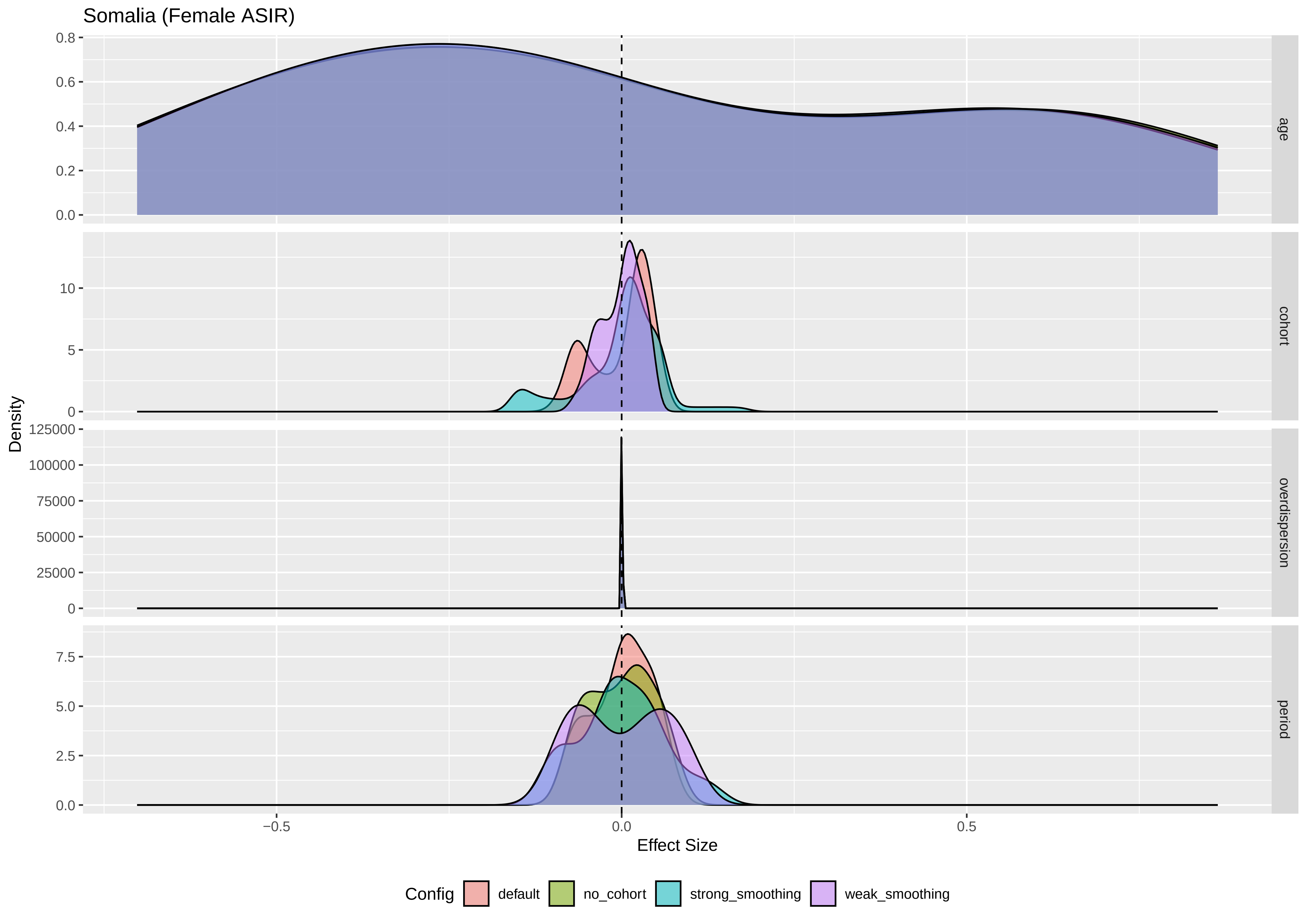
Somalia (Female ASIR)
Density
Effect Size
age
cohort
overdispersion
period
0.8
0.6
0.4
0.2
0.0
10
5
0
125000
100000
75000
50000
25000
0
7.5
5.0
2.5
0.0
−0.5
0.0
0.5
Config
default
no_cohort
strong_smoothing
weak_smoothing

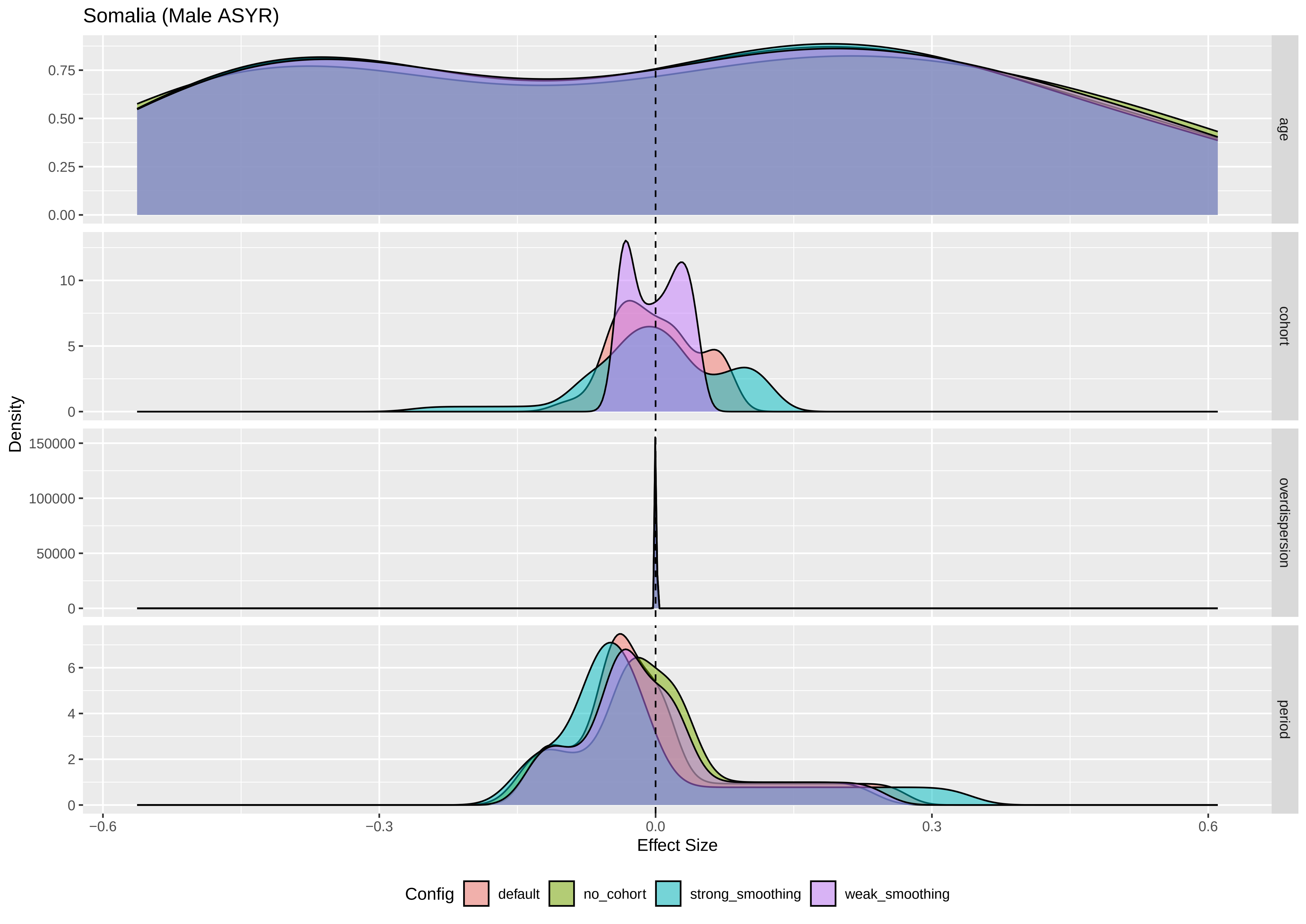

Somalia (Male ASYR)
0.75
0.50
0.25
0.00
age
10
5
0
cohort
150000
100000
50000
0
overdispersion
6
4
2
0
period
Density
−0.6
−0.3
0.0
0.3
0.6
Effect Size
Config
default
no_cohort
strong_smoothing
weak_smoothing

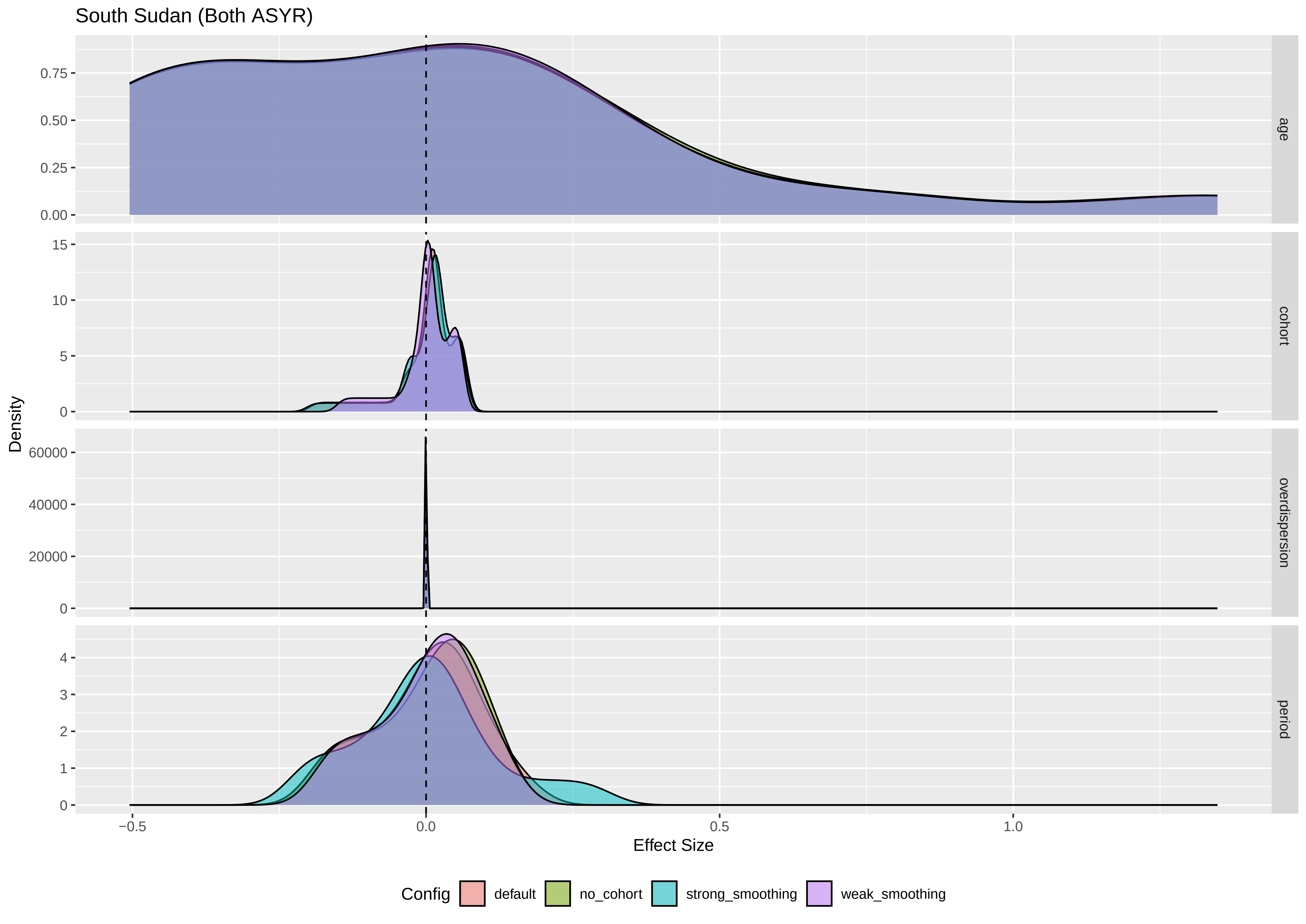
South Sudan (Both ASYR)
age
cohort
overdispersion
period
Density
Effect Size
0.75
0.50
0.25
0.00
15
10
5
0
60000
40000
20000
0
4
3
2
1
0
−0.5
0.0
0.5
1.0
Config
default
no_cohort
strong_smoothing
weak_smoothing

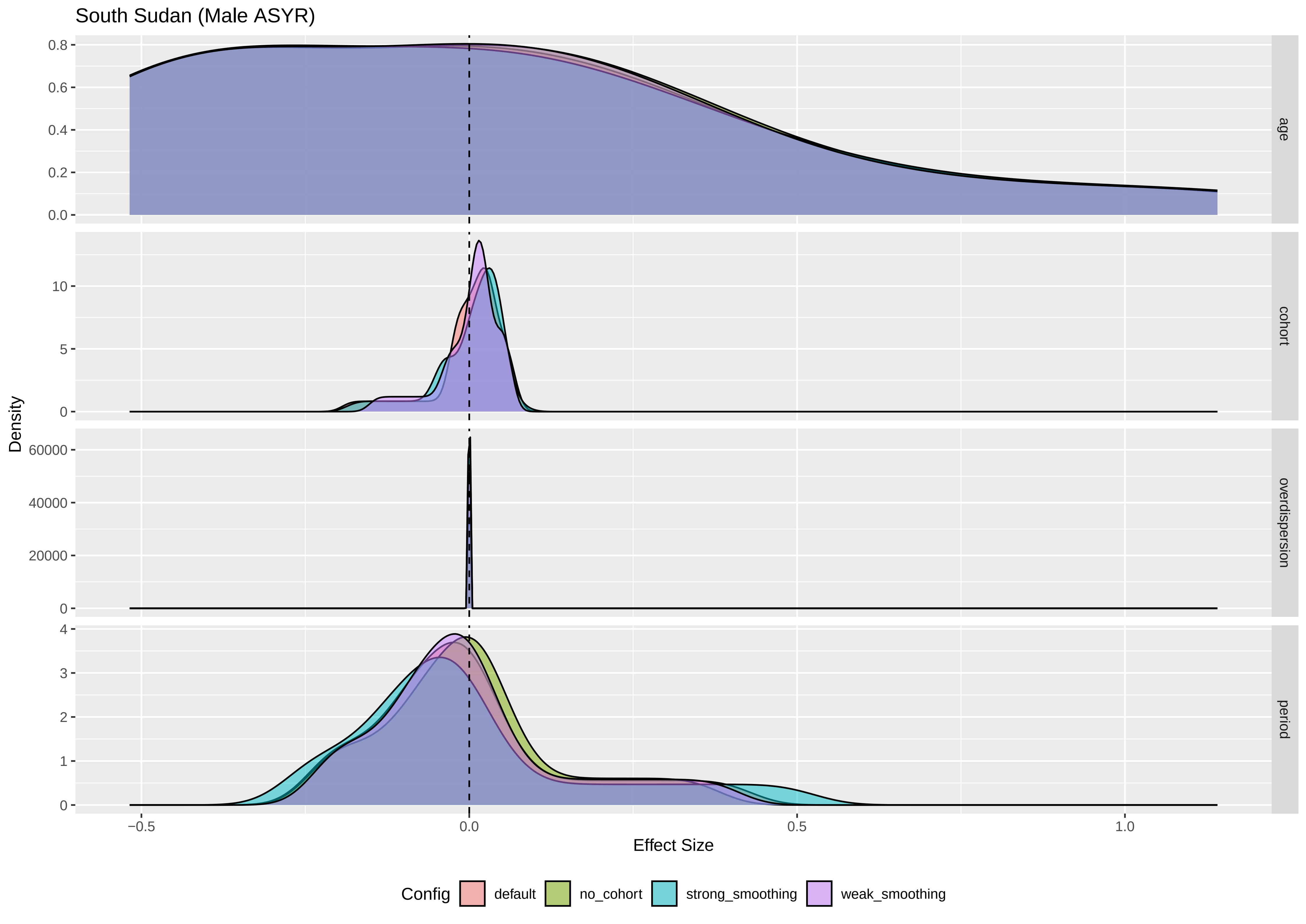

South Sudan (Male ASYR)
Density
Effect Size
age
cohort
overdispersion
period
0.0
0.2
0.4
0.6
0.8
0
5
10
20000
40000
60000
1
2
3
4
−0.5
0.0
0.5
1.0
Config
default
no_cohort
strong_smoothing
weak_smoothing

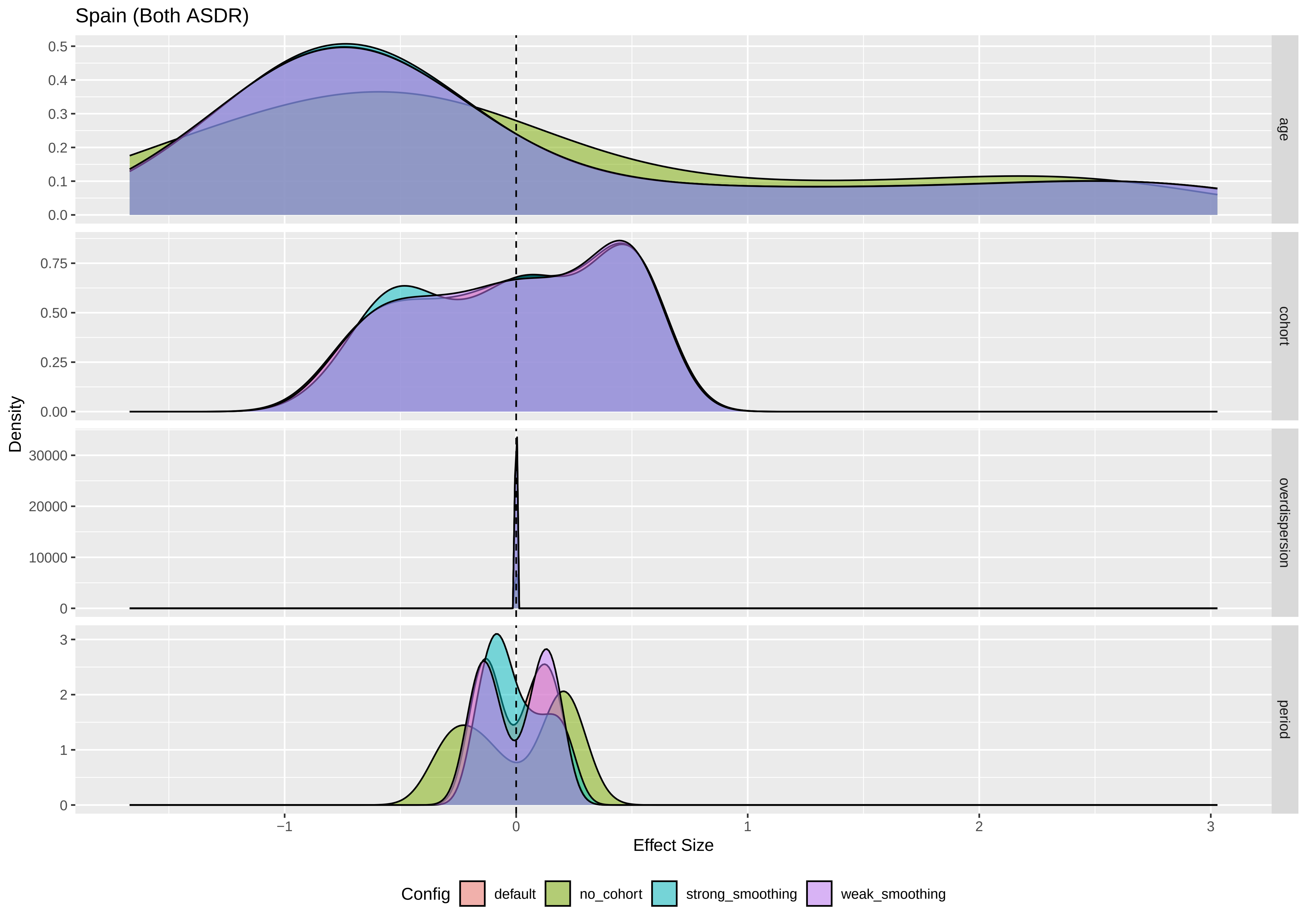

Spain (Both ASDR)
Density
age
cohort
overdispersion
period
0.0
0.1
0.2
0.3
0.4
0.5
0.00
0.25
0.50
0.75
0
10000
20000
30000
0
1
2
3
−1
0
1
2
3
Effect Size
Config
default
no_cohort
strong_smoothing
weak_smoothing

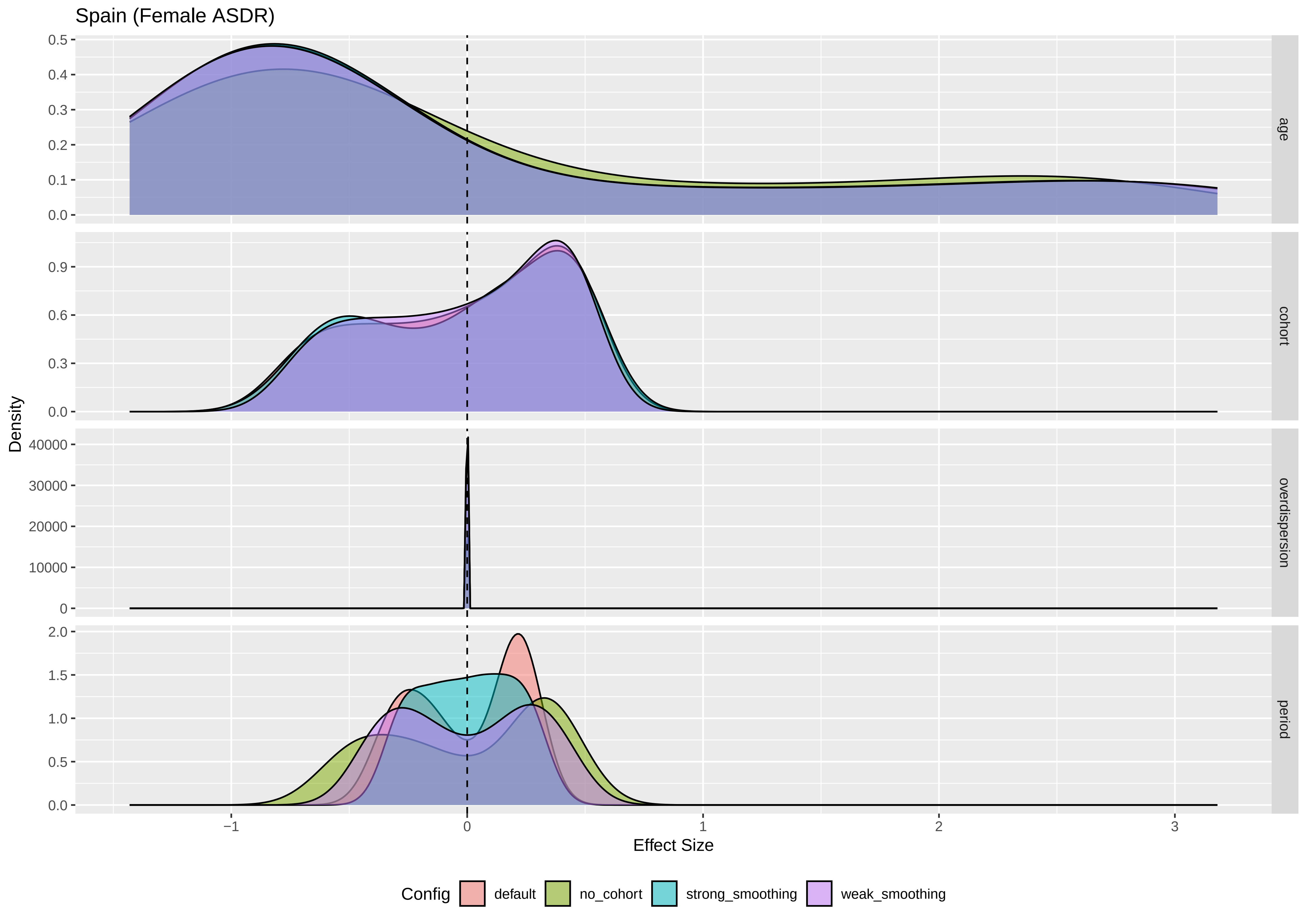

Spain (Female ASDR)
Density
Effect Size
age
cohort
overdispersion
period
0.0
0.1
0.2
0.3
0.4
0.5
0.3
0.6
0.9
0
10000
20000
30000
40000
0.5
1.0
1.5
2.0
−1
0
1
2
3
Config
default
no_cohort
strong_smoothing
weak_smoothing

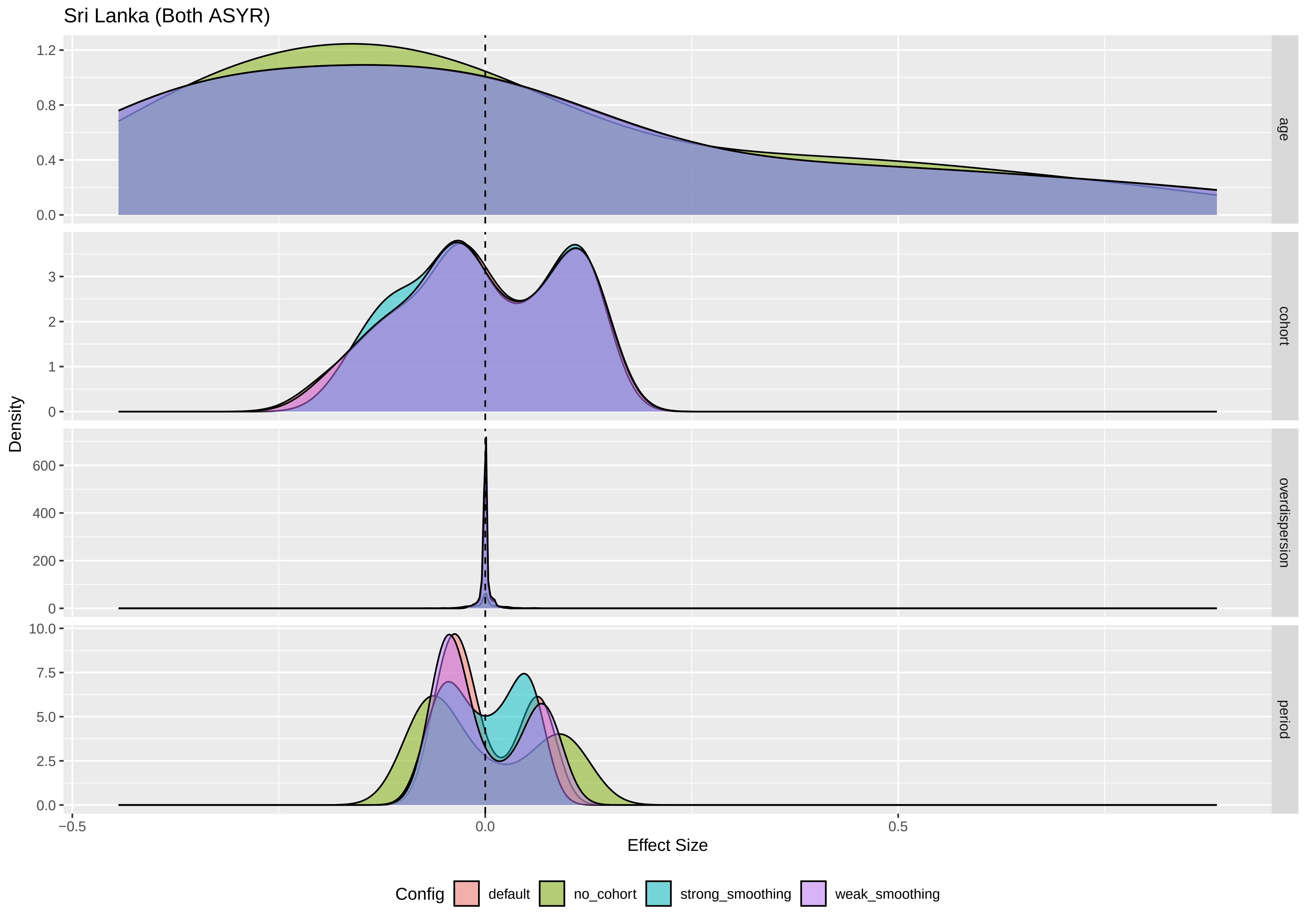
Sri Lanka (Both ASYR)
Density
Effect Size
age
cohort
overdispersion
period
1.2
0.8
0.4
0.0
3
2
1
0
600
400
200
0
10.0
7.5
5.0
2.5
0.0
-0.5
0.0
0.5
Config
default
no_cohort
strong_smoothing
weak_smoothing

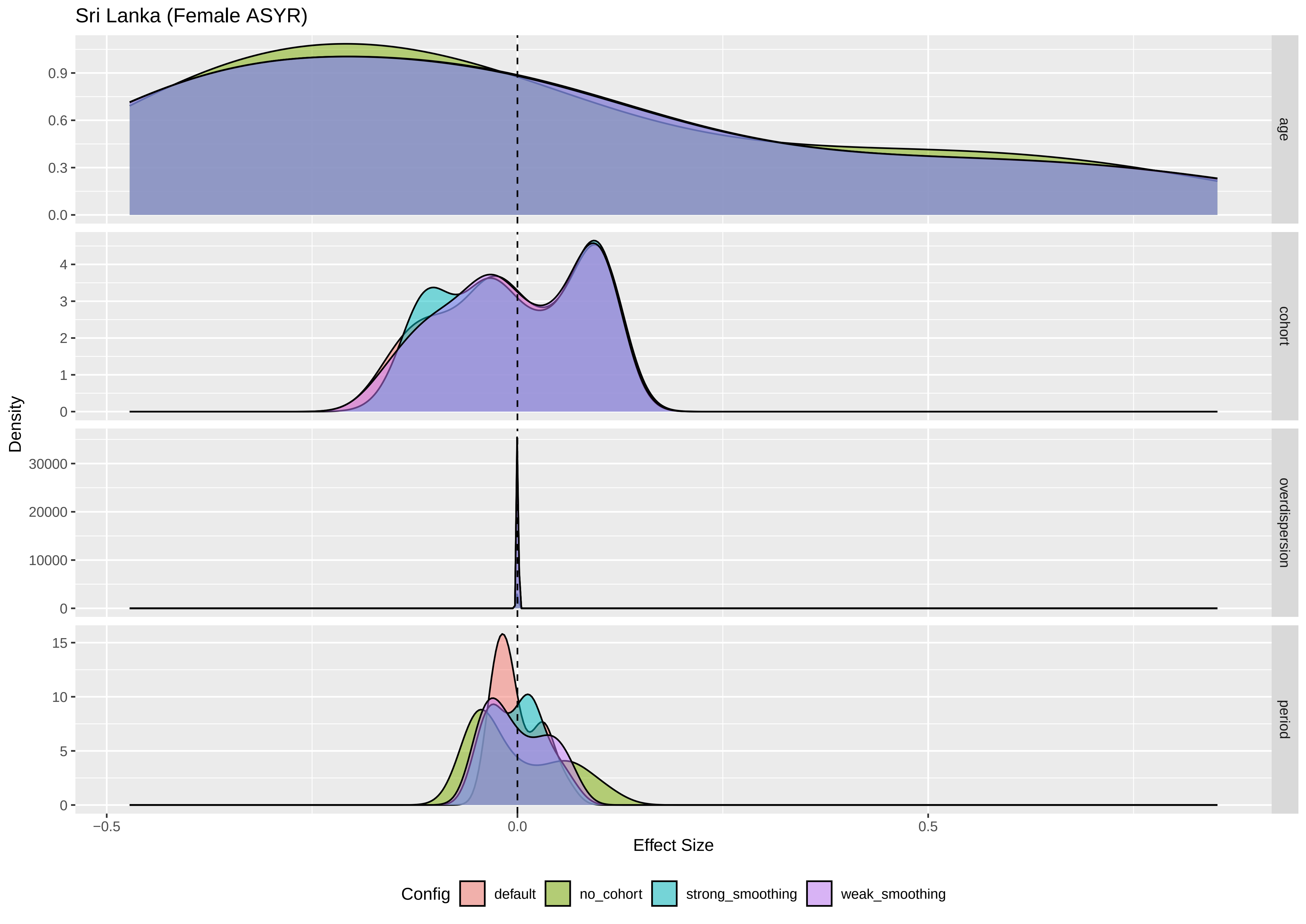

Sri Lanka (Female ASYR)
age
cohort
overdispersion
period
Density
Effect Size
-0.5
0.0
0.5
0.0
0.3
0.6
0.9
0
1
2
3
4
0
10000
20000
30000
0
5
10
15
Config
default
no_cohort
strong_smoothing
weak_smoothing

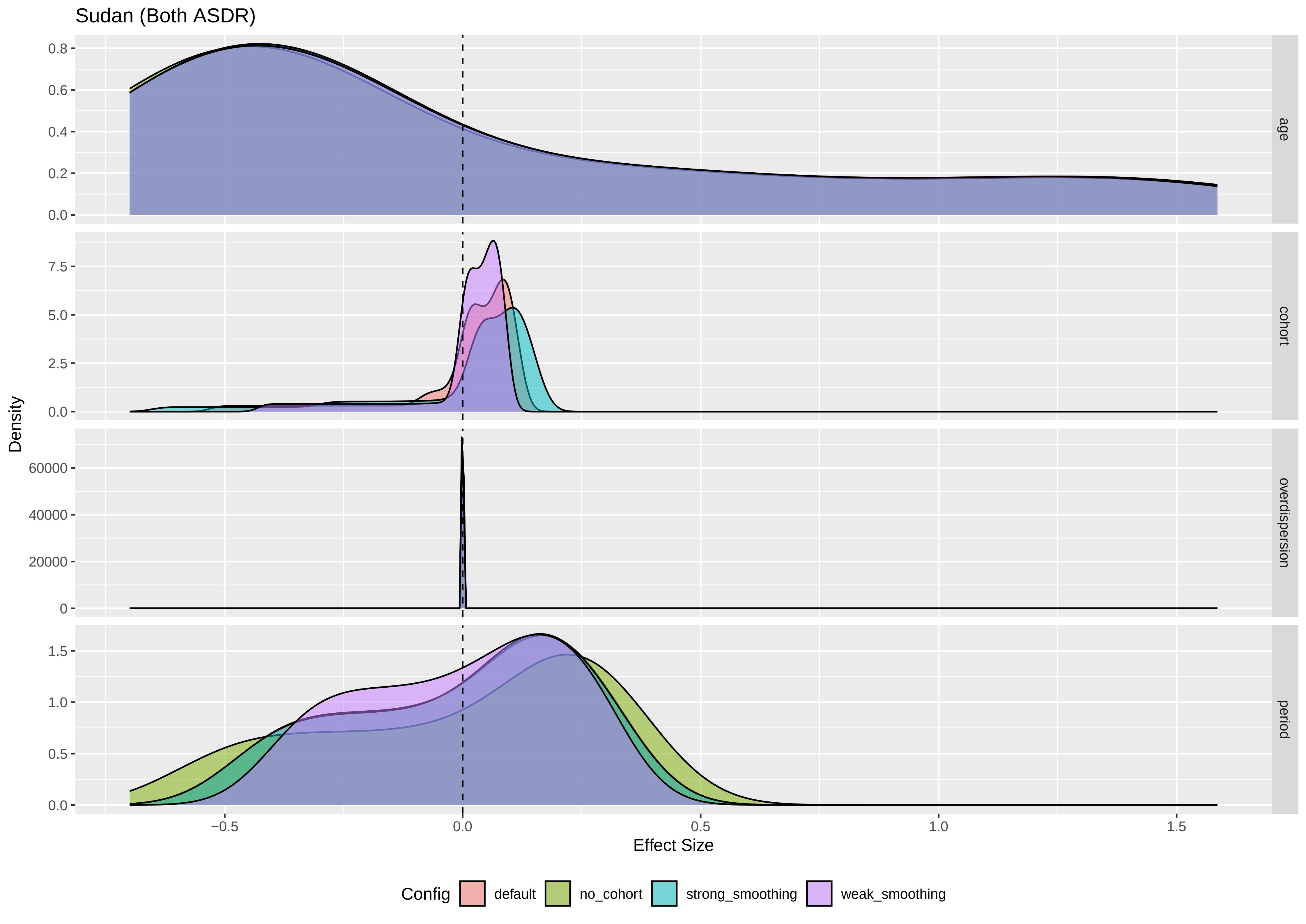

Sudan (Both ASDR)
0.8
0.6
0.4
0.2
0.0
age
7.5
5.0
2.5
0.0
cohort
60000
40000
20000
0
overdispersion
1.5
1.0
0.5
0.0
period
Density
−0.5
0.0
0.5
1.0
1.5
Effect Size
Config
default
no_cohort
strong_smoothing
weak_smoothing

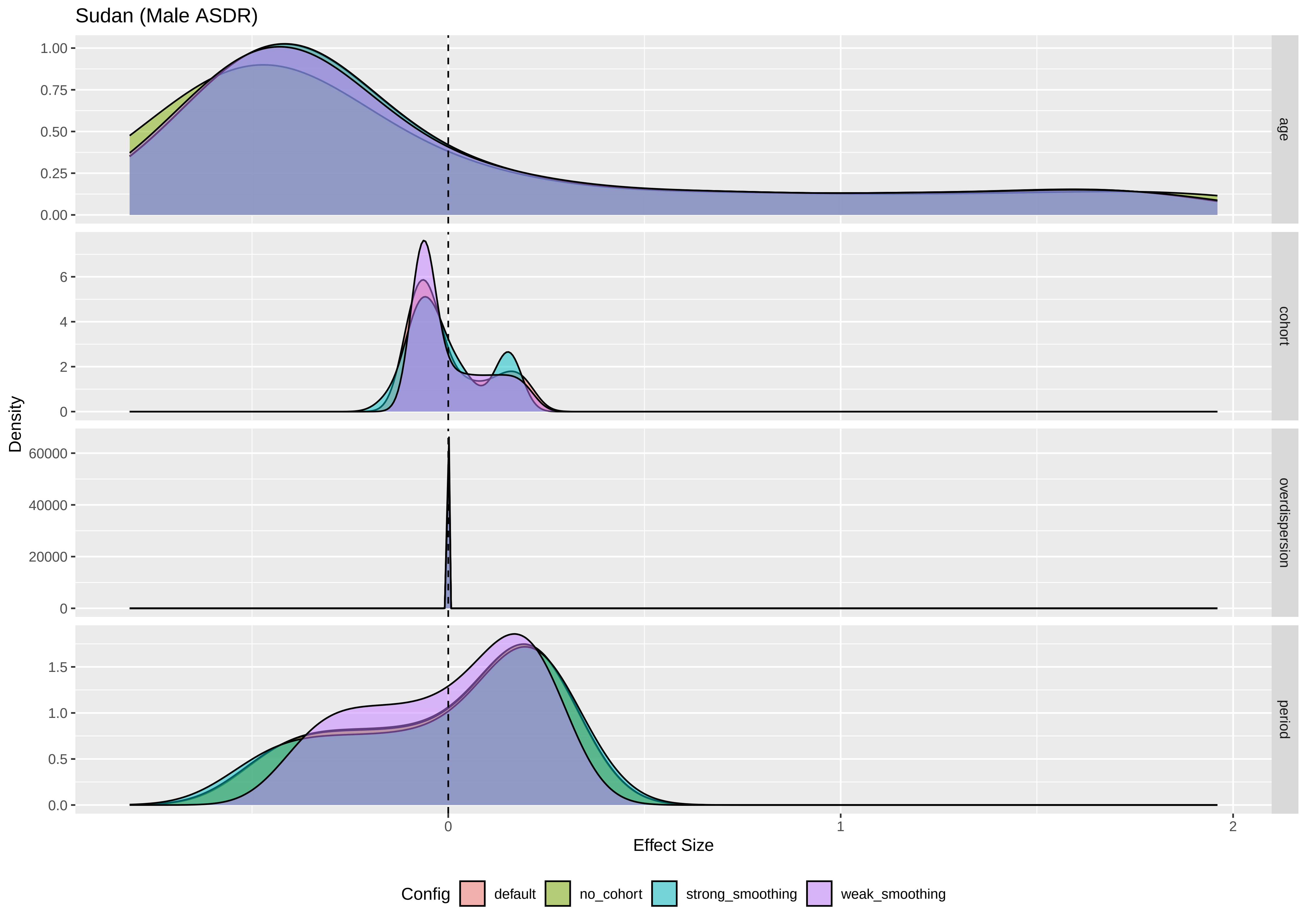

Sudan (Male ASDR)
1.00
0.75
0.50
0.25
0.00
age
6
4
2
0
cohort
60000
40000
20000
0
overdispersion
1.5
1.0
0.5
0.0
period
Density
0
1
2
Effect Size
Config
default
no_cohort
strong_smoothing
weak_smoothing

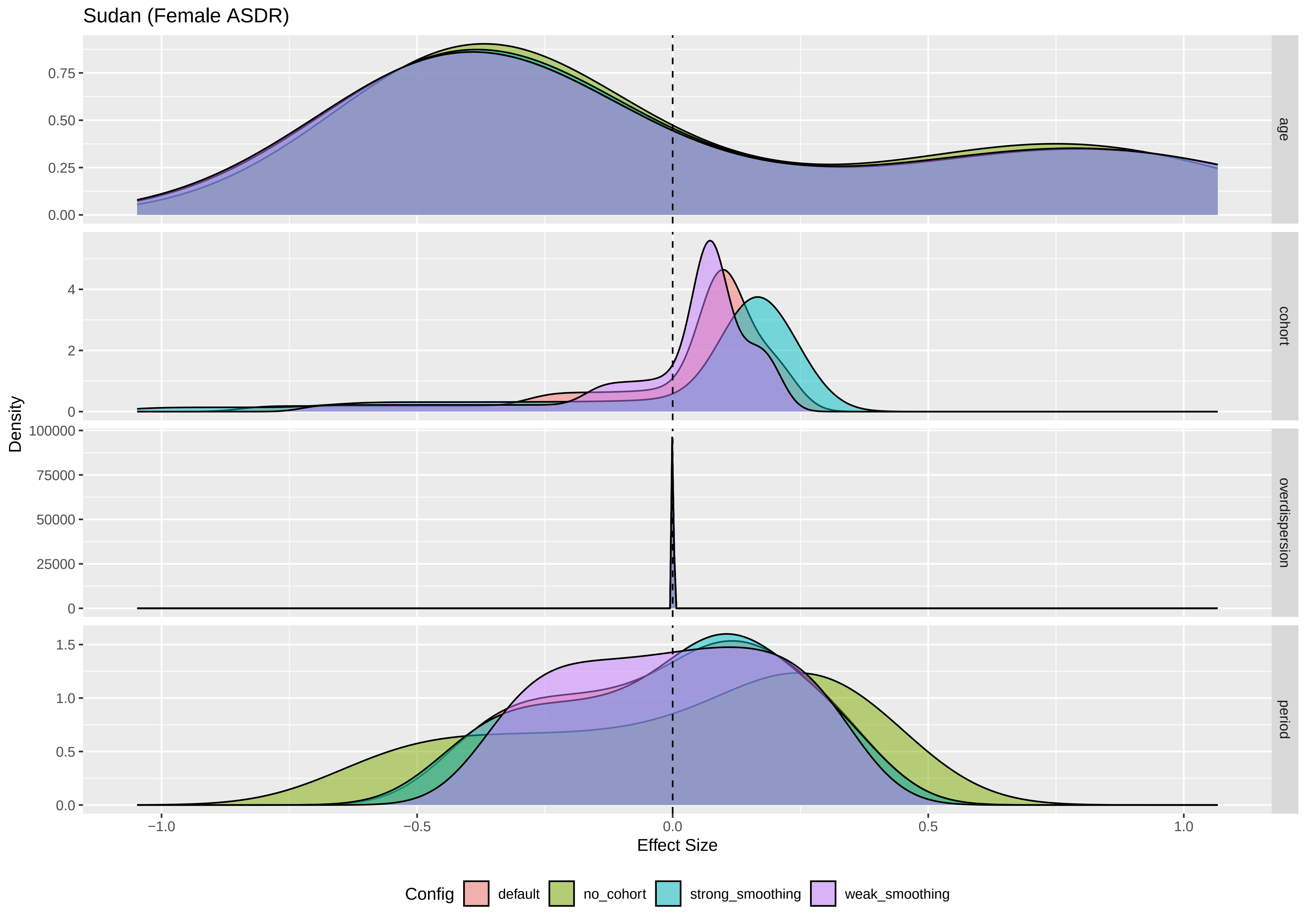

Sudan (Female ASDR)
age
cohort
overdispersion
period
Density
Effect Size
0.00
0.25
0.50
0.75
0
2
4
0
25000
50000
75000
100000
0.0
0.5
1.0
1.5
−1.0
−0.5
0.0
0.5
1.0
Config
default
no_cohort
strong_smoothing
weak_smoothing

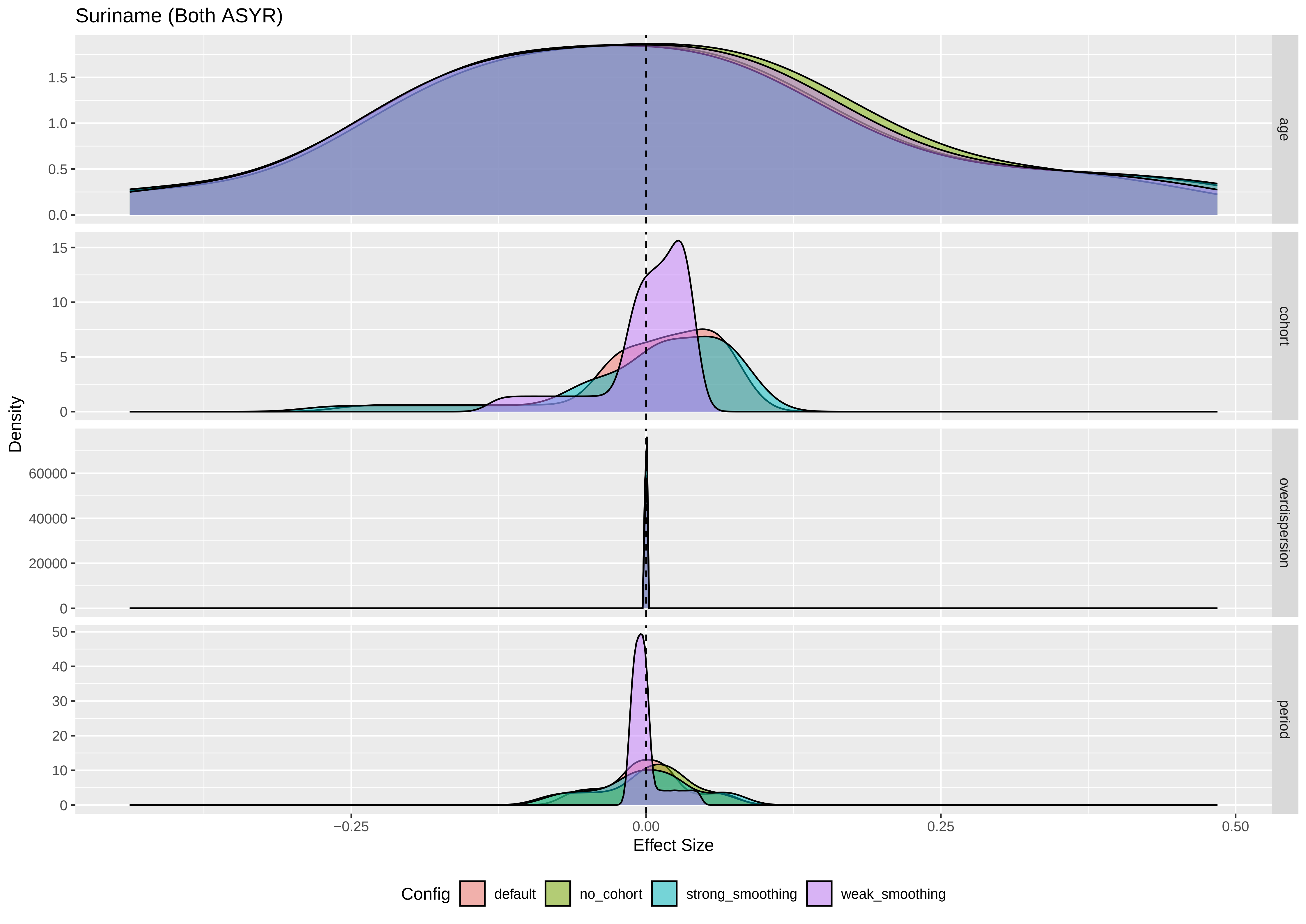

Suriname (Both ASYR)
Density
Effect Size
age
cohort
overdispersion
period
0.0
0.5
1.0
1.5
0
5
10
15
0
20000
40000
60000
0
10
20
30
40
50
-0.25
0.00
0.25
0.50
Config
default
no_cohort
strong_smoothing
weak_smoothing

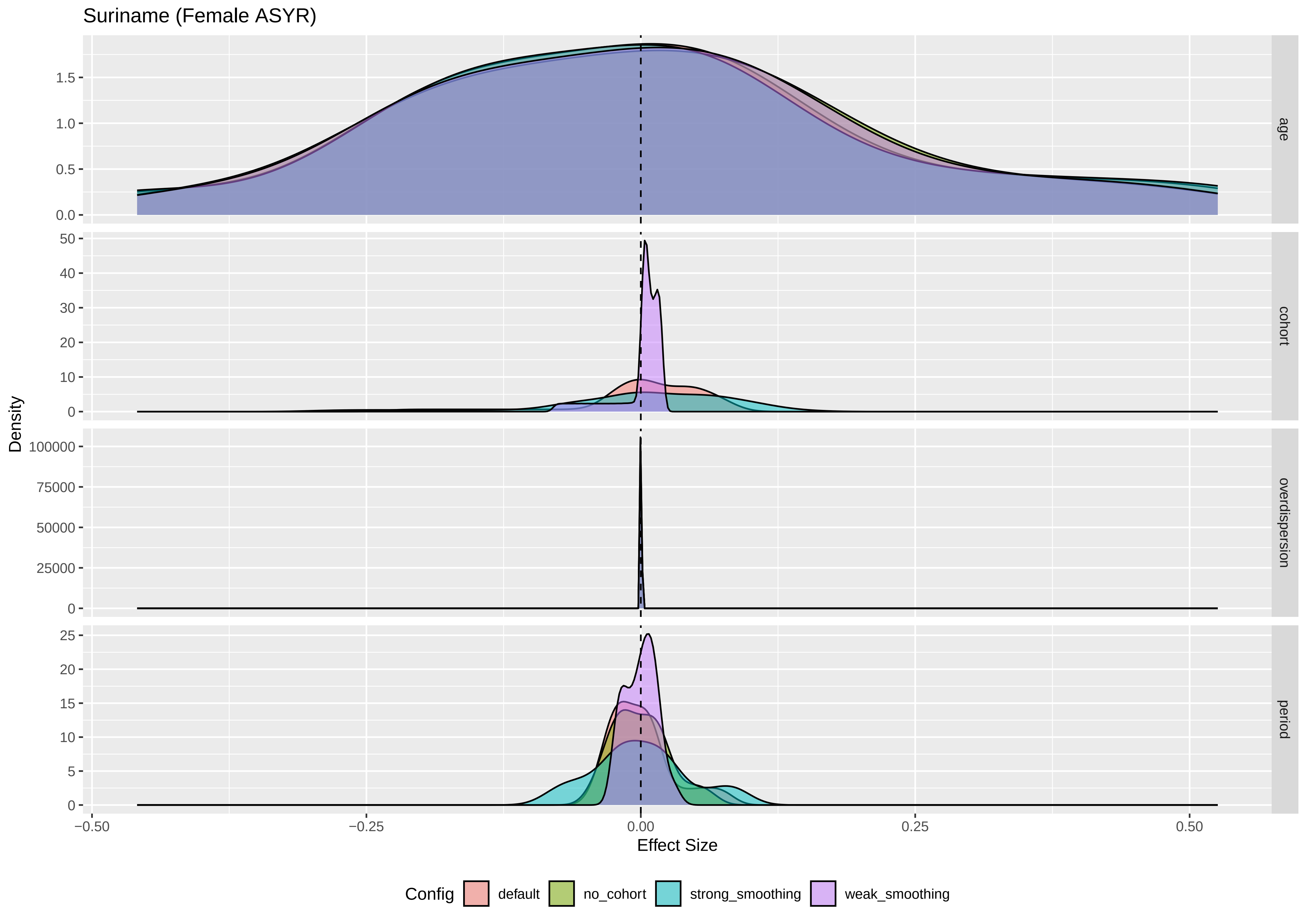

Suriname (Female ASYR)
age
cohort
overdispersion
period
Density
Effect Size
1.5
1.0
0.5
0.0
50
40
30
20
10
0
100000
75000
50000
25000
25
20
15
5
−0.50
−0.25
0.00
0.25
0.50
Config
default
no_cohort
strong_smoothing
weak_smoothing

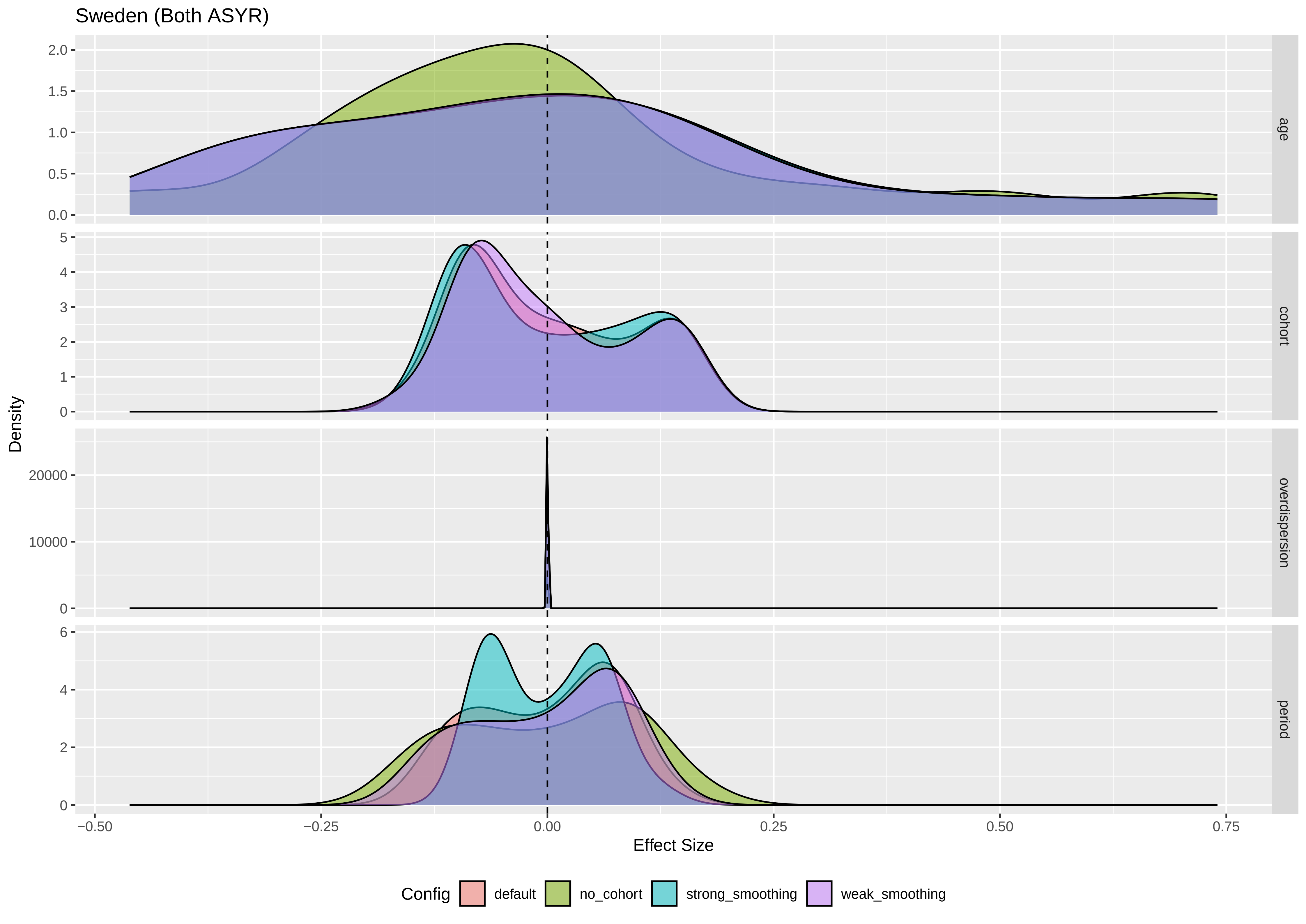

Sweden (Both ASYR)
Density
Effect Size
age
cohort
overdispersion
period
2.0
1.5
1.0
0.5
0.0
5
4
3
2
1
0
20000
10000
0
6
4
2
0
−0.50
−0.25
0.00
0.25
0.50
0.75
Config
default
no_cohort
strong_smoothing
weak_smoothing

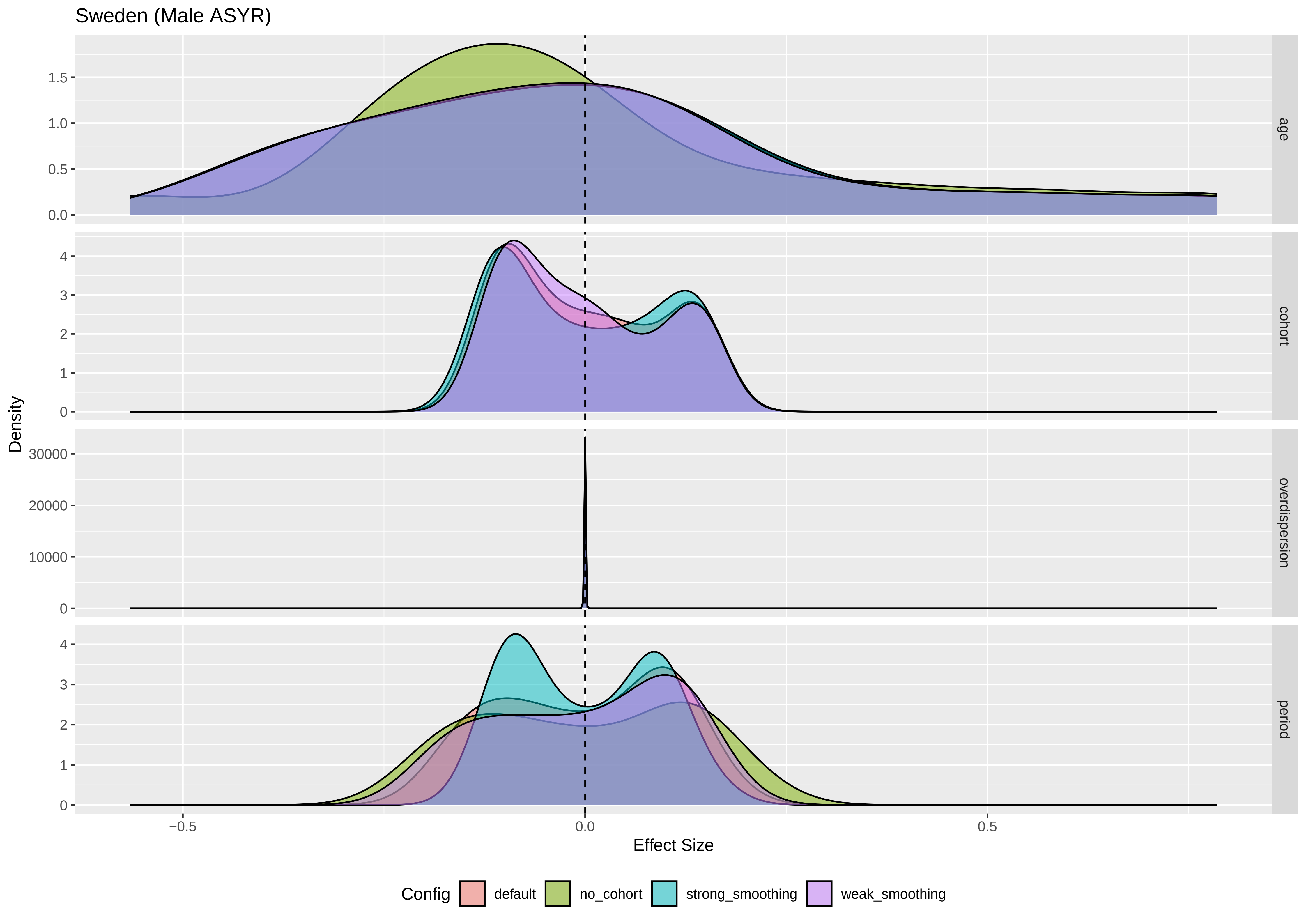

Sweden (Male ASYR)
Density
Effect Size
age
cohort
overdispersion
period
−0.5
0.0
0.5
1.5
1.0
0.5
0.0
4
3
2
1
0
30000
20000
10000
0
Config
default
no_cohort
strong_smoothing
weak_smoothing

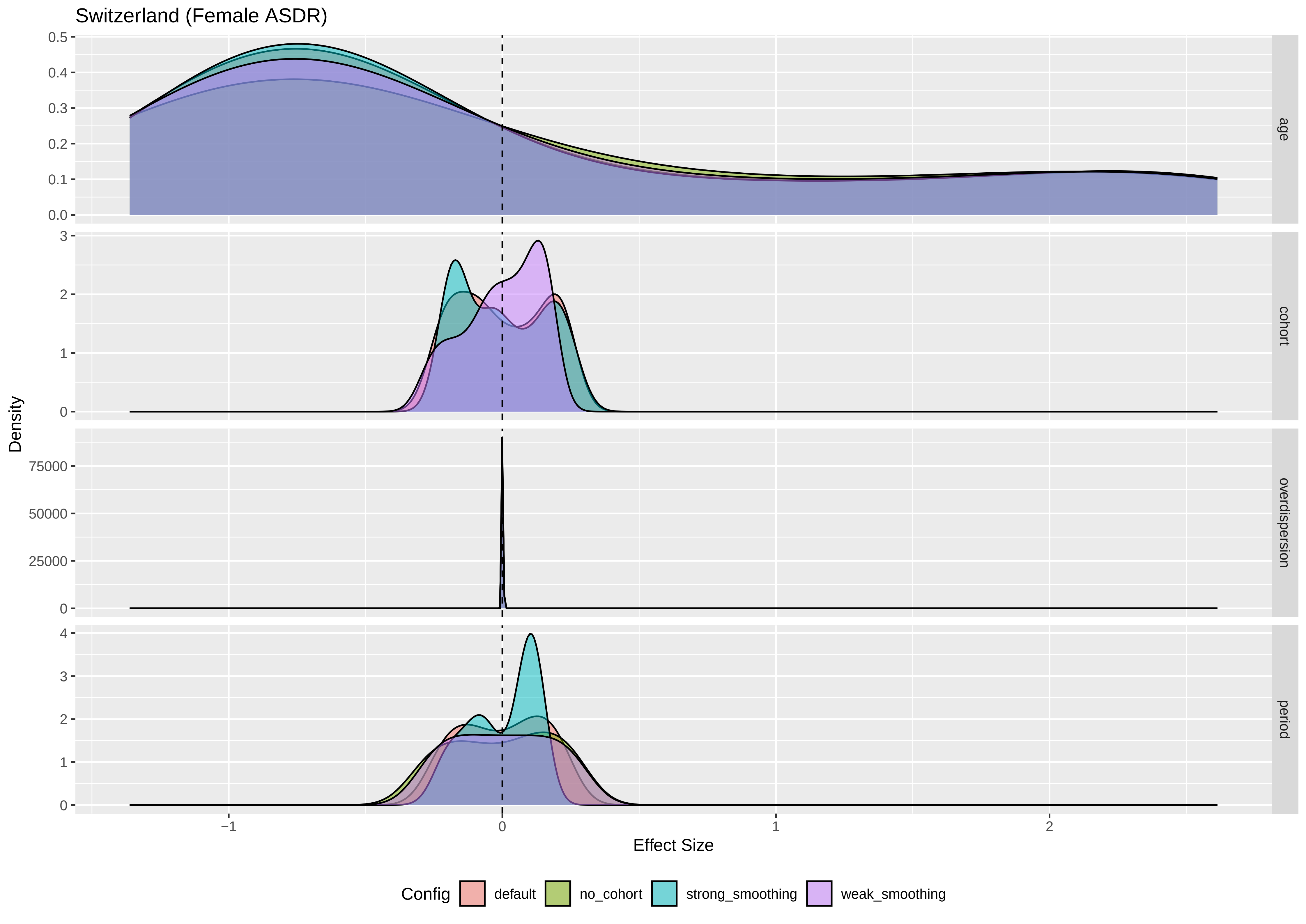

Switzerland (Female ASDR)
Density
Effect Size
age
cohort
overdispersion
period
0.0
0.1
0.2
0.3
0.4
0.5
0
1
2
3
0
25000
50000
75000
0
1
2
3
4
−1
0
1
2
Config
default
no_cohort
strong_smoothing
weak_smoothing

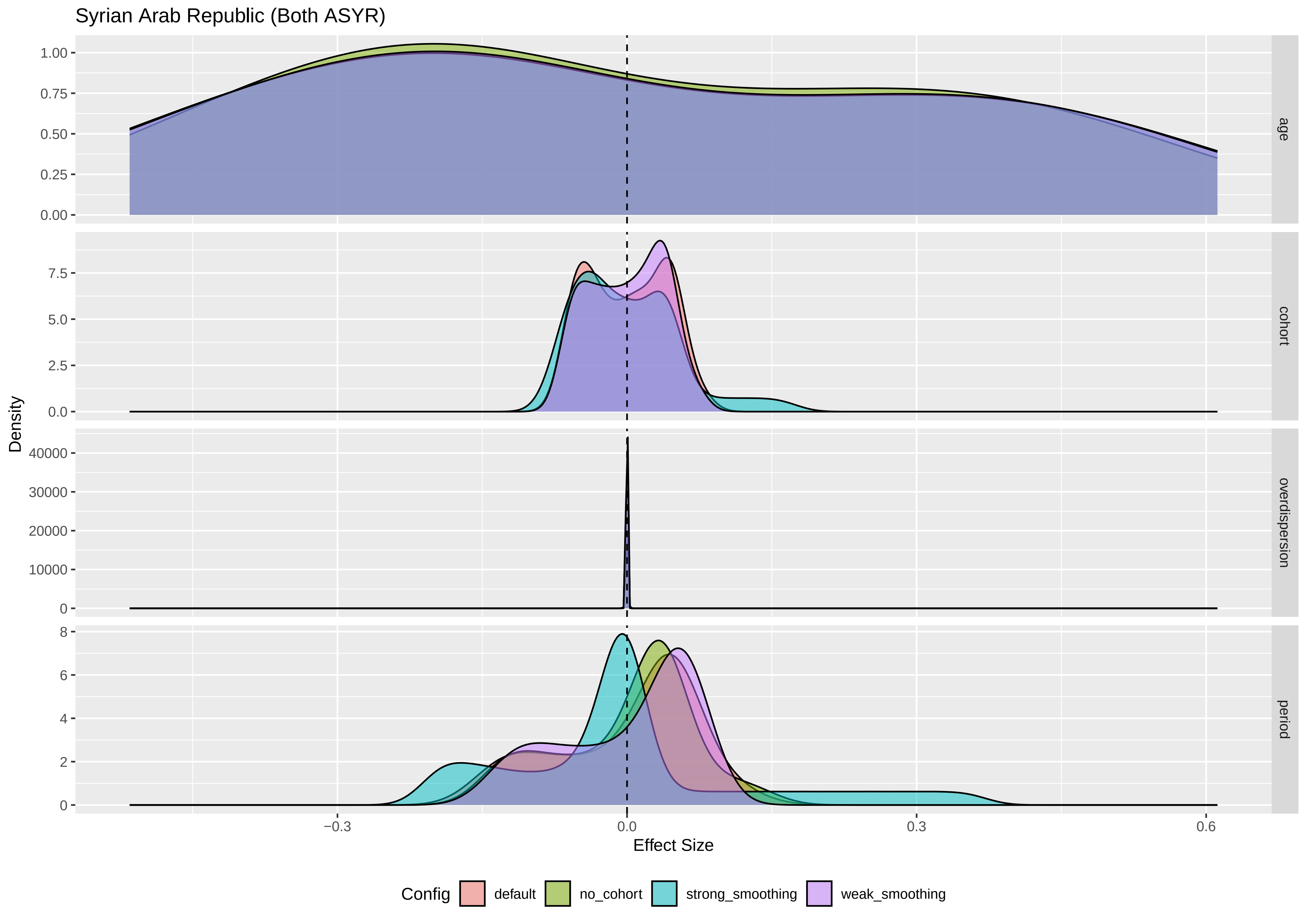

Syrian Arab Republic (Both ASYR)
Density
Effect Size
age
cohort
overdispersion
period
1.00
0.75
0.50
0.25
0.00
7.5
5.0
2.5
0.0
40000
30000
20000
10000
0
8
6
4
2
0
−0.3
0.0
0.3
0.6
Config
default
no_cohort
strong_smoothing
weak_smoothing

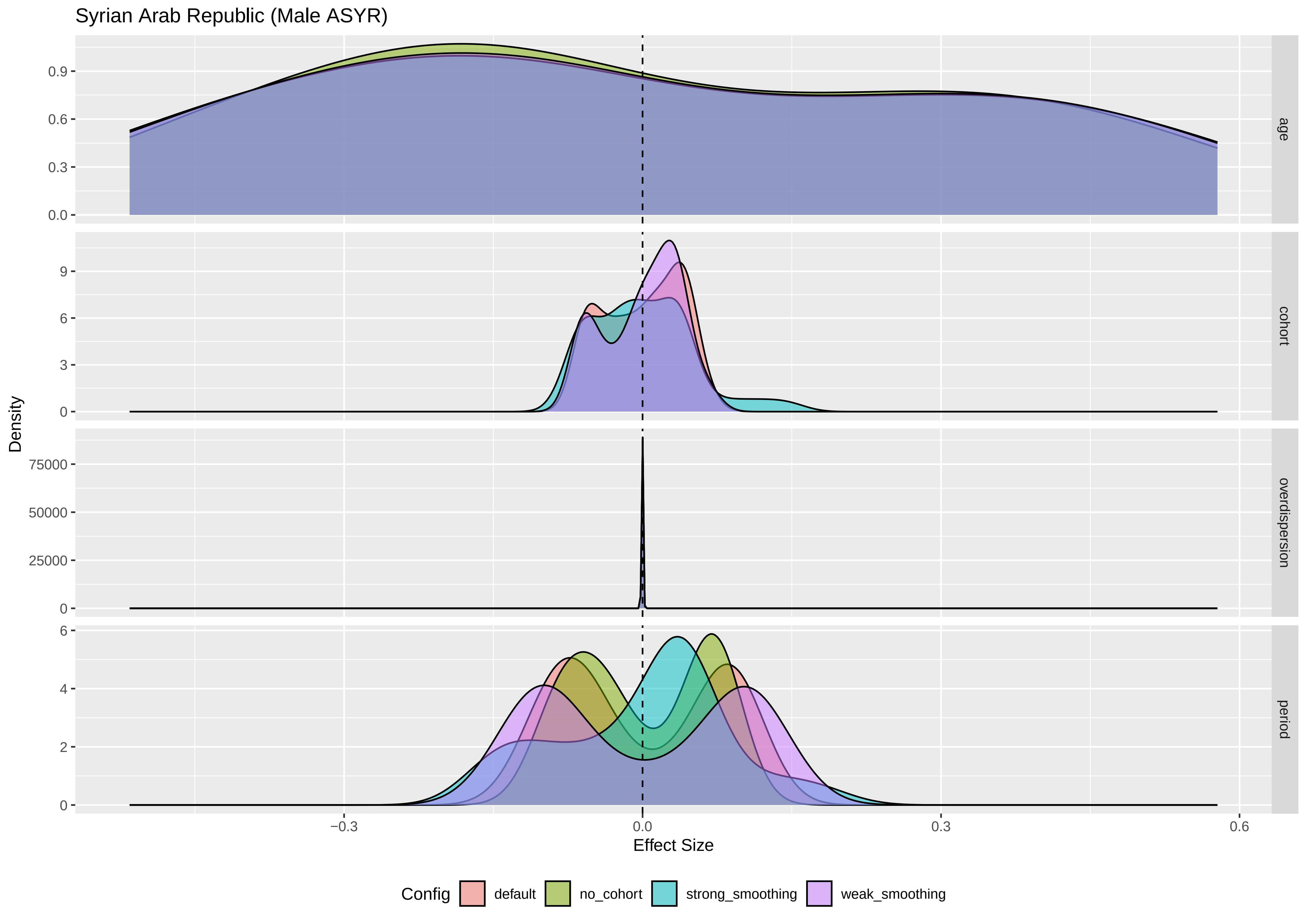

Syrian Arab Republic (Male ASYR)
Density
Effect Size
age
cohort
overdispersion
period
0.0
0.3
0.6
0.9
0
3
6
9
0
25000
50000
75000
0
2
4
6
−0.3
0.0
0.3
0.6
Config
default
no_cohort
strong_smoothing
weak_smoothing

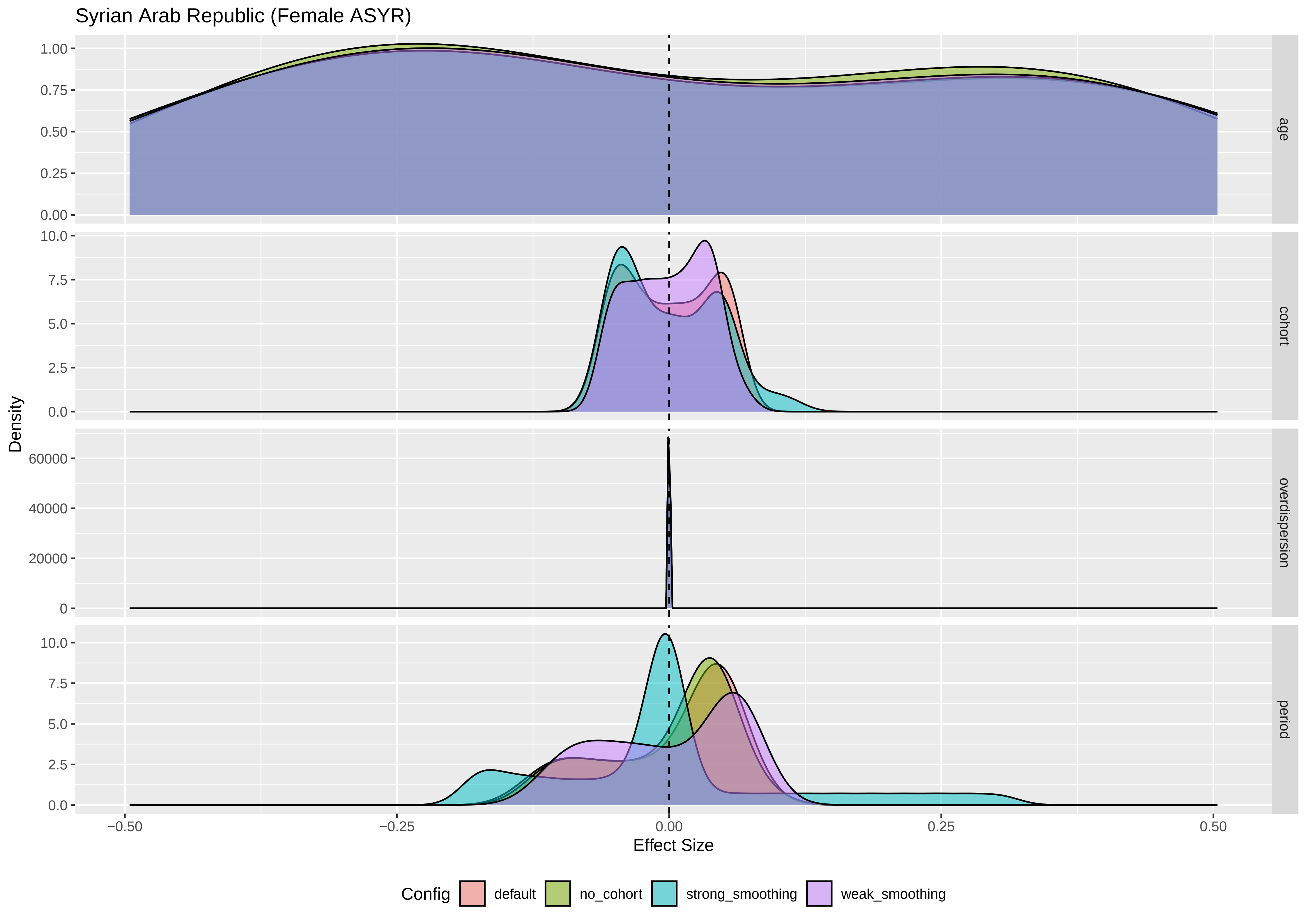

Syrian Arab Republic (Female ASYR)
age
cohort
overdispersion
period
Density
Effect Size
1.00
0.75
0.50
0.25
0.00
10.0
7.5
5.0
2.5
0.0
60000
40000
20000
0
−0.50
−0.25
0.00
0.25
0.50
Config
default
no_cohort
strong_smoothing
weak_smoothing

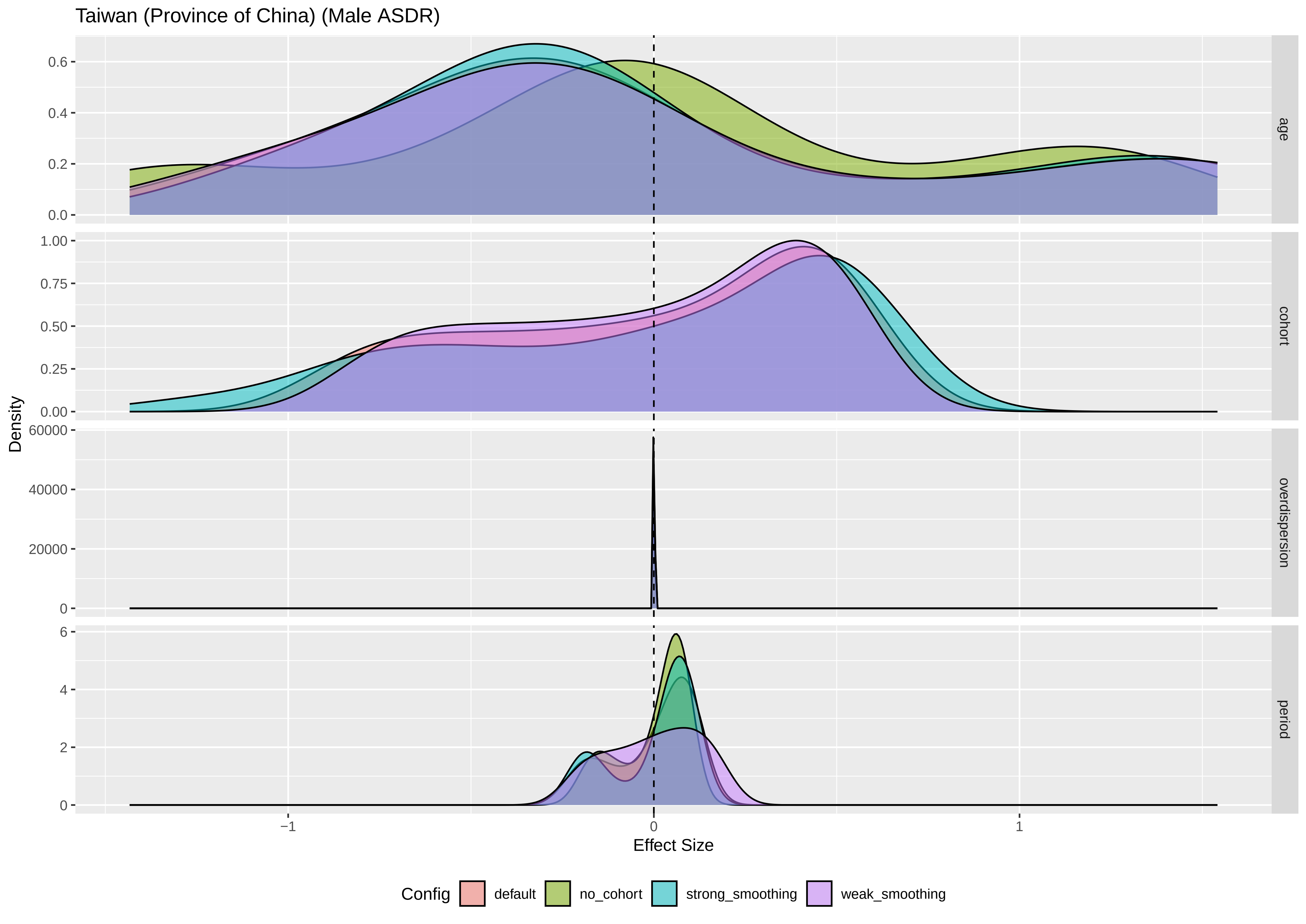
Taiwan (Province of China) (Male ASDR)
Density
Effect Size
age
cohort
overdispersion
period
0.0
0.2
0.4
0.6
0.00
0.25
0.50
0.75
1.00
0
20000
40000
60000
0
2
4
6
−1
0
1
Config
default
no_cohort
strong_smoothing
weak_smoothing

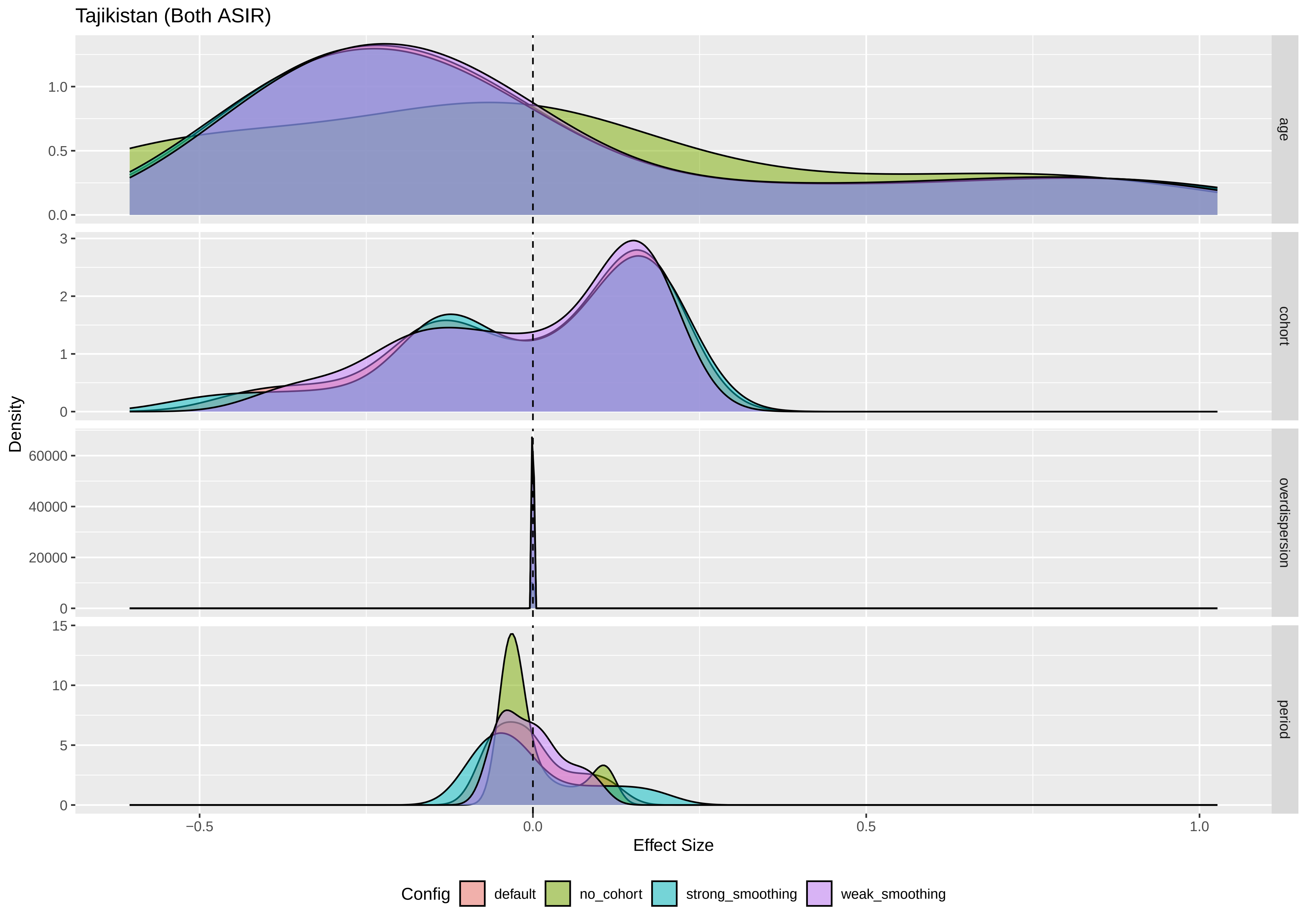

Tajikistan (Both ASIR)
Density
Effect Size
age
cohort
overdispersion
period
1.0
0.5
0.0
3
2
1
0
60000
40000
20000
0
15
10
5
0
−0.5
0.0
0.5
1.0
Config
default
no_cohort
strong_smoothing
weak_smoothing

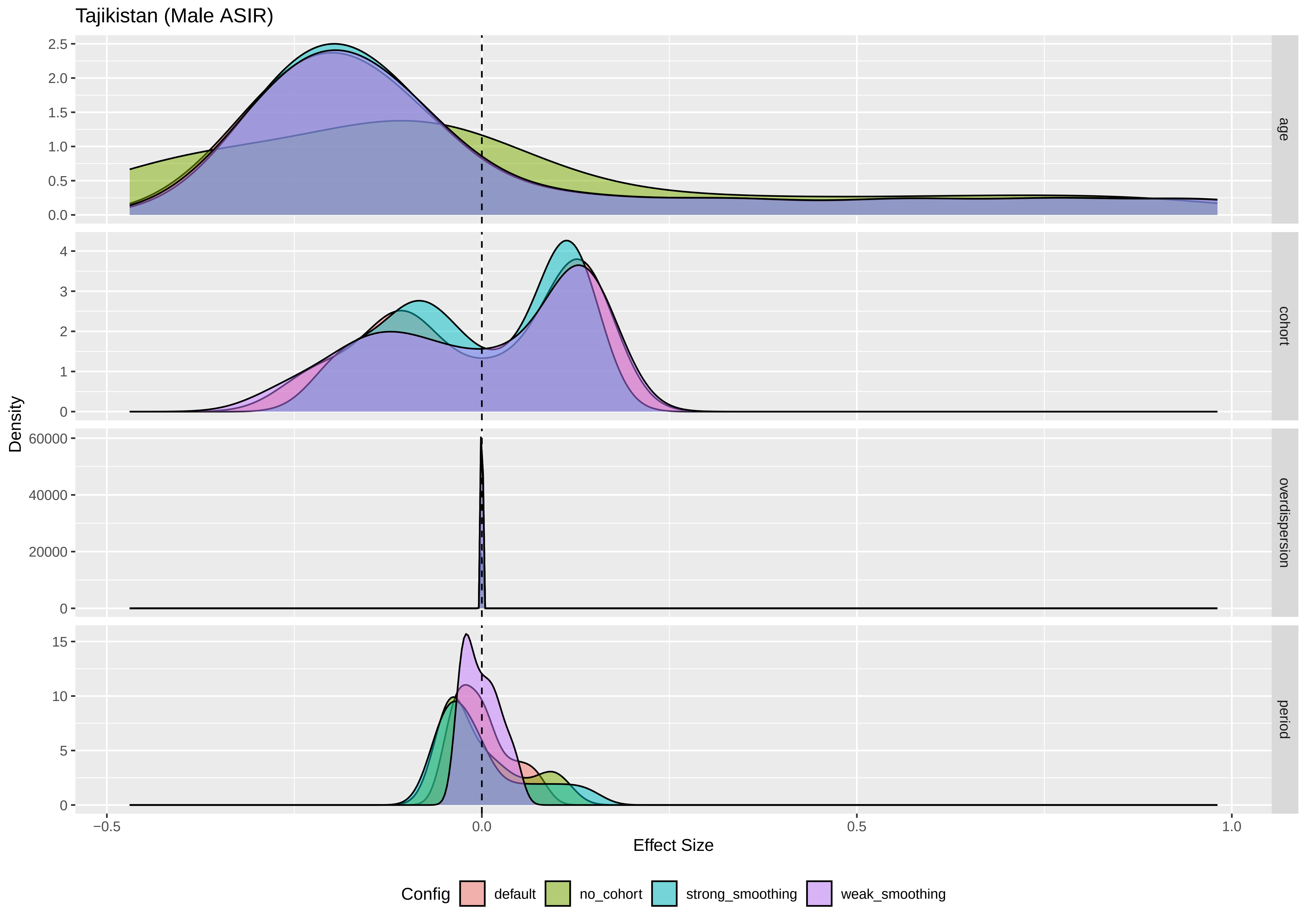
Tajikistan (Male ASIR)
age
cohort
overdispersion
period
Density
Effect Size
−0.5
0.0
0.5
1.0
2.5
2.0
1.5
1.0
0.5
0.0
4
3
2
1
0
60000
40000
20000
0
15
10
5
0
Config
default
no_cohort
strong_smoothing
weak_smoothing

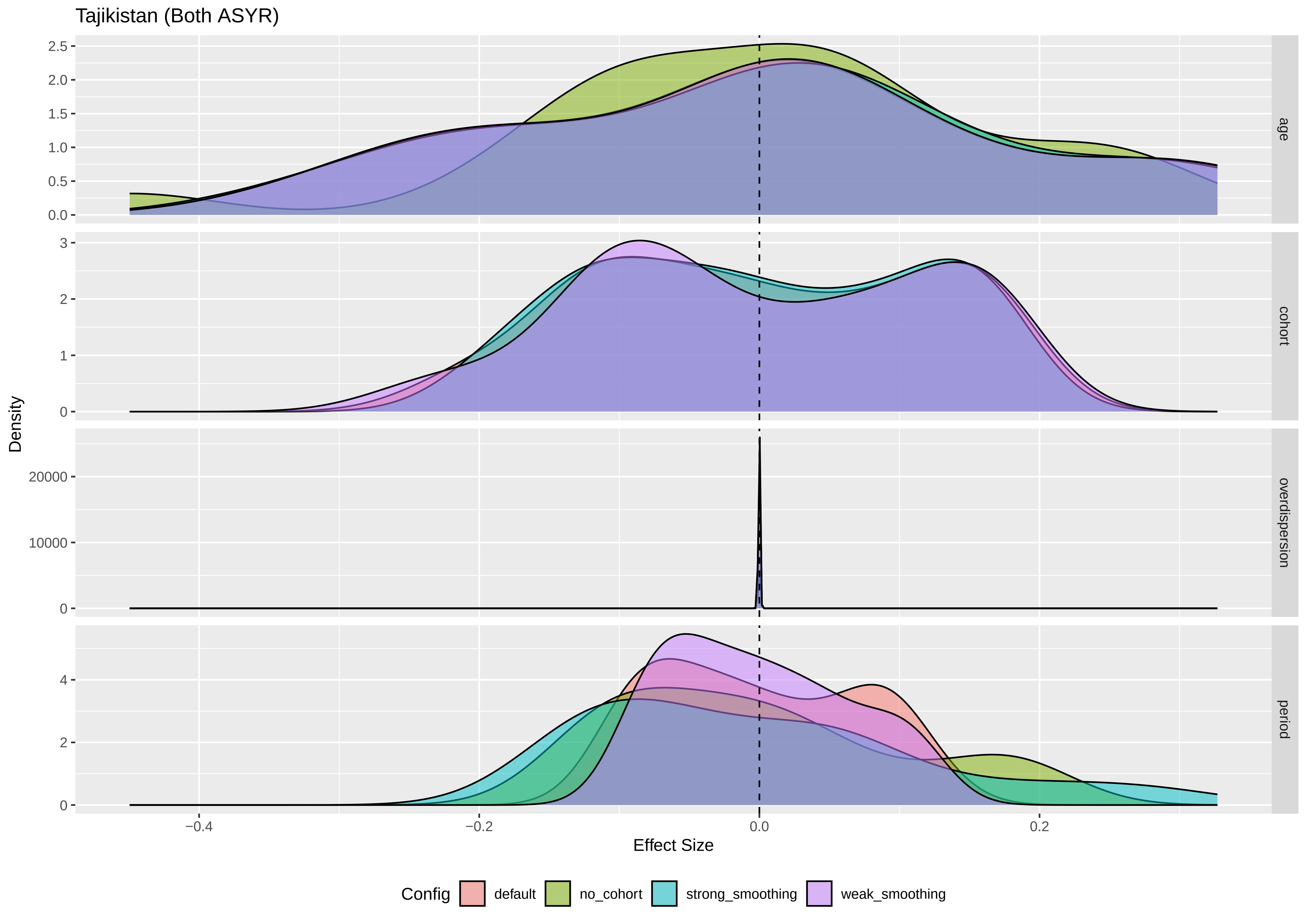

Tajikistan (Both ASYR)
Density
Effect Size
age
cohort
overdispersion
period
2.5
2.0
1.5
1.0
0.5
0.0
3
2
1
0
20000
10000
0
4
2
0
−0.4
−0.2
0.0
0.2
Config
default
no_cohort
strong_smoothing
weak_smoothing

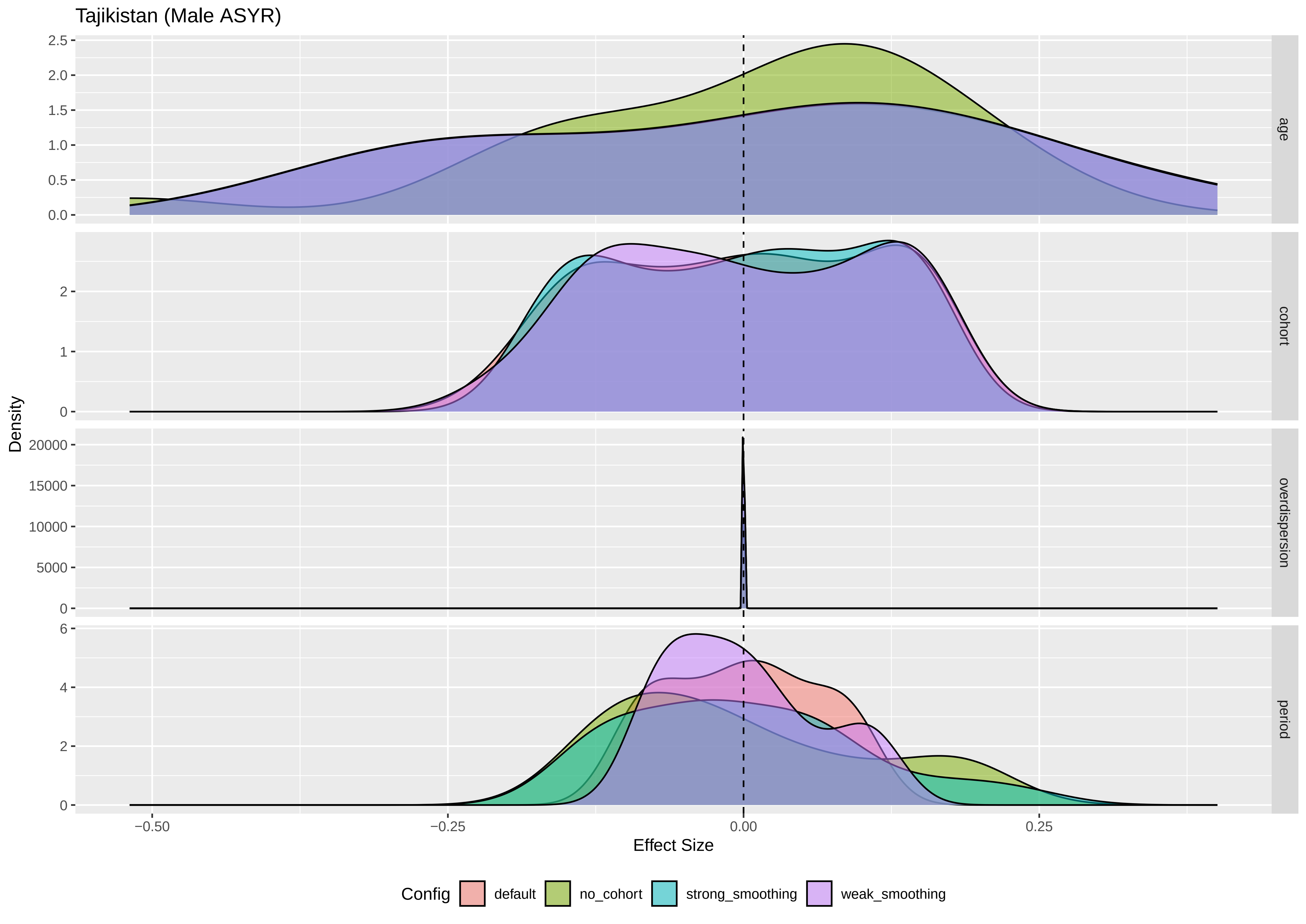

Tajikistan (Male ASYR)
Density
Effect Size
age
cohort
overdispersion
period
−0.50
−0.25
0.00
0.25
Config
default
no_cohort
strong_smoothing
weak_smoothing

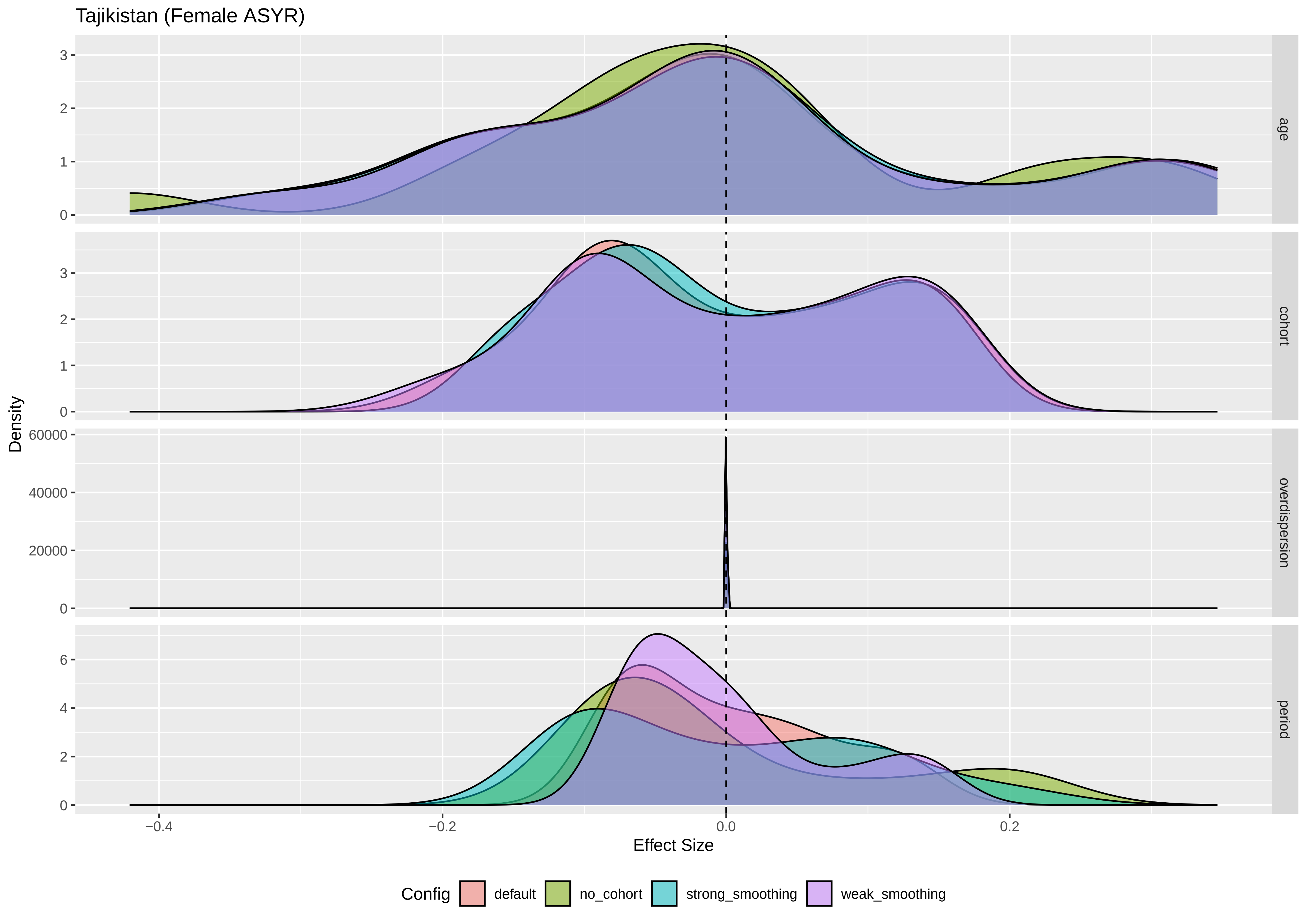

Tajikistan (Female ASYR)
Density
Effect Size
age
cohort
overdispersion
period
3
2
1
0
60000
40000
20000
6
4
−0.4
−0.2
0.0
0.2
Config
default
no_cohort
strong_smoothing
weak_smoothing

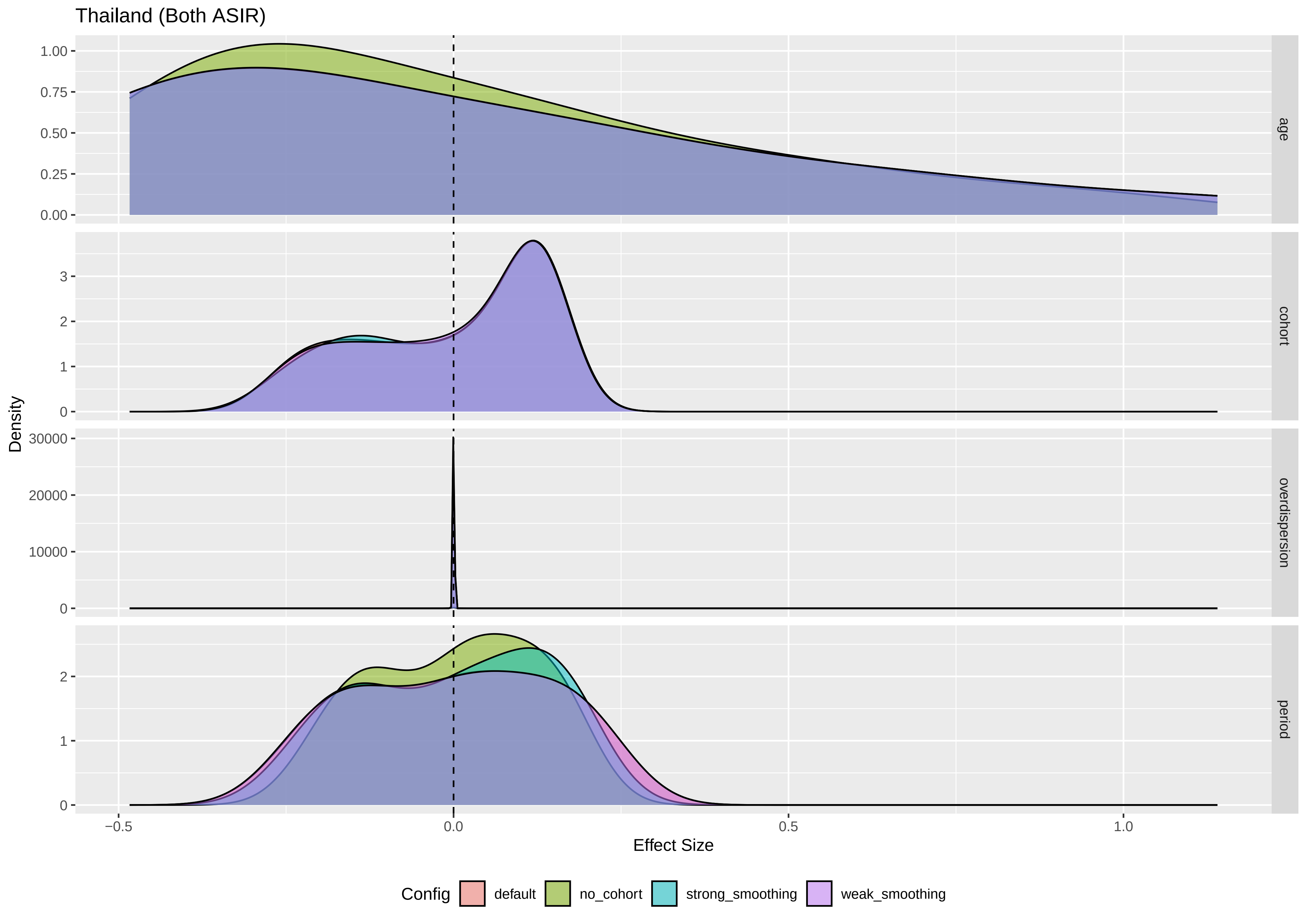

Thailand (Both ASIR)
1.00
0.75
0.50
0.25
0.00
age
3
2
1
0
cohort
30000
20000
10000
0
overdispersion
2
1
0
period
Density
−0.5
0.0
0.5
1.0
Effect Size
Config
default
no_cohort
strong_smoothing
weak_smoothing

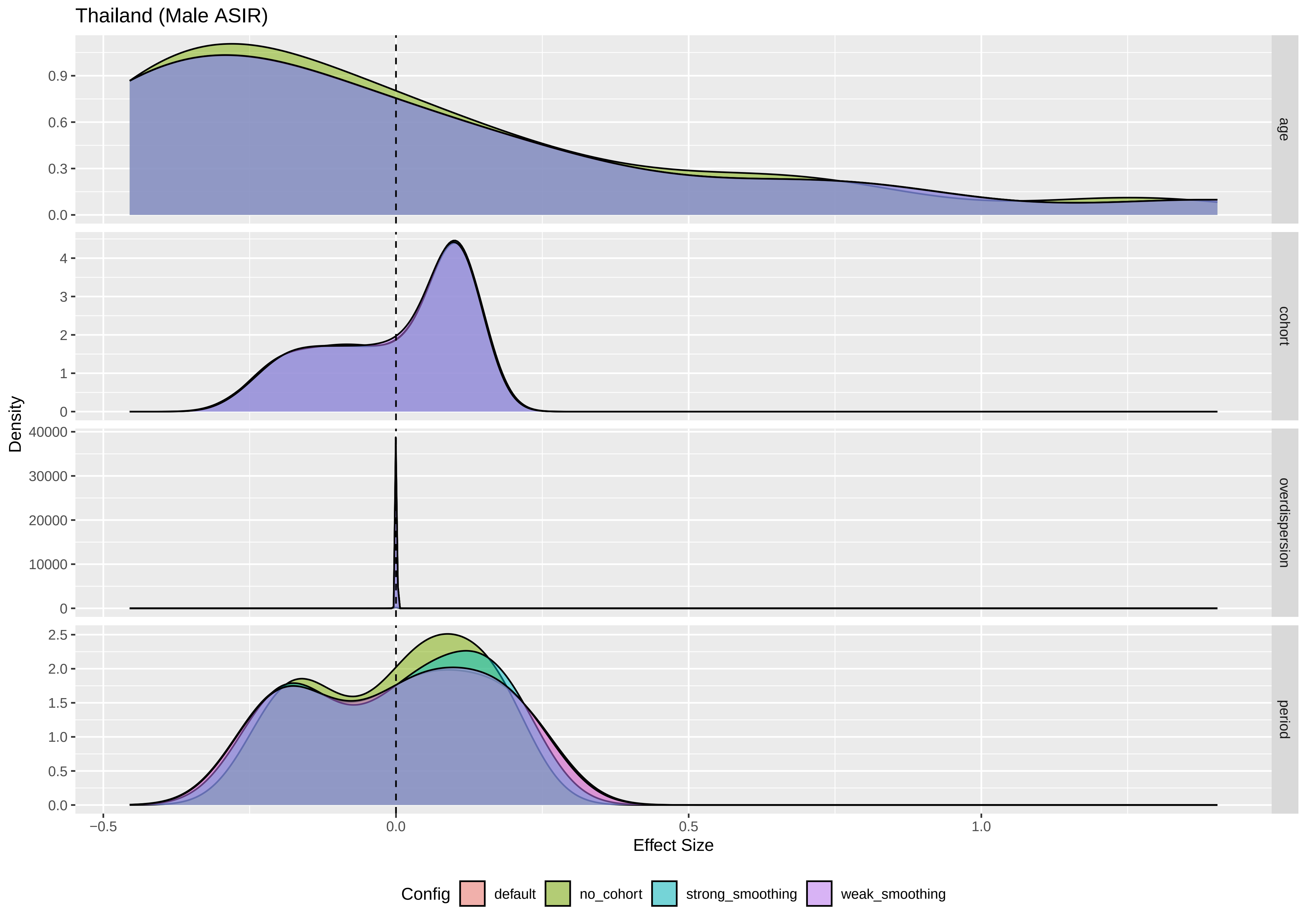

Thailand (Male ASIR)
Density
Effect Size
age
cohort
overdispersion
period
0.0
0.3
0.6
0.9
0
1
2
3
4
10000
20000
30000
40000
0.5
1.0
1.5
2.0
2.5
−0.5
0.0
0.5
1.0
Config
default
no_cohort
strong_smoothing
weak_smoothing

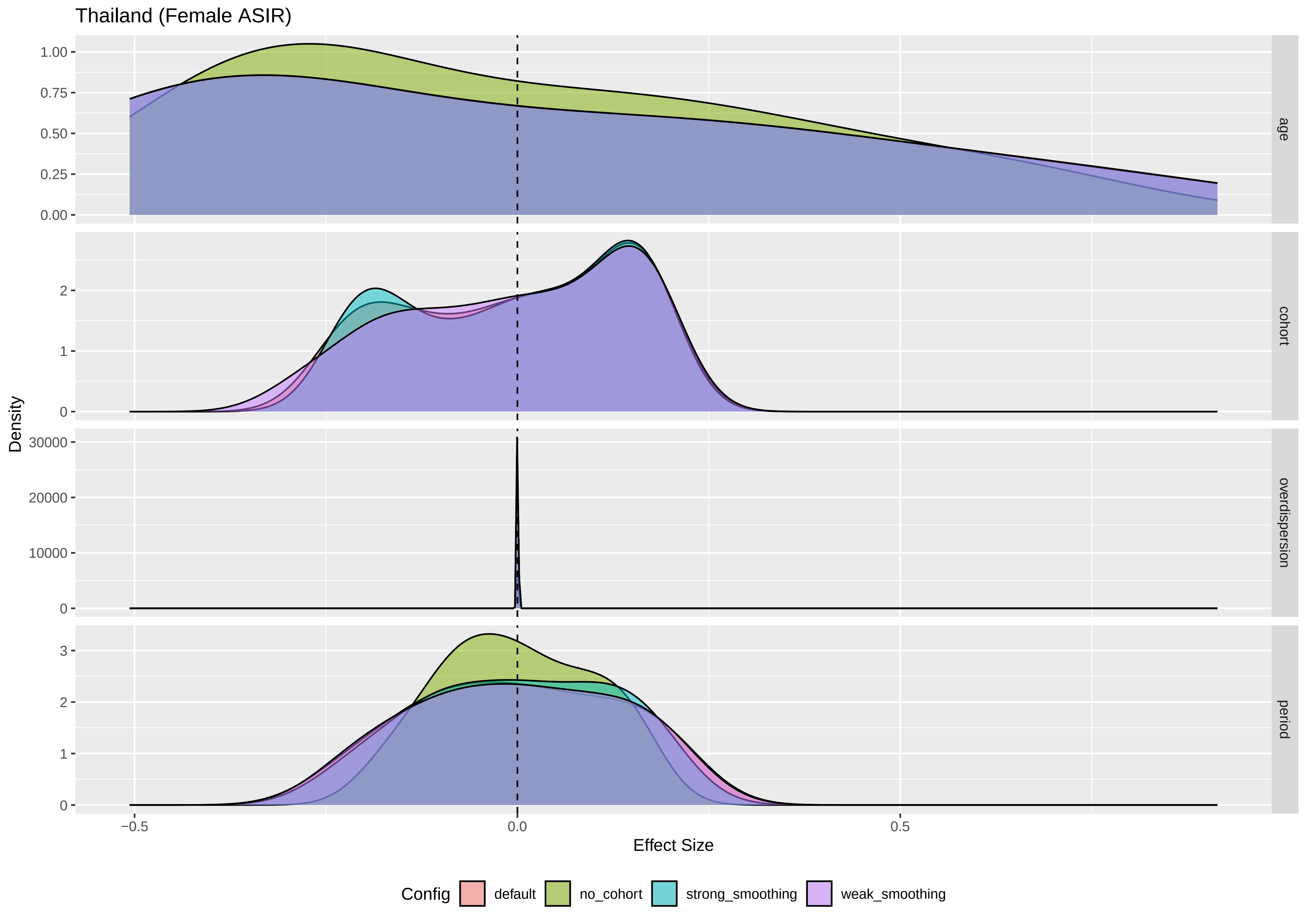
Thailand (Female ASIR)
Density
Effect Size
age
cohort
overdispersion
period
1.00
0.75
0.50
0.25
0.00
2
1
0
30000
20000
10000
0
3
2
1
0
−0.5
0.0
0.5
Config
default
no_cohort
strong_smoothing
weak_smoothing

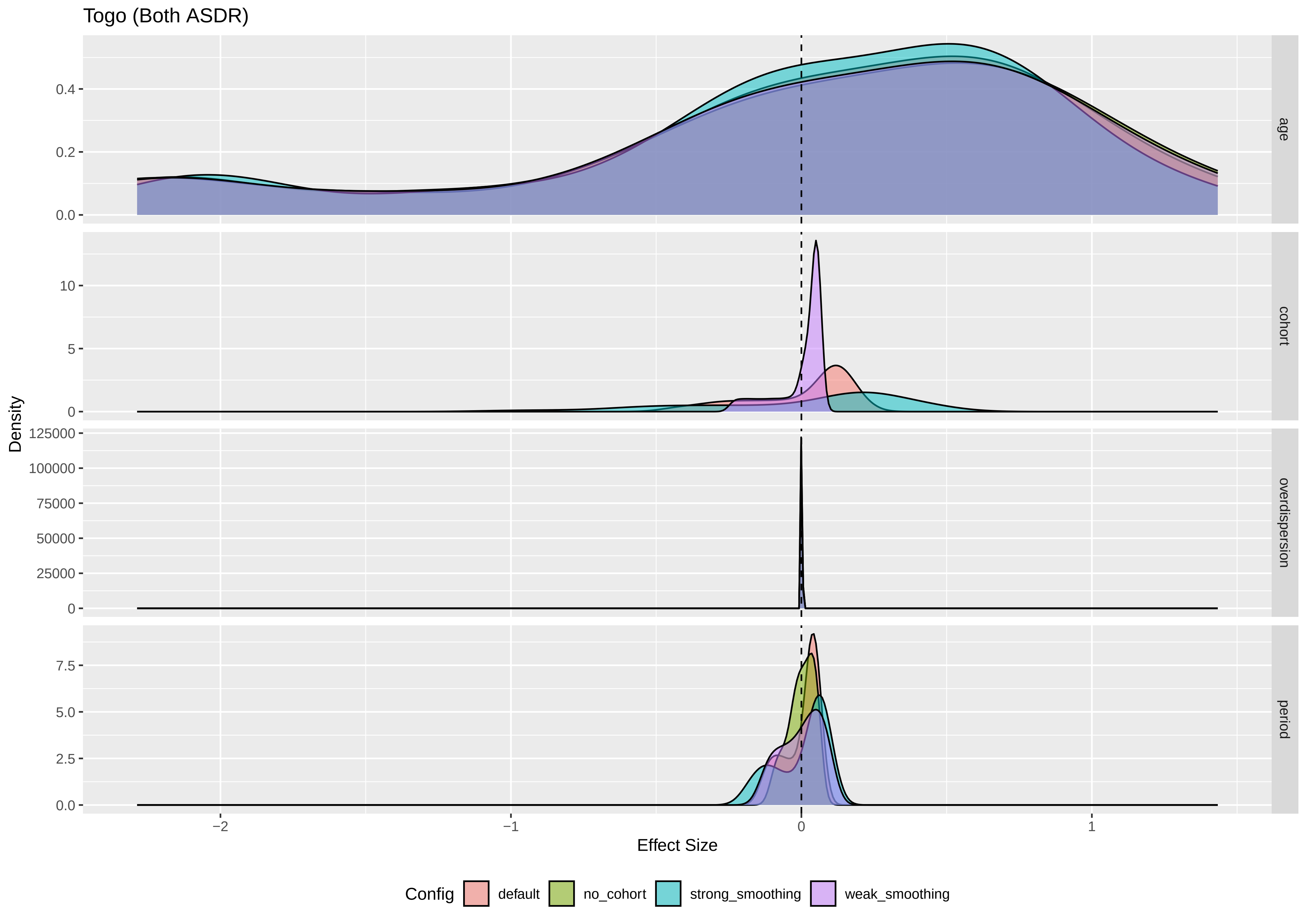

Togo (Both ASDR)
Density
Effect Size
age
cohort
overdispersion
period
0.0
0.2
0.4
0
5
10
25000
50000
75000
100000
125000
2.5
5.0
7.5
−2
−1
0
1
Config
default
no_cohort
strong_smoothing
weak_smoothing

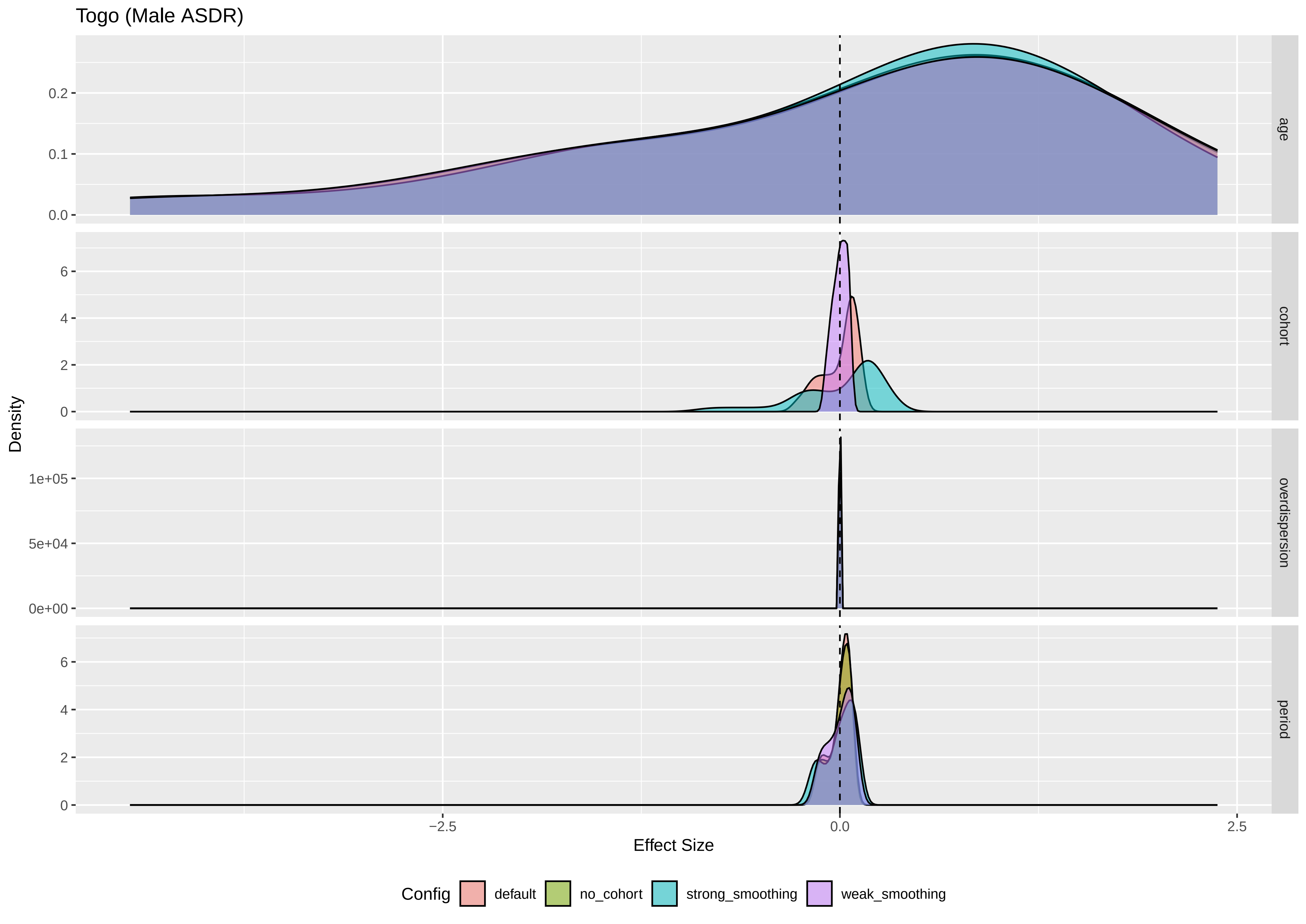

Togo (Male ASDR)
Density
Effect Size
age
cohort
overdispersion
period
0.0
0.1
0.2
0
2
4
6
0e+00
5e+04
1e+05
−2.5
0.0
2.5
Config
default
no_cohort
strong_smoothing
weak_smoothing

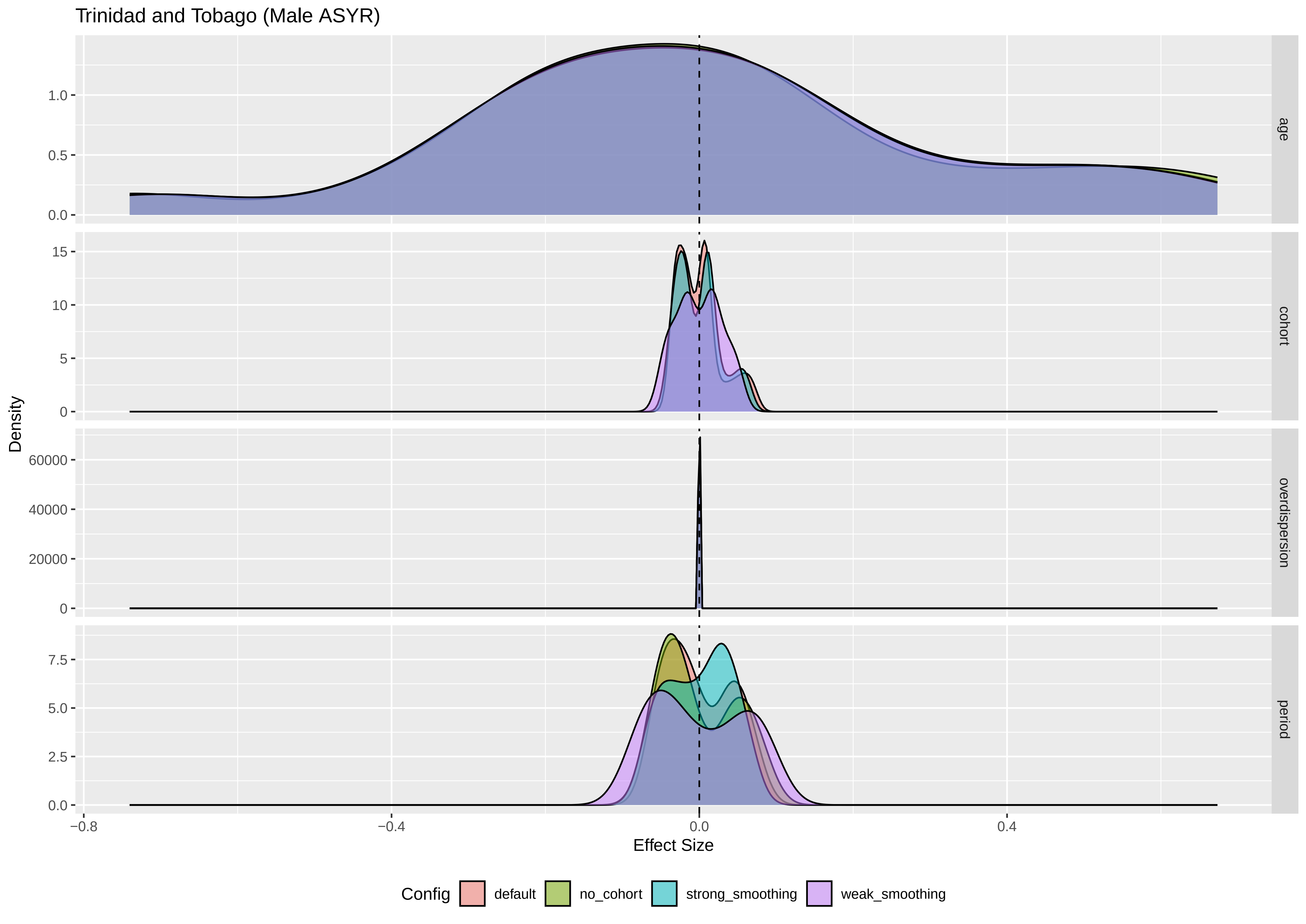

Trinidad and Tobago (Male ASYR)
age
cohort
overdispersion
period
Density
Effect Size
1.0
0.5
0.0
15
10
5
0
60000
40000
20000
0
7.5
5.0
2.5
0.0
−0.8
−0.4
0.0
0.4
Config
default
no_cohort
strong_smoothing
weak_smoothing

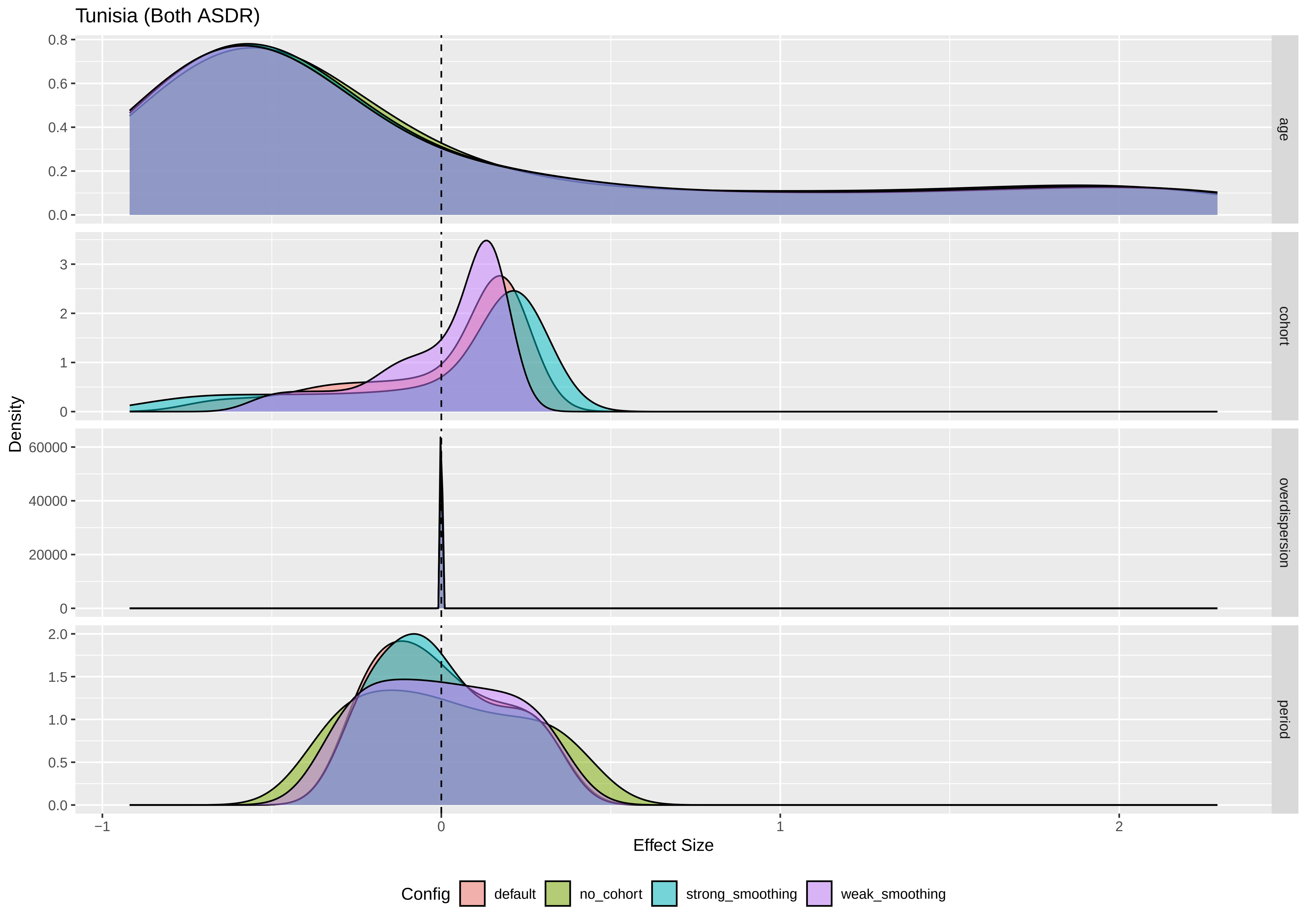

Tunisia (Both ASDR)
Density
Effect Size
age
cohort
overdispersion
period
0.8
0.6
0.4
0.2
0.0
3
2
1
0
60000
40000
20000
0
2.0
1.5
1.0
0.5
0.0
−1
0
1
2
Config
default
no_cohort
strong_smoothing
weak_smoothing

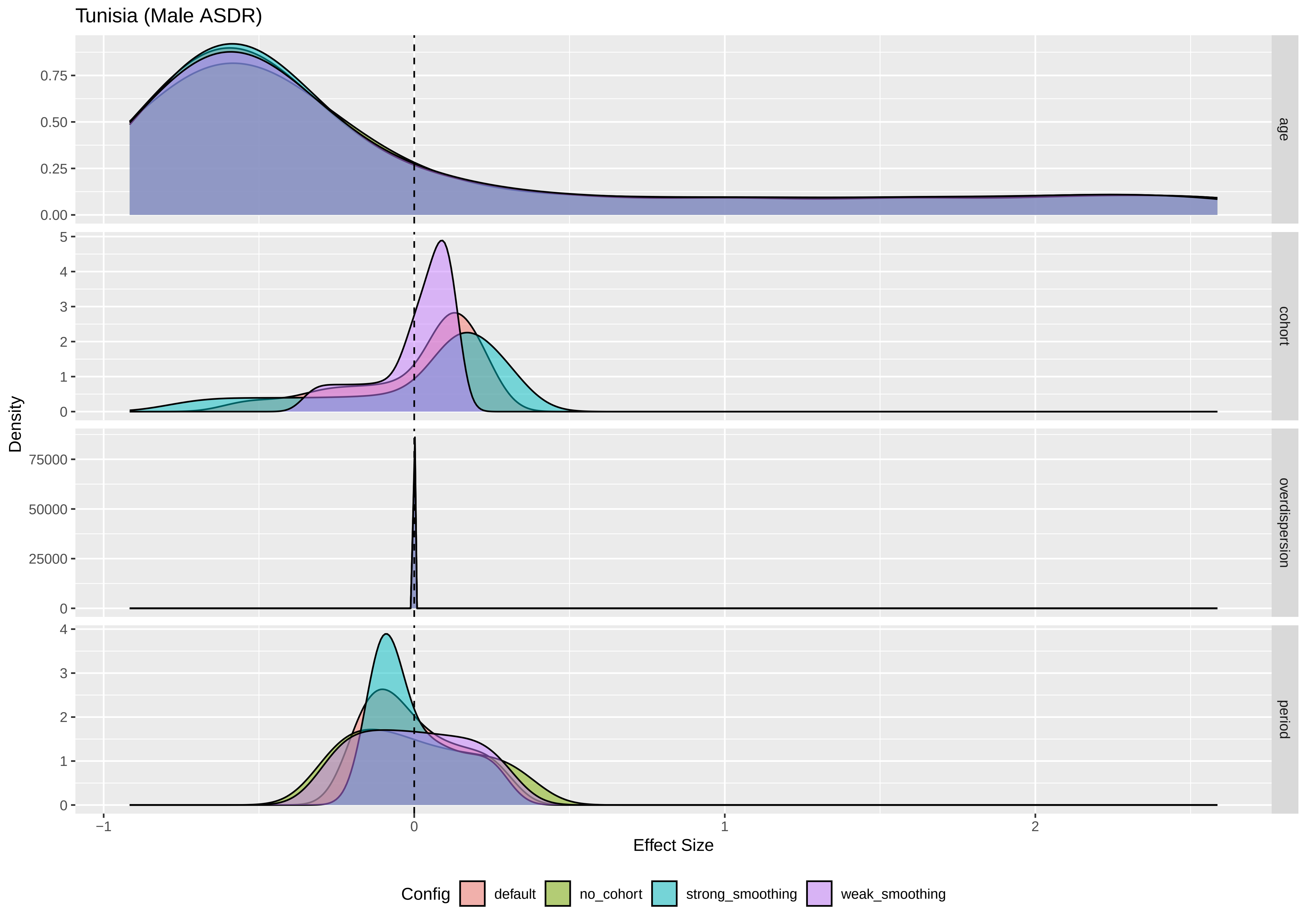

Tunisia (Male ASDR)
age
cohort
overdispersion
period
Density
0.75
0.50
0.25
0.00
5
4
3
2
1
0
75000
50000
25000
0
4
3
2
1
0
−1
0
1
2
Effect Size
Config
default
no_cohort
strong_smoothing
weak_smoothing

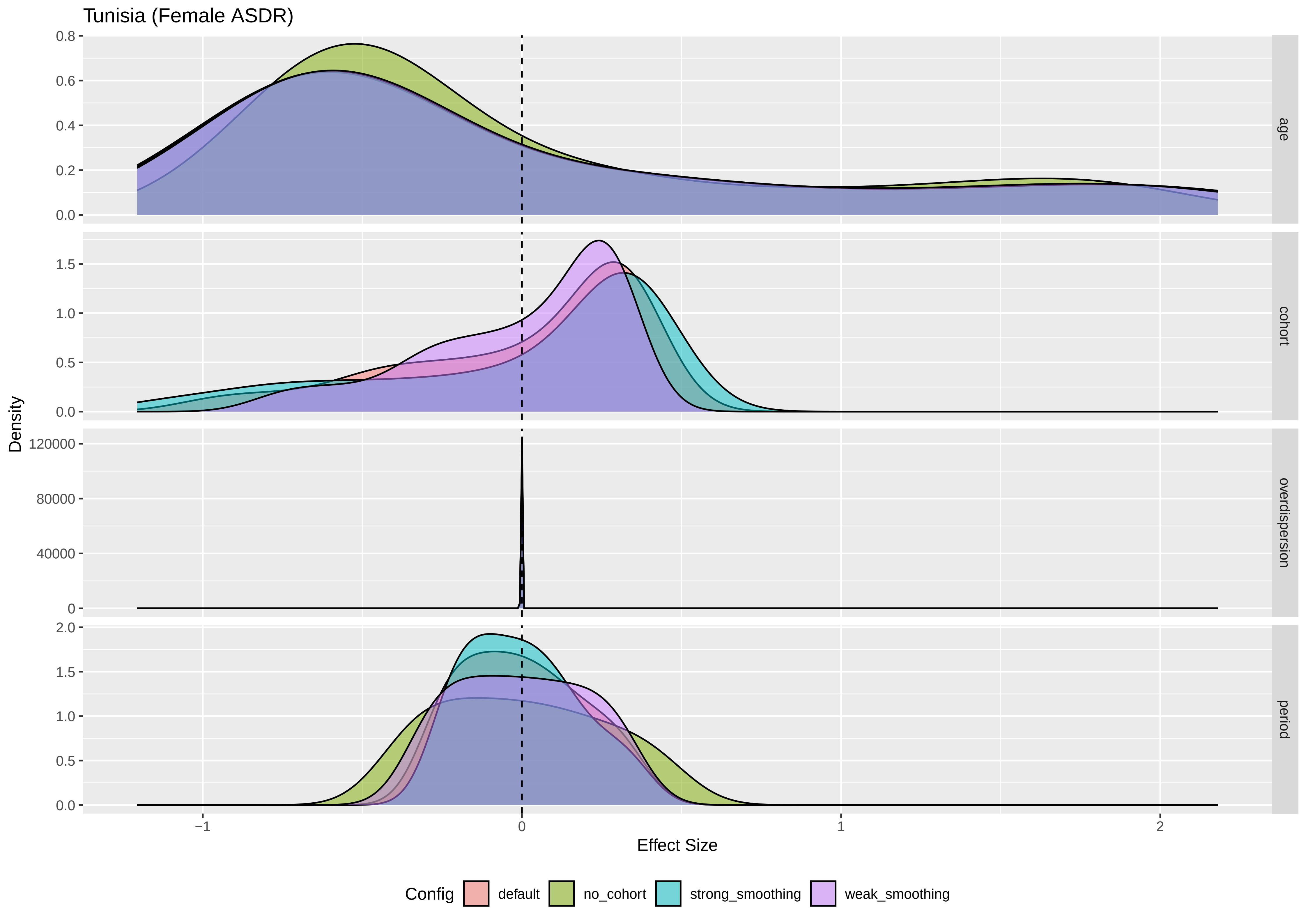
Tunisia (Female ASDR)
Density
Effect Size
age
cohort
overdispersion
period
0.0
0.2
0.4
0.6
0.8
0.5
1.0
1.5
0
40000
80000
120000
2.0
−1
0
1
2
Config
default
no_cohort
strong_smoothing
weak_smoothing

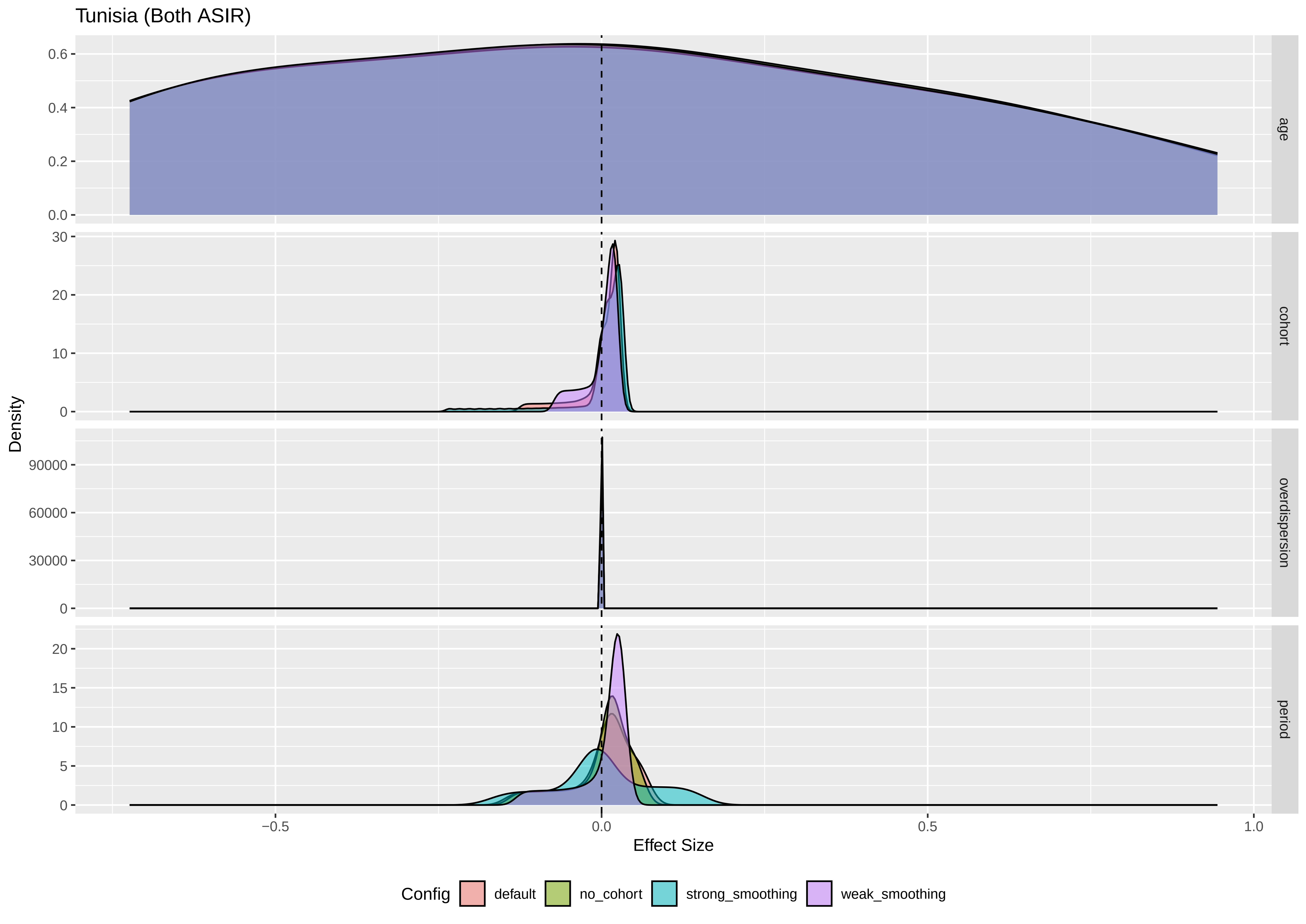

Tunisia (Both ASIR)
0.6
0.4
0.2
0.0
age
30
20
10
0
cohort
90000
60000
30000
0
overdispersion
20
15
10
5
0
period
Density
−0.5
0.0
0.5
1.0
Effect Size
Config
default
no_cohort
strong_smoothing
weak_smoothing

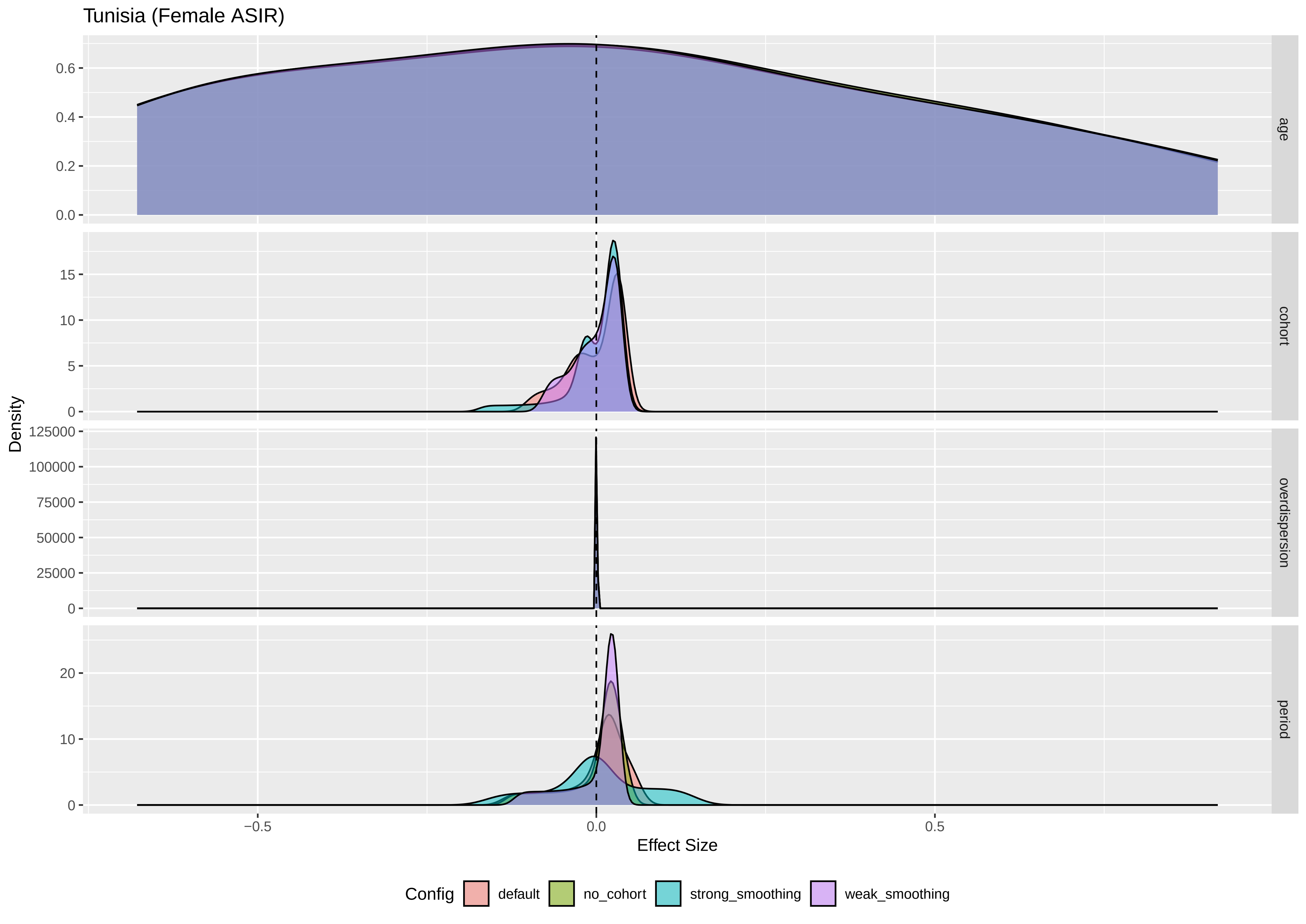
Tunisia (Female ASIR)
age
cohort
overdispersion
period
Density
Effect Size
−0.5
0.0
0.5
0.0
0.2
0.4
0.6
0
5
10
15
0
25000
50000
75000
100000
125000
0
10
20
Config
default
no_cohort
strong_smoothing
weak_smoothing

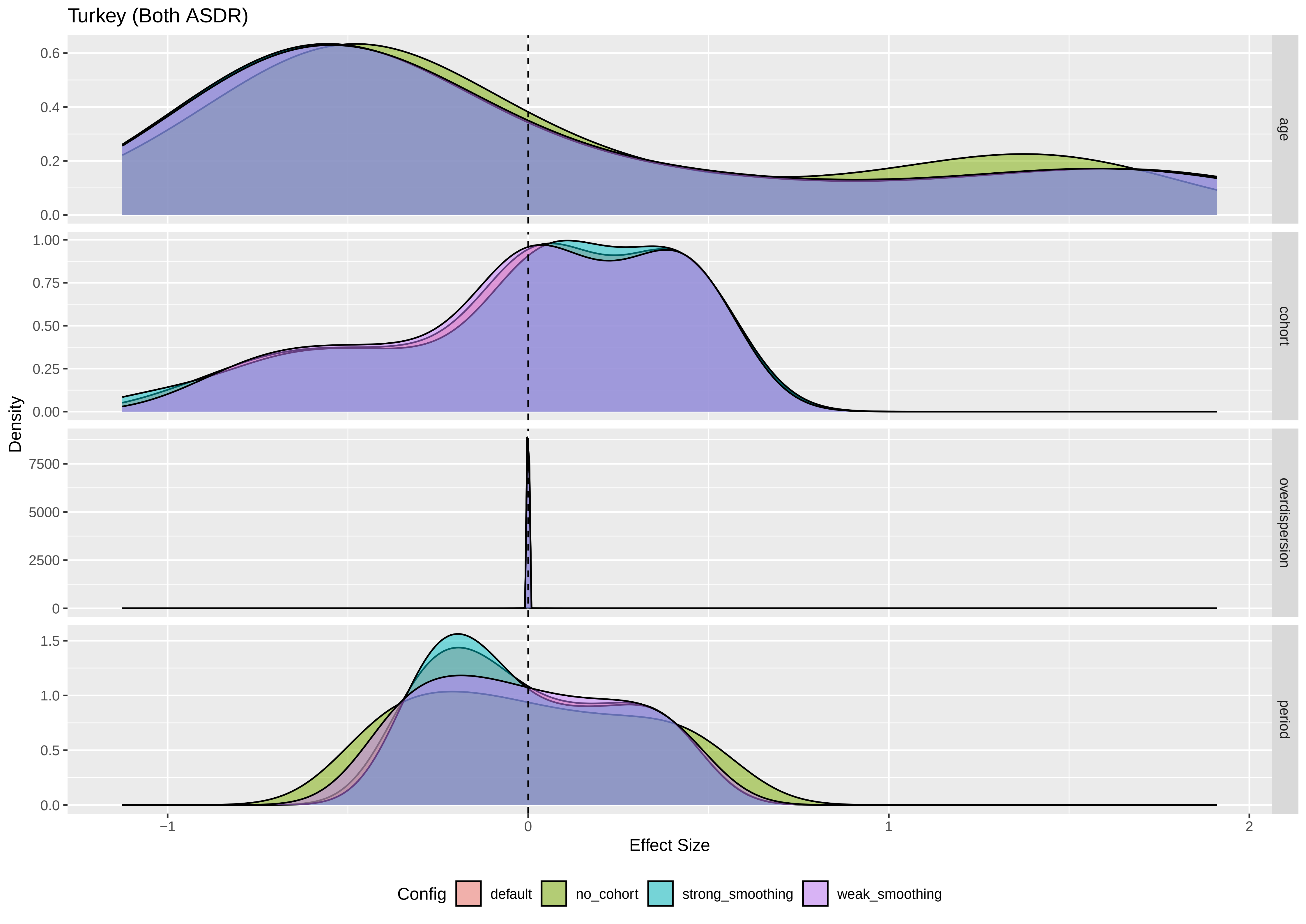

Turkey (Both ASDR)
0.6
0.4
0.2
0.0
age
1.00
0.75
0.50
0.25
0.00
cohort
Density
7500
5000
2500
0
overdispersion
1.5
1.0
0.5
0.0
period
−1
0
1
2
Effect Size
Config
default
no_cohort
strong_smoothing
weak_smoothing

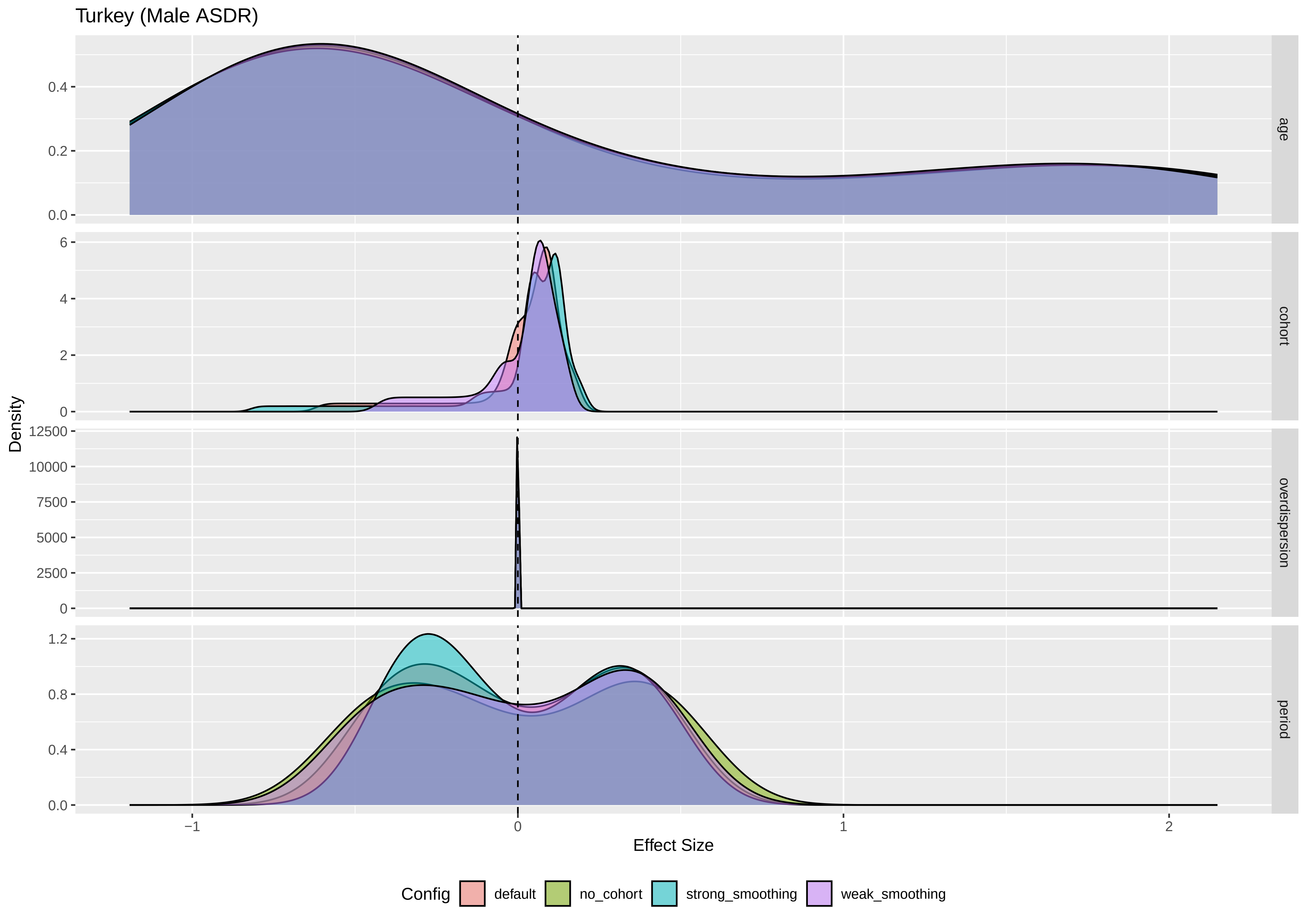

Turkey (Male ASDR)
age
cohort
overdispersion
period
Density
Effect Size
0.0
0.2
0.4
0
2
4
6
0
2500
5000
7500
10000
12500
0.0
0.4
0.8
1.2
−1
0
1
2
Config
default
no_cohort
strong_smoothing
weak_smoothing

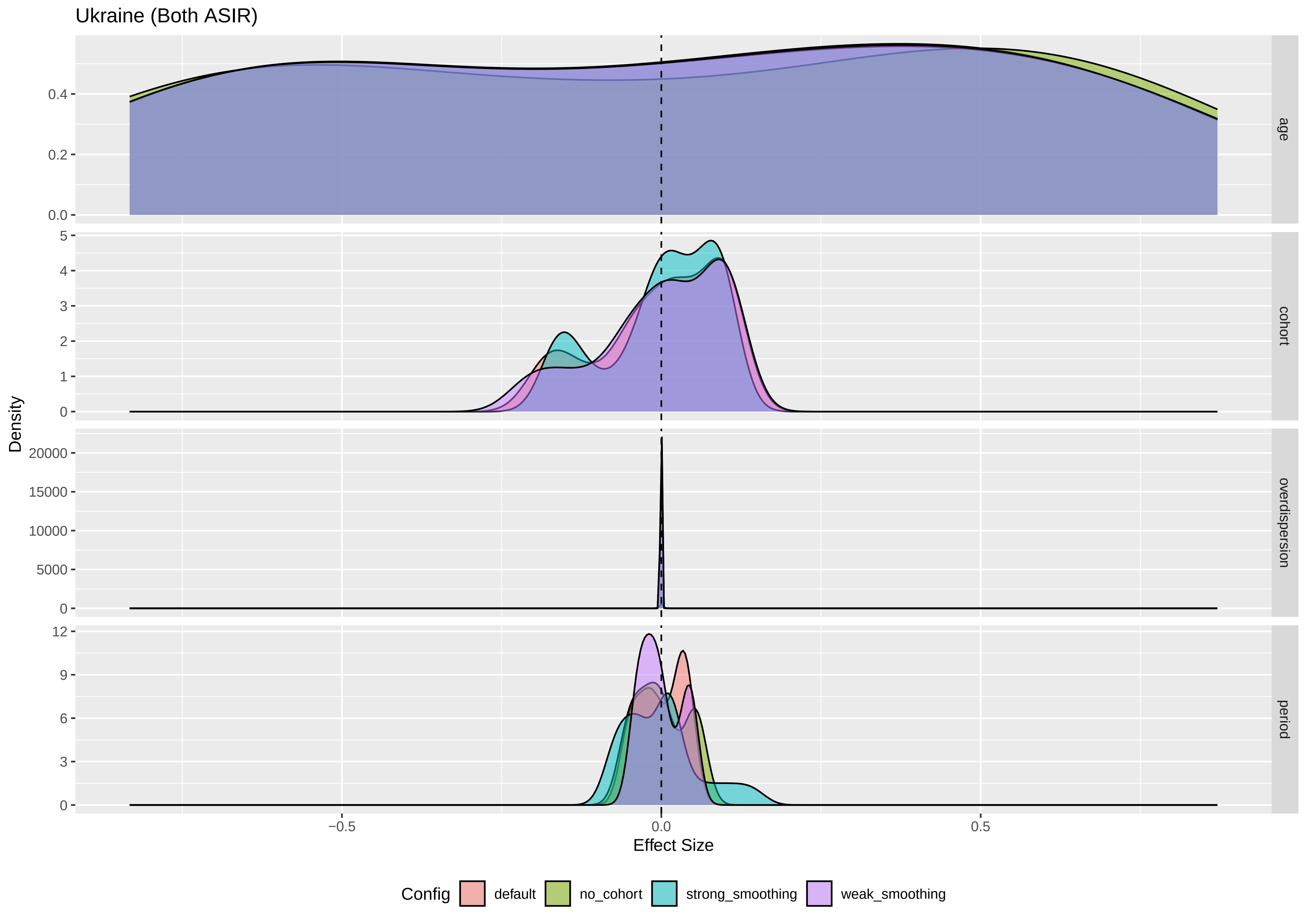

Ukraine (Both ASIR)
age
cohort
overdispersion
period
Density
Effect Size
0.0
0.2
0.4
0
1
2
3
4
5
0
5000
10000
15000
20000
0
3
6
9
12
−0.5
0.0
0.5
Config
default
no_cohort
strong_smoothing
weak_smoothing

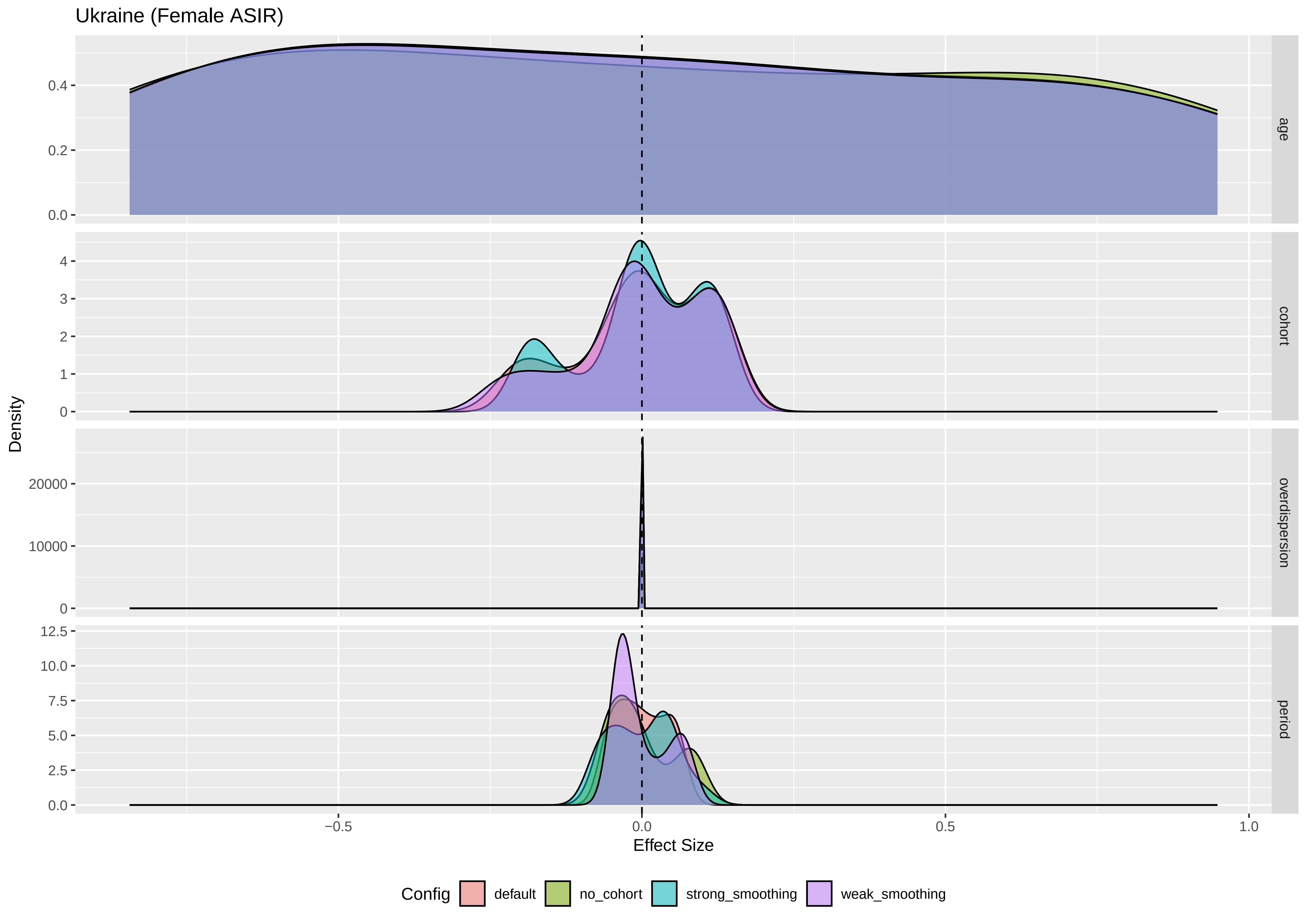

Ukraine (Female ASIR)
age
cohort
overdispersion
period
Density
Effect Size
0.0
0.2
0.4
0
1
2
3
4
10000
20000
2.5
5.0
7.5
10.0
12.5
−0.5
0.0
0.5
1.0
Config
default
no_cohort
strong_smoothing
weak_smoothing

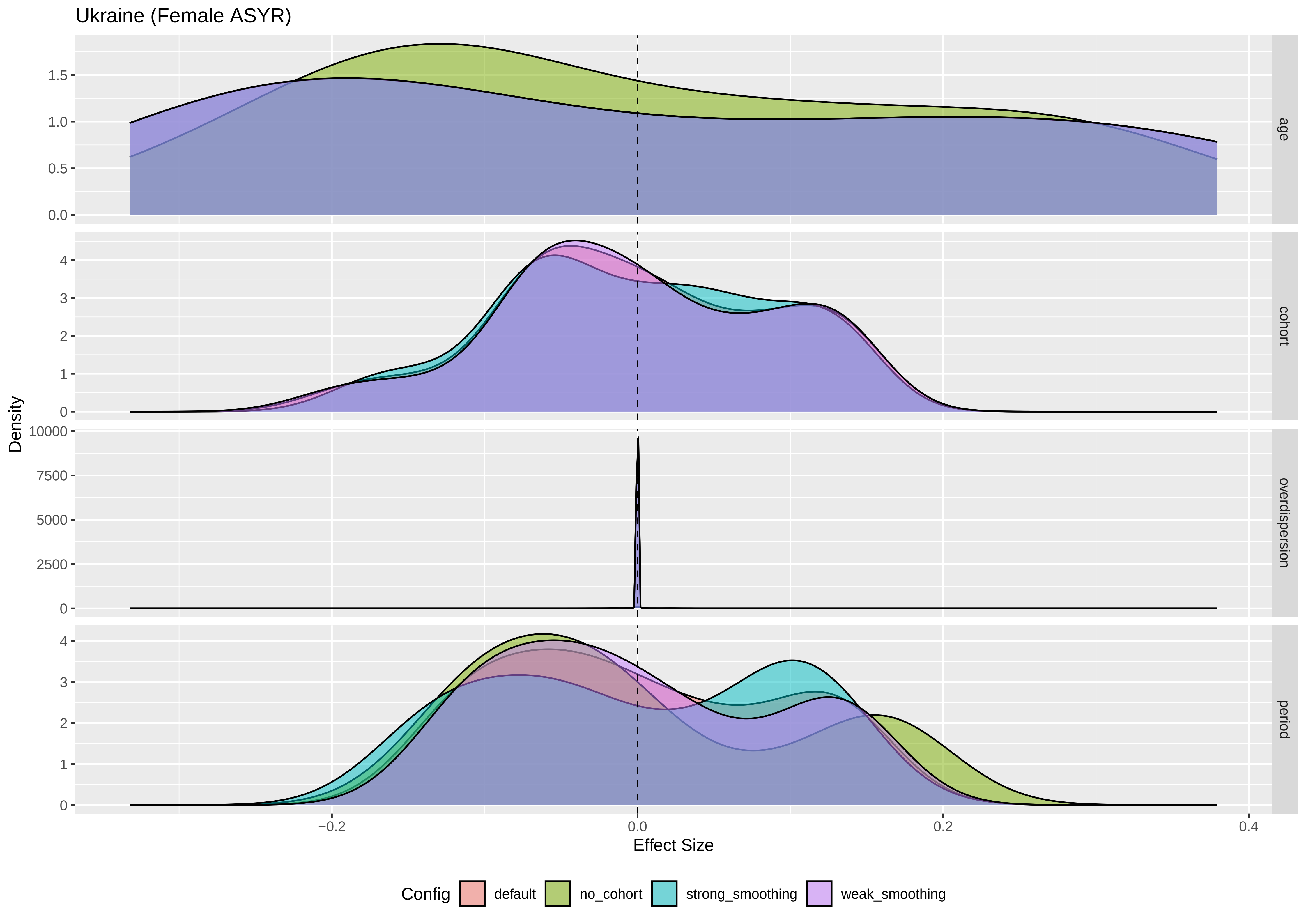

Ukraine (Female ASYR)
Density
Effect Size
age
cohort
overdispersion
period
1.5
1.0
0.5
0.0
4
3
2
1
0
10000
7500
5000
2500
−0.2
0.0
0.2
0.4
Config
default
no_cohort
strong_smoothing
weak_smoothing

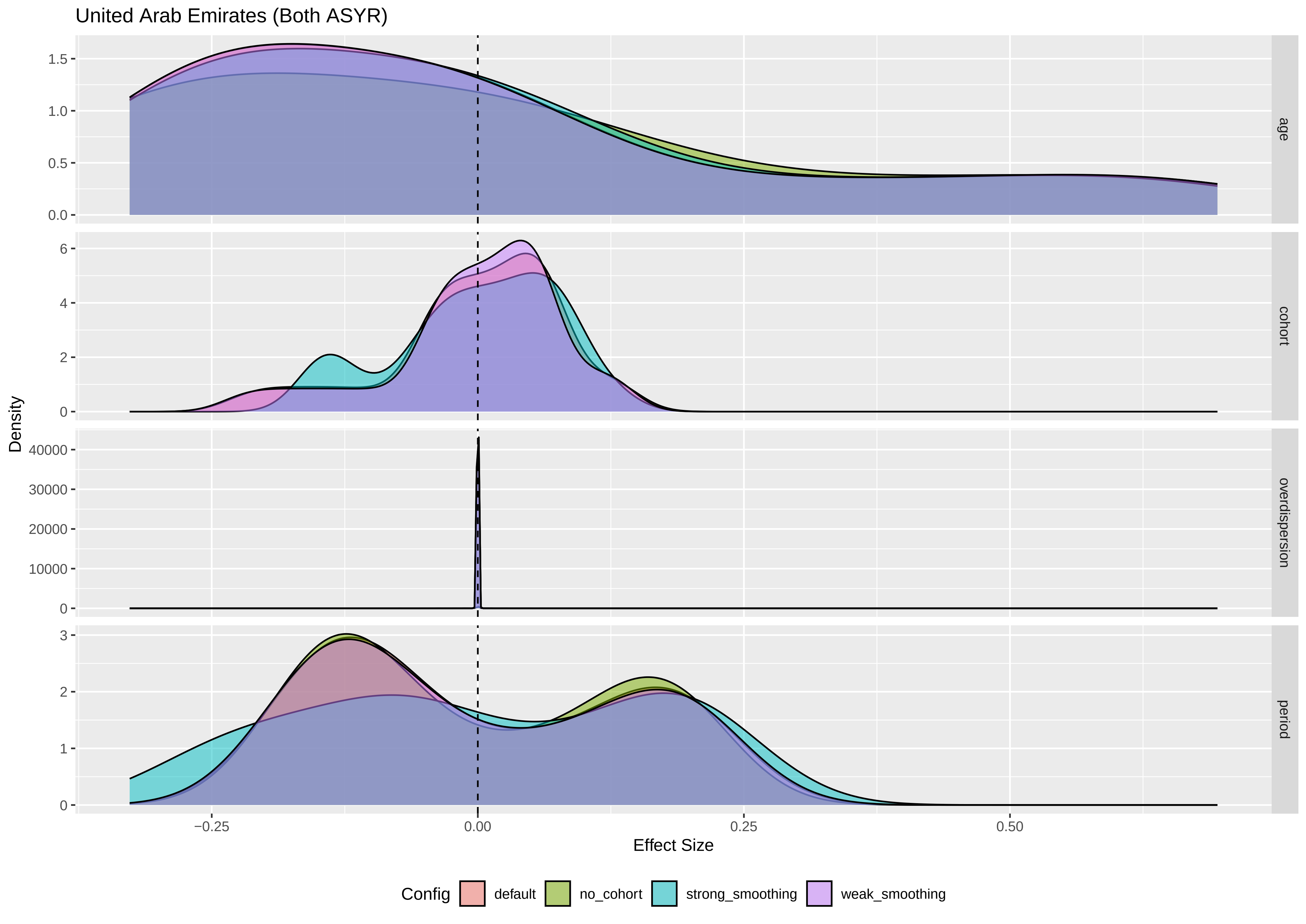
United Arab Emirates (Both ASYR)
age
cohort
overdispersion
period
Density
Effect Size
−0.25
0.00
0.25
0.50
0.0
0.5
1.0
1.5
0
2
4
6
0
10000
20000
30000
40000
0
1
2
3
Config
default
no_cohort
strong_smoothing
weak_smoothing

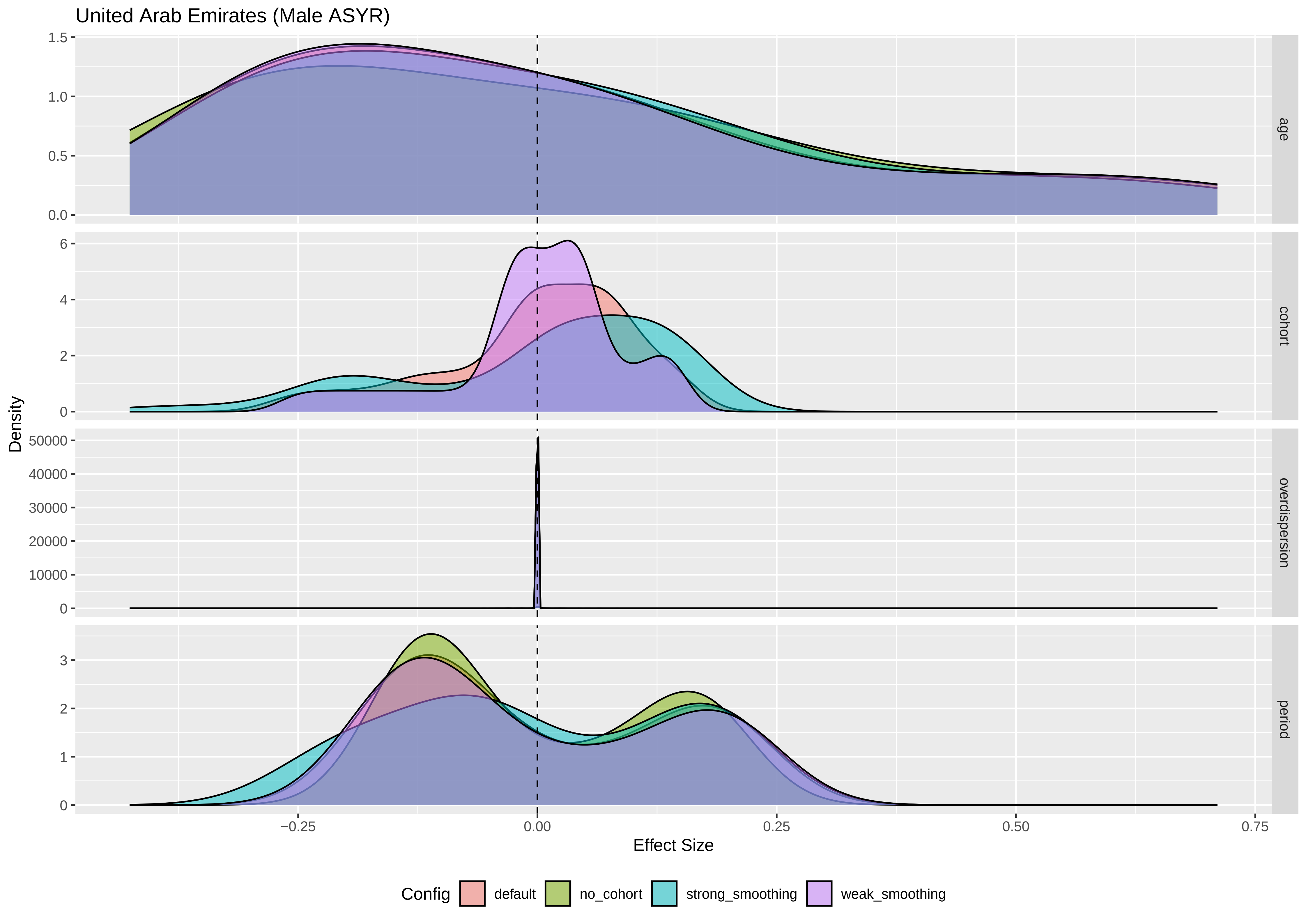

United Arab Emirates (Male ASYR)
Density
Effect Size
age
cohort
overdispersion
period
1.5
1.0
0.5
0.0
6
4
2
0
50000
40000
30000
20000
10000
0
3
2
1
0
−0.25
0.00
0.25
0.50
0.75
Config
default
no_cohort
strong_smoothing
weak_smoothing

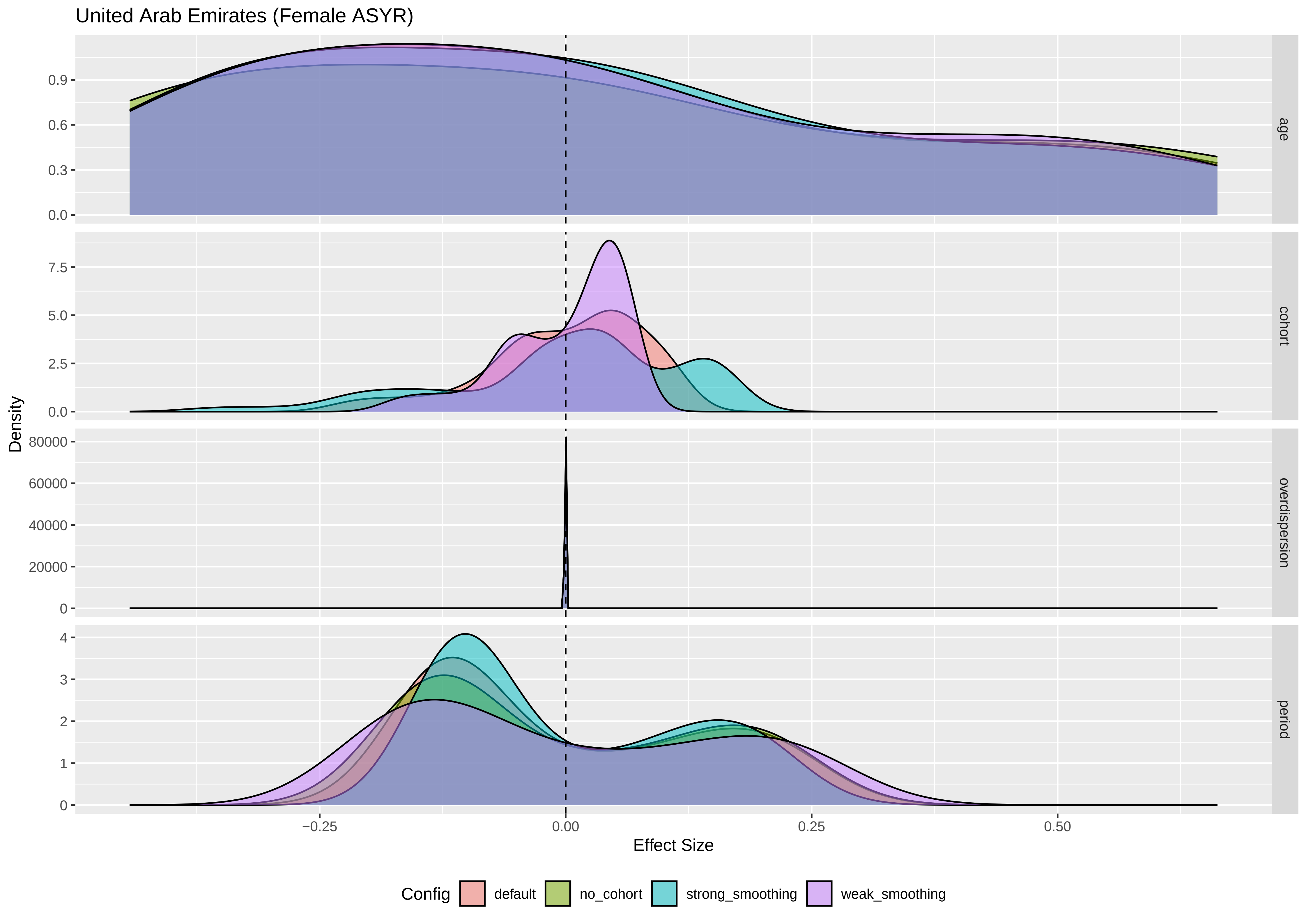

United Arab Emirates (Female ASYR)
Density
Effect Size
age
cohort
overdispersion
period
0.0
0.3
0.6
0.9
2.5
5.0
7.5
0
20000
40000
60000
80000
1
2
3
4
−0.25
0.00
0.25
0.50
Config
default
no_cohort
strong_smoothing
weak_smoothing

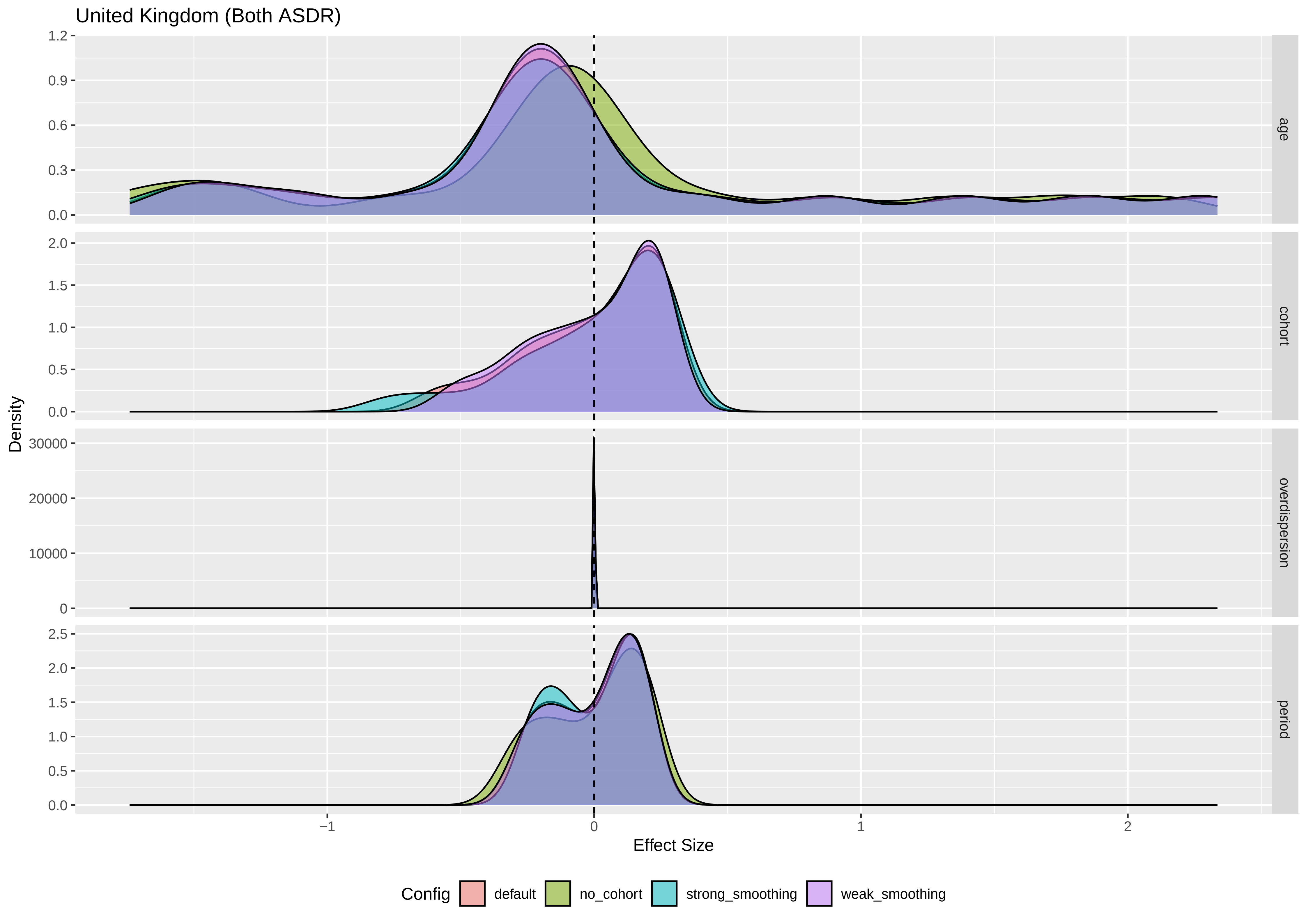
United Kingdom (Both ASDR)
Density
Effect Size
age
cohort
overdispersion
period
1.2
0.9
0.6
0.3
0.0
2.0
1.5
1.0
0.5
0.0
30000
20000
10000
0
2.5
2.0
1.5
1.0
0.5
0.0
−1
0
1
2
Config
default
no_cohort
strong_smoothing
weak_smoothing

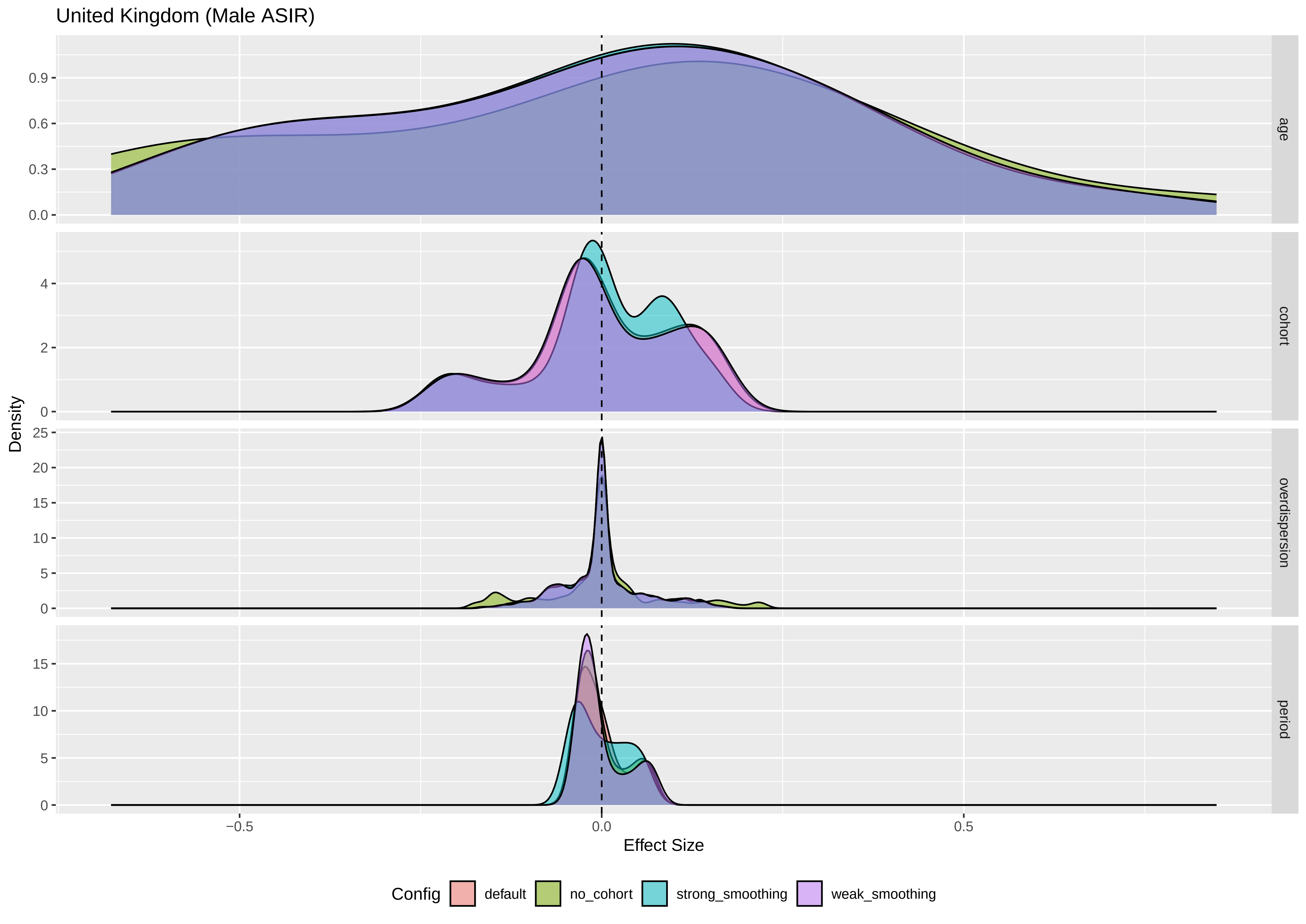

United Kingdom (Male ASIR)
Density
Effect Size
age
cohort
overdispersion
period
0.0
0.3
0.6
0.9
0
2
4
5
10
15
20
25
−0.5
0.0
0.5
Config
default
no_cohort
strong_smoothing
weak_smoothing

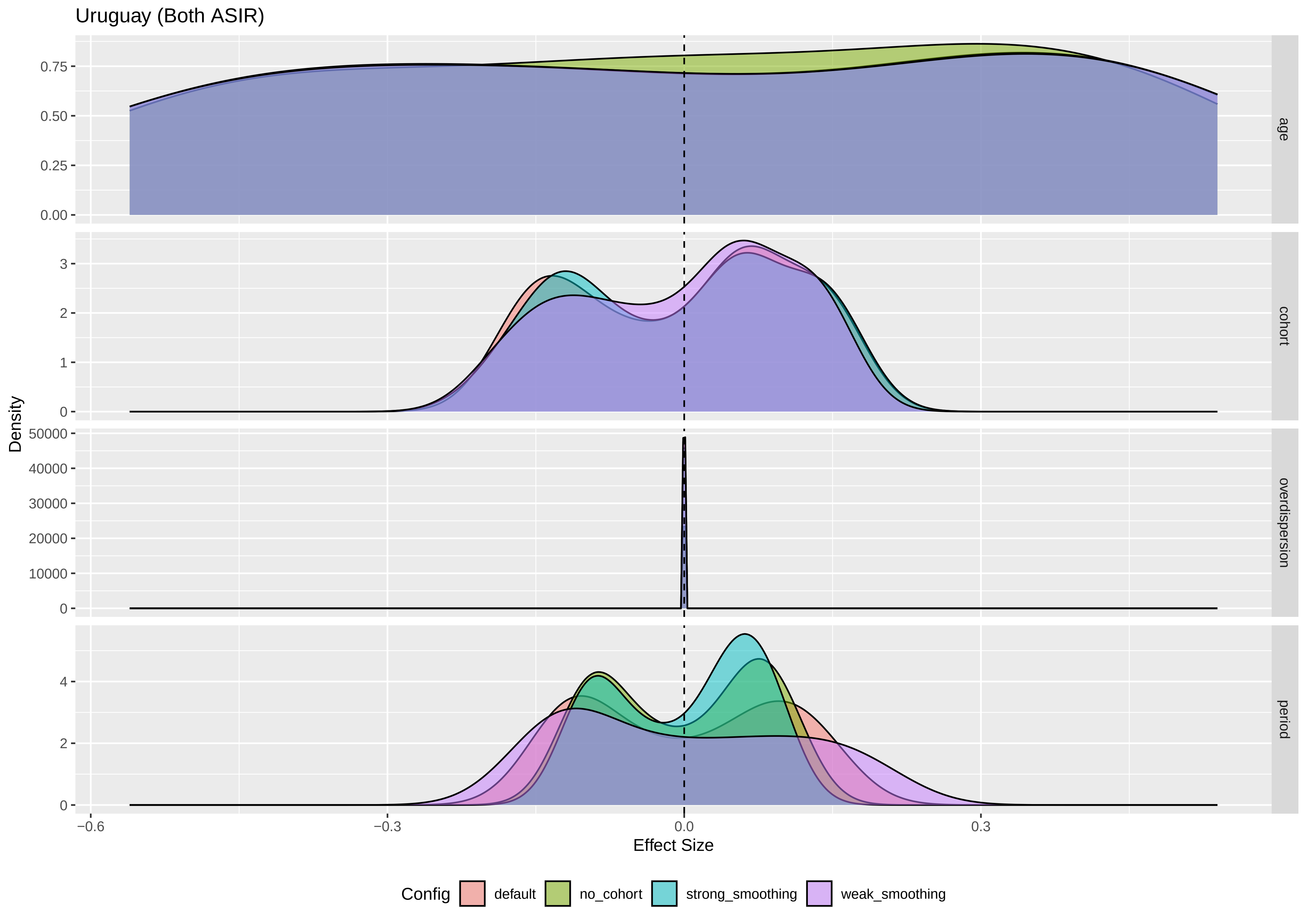

Uruguay (Both ASIR)
age
cohort
overdispersion
period
Density
0.00
0.25
0.50
0.75
0
1
2
3
0
10000
20000
30000
40000
50000
0
2
4
-0.6
-0.3
0.0
0.3
Effect Size
Config
default
no_cohort
strong_smoothing
weak_smoothing

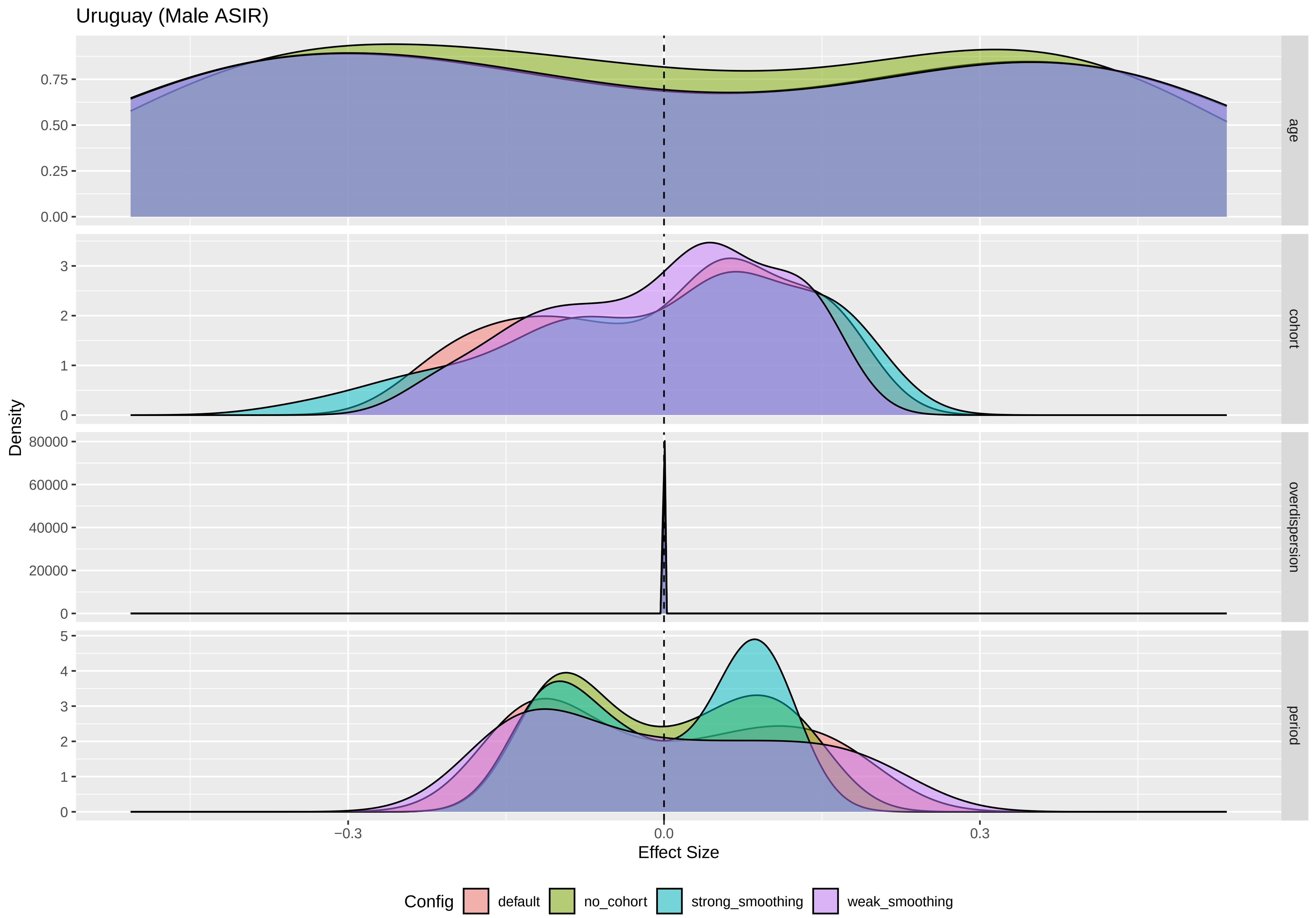

Uruguay (Male ASIR)
age
cohort
overdispersion
period
Density
Effect Size
−0.3
0.0
0.3
0.00
0.25
0.50
0.75
0
1
2
3
20000
40000
60000
80000
4
5
Config
default
no_cohort
strong_smoothing
weak_smoothing

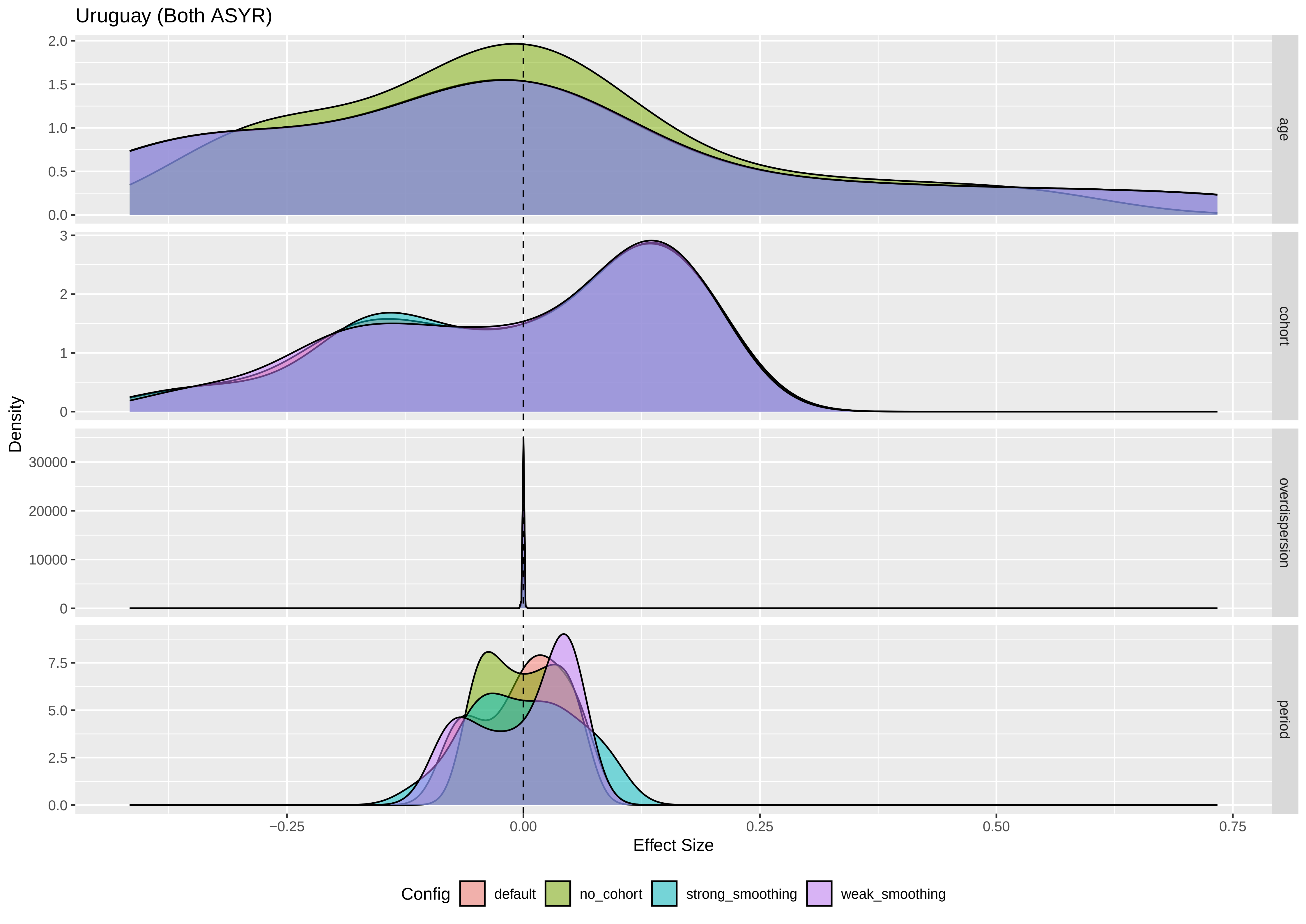

Uruguay (Both ASYR)
Density
Effect Size
age
cohort
overdispersion
period
2.0
1.5
1.0
0.5
0.0
3
2
1
0
30000
20000
10000
0
7.5
5.0
2.5
0.0
−0.25
0.00
0.25
0.50
0.75
Config
default
no_cohort
strong_smoothing
weak_smoothing

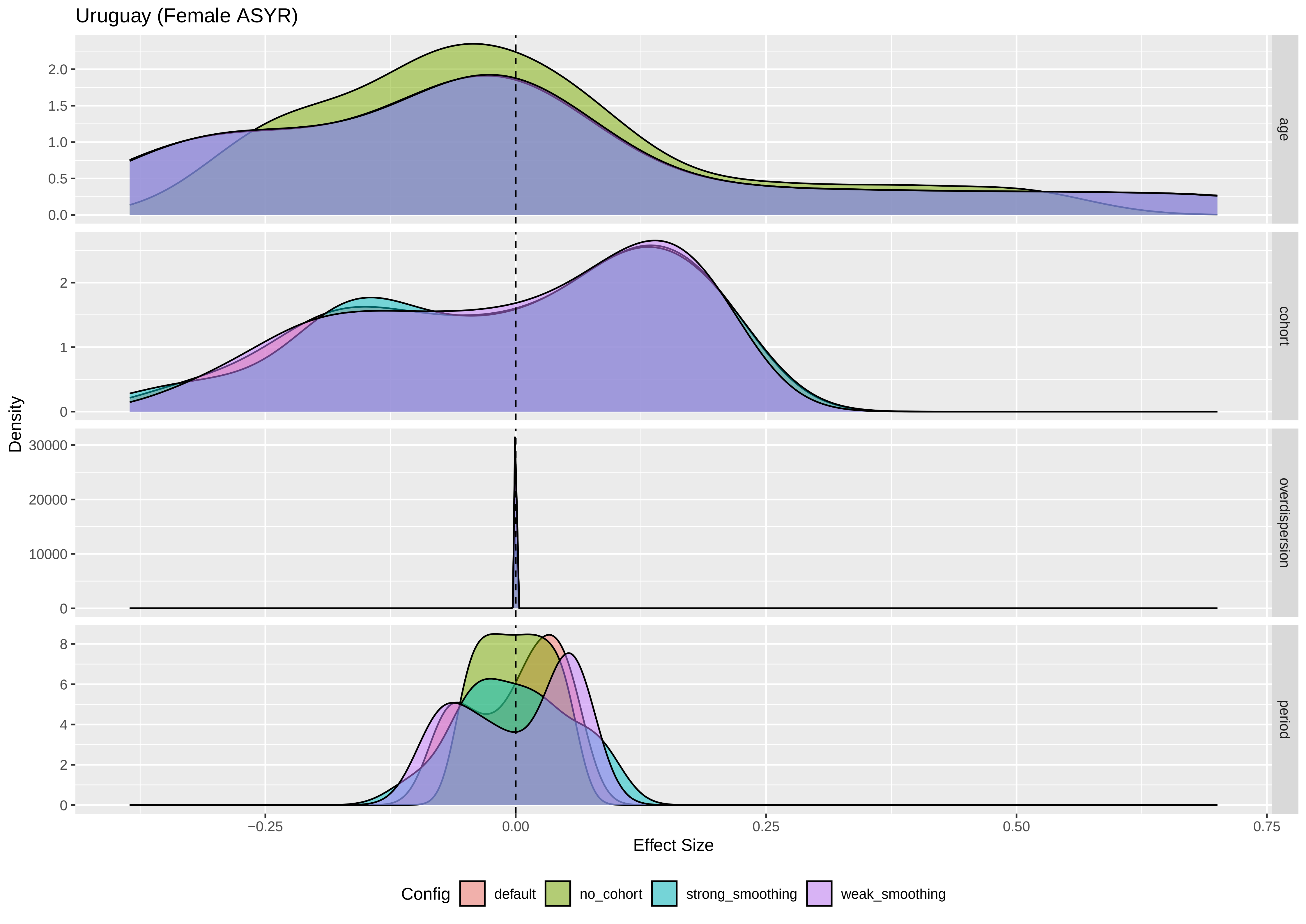
Uruguay (Female ASYR)
age
cohort
overdispersion
period
Density
Effect Size
−0.25
0.00
0.25
0.50
0.75
0.0
0.5
1.0
1.5
2.0
0
1
2
0
10000
20000
30000
0
2
4
6
8
Config
default
no_cohort
strong_smoothing
weak_smoothing

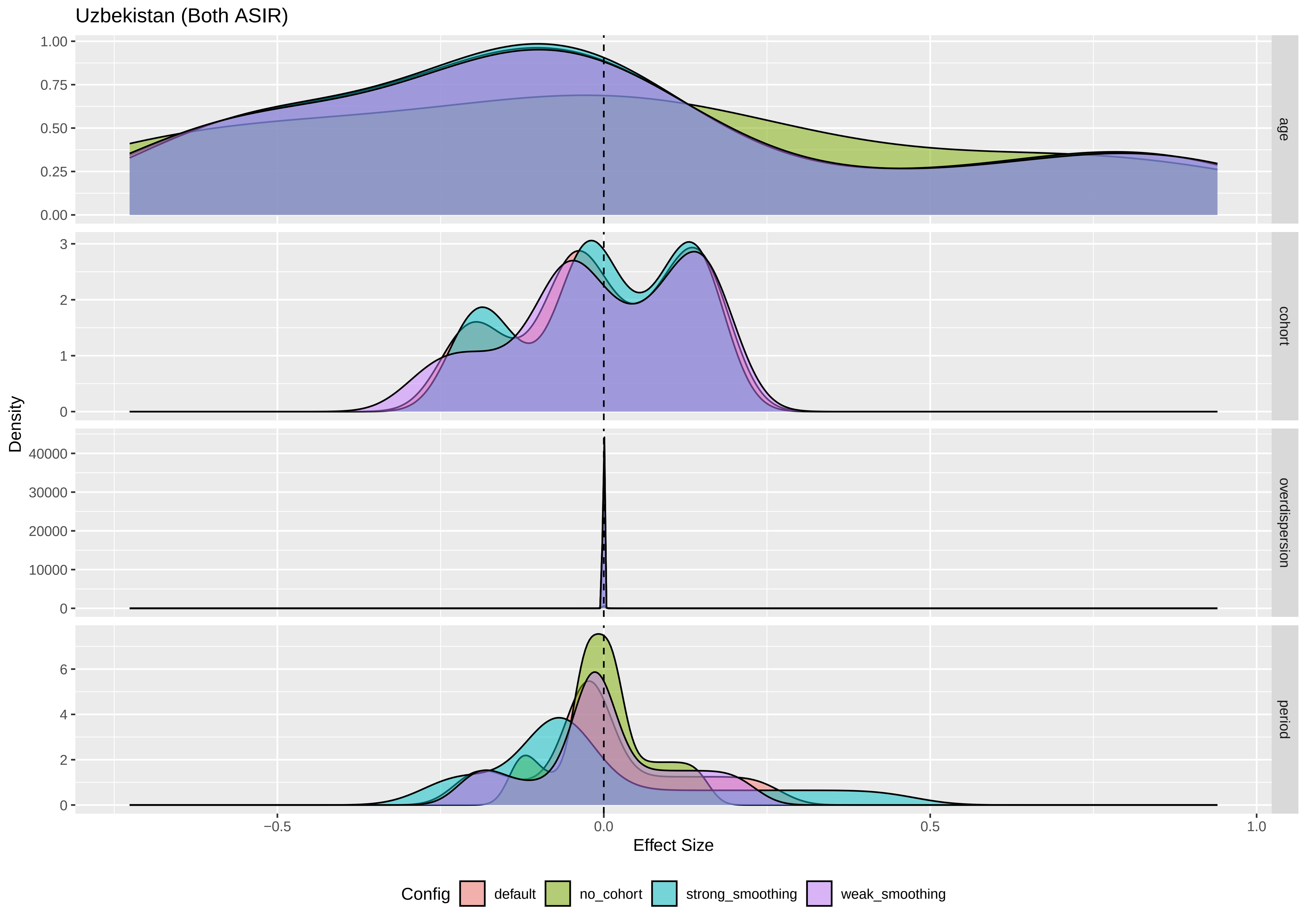

Uzbekistan (Both ASIR)
Density
Effect Size
age
cohort
overdispersion
period
1.00
0.75
0.50
0.25
0.00
3
2
1
0
40000
30000
20000
10000
0
6
4
2
0
−0.5
0.0
0.5
1.0
Config
default
no_cohort
strong_smoothing
weak_smoothing

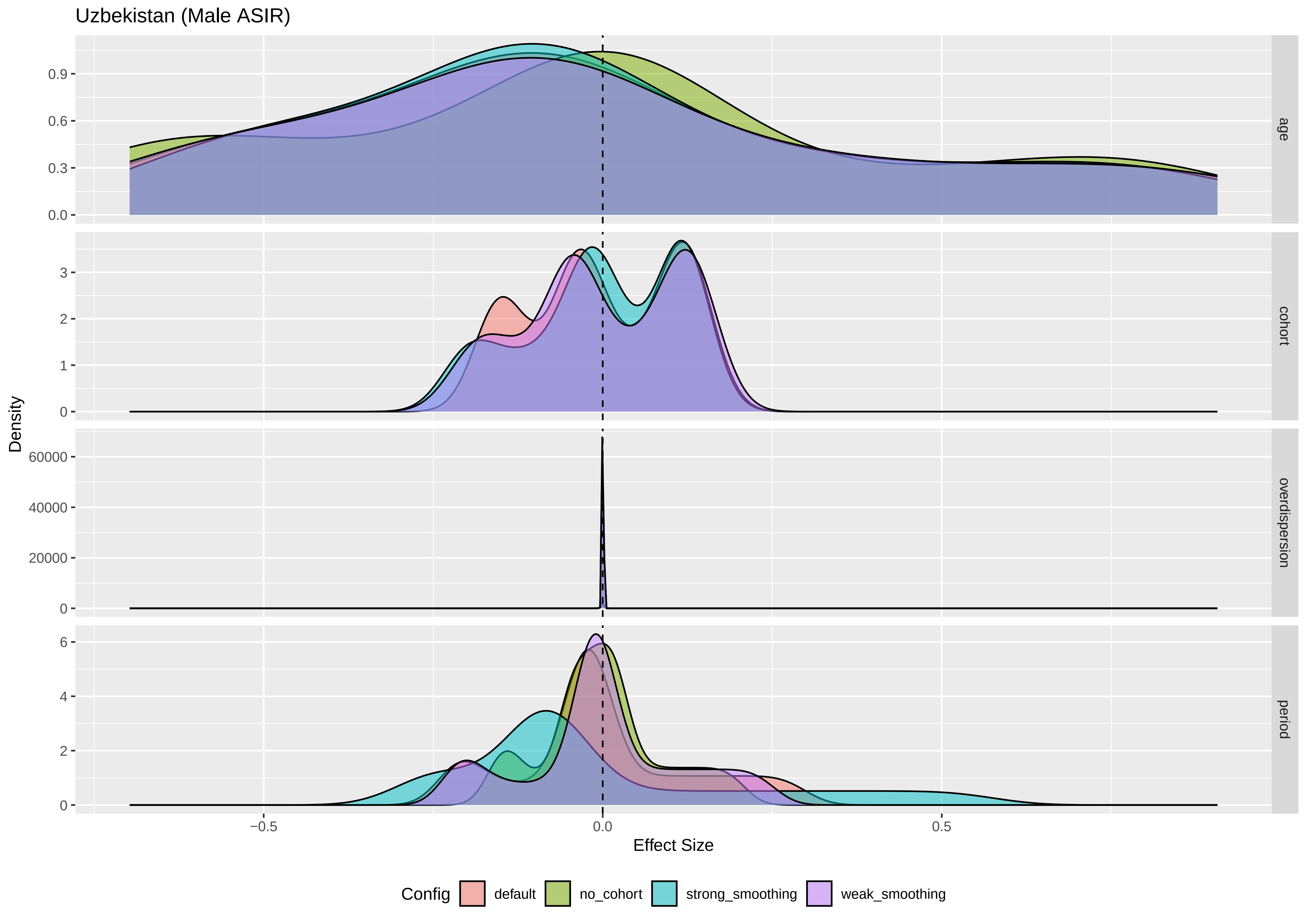

Uzbekistan (Male ASIR)
age
cohort
overdispersion
period
Density
Effect Size
0.0
0.3
0.6
0.9
0
1
2
3
0
20000
40000
60000
0
2
4
6
−0.5
0.0
0.5
Config
default
no_cohort
strong_smoothing
weak_smoothing

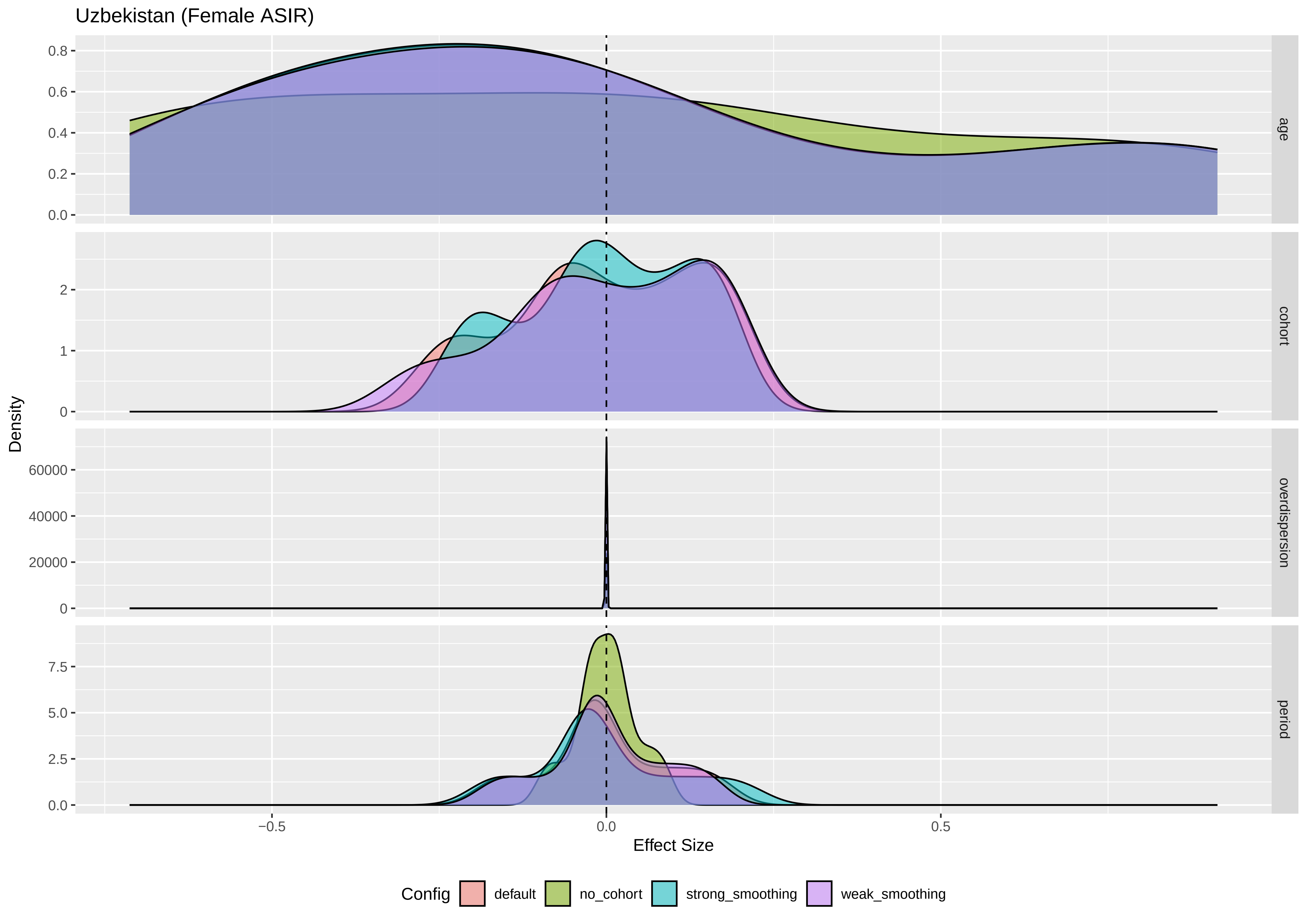

Uzbekistan (Female ASIR)
Density
Effect Size
age
cohort
overdispersion
period
0.0
0.2
0.4
0.6
0.8
0
1
2
0
20000
40000
60000
0.0
2.5
5.0
7.5
−0.5
0.0
0.5
Config
default
no_cohort
strong_smoothing
weak_smoothing

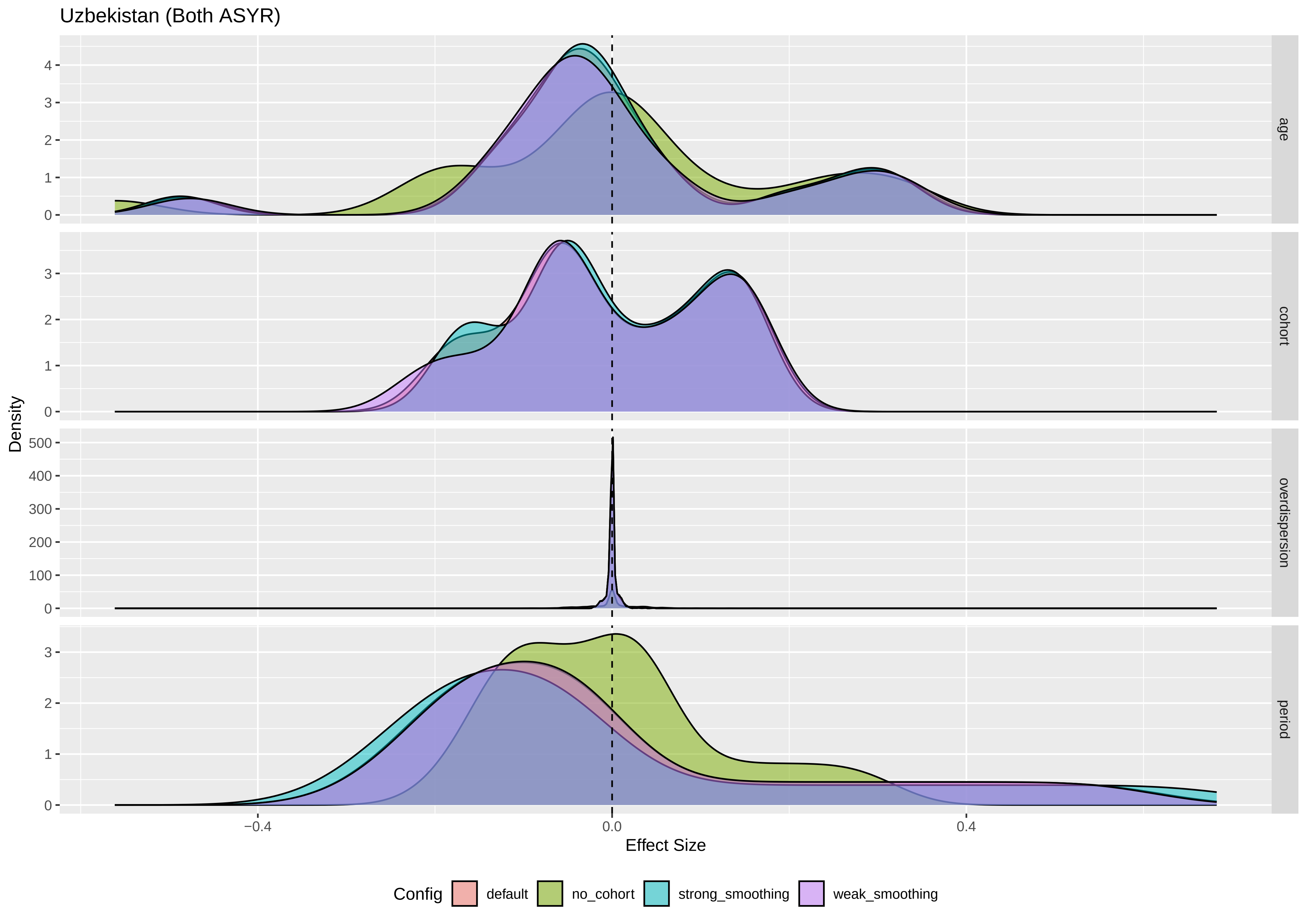
Uzbekistan (Both ASYR)
age
cohort
overdispersion
period
Density
Effect Size
-0.4
0.0
0.4
0
1
2
3
4
100
200
300
400
500
Config
default
no_cohort
strong_smoothing
weak_smoothing

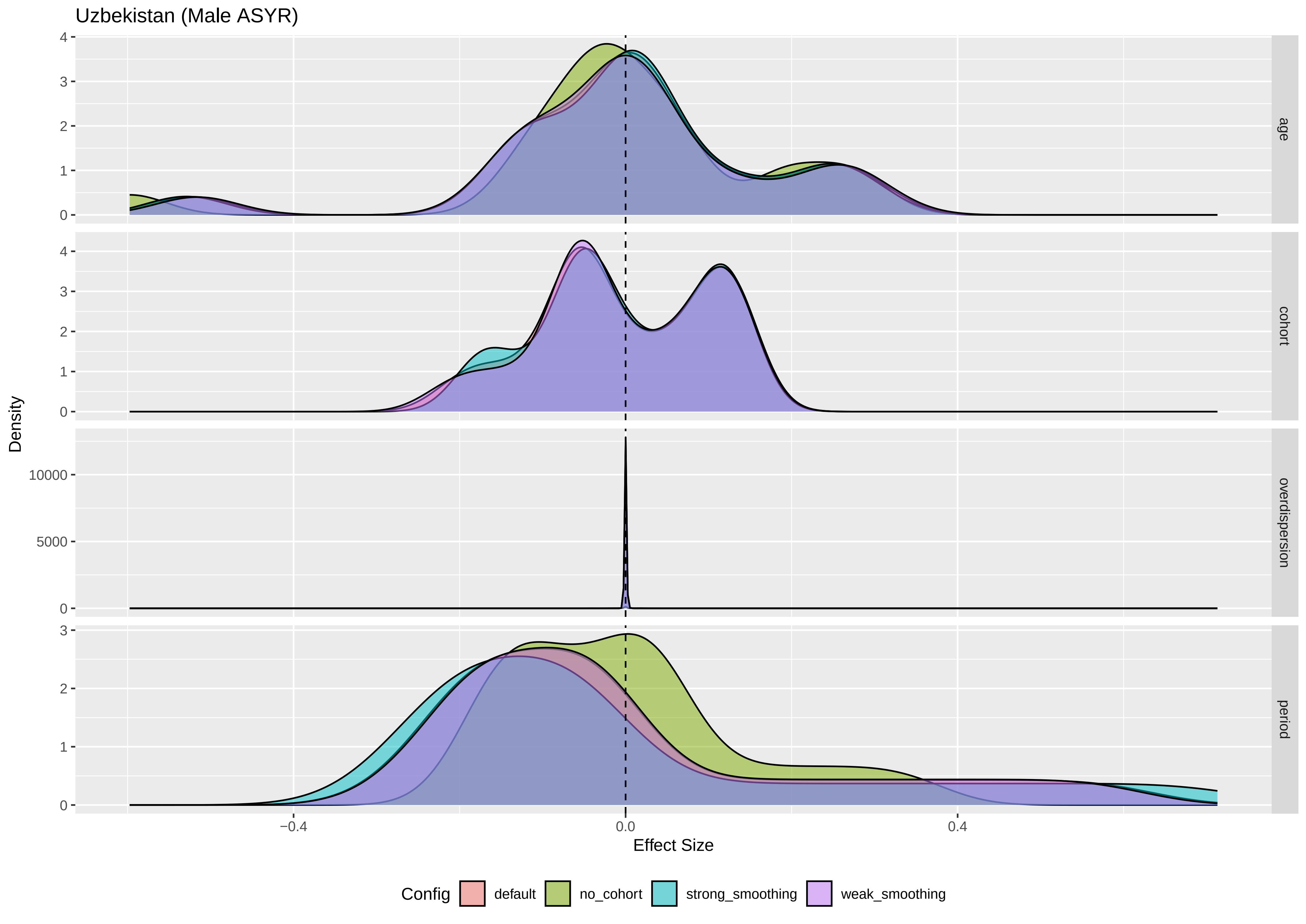

Uzbekistan (Male ASYR)
Density
Effect Size
age
cohort
overdispersion
period
−0.4
0.0
0.4
0
1
2
3
4
5000
10000
Config
default
no_cohort
strong_smoothing
weak_smoothing

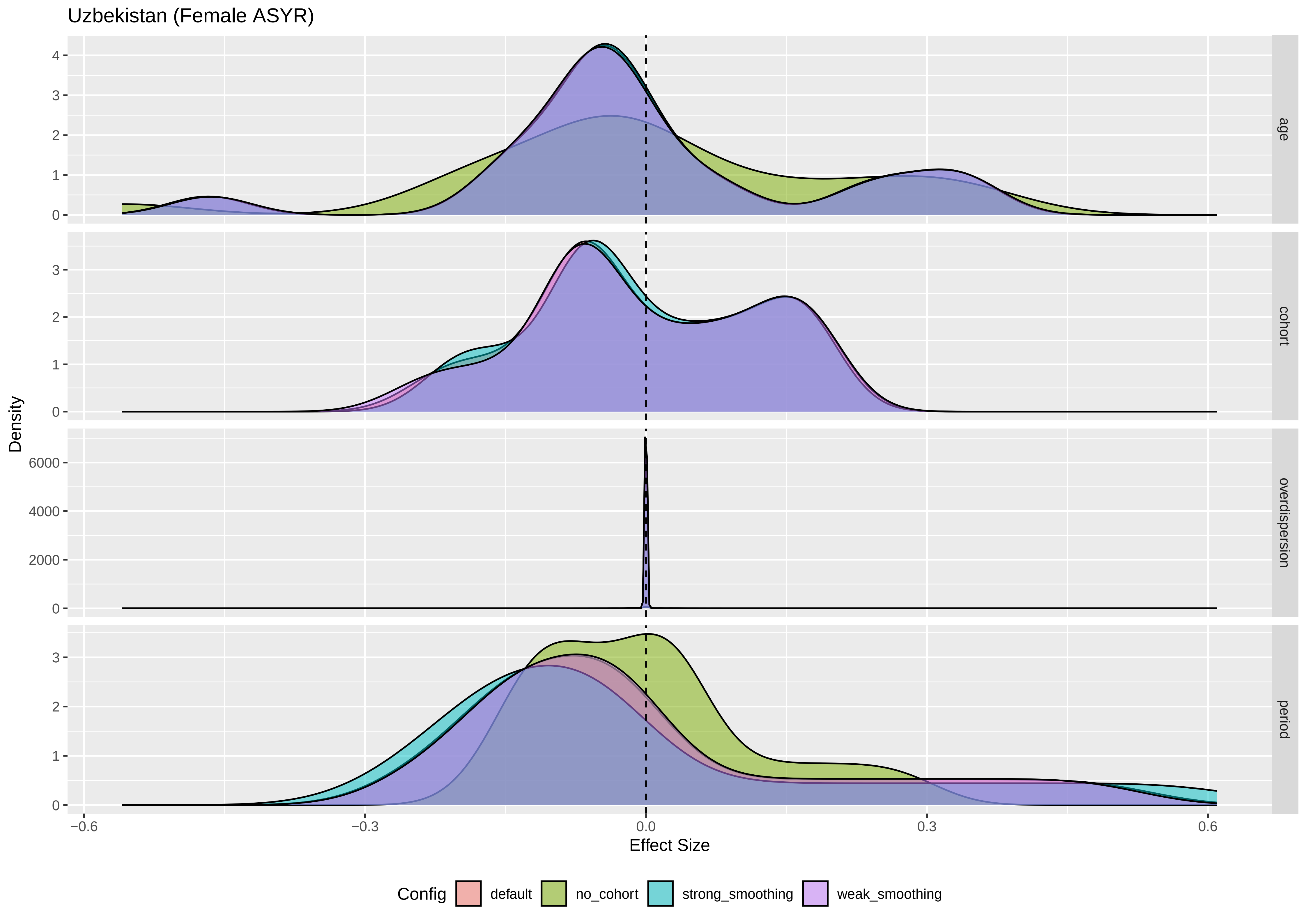

Uzbekistan (Female ASYR)
Density
Effect Size
age
cohort
overdispersion
period
−0.6
−0.3
0.0
0.3
0.6
0
1
2
3
4
0
1
2
3
0
2000
4000
6000
0
1
2
3
Config
default
no_cohort
strong_smoothing
weak_smoothing

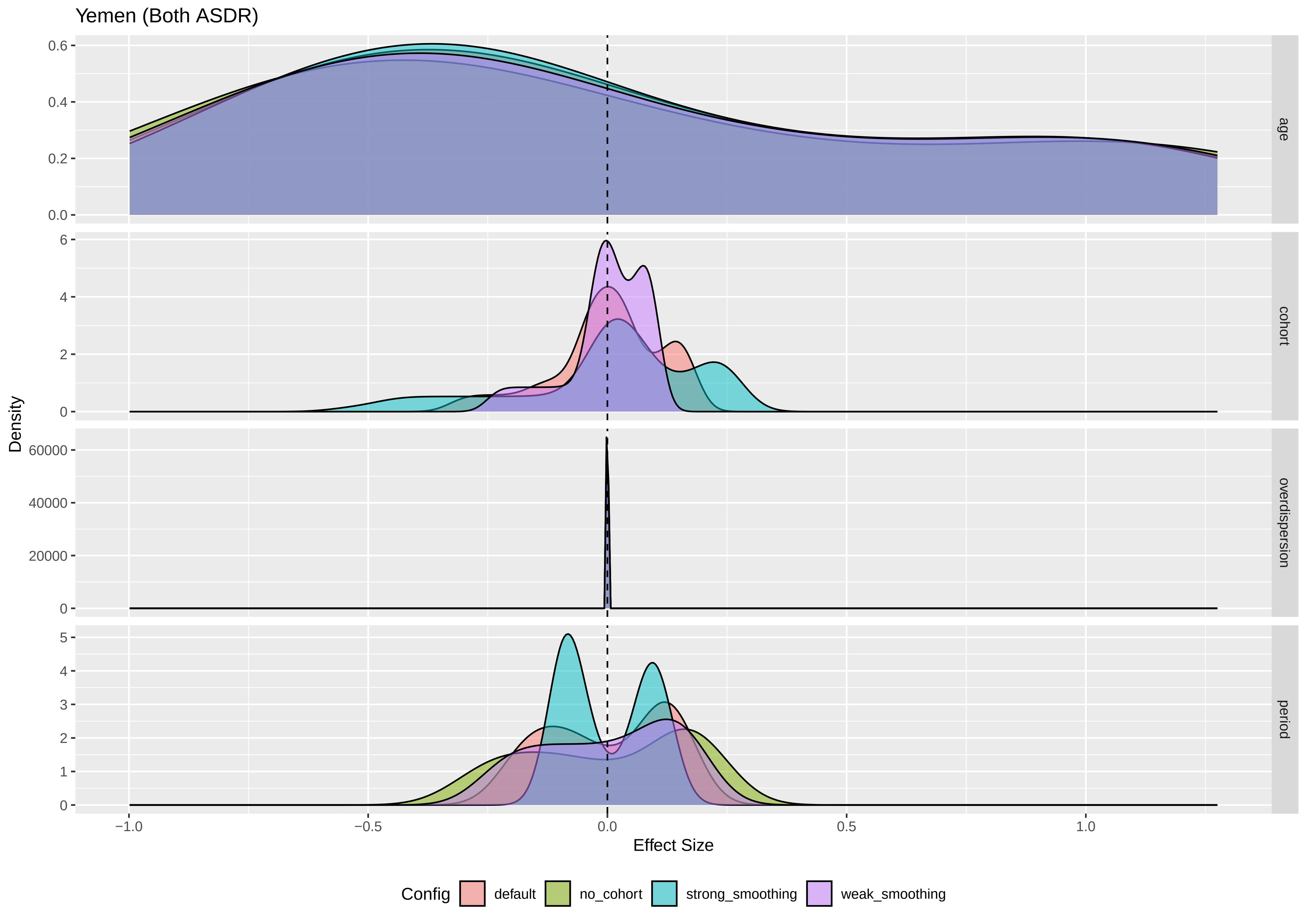

Yemen (Both ASDR)
Density
Effect Size
age
cohort
overdispersion
period
0.0
0.2
0.4
0.6
0
2
4
6
0
20000
40000
60000
0
1
2
3
4
5
−1.0
−0.5
0.0
0.5
1.0
Config
default
no_cohort
strong_smoothing
weak_smoothing

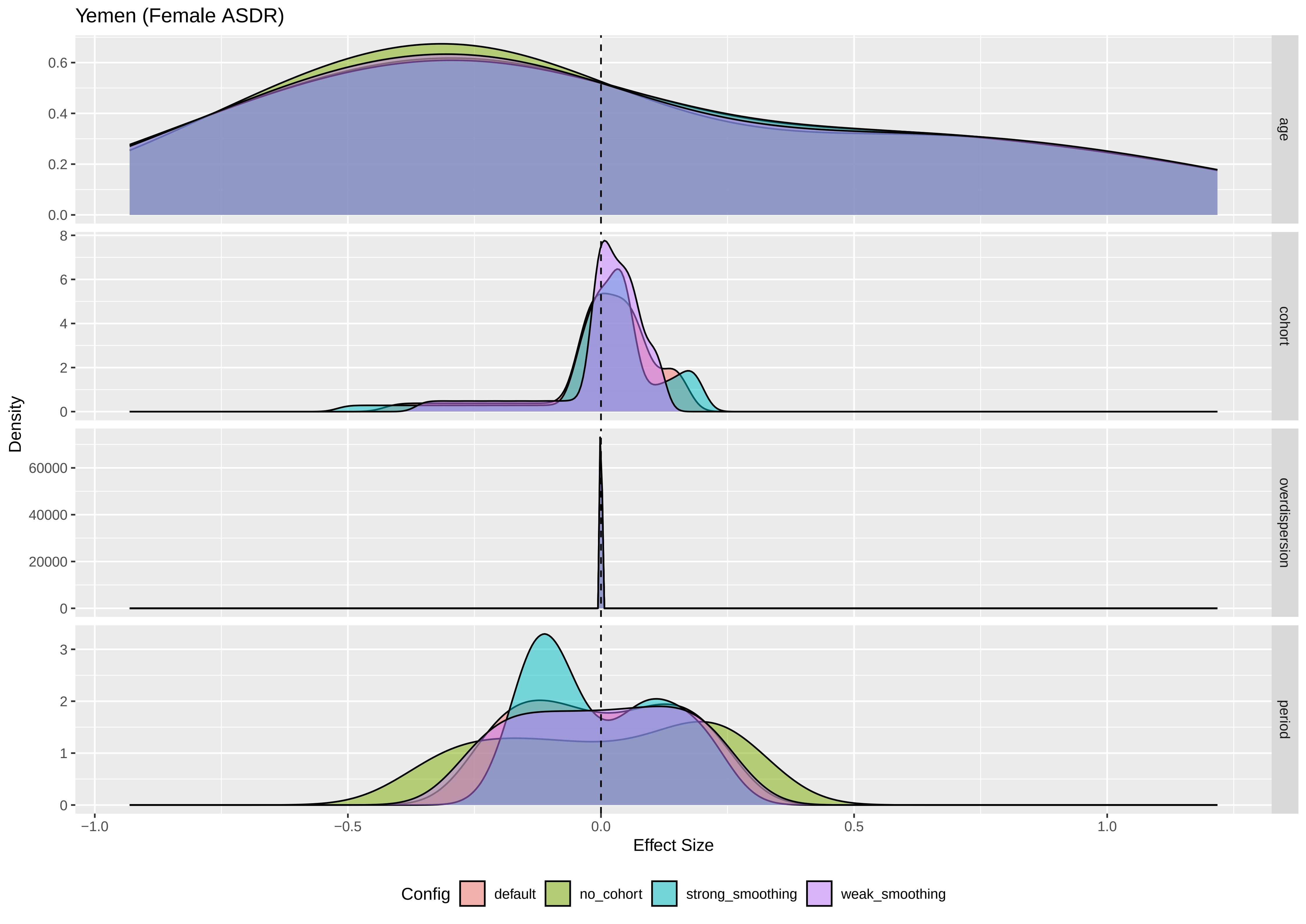
Yemen (Female ASDR)
age
cohort
overdispersion
period
Density
Effect Size
−1.0
−0.5
0.0
0.5
1.0
0.0
0.2
0.4
0.6
0
2
4
6
8
0
20000
40000
60000
0
1
2
3
Config
default
no_cohort
strong_smoothing
weak_smoothing

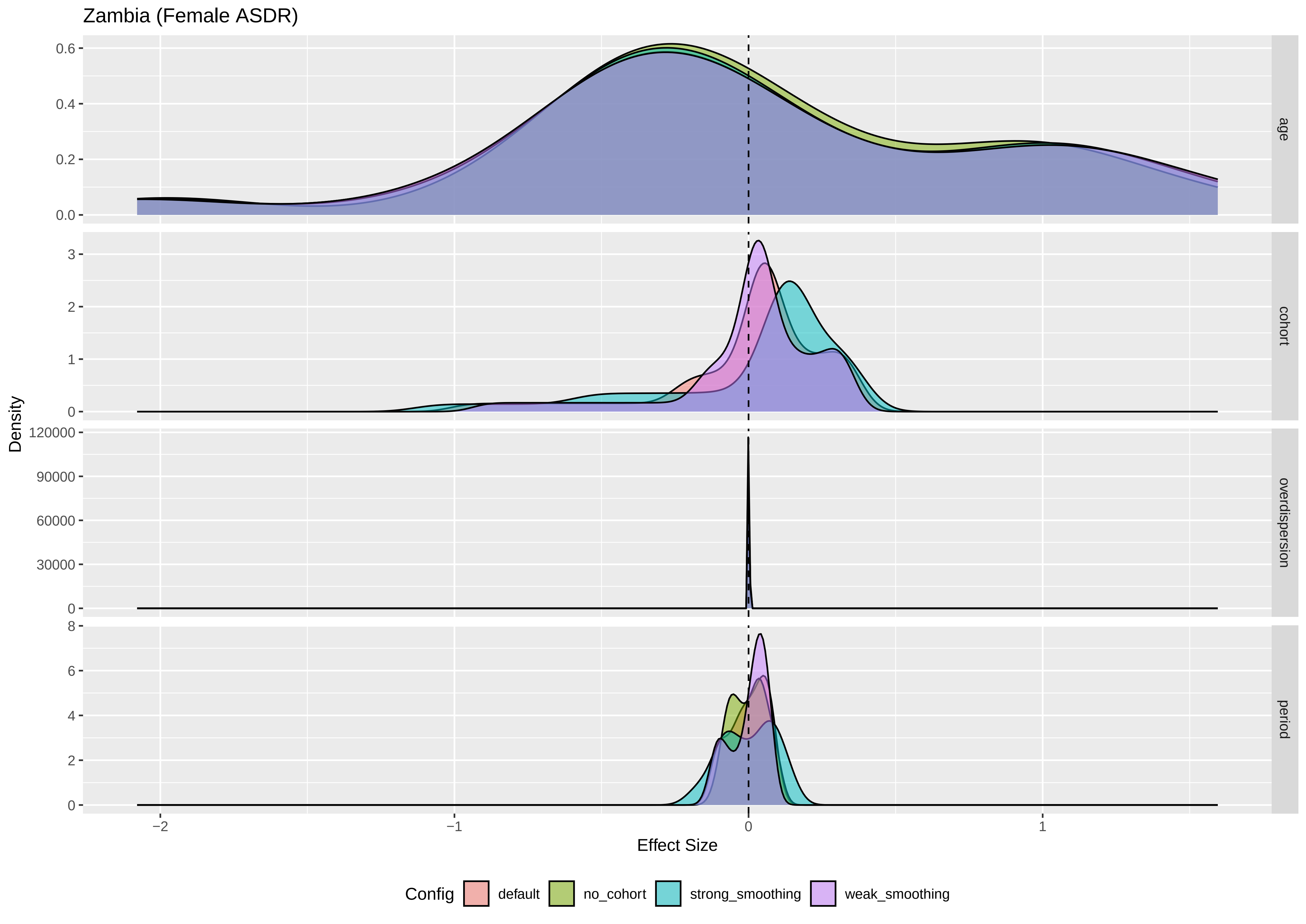

Zambia (Female ASDR)
Density
Effect Size
age
cohort
overdispersion
period
0.6
0.4
0.2
0.0
3
2
1
0
120000
90000
60000
30000
0
8
6
4
2
0
−2
−1
0
1
Config
default
no_cohort
strong_smoothing
weak_smoothing

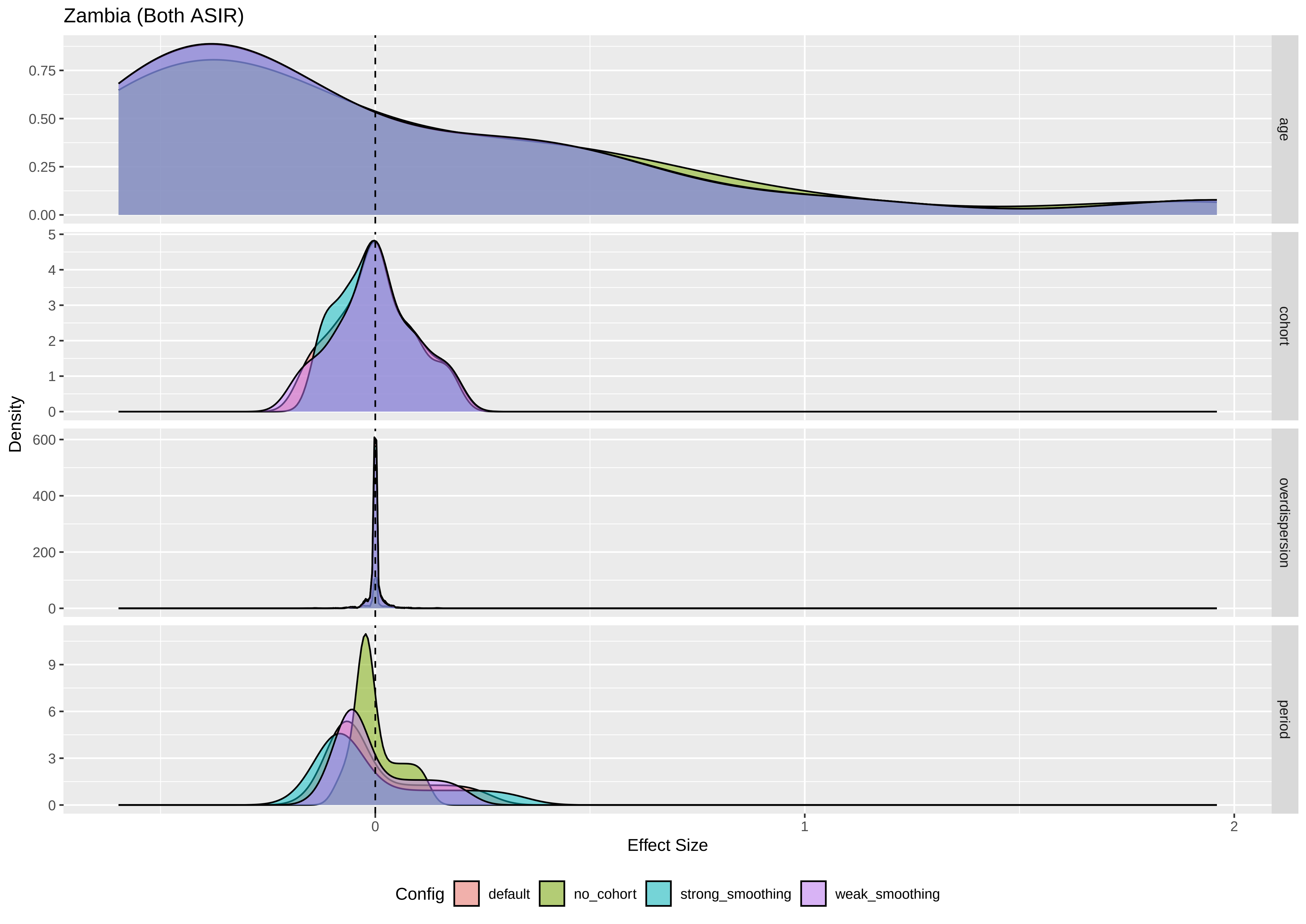

Zambia (Both ASIR)
age
cohort
overdispersion
period
Density
Effect Size
0.00
0.25
0.50
0.75
0
1
2
3
4
5
0
200
400
600
0
3
6
9
0
1
2
Config
default
no_cohort
strong_smoothing
weak_smoothing

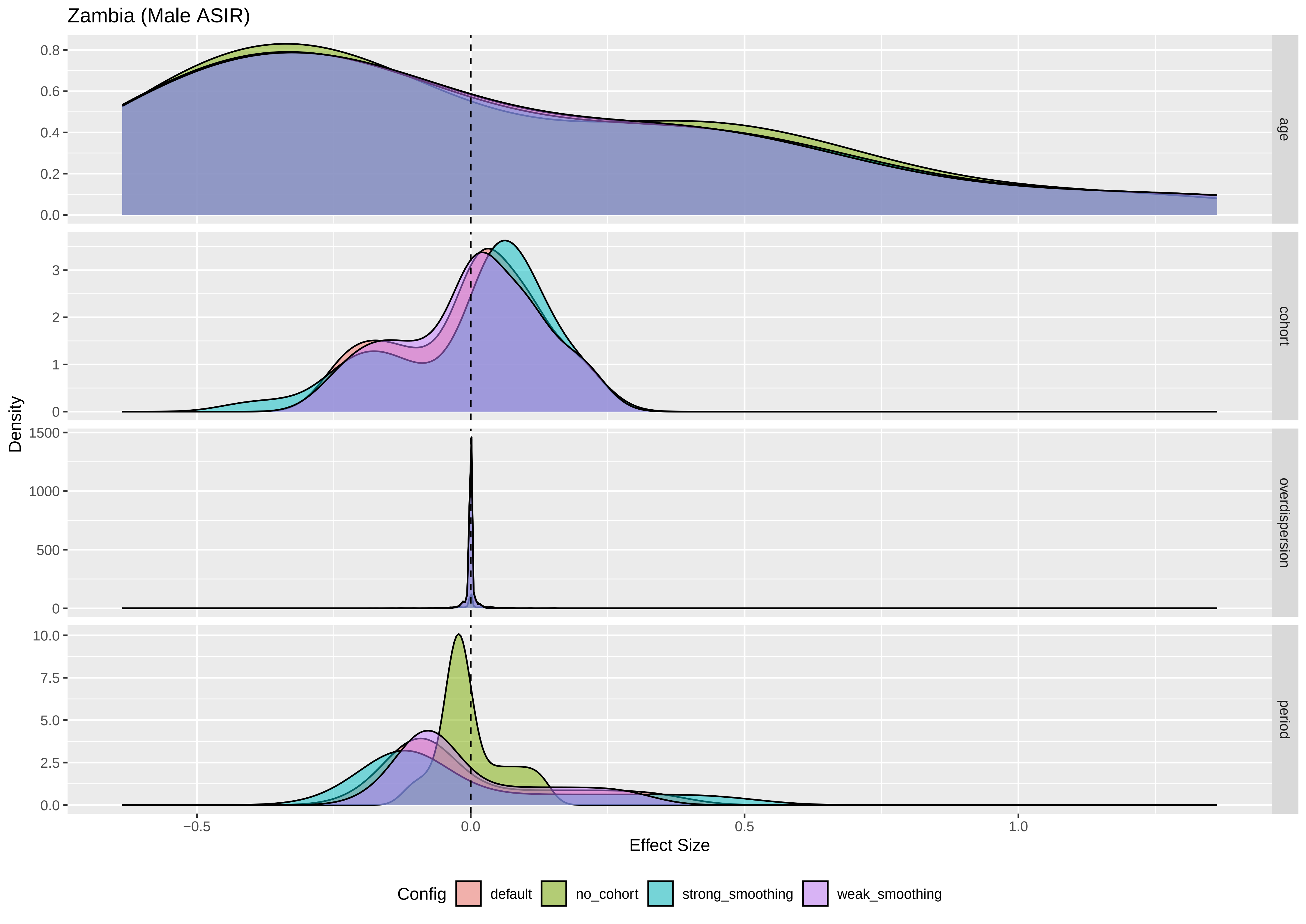

Zambia (Male ASIR)
Density
Effect Size
age
cohort
overdispersion
period
0.0
0.2
0.4
0.6
0.8
0
1
2
3
500
1000
1500
2.5
5.0
7.5
10.0
−0.5
0.0
0.5
1.0
Config
default
no_cohort
strong_smoothing
weak_smoothing

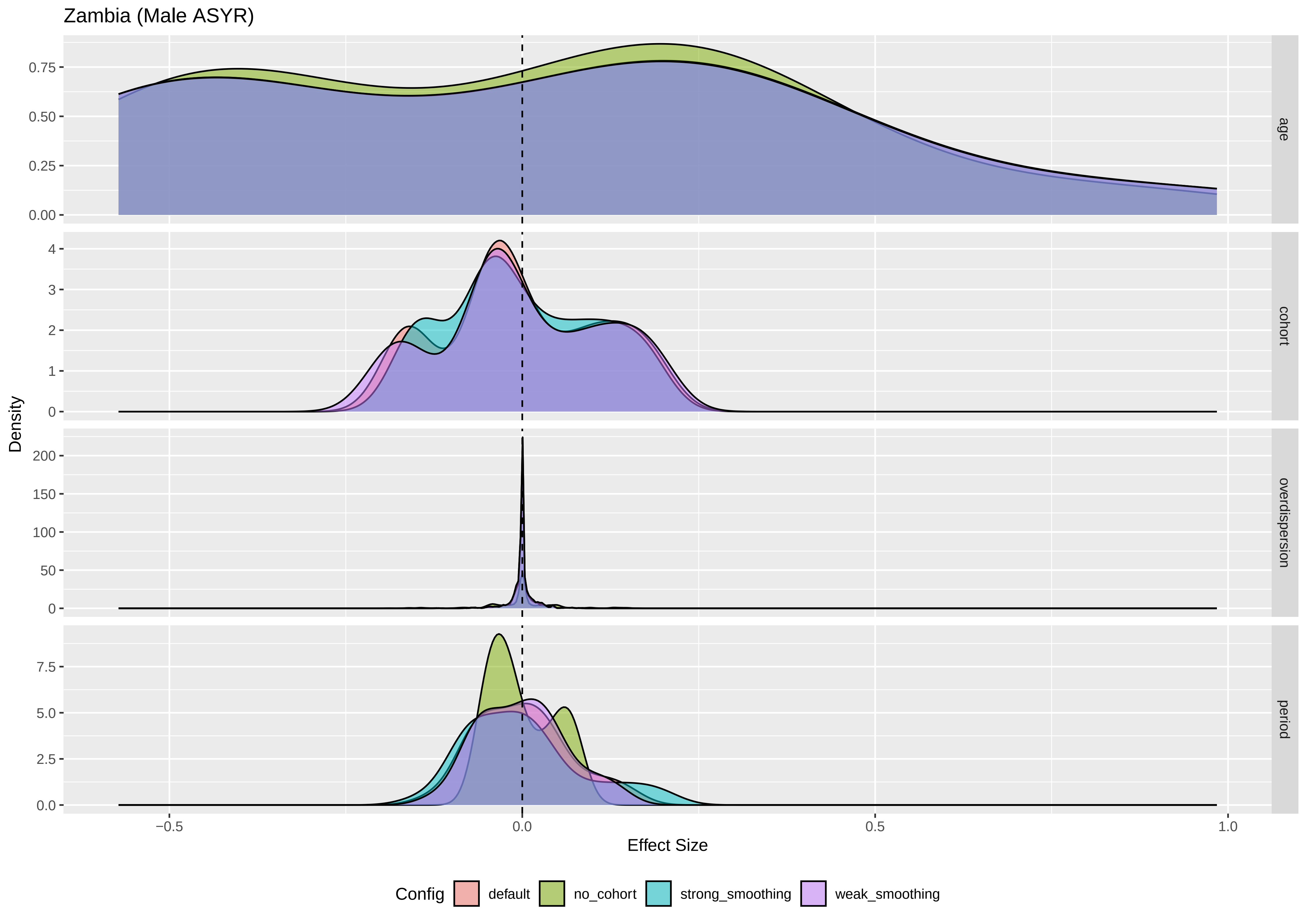
Zambia (Male ASYR)
age
cohort
overdispersion
period
Density
0.00
0.25
0.50
0.75
0
1
2
3
4
0
50
100
150
200
0.0
2.5
5.0
7.5
−0.5
0.0
0.5
1.0
Effect Size
Config
default
no_cohort
strong_smoothing
weak_smoothing

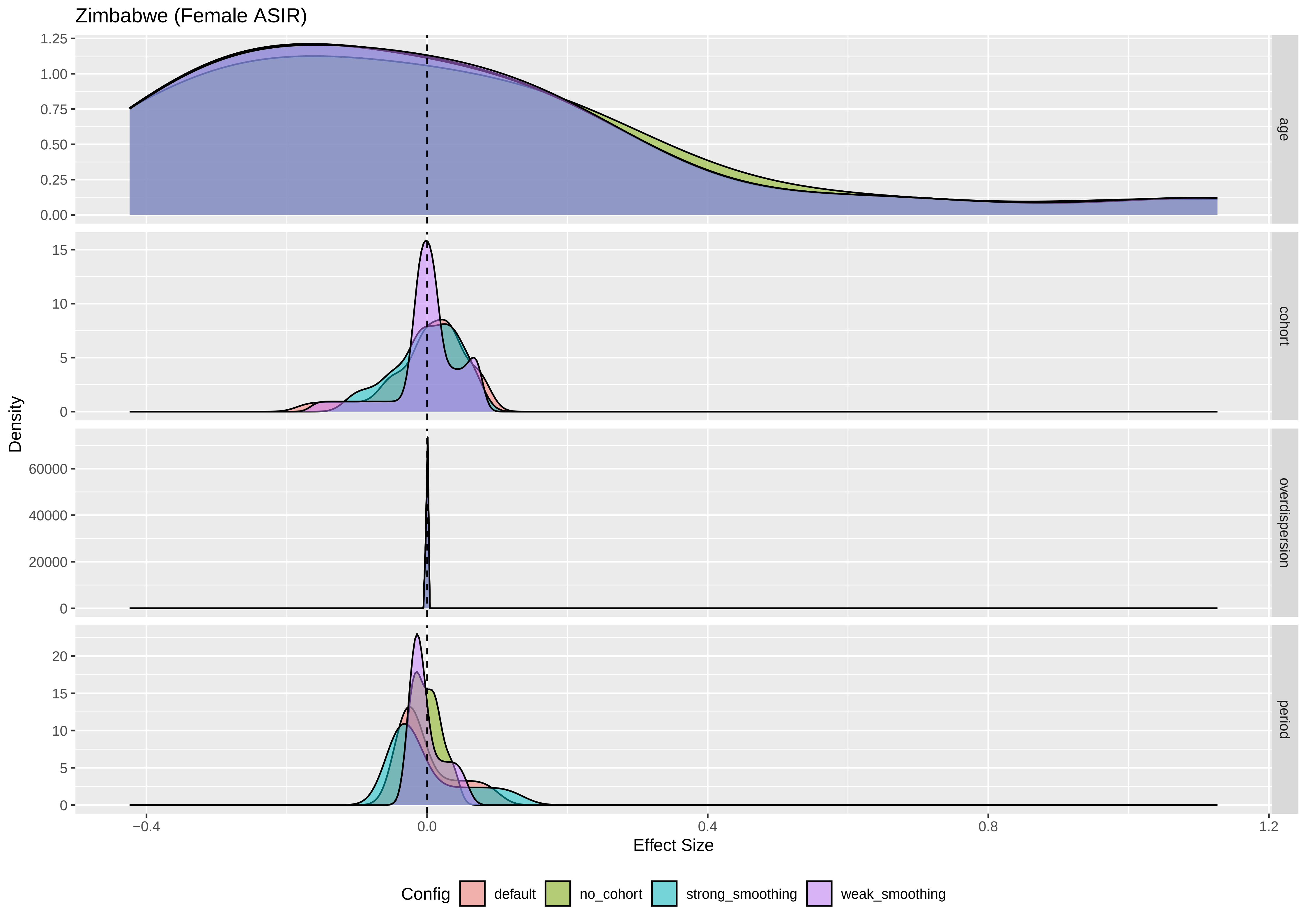

Zimbabwe (Female ASIR)
Density
Effect Size
age
cohort
overdispersion
period
1.25
1.00
0.75
0.50
0.25
0.00
15
10
5
0
60000
40000
20000
0
20
15
10
5
0
−0.4
0.0
0.4
0.8
1.2
Config
default
no_cohort
strong_smoothing
weak_smoothing
