# Supplementary material for: Global, regional, and national epilepsy of unknown cause incidence and mortality, 1990–2036: cross-national health inequalities and predictive analytics
Source: Front Neurol. 2025 Jun 30;16:1526984. doi: 10.3389/fneur.2025.1526984 (PMC12256229; doi:10.3389/fneur.2025.1526984)
Supplement: Supplementary file 4 [file Data_Sheet_4.pdf]

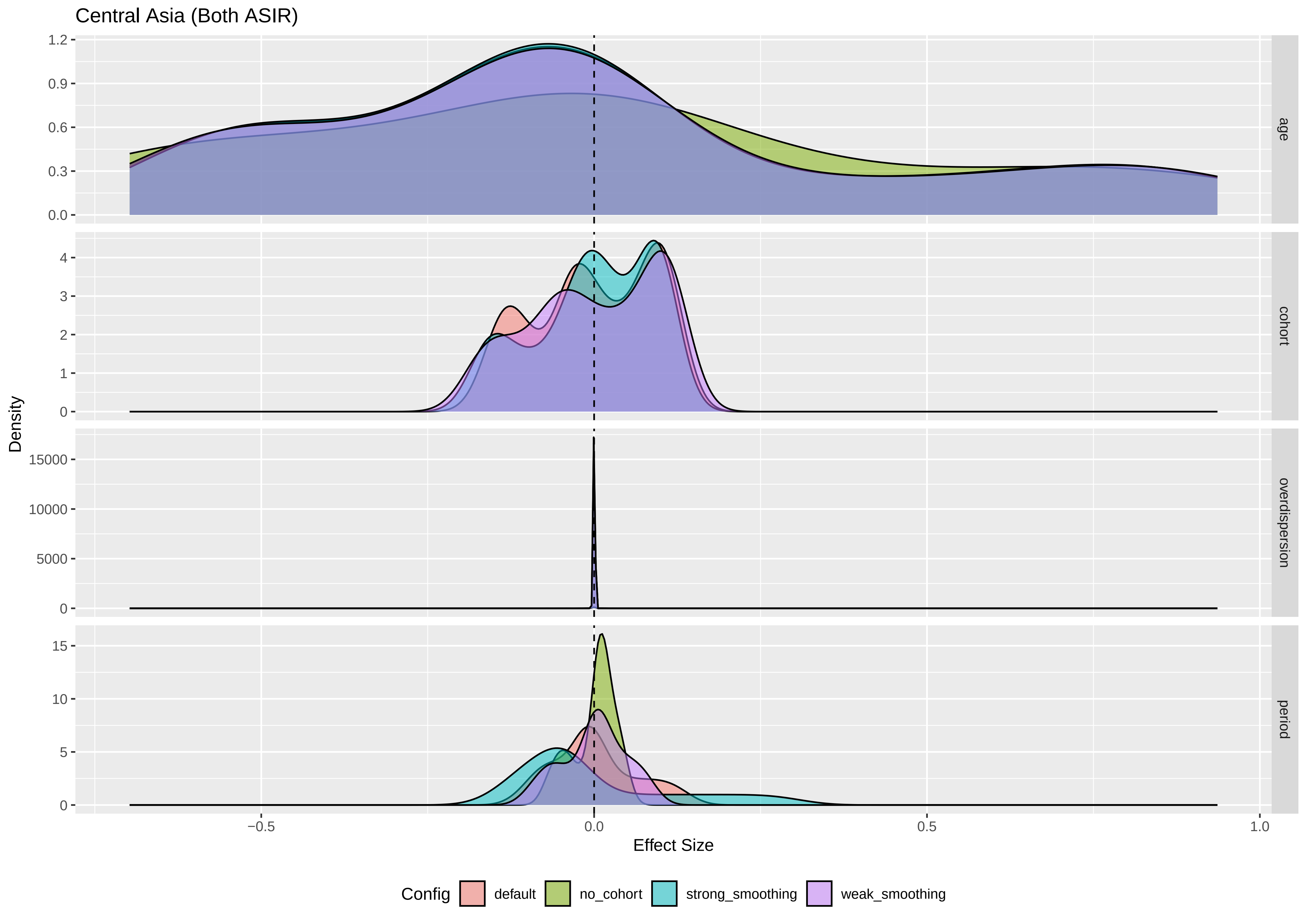
Central Asia (Both ASIR)
Density
Effect Size
age
cohort
overdispersion
period
1.2
0.9
0.6
0.3
0.0
4
3
2
1
0
15000
10000
5000
0
15
10
5
0
−0.5
0.0
0.5
1.0
Config
default
no_cohort
strong_smoothing
weak_smoothing

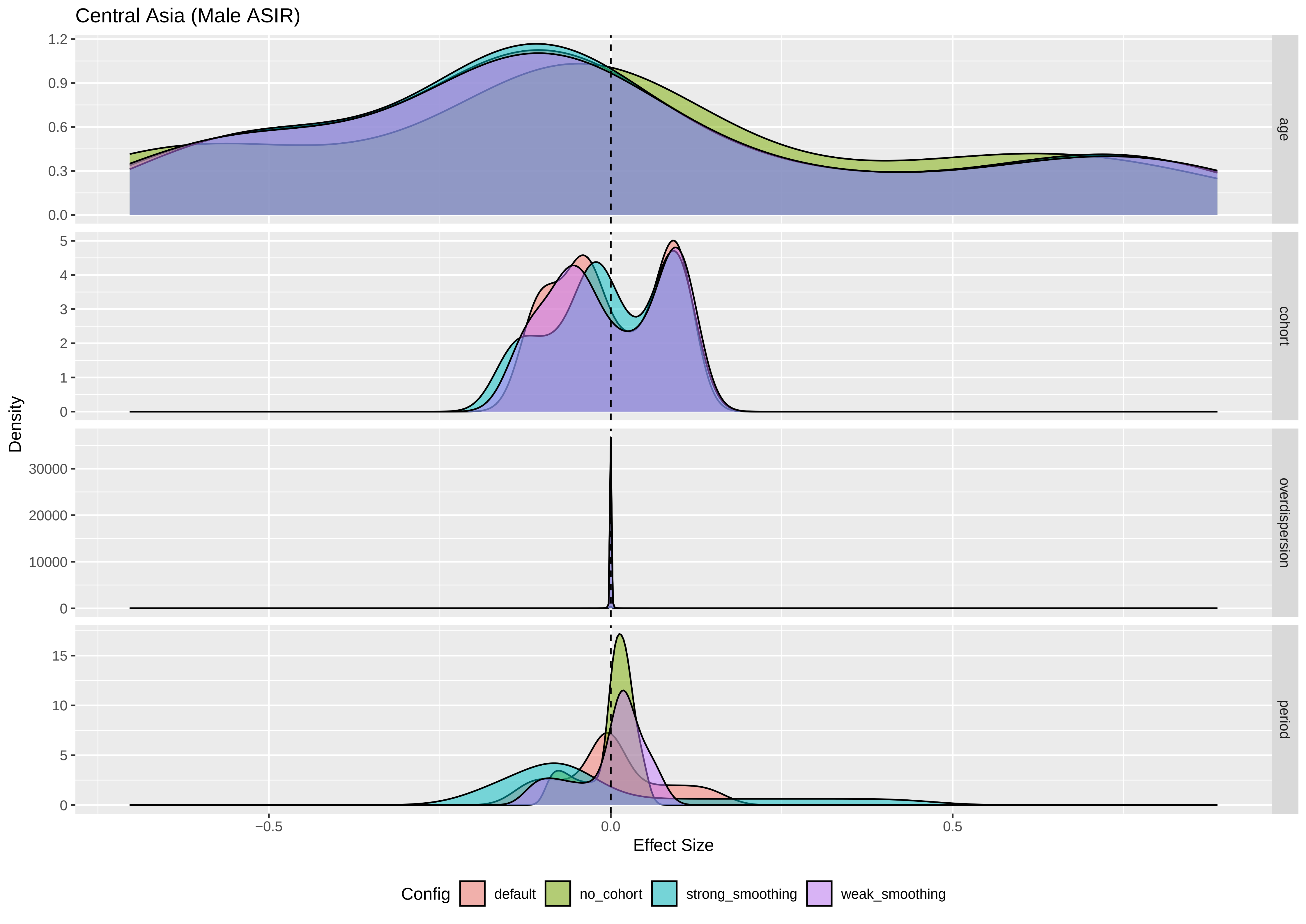

Central Asia (Male ASIR)
Density
Effect Size
age
cohort
overdispersion
period
1.2
0.9
0.6
0.3
0.0
5
4
3
2
1
0
30000
20000
10000
0
15
10
5
0
−0.5
0.0
0.5
Config
default
no_cohort
strong_smoothing
weak_smoothing

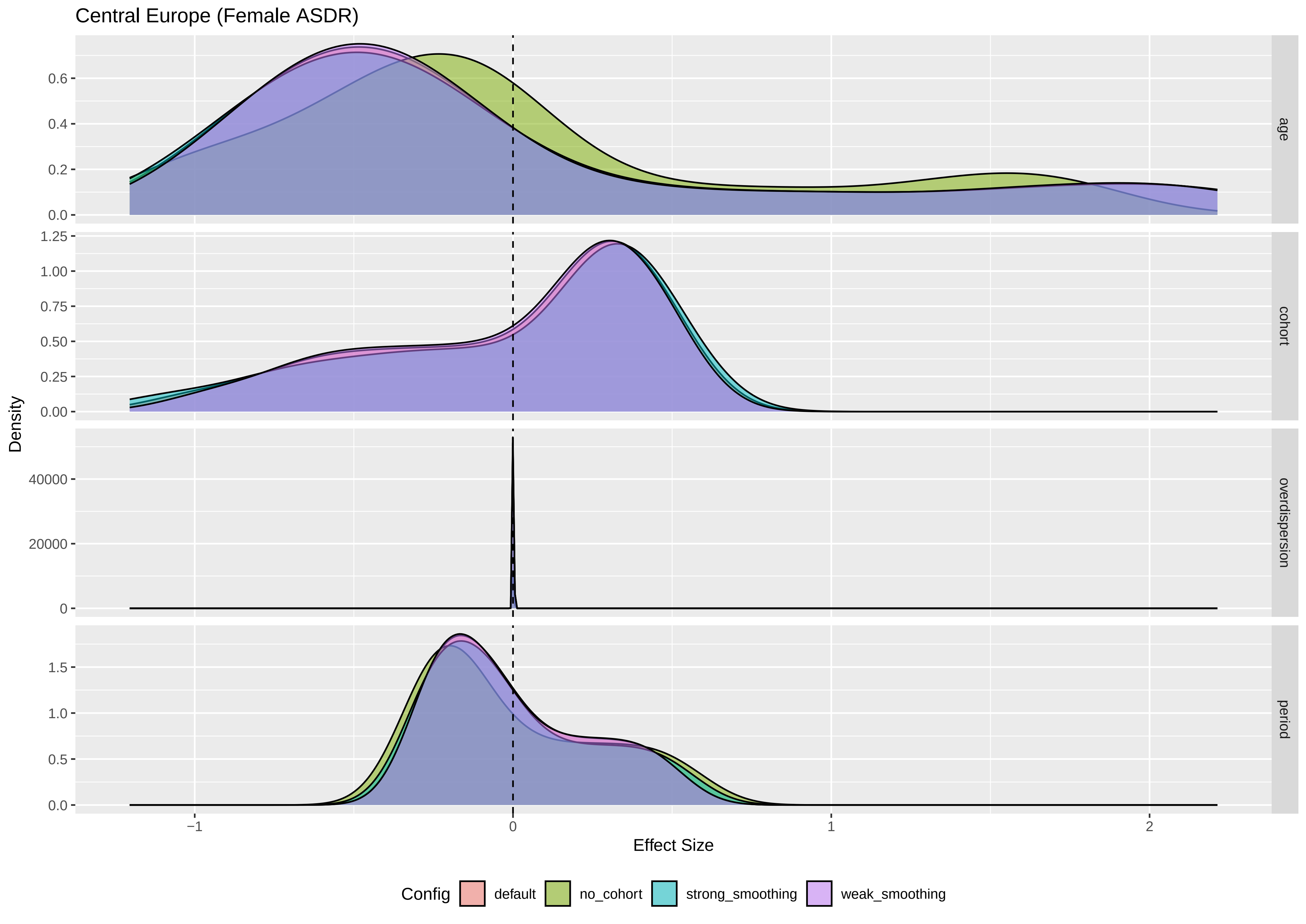

Central Europe (Female ASDR)
age
cohort
overdispersion
period
Density
Effect Size
0.0
0.2
0.4
0.6
0.00
0.25
0.50
0.75
1.00
1.25
0
20000
40000
0.0
0.5
1.0
1.5
−1
0
1
2
Config
default
no_cohort
strong_smoothing
weak_smoothing

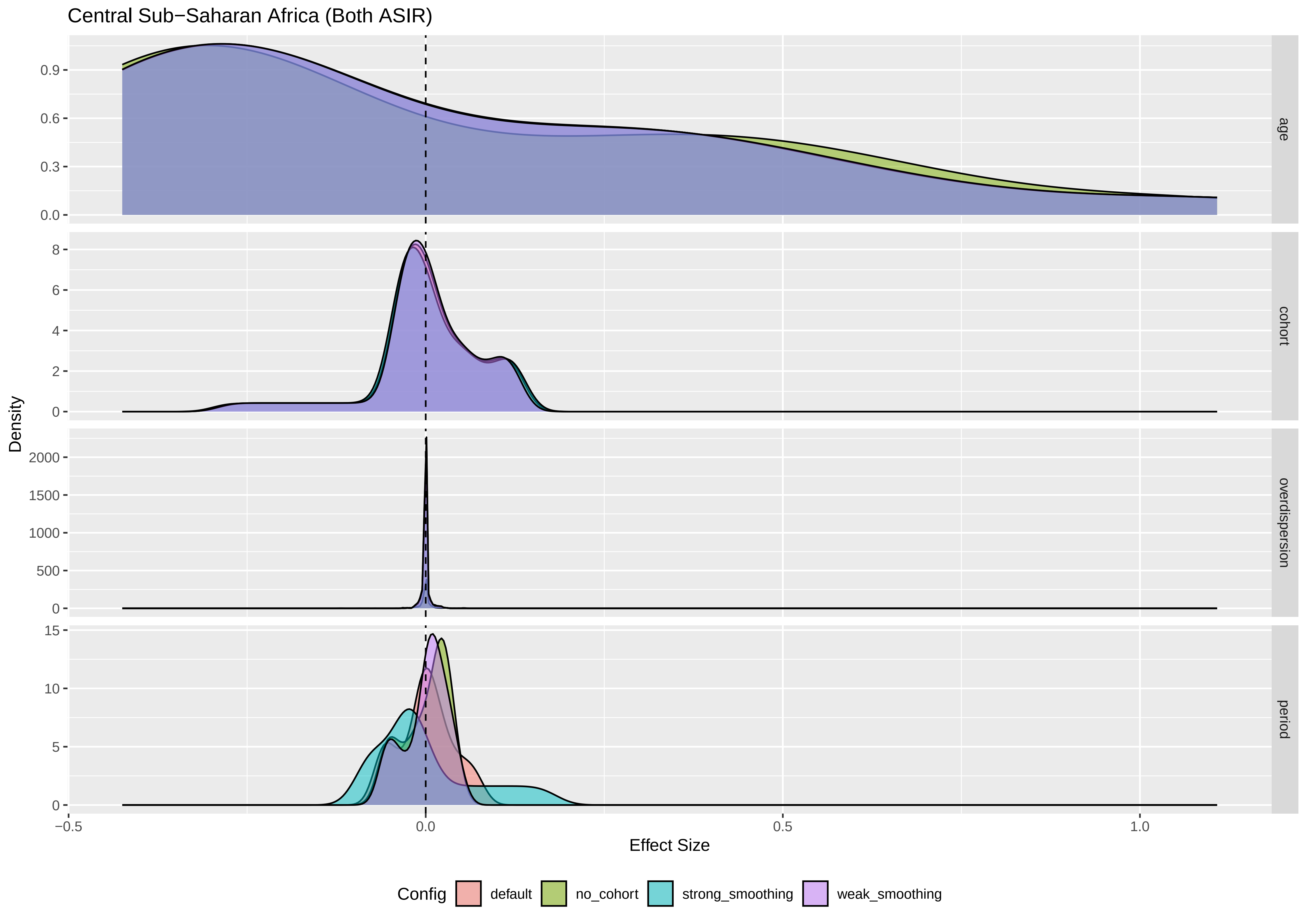

Central Sub−Saharan Africa (Both ASIR)
age
cohort
overdispersion
period
Density
0.0
0.3
0.6
0.9
0
2
4
6
8
0
500
1000
1500
2000
0
5
10
15
−0.5
0.0
0.5
1.0
Effect Size
Config
default
no_cohort
strong_smoothing
weak_smoothing

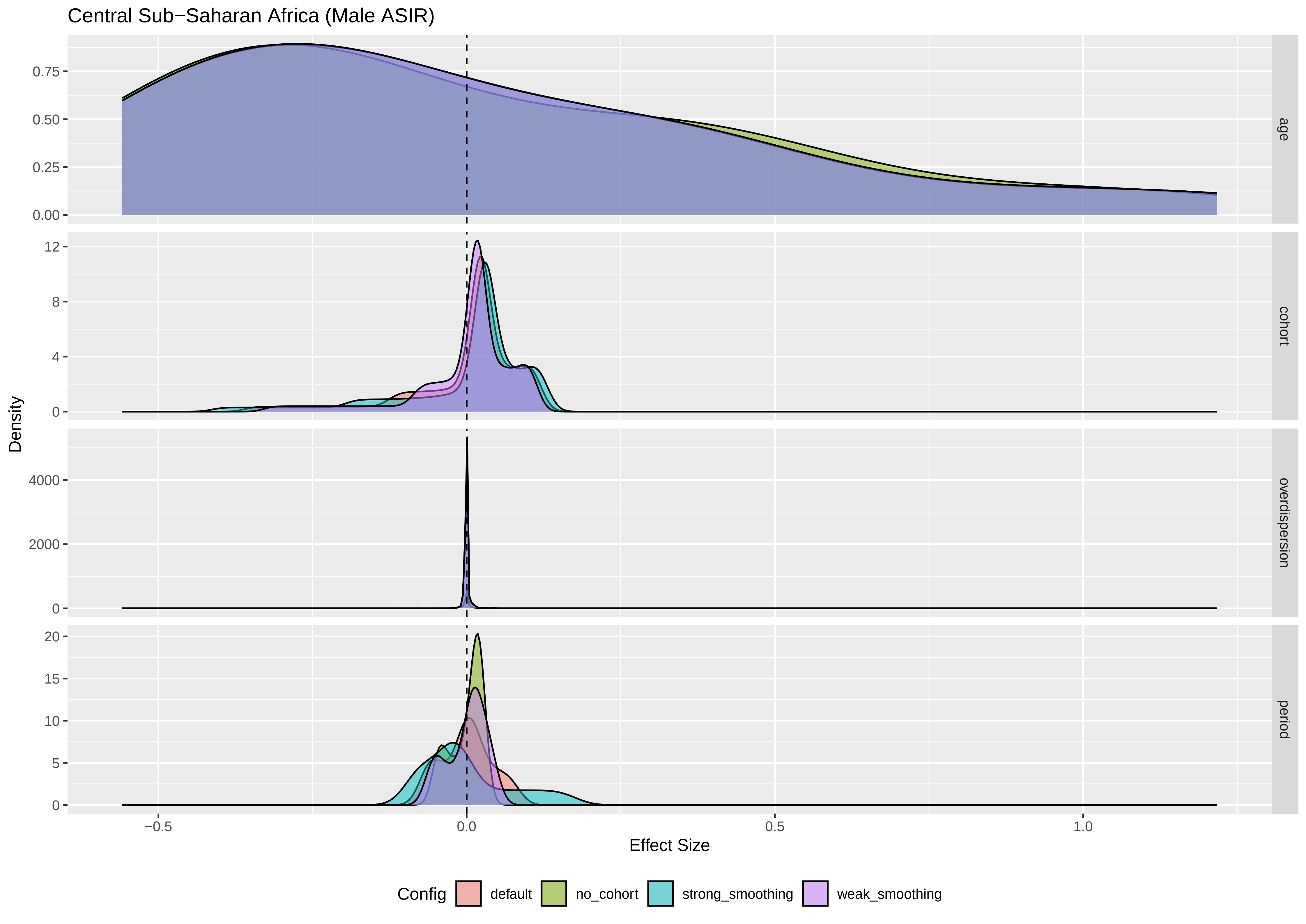

Central Sub−Saharan Africa (Male ASIR)
age
cohort
overdispersion
period
Density
Effect Size
0.00
0.25
0.50
0.75
0
4
8
12
0
2000
4000
0
5
10
15
20
−0.5
0.0
0.5
1.0
Config
default
no_cohort
strong_smoothing
weak_smoothing

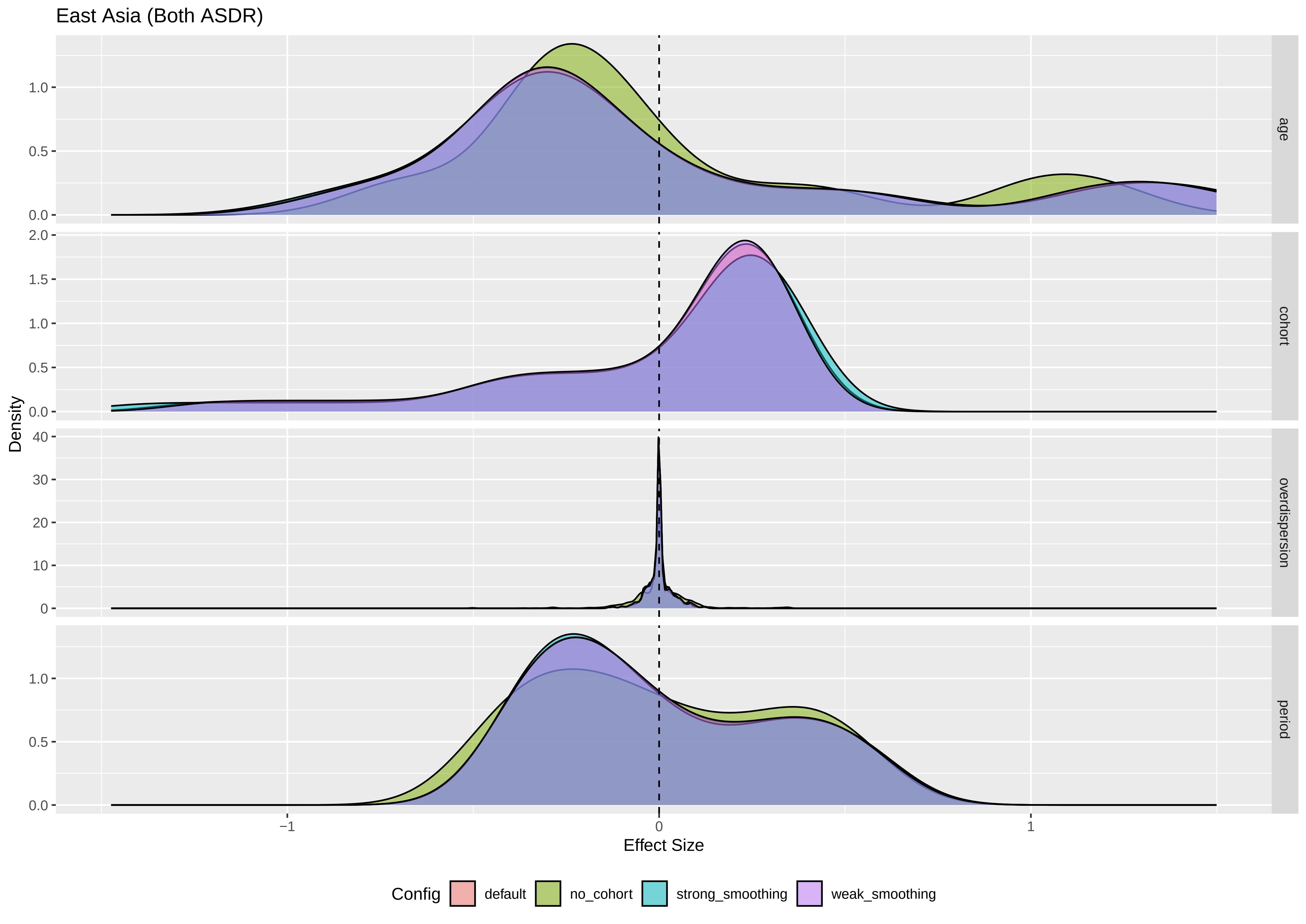

East Asia (Both ASDR)
age
cohort
overdispersion
period
Density
Effect Size
−1
0
1
0.0
0.5
1.0
1.5
2.0
0
10
20
30
40
Config
default
no_cohort
strong_smoothing
weak_smoothing

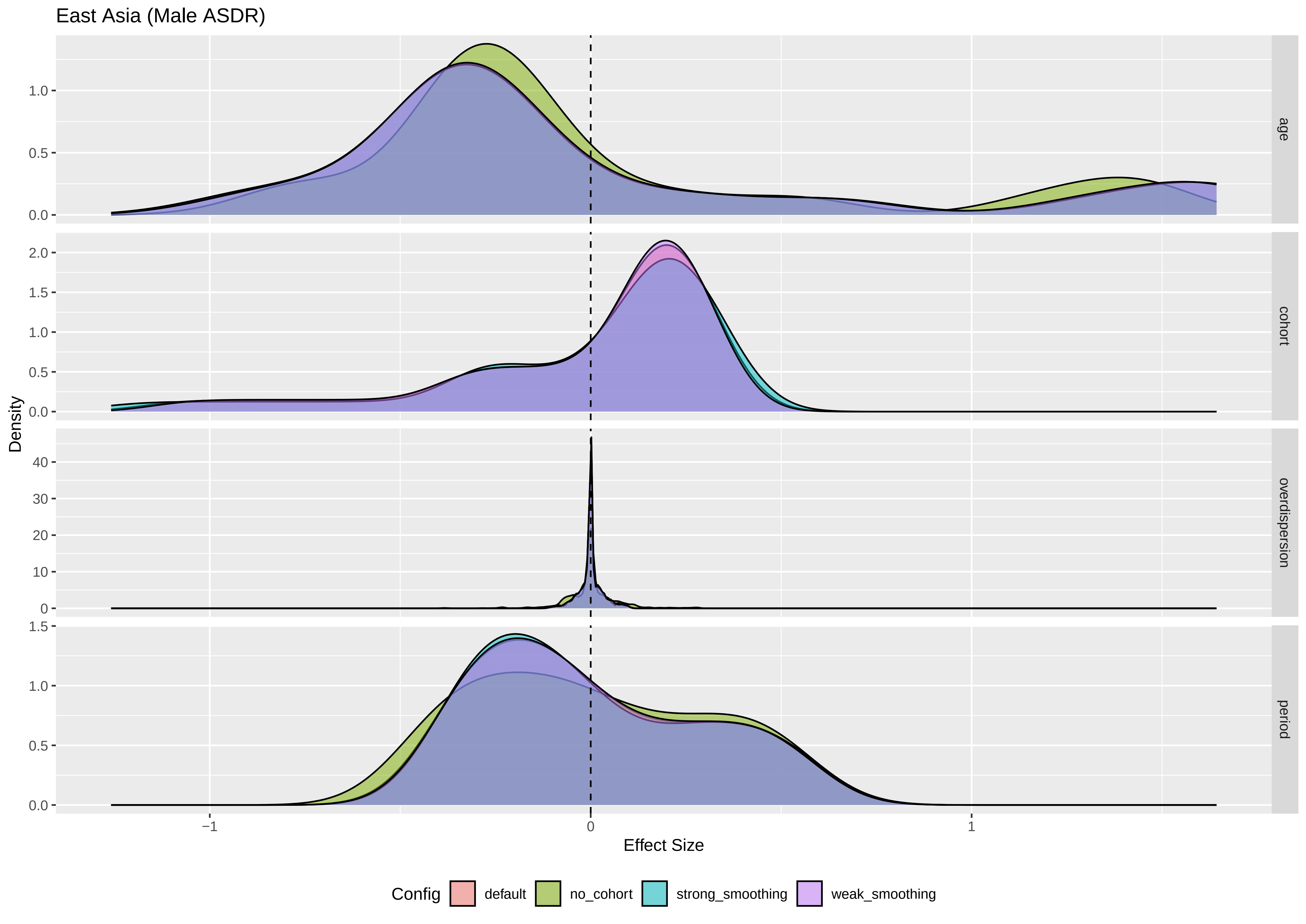
East Asia (Male ASDR)
Density
Effect Size
age
cohort
overdispersion
period
Config
default
no_cohort
strong_smoothing
weak_smoothing
−1
0
1
0.0
0.5
1.0
1.5
2.0
0
10
20
30
40

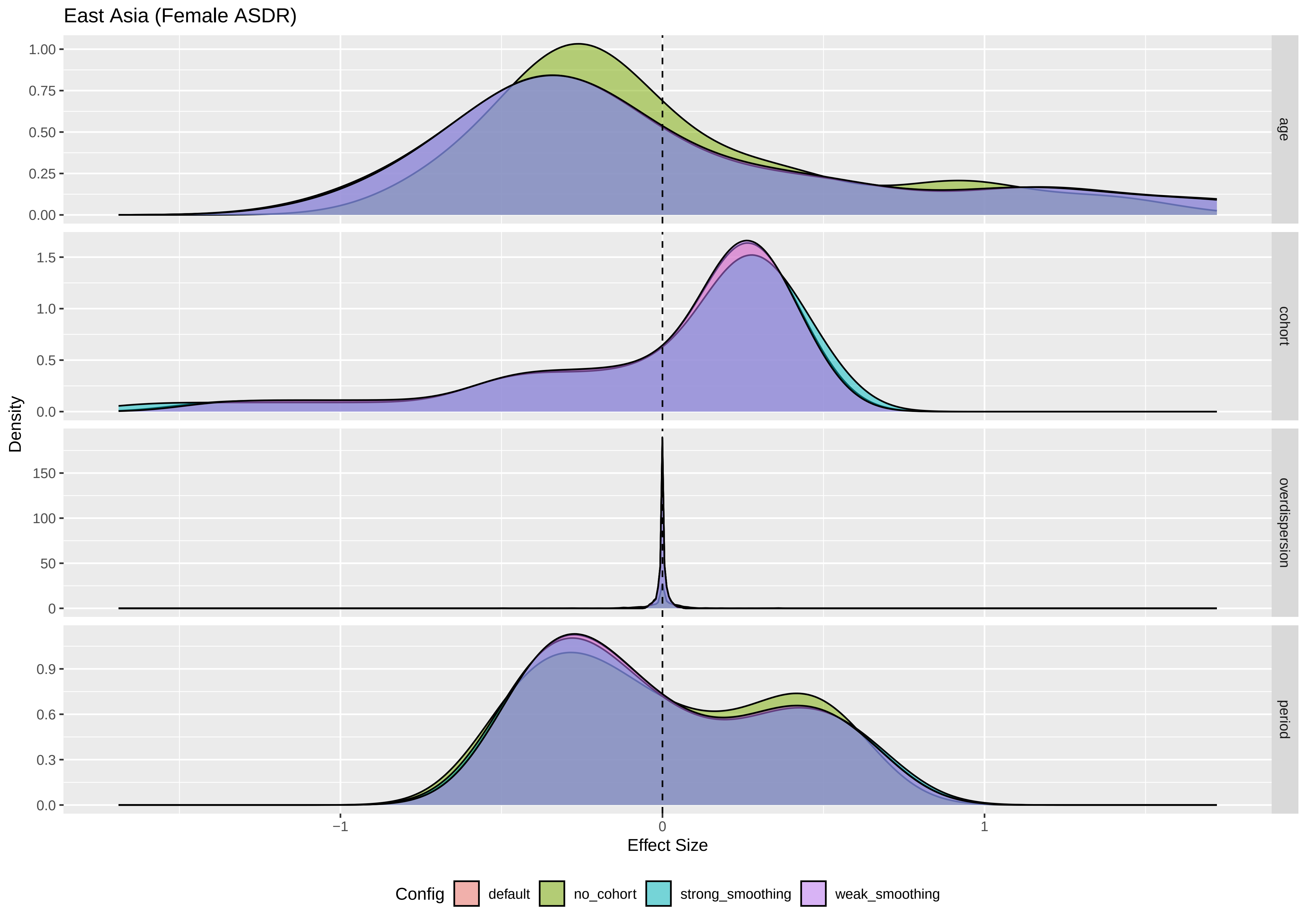

East Asia (Female ASDR)
age
cohort
overdispersion
period
Density
Effect Size
−1
0
1
1.00
0.75
0.50
0.25
0.00
1.5
1.0
0.5
0.0
150
100
50
0
0.9
0.6
0.3
0.0
Config
default
no_cohort
strong_smoothing
weak_smoothing

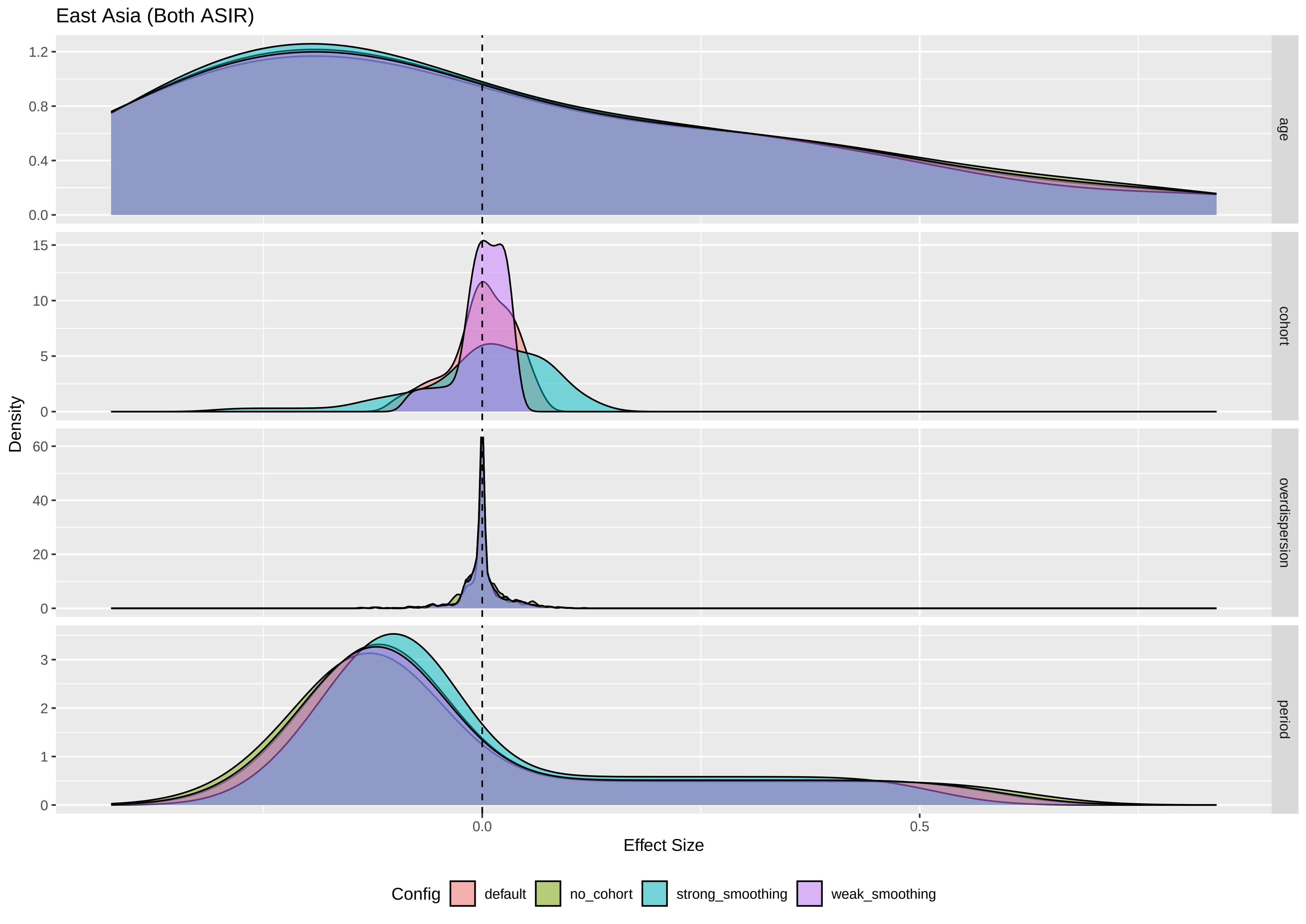

East Asia (Both ASIR)
1.2
0.8
0.4
0.0
age
15
10
5
0
cohort
60
40
20
0
overdispersion
3
2
1
0
period
Density
0.0
0.5
Effect Size
Config
default
no_cohort
strong_smoothing
weak_smoothing

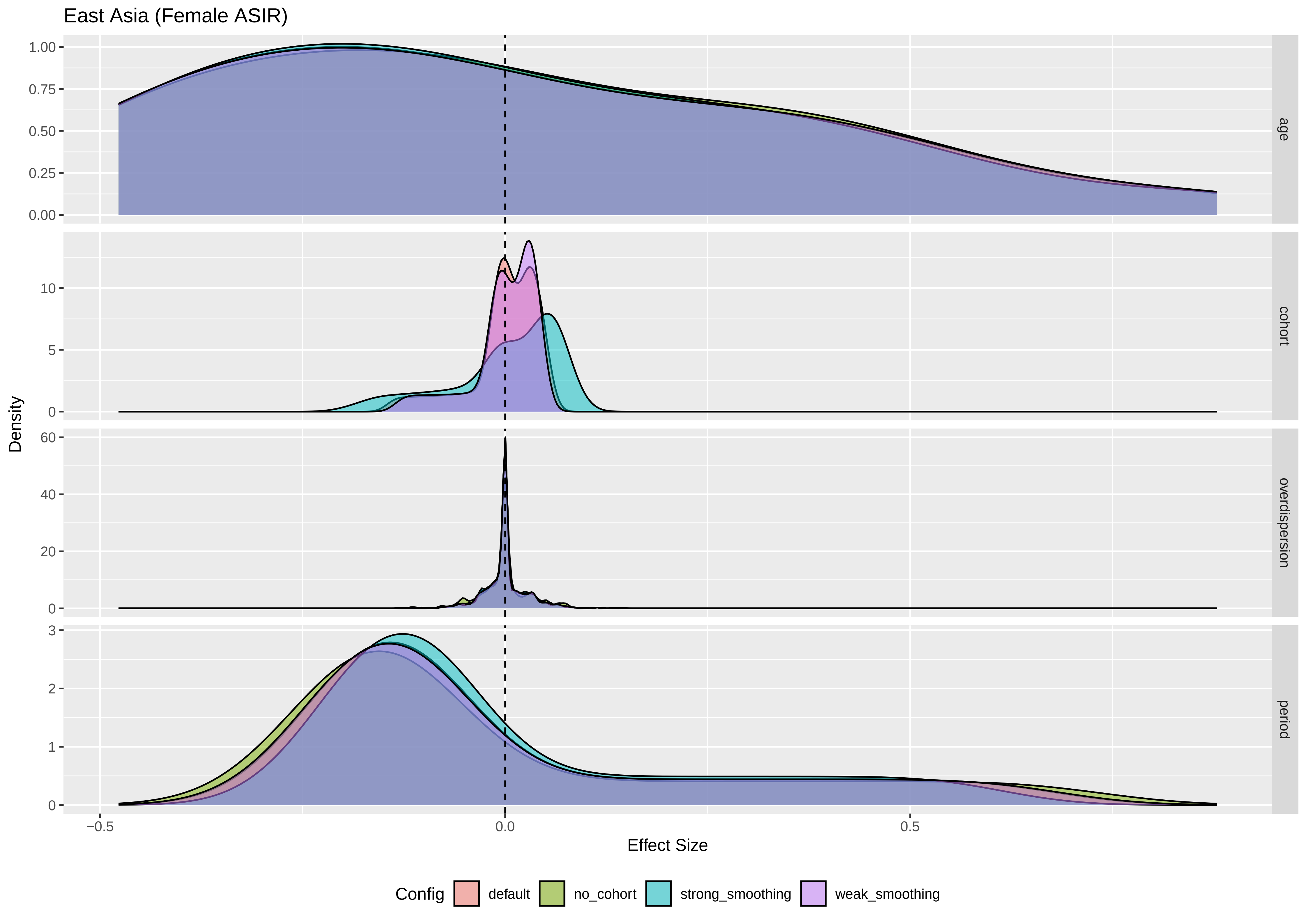
East Asia (Female ASIR)
Density
Effect Size
age
cohort
overdispersion
period
1.00
0.75
0.50
0.25
0.00
10
5
0
60
40
20
0
3
2
1
0
−0.5
0.0
0.5
Config
default
no_cohort
strong_smoothing
weak_smoothing

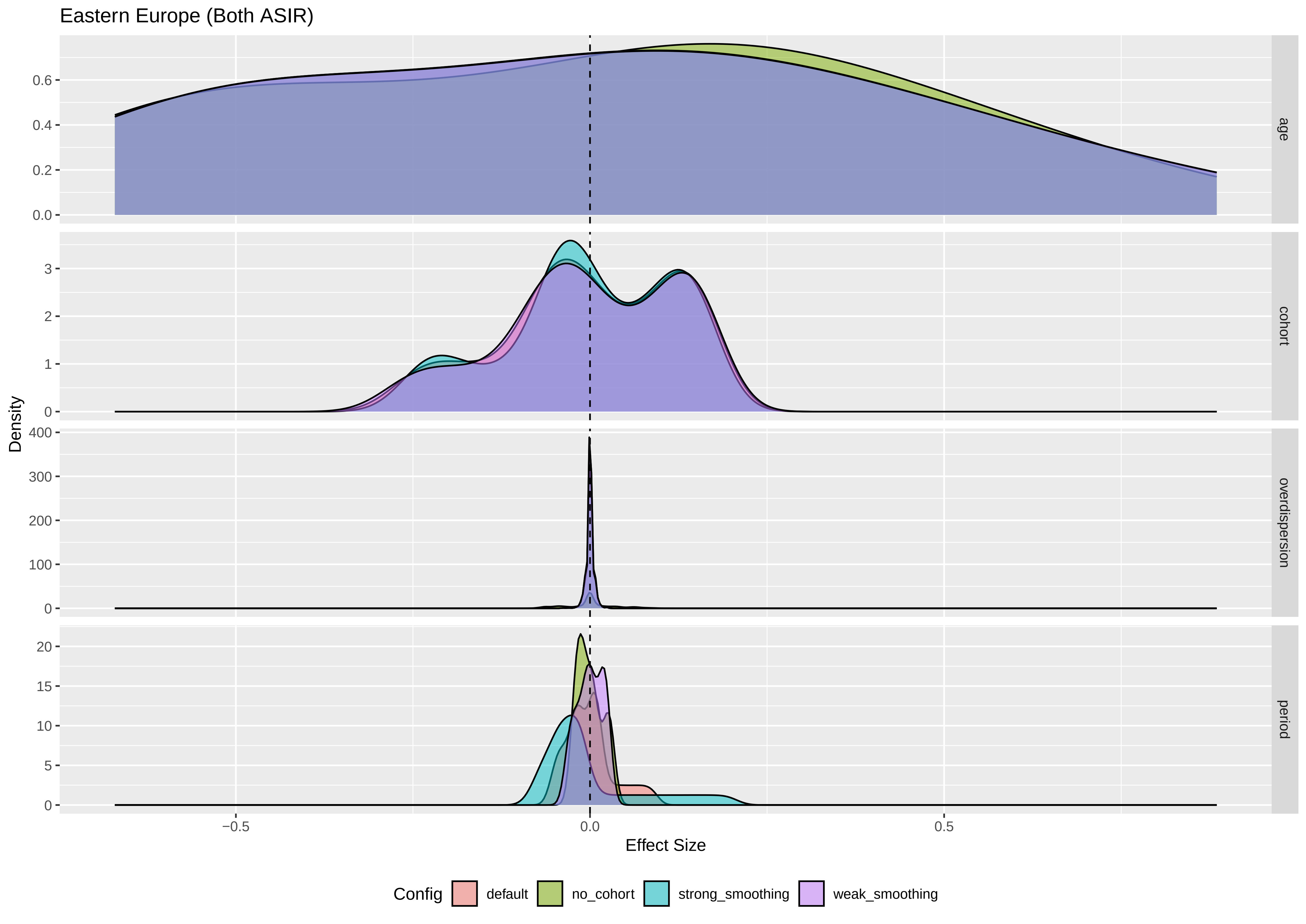

Eastern Europe (Both ASIR)
age
cohort
overdispersion
period
Density
Effect Size
−0.5
0.0
0.5
0.0
0.2
0.4
0.6
0
1
2
3
0
100
200
300
400
0
5
10
15
20
Config
default
no_cohort
strong_smoothing
weak_smoothing

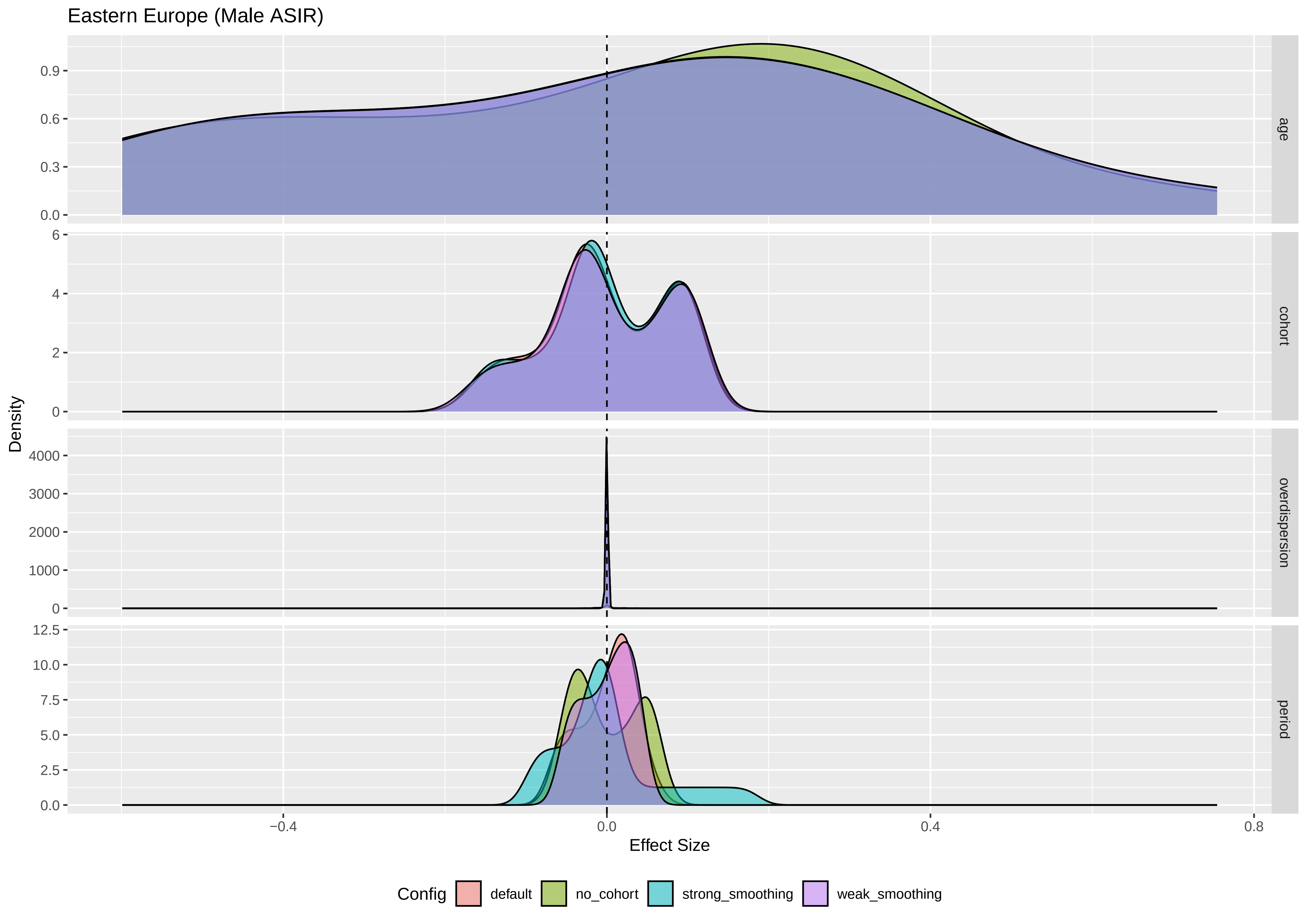

Eastern Europe (Male ASIR)
age
cohort
overdispersion
period
Density
Effect Size
-0.4
0.0
0.4
0.8
0.0
0.3
0.6
0.9
0
2
4
6
0
1000
2000
3000
4000
0.0
2.5
5.0
7.5
10.0
12.5
Config
default
no_cohort
strong_smoothing
weak_smoothing

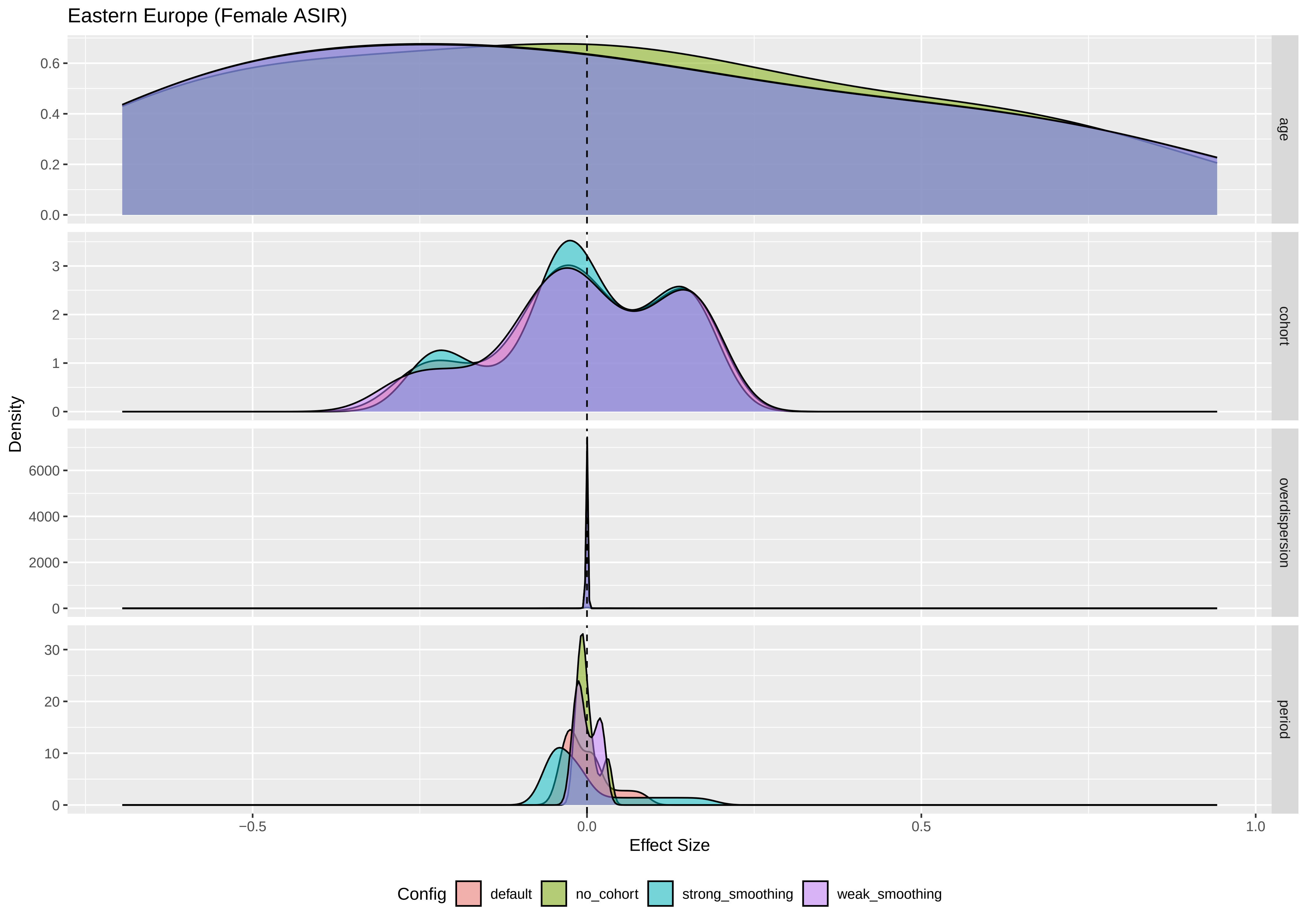

Eastern Europe (Female ASIR)
Density
Effect Size
age
cohort
overdispersion
period
0.0
0.2
0.4
0.6
0
1
2
3
0
2000
4000
6000
0
10
20
30
−0.5
0.0
0.5
1.0
Config
default
no_cohort
strong_smoothing
weak_smoothing

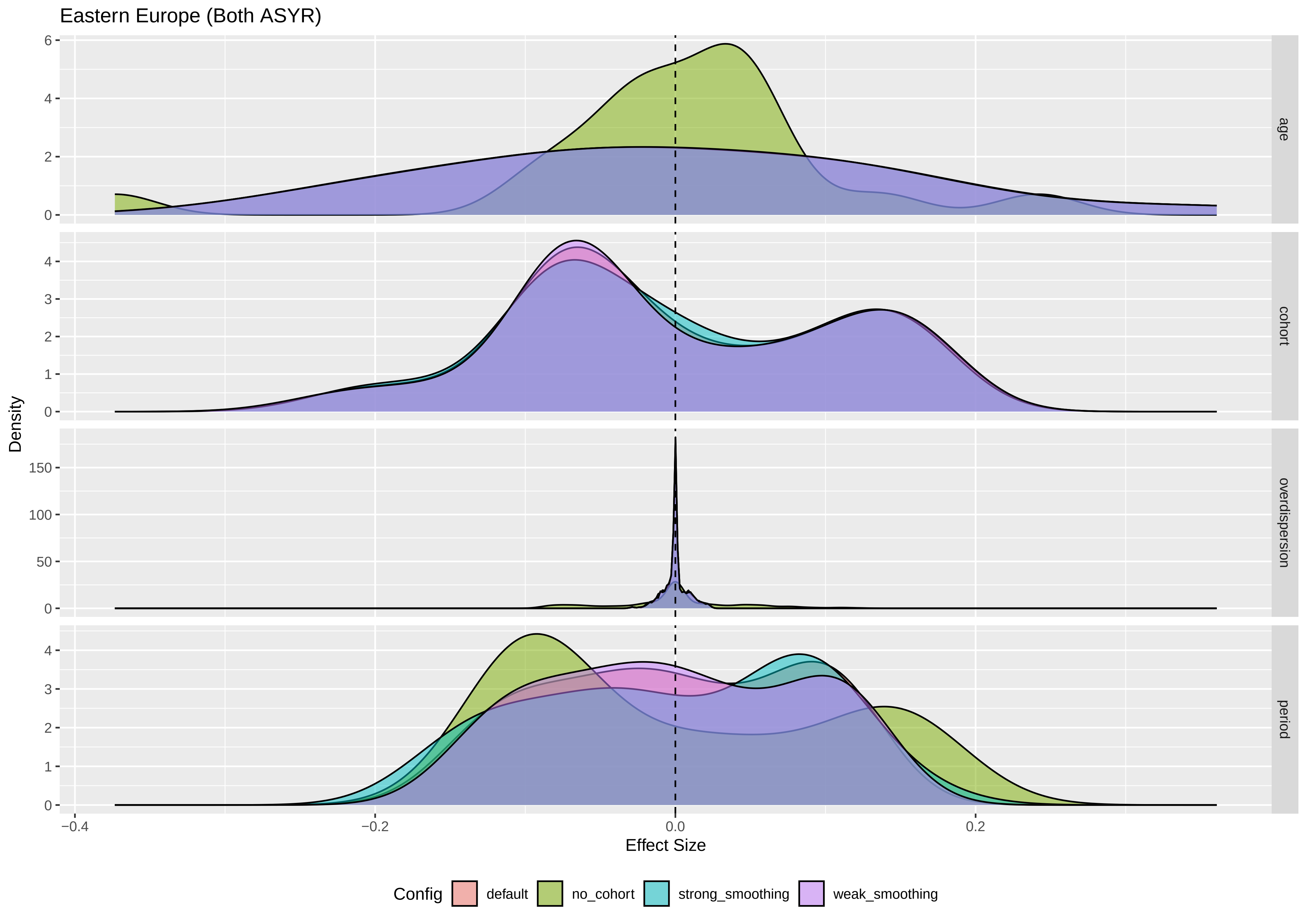
Eastern Europe (Both ASYR)
age
cohort
overdispersion
period
Density
Effect Size
0
2
4
6
1
3
50
100
150
−0.4
−0.2
0.0
0.2
Config
default
no_cohort
strong_smoothing
weak_smoothing

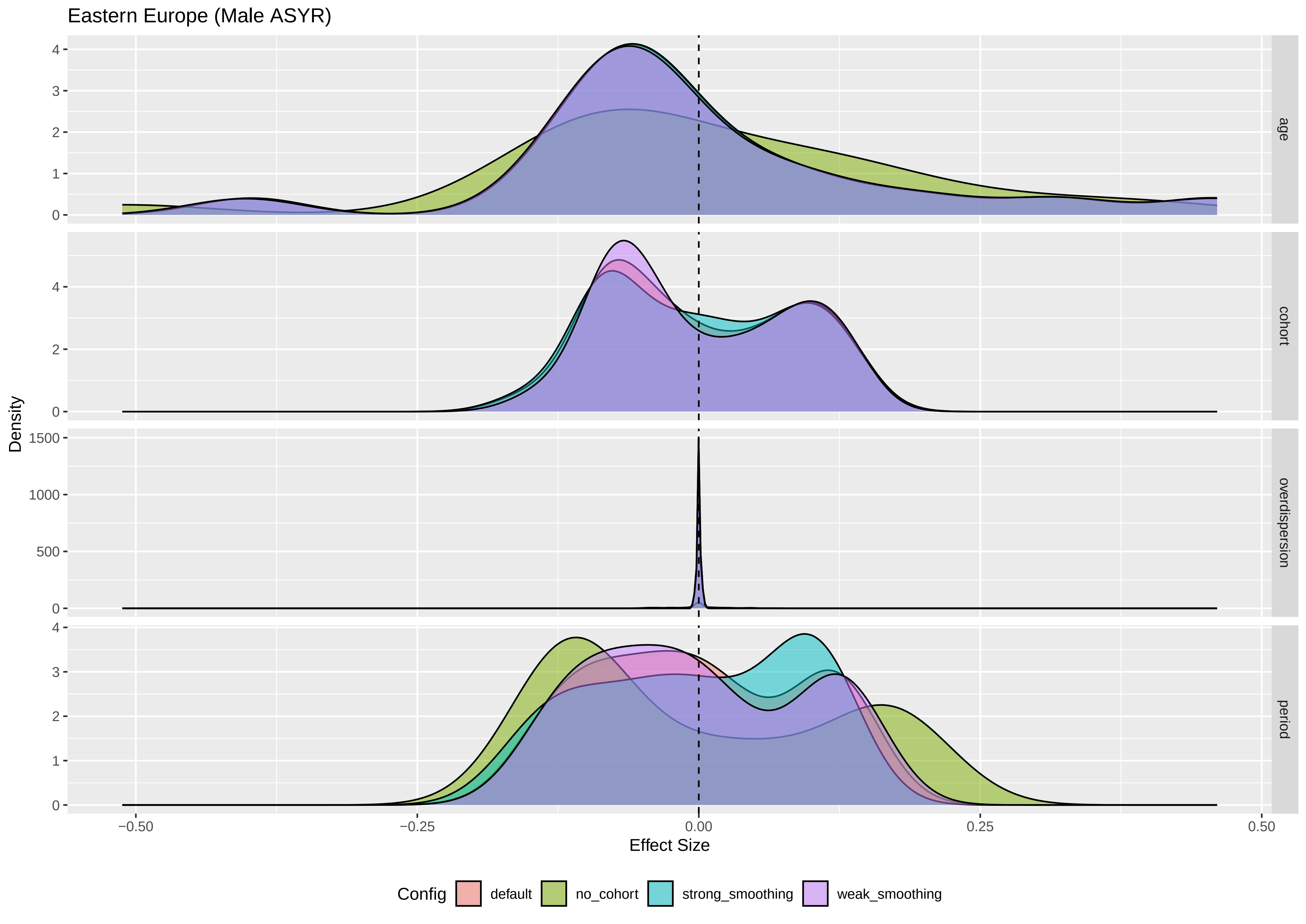

Eastern Europe (Male ASYR)
age
cohort
overdispersion
period
Density
Effect Size
-0.50
-0.25
0.00
0.25
0.50
0
1
2
3
4
500
1000
1500
Config
default
no_cohort
strong_smoothing
weak_smoothing

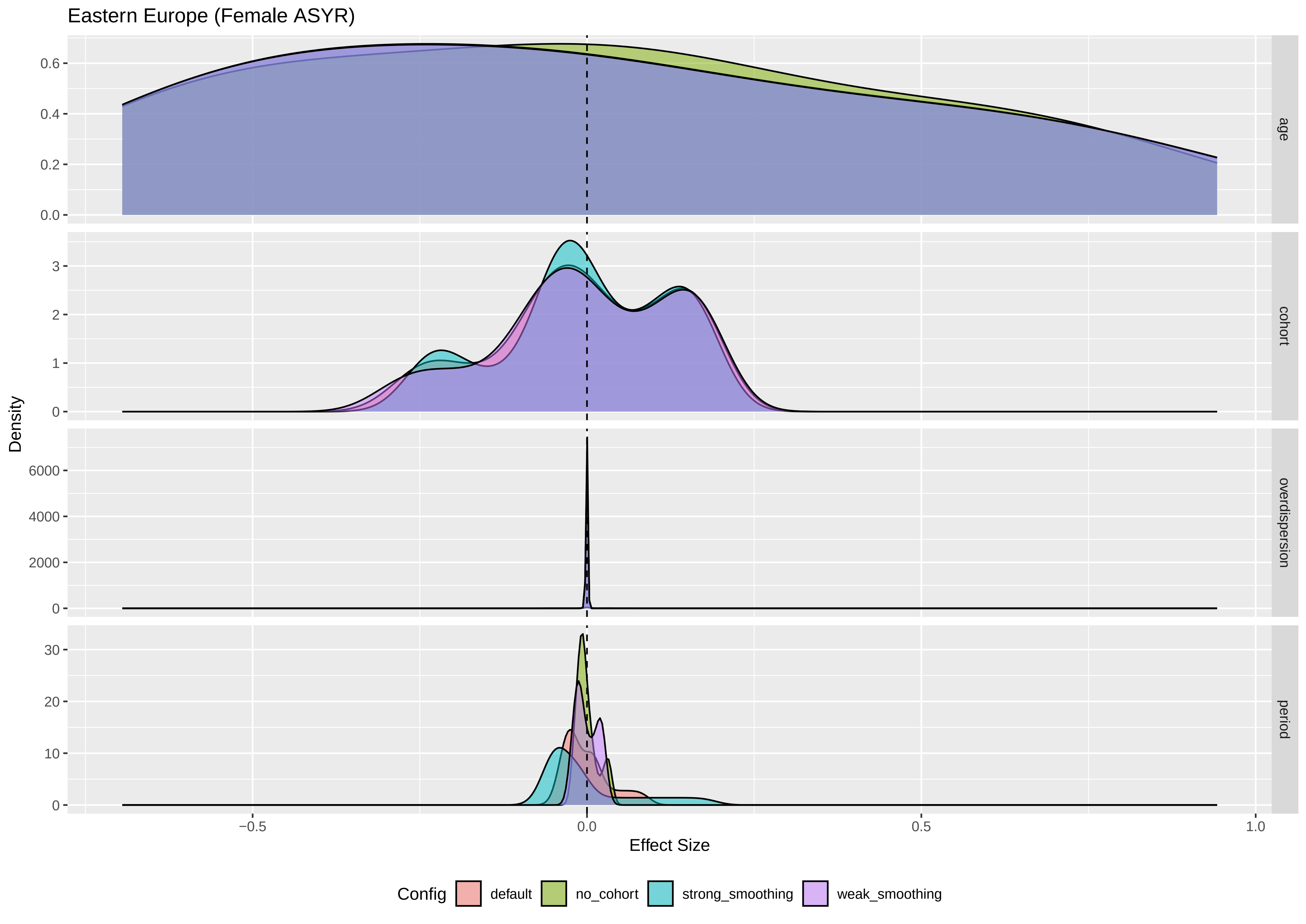
Eastern Europe (Female ASYR)
Density
Effect Size
age
cohort
overdispersion
period
0.0
0.2
0.4
0.6
0
1
2
3
0
2000
4000
6000
0
10
20
30
−0.5
0.0
0.5
1.0
Config
default
no_cohort
strong_smoothing
weak_smoothing

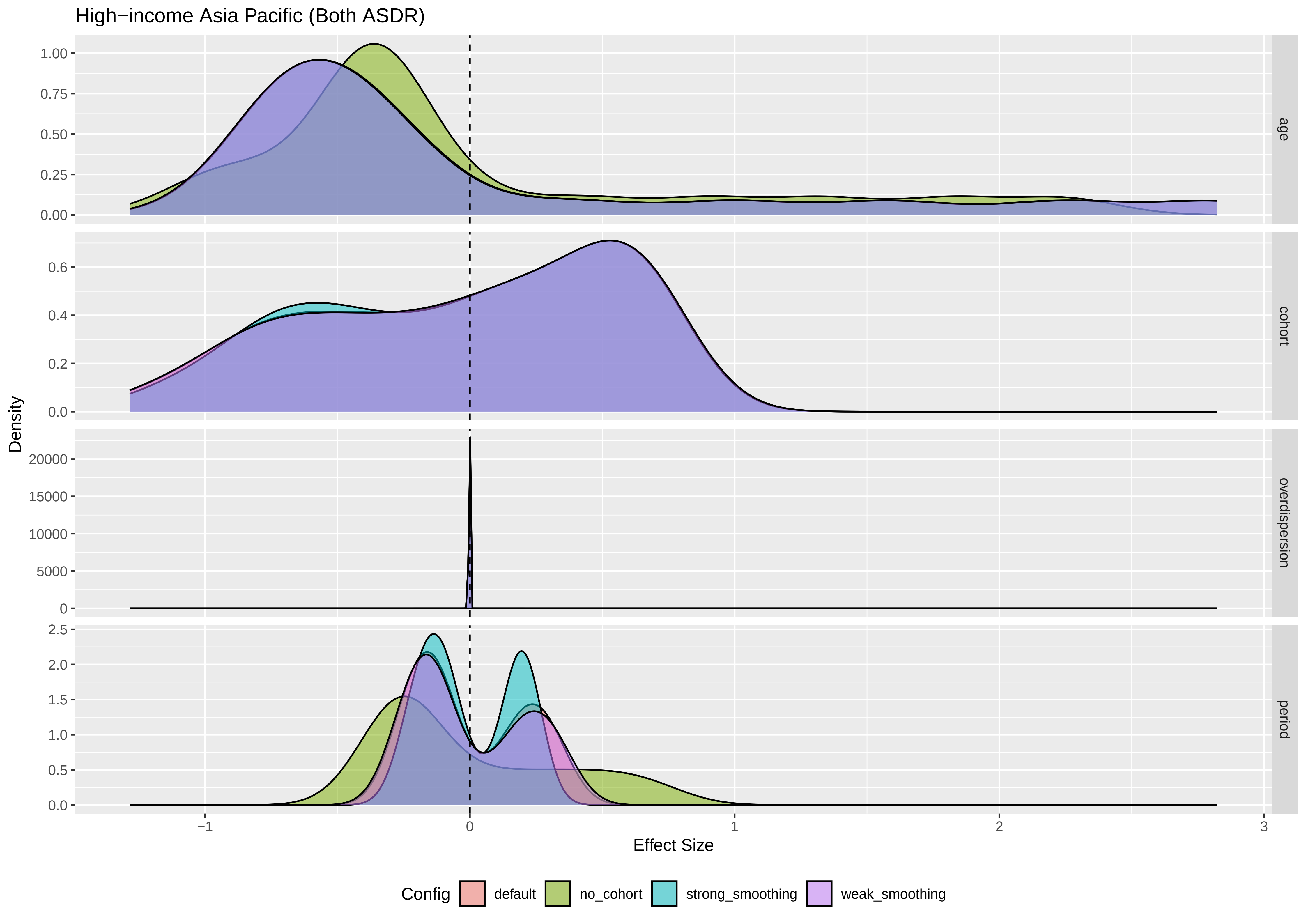

High−income Asia Pacific (Both ASDR)
Density
Effect Size
age
cohort
overdispersion
period
1.00
0.75
0.50
0.25
0.00
0.6
0.4
0.2
0.0
20000
15000
10000
5000
0
2.5
2.0
1.5
1.0
0.5
0.0
−1
0
1
2
3
Config
default
no_cohort
strong_smoothing
weak_smoothing

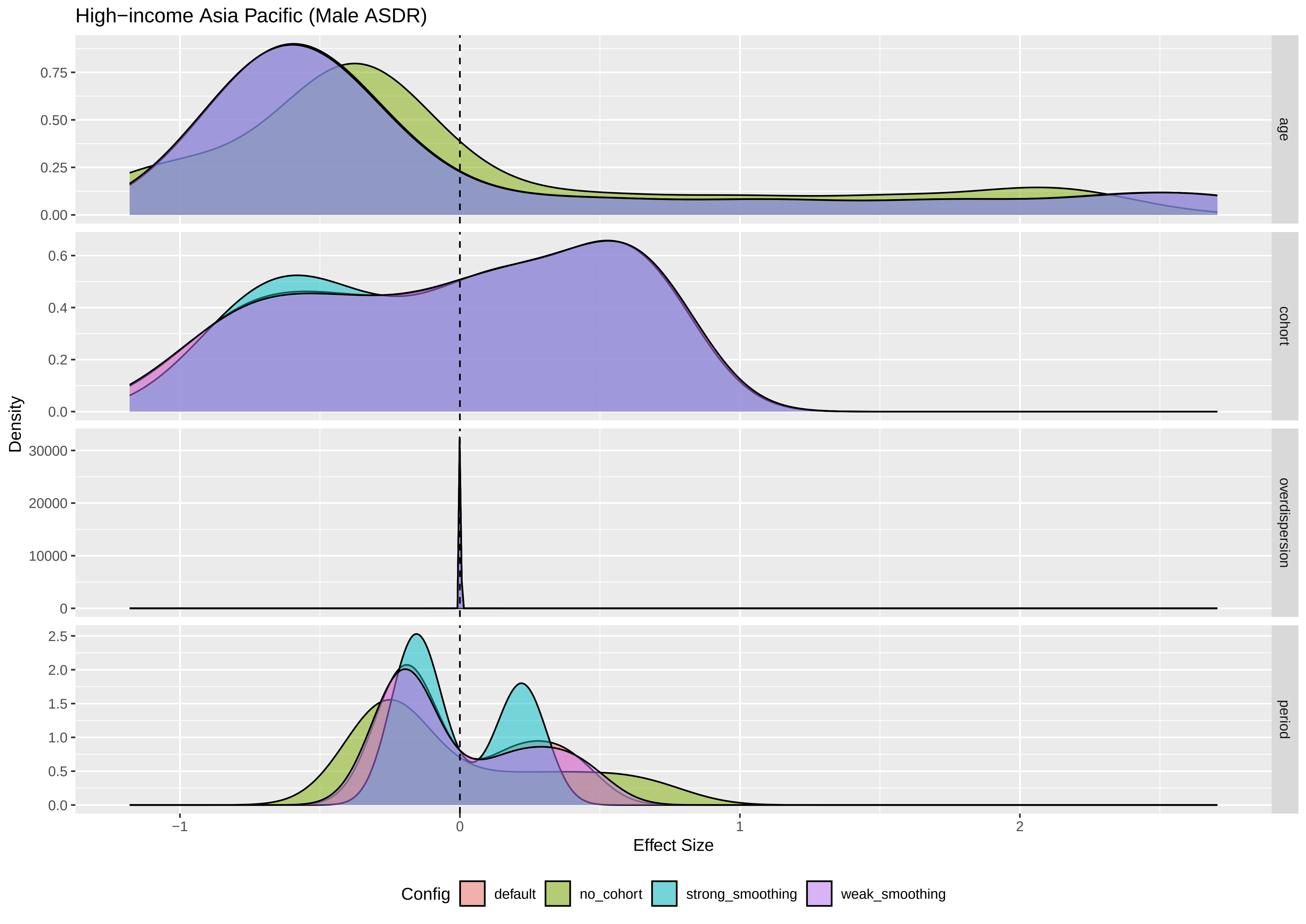
High−income Asia Pacific (Male ASDR)
age
cohort
overdispersion
period
Density
Effect Size
0.00
0.25
0.50
0.75
0.0
0.2
0.4
0.6
0
10000
20000
30000
0.0
0.5
1.0
1.5
2.0
2.5
−1
0
1
2
Config
default
no_cohort
strong_smoothing
weak_smoothing

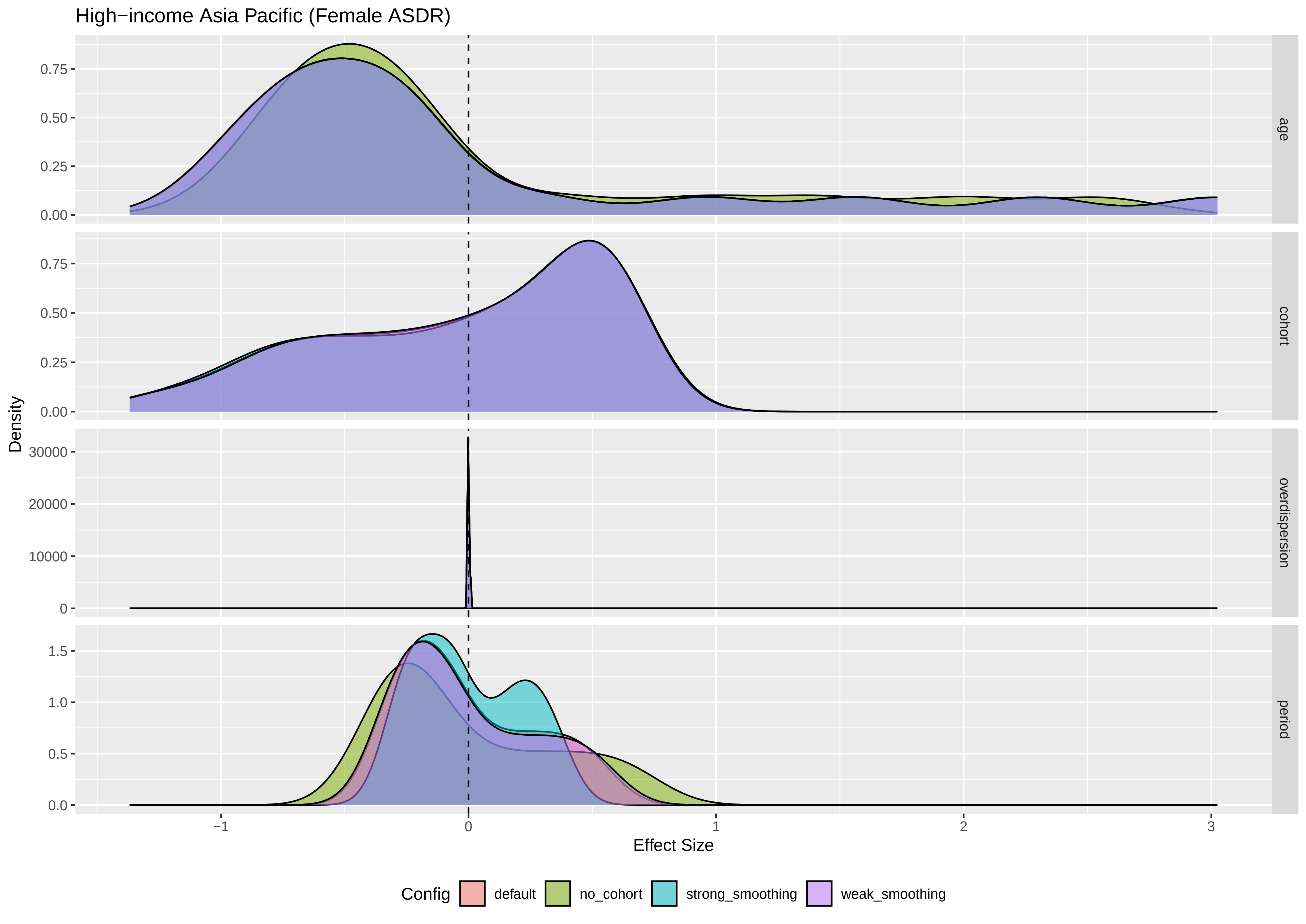

High−income Asia Pacific (Female ASDR)
age
cohort
overdispersion
period
Density
Effect Size
0.00
0.25
0.50
0.75
0
10000
20000
30000
0.0
0.5
1.0
1.5
−1
0
1
2
3
Config
default
no_cohort
strong_smoothing
weak_smoothing

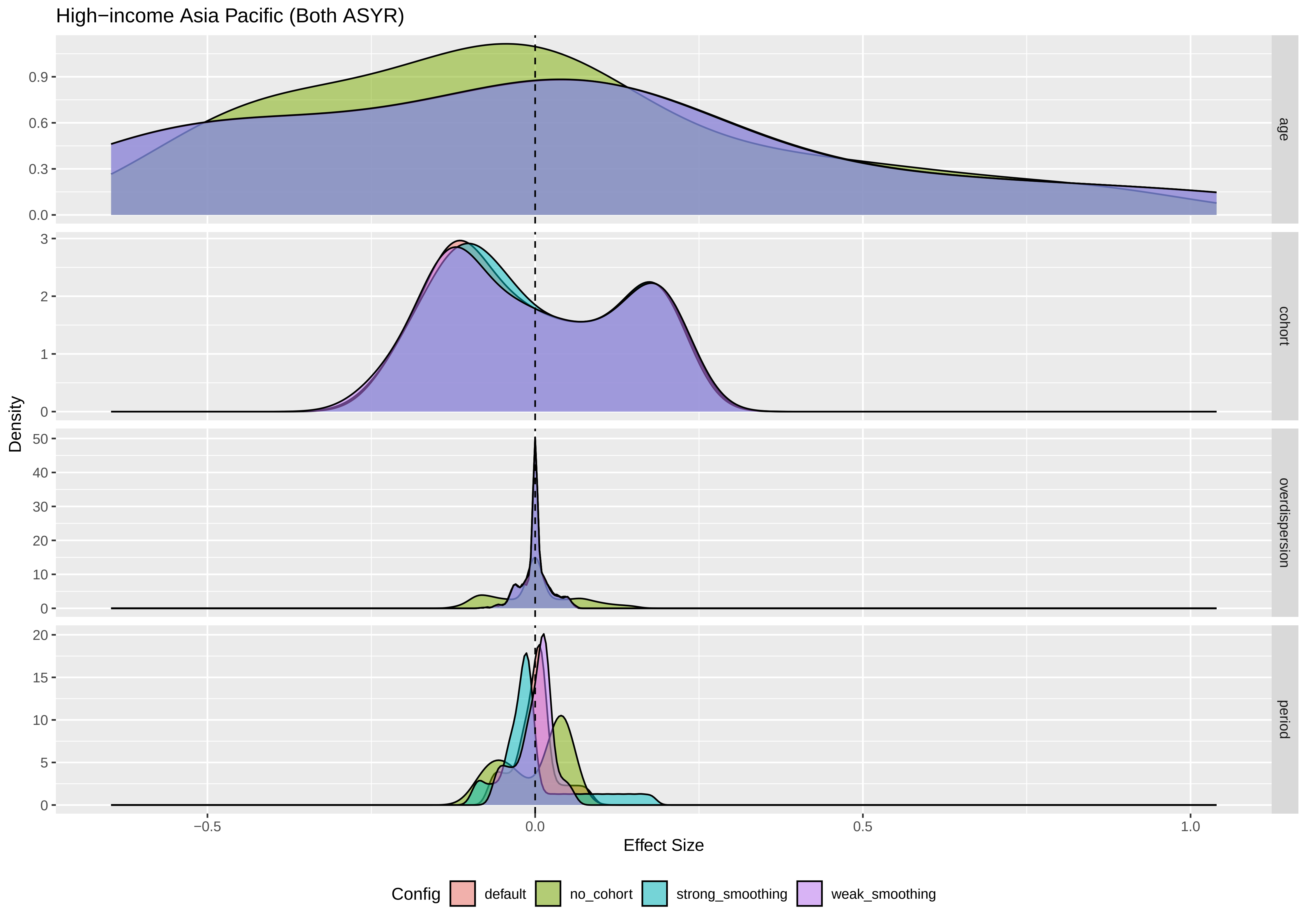

High−income Asia Pacific (Both ASYR)
age
cohort
overdispersion
period
Density
Effect Size
0.0
0.3
0.6
0.9
0
1
2
3
0
10
20
30
40
50
0
5
10
15
20
−0.5
0.0
0.5
1.0
Config
default
no_cohort
strong_smoothing
weak_smoothing

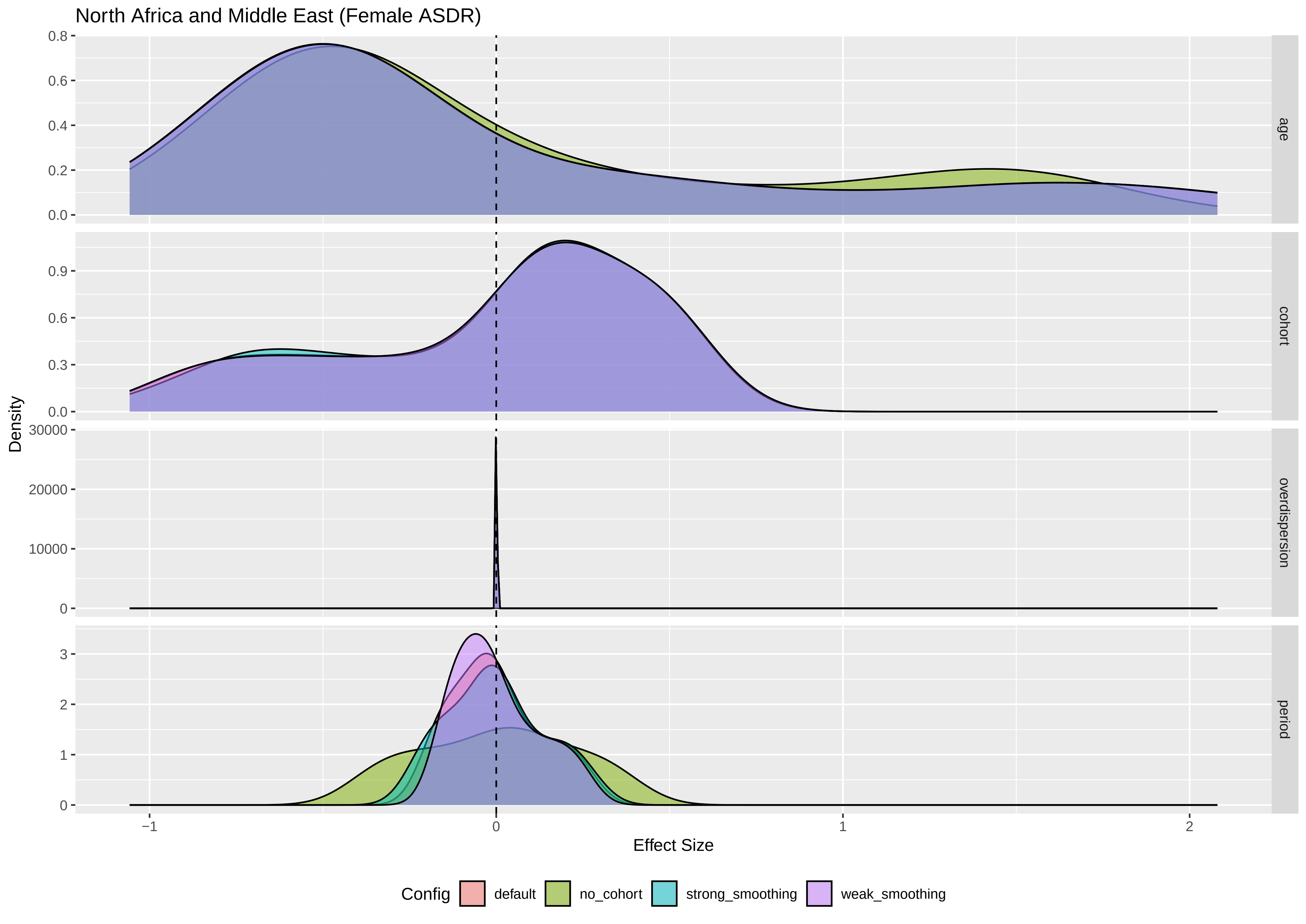

North Africa and Middle East (Female ASDR)
Density
Effect Size
age
cohort
overdispersion
period
0.0
0.2
0.4
0.6
0.8
0.0
0.3
0.6
0.9
0
10000
20000
30000
0
1
2
3
−1
0
1
2
Config
default
no_cohort
strong_smoothing
weak_smoothing

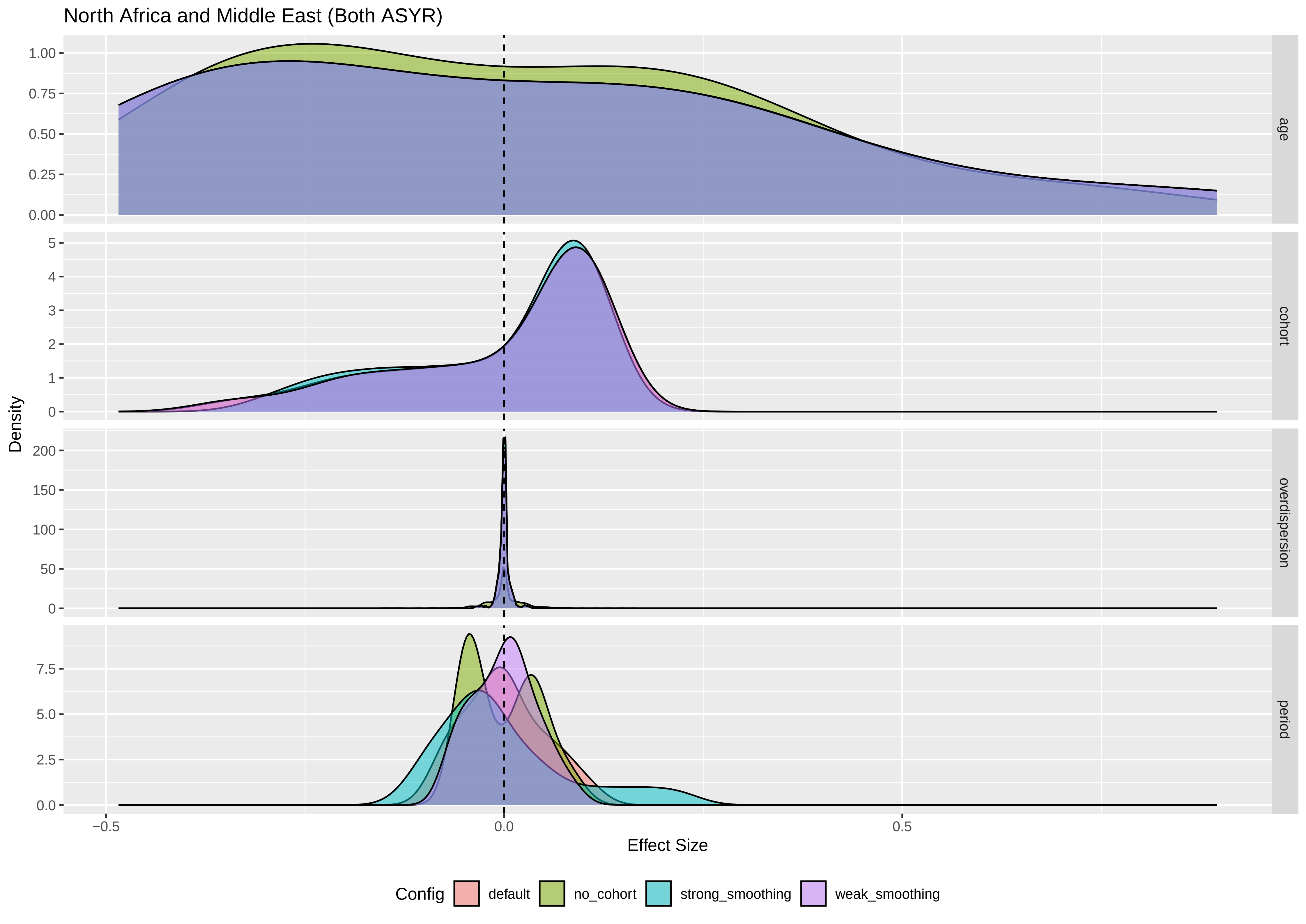
North Africa and Middle East (Both ASYR)
Density
Effect Size
age
cohort
overdispersion
period
1.00
0.75
0.50
0.25
0.00
5
4
3
2
1
0
200
150
100
50
0
7.5
5.0
2.5
0.0
−0.5
0.0
0.5
Config
default
no_cohort
strong_smoothing
weak_smoothing

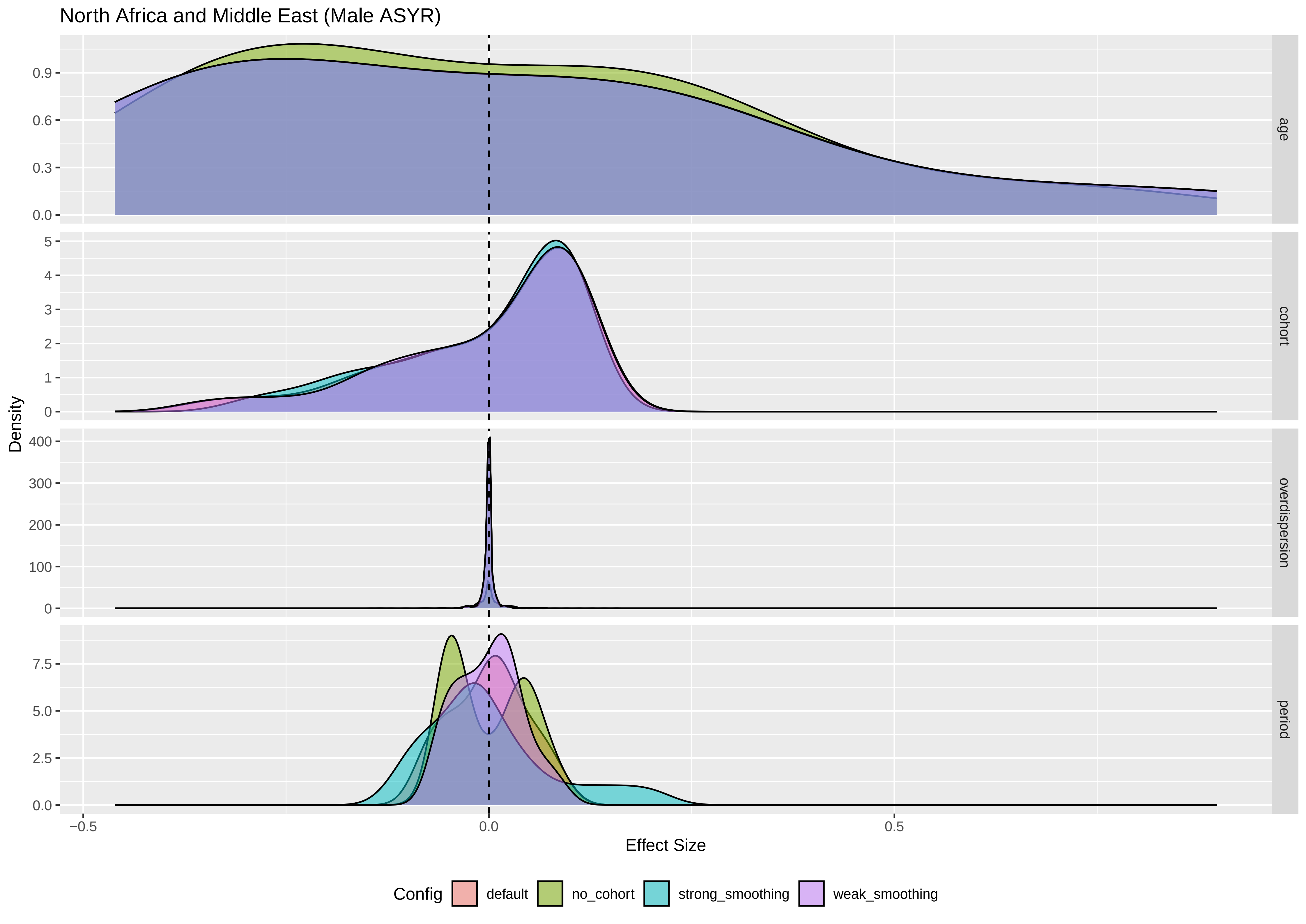

North Africa and Middle East (Male ASYR)
Density
Effect Size
age
cohort
overdispersion
period
0.0
0.3
0.6
0.9
0
1
2
3
4
5
100
200
300
400
2.5
5.0
7.5
−0.5
0.0
0.5
Config
default
no_cohort
strong_smoothing
weak_smoothing

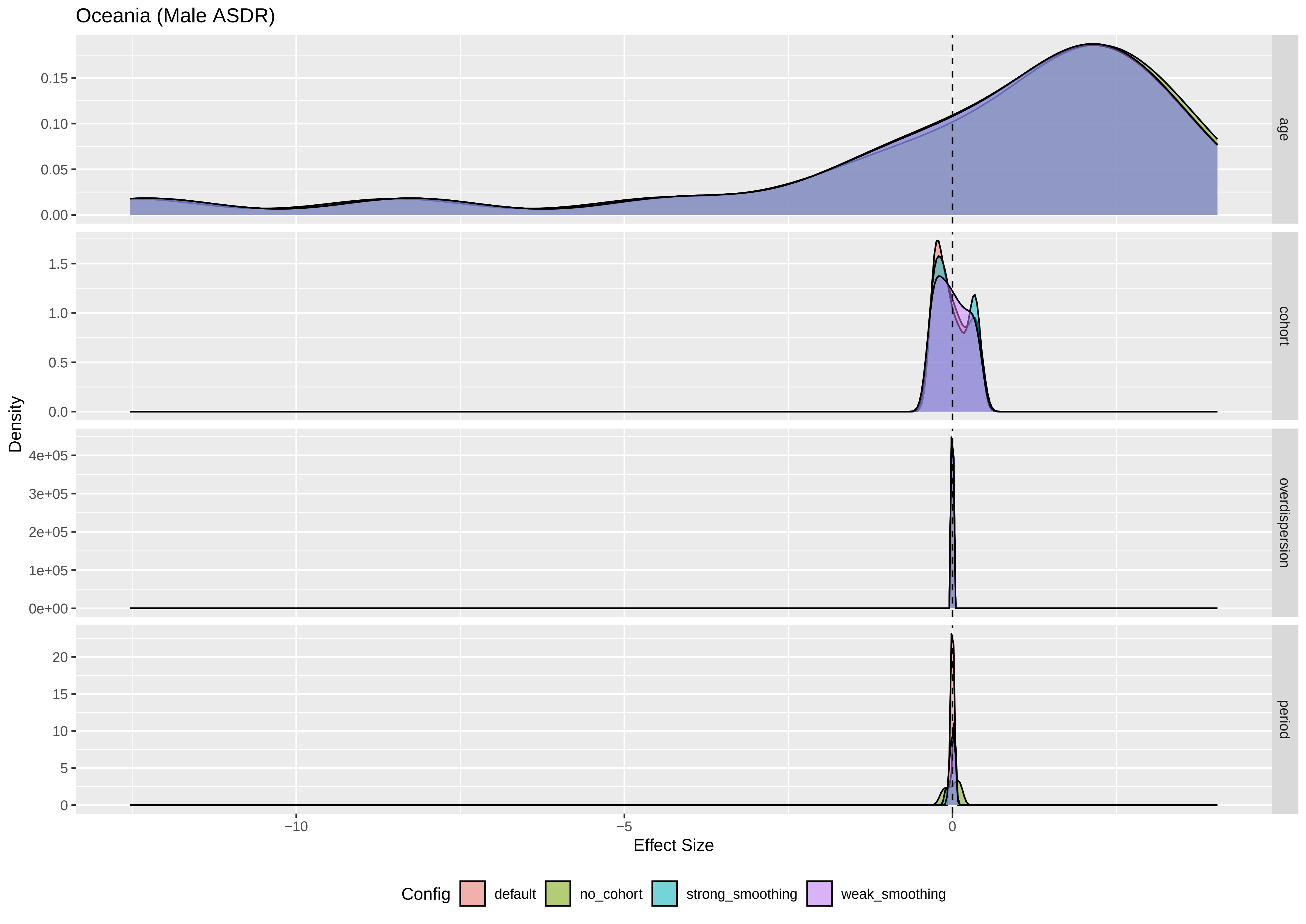

Oceania (Male ASDR)
Density
Effect Size
age
cohort
overdispersion
period
0.00
0.05
0.10
0.15
0.0
0.5
1.0
1.5
0e+00
1e+05
2e+05
3e+05
4e+05
0
5
10
15
20
−10
−5
0
Config
default
no_cohort
strong_smoothing
weak_smoothing

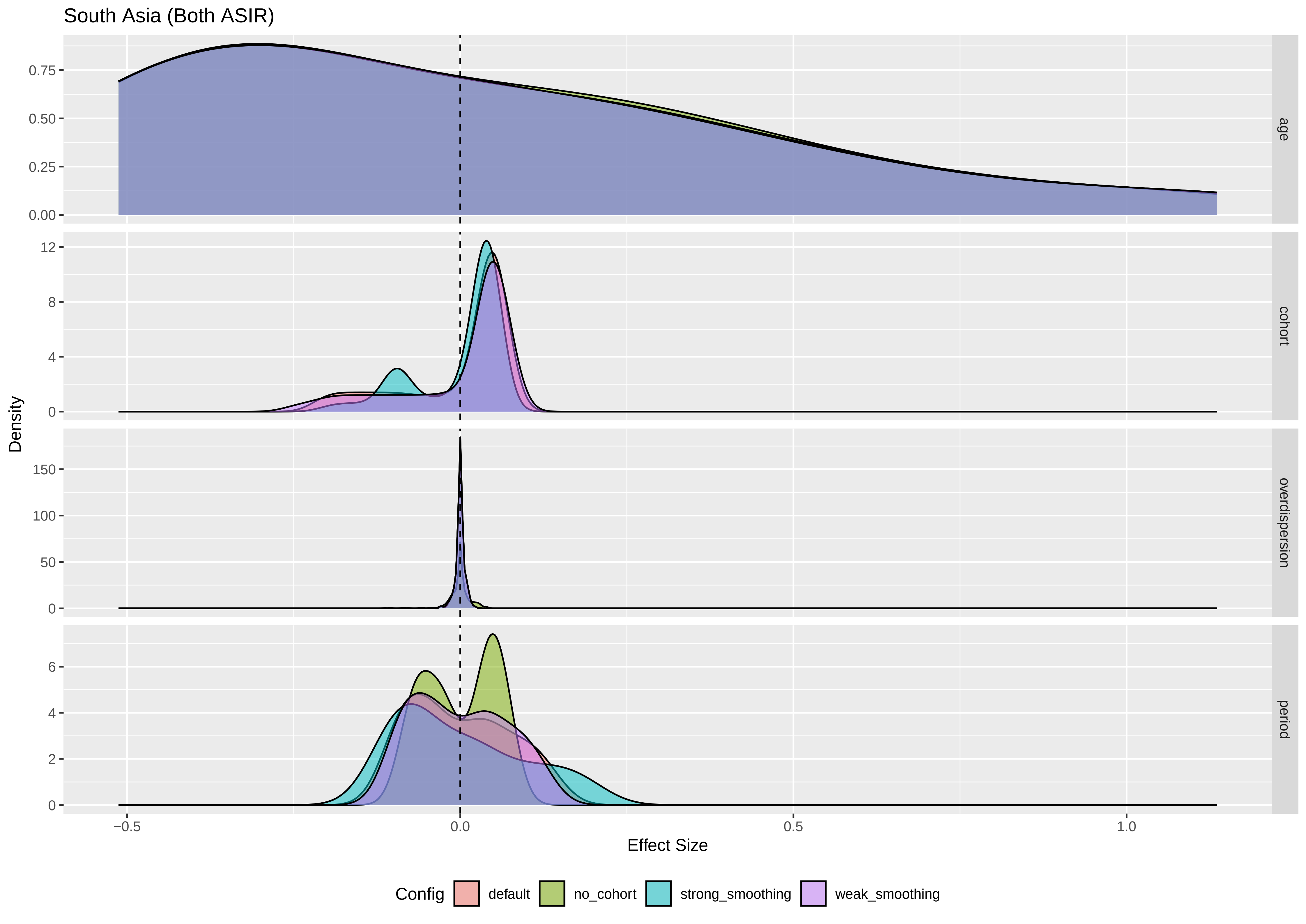
South Asia (Both ASIR)
Density
age
cohort
overdispersion
period
0.00
0.25
0.50
0.75
0
4
8
12
0
50
100
150
0
2
4
6
-0.5
0.0
0.5
1.0
Effect Size
Config
default
no_cohort
strong_smoothing
weak_smoothing

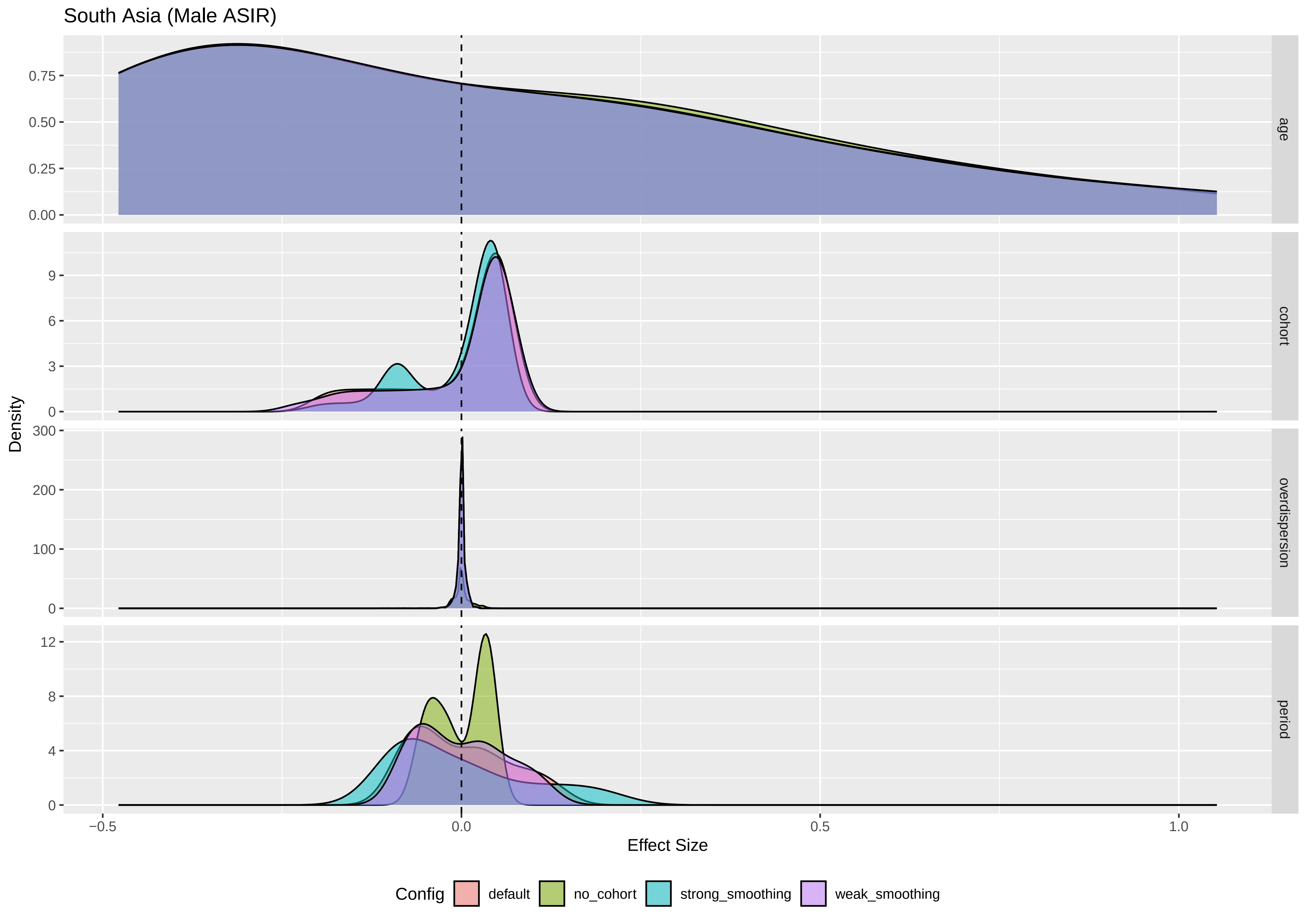

South Asia (Male ASIR)
age
cohort
overdispersion
period
Density
Effect Size
0.00
0.25
0.50
0.75
0
3
6
9
0
100
200
300
0
4
8
12
−0.5
0.0
0.5
1.0
Config
default
no_cohort
strong_smoothing
weak_smoothing

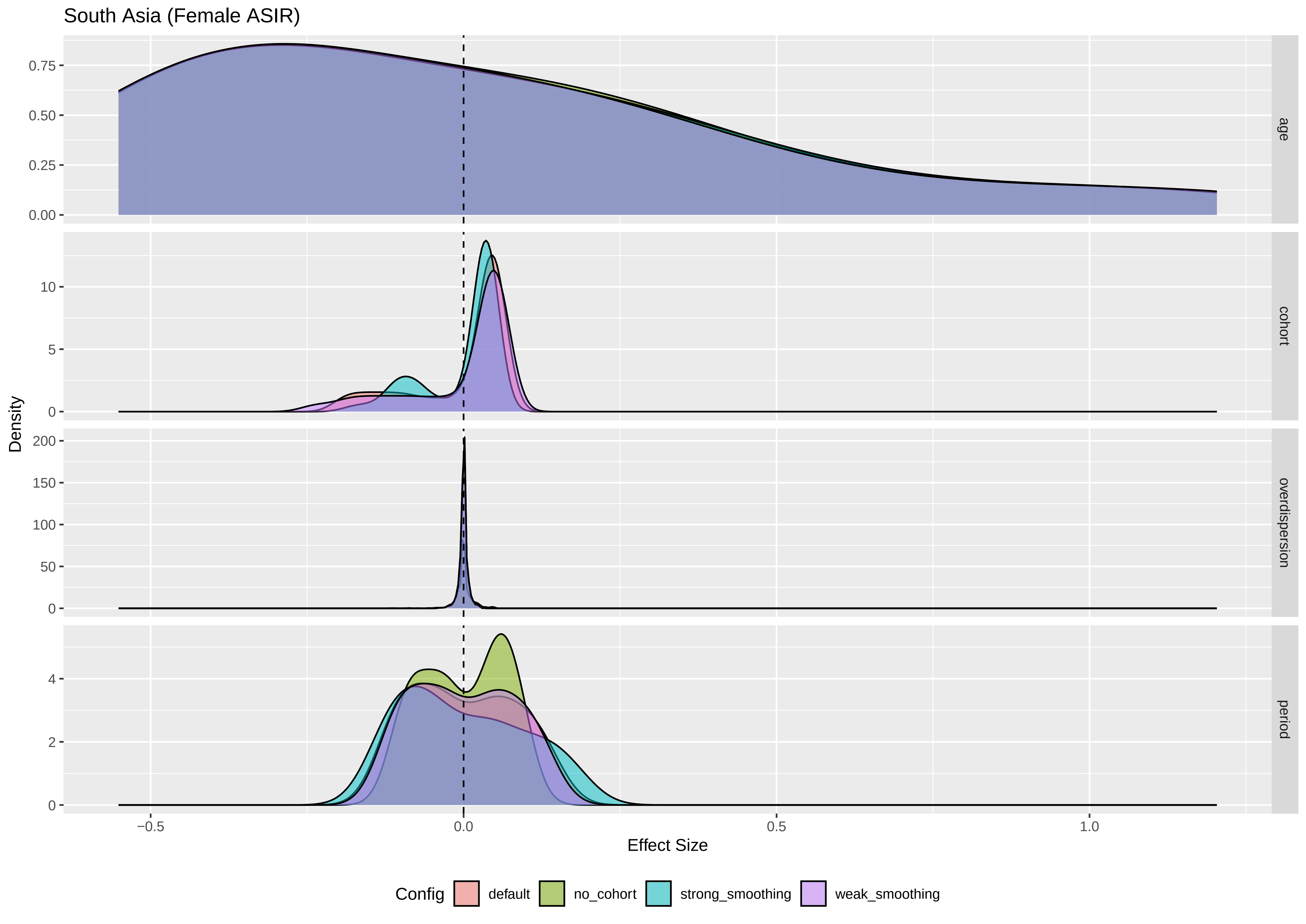
South Asia (Female ASIR)
age
cohort
overdispersion
period
Density
Effect Size
0.00
0.25
0.50
0.75
0
5
10
0
50
100
150
200
0
2
4
−0.5
0.0
0.5
1.0
Config
default
no_cohort
strong_smoothing
weak_smoothing

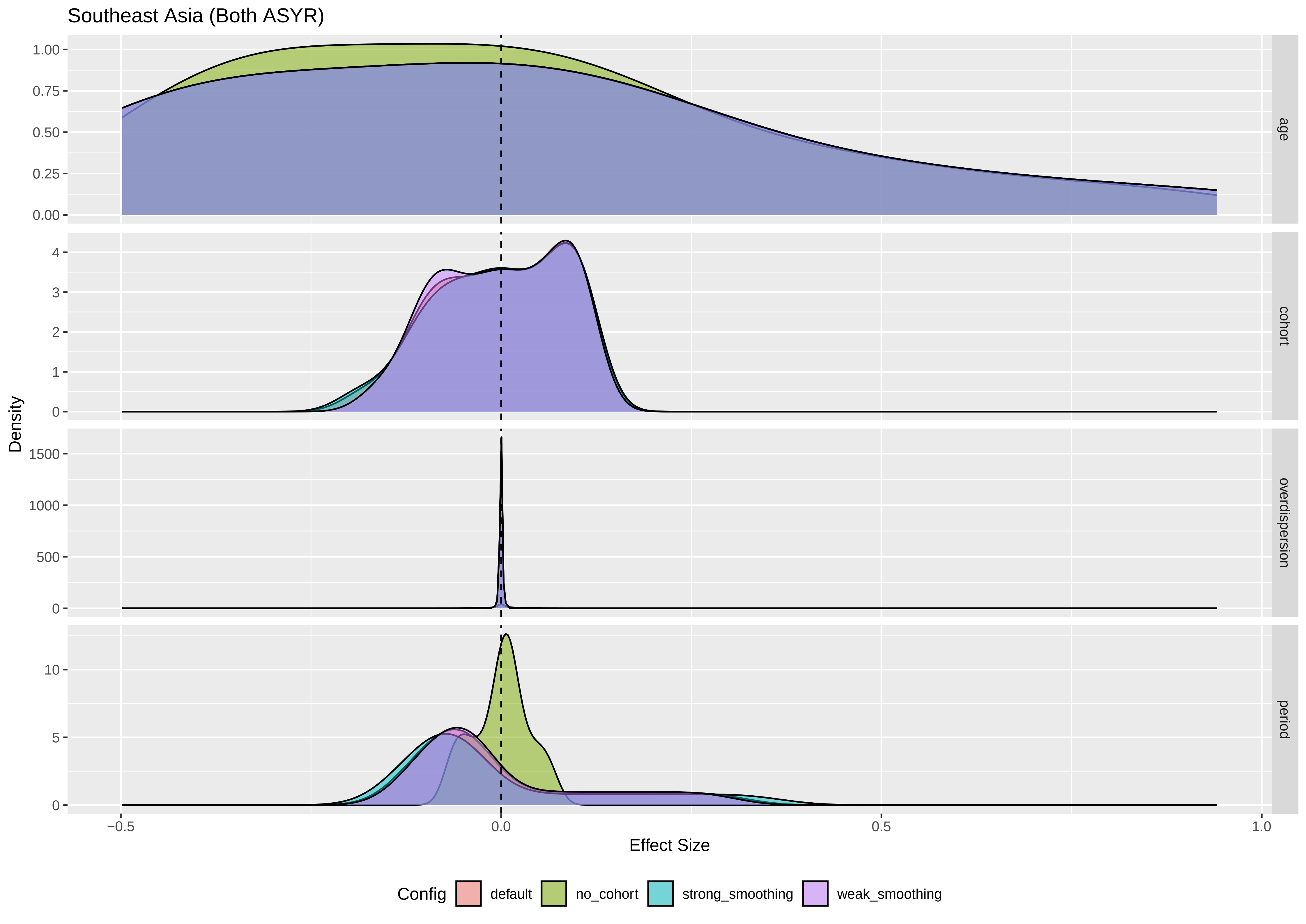
Southeast Asia (Both ASYR)
Density
Effect Size
age
cohort
overdispersion
period
1.00
0.75
0.50
0.25
0.00
4
3
2
1
0
1500
1000
500
0
10
5
0
−0.5
0.0
0.5
1.0
Config
default
no_cohort
strong_smoothing
weak_smoothing

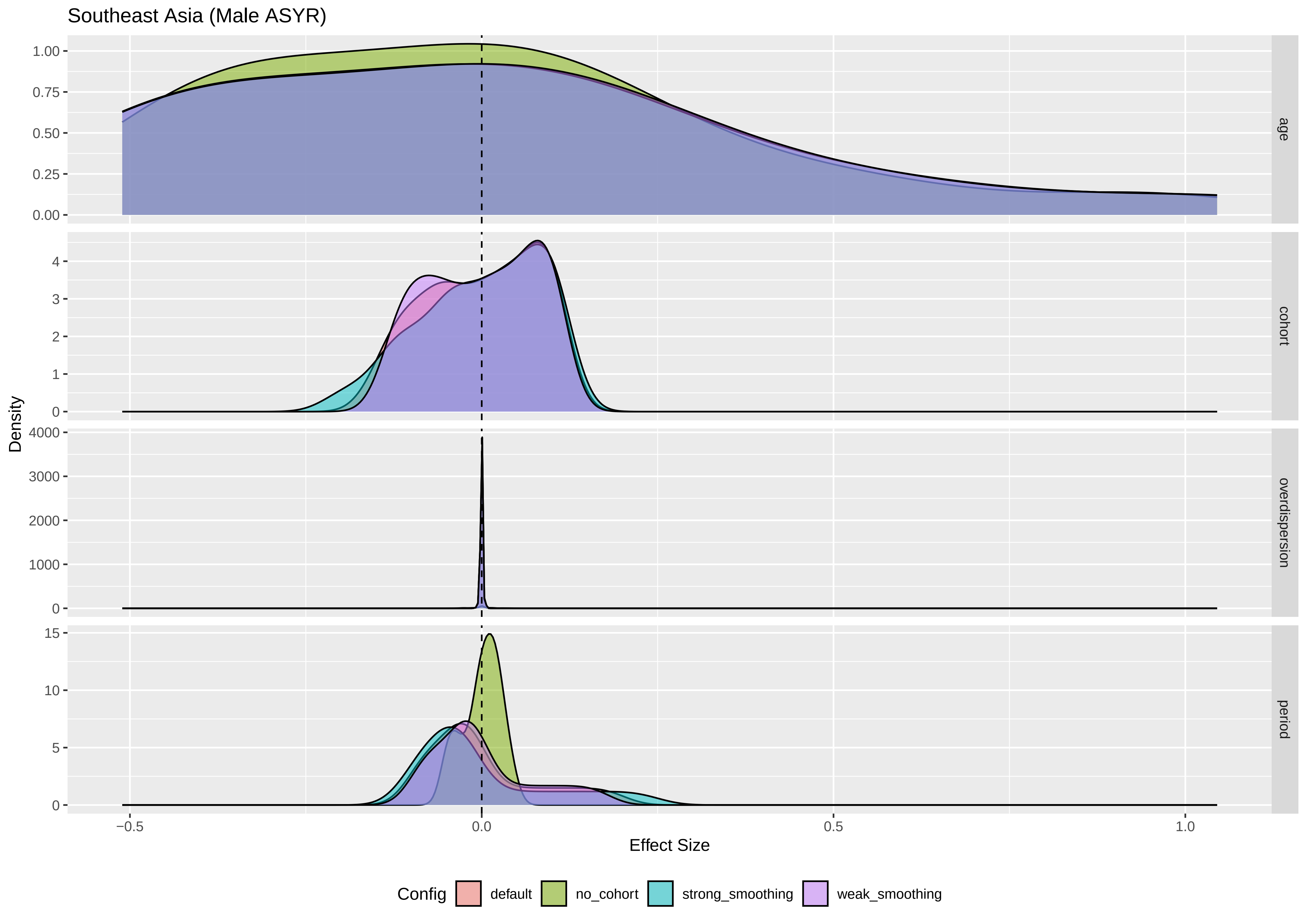
Southeast Asia (Male ASYR)
Density
Effect Size
age
cohort
overdispersion
period
1.00
0.75
0.50
0.25
0.00
4
3
2
1
0
4000
3000
2000
1000
0
15
10
5
0
−0.5
0.0
0.5
1.0
Config
default
no_cohort
strong_smoothing
weak_smoothing

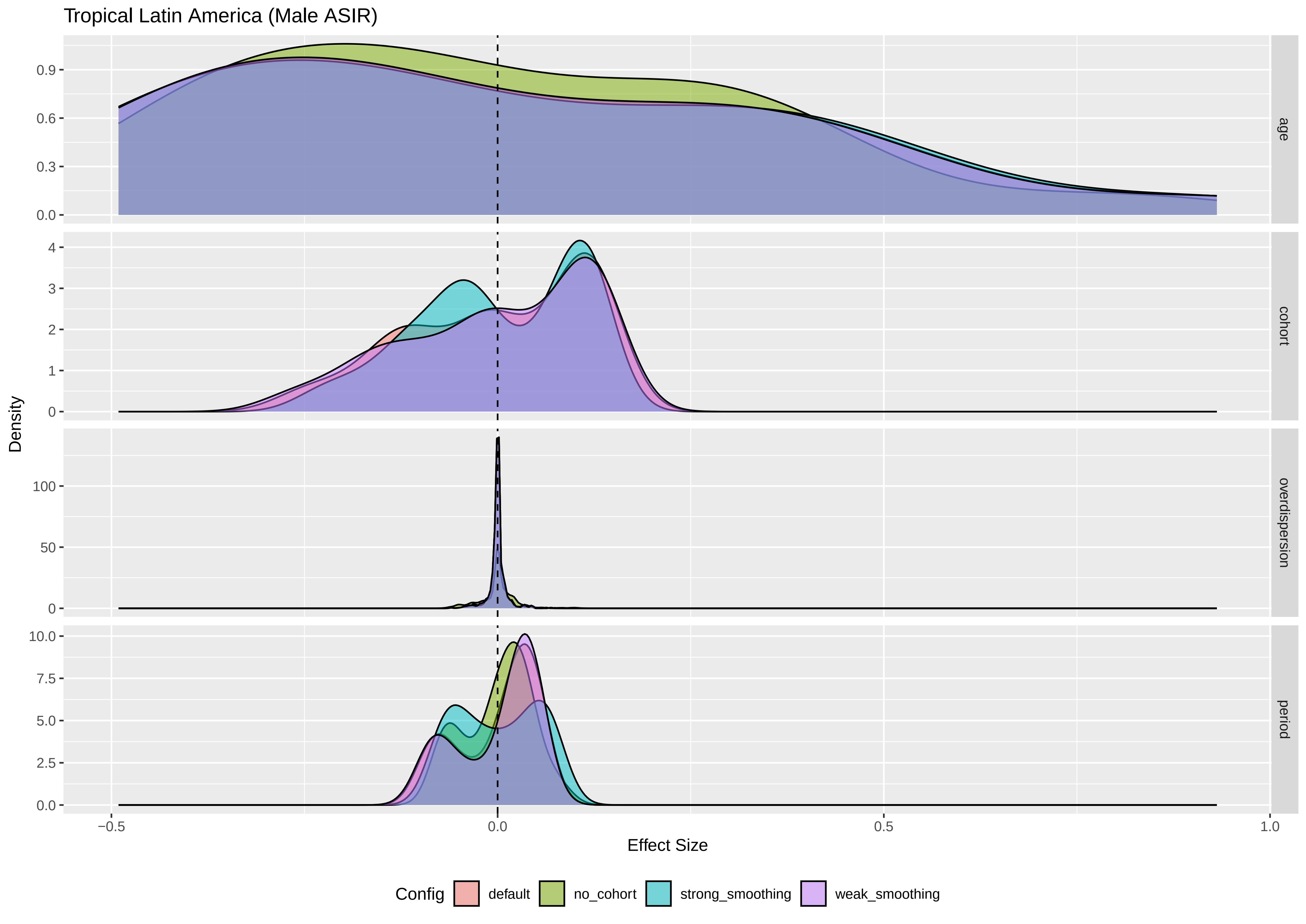

Tropical Latin America (Male ASIR)
Density
Effect Size
age
cohort
overdispersion
period
0.0
0.3
0.6
0.9
0
1
2
3
4
50
100
2.5
5.0
7.5
10.0
−0.5
0.0
0.5
1.0
Config
default
no_cohort
strong_smoothing
weak_smoothing

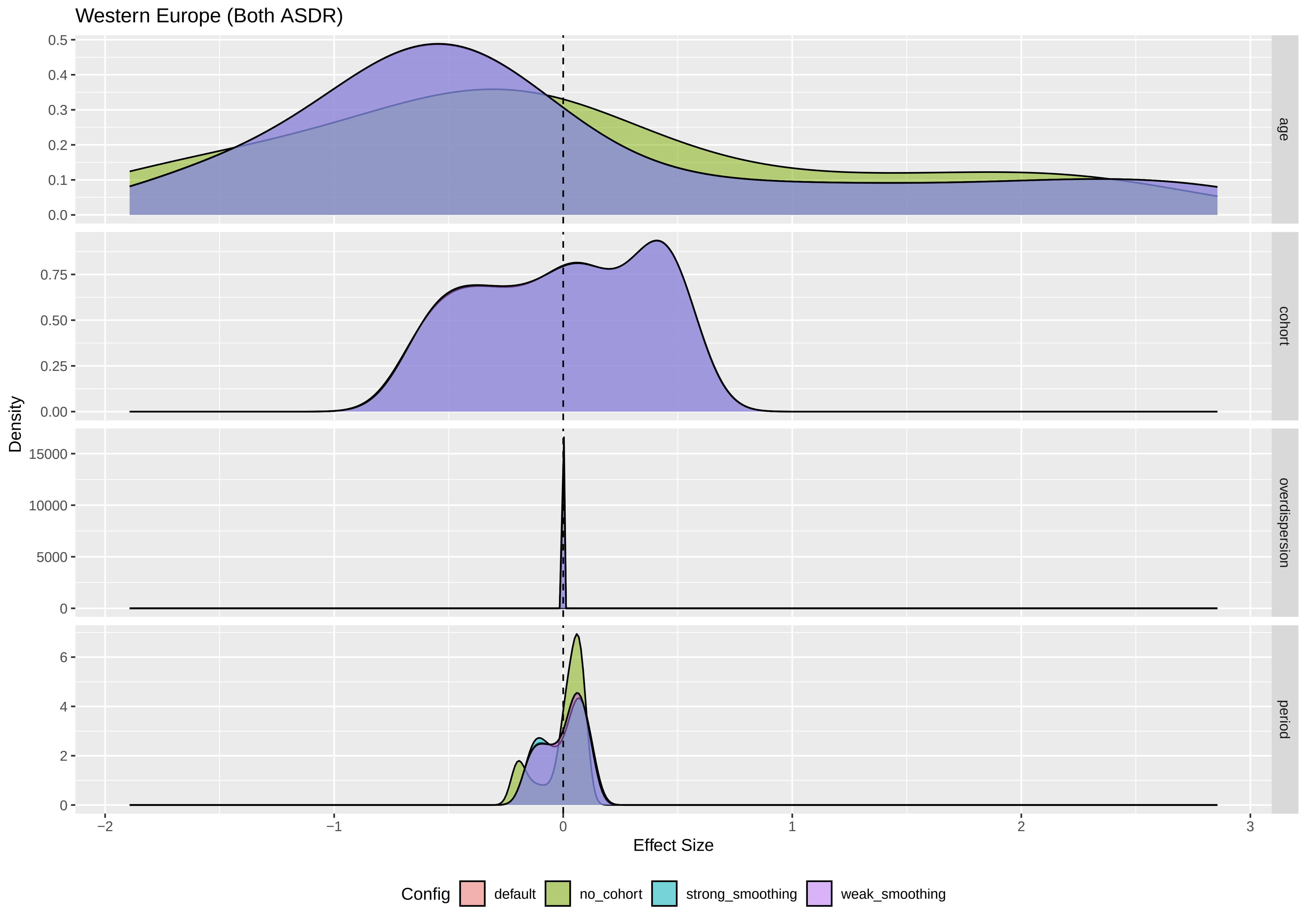

Western Europe (Both ASDR)
age
cohort
overdispersion
period
Density
Effect Size
0.0
0.1
0.2
0.3
0.4
0.5
0.00
0.25
0.50
0.75
0
5000
10000
15000
0
2
4
6
−2
−1
0
1
2
3
Config
default
no_cohort
strong_smoothing
weak_smoothing

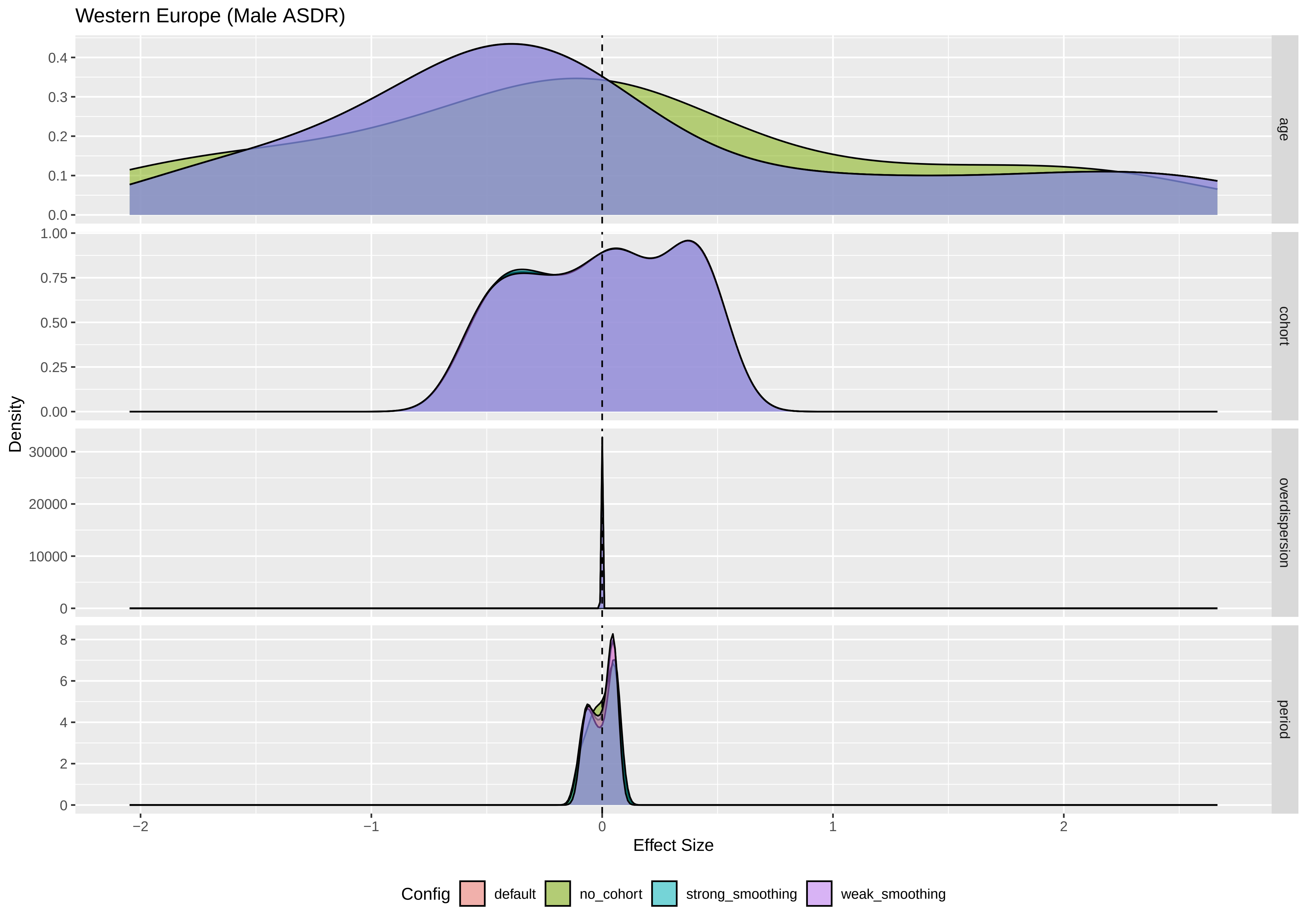
Western Europe (Male ASDR)
Density
Effect Size
age
cohort
overdispersion
period
Config
default
no_cohort
strong_smoothing
weak_smoothing

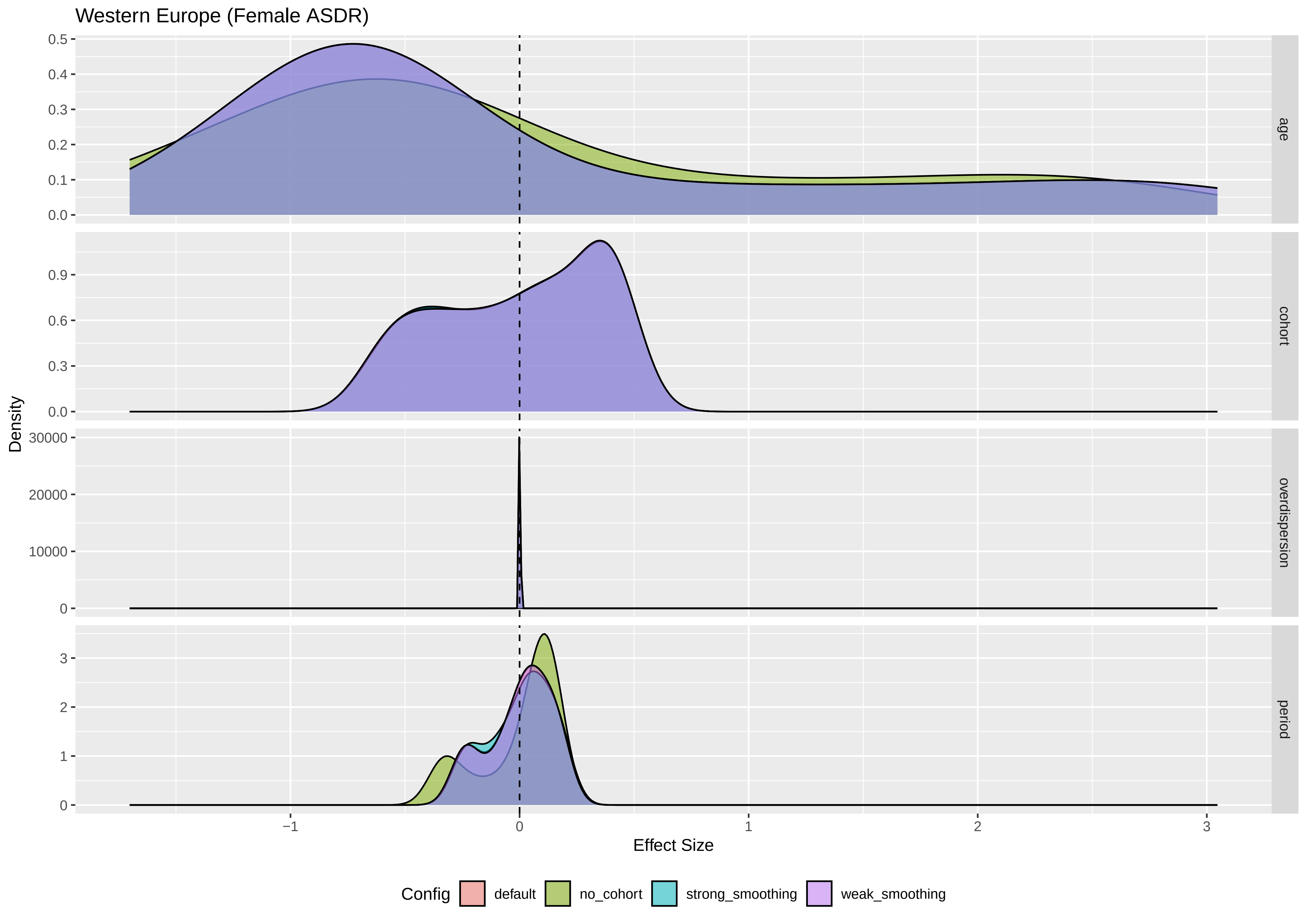

Western Europe (Female ASDR)
0.5
0.4
0.3
0.2
0.1
0.0
age
0.9
0.6
0.3
0.0
cohort
30000
20000
10000
0
overdispersion
3
2
1
0
period
Density
−1
0
1
2
3
Effect Size
Config
default
no_cohort
strong_smoothing
weak_smoothing
